# Single-cell transcriptome analysis suggests cells of the tumor microenvironment as a major discriminator between brain and extracranial melanoma metastases

**DOI:** 10.1186/s13062-025-00691-2

**Published:** 2025-09-16

**Authors:** Konrad Grützmann, Michael Seifert

**Affiliations:** Institute for Medical Informatics and Biometry (IMB), Faculty of Medicine Carl Gustav Carus, Technische Universität Dresden, Dresden, Germany

**Keywords:** Melanoma metastases, Brain metastasis, Extracranial metastasis, Computational comparative gene expression analysis, Single-cell RNA sequencing, Single-nucleus RNA sequencing

## Abstract

**Background:**

Despite therapeutic advances, metastatic melanoma, and particularly brain metastasis (MBM), remains a lethal burden for patients. Existing single-cell studies offer a more detailed view of melanoma and its microenvironment, which is crucial to improve diagnosis and treatment.

**Results:**

We here present a computational reanalysis of single-nucleus data comparing 15 MBM and 10 extracranial melanoma metastases (ECM), considering recent best practice recommendations. We used cell type-specific pseudobulking and omit imputation during patient integration to gain complementary insights. Interestingly, our analysis revealed high homogeneity in tumor cell expression profiles within and between MBM and ECM. However, MBM displayed even higher homogeneity but a more flexible energy metabolism, suggesting a specific metastatic adaptation to the putatively more restricted brain microenvironment. While tumor cells were homogeneous, the metastasis microenvironment, especially lymphocytes and related immune-tumor interaction pathways, exhibited greater divergence between MBM and ECM. Overall, this suggests that major differences between MBM and ECM are potentially driven by variations in their microenvironment. Finally, a comparison of single-cell data to previous bulk studies, including their deconvoluted putative cell types, showed significant differences, potentially causing divergent conclusions.

**Conclusion:**

Our study contributed to refine the understanding of differences between MBM and ECM, suggesting these are potentially more influenced by their local microenvironments. Future research and therapies could possibly focus on the metabolic flexibility of melanoma brain metastases and patient-specific immune pathway alterations.

**Supplementary Information:**

The online version contains supplementary material available at 10.1186/s13062-025-00691-2.

## Background

Melanoma is the most lethal skin cancer [1] and exhibits the third-highest incidence of brain metastasis, trailing lung and breast cancer [2]. While local melanoma can be treated well, metastatic melanoma has a low five-year survival rate of 20% [3]. The development of targeted therapies [4] and modern immunotherapy were breakthroughs that substantially improved the five-year overall patient survival [5, 6]. However, a significant proportion of patients with metastatic melanoma experience recurrence of existing brain metastases or develop new ones [7]. Initial treatment responses of melanoma brain metastases are often short-lived compared to those seen in other organ metastases, resulting in a substantial decline in overall patient survival [8, 9]. A better understanding of the molecular and systemic differences between melanoma brain (MBM) and extracranial metastases (ECM) is crucial for advancing treatment options, and, ultimately, patient outcomes. Studies focusing on the genetic differences have, for example, found recurrent driver gene mutations [10] and a higher *BRAF* mutation rate [11] in brain metastases. Gene expression analyses identified immune suppression, oxidative phosphorylation [12], and PI3K/Akt signaling as deregulated in MBM compared to ECM [13–15]. Also, epigenetic alterations significantly influence melanoma metastasis, including brain metastases [16–18]. DNA methylation changes exhibit variability between patients and even between intra- and extracranial metastases within patients [18], potentially contributing to the unique characteristics of brain metastases [14, 19].

Tumors do not represent isolated homogeneous tissues. They are usually very heterogeneous and carry out bidirectional interactions with their microenvironment. Understanding this interplay is crucial for advancing insights into melanoma metastasis. Traditional bulk tumor tissue analysis examines cell populations collectively, yielding only averaged signals and failing to capture intra-tumoral heterogeneity [20]. Additionally, samples from tumor dissections are rarely 100% pure, often containing, e.g., immune cells, thus introducing potential biases into bulk analyses. Single-cell RNA sequencing (scRNA-seq) is becoming a gold standard for evaluating tumor heterogeneity and evolution, thereby facilitating personalized medicine [21], including advancements for therapies of melanoma [22]. Tirosh et al. were the first to explore expression variation in the tumor and microenvironment of primary and metastatic melanoma by scRNA-seq [23]. This technique enables a precise dissection and characterization of the melanoma microenvironment. For example, a gradual shift of many CD4 + and CD8 + T cells towards dysfunctional states and a strong corresponding link to tumor reactivity were found [24]. scRNA-seq enables a detailed study of expression dynamics of melanoma during genesis and evolution [25]. scRNA-seq also helped to unravel the microenvironment’s influence on melanoma therapy response. For example, a monocyte subtype predictive of PD-1 immunotherapy response [26] and new prognostic expression markers in Tregs of melanoma were found [27]. Further, the distribution of cancer-associated fibroblast expression markers was verified, corroborating their use for prognosis and therapy response [28]. Also, the rarer uveal, acral, and conjunctival melanoma subtypes were studied in more detail with the help of scRNA-seq [29–31]. A recent study by Biermann et al. found that MBM exhibit increased chromosomal instability, a neuronal-like phenotype, and spatially variable metabolic activity compared to extracranial metastases [32].

scRNA-seq captures unprecedented numbers of cells but suffers from much higher sparsity, expression variability, and technical variation compared to traditional RNA-seq [33]. Integration methods aim to correct technical variations to improve sample comparability. They also address the problem of missing values by imputation [34, 35]. However, this may artificially reduce variance and inflate downstream p-values. Furthermore, methods that consider individual cells as independent observations [36, 37] further inflate p-values in differential expression testing. Techniques using “pseudobulking” can circumvent this [38]. Thus, recognizing these challenges, best practices for scRNA-seq data analysis [39] are continually evolving and improving [40–42].

Especially the study by Biermann et al. provides a rich multi-omic single-cell reference of 22 melanoma brain and 10 extracranial metastases, available for further exploration and comparative analysis of the metastatic sites [32]. We avoid the above-mentioned difficulties by only using measured, but not imputed, expression values after expression integration, and also “pseudobulk” cell type-specific expression values within each sample. We reanalyze the single-nucleus expression data with these alternative computational strategies to gain new perspectives to characterize and understand molecular differences between brain and extracranial metastases. We find that tumor cells display highly similar expression profiles with limited variation between MBM and ECM. Notably, MBM tumor cells exhibit even greater uniformity than ECM, indicating a restrictive brain microenvironment for melanoma metastasis. The subtle expression differences in tumor cells are associated with energy metabolism, especially oxidative phosphorylation, adherens junctions, and neuroinflammation. Conversely, cell types of the microenvironment show significant divergence and appear to drive the distinction between the MBM and ECM phenotypes. Specifically, the lymphocyte lineages in MBM and ECM differ by a wide spectrum of immune regulatory processes, encompassing proliferation, expansion, and inflammation. Additionally, endothelial cells indicate a divergent development of blood vessels. Finally, we contribute another added value by comparing the single-cell data with previous bulk studies and with their deconvoluted putative cell types, demonstrating significant differences. This potentially leads to divergent conclusions, highlighting the need for further single-cell studies for a more nuanced understanding of melanoma metastases.

## Methods

### Single-cell data acquisition, integration, and removal of imputed expression values

Unprocessed single-nucleus RNA (snRNA) count data were downloaded from NCBI’s GEO database (GSE200218). Processing was done based on the code provided by Biermann et al. (https://github.com/IzarLab/Melanoma_Brain_Metastasis), mainly using the Seurat R package (v4.3.0) [34]. Briefly, cells with 500—10,000 genes, 1000–60,000 counts, and < 10% mitochondrial reads were kept. After removing doublets with Scrublet v0.2.1 [43] and DoubletFinder v2.0.3 [44], data were normalized with the ‘NormalizeData’ function using the ‘LogNormalize’ method and scaled with the ‘ScaleData’ function. Then, metadata (GSE200218_sc_sn_metadata.csv.gz) that include cell type annotations were merged to the data. Samples MBM21 and MBM10 were removed as they had too few tumor cells (2 and 82, respectively). Data integration was done with reciprocal principal component analysis (RPCA) [34], which allowed us to keep the top 17,500 variable genes compared to only 2,000 in the original analysis using the methods “SelectIntegrationFeatures”, “FindIntegrationAnchors”, and “IntegrateData”. Cell identities ‘Low-quality cells’, ‘Doublets’, ‘Contamination’, and ‘Undetermined’ were removed. Next, PCA and uniform manifold approximation and projection (UMAP) reduction were performed with 80 principal components. Expression matrix entries (gene/cell) with missing values in the raw data were set to NA to prevent downstream use of artificial values imputed by RPCA. Data were then aggregated (“pseudobulked”) by patient and cell type, calculating mean expression across all cells within each type. Genes of a patient and cell type were retained only if expressed in at least 10 cells. In addition to UMAP and PCA plots of the single-cell data, PCA with 10 principal components was calculated for the aggregated expression data using the NIPALS R package [45] and the first two components were plotted. This was done for the expression data of tumor cells and for data summed over all cell types. The complementary UMAP and PCA plots allow potential outlier detection. A comparison to the Methods of the original study by Biermann et al. is provided in Appendix 1. The original code for processing was archived at GitHub (https://github.com/konradgrutz/single_cell_melanoma_metastasis, 10.5281/zenodo.17092986) and data deposited at Zenodo (10.5281/zenodo.16981813).

### Differential gene expression analysis

Differential gene expression analysis comparing brain with extracranial metastases was done for each cell type (cell_type_int of the annotation) applying limma [46] on the aggregated data. Only genes with aggregated expression values in at least four patients per group were tested. Adjustment for multiple testing within cell type results was done using the false discovery rate (FDR) [47] with the cutoffs 0.05, 0.1, and 0.2. Genes were annotated with information from the Ensembl database v114 and Cancer Gene Census v101 [48]. The differential expression analysis was repeated once adjusting for treatment as cofactor and once excluding the five treated ECM patients. In the latter version, genes with aggregated values from at least three patients per group were tested accounting for the few remaining ECM samples. The FDR cutoff was set to 0.1. The differential gene expression analysis was also repeated with preprocessed, non-integrated data that was aggregated in the same way to evaluate a potential bias from the sample integration.

### Correlation of expression profiles

Pairwise Pearson correlations were calculated between patients’ aggregated expression profiles, and their distribution was presented as violin plots using the ggplot2 R package [49]. For single-cell data correlation, expression correlation (Pearson) was calculated for each cell type by averaging the correlation of 100 randomly selected cell pairs from each possible sample pairs. We applied a mixed-effects model with patient pairs as a random effect and group as a fixed effect using the lmer, emmeans, and pairs functions from the lmerTest [50] and emmeans packages [51] with standard parameters. FDR was calculated for multiple test correction over all performed tests [47].

### Cell type-specific expression signatures defining the brain and extracranial melanoma metastasis phenotypes

A cluster analysis was performed to find which cell types define the brain and extracranial phenotypes most. The top 50 differentially expressed genes (lowest p-value) of each cell type were subjected to hierarchical cluster analysis, including heatmap plots (ComplexHeatmap R package [52]) using the aggregated expression with Euclidean distance and complete linkage. Cluster stability was assessed with pvclust [53]. Genes were annotated with information about cancer signaling pathways, transcription (co-) factors, oncogenes, tumor suppressors, and the Cancer Gene Census [48] collected by Grützmann et al. [54].

### PubMed annotations of differentially expressed genes and signature genes

The NCBI PubMed database was searched for publications about the differentially expressed genes and signature genes with at least two tumor associations (see below). For each gene, an initial search used the term “gene symbol AND melanoma”. If no results were found, the search was repeated with “gene symbol AND tumor”. Up to ten bibliographic entries, including abstracts, were downloaded per gene. This information was converted into an HTML document, highlighting gene names, their synonyms, and the keyword fragments “melan”, “tumor”, “cancer”, and “neopla”.

### Functional enrichment analysis of cell type-specific differentially expressed genes

Functional enrichment analysis was performed using gprofiler2 [55] for each cell type separately. All genes were ranked by unadjusted differential expression p-value and subjected to incremental enrichment testing (gost function, ordered_query = TRUE, domain_scope = ‘custom’, custom background: all 17,500 genes). This way, enrichment was assessed for Gene Ontology [56] (GO) molecular function and biological process terms, KEGG pathways [57], and WikiPathways [58] among the genes with the highest differential gene expression potential. Genes were not separated into more highly and more lowly expressed in MBM compared to ECM. Significant results were determined using a multi-testing adjusted p-value of < 0.05 (g_SCS correction). TreeMap [59] visualizations of enriched GO terms were generated using the treemap R package, following GO term redundancy reduction with rrvgo [60] (‘Rel’ similarity, threshold = 0.7, score = -log10(adjusted p-value)).

### Expression patterns of cancer-associated signature genes across cell types and association with known melanoma signatures

The top 50 differentially expressed genes (lowest p-value) of each cell type were restricted to genes annotated as oncogene, tumor suppressor, COSMIC Cancer Census gene [48], or genes involved in cancer signaling (annotation from Grützmann et al. [54]). Two gene lists were compiled: one with genes with at least two such annotations for the main results and a larger one with genes with at least one annotation for supplementary results. Then, the expression of these genes across all cell types was inspected by averaging the aggregated expression over patient samples within the MBM and ECM groups separately for each gene and cell type. Dotplots were generated with the ggplot2 R package [49], coding expression strength as color gradient and the proportion of patients expressing the gene in each group as dot size. Further, the signature genes with at least two cancer annotations were subjected to the WIMMS server (https://wimms.tanlab.org/) to determine associations with the seven principal classes of melanocytic gene signatures of previously identified phenotypes (Neuro, Mitotic/MYC, Invasive, Hypometabolic, Differentiated, AXL, and Amelanotic) [61].

### Cell type-resolved expression patterns of candidate genes of previous bulk studies

A list of candidate genes identified in prior bulk tissue analyses that also appeared in the data analyzed here was compiled. It comprised 20 genes predicted to be differentially expressed between MBM and ECM in at least two of the three considered studies [12, 13, 15]. A dotplot was generated displaying the average aggregated expression in the MBM and ECM patient groups in the data presented here, coding expression strength as color gradient and the proportion of expressing patients in each group as dot size. Further, bulk expression of the single-cell data was simulated for a better comparison to the previous studies by summing average per-cell-type expression levels and then calculating log_2_-fold-changes. In addition, the bulk expression profiles from two of the three studies [13, 15] were computationally deconvoluted using CIBERSORTx [62] with standard settings to predict potentially included cell types and their specific expression profiles. The required cell type-resolved, single-cell reference transcriptomes from the melanoma study by Tirosh et al. [23] were provided by CIBERSORTx.

## Results

### Joint snRNA-seq integration of melanoma brain and extracranial metastasis

**Fig. 1:**
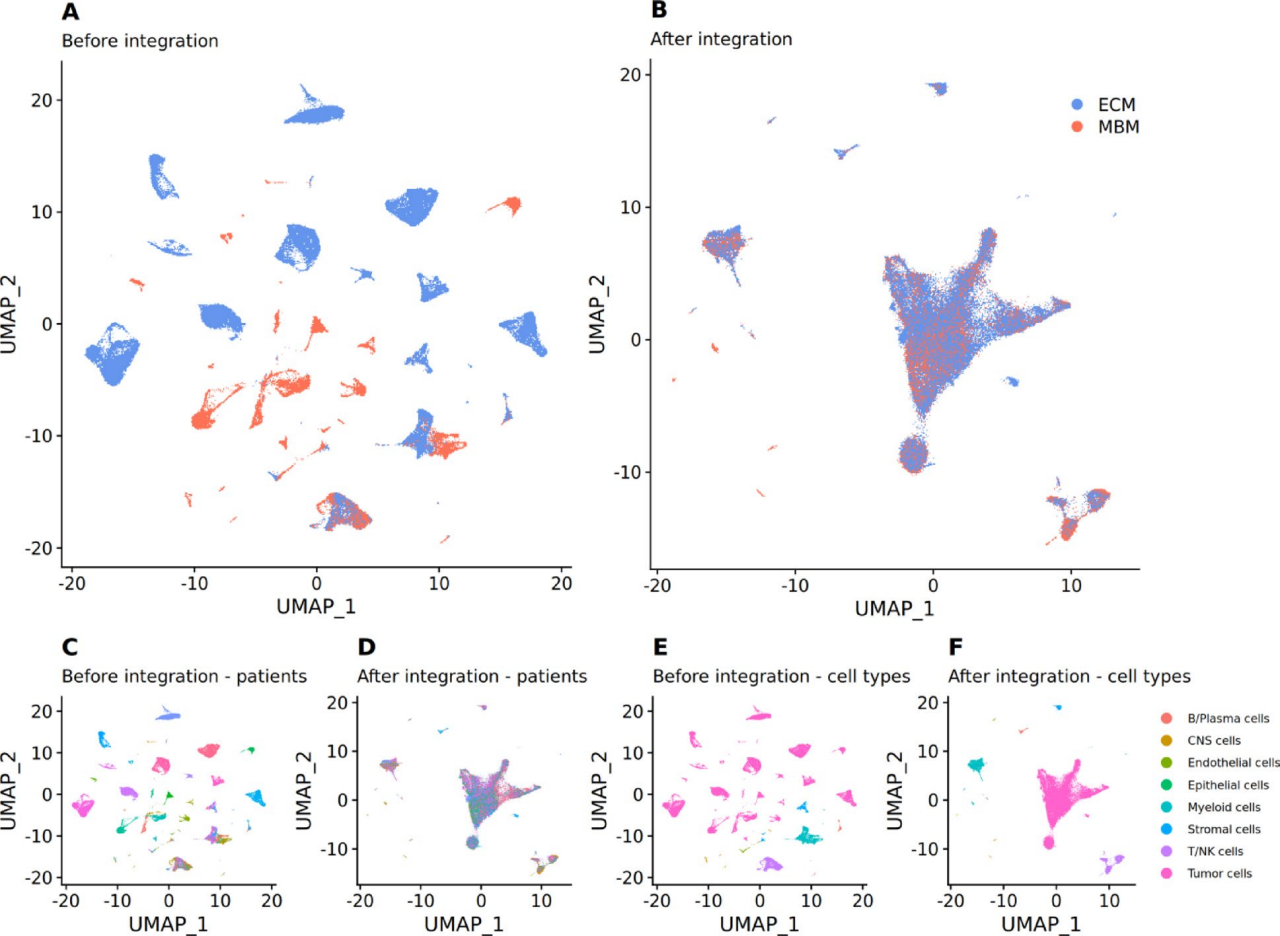
UMAP plots of the single-cell data before (**A**) and after (**B**) integration of patient samples highlighting cells of MBM (blue) and ECM (red) samples. Subplots C-F show the same UMAP components before (**C**, **E**) and after (**D**, **F**) data integration. Colors in subplots C and D highlight patients; colors in E and F highlight cell types. Cell types: CNS—central nervous system, NK cells—natural killer cells

Single-nucleus RNA (snRNA) expression data of 15 patients with melanoma brain metastases (MBM) and 10 extracranial melanoma metastases (ECM) from Biermann et al. [32] were integrated (Fig. 1). The resulting data encompassed expression values for 17,500 genes across an average of 4,088 cells per patient. It captured the diverse cell types within the tumor microenvironment, for example, epithelial and stromal cells, B cells, T cells, and natural killer cells. The overall expression profiles of patients were highly similar (Fig. 1D), while cell types could be distinguished clearly (Fig. 1F). PCA plots of the single-cell data did not show a global bias with respect to individual patients or patient groups (Suppl. Figure 1A and B). Overall, due to the snRNA data sparsity, a median of 88% of expression values were imputed by the integration method. In the following, imputed values were removed, retaining only true measurements for subsequent analyses. Additionally, the expression data were pseudobulked by patient and cell type to ensure a robust data basis for these analyses (Suppl. Table 1). PCA plots of the pseudobulked expression data of tumor cells and of cell type-mixed data revealed that MBM, ECM and previously treated ECM samples did not show clear outliers that could cause a global bias (Suppl. Figure 1C and D). A comparison to the Results of the original study by Biermann et al. [32] is provided in Appendix 1.

### Brain and extracranial metastases differ by microenvironment rather than tumor gene expression

**Fig. 2:**
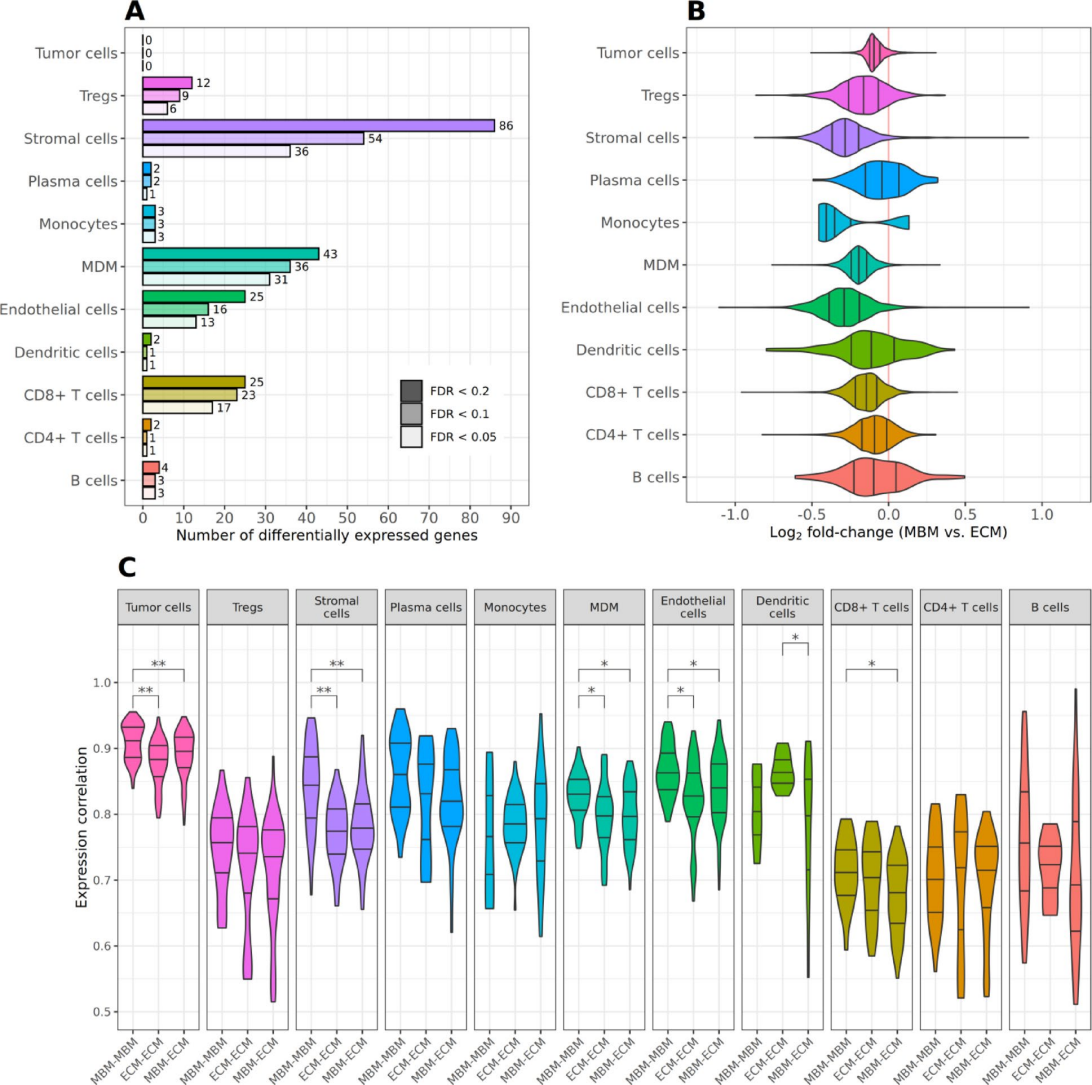
**A**–**C** Results of the differential gene expression analysis comparing the aggregated expression of tumor cells of MBM with ECM patient samples. **A** Number of significantly differentially expressed genes for different adjusted p-value cutoffs (FDR: false discovery rate). **B** Distribution of the log_2_-fold-changes of all tested genes. **C** Comparison of the correlation of gene expression profiles of MBM and ECM patients for different cell types. Unpaired t-tests with FDR correction compared the correlation distributions between MBM and ECM groups within each cell type (‘*’ FDR < 0.05, ‘**’ FDR < 0.001)

Next, we performed a differential gene expression analysis for 11 shared cell types, comparing MBM and ECM patients. Surprisingly, no gene showed significant expression differences between the tumor cells of MBM and ECM patients, neither with a FDR < 0.05, nor with relaxed cutoffs of 0.1 and 0.2 (Fig. 2A, Suppl. Table 2). By contrast, several differentially expressed genes were found in the other cell types, especially stromal cells (36 genes), monocyte-derived macrophages (MDM, 31 genes), CD8 + T cells (17 genes), and endothelial cells (13 genes) (FDR < 0.05, Fig. 2A, Suppl. Figure 2, Suppl. Table 2). In all cell types, a clearly greater number of genes exhibited lower expression in MBM compared to ECM than vice versa (Fig. 2B, Suppl. Figure 2, Suppl. Table 2). Most of these genes were protein-coding, but a few differentially expressed long non-coding RNAs were found, too.

Because of the absence of differentially expressed genes in tumor cells, we further investigated if tumor cells would be much more similar within and across the MBM and ECM groups than the other cell types. Globally, expression differences between MBM and ECM were indeed low and in a narrow range in tumor cells compared to the other cell types (Fig. 2B). A comparative correlation analysis of the patients’ cell type-specific pseudobulked expression profiles showed that tumor cells were much more similar than other cell types (Fig. 2C). This was true for patients within and across the MBM and ECM groups. Tumor cells may be more homogeneous in their functional alterations, and hence their expression profiles, compared to, for example, CD8 + T cells with their specialized subtypes. Stratification showed that CD8 + TOX + and CD8 + TCF7 + subtypes [32] showed higher expression correlation than their parent CD8 + T cell population in the single-cell data (Suppl. Figure 3^3^), indicating greater expression uniformity in specialized subtypes. Similarly, expression uniformity of tumor cells could arise from their high specialization. Finally, across almost all cell types, MBM-MBM profile pairs exhibited higher correlations compared to ECM-ECM and MBM-ECM pairs (Fig. 2C). The same but less pronounced observation was made inspecting the single cell-resolved data (Suppl. Figure 3A). Tumor cells of MBM may exhibit higher expression similarity due to a more restrictive environment compared to the mainly subcutaneous ECM of the cohort.

A small set of common differentially expressed genes was observed across several cell types. Specifically, *FKBP5* (alias *FKBP51*) was shared by Tregs, stromal cells, monocytes, CD8 + /CD4 + T cells, and MDMs. FKBP5 modulates immune response via PD-L1 [63] and promotes stemness and metastatic potential in melanoma [64]. *SYTL3* and *PBX4* were common to Tregs and CD8 + T cells, while *SLA*, *SMAP2*, and *CCND3* were shared between MDM and CD8 + T cells. Notably, *CCND3* is strongly associated with cancer, with roles as a tumor suppressor, oncogene, Cancer Census gene, and in cancer signaling. Further, an involvement in melanoma was shown [65, 66]. Melanoma or tumor literature was compiled for each differentially expressed gene (Additional File 1).

Further, we tested whether the treatment status of five of the ten ECM patients influenced the analysis by performing two alternative analyses: one excluding the treated patients and another adjusting for treatment as cofactor. Both scenarios yielded very similar numbers of differentially expressed genes, as well as high correlations of log_2_-fold-changes and FDRs with those from the main analysis (Suppl. Table 2). Therefore, the subsequent analyses are based on the simpler original analysis to maximize statistical power.

In addition, we tested if the integration of all samples and cell types at once may have introduced a bias resulting in the differential expression trends that we saw. Therefore, preprocessed, non-integrated data were pseudobulked and subjected to differential expression analysis. The resulting log_2_ fold-changes (Suppl. Figure 4) showed trends consistent with our main analysis: a global tendency for lower gene expression in MBM compared to ECM patients, and similarly small gene expression differences in tumor cells. This confirmed that the data integration step did not introduce a global bias.

### Expression signatures of the tumor microenvironment distinguish brain from extracranial metastases

**Fig. 3:**
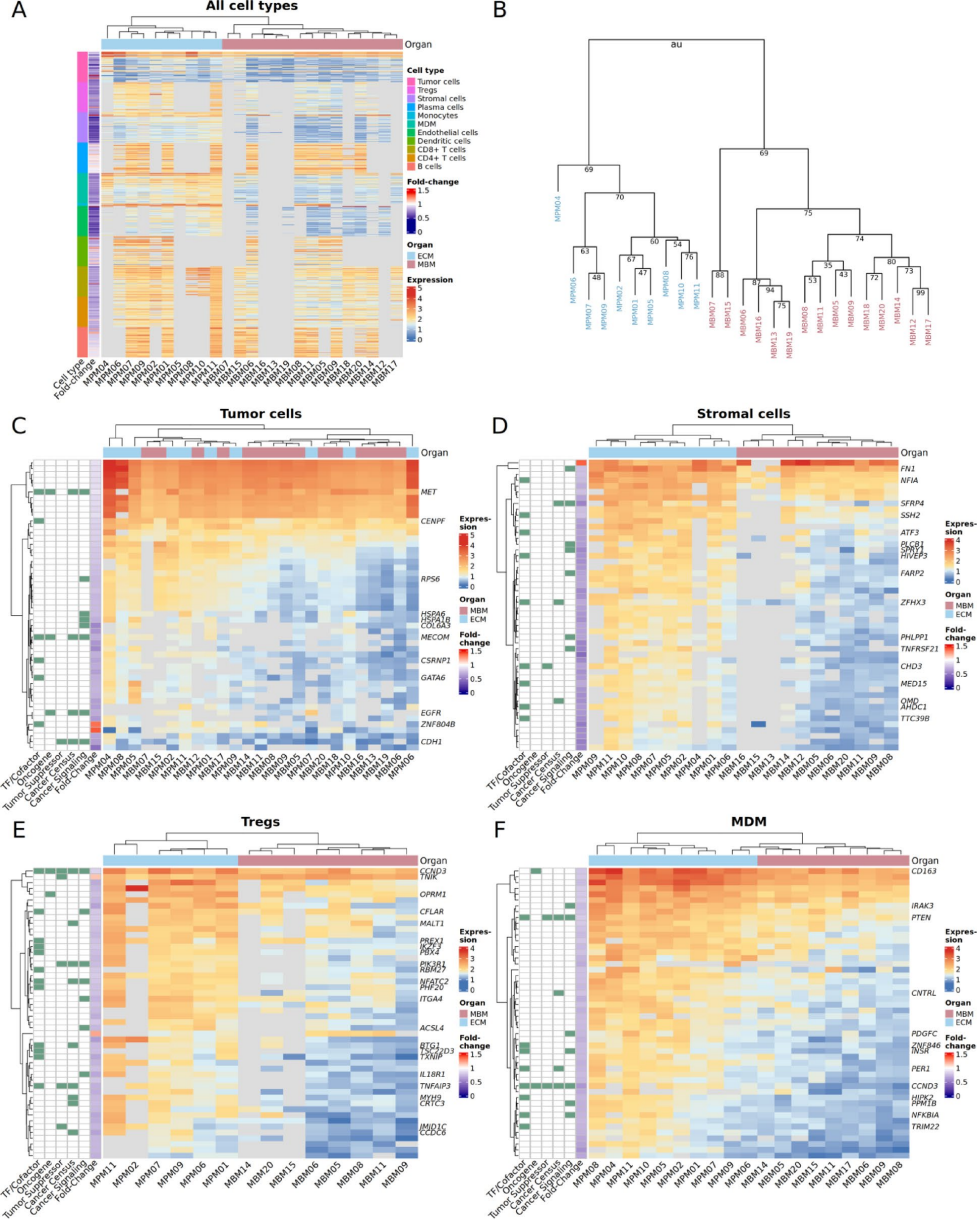
Heatmaps from hierarchical clustering of samples based on differentially expressed genes (lowest p-value) of each cell type comparing MBM and ECM. Euclidean distance and complete linkage were used. MBM and ECM samples were clearly separated by these signature genes, except for tumor cells. Gray areas represent genes not expressed or expressed in less than 10 cells. Fold changes were calculated as the ratio of average MBM to ECM gene expression. **A** Hierarchical clustering of samples using the combined set of the top 50 differentially expressed genes from each cell type. **B** Dendrogram from the performed cluster stability analysis. AU-values (blue) of subclusters were obtained by multiscale bootstrap resampling (pvclust [53]), where 100 represents a perfectly stable subcluster. Most subclusters were moderately stable. **C**–**F** Using the top 50 cell type-specific differentially expressed genes alone, MBM and ECM phenotypes were well separated, excluding tumor cells. Cancer-associated genes and their functions are highlighted

Next, we further investigated how the diverse cell types contribute to the definition of the MBM and ECM phenotypes. We performed hierarchical clustering of each cell type using the top 50 genes with the lowest p-values from the differential expression analysis. Genes that effectively separate MBM and ECM can help to understand the processes defining their phenotypes and serve as putative candidates for future intervention studies. MBM and ECM were separated perfectly using the top genes of all cell types together (Fig. 3A). We thus call these “signature genes” distinguishing the MBM and ECM phenotypes. A cluster stability analysis consistently split the top level into the MBM and ECM groups and found a moderate stability of the subclusters (Fig. 3B). Considering the top 50 genes of tumor cells alone did not separate the two phenotypes. By contrast, MBM and ECM could be perfectly distinguished by clustering only based on the top genes of their regulatory T cells, stromal cells, MDM, and endothelial cells. CD8 + and CD4 + T cells, dendritic cells, and B cells also showed very good separation capabilities (Suppl. Figure 5). This reaffirmed that cells of the metastasis microenvironment rather than tumor cells contribute to the phenotypic difference between brain and extracranial metastasis. Many of these cell type-specific MBM-ECM signature genes encode transcription factors, co-factors, oncogenes, and tumor suppressors and are involved in cancer signaling. Furthermore, many are listed in the Cancer Gene Census database as causally implicated in cancer (Fig. 3D–F, Suppl. Figures 5). Melanoma or tumor literature was compiled for each signature gene (Additional File 1).

### Enrichment analysis uncovers divergent immune and tumor processes in brain and extracranial metastases

Functional enrichment analysis was performed to further elucidate the altered biological processes between MBM and ECM. For an unbiased view and to gain new insights, a vast collection of gene sets was tested rather than focusing solely on melanoma-related pathways from literature. Enrichment of Gene Ontology (GO) molecular function and biological process terms, KEGG pathways, and WikiPathways was assessed using all genes ranked by unadjusted differential expression p-value, irrespective of their expression direction. A total of 669 terms were significantly enriched, particularly in tumor cells, regulatory T cells, CD4 + T cells, B cells, and endothelial cells (Suppl. Table 3).

**Fig. 4:**
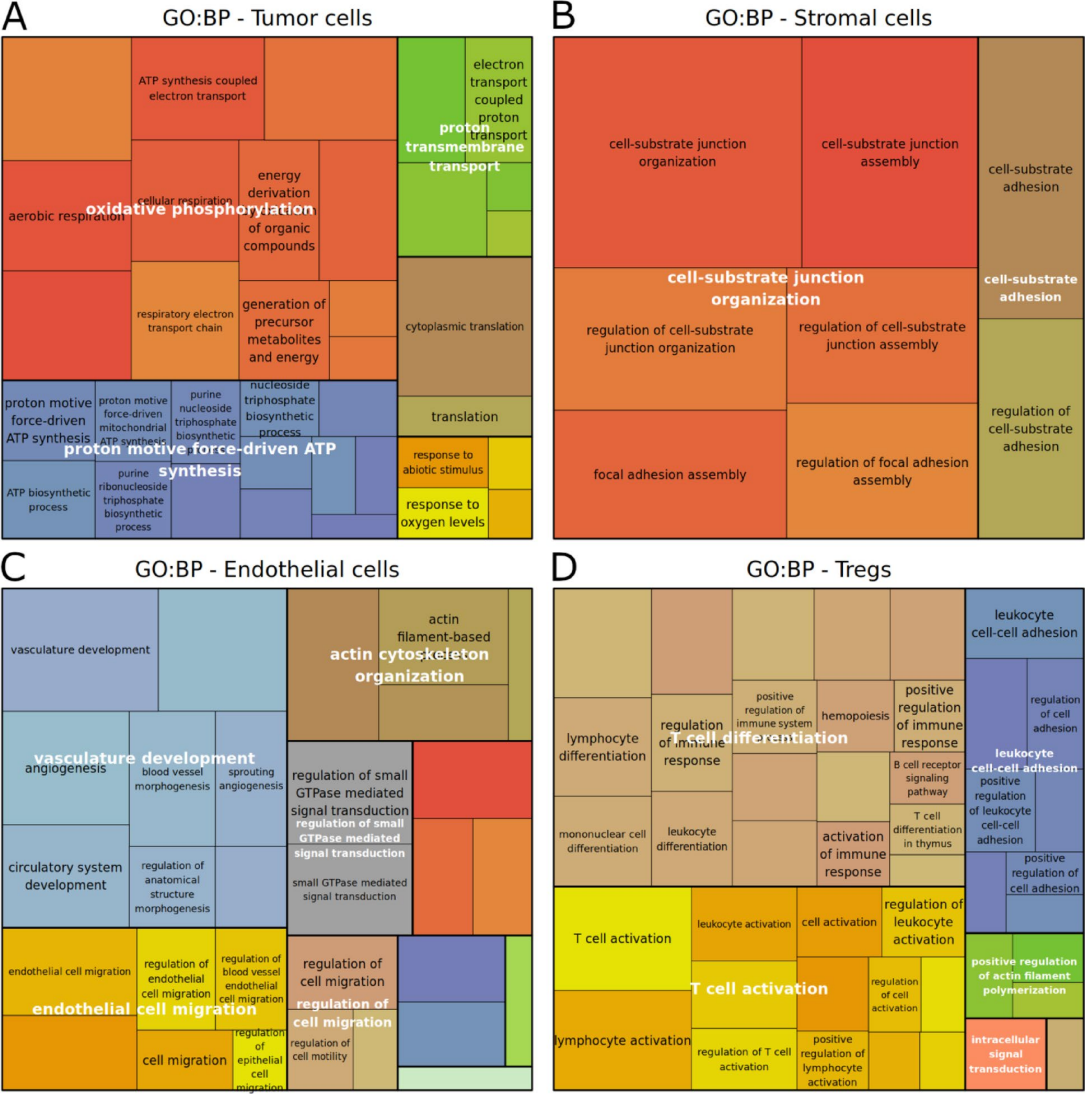
TreeMap visualization of Gene Ontology (GO) biological process (BP) terms functionally enriched in various cell types. Enrichment analysis using gprofiler2 was based on all genes ranked by differential expression p-value (MBM vs. ECM) within each cell type including up- and downregulated genes likewise. Following GO term redundancy reduction (rrvgo R package [60]), the TreeMap clustered enriched GO terms by semantic similarity, indicated by similar color shades and representative white text labels. The area size reflects the ranking score: -log_10_(adjusted p-value) from the enrichment test (g:SCS correction). The panels display results of the different cell types: tumor (**A**), stromal (**B**), endothelial (**C**), and regulatory T cells (**D**). Color match across plots is coincidental. Note, most underlying genes were more lowly expressed in MBM compared to ECM, suggesting that most pathways were also more lowly expressed (Suppl. Table 3, last sheet), indicating immune suppression on average

Enriched GO terms in tumor cells included oxidative phosphorylation, ATP synthesis, translation, and proton transmembrane transport. MAPK signaling pathway, adherens junction, and neuroinflammation were notably enriched KEGG pathways (Fig. 4A, Suppl. Figure 6A/B, Suppl. Table 3), the latter also prominent in MDM and regulatory T cells. Stromal cells showed significant enrichment in focal adhesion and cell-substrate junction/adhesion, as well as pathways crucial for cancer proliferation and migration: EGFR, PDGFR, VEGFR, and TGF beta signaling (Fig. 4B, Suppl. Figure 6E, Suppl. Table 3).

Lymphocyte lineage cells (CD8 + , CD4 + , regulatory T cells) shared enrichment in T cell receptor signaling, lymphocyte activation/differentiation, and especially Th1/Th2/Th17 cell differentiation. CD4 + T cells displayed enrichment of multiple interleukin signaling pathways. All three lymphocyte cell types showed enrichment in pathways strongly involved in immune cell response to cancer, like PD-L1/PD-1 checkpoint, ErbB, MAPK, TNF, PI3K/Akt/mTOR, EGFR, VEGF, NF-kappa B signaling. Together, this indicated a substantial difference in a broad spectrum of immune regulatory processes between MBM and ECM, encompassing proliferation, expansion, inflammation, and general immune cell communication (Fig. 4D, Suppl. Figure 6C/D/I/J, Suppl. Table 3).

Finally, enrichment in endothelial cells suggested a distinct blood vessel development in MBM compared to ECM metastases (Fig. 4C, Suppl. Figure 6F/G, Suppl. Table 3). The complete list of enriched terms is in Suppl. Table 3. No separate enrichment was performed for more highly and lowly expressed genes. However, as the vast majority of the genes within most of these enriched pathways exhibit lower expression in MBM compared to ECM patients (last sheet Suppl. Table 3), most pathways were more lowly expressed, too, indicating a rather global immune suppression in MBM.

### Celltype-specific MBM-ECM signature genes show rather global expression patterns and associate with known melanocytic phenotype signatures

**Fig. 5:**
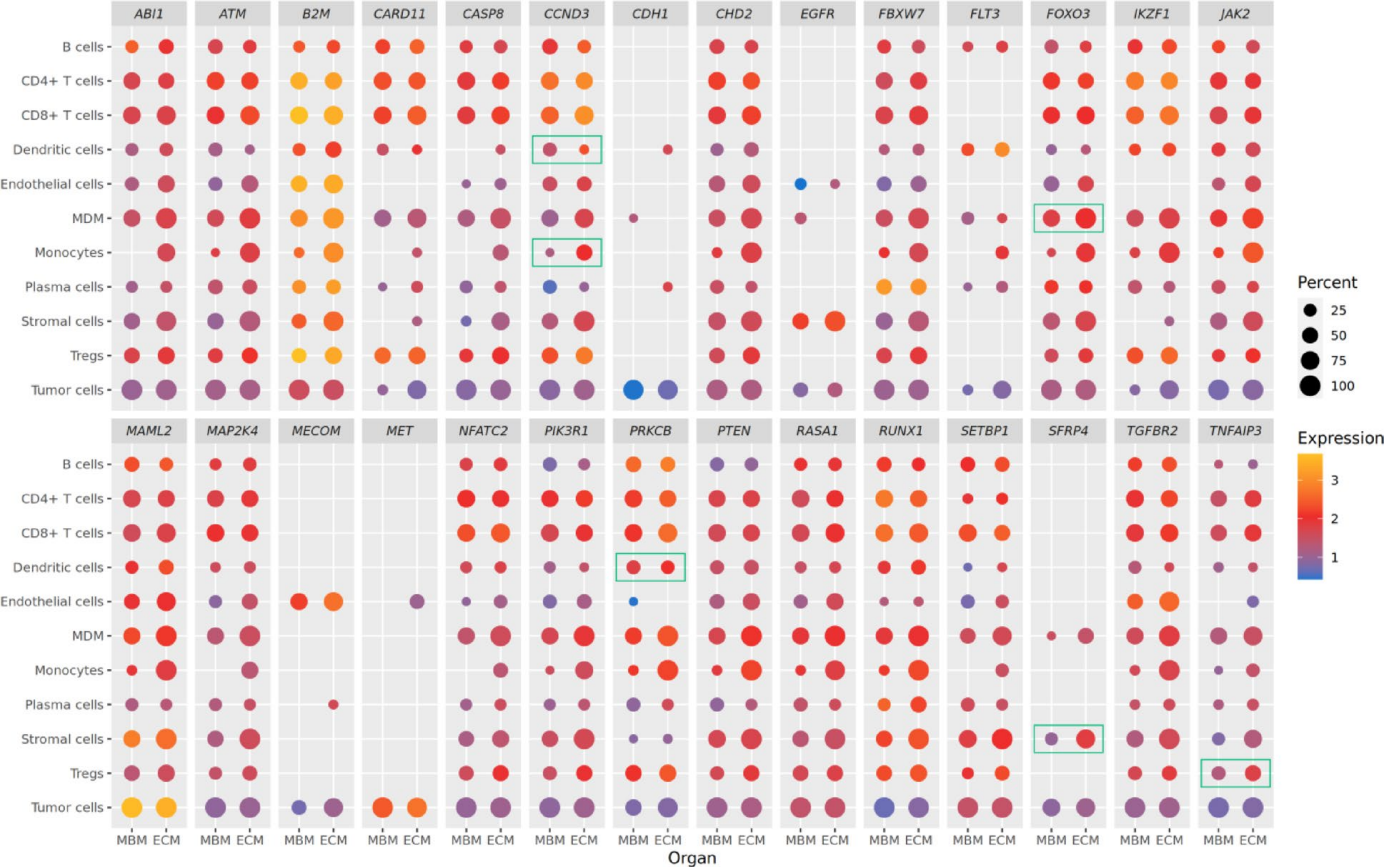
Expression patterns of cancer-related signature genes across cell types. The dotplot displays expression levels (indicated by color) of signature genes of at least one cell type (MBM vs. ECM, top 50 genes with lowest p-value) that also have at least two cancer-related annotations (oncogene, tumor suppressor, Cancer Census, cancer signaling). Circle size represents the percentage of MBM and ECM patients expressing each gene. Green boxes indicate significantly differentially expressed genes (FDR < 0.05) in the given cell type. Suppl. Figure 5 provides a more comprehensive view of signature genes with at least one cancer-related annotation

Next, we investigated the expression patterns of cancer-associated signature genes across all different cell types to characterize expression differences between cancer cells and non-cancer cells in more detail. We visualized genes with at least one association (Suppl. Figure 7) and a more stringent set with at least two associations (Fig. 5). While those genes were only differentially expressed in one or a few cell types, they were frequently expressed in most cell types in MBM and ECM patients. Notably, some genes (e.g., *MET*, *HSPA6*, *ID1*, *MECOM*, *POU2AF1*, *SFRP4*, *CDH1*) exhibited expression predominantly in tumor cells and a limited number of other cell types. We further analyzed the cancer-associated signature genes (Fig. 5), which differed in their expression between MBM and ECM for at least one cell type, with respect to the seven principal classes of gene signatures that were shown to correlate with previously identified melanocytic phenotypes [61]. This revealed positive associations with the Invasive, Neuro, AXL, Amelanotic, and Mitotic/MYC principal classes, whereas negative associations were revealed for the two principal classes Hypometabolic and Differentiated (Suppl. Figure 8).

### Comparison of candidate genes show divergent but also confirmatory trends for previous bulk RNA studies and their deconvoluted cell types

**Fig. 6:**
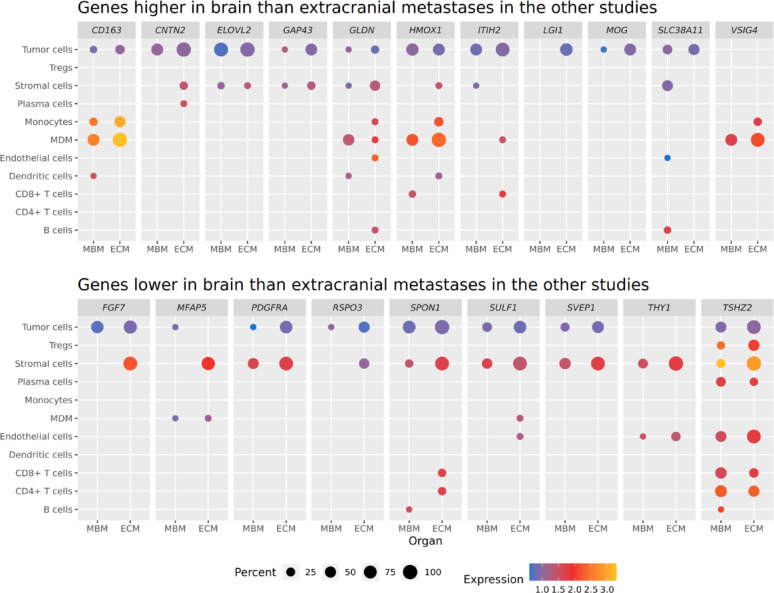
Expression patterns in aggregated single-cell data for differentially expressed genes from previous bulk transcriptome studies. The dotplot displays expression levels (indicated by color) of genes identified as differentially expressed in MBM versus ECM in at least two of three prior studies [12, 13, 15], visualized across cell types in our aggregated single-cell dataset. Expression strength is indicated by color. Circle size represents the percentage of MBM and ECM patients expressing the gene

While some previously published bulk tissue analyses offered valuable insights into melanoma metastases, they can obscure critical cell type-specific dynamics. Therefore, we revisited candidate genes from three prior bulk tissue studies [12, 13, 15] to gain a more granular understanding by examining their behavior within our cell type-resolved data. We compiled a list of 20 genes differentially expressed in MBM and ECM in at least two of the three studies that were also present in our single-cell data set. Gene expression strength and ratio of presence in MBM and ECM samples were plotted for each cell type of the single-cell data (Fig. 6). While *CD163* was a signature gene in our MDM population, its expression was lower in MDMs, tumor cells, and monocytes from brain versus extracranial metastases. Many other candidate genes were predominantly expressed in tumor but also stromal cells. Conversely, some genes (*VSIG4*, *MFAP5*, *THY1*, *TSHZ2*) showed little to no expression in tumor cells. Contrary to the bulk tissue findings, most of the genes exhibited lower expression in MBM compared to ECM in all cell types of our data. Simulating bulk expression by summing average per-cell-type expression revealed a similar trend: genes previously reported as more highly expressed in MBM showed slightly lower expression in the simulated data, while the direction was confirmed for genes with lower expression (Suppl. Figure 9, Suppl. Table 4). Vice versa, the bulk expression profiles from two of these studies [13, 15] were publicly available and therefore computationally deconvoluted using CIBERSORTx [62] to predict potentially included cell types and their specific expression profiles. The deconvolution only worked using the single-cell melanoma cell type signatures from [23] as reference, directly provided by CIBERSORTx, whereas the tool was not able to generate a basis for deconvolution from the single-cell data of our study. However, the results of CIBERSORTx confirmed a high tumor content of at least 80% for the majority of the bulk samples (Suppl. Figure 10), but an estimation of cell type-specific expression levels was only possible for few of the 20 genes (Suppl. Figure 11). For example, the increased expression of *GAP43* and reduced expression of *FGF7* in tumor cells of MBM compared to ECM (Fig. 6) were also observed in tumor cells dissected from the bulk transcriptomes from [15] (Suppl. Figure 11). Further, similar expression levels of *ITIH2*, *LGI1*, *MOG*, *SLC38A11* and *RSPO3* in tumor cells of MBM compared to ECM (Fig. 6) were also observed in the dissected tumor cells from the bulk transcriptomes from [13], but the reduced expression of *FGF7* in MBM tumor cells was not confirmed (Suppl. Figure 11). Overall, the behavior of some candidate genes from the previous bulk transcriptome studies was confirmed in their corresponding deconvoluted single-cell expression profiles. However, expression of only few genes could be predicted for different cell types, which limits the comparison and its interpretation.

## Discussion

Despite advances in diagnosis and therapy, metastatic melanoma is an ongoing challenge for patients and clinical decisions [1]. Melanoma brain metastases (MBM) exhibit lower response rates and higher recurrence compared to extracranial metastases (ECM), thus worsening overall survival [7–9]. Understanding the molecular and systemic differences between these metastatic sites is crucial to improve diagnosis and treatment. Recent single-cell studies offer a more detailed view of tumors and their microenvironment. Several studies on melanoma already exist [22–32]. Especially, Biermann et al. provided the first scRNA-seq comparison of MBM and ECM [32], yielding rich data for further exploration. Therefore, we presented here a computational reanalysis of their single-nucleus data of 15 MBM and 10 ECM patients. Informed by recent best practice recommendations, we employed a different computational approach to gain insights from a complementary perspective. Appendix 1 provides a comparison to the Methods and Results of the original study by Biermann et al. [32].

The key novel insight from our study is the minimal difference observed between MBM and ECM tumor cells. In contrast, cells of the tumor microenvironment displayed greater divergence across a broad spectrum of processes. Surprisingly, we found no significantly differentially expressed gene in tumor cells between the two metastatic sites, despite their central role in melanoma and previous reports of differences in bulk transcriptome analyses [12, 13, 15] and in the original single-cell study by Biermann et al. [32]. This might be attributed to the limited number of patients in our study and the application of recent best practice recommendations for the analysis of single-cell data. However, the original single-cell study used raw expression counts and treated every cell as independent measurement, which inflated p-values. We addressed this issue by consistently using integrated expression values and pseudobulking. Nonetheless, besides these potential technical causes, we observed that tumor cell profiles were very homogeneous among patients. The overall gene expression differences between MBM and ECM in tumor cells were small, a trend which was also notable in the original single-cell analysis [32]. The tumor environment might impose stronger selective pressures, limiting divergence of melanoma metastasis. Consequently, signature genes from tumor cells alone could not effectively separate MBM and ECM patients. Notably, tumor cell profiles showed even greater homogeneity in MBM compared to ECM patients, suggesting that the brain environment imposes even stronger selective pressure than the extracranial, mainly subcutaneous, sites. The brain is a crucial organ, protected by the blood–brain barrier, a multitude of signaling molecules, and specialized protective cells [67, 68]. Thus, tumor cells likely have to adapt even more strongly to the brain, limiting their divergence further. Previously, brain-like behavior of tumor cells was reported in bulk tissue transcriptome and methylome analyses comparing MBM with ECM [15, 19] and in breast cancer-derived brain metastases [69]. Biermann et al. reported a neuronal-like metaprogram in a subset of their single-cell MBM patient data [32]. However, this was rather not apparent in our reanalysis, where neuroinflammation, neurodegeneration, and retrograde endocannabinoid signaling were the most closely related enriched pathways.

Despite subtle gene expression differences in tumor cells, pathway analysis revealed enrichment of cancer-relevant pathways, notably an altered energy metabolism in MBM versus ECM. Specifically, oxidative phosphorylation (OxPhos) and the related aerobic respiration and response to hypoxia were prominent in MBM, consistent with prior findings in matched and unmatched MBM-ECM pairs [12]. Looking specifically into tumor cells, Biermann et al. found overall enrichment of OxPhos in MBM but also reported heterogeneity and found some ECM with strong expression [32]. Furthermore, the co-enrichment of hypoxia response in our analysis suggests potential oxygen limitations. Melanoma shows high metabolic adaptability by switching between glycolysis and OxPhos [70]. Additionally, OxPhos upregulation can mediate resistance to targeted and immune therapies [70]. MBM may be more capable of controlling energy supply, and hence, OxPhos inhibitors, which have shown efficacy in some tumors [71], could improve therapy response particularly in MBM. It must be emphasized that due to the small expression differences between MBM and ECM in tumor cells, the results from the pathway analysis are exploratory and need further confirmation through additional studies.

Unlike tumor cells, cells of the tumor microenvironment showed stronger and more consistent differences between MBM and ECM, evident in global gene expression, the number of differentially expressed genes, and expression profile heterogeneity. Notably, lymphocytes displayed substantial variation across many immune regulatory processes. There was enrichment in T cell receptor signaling and lymphocyte activation/differentiation, especially Th1, Th2, and Th17. This indicated broad differences in microenvironment interactions coordinating CD4 + T cell behavior, further supported by the many interleukin pathways differing between MBM and ECM. Regarding the potential therapeutic use of the findings, there is evidence of anti-metastatic effects of IL-9 in melanomas [72]. Secondly, IL-2 is itself an approved drug against metastatic melanoma with limited effect and toxicity, but oncolytic viruses promise improved delivery [73]. And thirdly, IL-6 signaling diverged between MBM and ECM. There is evidence that the E2F1-STAT3/IL-6 axis fosters an immunosuppressive microenvironment by promoting Th2 and inhibiting Th1 cell infiltration in primary and metastatic melanoma [74]. Furthermore, numerous immune-tumor interaction pathways (e.g., MAPK, PI3K/Akt/mTOR, ErbB/EGFR) varied between MBM and ECM. PD-L1/PD-1 checkpoint signaling indicated a diverging immune suppression between MBM and ECM, which is relevant for therapy decision. Differences in TNF alpha and NF-kappa B signaling suggested diverging inflammation grades of the metastatic micromilieus [75]. Accordingly, the “neuroinflammation” WikiPathway differed in MDM, Tregs, and tumor cells. Finally, VEGF and ErbB signaling diverged in Tregs, CD4 + T cells, and stromal cells, and pathways of blood vessel development differed in endothelial cells between MBM and ECM. This hints at angiogenesis, which could help the melanoma to further grow and metastasize. Overall, MBM and ECM exhibited different metastasis microenvironments that potentially contribute to the observed distinct T cell profiles. This underscores the need to identify individually affected pathways for personalized therapy prediction.

We also examined the expression of the signature genes (lowest differential expression p-values) with known cancer association across cell types. Although defined as signature genes for single cell types, most were expressed in MBM and ECM across all cell types. However, some genes were predominantly expressed in tumor cells and were highly relevant for melanoma. For example, higher expression of *MET* might be associated with metastatic melanoma [76]. *EGFR* and its pathway promote development and progression of several cancers [77], and it associates with poor prognosis in melanoma [78]. *CDH1* is a known invasion-suppressor gene, and its loss or abnormal expression can increase the migration capabilities of melanoma cells [79]. Other genes have known roles in non-tumor cells, as for example *CD163.* Tumor-associated CD163 + macrophages are immunosuppressive in melanoma and other tumors [80], and higher expression associates with lower overall survival in melanoma [81]. Finally, we found that the cancer-related MBM vs. ECM signature genes had positive associations with five of seven previously identified principal signature gene classes of melanocytic phenotypes (Invasive, Neuro, AXL, Amelanotic, and Mitotic/MYC) [61]. Overall, these cell type-specific signature genes are strong candidates to further study their contribution to the more aggressive brain metastases.

We found that most of the genes reported as differentially expressed in MBM compared to ECM by previously published bulk tissue analyses [12, 13, 15] were primarily expressed in tumor and stromal cells, though some had little to no tumor cell expression. *TSHZ2*, however, was expressed across many cell types. This suggests that bulk analysis can yield gene candidates not solely relevant to tumor cells. Indeed, a study on pediatric brain tumors found confounding effects on differential expression analysis due to varying proportions of specific cell type populations [82]. Underappreciation of cellular complexities and false attribution of gene expression to tumor cells are significant challenges in bulk expression studies [83]. In contrast, single-cell analysis offers more targeted, and thus promising, candidates. We observed a global tendency of lower gene expression in MBM compared to ECM, which is also in line with the trend that some of the gene candidates of previous bulk studies showed lower MBM expression. Of all candidates with higher MBM expression in the previous bulk studies, *GAP43* and *SLC38A11* showed the same behavior in our single-cell-based analysis. Computational deconvolution of two of the previous bulk studies confirmed the higher expression of *GAP43* in MBM compared to ECM in the deconvoluted tumor cells. However, cell type-specific expression deconvolution is difficult and overinterpretation should be avoided. Overall, we here generally found again hints that candidate genes of bulk studies may often not come from the targeted tumor cells themselves.

In this study, we processed single-cell data from Biermann et al. using an alternative approach informed by recent best practices for the analysis of this kind of data. While scRNA-seq provides high cellular resolution, the high rates of zero counts pose a big challenge. Integration methods that make patient samples comparable often address the problem of dropouts by imputing expression values, which we, however, chose to omit. While imputation often yields plausible values, it can reduce variance within replicates, potentially inflating downstream p-values. The second change was to pseudobulk cell type-resolved expression. This impedes, e.g., trajectory analysis. However, it aligned with our focus on the identification of cell type-specific differences between MBM and ECM. Pseudobulking approaches account better for biological replicate variability [38]. In this respect, they outperform individual cell comparisons that use methods like Wilcoxon rank-sum test [36] or MAST [37], which can inflate p-values by treating cells as independent. Biermann et al. used MAST on raw counts, resulting in long gene lists requiring strict p-value correction. Our analysis yielded shorter lists, and p-value ranks were used to define signature genes. Ultimately, the optimal single-cell analysis strategy depends on the goal of a study and different approaches can lead to complementary results. Future advances in the quality of single-cell transcriptomics and new statistical approaches will certainly help to perform differential expression analysis across thousands of cells with limited replicates.

## Conclusions

Our computational study contributed to a more accurate characterization of gene expression differences between MBM and ECM at the level of single cell types, indicating that the phenotypes are potentially more strongly influenced by variations in their metastasis microenvironments. Promising directions for future research and therapy development could involve targeting the metabolic flexibility of MBM and patient-specific immune pathway alterations.

## Supplementary Information

Below is the link to the electronic supplementary material.

Supplementary Material 1

Supplementary Material 2

Supplementary Material 3

Supplementary Material 4

Supplementary Material 5

Supplementary Material 6

## Data Availability

All data generated and analyzed in this study are included in this published article, its supplementary information or online at Zenodo (10.5281/zenodo.16981813). All original code has been deposited at GitHub (https://github.com/konradgrutz/single_cell_melanoma_metastasis, 10.5281/zenodo.17092986).

## Abbreviations

Akt: Protein kinase B
CNS: Central nervous system
E2F1: E2F Transcription Factor 1
ECM: Extracranial melanoma metastasis/metastases
EGFR: Epidermal growth factor receptor
FDR: False discovery rate
GEO: Gene Expression Omnibus
GO: Gene ontology
IL: Interleukin
KEGG: Kyoto Encyclopedia of Genes and Genomes
MAPK: Mitogen-activated protein kinase
MBM: Melanoma brain metastasis/metastases
MDM: Monocyte-derived macrophages
mTOR: Mammalian target of rapamycin
NF-kappa B: Nuclear Factor kappa-light-chain-enhancer of activated B cells
NK cells: Natural killer cells
OxPhos: Oxidative phosphorylation
PCA: Principal component analysis
PD-1: Programmed cell death protein 1
PDGFR: Platelet-derived growth factor receptor
PD-L1: Programmed death-ligand 1
PI3K: Phosphatidylinositol-3-Kinase
RPCA: Reciprocal principal component analysis
scRNA-seq: Single-cell RNA sequencing
snRNA: Single-nucleus RNA
STAT3: Signal transducer and activator of transcription 3
TGF beta: Transforming growth factor beta
Th1/Th17/Th2: T helper cell type 1/2/17
TNF: Tumor necrosis factor
Tregs: Regulatory T cells
UMAP: Uniform manifold approximation and projection
VEGF: Vascular endothelial growth factor
VEGFR: Vascular endothelial growth factor receptor
