# Supplementary material for: Single-cell transcriptome analysis suggests cells of the tumor microenvironment as a major discriminator between brain and extracranial melanoma metastases
Source: Biol Direct. 2025 Sep 16;20:97. doi: 10.1186/s13062-025-00691-2 (PMC12439397; doi:10.1186/s13062-025-00691-2)
Supplement: Supplementary file 1 — Supplementary Material 1 [file 13062_2025_691_MOESM1_ESM.html]

DEG pubmed abstr. melanoma | tumor 


**Legend**  
 Melanoma   
Gene   
Tumor | Cancer

# ABI1

**Signature gene in:** B cells
  
**Differentially expressed in:** no cell type
  
  
**ABI1**
-
1
of
5
  

### [Regulator proteins of actin dynamics as possible targets of antineoplastic therapies].

1. Pathologe. 2018 Dec;39(Suppl 2):225-230. doi: 10.1007/s00292-018-0495-x
  
  
[Article in German]
  
  
Steinestel K(1).
  
  
Author information: (1)Institut für Pathologie und Molekularpathologie, Bundeswehrkrankenhaus Ulm, Oberer Eselsberg 40, 89081, Ulm, Deutschland. konradsteinestel@bundeswehr.org.
  
  
  
  
**ABI1**
-
2
of
5
  

### Lamellipodin and the Scar/WAVE complex cooperate to promote cell migration in vivo.

2. J Cell Biol. 2013 Nov 25;203(4):673-89. doi: 10.1083/jcb.201304051 Epub 2013 Nov 18.
  
  
Law AL(1), Vehlow A, Kotini M, Dodgson L, Soong D, Theveneau E, Bodo C, Taylor E, Navarro C, Perera ...
  
  
Author information: (1)Randall Division of Cell and Molecular Biophysics, and 2 British Heart Founda ...
  
  
Cell migration is essential for development, but its deregulation causes metastasis. The Scar/WAVE complex is absolutely required for lamellipodia and is a key effector in cell migration, but its regulation in vivo is enigmatic. Lamellipodin (Lpd) controls lamellipodium formation through an unknown mechanism. Here, we report that Lpd directly binds active Rac, which regulates a direct interaction between Lpd and the Scar/WAVE complex via Abi. Consequently, Lpd controls lamellipodium size, cell migration speed, and persistence via Scar/WAVE in vitro. Moreover, Lpd knockout mice display defective pigmentation because fewer migrating neural crest-derived melanoblasts reach their target during development. Consistently, Lpd regulates mesenchymal neural crest cell migration cell autonomously in Xenopus laevis via the Scar/WAVE complex. Further, Lpd's Drosophila melanogaster orthologue Pico binds Scar, and both regulate collective epithelial border cell migration. Pico also controls directed cell protrusions of border cell clusters in a Scar-dependent manner. Taken together, Lpd is an essential, evolutionary conserved regulator of the Scar/WAVE complex during cell migration in vivo.
  
  
  
  
**ABI1**
-
3
of
5
  

### Libraries enriched for alternatively spliced exons reveal splicing patterns in melanocytes and melanomas.

3. Nat Methods. 2004 Dec;1(3):233-9. doi: 10.1038/nmeth719 Epub 2004 Nov 18.
  
  
Watahiki A(1), Waki K, Hayatsu N, Shiraki T, Kondo S, Nakamura M, Sasaki D, Arakawa T, Kawai J, Harb ...
  
  
Author information: (1)Genome Science Laboratory, RIKEN, Wako main campus, 2-1 Hirosawa, Wako, Saita ...
  
  
It is becoming increasingly clear that alternative splicing enables the complex development and homeostasis of higher organisms. To gain a better understanding of how splicing contributes to regulatory pathways, we have developed an alternative splicing library approach for the identification of alternatively spliced exons and their flanking regions by alternative splicing sequence enriched tags sequencing. Here, we have applied our approach to mouse melan-c melanocyte and B16-F10Y melanoma cell lines, in which 5,401 genes were found to be alternatively spliced. These genes include those encoding important regulatory factors such as cyclin D2, Ilk, MAPK12, MAPK14, RAB4, melastatin 1 and previously unidentified splicing events for 436 genes. Real-time PCR further identified cell line-specific exons for Tmc6, ABI1, Sorbs1, Ndel1 and Snx16. Thus, the ASL approach proved effective in identifying splicing events, which suggest that alternative splicing is important in melanoma development.
  
  
  
  
**ABI1**
-
4
of
5
  

### IRSp53/Eps8 complex is important for positive regulation of Rac and cancer cell motility/invasiveness.

4. Cancer Res. 2004 Aug 1;64(15):5237-44. doi: 10.1158/0008-5472.CAN-04-0327
  
  
Funato Y(1), Terabayashi T, Suenaga N, Seiki M, Takenawa T, Miki H.
  
  
Author information: (1)Division of Cancer Genomics, Biochemistry, and Cancer Cell Research, Institut ...
  
  
IRSp53 has been characterized as an adaptor protein that links Rho-family small GTPases, such as Rac, to reorganization of the actin cytoskeleton. Here, we search for other binding partners for the IRSp53 SH3 domain and identify Eps8 as the major binding protein in fibroblasts and various cancer cell lines. Eps8 has been shown to form a Rac-specific guanine nucleotide exchange factor complex with ABI-1 and Sos-1, which seems essential for ruffling formation induced by oncogenic Ras. We confirm the IRSp53/Eps8 complex formation in vivo and the direct association between Eps8 NH(2)-terminal proline-rich sequence and IRSp53 SH3 domain. This complex synergistically activates Rac by reinforcing the formation of the Eps8/ABI-1/Sos-1 Rac-guanine nucleotide exchange factor complex, which mediates positive regulation of Rac activity. In addition, IRSp53/Eps8 complex formation as determined by fluorescent resonance energy transfer analysis, occurs at the leading edge of motile cells, and the motility and invasiveness of HT1080 fibrosarcoma cells are suppressed by inhibiting complex formation. These findings implicate the importance of the IRSp53/Eps8 complex in Rac activation and metastatic behavior of the malignant tumor cells.
  
  
  
  
**ABI1**
-
5
of
5
  

### The Abl interactor proteins localize to sites of actin polymerization at the tips of lamellipodia and filopodia.

5. Curr Biol. 2001 Jun 5;11(11):891-5. doi: 10.1016/s0960-9822(01)00239-1
  
  
Stradal T(1), Courtney KD, Rottner K, Hahne P, Small JV, Pendergast AM.
  
  
Author information: (1)Institute of Molecular Biology, Austrian Academy of Sciences, 5020, Salzburg, ...
  
  
Comment in Nat Rev Mol Cell Biol. 2011 Jan;12(1):8. doi: 10.1038/nrm3026.
  
  
  
  
  

---

  

# ATM

**Signature gene in:** Plasma cells
  
**Differentially expressed in:** no cell type
  
  
**ATM**
-
1
of
10
  

### Phase II Study of Niraparib in Patients With Advanced Melanoma With Homologous Recombination Pathway Gene Mutations.

1. JCO Precis Oncol. 2025 May;9:e2400658. doi: 10.1200/PO-24-00658 Epub 2025 May 15.
  
  
Kim KB(1)(2), Desprez PY(2), de Semir D(2), Woo RWL(2), Sharma A(2), Jones R(2), Caressi C(2), Nosra ...
  
  
Author information: (1)Center for Melanoma Research and Treatment, Sutter California Pacific Medical ...
  
  
PURPOSE: Patients with metastatic melanoma who progress on checkpoint inhibitors and BRAF-targeting drugs have limited therapeutic options. Up to one third of melanomas harbor at least one molecular aberration in the homologous recombination (HR) pathway, leading to HR deficiency. PATIENTS AND METHODS: In this single-arm trial, we assessed the overall response rate to niraparib in patients with metastatic melanoma, harboring a genetic alteration in the HR pathway (ARID1A/B, ARID2, ATM, ATR, ATRX, BARD1, BRCA1/2, BAP1, BRIP1, CHEK2, FANCD2, MRE11A, RAD50, RAD51, RAD54B, or PALB2) who had disease progression after PD-1 blockade or BRAF/MEK inhibition if BRAF-mutant. Niraparib was administered orally at 300 mg or 200 mg daily, based on body weight and platelet count. RESULTS: Fourteen patients were accrued to the trial, which was discontinued because of slow accrual. The median age was 71 years. Nine patients had an Eastern Cooperative Oncology Group performance status of 1. Eleven patients had elevated lactate dehydrogenase levels. Ten patients had nonuveal melanoma and four had uveal melanoma. Two (14%) had a partial response and seven (50%) had stable disease, with a disease control rate of 64%. The median progression-free survival was 16 weeks. Among the patients with nonuveal melanoma, two (20%) achieved partial response with a time to progression of 32 and 24 weeks, while five (50%) had stable disease lasting 16-98 weeks. None of the four patients with uveal melanoma responded. There were no unexpected adverse events related to niraparib treatment. Notably, one responder with an ARID1A mutation had detectable circulating tumor DNA at baseline, which became undetectable during treatment. CONCLUSION: Despite the small sample size, our results indicate a promising signal for single agent niraparib in patients with pretreated nonuveal metastatic melanoma with HR gene mutations.
  
  
  
  
**ATM**
-
2
of
10
  

### Short Report: The Variants in CHEK2 in Metastatic Uveal Melanoma.

2. J Clin Med. 2025 Apr 18;14(8):2815. doi: 10.3390/jcm14082815
  
  
Terai M(1), Seedor R(1), Ashraf U(2), Hubbard G(2), Koshkin S(1), Orloff M(1), Sato T(1).
  
  
Author information: (1)Department of Medical Oncology, Sidney Kimmel Comprehensive Cancer Center at ...
  
  
Background: Uveal melanoma (UM) is a rare subtype of melanoma with distinct clinical and molecular features compared to other melanoma subtypes. UM tumors are frequently detected with mutations in GNA11, GNAQ, EIF1AX, BAP1, and SF3B1 instead of the typical mutations associated with cutaneous melanoma. Although hereditary UM is rare, germline BAP1 loss predisposes patients to UM and various other cancers. The CHEK2 (Checkpoint kinase 2) gene that encodes the protein CHK2, a serine-threonine kinase, is a cell cycle checkpoint regulator that acts as a tumor suppressor. CHK2 is involved in DNA repair, cell cycle arrest, or apoptosis in response to DNA damage. CHEK2 mutations have been linked to various cancers. While there is no strong evidence that CHEK2 mutations increase the risk of melanoma, two cases of germline CHEK2 mutations in UM patients have been reported. However, the incidence of CHEK2 variants in metastatic UM (MUM) has not been investigated. Thus, we conducted a retrospective analysis of patients with MUM and CHEK2 variants to understand this link better. Methods: We collected MUM cases from 2016 to 2024 from institutional databases. Tissues underwent analyses of molecular and genomic features, including tumor mutational burden, and were performed by a Clinically Certified Laboratory. Next-generation sequencing and variant calling were conducted to identify CHEK2 variants. Results: In this study, we reported ten patients with CHEK2 variants among 740 metastatic UM patients (1.4%) and four primary UM patients with CHEK2 germline mutations. Conclusions: Although rare, UM patients with an abnormal ATM-CHEK2 axis might receive clinical benefits from medications that target DNA repair mechanisms.
  
  
  
  
**ATM**
-
3
of
10
  

### Primary Malignant Melanoma of the Uterine Cervix with S100 (Protein Marker Seen in Women with Melanoma) Negative Status and Novel ATM Gene Mutation: Case Report and Literature Review.

3. Int J Womens Health. 2025 Apr 8;17:983-999. doi: 10.2147/IJWH.S499393 eCollection 2025.
  
  
Wang L(1).
  
  
Author information: (1)Department of Gynecology and Obstetrics, Tianjin NanKai Hospital, Tianjin, 30 ...
  
  
Malignant melanoma (MM) is a relatively common malignant tumor. It mostly occurs in the skin, uvea of the eye, oral cavity, esophagus and anus, etc. Primary melanoma of the uterine cervix is very rare, with only more than 100 cases reported worldwide so far. In this article, we report a 42-year-old patient with primary cervical malignant melanoma. This case recorded the patient's entire process from onset, surgery, progression, treatment, deterioration, and death. The patient began to seek medical treatment after the discovery of cervical vegetations and eventually died of brain metastasis. The patient was negative for S100 by immunohistochemistry and had a frameshift mutation in the Ataxia-telangiectasia mutated (ATM) gene by genetic testing. This has never been described in previous cases and is reported for the first time.
  
  
  
  
**ATM**
-
4
of
10
  

### Atypical Fibroxanthoma-Like Melanoma: A Rare Subtype of High-Cumulative Sun Damage Melanoma With Partial Dedifferentiation and an Aggressive Molecular Profile.

4. Cureus. 2025 Feb 21;17(2):e79414. doi: 10.7759/cureus.79414 eCollection 2025 Feb.
  
  
Shendrik I(1), Bobkova S(2), Oldham EP(3), Roberts J(4).
  
  
Author information: (1)Dermatopathology Section, Regional Medical Laboratory, Inc. & Pathology Labor ...
  
  
We report a rare case of atypical fibroxanthoma (AFX)-like melanoma on the scalp of a 75-year-old man. A biopsy of an erythematous, tender nodule on the left posterior parietal scalp revealed an ulcerated nodular lesion composed of epithelioid and spindle cells with pleomorphic nuclei arranged in a fascicular pattern. The tumor's architectural and cytologic features, including the presence of an epidermal collarette, marked anisonucleosis, and numerous atypical mitoses, closely resembled those of AFX. Immunohistochemical analysis demonstrated SOX10 positivity and negativity for other melanocytic markers. Molecular profiling confirmed the diagnosis of melanoma and identified mutations in the TERT promoter, NRAS, NF1, PBRM1, FAT1, and ATM genes. The tumor was categorized as Class 2B by the DecisionDx-Melanoma test (Castle Biosciences, Friendswood, Texas), indicating a high risk of recurrence and metastasis. Sentinel lymph node excision revealed metastatic melanoma in two of the five examined nodes, further supporting the aggressive biological nature of this neoplasm. This case illustrates the phenomenon of melanoma dedifferentiation, resulting in an AFX-like appearance. We discuss potential molecular mechanisms underlying this transformation, with mutations in NF1, PBRM1, and FAT1 likely contributing to the tumor's atypical morphology and loss of melanocytic markers. The high tumor mutational burden and aggressive molecular profile align with the reported poor prognosis of dedifferentiated melanomas. Recognizing this rare variant is critical for accurate diagnosis, effective patient management, and prognosis assessment.
  
  
  
  
**ATM**
-
5
of
10
  

### Germline variants in CDKN2A wild-type melanoma prone families.

5. Mol Oncol. 2025 May;19(5):1493-1507. doi: 10.1002/1878-0261.70020 Epub 2025 Mar 12.
  
  
Iversen GT(1)(2), Loeng M(3), Holth AL(3), Lønning PE(1)(2), Geisler J(3)(4), Knappskog S(1)(2).
  
  
Author information: (1)Department of Clinical Science, K.G. Jebsen Center for Genome-Directed Cancer ...
  
  
Germline pathogenic variants in CDKN2A are well established as an underlying cause of familial malignant melanoma. While pathogenic variants in other genes have also been linked to melanoma, most familial cases remain unexplained. We assessed pathogenic germline variants in 360 cancer-related genes in 56 Norwegian melanoma-prone families. The index cases were selected based on familial history of melanoma and/or multiple primary melanomas, along with previous negative tests for pathogenic CDKN2A variants. We found 6 out of 56 index individuals to carry germline pathogenic or likely pathogenic variants in BRCA2, MRE11, ATM, MSH2, CHEK2, and AR. One family member with melanoma (not index case) carried a pathogenic variant in MAP3K6. In addition, we found a high fraction of variants previously considered benign and/or as variants of uncertain significance in xeroderma pigmentosum-related genes. In particular, XPCL48F was found in 8 indexes; thus, the allele fraction (0.07) was significantly higher than in comparable healthy populations (0.02-0.03; P-values from 0.007 to 0.014). In conclusion, we found that several melanoma-prone families have pathogenic variants in genes not usually linked to melanoma.
  
  
  
  
**ATM**
-
6
of
10
  

### Genetic Landscape of Mucosal Melanoma: Identifying Pathogenic Germline Variants.

6. Pigment Cell Melanoma Res. 2025 Mar;38(2):e70007. doi: 10.1111/pcmr.70007
  
  
Ribaudo I(1), Arbesman M(1), Ni Y(1), Isaacs J(1), Kennedy LB(1), Ko J(1), Funchain P(2), Truong TG( ...
  
  
Author information: (1)Cleveland Clinic, Cleveland, Ohio, USA. (2)Stanford University, Palo Alto, Ca ...
  
  
Mucosal melanomas (MM) are rare but aggressive malignancies, comprising only 1.3% of all melanoma diagnoses, with a poor 5-year survival rate below 20%. MM lacks identifiable risk factors, presents with distinct mutational profiles, and is often diagnosed at an advanced stage, contributing to worse outcomes. This study explores the prevalence of pathogenic germline variants associated with melanoma and general cancer susceptibility in a cohort of 16 MM patients enrolled in the Gross Family Melanoma Registry at Cleveland Clinic between 2017 and 2023. Germline testing was performed using an ≥ 81 gene panel, including 12 genes with established or preliminary melanoma predisposition evidence. Our findings reveal a high prevalence (50%) of pathogenic germline variants among MM patients, with CHEK2 and APC variants identified in 12.5% of cases each, and individual variants detected in MUTYH, ATM, RB1, and RECQL4. These results suggest a germline-driven cancer susceptibility in MM, exceeding the 15% prevalence observed in cutaneous melanoma using the same inclusion criteria.
  
  
  
  
**ATM**
-
7
of
10
  

### Considerations for hereditary breast and ovarian cancer syndrome molecular diagnosis: experience from the clinical practice.

7. Breast Cancer Res Treat. 2025 Apr;210(3):507-519. doi: 10.1007/s10549-025-07643-4 Epub 2025 Feb 24.
  
  
Potrony M(1)(2), Morales-Romero B(1)(2), Moreno L(3), Pastor B(3)(4)(5), Grau E(3), Badenas C(1)(2), ...
  
  
Author information: (1)Biochemistry and Molecular Genetics Department, Hospital Clínic de Barcelona, ...
  
  
PURPOSE: The implementation of the next-generation sequencing (NGS) in clinical practice has improved the genetic diagnosis of Hereditary Breast and Ovarian Cancer Syndrome (HBOC). We aimed to evaluate the diagnostic outcomes of using an NGS cancer gene panel in clinical practice for patients selected based on personal and/or family history of breast, ovarian, prostate, melanoma, and other HBOC-associated cancers. METHODS: The study series included 2561 consecutive Spanish individuals referred for genetic testing, comprising 2445 cancer patients and 116 healthy individuals with family history of HBOC. Eleven HBOC susceptibility genes (BRCA1, BRCA2, PALB2, ATM, CHEK2, BARD1, BRIP1, RAD51C, RAD51D, TP53, and PTEN) and three Lynch Syndrome genes (MLH1, MSH2, and MSH6) available for opportunistic testing were analyzed using a commercial Hereditary Cancer Panel and an in-house bioinformatics pipeline. RESULTS: Overall, the diagnostic yield was 11.0% in cancer patients and 8.6% in healthy individuals with a family history of breast/ovarian cancer. Pathogenic variants in high-risk genes were more frequent in patients with multiple HBOC tumors and a family history of different HBOC cancers. Additionally, we diagnosed five families with Lynch syndrome through opportunistic testing. CONCLUSION: Testing cancer susceptibility genes using an agnostic strategy confers a diagnostic benefit for hereditary cancer syndromes compared to phenotype-driven test, without adding complexity to the study. The analysis of healthy individuals with a family history of HBOC detects pathogenic variants in a cost-efficient percentage of cases, resulting in a good alternative strategy when the index case is unavailable.
  
  
  
  
**ATM**
-
8
of
10
  

### PARP4 deficiency enhances sensitivity to ATM inhibitor by impairing DNA damage repair in melanoma.

8. Cell Death Discov. 2025 Jan 30;11(1):35. doi: 10.1038/s41420-025-02296-0
  
  
Li Y(#)(1), Liu Y(#)(1), Ma J(1), Yang Y(1), Yue Q(1), Zhu G(1), Guo W(1), Gao T(1), Shi Q(2), Li C( ...
  
  
Author information: (1)Department of Dermatology, Xijing Hospital, Fourth Military Medical Universit ...
  
  
Besides the important pathogenic mechanisms of melanoma, including BRAF-driven and immunosuppressive microenvironment, genomic instability and abnormal DNA double-strand breaks (DSB) repair are significant driving forces for its occurrence and development. This suggests investigating novel therapeutic strategies from the synthetic lethality perspective. Poly (ADP-ribose) polymerase 4 (PARP4) is known to be a member of the PARP protein family. The low expression of PARP4 is significantly associated with defective DSB repair markers and poor prognosis in melanoma. Further research revealed that PARP4 plays a role in DSB repair by regulating the non-homologous end joining (NHEJ) pathway through its involvement in Ku80 mono-ADP-ribosylation. Moreover, from a synthetic lethality perspective, PARP4 expression is associated with ATM inhibitor sensitivity. Overall, our study provides new and valuable insights into the function of PARP4 and melanoma pathogenesis and suggests that ATM inhibitor may be a promising therapeutic approach for treating melanoma with low PARP4 expression.
  
  
  
  
**ATM**
-
9
of
10
  

### Germline Pathogenic Variants in Patients with Pancreatic Ductal Adenocarcinoma and Extra-Pancreatic Malignancies: A Nationwide Database Analysis.

9. Mod Pathol. 2025 Jan 9;38(5):100709. doi: 10.1016/j.modpat.2025.100709 Online ahead of print.
  
  
Kryklyva V(1), Pflüger MJ(2), Ouchene H(3), Volleberg-Gorissen H(3), Mensenkamp AR(3), Jonker MA(4), ...
  
  
Author information: (1)Department of Pathology, Research Institute for Medical Innovation, Radboud U ...
  
  
Pancreatic ductal adenocarcinoma (PDAC) is a lethal disease. Approximately 10% of affected individuals have an inherited component. Deleterious germline variants increase the lifetime risk for PDAC and are often associated with an elevated risk for extra-pancreatic malignancies. In this study, we aimed to determine the prevalence and impact of germline pathogenic variants (gPVs) in patients with PDAC and extra-pancreatic malignancies. Using tissue samples and longitudinal data from a nationwide pathology database, we identified patients with PDAC and a set of 7 extra-pancreatic malignancies to investigate the presence of gPVs in 25 cancer susceptibility genes with targeted next-generation sequencing. Of 473 patients with PDAC and at least 1 extra-pancreatic malignancy, 75 (16%) had gPVs. These were predominantly in ATM (n = 22), CDKN2A (n = 14), BRCA2 (n = 10), or CHEK2 (n = 10) genes. The combination of PDAC and ovarian carcinoma carried the highest prevalence of gPVs (4 of 10; 40%), followed by PDAC and melanoma (15 of 53; 28%), and PDAC and gastric cancer (2 of 9; 22%). Patients with PDAC and certain extra-pancreatic malignancies carry a higher burden of gPVs than unselected PDAC cohorts. This is a group that very likely benefits from genetic testing because germline status can have important diagnostic and therapeutic implications for affected individuals and their family members.
  
  
  
  
**ATM**
-
10
of
10
  

### PARP inhibitors in testicular germ cell tumors: what we know and what we are looking for.

10. Front Genet. 2024 Nov 29;15:1480417. doi: 10.3389/fgene.2024.1480417 eCollection 2024.
  
  
Parola S(1), Oing C(2), Rescigno P(2), Feliciano S(1), Carlino F(1), Pompella L(1), Marretta AL(1), ...
  
  
Author information: (1)Medical Oncology Unit, Ospedale Ave Gratia Plena, ASL Caserta, San Felice a C ...
  
  
Testicular germ cell tumors (TGCTs), the most common malignancies affecting young men, are characterized by high sensitivity to cisplatin-based chemotherapy, which leads to high cure rates even in metastatic disease. However, approximately 30% of patients with metastatic TGCTs relapse after first-line treatment and those who can be defined as platinum-refractory patients face a very dismal prognosis with only limited chemotherapy-based treatment options and an overall survival of few months. Hence, to understand the mechanisms underlying cisplatin resistance is crucial for developing new treatment strategies. This narrative review explores the potential role of PARP inhibitors (PARPis) in overcoming cisplatin resistance in TGCTs, starting from the rationale of their ability to induce DNA damage in cells with homologous recombination repair (HRR). Thus far, PARPis have failed to show meaningful clinical activity in platinum-refractory TGCT patients, either alone or in combination with chemotherapy. However, few responses to PARPis in TGCTs have been detected in patients with BRCA1/2, ATM or CHEK2 mutations, reinforcing the idea that patients should be optimally selected for tailored treatments in the era of personalized medicine. Future preclinical and clinical research is needed to further investigate the molecular mechanisms of cisplatin resistance and to identify novel therapeutic strategies in resistant/refractory TGCTs patients.
  
  
  
  
  

---

  

# B2M

**Signature gene in:** Plasma cells
  
**Differentially expressed in:** no cell type
  
  
**B2M**
-
1
of
10
  

### Intratumoral IL12 mRNA administration activates innate and adaptive pathways in checkpoint inhibitor resistant tumors resulting in complete responses.

1. Res Sq [Preprint]. 2025 Apr 17:rs.3.rs-6024931. doi: 10.21203/rs.3.rs-6024931/v1
  
  
Lakshmipathi J(1), Santha S(1), Li M(1), Qian Y(1), Roy SF(1), Luheshi N(2), Politi K(1), Bosenberg ...
  
  
Author information: (1)Yale School of Medicine. (2)AstraZeneca (United Kingdom).
  
  
Despite the proven clinical activity of checkpoint inhibitors (ICIs) in several cancer indications, frequent occurrence of primary and secondary resistance reduces their overall effectiveness. Development of ICI resistance has been attributed mainly to genetic or epigenic alterations that affect the tumor antigen presentation machinery leading to diminished anti-tumor immune responses. There is an urgent need for new approaches which can either re-sensitize resistant tumors to the ICIs or engage alternate immune pathways to inhibit tumors. Intratumoral delivery of nanoparticle encapsulated murine IL-12 (mIL-12) mRNA induces powerful anti-tumor immune responses in murine tumor models and the human version of this drug results in objective responses in patients with advanced disease. Here, we tested the efficacy of mIL12 mRNA as a single agent and in combination with anti-PD-L1 antibodies in ICI sensitive Yummer1.7 melanoma and MC38 colorectal murine tumors and in ICI resistant, β2-microglobulin (B2M) knockout versions of these models. mIL12 mRNA monotherapy was sufficient to cause complete responses (CRs) in ≥ 60% of both ICI sensitive or resistant Yummer1.7 melanoma and MC38 colorectal carcinoma tumors. The mIL12 mRNA treatment resulted in potent upregulation of TH1 type cytokines and chemokines. A reduction in number of Tregs, increase in numbers and activation state of both cytotoxic T cells (CTLs) as well as tumor associated macrophages (TAMs) was observed indicating enhanced anti-tumor, cell-based immune responses in the tumor microenvironment. This mIL-12 induced concerted immune activation was associated with a robust killing and phagocytosis of tumor cells resulting in durable CRs. These observations suggest that intratumoral IL12mRNA therapy may benefit patients with ICI resistant cancers.
  
  
  
  
**B2M**
-
2
of
10
  

### Population analysis and immunologic landscape of melanoma in people living with HIV.

2. bioRxiv [Preprint]. 2025 Apr 22:2025.04.17.648995. doi: 10.1101/2025.04.17.648995
  
  
Barger LN, Wang D, Saravia A, Mezzano V, Ward G, Loomis C, Feldman C, Tuluc M, Seedor RS, Gaskill PJ ...
  
  
People living with HIV (PLWH) diagnosed with melanoma have consistently exhibited worse clinical out ...
  
  
DOI: 10.1101/2025.04.17.648995 PMCID: PMC12045344 PMID: 40313919
  
  
  
  
**B2M**
-
3
of
10
  

### Clinico-genomic features predict distinct metastatic phenotypes in cutaneous melanoma.

3. bioRxiv [Preprint]. 2025 Jan 27:2025.01.24.633441. doi: 10.1101/2025.01.24.633441
  
  
Aprati TJ(1)(2)(3), Day CP(4), Lee D(5), Pan A(5), Jee J(6), Tarantino G(1)(2)(3), Manos MP(1), Faul ...
  
  
Author information: (1)Department of Medical Oncology, Dana-Farber Cancer Institute, Boston, MA, USA ...
  
  
Metastasis drives mortality and morbidity in cancer. While some patients develop broad metastatic disease across multiple organs, others exhibit organ-specific spread. To identify mechanisms underlying metastatic organotropism, we analyzed clinico-genomic data from over 7,000 patients with metastatic cutaneous melanoma in three independent cohorts (one primary discovery and two validation cohorts including a nationwide electronic health record-derived deidentified database), leveraging machine learning approaches to clinical data. We found that female sex and increased tumor mutational burden associate with decreased metastatic potential, while older age associates with more lung and adrenal metastases. Using unsupervised analyses, patients clustered into five metastatic patterns: a "highly metastatic" cluster characterized by involvement of many organs, a "low metastatic" cluster characterized by few metastatic sites (mostly lymph node metastases), and three additional clusters each characterized by metastasis to specific sites (brain, lung, liver). Mutations in B2M and PTEN associated with increased overall metastatic potential. PTEN mutations were also associated with brain metastases but were enriched only in the "highly metastatic" cluster and not the brain-specific cluster. Mutations in GNAQ or GNA11 (GNA) associated with increased liver metastasis. To validate this association, we tested and demonstrated liver tropism in two GNA-mutant genetically engineered cutaneous melanoma mouse models of metastasis. Overall, our study elucidates distinct phenotypes of metastasis in patients with melanoma and identifies novel clinical and genomic associations that illuminate the clinical drivers of metastatic organotropism.
  
  
  
  
**B2M**
-
4
of
10
  

### Genomic mediators of acquired resistance to immunotherapy in metastatic melanoma.

4. Cancer Cell. 2025 Feb 10;43(2):308-316.e6. doi: 10.1016/j.ccell.2025.01.009
  
  
Schiantarelli J(1), Benamar M(1), Park J(1), Sax HE(2), Oliveira G(1), Bosma-Moody A(1), Campbell KM ...
  
  
Author information: (1)Department of Medical Oncology, Dana-Farber Cancer Institute, Harvard Medical ...
  
  
Although some patients with metastatic melanoma experience durable responses to immune checkpoint inhibitors (ICIs), most exhibit intrinsic or acquired resistance to these therapies. Here, we compare somatic genomic profiles from matched pre-treatment and post-resistance tumor biopsies in patients (n = 25) with metastatic melanoma who exhibited heterogeneous ICI responses to nominate additional mediators of acquired resistance. We find that several acquired resistance tumors exhibit defects in B2M or JAK1/2, consistent with prior findings. We also discover resistance-associated mutations in SEC24C and SEC24D in 3 patients. SEC24 has an essential role in the trafficking of the dsDNA sensor STING and has been linked to interferonopathies. Melanoma cells engineered to express the SEC24C mutations observed in patients exhibit diminished STING signaling, including decreased type I interferon production, antigen presentation, and a reduced capacity to activate cytotoxic T cells. This study nominates a role for aberrant STING trafficking in acquired resistance to ICIs.
  
  
  
  
**B2M**
-
5
of
10
  

### Craters on the melanoma surface facilitate tumor-immune interactions and demonstrate pathologic response to checkpoint blockade in humans.

5. bioRxiv [Preprint]. 2024 Sep 19:2024.09.18.613595. doi: 10.1101/2024.09.18.613595
  
  
Ludin A(1)(2)(3), Stirtz GL(1)(3), Tal A(1), Nirmal AJ(4)(5)(6)(7), Besson N(8), Jones SM(8), Pfaff ...
  
  
Author information: (1)Harvard Stem Cell and Regenerative Biology Department, Harvard University; Bo ...
  
  
Immunotherapy leads to cancer eradication despite the tumor's immunosuppressive environment. Here, we used extended long-term in-vivo imaging and high-resolution spatial transcriptomics of endogenous melanoma in zebrafish, and multiplex imaging of human melanoma, to identify domains that facilitate immune response during immunotherapy. We identified crater-shaped pockets at the margins of zebrafish and human melanoma, rich with beta-2 microglobulin (B2M) and antigen recognition molecules. The craters harbor the highest density of CD8+ T cells in the tumor. In zebrafish, CD8+ T cells formed prolonged interactions with melanoma cells within craters, characteristic of antigen recognition. Following immunostimulatory treatment, the craters enlarged and became the major site of activated CD8+ T cell accumulation and tumor killing that was B2M dependent. In humans, craters predicted immune response to ICB therapy, showing response better than high T cell infiltration. This marks craters as potential new diagnostic tool for immunotherapy success and targets to enhance ICB response.
  
  
  
  
**B2M**
-
6
of
10
  

### Tumor-Localized Interleukin-2 and Interleukin-12 Combine with Radiation Therapy to Safely Potentiate Regression of Advanced Malignant Melanoma in Pet Dogs.

6. Clin Cancer Res. 2024 Sep 13;30(18):4029-4043. doi: 10.1158/1078-0432.CCR-24-0861
  
  
Stinson JA(#)(1)(2), Barbosa MMP(#)(3), Sheen A(1)(2), Momin N(1)(2)(4), Fink E(1)(5), Hampel J(3), ...
  
  
Author information: (1)Koch Institute for Integrative Cancer Research, Massachusetts Institute of Te ...
  
  
Update of bioRxiv. 2024 Feb 14:2024.02.12.579965. doi: 10.1101/2024.02.12.579965.
  
  
  
  
**B2M**
-
7
of
10
  

### IFNγ mediates the resistance of tumor cells to distinct NK cell subsets.

7. J Immunother Cancer. 2024 Jul 1;12(7):e009410. doi: 10.1136/jitc-2024-009410
  
  
Hofman T(1), Ng SW(2), Garcés-Lázaro I(1), Heigwer F(2)(3), Boutros M(2), Cerwenka A(4)(5).
  
  
Author information: (1)Department of Immunobiochemistry, Mannheim Institute for Innate Immunoscience ...
  
  
BACKGROUND: Immune checkpoint blockade targeting the adaptive immune system has revolutionized the treatment of cancer. Despite impressive clinical benefits observed, patient subgroups remain non-responsive underscoring the necessity for combinational therapies harnessing additional immune cells. Natural killer (NK) cells are emerging tools for cancer therapy. However, only subpopulations of NK cells that are differentially controlled by inhibitory receptors exert reactivity against particular cancer types. How to leverage the complete anti-tumor potential of all NK cell subsets without favoring the emergence of NK cell-resistant tumor cells remains unresolved. METHODS: We performed a genome-wide CRISPR/Cas9 knockout resistance screen in melanoma cells in co-cultures with human primary NK cells. We comprehensively evaluated factors regulating tumor resistance and susceptibility by focusing on NK cell subsets in an allogenic setting. Moreover, we tested therapeutic blocking antibodies currently used in clinical trials. RESULTS: Melanoma cells deficient in antigen-presenting or the IFNγ-signaling pathways were depleted in remaining NK cell-co-cultured melanoma cells and displayed enhanced sensitivity to NK cells. Treatment with IFNγ induced potent resistance of melanoma cells to resting, IL-2-cultured and ADCC-activated NK cells that depended on B2M required for the expression of both classical and non-classical MHC-I. IFNγ-induced expression of HLA-E mediated the resistance of melanoma cells to the NKG2A+ KIR- and partially to the NKG2A+ KIR+ NK cell subset. The expression of classical MHC-I by itself was sufficient for the inhibition of the NKG2A- KIR+, but not the NKG2A+ KIR+ NK cell subset. Treatment of NK cells with monalizumab, an NKG2A blocking mAb, enhanced the reactivity of a corresponding subset of NK cells. The combination of monalizumab with lirilumab, blocking KIR2 receptors, together with DX9, blocking KIR3DL1, was required to restore cytotoxicity of all NK cell subsets against IFNγ-induced resistant tumor cells in melanoma and tumors of different origins. CONCLUSION: Our data reveal that in the context of NK cells, IFNγ induces the resistance of tumor cells by the upregulation of classical and non-classical MHC-I. Moreover, we reveal insights into NK cell subset reactivity and propose a therapeutic strategy involving combinational monalizumab/lirilumab/DX9 treatment to fully restore the antitumor response across NK cell subsets.
  
  
  
  
**B2M**
-
8
of
10
  

### Malignant features of minipig melanomas prior to spontaneous regression.

8. Sci Rep. 2024 Apr 22;14(1):9240. doi: 10.1038/s41598-024-59741-w
  
  
Débare H(1), Blanc F(1), Piton G(1)(2)(3), Leplat JJ(1)(2), Vincent-Naulleau S(1)(2)(3), Rivière J(1 ...
  
  
Author information: (1)Université Paris-Saclay, INRAE, AgroParisTech, GABI, 78350, Jouy-en-Josas, Fr ...
  
  
In MeLiM minipigs, melanomas develop around birth, can metastasize, and have histopathologic characteristics similar to humans. Interestingly, MeLiM melanomas eventually regress. This favorable outcome raises the question of their malignancy, which we investigated. We clinically followed tens of tumors from onset to first signs of regression. Transcriptome analysis revealed an enrichment of all cancer hallmarks in melanomas, although no activating or suppressing somatic mutation were found in common driver genes. Analysis of tumor cell genomes revealed high mutation rates without UV signature. Canonical proliferative, survival and angiogenic pathways were detected in MeLiM tumor cells all along progression stages. Functionally, we show that MeLiM melanoma cells are capable to grow in immunocompromised mice, with serial passages and for a longer time than in MeLiM pigs. Pigs set in place an immune response during progression with dense infiltration by myeloid cells while melanoma cells are deficient in B2M expression. To conclude, our data on MeLiM melanomas reveal several malignancy characteristics. The combination of these features with the successful spontaneous regression of these tumors make it an outstanding model to study an efficient anti-tumor immune response.
  
  
  
  
**B2M**
-
9
of
10
  

### Differentially Expressed Genes Involved in Primary Resistance to Immunotherapy in Patients with Advanced-Stage Pulmonary Cancer.

9. Int J Mol Sci. 2024 Feb 8;25(4):2048. doi: 10.3390/ijms25042048
  
  
Chinchilla-Tábora LM(1), Montero JC(1)(2), Corchete LA(3), González-Morais I(1), Del Barco Morillo E ...
  
  
Author information: (1)Department of Pathology, Institute for Biomedical Research of Salamanca (IBSA ...
  
  
In the last few years, nivolumab has become the standard of care for advanced-stage lung cancer patients. Unfortunately, up to 60% of patients do not respond to this treatment. In our study, we identified variations in gene expression related to primary resistance to immunotherapy. Bronchoscopy biopsies were obtained from advanced non-small cell lung cancer (NSCLC) patients previously characterized as responders or non-responders after nivolumab treatment. Ten tumor biopsies (from three responders and seven non-responders) were analyzed by the differential expression of 760 genes using the NanoString nCounter platform. These genes are known to be involved in the response to anti-PD1/PD-L1 therapy. All the patients were treated with nivolumab. Examining the dysregulated expression of 24 genes made it possible to predict the response to nivolumab treatment. Supervised analysis of the gene expression profile (GEP) revealed that responder patients had significantly higher levels of expression of CXCL11, NT5E, KLRK1, CD3G, GZMA, IDO1, LCK, CXCL9, GNLY, ITGAL, HLA-DRB1, CXCR6, IFNG, CD8A, ITK, B2M, HLA-B, and HLA-A than did non-responder patients. In contrast, PNOC, CD19, TP73, ARG1, FCRL2, and PTGER1 genes had significantly lower expression levels than non-responder patients. These findings were validated as predictive biomarkers in an independent series of 201 patients treated with nivolumab (22 hepatocellular carcinomas, 14 non-squamous cell lung carcinomas, 5 head and neck squamous cell carcinomas, 1 ureter/renal pelvis carcinoma, 120 melanomas, 4 bladder carcinomas, 31 renal cell carcinomas, and 4 squamous cell lung carcinomas). ROC curve analysis showed that the expression levels of ITK, NT5E, ITGAL, and CD8A were the best predictors of response to nivolumab. Further, 13/24 genes showed an adverse impact on overall survival (OS) in an independent, large series of patients with NSCLC (2166 cases). In summary, we found a strong association between the global GEP of advanced NSCLC and the response to nivolumab. The classification of NSCLC patients based on GEP enabled us to identify those patients who genuinely benefited from treatment with immune checkpoint inhibitors (ICIs). We also demonstrated that abnormal expression of most of the markers comprising the genomic signature has an adverse influence on OS, making them significant markers for therapeutic decision-making. Additional prospective studies in larger series of patients are required to confirm the clinical utility of these biomarkers.
  
  
  
  
**B2M**
-
10
of
10
  

### Tumor characteristics of dissociated response to immune checkpoint inhibition in advanced melanoma.

10. Cancer Immunol Immunother. 2024 Jan 27;73(2):28. doi: 10.1007/s00262-023-03581-6
  
  
Versluis JM(1), Hoefsmit EP(2), Shehwana H(2), Dimitriadis P(2), Sanders J(3), Broeks A(4), Blank CU ...
  
  
Author information: (1)Department of Medical Oncology, Netherlands Cancer Institute, Amsterdam, The ...
  
  
INTRODUCTION: Immune checkpoint inhibition (ICI) has improved patients' outcomes in advanced melanoma, often resulting in durable response. However, not all patients have durable responses and the patients with dissociated response are a valuable subgroup to identify mechanisms of ICI resistance. METHODS: Stage IV melanoma patients treated with ICI and dissociated response were retrospectively screened for available samples containing sufficient tumor at least at two time-points. Included were one patient with metachronous regressive and progressive lesions at the same site, two patients with regressive and novel lesion at different sites, and three patients with regressive and progressive lesions at different sites. In addition, four patients with acquired resistant tumor samples without a matched second sample were included. RESULTS: In the majority of patients, the progressive tumor lesion contained higher CD8+ T cell counts/mm2 and interferon-gamma (IFNγ) signature level, but similar tumor PD-L1 expression. The tumor mutational burden levels were in 2 out 3 lesions higher compared to the corresponding regressive tumors lesion. In the acquired tumor lesions, high CD8+/mm2 and relatively high IFNγ signature levels were observed. In one patient in both the B2M and PTEN gene a stop gaining mutation and in another patient a pathogenic POLE mutation were found. CONCLUSION: Intrapatient comparison of progressive versus regressive lesions indicates no defect in tumor T cell infiltration, and in general no tumor immune exclusion were observed.
  
  
  
  
  

---

  

# CARD11

**Signature gene in:** B cells
  
**Differentially expressed in:** no cell type
  
  
**CARD11**
-
1
of
8
  

### Eight gene mutation-based polygenic hazard score as a potential predictor for immune checkpoint inhibitor therapy outcome in metastatic melanoma.

1. Front Mol Biosci. 2022 Sep 2;9:1001792. doi: 10.3389/fmolb.2022.1001792 eCollection 2022.
  
  
Zhao L(1)(2)(3), Luo T(4), Jiang J(1), Wu J(1), Zhang X(2)(3).
  
  
Author information: (1)Department of Oncology, Ruijin Hospital, Shanghai Jiao Tong University School ...
  
  
Background: Immune checkpoint inhibitor (ICI) therapies have revolutionized the treatment of metastatic cutaneous melanoma, but have only benefitted a subset of them. Gene mutations were reported to impact the ICI therapy outcomes in metastatic melanoma but have not been fully investigated. Hence, we systematically analyzed the impact of cancer-related gene mutations on the clinical outcome in metastatic melanoma patients who underwent ICI therapies. Methods: Publicly available discovery and validation cohorts (312 patients and 110 patients respectively, all the patients received ICI therapies) were included in this study. Cox proportional hazards regression analysis was used to assess the association of 468 cancer-related gene mutations with overall survival (OS) in the discovery cohort, and the polygenic hazard score (PHS) was constructed subsequently, and validated in the validation cohort. The Tumor Immune Estimation Resource (TIMER) online tools, which are based on The Cancer Genome Atlas database, were used to analyze the impact of gene mutations on tumor-infiltrated immune cells in melanoma samples. Results: We found eight gene mutations that were significantly associated with the overall survival (BAP1, CARD11, IGF1R, KMT2D, PTPRD, PTPRT, ROS1, and TERT, p < 0.05, mutation frequency >0.05). The PHS, which was based on these genes, was found to effectively discriminate the subset which benefited most from ICI therapies (HR = 1·54, 95%CI, 1.25-1.95; p < 0.001). After adjusting with age, sex, ICI regimes, and tumor mutation burden (TMB), we found that PHS was an independent predictor for the outcome of ICI therapies (adjusted HR = 1.84, 95%CI, 1.22-2.79; p = 0.004). The PHS was validated in the validation cohort (log-Rank p = 0.038). Further research found that CARD11 and PTPRD mutations were significantly associated with more tumor-infiltrated immune cells in melanoma samples. Conclusion: For the first time, we have shown that PHS can independently and effectively predict the ICI therapy outcome in metastatic melanoma, which once validated by larger research, may help the decision-making process in melanoma.
  
  
  
  
**CARD11**
-
2
of
8
  

### Analysis of melanoma tumor antigens and immune subtypes for the development of mRNA vaccine.

2. Invest New Drugs. 2022 Dec;40(6):1173-1184. doi: 10.1007/s10637-022-01290-y Epub 2022 Aug 13.
  
  
Ping H(#)(1), Yu W(#)(1), Gong X(1), Tong X(1), Lin C(1), Chen Z(1), Cai C(1), Guo K(1), Ke H(2)(3)( ...
  
  
Author information: (1)Department of Infectious Disease, Zhongnan Hospital of Wuhan University, Hube ...
  
  
Erratum in Invest New Drugs. 2022 Dec;40(6):1356. doi: 10.1007/s10637-022-01296-6.
  
  
  
  
**CARD11**
-
3
of
8
  

### CARD11 is a prognostic biomarker and correlated with immune infiltrates in uveal melanoma.

3. PLoS One. 2021 Aug 9;16(8):e0255293. doi: 10.1371/journal.pone.0255293 eCollection 2021.
  
  
Shi X(1)(2), Xia S(1), Chu Y(3), Yang N(2)(4), Zheng J(2), Chen Q(2), Fen Z(2), Jiang Y(5), Fang S(6 ...
  
  
Author information: (1)Clinical Laboratory of Integrative Medicine, The First Affiliated Hospital of ...
  
  
Uveal melanoma (UVM), the most common primary intraocular malignancy, has a high mortality because of a high propensity to metastasize. Our study analyzed prognostic value and immune-related characteristics of CARD11 in UVM, hoping to provide a potential management and research direction. The RNA-sequence data of 80 UVM patients were downloaded from The Cancer Genome Atlas database and divided them into high- and low-expression groups. We analyzed the differentially expressed genes, enrichment analyses and the infiltration of immune cells using the R package and Gene-Set Enrichment Analysis. A clinical prediction nomogram and protein-protein interaction network were constructed and the first 8 genes were considered as the hub-genes. Finally, we constructed a competing endogenous RNA (ceRNA) network by Cytoscape and analyzed the statistical data via the R software. Here we found that CARD11 expression had notable correlation with UVM clinicopathological features, which was also an independent predictor for overall survival (OS). Intriguingly, CARD11 had a positively correlation to autophagy, cellular senescence and apoptosis. Infiltration of monocytes was significantly higher in low CARD11 expression group, and infiltration of T cells regulatory was lower in the same group. Functional enrichment analyses revealed that CARD11 was positively related to T cell activation pathways and cell adhesion molecules. The expressions of hub-genes were all increased in the high CARD11 expression group and the ceRNA network showed the interaction among mRNA, miRNA and lncRNA. These findings show that high CARD11 expression in UVM is associated with poor OS, indicating that CARD11 may serve as a potential biomarker for the diagnosis and prognosis of the UVM.
  
  
  
  
**CARD11**
-
4
of
8
  

### CARD11 alteration as a candidate biomarker of skin cutaneous melanoma treated with immune checkpoint blockade.

4. Am J Transl Res. 2021 Jan 15;13(1):286-300. eCollection 2021.
  
  
Si Y(1)(2), Lin A(1), Ding W(1), Meng H(1), Luo P(1), Zhang J(1).
  
  
Author information: (1)Department of Oncology, Zhujiang Hospital, Southern Medical University Guangz ...
  
  
BACKGROUND: Immune checkpoint inhibitors (ICIs) can be problematic, including a lack of sustained clinical response, in the treatment of skin cutaneous melanoma (SKCM) patients; therefore, predictive biomarkers are urgently needed. Recently, gene mutations identified by melanoma genomic analysis have shown great predictive potential. METHODS: We collected an immunotherapy cohort and The Cancer Genome Atlas (TCGA)-SKCM cohort from published studies and tested the predictive function of the CARD11 mutation. We then further studied the association between the CARD11 mutation and tumor immunogenicity by studying related genes and pathways in the tumor microenvironment (TME). RESULTS: In the immunotherapy and TCGA-SKCM cohorts, patients with CARD11-mutant (MT) tumors had longer overall survival (OS) and a better prognosis than those with CARD11-wild-type (WT) tumors. CARD11-MT tumors had higher immunogenicity, and gene expression related to immunosuppression was significantly downregulated in CARD11-MT tumors. We found that immunosuppression-related pathways were significantly downregulated in CARD11-MT tumors, while immune activation-related pathways were significantly upregulated. Additionally, CARD11-MT tumors had more DNA damage response and repair (DDR) pathway mutations. CONCLUSIONS: CARD11 mutation is associated with longer OS and a better prognosis after ICI treatment. Therefore, the CARD11 gene can be used as a biomarker for predicting the efficacy of ICIs in SKCM patients.
  
  
  
  
**CARD11**
-
5
of
8
  

### Bcl10-controlled Malt1 paracaspase activity is key for the immune suppressive function of regulatory T cells.

5. Nat Commun. 2019 May 28;10(1):2352. doi: 10.1038/s41467-019-10203-2
  
  
Rosenbaum M(1)(2), Gewies A(1)(3)(4), Pechloff K(1)(2)(3), Heuser C(2)(5)(6)(7), Engleitner T(2)(8), ...
  
  
Author information: (1)Institute of Clinical Chemistry and Pathobiochemistry, TUM School of Medicine ...
  
  
Regulatory T cells (Tregs) have crucial functions in the inhibition of immune responses. Their development and suppressive functions are controlled by the T cell receptor (TCR), but the TCR signaling mechanisms that mediate these effects remain ill-defined. Here we show that CARD11-BCL10-MALT1 (CBM) signaling mediates TCR-induced NF-κB activation in Tregs and controls the conversion of resting Tregs to effector Tregs under homeostatic conditions. However, in inflammatory milieus, cytokines can bypass the CBM requirement for this differentiation step. By contrast, CBM signaling, in a MALT1 protease-dependent manner, is essential for mediating the suppressive function of Tregs. In malignant melanoma models, acute genetic blockade of BCL10 signaling selectively in Tregs or pharmacological MALT1 inhibition enhances anti-tumor immune responses. Together, our data uncover a segregation of Treg differentiation and suppressive function at the CBM complex level, and provide a rationale to explore MALT1 inhibitors for cancer immunotherapy.
  
  
  
  
**CARD11**
-
6
of
8
  

### The Prognostic Significance of Low-Frequency Somatic Mutations in Metastatic Cutaneous Melanoma.

6. Front Oncol. 2019 Jan 4;8:584. doi: 10.3389/fonc.2018.00584 eCollection 2018.
  
  
Zhao X(1), Little P(2), Hoyle AP(1), Pegna GJ(3), Hayward MC(1), Ivanova A(1)(2), Parker JS(1), Marr ...
  
  
Author information: (1)Lineberger Comprehensive Cancer Center, The University of North Carolina at C ...
  
  
Background: Little is known about the prognostic significance of somatically mutated genes in metastatic melanoma (MM). We have employed a combined clinical and bioinformatics approach on tumor samples from cutaneous melanoma (SKCM) as part of The Cancer Genome Atlas project (TCGA) to identify mutated genes with potential clinical relevance. Methods: After limiting our DNA sequencing analysis to MM samples (n = 356) and to the CANCER CENSUS gene list, we filtered out mutations with low functional significance (snpEFF). We performed Cox analysis on 53 genes that were mutated in ≥3% of samples, and had ≥50% difference in incidence of mutations in deceased subjects versus alive subjects. Results: Four genes were potentially prognostic [RAC1, FGFR1, CARD11, CIITA; false discovery rate (FDR) < 0.2]. We identified 18 additional genes (e.g., SPEN, PDGFRB, GNAS, MAP2K1, EGFR, TSC2) that were less likely to have prognostic value (FDR < 0.4). Most somatic mutations in these 22 genes were infrequent (< 10%), associated with high somatic mutation burden, and were evenly distributed across all exons, except for RAC1 and MAP2K1. Mutations in only 9 of these 22 genes were also identified by RNA sequencing in >75% of the samples that exhibited corresponding DNA mutations. The low frequency, UV signature type and RNA expression of the 22 genes in MM samples were confirmed in a separate multi-institution validation cohort (n = 413). An underpowered analysis within a subset of this validation cohort with available patient follow-up (n = 224) showed that somatic mutations in SPEN and RAC1 reached borderline prognostic significance [log-rank favorable (p = 0.09) and adverse (p = 0.07), respectively]. Somatic mutations in SPEN, and to a lesser extent RAC1, were not associated with definite gene copy number or RNA expression alterations. High (>2+) nuclear plus cytoplasmic expression intensity for SPEN was associated with longer melanoma-specific overall survival (OS) compared to lower (≤ 2+) nuclear intensity (p = 0.048). We conclude that expressed somatic mutations in infrequently mutated genes beyond the well-characterized ones (e.g., BRAF, RAS, CDKN2A, PTEN, TP53), such as RAC1 and SPEN, may have prognostic significance in MM.
  
  
  
  
**CARD11**
-
7
of
8
  

### The Role of Human Papillomaviruses and Polyomaviruses in BRAF-Inhibitor Induced Cutaneous Squamous Cell Carcinoma and Benign Squamoproliferative Lesions.

7. Front Microbiol. 2018 Aug 14;9:1806. doi: 10.3389/fmicb.2018.01806 eCollection 2018.
  
  
Purdie KJ(1), Proby CM(2), Rizvi H(3), Griffin H(4), Doorbar J(4), Sommerlad M(5), Feltkamp MC(6), d ...
  
  
Author information: (1)Centre for Cell Biology and Cutaneous Research, Blizard Institute, Barts and ...
  
  
Background: Human papillomavirus (HPV) has long been proposed as a cofactor in the pathogenesis of cutaneous squamous cell carcinoma (cSCC). More recently, the striking clinico-pathological features of cSCCs that complicate treatment of metastatic melanoma with inhibitors targeting BRAF mutations (BRAFi) has prompted speculation concerning a pathogenic role for oncogenic viruses. Here, we investigate HPV and human polyomaviruses (HPyV) and correlate with clinical, histologic, and genetic features in BRAFi-associated cSCC. Materials and Methods: Patients receiving BRAFi treatment were recruited at Barts Health NHS Trust. HPV DNA was detected in microdissected frozen samples using reverse line probe technology and degenerate and nested PCR. HPV immunohistochemistry was performed in a subset of samples. Quantitative PCR was performed to determine the presence and viral load of HPyVs with affinity for the skin (HPyV6, HPyV7, HPyV9, MCPyV, and TSPyV). These data were correlated with previous genetic mutational analysis of H, K and NRAS, NOTCH1/2, TP53, CDKN2A, CARD11, CREBBP, TGFBR1/2. Chromosomal aberrations were profiled using single nucleotide polymorphism (SNP) arrays. Results: Forty-five skin lesions from seven patients treated with single agent vemurafenib in 2012-2013 were analyzed: 12 cSCC, 19 viral warts (VW), 2 actinic keratosis (AK), 5 verrucous keratosis/other squamoproliferative (VK/SP) lesions, one melanocytic lesion and 6 normal skin samples. Significant histologic features of viral infection were seen in 10/12 (83%) cSCC. HPV DNA was detected in 18/19 (95%) VW/SP, 9/12 (75%) cSCC, 4/5 (80%) SP, and 3/6 (50%) normal skin samples and in 1/12 cases assessed by immunohistochemistry. HPyV was co-detected in 22/30 (73%) of samples, usually at low viral load, with MCPyV and HPyV7 the most common. SNP arrays confirmed low levels of chromosomal abnormality and there was no significant correlation between HPV or HPyV detection and individual gene mutations or overall mutational burden. Conclusion: Despite supportive clinicopathologic evidence, the role for HPV and HPyV infection in the pathogenesis of BRAFi-induced squamoproliferative lesions remains uncertain. Synergistic oncogenic mechanisms are plausible although speculative. Nonetheless, with the prospect of a significant increase in the adjuvant use of these drugs, further research is justified and may provide insight into the pathogenesis of other BRAFi-associated malignancies.
  
  
  
  
**CARD11**
-
8
of
8
  

### Mechanisms of NKT cell anergy induction involve Cbl-b-promoted monoubiquitination of CARMA1.

8. Proc Natl Acad Sci U S A. 2009 Oct 20;106(42):17847-51. doi: 10.1073/pnas.0904078106 Epub 2009 Oct 7.
  
  
Kojo S(1), Elly C, Harada Y, Langdon WY, Kronenberg M, Liu YC.
  
  
Author information: (1)La Jolla Institute for Allergy and Immunology, La Jolla, CA 92037, USA.
  
  
Repeated injection of alpha-galactosylceramide, an agonistic ligand for natural killer T (NKT) cells, results in long-term unresponsiveness or anergy, which severely limits its clinical application. However, the molecular mechanisms leading to NKT anergy induction remain unclear. We show here that the decreased IFN-gamma production and failed tumor rejection observed in anergized NKT cells are rescued by Cbl-b deficiency. Cbl-b E3 ligase activity is critical for the anergy induction, as revealed by the similarity between Cbl-b(-/-) and its RING finger mutant NKT cells. Cbl-b binds and promotes monoubiquitination to CARMA1, a critical signaling molecule in NFkappaB activation. Ubiquitin conjugation to CARMA1 disrupts its complex formation with Bcl10 without affecting its protein stability. In addition, CARMA1(-/-) NKT cells are defective in IFN-gamma production. The study identifies an important signaling pathway linking Cbl-b-induced monoubiquitination to NFkappaB activation in NKT cell anergy induction, which may help design approaches for human cancer therapy.
  
  
  
  
  

---

  

# CASP8

**Signature gene in:** CD4+ T cells
  
**Differentially expressed in:** no cell type
  
  
**CASP8**
-
1
of
10
  

### Novel Variants in Medium and Low Penetrance Predisposing Genes in a Hungarian Malignant Melanoma Cohort With Increased Risk.

1. Pigment Cell Melanoma Res. 2025 Jan;38(1):e13214. doi: 10.1111/pcmr.13214 Epub 2024 Nov 28.
  
  
Bokor BA(1), Abdolreza A(1)(2), Kaptás F(1), Pál M(1)(2), Battyani Z(3), Széll M(1)(2), Nagy N(1)(2) ...
  
  
Author information: (1)Department of Medical Genetics, University of Szeged, Szeged, Hungary. (2)HUN ...
  
  
Both germline and somatic variants contribute to the genetic background and pathogenesis of melanoma. Germline variants include the presence of rare pathogenic or likely pathogenic variants of high, medium, and low penetrance melanoma-predisposing genes. Rare variants of high penetrance melanoma-predisposing genes are associated with melanoma development, whereas the medium and low penetrance predisposing genes can significantly increase melanoma risk. In this study, we clarified the germline genetic background of a Hungarian melanoma cohort (n = 17). Using a gene panel of 30 melanoma-predisposing genes, germline genetic variants were identified in 10 of the 17 patients (58.82%). A novel, likely pathogenic, missense variant (p.Y143C) in a medium penetrance melanoma-predisposing gene, melanocortin 1 receptor gene (MC1R), and two novel, likely pathogenic nonsense variants in low penetrance genes, p.Q218Ter in caspase 8 (CASP8) and p.Q40Ter in the fat mass- and obesity-associated (FTO) gene were detected. This study highlights the importance of elucidating the germline genetic background of melanoma, which may improve prediction of individual risk and the risk of family members and to optimize preventive, screening, and therapeutic measures for each patient and melanoma-prone families.
  
  
  
  
**CASP8**
-
2
of
10
  

### Multi-regional genomic and transcriptomic characterization of a melanoma-associated oral cavity cancer provide evidence for CASP8 alteration-mediated field cancerization.

2. Hum Genomics. 2024 Sep 7;18(1):96. doi: 10.1186/s40246-024-00668-8
  
  
Chakravarty S(1)(2), Ghosh A(1)(2), Das C(1), Das S(1), Patra S(1), Maitra A(1), Ghose S(3), Biswas ...
  
  
Author information: (1)Biotechnology Research and Innovation Council, National Institute of Biomedic ...
  
  
BACKGROUND: Precancerous and malignant tumours arise within the oral cavity from a predisposed "field" of epithelial cells upon exposure to carcinogenic stimulus. This phenomenon is known as "Field Cancerization". The molecular genomic and transcriptomic alterations that lead to field cancerization and tumour progression is unknown in Indian Oral squamous cell carcinoma (OSCC) patients. METHODS: We have performed whole exome sequencing, copy-number variation array and whole transcriptome sequencing from five tumours and dysplastic lesions (sampled from distinct anatomical subsites - one each from buccal anterior and posterior alveolus, dorsum of tongue-mucosal melanoma, lip and left buccal mucosa) and blood from a rare OSCC patient with field cancerization. RESULTS: A missense CASP8 gene mutation (p.S375F) was observed to be the initiating event in oral tumour field development. APOBEC mutation signatures, arm-level copy number alterations, depletion of CD8 + T cells and activated NK cells and enrichment of pro-inflammatory mast cells were features of early-originating tumours. Pharmacological inhibition of CASP8 protein in a CASP8-wild type OSCC cell line showed enhanced levels of cellular migration and viability. CONCLUSION: CASP8 alterations are the earliest driving events in oral field carcinogenesis, whereas additional somatic mutational, copy number and transcriptomic alterations ultimately lead to OSCC tumour formation and progression.
  
  
  
  
**CASP8**
-
3
of
10
  

### Ibrutinib Modulates Proliferation, Migration, Mitochondrial Homeostasis, and Apoptosis in Melanoma Cells.

3. Biomedicines. 2024 May 4;12(5):1012. doi: 10.3390/biomedicines12051012
  
  
Lins FV(1)(2), Bispo ECI(1), Rodrigues NS(1), Silva MVS(3), Carvalho JL(3), Gelfuso GM(4), Saldanha- ...
  
  
Author information: (1)Laboratório de Hematologia e Células-Tronco, Faculdade de Ciências da Saúde, ...
  
  
Ibrutinib, a tyrosine kinase inhibitor with a broad spectrum of action, has been successfully explored to treat hematological and solid cancers. Herein, we investigated the anti-cancer effect of Ibrutinib on melanoma cell lines. Cytotoxicity was evaluated using the MTT assay. Apoptosis, mitochondrial membrane potential, reactive oxygen species (ROS) production, cell proliferation, and cell cycle stages were determined by flow cytometry. LDH release and Caspase 3/7 activity were determined by colorimetric and luminescent assays, respectively. Cell migration was evaluated by wound scratch assay. Gene expression was determined by real-time PCR. Gene Ontology (GO) enrichment analysis of melanoma clinical samples was performed using the Database for Annotation, Visualization and Integrated Discovery (DAVID). MTT assays showed that Ibrutinib is toxic for MeWo, SK-MEL-28, and WM164 cells. The annexin V/PI staining, Caspase 3/7 activity, and LDH release in MeWo cells revealed that apoptosis is the primary mechanism of death caused by Ibrutinib. Corroborating such observation, we identified that Ibrutinib treatment impairs the mitochondrial membrane potential of such cells and significantly increases the transcriptional levels of the pro-apoptotic factors ATM, HRK, BAX, BAK, CASP3, and CASP8. Furthermore, Ibrutinib showed antimetastatic potential by inhibiting the migration of MeWo cells. Finally, we performed a functional enrichment analysis and identified that the differential expression of Ibrutinib-target molecules is associated with enrichment of apoptosis and necrosis pathways in melanoma samples. Taken together, our results clearly suggest that Ibrutinib can be successfully explored as an effective therapeutic approach for melanomas.
  
  
  
  
**CASP8**
-
4
of
10
  

### Transcriptome-Based Treatment for Melanoma With Brain Metastasis: A Case Report.

4. Cureus. 2024 Mar 19;16(3):e56494. doi: 10.7759/cureus.56494 eCollection 2024 Mar.
  
  
Ayass MA(1)(2), Melendez K(3), Griko N(4), Zhang J(4), Abi-Mosleh L(4).
  
  
Author information: (1)Respiratory Medicine/Internal Medicine, Pulmonology, Nephrology, Ayass Biosci ...
  
  
Malignant melanoma with brain metastasis has a high mortality rate. New approaches for diagnosis and treatment are urgently required to improve prognosis. Here we present a 60-year-old male with metastatic melanoma to the brain. Using a transcriptomics pipeline, we analyzed whole blood and resected tumor tissue, identifying enriched gene expression biomarkers and pathways - including seven upregulated ( BRAF, CDK4, EIF1AX, IK, NRAS, PIK3R2, and TP53) and 11 downregulated (CASP8, CDK10, CDKN2A, CTLA4, GNA11, HERC2, IRF4, MC1R, PLA2G6, RREB1, and TPCN2) genes in the blood (across 15 pathways), showing 14% enrichment, and 16 upregulated (CCND1, CDK4, CTLA4, EIF1AX, IK, IRF4, MITF, NRAS, PIK3CB, PIK3R2, PMEL, RREB1, SLC45A2, SOX10, TYR, and TYRP1) and three downregulated ( GNA11, KITLG, and PLA2G6) genes in tissue (across 17 pathways), showing 33% enrichment, with five shared markers and 12 shared pathways. The model connected CDK4 pathway overactivity observed in both samples to inhibitors like ribociclib, abemaciclib, and palbociclib as putative treatments. By enabling objective personalized therapy selection, this approach shows great promise for advancing patient outcomes.
  
  
  
  
**CASP8**
-
5
of
10
  

### TNF and IFNγ-induced cell death requires IRF1 and ELAVL1 to promote CASP8 expression.

5. J Cell Biol. 2024 Mar 4;223(3):e202305026. doi: 10.1083/jcb.202305026 Epub 2024 Feb 6.
  
  
Deng B(#)(1), Wang J(#)(1), Yang T(#)(1), Deng Z(#)(1), Yuan J(1), Zhang B(1), Zhou Z(1), Chen F(1), ...
  
  
Author information: (1)State Key Laboratory of Molecular Developmental Biology, Institute of Genetic ...
  
  
Comment in J Cell Biol. 2024 Mar 4;223(3):e202401127. doi: 10.1083/jcb.202401127.
  
  
  
  
**CASP8**
-
6
of
10
  

### Does Boric Acid Inhibit Cell Proliferation on MCF-7 and MDA-MB-231 Cells in Monolayer and Spheroid Cultures by Using Apoptosis Pathways?

6. Biol Trace Elem Res. 2024 May;202(5):2008-2021. doi: 10.1007/s12011-023-03810-0 Epub 2023 Aug 12.
  
  
Bayram D(1), Özgöçmen M(2), Çelik DA(3), Sarman E(4), Sevimli M(2).
  
  
Author information: (1)Department of Histology and Embryology, Faculty of Medicine, Süleyman Demirel ...
  
  
Most breast cancers originate in the lobules or ducts of the breast. Breast cancer as the second main cause of death among women in the world is the most common kind of cancer in women. Studies have been conducted to find the optimal treatment for breast cancer. Moreover, the therapeutic effects of different drugs and substances on this disease have been intensively researched. Boric acid accounts for 96% of the boron content in body fluids, and its derivatives are absorbed by the human body. It is assumed to be represented as (B(OH)2). Experimental studies have shown a reduction of cell proliferation and stimulation of apoptosis in some melanoma, prostate, and colon cancer cell lines through boric acid. The aim of this study was to investigate if boric acid could be used for treating breast cancer. The impacts of boric acid on the human breast carcinoma cell lines MCF-7 and MDA-MB-231 were studied with TUNEL, BrdU, caspase-3, and endo-G immunohistochemical studies in 3D and 2D culture systems. Furthermore, we conducted a qRT-PCR study to show changes in the expression of some genes involved in apoptosis. Suppression of cell proliferation through boric acid-inducing apoptosis was observed both in 3D and 2D culture conditions. These results are compatible with the gene expression results. The ENDOG, CASP3, CASP8, and CASP9 gene expression significantly changed at all time intervals in MCF-7 and MD-MB-231 cell lines boric acid can potentially treat breast cancer as an anti-cancer agent candidate.
  
  
  
  
**CASP8**
-
7
of
10
  

### Immune regulator IRF1 contributes to ZBP1-, AIM2-, RIPK1-, and NLRP12-PANoptosome activation and inflammatory cell death (PANoptosis).

7. J Biol Chem. 2023 Sep;299(9):105141. doi: 10.1016/j.jbc.2023.105141 Epub 2023 Aug 7.
  
  
Sharma BR(1), Karki R(1), Rajesh Y(1), Kanneganti TD(2).
  
  
Author information: (1)Department of Immunology, St Jude Children's Research Hospital, Memphis, Tenn ...
  
  
The innate immune system provides the first line of defense against pathogens and cellular insults and is activated by pattern recognition receptors sensing pathogen- or damage-associated molecular patterns. This activation can result in inflammation via cytokine release as well as the induction of lytic regulated cell death (RCD). Innate immune signaling can also induce the expression of interferon regulatory factor 1 (IRF1), an important molecule in regulating downstream inflammation and cell death. While IRF1 has been shown to modulate some RCD pathways, a comprehensive evaluation of its role in inflammatory cell death pathways is lacking. Here, we examined the role of IRF1 in cell death during inflammasome and PANoptosome activation using live cell imaging, Western blotting, and ELISA in primary murine macrophages. IRF1 contributed to the induction of ZBP1- (Z-DNA binding protein 1), AIM2- (absent in melanoma-2), RIPK1- (receptor interacting protein kinase 1), and NLRP12 (NOD-like receptor family, pyrin domain-containing 12)-PANoptosome activation and PANoptosis. Furthermore, IRF1 regulated the cell death under conditions where inflammasomes, along with caspase-8 and RIPK3, act as integral components of PANoptosomes to drive PANoptosis. However, it was dispensable for other inflammasomes that form independent of the PANoptosome to drive pyroptosis. Overall, these findings define IRF1 as an upstream regulator of PANoptosis and suggest that modulating the activation of molecules in the IRF1 pathway could be used as a strategy to treat inflammatory and infectious diseases associated with aberrant inflammatory cell death.
  
  
  
  
**CASP8**
-
8
of
10
  

### Rosmarinic acid decreases viability, inhibits migration and modulates expression of apoptosis-related CASP8/CASP3/NLRP3 genes in human metastatic melanoma cells.

8. Chem Biol Interact. 2023 Apr 25;375:110427. doi: 10.1016/j.cbi.2023.110427 Epub 2023 Feb 28.
  
  
da Silva GB(1), Manica D(1), da Silva AP(2), Marafon F(2), Moreno M(1), Bagatini MD(3).
  
  
Author information: (1)Postgraduate Program in Biomedical Sciences, Federal University of Fronteira ...
  
  
Cutaneous melanoma is the most aggressive type of skin cancer; it is difficult to treat, and has been highlighted in recent years due to increasing numbers of cases worldwide. The use of antitumoral therapeutics for this neoplasm has been associated with severe side effects, low quality of life, and resistance. We aimed in this study to explore the effect of the phenolic compound rosmarinic acid (RA) on human metastatic melanoma cells. SK-MEL-28 melanoma cells were treated for 24 h with different concentrations of RA. In parallel, peripheral blood mononuclear cells (PBMCs) also were treated with RA under the same experimental conditions to verify the cytotoxic effect on non-tumoral cells. Then, we assessed cell viability and migration, levels of intracellular and extracellular reactive oxygen species (ROS), as well as nitric oxide (NOx), non-protein thiols (NPSH), and total thiol (PSH). Gene expression of the caspase 8, caspase 3 and NLRP3 inflammasome was evaluated by RT-qPCR. The enzymatic activity of the caspase 3 protein was assessed by a sensitive fluorescent assay. Fluorescence microscopy was employed to corroborate the effects of RA on melanoma cell viability, mitochondria transmembrane potential and apoptotic bodies formation. We found that RA potently reduces melanoma cell viability and migration after 24 h of treatment. On the other hand, it has no cytotoxic effect on non-tumoral cells. The fluorescence micrographics indicated that RA reduces transmembrane potential of mitochondria and induces apoptotic bodies formation. Moreover, RA significantly decreases intracellular and extracellular ROS levels, and increases the antioxidant defenders NPSH and PSH. A remarkable feature found in our study was that RA strongly upregulates the gene expression of the caspase 8 and caspase 3, and downregulates NLRP3 inflammasome expression. Similar to gene expression, RA greatly increases the enzymatic activity of caspase 3 protein. Taken together, we have shown for the first time that RA reduces cell viability and migration of human metastatic melanoma cells, in addition to modulates apoptosis-related gene expression. We suggest that RA may have the potential to be used in a therapeutic perspective, particularly for CM cell treatment.
  
  
  
  
**CASP8**
-
9
of
10
  

### Pancancer transcriptomic profiling identifies key PANoptosis markers as therapeutic targets for oncology.

9. NAR Cancer. 2022 Nov 1;4(4):zcac033. doi: 10.1093/narcan/zcac033 eCollection 2022 Dec.
  
  
Mall R(1), Bynigeri RR(1), Karki R(1), Malireddi RKS(1), Sharma BR(1), Kanneganti TD(1).
  
  
Author information: (1)Department of Immunology, St. Jude Children's Research Hospital, Memphis, TN  ...
  
  
Resistance to programmed cell death (PCD) is a hallmark of cancer. While some PCD components are prognostic in cancer, the roles of many molecules can be masked by redundancies and crosstalks between PCD pathways, impeding the development of targeted therapeutics. Recent studies characterizing these redundancies have identified PANoptosis, a unique innate immune-mediated inflammatory PCD pathway that integrates components from other PCD pathways. Here, we designed a systematic computational framework to determine the pancancer clinical significance of PANoptosis and identify targetable biomarkers. We found that high expression of PANoptosis genes was detrimental in low grade glioma (LGG) and kidney renal cell carcinoma (KIRC). ZBP1, ADAR, CASP2, CASP3, CASP4, CASP8 and GSDMD expression consistently had negative effects on prognosis in LGG across multiple survival models, while AIM2, CASP3, CASP4 and TNFRSF10 expression had negative effects for KIRC. Conversely, high expression of PANoptosis genes was beneficial in skin cutaneous melanoma (SKCM), with ZBP1, NLRP1, CASP8 and GSDMD expression consistently having positive prognostic effects. As a therapeutic proof-of-concept, we treated melanoma cells with combination therapy that activates ZBP1 and showed that this treatment induced PANoptosis. Overall, through our systematic framework, we identified and validated key innate immune biomarkers from PANoptosis which can be targeted to improve patient outcomes in cancers.
  
  
  
  
**CASP8**
-
10
of
10
  

### Sialidase NEU1 May Serve as a Potential Biomarker of Proliferation, Migration and Prognosis in Melanoma.

10. World J Oncol. 2022 Aug;13(4):222-234. doi: 10.14740/wjon1509 Epub 2022 Aug 23.
  
  
Peng Q(1)(2), Gao L(3)(2), Cheng HB(4)(5)(2), Wang JS(4)(6), Wang J(4)(7).
  
  
Author information: (1)Hunan Cancer Hospital and the Affiliated Cancer Hospital of Xiangya School of ...
  
  
BACKGROUND: Melanoma is a kind of malignant tumor with high mortality originating from melanocytes. It is urgent to find new molecular biomarkers for prognosis and new treatment methods for melanoma. As an important molecule of sialidase family, neuraminidase-1 (NEU1) has been found to play an important role in regulating the occurrence and progression of tumors, but the role of NEU1 in melanoma is not sure. METHODS: The expression level of NEU1 in melanoma and normal tissues was evaluated by analyzing the expression data from ONCOMINE, UALCAN and GEPIA database. The mutation, copy number alteration and gene correlation of NEU1 in melanoma were evaluated by analyzing the melanoma data from cBioPortal database The protein expression levels of NEU1 were further validated by immunohistochemical (IHC) staining data from The Human Protein Atlas database. The melanoma data in TIMER 2.0 database were used to analyze the correlation between NEU1 expression and immune cell infiltration. The proliferative and migratory abilities of melanoma cells were examined by cell proliferation and migration assay in vitro and nude mice. RESULTS: We discovered that NEU1 was highly expressed in melanoma samples compared with normal samples. The alteration frequency of NEU1 in melanoma patients reached 18%, and most of them were "mutation" type. The expression of NEU1 was positively correlated with the overall survival of patients with melanoma. The expression of NEU1 was positively correlated with the expression of proliferation marker CDK2 and epithelial-mesenchymal transition marker CD44 and negatively correlated with the expression of apoptosis marker CASP3 and CASP8. Moreover, the expression level of NEU1 was related to the infiltration of immune cells in melanoma. Knockdown of NEU1 attenuated the in vitro proliferative and migratory abilities of melanoma cells, as well as in vivo tumor progression of melanoma cells. CONCLUSIONS: These findings suggest that NEU1 may play a key role in the development of melanoma and may be used as a prognostic target of melanoma.
  
  
  
  
  

---

  

# CD74

**Signature gene in:** B cells, Dendritic cells
  
**Differentially expressed in:** no cell type
  
  
**CD74**
-
1
of
10
  

### Characterizing spatial immune architecture in metastatic melanoma using high-dimensional multiplex imaging.

1. Front Immunol. 2025 Apr 29;16:1560778. doi: 10.3389/fimmu.2025.1560778 eCollection 2025.
  
  
Eliason J(1), Krishnan S(1), Fukuda Y(2), Bustos MA(3), Winkowski D(4), Cho S(5), Basi A(5)(6), Bair ...
  
  
Author information: (1)Department of Computational Medicine and Bioinformatics, University of Michig ...
  
  
INTRODUCTION: Immune checkpoint inhibitors (ICIs) have significantly improved survival for patients with metastatic melanoma, yet many experienceresistance due to immunosuppressive mechanisms within the tumor immune microenvironment (TIME). Understanding how the spatial architecture of immune and inflammatory components changes across disease stages may reveal novel prognostic biomarkers and therapeutic targets. METHODS: We performed high-dimensional spatial profiling of two melanoma tissue microarrays (TMAs), representing Stage III (n = 157) and Stage IV (n = 248) metastatic tumors. Using imaging mass cytometry (IMC) and multiplex immunofluorescence (mIF), we characterized the phenotypic, functional, and spatial properties of the TIME. Cellular neighborhoods were defined by inflammatory marker expression, and spatial interactions between immune and tumor cells were quantified using nearest-neighbor functions (G-cross). Associations with survival were assessed using Cox proportional hazards models with robust variance estimation. RESULTS: Stage IV tumors exhibited a distinct immune landscape, with increased CD74- and MIF-enriched inflammatory neighborhoods and reduced iNOS-associated regions compared to Stage III. Cytotoxic T lymphocytes (CTLs) and tumor cells were more prevalent in Stage IV TIME, while B cells and NK cells were depleted. Spatial analysis revealed that CTL-Th cell, NK-T cell, and B-NK cell interactions were linked to improved survival, whereas macrophage aggregation and excessive B-Th cell clustering in inflammatory regions correlated with worse outcomes. Organ-specific analyses showed that CTL infiltration near tumor cells predicted survival in gastrointestinal metastases, while NK-T cell interactions were prognostic in lymph node and skin metastases. DISCUSSION: Our results reveal stage-specific shifts in immune composition and spatial organization within the melanoma TIME. In advanced disease, immunosuppressive neighborhoods emerge alongside changes in immune cell localization, with spatial patterns of immune coordination-particularly involving CTLs, NK cells, and B cells-strongly predicting survival. These findings highlight spatial biomarkers that may refine patient stratification and guide combination immunotherapy strategies targeting the inflammatory architecture of the TIME.
  
  
  
  
**CD74**
-
2
of
10
  

### Transcriptomic signatures in peripheral CD4(+)T-lymphocytes may reflect melanoma staging and immunotherapy responsiveness prior to ICI initiation.

2. Front Immunol. 2025 Mar 28;16:1529707. doi: 10.3389/fimmu.2025.1529707 eCollection 2025.
  
  
Palli E(#)(1), Lavigne M(#)(2), Verginis P(2), Alissafi T(3), Anastasopoulou A(1), Lyrarakis G(1), K ...
  
  
Author information: (1)First Department of Internal Medicine, Laikon General Hospital, National and ...
  
  
BACKGROUND AND PURPOSE: Promoting adaptive immunity with ICIs has drastically improved melanoma prognosis, but not for all patients. Some cases relapse in the first few months, while others keep durable benefit, even after immunotherapy discontinuation. To identify cellular/molecular signatures in peripheral blood that could differentiate advanced from metastatic melanoma and predict dynamics for primary/secondary immune escape, we examined 100 consecutive patients with stage III/IV melanoma scheduled to start ICIs. MATERIALS AND METHODS: At melanoma diagnosis, a multiparameter flow cytometric analysis and purification scheme using standard conjugated antibodies were performed for all individuals prior to ICI initiation. In each stage(III/IV) according to their RFS/PFS, we retrospectively selected the cases with the clearest clinical outcomes and focused our analysis on the extreme responders(n=7) and non-responders(n=7) to characterize the transcriptomes of circulating CD4+T-cells by bulk RNA-seq, Differential Expression Analysis(DEA)and Gene Ontology(GO)enrichment analysis. Based on our selected patient cohort, we examined for differentially expressed genes(DEGs)and key-pathways that appear preferentially activated in stage III vs. IV melanoma, and in long vs. short immunotherapy responders. RESULTS: Although circulating immune-cells did not numerically differ in both sets of analysis(staging and ICI responsiveness), DEA and GO data showed that patients could be clustered separately, identifying 189vs.92 DEGs in stage IV/III and 101vs.47 DEGs in early progressors/long responders. These DEGs were functionally implicated in distinct pathways. For metastatic cases: inflammatory response(logp-value=-9.2:ADGRE5/2,CYBA,GRN,HMOX1,IRF5,ITGAM), adaptive immunity(logp-value=-7.7:CD1C,CD74,CYBB,NCF2,CTSA,S100A8/9,BCL3,FCER1G), T-cell activation(logp-value=-6.3:BCL3,CD1C,CD74,FCER1G,FGL2)and lipid metabolism/catabolism(logp-value=-2.5/-2.6:ARF3,GPX1,MVD,OCRL,PCCB,CTSA,PNPLA2,NAGLU,GBA2,ABHD4); while in early-progressors to ICIs: immune effector processing(logp-value=-13.7:BCL6,FGR,HLA-DQA1/DQB1,HLA-DRA,HLA-DRB1/DRB5,NKG7,SLC11A1,TYROBP,SPON2,HAVCR2),PD-1(logp-value=-10.2:HLA-DQA1/DQB1,HLA-DRA,HLA-DRB1/DRB5)and IFN signaling(logp-value=-8.5: HLA-DQA1/DQB1,HLA-DRA,HLA-DRB1/DRB5,NCAM1,IFITM3),positive regulation of T-cell activation(logp-value=-7.7:BCL6,HLA-DQA1/DQB1,HLA-DRA,HLA-DRB1/DRB5,SASH3,HAVCR2)and CD28 co-stimulation(logp-value=-10.3:HLA-DQA1/DQB1,HLA-DRA,HLA-DRB1/DRB5), supporting an immune-mediated behavior. CONCLUSIONS: Specific pathways and marker genes in the peripheral CD4+T-cells may predetermine melanoma staging and immunotherapy resistance.
  
  
  
  
**CD74**
-
3
of
10
  

### Developing a risk score using liquid biopsy biomarkers for selecting Immunotherapy responders and stratifying disease progression risk in metastatic melanoma patients.

3. J Exp Clin Cancer Res. 2025 Feb 5;44(1):40. doi: 10.1186/s13046-025-03306-w
  
  
Azzariti A(1), De Summa S(2), Marvulli TM(3), De Risi I(4), De Palma G(5), Di Fonte R(6), Fasano R(6 ...
  
  
Author information: (1)Experimental Pharmacology Laboratory, IRCCS Istituto Tumori Giovanni Paolo II ...
  
  
BACKGROUND: Despite the high response rate to PD-1 blockade therapy in metastatic melanoma (MM) patients, a significant proportion of patients do not respond. Identifying biomarkers to predict patient response is crucial, ideally through non-invasive methods such as liquid biopsy. METHODS: Soluble forms of PD1, PD-L1, LAG-3, CTLA-4, CD4, CD73, and CD74 were quantified using ELISA assay in plasma of a cohort of 110 MM patients, at baseline, to investigate possible correlations with clinical outcomes. A clinical risk prediction model was applied and validated in pilot studies. RESULTS: No biomarker showed statistically significant differences between responders and non-responders. However, high number of significant correlations were observed among certain biomarkers in non-responders. Through univariate and multivariate Cox analyses, we identified sPD-L1, sCTLA-4, sCD73, and sCD74 as independent biomarkers predicting progression-free survival and overall survival. According to ROC analysis we discovered that, except for sCD73, values of sPD-L1, sCTLA-4, and sCD74 lower than the cut-off predicted lower disease progression and reduced mortality. A comprehensive risk score for predicting progression-free survival was developed by incorporating the values ​​of the two identified independent factors, sCTLA-4 and sCD74, which significantly improved the accuracy of outcome prediction. Pilot validations highlighted the potential use of the risk score in treatment-naive individuals and long responders. CONCLUSION: In summary, risk score based on circulating sCTLA-4 and sCD74 reflects the response to immune checkpoint inhibitor (ICI) therapy in MM patients. If confirmed, through further validation, these findings could assist in recommending therapy to patients likely to experience a long-lasting response.
  
  
  
  
**CD74**
-
4
of
10
  

### Identification of feature genes and molecular mechanisms involved in cell communication in uveal melanoma through analysis of single‑cell sequencing data.

4. Oncol Lett. 2024 Aug 20;28(5):503. doi: 10.3892/ol.2024.14636 eCollection 2024 Nov.
  
  
Lyu N(1)(2)(3), Wu J(1)(2)(3), Dai Y(1)(2)(3), Fan Y(1)(2)(3), Lyu Z(4), Gu J(1)(2)(3), Cheng J(1)(2 ...
  
  
Author information: (1)Eye Institute and Department of Ophthalmology, Eye & ENT Hospital, Fudan Univ ...
  
  
Uveal melanoma (UM) is a highly metastatic cancer with resistance to immunotherapy. The present study aimed to identify novel feature genes and molecular mechanisms in UM through analysis of single-cell sequencing data. For this purpose, data were downloaded from The Cancer Genome Atlas and National Center for Biotechnology Information Gene Expression Omnibus public databases. The statistical analysis function of the CellPhoneDB software package was used to analyze the ligand-receptor relationships of the feature genes. The Metascape database was used to perform the functional annotation of notable gene sets. The randomForestSRC package and random survival forest algorithm were applied to screen feature genes. The CIBERSORT algorithm was used to analyze the RNA-sequencing data and infer the relative proportions of the 22 immune-infiltrating cell types. In vitro, small interfering RNAs were used to knockdown the expression of target genes in C918 cells. The migration capability and viability of these cells were then assessed by gap closure and Cell Counting Kit-8 assays. In total, 13 single-cell sample subtypes were clustered by t-distributed Stochastic Neighbor Embedding and annotated by the R package, SingleR, into 7 cell categories: Tissue stem cells, epithelial cells, fibroblasts, macrophages, natural killer cells, neurons and endothelial cells. The interactions in NK cells|Endothelial cells, Neurons|Endothelial cells, CD74\_APP, and SPP1\_PTGER4 were more significant than those in the other subsets. T-Box transcription factor 2, tropomyosin 4, plexin D1 (PLXND1), G protein subunit α I2 (GNAI2) and SEC14-like lipid binding 1 were identified as the feature genes in UM. These marker genes were found to be significantly enriched in pathways such as vasculature development, focal adhesion and cell adhesion molecule binding. Significant correlations were observed between key genes and immune cells as well as immune factors. Relationships were also observed between the expression levels of the key genes and multiple disease-related genes. Knockdown of PLXND1 and GNAI2 expression led to significantly lower viability and gap closure rates of C918 cells. Therefore, the results of the present study uncovered cell communication between endothelial cells and other cell types, identified innovative key genes and provided potential targets of gene therapy in UM.
  
  
  
  
**CD74**
-
5
of
10
  

### The cytoglobin-dependent transcriptome in melanoma indicates a protective function associated with oxidative stress, inflammation and cancer-associated pathways.

5. Sci Rep. 2024 Aug 6;14(1):18175. doi: 10.1038/s41598-024-69224-7
  
  
De Backer J(1)(2), Hoogewijs D(3).
  
  
Author information: (1)Section of Medicine, Department of Endocrinology, Metabolism and Cardiovascul ...
  
  
Cytoglobin (CYGB) is a member of the oxygen-binding globin superfamily. In this study we generated stable CYGB overexpressing A375 melanoma cells and performed RNA-sequencing to comprehensively explore the CYGB-dependent transcriptome. Our findings reveal that ectopic expression of CYGB dysregulated multiple cancer-associated genes, including the mTORC1 and AKT/mTOR signaling pathways, which are frequently overactivated in tumors. Moreover, several cancer-associated pathways, such as epithelial-mesenchymal transition (EMT) mediated by CSPG4, were downregulated upon CYGB overexpression. Intriguingly, ectopic expression suggested anti-inflammatory potential of CYGB, as exemplified by downregulation of key inflammasome-associated genes, including NLRP1, CASP1 and CD74, which play pivotal roles in cytokine regulation and inflammasome activation. Consistent with established globin functions, CYGB appears to be involved in redox homeostasis. Furthermore, our study indicates CYGB's association to DNA repair mechanisms and its regulation of NOX4, reinforcing its functional versatility. Additionally, multiple significantly enriched pathways in CYGB overexpressing cells were consistently dysregulated in opposite direction in CYGB depleted cells. Collectively, our RNA-sequencing based investigations illustrate the diverse functions of CYGB in melanoma cells, pointing to its putative roles in cellular protection against oxidative stress, inflammation, and cancer-associated pathways. These findings pave the way for further research into the physiological role of CYGB and its potential as a candidate therapeutic target in melanoma.
  
  
  
  
**CD74**
-
6
of
10
  

### Detection of neoplastic-immune hybrid cells with metastatic properties in uveal melanoma.

6. Biomark Res. 2024 Jul 20;12(1):67. doi: 10.1186/s40364-024-00609-6
  
  
Anderson AN(1), Conley P(2), Klocke CD(2), Sengupta SK(1), Pang A(1), Farley HC(1)(3), Gillingham AR ...
  
  
Author information: (1)Department of Cell, Developmental and Cancer Biology, Oregon Health and Scien ...
  
  
Update of Res Sq. 2023 Dec 06:rs.3.rs-3694879. doi: 10.21203/rs.3.rs-3694879/v1.
  
  
  
  
**CD74**
-
7
of
10
  

### Prognostic and therapeutic insights into MIF, DDT, and CD74 in melanoma.

7. Oncotarget. 2024 Jul 19;15:507-520. doi: 10.18632/oncotarget.28615
  
  
Valdez CN(1), Sánchez-Zuno GA(2), Osmani L(2), Ibrahim W(3), Galan A(3), Bacchiocchi A(3), Halaban R ...
  
  
Author information: (1)School of Medicine, Yale University, New Haven, CT 06520, USA. (2)Department ...
  
  
Macrophage Migration Inhibitory Factor (MIF) and its homolog D-dopachrome Tautomerase (DDT) have been implicated as drivers of tumor progression across a variety of cancers. Recent evidence suggests MIF as a therapeutic target in immune checkpoint inhibition (ICI) resistant melanomas, however clinical evidence of MIF and particularly of DDT remain limited. This retrospective study analyzed 97 patients treated at Yale for melanoma between 2002-2020. Bulk-RNA sequencing of patient tumor samples from the Skin Cancer SPORE Biorepository was used to evaluate for differential gene expression of MIF, DDT, CD74, and selected inflammatory markers, and gene expression was correlated with patient survival outcomes. Our findings revealed a strong correlation between MIF and DDT levels, with no statistically significant difference across common melanoma mutations and subtypes. Improved survival was associated with lower MIF and DDT levels and higher CD74:MIF and CD74:DDT levels. High CD74:DDT and CD74:MIF levels were also associated with enrichment of infiltrating inflammatory cell markers. These data suggest DDT as a novel target in immune therapy. Dual MIF and DDT blockade may provide synergistic responses in patients with melanoma, irrespective of common mutations, and may overcome ICI resistance. These markers may also provide prognostic value for further biomarker development.
  
  
  
  
**CD74**
-
8
of
10
  

### The role of macrophage migration inhibitory factor family and CD74 in the pathogenesis of melanoma.

8. Exp Dermatol. 2024 Jun;33(6):e15122. doi: 10.1111/exd.15122
  
  
Tanese K(1), Ogata D(2).
  
  
Author information: (1)Department of Dermatology, Toho University School of Medicine, Tokyo, Japan. ...
  
  
Melanoma is an aggressive tumour with poor prognosis that arises from the malignant transformation of melanocytes. Over the past few decades, intense research into the pathogenesis of melanoma has led to the development of BRAF and immune checkpoint inhibitors, including antibodies against programmed cell death protein 1 (PD-1) and cytotoxic T lymphocyte-associated protein 4 (CTLA-4), which have shown clinically significant efficacy. However, some tumours do not respond to these therapies initially or become treatment resistant. Most melanoma tissues appear to possess biological characteristics that allow them to evade these treatments, and identifying these characteristics is one of the major challenges facing cancer researchers. One such characteristic that has recently gained attention is the role of macrophage migration inhibitory factor (MIF) and its receptor CD74. This review outlines the cellular and molecular functions of CD74, MIF and their family of proteins. We then review their roles in tumours based on previous reports, highlight their pathological significance in melanoma and discuss their potential as therapeutic targets.
  
  
  
  
**CD74**
-
9
of
10
  

### MIF and CD74 as Emerging Biomarkers for Immune Checkpoint Blockade Therapy.

9. Cancers (Basel). 2024 May 4;16(9):1773. doi: 10.3390/cancers16091773
  
  
Fey RM(1), Nichols RA(1), Tran TT(2), Vandenbark AA(3)(4)(5), Kulkarni RP(1)(6)(7)(8).
  
  
Author information: (1)Department of Dermatology, Oregon Health & Science University, Portland, OR 9 ...
  
  
Immune checkpoint blockade (ICB) therapy is used to treat a wide range of cancers; however, some patients are at risk of developing treatment resistance and/or immune-related adverse events (irAEs). Thus, there is a great need for the identification of reliable predictive biomarkers for response and toxicity. The cytokine MIF (macrophage migration inhibitory factor) and its cognate receptor CD74 are intimately connected with cancer progression and have previously been proposed as prognostic biomarkers for patient outcome in various cancers, including solid tumors such as malignant melanoma. Here, we assess their potential as predictive biomarkers for response to ICB therapy and irAE development. We provide a brief overview of their function and roles in the context of cancer and autoimmune disease. We also review the evidence showing that MIF and CD74 may be of use as predictive biomarkers of patient response to ICB therapy and irAE development. We also highlight that careful consideration is required when assessing the potential of serum MIF levels as a biomarker due to its reported circadian expression in human plasma. Finally, we suggest future directions for the establishment of MIF and CD74 as predictive biomarkers for ICB therapy and irAE development to guide further research in this field.
  
  
  
  
**CD74**
-
10
of
10
  

### Expert Consensus on the Diagnosis and Treatment of NRG1/2 Gene Fusion Solid Tumors.

10. Glob Med Genet. 2024 Feb 27;11(1):86-99. doi: 10.1055/s-0044-1781457 eCollection 2024 Jan.
  
  
Xu C(1)(2), Wang Q(3), Wang D(2), Wang W(4), Fang W(5), Li Z(6), Liu A(7), Yu J(8), Zhong W(9), Wang ...
  
  
Author information: (1)Department of Scientific Research, Institute of Cancer and Basic Medicine, Ch ...
  
  
The fusion genes NRG1 and NRG2 , members of the epidermal growth factor (EGF) receptor family, have emerged as key drivers in cancer. Upon fusion, NRG1 retains its EGF-like active domain, binds to the ERBB ligand family, and triggers intracellular signaling cascades, promoting uncontrolled cell proliferation. The incidence of NRG1 gene fusion varies across cancer types, with lung cancer being the most prevalent at 0.19 to 0.27%. CD74 and SLC3A2 are the most frequently observed fusion partners. RNA-based next-generation sequencing is the primary method for detecting NRG1 and NRG2 gene fusions, whereas pERBB3 immunohistochemistry can serve as a rapid prescreening tool for identifying NRG1 -positive patients. Currently, there are no approved targeted drugs for NRG1 and NRG2 . Common treatment approaches involve pan-ERBB inhibitors, small molecule inhibitors targeting ERBB2 or ERBB3, and monoclonal antibodies. Given the current landscape of NRG1 and NRG2 in solid tumors, a consensus among diagnostic and treatment experts is proposed, and clinical trials hold promise for benefiting more patients with NRG1 and NRG2 gene fusion solid tumors.
  
  
  
  
  

---

  

# CDH1

**Signature gene in:** Tumor cells
  
**Differentially expressed in:** no cell type
  
  
**CDH1**
-
1
of
10
  

### Novel Risk Factors for Uveal Melanoma in Adolescent and Young Adult Patients: A Comprehensive Case-Control Analysis.

1. Ophthalmol Sci. 2024 Dec 24;5(4):100687. doi: 10.1016/j.xops.2024.100687 eCollection 2025 Jul-Aug.
  
  
Nisanova A(1)(2), Park SS(2), Amin A(1)(2), Zako C(1), Wilson MD(3), Scholey J(1), Afshar AR(4), Tsa ...
  
  
Author information: (1)Department of Radiation Oncology, University of California San Francisco, San ...
  
  
PURPOSE: To identify risk factors associated with uveal melanoma (UM) in adolescents and young adults (AYAs). DESIGN: A retrospective case-control study. PARTICIPANTS: Two hundred forty-seven UM patients aged 13 to 45 treated with proton beam radiation therapy and 401 age- and sex-matched controls at a tertiary academic center. METHODS: We obtained demographic and genetic data, environmental exposures, and social, medical, and ocular history via retrospective chart review and phone follow-up. MAIN OUTCOME MEASURES: The main outcome measures included the prevalence and odds ratios (ORs) of the investigated risk factors in UM patients compared with controls. RESULTS: The median age of UM diagnosis was 38 years (range: 13-45 years); the median follow-up was 102 months (range: 3-329 months). Identified novel risk factors for UM included family history of cutaneous melanoma (OR = 3.06, P = 0.002), Ashkenazi Jewish ancestry (2.98, P = 0.02), prior eye trauma (2.94, P = 0.01), secondhand cigarette smoke exposure (2.39, P < 0.001), and previous head and neck surgery (1.81, P = 0.007). Some known risk factors identified include choroidal nevi (11.39, P < 0.001), light eye color (4.69, P < 0.001), White race (4.63, P < 0.001), outdoor sunlight exposure (4.20, P < 0.001), recent pregnancy (4.0, P = 0.002), occupational (2.39, P = 0.003) and toxic chemical (2.27, P = 0.03) exposures, family history of any cancer (2.16, P < 0.001), lack of ultraviolet-blocking eyewear use (2.13, P = 0.01), indoor tanning (2.10, P = 0.03), and propensity to sunburn (1.89, P < 0.05). The prevalence of oculodermal melanocytosis (P = 0.03) and family history of UM (P < 0.001) were significantly greater in UM patients than in controls. Uveal melanoma T-categories were as follows: 39% T1, 37% T2, 19% T3, and 5% T4. Gene expression profiling was available in 64 patients and showed 59% class 1A, 19% class 1B, and 22% class 2 tumors. Thirteen patients underwent genetic screening; identified germline mutations included CDH1, NF1, and PALB2. The estimated 10-year metastasis-free progression rate and overall survival were 80% and 81%, respectively. CONCLUSIONS: This study identified several novel risk factors for UM in AYAs and confirmed select established risk factors seen in UM patients of all ages. To the best of our knowledge, this is the first explicit and comprehensive investigation of risk factors among a younger cohort and may help further elucidate UM pathogenesis. FINANCIAL DISCLOSURES: Proprietary or commercial disclosure may be found in the Footnotes and Disclosures at the end of this article.
  
  
  
  
**CDH1**
-
2
of
10
  

### Malignant behaviors and immune response in melanoma: Epstein-Barr virus induced gene 3 as a therapeutic target based on an in-vitro exploration.

2. PeerJ. 2024 Dec 23;12:e18730. doi: 10.7717/peerj.18730 eCollection 2024.
  
  
Zhang Y(1), Cheng F(1), Cai X(2), Wu J(1).
  
  
Author information: (1)Department of Medical Aesthetics, Affiliated Hospital of Chengdu University o ...
  
  
BACKGROUND: Epstein-Barr virus induced gene 3 (EBI3), a member of the IL-12 family, is known to be involved in malignant progression in a variety of cancers, but its role in melanoma is unclear. The aim of this study was to explore the effects of EBI3 on the malignant phenotype melanoma to reveal its potential as a therapeutic target. METHODS: In this study, we used bioinformatics to analyze the expression of EBI3 in pan-cancer and verified its expression level in melanoma cells by reverse transcription-quantitative polymerase chain reaction (RT-qPCR). Subsequently, the effects of EBI3 knockdown on cell proliferation, migration and invasion were detected using the Cell Counting Kit-8 (CCK-8) and Transwell assays. Changes in immune-related cytokines were detected by ELISA, and macrophage polarization was observed using immunofluorescence. Finally, the phosphorylation levels of signaling pathways such as Smad3, STAT6 and cGAS-STING were analyzed by Western blot. RESULTS: EBI3 was evidently highly-expressed in melanoma, and silencing of EBI3 could visibly suppress the survival and migration/invasion of melanoma cells, concurrent with the increased levels of BAX and CDH1 and the decreased expressions of BCL2 and CDH2. Meanwhile, EBI3 knockdown diminished the phosphorylation levels of both Smad3 and STAT6 and the levels of immune response-relevant cytokines in melanoma cells, while aggravating the macrophage M1 polarization and the expression of cGAS, p-STING and p-IRE1 α in THP-1 monocyte-derived macrophages co-cultured with EBI3-silenced melanoma cells. CONCLUSION: This study filled the blank on the involvement of EBI3 in melanoma, hinting the possibility of controlling EBI3 as a therapeutic strategy in the management of melanoma.
  
  
  
  
**CDH1**
-
3
of
10
  

### Multimodal tumor suppression by METTL3 gene knockdown in melanoma and colon cancer cells.

3. Histochem Cell Biol. 2024 Dec 25;163(1):21. doi: 10.1007/s00418-024-02346-1
  
  
Bazargani A(1), Taha MF(1), Soltani BM(2), Javeri A(3).
  
  
Author information: (1)Department of Stem Cells and Regenerative Medicine, Institute for Medical Bio ...
  
  
METTL3, an m6A methyltransferase, is integral to the regulation of messenger RNA (mRNA) biogenesis, degradation, and translation through the N6-methyladenosine (m6A) modification. Alterations in m6A homeostasis have been implicated in the development, progression, invasion, and metastasis of certain cancers. The present research aims to examine the consequences of METTL3 knockdown using short hairpin RNA (shRNA) on the proliferation and invasive capabilities of human colorectal and melanoma cancer cell lines. A specific shRNA against METTL3 mRNA was designed and inserted into an expression vector. Highly invasive colorectal cancer cell line SW480 and melanoma cell line A375 were cultured and transfected by METTL3-shRNA and scramble-control vectors and kept under culture condition for 2 weeks. The cells were harvested for analysis of gene expression by quantitative polymerase chain reaction (qPCR), invasion assay using three-dimensional (3D) spheroid assay and cell cycle and apoptosis analyses. In the METTL3-shRNA transfected cells, the expression of METTL3, VIM, SNAI1, SNAI2, ZEB1, CDH1, and TGFB1 genes were downregulated significantly compared with the scramble-control transfected cells. Expression of b-catenin, N-cadherin, vimentin, ZEB1, pro- and active MMP2, OCT4A, SOX2, and MYC proteins were also downregulated following METTL3 knockdown. Transfection by METTL3-shRNA reduced proliferation rate of the cells and increased the apoptotic rate significantly. Both migration and invasion rate of the cancer cells transfected with METTL3-shRNA were significantly decreased. These findings highlight the pro-oncogenic function of METTL3 in colorectal and melanoma cancer cells, indicating that inhibiting METTL3 could be a promising approach for tumor suppression across multiple cancer types; nonetheless, further investigation is essential to confirm these observations.
  
  
  
  
**CDH1**
-
4
of
10
  

### Expression of Markers Associated with Epithelial-Mesenchymal Transition and Extracellular Matrix Degradation in Human Uveal Melanoma.

4. Bull Exp Biol Med. 2024 Oct;177(6):774-779. doi: 10.1007/s10517-024-06266-x Epub 2024 Oct 23.
  
  
Shatruk AY(1), Bgatova NP(2), Yeremina AV(3), Trunov AN(3), Chernykh VV(3), Taskaeva IS(2).
  
  
Author information: (1)Research Institute of Clinical and Experimental Lymphology - Branch of the In ...
  
  
The expression of markers associated with epithelial-mesenchymal transition (EMT) and extracellular matrix degradation in human uveal melanoma tissue samples and postequatorial zone of the choroid was assessed by immunohistochemical staining. Increased expression of EMT markers E-cadherin and vimentin was observed in the tumor. The ratio of MMP-9 to TIMP-1 proteins related to the extracellular matrix degradation was higher in the tumor. These results may indicate activation of EMT-like process in the uveal melanoma cells and degradation of the extracellular matrix, which can contribute to the development of collective invasion in uveal melanoma.
  
  
  
  
**CDH1**
-
5
of
10
  

### Clinicopathological and Molecular Features of Penile Melanoma With a Proposed Staging System.

5. Am J Surg Pathol. 2024 Jul 1;48(7):825-833. doi: 10.1097/PAS.0000000000002247 Epub 2024 May 29.
  
  
Cornejo KM(1), Goyal A(2), Valencia Guerrero A(3), Astudillo M(4), Dias-Santagata D(1), Johnson MM(5 ...
  
  
Author information: (1)Department of Pathology, Massachusetts General Hospital and Harvard Medical S ...
  
  
Penile melanomas (PM) are an exceedingly rare subtype of mucosal melanoma (MM), and we reviewed the clinicopathologic features and molecular profile in 8 PMs. The patient ages ranged from 46 to 78 (mean: 62.8) years with involvement on the glans (n=5; 62.5%), penile urethra (n=2; 25%), and foreskin (n=1, 12.5%). Tumor depth ranged from 1.6 to 10.0 (mean: 5.25) mm. Most of the patients underwent partial penectomy (n=6; 75%) and sentinel lymph node (LN) biopsy N=7; 87.5%). Seven patients had metastatic disease at diagnosis, 6 involving LNs and 1 the adrenal gland, and 4 died of disease with a mean follow-up period of 40.5 (2 to 95) months. Five of 7 (71%) cases identified 15 molecular alterations within KIT , CDKN2A , NF1 , PTEN , and APC (n=2 each), and NRAS , MAP3K1 , CDH1 , MSH6 , and TERT (n=1 each). Two cases were not found to harbor genetic aberrations, and 1 case failed testing. In addition, we reviewed the English literature and included 93 cases with a reported depth of invasion and follow-up. A total of 101 PMs were analyzed for prognostic parameters, and the overall survival was significantly worse in patients with LN metastasis (P=0.0008), distant metastasis (P=0.0016), and greater depth of invasion (P=0.0222) based upon T-stage. While T4 conferred substantially worse survival, the delineation of the survival curves between T2 and T3 was less clear, and combining T2+T3 disease had a strong prognostic impact ( P =0.0024). Prognostic parameters used in the staging of cutaneous melanomas may also be used in PMs. An alternative staging system expanding the inclusion criteria for T2 might provide a more accurate prognostic stratification.
  
  
  
  
**CDH1**
-
6
of
10
  

### The effect of microRNA-9 overexpression on inhibition of melanoma cancer stem cells tumorigenicity.

6. Melanoma Res. 2024 Apr 1;34(2):105-117. doi: 10.1097/CMR.0000000000000931 Epub 2024 Jan 15.
  
  
Parisa S(1), Amirhossein I, Hamed YS, Javad F, Masoumeh A, Pardis K, Mahshad D, Faezeh K, Marzieh E.
  
  
Author information: (1)Department of Regenerative Medicine, Cell Science Research Center, Royan Inst ...
  
  
Most of the studies have reported the downregulation of miR-9 in metastatic melanomas compared to primary tumors. They indicated that miR-9 negatively regulates the epithelial-to-mesenchymal transition (EMT) by inhibiting SNAIL1 expression and consequently promotes CDH1 expression. Since the process of EMT is associated to stem cell features, it could be interesting to study the effect of miR-9 on melanoma cancer stem cells. In the present study, we examined the effects of miR-9 manipulation on the stemness potential of melanoma cells. Our data demonstrated that the overexpression of miR-9 in A375 and NA8 cells significantly inhibits the ability of proliferation, self-renewal, migration, and tumorigenicity of melanoma cells which was concomitant with changes in the level of BRAF , some EMT factors, and stemness genes. Likewise, the reduction of miR-9 levels led to an increase in cell proliferation, colony and sphere formation, and the ability of cell migration and tumorigenicity. In conclusion, our results specified the role of miR-9 as a tumor suppressor miRNA to inhibit many aspects of melanoma stem cells, and therefore, it could be a potential candidate for the suppression of melanoma growth and progression.
  
  
  
  
**CDH1**
-
7
of
10
  

### Novel cellular systems unveil mucosal melanoma initiating cells and a role for PI3K/Akt/mTOR pathway in mucosal melanoma fitness.

7. J Transl Med. 2024 Jan 8;22(1):35. doi: 10.1186/s12967-023-04784-2
  
  
Monti M(#)(1), Benerini Gatta L(#)(1)(2), Bugatti M(1), Pezzali I(1), Picinoli S(1), Manfredi M(3)(4 ...
  
  
Author information: (1)Department of Molecular and Translational Medicine, University of Brescia, Br ...
  
  
BACKGROUND: Mucosal Melanomas (MM) are highly aggressive neoplasms arising from mucosal melanocytes. Current treatments offer a limited survival benefit for patients with advanced MM; moreover, the lack of pre-clinical cellular systems has significantly limited the understanding of their immunobiology. METHODS: Five novel cell lines were obtained from patient-derived biopsies of MM arising in the sino-nasal mucosa and designated as SN-MM1-5. The morphology, ultrastructure and melanocytic identity of SN-MM cell lines were validated by transmission electron microscopy and immunohistochemistry. Moreover, in vivo tumorigenicity of SN-MM1-5 was tested by subcutaneous injection in NOD/SCID mice. Molecular characterization of SN-MM cell lines was performed by a mass-spectrometry proteomic approach, and their sensitivity to PI3K chemical inhibitor LY294002 was validated by Akt activation, measured by pAkt(Ser473) and pAkt(Thr308) in immunoblots, and MTS assay. RESULTS: This study reports the validation and functional characterization of five newly generated SN-MM cell lines. Compared to the normal counterpart, the proteomic profile of SN-MM is consistent with transformed melanocytes showing a heterogeneous degree of melanocytic differentiation and activation of cancer-related pathways. All SN-MM cell lines resulted tumorigenic in vivo and display recurrent structural variants according to aCGH analysis. Of relevance, the microscopic analysis of the corresponding xenotransplants allowed the identification of clusters of MITF-/CDH1-/CDH2 + /ZEB1 + /CD271 + cells, supporting the existence of melanoma-initiating cells also in MM, as confirmed in clinical samples. In vitro, SN-MM cell lines were sensitive to cisplatin, but not to temozolomide. Moreover, the proteomic analysis of SN-MM cell lines revealed that RICTOR, a subunit of mTORC2 complex, is the most significantly activated upstream regulator, suggesting a relevant role for the PI3K-Akt-mTOR pathway in these neoplasms. Consistently, phosphorylation of NDRG1 and Akt activation was observed in SN-MM, the latter being constitutive and sustained by PTEN loss in SN-MM2 and SN-MM3. The cell viability impairment induced by LY294002 confirmed a functional role for the PI3K-Akt-mTOR pathway in SN-MM cell lines. CONCLUSIONS: Overall, these novel and unique cellular systems represent relevant experimental tools for a better understanding of the biology of these neoplasms and, as an extension, to MM from other sites.
  
  
  
  
**CDH1**
-
8
of
10
  

### Factors Affecting Recurrence and Survival for Patients with High-Risk Stage II Melanoma.

8. Ann Surg Oncol. 2024 Apr;31(4):2713-2726. doi: 10.1245/s10434-023-14724-5 Epub 2023 Dec 29.
  
  
Dedeilia A(#)(1)(2)(3), Lwin T(4), Li S(1), Tarantino G(3)(5), Tunsiricharoengul S(6), Lawless A(7), ...
  
  
Author information: (1)Division of Gastrointestinal and Oncologic Surgery, Department of Surgery, Ma ...
  
  
BACKGROUND: In the current era of effective adjuvant therapies and de-escalation of surgery, distinguishing which patients with high-risk stage II melanoma are at increased risk of recurrence after excision of the primary lesion is essential to determining appropriate treatment and surveillance plans. METHODS: A single-center retrospective study analyzed patients with stage IIB or IIC melanoma. Demographic and tumor data were collected, and genomic analysis of formalin-fixed, paraffin-embedded tissue samples was performed via an internal next-generation sequencing (NGS) platform (SNaPshot). The end points examined were relapse-free survival (RFS), distant metastasis-free survival (DMFS), overall survival (OS), and melanoma-specific survival (MSS). Uni- and multivariable Cox regressions were performed to calculate the hazard ratios. RESULTS: The study included 92 patients with a median age of 69 years and a male/female ratio of 2:1. A Breslow depth greater than 4 mm, a higher mitotic rate, an advanced T stage, and a KIT mutation had a negative impact on RFS. A primary lesion in the head and neck, a mitotic rate exceeding 10 mitoses per mm2, a CDH1 mutation, or a KIT mutation was significantly associated with a shorter DMFS. Overall survival was significantly lower with older age at diagnosis and a higher mitotic rate. An older age at diagnosis also had a negative impact on MSS. CONCLUSION: Traditional histopathologic factors and specific tumor mutations displayed a significant correlation with disease recurrence and survival for patients with high-risk stage II melanoma. This study supported the use of genomic testing of high-risk stage II melanomas for prognostic prediction and risk stratification.
  
  
  
  
**CDH1**
-
9
of
10
  

### Screening for Mutations in Hereditary Cancer Susceptibility Genes in a Region with High Endogamy in Brazil.

9. Glob Med Genet. 2023 Dec 8;10(4):376-381. doi: 10.1055/s-0043-1777449 eCollection 2023 Dec.
  
  
Oliveira P(1), Correa P(2), Acosta A(2), Freitas J(3), Machado-Lopes T(4), Bomfim-Palma T(4), Ribeir ...
  
  
Author information: (1)Department of Biology, State University of Feira de Santana, Bahia, Brazil. ( ...
  
  
Introduction  Cancer is a multifactorial disease dependent on the influence of genetic and environmental factors. About 10% of cancers are associated with germline mutations, which predispose to a higher risk of developing cancer. Currently, the use of panels that identify susceptibility and/or association genes cancer has been increasingly used, both in clinical practice and in scientific research. Objective  To investigate genetic mutations in patients with a profile for hereditary cancer in individuals from a region of northeast Brazil, where there is a high frequency of endogenous and consanguineous marriages. Methods  A set of 17 genes ( BRCA1 , BRCA2 , APC , TP53 , PTEN , RET , VHL , RB1 , CDKN2 , CDH1 , CHEK2 , MLH1 , MSH2 , MSH6 , MUTYH , XPA , and XPC ) associated with cancer and hereditary syndromes were analyzed. Fifteen patients with a hereditary cancer profile were evaluated. Results  The pathogenic variant found was c.1187G > A (p.Gly396Asp), rs36053993 in the MUTYH gene in a male patient diagnosed with melanoma at the age of 43 years and a family history for this tumor. This gene encodes an important enzyme related to DNA repair and has been associated with other types of cancer, this is the first report of an association with melanoma, the biological plausibility of this association is given once the MUTYH protein is expressed in the skin tissue and is responsible for repairing damage caused, for example, by sun exposure. Conclusion  The results of this study suggest that this mutation may be important for the hereditary predisposition to melanoma, but a broader investigation of this mutation is needed.
  
  
  
  
**CDH1**
-
10
of
10
  

### Prostaglandin E2 Exposure Disrupts E-Cadherin/Caveolin-1-Mediated Tumor Suppression to Favor Caveolin-1-Enhanced Migration, Invasion, and Metastasis in Melanoma Models.

10. Int J Mol Sci. 2023 Nov 29;24(23):16947. doi: 10.3390/ijms242316947
  
  
Lobos-González L(1)(2), Oróstica L(3)(4), Díaz-Valdivia N(2)(3), Rojas-Celis V(2)(3), Campos A(2)(5) ...
  
  
Author information: (1)Centro de Medicina Regenerativa, Facultad de Medicina-Clínica Alemana, Univer ...
  
  
Caveolin-1 (CAV1) is a membrane-bound protein that suppresses tumor development yet also promotes metastasis. E-cadherin is important in CAV1-dependent tumor suppression and prevents CAV1-enhanced lung metastasis. Here, we used murine B16F10 and human A375 melanoma cells with low levels of endogenous CAV1 and E-cadherin to unravel how co-expression of E-cadherin modulates CAV1 function in vitro and in vivo in WT C57BL/6 or Rag-/- immunodeficient mice and how a pro-inflammatory environment generated by treating cells with prostaglandin E2 (PGE2) alters CAV1 function in the presence of E-cadherin. CAV1 expression augmented migration, invasion, and metastasis of melanoma cells, and these effects were abolished via transient co-expression of E-cadherin. Importantly, exposure of cells to PGE2 reverted the effects of E-cadherin expression and increased CAV1 phosphorylation on tyrosine-14 and metastasis. Moreover, PGE2 administration blocked the ability of the CAV1/E-cadherin complex to prevent tumor formation. Therefore, our results support the notion that PGE2 can override the tumor suppressor potential of the E-cadherin/CAV1 complex and that CAV1 released from the complex is phosphorylated on tyrosine-14 and promotes migration/invasion/metastasis. These observations provide direct evidence showing how a pro-inflammatory environment caused here via PGE2 administration can convert a potent tumor suppressor complex into a promoter of malignant cell behavior.
  
  
  
  
  

---

  

# DDX5

**Signature gene in:** Dendritic cells
  
**Differentially expressed in:** no cell type
  
  
**DDX5**
-
1
of
3
  

### Diagnostic Power of MicroRNAs in Melanoma: Integrating Machine Learning for Enhanced Accuracy and Pathway Analysis.

1. J Cell Mol Med. 2025 Jan;29(2):e70367. doi: 10.1111/jcmm.70367
  
  
Rafiepoor H(1), Ghorbankhanloo A(1), Soleimani Dorcheh S(2), Angouraj Taghavi E(1), Ghanadan A(3), S ...
  
  
Author information: (1)Cancer Biology Research Center, Cancer Institute, Tehran University of Medica ...
  
  
This study identifies microRNAs (miRNAs) with significant discriminatory power in distinguishing melanoma from nevus, notably hsa-miR-26a and hsa-miR-211, which have exhibited diagnostic potential with accuracy of 81% and 78% respectively. To enhance diagnostic accuracy, we integrated miRNAs into various machine-learning (ML) models. Incorporating miRNAs with AUC scores above 0.70 significantly improved diagnostic accuracy to 94%, with a sensitivity of 91%. These findings underscore the potential of ML models to leverage miRNA data for enhanced melanoma diagnosis. Additionally, using the miRNet tool, we constructed a network of miRNA-miRNA interactions, revealing 170 key genes in melanoma pathophysiology. Protein-protein interaction network analysis via Cytoscape identified hub genes including MYC, BRCA1, JUN, AURKB, CDKN2A, DDX5, MAPK14, DDX3X, DDX6, FOXM1 and GSK3B. The identification of hub genes and their interactions with miRNAs enhances our understanding of the molecular mechanisms driving melanoma. Pathway enrichment analyses highlighted key pathways associated with differentially expressed miRNAs, including the PI3K/AKT, TGF-beta signalling pathway and cell cycle regulation. These pathways are implicated in melanoma development and progression, reinforcing the significance of our findings. The functional enrichment of miRNAs suggests their critical role in modulating essential pathways in melanoma, suggesting their potential as therapeutic targets.
  
  
  
  
**DDX5**
-
2
of
3
  

### DEAD/DEAH-box helicase 5 is hijacked by an avian oncogenic herpesvirus to inhibit interferon beta production and promote viral replication.

2. Dev Comp Immunol. 2021 Jun;119:104048. doi: 10.1016/j.dci.2021.104048 Epub 2021 Feb 17.
  
  
Xu J(1), Cai Y(1), Ma Z(2), Jiang B(1), Liu W(1), Cheng J(1), Jin H(1), Li Y(3).
  
  
Author information: (1)Institute of Animal Husbandry and Veterinary Medicine, Beijing Academy of Agr ...
  
  
DEAD-box helicase 5 (DDX5) plays a significant role in tumorigenesis and regulates viral replication of several viruses. An avian oncogenic herpesvirus, Marek's disease virus (MDV), is widely known to cause immunosuppression and lymphoma in chickens. However, the underlying mechanisms of how DDX5 plays a role in viral replication remain unclear. In this study, we show that MDV inhibits the production of interferon beta (IFN-β) in chicken embryo fibroblasts (CEFs) by increasing the expression level and promoting the nuclear aggregation of DDX5. We further reveal how DDX5 down-regulates melanoma differentiation-associated gene 5/toll-like receptor 3 signaling through the fundamental transcription factor, interferon regulatory factor 1. MDV replication is suppressed, and the production of IFN-β is promoted in the DDX5 absented CEFs. Taken together, our investigations demonstrate that MDV inhibits IFN-β production by targeting DDX5-mediated signaling to facilitate viral replication, which offers a novel insight into the mechanism by which an avian oncogenic herpesvirus replicates in chicken cells.
  
  
  
  
**DDX5**
-
3
of
3
  

### Preventing the spontaneous modification of an HLA-A2-restricted peptide at an N-terminal glutamine or an internal cysteine residue enhances peptide antigenicity.

3. J Immunother. 2004 May-Jun;27(3):177-83. doi: 10.1097/00002371-200405000-00001
  
  
Thompson LW(1), Hogan KT, Caldwell JA, Pierce RA, Hendrickson RC, Deacon DH, Settlage RE, Brinckerho ...
  
  
Author information: (1)Department of Surgery, University of Virginia, Charlottesville 22908, USA.
  
  
The p68-derived peptide, QIVDVCHDV, was identified by a reverse immunology approach as capable of reconstituting an epitope recognized by the melanoma-reactive cytotoxic T lymphocyte (CTL) line VMM5. The peptide has not been demonstrated definitively on the cell surface by mass spectrometry; thus, it is not yet considered appropriate for use in human melanoma vaccines. Interestingly, however, the antigenicity of this peptide was affected by spontaneous modifications at two distinct residues. Spontaneous modification of the QIVDVCHDV peptide can occur at the cysteine residue at position 6 or at the N-terminal glutamine residue, and both modifications dramatically affect CTL recognition. Avoidance of an acidic environment prevents the conversion of the N-terminal glutamine residue to pyroglutamic acid, a conversion that inhibits binding of the peptide to HLA-A2 and diminishes recognition by CTLs. Substitution of asparagine for the N-terminal glutamine and substitution of serine for the cysteine were shown to enhance the binding of the peptide to HLA-A2 and to enhance the recognition of the peptide by CTLs. These findings suggest general strategies for enhancing the antigenicity of other peptides containing similar amino acids in their sequence.
  
  
  
  
  

---

  

# EBF1

**Signature gene in:** B cells
  
**Differentially expressed in:** no cell type
  
  
**EBF1**
-
1
of
1
  

### Integrative RNA profiling of TBEV-infected neurons and astrocytes reveals potential pathogenic effectors.

1. Comput Struct Biotechnol J. 2022 May 30;20:2759-2777. doi: 10.1016/j.csbj.2022.05.052 eCollection 2022.
  
  
Selinger M(1)(2), Věchtová P(1), Tykalová H(1)(2), Ošlejšková P(1), Rumlová M(3), Štěrba J(1), Grubh ...
  
  
Author information: (1)Faculty of Science, University of South Bohemia in České Budějovice, Branišov ...
  
  
Tick-borne encephalitis virus (TBEV), the most medically relevant tick-transmitted flavivirus in Eurasia, targets the host central nervous system and frequently causes severe encephalitis. The severity of TBEV-induced neuropathogenesis is highly cell-type specific and the exact mechanism responsible for such differences has not been fully described yet. Thus, we performed a comprehensive analysis of alterations in host poly-(A)/miRNA/lncRNA expression upon TBEV infection in vitro in human primary neurons (high cytopathic effect) and astrocytes (low cytopathic effect). Infection with severe but not mild TBEV strain resulted in a high neuronal death rate. In comparison, infection with either of TBEV strains in human astrocytes did not. Differential expression and splicing analyses with an in silico prediction of miRNA/mRNA/lncRNA/vd-sRNA networks found significant changes in inflammatory and immune response pathways, nervous system development and regulation of mitosis in TBEV Hypr-infected neurons. Candidate mechanisms responsible for the aforementioned phenomena include specific regulation of host mRNA levels via differentially expressed miRNAs/lncRNAs or vd-sRNAs mimicking endogenous miRNAs and virus-driven modulation of host pre-mRNA splicing. We suggest that these factors are responsible for the observed differences in the virulence manifestation of both TBEV strains in different cell lines. This work brings the first complex overview of alterations in the transcriptome of human astrocytes and neurons during the infection by two TBEV strains of different virulence. The resulting data could serve as a starting point for further studies dealing with the mechanism of TBEV-host interactions and the related processes of TBEV pathogenesis.
  
  
  
  
  

---

  

# EGFR

**Signature gene in:** Tumor cells
  
**Differentially expressed in:** no cell type
  
  
**EGFR**
-
1
of
10
  

### Sensitivity to immune checkpoint inhibitors in BRAF/MEK inhibitor refractory melanoma.

1. J Immunother Cancer. 2025 May 15;13(5):e011551. doi: 10.1136/jitc-2025-011551
  
  
Patel RP(1)(2), Lim LRJ(1)(2), Saleh R(1), Schenk D(1), Lee MK(1)(2), Lelliott E(3)(4)(5)(6), Rao AD ...
  
  
Author information: (1)Peter MacCallum Cancer Centre, Melbourne, Victoria, Australia. (2)Sir Peter M ...
  
  
BACKGROUND: Resistance to BRAF and MEK inhibitors (BRAFi/MEKi) in metastatic melanoma frequently results in cross-resistance to immune checkpoint inhibitors (ICI), limiting effective treatment options. However, a subset of BRAFi/MEKi-resistant patients remains responsive to second-line ICI, suggesting heterogeneous underlying resistance mechanisms. This study aimed to explore the tumor immune microenvironment in BRAFi/MEKi-resistant melanoma to uncover factors influencing sensitivity to second-line ICI therapy. METHOD: To investigate mechanisms underlying resistance and responsiveness to second-line ICIs, BRAFi/MEKi-resistant melanoma mouse models were used. Flow cytometry was employed to analyze immune cell populations within the tumor microenvironment, focusing on changes in CD8+T effector cells and other key immune subsets. RNA sequencing was performed to profile transcriptomic changes in resistant tumors, providing insights into the signaling pathways associated with resistance. Clinical samples from BRAFi/MEKi-resistant patients were further evaluated for correlations between immune profiles and key signaling pathways to support findings from the preclinical models. RESULTS: Using BRAFi/MEKi-resistant melanoma mouse models, we observed distinct alterations in the tumor-immune microenvironment. Tumors exhibiting resistance showed a significant increase in CD8+T effector cells following BRAFi/MEKi treatment, suggesting an immune-stimulatory response. Mechanistic analysis identified the activation of the EGFR-STAT signaling pathway as a key driver of intrinsic resistance in these models. Notably, these tumors retained sensitivity to second-line ICI therapy, contrasting with NRAS-driven BRAFi/MEKi-resistant tumors, which demonstrated cross-resistance to ICIs. Supporting these findings, clinical samples from BRAFi/MEKi-resistant patients revealed a correlation between elevated EGFR activation and higher immune scores, indicating potential sensitivity to ICI therapy in this subset of patients. CONCLUSION: EGFR overexpression emerges as a potential predictive biomarker for responsiveness to second-line ICIs in BRAFi/MEKi-resistant melanoma. These findings underscore the need for stratified therapeutic approaches and highlight EGFR as a target for improving outcomes in ICI therapy.
  
  
  
  
**EGFR**
-
2
of
10
  

### HER2 expression in different cell lines at different inoculation sites assessed by [(52)Mn]Mn-DOTAGA(anhydride)-trastuzumab.

2. Pathol Oncol Res. 2025 Apr 29;31:1611999. doi: 10.3389/pore.2025.1611999 eCollection 2025.
  
  
Ngô TM(1)(2), Vágner A(3), Nagy G(3), Ország G(3), Nagy T(2)(3), Szoboszlai Z(3), Csikos C(1)(2), Vá ...
  
  
Author information: (1)Gyula Petrányi Doctoral School of Clinical Immunology and Allergology, Facult ...
  
  
PURPOSE: Positron emission tomography (PET) hybrid imaging targeting HER2 requires antibodies labelled with longer half-life isotopes. With a suitable radiation profile, 52Mn coupled with DOTAGA as a bifunctional chelator is a potential candidate. In this study, we investigated the tumor HER2 specificity and the temporal biodistribution of the [52Mn]Mn-DOTAGA(anhydride)-trastuzumab in preclinical models. METHODS: PET/MRI and PET/CT were performed on SCID mice bearing orthotopic and ectopic HER2-positive and ectopic HER2-negative tumors at 4, 24, 48, 72, and 120 h post-injection with [52Mn]Mn-DOTAGA(anhydride)-trastuzumab. Melanoma xenografts were included for comparison of specificity. RESULTS: In vivo biodistribution demonstrated strong contrast in HER2-positive tumors, particularly in orthotopic tumors, where uptake was significantly higher than in the blood pool and other organs from 24 h onwards and consistently higher than in ectopic HER2-positive tumors at all time points. Significantly higher tumor-to-blood and tumor-to-muscle ratios were observed in HER2-positive ectopic tumors compared to HER2-negative tumors but only at 4 and 24 h; the differences were likely due to non-specific binding of the tracer. The ratios for orthotopic HER2-positive tumors were significantly higher than those for ectopic HER2-negative tumors and melanoma at all time points. However, the differences between HER2-positive and HER2-negative tumors decreased at later time points. CONCLUSION: These results suggest that [52Mn]Mn-DOTAGA(anhydride)-trastuzumab demonstrates efficient tumor-to-background contrast, emphasize the higher tumor uptake observed in orthotopic tumors, and highlight the influence of tumor environment characteristics on uptake.
  
  
  
  
**EGFR**
-
3
of
10
  

### Protein tyrosine phosphatase receptor type kappa (PTPRK) revisited: evolving insights into structure, function, and pathology.

3. J Transl Med. 2025 May 12;23(1):534. doi: 10.1186/s12967-025-06496-1
  
  
Zheng C(#)(1)(2), Liu T(#)(1), Wang AQ(1), Chen XA(1), Zhang RZ(1), Wang XC(1), Lv CY(2), Pan RL(1)( ...
  
  
Author information: (1)School of Basic Medical Sciences, Wenzhou Medical University, Wenzhou, 325035 ...
  
  
Protein Tyrosine Phosphatase Receptor Type Kappa (PTPRK) is a membrane-bound tyrosine phosphatase encoded by the frequently deleted region of chromosome 6q, which plays a crucial role in regulating cell signaling, adhesion, and immune response. Structurally, PTPRK comprises with an extracellular domain involved in cell-cell adhesion, a transmembrane region, and two intracellular catalytic domains responsible for its phosphatase activity. Notably, PTPRK undergoes proteolytic cleavage by Furin and ADAM10, resulting in the generation of an extracellular E-subunit and a P-subunit. Further processing by γ-secretase releases the intracellular PIC, which plays a pivotal role in regulating β-catenin signaling within the nucleus. PTPRK is widely recognized for its tumor-suppressive properties across various cancers, including colorectal, lung, ovarian, and melanoma. Despite its function as a tumor suppressor, the expression and activity of PTPRK exhibit considerable variability across different cancer types and stages. It exerts its effects by dephosphorylating key signaling molecules such as EGFR, STAT3, CD133 and β-catenin, thereby inhibiting cancer cell proliferation, survival, and metastasis. Beyond its role in cancer, PTPRK is also involved in immune regulation, particularly in the development of CD4 + T cells, and has been implicated in autoimmune diseases such as multiple sclerosis. In the nervous system, PTPRK is linked to neurite outgrowth and synaptic transmission, with genetic polymorphisms in PTPRK associated with an increased risk of neurodegenerative diseases like Alzheimer's disease. Given its extensive involvement in cancer biology, immune regulation, and neurodevelopment, PTPRK presents a promising therapeutic target. Strategies aimed at restoring its activity or targeting PTPRK might offer new approaches for current cancer therapies and overcome drug resistance. In this review, we elucidate the structural characteristics and functional roles of PTPRK in cellular signaling and disease pathogenesis. The variability of PTPRK suggests that the regulatory mechanisms governing its activity are intricate and worth further comprehensive investigation.
  
  
  
  
**EGFR**
-
4
of
10
  

### Design and synthesis of novel 2-S-alkylated Quinazolinones as dual BRAF(V600E) and EGFR inhibitors in melanoma: Mechanistic insights from apoptosis and cell cycle modulation.

4. Bioorg Chem. 2025 Apr 28;161:108526. doi: 10.1016/j.bioorg.2025.108526 Online ahead of print.
  
  
El-Shafey HW(1), Al-Sanea MM(2), Elnagar MR(3), Gendy AM(4), Serag MI(5), Almatary AM(6), Khalaf MA( ...
  
  
Author information: (1)Department of Pharmaceutical Organic Chemistry, Faculty of Pharmacy, Mansoura ...
  
  
Melanoma, an aggressive and highly metastatic form of skin cancer, remains challenging to treat due to its resistance to conventional therapies and frequent mutations in the BRAF signaling pathway. In this study, we report the design and synthesis of a novel series of thirteen quinazolinone derivatives, featuring a phenyl thiazole moiety linked via a triazole acetamide spacer. These compounds were developed as potential dual inhibitors of BRAFV600E and EGFR, which should offer a promising therapeutic strategy for melanoma treatment. The antiproliferative activity of these compounds was evaluated against the NCI-60 cell line panel, with six compounds advancing to a five-dose screening. Three compounds, 7k, 7l, and 7m, exhibited broad-spectrum anticancer activity, with mean growth inhibition (GI%) exceeding 100 %. Compound 7l demonstrated exceptional efficacy against melanoma subpanels (GI% = 152 %) and potent dual kinase inhibition, with IC50 values of 0.048 μM against B-RAFV600E and 0.037 μM against EGFR. In vitro studies of compound 7l revealed significant cytotoxicity against MALME-3 M (IC50 = 3.16 μM) and LOX-IMVI (IC50 = 2.50 μM) melanoma cell lines, with minimal toxicity towards normal Vero cells. Cell cycle analysis showed G1-phase arrest and disrupted DNA synthesis in melanoma cells, while apoptosis assays demonstrated a dramatic increase in early apoptotic cells from 7.28 % to 40.69 %. Compound 7l modulated key apoptotic markers, increasing the BAX/Bcl-2 ratio by 14.42-fold and elevating caspase 3 and 9 levels, indicating its potential to overcome drug resistance and enhance therapeutic efficacy in melanoma treatment.
  
  
  
  
**EGFR**
-
5
of
10
  

### Small Molecule B-RAF Inhibitors as Anti-Cancer Therapeutics: Advances in Discovery, Development, and Mechanistic Insights.

5. Int J Mol Sci. 2025 Mar 16;26(6):2676. doi: 10.3390/ijms26062676
  
  
Anaya YA(1)(2)(3)(4), Bracho RP(1)(3)(4), Chauhan SC(3)(4), Tripathi MK(3)(4), Bandyopadhyay D(1)(5) ...
  
  
Author information: (1)School of Integrative Biological and Chemical Sciences, The University of Tex ...
  
  
B-RAF is a serine/threonine kinase that plays a crucial role in the MAPK signaling pathway, regulating cell proliferation and survival. Mutations in B-RAF, particularly V600E, are associated with several malignancies, including melanoma, colorectal cancer, and non-small cell lung cancer, making it a key therapeutic target. The development of B-RAF inhibitors, such as Vemurafenib, Dabrafenib, and second-generation inhibitors like Encorafenib, has led to significant advancements in targeted cancer therapy. However, acquired resistance, driven by MAPK pathway reactivation, RAF dimerization, and alternative signaling pathways, remains a major challenge. This review explores the molecular mechanisms of B-RAF inhibitors, their therapeutic efficacy, and resistance mechanisms, emphasizing the importance of combination strategies to enhance treatment outcomes. The current standard of care involves B-RAF and MEK inhibitors, with additional therapies such as EGFR inhibitors and immune checkpoint blockades showing potential in overcoming resistance. Emerging pan-RAF and brain-penetrant inhibitors offer new opportunities for treating refractory cancers, while precision medicine approaches, including genomic profiling and liquid biopsies, are shaping the future of B-RAF-targeted therapy.
  
  
  
  
**EGFR**
-
6
of
10
  

### Expanding the Therapeutic Reach of Chimeric Antigen Receptor T-Cells and Bispecific T-Cell Engagers Across Solid Tumors.

6. JCO Precis Oncol. 2025 Mar;9:e2400753. doi: 10.1200/PO-24-00753 Epub 2025 Mar 26.
  
  
Gerber WK(1), Xie Y(1), Patel SA(1)(2)(3).
  
  
Author information: (1)Department of Medicine, Division of Hematology/Oncology, UMass Memorial Medic ...
  
  
The introduction of T-cell-based therapeutics in hematologic malignancies has led to improvements in outcomes for patients with acute leukemia, lymphoma, and multiple myeloma. To date, the Food and Drug Administration (FDA) has approved seven chimeric antigen receptor-T (CAR-T) cell therapies and seven bispecific T-cell engagers (BiTEs) across a variety of hematologic malignancies; however, the extension of CAR-T therapies and BiTEs to the solid tumor arena has been somewhat limited. In this review, we discuss the landmark data that led to the commercialization of four novel FDA-approved T-cell-based therapeutics in solid malignancies, including tarlatamab for small cell lung cancer, afamitresgene autoleucel for synovial sarcoma, lifileucel for metastatic melanoma, and tebentafusp for metastatic uveal melanoma. We discuss the targetable antigen landscape of CAR-T therapies and BiTEs under investigation in solid malignancies. We explore the translational potential for various CARs under active investigation, including human epidermal growth factor receptor 2-directed CARs in breast cancer, prostate stem cell antigen-directed CARs for prostate cancer, epidermal growth factor receptor (EGFR)-IL13Ra2 and EGFR-vIII CARs for glioblastoma, and GD2-directed CARs for neuroblastoma. We glean from lessons learned for existing CAR-T therapies and BiTEs for hematologic malignancies and emphasize solutions toward facilitating the clinical rollout of T-cell-based therapies in solid tumors, including scalability to meet the growing needs of clinical oncology. Some solutions include addressing on-target, off-tumor toxicity; improving the manufacturing of CARs; optimizing the tissue-specific tumor microenvironment by combating immune desert tumors; and discovering natural tumor neoantigens and non-self-epitopes generated by tumor-specific mutations. These concepts can help provide transformative benefits for patients with solid malignancies in the coming years.
  
  
  
  
**EGFR**
-
7
of
10
  

### The impact of the immunological context on outcomes of solid cancer patients treated with genotype-matched targeted therapies: a comprehensive review.

7. Ann Oncol. 2025 Mar 19:S0923-7534(25)00113-9. doi: 10.1016/j.annonc.2025.03.007 Online ahead of print.
  
  
Mubarak O(1), Middleton GW(2).
  
  
Author information: (1)Department of Immunology and Immunotherapy, College of Medicine and Health, U ...
  
  
BACKGROUND: Outcomes with genotype-matched targeted therapy in solid cancer patients are heterogeneous: some have exceptional responses, whereas others have primary progression. This review explores the immunobiological features which may underlie this differential response. METHODS: We conducted a literature review of studies assessing the impact of immune context following searches on Web of Science, Medline and Embase. Relevant outcomes include response, progression-free survival and overall survival. Data were extracted from multivariate analysis, univariate analysis or directly from Kaplan-Meier curves. Meta-analyses were carried out where three or more studies analysed the same immune factor for the same cancer type. The remaining studies were analysed descriptively. RESULTS: In the adjuvant setting, assessment of the immune context does not highlight a group failing to derive benefit for the use of dabrafenib/trametinib after resection of BRAFV600E melanoma. Differential gene expression in exceptional responders show enrichment of genes associated with immune activation. BRAFV600E colorectal cancer patients with high cytolytic scores benefit from the addition of MEK inhibition whereas those with low scores fare better without. High programmed death-ligand 1 (PD-L1) expression is predictive of inferior outcomes to epidermal growth factor receptor (EGFR), ALK and G12C tyrosine kinase inhibitors. EGFR-mutant patients with high CD8+ T cells and PD-L1 positivity have very poor outcomes. Stromal tumour-infiltrating lymphocytes predict for efficacy of stromal-poor tumours in human epidermal growth factor receptor 2 (HER2)-positive breast cancer treated with short-course adjuvant trastuzumab. High immune metagene and single immune gene expression are predictive of benefit for chemotherapy plus trastuzumab, but not chemotherapy alone. The addition of pertuzumab or lapatanib appears to be beneficial in those with immune non-enriched microenvironments. High major histocompatibility complex (MHC)-I is negatively predictive and high MHC-II is positively predictive of outcomes with trastuzumab-based therapy. CONCLUSIONS: To our knowledge, this is the first review assessing immunological context as a biomarker for targeted therapy. The results of this review represent an important resource to aid future translational studies in advancing stratified precision medicine oncology.
  
  
  
  
**EGFR**
-
8
of
10
  

### Revealing the role of RAB27 in HER receptor family expression and signaling in melanoma cells.

8. Cell Commun Signal. 2025 Mar 4;23(1):118. doi: 10.1186/s12964-025-02064-8
  
  
Horodecka K(1), Czernek L(2), Pęczek Ł(2), Klink M(3).
  
  
Author information: (1)Centre of Molecular and Macromolecular Studies, Polish Academy of Sciences, S ...
  
  
BACKGROUND: Alterations in signalling pathways fuel the growth and progression of melanoma. Therefore, understanding these processes is essential for developing effective treatment strategies. RAB27A and RAB27B are known to possess oncogenic effects by modulating cancer cell proliferation, invasion and drug resistance in various types of cancer, including melanoma. These proteins are mostly acknowledged as coordinators of the vesicular trafficking, however, their function in cellular signaling is less recognized. Therefore we aimed to investigate the relationship between RAB27 and oncogenic or signalling proteins in melanoma cells. METHODS: We generated RAB27A knockout (KO) in SkMel28, A375, and patient-derived DMBC12 melanoma cell lines. Additionally, a double RAB27A/B knockout (dKO) A375 cell line was created. Firstly, we applied the Proteome Profiler array to identify proteins differentially expressed upon RAB27A/B loss. Subsequently, we picked selected specific proteins for a further in-depth analysis using RT-PCR, Western blot, and flow cytometry. RESULTS: We found that silencing RAB27 markedly decreased the levels of various intracellular proteins linked with proliferation, invasion, angiogenesis, adhesion, or EMT at a cell-line dependent level. Among others, we observed a link between the expression of RAB27 and EGFR, HER2 and HER3. Altered levels of HER receptors disturbed the downstream signaling pathways by reducing the phosphorylation of AKT and ERK1/2 proteins. CONCLUSIONS: Our findings present novel, previously unpublished data on the relationship between HER family receptor expression and potential activity, and the involvement of RAB27 in melanoma cells.
  
  
  
  
**EGFR**
-
9
of
10
  

### EGFR influences the resistance to targeted therapy in BRAF (V600E) melanomas by regulating the ferroptosis process.

9. Arch Dermatol Res. 2025 Mar 1;317(1):514. doi: 10.1007/s00403-025-03895-8
  
  
Sun Y(#)(1), Yu H(#)(1), Zhou Y(1), Bao J(2), Qian X(3)(4).
  
  
Author information: (1)Department of Dermotology, Nanjing Drum Tower Hospital, Clinical College of N ...
  
  
To identify genes differentially expressed between resistant and sensitive BRAF V600E melanoma cell lines using bioinformatics tools applied to GEO data. We retrieved and downloaded the target gene set (GSE45558) from the GEO database and used R software to filter differentially expressed genes (DEGs) between BRAF V600E melanoma cell lines resistant. The identified DEGs were subjected to GO functional enrichment analysis (including biological processes, molecular functions, and cellular components) and Kyoto Encyclopedia of Genes and Genomes (KEGG) pathway analysis, utilizing R software. Protein-protein interaction networks for the DEGs were generated using the STRING online database. Top hub genes were cross-referenced with genes related to ferroptosis from the FerrDb database to identify DEGs linked to ferroptosis in resistant melanoma cells. From the GEO database analysis, we identified the top 100 DEGs between BRAF V600E melanoma cell lines, including 50 downregulated and 50 upregulated DEGs. Using STRING and Cytoscape, we identified the top 10 hub genes: IL6, IL1B, CCL2, MMP2, TGFB2, EGFR, POSTN, SERPINE1, COL1A2, and MITF. Cross-referencing with the FerrDb database, we found that IL6 and EGFR are differentially expressed genes related to ferroptosis in resistant melanoma cells. Validation through clinical samples and in vitro experiments confirmed the high expression of the ferroptosis-related gene EGFR as a potential biomarker for resistance to targeted therapy in BRAFV600E melanoma. Bioinformatics analysis identified key resistance genes in BRAFV600E melanoma targeted therapy, demonstrating the impact of ferroptosis-related gene EGFR on the resistance of BRAFV600E melanoma.
  
  
  
  
**EGFR**
-
10
of
10
  

### Survival expectations in melanoma patients: a molecular prognostic model associated with aging.

10. Discov Oncol. 2025 Feb 28;16(1):253. doi: 10.1007/s12672-025-01971-z
  
  
Zhang N(#)(1), Qiu X(#)(2), Chen X(1), Du C(3), Dong J(4), Li X(5), Chen B(4), Zhang L(6), Zhang Y(7 ...
  
  
Author information: (1)Clinical Laboratory, Jiaxing Hospital of Traditional Chinese Medicine, Zhejia ...
  
  
BACKGROUND: Aging and long non-coding RNAs (lncRNAs) are research hotspots in melanoma. However, no study has so far explored the relationship between melanoma prognosis and aging-related lncRNAs (ARLs). METHODS: The Cancer Genome Atlas database, the GTEx database, and the HAGR database were used in this study in a combined manner. Univariate and multivariate cox regression analyses were used to screen out lncRNA signatures associated with overall survival (OS) in the primary dataset. The risk scoring model was analyzed by risk stratification and tested internally. The protein expression levels of possible target genes of ARLs were verified by immunohistochemistry analysis in HPA database. Finally, gene enrichment analysis was performed. RESULTS: In the primary dataset, five OS-related lncRNA signatures (AC011481.1, USP30-AS1, EBLN3P, LINC01527, HLA-DQB1-AS1) were screened out. The survival curve showed that the high-risk group had a worse prognosis than the low-risk group. The immunohistochemical analysis revealed that reduced expression of Epidermal Growth Factor Receptor (EGFR), along with increased expression of Activating Transcription Factor 2 (ATF2) and DNA Polymerase Delta 1 (POLD1), was linked to a worse prognosis. Finally, enrichment analysis revealed that OS-related DELs were significantly enriched in the regulation of reactive oxygen metabolism, etc. The ARGs were significantly activated in the SKCM tissues. The regulation of aging in melanoma cells may be realized through ferroptosis, immunity, and autophagy and so on. CONCLUSION: The ARL signature obtained in this study had better prognostic ability than individual clinical features.
  
  
  
  
  

---

  

# FBXW7

**Signature gene in:** Plasma cells
  
**Differentially expressed in:** no cell type
  
  
**FBXW7**
-
1
of
10
  

### Baicalin alleviates acute lung injury in vivo and in vitro.

1. Int Immunopharmacol. 2024 Dec 25;143(Pt 1):113128. doi: 10.1016/j.intimp.2024.113128 Epub 2024 Oct 4.
  
  
Wang S(1), Wu M(1), Ding J(1), Tan W(2), Jiang H(3).
  
  
Author information: (1)Department of Emergency, Shanghai Pulmonary Hospital, Tongji University Schoo ...
  
  
The aim of the present study was to evaluate the effects and mechanisms of Baicalin (BA) on acute lung injury (ALI). ALI model was established by lipopolysaccharide (LPS) and proteomics, immunoprecipitation and F-box/WD repeat containing protein 7 （FBXW7） knockout (KO) mice and FBXW7 silence mouse lung epithelial (MLE-12) cells were used to investigate the mechanisms of BA on acute lung injury ALI. The results showed that 218 differentially expressed proteins were identified in the lung tissue of ALI mice and FBXW7 was one of the changed most proteins and was significantly decreased in in the lung tissue of ALI mice. It was also found that FBXW7 had protective effects on ALI via inhibition of Absent in Melanoma 2 （AIM2） inflammasomes also found that BA mitigated ALI via FBXW7/AIM2 signal pathway. In conclusion, FBXW7 as a key marker was identified in ALI and has a protective effect on ALI and BA regulated FBXW7/AIM2 signal pathway to alleviate ALI. This study provided a new method for treating ALI.
  
  
  
  
**FBXW7**
-
2
of
10
  

### Melanoma genomics - will we go beyond BRAF in clinics?

2. J Cancer Res Clin Oncol. 2024 Sep 28;150(9):433. doi: 10.1007/s00432-024-05957-2
  
  
Mirek J(1), Bal W(2), Olbryt M(3).
  
  
Author information: (1)Center for Translational Research and Molecular Biology of Cancer, Maria Sklo ...
  
  
Erratum in J Cancer Res Clin Oncol. 2025 Feb 13;151(2):81. doi: 10.1007/s00432-025-06112-1.
  
  
  
  
**FBXW7**
-
3
of
10
  

### Identifying novel circadian rhythm biomarkers for diagnosis and prognosis of melanoma by an integrated bioinformatics and machine learning approach.

3. Aging (Albany NY). 2024 Jun 20;16(16):11824-11842. doi: 10.18632/aging.205961 Epub 2024 Jun 20.
  
  
Xu Y(1), Zeng C(1), Bin J(1), Tang H(1), Li W(1).
  
  
Author information: (1)Department of Plastic Surgery, Second People’s Hospital of Hunan Provi ...
  
  
Melanoma is a highly malignant skin tumor with poor prognosis. Circadian rhythm is closely related to melanoma pathogenesis. This study aimed to identify key circadian rhythm genes (CRGs) in melanoma and explore their potential as diagnostic and prognostic biomarkers. Microarray data of melanoma tissues and normal skins were obtained. Differentially expressed genes were identified and weighted gene co-expression network analysis (WGCNA) was performed to screen hub genes associated with melanoma. By overlapping hub genes with known CRGs, 125 melanoma-related CRGs were identified. Functional enrichment analysis revealed these CRGs were mainly involved in circadian rhythm and other cancer-related pathways. Three machine learning algorithms including LASSO regression, support vector machine-recursive feature elimination (SVM-RFE), and random forest were utilized to select key CRGs. Six CRGs (ABCC2, CA14, EGR3, FBXW7, LDHB, and PSEN2) were identified as key CRGs for melanoma diagnosis and prognosis. Diagnostic values of key CRGs were evaluated by ROC analysis in training and validation sets. Prognostic values of key CRGs were assessed by survival analysis and a multivariate Cox regression prognostic model was constructed. The prognostic model could effectively stratify melanoma patients into high- and low-risk groups with significantly different survival. A nomogram integrating clinical variables and risk score was built to predict 3-, 5- and 10-year overall survival of melanoma patients. In summary, six CRGs were identified as key genes associated with melanoma pathogenesis and may serve as promising diagnostic and prognostic biomarkers. The prognostic model and nomogram could facilitate personalized prognosis evaluation of melanoma patients.
  
  
  
  
**FBXW7**
-
4
of
10
  

### Increased Expression and Prognostic Significance of BYSL in Melanoma.

4. J Immunother. 2024 Oct 1;47(8):279-302. doi: 10.1097/CJI.0000000000000530 Epub 2024 Jul 9.
  
  
Wang ZZ(1), Yao GT(2), Wang LZ(3), Zhu YJ(3), Chen JH(1).
  
  
Author information: (1)Department of Dermatology, School of Medicine, Shanghai Fourth People's Hospi ...
  
  
We evaluated the BYSL content and underlying mechanism in melanoma (SKCM) overall survival (OS). In this study, we used a comprehensive approach combining bioinformatics tools, including miRNA estimation, quantitative real-time polymerase chain reaction (qRT-PCR) of miRNAs, E3 ligase estimation, STRING analysis, TIMER analysis, examination of associated upstream modulators, protein-protein interaction (PPI) analysis, as well as retrospective and survival analyses, alongside clinical sample validation. These methods were used to investigate the content of BYSL, its methylation status, its relation to patient outcome, and its immunologic significance in tumors. Our findings revealed that BYSL expression is negatively regulated by BYSL methylation. Analysis of 468 cases of SKCM RNA sequencing samples demonstrated that enhanced BYSL expression was associated with higher tumor grade. We identified several miRNAs, namely hsa-miR-146b-3p, hsa-miR-342-3p, hsa-miR-511-5p, hsa-miR-3690, and hsa-miR-193a-5p, which showed a strong association with BYSL levels. Furthermore, we predicted the E3 ubiquitin ligase of BYSL and identified CBL, FBXW7, FZR1, KLHL3, and MARCH1 as potential modulators of BYSL. Through our investigation, we discovered that PNO1, RIOK2, TSR1, WDR3, and NOB1 proteins were strongly associated with BYSL expression. In addition, we found a close association between BYSL levels and certain immune cells, particularly dendritic cells (DCs). Notably, we observed a significant negative correlation between miR-146b-3p and BYSL mRNA expression in SKCM sera samples. Collectively, based on the previously shown evidences, BYSL can serve as a robust bioindicator of SKCM patient prognosis, and it potentially contributes to immune cell invasion in SKCM.
  
  
  
  
**FBXW7**
-
5
of
10
  

### Overcoming BRAF and CDK4/6 inhibitor resistance by inhibiting MAP3K3-dependent protection against YAP lysosomal degradation.

5. Exp Mol Med. 2024 Apr;56(4):987-1000. doi: 10.1038/s12276-024-01210-5 Epub 2024 Apr 16.
  
  
Park S(#)(1)(2), Ryu WJ(#)(3), Kim TY(3), Hwang Y(3), Han HJ(3), Lee JD(3), Kim GM(4), Sohn J(4), Ki ...
  
  
Author information: (1)Department of Dermatology, Chonnam National University Medical School, Gwangj ...
  
  
Transcriptional programs governed by YAP play key roles in conferring resistance to various molecular-targeted anticancer agents. Strategies aimed at inhibiting YAP activity have garnered substantial interest as a means to overcome drug resistance. However, despite extensive research into the canonical Hippo-YAP pathway, few clinical agents are currently available to counteract YAP-associated drug resistance. Here, we present a novel mechanism of YAP stability regulation by MAP3K3 that is independent of Hippo kinases. Furthermore, we identified MAP3K3 as a target for overcoming anticancer drug resistance. Depletion of MAP3K3 led to a substantial reduction in the YAP protein level in melanoma and breast cancer cells. Mass spectrometry analysis revealed that MAP3K3 phosphorylates YAP at serine 405. This MAP3K3-mediated phosphorylation event hindered the binding of the E3 ubiquitin ligase FBXW7 to YAP, thereby preventing its p62-mediated lysosomal degradation. Robust YAP activation was observed in CDK4/6 inhibitor-resistant luminal breast cancer cells. Knockdown or pharmacological inhibition of MAP3K3 effectively suppressed YAP activity and restored CDK4/6 inhibitor sensitivity. Similarly, elevated MAP3K3 expression supported the prosurvival activity of YAP in BRAF inhibitor-resistant melanoma cells. Inhibition of MAP3K3 decreased YAP-dependent cell proliferation and successfully restored BRAF inhibitor sensitivity. In conclusion, our study reveals a previously unrecognized mechanism for the regulation of YAP stability, suggesting MAP3K3 inhibition as a promising strategy for overcoming resistance to CDK4/6 and BRAF inhibitors in cancer treatment.
  
  
  
  
**FBXW7**
-
6
of
10
  

### Clinical significance of genetic profiling based on different anatomic sites in patients with mucosal melanoma who received or did not receive immune checkpoint inhibitors.

6. Cancer Cell Int. 2023 Aug 30;23(1):187. doi: 10.1186/s12935-023-03032-3
  
  
Wang HY(1)(2), Liu Y(3)(4), Deng L(4), Jiang K(4), Yang XH(4), Wu XY(4), Guo KH(5), Wang F(6).
  
  
Author information: (1)Department of Pathology, Guangdong Provincial Clinical Research Center for Ch ...
  
  
BACKGROUND: To date, data on the efficacy of targeted therapies for mucosal melanoma (MM) are limited. In this study, we analyzed genetic alterations according to the primary site of origin, which could provide clues for targeted therapy for MM. METHODS: We conducted a retrospective cohort study of 112 patients with MM. Targeted sequencing was performed to analyze genetic aberrations. Kaplan-Meier analysis was conducted with the log-rank test to compare the significance among subgroups. RESULTS: In total, 112 patients with MM were included according to the anatomic sites: 38 (33.9%) in the head and neck, 22 (19.6%) in the genitourinary tract, 21 (18.8%) in the anorectum, 19 (17.0%) in the esophagus, 10 (8.9%) in the uvea, and 2 (1.8%) in the small bowel. The most significantly mutated genes included BRAF (17%), KIT (15%), RAS (15%), TP53 (13%), NF1 (12%), SF3B1 (11%), GNA11 (7%), GNAQ (5%), and FBXW7 (4%). A large number of chromosomal structural variants was found. The anatomic sites of esophagus and small bowel were independent risk factors for progression-free survival (PFS, hazard ratio [HR] 4.78, 95% confidence interval [CI] 2.42-9.45, P < 0.0001) and overall survival (OS, HR 5.26, 95% CI 2.51-11.03, P < 0.0001). Casitas B-lineage lymphoma (CBL) mutants showed significantly poorer PFS and OS. In contrast, MM patients who received immune checkpoint inhibitors (ICIs) had a significantly more favorable OS (HR 0.39, 95% CI 0.20-0.75, P = 0.008). CONCLUSIONS: Our findings reveal the genetic features of patients with MM, mainly across six anatomic sites, offering a potential avenue for targeted therapies.
  
  
  
  
**FBXW7**
-
7
of
10
  

### Whole-Exome Sequencing and cfDNA Analysis Uncover Genetic Determinants of Melanoma Therapy Response in a Real-World Setting.

7. Int J Mol Sci. 2023 Feb 21;24(5):4302. doi: 10.3390/ijms24054302
  
  
Vanni I(1), Pastorino L(1)(2), Tanda ET(2)(3), Andreotti V(1), Dalmasso B(1), Solari N(4), Mascherin ...
  
  
Author information: (1)Genetics of Rare Cancers, IRCCS Ospedale Policlinico San Martino, 16132 Genoa ...
  
  
Although several studies have explored the molecular landscape of metastatic melanoma, the genetic determinants of therapy resistance are still largely unknown. Here, we aimed to determine the contribution of whole-exome sequencing and circulating free DNA (cfDNA) analysis in predicting response to therapy in a consecutive real-world cohort of 36 patients, undergoing fresh tissue biopsy and followed during treatment. Although the underpowered sample size limited statistical analysis, samples from non-responders had higher copy number variations and mutations in melanoma driver genes compared to responders in the BRAF V600+ subset. In the BRAF V600- subset, Tumor Mutational Burden (TMB) was twice that in responders vs. non-responders. Genomic layout revealed commonly known and novel potential intrinsic/acquired resistance driver gene variants. Among these, RAC1, FBXW7, GNAQ mutations, and BRAF/PTEN amplification/deletion were present in 42% and 67% of patients, respectively. Both Loss of Heterozygosity (LOH) load and tumor ploidy were inversely associated with TMB. In immunotherapy-treated patients, samples from responders showed higher TMB and lower LOH and were more frequently diploid compared to non-responders. Secondary germline testing and cfDNA analysis proved their efficacy in finding germline predisposing variants carriers (8.3%) and following dynamic changes during treatment as a surrogate of tissue biopsy, respectively.
  
  
  
  
**FBXW7**
-
8
of
10
  

### Molecular profiling of primary uveal melanoma: results of a Polish cohort.

8. Melanoma Res. 2023 Apr 1;33(2):104-115. doi: 10.1097/CMR.0000000000000874 Epub 2023 Jan 30.
  
  
Kowalik A(1)(2), Karpinski P(3), Markiewicz A(4), Orlowska-Heitzman J(5), Romanowska-Dixon B(4), Don ...
  
  
Author information: (1)Department of Molecular Diagnostics, Holy Cross Cancer Center. (2)Division of ...
  
  
There is no published data regarding the molecular alterations of Polish patients with primary uveal melanoma. We performed whole exome sequencing of 20 primary uveal melanomas (UMs), 10 metastasizing and 10 non-metastasizing cases to identify significant molecular alterations. We detected mutations and copy number variants in the BAP1 gene in 50% (10 cases) of the cases. GNA11 mutations were detected in 50% (10 cases) including nine p.Q209L and one p.R183C. GNAQ mutations gene were detected in 40% (8 cases) and all were p.Q209P. SF3B1, EIF1AX, PLCB4 , and PALB2 mutations were detected in one case each. Genetic aberrations of FBXW7 were detected in 55% of cases, with copy number loss of 10 and missense mutation in one. Gain or loss of copy number was observed in 60%, 60%, and 10% of cases in MYC, MLH1 , and CDKN2A genes, respectively. BAP1 and GNAQ tumor suppressor genes are more often mutated in UM with metastasis, while GNA11 mutations are more frequently detected in non-metastasizing tumors. MYC copy gain was present twice as frequently (80% versus 40%) in cases with versus those without metastases. BAP1 mutation correlated with worse overall survival; while GNA11 mutation and CDKN2A loss correlated with better and worse progression-free survival, respectively. We have confirmed BAP1 prognostic potential and documented frequent MYC amplification in metastasizing cases. Although GNA11 mutation and CDKN2A loss significantly correlated with progression-free survival in our study, our sample size is small. The prognostic significance of GNAQ/GNA11 mutation and CDKN2A loss would require further investigation.
  
  
  
  
**FBXW7**
-
9
of
10
  

### Identification and validation of a ferroptosis-related gene signature for predicting survival in skin cutaneous melanoma.

9. Cancer Med. 2022 Sep;11(18):3529-3541. doi: 10.1002/cam4.4706 Epub 2022 Apr 4.
  
  
Ping S(1), Wang S(1), Zhao Y(1), He J(1), Li G(1), Li D(2), Wei Z(1), Chen J(3).
  
  
Author information: (1)Department of Orthopaedics, Liyuan Hospital, Tongji Medical College, Huazhong ...
  
  
PURPOSE: Ferroptosis plays a crucial role in the initiation and progression of melanoma. This study developed a robust signature with ferroptosis-related genes (FRGs) and assessed the ability of this signature to predict OS in patients with skin cutaneous melanoma (SKCM). METHODS: RNA-sequencing data and clinical information of melanoma patients were extracted from TCGA, GEO, and GTEx. Univariate, multivariate, and LASSO regression analyses were conducted to identify the gene signature. A 10 FRG signature was an independent and strong predictor of survival. The predictive performance was assessed using ROC curve. The functions of this gene signature were assessed by GO and KEGG analysis. The statuses of low-risk and high-risk groups according to the gene signature were compared by GSEA. In addition, we investigated the possible relationship of FRGs with immunotherapy efficacy. RESULTS: A prognostic signature with 10 FRGs (CYBB, IFNG, FBXW7, ARNTL, PROM2, GPX2, JDP2, SLC7A5, TUBE1, and HAMP) was identified by Cox regression analysis. This signature had a higher prediction efficiency than clinicopathological features (AUC = 0.70). The enrichment analyses of DEGs indicated that ferroptosis-related immune pathways were largely enriched. Furthermore, GSEA showed that ferroptosis was associated with immunosuppression in the high-risk group. Finally, immune checkpoints such as PDCD-1 (PD-1), CTLA4, CD274 (PD-L1), and LAG3 were also differential expression in two risk groups. CONCLUSIONS: The 10 FRGs signature were a strong predictor of OS in SKCM and could be used to predict therapeutic targets for melanoma.
  
  
  
  
**FBXW7**
-
10
of
10
  

### Prognostic Values of G-Protein Mutations in Metastatic Uveal Melanoma.

10. Cancers (Basel). 2021 Nov 17;13(22):5749. doi: 10.3390/cancers13225749
  
  
Terai M(1), Shimada A(2), Chervoneva I(2), Hulse L(1), Danielson M(1), Swensen J(3), Orloff M(1), We ...
  
  
Author information: (1)Department of Medical Oncology, Sidney Kimmel Cancer Center, Thomas Jefferson ...
  
  
Uveal melanoma is the most common primary ocular malignancy in adults, characterized by gene mutations in G protein subunit alpha q (GNAQ) and G protein subunit alpha 11 (GNA11). Although they are considered to be driver mutations, their role in MUM remains elusive. We investigated key somatic mutations of MUM and their impact on patients' survival after development of systemic metastasis (Met-to-Death). Metastatic lesions from 87 MUM patients were analyzed by next generation sequencing (NGS). GNA11 (41/87) and GNAQ (39/87) mutations were most predominantly seen in MUM. Most GNA11 mutations were Q209L (36/41), whereas GNAQ mutations comprised Q209L (14/39) and Q209P (21/39). Epigenetic pathway mutations BAP1 (42/66), SF3B1 (11/66), FBXW7 (2/87), PBRM1 (1/66), and SETD2 (1/66) were found. No specimen had the EIF1AX mutation. Interestingly, Met-to-Death was longer in patients with GNAQ Q209P compared to GNAQ/GNA11 Q209L mutations, suggesting the difference in mutation type in GNAQ/GNA11 might determine the prognosis of MUM. Structural alterations of the GNAQ/GNA11 protein and their impact on survival of MUM patients should be further investigated.
  
  
  
  
  

---

  

# FLI1

**Signature gene in:** B cells
  
**Differentially expressed in:** no cell type
  
  
**FLI1**
-
1
of
10
  

### Utility of Protein Kinase C Beta II Immunohistochemistry in Differential Diagnosis of Ewing Sarcoma.

1. Am J Surg Pathol. 2025 Apr 15. doi: 10.1097/PAS.0000000000002400 Online ahead of print.
  
  
Lasota J(1), Krupińska M(2), Kaczorowski M(1)(3), Chłopek M(1)(4), Kinkor Z(5), Švajdler M(5)(6), Pe ...
  
  
Author information: (1)Laboratory of Pathology, National Cancer Institute, Bethesda, MD. (2)Departme ...
  
  
The diagnosis of Ewing sarcoma can be challenging, particularly when the tumor is present in an atypical location and resembles histologic mimics. The hallmark feature of Ewing sarcoma is chromosomal translocation, t(11;22)(q24;q12), involving EWSR1 and ETS gene family members. For decades, fluorescence in situ hybridization with a break-apart EWSR1 probe has been the diagnostic gold standard. However, EWSR1 rearrangements have been identified in other malignancies; thus, the detection of chimeric EWSR1 transcripts has become a preferable approach. Occasionally, insufficient tissue, severe RNA degradation, or economic constraints hamper molecular testing. This study evaluated Protein Kinase C Beta II (PKC β II) expression in >1000 tumors and assessed the utility of PKC β II immunohistochemistry in the differential diagnosis of Ewing sarcoma. Tumors harboring EWSR1::FLI1 (n=26), EWSR1::ERG, EWSR1::ETV4 (n=1), and FUS::ERG (n=6) fusions were evaluated, revealing strong diffuse immunoreactivity, although a patchy pattern was seen in 3 cases. Undifferentiated round cell sarcomas (n=46), including BCOR-, CIC-, NFATC2-, NUTM1-, and PATZ1 rearranged/fusion-sarcomas were negative. Two of the 130 synovial sarcomas, including 1 with a poorly differentiated morphology, showed diffuse, moderate-to-strong positivity. One of the 26 poorly differentiated carcinomas from the head and neck region, probably small cell lung carcinoma metastasis, showed strong PKC β II expression. Neuroblastomas (>50%) expressed PKC β II, although none showed a strong diffuse pattern. Diffuse moderate-to-strong immunoreactivity was observed in 2 sarcomatoid mesotheliomas and 2 metastatic melanomas. Diffuse but weak staining was observed in 73% (11/15) of the T-cell lymphoblastic lymphomas, including 10 CD99-positive cases. Similarly, weak predominantly patchy staining was seen in half (40/80) of other non-Hodgkin lymphomas and sporadically in embryonal rhabdomyosarcoma, Merkel cell carcinoma, small cell lung carcinoma, and Wilms tumor. Thus, diffuse and strong PKC β II immunoreactivity appears to be a reliable diagnostic marker for distinguishing classic Ewing sarcoma from histologic mimics.
  
  
  
  
**FLI1**
-
2
of
10
  

### Case report: successful treatment of primary intradural extramedullary extraskeletal Ewing sarcoma in adult patient with intralesional surgery, chemotherapy, and proton beam therapy of the cerebrospinal axis.

2. Ther Adv Med Oncol. 2024 Nov 16;16:17588359241297868. doi: 10.1177/17588359241297868 eCollection 2024.
  
  
Ziomek M(1), Placzke J(1), Urbanek K(2), Skóra T(2), Rutkowski P(1), Spałek MJ(3)(4).
  
  
Author information: (1)Department of Soft Tissue/Bone Sarcoma and Melanoma, Maria Skłodowska-Curie N ...
  
  
Ewing sarcoma is a rare malignant neoplasm that primarily affects bone in children. Extraskeletal location is less common, while intradural extramedullary Ewing sarcoma (IEES) in adults is a casuistic phenomenon. Due to its rarity, a standardized treatment strategy for IEES has not been established. The clinical use of proton beam therapy (PBT) for craniospinal irradiation (CSI) in the treatment of IEES has not been reported in the literature. A 41-year-old previously healthy man presented with disabling gluteal and lower extremity pain, decreased sensation, and progressive paraparesis without sphincter dysfunction. Imaging showed intradural extramedullary spinal lesions. The patient underwent urgent surgery. Histology and immunohistochemistry suggested a poorly differentiated neuroendocrine tumor. Negative chromogranin staining and a high Ki67 index prompted further investigation. Next-generation sequencing later confirmed an EWSR1/FLI1 translocation, leading to the diagnosis of extraskeletal Ewing sarcoma. The patient received standardized chemotherapy with marked clinical improvement. PBT CSI was initiated but was interrupted due to COVID-19 and other complications. At 20 months follow-up, no recurrence was observed, and the patient reported an active life. Despite intra-spinal spread and multiple complications, intensive chemotherapy combined with PBT CSI led to a favorable outcome. CSI rather than focal radiotherapy should be considered for patients with IEES limited to the cerebrospinal axis. PBT may be used as an alternative to photon radiotherapy to better spare organs at risk.
  
  
  
  
**FLI1**
-
3
of
10
  

### [Detection of EWSR1 gene rearrangement by fluorescence in situ hybridization in bone and soft tissue tumors: clinical application evaluation and atypical signal analysis].

3. Zhonghua Bing Li Xue Za Zhi. 2024 May 8;53(5):458-463. doi: 10.3760/cma.j.cn112151-20231025-00308
  
  
[Article in Chinese; Abstract available in Chinese from the publisher]
  
  
Li L(1), Zhang M(1), Zhang TT(1), Ding Y(1).
  
  
Author information: (1)Department of Pathology, Beijing Jishuitan Hospital, Capital Medical University, Beijing 100035, China.
  
  
  
  
**FLI1**
-
4
of
10
  

### Innovative breakthroughs facilitated by single-cell multi-omics: manipulating natural killer cell functionality correlates with a novel subcategory of melanoma cells.

4. Front Immunol. 2023 Jun 26;14:1196892. doi: 10.3389/fimmu.2023.1196892 eCollection 2023.
  
  
Zhao Z(1)(2), Ding Y(3)(4), Tran LJ(5), Chai G(1)(2), Lin L(1)(2).
  
  
Author information: (1)Department of Plastic and Reconstructive Surgery, Shanghai Ninth People's Hos ...
  
  
BACKGROUND: Melanoma is typically regarded as the most dangerous form of skin cancer. Although surgical removal of in situ lesions can be used to effectively treat metastatic disease, this condition is still difficult to cure. Melanoma cells are removed in great part due to the action of natural killer (NK) and T cells on the immune system. Still, not much is known about how the activity of NK cell-related pathways changes in melanoma tissue. Thus, we performed a single-cell multi-omics analysis on human melanoma cells in this study to explore the modulation of NK cell activity. MATERIALS AND METHODS: Cells in which mitochondrial genes comprised > 20% of the total number of expressed genes were removed. Gene ontology (GO), gene set enrichment analysis (GSEA), gene set variation analysis (GSVA), and AUCcell analysis of differentially expressed genes (DEGs) in melanoma subtypes were performed. The CellChat package was used to predict cell-cell contact between NK cell and melanoma cell subtypes. Monocle program analyzed the pseudotime trajectories of melanoma cells. In addition, CytoTRACE was used to determine the recommended time order of melanoma cells. InferCNV was utilized to calculate the CNV level of melanoma cell subtypes. Python package pySCENIC was used to assess the enrichment of transcription factors and the activity of regulons in melanoma cell subtypes. Furthermore, the cell function experiment was used to confirm the function of TBX21 in both A375 and WM-115 melanoma cell lines. RESULTS: Following batch effect correction, 26,161 cells were separated into 28 clusters and designated as melanoma cells, neural cells, fibroblasts, endothelial cells, NK cells, CD4+ T cells, CD8+ T cells, B cells, plasma cells, monocytes and macrophages, and dendritic cells. A total of 10137 melanoma cells were further grouped into seven subtypes, i.e., C0 Melanoma BIRC7, C1 Melanoma CDH19, C2 Melanoma EDNRB, C3 Melanoma BIRC5, C4 Melanoma CORO1A, C5 Melanoma MAGEA4, and C6 Melanoma GJB2. The results of AUCell, GSEA, and GSVA suggested that C4 Melanoma CORO1A may be more sensitive to NK and T cells through positive regulation of NK and T cell-mediated immunity, while other subtypes of melanoma may be more resistant to NK cells. This suggests that the intratumor heterogeneity (ITH) of melanoma-induced activity and the difference in NK cell-mediated cytotoxicity may have caused NK cell defects. Transcription factor enrichment analysis indicated that TBX21 was the most important TF in C4 Melanoma CORO1A and was also associated with M1 modules. In vitro experiments further showed that TBX21 knockdown dramatically decreases melanoma cells' proliferation, invasion, and migration. CONCLUSION: The differences in NK and T cell-mediated immunity and cytotoxicity between C4 Melanoma CORO1A and other melanoma cell subtypes may offer a new perspective on the ITH of melanoma-induced metastatic activity. In addition, the protective factors of skin melanoma, STAT1, IRF1, and FLI1, may modulate melanoma cell responses to NK or T cells.
  
  
  
  
**FLI1**
-
5
of
10
  

### FLI-1/Melan-A dual stain is an alternative to PRAME in differentiating metastatic melanoma from nodal nevus: A monocentric retrospective study.

5. J Cutan Pathol. 2023 Mar;50(3):247-258. doi: 10.1111/cup.14373 Epub 2022 Dec 21.
  
  
Zengin HB(1), Yildiz B(1), Pukhalskaya T(1), Smoller BR(1).
  
  
Author information: (1)Department of Pathology and Laboratory Medicine, University of Rochester Medi ...
  
  
Melanocytic nevi existing in lymph nodes create a diagnostic challenge by mimicking metastases. PReferentially expressed Antigen in MElanoma (PRAME) immunohistochemical (IHC) stain can differentiate one from another. FLI-1 IHC expression has been shown in malignant melanoma with variable sensitivity while melanocytic nevi were reported to be negative. We hypothesized that FLI-1/Melan-A dual IHC staining may be used in the distinction of metastatic melanoma from nodal nevi and can be an alternative and/or complementary to PRAME. In this study, we examined 13 lymph nodes with metastatic melanoma and 13 lymph nodes with benign deposits. We stained all of the lymph nodes with FLI-1, FLI-1/Melan-A dual, and PRAME IHC stains. In addition, we stained paired skin samples of the metastatic lymph nodes with FLI-1 and PRAME. In primary cutaneous melanomas, 11 of 13 were positive for FLI-1 and PRAME expression (85%). Malignant cells in 12 and 13 lymph nodes showed positive expression of PRAME and FLI-1, respectively. Only one case with a nevic cell deposit was weakly positive for FLI-1 and the remaining benign cases were negative for both FLI-1 and PRAME. Our results show that FLI-1/Melan-A dual stain is as sensitive and specific as PRAME in distinguishing lymph nodes with metastatic melanoma from nodal nevi. Further studies with larger case numbers are needed to support our significant results.
  
  
  
  
**FLI1**
-
6
of
10
  

### NRAS mutant melanoma arising in a giant congenital melanocytic nevus in an infant.

6. Indian J Pathol Microbiol. 2021 Apr-Jun;64(2):402-405. doi: 10.4103/IJPM.IJPM\_38\_20
  
  
Bansal N(1), Das M(1), Chatterjee U(1), Mallick MG(1), Arora N(2), Mishra DK(2).
  
  
Author information: (1)Department of Pathology, Institute of Post Graduate Medical Education and Res ...
  
  
Pediatric melanomas are uncommon and sometimes arise in the background of giant congenital melanocytic nevus (GCMN). A 1-year-old girl was born with GCMN affecting her left half of the face and smaller nodules affecting trunk, hands, and feet. She developed an ulcerated lesion on the left temporoparietal scalp. The lesion showed features of GCMN along with large nests of a tumor composed of round cells with a vesicular nucleus, prominent nucleolus, plentiful mitoses, and areas of necrosis. Immunostaining for desmin, LCA, CD 20, CD 34, CD 99, BCL-2, and FLI1 was negative. Tumor cells showed immunopositivity for S-100 and HMB-45 confirming the diagnosis of melanoma. Immunostaining for BRAF V600E was negative; however, NRAS mutation was detected on next-generation sequencing. Unlike adult melanomas BRAF mutations are rare but NRAS mutations have been reported in pediatric melanomas. Adjunctive molecular testing will be important to understand the genetic basis of this disease and future targeted therapy.
  
  
  
  
**FLI1**
-
7
of
10
  

### Targeting Pan-ETS Factors Inhibits Melanoma Progression.

7. Cancer Res. 2021 Apr 15;81(8):2071-2085. doi: 10.1158/0008-5472.CAN-19-1668 Epub 2021 Feb 1.
  
  
Huang L(#)(1), Zhai Y(#)(1), La J(#)(1), Lui JW(1)(2)(3), Moore SPG(1), Little EC(2), Xiao S(2), Har ...
  
  
Author information: (1)Department of Dermatology, Boston University, Boston, Massachusetts. (2)Secti ...
  
  
The failure of once promising target-specific therapeutic strategies often arises from redundancies in gene expression pathways. Even with new melanoma treatments, many patients are not responsive or develop resistance, leading to disease progression in terms of growth and metastasis. We previously discovered that the transcription factors ETS1 and PAX3 drive melanoma growth and metastasis by promoting the expression of the MET receptor. Here, we find that there are multiple ETS family members expressed in melanoma and that these factors have redundant functions. The small molecule YK-4-279, initially developed to target the ETS gene-containing translocation product EWS-FLI1, significantly inhibited cellular growth, invasion, and ETS factor function in melanoma cell lines and a clinically relevant transgenic mouse model, BrafCA;Tyr-CreERT2;Ptenf/f. One of the antitumor effects of YK-4-279 in melanoma is achieved via interference of multiple ETS family members with PAX3 and the expression of the PAX3-ETS downstream gene MET. Expression of exogenous MET provided partial rescue of the effects of YK-4-279, further supporting that MET loss is a significant contributor to the antitumor effects of the drug. This is the first study identifying multiple overlapping functions of the ETS family promoting melanoma. In addition, targeting all factors, rather than individual members, demonstrated impactful deleterious consequences in melanoma progression. Given that multiple ETS factors are known to have oncogenic functions in other malignancies, these findings have a high therapeutic impact. SIGNIFICANCE: These findings identify YK-4-279 as a promising therapeutic agent against melanoma by targeting multiple ETS family members and blocking their ability to act as transcription factors.
  
  
  
  
**FLI1**
-
8
of
10
  

### The β-Carboline Harmine Induces Actin Dynamic Remodeling and Abrogates the Malignant Phenotype in Tumorigenic Cells.

8. Cells. 2020 May 8;9(5):1168. doi: 10.3390/cells9051168
  
  
Le Moigne R(1), Subra F(1), Karam M(2), Auclair C(2)(3).
  
  
Author information: (1)Centre National de la Recherche Scientifique, CNRS UMR 8113, Laboratoire de B ...
  
  
Numerous studies have shown that alteration of actin remodeling plays a pivotal role in the regulation of morphologic and phenotypic changes leading to malignancy. In the present study, we searched for drugs that can regulate actin polymerization and reverse the malignant phenotype in cancer cells. We developed a cell-free high-throughput screening assay for the identification of compounds that induce the actin polymerization in vitro, by fluorescence anisotropy. Then, the potential of the hit compound to restore the actin cytoskeleton and reverse the malignant phenotype was checked in EWS-Fli1-transformed fibroblasts and in B16-F10 melanoma cells. A β-carboline extracted from Peganum harmala (i.e., harmine) is identified as a stimulator of actin polymerization through a mechanism independent of actin binding and requiring intracellular factors involved in a process that regulates actin kinetics. Treatment of malignant cells with non-cytotoxic concentrations of harmine induces the recovery of a non-malignant cell morphology accompanied by reorganization of the actin cytoskeleton, rescued cell-cell adhesion, inhibition of cell motility and loss of anchorage-independent growth. In conclusion, harmine induces the reversion of the malignant phenotype by a process involving the modulation of actin dynamics and is a potential anti-tumor agent acting principally through a non-cytotoxic process.
  
  
  
  
**FLI1**
-
9
of
10
  

### RhB-encapsulating silica nanoparticles modified with PEG impact the vascular endothelial function in endothelial cells and zebrafish model.

9. Sci Total Environ. 2020 Apr 1;711:134493. doi: 10.1016/j.scitotenv.2019.134493 Epub 2019 Oct 4.
  
  
Liang S(1), Chen Y(1), Zhang S(2), Cao Y(1), Duan J(3), Wang Y(4), Sun Z(5).
  
  
Author information: (1)Department of Toxicology and Sanitary Chemistry, School of Public Health, Cap ...
  
  
Silica nanoparticles (SiNPs) have been widely used in human health related products, such as food additives, cosmetics and even drug delivery, gene therapy or bioimaging. Recently, a first-in-human clinical trial based on polyethylene glycol (PEG)-modified SiNPs had been approved by US FDA to trace melanoma. However, as a nano-based drug delivery system, its biocompatibility and vascular toxicity are still largely unknown. Thus, we synthesized the fluorescent SiNPs to explore the biocompatibility and vascular endothelial function, and compare different biological effects caused by PEG-modified and unmodified SiNPs in cells and zebrafish model. The characterizations of SiNPs and PEG-modified SiNPs were analyzed by TEM, SEM, AFM and DLS, which exhibited relatively good stable and dispersive. Compared with SiNPs, PEG-modified SiNPs had markedly reduced the inflammatory response and vascular damage in Tg (fli-1: EGFP) and Tg (mpo: GFP) transgenic zebrafish lines, respectively. Consistent with the in vivo results, the PEG-modified SiNPs had been found to significantly decline the levels of ROS, inflammatory cytokines and mitochondrial-mediated apoptosis in vascular endothelial cells compared to SiNPs, and the ROS scavenger NAC could effectively alleviate the above adverse effects induced by nanoparticles. Our results suggested that the PEG-modified SiNPs could become more safety via increasing the biocompatibility and decreasing cellular toxicities in living organisms.
  
  
  
  
**FLI1**
-
10
of
10
  

### Identifying FL11 subtype by characterizing tumor immune microenvironment in prostate adenocarcinoma via Chou's 5-steps rule.

10. Genomics. 2020 Mar;112(2):1500-1515. doi: 10.1016/j.ygeno.2019.08.021 Epub 2019 Aug 28.
  
  
Yang L(1), Lv Y(2), Wang S(2), Zhang Q(2), Pan Y(2), Su D(2), Lu Q(2), Zuo Y(3).
  
  
Author information: (1)College of Bioinformatics Science and Technology, Harbin Medical University, ...
  
  
Prostate cancer is one of the leading causes of death in men worldwide, revealing a substantial heterogeneity in terms of molecular and clinical behaviors. Tumor infiltrating immune cell is associated with prognosis and response to immunotherapy in several cancer types. However, until now, the immune infiltrate profile of distinct subtypes for prostate cancer remains poorly characterized. In this study, using immune infiltration profiles as well as transcriptomic datasets, we characterized this subtype of prostate tumors. We observed that the FLI1 subtype of prostate tumors was highly enriched in immune system processes, immune related KEGG pathways and biological processes. We also expanded this approach to explore the immune infiltration profile of the high FLI1 expression subtype for skin cutaneous melanoma, similar results were found. Investigation of the association of immune infiltration features with the FLI1 expression demonstrated that many important features were associated with the FLI1 expression.
  
  
  
  
  

---

  

# FLT3

**Signature gene in:** Dendritic cells
  
**Differentially expressed in:** no cell type
  
  
**FLT3**
-
1
of
10
  

### Enhancing antitumor immunity through chemotherapeutic-derived lipid nanoparticle-induced immunogenic cell death and CD40L/Flt3L mRNA-mediated dendritic cell activation.

1. J Control Release. 2025 Jun 10;382:113684. doi: 10.1016/j.jconrel.2025.113684 Epub 2025 Apr 2.
  
  
Hou X(1), Wang C(1), Zhong Y(1), Wang L(1), Kang DD(2), Lubitz G(3), Xue Y(1), Liu Z(1), Wang S(1), ...
  
  
Author information: (1)Marc and Jennifer Lipschultz Precision Immunology Institute, Icahn School of ...
  
  
Dendritic cells (DCs) are essential for inducing effective antitumor T cell responses. However, the immunosuppressive tumor microenvironment (TME) hinders DC recruitment and maturation, facilitating tumor progression and spread. This study investigates the synergistic potential of immunogenic cell death (ICD), triggered by chemotherapeutic-derived lipid nanoparticles (LNPs), in combination with Flt3L and CD40L mRNA delivery to enhance DC mobilization and activation, reprogram the TME, and ultimately promote robust antitumor T cell responses. The optimized LNP formulation, GEM5Q7, efficiently delivered mRNA and induced ICD in melanoma cells. Intratumoral administration of GEM5Q7, encapsulating Flt3L and CD40L mRNAs, elevated pro-inflammatory cytokine and chemokine secretion, driving the infiltration and activation of cross-presenting DCs, which are critical for priming T cells. In a subcutaneous melanoma model, this approach led to significant tumor suppression and a 40 % complete response rate. This strategy holds promise for enhancing cancer immunotherapies by reprogramming the TME and inducing durable antitumor T cell immunity.
  
  
  
  
**FLT3**
-
2
of
10
  

### FLT3 mutation-related immune checkpoint molecule absent in melanoma 2 (AIM2) contributes to immune infiltration in pediatric and adult acute myeloid leukemia: evidence from bioinformatics analysis.

2. Transl Cancer Res. 2024 Nov 30;13(11):6255-6272. doi: 10.21037/tcr-24-1403 Epub 2024 Nov 27.
  
  
Zhao J(1), Cui Y(2), Zhou H(1), Zhou D(2), Che Z(2), Zhang N(2), Yun Q(2), Machado-Neto JA(3), Damia ...
  
  
Author information: (1)Department of Pediatrics, Changzhou Children's Hospital Affiliated to Nantong ...
  
  
BACKGROUND: The use of FMS-like tyrosine kinase 3 (FLT3) as a crucial target for kinase inhibitors is well established, but its association with immune infiltration remains unclear. This study aimed to explore the relationship between FLT3 mutations and immune checkpoint molecules (ICMs) in patients with acute myeloid leukemia (AML). METHODS: The Cancer Genome Atlas (TCGA) and Genotype-Tissue Expression (GTEx) databases were used to identify the ICMs associated with FLT3 mutations. A Gene Ontology (GO) analysis, Kyoto Encyclopedia of Genes and Genomes (KEGG) analysis, and gene set enrichment analysis (GSEA) were conducted to analyze the signaling pathways related to the ICMs. The single-sample GSEA (ssGSEA), Cibersort, and estimate algorithms were used to assess immune cell infiltration in AML. RESULTS: Absent in melanoma 2 (AIM2) exhibits elevated expression levels in AML patients harboring FLT3 mutation, contributing significantly to the progress of AML and establishing of an immunosuppressive microenvironment. AIM2 expression significantly correlated with sensitivity of clinically relevant drugs in ex vivo assays of AML. Additionally, AIM2 demonstrates substantial prognostic value and holds promise as a prospective immunotherapeutic target for AML. Our findings indicate a significant correlation between AIM2 and immune infiltration in AML cases, potentially affecting the presence of neutrophils, macrophages, effector memory T cells (Tem), and monocytes. Furthermore, AIM2 is closely linked to various signaling pathways, such as immune cytokine release, immune antigen presentation, and inflammasome signaling, which could play a role in immune cell enrichment in AML. CONCLUSIONS: Our study identified AMI2 as an ICM linked to FLT3 mutations. AMI2 may be involved in the activation of suppressive immune cell populations, such as macrophages, neutrophils, and monocytes. AIM2 could serve as a promising immunotherapeutic target for combination therapy with FLT3 inhibitors in AML.
  
  
  
  
**FLT3**
-
3
of
10
  

### The causal relationship between immune cells mediating FIT3L, CCL4, OSM, and skin-derived deteriorated tumors.

3. Skin Res Technol. 2024 Jul;30(7):e13774. doi: 10.1111/srt.13774
  
  
Gong W(1)(2), Zhou J(3), Hou Y(1)(2), Zhang J(1)(2), He P(2), Yu Q(1)(2).
  
  
Author information: (1)School of Management, Shanxi Medical University, Jinzhong, Shanxi, China. (2) ...
  
  
OBJECTIVE: Observational studies have identified a dual effect of circulating inflammatory proteins and immune cells on cancer progression. However, the specific mechanisms of action have not been clarified in the exacerbation of cutaneous-origin tumors. Therefore, this study aims to investigate whether the causal relationship between circulating inflammatory factors and basal cell carcinoma (BCC), cutaneous malignant melanoma (SKCM), and cutaneous squamous cell carcinoma (cSCC) is regulated by immune cells. METHODS: This study employed the Two-Sample Mendelian Randomization (TSMR) approach to investigate the causal relationships between 91 circulating inflammatory factors and three prevalent types of skin cancer from a genetic perspective. Bayesian Weighted Mendelian Randomization (BWMR) was also used to validate correlation and reverse MR to assess inverse relationships. Subsequent sensitivity analyses were conducted to limit the impact of heterogeneity and pleiotropy. Finally, the two-step Mendelian Randomization (two-step MR) method was utilized to ascertain the mediating effects of specific immune cell traits in the causal pathways linking circulating inflammatory factors with BCC, SKCM, and cSCC. RESULTS: The Inverse Variance Weighted (IVW) method and the Bayesian Weighted Algorithm collectively identified nine inflammatory factors causally associated with BCC, SKCM, and cSCC. The results from Cochran's Q test, mendelian randomization pleiotropy residual sum and outlier (MR-PRESSO), and MR-Egger intercept were not statistically significant (p < 0.05). Additionally, the proportions mediated by CD4+ CD8dim T cell %leukocyte, CD4-CD8-Natural Killer T %T cell, and CD20 on IgD-CD38-B cell for FIt3L, CCL4, and OSM were 9.26%, 8.96%, and 10.16%, respectively. CONCLUSION: Immune cell levels potentially play a role in the modulation process between circulating inflammatory proteins and cutaneous-origin exacerbated tumors. This finding offers a new perspective for the in-depth exploration of cutaneous malignancies.
  
  
  
  
**FLT3**
-
4
of
10
  

### FLT3L-dependent dendritic cells control tumor immunity by modulating Treg and NK cell homeostasis.

4. Cell Rep Med. 2023 Dec 19;4(12):101256. doi: 10.1016/j.xcrm.2023.101256
  
  
Régnier P(1), Vetillard M(2), Bansard A(3), Pierre E(4), Li X(5), Cagnard N(6), Gautier EL(7), Guerm ...
  
  
Author information: (1)Institut Necker Enfants Malades, INSERM U1151, CNRS UMR-8253, Université Pari ...
  
  
Comment in Cell Rep Med. 2024 Jan 16;5(1):101378. doi: 10.1016/j.xcrm.2023.101378.
  
  
  
  
**FLT3**
-
5
of
10
  

### Antigen-loaded Monocyte Administration and FLT3 Ligand Augment the Antitumor Efficacy of Immune Checkpoint Blockade in a Murine Melanoma Model.

5. J Immunother. 2023 Nov-Dec 01;46(9):333-340. doi: 10.1097/CJI.0000000000000487 Epub 2023 Sep 20.
  
  
D'Anniballe VM(1)(2), Huang MN(3), Lueck BD(1)(2), Nicholson LT(4), McFatridge I(1)(2), Gunn MD(1)(2 ...
  
  
Author information: (1)Department of Immunology, Duke University Medical Center, Durham, NC. (2)Divi ...
  
  
Undifferentiated monocytes can be loaded with tumor antigens (Ag) and administered intravenously to induce antitumor cytotoxic T lymphocyte (CTL) responses. This vaccination strategy exploits an endogenous Ag cross-presentation pathway, where Ag-loaded monocytes (monocyte vaccines) transfer their Ag to resident splenic dendritic cells (DC), which then stimulate robust CD8 + CTL responses. In this study, we investigated whether monocyte vaccination in combination with CDX-301, a DC-expanding cytokine Fms-like tyrosine kinase 3 ligand (Flt3L), could improve the antitumor efficacy of anti-programmed cell death (anti-PD-1) immune checkpoint blockade. We found that Flt3L expanded splenic DC over 40-fold in vivo and doubled the number of circulating Ag-specific T cells when administered before monocyte vaccination in C57BL/6 mice. In addition, OVA-monocyte vaccination combined with either anti-PD-1, anti-programmed cell death ligand 1 (anti-PD-L1), or anti-cytotoxic T lymphocyte antigen-4 (anti-CTLA-4) suppressed subcutaneous B16/F10-OVA tumor growth to a greater extent than checkpoint blockade alone. When administered together, OVA-monocyte vaccination improved the antitumor efficacy of Flt3L and anti-PD-1 in terms of circulating Ag-specific CD8 + T cell frequency and inhibition of subcutaneous B16/F10-OVA tumor growth. To our knowledge, this is the first demonstration that a cancer vaccine strategy and Flt3L can improve the antitumor efficacy of anti-PD-1. The findings presented here warrant further study of how monocyte vaccines can improve Flt3L and immune checkpoint blockade as they enter clinical trials.
  
  
  
  
**FLT3**
-
6
of
10
  

### Skin-Grafting and Dendritic Cell "Boosted" Humanized Mouse Models Allow the Pre-Clinical Evaluation of Therapeutic Cancer Vaccines.

6. Cells. 2023 Aug 18;12(16):2094. doi: 10.3390/cells12162094
  
  
Zeng B(1)(2), Moi D(1)(2), Tolley L(2), Molotkov N(2), Frazer IH(2), Perry C(3)(4), Dolcetti R(1)(2) ...
  
  
Author information: (1)Peter MacCallum Cancer Centre, Melbourne, VIC 3000, Australia. (2)Frazer Inst ...
  
  
Vaccines have been hailed as one of the most remarkable medical advancements in human history, and their potential for treating cancer by generating or expanding anti-tumor T cells has garnered significant interest in recent years. However, the limited efficacy of therapeutic cancer vaccines in clinical trials can be partially attributed to the inadequacy of current preclinical mouse models in recapitulating the complexities of the human immune system. In this study, we developed two innovative humanized mouse models to assess the immunogenicity and therapeutic effectiveness of vaccines targeting human papillomavirus (HPV16) antigens and delivering tumor antigens to human CD141+ dendritic cells (DCs). Both models were based on the transference of human peripheral blood mononuclear cells (PBMCs) into immunocompromised HLA-A\*02-NSG mice (NSG-A2), where the use of fresh PBMCs boosted the engraftment of human cells up to 80%. The dynamics of immune cells in the PBMC-hu-NSG-A2 mice demonstrated that T cells constituted the vast majority of engrafted cells, which progressively expanded over time and retained their responsiveness to ex vivo stimulation. Using the PBMC-hu-NSG-A2 system, we generated a hyperplastic skin graft model expressing the HPV16-E7 oncogene. Remarkably, human cells populated the skin grafts, and upon vaccination with a DNA vaccine encoding an HPV16-E6/E7 protein, rapid rejection targeted to the E7-expressing skin was detected, underscoring the capacity of the model to mount a vaccine-specific response. To overcome the decline in DC numbers observed over time in PBMC-hu-NSG-A2 animals, we augmented the abundance of CD141+ DCs, the specific targets of our tailored nanoemulsions (TNEs), by transferring additional autologous PBMCs pre-treated in vitro with the growth factor Flt3-L. The Flt3-L treatment bolstered CD141+ DC numbers, leading to potent antigen-specific CD4+ and CD8+ T cell responses in vivo, which caused the regression of pre-established triple-negative breast cancer and melanoma tumors following CD141+ DC-targeting TNE vaccination. Notably, using HLA-A\*02-matching PBMCs for humanizing NSG-A2 mice resulted in a delayed onset of graft-versus-host disease and enhanced the efficacy of the TNE vaccination compared with the parental NSG strain. In conclusion, we successfully established two humanized mouse models that exhibited strong antigen-specific responses and demonstrated tumor regression following vaccination. These models serve as valuable platforms for assessing the efficacy of therapeutic cancer vaccines targeting HPV16-dysplastic skin and diverse tumor antigens specifically delivered to CD141+ DCs.
  
  
  
  
**FLT3**
-
7
of
10
  

### Design, synthesis, and biological evaluation with molecular dynamics study of novel pyrazolo[3,4-d]pyrimidine derivatives as anti-cancer agents.

7. RSC Adv. 2023 Jun 7;13(25):17074-17096. doi: 10.1039/d3ra00446e eCollection 2023 Jun 5.
  
  
Shaban RM(1), Samir N(2), Nissan YM(1)(3), Abouzid KAM(2).
  
  
Author information: (1)Pharmaceutical Organic Chemistry Department, Faculty of Pharmacy, October Uni ...
  
  
In continuation of our efforts to discover new structural chemotypes with significant chemotherapeutic activities, a novel series of pyrazolo[3,4-d]pyrimidine-based compounds linked to a piperazine ring, bearing different aromatic moieties, through different linkages was designed and synthesized as FLT3 inhibitors. All of the newly synthesized compounds were evaluated for their cytotoxicity on 60-NCI cell lines. Compounds with the piperazine acetamide linkage XIIa-f & XVI exhibited a remarkable anticancer activity among all of the tested compounds, especially against non-small cell lung cancer, melanoma, leukemia and renal cancer models. Furthermore, compound XVI (NSC no - 833644) was further screened with a 5-dose assay on nine subpanels and exhibited a GI50 between 1.17 and 18.40 μM. On the other hand, molecular docking and dynamics studies were performed to predict the binding mode of the newly synthesized compounds in the FLT3 binding domain. Finally, through a predictive kinetic study, several ADME descriptors were calculated.
  
  
  
  
**FLT3**
-
8
of
10
  

### Enrichment of Large Numbers of Splenic Mouse Dendritic Cells After Injection of Flt3L-Producing Tumor Cells.

8. Methods Mol Biol. 2023;2618:173-186. doi: 10.1007/978-1-0716-2938-3\_13
  
  
Santa P(1), Roubertie A(1), Loizon S(1), Garreau A(1), Ferriere A(1), Duluc D(2), Sisirak V(3).
  
  
Author information: (1)Université de Bordeaux, CNRS, ImmunoConcEpT, UMR 5164, Bordeaux, France. (2)U ...
  
  
Dendritic cells (DCs) are antigen-presenting cells (APCs) that shape innate and adaptive immunity. There are multiple subsets of DCs distinguished according to their phenotype and functional specialization. DCs are present in lymphoid organs and across multiple tissues. However, their frequency and numbers at these sites are very low making their functional study difficult. Multiple protocols have been developed to generate DCs in vitro from bone marrow progenitors, but they do not fully recapitulate DC complexity found in vivo. Therefore, directly amplifying endogenous DCs in vivo appears as an option to overcome this specific caveat. In this chapter, we describe a protocol to amplify murine DCs in vivo by the injection of a B16 melanoma cell line expressing the trophic factor FMS-like tyrosine kinase 3 ligand (Flt3L). We have also compared two methods of magnetic sorting of amplified DCs, both giving high yields of total murine DCs, but different representation of the main DC subsets found in vivo.
  
  
  
  
**FLT3**
-
9
of
10
  

### Exome sequencing analysis of gastric primary myeloid sarcoma with monocytic differentiation with altered immunophenotype after chemotherapy: case report.

9. Diagn Pathol. 2023 Mar 4;18(1):35. doi: 10.1186/s13000-023-01311-1
  
  
Li X(#)(1), Zhang H(#)(2), Cui Y(#)(3), Zhang H(1), Wang Y(1), Ding M(1), Zhu X(1), Zhang R(1), Hu Q ...
  
  
Author information: (1)Department of Pathology, The First Affiliated Hospital, Shihezi University Sc ...
  
  
Erratum in Diagn Pathol. 2023 Mar 28;18(1):38. doi: 10.1186/s13000-023-01326-8.
  
  
  
  
**FLT3**
-
10
of
10
  

### Analyzing Prognostic Hub Genes in the Microenvironment of Cutaneous Melanoma by Computer Integrated Bioinformatics.

10. Comput Intell Neurosci. 2022 Mar 8;2022:4493347. doi: 10.1155/2022/4493347 eCollection 2022.
  
  
Li G(1), Zhang J(1), Liu Y(1), Cheng X(1), Sun K(1), Hong W(1), Sha K(2).
  
  
Author information: (1)Affiliated Hospital of Jiujiang University, No. 57 Xunyang East Road, Jiujian ...
  
  
Cutaneous melanoma (CM) is attracting increasing attention due to high mortality. In response to this, we synthetically analyze the CM dataset from the TCGA database and explore microenvironment-related genes that effectively predict patient prognosis. Immune/stromal scores of cases are calculated using the ESTIMATE algorithm and are significantly associated with overall patient survival. Then, differentially expressed genes are identified by comparing the immune score and stromal score, also prognostic genes are subsequently screened. Functional analysis shows that these genes are enriched in different activities of immune system. Moreover, 19 prognosis-related hub genes are extracted from the protein-protein interaction network, of which four unreported genes (IL7R, FLT3, C1QC, and HLA-DRB5) are chosen for validation. A significant negative relationship is found between the expression levels of the 4 genes and pathological stages, notably T grade. Furthermore, the K-M plots and TIMER results show that these genes have favorable value for CM prognosis. In conclusion, these results give a novel insight into CM and identify IL7R, FLT3, C1QC, and HLA-DRB5 as crucial roles for the diagnosis and treatment of CM.
  
  
  
  
  

---

  

# FOS

**Signature gene in:** Endothelial cells
  
**Differentially expressed in:** no cell type
  
  
**FOS**
-
1
of
10
  

### Lactylation of LSD1 is an acquired epigenetic vulnerability of BRAFi/MEKi-resistant melanoma.

1. Dev Cell. 2025 Mar 20:S1534-5807(25)00121-2. doi: 10.1016/j.devcel.2025.02.016 Online ahead of print.
  
  
Li A(1), Gong Z(2), Long Y(1), Li Y(3), Liu C(1), Lu X(1), Li Q(1), He X(4), Lu H(5), Wu K(6), Nie Y ...
  
  
Author information: (1)State Key Laboratory of Cellular Stress Biology, School of Life Sciences, Xia ...
  
  
BRAFV600E mutant melanomas treated with BRAF inhibitor (BRAFi) and MEK inhibitor (MEKi) almost invariably develop drug resistance, accompanied by restored glucose metabolism. How resumed glycolysis controls acquired resistance remains unknown. Here, we identify that lysine-specific demethylase 1 (LSD1) lactylation, induced by re-accumulated lactate in both human and murine BRAFi/MEKi-resistant melanoma cells, selectively drives survival via epigenetic reprogramming. Mechanistically, lactylation of LSD1 promotes its interaction with Fos-related antigen 1 (FosL1), preventing its degradation by E3 ligase tripartite-motif-containing protein 21 (TRIM21) and selectively enhancing its genomic enrichment. We further demonstrate that lactylated LSD1 co-directs gene transcription with FosL1 to repress ferroptosis via interfering with transferrin receptor protein 1 (TFRC)-mediated iron uptake. LSD1 inhibition activates ferroptosis, resulting in drastic regression of drug-resistant murine melanoma when combined with immunotherapy. Our results highlight a crucial role of metabolic rewiring-induced epigenetic reprogramming as a bypass resistance mechanism in BRAFi/MEKi-resistant melanoma, providing a therapeutically actionable strategy to overcome resistance to targeted therapy and immunotherapy.
  
  
  
  
**FOS**
-
2
of
10
  

### Mitochondrial respiratory complex III sustains IL-10 production in activated macrophages and promotes tumor-mediated immune evasion.

2. Sci Adv. 2025 Jan 24;11(4):eadq7307. doi: 10.1126/sciadv.adq7307 Epub 2025 Jan 22.
  
  
Zotta A(1), Toller-Kawahisa J(1), Palsson-McDermott EM(1), O'Carroll SM(1), Henry ÓC(1), Day EA(1)(2 ...
  
  
Author information: (1)School of Biochemistry and Immunology, Trinity Biomedical Sciences Institute, ...
  
  
The cytokine interleukin-10 (IL-10) limits the immune response and promotes resolution of acute inflammation. Because of its immunosuppressive effects, IL-10 up-regulation is a common feature of tumor progression and metastasis. Recently, IL-10 regulation has been shown to depend on mitochondria and redox-sensitive signals. We have found that Suppressor of site IIIQo Electron Leak 1.2 (S3QEL 1.2), a specific inhibitor of reactive oxygen species (ROS) production from mitochondrial complex III, and myxothiazol, a complex III inhibitor, decrease IL-10 in lipopolysaccharide (LPS)-activated macrophages. IL-10 down-regulation is likely to be mediated by suppression of c-Fos, which is a subunit of activator protein 1 (AP1), a transcription factor required for IL-10 gene expression. S3QEL 1.2 impairs IL-10 production in vivo after LPS challenge and promotes the survival of mice bearing B16F10 melanoma by lowering tumor growth. Our data identify a link between complex III-dependent ROS generation and IL-10 production in macrophages, the targeting of which could have potential in boosting antitumor immunity.
  
  
  
  
**FOS**
-
3
of
10
  

### Curcumin alleviates inflammatory effects of ketamine anesthesia in postnatal rats.

3. Vet Res Forum. 2024;15(9):473-480. doi: 10.30466/vrf.2024.2018359.4107 Epub 2024 Sep 15.
  
  
Afshar Ghahremani S(1), Raisi A(1), Minaei Beirami S(2)(3), Kahroba H(4), Mardani M(5), Dezfoulian O ...
  
  
Author information: (1)Department of Clinical Sciences, Faculty of Veterinary Medicine, Lorestan Uni ...
  
  
Curcumin has been employed in traditional medicine for over a millennium to treat various ailments, and its global use is now widespread. Chinese medicine relies heavily on curcumin as a primary element and uses it to cure infectious diseases, skin disorders, depression, and stress. It has cardioprotective, neuroprotective, and anti-diabetic properties, as well as pharmacological effects on disorders like type II diabetes, atherosclerosis, and human immunodeficiency virus replication. The anti-cancer activity of curcumin has been studied extensively with notable improvements in gastrointestinal, melanoma, urogenital, breast, and lung malignancies. We investigated the anti-inflammatory effects of curcumin on expression of tumor necrosis factor (TNF)-α, c-Fos, and interleukin (IL)-6 genes in brain and liver tissue owing to the effects of ketamine anesthesia on postnatal rats. The thalamic and hepatic tissues were collected without anesthesia, immediately after anesthesia, and 4 and 12 hr after anesthesia in control and curcumin treated postnatal rats. The results showed that glucose, triglyceride, high- and low-density lipoprotein levels were lowered with curcumin treatment. We also found that ketamine increased c-Fos and inflammatory cytokines like TNF-α and IL-6, all of which contribute to inflammation. Brain and liver immunohistochemistry studies confirmed the real-time polymerase chain reaction findings. Curcumin injections alone may be effective in decreasing ketamine-induced inflammation in both brain and liver tissues.
  
  
  
  
**FOS**
-
4
of
10
  

### Tumor-infiltrating nerves functionally alter brain circuits and modulate behavior in a mouse model of head-and-neck cancer.

4. Elife. 2024 Sep 20;13:RP97916. doi: 10.7554/eLife.97916
  
  
Barr J(#)(1), Walz A(#)(1), Restaino AC(1)(2), Amit M(3), Barclay SM(1), Vichaya EG(4), Spanos WC(1) ...
  
  
Author information: (1)Sanford Research, Cancer Biology and Immunotherapies Group, Sioux Falls, Siou ...
  
  
Update of bioRxiv. 2024 Jun 07:2023.10.18.562990. doi: 10.1101/2023.10.18.562990.
  
  
  
  
**FOS**
-
5
of
10
  

### Eravacycline improves the efficacy of anti-PD1 immunotherapy via AP1/CCL5 mediated M1 macrophage polarization in melanoma.

5. Biomaterials. 2025 Mar;314:122815. doi: 10.1016/j.biomaterials.2024.122815 Epub 2024 Sep 11.
  
  
Liu N(1), Yan M(1), Lu C(1), Tao Q(1), Wu J(1), Zhou Z(2), Chen J(3), Chen X(4), Peng C(5).
  
  
Author information: (1)Department of Dermatology, Xiangya Hospital, Central South University, Changs ...
  
  
Screening approved library is a promising and safe strategy to overcome the limitation of low response rate and drug resistance in immunotherapy. Accumulating evidence showed that the application of antibiotics has been considered to reduce the effectiveness of anti-PD1 immunotherapy in tumor treatment, however, in this study, an antibiotic drug (Eravacycline, ERV) was identified to improve the efficacy of anti-PD1 immunotherapy in melanoma through screening approved library. Administration of ERV significantly attenuated melanoma cells growth as well as directly or indirectly benefited M1 macrophage polarization. Meanwhile, ERV treatment significantly induced cellular autophagy via damage of mitochondria, leading to up-regulation of ROS production, subsequently, raised CCL5 secretion through elevation AP1 binding to CCL5 promoter via p38 or JNK1/2 activation. Knockdown of Ccl5 expression attenuated ERV triggered M1 macrophage polarization in melanoma cells. Clinical analysis revealed a positive association between high expression of CCL5 and improved prognosis as well as a favorable anti-PD1 therapy in melanoma patients. As expected, application of ERV improved the efficacy of anti-PD1. Overall, our results approved that ERV enhances the efficacy of anti-PD1 immunotherapy in melanoma by promoting the polarization of M1 macrophages, which provided novel therapeutic strategy for improving the effectiveness of melanoma anti-PD1 immunotherapy.
  
  
  
  
**FOS**
-
6
of
10
  

### The Effect of Cell-Free DNA from Blood Serum of Mice with Metastatic Melanoma on Enhancement of Oncogenic Properties of Melanoma Cells.

6. Biochemistry (Mosc). 2023 Jul;88(7):995-1007. doi: 10.1134/S0006297923070118
  
  
Filatova AA(1)(2), Alekseeva LA(3), Savin IA(4), Sen'kova AV(5), Zenkova MA(6), Mironova NL(7).
  
  
Author information: (1)Institute of Chemical Biology and Fundamental Medicine, Siberian Branch of th ...
  
  
Currently, a significant increase in the levels of circulating cell-free DNA (cfDNA) in the blood of patients is considered as a generally recognized marker of the development of oncological diseases. Although the tumor-associated cfDNA has been well studied, its biological functions remain unclear. In this work, we investigated the effect of cfDNA isolated from the blood serum of the mice with B16-F10 metastatic melanoma on the properties of the B16-F10 melanoma cells in vitro. It was found that the profile of cfDNA isolated from the blood serum of mice with melanoma differs significantly from the cfDNA isolated from the blood serum of healthy mice, and is similar to the genomic DNA of B16 cells with regards to abundance of oncogenes and mobile genetic elements (MGE). It was shown that the cfDNA of mice with melanoma penetrated into B16 cells, resulting in the increase in abundance of oncogenes and MGE fragments, and caused 5-fold increase of the mRNA level of the secreted DNase Dnase1l3 and a slight increase of the mRNA level of the Jun, FOS, Ras, and Myc oncogenes. cfDNA of the healthy mice caused increase of the mRNA level of intracellular regulatory DNase EndoG and 4-fold increase of the mRNA level of FOS and Ras oncogenes, which are well-known triggers of a large number of signal cascades, from apoptosis inhibition to increased tumor cell proliferation. Thus, it is obvious that the circulating cfDNA of tumor origin is able to penetrate into the cells and, despite the fact that no changes were found in the level of viability and migration activity of the tumor cells, cfDNA, even with a single exposure, can cause changes at the cellular level that increase oncogenicity of the recipient cells.
  
  
  
  
**FOS**
-
7
of
10
  

### Synthesis of Tricyclic Pterolobirin H Analogue: Evaluation of Anticancer and Anti-Inflammatory Activities and Molecular Docking Investigations.

7. Molecules. 2023 Aug 23;28(17):6208. doi: 10.3390/molecules28176208
  
  
Zentar H(1)(2), Jannus F(2), Medina-O'Donnell M(2), El Mansouri AE(3), Fernández A(1), Justicia J(1) ...
  
  
Author information: (1)Departamento de Química Orgánica, Facultad de Ciencias, Instituto de Biotecno ...
  
  
Pterolobirin H (3), a cassane diterpene isolated from the roots of Pterolobium macropterum, exhibits important anti-inflammatory and anticancer properties. However, its relatively complex tetracyclic structure makes it difficult to obtain by chemical synthesis, thus limiting the studies of its biological activities. Therefore, we present here a short route to obtain a rational simplification of pterolobirin H (3) and some intermediates. The anti-inflammatory activity of these compounds was assayed in LPS-stimulated RAW 264.7 macrophages. All compounds showed potent inhibition of NO production, with percentages between 54 to 100% at sub-cytotoxic concentrations. The highest anti-inflammatory effect was shown for compounds 15 and 16. The simplified analog 16 revealed potential NO inhibition properties, being 2.34 higher than that of natural cassane pterolobirin H (3). On the other hand, hydroxyphenol 15 was also demonstrated to be the strongest NO inhibitor in RAW 264.7 macrophages (IC50 NO = 0.62 ± 0.21 μg/mL), with an IC50NO value 28.3 times lower than that of pterolobirin H (3). Moreover, the anticancer potential of these compounds was evaluated in three cancer cell lines: HT29 colon cancer cells, Hep-G2 hepatoma cells, and B16-F10 murine melanoma cells. Intermediate 15 was the most active against all the selected tumor cell lines. Compound 15 revealed the highest cytotoxic effect with the lowest IC50 value (IC50 = 2.45 ± 0.29 μg/mL in HT29 cells) and displayed an important apoptotic effect through an extrinsic pathway, as evidenced in the flow cytometry analysis. Furthermore, the Hoechst staining assay showed that analog 15 triggered morphological changes, including nuclear fragmentation and chromatin condensation, in treated HT29 cells. Finally, the in silico studies demonstrated that cassane analogs exhibit promising binding affinities and docking performance with iNOS and caspase 8, which confirms the obtained experimental results.
  
  
  
  
**FOS**
-
8
of
10
  

### Eubacterium rectale Improves the Efficacy of Anti-PD1 Immunotherapy in Melanoma via l-Serine-Mediated NK Cell Activation.

8. Research (Wash D C). 2023 Apr 28;6:0127. doi: 10.34133/research.0127 eCollection 2023.
  
  
Liu N(1)(2)(3)(4)(5), Chen L(1)(5), Yan M(1)(2)(3)(4)(5), Tao Q(1)(3)(4), Wu J(1)(3)(4), Chen J(1)(3 ...
  
  
Author information: (1)Department of Clinical Pharmacology, Xiangya Hospital, Central South Universi ...
  
  
Natural killer (NK) cells, as key immune cells, play essential roles in tumor cell immune escape and immunotherapy. Accumulating evidence has demonstrated that the gut microbiota community affects the efficacy of anti-PD1 immunotherapy and that remodeling the gut microbiota is a promising strategy to enhance anti-PD1 immunotherapy responsiveness in advanced melanoma patients; however, the details of the mechanism remain elusive. In this study, we found that Eubacterium rectale was significantly enriched in melanoma patients who responded to anti-PD1 immunotherapy and that a high E. rectale abundance was related to longer survival in melanoma patients. Furthermore, administration of E. rectale remarkably improved the efficacy of anti-PD1 therapy and increased the overall survival of tumor-bearing mice; moreover, application of E. rectale led to a significant accumulation of NK cells in the tumor microenvironment. Interestingly, conditioned medium isolated from an E. rectale culture system dramatically enhanced NK cell function. Gas chromatography-mass spectrometry/ultrahigh performance liquid chromatography-tandem mass spectrometry-based metabolomic analysis showed that l-serine production was significantly decreased in the E. rectale group; moreover, administration of an l-serine synthesis inhibitor dramatically increased NK cell activation, which enhanced anti-PD1 immunotherapy effects. Mechanistically, supplementation with l-serine or application of an l-serine synthesis inhibitor affected NK cell activation through FOS/Fosl. In summary, our findings reveal the role of bacteria-modulated serine metabolic signaling in NK cell activation and provide a novel therapeutic strategy to improve the efficacy of anti-PD1 immunotherapy in melanoma.
  
  
  
  
**FOS**
-
9
of
10
  

### GDNF family receptor alpha-like antagonist antibody alleviates chemotherapy-induced cachexia in melanoma-bearing mice.

9. J Cachexia Sarcopenia Muscle. 2023 Jun;14(3):1441-1453. doi: 10.1002/jcsm.13219 Epub 2023 Apr 5.
  
  
Lee BY(1), Jeong J(1), Jung I(1), Cho H(2), Jung D(3), Shin J(1), Park JK(1), Park E(1), Noh S(1), S ...
  
  
Author information: (1)Department of New Biology, Daegu Gyeongbuk Institute of Science and Technolog ...
  
  
BACKGROUND: Patients with cancer undergoing chemotherapy experience cachexia with anorexia, body weight loss, and the depletion of skeletal muscles and adipose tissues. Effective treatment strategies for chemotherapy-induced cachexia are scarce. The growth differentiation factor 15 (GDF15)/GDNF family receptor alpha-like (GFRAL)/rearranged during transfection (RET) axis is a critical signalling pathway in chemotherapy-induced cachexia. In this study, we developed a fully human GFRAL antagonist antibody and investigated whether it inhibits the GDF15/GFRAL/RET axis, thereby alleviating chemotherapy-induced cachexia in tumour-bearing mice. METHODS: Anti-GFRAL antibodies were selected via biopanning, using a human combinatorial antibody phage library. The potent GFRAL antagonist antibody A11 was selected via a reporter cell assay and its inhibitory activity of GDF15-induced signalling was evaluated using western blotting. To investigate the in vivo function of A11, a tumour-bearing mouse model was established by inoculating 8-week-old male C57BL/6 mice with B16F10 cells (n = 10-16 mice per group). A11 was administered subcutaneously (10 mg/kg) 1 day before intraperitoneal treatment with cisplatin (10 mg/kg). Animals were assessed for changes in food intake, body weight, and tumour volume. Plasma and key metabolic tissues such as skeletal muscles and adipose tissues were collected for protein and mRNA expression analysis. RESULTS: A11 reduced serum response element-luciferase reporter activity up to 74% (P < 0.005) in a dose-dependent manner and blocked RET phosphorylation up to 87% (P = 0.0593), AKT phosphorylation up to 28% (P = 0.0593) and extracellular signal regulatory kinase phosphorylation up to 75% (P = 0.0636). A11 inhibited the action of cisplatin-induced GDF15 on the brainstem and decreased GFRAL-positive neuron population expressing c-Fos in the area postrema and nucleus of the solitary tract by 62% in vivo (P < 0.05). In a melanoma mouse model treated with cisplatin, A11 recovered anorexia by 21% (P < 0.05) and tumour-free body weight loss by 13% (P < 0.05). A11 significantly improved the cisplatin-induced loss of skeletal muscles (quadriceps: 21%, gastrocnemius: 9%, soleus: 13%, P < 0.05) and adipose tissues (epididymal white adipose tissue: 37%, inguinal white adipose tissue: 51%, P < 0.05). CONCLUSIONS: Our study suggests that GFRAL antagonist antibody may alleviate chemotherapy-induced cachexia, providing a novel therapeutic approach for patients with cancer experiencing chemotherapy-induced cachexia.
  
  
  
  
**FOS**
-
10
of
10
  

### Epigenetic modification of gene expression in cancer cells by terahertz demethylation.

10. Sci Rep. 2023 Mar 26;13(1):4930. doi: 10.1038/s41598-023-31828-w
  
  
Cheon H(1), Hur JK(2), Hwang W(3), Yang HJ(4), Son JH(5).
  
  
Author information: (1)Biomedical Engineering Research Center, Asan Medical Center, 88, Olympic-ro 4 ...
  
  
Terahertz (THz) radiation can affect the degree of DNA methylation, the spectral characteristics of which exist in the terahertz region. DNA methylation is an epigenetic modification in which a methyl (CH3) group is attached to cytosine, a nucleobase in human DNA. Appropriately controlled DNA methylation leads to proper regulation of gene expression. However, abnormal gene expression that departs from controlled genetic transcription through aberrant DNA methylation may occur in cancer or other diseases. In this study, we demonstrate the modification of gene expression in cells by THz demethylation using resonant THz radiation. Using an enzyme-linked immunosorbent assay, we observed changes in the degree of global DNA methylation in the SK-MEL-3 melanoma cell line under irradiation with 1.6-THz radiation with limited spectral bandwidth. Resonant THz radiation demethylated living melanoma cells by 19%, with no significant occurrence of apurinic/apyrimidinic sites, and the demethylation ratio was linearly proportional to the power of THz radiation. THz demethylation downregulates FOS, JUN, and CXCL8 genes, which are involved in cancer and apoptosis pathways. Our results show that THz demethylation has the potential to be a gene expression modifier with promising applications in cancer treatment.
  
  
  
  
  

---

  

# HMGB1

**Signature gene in:** CD4+ T cells
  
**Differentially expressed in:** no cell type
  
  
**HMGB1**
-
1
of
10
  

### A fermented Mistletoe (Viscum album L.) extract elicits markers characteristic for immunogenic cell death driven by endoplasmic reticulum stress in vitro.

1. BMC Complement Med Ther. 2025 May 14;25(1):175. doi: 10.1186/s12906-025-04909-8
  
  
Weissenstein U(1), Tschumi S(2), Leonhard B(2), Baumgartner S(2)(3).
  
  
Author information: (1)Society for Cancer Research, Arlesheim, Switzerland. u.weissenstein@vfk.ch. ( ...
  
  
BACKGROUND: Immune evasion is a characteristic hallmark of cancer. Immunotherapies aim to activate and support the body's immune system to recognize and fight tumor cells. Induction of immunogenic cell death (ICD) and the associated activation of danger signaling pathways can increase the immunogenicity of tumor cells. Therapeutic ICD stimuli activate endoplasmic reticulum stress pathways and apoptosis leading to the cellular expression of damage-associated molecular patterns (DAMPs). The aim of our in vitro study was to investigate whether mistletoe extracts induce characteristics of immunogenic tumor cell death in cancer cell lines. METHODS: Three human breast cancer cell lines and one murine melanoma cell line (SKBR3, MDA-MB-231, MCF-7, and B16F10) were treated with aqueous, fermented Viscum album extract (VAE: Iscador Qu spec.) and taxol or tunicamycin as positive controls, respectively. To investigate whether VAE induces ribotoxic stress, we measured the ER stress regulators p-eIF2a, ATF4, and CHOP by Western blot. Cell surface exposure of DAMPs (calreticulin, heat shock proteins hsp70 and hsp90), apoptosis and induction of mitochondrial reactive oxygen species (ROS) were assessed by flow cytometry. HMGB1 and ATP were quantified by ELISA and chemiluminescence assay, respectively. RESULTS: Treatment with VAE resulted in phosphorylation of eIF2α in all cancer cell lines tested and increased calreticulin (CRT) exposure on the surface of pre-apoptotic SKBR3 breast cancer and B16F10 mouse melanoma cells. VAE exerted a concentration-dependent effect in all cell lines, resulting in a significantly increased exposure of three DAMPs (CRT, hsp70 and hsp90) on the surface of early apoptotic cells. Furthermore, VAE elevated mitochondrial ROS production and the release of ATP. HMGB1 release was not induced by VAE. CONCLUSIONS: In this in vitro study, we demonstrated for the first time the potential of a mistletoe extract to induce surrogate markers of immunogenic cancer cell death. This is a primary step in investigating the potential of VAEs to contribute to ICD-induced tumor-specific immune activation.
  
  
  
  
**HMGB1**
-
2
of
10
  

### Blocking Nitrosylation Induces Immunogenic Cell Death by Sensitizing NRAS-Mutant Melanoma to MEK Inhibitors.

2. Cancer Res. 2025 Apr 27. doi: 10.1158/0008-5472.CAN-24-0693 Online ahead of print.
  
  
Srivastava J(1), Yadav VK(2), Jimenez RV(1), Phadatare PR(3), Inamdar NA(4), Young MM(5), Bacchiocch ...
  
  
Author information: (1)Moffitt Cancer Center, Tampa, FL, United States. (2)Moffitt Cancer Center, ta ...
  
  
Activating NRAS mutations occur in 15-25% of all melanomas. However, this subtype remains refractory to existing therapeutics, including immunotherapy and RAS inhibitors; therefore, identifying innovative treatment strategies is of utmost importance. We investigated the role of nitrosylation, a nitric oxide-induced post-translational modification, in melanoma progression and therapeutic resistance. Inhibiting nitrosylation sensitized NRAS-mutant melanomas to targeted MEK inhibitors (MEKi), leading to sustained downregulation of the ERK-MAPK pathway, along with concomitant de-nitrosylation of NRAS, MEK, ERK, RSK1, and DUSPs. Global nitrosylome profiling using mass-spectrometry revealed nitrosylation of multiple ERK regulators. Gain- and loss-of-function studies confirmed a positive association between nitrosylation and ERK activation. ERK and MEK proteins harbored potential nitrosylation sites, mutation of which abrogated their phosphorylation and inhibited cell growth. The nitrosylome also contained death-associated molecular patterns (DAMPs), factors known to induce immunogenic cell death (ICD). Notably, nitrosylation inhibition combined with MEKi markedly inhibited Nras-mutant melanoma growth in an immunocompetent mouse model. This was accompanied by downregulated MEK-ERK signaling and extracellular release of DAMPs like calreticulin, phospho-eIF2α, and HMGB1, confirming ICD induction. Furthermore, the combination significantly increased the repertoire of CD8+ T cells, dendritic cells (DCs), and macrophages in the tumor microenvironment, which was validated in co-cultures of DCs and T-lymphocytes. In conclusion, the current study demonstrates that nitrosylation inhibition sensitizes NRAS-mutant melanomas to targeted MEKi-induced cell death and causes the release of non-nitrosylated (active) DAMPs that induce a potent anti-melanoma immune response via ICD. These findings highlight potential therapeutic vulnerabilities in the currently untreatable NRAS-mutant melanoma subtype.
  
  
  
  
**HMGB1**
-
3
of
10
  

### Nucleus-targeted ruthenium(II) complex triggers immunogenic cell death and sensitizes melanoma to anti-PD-1 therapy by activating cGAS-STING pathway.

3. J Inorg Biochem. 2025 Jun;267:112871. doi: 10.1016/j.jinorgbio.2025.112871 Epub 2025 Feb 25.
  
  
Wang B(1), Tang X(1), Xiao C(1), Yu Z(1), Bo H(1), Wang J(2), Wang J(3).
  
  
Author information: (1)Guangdong Provincial Key Laboratory for Research and Evaluation of Pharmaceut ...
  
  
A significant challenge in the treatment of melanoma with immune checkpoint blockades (ICBs) is the limited T cells response often observed in immunologically "cold" tumors. By leveraging the immunogenicity of immunogenic cell death (ICD), which increases the susceptibility of tumor cells to ICBs, this study investigated the potential of a nucleus-targeted ruthenium(II) complex (Ru1) as an inducer of ICD. Treatment with Ru1 induced DNA damage in melanoma cells, activating the cyclic GMP-AMP synthase-stimulator of the interferon genes (cGAS-STING) pathway. This triggered endoplasmic reticulum (ER) stress, leading to ICD. Ru1-treated dying melanoma cells exhibited characteristics such as cell exposure of calreticulin (CRT) on the cell surface, release of adenosine triphosphate (ATP), and secretion of high-mobility group box 1 (HMGB1). Vaccination with Ru1-treated, dying melanoma cells elicited robust antitumor immune responses, as evidenced by CD8+ T cells activation, reduced Foxp3+ T cells count, and the development of a memory immune response that protected mice from subsequent melanoma challenges. Combining Ru1 with anti-PD-1 therapy significantly promoted T cells infiltration, enhanced dendritic cell activation, and reduced tumor-associated immunosuppressive factors, indicating a reprogramming of the tumor microenvironment. These findings suggest that Ru1 is a promising therapeutic agent for treating "cold" tumors in cancer chemoimmunotherapy.
  
  
  
  
**HMGB1**
-
4
of
10
  

### Effectiveness and Safety of Oral Compound Glycyrrhizin Followed by Phototherapy for the Treatment of Progressive Vitiligo in Children.

4. Pigment Cell Melanoma Res. 2025 Mar;38(2):e13226. doi: 10.1111/pcmr.13226
  
  
Zhang L(1), Zhang J(1), Liu S(1), Shi Z(1), Zhu Y(1), Jiang M(1), Xiang L(1).
  
  
Author information: (1)Department of Dermatology, Huashan Hospital, Fudan University, Shanghai, Chin ...
  
  
Childhood vitiligo, distinct from its adult counterpart, presents unique treatment challenges. Glycyrrhizin inhibits the release of high-mobility group box 1 (HMGB1) protein from keratinocytes, preventing melanocyte apoptosis and autophagy. Furthermore, the orally administered compound glycyrrhizin (OCG) effectively treats various autoimmune disorders, demonstrating long-term efficacy, safety, and tolerability. This study compared the efficacy of OCG and oral prednisone (OP), followed by phototherapy, in patients with progressive childhood vitiligo at 52 weeks' follow-up. Fifty children with vitiligo were randomized into two groups according to treatment: OCG (50-150 mg/day) followed by phototherapy (n = 25) and OP (5-10 mg/day) followed by phototherapy (n = 25). At Week 24, a halt in disease progression (HDP) was observed in 20 (80%) patients in the OCG group and 21 (84%) in the OP group, with no significant difference (p > 0.99). However, the mean time to achieve HDP was significantly shorter in the OP group than in the OCG group (14.73 ± 4.84 vs. 19.13 ± 4.82 weeks; p < 0.01). In addition, serum HMGB1 concentrations were significantly reduced after treatment with OCG at Week 24 (3.02 ± 0.83 vs. 0.95 ± 0.36 ng/mL [p < 0.01]; OP, 2.79 ± 0.16 vs. 1.03 ± 0.34 ng/mL [p < 0.01]). The decline in Vitiligo Area Scoring Index (VASI) score at the end of follow-up (i.e., Week 52) did not show a statistically significant difference between the OCG and OP groups (52.31% ± 14.86% vs. 55.71% ± 21.23%; p = 0.55). The therapeutic response of the clinical markers of progression was good and comparable between the OCG and OP groups. OCG demonstrated similar efficacy to OP followed by phototherapy in controlling disease activity and promoting repigmentation in children with vitiligo at 52 weeks of follow-up. Trial Registration: ChiCTR2400086844.
  
  
  
  
**HMGB1**
-
5
of
10
  

### Tumor Vaccine Exploiting Membranes with Influenza Virus-Induced Immunogenic Cell Death to Decorate Polylactic Coglycolic Acid Nanoparticles.

5. ACS Nano. 2025 Jan 28;19(3):3115-3134. doi: 10.1021/acsnano.4c00654 Epub 2025 Jan 13.
  
  
Yang Y(1), Hu Y(1)(2), Yang Y(1)(3), Liu Q(1)(3), Zheng P(1), Yang Z(1), Duan B(1)(3), He J(1), Li W ...
  
  
Author information: (1)Laboratory of Molecular Immunology, Institute of Medical Biology, Chinese Aca ...
  
  
Immunogenic cell death (ICD) of tumor cells, which is characterized by releasing immunostimulatory "find me" and "eat me" signals, expressing proinflammatory cytokines and providing personalized and broad-spectrum tumor antigens draws increasing attention in developing a tumor vaccine. In this study, we aimed to investigate whether the influenza virus (IAV) is efficient enough to induce ICD in tumor cells and an extra modification of IAV components such as hemeagglutinin (HA) will be helpful for the ICD-induced cells to elicit robust antitumor effects; in addition, to evaluate whether the membrane-engineering polylactic coglycolic acid nanoparticles (PLGA NPs) simulating ICD immune stimulation mechanisms hold the potential to be a promising vaccine candidate, a mouse melanoma cell line (B16-F10 cell) was infected with IAV rescued by the reverse genetic system, and the prepared cells and membrane-modified PLGA NPs were used separately to immunize the melanoma-bearing mice. IAV-infected tumor cells exhibit dying status, releasing high mobility group box-1 (HMGB1) and adenosine triphosphate (ATP), and exposing calreticulin (CRT), IAV hemeagglutinin (HA), and tumor antigens like tyrosinase-related protein 2 (TRP2). IAV-induced ICD cells enhance biomass-derived carbon (BMDCs) migration, antigen uptake, cross-presentation, and maturation in vitro. Furthermore, immunization with IAV-induced ICD cells effectively suppressed tumor growth in melanoma-bearing mice. The isolated cell membrane inherited the immunological characteristics from the ICD cells and elicited robust antitumor immune responses through decorating PLGA NPs loading with a tumor-specific helper T-cell peptide and supplemented with ATP in a hydrogel system. This study indicated a promising strategy for developing cell-based and personalized tumor vaccines through fully taking advantage of the immune stimulation mechanisms of ICD occurrence in tumor cells, IAV modification, and nanoscale delivery.
  
  
  
  
**HMGB1**
-
6
of
10
  

### MLLT3 Regulates Melanoma Stemness and Progression by Inhibiting HMGB1 Nuclear Entry and MAGEA1 M(5)C Modification.

6. Adv Sci (Weinh). 2025 Mar;12(10):e2408529. doi: 10.1002/advs.202408529 Epub 2024 Dec 24.
  
  
Li Y(1)(2)(3), Liu H(4), Li J(1)(3), Fu C(1)(3), Jiang B(2), Chen B(2), Zou Y(2), Yu B(2), Song B(1) ...
  
  
Author information: (1)Institute of Biomedical and Health Engineering, Shen Zhen Institutes of Advan ...
  
  
Melanoma stem cells are a kind of cells with self-renewal and multi-directional differentiation potential. They are one of the key factors in the occurrence, development and metastasis of melanoma. This study demonstrates that MLLT3 is a transcription factor that regulates the stemness and progression of melanoma. MLLT3 interacted with HMGB1 to inhibit its entry into the nucleus, MLLT3 interacted with YBX1 to inhibit its reading of m5C of MAGEA1, thereby inhibiting the mRNA stability of MAGEA1, and directly transcribed P53 to inhibit the stemness, proliferation and metastasis of melanoma cells. This study further explored the potential mechanism of the interaction between miR-542-3p/miR-3922-3p and MLLT3. Furthermore, the scRNA-seq of melanoma cells with MLLT3 knock-out resulted in important changes in cell subsets, activating the TP53 and MAPK pathways and transforming into stem cells. The results indicate that the transcription factor MLLT3 is a suppressor gene that regulates the stemness and progression of melanoma, and is expected to become a target for melanoma therapy.
  
  
  
  
**HMGB1**
-
7
of
10
  

### Metabolic Inhibition Induces Pyroptosis in Uveal Melanoma.

7. Mol Cancer Res. 2025 Apr 1;23(4):350-362. doi: 10.1158/1541-7786.MCR-24-0508
  
  
Varney SD(1), Erkes DA(1), Mersky GL(1), Mustafa MU(1), Chua V(1)(2)(3), Chervoneva I(4), Purwin TJ( ...
  
  
Author information: (1)Department of Pharmacology, Physiology, and Cancer Biology, Thomas Jefferson ...
  
  
Few treatment options are available for patients with metastatic uveal melanoma. Although the bispecific tebentafusp is FDA approved, immunotherapy has largely failed, likely given the poorly immunogenic nature of uveal melanoma. Treatment options that improve the recognition of uveal melanoma by the immune system may be key to reducing disease burden. We investigated whether uveal melanoma has the ability to undergo pyroptosis, a form of immunogenic cell death. Publicly available patient data and cell line analysis showed that uveal melanoma expressed the machinery needed for pyroptosis, including gasdermins D and E (GSDMD and E), caspases 1, 3, 4, and 8, and ninjurin-1. We induced cleavage of GSDMs in uveal melanoma cell lines treated with metabolic inhibitors. In particular, the carnitine palmitoyltransferase 1 (CPT1) inhibitor, etomoxir, induced propidium iodide uptake, caspase 3 cleavage, and the release of HMGB1 and IL-1β, indicating that the observed cleavage of GSDMs led to pyroptosis. Importantly, a gene signature reflecting CPT1A activity correlated with poor prognosis in patients with uveal melanoma and knockdown of CPT1A also induced pyroptosis. Etomoxir-induced pyroptosis was dependent on GSDME but not on GSDMD, and a pyroptosis gene signature correlated with immune infiltration and improved response to immune checkpoint blockade in a set of patients with uveal melanoma. Together, these data show that metabolic inhibitors can induce pyroptosis in uveal melanoma cell lines, potentially offering an approach to enhance inflammation-mediated immune targeting in patients with metastatic uveal melanoma. Implications: Induction of pyroptosis by metabolic inhibition may alter the tumor immune microenvironment and improve the efficacy of immunotherapy in uveal melanoma.
  
  
  
  
**HMGB1**
-
8
of
10
  

### Combination of cowpea mosaic virus (CPMV) intratumoral therapy and oxaliplatin chemotherapy.

8. Mater Adv. 2024 Jun 7;5(11):4878-4888. doi: 10.1039/d4ma00427b Epub 2024 May 7.
  
  
Moreno-Gonzalez MA(1)(2)(3)(4)(5), Zhao Z(1)(2)(3)(4)(5), Caparco AA(1)(2)(3)(6), Steinmetz NF(1)(7) ...
  
  
Author information: (1)Department of NanoEngineering, University of California San Diego, 9500 Gilma ...
  
  
Cowpea mosaic virus is a potent intratumoral immunotherapy agent that has shown promise in preclinical studies and canine cancer trials with tumor- and tissue-agnostic efficacy. As we move towards the clinic, it is imperative to investigate combination strategies that synergize to further improve the potency of the approach. Here, we combined CPMV with the clinically approved chemotherapeutic agent oxaliplatin. CPMV's ability to recruit and activate naive immune cells synergized with oxaliplatin's ability to induce immunogenic cell death in the ID8-Defb29/Vegf-A ovarian and B16F10 melanoma murine cancer models with an increase of median survival of 57.7% and 162.2%, respectively. The combination therapy outperformed the CPMV or oxaliplatin monotherapy, and achieved a percent difference in tumor burden of 26.1% and 170.6% in the ID8-Defb29/Vegf-A ovarian and B16F10 melanoma models, respectively. Immunofluorescence staining of treated tumor sections elucidated the role of damage associated molecular patterns (calreticulin and HMGB1), innate immune cells (myeloid cells - likely neutrophils, NK cells, and macrophages), and regulatory T cells (Tregs) as a function of the treatment regimen. Overall, our proposed combination therapy modulated the dormant tumor microenvironment which resulted in effective tumor cell death. This study demonstrates the potential for clinical combination of chemotherapy and CPMV intratumoral immunotherapy.
  
  
  
  
**HMGB1**
-
9
of
10
  

### Neoantigen sequestrated autophagosomes as therapeutic cancer vaccines.

9. J Control Release. 2024 Dec;376:369-381. doi: 10.1016/j.jconrel.2024.10.019 Epub 2024 Oct 19.
  
  
Zhang J(1), Cao J(1), Wang L(1), Li S(1), Meng F(2), Liang X(3), Jiang H(4), Luo R(1), Zhu D(1), Zha ...
  
  
Author information: (1)State Key Laboratory of Advanced Medical Materials and Devices, Tianjin Key L ...
  
  
Neoantigens serve as ideal personalized cancer vaccines because of their high immunogenicity, ability to evade central thymic tolerance, and minimal risk of eliciting autoimmune responses. Herein, we describe a genetically engineered autophagosome-based neoantigen vaccine (APNV) in combination with an immune checkpoint inhibitor (anti-PD-1 antibody) for cancer immunotherapy. The APNV was derived from engineered NIH 3T3 cells, which co-express melanoma neoantigens and autophagosome maker microtubule-associated proteins 1 A/1B light chain 3B (LC3), from which the LC3-labeled neoantigen-autophagosomes were isolated. These purified autophagosomes, in conjunction with vaccine adjuvants high-mobility group box 1 (HMGB1) and granulocyte-macrophage colony-stimulating factor (GM-CSF), were integrated into a hydrogel to create an APNV. The APNV effectively activated dendritic cells both in vitro and in vivo. Moreover, APNV, in combination with checkpoint blockade therapy, significantly hampered post-surgical tumor recurrence in a subcutaneous melanoma tumor model and effectively impeded metastatic progression in a melanoma lung metastasis model. This APNV may be conducive to making personalized therapeutic neoantigen vaccines for cancer immunotherapy.
  
  
  
  
**HMGB1**
-
10
of
10
  

### Induction of immunogenic cell death and enhancement of the radiation-induced immunogenicity by chrysin in melanoma cancer cells.

10. Sci Rep. 2024 Oct 5;14(1):23231. doi: 10.1038/s41598-024-72697-1
  
  
Jafari S(1), Ardakan AK(2), Aghdam EM(3)(4), Mesbahi A(5), Montazersaheb S(#)(6), Molavi O(#)(7)(8).
  
  
Author information: (1)Nutrition Research Center, Tabriz University of Medical Sciences, Tabriz, Ira ...
  
  
Chrysin is a natural flavonoid with anti-cancer effects. Despite its beneficial effects, little information is available regarding its immunogenic cell death (ICD) properties. In this work, we hypothesized that chrysin can potentiate radiotherapy(RT)-induced immunogenicity in melanoma cell line (B16-F10). We examined the effects of chrysin alone and in combination with radiation on ICD induction in B16-F10 cells. Cell viability was assessed using an MTT assay. Cell apoptosis and calreticulin (CRT) exposure were determined using flow cytometry. Western blotting and ELISA assay were employed to examine changes in protein expression. Combination therapy exhibited a synergistic effect, with an optimum combination index of 0.66. The synergistic anti-cancer effect correlated with increased cell apoptosis in cancer cells. Compared to the untreated control, chrysin alone and in combination with RT induced higher levels of DAMPs, such as CRT, HSP70, HMGB1, and ATP. The protein expression of p-STAT3/STAT3 and PD-L1 was reduced in B16-F10 cells exposed to chrysin alone and in combination with RT. Conditioned media from B16-F10 cells exposed to mono-and combination treatments elicited IL-12 secretion in dendritic cells (DCs), inducing a Th1 response. Our findings revealed that chrysin could induce ICD and intensify the RT-induced immunogenicity.
  
  
  
  
  

---

  

# ID1

**Signature gene in:** Endothelial cells
  
**Differentially expressed in:** no cell type
  
  
**ID1**
-
1
of
10
  

### An attractor state zone precedes neural crest fate in melanoma initiation.

1. bioRxiv [Preprint]. 2024 Oct 25:2024.10.22.618007. doi: 10.1101/2024.10.22.618007
  
  
McConnell AM(1)(2)(3), Chassé MH(1)(2)(3), Noonan HR(1)(2)(3)(4), Mito JK(1)(2)(3)(5), Barbano J(1), ...
  
  
Author information: (1)Stem Cell Program and Division of Hematology/Oncology, Children's Hospital Bo ...
  
  
The field cancerization theory suggests that a group of cells containing oncogenic mutations are predisposed to transformation1, 2. We previously identified single cells in BRAF V600E ;p53 -/- zebrafish that reactivate an embryonic neural crest state before initiating melanoma3-5. Here we show that single cells reactivate the neural crest fate from within large fields of adjacent abnormal melanocytes, which we term the "cancer precursor zone." These cancer precursor zone melanocytes have an aberrant morphology, dysplastic nuclei, and altered gene expression. Using single cell RNA-seq and ATAC-seq, we defined a distinct transcriptional cell attractor state for cancer precursor zones and validated the stage-specific gene expression initiation signatures in human melanoma. We identify the cancer precursor zone driver, ID1, which binds to TCF12 and inhibits downstream targets important for the maintenance of melanocyte morphology and cell cycle control. Examination of patient samples revealed precursor melanocytes expressing ID1, often surrounding invasive melanoma, indicating a role for ID1 in early melanomagenesis. This work reveals a surprising field effect of melanoma initiation in vivo in which tumors arise from within a zone of morphologically distinct, but clinically covert, precursors with altered transcriptional fate. Our studies identify novel targets that could improve early diagnosis and prevention of melanoma.
  
  
  
  
**ID1**
-
2
of
10
  

### Strong Activation of ID1, ID2, and ID3 Genes Is Coupled with the Formation of Vasculogenic Mimicry Phenotype in Melanoma Cells.

2. Int J Mol Sci. 2024 Aug 27;25(17):9291. doi: 10.3390/ijms25179291
  
  
Tchurikov NA(1), Vartanian AA(2), Klushevskaya ES(1), Alembekov IR(1), Kretova AN(1), Lukicheva VN(1 ...
  
  
Author information: (1)Department of Epigenetic Mechanisms of Gene Expression Regulation, Engelhardt ...
  
  
Gene expression patterns are very sensitive to external influences and are reflected in phenotypic changes. It was previously described that transferring melanoma cells from a plastic surface to Matrigel led to formation of de novo vascular networks-vasculogenic mimicry-that are characteristic to a stemness phenotype in aggressive tumors. Up to now there was no detailed data about the gene signature accompanying this process. Here, we show that this transfer shortly led to extremely strong epigenetic changes in gene expression in the melanoma cells. We observed that on Matrigel numerous genes controlling ribosome biogenesis were upregulated. However, most of the activated genes were inhibitors of the differentiation genes (ID1, ID2, and ID3). At the same time, the genes that control differentiation were downregulated. Both the upregulated and the downregulated genes are simultaneously targeted by different transcription factors shaping sets of co-expressed genes. The specific group of downregulated genes shaping contacts with rDNA genes are also associated with the H3K27me3 mark and with numerous lincRNAs and miRNAs. We conclude that the stemness phenotype of melanoma cells is due to the downregulation of developmental genes and formation of dedifferentiated cells.
  
  
  
  
**ID1**
-
3
of
10
  

### PGC1α Loss Promotes Lung Cancer Metastasis through Epithelial-Mesenchymal Transition.

3. Cancers (Basel). 2021 Apr 8;13(8):1772. doi: 10.3390/cancers13081772
  
  
Oh TI(1)(2), Lee M(3)(4)(5), Lee YM(1), Kim GH(1)(2), Lee D(6), You JS(7), Kim SH(8), Choi M(8), Jan ...
  
  
Author information: (1)Department of Biomedical Chemistry, College of Biomedical & Health Science, K ...
  
  
PGC1α oppositely regulates cancer metastasis in melanoma, breast, and pancreatic cancer; however, little is known about its impact on lung cancer metastasis. Transcriptome and in vivo xenograft analysis show that a decreased PGC1α correlates with the epithelial-mesenchymal transition (EMT) and lung cancer metastasis. The deletion of a single Pgc1α allele in mice promotes bone metastasis of KrasG12D-driven lung cancer. Mechanistically, PGC1α predominantly activates ID1 expression, which interferes with TCF4-TWIST1 cooperation during EMT. Bioinformatic and clinical studies have shown that PGC1α and ID1 are downregulated in lung cancer, and correlate with a poor survival rate. Our study indicates that TCF4-TWIST1-mediated EMT, which is regulated by the PGC1α-ID1 transcriptional axis, is a potential diagnostic and therapeutic target for metastatic lung cancer.
  
  
  
  
**ID1**
-
4
of
10
  

### TBX3 Promotes Melanoma Migration by Transcriptional Activation of ID1, which Prevents Activation of E-Cadherin by MITF.

4. J Invest Dermatol. 2021 Sep;141(9):2250-2260.e2. doi: 10.1016/j.jid.2021.02.740 Epub 2021 Mar 17.
  
  
Peres J(1), Damerell V(1), Chauhan J(2), Popovic A(1), Desprez PY(3), Galibert MD(4), Goding CR(2), ...
  
  
Author information: (1)Department of Human Biology, Faculty of Health Sciences, University of Cape T ...
  
  
In melanoma, a phenotype switch from proliferation to invasion underpins metastasis, the major cause of melanoma-associated death. The transition from radial to vertical growth phase (invasive) melanoma is characterized by downregulation of both E-cadherin (CDH1) and MITF and upregulation of the key cancer-associated gene TBX3 and the phosphatidylinositol 3 kinase signaling pathway. Yet, whether and how these diverse events are linked remains poorly understood. Here, we show that TBX3 directly promotes expression of ID1, a dominant-negative regulator of basic helix-loop-helix transcription factors, and that ID1 decreases MITF binding and upregulation of CDH1. Significantly, we show that TBX3 activation of ID1 is necessary for TBX3 to enhance melanoma cell migration, and the mechanistic links between TBX3, ID1, MITF, and invasion revealed here are reflected in their expression in human melanomas. Our results reveal that melanoma migration is promoted through a TBX3-ID1-MITF-E-cadherin axis and that ID1-mediated repression of MITF activity may reinforce maintenance of an MITFLow phenotype associated with disease progression and therapy resistance.
  
  
  
  
**ID1**
-
5
of
10
  

### IL-1β Promotes Stemness of Tumor Cells by Activating Smad/ID1 Signaling Pathway.

5. Int J Med Sci. 2020 May 18;17(9):1257-1268. doi: 10.7150/ijms.44285 eCollection 2020.
  
  
Lu L(1)(2), Wang P(2), Zou Y(3), Zha Z(1), Huang H(2), Guan M(1)(2), Wu Y(1)(2), Liu G(1)(2).
  
  
Author information: (1)Department of Medical Oncology, Guangzhou First People's Hospital, School of ...
  
  
Background: IL-1β is reported to be involved in cancer development and distant metastasis. However, the underlying mechanism of IL-1β upon malignant behaviors remains largely unknown. In this study, we aimed to study whether IL-1β could enhance the stemness traits of tumor cells. Methods: The concentrations of serum IL-1β in head and neck squamous cell carcinoma (HNSCC) and melanoma patients were detected using ELISA assay. The effect and mechanisms of IL-1β on tumor cell growth, migration, invasion and stemness characters were studied using HNSCC cell SCC7 and melanoma cell B16-F10. The underlying mechanisms were further explored. Results: Enhanced concentrations of IL-1β were positively correlated with advanced tumor stage in both HNSCC and melanoma patients. IL-1β treatment led to a significant increase in tumor growth both in vitro and in vivo. IL-1β stimulation promoted cell proliferation, colony formation and tumorigenicity. In addition, IL-1β-stimulated tumor cells gained enhanced capabilities on wounding healing and invasion capabilities. Moreover, IL-1β stimulation promoted the stem-like capabilities of both HNSCC cells and melanoma cells, including the enrichment of aldehyde dehydrogenase+ (ALDH+) cells, up-regulation of stem cell related markers Nanog, OCT4, and SOX2, sphere formation and chemoresistance. Mechanistically, IL-1β treatment promoted the phosphorylation of Smad1/5/8 and activated its downstream target inhibitor of differentiation 1 (ID1). Silencing ID1 abrogated sphere formation and upregulated expression of stemness genes which were induced by IL-1β stimulation. Conclusion: Our data demonstrates that IL-1β promotes the stemness of HNSCC and melanoma cells through activating Smad/ID1 signal pathway.
  
  
  
  
**ID1**
-
6
of
10
  

### High expression of ID1 in monocytes is strongly associated with phenotypic and functional MDSC markers in advanced melanoma.

6. Cancer Immunol Immunother. 2020 Apr;69(4):513-522. doi: 10.1007/s00262-019-02476-9 Epub 2020 Jan 17.
  
  
Melief J(1), Pico de Coaña Y(2), Maas R(2)(3), Fennemann FL(2)(4), Wolodarski M(2)(5), Hansson J(2), ...
  
  
Author information: (1)Department of Oncology-Pathology, Karolinska Institute, Visionsgatan 4, 171 6 ...
  
  
The efficacy of immunotherapies for malignant melanoma is severely hampered by local and systemic immunosuppression mediated by myeloid-derived suppressor cells (MDSC). Inhibitor of differentiation 1 (ID1) is a transcriptional regulator that was shown to be centrally involved in the induction of immunosuppressive properties in myeloid cells in mice, while it was overexpressed in CD11b+ cells in the blood of late-stage melanoma patients. Therefore, we comprehensively assessed ID1 expression in PBMC from stage III and IV melanoma patients, and studied ID1 regulation in models for human monocyte differentiation towards monocyte-derived dendritic cells. A highly significant elevation of ID1 was observed in CD33+CD11b+CD14+HLA-DRlow monocytic MDSC in the blood of melanoma patients compared to their HLA-DRhigh counterparts, while expression of ID1 correlated positively with established MDSC markers S100A8/9 and iNOS. Moreover, expression of ID1 in monocytes significantly decreased in PBMC samples taken after surgical removal of melanoma metastases, compared to those taken before surgery. Finally, maturation of monocyte-derived DC coincided with a significant downregulation of ID1. Together, these data indicate that increased ID1 expression is strongly associated with expression of phenotypic and immunosuppressive markers of monocytic MDSC, while downregulation is associated with a more immunogenic myeloid phenotype. As such, ID1 may be an additional phenotypic marker for monocytic MDSC. Investigation of ID1 as a pharmacodynamic biomarker or its use as a target for modulating MDSC is warranted.
  
  
  
  
**ID1**
-
7
of
10
  

### Author Correction: Hyaluronic acid-CD44 interactions promote BMP4/7-dependent ID1/3 expression in melanoma cells.

7. Sci Rep. 2019 Dec 11;9(1):19220. doi: 10.1038/s41598-019-55812-5
  
  
Wu RL(1)(2), Sedlmeier G(1), Kyjacova L(1), Schmaus A(1)(3), Philipp J(1), Thiele W(1)(3), Garvalov ...
  
  
Author information: (1)European Center for Angioscience (ECAS), Medical Faculty of Mannheim, Heidelb ...
  
  
Erratum for Sci Rep. 2018 Oct 8;8(1):14913. doi: 10.1038/s41598-018-33337-7.
  
  
  
  
**ID1**
-
8
of
10
  

### ID1 Is Critical for Tumorigenesis and Regulates Chemoresistance in Glioblastoma.

8. Cancer Res. 2019 Aug 15;79(16):4057-4071. doi: 10.1158/0008-5472.CAN-18-1357 Epub 2019 Jul 10.
  
  
Sachdeva R(1)(2), Wu M(1), Smiljanic S(1)(2), Kaskun O(1), Ghannad-Zadeh K(1), Celebre A(1)(3), Isae ...
  
  
Author information: (1)The Arthur and Sonia Labatt Brain Tumor Research Center, Hospital for Sick Ch ...
  
  
Glioblastoma is the most common primary brain tumor in adults. While the introduction of temozolomide chemotherapy has increased long-term survivorship, treatment failure and rapid tumor recurrence remains universal. The transcriptional regulatory protein, inhibitor of DNA-binding-1 (ID1), is a key regulator of cell phenotype in cancer. We show that CRISPR-mediated knockout of ID1 in glioblastoma cells, breast adenocarcinoma cells, and melanoma cells dramatically reduced tumor progression in all three cancer systems through transcriptional downregulation of EGF, which resulted in decreased EGFR phosphorylation. Moreover, ID1-positive cells were enriched by chemotherapy and drove tumor recurrence in glioblastoma. Addition of the neuroleptic drug pimozide to inhibit ID1 expression enhanced the cytotoxic effects of temozolomide therapy on glioma cells and significantly prolonged time to tumor recurrence. Conclusively, these data suggest ID1 could be a promising therapeutic target in patients with glioblastoma. SIGNIFICANCE: These findings show that the transcriptional regulator ID1 is critical for glioblastoma initiation and chemoresistance and that inhibition of ID1 enhances the effect of temozolomide, delays tumor recurrence, and prolongs survival.
  
  
  
  
**ID1**
-
9
of
10
  

### Hyaluronic acid-CD44 interactions promote BMP4/7-dependent ID1/3 expression in melanoma cells.

9. Sci Rep. 2018 Oct 8;8(1):14913. doi: 10.1038/s41598-018-33337-7
  
  
Wu RL(1)(2), Sedlmeier G(1), Kyjacova L(1), Schmaus A(1)(3), Philipp J(1), Thiele W(1)(3), Garvalov ...
  
  
Author information: (1)European Center for Angioscience (ECAS), Medical Faculty of Mannheim, Heidelb ...
  
  
Erratum in Sci Rep. 2019 Dec 11;9(1):19220. doi: 10.1038/s41598-019-55812-5.
  
  
  
  
**ID1**
-
10
of
10
  

### Footprintless disruption of prosurvival genes in aneuploid cancer cells using CRISPR/Cas9 technology.

10. Biochem Cell Biol. 2016 Jun;94(3):289-96. doi: 10.1139/bcb-2015-0150 Epub 2016 Jun 2.
  
  
Krachulec JM(1), Sedlmeier G(1), Thiele W(1)(2), Sleeman JP(1)(2).
  
  
Author information: (1)a Centre for Biomedicine and Medical Technology Mannheim, Medical Faculty Man ...
  
  
CRISPR/Cas9 has emerged as a powerful methodology for the targeted editing of genomic DNA sequences. Nevertheless, the intrinsic inefficiency of transfection methods required to use this technique with cultured cells requires the selection and isolation of successfully modified cells, which invariably subjects the cells to stress. Here we report a workflow that allows the isolation of genomically modified cells, even where loss of functional alleles constitutes a selective disadvantage owing to impaired ability to survive stress. Using targeted disruption of the ID1 and Id3 genes in murine B16-F10 and Ret melanoma cell lines as an example, we show that the method allows for the footprintless isolation of CRISPR/Cas9-modified aneuploid cancer cells. We also provide evidence that serial CRISPR/Cas9 modifications can occur, for example when initial homologous recombination events introduce cryptic PAM sequences, and demonstrate that multiple alleles can be successfully targeted in aneuploid cancer cells. By sequencing individual alleles we also found evidence for CRISPR/Cas9-induced transposable element insertion, albeit at a low frequency. This workflow should have broad application in the functional analysis of prosurvival gene function in cultured cells.
  
  
  
  
  

---

  

# IKZF1

**Signature gene in:** B cells
  
**Differentially expressed in:** no cell type
  
  
**IKZF1**
-
1
of
6
  

### Novel IKZF3 transcriptomic signature correlates with positive outcomes of skin cutaneous melanoma: A pan-cancer analysis.

1. Front Genet. 2022 Oct 24;13:1036402. doi: 10.3389/fgene.2022.1036402 eCollection 2022.
  
  
Yang LK(1), Lin CX(1), Li SH(1), Liang JJ(1), Xiao LL(1), Xie GH(1), Liu HW(1), Liao X(1).
  
  
Author information: (1)Department of Plastic Surgery, The First Affiliated Hospital of Jinan Univers ...
  
  
To investigate the potential relationship between Ikaros family genes and skin cutaneous melanoma (SKCM), we undertook a pan-cancer analysis of the transcriptional signature and clinical data of melanoma through multiple databases. First, 10,327 transcriptomic samples from different cancers were included to determine the overall characteristics and clinical prognoses associated with Ikaros gene expression across cancer types. Second, differentially expressed genes analysis, prognostic evaluation, and gene set enrichment analysis were employed to investigate the role of Ikaros (IKZF) genes in SKCM. Third, we evaluated the relationship between Ikaros family genes and SKCM immune infiltrates and verified the findings using the GEO single-cell sequencing dataset. The results show that Ikaros genes were widely expressed among different cancer types with independently similar patterns as follows: 1. IKZF1 and IKZF3, and 2. IKZF2 and IKZF4-5. IKZF2 and IKZF5 were downregulated in the primary tumor, and IKZF1-3 expression decreased significantly as the T-stage or metastasis increased in SKCM. Moreover, high IKZF1-3 expression was associated with better overall survival, disease-specific survival, and progression-free interval. IKZF3 is an independent prognostic factor of SKCM. Among Ikaros genes, the expression of IKZF1 and IKZF3 positively correlated with the infiltration level of CD4+ T cells and CD8+ T cells, B cells, and Tregs in SKCM and negatively correlated with the infiltration level of M0 and M1 macrophages. Moreover, single-cell sequencing data analysis revealed that IKZF1 and IKZF3 were mainly expressed by immune cells. Correlation analysis shows the immune factors and drug responses associated with IKZF3 expression. In conclusion, the present study is the first, to our knowledge, to identify a pan-cancer genomic signature of the Ikaros gene family among different cancers. Expression of these family members, particularly high levels of IKZF3, indicate positive immunological status and beneficial clinical outcomes of SKCM. IKZF3 may therefore serve as potential targets for immunotherapy of melanoma.
  
  
  
  
**IKZF1**
-
2
of
6
  

### Immature tertiary lymphoid structure formation was increased in the melanoma tumor microenvironment of IKZF1 transgenic mice.

2. Transl Cancer Res. 2022 Jul;11(7):2388-2397. doi: 10.21037/tcr-22-1759
  
  
Yin S(1), Zhang C(2), Gao F(1).
  
  
Author information: (1)Department of Oncology, Shanghai 9th People's Hospital, Shanghai Jiao Tong Un ...
  
  
BACKGROUND: IKZF1 promotes the occurrence of lymphoma and is also related to the development of breast cancer, liver cancer, and ovarian cancer. It was hypothesized that IKZF1 influences tertiary lymphoid structures (TLSs) formation and development in the tumor immune microenvironment, and this effect of IKZF1 on the tumor immune microenvironment has not been explored. Using melanoma grafts as a model, we investigated the effect of IKZF1 on the immune microenvironment of melanoma. METHODS: The Cell Count Kit-8 (CCK8) assay was used to detect the effect IKZF1 overexpression in melanoma cells on cell proliferation. The IKZF1 overexpression vector was constructed by homologous recombination. After linearization, the overexpression vector was microinjected into the fertilized egg. Transgenic mice overexpressing IKZF1 were screened by tail identification. After melanoma B16 mouse cells were digested into single cells, the tumor was subcutaneously implanted in C57BL6/J-wild type (WT) mice and IKZF1 transgenic mice, and the tumor growth of the 2 groups was compared. The number of TLSs in the tumor tissues of mice was analyzed after hematoxylin-eosin (HE) staining. RESULTS: Overexpression of IKZF1 in melanoma cells did not affect cell proliferation. The IKZF1 overexpression vector pcDNA3.1-CAG-IKAROS was successfully constructed. Viable fertilized eggs were obtained after microinjection. Transgenic mice stably expressing IKZF1 were identified by polymerase chain reaction (PCR). Compared with WT mice, the tumor load of IKZF1 transgenic mice increased significantly. HE staining showed that the number of immature TLSs in melanomas of IKZF1 transgenic mice increased significantly. CONCLUSIONS: IKZF1 does not affect the proliferation of melanoma cells. Transgenic mice overexpressing IKZF1 were successfully constructed. IKZF1 is a key driver gene of the formation of immature TLS.
  
  
  
  
**IKZF1**
-
3
of
6
  

### Biomarkers Associated with Immune-Related Adverse Events under Checkpoint Inhibitors in Metastatic Melanoma.

3. Cancers (Basel). 2022 Jan 8;14(2):302. doi: 10.3390/cancers14020302
  
  
Wölffer M(1), Battke F(2), Schulze M(3), Feldhahn M(2), Flatz L(1), Martus P(4), Forschner A(1).
  
  
Author information: (1)Department of Dermatology, University Hospital Tuebingen, 72076 Tuebingen, Ge ...
  
  
Immune checkpoint inhibitors (ICI) have revolutionized the therapeutic landscape of metastatic melanoma. However, ICI are often associated with immune-related adverse events (IRAE) such as colitis, hepatitis, pancreatitis, hypophysitis, pneumonitis, thyroiditis, exanthema, nephritis, myositis, encephalitis, or myocarditis. Biomarkers associated with the occurrence of IRAE would be desirable. In the literature, there is only little data available and furthermore mostly speculative, especially in view of genetic alterations. Our major aim was to check for possible associations between NGS-based genetic alterations and IRAE. We therefore analyzed 95 melanoma patients with ICI and evaluated their NGS results. We checked the data in view of potential associations between copy number variations (CNVs), small variations (VARs), human leucocyte antigen (HLA), sex, blood count parameters, pre-existing autoimmune diseases and the occurrence of IRAE. We conducted a literature research on genetic alterations hypothesized to be associated with the occurrence of IRAE. In total, we identified 39 genes that have been discussed as hypothetical biomarkers. We compared the list of these 39 genes with the tumor panel that our patients had received and focused our study on those 16 genes that were also included in the tumor panel used for NGS. Therefore, we focused our analyses on the following genes: AIRE, TERT, SH2B3, LRRK2, IKZF1, SMAD3, JAK2, PRDM1, CTLA4, TSHR, FAN1, SLCO1B1, PDCD1, IL1RN, CD274, UNG. We obtained relevant results: female sex was significantly associated with the development of hepatitis, combined immunotherapy with colitis, increased total and relative monocytes at therapy initiation were significantly associated with the development of pancreatitis, the same, pre-existing autoimmune diseases. Further significant associations were as follows: HLA homozygosity (hepatitis), and VARs on SMAD3 (pancreatitis). Regarding CNVs, significant markers included PRDM1 deletions and IL1RN (IRAE), CD274 duplications and SLCO1B1 (hepatitis), PRDM1 and CD274 (encephalitis), and PRDM1, CD274, TSHR, and FAN1 (myositis). Myositis and encephalitis, both, were associated with alterations of PRDM1 and CD274, which might explain their joined appearance in clinical practice. The association between HLA homozygosity and IRAE was clarified by finding HLA-A homozygosity as determining factor. We identified several genetic alterations hypothesized in the literature to be associated with the development of IRAE and found significant results concerning pre-existing autoimmune diseases and specific blood count parameters. Our findings can help to better understand the development of IRAE in melanoma patients. NGS might be a useful screening tool, however, our findings have yet to be confirmed in larger studies.
  
  
  
  
**IKZF1**
-
4
of
6
  

### Human Vaccines & Immunotherapeutics: News.

4. Hum Vaccin Immunother. 2018;14(9):2099-2100. doi: 10.1080/21645515.2018.1518064
  
  
[No authors listed]
  
  
DOI: 10.1080/21645515.2018.1518064 PMCID: PMC6183266 PMID: 30285588 [Indexed for MEDLINE]
  
  
NA
  
  
  
  
**IKZF1**
-
5
of
6
  

### Network-based co-expression analysis for exploring the potential diagnostic biomarkers of metastatic melanoma.

5. PLoS One. 2018 Jan 29;13(1):e0190447. doi: 10.1371/journal.pone.0190447 eCollection 2018.
  
  
Wang LX(1), Li Y(2), Chen GZ(1).
  
  
Author information: (1)Department of Dermatology, The Affiliated Hospital of Qingdao University, Sha ...
  
  
Metastatic melanoma is an aggressive skin cancer and is one of the global malignancies with high mortality and morbidity. It is essential to identify and verify diagnostic biomarkers of early metastatic melanoma. Previous studies have systematically assessed protein biomarkers and mRNA-based expression characteristics. However, molecular markers for the early diagnosis of metastatic melanoma have not been identified. To explore potential regulatory targets, we have analyzed the gene microarray expression profiles of malignant melanoma samples by co-expression analysis based on the network approach. The differentially expressed genes (DEGs) were screened by the EdgeR package of R software. A weighted gene co-expression network analysis (WGCNA) was used for the identification of DEGs in the special gene modules and hub genes. Subsequently, a protein-protein interaction network was constructed to extract hub genes associated with gene modules. Finally, twenty-four important hub genes (RASGRP2, IKZF1, CXCR5, LTB, BLK, LINGO3, CCR6, P2RY10, RHOH, JUP, KRT14, PLA2G3, SPRR1A, KRT78, SFN, CLDN4, IL1RN, PKP3, CBLC, KRT16, TMEM79, KLK8, LYPD3 and LYPD5) were treated as valuable factors involved in the immune response and tumor cell development in tumorigenesis. In addition, a transcriptional regulatory network was constructed for these specific modules or hub genes, and a few core transcriptional regulators were found to be mostly associated with our hub genes, including GATA1, STAT1, SP1, and PSG1. In summary, our findings enhance our understanding of the biological process of malignant melanoma metastasis, enabling us to identify specific genes to use for diagnostic and prognostic markers and possibly for targeted therapy.
  
  
  
  
**IKZF1**
-
6
of
6
  

### Ikaros imposes a barrier to CD8+ T cell differentiation by restricting autocrine IL-2 production.

6. J Immunol. 2014 Jun 1;192(11):5118-29. doi: 10.4049/jimmunol.1301992 Epub 2014 Apr 28.
  
  
O'Brien S(1), Thomas RM(2), Wertheim GB(3), Zhang F(4), Shen H(4), Wells AD(5).
  
  
Author information: (1)Department of Pathology and Laboratory Medicine, Perelman School of Medicine, ...
  
  
Naive CD4(+) T cells require signals from the TCR and CD28 to produce IL-2, expand, and differentiate. However, these same signals are not sufficient to induce autocrine IL-2 production by naive CD8(+) T cells, which require cytokines provided by other cell types to drive their differentiation. The basis for failed autocrine IL-2 production by activated CD8(+) cells is unclear. We find that Ikaros, a transcriptional repressor that silences IL-2 in anergic CD4(+) T cells, also restricts autocrine IL-2 production by CD8(+) T cells. We find that CD8(+) T cell activation in vitro in the absence of exogenous cytokines and CD4 help leads to marked induction of Ikaros, a known repressor of the Il2 gene. Naive murine CD8 T cells haplo-insufficient for IKZF1 failed to upregulate Ikaros, produced autocrine IL-2, and differentiated in an IL-2-dependent manner into IFN-γ-producing CTLs in response to TCR/CD28 stimulation alone. Furthermore, IKZF1 haplo-insufficient CD8(+) T cells were more effective at controlling Listeria infection and B16 melanoma growth in vivo, and they could provide help to neighboring, non-IL-2-producing cells to differentiate into IFN-γ-producing effectors. Therefore, by repressing autocrine IL-2 production, Ikaros ensures that naive CD8(+) T cells remain dependent on licensing by APCs and CD4(+) T cells, and it may therefore act as a cell-intrinsic safeguard against inappropriate CTL differentiation and immunopathology.
  
  
  
  
  

---

  

# INSR

**Signature gene in:** MDM
  
**Differentially expressed in:** no cell type
  
  
**INSR**
-
1
of
3
  

### Genetic association of type 2 diabetes and antidiabetic drug target with skin cancer.

1. Front Med (Lausanne). 2024 Nov 21;11:1445853. doi: 10.3389/fmed.2024.1445853 eCollection 2024.
  
  
Zhao J(1), Zhang Y(2), Li J(3), Li Q(2), Teng Z(2).
  
  
Author information: (1)Department of Dermatology, Kunming City Maternal and Child Health Hospital, K ...
  
  
BACKGROUND: Several observational studies have suggested that type 2 diabetes (T2D) is a risk factor for skin cancer, and antidiabetic drugs may reduce skin cancer risk. Nevertheless, the findings remain ambiguous. This Mendelian randomization (MR) study aimed to investigate the causal association of T2D with skin cancer and evaluate the potential impact of antidiabetic drug targets on skin cancer. METHODS: Genetic variants associated with glycated hemoglobin (HbA1c), Type 2 Diabetes (T2D), and antidiabetic drug targets (KCNJ11, ABCC8, PPARG, INSR, GLP1R, SLC5A2, and DPP4) were sourced from genome-wide association studies in the UK Biobank and the DIAMANTE consortium. Genetic summary statistics on skin cancer were obtained from the FinnGen consortium. MR analysis was primarily performed leveraging the inverse-variance weighted method, with additional sensitivity analyses conducted. Summary data-based MR (SMR) was utilized to further investigate the association between antidiabetic drug target gene expression and skin cancer. Colocalization analysis was carried out to verify the robustness of the results. RESULTS: Genetically proxied elevated levels of HbA1c were found to be suggestively associated with a reduced risk of melanoma (OR: 0.886, 95% confidence interval (CI): 0.792-0.991, p = 0.0347). Additionally, genetically proxied T2D was notably associated with a lower risk of basal cell carcinoma (OR: 0.960, 95% CI: 0.928-0.992, p = 0.0147). The study also discovered that perturbation of the antidiabetic drug target SLC5A2 was significantly associated with an increased risk of basal cell carcinoma (for SLC5A2 perturbation equivalent to a 6.75 mmol/mol decrement in HbA1c: OR: 2.004, 95% CI: 1.270-3.161, p = 0.0027). However, this finding was not supported by colocalization analysis. Notably, no other drug target perturbations were found to be associated with skin cancer. Furthermore, SMR analysis failed to detect an association between antidiabetic drug target genes and skin cancer. CONCLUSION: The study suggests that higher HbA1c levels and T2D may be associated with a reduced risk of skin cancer. However, the results did not provide evidence to support the association between antidiabetic drug targets and skin cancer. Further evaluation of these drug targets is required to confirm the findings in this analysis.
  
  
  
  
**INSR**
-
2
of
3
  

### IGF1R/IR Mediates Resistance to BRAF and MEK Inhibitors in BRAF-Mutant Melanoma.

2. Cancers (Basel). 2021 Nov 22;13(22):5863. doi: 10.3390/cancers13225863
  
  
Patel H(1), Mishra R(1), Yacoub N(2), Alanazi S(1), Kilroy MK(1), Garrett JT(1).
  
  
Author information: (1)Department of Pharmaceutical Sciences, College of Pharmacy, University of Cin ...
  
  
The use of BRAF and MEK inhibitors for patients with BRAF-mutant melanoma is limited as patients relapse on treatment as quickly as 6 months due to acquired resistance. We generated trametinib and dabrafenib resistant melanoma (TDR) cell lines to the MEK and BRAF inhibitors, respectively. TDR cells exhibited increased viability and maintenance of downstream p-ERK and p-Akt as compared to parental cells. Receptor tyrosine kinase arrays revealed an increase in p-IGF1R and p-IR in the drug resistant cells versus drug sensitive cells. RNA-sequencing analysis identified IGF1R and INSR upregulated in resistant cell lines compared to parental cells. Analysis of TCGA PanCancer Atlas (skin cutaneous melanoma) showed that patients with a BRAF mutation and high levels of IGF1R and INSR had a worse overall survival. BMS-754807, an IGF1R/IR inhibitor, suppressed cell proliferation along with inhibition of intracellular p-Akt in TDR cells. Dual inhibition of IGF1R and INSR using siRNA reduced cell proliferation. The combination of dabrafenib, trametinib, and BMS-754807 treatment reduced in vivo xenograft tumor growth. Examining the role of IGF1R and IR in mediating resistance to BRAF and MEK inhibitors will expand possible treatment options to aid in long-term success for BRAF-mutant melanoma patients.
  
  
  
  
**INSR**
-
3
of
3
  

### The human VAV proto-oncogene maps to chromosome region 19p12----19p13.2.

3. Hum Genet. 1990 Nov;86(1):65-8. doi: 10.1007/BF00205175
  
  
Martinerie C(1), Cannizzaro LA, Croce CM, Huebner K, Katzav S, Barbacid M.
  
  
Author information: (1)Institut Curie, Centre Universitaire de Paris-Sud, Orsay, France.
  
  
A novel human oncogene, designated VAV, has been recently characterized. This oncogene was generated by a rearrangement within the 5' coding sequences of a normal cellular gene, the VAV proto-oncogene. The normal VAV gene is specifically expressed in hematopoietic cells regardless of their differentiation lineage. We now report that the VAV locus has been localized in the human genome at chromosome 19p12----19p13.2 by analysis of its segregation pattern in rodent-human somatic cell hybrids and by chromosomal in situ hybridization. The VAV locus might be closely linked to the insulin receptor (INSR) locus, as suggested by comigration of INSR and VAV high-molecular-weight DNA fragments after pulsed-field gel electrophoresis. The VAV chromosomal assignment is of interest because chromosome region 19p13 is involved in different karyotypic abnormalities in a variety of malignancies including melanomas and leukemias. The identification of a novel proto-oncogene that maps to that region will enable us to define whether VAV is involved in any of the translocations observed.
  
  
  
  
  

---

  

# JAK2

**Signature gene in:** Dendritic cells
  
**Differentially expressed in:** no cell type
  
  
**JAK2**
-
1
of
10
  

### Recurrent ERBB2 alterations are associated with esophageal adenocarcinoma brain metastases.

1. medRxiv [Preprint]. 2025 Feb 26:2025.02.19.25322558. doi: 10.1101/2025.02.19.25322558
  
  
Lawson NM(1), Ye L(1), Cho CY(1), Zhao B(1), Mitchell T(2), Martín-Barrio I(1), Beernaert B(3), Gupt ...
  
  
Author information: (1)Department of Neurosurgery, MD Anderson Cancer Center, Houston, TX, USA. (2)D ...
  
  
Brain metastases in esophageal adenocarcinoma (EAC) patients are associated with poor prognosis and remain understudied. We performed multi-omics analysis with whole-genome sequencing and single-cell spatial transcriptomics on the brain metastases and matched primary tumors. Our analysis identified ERBB2 as a recurrent oncogene in EAC brain metastases, with 9 out of 10 cases harboring amplifications. Single-cell whole-genome and multi-region sequencing revealed that ERBB2 alterations, occur early during disease progression and are associated with monoclonal seeding. Although the median survival in our cohort was 13 months, one patient on HER2 antibody-drug conjugate therapy remains a long-term survivor beyond 34 months. Interestingly, the sole patient without an ERBB2 alteration had JAK2 deletion, high T cell infiltration in the brain lesion, and survived 35 months after immune checkpoint therapy. Our findings have significant clinical implications for the treatment and management of EAC brain metastases.
  
  
  
  
**JAK2**
-
2
of
10
  

### Prognostic value and immune infiltration of a tumor microenvironment-related PTPN6 in metastatic melanoma.

2. Cancer Cell Int. 2024 Dec 28;24(1):435. doi: 10.1186/s12935-024-03625-6
  
  
Sun R(1), Wei S(2), Yu Y(1), Wang Z(1), Yao T(1), Zhang Y(1), Cui L(1), Ma X(3).
  
  
Author information: (1)Department of Plastic and Aesthetic Surgery, the Second Affiliated Hospital o ...
  
  
BACKGROUND: Cutaneous melanoma is one of the most invasive and lethal skin malignant tumors. Compared to primary melanoma, metastatic melanoma (MM) presents poorer treatment outcomes and a higher mortality rate. The tumor microenvironment (TME) plays a critical role in MM progression and immunotherapy resistance. This study focuses on the role of the TME-related gene PTPN6 in the prognosis and immunotherapy response of MM. METHODS: This study analyzed the RNA-seq and clinical data of MM patients from public databases, employing the ESTIMATE algorithm and bioinformatics tools to identify differentially expressed genes in the TME. PTPN6 was identified as a prognostic biomarker. Its expression and function were validated using in vitro and in vivo experiments. The role of PTPN6 in immune cell infiltration and its association with the JAK2-STAT3 pathway and immunotherapy response were also evaluated. RESULTS: PTPN6 expression was significantly lower in MM and associated with poor prognosis. In vitro, Overexpression of PTPN6 inhibited proliferation, migration, and invasion, while knockdown reversed these effects. In vivo, PTPN6 overexpression reduced tumor growth. Mechanistically, PTPN6 suppressed JAK2-STAT3 signaling pathway activation. High PTPN6 expression was positively associated with immune cell infiltration, improved immunotherapy response, and reduced PD-L1 expression. CONCLUSION: The gene PTPN6, associated with the tumor microenvironment, may serve as a promising prognostic biomarker and therapeutic target for MM.
  
  
  
  
**JAK2**
-
3
of
10
  

### Harnessing Tumor Cell-Derived Exosomes for Immune Rejection Management in Corneal Transplantation.

3. Adv Sci (Weinh). 2025 Jan;12(2):e2409207. doi: 10.1002/advs.202409207 Epub 2024 Nov 14.
  
  
Yang J(1), Kang H(2), Liu Y(3), Lu S(1), Wu H(4), Zhang B(4), He Y(3), Zhou W(1)(5).
  
  
Author information: (1)Xiangya School of Pharmaceutical Sciences, Central South University, Changsha ...
  
  
Transplantation remains the definitive treatment for end-stage organ failures, but its efficacy is frequently compromised by immune rejection. This study introduces a novel strategy by utilizing tumor-derived exosomes from B16-F10 melanoma cells (B16-Exo), diverging from the conventional use of immune cell-derived exosomes, to alleviate post-transplantation immune rejection. Utilizing murine corneal transplantation as a model, it is demonstrated that B16-Exo significantly reduces immune rejection, evidenced by decreased corneal opacity, neovascularization, and immune dysregulation, while enhancing postoperative survival. Proteomic analyses reveal differential expression of pivotal proteins in B16-Exo, notably the JAK2 protein within the JAK-STAT signaling pathway, which has been mechanistically demonstrated to amplify the activity of myeloid-derived suppressor cells (MDSCs) and inhibit T cell proliferation. These findings demonstrate the significant immunomodulatory effect of B16-Exo in transplant immunology, supporting the continued exploration of tumor-derived exosomes as a platform to uncover novel immunosuppressive mechanisms in transplantation.
  
  
  
  
**JAK2**
-
4
of
10
  

### AIM2 inflammasome regulated by the IFN-γ/JAK2/STAT1 pathway promotes activation and pyroptosis of monocytes in Coronary Artery Disease.

4. Immun Inflamm Dis. 2024 Jun;12(6):e1317. doi: 10.1002/iid3.1317
  
  
Zhao Y(1), Liang B(1)(2)(3), Sheng S(1), Wang C(1), Jin B(1), Zhang X(1), Cheng Y(1), Shen C(4), Zhe ...
  
  
Author information: (1)Center for Gene Diagnosis and Department of Clinical Laboratory Medicine, Zho ...
  
  
BACKGROUND: Numerous studies have demonstrated that Absent in Melanoma 2 (AIM2) is upregulated in aortic plaques, especially in Vascular Smooth Muscle Cells in Coronary Artery Disease (CAD), and is related to inflammasome-induced inflammation. However, the underlying mechanism of this phenomenon and the role of AIM2 in atherosclerosis remained unclear. METHODS: This study enrolled 133 CAD patients and 123 controls. We isolated Peripheral Blood Leukocytes (PBLs) and the mRNA expression of AIM2 inflammasome and its downstream genes (ASC, Caspase-1, IL-1β, and IL-18) were detected by real-time quantitative PCR (qPCR). We assessed correlations between AIM2 expressions and clinical characteristics by multiple linear regression and spearman's correlation. The THP-1 cells cultured in poly(dA:dT), A151, interferon-gamma (IFN-γ), AG490, or JC2-11. And then the mRNA and protein levels of AIM2, ASC, Caspase-1, IL-1β, IL-18, GSDMD, and STAT1 were analyzed by qPCR and Western blot analysis, respectively. The migration and adhesive capacity of THP-1 cells was assessed using an inverted microscope and an inverted fluorescence microscope, respectively. RESULTS: In this study, we found that expressions of components of AIM2 inflammasome and its downstream genes (ASC, Caspase-1, IL-1β, and IL-18), were all increased in PBLs of CAD patients, which indicated the inflammasome activation. AIM2 inflammasome activation further induced pyroptosis, and stimulated migration and adhesion in monocyte cell lines, which was regulated by IFN-γ probably through JAK2/STAT1 pathway. In addition, AIM2 expressions were positively correlated with systemic inflammatory indicators as an independent risk factor for CAD. CONCLUSIONS: In conclusion, increased AIM2 expression, induced by the IFN-γ/JAK2/STAT1 signal, orientates monocytes to inflammatory status or even pyroptosis through AIM2 inflammasome activation, which is involved in the development of CAD.
  
  
  
  
**JAK2**
-
5
of
10
  

### α5-nAChR/ADAM10 signaling mediates nicotine-related cutaneous melanoma progression via STAT3 activation.

5. Arch Dermatol Res. 2024 May 25;316(6):269. doi: 10.1007/s00403-024-03110-0
  
  
Li X(#)(1), Meng X(#)(1), Fan H(2), Wang Y(3), Jia Y(4), Jiao J(5), Ma X(6)(7)(8).
  
  
Author information: (1)Department of Dermatology, Central Hospital Affiliated to Shandong First Medi ...
  
  
Skin cutaneous melanoma (SKCM) is the skin malignancy with the highest mortality rate, and its morbidity rate is on the rise worldwide. Smoking is an independent marker of poor prognosis in melanoma. The α5-nicotinic acetylcholine receptor (α5-nAChR), one of the receptors for nicotine, is involved in the proliferation, migration and invasion of SKCM cells. Nicotine has been reported to promote the expression of a disintegrin and metalloproteinase 10 (ADAM10), which is the key gene involved in melanoma progression. Here, we explored the link between α5-nAChR and ADAM10 in nicotine-associated cutaneous melanoma. α5-nAChR expression was correlated with ADAM10 expression and lower survival in SKCM. α5-nAChR mediated nicotine-induced ADAM10 expression via STAT3. The α5-nAChR/ADAM10 signaling axis was involved in the stemness and migration of SKCM cells. Furthermore, α5-nAChR expression was associated with ADAM10 expression, EMT marker expression and stemness marker expression in nicotine-related mice homograft tissues. These results suggest the role of the α5-nAChR/ADAM10 signaling pathway in nicotine-induced melanoma progression.
  
  
  
  
**JAK2**
-
6
of
10
  

### In vitro study of cold atmospheric plasma-activated liquids inhibits malignant melanoma by affecting macrophage polarization through the ROS/JAK2/STAT1 pathway.

6. Biomed Pharmacother. 2024 Jun;175:116657. doi: 10.1016/j.biopha.2024.116657 Epub 2024 Apr 30.
  
  
Chen C(1), Zhou S(1), Yang X(1), Ren M(1), Qi Y(1), Mao Y(1), Yang C(2).
  
  
Author information: (1)Department of Dermatology and Venereology, the Second Affiliated Hospital of ...
  
  
Melanoma is a prevalent malignant skin tumor known for its high invasive ability and a high rate of metastasis, making clinical treatment exceptionally challenging. Tumor-associated macrophages (TAMs) are the most abundant immune cells in the tumor microenvironment and play a crucial role in tumor survival and development. Cold atmospheric plasma (CAP) is an emerging tool for tumor treatment that has garnered attention from scholars due to its interaction with non-tumor cells in the tumor microenvironment. Here, we used the macrophage lines THP-1 and RAW264.7, as well as the melanoma cell lines A375 and MV3, as research subjects to investigate the effect of plasma-activated liquid (PAL) on macrophage differentiation and its inhibitory effect on melanoma cell proliferation. We confirmed that the killing effect of PAL on melanoma cells was selective. Using flow cytometry and PCR, we discovered that PAL can influence macrophage differentiation. Through in vitro cell coculture, we demonstrated that PAL-treated macrophages can significantly impede tumor cell development and progression, and the effect is more potent than that of PAL directly targeting tumor cells. Furthermore, we have proposed the hypothesis that PAL promotes the differentiation of macrophages into the M1 type through the ROS/JAK2/STAT1 pathway. To test the hypothesis, we employed catalase and fludarabine to block different sites of the pathway. The results were then validated through Western Blot, qPCR and ELISA. This study illustrates that PAL therapy is an effective tumor immunotherapy and expands the scope of tumor immunotherapy. Furthermore, these findings establish a theoretical foundation for potential clinical applications of PAL.
  
  
  
  
**JAK2**
-
7
of
10
  

### Fucoxanthin induces human melanoma cytotoxicity by thwarting the JAK2/STAT3/BCL-xL signaling axis.

7. Environ Toxicol. 2024 Jun;39(6):3356-3366. doi: 10.1002/tox.24193 Epub 2024 Mar 5.
  
  
Kuo MY(1), Dai WC(2), Chang JL(3), Chang JS(4)(5), Lee TM(6), Chang CC(2)(5)(7)(8)(9)(10).
  
  
Author information: (1)Pediatric Surgery Division, Department of Surgery, Tungs' Taichung MetroHarbo ...
  
  
Melanoma is the most lethal skin malignancy. Fucoxanthin is a marine carotenoid with significant anticancer activities. Intriguingly, Fucoxanthin's impact on human melanoma remains elusive. Signal Transducer and Activator of Transcription 3 (STAT3) represents a promising target in cancer therapy due to its persistent activation in various cancers, including melanoma. Herein, we revealed that Fucoxanthin is cytotoxic to human melanoma cell lines A2758 and A375 while showing limited cytotoxicity to normal human melanocytes. Apoptosis is a primary reason for Fucoxanthin's melanoma cytotoxicity, as the pan-caspase inhibitor z-VAD-fmk drastically abrogated Fucoxanthin-elicited clonogenicity blockage. Besides, Fucoxanthin downregulated tyrosine 705-phosphorylated STAT3 (p-STAT3 (Y705)), either inherently present in melanoma cells or inducible by interleukin 6 (IL-6) stimulation. Notably, ectopic expression of STAT3-C, a dominant-active STAT3 mutant, abolished Fucoxanthin-elicited melanoma cell apoptosis and clonogenicity inhibition, supporting the pivotal role of STAT3 blockage in Fucoxanthin's melanoma cytotoxicity. Moreover, Fucoxanthin lowered BCL-xL levels by blocking STAT3 activation, while ectopic BCL-xL expression rescued melanoma cells from Fucoxanthin-induced killing. Lastly, Fucoxanthin was found to diminish the levels of JAK2 with dual phosphorylation at tyrosine residues 1007 and 1008 in melanoma cells, suggesting that Fucoxanthin impairs STAT3 signaling by blocking JAK2 activation. Collectively, we present the first evidence that Fucoxanthin is cytotoxic selectively against human melanoma cells while sparing normal melanocytes. Mechanistically, Fucoxanthin targets the JAK2/STAT3/BCL-xL antiapoptotic axis to provoke melanoma cell death. This discovery implicates the potential application of Fucoxanthin as a chemopreventive or therapeutic strategy for melanoma management.
  
  
  
  
**JAK2**
-
8
of
10
  

### A safety evaluation of ruxolitinib for the treatment of polycythemia vera.

8. Expert Opin Drug Saf. 2024 Jan;23(1):1-7. doi: 10.1080/14740338.2023.2299391 Epub 2023 Dec 29.
  
  
Boldrini V(1), Vannucchi AM(1)(2), Guglielmelli P(1)(2).
  
  
Author information: (1)Department of Experimental and Clinical Medicine, University of Florence, Flo ...
  
  
INTRODUCTION: Polycythemia Vera (PV) is a chronic myeloproliferative neoplasm hallmarked by deregulated proliferation of hematopoietic stem cells leading to prevalent expansion of red cell mass, increased rate of vascular events, splenomegaly, disease-associated symptoms, and risk of evolution to secondary myelofibrosis and blast phase. PV is pathogenetically associated with autonomously persistent activation of JAK2, which causes overproduction of blood cells and an inflammatory condition responsible for the clinical manifestations of the disease. Extensively supported by preclinical studies, targeting JAK2-dependent signaling represents a rational therapeutic approach to PV, finally leading to the approval of ruxolitinib, a JAK1/2 inhibitor. AREAS COVERED (LITERATURE RESEARCH): We analyzed reports of phase 2 and phase 3 trials with ruxolitinib in PV and relevant literature dealing with efficacy and safety aspects, including most recent real-world reports. EXPERT OPINION: Ruxolitinib is the only JAK2 inhibitor approved for the treatment of PV with well-known efficacy for splenomegaly, symptoms, and potentially reduction of vascular events. The treatment regimen is notably manageable and safe, with the most prevalent side effects primarily encompassing myelosuppression, hyperlipidemia, non-melanoma skin cancer and infections, mainly reactivation of Herpes Zoster. These effects necessitate ongoing surveillance and proactive preventive measures.
  
  
  
  
**JAK2**
-
9
of
10
  

### Identifying survival of pan-cancer patients under immunotherapy using genomic mutation signature with large sample cohorts.

9. J Mol Med (Berl). 2024 Jan;102(1):69-79. doi: 10.1007/s00109-023-02398-1 Epub 2023 Nov 18.
  
  
Zhang L(#)(1), Wang Y(#)(2), Wang L(1), Wang M(1), Li S(1), He J(1), Ji J(1), Li K(3), Cao L(4).
  
  
Author information: (1)Department of Epidemiology and Biostatistics, Public Health College, Harbin M ...
  
  
Although immune checkpoint inhibitors have led to durable clinical response in multiple cancers, only a small proportion of patients respond to this treatment. Therefore, we aim to develop a predictive model that utilizes gene mutation profiles to accurately identify the survival of pan-cancer patients with immunotherapy. Here, we develop and evaluate three different nomograms using two cohorts containing 1,594 cancer patients whose mutation profiles are obtained by MSK-IMPACT sequencing and 230 cancer patients receiving whole-exome sequencing, respectively. Using eighteen genes (SETD2, BRAF, NCOA3, LATS1, IL7R, CREBBP, TET1, EPHA7, KDM5C, MET, KMT2D, RET, PAK7, CSF1R, JAK2, FAT1, ASXL1 and SPEN), the first nomogram stratifies patients from both cohorts into High-Risk and Low-Risk groups. Pan-cancer patients in the High-Risk group exhibit significantly shorter overall survival and progression-free survival than patients in the Low-Risk group in both cohorts. Meanwhile, the first nomogram also accurately identifies the survival of patients with melanoma or lung cancer undergoing immunotherapy, or pan-cancer patients treated with anti-PD-1/PD-L1 inhibitor or anti-CTLA-4 inhibitor. The model proposed is not a prognostic model for the survival of pan-cancer patients without immunotherapy, but a simple, effective and robust predictive model for pan-cancer patients' survival under immunotherapy, and could provide valuable assistance for clinical practice.
  
  
  
  
**JAK2**
-
10
of
10
  

### Decoding the molecular mechanism of stypoldione against breast cancer through network pharmacology and experimental validation.

10. Saudi J Biol Sci. 2023 Dec;30(12):103848. doi: 10.1016/j.sjbs.2023.103848 Epub 2023 Oct 21.
  
  
Qayoom H(1), Alshehri B(2), Ul Haq B(1), Almilaibary A(3), Alkhanani M(4), Ahmad Mir M(1).
  
  
Author information: (1)Department of Bioresources, School of Biological Sciences, University of Kash ...
  
  
Breast cancer is the primary factor contributing to female mortality worldwide. The incidence has overtaken lung cancer. It is the most difficult illness due to its heterogeneity and is made up of several subtypes, including Luminal A and B, basal-like, Her-2 overexpressed and TNBC. Amongst different breast carcinoma subtypes, TNBC is the most deadly breast cancer subtype. The hostile nature of TNBC is mainly attributed to its lack of three hormonal receptors and hence lack of targeted therapy. Furthermore, the current diagnostic options like radiotherapy, surgery and chemotherapy render unsuccessful due to recurrence, treatment side effects and drug resistance. The majority of anticancer drugs come from natural sources or is developed from them, making nature a significant source of many medicines. Marine-based constituents such as nucleotides, proteins, peptides, and amides are receiving a lot of interest in the field of cancer treatment due to their bioactive properties. The role of stypoldione in this study as a prospective treatment for breast carcinoma was examined, and we sought to comprehend the molecular means/pathways this chemical employs in breast carcinoma. The most promising possibility for an anti-cancer treatment is stypoldione, a marine chemical produced from the brown alga Stypopodium zonale. We investigated stypoldione's mode of action in breast cancer using the network pharmacology method, and we confirmed our research by using a number of computational tools, including UALCAN, cBioportal, TIMER, docking, and simulation. The findings revealed 92 common targets between the chemical and breast cancer target network. Additionally, we found that stypoldione targets a number of unregulated genes in breast cancer, including: ESR1, HSP90AA1, CXCL8, PTGS2, APP, MDM2, JAK2, KDR, LCK, GRM5, MAPK14, KIT, and several signaling pathways such as FOXO signaling pathway, VEGF pathway, calcium signaling pathway, MAPK/ERK pathway and Neuroactive ligand-receptor interaction. The examined medication demonstrated a strong affinity for the major targets, according to a docking analysis. The best hit compound produced a stable protein-ligand pair, as predicted by molecular dynamics simulations. Our results are supported by the fact that when in-vitro assays were done on melanoma using stypoldione compound it was found that its mechanisms of action involved the PI3K/mTOR/Akt and NF-kB pathways. This study was set out to inspect the possible value of stypoldione as a breast cancer cure and to get a deeper understanding of the molecular mechanisms by which this drug acts on breast cancer.
  
  
  
  
  

---

  

# MAML2

**Signature gene in:** Dendritic cells
  
**Differentially expressed in:** no cell type
  
  
**MAML2**
-
1
of
6
  

### Genomic and Transcriptomic Profiling of Digital Papillary Adenocarcinomas Reveals Alterations in Matrix Remodeling and Metabolic Genes.

1. J Cutan Pathol. 2025 Apr;52(4):309-316. doi: 10.1111/cup.14782 Epub 2025 Jan 6.
  
  
Bayraktar EC(1), Aung PP(2), Gill P(2), Shen G(1), Vasudevaraja V(1), Lai Z(2), Chiriboga L(1), Ivan ...
  
  
Author information: (1)Department of Pathology and Dermatology, NYU Langone Medical Center, New York ...
  
  
BACKGROUND: Digital papillary adenocarcinoma (DPAC) is a rare but aggressive cutaneous malignant sweat gland neoplasm that occurs on acral sites. Despite its clinical significance, the cellular and genetic characteristics of DPAC remain incompletely understood. METHODS: We conducted a comprehensive genomic and transcriptomic analysis of DPAC (n = 14) using targeted next-generation DNA and RNA sequencing, along with gene expression profiling employing the Nanostring Technologies nCounter IO 360 Panel. Gene expression in DPAC was compared to that in hidradenoma (n = 10). Immunohistochemistry was employed to validate gene expression. RESULTS: Two out of eight DPACs showed fusion gene rearrangements (CRTC3::MAML2 and TRPS1::PLAG1). No uniform mutational signature was detected in DPAC. Comparative gene expression analysis revealed an enrichment of genes related to matrix remodeling, metabolism, and DNA damage repair. Hallmark pathway analysis demonstrated significant upregulation of E2F target genes in DPAC compared to hidradenoma (p = 0.00710). Human papillomavirus-42 was found to be positive in all of our tested DPAC cases. Immunohistochemistry confirmed increased protein expression of CD56, CDC20, and SOX10 in DPAC. Notably, most DPAC tumors also exhibited B-cell infiltration, as indicated by CD20 staining. CONCLUSIONS: Our findings reveal novel fusions and validate altered replication pathways related to HPV42 in DPAC.
  
  
  
  
**MAML2**
-
2
of
6
  

### Comprehensive mutational profiling identifies new driver events in cutaneous leiomyosarcoma.

2. Br J Dermatol. 2025 Jan 24;192(2):335-343. doi: 10.1093/bjd/ljae386
  
  
van der Weyden L(1), Del Castillo Velasco-Herrera M(1), Cheema S(1), Wong K(1), Boccacino JM(1), Off ...
  
  
Author information: (1)Wellcome Sanger Institute, Wellcome Genome Campus, Hinxton, UK. (2)Department ...
  
  
BACKGROUND: Cutaneous leiomyosarcoma (cLMS) is a rare soft-tissue neoplasm, showing smooth muscle differentiation, that arises from the mesenchymal cells of the dermis. To date, genetic investigation of these tumours has involved studies with small sample sizes and limited analyses that identified recurrent somatic mutations in RB1 and TP53, copy number gain of MYOCD and IGF1R, and copy number loss of PTEN. OBJECTIVES: To better understand the molecular pathogenesis of cLMS, we comprehensively explored the mutational landscape of these rare tumours to identify candidate driver events. METHODS: In this retrospective, multi-institutional study, we performed whole-exome sequencing and RNA sequencing in 38 cases of cLMS. RESULTS: TP53 and RB1 were identified as significantly mutated and thus represent validated driver genes of cLMS. COSMIC mutational signatures SBS7a/b and DBS1 were recurrent; thus, ultraviolet light exposure may be an aetiological factor driving cLMS. Analysis of significantly recurrent somatic copy number alterations, which represent candidate driver events, found focal (< 10 Mb) deletions encompassing TP53 and KDM6B, and amplifications encompassing ZMYM2, MYOCD, MAP2K4 and NCOR1. A larger (24 Mb) recurrent deletion encompassing CYLD was also identified as significant. Significantly recurrent broad copy number alterations, involving at least half of a chromosome arm, included deletions of 6p/q, 10p/q, 11q, 12q, 13q and 16p/q, and amplification of 15q. Notably PTEN is located on 10q, RB1 on 13q and IGFR1 on 15q. Fusion gene analysis identified recurrent CRTC1/CRTC3::MAML2 fusions, as well as many novel fusions in individual samples. CONCLUSIONS: Our analysis of the largest number of cases of cLMS to date highlights the importance of large cohort sizes and exploration beyond small targeted gene panels when performing molecular analyses, as it allowed a comprehensive exploration of the mutational landscape of these tumours and identification of novel candidate driver events. It also uniquely afforded the opportunity to compare the molecular phenotype of cLMS with LMS of other tissue types, such as uterine and soft-tissue LMS. Given that molecular profiling has resulted in the development of novel targeted treatment approaches for uterine and soft-tissue LMS, our study now allows the same opportunities to become available for patients with cLMS.
  
  
  
  
**MAML2**
-
3
of
6
  

### Faecal Proteomics and Functional Analysis of Equine Melanocytic Neoplasm in Grey Horses.

3. Vet Sci. 2022 Feb 21;9(2):94. doi: 10.3390/vetsci9020094
  
  
Tesena P(1)(2), Kingkaw A(3), Phaonakrop N(4), Roytrakul S(4), Limudomporn P(5), Vongsangnak W(5)(6) ...
  
  
Author information: (1)Graduate Student in Animal Health and Biomedical Science Program, Faculty of ...
  
  
Equine melanocytic neoplasm (EMN) is a common disease in older grey horses. The purpose of this study was to examine the potential proteins throughout EMN stages from faecal proteomic outlining using functional analysis. Faecal samples were collected from the rectum of 25 grey horses divided into three groups; normal group without EMN (n = 10), mild EMN (n = 6) and severe EMN (n = 9). Based on the results, 5910 annotated proteins out of 8509 total proteins were assessed from proteomic profiling. We observed differentially expressed proteins (DEPs) between the normal group and the EMN group, and 109 significant proteins were obtained, of which 28 and 81 were involved in metabolic and non-metabolic functions, respectively. We found 10 proteins that play a key role in lipid metabolism, affecting the tumour microenvironment and, consequently, melanoma progression. Interestingly, FOSL1 (FOS like 1, AP-1 transcription factor subunit) was considered as a potential highly expressed protein in a mild EMN group involved in melanocytes cell and related melanoma. Diacylglycerol kinase (DGKB), TGc domain-containing protein (Tgm2), structural maintenance of chromosomes 4 (SMC4) and mastermind-like transcriptional coactivator 2 (MAML2) were related to lipid metabolism, facilitating melanoma development in the severe-EMN group. In conclusion, these potential proteins can be used as candidate biomarkers for the monitoring of early EMN, the development of EMN, further prevention and treatment.
  
  
  
  
**MAML2**
-
4
of
6
  

### Mucoepidermoid carcinoma with extensive spindled morphology and melanocytic marker expression.

4. Hum Pathol. 2017 Sep;67:181-186. doi: 10.1016/j.humpath.2017.03.010 Epub 2017 Apr 12.
  
  
Oide T(1), Hiroshima K(2), Takahashi Y(3), Fugo K(4), Yamatoji M(5), Kasamatsu A(6), Endo-Sakamoto Y ...
  
  
Author information: (1)Department of Pathology, Tokyo Women's Medical University Yachiyo Medical Cen ...
  
  
Mucoepidermoid carcinoma (MEC) is the most common malignant neoplasm of the salivary gland. Albeit common, histologic variants have rarely been noted in MEC. Here, we report a 49-year-old man with a sublingual gland tumor. Histologically, the tumor was composed of spindle cells arranged in interlacing fascicules or globular nests. A few bland small glands containing mucous cells were also scattered. The spindle tumor cells completely lacked immunoreactivity for cytokeratin, and exhibited immunoreactivity for vimentin, S-100, HMB-45, Melan A, and SOX10. The tumor was initially suspected to be clear cell sarcoma, malignant melanoma, or perivascular epithelioid cell tumor with a few entrapped nonneoplastic duct epitheliums. However, reverse-transcription polymerase chain reaction revealed the CRTC3-MAML2 fusion gene product diagnostic of MEC. In fact, a very minor component of the epithelial cells was reminiscent of conventional MEC, whereas major spindled tumor cells possessed markedly altered differentiation. This is the first case report of MEC with extensive spindled morphology and melanocytic marker expression.
  
  
  
  
**MAML2**
-
5
of
6
  

### Functional Variants in Notch Pathway Genes NCOR2, NCSTN, and MAML2 Predict Survival of Patients with Cutaneous Melanoma.

5. Cancer Epidemiol Biomarkers Prev. 2015 Jul;24(7):1101-10. doi: 10.1158/1055-9965.EPI-14-1380-T Epub 2015 May 7.
  
  
Zhang W(1), Liu H(2), Liu Z(2), Zhu D(3), Amos CI(3), Fang S(4), Lee JE(4), Wei Q(5).
  
  
Author information: (1)Department of Medicine, Duke University School of Medicine and Duke Cancer In ...
  
  
BACKGROUND: The Notch signaling pathway is constitutively activated in human cutaneous melanoma to promote growth and aggressive metastatic potential of primary melanoma cells. Therefore, genetic variants in Notch pathway genes may affect the prognosis of cutaneous melanoma patients. METHODS: We identified 6,256 SNPs in 48 Notch genes in 858 cutaneous melanoma patients included in a previously published cutaneous melanoma genome-wide association study dataset. Multivariate and stepwise Cox proportional hazards regression and false-positive report probability corrections were performed to evaluate associations between putative functional SNPs and cutaneous melanoma disease-specific survival. Receiver operating characteristic curve was constructed, and area under the curve was used to assess the classification performance of the model. RESULTS: Four putative functional SNPs of Notch pathway genes had independent and joint predictive roles in survival of cutaneous melanoma patients. The most significant variant was NCOR2 rs2342924 T>C (adjusted HR, 2.71; 95% confidence interval, 1.73-4.23; Ptrend = 9.62 × 10(-7)), followed by NCSTN rs1124379 G>A, NCOR2 rs10846684 G>A, and MAML2 rs7953425 G>A (Ptrend = 0.005, 0.005, and 0.013, respectively). The receiver operating characteristic analysis revealed that area under the curve was significantly increased after adding the combined unfavorable genotype score to the model containing the known clinicopathologic factors. CONCLUSIONS: Our results suggest that SNPs in Notch pathway genes may be predictors of cutaneous melanoma disease-specific survival. IMPACT: Our discovery offers a translational potential for using genetic variants in Notch pathway genes as a genotype score of biomarkers for developing an improved prognostic assessment and personalized management of cutaneous melanoma patients.
  
  
  
  
**MAML2**
-
6
of
6
  

### CRTC1/MAML2 fusion transcript in Warthin's tumor and mucoepidermoid carcinoma: evidence for a common genetic association.

6. Genes Chromosomes Cancer. 2008 Apr;47(4):309-14. doi: 10.1002/gcc.20534
  
  
Bell D(1), Luna MA, Weber RS, Kaye FJ, El-Naggar AK.
  
  
Author information: (1)Department of Pathology, The University of Texas M. D. Anderson Cancer Center ...
  
  
Translocations and gene fusions have an important early role in tumorigenesis. The t(11;19) translocation and its CRTC1/MAML2 fusion transcript have been identified in several examples of both Warthin's tumor and mucoepidermoid carcinoma and are believed to be associated with the development of a subset of these tumors. To determine whether Warthin's tumor and mucoepidermoid carcinoma are genetically related, we used reverse transcriptase-polymerase chain reaction and DNA sequencing to analyze microdissected components of three tumors consisting of Warthin's tumor and mucoepidermoid carcinoma. We also investigated a metastatic melanoma to Warthin's tumor and a Warthin's carcinoma of the parotid gland for comparison. The fusion transcript was identified in both Warthin's tumor and matching mucoepidermoid carcinoma components of all three tumors, in the Warthin's carcinoma, and in the Warthin's tumor component but not in the metastatic melanoma. The results provide evidence for a link between the t(11;19) fusion gene and the development of a subset of Warthin's tumors with concurrent mucoepidermoid carcinoma and possible malignant transformation to Warthin's carcinoma. This article contains Supplementary Material available at http://www.interscience.wiley.com/jpages/1045-2257/suppmat.
  
  
  
  
  

---

  

# MAP2K4

**Signature gene in:** Endothelial cells
  
**Differentially expressed in:** no cell type
  
  
**MAP2K4**
-
1
of
10
  

### Clinical outcomes and genomic profiles of MAP2K1-mutated primary cutaneous melanocytic tumours.

1. EBioMedicine. 2025 Apr;114:105643. doi: 10.1016/j.ebiom.2025.105643 Epub 2025 Mar 18.
  
  
Ebbelaar CF(1), Jansen AML(2), Speet LCM(3), Schutgens F(4), Zoetemeyer S(5), Cleton-Jansen AM(2), v ...
  
  
Author information: (1)Department of Dermatology, Leiden University Medical Centre, Leiden, the Neth ...
  
  
BACKGROUND: Primary cutaneous melanocytic tumours harbouring MAP2K1 mutations without second-hit genomic alterations represent a subclass of neoplasms with poorly understood biological behaviour. This study aimed to investigate the clinical outcomes and genomic characteristics of these tumours. METHODS: This cohort study included primary cutaneous melanocytic tumours with MAP2K1 mutations from patients at two academic centres (Leiden University Medical Centre and University Medical Centre Utrecht). These mutations were categorised into three functional classes: Class I (RAF-dependent), Class II (RAF-regulated), and Class III (RAF-independent). Tumours underwent histopathological evaluation, next-generation sequencing (NGS), and copy number variation (CNV) analysis and were categorised as non-melanoma or melanoma. Clinical outcomes were assessed for each mutation class during follow-up visits and through the Dutch Pathology Database (PALGA) using the composite outcome of metastatic melanoma (recurrence, metastasis, or melanoma-related death). FINDINGS: A total of 102 patients were included, with tumours classified as melanoma in 52 (51%) and non-melanoma in 50 (49%). The tumours displayed spitzoid histomorphology in over two-thirds of cases and harboured 31 distinct MAP2K1 mutations: 20 Class I (19.6%), 56 Class II (54.9%), and 26 Class III (25.5%). Class I mutations exclusively co-occurred with BRAF or NRAS mutations, while Class II and III mutations often acted as sole tumour drivers. Of the tumours with Class I mutations, 95% were classified as melanoma, which was less frequently the case for Class II (risk ratio [RR] 0.43 [95% CI: 0.31-0.60], p < 0.001) and Class III mutations (RR 0.40 [95% CI: 0.25-0.67], p < 0.001). MAP2K1 mutation Class and TERT-p mutation status were independent predictors for the composite outcome. Compared to Class I mutations, Class II mutations were negatively associated with the composite outcome (odds ratio [OR] 0.16 [95% CI: 0.03-0.75], p = 0.03), whereas Class III mutations were not associated (OR 0.31 [95% CI: 0.05-1.54], p = 0.16). TERT-p mutations were positively associated with the composite outcome (OR 23.1, 95% CI: 3.99-439.8, p < 0.005). INTERPRETATION: Class I MAP2K1 mutations typically occur alongside other MAPK pathway mutations and may contribute to aggressive melanoma behaviour. In contrast, Class II and III MAP2K1 mutations can independently drive melanocytic tumourigenesis with a potential for metastasis, aligning with conventional melanomagenesis pathways, despite their frequent spitzoid histomorphology. FUNDING: This research was supported by the Hanarth Fund.
  
  
  
  
**MAP2K4**
-
2
of
10
  

### Large scale multi-omic analysis identifies anatomic differences and immunogenic potential in subtypes of leiomyosarcoma.

2. Clin Cancer Res. 2025 Mar 18. doi: 10.1158/1078-0432.CCR-24-2503 Online ahead of print.
  
  
Lagos G(1), Groisberg R(2), Elliott A(3), Dizon DS(4), Seeber A(5), Gibney GT(6), von Mehren M(7), C ...
  
  
Author information: (1)Brown University, Providence, RI, United States. (2)Rutgers Cancer Institute ...
  
  
PURPOSE: Comprehensive molecular profiling was used to define the genomic and immune landscapes of leiomyosarcomas (LMS) by anatomic subtype, which have not been completely characterized. DESIGN: 1115 LMS samples, categorized into uterine (uLMS), retroperitoneal (rpLMS), or other (oLMS), underwent DNA/RNA sequencing (Caris Life Sciences). Genomic/transcriptomic profiles were compared across subtypes. Immune profiling was compared to melanoma (n=1255), an immunogenic tumor. Insurance claims data were used to infer real-world outcomes with immune checkpoint inhibitors (ICI) in LMS. RESULTS: uLMS (n=701) were molecularly distinct from rpLMS (n=166) and oLMS (n=248). RB1 mutations and MAP2K4 copy number amplification were more common in non-uLMS. MED12 mutations were almost exclusive to uLMS. Traditional ICI response biomarkers (i.e. PD-L1) didn't vary by anatomic site. Non-uLMS demonstrated upregulated immune-related gene sets, including interferon and inflammatory response pathways, and higher immune cell infiltration, especially CD8+ T cells and B cells (>2-fold increase, p<0.0001). LMS had lower immune cell abundance and T-cell inflamed scores (TIS) compared to melanoma, though 11% of oLMS samples had high TIS scores. In a real-world cohort (n=138), 29% of LMS patients receiving ICI were treated >6 months, indicating potential clinical benefit. CONCLUSION: Comprehensive profiling suggested that uLMS represents a molecularly distinct disease from non-uLMS. While traditional ICI response biomarkers were similar across anatomic subtypes, uLMS were immune cold compared to non-uLMS. Signals for ICI responsiveness, such as high TIS and immune cell abundance, in some tumors suggest that further research into immunotherapies for LMS is warranted.
  
  
  
  
**MAP2K4**
-
3
of
10
  

### Role of Targeted Sequencing in Routine Diagnostics of Spitz Melanocytic Neoplasms-An Analysis of 70 Cases.

3. J Cutan Pathol. 2025 Feb;52(2):141-153. doi: 10.1111/cup.14750 Epub 2024 Nov 7.
  
  
Šekoranja D(1), Zupan A(1), Matjašič A(1), Boštjančič E(1), Calonje E(2), Pižem J(1).
  
  
Author information: (1)Institute of Pathology, Faculty of Medicine, University of Ljubljana, Ljublja ...
  
  
BACKGROUND: There is growing evidence that the Spitz group of melanocytic neoplasms should be restricted to those harboring kinase receptor fusions and HRAS mutations/11p15 amplification. The presence of genomic alterations characteristic of conventional melanomas (BRAF and NRAS mutations) precludes a diagnosis of a Spitz neoplasm. It is often challenging to distinguish Spitz neoplasms from conventional melanomas with spitzoid morphology on histopathological grounds alone. METHODS: We report a series of 70 consecutive melanocytic tumors in which targeted sequencing was indicated to distinguish Spitz from spitzoid neoplasms and to classify Spitz neoplasms along the biological spectrum. RESULTS: Final diagnoses incorporating molecular results included 12 conventional melanomas (nine of which with NRAS mutations), five Spitz melanomas, 35 atypical Spitz tumors, eight Spitz nevi, three melanocytic tumors with a MAP2K1 mutation, and seven desmoplastic Spitz nevi/tumors. There were significant discrepancies between initial diagnoses and final diagnoses after incorporating molecular results in 24 (34%) cases, including nine conventional melanomas favored to be Spitz neoplasms and nine Spitz neoplasms favored to be conventional melanomas. CONCLUSIONS: It is often not possible to reliably distinguish Spitz neoplasms from spitzoid melanocytic tumors without identifying their driver genomic alterations. Applying next-generation sequencing in diagnostically problematic tumors improves diagnostic accuracy.
  
  
  
  
**MAP2K4**
-
4
of
10
  

### Comprehensive mutational profiling identifies new driver events in cutaneous leiomyosarcoma.

4. Br J Dermatol. 2025 Jan 24;192(2):335-343. doi: 10.1093/bjd/ljae386
  
  
van der Weyden L(1), Del Castillo Velasco-Herrera M(1), Cheema S(1), Wong K(1), Boccacino JM(1), Off ...
  
  
Author information: (1)Wellcome Sanger Institute, Wellcome Genome Campus, Hinxton, UK. (2)Department ...
  
  
BACKGROUND: Cutaneous leiomyosarcoma (cLMS) is a rare soft-tissue neoplasm, showing smooth muscle differentiation, that arises from the mesenchymal cells of the dermis. To date, genetic investigation of these tumours has involved studies with small sample sizes and limited analyses that identified recurrent somatic mutations in RB1 and TP53, copy number gain of MYOCD and IGF1R, and copy number loss of PTEN. OBJECTIVES: To better understand the molecular pathogenesis of cLMS, we comprehensively explored the mutational landscape of these rare tumours to identify candidate driver events. METHODS: In this retrospective, multi-institutional study, we performed whole-exome sequencing and RNA sequencing in 38 cases of cLMS. RESULTS: TP53 and RB1 were identified as significantly mutated and thus represent validated driver genes of cLMS. COSMIC mutational signatures SBS7a/b and DBS1 were recurrent; thus, ultraviolet light exposure may be an aetiological factor driving cLMS. Analysis of significantly recurrent somatic copy number alterations, which represent candidate driver events, found focal (< 10 Mb) deletions encompassing TP53 and KDM6B, and amplifications encompassing ZMYM2, MYOCD, MAP2K4 and NCOR1. A larger (24 Mb) recurrent deletion encompassing CYLD was also identified as significant. Significantly recurrent broad copy number alterations, involving at least half of a chromosome arm, included deletions of 6p/q, 10p/q, 11q, 12q, 13q and 16p/q, and amplification of 15q. Notably PTEN is located on 10q, RB1 on 13q and IGFR1 on 15q. Fusion gene analysis identified recurrent CRTC1/CRTC3::MAML2 fusions, as well as many novel fusions in individual samples. CONCLUSIONS: Our analysis of the largest number of cases of cLMS to date highlights the importance of large cohort sizes and exploration beyond small targeted gene panels when performing molecular analyses, as it allowed a comprehensive exploration of the mutational landscape of these tumours and identification of novel candidate driver events. It also uniquely afforded the opportunity to compare the molecular phenotype of cLMS with LMS of other tissue types, such as uterine and soft-tissue LMS. Given that molecular profiling has resulted in the development of novel targeted treatment approaches for uterine and soft-tissue LMS, our study now allows the same opportunities to become available for patients with cLMS.
  
  
  
  
**MAP2K4**
-
5
of
10
  

### An Unexpected Finding of a PTPN11 Germline Mutation in a Patient With a Melanocytic Lesion With a Somatic MAP2K1 Mutation. Coincidence or Not?

5. J Cutan Pathol. 2025 Jan;52(1):20-23. doi: 10.1111/cup.14730 Epub 2024 Oct 11.
  
  
van der Woude S(1), Klein Wassink-Ruiter JS(2), Kluiver J(1), de Jonge M(3), Diercks GFH(1).
  
  
Author information: (1)Department of Pathology, University Medical Center Groningen, University of G ...
  
  
Melanocytic tumors are a diverse group of lesions and are traditionally classified based on a combination of clinical presentation as well as histological examination. More recently, molecular diagnostics has become an increasingly important part of differentiating different melanocytic lesions in the current WHO standards. This molecular testing, however, can result in unexpected findings. In this report, we describe that molecular testing of a clinical atypical melanocytic lesion showed a mutation in the MAP2K1 gene as well as an unexpected germline mutation in PTPN11, indicative of Noonan syndrome. Based on these findings we concluded that the patient had a MAP2K1 associated melanocytic lesion with Noonan syndrome as an incidental finding. Melanomas are classically not associated with Noonan syndrome. However, we hypothesized that the germline mutations of PTPN11 and the somatic second hit mutation in the MAP2K1 genes might be involved in the formation of the aforementioned lesion. As they are both part of the RAS-MAPK pathway. Furthermore, with the expansion of molecular diagnostics in melanomas, we expect to find an increase in unexpected (germline) mutations.
  
  
  
  
**MAP2K4**
-
6
of
10
  

### Sclerosing melanocytic tumors with MAP2K1 in-frame deletions and copy number gains in 15q: A distinctive pathway of nevogenesis.

6. J Cutan Pathol. 2024 Apr;51(4):284-287. doi: 10.1111/cup.14586 Epub 2023 Dec 27.
  
  
Hamad J(1), Shaw B(2), Kohen L(1), Linos K(3), Friedman BJ(1).
  
  
Author information: (1)Department of Dermatology, Henry Ford Health, Detroit, Michigan, USA. (2)Depa ...
  
  
Comment on J Cutan Pathol. 2023 Dec;50(12):1083-1093. doi: 10.1111/cup.14502.
  
  
  
  
**MAP2K4**
-
7
of
10
  

### Clinical, Morphologic, and Molecular Features of Benign and Intermediate-grade Melanocytic Tumors With Activating Mutations in MAP2K1.

7. Am J Surg Pathol. 2023 Dec 1;47(12):1438-1448. doi: 10.1097/PAS.0000000000002131 Epub 2023 Sep 29.
  
  
Fumero-Velázquez M(1), Hagstrom M(1), Dhillon S(1), Geraminejad T(1), Olivares S(1), Donati M(2), No ...
  
  
Author information: (1)Department of Dermatology, Feinberg School of Medicine, Northwestern Universi ...
  
  
Activating mutations in MAP2K1 can be seen in benign and intermediate-grade melanocytic neoplasms with spitzoid morphology. We analyzed the clinical, histopathologic, and genetic features for 16 cases of benign and intermediate-grade melanocytic tumors harboring activating MAP2K1 mutations. We compared them to Spitz neoplasms with characteristic Spitz fusions or HRAS mutation. We also compared the mutational pattern of benign and intermediate-grade MAP2K1 -mutated neoplasms and melanomas with activating MAP2K1 mutations. Among the 16 cases, the favored morphologic diagnosis was Spitz nevus (8/16), atypical Spitz tumors (6/16), and deep penetrating nevus (2/16). The 2 most common architectural patterns seen included a plaque-like silhouette with fibroplasia around the rete reminiscent of a dysplastic nevus (n=7) or a wedge-shaped or nodular pattern with the plexiform arrangement of the nests aggregating around the adnexa or neurovascular bundle (n=8). The cases with dysplastic architecture and spitzoid cytology resembled dysplastic Spitz nevi. Compared with true Spitz neoplasms, MAP2K1 -mutated neoplasms occurred in older age groups and had more frequent pagetosis and a lower average mitotic count. The most common type of mutation in the benign and intermediate-grade cases in the literature involves an in-frame deletion, while, in melanomas, missense mutations are predominant. Benign and intermediate-grade melanocytic neoplasms with activating mutations in MAP2K1 can have morphologic overlap with Spitz neoplasms. A significant proportion of melanomas also have activating MAP2K1 mutations. In-frame deletions are predominantly seen in the benign and intermediate-grade cases, and missense mutations are predominantly seen in melanomas.
  
  
  
  
**MAP2K4**
-
8
of
10
  

### MAP2K1-mutated melanocytic tumors have reproducible histopathologic features and share similarities with melanocytic tumors with BRAF V600E mutations.

8. J Cutan Pathol. 2023 Dec;50(12):1083-1093. doi: 10.1111/cup.14502 Epub 2023 Aug 11.
  
  
Alomari AK(1)(2), Harms PW(3)(4), Andea AA(3)(4), Warren SJ(1)(2).
  
  
Author information: (1)Department of Pathology, Indiana University School of Medicine, Indianapolis, ...
  
  
Comment in J Cutan Pathol. 2024 Apr;51(4):284-287. doi: 10.1111/cup.14586.
  
  
  
  
**MAP2K4**
-
9
of
10
  

### Response and Resistance to Trametinib in MAP2K1-Mutant Triple-Negative Melanoma.

9. Int J Mol Sci. 2023 Feb 24;24(5):4520. doi: 10.3390/ijms24054520
  
  
Krebs FS(1)(2), Moura B(3), Missiaglia E(4)(5), Aedo-Lopez V(6), Michielin O(5)(7)(8)(9)(10), Tsanto ...
  
  
Author information: (1)Computer-Aided Molecular Engineering, Department of Oncology UNIL-CHUV, Unive ...
  
  
The development of targeted therapies for non-BRAF p.Val600-mutant melanomas remains a challenge. Triple wildtype (TWT) melanomas that lack mutations in BRAF, NRAS, or NF1 form 10% of human melanomas and are heterogeneous in their genomic drivers. MAP2K1 mutations are enriched in BRAF-mutant melanoma and function as an innate or adaptive resistance mechanism to BRAF inhibition. Here we report the case of a patient with TWT melanoma with a bona fide MAP2K1 mutation without any BRAF mutations. We performed a structural analysis to validate that the MEK inhibitor trametinib could block this mutation. Although the patient initially responded to trametinib, he eventually progressed. The presence of a CDKN2A deletion prompted us to combine a CDK4/6 inhibitor, palbociclib, with trametinib but without clinical benefit. Genomic analysis at progression showed multiple novel copy number alterations. Our case illustrates the challenges of combining MEK1 and CDK4/6 inhibitors in case of resistance to MEK inhibitor monotherapy.
  
  
  
  
**MAP2K4**
-
10
of
10
  

### High-Throughput Functional Evaluation of MAP2K1 Variants in Cancer.

10. Mol Cancer Ther. 2023 Feb 1;22(2):227-239. doi: 10.1158/1535-7163.MCT-22-0302
  
  
Mizuno S(1)(2)(3), Ikegami M(1)(4), Koyama T(5), Sunami K(6), Ogata D(7), Kage H(8), Yanagaki M(1)(9 ...
  
  
Author information: (1)Division of Cellular Signaling, National Cancer Center Research Institute, Ts ...
  
  
Comment in 1535-7163. doi: 10.1158/1535-7163.MCT-22-2-HI.
  
  
  
  
  

---

  

# MECOM

**Signature gene in:** Tumor cells
  
**Differentially expressed in:** no cell type
  
  
**MECOM**
-
1
of
4
  

### Patient-specific identification of genome-wide DNA-methylation differences between intracranial and extracranial melanoma metastases.

1. Sci Rep. 2023 Jan 9;13(1):444. doi: 10.1038/s41598-022-24940-w
  
  
Kraft T(1), Grützmann K(1), Meinhardt M(2), Meier F(3)(4), Westphal D(3)(4), Seifert M(5)(6).
  
  
Author information: (1)Institute for Medical Informatics and Biometry (IMB), Carl Gustav Carus Facul ...
  
  
Melanomas frequently metastasize to distant organs and especially intracranial metastases still represent a major clinical challenge. Epigenetic reprogramming of intracranial metastases is thought to be involved in therapy failure, but so far only little is known about patient-specific DNA-methylation differences between intra- and extracranial melanoma metastases. Hierarchical clustering of the methylomes of 24 patient-matched intra- and extracranial melanoma metastases pairs revealed that intra- and extracranial metastases of individual patients were more similar to each other than to metastases in the same tissue from other patients. Therefore, a personalized analysis of each metastases pair was done by a Hidden Markov Model to classify methylation levels of individual CpGs as decreased, unchanged or increased in the intra- compared to the extracranial metastasis. The predicted DNA-methylation alterations were highly patient-specific differing in the number and methylation states of altered CpGs. Nevertheless, four important general observations were made: (i) intracranial metastases of most patients mainly showed a reduction of DNA-methylation, (ii) cytokine signaling was most frequently affected by differential methylation in individual metastases pairs, but also MAPK, PI3K/Akt and ECM signaling were often altered, (iii) frequently affected genes were mainly involved in signaling, growth, adhesion or apoptosis, and (iv) an enrichment of functional terms related to channel and transporter activities supports previous findings for a brain-like phenotype. In addition, the derived set of 17 signaling pathway genes that distinguished intra- from extracranial metastases in more than 50% of patients included well-known oncogenes (e.g. PRKCA, DUSP6, BMP4) and several other genes known from neuronal disorders (e.g. EIF4B, SGK1, CACNG8). Moreover, associations of gene body methylation alterations with corresponding gene expression changes revealed that especially the three signaling pathway genes JAK3, MECOM, and TNXB differ strongly in their expression between patient-matched intra- and extracranial metastases. Our analysis contributes to an in-depth characterization of DNA-methylation differences between patient-matched intra- and extracranial melanoma metastases and may provide a basis for future experimental studies to identify targets for new therapeutic approaches.
  
  
  
  
**MECOM**
-
2
of
4
  

### Machine learning approach informs biology of cancer drug response.

2. BMC Bioinformatics. 2022 May 17;23(1):184. doi: 10.1186/s12859-022-04720-z
  
  
Zhu EY(1)(2)(3)(4), Dupuy AJ(5)(6).
  
  
Author information: (1)Department of Anatomy and Cell Biology, The University of Iowa, Iowa City, IA ...
  
  
BACKGROUND: The mechanism of action for most cancer drugs is not clear. Large-scale pharmacogenomic cancer cell line datasets offer a rich resource to obtain this knowledge. Here, we present an analysis strategy for revealing biological pathways that contribute to drug response using publicly available pharmacogenomic cancer cell line datasets. METHODS: We present a custom machine-learning based approach for identifying biological pathways involved in cancer drug response. We test the utility of our approach with a pan-cancer analysis of ML210, an inhibitor of GPX4, and a melanoma-focused analysis of inhibitors of BRAFV600. We apply our approach to reveal determinants of drug resistance to microtubule inhibitors. RESULTS: Our method implicated lipid metabolism and Rac1/cytoskeleton signaling in the context of ML210 and BRAF inhibitor response, respectively. These findings are consistent with current knowledge of how these drugs work. For microtubule inhibitors, our approach implicated Notch and Akt signaling as pathways that associated with response. CONCLUSIONS: Our results demonstrate the utility of combining informed feature selection and machine learning algorithms in understanding cancer drug response.
  
  
  
  
**MECOM**
-
3
of
4
  

### Targeted next-generation sequencing reveals high frequency of mutations in epigenetic regulators across treatment-naïve patient melanomas.

3. Clin Epigenetics. 2015 Jun 9;7(1):59. doi: 10.1186/s13148-015-0091-3 eCollection 2015.
  
  
Lee JJ(1), Sholl LM(1), Lindeman NI(1), Granter SR(1), Laga AC(1), Shivdasani P(1), Chin G(1), Luke ...
  
  
Author information: (1)Department of Pathology, Brigham and Women's Hospital, Harvard Medical School ...
  
  
BACKGROUND: Recent developments in genomic sequencing have advanced our understanding of the mutations underlying human malignancy. Melanoma is a prototype of an aggressive, genetically heterogeneous cancer notorious for its biologic plasticity and predilection towards developing resistance to targeted therapies. Evidence is rapidly accumulating that dysregulated epigenetic mechanisms (DNA methylation/demethylation, histone modification, non-coding RNAs) may play a central role in the pathogenesis of melanoma. Therefore, we sought to characterize the frequency and nature of mutations in epigenetic regulators in clinical, treatment-naïve, patient melanoma specimens obtained from one academic institution. RESULTS: Targeted next-generation sequencing for 275 known and investigative cancer genes (of which 41 genes, or 14.9 %, encoded an epigenetic regulator) of 38 treatment-naïve patient melanoma samples revealed that 22.3 % (165 of 740) of all non-silent mutations affected an epigenetic regulator. The most frequently mutated genes were BRAF, MECOM, NRAS, TP53, MLL2, and CDKN2A. Of the 40 most commonly mutated genes, 12 (30.0 %) encoded epigenetic regulators, including genes encoding enzymes involved in histone modification (MECOM, MLL2, SETD2), chromatin remodeling (ARID1B, ARID2), and DNA methylation and demethylation (TET2, IDH1). Among the 38 patient melanoma samples, 35 (92.1 %) harbored at least one mutation in an epigenetic regulator. The genes with the highest number of total UVB-signature mutations encoded epigenetic regulators, including MLL2 (100 %, 16 of 16) and MECOM (82.6 %, 19 of 23). Moreover, on average, epigenetic genes harbored a significantly greater number of UVB-signature mutations per gene than non-epigenetic genes (3.7 versus 2.4, respectively; p = 0.01). Bioinformatics analysis of The Cancer Genome Atlas (TCGA) melanoma mutation dataset also revealed a frequency of mutations in the 41 epigenetic genes comparable to that found within our cohort of patient melanoma samples. CONCLUSIONS: Our study identified a high prevalence of somatic mutations in genes encoding epigenetic regulators, including those involved in DNA demethylation, histone modification, chromatin remodeling, and microRNA processing. Moreover, UVB-signature mutations were found more commonly among epigenetic genes than in non-epigenetic genes. Taken together, these findings further implicate epigenetic mechanisms, particularly those involving the chromatin-remodeling enzyme MECOM/EVI1 and histone-modifying enzyme MLL2, in the pathobiology of melanoma.
  
  
  
  
**MECOM**
-
4
of
4
  

### Expression of the zinc finger gene EVI-1 in ovarian and other cancers.

4. Br J Cancer. 1996 Nov;74(10):1518-25. doi: 10.1038/bjc.1996.583
  
  
Brooks DJ(1), Woodward S, Thompson FH, Dos Santos B, Russell M, Yang JM, Guan XY, Trent J, Alberts D ...
  
  
Author information: (1)Department of Medicine, University of Arizona and Arizona Cancer Center, Tucs ...
  
  
The EVI-1 gene was originally detected as an ectopic viral insertion site and encodes a nuclear zinc finger DNA-binding protein. Previous studies showed restricted EVI-1 RNA or protein expression during ontogeny; in a kidney and an endometrial carcinoma cell line; and in normal murine oocytes and kidney cells. EVI-1 expression was also detected in a subset of acute myeloid leukaemias (AMLs) and myelodysplasia. Because EVI-1 is expressed in the urogenital tract during development, we examined ovarian cancers and normal ovaries for EVI-1 RNA expression using reverse transcription polymerase chain reaction (RT-PCR) and RNAase protection. Chromosome abnormalities were examined using karyotypes and whole chromosome 3 and 3q26 fluorescence in situ hybridisation (FISH). RNA from six primary ovarian tumours, five normal ovaries and 47 tumour cell lines (25 ovarian, seven melanoma, three prostate, seven breast and one each of bladder, endometrial, lung, epidermoid and histiocytic lymphoma) was studied. Five of six primary ovarian tumours, three of five normal ovaries and 22 of 25 ovarian cell lines expressed EVI-1 RNA. A variety of other non-haematological cancers also expressed EVI-1 RNA. Immunostaining of ovarian cancer cell lines revealed nuclear EVI-1 protein. In contrast, normal ovary stained primarily within oocytes and faintly in stroma. Primary ovarian tumours showed nuclear and intense, diffuse cytoplasmic staining. Quantitation of EVI-1 RNA, performed using RNAase protection, showed ovarian carcinoma cells expressed 0 to 40 times the EVI-1 RNA in normal ovary, and 0-6 times the levels in leukaemia cell lines. Southern analyses of ovarian carcinoma cell lines showed no amplification or rearrangements involving EVI-1. In some acute leukaemias, activation of EVI-1 transcription is associated with translocations involving 3q26, the site of the EVI-1 gene. Ovarian carcinoma karyotypes showed one line with quadruplication 3(q24q27), but no other clonal structural rearrangements involving 3q26. However, whole chromsome 3 and 3q26 FISH performed on lines with high EVI-1 expression showed translocations involving chromosome 3q26. EVI-1 is overexpressed in ovarian cancer compared with normal ovaries, suggesting a role for EVI-1 in solid tumour carcinogenesis or progression. Mechanisms underlying EVI-1 overexpression remain unclear, but may include rearrangements involving chromosome 3q26.
  
  
  
  
  

---

  

# MEF2C

**Signature gene in:** B cells, Plasma cells
  
**Differentially expressed in:** no cell type
  
  
**MEF2C**
-
1
of
1
  

### Molecular Features of Cancer-associated Fibroblast Subtypes and their Implication on Cancer Pathogenesis, Prognosis, and Immunotherapy Resistance.

1. Clin Cancer Res. 2021 May 1;27(9):2636-2647. doi: 10.1158/1078-0432.CCR-20-4226 Epub 2021 Feb 23.
  
  
Galbo PM Jr(1)(2), Zang X(3)(4), Zheng D(5)(6).
  
  
Author information: (1)Department of Genetics, Albert Einstein College of Medicine, Bronx, New York. ...
  
  
PURPOSE: Cancer-associated fibroblasts (CAFs) are an important component of the tumor microenvironment, but a systematic investigation of their molecular characteristics and clinical relevance are lacking. Here, we sought to compare CAFs across multiple cancer types to identify critical molecular pathways activated in CAF subtypes, which may contribute to clinical outcome, disease progression, and immunotherapy resistance. EXPERIMENTAL DESIGN: We performed integrated analysis of CAFs from melanoma, head and neck squamous cell carcinoma, and lung cancer, and identified the molecular characteristics that are distinctly active in each CAF subtype. Gene signatures for individual CAF subtypes were identified and used to study the association of subtype abundance with clinical outcome and immunotherapy resistance. RESULTS: We identified six CAF subtypes (pan-CAF) shared across cancer types and uncovered the molecular characteristics and genetic pathways distinguishing them. Interestingly, these CAF subtypes express distinct immunosuppressive factors, such as CXCL12 and CXLC14, and stem cell-promoting factor IL6. In addition, we identified novel transcriptional drivers (MEF2C, TWIST1, NR1H3, RELB, and FOXM1) key to CAF heterogeneity. Furthermore, we showed that CAF subtypes were associated with different clinical outcomes and uncovered key molecular pathways that could activate or suppress cancer progression or were involved in resistance to anti-PD1 or anti-PD-L1 immunotherapy. CONCLUSIONS: Our study identifies the molecular characteristics of CAF subtypes shared across several cancer types, implicates cancer types that may benefit from CAF subtype targeted therapies, and identifies specific CAF subtypes associated with immunotherapy resistance.
  
  
  
  
  

---

  

# MET

**Signature gene in:** Tumor cells
  
**Differentially expressed in:** no cell type
  
  
**MET**
-
1
of
10
  

### Loss of viability and impairment of the cell cycle by combining metabolic modulators in canine and feline melanoma cells.

1. Res Vet Sci. 2025 May 11;191:105691. doi: 10.1016/j.rvsc.2025.105691 Online ahead of print.
  
  
Pan MD(1), Arbe MF(1), Salamone GV(2), Glikin GC(1), Finocchiaro LME(1), Villaverde MS(3).
  
  
Author information: (1)Universidad de Buenos Aires, Facultad de Medicina, Instituto de Oncología Dr. ...
  
  
Despite encouraging advances during the last decade, clinical management of malignant human, canine and feline melanoma continues to be a challenge. Thus, new therapeutic development is required. One of the hallmarks of cancer is metabolic rearrangement, including increased glucose metabolism. This metabolic alteration seems to be involved not only in cell proliferation but also in drug resistance, thus offering potential therapeutic targets. The aim of the present work was to investigate the in vitro effects of a combination of metformin (MET, an antidiabetic drug and OXPHOS inhibitor), 2-deoxyglucose (2DG, an HK inhibitor) and 6-aminonicotinamide (6AN, a G6PDH inhibitor) on two melanoma cell lines, Sc (canine) and Dc (feline) derived from spontaneous tumors. We found that both 2DG and MET treatment significantly decreased the cell viability of both cell lines (p < 0.05) in a concentration-dependent manner, whereas 6AN as monotherapy only significantly affected Sc. In addition, the effect of MET was significantly potentiated (p < 0.05) by the combination with both 2DG and 6AN in both cell lines. MET/2DG and MET/6AN significantly affected the cell cycle and increased the percentage of the subG0 population. These results support further studies to investigate the potential use of these metabolic drugs in a veterinary clinical setting.
  
  
  
  
**MET**
-
2
of
10
  

### Immune checkpoint inhibitor-associated Vogt-Koyanagi-Harada-like syndrome: A descriptive systematic review.

2. J Ophthalmic Inflamm Infect. 2025 May 12;15(1):44. doi: 10.1186/s12348-025-00484-8
  
  
Zhang HA(1)(2), Yuan AT(3), Chiasson N(4), Wu KY(5)(6)(7), Kalevar A(8).
  
  
Author information: (1)Department of Ophthalmology and Vision Sciences, University of Toronto, Toron ...
  
  
TOPIC: Vogt-Koyanagi-Harada (VKH)-like uveitis is uniquely reported with immune checkpoint inhibitors (ICI) and BRAF/MEK inhibitors. This article aims to provide a comprehensive portrait of the comorbidities, ocular presentations, treatments, and visual outcomes of patients with VKH-like uveitis following ICI therapy. CLINICAL RELEVANCE: ICIs are increasingly used in cancer therapy, but poorly understood ocular immune-related adverse events (irAEs) can lead to suspension of treatment and be vision-threatening. METHODS: We conducted a systematic review (PROSPERO #CRD42024558269) according to PRISMA guidelines. MEDLINE, Embase, CENTRAL, and Web of Science were searched for English articles published up to June 28, 2024. All study designs reporting on incident VKH-like uveitis following ICI were included. Risk of Bias was assessed using a tool modified from Murad et al. (2018). RESULTS: Of 865 articles, we included 42 articles (4 observational studies, 28 case reports, 6 case series, 3 letters, and 1 editorial) from 12 countries, comprising 52 patients. The mean age was 60.0 ± 11.9 years, and 32 (61.5%) were females. Thirty-six (69.2%) had melanoma, and most were undergoing treatment with a PD-1 inhibitor alone (n = 33, 63.5%) or in combination with a CTLA-4 inhibitor (n = 10, 19.2%). The mean duration of ICI treatment before VKH-like uveitis symptoms was 22.2 ± 29.6 weeks, and the mean duration of ocular symptoms was 16.7 ± 18.6 weeks, with wide variation. Overall, 43 patients (73.1%) had imaging or exams suggesting bilateral involvement and 21 cases (40.4%) suggesting panuveitis. Only 31 cases (59.6%) MET the acute initial-onset uveitis criteria, and 15 (28.8%) MET the chronic phase criteria. Most (n = 47, 90.4%) required systemic or intravitreal steroids, termination of ICI (n = 31, 59.6%), and experienced full resolution or remission of visual symptoms (n = 43, 82.7%). Most articles (n = 40, 95.2%) were judged to be at medium risk of bias. CONCLUSION: This descriptive systematic review consisted mostly of case reports, but it confirmed that a high proportion of VKH-like uveitis occur with PD-1 inhibitors and melanoma patients. VKH-like uveitis can lead to suspension of treatment. Further collaboration between oncologists and ophthalmologists is needed in the continuum of cancer care.
  
  
  
  
**MET**
-
3
of
10
  

### HGF/c-Met Axis-Targeted Nanotherapy via GSH-Responsive Polymer Platforms Suppresses Uveal Melanoma Metastasis.

3. Adv Healthc Mater. 2025 May 8:e2405056. doi: 10.1002/adhm.202405056 Online ahead of print.
  
  
Tao H(1), Liu H(1), Zhang H(2)(3), Ren H(1), Wen B(1), Zhang J(1), Du J(1), Cai Z(1), Deng Z(1).
  
  
Author information: (1)Department of Ophthalmology, The Third Xiangya Hospital, Central South Univer ...
  
  
Uveal melanoma (UM), a malignant tumor originating within the ocular, characterizes high metastasis and lethality among patients. Cancer stem cells (CSCs) distinguished by the c-Met protein are believed to mediate tumor metastasis in UM. However, the low bioavailability of c-Met inhibitors like Crizotilib (Criz) limits their clinical application. Herein, a GSH-responsive nanoparticle named NP@Oxa/Criz to precisely deliver Criz and Oxaliplatin (Oxa) is synthesized in this study. The dual-action mechanism of NP@Oxa/Criz inhibits the HGF/c-Met axis to prevent the nuclear translocation of β-Catenin, thereby reducing the transcription of metastasis-associated genes and undermining the stemness and metastasis of UM cells. Simultaneously, NP@Oxa/Criz induces immunogenic cell death to boost anti-tumor immunity. In vivo studies demonstrate that NP@Oxa/Criz can accumulate in tumor sites, significantly eradicating the primary UM in the ocular and suppressing the metastasis UM in the liver and peritoneal. The outcomes from this work illuminate the therapeutic mechanisms of NP@Oxa/Criz and provide a precise and potent nanotherapeutic strategy for clinical treatment and research in highly metastatic UM.
  
  
  
  
**MET**
-
4
of
10
  

### Diagnostic Accuracy of Novel Optical Imaging Techniques for Melanoma Detection: A Systematic Review and Meta-Analysis.

4. Int J Dermatol. 2025 May 7. doi: 10.1111/ijd.17828 Online ahead of print.
  
  
Varga NN(1)(2), Gulyás L(1)(2), Meznerics FA(1)(2), Barkovskij-Jakobsen KS(1)(2), Szabó B(2)(3), Heg ...
  
  
Author information: (1)Department of Dermatology, Venereology and Dermatooncology, Faculty of Medici ...
  
  
The incidence of melanoma is increasing worldwide, requiring early detection to improve survival rates. Although dermoscopy is the standard non-invasive tool for diagnosing melanoma, it relies on experience and skill. Advances in optical imaging technologies and artificial intelligence have the potential to improve diagnostic accuracy. Our objective was to compare the diagnostic accuracy of novel non-invasive optical imaging techniques for melanoma detection. A systematic literature search was conducted in three databases (Medline, Embase, and CENTRAL) on November 15, 2023. Inclusion criteria focused on studies comparing the accuracy of optical imaging methods against histopathology. Outcomes consisted of measures of diagnostic accuracy. Random-effects meta-analyses were performed for each method with 95% confidence intervals to summarize all relevant effect sizes. Of the 16,239 records, 141 articles MET the inclusion criteria, of which 138 articles were eligible for the meta-analysis. Reflectance confocal microscopy (RCM) and dermoscopy combined with artificial intelligence (DSC + AI) had the highest sensitivity (0.93), with DSC + AI showing higher specificity (0.77 [0.70-0.83]) than RCM (0.749 [0.7475-0.7504]). Multispectral imaging combined with AI also showed high sensitivity (0.92 [0.82-0.97]) and relatively high specificity (0.80 [0.67-0.89]). Standalone dermoscopy exhibited balanced sensitivity (0.87 [0.84-0.90]) and specificity (0.82 [0.78-0.86]). In melanoma diagnosis, both RCM and DSC + AI can serve as second-step optical evaluation methods for suspicious lesions following initial screening with DSC. By maintaining a strong emphasis on multimodal imaging, healthcare providers could improve early detection and outcomes for patients with melanoma.
  
  
  
  
**MET**
-
5
of
10
  

### Surgical technique, outcome, complications, and recurrence rate for removal of extensive perianal melanomas: 50 treated horses.

5. J Am Vet Med Assoc. 2025 May 7:1-8. doi: 10.2460/javma.24.12.0816 Online ahead of print.
  
  
Haegeman L(1), Foucaud M(1), Joostens Z(1), Declercq J(1), Vinardell T(2), Kadic D(1), Mariën T(1).
  
  
Author information: (1)1Equitom Equine Clinic, Lummen, Belgium. (2)2Equine Care Group, Lummen, Belgi ...
  
  
OBJECTIVE: To describe surgical treatment and outcome for horses with extensive perianal melanomas. ANIMALS: Gray horses treated surgically for extensive perianal melanoma between July 1, 2020, and July 31, 2023, were eligible. Horses without the entire perianal skin covered with coalescing or ulcerating melanomas were excluded. CLINICAL PRESENTATION: 59 horses (30 mares, 27 geldings, and 2 stallions) MET the inclusion criteria. Median age was 16 years (range, 7 to 28 years). Clinical signs included tenesmus (n = 53), weight loss (3), and hind limb lameness (1). Six horses presented with no complaints; in these horses, surgery was performed preventively. Time since melanoma first appeared was < 5 years (n = 21), 5 to 10 years (18), > 10 years (9), or unknown (11). Tails were affected in all horses. Melanoma occurred elsewhere for 58 horses. All horses underwent presurgical thoracic and abdominal ultrasonography. RESULTS: Horses underwent standing sedation combined with epidural anesthesia (n = 51) or general anesthesia (8). Surgery involved circumferential incisions at the junction between the skin and melanomas and mucocutaneous junction of the rectum, blunt and sharp dissection to remove the masses, and rectal reconstruction. Perirectal tumors were removed via separate vertical incisions. Tail amputation was performed for 13 horses. Follow-up data (median, 19 months; range, 6 to 48 months) were available for 50 horses; clinical signs improved for 44 (88%), recurrence was observed for 6 (12%), and complications occurred for 15 (30%). CLINICAL RELEVANCE: Results indicated that this surgical procedure is a viable treatment option for horses with extensive perianal melanoma.
  
  
  
  
**MET**
-
6
of
10
  

### Early Compliance with Lung Cancer Lymph Node Standard 5.8: An Analysis of 2022 and 2023 Commission on Cancer Site Reviews.

6. J Thorac Cardiovasc Surg. 2025 Apr 29:S0022-5223(25)00340-X. doi: 10.1016/j.jtcvs.2025.04.041 Online ahead of print.
  
  
Baskin AS(1), Funk EC(2), Francescatti AB(2), Sinco BR(3), Palis BE(2), Hieken TJ(4), Katz MHG(5), B ...
  
  
Author information: (1)Department of Surgery, University of California, San Francisco, San Francisco ...
  
  
OBJECTIVE: To address variability in nodal staging during curative-intent lung cancer resections, the Commission on Cancer (CoC) implemented Standard 5.8 in 2021, requiring lymph nodes be sampled from ≥3 mediastinal stations and ≥1 hilar station and documented in a synoptic pathology report. We assessed compliance data from recent site reviews to evaluate the early implementation of Standard 5.8 through a federally funded collaborative. METHODS: Hospital compliance rates with Standard 5.8 were extracted from a repository of site reviewers' documentation of CoC site visits performed in 2022 and 2023. Each review included up to 7 randomly selected pathology reports eligible for the standard. For a site to be compliant, 5 of 7 pathology reports reviewed in 2022 must have MET Standard 5.8, which increased to 6 of 7 reports in 2023 and beyond. RESULTS: Overall, 652 site visits occurred in 2022 and 2023. Sites without eligible cases (n=148, 23%) were excluded. Among 504 eligible sites, 272 (54%) were found compliant, and 232 (46%) were found non-compliant. Of non-compliant sites, the median percentage of pathology reports meeting the standard was 29%. From 2022 to 2023, the median percentage of adherent pathology reports increased; however, overall compliance rates dipped in 2023, as the threshold needed for site compliance rose. CONCLUSIONS: With almost half of CoC-accredited sites non-compliant, there is a real opportunity to improve the quality of surgical lymph node evaluations. This aligns with growing efforts to support hospitals and surgeons with quality improvement tools and resources for Standard 5.8.
  
  
  
  
**MET**
-
7
of
10
  

### Impact of Parkinson's disease diagnosis validity on the association with cancer: A systematic review and meta-analysis.

7. Parkinsonism Relat Disord. 2025 Apr 22:107846. doi: 10.1016/j.parkreldis.2025.107846 Online ahead of print.
  
  
Mehdiyeva A(1), Kaasinen V(2), Heervä E(3), Sipilä JOT(4).
  
  
Author information: (1)Department of Neurology, Siun Sote North Karelia Central Hospital, Joensuu, F ...
  
  
BACKGROUND: Meta-analyses have reported lower cancer incidence in patients with Parkinson's disease (PD) compared to the general population but with considerable data heterogeneity. OBJECTIVE: To explore how the validity of the PD diagnoses is related to the association with cancer. METHODS: We conducted a systematic review and meta-analysis in which studies were stratified into groups based on the diagnostic validity of Parkinson's disease. Studies investigating mortality data and those examining cancer risk within certain genetic subgroups of PD were excluded. RESULTS: Thirty-four articles encompassing 533,102 patients with PD from 11 countries MET the inclusion criteria. Stratified analyses revealed no association between PD and overall cancer risk preceding or following the PD diagnosis in studies using validated PD data. Studies utilizing less robust PD identification methods, the majority of which were cohort studies, demonstrated a neutral or decreased cancer risk among PD patients. In the studies with the most rigorous PD validation organ-specific analyses showed an increased risk of cutaneous melanoma but no decreased risk in any type of cancer. The positive association between PD and melanoma was more pronounced in the studies with more robust PD diagnosis validity. CONCLUSIONS: The reported associations between PD and cancer are substantially influenced by the quality of PD data. Future investigations should concentrate on organ-specific cancers, instead of pooling cancers together, and use only PD cohorts with validated diagnosis.
  
  
  
  
**MET**
-
8
of
10
  

### A pattern of local failure after preoperative 5 × 5 Gy in soft tissue sarcomas: A long-term real-world experience.

8. Clin Transl Radiat Oncol. 2025 Apr 9;53:100954. doi: 10.1016/j.ctro.2025.100954 eCollection 2025 Jul.
  
  
Zasadziński K(1), Borkowska A(1)(2), Morysiński T(1)(2), Koseła-Paterczyk H(2), Rutkowski P(2), Spał ...
  
  
Author information: (1)Department of Radiotherapy I, Maria Skłodowska-Curie National Research Instit ...
  
  
INTRODUCTION: Preoperative radiotherapy (RT) is used to improve local control (LC) and facilitate limb-sparing procedures in patients with localized soft tissue sarcomas (STS). While conventional preoperative RT delivers 50-50.4 Gy in 25-28 fractions, alternative hypofractionated regimens are under investigation. A 5x5 Gy regimen has been investigated in STS, but its long-term LC rates appear suboptimal. The aim of this study is to analyze the characteristics of patients with local recurrence (LR) after 5x5 Gy and to identify potential RT-related factors affecting efficacy. METHODS: We retrospectively analyzed patients who received 5x5 Gy and underwent surgery for localized extremity and truncal STS in three clinical trials and institutional records. Patient, tumor, and treatment characteristics were evaluated. We assessed the quality of RT plans and recurrence patterns. RESULTS: Among 174 patients who experienced LR after 5x5 Gy, pleomorphic sarcoma (23 %), myxofibrosarcoma (17.8 %), and malignant peripheral nerve sheath tumor (12 %) were the most common pathologic diagnoses. No LR was observed in patients with myxoid liposarcoma. Almost all analyzed plans MET the quality criteria. Most patients (86.2 %) had in-volume recurrences, suggesting inadequate tumor cell eradication rather than insufficient margins or poor target coverage. Dose equivalence analysis suggested that 5x5 Gy (EQD2 = 37.5 Gy for STS, assuming alpha/beta ratio of 4 Gy) may be insufficient, especially for radioresistant subtypes. CONCLUSIONS: The primary factor contributing to LR after 5x5 Gy appears to be insufficient total dose. Future clinical trials should explore dose escalation beyond 5 Gy per fraction, except in myxoid liposarcoma where 5x5 Gy remains effective.
  
  
  
  
**MET**
-
9
of
10
  

### Changes in Melanocytic Nevi and Melanoma Associated With Pregnancy: A Scoping Review.

9. J Cutan Med Surg. 2025 Apr 20:12034754251335590. doi: 10.1177/12034754251335590 Online ahead of print.
  
  
Block BR(1), Powers CM(1), Hu BD(1), Chang A(1), Lambert R(1), Verma H(1), Rabinowitz G(1), Orloff J ...
  
  
Author information: (1)Department of Dermatology, Icahn School of Medicine at Mount Sinai, New York, ...
  
  
Pregnancy is a transformative period characterized by numerous physiological and hormonal changes, including those affecting the skin. Among these changes, alterations in melanocytic nevi and the potential risk of melanoma are areas of significant interest and ambiguity. This scoping review aims to synthesize existing literature on these topics to provide a comprehensive understanding and identify gaps in knowledge. Following the Preferred Reporting Items for Systematic Reviews and Meta-analyses Extension for Scoping Reviews guidelines, a systematic search was conducted; out of 4824 identified articles, 80 MET the inclusion criteria. While some studies suggest hormonal influences might accelerate melanocyte growth, others argue that perceived growth is due to mechanical stretching of the skin, and still others found no significant size changes. Pigmentary changes in nevi are more consistently reported, though some researchers advocate viewing them with suspicion until proven benign. The risk of developing pregnancy-associated melanoma is contentious, with some studies proposing that hormonal changes and gestational immunosuppression increase susceptibility, but the evidence remains inconclusive. Prognostic outcomes are equally disputed, with conflicting findings on pregnancy-associated melanoma thickness, recurrence rates, and survival outcomes. We also performed a novel semi-quantitative analysis to assess attitudes on the risk and prognosis of pregnancy-associated melanoma in the literature, demonstrating that authors of narrative reviews are more likely to oppose theories of pregnancy as a trigger for melanoma development or progression than researchers who conducted the primary investigations. These inconsistencies in the literature highlight the need for further research with standardized methodologies and consideration of confounding factors.
  
  
  
  
**MET**
-
10
of
10
  

### Application of 3D printing for personalized boluses in radiotherapy: a systematic review.

10. Rep Pract Oncol Radiother. 2025 Mar 21;30(1):100-113. doi: 10.5603/rpor.104014 eCollection 2025.
  
  
Bochyńska A(1)(2), Zawadzka A(1), Kukołowicz P(1), Spałek MJ(2).
  
  
Author information: (1)Medical Physics Department, Maria Skłodowska-Curie National Research Institut ...
  
  
The goal of this study was to evaluate the current literature covering the topic of 3D-printed radiotherapy boluses in the context of fabrication methods, materials, and clinical outcomes. This systematic review followed the PRISMA 2020 guidelines. Data were extracted for authors, publication details, application type, printing technique and materials, study type, radiation type, reported outcomes and implementation difficulties. The search yielded 161 articles, 52 of which MET the inclusion criteria. Publications on 3D printing for customized boluses have increased since 2014, with the most articles from the United States (21%). Most studies (80.8%) focused on manufacturing custom boluses and testing 3D printing materials, whereas 19.2% explored creating molds for boluses. CT scans were the primary method for defining the bolus area (88.6%). The publications included three study types: dosimetric evaluations, evaluations with anthropomorphic phantoms, and clinical case studies. Fused Deposition Modeling (FDM) was the most common printing technique (88.1%), with Polylactic Acid (PLA) being the most frequently used material (57.1%). Challenges included ensuring proper fit, assessing material properties, and managing printing time. The outcomes of this review suggest that 3D printing technology holds significant promise for improving radiotherapy by creating custom-fit boluses. 3D-printed boluses demonstrated notable advantages, such as improved dose distribution, better bolus conformity, and reduced setup times. However, several limitations have been identified, including considerable variability in study designs, making it challenging to draw generalized conclusions. Some studies had small sample sizes or did not clearly report methodological details. Addressing these issues will help to optimize technology's implementation.
  
  
  
  
  

---

  

# MGMT

**Signature gene in:** CD4+ T cells
  
**Differentially expressed in:** no cell type
  
  
**MGMT**
-
1
of
10
  

### Neoadjuvant triplet immune checkpoint blockade in newly diagnosed glioblastoma.

1. Nat Med. 2025 Feb 27. doi: 10.1038/s41591-025-03512-1 Online ahead of print.
  
  
Long GV(1)(2)(3)(4)(5), Shklovskaya E(6)(7), Satgunaseelan L(8)(9), Mao Y(6)(8)(10), da Silva IP(6)( ...
  
  
Author information: (1)Melanoma Institute Australia, University of Sydney, Sydney, New South Wales, ...
  
  
Glioblastoma (GBM) is an aggressive primary adult brain tumor that rapidly recurs after standard-of-care treatments, including surgery, chemotherapy and radiotherapy. While immune checkpoint inhibitor therapies have transformed outcomes in many tumor types, particularly when used neoadjuvantly or as a first-line treatment, including in melanoma brain metastases, they have shown limited efficacy in patients with resected or recurrent GBM. The lack of efficacy has been attributed to the scarcity of tumor-infiltrating lymphocytes (TILs), an immunosuppressive tumor microenvironment and low tumor mutation burden typical of GBM tumors, plus exclusion of large molecules from the brain parenchyma. We hypothesized that upfront neoadjuvant combination immunotherapy, administered with disease in situ, could induce a stronger immune response than treatment given after resection or after recurrence. Here, we present a case of newly diagnosed IDH-wild-type, MGMT promoter unmethylated GBM, treated with a single dose of neoadjuvant triplet immunotherapy (anti-programmed cell death protein 1 plus anti-cytotoxic T-lymphocyte protein 4 plus anti-lymphocyte-activation gene 3) followed by maximal safe resection 12 days later. The anti-programmed cell death protein 1 drug was bound to TILs in the resected GBM and there was marked TIL infiltration and activation compared with the baseline biopsy. After 17 months, there is no definitive sign of recurrence. If used first line, before safe maximal resection, checkpoint inhibitors are capable of immune activation in GBM and may induce a response. A clinical trial of first-line neoadjuvant combination checkpoint inhibitor therapy in newly diagnosed GBM is planned (GIANT; trial registration no. NCT06816927 ).
  
  
  
  
**MGMT**
-
2
of
10
  

### Temozolomide-Promoted MGMT Transcription Contributes to Chemoresistance by Activating the ERK Signalling Pathway in Malignant Melanoma.

2. J Cell Mol Med. 2025 Feb;29(3):e70380. doi: 10.1111/jcmm.70380
  
  
Deng M(1)(2)(3), Ren B(4)(5), Zhao J(6), Guo X(7), Yang Y(4), Shi H(1)(2), Bian X(4), Wu M(4), Xu C( ...
  
  
Author information: (1)Department of Oncology, The Fourth Affiliated Hospital of Soochow University, ...
  
  
Tumour cells possess a multitude of chemoresistance mechanisms, which could plausibly contribute to the ineffectiveness of chemotherapy. O6-methylguanine-DNA methyltransferase (MGMT) is an important effector protein associated with Temozolomide (TMZ) resistance in various tumours. To some extent, the expression level of MGMT determines the sensitivity of cells to TMZ, but the mechanism of its expression regulation has not been fully elucidated. Cultured malignant melanoma cell lines A375 and Sk-MEL28 were employed. A luciferase assay was used to detect the transcriptional activity of the MGMT promoter. Western blotting was used to compare the expression levels of phosphorylated ERK1/2 (P-ERK1/2) after TMZ treatment. Immunofluorescent staining was used to detect TMZ-induced DNA damage protein levels. The sensitivity of melanoma cells to TMZ was detected by MTT assay and animal experiments. The expression of MGMT mRNA was tested by Quantitative real-time PCR (RT-qPCR). Flow cytometry was used to measure the apoptosis of TMZ-treated cells. TMZ enhanced the transcription of MGMT through activating the ERK pathway. ERK inhibitors U0126 and vemurafenib (vMF) inhibited the TMZ induced transcription of MGMT. The expression of MGMT and p-ERK1/2 was closely related in human MM tissues. vMF increased the sensitivity of melanoma (MM) to TMZ in vitro and in vivo through downregulating MGMT and promoting the TMZ induced DNA damage in MM. TMZ-promoted MGMT transcription contributed to instinctive chemoresistance by activating the ERK signalling pathway in malignant melanoma. Our study indicates that the use of the ERK inhibitor in combination with TMZ could potentially enhance the effectiveness of clinical treatment for malignant melanoma.
  
  
  
  
**MGMT**
-
3
of
10
  

### Protocatechuic aldehyde sensitizes BRAF-mutant melanoma cells to temozolomide through inducing FANCD2 degradation.

3. Med Oncol. 2025 Jan 18;42(2):48. doi: 10.1007/s12032-025-02601-y
  
  
Yang J(1)(2)(3), Zeng X(1)(2)(3), Pei J(1)(2)(4), Su Z(1)(2)(3), Liu Q(1)(2)(4), Zhang Y(5), Yang Y( ...
  
  
Author information: (1)Engineering Research Center of Sichuan-Tibet Traditional Medicinal Plant, Che ...
  
  
Temozolomide (TMZ)-based chemotherapy is a primary regimen for melanoma patients who have failed targeted therapy or immunotherapy. However, the low response rate of TMZ-based chemotherapy challenges the patients' prognosis. BRAFV600E mutation is the most frequently mutated site in melanoma. This study investigates the synergistic effect of protocatechuic aldehyde (PA) and temozolomide (TMZ) in killing BRAFV600E mutant melanoma cells and BRAF inhibitor-resistant melanoma cells as well as the underlying molecular mechanisms. We report that PA synergistically promoted TMZ cytotoxicity to both BRAF inhibitor-sensitive and BRAF inhibitor-resistant melanoma cells. Combination of PA and TMZ increased DNA double-strand breaks and elevated apoptosis. Mechanism study reveals that PA promoted TMZ cytotoxicity through inducing FANCD2 degradation. Our results suggest that PA is a potential compound for melanoma combinational chemotherapy, regardless of O-6-methylguanine-DNA methyltransferase (MGMT) status.
  
  
  
  
**MGMT**
-
4
of
10
  

### Gliosarcoma: A Multi-Institutional Analysis on Clinical Outcomes and Prognostic Factors.

4. Cancer Med. 2024 Nov;13(22):e70347. doi: 10.1002/cam4.70347
  
  
Roohani S(1)(2)(3), Mirwald M(1), Ehret F(1)(3), Fink C(4)(5)(6), König L(4)(5)(6), Striefler JK(7), ...
  
  
Author information: (1)Department of Radiation Oncology, Charité-Universitätsmedizin Berlin, Corpora ...
  
  
PURPOSE: This study describes oncological outcomes and investigates prognostic factors for patients with gliosarcomas (GSM). METHODS: Histopathologically confirmed GSM patients who underwent treatment at five European institutions were retrospectively analyzed. RESULTS: We analyzed 170 patients with a median clinical follow-up time of 9.2 months. The majority received surgery (94.1%), postoperative radiotherapy (pRT, 81.8%), and temozolomide (TMZ)-based postoperative chemotherapy (66.5%). The median overall survival (OS) and progression-free survival (PFS) were 12.3 and 6.6 months, respectively. In the multivariable Cox regression analysis (MVA), the following factors were significantly associated with OS: age per year (hazard ratio (HR): 1.03, p < 0.001), subtotal resection (STR) versus biopsy only (HR: 0.15, p = 0.018), gross total resection (GTR) versus biopsy only (HR: 0.13, p = 0.011), pRT versus no pRT (HR: 0.20, p < 0.001), postoperative TMZ-based chemotherapy versus no postoperative chemotherapy (HR: 0.44, p = 0.003), MGMT promoter non-methylated versus methylated (HR: 1.79, p = 0.05), and tumor diameter per cm (HR: 1.15, p = 0.046). For PFS, the following factors were significantly associated in the MVA: GTR versus biopsy only (HR: 0.19, p = 0.026), pRT versus no pRT (HR: 0.36, p = 0.006), postoperative TMZ-based chemotherapy vs. no postoperative chemotherapy (HR: 0.39, p < 0.001), MGMT promoter status unknown versus methylated (HR: 1.69, p = 0.034), and tumor diameter per cm (HR: 1.18, p = 0.016). Sex, primary or secondary GSM, and TP53 mutational status were not significantly associated with OS or PFS. CONCLUSIONS: Trimodal therapy comprising surgical resection, pRT and TMZ-based chemotherapy appears to have the most beneficial effect on survival in GSM patients. Smaller tumor size, younger age and methylated MGMT promoters are associated with improved survival. To our knowledge, this is the largest multi-institutional cohort study investigating outcomes and prognostic factors for GSM.
  
  
  
  
**MGMT**
-
5
of
10
  

### Prognostic Impact of TERT Promoter Mutations in Adult-Type Diffuse Gliomas Based on WHO2021 Criteria.

5. Cancers (Basel). 2024 May 27;16(11):2032. doi: 10.3390/cancers16112032
  
  
Lee Y(1), Park CK(2), Park SH(3)(4).
  
  
Author information: (1)Department of Hospital Pathology, St. Vincent's Hospital, The Catholic Univer ...
  
  
Mutation in the telomerase reverse transcriptase promoter (TERTp )is commonly observed in various malignancies, such as central nervous system (CNS) tumors, malignant melanoma, bladder cancer, and thyroid carcinoma. These mutations are recognized as significant poor prognostic factors for these tumors. In this investigation, a total of 528 cases of adult-type diffuse gliomas diagnosed at a single institution were reclassified according to the 2021 WHO classifications of CNS tumors, 5th edition (WHO2021). The study analyzed clinicopathological and genetic features, including TERTp mutations in each tumor. The impact of known prognostic factors on patient outcomes was analyzed through Kaplan-Meier survival and Cox regression analysis. TERTp mutations were predominantly identified in 94.1% of oligodendrogliomas (ODG), followed by 66.3% in glioblastoma, IDH-wildtype (GBM-IDHwt), and 9.2% of astrocytomas, IDH-mutant (A-IDHm). When considering A-IDHm and GBM as astrocytic tumors (Group 1) and ODGs (Group 2), TERTp mutations emerged as a significant adverse prognostic factor (p = 0.013) in Group 1. However, within each GBM-IDHwt and A-IDHm, the presence of TERTp mutations did not significantly impact patient prognosis (p = 0.215 and 0.268, respectively). Due to the high frequency of TERTp mutations in Group 2 (ODG) and their consistent prolonged survival, a statistical analysis to evaluate their impact on overall survival was deemed impractical. When considering MGMTp status, the combined TERTp-mutated and MGMTp-unmethylated group exhibited the worst prognosis in OS (p = 0.018) and PFS (p = 0.034) of GBM. This study confirmed that the classification of tumors according to the WHO2021 criteria effectively reflected prognosis. Both uni- and multivariate analyses in GBM, age, MGMTp methylation, and CDKN2A/B homozygous deletion were statistically significant prognostic factors while in univariate analysis in A-IDHm, grade 4, the Ki-67 index and MYCN amplifications were statistically significant prognostic factors. This study suggests that it is important to classify and manage tumors based on their genetic characteristics in adult-type diffuse gliomas.
  
  
  
  
**MGMT**
-
6
of
10
  

### Revisiting Temozolomide's role in solid tumors: Old is gold?

6. J Cancer. 2024 Apr 22;15(11):3254-3271. doi: 10.7150/jca.94109 eCollection 2024.
  
  
Matthaios D(1), Balgkouranidou I(2), Neanidis K(3), Sofis A(4), Pikouli A(5), Romanidis K(6), Pappa ...
  
  
Author information: (1)Department of Oncology, General Hospital of Rhodes, Rhodes, Greece. (2)Depart ...
  
  
Temozolomide is an imidazotetrazine with a long history in oncology especially for the high grade malignant glioma and metastatic melanoma. However, last year's new indications for its use are added. Its optimum pharmacodynamic profile, its ability to penetrate the blood-brain barrier, the existence of methylation of MGMT in solid tumors which enhances its efficacy, the identification of new agents that can overcome temozolomide's resistance, the promising role of temozolomide in turning immune cold tumors to hot ones, are leading to expand its use in other solid tumors, giving oncologists an additional tool for the treatment of advanced and aggressive neoplasms.
  
  
  
  
**MGMT**
-
7
of
10
  

### MGMT inhibition regulates radioresponse in GBM, GSC, and melanoma.

7. Sci Rep. 2024 May 29;14(1):12363. doi: 10.1038/s41598-024-61240-x
  
  
Yun HS(1), Kramp TR(1), Palanichamy K(2), Tofilon PJ(1), Camphausen K(3).
  
  
Author information: (1)Radiation Oncology Branch, National Cancer Institute, 10 Center Drive, 9000 R ...
  
  
Radiotherapy is the standard treatment for glioblastoma (GBM), but the overall survival rate for radiotherapy treated GBM patients is poor. The use of adjuvant and concomitant temozolomide (TMZ) improves the outcome; however, the effectiveness of this treatment varies according to MGMT levels. Herein, we evaluated whether MGMT expression affected the radioresponse of human GBM, GBM stem-like cells (GSCs), and melanoma. Our results indicated a correlation between MGMT promoter methylation status and MGMT expression. MGMT-producing cell lines ACPK1, GBMJ1, A375, and MM415 displayed enhanced radiosensitivity when MGMT was silenced using siRNA or when inhibited by lomeguatrib, whereas the OSU61, NSC11, WM852, and WM266-4 cell lines, which do not normally produce MGMT, displayed reduced radiosensitivity when MGMT was overexpressed. Mechanistically lomeguatrib prolonged radiation-induced γH2AX retention in MGMT-producing cells without specific cell cycle changes, suggesting that lomeguatrib-induced radiosensitization in these cells is due to radiation-induced DNA double-stranded break (DSB) repair inhibition. The DNA-DSB repair inhibition resulted in cell death via mitotic catastrophe in MGMT-producing cells. Overall, our results demonstrate that MGMT expression regulates radioresponse in GBM, GSC, and melanoma, implying a role for MGMT as a target for radiosensitization.
  
  
  
  
**MGMT**
-
8
of
10
  

### Prognostic gene biomarkers for c-Src inhibitor Si162 sensitivity in melanoma cells.

8. Turk J Biol. 2023 Nov 6;48(1):13-23. doi: 10.55730/1300-0152.2678 eCollection 2024.
  
  
Türk S(1), Yilmaz A(2), Malkan ÜY(3), Uçar G(1), Türk C(2).
  
  
Author information: (1)Department of Biochemistry, Faculty of Pharmacy, Hacettepe University, Ankara ...
  
  
BACKGROUND/AIM: Early detection and treatment are crucial in combating malignant melanoma. Src is an important therapeutic target in melanoma due to its association with cancer progression. However, developing effective Src-targeting drugs remains challenging and personalized medicine relies on biomarkers and targeted therapies for precise and effective treatment. This study focuses on Si162, a newly synthesized c-Src inhibitor, to identify reliable biomarkers for predicting Si162 sensitivity and explore associated biological characteristics and pathways in melanoma cells. MATERIALS AND METHODS: Primary melanoma cells (M1, M21, M24, M84, M133, M307, and M2025) were obtained from patients diagnosed with melanoma. Si162 cytotoxicity tests were performed using luminescent adenosine triphosphate detection and the half-maximal inhibitory concentration (IC50) values were calculated. Gene expression profiles were analyzed using microarray-based gene expression data. Differentially expressed genes between the resistant and sensitive groups were identified using Pearson correlation analysis. Gene coexpression, interactions, and pathways were investigated through clustering, network, and pathway analyses. Biological functions were examined using the Database for Annotation, Visualization, and Integrated Discovery. Molecular pathways associated with different responses to Si162 were identified using gene set enrichment analysis. The gene expressions were validated using reverse transcription-quantitative polymerase chain reaction. RESULTS: The cells revealed significant differences in response to Si162 based on the IC50 values (p < 0.05). A total of 36 differentially expressed genes associated with Si162 susceptibility were identified. Distinct expression patterns between the sensitive and resistant groups were observed in 9 genes (LRBA, MGMT, CAND1, ADD1, SETD2, CNTN6, FGF18, C18orf25, and RPL13). Coexpression among the differentially expressed genes was highlighted, and 9 genes associated with molecular pathways, including EMT, transforming growth factor-beta (TGF-β) signaling, and ribosomal protein synthesis, between groups. Genes involved in dysregulated immune response were observed in the resistant group. The involvement of 5 genes (ADD1, CNTN6, FGF18, C18orf25, and RPL13) in Si162 resistance was confirmed through qRT-PCR validation. CONCLUSION: These findings contribute to our understanding of the underlying biological differences among melanoma cells and suggest potential biomarkers and pathways associated with Si162 response and resistance.
  
  
  
  
**MGMT**
-
9
of
10
  

### The Impact of O6-Methylguanine-DNA Methyltransferase (MGMT) Promoter Methylation on the Outcomes of Patients with Leiomyosarcoma Treated with Dacarbazine.

9. Cells. 2023 Jun 15;12(12):1635. doi: 10.3390/cells12121635
  
  
Cannella L(1), Della Monica R(2), Marretta AL(3), Iervolino D(4), Vincenzi B(5), De Chiara AR(6), Cl ...
  
  
Author information: (1)S.C. Sarcomi e Tumori Rari, Istituto Nazionale Tumori-IRCCS-Fondazione "G. Pa ...
  
  
Dacarbazine is an important drug in the therapeutic landscape of leiomyosarcoma (LMS). Alkylating agents are subjected to resistance mechanisms based on anti-apoptotic pathways and repair mechanisms, including the DNA repair enzyme O6-methylguanine-DNA methyltransferase (MGMT). In this retrospective study, the methylation status of the MGMT promoter in histological tumor samples from patients with LMS, dacarbazine-based regimens-treated, was measured and correlated with clinical outcomes aimed at optimizing the use of dacarbazine in soft tissue sarcomas. The patients with unmethylated MGMT had better outcomes than those with methylated MGMT. Patients without MGMT methylation had better Progression Free Survival (PFS) when aged ≥62 years compared to those aged <62 years, while PFS of patients with methylated MGMT was less favorable independently of age (p = 0.0054). The patients without a methylated MGMT gene had higher Disease control rate (DCR). These results are not in agreement with the role of the methylated MGMT gene in other tumors, and with this study, we demonstrated the correlation between methylated MGMT and poor prognosis; despite that, sample smallness, heterogeneity of LMS and of treatment history could be selection bias. Predictive markers of response to chemotherapies in sarcomas remain an unmet need.
  
  
  
  
**MGMT**
-
10
of
10
  

### Protocatechuic aldehyde acts synergistically with dacarbazine to augment DNA double-strand breaks and promote apoptosis in cutaneous melanoma cells.

10. BMC Complement Med Ther. 2023 Apr 6;23(1):111. doi: 10.1186/s12906-023-03933-w
  
  
Pei J(#)(1)(2)(3), Su Z(#)(1)(2)(4), Zeng X(#)(1)(2)(4), Zhong Y(1)(2)(3), Zhang Y(5), Yang Y(1)(2)( ...
  
  
Author information: (1)Engineering Research Center of Sichuan-Tibet Traditional Medicinal Plant, Che ...
  
  
Erratum in BMC Complement Med Ther. 2023 Apr 27;23(1):133. doi: 10.1186/s12906-023-03965-2.
  
  
  
  
  

---

  

# NCOA3

**Signature gene in:** B cells
  
**Differentially expressed in:** no cell type
  
  
**NCOA3**
-
1
of
10
  

### The Molecular Evolution of Melanoma Distant Metastases.

1. J Invest Dermatol. 2024 Nov;144(11):2530-2540.e1. doi: 10.1016/j.jid.2024.03.029 Epub 2024 Apr 4.
  
  
Bezrookove V(1), Kianian S(1), McGeever L(2), Jones R(2), Caressi C(1), Nosrati M(1), Kim KB(1), Leo ...
  
  
Author information: (1)Center for Melanoma Research and Treatment, California Pacific Medical Center ...
  
  
The evolution of primary melanoma to lymph node and distant metastasis is incompletely understood. We examined the genomic diversity in melanoma progression in matched primary melanomas and lymph node and distant metastases from 17 patients. FISH analysis revealed cancer cell fractions with monotonic copy number alterations, including PHIP gain and PTEN loss, in the metastatic cascade. By contrast, the cancer cell fraction with copy number alterations for BPTF and MITF was reduced in lymph node metastases but increased in distant metastases. Separately, the cancer cell fraction with NCOA3 copy number alteration was comparable between primary tumors and lymph node metastases yet increased in distant metastases. These results suggest enrichment of the phosphoinositide 3-kinase and MITF pathways in the transition through the metastatic cascade. By contrast, next-generation sequencing analysis did not identify a consistent pattern of changes in variant allele frequency while revealing several intriguing findings, including decreased variant allele frequency in distant metastases and distinct drivers in lymph node versus distant metastases. These results provide evidence that distant melanoma metastasis does not always emanate from lymph node metastasis. These results enhance our understanding of clonal patterns of melanoma metastasis, with possible implications for targeted therapy and metastasis competency.
  
  
  
  
**NCOA3**
-
2
of
10
  

### Identifying survival of pan-cancer patients under immunotherapy using genomic mutation signature with large sample cohorts.

2. J Mol Med (Berl). 2024 Jan;102(1):69-79. doi: 10.1007/s00109-023-02398-1 Epub 2023 Nov 18.
  
  
Zhang L(#)(1), Wang Y(#)(2), Wang L(1), Wang M(1), Li S(1), He J(1), Ji J(1), Li K(3), Cao L(4).
  
  
Author information: (1)Department of Epidemiology and Biostatistics, Public Health College, Harbin M ...
  
  
Although immune checkpoint inhibitors have led to durable clinical response in multiple cancers, only a small proportion of patients respond to this treatment. Therefore, we aim to develop a predictive model that utilizes gene mutation profiles to accurately identify the survival of pan-cancer patients with immunotherapy. Here, we develop and evaluate three different nomograms using two cohorts containing 1,594 cancer patients whose mutation profiles are obtained by MSK-IMPACT sequencing and 230 cancer patients receiving whole-exome sequencing, respectively. Using eighteen genes (SETD2, BRAF, NCOA3, LATS1, IL7R, CREBBP, TET1, EPHA7, KDM5C, MET, KMT2D, RET, PAK7, CSF1R, JAK2, FAT1, ASXL1 and SPEN), the first nomogram stratifies patients from both cohorts into High-Risk and Low-Risk groups. Pan-cancer patients in the High-Risk group exhibit significantly shorter overall survival and progression-free survival than patients in the Low-Risk group in both cohorts. Meanwhile, the first nomogram also accurately identifies the survival of patients with melanoma or lung cancer undergoing immunotherapy, or pan-cancer patients treated with anti-PD-1/PD-L1 inhibitor or anti-CTLA-4 inhibitor. The model proposed is not a prognostic model for the survival of pan-cancer patients without immunotherapy, but a simple, effective and robust predictive model for pan-cancer patients' survival under immunotherapy, and could provide valuable assistance for clinical practice.
  
  
  
  
**NCOA3**
-
3
of
10
  

### Patients deriving long-term benefit from immune checkpoint inhibitors demonstrate conserved patterns of site-specific mutations.

3. Sci Rep. 2022 Jul 7;12(1):11490. doi: 10.1038/s41598-022-15714-5
  
  
Principe DR(1).
  
  
Author information: (1)University of Illinois College of Medicine, 840 South Wood Street, 601 CSB, C ...
  
  
Immune checkpoint inhibitors (ICIs) have revolutionized cancer therapy and are now the preferred treatment for several tumor types. Though ICIs have shown remarkable efficacy in several cancer histologies, in many cases providing long-term disease control, not all patients will derive clinical benefit from such approaches. Given the lack of a reliable predictive biomarker for therapeutic responses to ICIs, we conducted a retrospective analysis of publicly available genomic data from a large pan-cancer cohort of patients receiving ICI-based immunotherapy. Consistent with previous results, patients in the combined cohort deriving a long-term survival benefit from ICIs were more likely to have a higher tumor mutational burden (TMB). However, this was not uniform across tumor-types, failing to predict for long-term survivorship in most non-melanoma cancers. Interestingly, long-term survivors in most cancers had conserved patterns of mutations affecting several genes. In melanoma, this included mutations affecting TET1 or PTPRD. In patients with colorectal cancer, mutations affecting TET1, RNF43, NCOA3, LATS1, NOTCH3, or CREBBP were also associated with improved prognosis, as were mutations affecting PTPRD, EPHA7, NTRK3, or ZFHX3 in non-small cell lung cancer, RNF43, LATS1, or CREBBP mutations in bladder cancer, and VHL mutations in renal cell carcinoma patients. Thus, this study identified several genes that may have utility as predictive biomarkers for therapeutic responses in patients receiving ICIs. As many have no known relationship to immunotherapy or ICIs, these genes warrant continued exploration, particularly for cancers in which established biomarkers such as PD-L1 expression or TMB have little predictive value.
  
  
  
  
**NCOA3**
-
4
of
10
  

### NCOA3, a new player in melanoma susceptibility and a therapeutic target.

4. Cancer Gene Ther. 2022 May;29(5):399-401. doi: 10.1038/s41417-022-00449-2 Epub 2022 Mar 23.
  
  
Bezrookove V(1), Kashani-Sabet M(2).
  
  
Author information: (1)Center for Melanoma Research and Treatment, California Pacific Medical Center ...
  
  
DOI: 10.1038/s41417-022-00449-2 PMCID: PMC9117460 PMID: 35322161 [Indexed for MEDLINE]
  
  
  
  
**NCOA3**
-
5
of
10
  

### Spatial transcriptomics using combinatorial fluorescence spectral and lifetime encoding, imaging and analysis.

5. Nat Commun. 2022 Jan 10;13(1):169. doi: 10.1038/s41467-021-27798-0
  
  
Vu T(#)(1)(2), Vallmitjana A(#)(1)(3), Gu J(#)(2)(4), La K(1), Xu Q(1), Flores J(2)(5), Zimak J(6), ...
  
  
Author information: (1)Department of Biomedical Engineering, University of California, Irvine, Irvin ...
  
  
Multiplexed mRNA profiling in the spatial context provides new information enabling basic research and clinical applications. Unfortunately, existing spatial transcriptomics methods are limited due to either low multiplexing or complexity. Here, we introduce a spatialomics technology, termed Multi Omic Single-scan Assay with Integrated Combinatorial Analysis (MOSAICA), that integrates in situ labeling of mRNA and protein markers in cells or tissues with combinatorial fluorescence spectral and lifetime encoded probes, spectral and time-resolved fluorescence imaging, and machine learning-based decoding. We demonstrate MOSAICA's multiplexing scalability in detecting 10-plex targets in fixed colorectal cancer cells using combinatorial labeling of five fluorophores with facile error-detection and removal of autofluorescence. MOSAICA's analysis is strongly correlated with sequencing data (Pearson's r = 0.96) and was further benchmarked using RNAscopeTM and LGC StellarisTM. We further apply MOSAICA for multiplexed analysis of clinical melanoma Formalin-Fixed Paraffin-Embedded (FFPE) tissues. We finally demonstrate simultaneous co-detection of protein and mRNA in cancer cells.
  
  
  
  
**NCOA3**
-
6
of
10
  

### Nuclear Receptor Coactivator NCOA3 Regulates UV Radiation-Induced DNA Damage and Melanoma Susceptibility.

6. Cancer Res. 2021 Jun 1;81(11):2956-2969. doi: 10.1158/0008-5472.CAN-20-3450 Epub 2021 Mar 25.
  
  
de Semir D(1)(2), Bezrookove V(1)(2), Nosrati M(1)(2), Dar AA(1)(2), Miller JR 3rd(1)(2), Leong SP(1 ...
  
  
Author information: (1)Center for Melanoma Research and Treatment, California Pacific Medical Center ...
  
  
Melanoma occurs as a consequence of inherited susceptibility to the disease and exposure to UV radiation (UVR) and is characterized by uncontrolled cellular proliferation and a high mutational load. The precise mechanisms by which UVR contributes to the development of melanoma remain poorly understood. Here we show that activation of nuclear receptor coactivator 3 (NCOA3) promotes melanomagenesis through regulation of UVR sensitivity, cell-cycle progression, and circumvention of the DNA damage response (DDR). Downregulation of NCOA3 expression, either by genetic silencing or small-molecule inhibition, significantly suppressed melanoma proliferation in melanoma cell lines and patient-derived xenografts. NCOA3 silencing suppressed expression of xeroderma pigmentosum C and increased melanoma cell sensitivity to UVR. Suppression of NCOA3 expression led to activation of DDR effectors and reduced expression of cyclin B1, resulting in G2-M arrest and mitotic catastrophe. A SNP in NCOA3 (T960T) reduced NCOA3 protein expression and was associated with decreased melanoma risk, given a significantly lower prevalence in a familial melanoma cohort than in a control cohort without cancer. Overexpression of wild-type NCOA3 promoted melanocyte survival following UVR and was accompanied by increased levels of UVR-induced DNA damage, both of which were attenuated by overexpression of NCOA3 (T960T). These results describe NCOA3-regulated pathways by which melanoma can develop, with germline NCOA3 polymorphisms enabling enhanced melanocyte survival in the setting of UVR exposure, despite an increased mutational burden. They also identify NCOA3 as a novel therapeutic target for melanoma. SIGNIFICANCE: This study explores NCOA3 as a regulator of the DDR and a therapeutic target in melanoma, where activation of NCOA3 contributes to melanoma development following exposure to ultraviolet light.
  
  
  
  
**NCOA3**
-
7
of
10
  

### SRC-3 Functions as a Coactivator of T-bet by Regulating the Maturation and Antitumor Activity of Natural Killer Cells.

7. Cancer Immunol Res. 2020 Sep;8(9):1150-1162. doi: 10.1158/2326-6066.CIR-20-0181 Epub 2020 Jun 19.
  
  
Hu M(1), Lu Y(1), Qi Y(1), Zhang Z(1), Wang S(1), Xu Y(1), Chen F(1), Tang Y(1), Chen S(1), Chen M(1 ...
  
  
Author information: (1)State Key Laboratory of Trauma, Burns and Combined Injury, Institute of Combi ...
  
  
Natural killer (NK)-cell development and maturation is a well-organized process. The steroid receptor coactivator 3 (SRC-3) is a regulator of the hematopoietic and immune systems; however, its role in NK cells is poorly understood. Here, SRC-3 displayed increased nuclear translocation in NK cells during terminal differentiation and upon inflammatory cytokine stimulation. Targeted deletion of SRC-3 altered normal NK-cell distribution and compromised NK-cell maturation. SRC-3 deficiency led to significantly impaired NK-cell functions, especially their antitumor activity. The expression of several critical T-bet target genes, including Zeb2, Prdm1, and S1pr5, but not T-bet itself, was markedly decreased in NK cells in the absence of SRC-3. There was a physiologic interaction between SRC-3 and T-bet proteins, where SRC-3 was recruited by T-bet to regulate the transcription of the aforementioned genes. Collectively, our findings unmask a previously unrecognized role of SRC-3 as a coactivator of T-bet in NK-cell biology and indicate that targeting SRC-3 may be a promising strategy to increase the tumor surveillance function of NK cells.
  
  
  
  
**NCOA3**
-
8
of
10
  

### Prospective Validation of Molecular Prognostic Markers in Cutaneous Melanoma: A Correlative Analysis of E1690.

8. Clin Cancer Res. 2017 Nov 15;23(22):6888-6892. doi: 10.1158/1078-0432.CCR-17-1317 Epub 2017 Aug 8.
  
  
Kashani-Sabet M(1), Nosrati M(2), Miller JR 3rd(2), Sagebiel RW(2), Leong SPL(2), Lesniak A(3), Tong ...
  
  
Author information: (1)Center for Melanoma Research and Treatment, California Pacific Medical Center ...
  
  
Purpose: To validate the prognostic impact of combined expression levels of three markers (SPP1, RGS1, and NCOA3) in melanoma specimens from patients enrolled in the E1690 clinical trial of high-dose or low-dose IFNα-2b versus observation.Experimental Design: Tissue was available from 248 patients. Marker expression was determined by digital imaging of immunohistochemically stained slides. The prognostic impact of each marker was first assessed by recording its expression value relative to the median. A multimarker index was then developed to combine marker expression levels by counting for each patient the number of markers with high expression. The impact of the multimarker index on relapse-free survival (RFS) and overall survival (OS) was assessed using Kaplan-Meier analysis, and both univariate and multivariate Cox regression analyses.Results: By Kaplan-Meier analysis, high multimarker expression scores were significantly predictive of RFS (P < 0.001) and OS (P < 0.001). Stepwise multivariate Cox regression analysis with backward elimination that included routine clinical and histologic prognostic factors revealed high multimarker expression scores and tumor thickness as the only factors significantly and independently predicting RFS and OS. Stepwise multivariate Cox regression analyses that also included treatment type and number of positive lymph nodes generated identical results for both RFS and OS. In the molecularly defined low-risk subgroup, patients treated with high-dose IFN had a significantly improved RFS compared with patients in the other two subgroups (P < 0.05).Conclusions: These results validate the independent impact of combined expression levels of SPP1, RGS1, and NCOA3 on survival of melanoma in a prospectively collected cohort. Clin Cancer Res; 23(22); 6888-92. ©2017 AACR.
  
  
  
  
**NCOA3**
-
9
of
10
  

### miR-137 Targets p160 Steroid Receptor Coactivators SRC1, SRC2, and SRC3 and Inhibits Cell Proliferation.

9. Mol Endocrinol. 2015 Aug;29(8):1170-83. doi: 10.1210/me.2015-1080 Epub 2015 Jun 12.
  
  
Eedunuri VK(1), Rajapakshe K(1), Fiskus W(1), Geng C(1), Chew SA(1), Foley C(1), Shah SS(1), Shou J( ...
  
  
Author information: (1)Adrienne Helis Malvin Medical Research Foundation (V.K.E.), New Orleans, Loui ...
  
  
The p160 family of steroid receptor coactivators (SRCs) are pleiotropic transcription factor coactivators and "master regulators" of gene expression that promote cancer cell proliferation, survival, metabolism, migration, invasion, and metastasis. Cancers with high p160 SRC expression exhibit poor clinical outcomes and resistance to therapy, highlighting the SRCs as critical oncogenic drivers and, thus, therapeutic targets. microRNAs are important epigenetic regulators of protein expression. To examine the regulation of p160 SRCs by microRNAs, we used and combined 4 prediction algorithms to identify microRNAs that could target SRC1, SRC2, and SRC3 expression. For validation of these predictions, we assessed p160 SRC protein expression and cell viability after transfection of corresponding microRNA mimetics in breast cancer, uveal melanoma, and prostate cancer (PC) cell lines. Transfection of selected microRNA mimetics into breast cancer, uveal melanoma, and PC cells depleted SRC protein expression levels and exerted potent antiproliferative activity in these cell types. In particular, microRNA-137 (miR-137) depleted expression of SRC1, SRC2, and very potently, SRC3. The latter effect can be attributed to the presence of 3 miR-137 recognition sequences within the SRC3 3'-untranslated region. Using reverse phase protein array analysis, we identified a network of proteins, in addition to SRC3, that were modulated by miR-137 in PC cells. We also found that miR-137 and its host gene are epigenetically silenced in human cancer specimens and cell lines. These results support the development and testing of microRNA-based therapies (in particular based on restoring miR-137 levels) for targeting the oncogenic family of p160 SRCs in cancer.
  
  
  
  
**NCOA3**
-
10
of
10
  

### A multimarker prognostic assay for primary cutaneous melanoma.

10. Clin Cancer Res. 2009 Nov 15;15(22):6987-92. doi: 10.1158/1078-0432.CCR-09-1777 Epub 2009 Nov 3.
  
  
Kashani-Sabet M(1), Venna S, Nosrati M, Rangel J, Sucker A, Egberts F, Baehner FL, Simko J, Leong SP ...
  
  
Author information: (1)Auerback Melanoma Research Laboratory and Melanoma Center, Comprehensive Canc ...
  
  
PURPOSE: To determine the prognostic significance of a multimarker assay incorporating expression levels of three molecular markers in primary cutaneous melanoma. EXPERIMENTAL DESIGN: We assessed expression levels of NCOA3, SPP1, and RGS1 using immunohistochemical analysis in a tissue microarray cohort of 395 patients. For each marker, we identified optimal cut-points for expression intensity to predict disease-specific survival (DSS) and, as a secondary endpoint, sentinel lymph node (SLN) status. The cumulative overexpression of all three markers was embodied in a multimarker index, and its prognostic effect on DSS and SLN status was assessed using Cox regression, Kaplan-Meier analysis, and logistic regression. The prognostic effect of this multimarker assay on DSS was assessed in an independent cohort of 141 patients, in which marker expression levels were scored using immunohistochemical analysis of stained tissue sections. RESULTS: Increasing multimarker index scores were significantly predictive of reduced DSS and increased SLN metastasis in the 395-patient cohort. Multivariate logistic regression analysis revealed multimarker expression scores as an independent predictor of SLN status (P = 0.001). Multivariate Cox regression analysis showed the independent effect of the multimarker index on DSS (P < 0.001). The multimarker index was the most significant factor predicting DSS when compared with other clinical and histologic factors, including SLN status (P = 0.002). Multimarker expression scores were also the most significantly predictive of DSS in the independent cohort (P = 0.01). CONCLUSIONS: These results describe a multimarker assay with independent prognostic effect on the prediction of survival associated with melanoma in two distinct cohorts.
  
  
  
  
  

---

  

# NFATC2

**Signature gene in:** Tregs
  
**Differentially expressed in:** no cell type
  
  
**NFATC2**
-
1
of
10
  

### Utility of Protein Kinase C Beta II Immunohistochemistry in Differential Diagnosis of Ewing Sarcoma.

1. Am J Surg Pathol. 2025 Apr 15. doi: 10.1097/PAS.0000000000002400 Online ahead of print.
  
  
Lasota J(1), Krupińska M(2), Kaczorowski M(1)(3), Chłopek M(1)(4), Kinkor Z(5), Švajdler M(5)(6), Pe ...
  
  
Author information: (1)Laboratory of Pathology, National Cancer Institute, Bethesda, MD. (2)Departme ...
  
  
The diagnosis of Ewing sarcoma can be challenging, particularly when the tumor is present in an atypical location and resembles histologic mimics. The hallmark feature of Ewing sarcoma is chromosomal translocation, t(11;22)(q24;q12), involving EWSR1 and ETS gene family members. For decades, fluorescence in situ hybridization with a break-apart EWSR1 probe has been the diagnostic gold standard. However, EWSR1 rearrangements have been identified in other malignancies; thus, the detection of chimeric EWSR1 transcripts has become a preferable approach. Occasionally, insufficient tissue, severe RNA degradation, or economic constraints hamper molecular testing. This study evaluated Protein Kinase C Beta II (PKC β II) expression in >1000 tumors and assessed the utility of PKC β II immunohistochemistry in the differential diagnosis of Ewing sarcoma. Tumors harboring EWSR1::FLI1 (n=26), EWSR1::ERG, EWSR1::ETV4 (n=1), and FUS::ERG (n=6) fusions were evaluated, revealing strong diffuse immunoreactivity, although a patchy pattern was seen in 3 cases. Undifferentiated round cell sarcomas (n=46), including BCOR-, CIC-, NFATC2-, NUTM1-, and PATZ1 rearranged/fusion-sarcomas were negative. Two of the 130 synovial sarcomas, including 1 with a poorly differentiated morphology, showed diffuse, moderate-to-strong positivity. One of the 26 poorly differentiated carcinomas from the head and neck region, probably small cell lung carcinoma metastasis, showed strong PKC β II expression. Neuroblastomas (>50%) expressed PKC β II, although none showed a strong diffuse pattern. Diffuse moderate-to-strong immunoreactivity was observed in 2 sarcomatoid mesotheliomas and 2 metastatic melanomas. Diffuse but weak staining was observed in 73% (11/15) of the T-cell lymphoblastic lymphomas, including 10 CD99-positive cases. Similarly, weak predominantly patchy staining was seen in half (40/80) of other non-Hodgkin lymphomas and sporadically in embryonal rhabdomyosarcoma, Merkel cell carcinoma, small cell lung carcinoma, and Wilms tumor. Thus, diffuse and strong PKC β II immunoreactivity appears to be a reliable diagnostic marker for distinguishing classic Ewing sarcoma from histologic mimics.
  
  
  
  
**NFATC2**
-
2
of
10
  

### NFATC2 target gene signature correlates with immune checkpoint blockade resistance in melanoma.

2. Am J Cancer Res. 2025 Jan 15;15(1):311-321. doi: 10.62347/EYML2689 eCollection 2025.
  
  
Lawal B(1)(2), Wang Y(1)(2), Lotfinejad P(1)(2), Sharma R(1)(2), Yang C(1)(2), Annasamudram A(1)(2), ...
  
  
Author information: (1)UPMC Hillman Cancer Center, University of Pittsburgh Pittsburgh, PA 15213, US ...
  
  
Immune checkpoint inhibitors (ICIs), such as anti-PD-1 and anti-CTLA-4, have significantly advanced melanoma treatment by reactivating the immune system to target cancer cells. However, a substantial portion of patients do not respond or develop resistance, highlighting the need for more effective predictive biomarkers. Dysregulation of transcriptional programs has been implicated in cancer progression and immune evasion, with transcription factors (TFs) playing a crucial role. In this study, we investigated transcriptional gene signatures (TGSs) for their potential to predict ICI resistance in melanoma by analyzing two independent clinical trial datasets. Among the identified TFs, NFATC2 (Nuclear Factor of Activated T Cells 2) was observed to be a promising marker for resistance to anti-PD-1 therapy. NFATC2, a regulator of T cell activation, may be co-opted by melanoma cells to evade immune surveillance. Our analysis indicated that elevated NFATC2 TGS scores were associated with ICI resistance and poorer survival outcomes across multiple melanoma cohorts. Validation in independent datasets further suggested NFATC2's potential predictive value, particularly in patients without liver metastasis or with prior anti-CTLA-4 therapy. Elevated NFATC2 TGS scores also correlated with reduced immune cell infiltration, specifically of CD8+ T cells, increased markers of T cell exhaustion, and higher tumor purity. These findings support NFATC2 TGS as a candidate biomarker for stratifying melanoma patients and potentially informing ICI therapy response. Further research into NFATC2-associated immune evasion mechanisms may offer insights for overcoming resistance to immunotherapy.
  
  
  
  
**NFATC2**
-
3
of
10
  

### [Detection of EWSR1 gene rearrangement by fluorescence in situ hybridization in bone and soft tissue tumors: clinical application evaluation and atypical signal analysis].

3. Zhonghua Bing Li Xue Za Zhi. 2024 May 8;53(5):458-463. doi: 10.3760/cma.j.cn112151-20231025-00308
  
  
[Article in Chinese; Abstract available in Chinese from the publisher]
  
  
Li L(1), Zhang M(1), Zhang TT(1), Ding Y(1).
  
  
Author information: (1)Department of Pathology, Beijing Jishuitan Hospital, Capital Medical University, Beijing 100035, China.
  
  
  
  
**NFATC2**
-
4
of
10
  

### PARP1 Is a Prognostic Marker and Targets NFATC2 to Promote Carcinogenesis in Melanoma.

4. Genet Test Mol Biomarkers. 2022 Nov;26(11):503-511. doi: 10.1089/gtmb.2021.0214 Epub 2022 Nov 17.
  
  
Mou K(1), Zhou Y(1), Mu X(1), Zhang J(1), Wang L(1), Ge R(1).
  
  
Author information: (1)Department of Dermatology, The First Affiliated Hospital of Xi'an Jiaotong Un ...
  
  
Background: Melanoma is the most lethal skin tumor. PARP1 plays an oncogenic role in tumors, but the mechanism of PARP1 in melanoma remains unclear. Explicating the functional mechanism of PARP1 might highlight new targets for improving the survival rate of melanoma patients. Methods: The expression level of PARP1 and its correlation with prognosis of melanoma were examined using the TCGA dataset, the GEO12391 dataset, and western blot analysis. The differentially expressed genes (DEGs) following PARP1 intervention were identified via microarray analyses. GSEA, GO, and KEGG pathway enrichment analyses of the DEGs were performed. Theses DEGs were also compared with PARP1-related DEGs in the GEO59455 dataset and correlation analyses were performed to identify the intersection of the two gene sets to identify PARP1 target genes. The regulatory relationship between PARP1 and its target gene NFATC2 was examined by Western blot analysis and RT-qPCR. A CCK8 assay was used to determine the biological role of PARP1 and its target, NFATC2, in melanoma. Results: We demonstrated that PARP1 was overexpressed in both melanoma tissues and melanoma cell lines, and that upregulated expression of PARP1 was associated with higher pathological stages and unfavorable prognosis of melanoma. Five genes (NFATC2, ITGAX, CYP26B1, SYT17, and VGF) were identified as PARP1 target genes, and NFATC2 expression was confirmed to be regulated by PARP1. A CCK8 assay showed that by down-regulating expression of PARP1 or NFATC2 that melanoma proliferation was inhibited. Conclusions: Taken together, the results of this study demonstrated that PARP1 expression is a prognostic marker and contributes to melanoma proliferation through the regulation of NFATC2.
  
  
  
  
**NFATC2**
-
5
of
10
  

### Robust gene expression programs underlie recurrent cell states and phenotype switching in melanoma.

5. Nat Cell Biol. 2020 Aug;22(8):986-998. doi: 10.1038/s41556-020-0547-3 Epub 2020 Aug 3.
  
  
Wouters J(#)(1)(2), Kalender-Atak Z(#)(1)(2)(3), Minnoye L(1)(2), Spanier KI(1)(2), De Waegeneer M(1 ...
  
  
Author information: (1)Center for Brain & Disease Research, VIB-KU Leuven, Leuven, Belgium. (2)Depar ...
  
  
Comment in Nat Cell Biol. 2020 Aug;22(8):913-914. doi: 10.1038/s41556-020-0556-2.
  
  
  
  
**NFATC2**
-
6
of
10
  

### Redox signals at the ER-mitochondria interface control melanoma progression.

6. EMBO J. 2019 Aug 1;38(15):e100871. doi: 10.15252/embj.2018100871 Epub 2019 Jul 15.
  
  
Zhang X(1)(2), Gibhardt CS(1), Will T(3), Stanisz H(4), Körbel C(5), Mitkovski M(6), Stejerean I(1), ...
  
  
Author information: (1)Molecular Physiology, Institute of Cardiovascular Physiology, University Medi ...
  
  
Reactive oxygen species (ROS) are emerging as important regulators of cancer growth and metastatic spread. However, how cells integrate redox signals to affect cancer progression is not fully understood. Mitochondria are cellular redox hubs, which are highly regulated by interactions with neighboring organelles. Here, we investigated how ROS at the endoplasmic reticulum (ER)-mitochondria interface are generated and translated to affect melanoma outcome. We show that TMX1 and TMX3 oxidoreductases, which promote ER-mitochondria communication, are upregulated in melanoma cells and patient samples. TMX knockdown altered mitochondrial organization, enhanced bioenergetics, and elevated mitochondrial- and NOX4-derived ROS. The TMX-knockdown-induced oxidative stress suppressed melanoma proliferation, migration, and xenograft tumor growth by inhibiting NFAT1. Furthermore, we identified NFAT1-positive and NFAT1-negative melanoma subgroups, wherein NFAT1 expression correlates with melanoma stage and metastatic potential. Integrative bioinformatics revealed that genes coding for mitochondrial- and redox-related proteins are under NFAT1 control and indicated that TMX1, TMX3, and NFAT1 are associated with poor disease outcome. Our study unravels a novel redox-controlled ER-mitochondria-NFAT1 signaling loop that regulates melanoma pathobiology and provides biomarkers indicative of aggressive disease.
  
  
  
  
**NFATC2**
-
7
of
10
  

### An actionable axis linking NFATC2 to EZH2 controls the EMT-like program of melanoma cells.

7. Oncogene. 2019 May;38(22):4384-4396. doi: 10.1038/s41388-019-0729-2 Epub 2019 Feb 1.
  
  
Perotti V(1), Baldassari P(1), Molla A(1), Nicolini G(1), Bersani I(1), Grazia G(1), Benigni F(2), M ...
  
  
Author information: (1)Department of Research, Human Tumors Immunobiology Unit, Milan, Italy. (2)HuM ...
  
  
Discovery of new actionable targets and functional networks in melanoma is an urgent need as only a fraction of metastatic patients achieves durable clinical benefit by targeted therapy or immunotherapy approaches. Here we show that NFATC2 expression is associated with an EMT-like transcriptional program and with an invasive melanoma phenotype, as shown by analysis of melanoma cell lines at the mRNA and protein levels, interrogation of the TCGA melanoma dataset and characterization of melanoma lesions by immunohistochemistry. Gene silencing or pharmacological inhibition of NFATC2 downregulated EMT-related genes and AXL, and suppressed c-Myc, FOXM1, and EZH2. Targeting of c-Myc suppressed FOXM1 and EZH2, while targeting of FOXM1 suppressed EZH2. Inhibition of c-Myc, or FOXM1, or EZH2 downregulated EMT-related gene expression, upregulated MITF and suppressed migratory and invasive activity of neoplastic cells. Stable silencing of NFATC2 impaired melanoma cell proliferation in vitro and tumor growth in vivo in SCID mice. In NFATC2+ EZH2+ melanoma cell lines pharmacological co-targeting of NFATC2 and EZH2 exerted strong anti-proliferative and pro-apoptotic activity, irrespective of BRAF or NRAS mutations and of BRAF inhibitor resistance. These results provide preclinical evidence for a role of NFATC2 in shaping the EMT-like melanoma phenotype and reveal a targetable vulnerability associated with NFATC2 and EZH2 expression in melanoma cells belonging to different mutational subsets.
  
  
  
  
**NFATC2**
-
8
of
10
  

### Correction to: NFATC2 is an intrinsic regulator of melanoma dedifferentiation.

8. Oncogene. 2019 May;38(19):3763-3764. doi: 10.1038/s41388-019-0679-8
  
  
Perotti V(1), Baldassari P(1), Molla A(1), Vegetti C(1), Bersani I(1), Maurichi A(2), Santinami M(2) ...
  
  
Author information: (1)Human Tumors Immunobiology Unit, Department of Experimental Oncology and Mole ...
  
  
Erratum for Oncogene. 2016 Jun 2;35(22):2862-72. doi: 10.1038/onc.2015.355.
  
  
  
  
**NFATC2**
-
9
of
10
  

### NFAT1 enhances the effects of tumor-associated macrophages on promoting malignant melanoma growth and metastasis.

9. Biosci Rep. 2018 Dec 18;38(6):BSR20181604. doi: 10.1042/BSR20181604 Print 2018 Dec 21.
  
  
Liu H(1), Yang L(2), Qi M(3), Zhang J(4).
  
  
Author information: (1)Department of Dermatology, Xiangya Hospital, Central South University, Changs ...
  
  
Tumor-associated macrophages (TAMs) play substantial roles in tumor growth, invasion, and metastasis. Nuclear factor of activated T cell (NFAT1) has been shown to promote melanoma growth and metastasis in vivo We herein aim to investigate whether NFAT1 is capable to promote melanoma growth and metastasis by influencing TAM properties. Melanoma-conditioned TAMs were obtained from human monocytes after incubation with conditioned medium from A375 cell culture. The phenotype of the macrophages was detected. Cell proliferation, migration, and invasion were evaluated. Human malignant melanoma tissues exhibited increased CD68+-macrophage infiltration and NFAT1 expression compared with the normal pigmented nevus tissues. Melanoma-conditioned TAMs displayed M2-like phenotype. Melanoma-conditioned TAMs also promoted proliferation, migration, and invasion of human malignant melanoma cell lines A375 and WM451. Furthermore, NFAT1 expression in TAMs was significantly increased compared with the M0 group. NFAT1 overexpression significantly strengthened the melanoma-conditioned TAM-mediated promotion of cell migration and invasion in A375 and WM451 cells, whereas NFAT1 knockdown exerted the opposite effects. Moreover, NFAT1 overexpression in melanoma-conditioned TAMs promoted CD68+-macrophage infiltration, tumor growth, and metastasis in vivo NFAT1 may play a critical role in enhancing the TAM-mediated promotion of growth and metastasis in malignant melanoma.
  
  
  
  
**NFATC2**
-
10
of
10
  

### NFAT1 Directly Regulates IL8 and MMP3 to Promote Melanoma Tumor Growth and Metastasis.

10. Cancer Res. 2016 Jun 1;76(11):3145-55. doi: 10.1158/0008-5472.CAN-15-2511 Epub 2016 Mar 24.
  
  
Shoshan E(1), Braeuer RR(1), Kamiya T(1), Mobley AK(1), Huang L(1), Vasquez ME(1), Velazquez-Torres ...
  
  
Author information: (1)Department of Cancer Biology, The University of Texas MD Anderson Cancer Cent ...
  
  
Nuclear factor of activated T cell (NFAT1, NFATC2) is a transcription factor that binds and positively regulates IL2 expression during T-cell activation. NFAT1 has important roles in both innate and adaptive immune responses, but its involvement in cancer is not completely understood. We previously demonstrated that NFAT1 contributes to melanoma growth and metastasis by regulating the autotaxin gene (Enpp2). Here, we report a strong correlation between NFAT1 expression and metastatic potential in melanoma cell lines and tumor specimens. To elucidate the mechanisms underlying NFAT1 overexpression during melanoma progression, we conducted a microarray on a highly metastatic melanoma cell line in which NFAT1 expression was stably silenced. We identified and validated two downstream targets of NFAT1, IL8, and MMP3. Accordingly, NFAT1 depletion in metastatic melanoma cell lines was associated with reduced IL8 and MMP3 expression, whereas NFAT1 overexpression in a weakly metastatic cell line induced expression of these targets. Restoration of NFAT1 expression recovered IL8 and MMP3 expression levels back to baseline, indicating that both are direct targets of NFAT1. Moreover, in vivo studies demonstrated that NFAT1 and MMP3 promoted melanoma tumor growth and lung metastasis. Collectively, our findings assign a new role for NFAT1 in melanoma progression, underscoring the multifaceted functions that immunomodulatory factors may acquire in an unpredictable tumor microenvironment. Cancer Res; 76(11); 3145-55. ©2016 AACR.
  
  
  
  
  

---

  

# NFKBIA

**Signature gene in:** MDM
  
**Differentially expressed in:** no cell type
  
  
**NFKBIA**
-
1
of
10
  

### Bioinformatics data combined with single-cell analysis reveals patterns of immunoinflammatory infiltration and cell death in melanoma.

1. Int Immunopharmacol. 2024 Dec 25;143(Pt 2):113347. doi: 10.1016/j.intimp.2024.113347 Epub 2024 Oct 16.
  
  
Jin L(1), Yang G(2), Liu Y(2), Rang Z(2), Cui F(3).
  
  
Author information: (1)Department of Dermatology, The Affiliated Hospital of Southwest Medical Unive ...
  
  
BACKGRUOND: Melanoma is a common cancer in dermatology, but its molecular mechanisms remain poorly explained. AIM: Utilizing single-cell analytics and bioinformatics, the work sought to discover the immunological infiltration and cellular molecular mechanisms of melanoma. METHODS: Melanoma genes databases were downloaded from GeneCards, and gene expression profiles were chosen from the Gene Expression Omnibus (GSE244889). Establishing and analyzing protein-protein interaction networks for functional enrichment made use of the Kyoto Encyclopedia of Genes and Genomes (KEGG) and Gene Ontology (GO) databases. The process assesses the immunological cell infiltration variations between normal and malignant samples by Immune Cell AI software program. Different cell type differences were clarified by cell quality control, filtration, removal of batch effects and cell clustering analysis using single cell analysis techniques. RESULTS: Using a variety of machine learning techniques, 20 differentially expressed hub genes were found; among these, TP53, HSP90AB1, HSPA4, RHOA, CCND1, CYCS, PPARG, NFKBIA, CAV1, ANXA5, ENO1, ITGAM, YWHAZ, RELA, SOD1, and VDAC1 were found to be significantly significant. The results of enrichment analysis demonstrated that immune response and inflammatory response were strongly associated with melanoma. Animal mitophagy, ferroptosis, the PI3K-Akt signaling pathway, and the HIF-1 signaling pathway were the primary signaling pathways implicated. Cells of immunity, T-cells, lymphocytes, B-cells, NK-cells, monocytes, and macrophages were shown to be significantly infiltrated in melanoma patients, according to analysis. Single cell analysis also demonstrated that ferroptosis is a significant mechanism of cell death that contributes to the advancement of melanoma and that macrophages are important in the disease. CONCLUSION: In summary, different immune cell infiltrations-particularly macrophages-have a significant impact on the onset and course of melanoma, and our findings may help direct future investigations into melanoma macrophages.
  
  
  
  
**NFKBIA**
-
2
of
10
  

### Exploring and validating the metastasis mechanism of pathenolide interfering with cutaneous melanoma through ER stress-dependent apoptosis based on the network pharmacology.

2. Phytochem Anal. 2023 Oct;34(7):745-754. doi: 10.1002/pca.3193 Epub 2022 Dec 7.
  
  
Liu LP(1), Liu N(2), Zhang HN(3), Li D(1).
  
  
Author information: (1)Institute of Integrated Medicine, Medical College, Qingdao University, Qingda ...
  
  
BACKGROUND: This study aimed to explore the mechanism of parthenolide in inhibiting melanoma metastasis through network pharmacology and cell experiment. MATERIALS AND METHODS: This research obtained the targets of the drug from the HERB database and PubChem database, the differential expression gene of metastatic cutaneous melanoma was obtained by differentially expression gene analysis of four Gene Expression Omnibus (GEO) datasets. The intersection of drug targets and differentially expression genes were considered to be related to drugs that inhibit metastasis of cutaneous melanoma. The STRING database was used to construct the protein-protein interaction (PPI) network, and cytohubba package in Cytoscape software was used to rank the PPI network targets. The enrichment analysis was used to screen out the relevance Kyoto Encyclopedia of Genes and Genomes (KEGG) pathway and gene ontology to explain the underlying mechanism of drug inhibiting the cutaneous melanoma metastatic; cell viability, apoptosis, cell migration and protein levels were assessed using cell counting kit-8 (CCK-8) assay, Annexin V-FITC/PI assay, wound healing assay, respectively. Finally, combining pathway maps and literature, we detected ATF4 and proteins upstream and downstream of ATF4 through Western blot. RESULTS: A total of 87 targets were screened out from the drug databases, and a total of 1635 differentially expression genes was obtained from the differentially expression genes analysis of GEO datasets, a total of nine targets (VEGFA, ANXA5, ICAM1, SELE, NFKBIA, ATF4, CTNNB1, SELP and HPGDS) were considered to be related to drugs that inhibit metastasis of cutaneous melanoma. The result of enrichment analysis showed that the drug inhibits the metastatic of cutaneous melanoma through multiple pathways such as TNF signalling pathway, lipid and atherosclerosis and fluid shear stress and atherosclerosis, relevance multiple biological processes, cellular components and molecular function; cell experiments showed that parthenolide could inhibit tumour cell migration and induce a decrease of cell viability. Flow cytometry results showed that parthenolide induced tumour cell apoptosis. Western blot results suggested that parthenolide exerted therapeutic effects by regulating ATF4 protein and its upstream and downstream proteins, namely endoplasmic reticulum (ER) stress signalling pathway. CONCLUSION: Parthenolide induces ER stress-dependent apoptosis in melanoma cells.
  
  
  
  
**NFKBIA**
-
3
of
10
  

### SMRT and NCoR1 fine-tune inflammatory versus tolerogenic balance in dendritic cells by differentially regulating STAT3 signaling.

3. Front Immunol. 2022 Sep 27;13:910705. doi: 10.3389/fimmu.2022.910705 eCollection 2022.
  
  
Jha A(1)(2), Ahad A(1)(2), Mishra GP(1)(3), Sen K(4), Smita S(1)(2), Minz AP(1), Biswas VK(1)(3), Tr ...
  
  
Author information: (1)Immuno-genomics & Systems Biology laboratory, Institute of Life Sciences (ILS ...
  
  
Dendritic cell (DC) fine-tunes inflammatory versus tolerogenic responses to protect from immune-pathology. However, the role of co-regulators in maintaining this balance is unexplored. NCoR1-mediated repression of DC immune-tolerance has been recently reported. Here we found that depletion of NCoR1 paralog SMRT (NCoR2) enhanced cDC1 activation and expression of IL-6, IL-12 and IL-23 while concomitantly decreasing IL-10 expression/secretion. Consequently, co-cultured CD4+ and CD8+ T-cells depicted enhanced Th1/Th17 frequency and cytotoxicity, respectively. Comparative genomic and transcriptomic analysis demonstrated differential regulation of IL-10 by SMRT and NCoR1. SMRT depletion represses mTOR-STAT3-IL10 signaling in cDC1 by down-regulating NR4A1. Besides, NFKBIA and Socs3 were down-regulated in Ncor2 (Smrt) depleted cDC1, supporting increased production of inflammatory cytokines. Moreover, studies in mice showed, adoptive transfer of SMRT depleted cDC1 in OVA-DTH induced footpad inflammation led to increased Th1/Th17 and reduced tumor burden after B16 melanoma injection by enhancing oncolytic CD8+ T-cell frequency, respectively. We also depicted decreased Ncor2 expression in Rheumatoid Arthritis, a Th1/Th17 disease.
  
  
  
  
**NFKBIA**
-
4
of
10
  

### NF-кB pathway genes expression in chicken erythrocytes infected with avian influenza virus subtype H9N2.

4. Br Poult Sci. 2021 Oct;62(5):666-671. doi: 10.1080/00071668.2021.1902478 Epub 2021 May 19.
  
  
Khan A(1), Jahejo AR(1), Qiao ML(1), Han XY(1), Cheng QQ(1), Mangi RA(1), Qadir MF(1), Zhang D(1), B ...
  
  
Author information: (1)College of Veterinary Medicine, Shanxi Agricultural University, Jinzhong, Chi ...
  
  
NA
  
  
  
  
**NFKBIA**
-
5
of
10
  

### DOI: 10.1080/00071668.2021.1902478 PMID: 33843365 [Indexed for MEDLINE]

1. Chicken erythrocytes in blood vessels are the most abundant circulating cells, which participate in the host's immune responses. The transcription factor nuclear factor-kappa B (NF-κB) plays a vital role in the inflammatory response following viral infections. However, the expression of the NF-κB pathway, and other immune-related genes in chicken erythrocytes infected with low pathogenic avian influenza virus (LPAIV H9N2), has not been extensively studied.2. The following study determined the interaction of LPAIV H9N2 with chicken erythrocytes using indirect immunofluorescence microscopy. This was followed by investigating myeloid differentiation primary response 88 (MyD88), C-C motif chemokine ligand 5 (CCL5), melanoma differentiation-associated protein 5 (MDA5), the inhibitor of nuclear factor-kappa B kinase subunit epsilon (IKBKE), NF-κB inhibitor alpha (NFKBIA), NF-κB inhibitor epsilon (NFKBIE), interferon-alpha (IFN-α), colony-stimulating factor 3 (CSF3) and tumour necrosis factor receptor-associated factor 6 (TRAF6) by mRNA expression using quantitative real-time PCR (qRT-PCR) at four different time intervals (0, 2, 6 and 10 h).3. There was a significant interaction between erythrocytes and LPAIV H9N2 virus. Furthermore, the mRNA expression of the NF-κB pathway and other immune-related genes were significantly up-regulated at 2 h post-infection in infected chicken erythrocytes, except for TRAF6, which were significantly downregulated. While at 0 h post-infection, IFN-α and CSF3 were significantly upregulated, whereas NFKBIA was significantly downregulated. Further expression of MDA5, CCL5 and NFKBIA was upregulated, while TRAF6 was downregulated at 6 h post-infection. In infected erythrocytes, expression of MyD88, CCL5 and IKBKE was upregulated. However, IFN-α and TRAF6 were downregulated at 10 h post-infection.4. These results give initial evidence that the NF-κB pathway, and other genes related to immunity, in chicken erythrocytes may contribute to LPAIV subtype H9N2 and induce host immune responses.
  
  
NA
  
  
NA
  
  
NA
  
  
  
  
**NFKBIA**
-
6
of
10
  

### Deuterium Oxide (D(2)O) Induces Early Stress Response Gene Expression and Impairs Growth and Metastasis of Experimental Malignant Melanoma.

5. Cancers (Basel). 2021 Feb 3;13(4):605. doi: 10.3390/cancers13040605
  
  
Jandova J(1), Hua AB(1), Fimbres J(1), Wondrak GT(1).
  
  
Author information: (1)Department of Pharmacology and Toxicology, College of Pharmacy & UA Cancer Ce ...
  
  
There are two stable isotopes of hydrogen, protium (1H) and deuterium (2H; D). Cellular stress response dysregulation in cancer represents both a major pathological driving force and a promising therapeutic target, but the molecular consequences and potential therapeutic impact of deuterium (2H)-stress on cancer cells remain largely unexplored. We have examined the anti-proliferative and apoptogenic effects of deuterium oxide (D2O; 'heavy water') together with stress response gene expression profiling in panels of malignant melanoma (A375V600E, A375NRAS, G361, LOX-IMVI), and pancreatic ductal adenocarcinoma (PANC-1, Capan-2, or MIA PaCa-2) cells with inclusion of human diploid Hs27 skin fibroblasts. Moreover, we have examined the efficacy of D2O-based pharmacological intervention in murine models of human melanoma tumor growth and metastasis. D2O-induction of apoptosis was substantiated by AV-PI flow cytometry, immunodetection of PARP-1, and pro-caspase 3 cleavage, and rescue by pan-caspase inhibition. Differential array analysis revealed early modulation of stress response gene expression in both A375 melanoma and PANC-1 adenocarcinoma cells elicited by D2O (90%; ≤6 h) (upregulated: CDKN1A, DDIT3, EGR1, GADD45A, HMOX1, NFKBIA, or SOD2 (up to 9-fold; p < 0.01)) confirmed by independent RT-qPCR analysis. Immunoblot analysis revealed rapid onset of D2O-induced stress response phospho-protein activation (p-ERK, p-JNK, p-eIF2α, or p-H2AX) or attenuation (p-AKT). Feasibility of D2O-based chemotherapeutic intervention (drinking water (30% w/w)) was demonstrated in a severe combined immunodeficiency (SCID) mouse melanoma metastasis model using luciferase-expressing A375-Luc2 cells. Lung tumor burden (visualized by bioluminescence imaging) was attenuated by D2O, and inhibition of invasiveness was also confirmed in an in vitro Matrigel transwell invasion assay. D2O supplementation also suppressed tumor growth in a murine xenograft model of human melanoma, and median survival was significantly increased without causing adverse effects. These data demonstrate for the first time that systemic D2O administration impairs growth and metastasis of malignant melanoma through the pharmacological induction of deuterium (2H)-stress.
  
  
  
  
**NFKBIA**
-
7
of
10
  

### Epigallocatechin-3-gallate(EGCG) suppresses melanoma cell growth and metastasis by targeting TRAF6 activity.

6. Oncotarget. 2016 Nov 29;7(48):79557-79571. doi: 10.18632/oncotarget.12836
  
  
Zhang J(1)(2), Lei Z(1)(2), Huang Z(3), Zhang X(1)(2), Zhou Y(1)(2), Luo Z(1)(2), Zeng W(1)(2), Su J ...
  
  
Author information: (1)The Department of Dermatology, Xiangya Hospital, Central South University, Ch ...
  
  
TRAF6 (TNF Receptor-Associated Factor 6) is an E3 ubiquitin ligase that contains a Ring domain, induces K63-linked polyubiquitination, and plays a critical role in signaling transduction. Our previous results demonstrated that TRAF6 is overexpressed in melanoma and that TRAF6 knockdown dramatically attenuates tumor cell growth and metastasis. In this study, we found that EGCG can directly bind to TRAF6, and a computational model of the interaction between EGCG and TRAF6 revealed that EGCG probably interacts with TRAF6 at the residues of Gln54, Gly55, Asp57 ILe72, Cys73 and Lys96. Among these amino acids, mutation of Gln54, Asp57, ILe72 in TRAF6 could destroy EGCG bound to TRAF6, furthermore, our results demonstrated that EGCG significantly attenuates interaction between TRAF6 and UBC13(E2) and suppresses TRAF6 E3 ubiquitin ligase activity in vivo and in vitro. Additionally, the phosphorylation of IκBα, p-TAK1 expression are decreased and the nuclear translocation of p65 and p50 is blocked by treatment with EGCG, leading to inactivation of the NF-κB pathway. Moreover, EGCG significantly inhibits cell growth as well as the migration and invasion of melanoma cells. Taken together, these findings show that EGCG is a novel E3 ubiquitin ligase inhibitor that could be used to target TRAF6 for chemotherapy or the prevention of melanoma.
  
  
  
  
**NFKBIA**
-
8
of
10
  

### Hinokitiol, a tropolone derivative, inhibits mouse melanoma (B16-F10) cell migration and in vivo tumor formation.

7. Eur J Pharmacol. 2015 Jan 5;746:148-57. doi: 10.1016/j.ejphar.2014.11.011 Epub 2014 Nov 20.
  
  
Huang CH(1), Lu SH(2), Chang CC(3), Thomas PA(4), Jayakumar T(5), Sheu JR(6).
  
  
Author information: (1)Graduate Institute of Medical Sciences, Taipei Medical University, Taipei 110 ...
  
  
Invasion and metastasis are the major causes of treatment failure in patients with cancer. Hinokitiol, a natural bioactive compound found in Chamacyparis taiwanensis, has been used in hair tonics, cosmetics, and food as an antimicrobial agent. In this study, we investigated the effects and possible mechanisms of action of hinokitiol on migration by the metastatic melanoma cell line, B16-F10, in which matrix metalloproteinase-1 (MMP-1) is found to be highly- expressed. Treatment with hinokitiol revealed a concentration-dependent inhibition of migration of B16-F10 melanoma cells. Hinokitiol appeared to achieve this effect by reducing the expression of MMP-1 and by suppressing the phosphorylation of mitogen- activated protein kinase (MAPK) signaling molecules such as extracellular signal-regulated kinase (ERK) 1/2, p38 MAPK and c-Jun N-terminal kinases (JNK). On the other hand, hinokitiol treatment reversed IκB-α degradation and inhibited the phosphorylation of p65 nuclear factor kappa B (NF-κB) and cJun in B16-F10 cells. In addition, hinokitiol suppressed the translocation of p65 NF-κB from the cytosol to the nucleus, suggesting reduced NF-κB activation. Consistent with these in vitro findings, our in vivo study demonstrated that hinokitiol treatment significantly reduced the total number of mouse lung metastatic nodules and improved histological alterations in B16-F10 injected C57BL/6 mice. These findings suggest that treatment of B16-F10 cells with hinokitiol significantly inhibits metastasis, possibly by blocking MMP-1 activation, MAPK signaling pathways and inhibition of the transcription factors, NF-κB and c-Jun, involved in cancer cell migration. These results may accelerate the development of novel therapeutic agents for the treatment of malignant cancers.
  
  
  
  
**NFKBIA**
-
9
of
10
  

### Hyaluronan-binding protein 1 (HABP1/p32/gC1qR) induces melanoma cell migration and tumor growth by NF-kappa B dependent MMP-2 activation through integrin α(v)β(3) interaction.

8. Cell Signal. 2011 Oct;23(10):1563-77. doi: 10.1016/j.cellsig.2011.04.009 Epub 2011 May 6.
  
  
Prakash M(1), Kale S, Ghosh I, Kundu GC, Datta K.
  
  
Author information: (1)School of Environmental Sciences, Jawaharlal Nehru University, New Delhi, Ind ...
  
  
Cell migration is the hallmark of cancer regulating anchorage independent growth and invasiveness of tumor cells. Hyaluronan (HA), an ECM polysaccharide is shown to regulate this process. In the present report, we demonstrated, supplementation of purified recombinant hyaluronan binding protein 1(HABP1/p32/gC1qR) from human fibroblast cDNA enhanced migration potential of highly invasive melanoma (B16F10) cells. Exogenous HABP1 adhered to the cell surface transiently and was shown to interact and colocalize with α(v)β(3) integrin, a regulatory molecule of cell migration. In HABP1 treated cells, the phosphorylation of nuclear factor inducing kinase (NIK) and IκBα was observed, followed by nuclear translocation of p65 subunit of NFκB, along with its DNA-binding and transactivation, resulting in upregulation of MT1-MMP expression and finally MMP-2 activation. To substantiate our findings, prior to HABP1 treatment, the expression of NIK was reduced by small interfering RNA mediated knockdown and confirmed the inhibition of nuclear translocation of p65 subunit of NFκB and upregulation of MT1-MMP expression. In addition, the use of curcumin, an anti-cancer drug, or GRGDSP, the blocking peptide along with exogenous HABP1, inhibited such NFκB-dependent pathway, confirming that HABP1-induced cell migration is α(v)β(3) integrin-mediated and downstream signaling by NFκB. Finally, we translated the in vitro data in mice model and observed enhanced tumor growth with higher MT1-MMP expression and MMP-2 activation in the tumors upon injection of HABP1 treated melanoma cells. The treatment of curcumin, the anticancer drug along with HABP1, inhibited the migration, expression of MT1-MMP and activation of MMP-2 and finally tumor growth supports the involvement of HABP1 in tumor formation.
  
  
  
  
**NFKBIA**
-
10
of
10
  

### Characterization and mechanistic studies of a novel melanoma-targeting construct containing IκBa for specific inhibition of nuclear factor-κB activity.

9. Neoplasia. 2010 Oct;12(10):766-77. doi: 10.1593/neo.10214
  
  
Zhou H(1), Liu Y, Cheung LH, Kim S, Zhang W, Mohamedali KA, Anand P, Hittelman WN, Aggarwal BB, Rose ...
  
  
Author information: (1)Immunopharmacology & Targeted Therapy Laboratory, Department of Experimental ...
  
  
The transcription factor nuclear factor-κB (NF-κB) is a central mediator of growth and homeostasis for both normal and neoplastic cells. IκBα is the natural intracellular inhibitor of NF-κB and can effectively complex with and thereby inhibit the biologic activity and translocation of NF-κB to the nucleus. We designed a fusion protein designated IκBα/scFvMEL composing of human IκBα and the single-chain antibody scFvMEL, targets melanoma gp240 antigen. Cells treated with IκBα/scFvMEL before irradiation showed specifically inhibition of both constitutive and radiation-induced NF-κB activity on gp240 antigen-positive A375M cells. Pretreatment of A375M cells with IκBα/scFvMEL significantly sensitized melanoma cells to ionizing radiation assessed using a clonogenic survival assay. Mechanistic studies showed that IκBα/scFvMEL, when exogenously added to A375M cells, could be coimmunoprecipitated with the p65 subunit of NF-κB. IκBα/scFvMEL inhibited in a time and/or dose-dependent manner of tumor necrosis factor α- or radiation-induced NF-κB activity in vitro. IκBα/scFvMEL was also shown to specifically inhibit the translocation of the NF-κB p65 subunit to the cell nucleus and NF-κB-mediated gene transcription. Further, initial studies showed that mice bearing well-established A375M xenografts were treated (intravenously) with IκBα/scFvMEL and showed a significant suppression of tumor growth. We also observed a decrease in levels of Bcl-2 and Bcl-XL signaling events downstream of NF-κB in the tumor model. These studies demonstrate for the first time that tumor cell-targeted delivery of IκBα may be beneficial for the treatment of melanoma when combined with standard anticancer therapies such as radiation.
  
  
  
  
  

---

  

# PIK3R1

**Signature gene in:** Tregs
  
**Differentially expressed in:** no cell type
  
  
**PIK3R1**
-
1
of
10
  

### Mapping variant effects on anti-tumor hallmarks of primary human T cells with base-editing screens.

1. Nat Biotechnol. 2025 Mar;43(3):384-395. doi: 10.1038/s41587-024-02235-x Epub 2024 May 23.
  
  
Walsh ZH(#)(1)(2)(3)(4), Shah P(#)(1)(2)(3)(4), Kothapalli N(1), Shah SB(1), Nikolenyi G(5), Brodtma ...
  
  
Author information: (1)Columbia University Vagelos College of Physicians and Surgeons, New York, NY, ...
  
  
Update of bioRxiv. 2023 Dec 14:2023.12.13.571465. doi: 10.1101/2023.12.13.571465.
  
  
  
  
**PIK3R1**
-
2
of
10
  

### Basigin is necessary for normal decidualization of human uterine stromal cells.

2. Hum Reprod. 2022 Nov 24;37(12):2885-2898. doi: 10.1093/humrep/deac229
  
  
Yang S(1)(2), Bi J(1), Drnevich J(3), Li K(1), Nowak RA(1).
  
  
Author information: (1)Department of Animal Sciences, University of Illinois, Urbana, IL, USA. (2)De ...
  
  
STUDY QUESTION: Does basigin (BSG) regulate human endometrial stromal cell (HESC) decidualization in vitro? SUMMARY ANSWER: BSG regulates HESCs proliferation and decidualization. WHAT IS KNOWN ALREADY: Studies have shown that in the human endometrium, BSG expression is menstrual-cycle dependent and its expression was significantly lower in uterine endometrium during the luteal phase of women experiencing multiple implantation failures after IVF than in women with normal fertility. STUDY DESIGN, SIZE, DURATION: We utilized a telomerase-immortalized HESCs in an in vitro cell culture model system to investigate whether BSG regulates decidualization of stromal cells. Further, we used microarray analysis to identify changes in the gene expression profile of HESCs treated with BSG small interfering RNA (siRNA). All experiments were repeated at least three times. PARTICIPANTS/MATERIALS, SETTING, METHODS: The effect of BSG knockdown (using siRNA) on HESC proliferation was determined by counting cell number and by tritiated thymidine incorporation assays. The effect of BSG on decidualization of HESCs was determined by RT-qPCR for the decidualization markers insulin-like growth factor-binding protein 1 (IGFBP1) and prolactin (PRL). Immunoblotting was used to determine the effect of BSG siRNA on the expression of MMP-2,3. Microarray analysis was used to identify BSG-regulated genes in HESCs at Day 6 of decidualization. Functional and pathway enrichment analyses were then carried out on the differentially expressed genes (DEGs). The STRING online database was used to analyze protein-protein interaction (PPI) between DEG-encoded proteins, and CytoScape software was used to visualize the interaction. MCODE and CytoHubba were used to construct functional modules and screen hub genes separately. Several BSG-regulated genes identified in the microarray analysis were confirmed by qPCR. MAIN RESULTS AND THE ROLE OF CHANCE: Knockdown of BSG expression in cultured stromal cells by siRNA significantly (P < 0.05) inhibited HESC proliferation, disrupted cell decidualization and down-regulated MMP-2 and MMP-3 expression. Microarray analysis identified 721 genes that were down-regulated, and 484 genes up-regulated with P < 0.05 in BSG siRNA treated HESCs. GO term enrichment analysis showed that the DEGs were significantly enriched in cell communication, signaling transduction and regulation, response to stimulus, cell adhesion, anatomical structure morphogenesis, extracellular matrix organization, as well as other functional pathways. KEGG pathway analysis identified upregulated gene enriched in pathways such as the MAPK signaling pathway, colorectal cancer, melanoma and axon guidance. In contrast, downregulated genes were mainly enriched in pathways including ECM-receptor interaction, PI3K-Akt signaling pathway, pathways in cancer, antigen processing, type I diabetes mellitus and focal adhesion. The top 10 hub nodes were identified using 12 methods analyses. The hub genes that showed up in two methods were screened out. Among these genes, upregulated genes included EGFR, HSP90AA1, CCND1, PXN, PRKACB, MGAT4A, EVA1A, LGALS1, STC2, HSPA4; downregulated genes included WNT4/5, FOXO1, CDK1, PIK3R1, IGF1, JAK2, LAMB1, ITGAV, HGF, MXRA8, TMEM132A, UBE2C, QSOX1, ERBB2, GNB4, HSP90B1, LAMB2, LAMC1 and ITGA1. Hub genes and module genes involved in the top three modules of PPI analysis were analyzed through the string database. Analysis showed that hub and module genes were related mainly to the WNT signaling pathway, PI3K-AKT signaling pathway and pathways in cancer. LARGE SCALE DATA: The microarray data set generated in this study has been published online at databank.illinois.edu. LIMITATIONS, REASONS FOR CAUTION: Most of the findings were obtained using an in vitro cell culture system that may not necessarily reflect in vivo functions. WIDER IMPLICATIONS OF THE FINDINGS: Our results demonstrate that BSG plays a vital role in decidualization and that downregulation of BSG in the uterine endometrium may be associated with infertility in women. The identified hub genes and pathways increase our understanding of the genetic etiology and molecular mechanisms underlying the regulation of decidualization by BSG. STUDY FUNDING/COMPETING INTEREST(S): This work was supported by the NIH U54 HD40093 (R.A.N.). The authors have no competing interests to declare.
  
  
  
  
**PIK3R1**
-
3
of
10
  

### Mechanism of Abelmoschus manihot L. in the Treatment of Contrast-Induced Nephropathy on the Basis of Network Pharmacology Analysis.

3. Front Nephrol. 2022 Apr 22;2:834513. doi: 10.3389/fneph.2022.834513 eCollection 2022.
  
  
Xu Z(1), Qian L(2), Niu R(1), Wang Y(1), Yang Y(1), Liu C(1), Lin X(1).
  
  
Author information: (1)Jiangsu Province Hospital of Chinese Medicine, Affiliated Hospital of Nanjing ...
  
  
BACKGROUND: Contrast-induced nephropathy (CIN) is increasingly seen in patients receiving contrast medium. Abelmoschus manihot (L.) Medik. (Malvaceae) and its preparations are widely used and effective in the treatment of various chronic kidney diseases and CIN in China. It is supposed to be an important adjuvant therapy for CIN. METHODS: PubMed and CNKI were searched for the main compounds of A. manihot L. The Swiss target prediction platform, OMIM, GeneCards, DisGeNET, and DrugBank databases were mined for information relevant to the prediction of targets that A. manihot L. in the treatment of CIN. Subsequently, STRING database was applied for the construction of the PPI protein interaction network, meanwhile, the core targets were screened. DAVID database was used to perform the GO function and Kegg signal pathway enrichment analysis. AutoDockTools and PYMOAL were used for molecular docking. Vitro experiments were used to verify the effect of TFA, the main active component of A. manihot L., in the intervention of iopromide-induced cells injury. RESULTS: A total of 17 chemical components and 133 potential targets in A. manihot L. were obtained. The top 15 proteins with higher degree value were selected from the PPI network model, AKT1, PIK3R1, EGFR, SRC,AR, APP, TNF, GAPDH, MMP9, and PTPN1, etc. may be core targets. The enrichment analysis indicated that A. manihot L. was involved in the regulation of PI3K/AKT signaling pathway, FoxO signaling pathway, VEGF signaling pathway, HIF-1, TNF signaling pathway, melanoma, hepatitis B, and other signaling pathways which were mainly associated with the regulation of transcription and apoptosis, protein phosphorylation, inflammatory response, aging, and cell proliferation. Molecular docking indicated that the key components and core targets had a good binding ability. The vitro experiments illustrated that TFA reduces iopromide induced renal tubular cell injury and apoptosis, which may be related to regulating the phosphorylation of AKT. CONCLUSION: The study preliminarily revealed the multi-component, multi-target, and multi-pathway synergistic effects of A. manihot L. on CIN, which provide theoretical reference and basis for the study of the pharmacological mechanism of A. manihot L. in the treatment of CIN.
  
  
  
  
**PIK3R1**
-
4
of
10
  

### Mutational landscape of primary and recurrent Ewing sarcoma.

4. Contemp Oncol (Pozn). 2021;25(4):241-248. doi: 10.5114/wo.2021.112234 Epub 2021 Dec 29.
  
  
Jagodzińska-Mucha P(1), Sobczuk P(1)(2), Mikuła M(3), Raciborska A(4), Dawidowska A(5), Kulecka M(3) ...
  
  
Author information: (1)Department of Soft Tissue/Bone Sarcoma and Melanoma, Maria Sklodowska-Curie N ...
  
  
INTRODUCTION: Ewing sarcoma (ES) is a highly aggressive malignancy of bone and soft tissues characterized by the presence of a genetic fusion involving the EWSR1 gene. More than one-third of patients develop distant metastases, which are associated with unfavorable prognosis. Knowledge about the disease's genetic landscape may help foster progress in using targeted therapies in the treatment of ES. AIM OF THE STUDY: The objective is to assess the mutational landscape of ES in pretreatment samples, tumor samples after neoadjuvant chemotherapy, and in metastatic/recurrent tumors in children and adults. MATERIAL AND METHODS: DNA from 39 formalin-fixed paraffin-embedded tumor samples of 22 patients (17 adults, 5 children) were analyzed by targeted next generation sequencing (NGS) using the Oncomine Comprehensive Assay v3gene panel. Additional functional analyses were performed between patient subgroups. RESULTS: All samples were characterized by low tumor mutation burden (< 10 mut/Mb). The most commonly mutated genes were PIK3R1 (59%) and POLE (50%). The most widely detected variants in biopsy samples were PIK3R1 T369I (50%), FGFR1 E159K, and TP53 at codon 72 (both in 27.3%). Additionally, the ATR,BRCA1, RAD50,ATM,CHEK1, and NBN genes showed a significantly higher number of mutations in ES. Mutations in PIK3R1 were significantly more frequent in adults, while mutations in the pathways responsible for cell cycle control, DNA repair, and transcriptional regulation were more frequent in children. CONCLUSIONS: Besides EWSR1 fusion, ES is characterized by numerous point mutations that are potential targets for precision medicine. There is high genomic heterogeneity that may explain differences in outcomes between patient subgroups.
  
  
  
  
**PIK3R1**
-
5
of
10
  

### Review of the European Society for Photodynamic Therapy (Euro-PDT) Annual Congress 2020.

5. Eur J Dermatol. 2021 Feb 1;31(1):17-21. doi: 10.1684/ejd.2021.3973
  
  
Morton CA(1), Szeimies RM(2), Braathen LR(3).
  
  
Author information: (1)Department of Dermatology, Stirling Community Hospital, Stirling, UK. (2)Depa ...
  
  
This article reviews the 2020 European Society for Photodynamic Therapy (Euro-PDT) Annual Congress. Cutting edge studies included assessment of immunohistochemical variables influencing response of basal cell carcinomas and Bowen's disease to PDT with p53, the only biomarker associated with good response in both conditions. A further study indicated that analysis of molecular markers, such as PIK3R1, could help select patients with actinic keratoses who demonstrate the best response to daylight PDT. Novel delivery protocols include artificial daylight, and laser-assisted and textile PDT. The meeting learnt of novel indications including antimicrobial PDT, as well as methods to optimise daylight PDT, including combination therapy for actinic keratoses. Adverse events were reviewed and options for painless and efficient PDT assessed, including the effect of reduced drug-light interval. A smartphone application was also evaluated which may be used to assist clinicians and patients in effective dosing and timing of daylight PDT via computational algorithms using data from earth observation satellites, to send light and ultraviolet dose information directly to patients' smart phones.
  
  
  
  
**PIK3R1**
-
6
of
10
  

### Diverse mechanisms activate the PI 3-kinase/mTOR pathway in melanomas: implications for the use of PI 3-kinase inhibitors to overcome resistance to inhibitors of BRAF and MEK.

6. BMC Cancer. 2021 Feb 6;21(1):136. doi: 10.1186/s12885-021-07826-4
  
  
Tran KB(1)(2)(3), Kolekar S(1), Jabed A(2), Jaynes P(2), Shih JH(2), Wang Q(2), Flanagan JU(1)(3), R ...
  
  
Author information: (1)Department of Molecular Medicine and Pathology, University of Auckland, Auckl ...
  
  
BACKGROUND: The PI 3-kinase (PI3K) pathway has been implicated as a target for melanoma therapy. METHODS: Given the high degree of genetic heterogeneity in melanoma, we sought to understand the breadth of variation in PI3K signalling in the large NZM panel of early passage cell lines developed from metastatic melanomas. RESULTS: We find the vast majority of lines show upregulation of this pathway, and this upregulation is achieved by a wide range of mechanisms. Expression of all class-IA PI3K isoforms was readily detected in these cell lines. A range of genetic changes in different components of the PI3K pathway was seen in different lines. Coding variants or amplification were identified in the PIK3CA gene, and amplification of the PK3CG gene was common. Deletions in the PIK3R1 and PIK3R2 regulatory subunits were also relatively common. Notably, no genetic variants were seen in the PIK3CD gene despite p110δ being expressed in many of the lines. Genetic variants were detected in a number of genes that encode phosphatases regulating the PI3K signalling, with reductions in copy number common in PTEN, INPP4B, INPP5J, PHLLP1 and PHLLP2 genes. While the pan-PI3K inhibitor ZSTK474 attenuated cell growth in all the lines tested, isoform-selective inhibition of p110α and p110δ inhibited cell growth in only a subset of the lines and the inhibition was only partial. This suggests that functional redundancy exists between PI3K isoforms. Furthermore, while ZSTK474 was initially effective in melanoma cells with induced resistance to vemurafenib, a subset of these cell lines concurrently developed partial resistance to PI3K inhibition. Importantly, mTOR-selective or mTOR/PI3K dual inhibitors effectively inhibited cell growth in all the lines, including those already resistant to BRAF inhibitors and ZSTK474. CONCLUSIONS: Overall, this indicates a high degree of diversity in the way the PI3K pathway is activated in different melanoma cell lines and that mTOR is the most effective point for targeting the growth via the PI3K pathway across all of these cell lines.
  
  
  
  
**PIK3R1**
-
7
of
10
  

### Genetic Profiling of Advanced Melanoma: Candidate Mutations for Predicting Sensitivity and Resistance to Targeted Therapy.

7. Target Oncol. 2020 Feb;15(1):101-113. doi: 10.1007/s11523-020-00695-0
  
  
Olbryt M(1), Pigłowski W(2)(3), Rajczykowski M(4), Pfeifer A(5), Student S(6)(7), Fiszer-Kierzkowska ...
  
  
Author information: (1)Center for Translational Research and Molecular Biology of Cancer, Maria Sklo ...
  
  
BACKGROUND: Molecularly targeted therapy has revolutionized the treatment of advanced melanoma. However, despite its high efficiency, a majority of patients experience relapse within 1 year of treatment because of acquired resistance, and approximately 10-25% patients gain no benefit from these agents owing to intrinsic resistance. This is mainly caused by the genetic heterogeneity of melanoma cells. OBJECTIVE: We aimed to validate the predictive significance of selected genes in advanced melanoma patients before treatment with BRAF/MEK inhibitors. PATIENTS AND METHODS: Archival DNA derived from 37 formalin-fixed paraffin-embedded pre-treatment advanced melanoma samples of patients treated with targeted therapy was used for next-generation sequencing analysis using the Ion Torrent platform. The AmpliSeq Custom Panel comprised coding sequences or hot spots of 23 melanoma genes: ATM, BRAF, CDK4, CDKN2A, CTNNB1, EGFR, HOXD8, HRAS, IDH1, KIT, KRAS, MAP3K8, MAP2K1, MAP2K2, MITF, MYC, NF1, NRAS, PAX5, PIK3R1, PTEN, RAC1, and RB1. The sequences were evaluated for genomic alterations and further validated using Sanger sequencing. RESULTS: Our analysis revealed non-BRAF genetic alterations in 28 out of 37 samples (75.7%). Genetic changes were identified in PTEN, CDK4, CDKN2A, CTNNB1, EGFR, HOXD8, HRAS, KIT, MAP2K1, MAP2K2, MITF, MYC, NF1, PAX5, RAC1, and RB1. Fifteen known pathogenic mutations (single nucleotide variants or indels) and 11 variants of unknown significance were detected. Statistical analysis revealed an association between the presence of pathogenic mutations and time to progression during treatment with combination therapy. CONCLUSIONS: Pathogenic mutations identified by gene panel sequencing have potential predictive value for targeted therapy of melanoma and are worth further validation in a larger series of cases. The role of some known mutations (e.g. CDK4R24, PTEN c.801 + 1G > A, CTNNB1S45F) as well as variants of unknown significance identified in this study (e.g. MITFR316K, KITG498S) in the generation of resistance to BRAF/MEK inhibitors should be further investigated.
  
  
  
  
**PIK3R1**
-
8
of
10
  

### The Small GTPase ARF6 Activates PI3K in Melanoma to Induce a Prometastatic State.

8. Cancer Res. 2019 Jun 1;79(11):2892-2908. doi: 10.1158/0008-5472.CAN-18-3026 Epub 2019 May 2.
  
  
Yoo JH(#)(1), Brady SW(#)(2)(3), Acosta-Alvarez L(4), Rogers A(4), Peng J(4), Sorensen LK(1), Wolff ...
  
  
Author information: (1)Department of Medicine, Program in Molecular Medicine, University of Utah, Sa ...
  
  
Melanoma has an unusual capacity to spread in early-stage disease, prompting aggressive clinical intervention in very thin primary tumors. Despite these proactive efforts, patients with low-risk, low-stage disease can still develop metastasis, indicating the presence of permissive cues for distant spread. Here, we show that constitutive activation of the small GTPase ARF6 (ARF6Q67L) is sufficient to accelerate metastasis in mice with BRAFV600E/Cdkn2aNULL melanoma at a similar incidence and severity to Pten loss, a major driver of PI3K activation and melanoma metastasis. ARF6Q67L promoted spontaneous metastasis from significantly smaller primary tumors than PTENNULL, implying an enhanced ability of ARF6-GTP to drive distant spread. ARF6 activation increased lung colonization from circulating melanoma cells, suggesting that the prometastatic function of ARF6 extends to late steps in metastasis. Unexpectedly, ARF6Q67L tumors showed upregulation of PIK3R1 expression, which encodes the p85 regulatory subunit of PI3K. Tumor cells expressing ARF6Q67L displayed increased PI3K protein levels and activity, enhanced PI3K distribution to cellular protrusions, and increased AKT activation in invadopodia. ARF6 is necessary and sufficient for activation of both PI3K and AKT, and PI3K and AKT are necessary for ARF6-mediated invasion. We provide evidence for aberrant ARF6 activation in human melanoma samples, which is associated with reduced survival. Our work reveals a previously unknown ARF6-PI3K-AKT proinvasive pathway, it demonstrates a critical role for ARF6 in multiple steps of the metastatic cascade, and it illuminates how melanoma cells can acquire an early metastatic phenotype in patients. SIGNIFICANCE: These findings reveal a prometastatic role for ARF6 independent of tumor growth, which may help explain how melanoma spreads distantly from thin, early-stage primary tumors.Graphical Abstract: http://cancerres.aacrjournals.org/content/canres/79/11/2892/F1.large.jpg.
  
  
  
  
**PIK3R1**
-
9
of
10
  

### Potential Mutations in Uveal Melanoma Identified Using Targeted Next-Generation Sequencing.

9. J Cancer. 2019 Jan 1;10(2):488-493. doi: 10.7150/jca.26967 eCollection 2019.
  
  
Yu J(1), Wu X(1), Yan J(1), Yu J(1), Yin T(1), Dai J(1), Ma M(1), Xu T(1), Yu H(1), Xu L(1), Yang L( ...
  
  
Author information: (1)Peking University Cancer Hospital & Institute, Key Laboratory of Carcinogenes ...
  
  
Background/objective: Uveal melanoma (UM) is the most common intraocular malignancy and has a high tendency to metastasize to the liver. Although primary tumours can be successfully treated, there is currently no effective treatment for metastatic UM. To gain insight into the genetics of UM, we performed the targeted next-generation sequencing (NGS) of UM samples from a non-Caucasian population. Methods: This study included tumour samples and blood samples from 107 UM patients at Peking University Cancer Hospital & Institute. Clinical data were collected. DNA was extracted from formalin-fixed, paraffin-embedded (FFPE) specimens. Using the HaloPlex Target Enrichment System (Agilent Technologies), NGS was performed to investigate mutations in a 35-gene panel composed of cancer-related genes. Results: Recurrent coding mutations were found in the known UM drivers GNAQ and GNA11. FOXO1, PIK3R1 and HIF1A were also found to harbour somatic mutations in more than 20% of patients, a result that may indicate previously undescribed associations between these genes and UM pathogenesis. Patients with HIF1A and FOXO1 mutations exhibited worse overall survival (OS). In multivariate analysis, FOXO1 mutation was an independent prognostic factor for OS (P<0.05) that was associated with an increase in the risk ratio by a factor of 1.35. Notably, we found that HIF1A and FOXO1 mutations were associated with metastatic transformation of UM (P<0.05 and P<0.001, respectively). Conclusion: Our findings from analyses of targeted NGS data shed new light on the molecular genetics of UM and facilitate the exploration of mutations associated with metastatic potential.
  
  
  
  
**PIK3R1**
-
10
of
10
  

### Expression profile and biological function of miR-455-5p in colorectal carcinoma.

10. Oncol Lett. 2019 Feb;17(2):2131-2140. doi: 10.3892/ol.2018.9862 Epub 2018 Dec 21.
  
  
Wang J(1), Lu Y(2), Zeng Y(2), Zhang L(3), Ke K(3), Guo Y(1).
  
  
Author information: (1)Department of Breast Surgery, Ningbo First Hospital, Ningbo, Zhejiang 315010, ...
  
  
Underexpression of microRNA-455-5p (miR-455-5p) in medullary thyroid carcinoma, melanoma, gastric cancer and additional cancer types has been reported, which may be associated with carcinoma development. The present study aimed to evaluate the expression profile and biological role of miR-455-5p in colorectal carcinoma. Carcinoma tissues and adjacent tissue specimens from 40 patients with colorectal cancer were randomly collected. Reverse transcription-quantitative polymerase chain reaction (RT-qPCR) analysis was conducted to detect the expression levels of miR-455-5p in colorectal carcinoma and adjacent normal tissues. The biological effects of miR-455-5p on selected colorectal cancer cells were assessed using bromodeoxyuridine assays, wound healing migration assays and flow cytometry. Bioinformatics analysis was implemented to predict the potential target genes of miR-455-5p in colorectal cancer. The expression levels of target genes were further validated by RT-qPCR and western blot analysis of the mRNA and protein levels. The results of the experiments demonstrated that miR-455-5p expression was downregulated in colorectal cancer tissues compared with adjacent normal tissues. In colorectal cancer cells (SW-480, HT-29 and HCT-116), miR-455-5p was observed to inhibit cell proliferation and migration while promoting cell apoptosis. Bioinformatics analysis predicted that the oncogene phosphoinositide-3-kinase regulatory subunit 1 (PIK3R1) was one of the top ranked target genes of miR-455-5p in colorectal cancer cells. This association was validated by RT-qPCR and western blotting. In vivo studies revealed that the expression level of miR-455-5p was significantly downregulated in human colorectal cancer. Further in vitro studies suggested that miR-455-5p may prevent the development of colorectal cancer by downregulating the oncogene PIK3R1. It was concluded that miR-455-5p may target and downregulate PIK3R1 in colorectal cancer.
  
  
  
  
  

---

  

# POU2AF1

**Signature gene in:** Plasma cells
  
**Differentially expressed in:** no cell type
  
  
**POU2AF1**
-
1
of
1
  

### Identification of four genes associated with cutaneous metastatic melanoma.

1. Open Med (Wars). 2020 Jun 11;15(1):531-539. doi: 10.1515/med-2020-0190 eCollection 2020.
  
  
Ji C(1), Li Y(1), Yang K(1), Gao Y(1), Sha Y(1), Xiao D(1), Liang X(2), Cheng Z(1).
  
  
Author information: (1)Department of Dermatology, Zhangjiagang TCM Hospital Affiliated to Nanjing Un ...
  
  
BACKGROUND: Cutaneous melanoma is an aggressive cancer with increasing incidence and mortality rates worldwide. Metastasis is one of the primary elements that influence the prognosis of patients with cutaneous melanoma. This study aims to clarify the potential mechanism underlying the low survival rate of metastatic melanoma and to search for novel target genes to improve the survival rate of patients with metastatic tumors. METHODS: Gene expression dataset and clinical data were downloaded from The Cancer Genome Atlas portal. Differentially expressed genes (DEGs) were identified, and their functions were studied through gene ontology and Kyoto Encyclopedia of Genes and Genomes enrichment analyses. Survival and multivariate Cox regression analyses were used to screen out candidate genes that could affect the prognosis of patients with metastatic melanoma. RESULTS: After a series of comprehensive statistical analysis, 464 DEGs were identified between primary tumor tissues and metastatic tissues. Survival and multivariate Cox regression analyses revealed four vital genes, namely, POU2AF1, ITGAL, CXCR2P1, and MZB1, that affect the prognosis of patients with metastatic melanoma. CONCLUSION: This study provides a new direction for studying the pathogenesis of metastatic melanoma. The genes related to cutaneous metastatic melanoma that affect the overall survival time of patients were identified.
  
  
  
  
  

---

  

# PTEN

**Signature gene in:** MDM
  
**Differentially expressed in:** no cell type
  
  
**PTEN**
-
1
of
10
  

### Federated SPARQL query performance evaluation for exploring disease model mouse: combining gene expression, orthology, and disease knowledge graphs.

1. BMC Med Inform Decis Mak. 2025 May 16;25(Suppl 1):189. doi: 10.1186/s12911-025-03013-8
  
  
Kushida T(1), de Farias TM(2)(3), Sima AC(2), Dessimoz C(2)(3), Chiba H(4), Bastian FB(2)(3), Masuya ...
  
  
Author information: (1)BioResource Research Center, RIKEN, Tsukuba-shi, Japan. tatsuya.kushida@riken ...
  
  
BACKGROUND: The RIKEN BRC develops and maintains the RIKEN BioResource MetaDatabase to help users explore appropriate target bioresources for their experiments and prepare precise and high-quality data infrastructures. The Swiss Institute of Bioinformatics develops two databases across multi-species for the study of gene expression and orthology: Bgee and Orthologous MAtrix (OMA, an orthology database). METHODS: This study combines the RIKEN BioResource data with Resource Description Framework (RDF) datasets from Bgee, a gene expression database, the OMA, the DisGeNET, a human gene-disease association, Mouse Genome Informatics (MGI), UniProt, and four disease ontologies in the RIKEN BioResource MetaDatabase. Our aim is to evaluate the distributed SPARQL query performance when exploring which model organisms are most appropriate for specific medical science research applications across the aforementioned interoperable datasets. More precisely in our biomedical use cases, we investigate disease-related genes, as well as anatomical parts where these genes are expressed and subsequently identify appropriate bioresource candidates available for specific disease research applications. RESULTS: We illustrate the above through two use cases targeting either Alzheimer's disease or melanoma. We identified 14 Alzheimer's disease-related genes that were expressed in the prefrontal cortex (e.g., APP and APOE) and 55 RIKEN bioresources, which were genetically modified mice related to these genes, predicted to be relevant to Alzheimer's disease research. Furthermore, executing a transitive search for the Uberon terms by using the Property Paths function, we identified 14 melanoma-related genes (e.g., HRAS and PTEN), and 12 anatomical parts in which these genes were expressed, such as the "skin of limb" as an example. Finally, we compared the performance of the federated SPARQL query via the remote Bgee SPARQL endpoint with the performance of a centralized SPARQL query using the Bgee dataset as part of the RIKEN BioResource MetaDatabase. CONCLUSIONS: As a result, we confirmed that the performance of the federated approach degraded. We concluded that we reduced the degradation of the query performance of the federated approach from the BioResource MetaDatabase to the SIB by refining the transferred data through a subquery and enhancing the server specifications thereby optimizing the triple store query evaluation.
  
  
  
  
**PTEN**
-
2
of
10
  

### Updates in clinical trial-explored chemopreventive agents for cutaneous melanoma: mechanisms affecting melanocytes.

2. Melanoma Manag. 2025 May 13;12(1):2505400. doi: 10.1080/20450885.2025.2505400 Epub 2025 May 16.
  
  
Ghajar-Rahimi G(1), Yusuf N(1)(2).
  
  
Author information: (1)Department of Dermatology, Heersink School of Medicine, University of Alabama ...
  
  
Cutaneous melanoma is a highly aggressive skin cancer with rising incidence, driven by risk factors such as ultraviolet exposure, genetic predisposition, and immunosuppression. While surgical excision remains the primary treatment, interest in chemoprevention strategies is growing. Numerous natural and synthetic agents have shown preclinical promise, but evaluating their effectiveness is challenging due to their systemic effects on multiple cell types. This review provides a focused examination of the melanocyte-specific mechanisms of select agents that have been tested in clinical trials for melanoma chemoprevention. We discuss various molecular and cellular mechanisms driving the anti-melanoma properties of nonsteroidal anti-inflammatory drugs, statins, sulforaphane, vitamin D, and N-acetylcysteine. Despite promising preclinical and early clinical data, challenges remain regarding precise mechanisms, optimal dosing, long-term safety, and patient selection. Future research should focus on refining melanoma prevention strategies through well-designed clinical trials and personalized approaches integrating genetic and molecular risk factors.
  
  
  
  
**PTEN**
-
3
of
10
  

### Unlocking the power of immune checkpoint inhibitors: Targeting YAP1 reduces anti-PD1 resistance in skin cutaneous melanoma.

3. Br J Pharmacol. 2025 May 5. doi: 10.1111/bph.70052 Online ahead of print.
  
  
Kovács SA(1)(2)(3), Kovács T(4)(5)(6)(7), Lánczky A(8), Paál Á(4)(5)(6)(7), Hegedűs ZI(4)(5)(6)(7), ...
  
  
Author information: (1)Department of Bioinformatics, Semmelweis University, Budapest, Hungary. (2)On ...
  
  
BACKGROUND AND PURPOSE: Immune checkpoint inhibitors, such as anti-PD1, revolutionized melanoma treatment. However, resistance and low response rates remain problems. Our goal was to pinpoint actionable biomarkers of resistance to anti-PD1 treatment and verify therapeutic effectiveness in vivo. EXPERIMENTAL APPROACH: Using receiver operating characteristic (ROC) and survival analysis in a database of 1434 samples, we identified the strongest resistance-associated genes. Inhibitors were evaluated in C57BL/6J mice using wild-type B16-F10, and BRAF, -PTEN, -CDKN2A-mutant YUMM1.7 melanoma cell lines. We investigated the synergistic impact of anti-PD1 therapy and yes-associated protein 1 (YAP1) inhibition by non-photoactivated Verteporfin. Tumour volume was determined at fixed cutoff points, normalized to body weights. KEY RESULTS: In the anti-PD1-treated melanoma cohort, YAP1 was the strongest druggable candidate overexpressed in non-responder patients (ROC AUC = 0.699, FC = 1.8, P=1.1E-8). The baseline YAP1 expression correlated with worse progression-free survival (HR = 2.51, P=1.2E-6, FDR = 1%), and overall survival (HR = 2.15, P = 1.2E-5, FDR = 1%). In YUMM1.7, combination of Verteporfin plus anti-PD1 reduced tumour size more than anti-PD1 monotherapy (P=0.008), or control (P=0.021). There was no difference between the cohorts in B16-F10 inoculated mice. We found increased expression of YAP1 in YUMM1.7 mice compared to B16-F10. The combination therapy induced a more-immune-inflamed phenotype characterized by increased expression of T cell and M1 macrophage markers. CONCLUSIONS AND IMPLICATIONS: Verteporfin with anti-PD1 exhibited antitumor potential by promoting a pro-inflammatory tumour microenvironment in melanoma. We believe that YAP1 acts as a master regulator of anti-PD1 resistance.
  
  
  
  
**PTEN**
-
4
of
10
  

### Endometriosis and cancer risk.

4. Eur J Cancer Prev. 2025 May 1;34(3):276-278. doi: 10.1097/CEJ.0000000000000965 Epub 2025 Mar 12.
  
  
Bogani G(1), Chiappa V(1), Raspagliesi F(1), Corso G(2)(3).
  
  
Author information: (1)Gynecologic Oncologic Unit, Fondazione IRCCS Istituto Nazionale dei Tumori. ( ...
  
  
Endometriosis is one of the most common gynecological benign disease. Epidemiological evidence suggests a potential association between endometriosis and cancer risk. Accumulating evidence highlighted the risk of ovarian cancer, particularly endometrioid and clear cell subtypes. Few studies reported a modest correlation between endometriosis and various solid tumors including, endometrial cancer, and melanoma. However, inconsistencies remain across studies. Additionally, some data indicate an increased risk of melanoma, basal cell carcinoma, and non-Hodgkin lymphoma, underscoring the multifaceted nature of cancer risk in women with endometriosis. The underlying mechanisms include chronic inflammation, oxidative stress, hormonal dysregulation, and genetic alterations, such as mutations in ARID1A , PTEN , and KRAS , which contribute to the shared pathology between endometriosis and cancer. This paper explores the complex association between endometriosis and cancer, focusing on specific malignancies. This review emphasizes the importance of understanding the shared mechanisms between endometriosis and cancer. Although most women with endometriosis will not develop cancer, further research is essential to unravel the molecular pathways linking these conditions and enhance long-term outcomes for affected women.
  
  
  
  
**PTEN**
-
5
of
10
  

### A Clinical, Morphologic, and Molecular Comparison of Bonafide Spitz Melanomas and ASTs in the Pediatric Population.

5. Am J Surg Pathol. 2025 Apr 24. doi: 10.1097/PAS.0000000000002381 Online ahead of print.
  
  
Beydoun HM(1), Jeyakumar JE(1), Addo A(1), Olivares S(1), Zhao L(2), Ma Y(2), Ko J(3), Bahrami A(4), ...
  
  
Author information: (1)Department of Dermatology, Feinberg School of Medicine. (2)Department of Prev ...
  
  
Pediatric Spitz melanoma (SM) with bonafide metastatic disease is rare. In this study, we assembled the largest cohort to date of pediatric SM with a verified Spitz-associated genomic driver and clinical follow-up demonstrating bonafide metastasis. We compared the clinical, morphologic, and molecular features of these SMs to a control cohort of 57 pediatric atypical Spitz tumors (ASTs). Pediatric SM patients were significantly older than AST patients (12 vs 8 years of age). While not statistically significant, SMs were more likely to be heavily pigmented (5/7 SMs vs 11/57 ASTs), to have a sheet-like growth pattern (3/7 SMs vs 8/57 ASTs), and have severe nuclear atypia (6/7 SMs vs 20/57 ASTs). SMs had significantly greater mitotic activity (avg of 4.3/mm 2 in SMs and 2.7/mm 2 in ASTs, P =0.008) and more frequent larger cell size ( P =0.006). However, none of these features were specific and could also be seen in ASTs. The presence of homozygous deletions of 9p21 in conjunction with TERT promoter hot spot mutations or PTEN deletions (n=2), as well as MYC overexpression or amplification (n=2) were only seen in the SMs and none of the ASTs. These findings were mutually exclusive in the SM group and mutually exclusive with the presence of complex chromosomal copy number aberrations, which were seen in the remaining 3 pediatric SMs. This study demonstrates that there are multiple pathways to malignancy for pediatric SMs and none of our commonly used biomarkers have a particularly high sensitivity. Hence, the optimal distinction of pediatric SM from ASTs will continue to require the integration of clinical, histologic, and molecular data.
  
  
  
  
**PTEN**
-
6
of
10
  

### Extended spectrum of cancers in PTEN hamartoma tumor syndrome.

6. NPJ Precis Oncol. 2025 Mar 6;9(1):61. doi: 10.1038/s41698-025-00847-3
  
  
Yehia L(1), Plitt G(2)(3)(4), Tushar AM(2), Liu D(2)(4), Joo J(2)(4), Ni Y(5), Patil S(6), Eng C(2)( ...
  
  
Author information: (1)Genomic Medicine Institute, Lerner Research Institute, Cleveland Clinic, Clev ...
  
  
PTEN hamartoma tumor syndrome (PHTS) is associated with increased lifetime risks of breast, thyroid, kidney, endometrial, and colorectal cancers, as well as melanoma (collectively, component cancers). We sought to characterize non-component cancers (NCC) in PHTS. Of 701 research participants with PHTS, 340 (49%) had cancer, with 101 (30%) having at least one NCC. Interestingly, 71 (70%) of those with NCC had at least one other PHTS component malignancy. Patients with pathogenic PTEN variants showed higher risks for prostate cancer and soft tissue sarcomas at younger ages than the general population. A literature survey showed independent cases of NCC in PHTS, with PTEN-related molecular changes including second-hit somatic PTEN alterations in a subset of various specimens. We recommend increased awareness regarding NCC in individuals with PHTS, particularly increased risks for prostate cancer and sarcoma. Further studies are needed to define age-related penetrance and accordingly, the appropriate strategies for cancer risk management.
  
  
  
  
**PTEN**
-
7
of
10
  

### Tumorigenesis Driven by BRAFV600E Requires Secondary Mutations That Overcome Its Feedback Inhibition of RAC1 and Migration.

7. Cancer Res. 2025 May 2;85(9):1611-1627. doi: 10.1158/0008-5472.CAN-24-2220
  
  
Gadal S(1), Boyer JA(2), Roy SF(3)(4), Outmezguine NA(1), Sharma M(1), Li H(5), Fan N(6), Chan E(6), ...
  
  
Author information: (1)Molecular Pharmacology Program, Sloan Kettering Institute for Cancer Research ...
  
  
Update of bioRxiv. 2024 Apr 20:2023.11.21.568071. doi: 10.1101/2023.11.21.568071.
  
  
  
  
**PTEN**
-
8
of
10
  

### Considerations for hereditary breast and ovarian cancer syndrome molecular diagnosis: experience from the clinical practice.

8. Breast Cancer Res Treat. 2025 Apr;210(3):507-519. doi: 10.1007/s10549-025-07643-4 Epub 2025 Feb 24.
  
  
Potrony M(1)(2), Morales-Romero B(1)(2), Moreno L(3), Pastor B(3)(4)(5), Grau E(3), Badenas C(1)(2), ...
  
  
Author information: (1)Biochemistry and Molecular Genetics Department, Hospital Clínic de Barcelona, ...
  
  
PURPOSE: The implementation of the next-generation sequencing (NGS) in clinical practice has improved the genetic diagnosis of Hereditary Breast and Ovarian Cancer Syndrome (HBOC). We aimed to evaluate the diagnostic outcomes of using an NGS cancer gene panel in clinical practice for patients selected based on personal and/or family history of breast, ovarian, prostate, melanoma, and other HBOC-associated cancers. METHODS: The study series included 2561 consecutive Spanish individuals referred for genetic testing, comprising 2445 cancer patients and 116 healthy individuals with family history of HBOC. Eleven HBOC susceptibility genes (BRCA1, BRCA2, PALB2, ATM, CHEK2, BARD1, BRIP1, RAD51C, RAD51D, TP53, and PTEN) and three Lynch Syndrome genes (MLH1, MSH2, and MSH6) available for opportunistic testing were analyzed using a commercial Hereditary Cancer Panel and an in-house bioinformatics pipeline. RESULTS: Overall, the diagnostic yield was 11.0% in cancer patients and 8.6% in healthy individuals with a family history of breast/ovarian cancer. Pathogenic variants in high-risk genes were more frequent in patients with multiple HBOC tumors and a family history of different HBOC cancers. Additionally, we diagnosed five families with Lynch syndrome through opportunistic testing. CONCLUSION: Testing cancer susceptibility genes using an agnostic strategy confers a diagnostic benefit for hereditary cancer syndromes compared to phenotype-driven test, without adding complexity to the study. The analysis of healthy individuals with a family history of HBOC detects pathogenic variants in a cost-efficient percentage of cases, resulting in a good alternative strategy when the index case is unavailable.
  
  
  
  
**PTEN**
-
9
of
10
  

### Clinico-genomic features predict distinct metastatic phenotypes in cutaneous melanoma.

9. bioRxiv [Preprint]. 2025 Jan 27:2025.01.24.633441. doi: 10.1101/2025.01.24.633441
  
  
Aprati TJ(1)(2)(3), Day CP(4), Lee D(5), Pan A(5), Jee J(6), Tarantino G(1)(2)(3), Manos MP(1), Faul ...
  
  
Author information: (1)Department of Medical Oncology, Dana-Farber Cancer Institute, Boston, MA, USA ...
  
  
Metastasis drives mortality and morbidity in cancer. While some patients develop broad metastatic disease across multiple organs, others exhibit organ-specific spread. To identify mechanisms underlying metastatic organotropism, we analyzed clinico-genomic data from over 7,000 patients with metastatic cutaneous melanoma in three independent cohorts (one primary discovery and two validation cohorts including a nationwide electronic health record-derived deidentified database), leveraging machine learning approaches to clinical data. We found that female sex and increased tumor mutational burden associate with decreased metastatic potential, while older age associates with more lung and adrenal metastases. Using unsupervised analyses, patients clustered into five metastatic patterns: a "highly metastatic" cluster characterized by involvement of many organs, a "low metastatic" cluster characterized by few metastatic sites (mostly lymph node metastases), and three additional clusters each characterized by metastasis to specific sites (brain, lung, liver). Mutations in B2M and PTEN associated with increased overall metastatic potential. PTEN mutations were also associated with brain metastases but were enriched only in the "highly metastatic" cluster and not the brain-specific cluster. Mutations in GNAQ or GNA11 (GNA) associated with increased liver metastasis. To validate this association, we tested and demonstrated liver tropism in two GNA-mutant genetically engineered cutaneous melanoma mouse models of metastasis. Overall, our study elucidates distinct phenotypes of metastasis in patients with melanoma and identifies novel clinical and genomic associations that illuminate the clinical drivers of metastatic organotropism.
  
  
  
  
**PTEN**
-
10
of
10
  

### Coordinate autophagy and translation inhibition enhance cell death in melanoma.

10. Folia Med Cracov. 2024 Dec 26;64(3):17-35. doi: 10.24425/fmc.2024.152163
  
  
Gil D(1), Zarzycka M(2), Lekka M(3).
  
  
Author information: (1)Chair of Medical Biochemistry, Jagiellonian University Medical College, Krakó ...
  
  
Melanoma treatments are necessary when surgically curable treatments are limited. The major challenge of targeted therapy for treating malignant melanoma is acquired drug resistance. Translation and autophagy pathways are interconnected and involved in developing cancer drug resistance. We hypothesized that coordinate inhibition of autophagy and translation would lead to a better anticancer effect. In the present study, we used chloroquine combined with two translation inhibitors (NVP-BEZ235 and CGP57380) acting at different signaling pathway levels, activating the translation. Our study was conducted for human melanoma cell lines with similar genomic alteration (BRAFV600E and PTEN loss). The combination of the drugs suppresses cell invasiveness and growth by inducing apoptosis. We showed multiple direct and indirect interactions, indicating the overlap and interaction between the translation machinery and autophagy. These data suggest that coordinated inhibition of translation and autophagy promotes apoptosis and may be a new therapeutic model for melanoma treatment.
  
  
  
  
  

---

  

# RASA1

**Signature gene in:** CD4+ T cells, CD8+ T cells
  
**Differentially expressed in:** no cell type
  
  
**RASA1**
-
1
of
3
  

### Promoterless Transposon Mutagenesis Drives Solid Cancers via Tumor Suppressor Inactivation.

1. Cancers (Basel). 2021 Jan 9;13(2):225. doi: 10.3390/cancers13020225
  
  
Aiderus A(1), Contreras-Sandoval AM(1), Meshey AL(1), Newberg JY(1)(2), Ward JM(3), Swing DA(4), Cop ...
  
  
Author information: (1)Department of Molecular Oncology, Moffitt Cancer Center & Research Institute, ...
  
  
A central challenge in cancer genomics is the systematic identification of single and cooperating tumor suppressor gene mutations driving cellular transformation and tumor progression in the absence of oncogenic driver mutation(s). Multiple in vitro and in vivo gene inactivation screens have enhanced our understanding of the tumor suppressor gene landscape in various cancers. However, these studies are limited to single or combination gene effects, specific organs, or require sensitizing mutations. In this study, we developed and utilized a Sleeping Beauty transposon mutagenesis system that functions only as a gene trap to exclusively inactivate tumor suppressor genes. Using whole body transposon mobilization in wild type mice, we observed that cumulative gene inactivation can drive tumorigenesis of solid cancers. We provide a quantitative landscape of the tumor suppressor genes inactivated in these cancers and show that, despite the absence of oncogenic drivers, these genes converge on key biological pathways and processes associated with cancer hallmarks.
  
  
  
  
**RASA1**
-
2
of
3
  

### Inactivation of RASA1 promotes melanoma tumorigenesis via R-Ras activation.

2. Oncotarget. 2016 Apr 26;7(17):23885-96. doi: 10.18632/oncotarget.8127
  
  
Sung H(1)(2), Kanchi KL(3), Wang X(1)(2), Hill KS(1)(2), Messina JL(2)(4)(5), Lee JH(6), Kim Y(7), D ...
  
  
Author information: (1)Department of Molecular Oncology, Moffitt Cancer Center, Tampa, FL, USA. (2)C ...
  
  
Inactivation of Ras GTPase activating proteins (RasGAPs) can activate Ras, increasing the risk for tumor development. Utilizing a melanoma whole genome sequencing (WGS) data from 13 patients, we identified two novel, clustered somatic missense mutations (Y472H and L481F) in RASA1 (RAS p21 protein activator 1, also called p120RasGAP). We have shown that wild type RASA1, but not identified mutants, suppresses soft agar colony formation and tumor growth of BRAF mutated melanoma cell lines via its RasGAP activity toward R-Ras (related RAS viral (r-ras) oncogene homolog) isoform. Moreover, R-Ras increased and RASA1 suppressed Ral-A activation among Ras downstream effectors. In addition to mutations, loss of RASA1 expression was frequently observed in metastatic melanoma samples on melanoma tissue microarray (TMA) and a low level of RASA1 mRNA expression was associated with decreased overall survival in melanoma patients with BRAF mutations. Thus, these data support that RASA1 is inactivated by mutation or by suppressed expression in melanoma and that RASA1 plays a tumor suppressive role by inhibiting R-Ras, a previously less appreciated member of the Ras small GTPases.
  
  
  
  
**RASA1**
-
3
of
3
  

### miRTrail--a comprehensive webserver for analyzing gene and miRNA patterns to enhance the understanding of regulatory mechanisms in diseases.

3. BMC Bioinformatics. 2012 Feb 22;13:36. doi: 10.1186/1471-2105-13-36
  
  
Laczny C(1), Leidinger P, Haas J, Ludwig N, Backes C, Gerasch A, Kaufmann M, Vogel B, Katus HA, Mede ...
  
  
Author information: (1)Center for Bioinformatics, Saarland University, Campus E2 1, 66041 Saarbrücke ...
  
  
BACKGROUND: Expression profiling provides new insights into regulatory and metabolic processes and in particular into pathogenic mechanisms associated with diseases. Besides genes, non-coding transcripts as microRNAs (miRNAs) gained increasing relevance in the last decade. To understand the regulatory processes of miRNAs on genes, integrative computer-aided approaches are essential, especially in the light of complex human diseases as cancer. RESULTS: Here, we present miRTrail, an integrative tool that allows for performing comprehensive analyses of interactions of genes and miRNAs based on expression profiles. The integrated analysis of mRNA and miRNA data should generate more robust and reliable results on deregulated pathogenic processes and may also offer novel insights into the regulatory interactions between miRNAs and genes. Our web-server excels in carrying out gene sets analysis, analysis of miRNA sets as well as the combination of both in a systems biology approach. To this end, miRTrail integrates information on 20.000 genes, almost 1.000 miRNAs, and roughly 280.000 putative interactions, for Homo sapiens and accordingly for Mus musculus and Danio rerio. The well-established, classical Chi-squared test is one of the central techniques of our tool for the joint consideration of miRNAs and their targets. For interactively visualizing obtained results, it relies on the network analyzers and viewers BiNA or Cytoscape-web, also enabling direct access to relevant literature. We demonstrated the potential of miRTrail by applying our tool to mRNA and miRNA data of malignant melanoma. MiRTrail identified several deregulated miRNAs that target deregulated mRNAs including miRNAs hsa-miR-23b and hsa-miR-223, which target the highest numbers of deregulated mRNAs and regulate the pathway "basal cell carcinoma". In addition, both miRNAs target genes like PTCH1 and RASA1 that are involved in many oncogenic processes. CONCLUSIONS: The application on melanoma samples demonstrates that the miRTrail platform may open avenues for investigating the regulatory interactions between genes and miRNAs for a wide range of human diseases. Moreover, miRTrail cannot only be applied to microarray based expression profiles, but also to NGS-based transcriptomic data. The program is freely available as web-server at mirtrail.bioinf.uni-sb.de.
  
  
  
  
  

---

  

# RERE

**Signature gene in:** B cells
  
**Differentially expressed in:** no cell type
  
  
**RERE**
-
1
of
1
  

### Variant of TYR and autoimmunity susceptibility loci in generalized vitiligo.

1. N Engl J Med. 2010 May 6;362(18):1686-97. doi: 10.1056/NEJMoa0908547 Epub 2010 Apr 21.
  
  
Jin Y(1), Birlea SA, Fain PR, Gowan K, Riccardi SL, Holland PJ, Mailloux CM, Sufit AJ, Hutton SM, Am ...
  
  
Author information: (1)Human Medical Genetics Program, School of Medicine, University of Colorado, P ...
  
  
BACKGROUND: Generalized vitiligo is an autoimmune disease characterized by melanocyte loss, which results in patchy depigmentation of skin and hair, and is associated with an elevated risk of other autoimmune diseases. METHODS: To identify generalized vitiligo susceptibility loci, we conducted a genomewide association study. We genotyped 579,146 single-nucleotide polymorphisms (SNPs) in 1514 patients with generalized vitiligo who were of European-derived white (CEU) ancestry and compared the genotypes with publicly available control genotypes from 2813 CEU persons. We then tested 50 SNPs in two replication sets, one comprising 677 independent CEU patients and 1106 CEU controls and the other comprising 183 CEU simplex trios with generalized vitiligo and 332 CEU multiplex families. RESULTS: We detected significant associations between generalized vitiligo and SNPs at several loci previously associated with other autoimmune diseases. These included genes encoding major-histocompatibility-complex class I molecules (P=9.05x10(-23)) and class II molecules (P=4.50x10(-34)), PTPN22 (P=1.31x10(-7)), LPP (P=1.01x10(-11)), IL2RA (P=2.78x10(-9)), UBASH3A (P=1.26x10(-9)), and C1QTNF6 (P=2.21x10(-16)). We also detected associations between generalized vitiligo and SNPs in two additional immune-related loci, RERE (P=7.07x10(-15)) and GZMB (P=3.44x10(-8)), and in a locus containing TYR (P=1.60x10(-18)), encoding tyrosinase. CONCLUSIONS: We observed associations between generalized vitiligo and markers implicating multiple genes, some associated with other autoimmune diseases and one (TYR) that may mediate target-cell specificity and indicate a mutually exclusive relationship between susceptibility to vitiligo and susceptibility to melanoma.
  
  
  
  
  

---

  

# RUNX1

**Signature gene in:** CD4+ T cells, Dendritic cells, Plasma cells
  
**Differentially expressed in:** no cell type
  
  
**RUNX1**
-
1
of
10
  

### Combatting cellular immortality in cancers by targeting the shelterin protein complex.

1. Biol Direct. 2024 Nov 22;19(1):120. doi: 10.1186/s13062-024-00552-4
  
  
Chakraborty S(1), Banerjee S(2).
  
  
Author information: (1)Department of Biotechnology, School of Bioscience and Technology, Vellore Ins ...
  
  
Shelterin proteins (TERF1, TERF2, TPP1, TINF2, POT1) protect telomeres, prevent unwarranted repair activation, and regulate telomerase activity. Alterations in these proteins can lead to cancer progression. This study uses an in-silico approach to examine shelterin in tumour samples across various cancers, employing mutation plots, phylogenetic trees, and sequence alignments. Network pharmacology identified TERF1 as an essential shelterin protein and transcription factors RUNX1, CTCF, and KDM2B as potential biomarkers due to their interactions with miRNAs and shelterin proteins. We performed MCODE analysis to identify subnetworks of ncRNAs interacting with the shelterin proteins. Shelterin expression predicted patient survival in 24 cancer types, with TERF1, TERF2, TINF2, and POT1 significantly expressed in testicular, AML, prostate, breast and renal cancers, respectively, and TPP1 in AML and skin cancer. Spearman and Pearson's analyses showed significant correlations of TERF1 across cancers, with near-significant correlations for all five proteins in different cancer datasets like breast cancer, kidney renal papillary and lung squamous cell carcinoma, skin cutaneous melanoma, etc.,. Shelterin expression correlated with patient survival in breast, renal, lung, skin, uterine, and gastric cancers. Insights into TPP1-associated glycans highlighted glycosylated sites contributing to tumorigenesis. This study provides molecular signatures for further functional and therapeutic research on shelterin, highlighting its potential as a target for anti-cancer therapies and promising prospects for cancer prognosis and prediction.
  
  
  
  
**RUNX1**
-
2
of
10
  

### TIMP3 Gene Polymorphisms of -1296 T > C and -915 A > G Increase the Susceptibility to Arsenic-Induced Skin Cancer: A Cohort Study and In Silico Analysis of Mutation Impacts.

2. Int J Mol Sci. 2022 Nov 29;23(23):14980. doi: 10.3390/ijms232314980
  
  
Wu MM(1)(2)(3), Chen CW(4), Chen CY(2), Lee CH(5), Chou M(3), Hsu LI(6), Lee TC(7), Chen CJ(8).
  
  
Author information: (1)Department of Public Health, School of Medicine, College of Medicine, Taipei ...
  
  
Long-term exposure to arsenic may induce several human cancers, including non-melanoma skin cancer. The tissue inhibitor of metalloproteinase (TIMP)-3, encoded by the TIMP3 gene, may inhibit tumor growth, invasion, and metastasis of several cancer types. In this study, we aimed to investigate effects of the TIMP3 -1296 T > C (rs9619311) and -915 A > G (rs2234921) single-nucleotide polymorphisms (SNPs) on skin cancer risk in an arsenic-exposed population, and to evaluate the influence of allele-specific changes by an in silico analysis. In total, 1078 study participants were followed up for a median of 15 years for newly diagnosed skin cancer. New cases were identified through linkage to the National Cancer Registry of Taiwan. A Cox regression analysis was used to evaluate the effects of TIMP3 variants. Transcription factor (TF) profiling of binding sites of allele-specific changes in SNPs was conducted using the JASPAR scan tool. We observed borderline associations between TIMP3 genotypes and skin cancer risk. However, when combined with high arsenic exposure levels, the rs9619311 C allele, rs2234921 G allele, or C-G haplotype groups exhibited a greater risk of developing skin cancer compared to the respective common homozygous genotype group. The in silico analysis revealed several TF motifs located at or flanking the two SNP sites. We validated that the C allele of rs9619311 attenuated the binding affinity of BACH2, MEIS2, NFE2L2, and PBX2 to the TIMP3 promoter, and that the G allele of rs2234921 reduced the affinity of E2F8 and RUNX1 to bind to the promoter. Our findings suggest significant modifications of the effect of the association between arsenic exposure and skin cancer risk by the TIMP3 rs9619311 and rs2234921 variants. The predicted TFs and their differential binding affinities to the TIMP3 promoter provide insights into how TIMP3 interacts with arsenic through TFs in skin cancer formation.
  
  
  
  
**RUNX1**
-
3
of
10
  

### Pan-cancer Landscape of the RUNX Protein Family Reveals their Potential as Carcinogenic Biomarkers and the Mechanisms Underlying their Action.

3. J Transl Int Med. 2022 Jul 10;10(2):156-174. doi: 10.2478/jtim-2022-0013 eCollection 2022 Jun.
  
  
Pan S(1), Sun S(2), Liu B(3), Hou Y(1).
  
  
Author information: (1)Department of Radiology, Shengjing Hospital of China Medical University, Shen ...
  
  
BACKGROUND: The RUNX family of transcription factors plays an important regulatory role in tumor development. Although the importance of RUNX in certain cancer types is well known, the pan-cancer landscape remains unclear. MATERIALS AND METHODS: Data from The Cancer Genome Atlas (TCGA) provides a pan-cancer overview of the RUNX genes. Hence, herein, we performed a pan-cancer analysis of abnormal RUNX expression and deciphered the potential regulatory mechanism. Specifically, we used TCGA multi-omics data combined with multiple online tools to analyze transcripts, genetic alterations, DNA methylation, clinical prognoses, miRNA networks, and potential target genes. RESULTS: RUNX genes are consistently overexpressed in esophageal, gastric, pancreatic, and pan-renal cancers. The total protein expression of RUNX1 in lung adenocarcinoma, kidney renal clear cell carcinoma (KIRC), and uterine corpus endometrial carcinoma (UCEC) is consistent with the mRNA expression results. Moreover, increased phosphorylation on the T14 and T18 residues of RUNX1 may represent potential pathogenic factors. The RUNX genes are significantly associated with survival in pan-renal cancer, brain lower-grade glioma, and uveal melanoma. Meanwhile, various mutations and posttranscriptional changes, including the RUNX1 D96 mutation in invasive breast carcinoma, the co-occurrence of RUNX gene mutations in UCEC, and methylation changes in the RUNX2 promoter in KIRC, may be associated with cancer development. Finally, analysis of epigenetic regulator co-expression, miRNA networks, and target genes revealed the carcinogenicity, abnormal expression, and direct regulation of RUNX genes. CONCLUSIONS: We successfully analyzed the pan-cancer abnormal expression and prognostic value of RUNX genes, thereby providing potential biomarkers for various cancers. Further, mutations revealed via genetic alteration analysis may serve as a basis for personalized patient therapies.
  
  
  
  
**RUNX1**
-
4
of
10
  

### [Clinical features and prognosis of childhood B-lineage acute lymphoblastic leukemia expressing the PRAME gene].

4. Zhongguo Dang Dai Er Ke Za Zhi. 2022 May 15;24(5):543-549. doi: 10.7499/j.issn.1008-8830.2111108
  
  
[Article in Chinese; Abstract available in Chinese from the publisher]
  
  
Zhang F(1), Lu AD(1), Zuo YX(1), Ding MM(1), Jia YP(1), Zhang LP(1).
  
  
Author information: (1)Department of Pediatrics, People's Hospital, Peking University, Beijing 100044, China.
  
  
  
  
**RUNX1**
-
5
of
10
  

### Blue light induces ROS mediated apoptosis and degradation of AML1-ETO oncoprotein in Kasumi-1 cells.

5. Med Oncol. 2022 Feb 12;39(5):52. doi: 10.1007/s12032-022-01650-x
  
  
Zhuang J(1), Xia L(2), Zou Z(2), Yin J(3).
  
  
Author information: (1)Department of Clinical Pharmacology, Key Laboratory of Clinical Cancer Pharma ...
  
  
Light-emitting diode (LED)-based therapies, particularly blue LEDs with wavelengths of 400-500 nm, have shown beneficial results in several cancers, including melanoma, lymphoid cells, and skin tumors. In this study, the cell viability and apoptosis of Kasumi-1 cells treated by blue light (BL) irradiation have been explored. Firstly, BL can specially inhibit the proliferation and promote the apoptosis of Kasumi-1 cells. Furthermore, the apoptosis was triggered by the production of reactive oxygen species and the decline of mitochondrial membrane potential which was regulated by the ratio of Bcl-2(Bcl-xL)/Bax; BL caused the cells' final apoptosis accompanied with the increased cleavage of caspase-3 and poly-ADP-ribose polymerase. Finally, BL induced the degradation of AML1-ETO dependent on the activation of caspase-3. These results are helpful for establishing a low toxicity and high efficiency strategy of BL irradiation for clinical treatment of Kasumi-1 cells.
  
  
  
  
**RUNX1**
-
6
of
10
  

### Proteogenomics Reveals Perturbed Signaling Networks in Malignant Melanoma Cells Resistant to BRAF Inhibition.

6. Mol Cell Proteomics. 2021;20:100163. doi: 10.1016/j.mcpro.2021.100163 Epub 2021 Oct 19.
  
  
Schmitt M(1), Sinnberg T(2), Bratl K(1), Zittlau K(1), Garbe C(2), Macek B(3), Nalpas NC(4).
  
  
Author information: (1)Quantitative Proteomics, University of Tuebingen, Tuebingen, Germany. (2)Divi ...
  
  
Analysis of nucleotide variants is a cornerstone of cancer medicine. Although only 2% of the genomic sequence is protein coding, mutations occurring in these regions have the potential to influence protein structure or modification status and may have severe impact on disease aetiology. Proteogenomics enables the analysis of sample-specific nonsynonymous nucleotide variants with regard to their effect at the proteome and phosphoproteome levels. Here, we developed a proof-of-concept proteogenomics workflow and applied it to the malignant melanoma cell line A375. Initially, we studied the resistance to serine/threonine-protein kinase B-raf (BRAF) inhibitor (BRAFi) vemurafenib in A375 cells. This allowed identification of several oncogenic nonsynonymous nucleotide variants, including a gain-of-function variant on aurora kinase A (AURKA) at F31I. We also detected significant changes in abundance among (phospho)proteins, which led to reactivation of the MAPK signaling pathway in BRAFi-resistant A375 cells. Upon reconstruction of the multiomic integrated signaling networks, we predicted drug therapies with the potential to disrupt BRAFi resistance mechanism in A375 cells. Notably, we showed that AURKA inhibition is effective and specific against BRAFi-resistant A375 cells. Subsequently, we investigated amino acid variants that interfere with protein posttranslational modification (PTM) status and potentially influence A375 cell signaling irrespective of BRAFi resistance. Mass spectrometry (MS) measurements confirmed variant-driven PTM changes in 12 proteins. Among them was the runt-related transcription factor 1 (RUNX1) displaying a variant on a known phosphorylation site S(Ph)276L. We confirmed the loss of phosphorylation site by MS and demonstrated the impact of this variant on RUNX1 interactome.
  
  
  
  
**RUNX1**
-
7
of
10
  

### Neoantigens in Hematologic Malignancies.

7. Front Immunol. 2020 Feb 14;11:121. doi: 10.3389/fimmu.2020.00121 eCollection 2020.
  
  
Biernacki MA(1)(2), Bleakley M(1)(3).
  
  
Author information: (1)Clinical Research Division, Fred Hutchinson Cancer Research Center, Seattle, ...
  
  
T cell cancer neoantigens are created from peptides derived from cancer-specific aberrant proteins, such as mutated and fusion proteins, presented in complex with human leukocyte antigens on the cancer cell surface. Because expression of the aberrant target protein is exclusive to malignant cells, immunotherapy directed against neoantigens should avoid "on-target, off-tumor" toxicity. The efficacy of neoantigen vaccines in melanoma and glioblastoma and of adoptive transfer of neoantigen-specific T cells in epithelial tumors indicates that neoantigens are valid therapeutic targets. Improvements in sequencing technology and innovations in antigen discovery approaches have facilitated the identification of neoantigens. In comparison to many solid tumors, hematologic malignancies have few mutations and thus fewer potential neoantigens. Despite this, neoantigens have been identified in a wide variety of hematologic malignancies. These include mutated nucleophosmin1 and PML-RARA in acute myeloid leukemia, ETV6-RUNX1 fusions and other mutated proteins in acute lymphoblastic leukemia, BCR-ABL1 fusions in chronic myeloid leukemia, driver mutations in myeloproliferative neoplasms, immunoglobulins in lymphomas, and proteins derived from patient-specific mutations in chronic lymphoid leukemias. We will review advances in the field of neoantigen discovery, describe the spectrum of identified neoantigens in hematologic malignancies, and discuss the potential of these neoantigens for clinical translation.
  
  
  
  
**RUNX1**
-
8
of
10
  

### Analysis of the proteasome activity and the turnover of the serotonin receptor 2B (HTR2B) in human uveal melanoma.

8. Exp Eye Res. 2019 Jul;184:72-77. doi: 10.1016/j.exer.2019.04.013 Epub 2019 Apr 16.
  
  
Le-Bel G(1), Benhassine M(2), Landreville S(3), Guérin SL(4).
  
  
Author information: (1)CUO-Recherche, Médecine Régénératrice, Centre de Recherche FRQS du CHU de Qué ...
  
  
Uveal melanoma (UM), although a very rare disease, remains a particularly aggressive type of cancer as near 50% of the UM presenting patients will also develop liver metastases within 15 years from the initial diagnostic. One of the most reliable predictive markers of UM at risk of evolving toward the formation of liver lesions is an abnormally elevated level of expression of the transcript encoding the 5-Hydroxytryptamine (serotonin) receptor 2B (HTR2B). In our previous study, we demonstrated that transcription of the HTR2B gene was under the regulatory influences of two transcription factors (TFs), NFI and RUNX1. However, the action of these TFs was insufficient to explain the elevated level of the HTR2B protein in metastatic UM cells or the discrepancies we observed between its expression at the transcriptional and protein levels, therefore suggesting that additional post-translational modifications may also contribute to the altered expression of HTR2B in UM cells. In the present study, we investigated whether the turnover of HTR2B by the proteasome could account at least in part for its deregulated expression. Microarray analyses performed with UM cell lines derived from both non-metastatic and metastatic UM primary tumors revealed important alterations in the expression of some of the transcripts encoding both the E3 ubiquitin ligases and the various subunits of the proteasome, and these modifications were further exacerbated by cell passaging in culture. These alterations also correlated with significant changes in the enzymatic activity of the proteasome. However, the highest proteasome activity and amount of ubiquitinated HTR2B observed in the metastatic T142 cell line, as revealed by immunoprecipitation of ubiquitinated proteins and Western blotting using the HTR2B antibody, apparently had little impact on the total content of HTR2B protein. This contrasts with the near total disappearance of this receptor in the non-metastatic T108 cell line. Our study therefore suggests that the inability of the proteasome to degrade HTR2B in metastatic UM cells might rely on an increased stability of the ubiquitinated receptor in these cells.
  
  
  
  
**RUNX1**
-
9
of
10
  

### Transcription of the Human 5-Hydroxytryptamine Receptor 2B (HTR2B) Gene Is under the Regulatory Influence of the Transcription Factors NFI and RUNX1 in Human Uveal Melanoma.

9. Int J Mol Sci. 2018 Oct 21;19(10):3272. doi: 10.3390/ijms19103272
  
  
Benhassine M(1), Guérin SL(2)(3).
  
  
Author information: (1)Centre Universitaire d'Ophtalmologie-Recherche (CUO-Recherche), Axe médecine ...
  
  
Because it accounts for 70% of all eye cancers, uveal melanoma (UM) is therefore the most common primary ocular malignancy. In this study, we investigated the molecular mechanisms leading to the aberrant expression of the gene encoding the serotonin receptor 2B (HTR2B), one of the most discriminating among the candidates from the class II gene signature, in metastatic and non-metastatic UM cell lines. Transfection analyses revealed that the upstream regulatory region of the HTR2B gene contains a combination of alternative positive and negative regulatory elements functional in HTR2B- but not in HTR23B⁺ UM cells. We demonstrated that both the transcription factors nuclear factor I (NFI) and Runt-related transcription factor I (RUNX1) interact with regulatory elements from the HTR2B gene to either activate (NFI) or repress (RUNX1) HTR2B expression in UM cells. The results of this study will help understand better the molecular mechanisms accounting for the abnormal expression of the HTR2B gene in uveal melanoma.
  
  
  
  
**RUNX1**
-
10
of
10
  

### The RUNX1/IL-34/CSF-1R axis is an autocrinally regulated modulator of resistance to BRAF-V600E inhibition in melanoma.

10. JCI Insight. 2018 Jul 26;3(14):e120422. doi: 10.1172/jci.insight.120422 eCollection 2018 Jul 26.
  
  
Giricz O(1), Mo Y(1), Dahlman KB(2), Cotto-Rios XM(3), Vardabasso C(4), Nguyen H(5), Matusow B(5), B ...
  
  
Author information: (1)Department of Medicine, Albert Einstein College of Medicine, Montefiore Medic ...
  
  
Resistance to current therapies still impacts a significant number of melanoma patients and can be regulated by epigenetic alterations. Analysis of global cytosine methylation in a cohort of primary melanomas revealed a pattern of early demethylation associated with overexpression of oncogenic transcripts. Loss of methylation and associated overexpression of the CSF 1 receptor (CSF1R) was seen in a majority of tumors and was driven by an alternative, endogenous viral promoter in a subset of samples. CSF1R was particularly elevated in melanomas with BRAF and other MAPK activating mutations. Furthermore, rebound ERK activation after BRAF inhibition was associated with RUNX1-mediated further upregulation of CSF-1R and its ligand IL-34. Importantly, increased CSF-1R and IL-34 overexpression were detected in an independent cohort of resistant melanomas. Inhibition of CSF-1R kinase or decreased CSF-1R expression by RNAi reduced 3-D growth and invasiveness of melanoma cells. Coinhibition of CSF-1R and BRAF resulted in synergistic efficacy in vivo. To our knowledge, our data unveil a previously unknown role for the autocrine-regulated CSF-1R in BRAF V600E resistance and provide a preclinical rationale for targeting this pathway in melanoma.
  
  
  
  
  

---

  

# SETBP1

**Signature gene in:** B cells
  
**Differentially expressed in:** no cell type
  
  
**SETBP1**
-
1
of
1
  

### First-hit SETBP1 mutations cause a myeloproliferative disorder with bone marrow fibrosis.

1. Blood. 2024 Apr 4;143(14):1399-1413. doi: 10.1182/blood.2023021349
  
  
Crespiatico I(1), Zaghi M(2), Mastini C(1), D'Aliberti D(1), Mauri M(1), Mercado CM(1), Fontana D(1) ...
  
  
Author information: (1)Department of Medicine and Surgery, University of Milan-Bicocca, Monza, Italy ...
  
  
Erratum in Blood. 2024 Jun 27;143(26):2810. doi: 10.1182/blood.2024025290.
  
  
  
  
  

---

  

# SMARCA2

**Signature gene in:** CD4+ T cells
  
**Differentially expressed in:** no cell type
  
  
**SMARCA2**
-
1
of
10
  

### Discovery of FHD-286, a First-in-Class, Orally Bioavailable, Allosteric Dual Inhibitor of the Brahma Homologue (BRM) and Brahma-Related Gene 1 (BRG1) ATPase Activity for the Treatment of SWItch/Sucrose Non-Fermentable (SWI/SNF) Dependent Cancers.

1. J Med Chem. 2025 Jan 23;68(2):1772-1792. doi: 10.1021/acs.jmedchem.4c02535 Epub 2025 Jan 12.
  
  
Vaswani RG(1), Huang DS(1), Anthony N(1), Xu L(1), Centore R(1), Schiller S(1), Li Z(1), Fan H(1), S ...
  
  
Author information: (1)Foghorn Therapeutics, 500 Technology Square, Suite 700, Cambridge, Massachuse ...
  
  
BRM (SMARCA2) and BRG1 (SMARCA4) are mutually exclusive ATPase subunits of the mSWI/SNF (BAF) chromatin remodeling complex. BAF is an attractive therapeutic target because of its role in transcription, and mutations in the subunits of BAF are common in cancer and neurological disorders. Herein, we report the discovery of compound 1 (FHD-286) as a potent allosteric inhibitor of the dual ATPase subunits from a high-throughput screening hit with a BRM IC50 of ∼27 μM. FHD-286 is an orally bioavailable compound with antitumor activity in mouse xenograft models of uveal melanoma and acute myeloid leukemia and is being evaluated in Phase 1 clinical trials.
  
  
  
  
**SMARCA2**
-
2
of
10
  

### Malignant melanoma arising from giant congenital melanocytic nevus with SMARCA2 gene mutation.

2. Asian J Surg. 2024 Jul;47(7):3320-3321. doi: 10.1016/j.asjsur.2024.03.171 Epub 2024 Apr 10.
  
  
Xie Y(1), Zhou Z(1), Deng D(2), Liu Y(3).
  
  
Author information: (1)Department of Pathology, Shanghai Skin Disease Hospital, School of Medicine, ...
  
  
DOI: 10.1016/j.asjsur.2024.03.171 PMID: 38604858 [Indexed for MEDLINE]
  
  
  
  
**SMARCA2**
-
3
of
10
  

### Cytomorphology of metastatic colonic MXD4::NUTM1-rearranged sarcoma.

3. Diagn Cytopathol. 2024 Jun;52(6):342-349. doi: 10.1002/dc.25320 Epub 2024 Apr 9.
  
  
Wilkinson ZA(1), Policarpio-Nicolas MLC(1).
  
  
Author information: (1)Department of Pathology, Cleveland Clinic, Cleveland, Ohio, USA.
  
  
This report describes the cytologic features of a recently described MXD4::NUTM1-rearranged colonic sarcoma metastatic to the midclavicular soft tissue. Thirteen years ago, a 65-year-old woman presented with a cecal mass and subsequent liver mass. The cecal mass was diagnosed as malignant undifferentiated spindled and epithelioid neoplasm based on morphology and lack of tumor immunoreactivity with a panel of epithelial, smooth muscle, skeletal, melanoma, hematologic, and GIST markers. The liver mass showed morphologic and immunophenotypic similarity to the epithelioid component of the patient's cecal mass, albeit devoid of the spindled component. Fine needle aspiration of the midclavicular soft tissue mass showed singly scattered to clustered epithelioid to rhabdoid tumor cells with centrally to eccentrically located nuclei, prominent nucleoli, and moderate eosinophilic cytoplasm. Immunohistochemical stains performed on the concurrent biopsy showed the tumor cells were positive for NUT and negative for all other additional markers with retained normal expression of SMARCA2 and SMARCA4. Next-generation sequencing showed the presence of MXD4::NUTM1 gene fusion. Due to the identical cytomorphologic findings with the epithelioid component of the patient's prior cecal and liver masses, the tumor was deemed as consistent with a NUTM1-rearranged sarcoma. To our knowledge, this case represents the first reported cytologic features of a NUTM1-rearranged sarcoma on fine needle aspiration. Familiarity with the cytologic features, inclusion of this entity in the differential diagnosis of tumors with epithelioid and/or rhabdoid morphology, and performance of ancillary tests (immunohistochemistry and molecular) will be helpful in arriving at the right diagnosis.
  
  
  
  
**SMARCA2**
-
4
of
10
  

### Undifferentiated large cell/rhabdoid carcinoma presenting in the intestines of patients with concurrent or recent non-small cell lung cancer (NSCLC): clinicopathologic and molecular analysis of 14 cases indicates an unusual pattern of dedifferentiated metastases.

4. Virchows Arch. 2021 Jul;479(1):157-167. doi: 10.1007/s00428-021-03032-6 Epub 2021 Jan 27.
  
  
Agaimy A(1), Daum O(2), Michal M(2), Schmidt MW(3), Stoehr R(4), Hartmann A(4), Lauwers GY(5).
  
  
Author information: (1)Institute of Pathology, Friedrich-Alexander-University Erlangen-Nürnberg, Uni ...
  
  
Undifferentiated carcinoma metastatic to the bowel is uncommon in surgical pathology practice and might be confused with primary gastrointestinal carcinoma, melanoma, lymphoma, and others. We present 14 cases of uni- (n = 9) or multifocal (n = 5) undifferentiated large cell/rhabdoid carcinoma presenting in the bowel of patients with concurrent (n = 9) or recent (diagnosed 1 to 25 months earlier; median, 4) non-small cell lung cancer (NSCLC). Patients were 6 females and 8 males, aged 52 to 85 years. Primary NSCLC was verified histologically in 10 cases and by imaging in 4. The undifferentiated histology was present in the lung biopsy in 4/10 patients (as sole pattern in 3 and combined with adenocarcinoma in 1) and was limited to the intestinal metastases in the remainder. PDL1 was strongly expressed in 7/9 cases (CPS: 41 to 100). Loss of at least one SWI/SNF subunit was detected in 7/13 cases (54%). SMARCA2 loss (n = 6) was most frequent and was combined with SMARCA4 loss in one case. PBRM1 loss was observed in one tumor. Successful molecular testing of 11 cases revealed BRAF mutations in 4 (3 were non-V600E variants), KRAS mutations in 3, and wildtype in 4. None had EGFR mutations. Analysis of 4 paired samples revealed concordant KRAS (2) and BRAF (1) mutations or wildtype (1). Our study indicates that undifferentiated carcinoma within the intestines of patients with concurrent/recent NSCLC represents dedifferentiated metastasis from the NSCLC. Recognition of this unusual presentation is cardinal to avoid misdiagnosis with inappropriate therapeutic and prognostic implications.
  
  
  
  
**SMARCA2**
-
5
of
10
  

### Patient with anomalous skin pigmentation expands the phenotype of ARID2 loss-of-function disorder, a SWI/SNF-related intellectual disability.

5. Am J Med Genet A. 2019 May;179(5):808-812. doi: 10.1002/ajmg.a.61075 Epub 2019 Mar 5.
  
  
Khazanchi R(1), Ronspies CA(2), Smith SC(2), Starr LJ(2).
  
  
Author information: (1)College of Medicine, University of Nebraska Medical Center, Omaha, Nebraska. ...
  
  
ARID2 loss-of-function is associated with a rare genetic disorder characterized in 14 reported patients to date. ARID2 encodes a member of the SWItch/sucrose non-fermentable chromatin remodeling complex. Other genes encoding subunits of this complex, such as ARID1A, ARID1B, and SMARCA2, are mutated in association with Coffin-Siris syndrome (CSS) and Nicolaides Baraitser syndrome (NCBRS) phenotypes. Previously reported ARID2 mutations manifested clinically with a CSS-like phenotype including intellectual disability, coarsened facial features, fifth toenail hypoplasia, and other recognizable dysmorphisms. However, heterogeneity exists between previously reported patients with some patients showing more overlapping features with NCBRS. Herein, we present a patient with a novel disease-causing ARID2 loss-of-function mutation. His clinical features included intellectual disability, coarse and dysmorphic facial features, toenail hypoplasia, ADHD, short stature, and delayed development consistent with prior reports. Our patient also presented with previously unreported clinical findings including ophthalmologic involvement, persistent fetal fingertip and toetip pads, and diffuse hyperpigmentary and hypopigmentary changes sparing his face, palms, and soles. The anomalous skin findings are particularly of interest given prior literature outlining the role of ARID2 in melanocyte homeostasis and melanoma. This clinical report and review of the literature is further affirming of the characteristic symptoms and expands the phenotype of this newly described and rare syndrome.
  
  
  
  
**SMARCA2**
-
6
of
10
  

### Dedifferentiated liposarcoma composed predominantly of rhabdoid/epithelioid cells: a frequently misdiagnosed highly aggressive variant.

6. Hum Pathol. 2018 Jul;77:20-27. doi: 10.1016/j.humpath.2017.12.025 Epub 2018 Jan 5.
  
  
Agaimy A(1), Michal M(2), Hadravsky L(3), Michal M(4).
  
  
Author information: (1)Institute of Pathology, University Hospital, 91054 Erlangen, Germany. Electro ...
  
  
Dedifferentiated liposarcoma is one of the most common sarcoma types in adults with a predilection for the retroperitoneum. We have recently encountered 6 cases of DDL composed predominantly of rounded, rhabdoid or epithelioid cells mimicking rhabdoid melanoma, epithelioid rhabdomyosarcoma or undifferentiated carcinoma. Patients were 5 males and one female aged 64 to 81 years (median, 68). Tumors originated in the retroperitoneum (n=5; 3 in the psoas muscle) and deep soft tissue of the thigh (n=1). All 3 patients with follow-up died of metastatic disease within 4 to 8 months. Preoperative biopsy diagnoses never suggested dedifferentiated liposarcoma as a possibility; instead carcinoma, rhabdomyosarcoma and lymphoma were on top of suggestions. Five resected tumors were composed predominantly (70%-100%) of anaplastic rounded to oval rhabdoid cells with prominent central nucleoli and paranuclear rhabdoid inclusions. Bi- and multinucleation was a constant feature. The background stroma showed variable myxoid changes and minor mixed inflammatory cells. Two cases showed homologous dedifferentiation and another had sclerosing spindle cell nodule but a well-differentiated lipomatous component was not seen in any. One biopsied case showed solely monotonous small round blue cells with scattered rhabdoid cells. Immunohistochemistry showed expression of MDM2 (6/6), CDK4 (5/6), pancytokeratin AE/1AE3 (4/6) and diffusely desmin and myogenin (2/6). All cases showed high-level co-amplification of MDM2/CDK4 by in situ hybridization. The SWI/SNF complex components (SMARCB1, SMARCA2, SMARCA4, ARID1A and PBRM1) were intact in all cases. This highly aggressive liposarcoma variant needs to be distinguished from a variety of neoplasms including undifferentiated carcinoma, melanoma, lymphoma, rhabdomyosarcoma and others.
  
  
  
  
**SMARCA2**
-
7
of
10
  

### Modulation of Brahma expression by the mitogen-activated protein kinase/extracellular signal regulated kinase pathway is associated with changes in melanoma proliferation.

7. Arch Biochem Biophys. 2014 Dec 1;563:125-35. doi: 10.1016/j.abb.2014.07.004 Epub 2014 Jul 12.
  
  
Mehrotra A(1), Saladi SV(1), Trivedi AR(1), Aras S(1), Qi H(1), Jayanthy A(2), Setaluri V(2), de la ...
  
  
Author information: (1)University of Toledo College of Medicine, Department of Biochemistry and Canc ...
  
  
Brahma (BRM) and Brahma-related gene 1(BRG1) are catalytic subunits of SWItch/sucrose non-fermentable (SWI/SNF) chromatin remodeling complexes. BRM is epigenetically silenced in a wide-range of tumors. Mutations in the v-raf murine sarcoma viral oncogene homolog B1 (BRAF) gene occur frequently in melanoma and lead to constitutive activation of the mitogen-activated protein kinase (MAPK)/extracellular signal regulated kinase (ERK1/2) pathway. We tested the hypothesis that BRM expression is modulated by oncogenic BRAF and phosphorylation of ERK1/2 in melanocytes and melanoma cells. Expression of oncogenic BRAF in melanocytes and melanoma cells that are wild-type for BRAF decreased BRM expression and increased BRG1 expression. Inhibition of mitogen-activated protein/extracellular signal-regulated kinase kinase (MEK) or selective inhibition of BRAF in melanoma cells that harbor oncogenic BRAF increased BRM expression and decreased BRG1 expression. Increased BRM expression was associated with increased histone acetylation on the BRM promoter. Over-expression of BRM in melanoma cells that harbor oncogenic BRAF promoted changes in cell cycle progression and apoptosis consistent with a tumor suppressive role. Upon inhibition of BRAF(V600E) with PLX4032, BRM promoted survival. PLX4032 induced changes in BRM function were correlated with increased acetylation of the BRM protein. This study provides insights into the epigenetic consequences of inhibiting oncogenic BRAF in melanoma through modulation of SWI/SNF subunit expression and function.
  
  
  
  
**SMARCA2**
-
8
of
10
  

### Inactivating mutations in SWI/SNF chromatin remodeling genes in human cancer.

8. Jpn J Clin Oncol. 2013 Sep;43(9):849-55. doi: 10.1093/jjco/hyt101 Epub 2013 Jul 30.
  
  
Oike T(1), Ogiwara H, Nakano T, Yokota J, Kohno T.
  
  
Author information: (1)Division of Genome Biology, National Cancer Center Research Institute, 1-1, T ...
  
  
Chromosomal deoxyribonucleic acid and histone proteins form a highly condensed structure known as chromatin. Chromatin remodeling proteins regulate deoxyribonucleic acid transcription, synthesis and repair by changing nucleosomal composition in an adenosine triphosphate-dependent manner and mediate access of deoxyribonucleic acid-binding proteins to deoxyribonucleic acid double strands. Recently, large-scale genome sequencing studies identified somatic mutations in genes encoding chromatin remodeling proteins in a variety of human solid cancers. Notably, inactivating mutations in genes encoding the catalytic and regulatory subunits of the switch/sucrose non-fermenting chromatin remodeling complex have been detected in several solid cancers: sucrose non-fermenting/switch/sucrose non-fermenting-related, matrix-associated, actin-dependent regulator of chromatin, subfamily b, member 1/Brahma-related gene 1-associated factor 47/integrase interactor 1 mutations in rhabdoid tumors; AT-rich interactive domain-containing protein 1 A/Brahma-related gene 1-associated factor 250a mutations in ovarian clear cell carcinoma, hepatocellular carcinoma and gastric adenocarcinoma; polybromo 1/Brahma-related gene 1-associated factor 180 mutations in renal clear cell carcinoma; Brahma-related gene 1/switch/sucrose non-fermenting-related, matrix-associated, actin-dependent regulator of chromatin, subfamily a, member 4 mutations in non-small-cell lung carcinoma and AT-rich interactive domain-containing protein 2/Brahma-related gene 1-associated factor 200 mutations in hepatocellular carcinoma and malignant melanoma. This suggests that the switch/sucrose non-fermenting complex has a tumor-suppressive function, and that switch/sucrose non-fermenting gene deficiencies may affect the properties of cancer cells, which could be of value for the development of novel therapeutic strategies.
  
  
  
  
**SMARCA2**
-
9
of
10
  

### MITF-independent pro-survival role of BRG1-containing SWI/SNF complex in melanoma cells.

9. PLoS One. 2013;8(1):e54110. doi: 10.1371/journal.pone.0054110 Epub 2013 Jan 17.
  
  
Ondrušová L(1), Vachtenheim J, Réda J, Záková P, Benková K.
  
  
Author information: (1)Laboratory of Transcription and Cell Signaling, Institute of Medical Biochemi ...
  
  
Metastasized malignant melanoma has a poor prognosis because of its intrinsic resistance to chemotherapy and radiotherapy. The central role in the melanoma transcriptional network has the transcription factor MITF (microphthalmia-associated transcription factor). It has been shown recently that the expression of MITF and some of its target genes require the SWI/SNF chromatin remodeling complex. Here we demonstrate that survival of melanoma cells requires functional SWI/SNF complex not only by supporting expression of MITF and its targets and but also by activating expression of prosurvival proteins not directly regulated by MITF. Microarray analysis revealed that besides the MITF-driven genes, expression of proteins like osteopontin, IGF1, TGFß2 and survivin, the factors known to be generally associated with progression of tumors and the antiapoptotic properties, were reduced in acute BRG1-depleted 501mel cells. Western blots and RT-PCR confirmed the microarray findings. These proteins have been verified to be expressed independently of MITF, because MITF depletion did not impair their expression. Because these genes are not regulated by MITF, the data suggests that loss of BRG1-based SWI/SNF complexes negatively affects survival pathways beyond the MITF cascade. Immunohistochemistry showed high expression of both BRM and BRG1 in primary melanomas. Exogenous CDK2, osteopontin, or IGF1 each alone partly relieved the block of proliferation imposed by BRG1 depletion, implicating that more factors, besides the MITF target genes, are involved in melanoma cell survival. Together these results demonstrate an essential role of SWI/SNF for the expression of MITF-dependent and MITF-independent prosurvival factors in melanoma cells and suggest that SWI/SNF may be a potential and effective target in melanoma therapy.
  
  
  
  
**SMARCA2**
-
10
of
10
  

### SWI/SNF chromatin remodeling complex is critical for the expression of microphthalmia-associated transcription factor in melanoma cells.

10. Biochem Biophys Res Commun. 2010 Feb 12;392(3):454-9. doi: 10.1016/j.bbrc.2010.01.048 Epub 2010 Jan 18.
  
  
Vachtenheim J(1), Ondrusová L, Borovanský J.
  
  
Author information: (1)Laboratory of Molecular Biology, University Hospital, Charles University, Pra ...
  
  
Erratum in Biochem Biophys Res Commun. 2010 May 14;395(4):585.
  
  
  
  
  

---

  

# TGFBR2

**Signature gene in:** B cells
  
**Differentially expressed in:** no cell type
  
  
**TGFBR2**
-
1
of
10
  

### Discovery of highly potent and ALK2/ALK1 selective kinase inhibitors using DNA-encoded chemistry technology.

1. Proc Natl Acad Sci U S A. 2024 Nov 19;121(47):e2413108121. doi: 10.1073/pnas.2413108121 Epub 2024 Nov 14.
  
  
Jimmidi R(#)(1)(2), Monsivais D(#)(1)(2), Ta HM(#)(1)(2), Sharma KL(1)(2), Bohren KM(1)(2), Chamakur ...
  
  
Author information: (1)Center for Drug Discovery, Baylor College of Medicine, Houston, TX 77030. (2) ...
  
  
Activin receptor type 1 (ACVR1; ALK2) and activin receptor like type 1 (ACVRL1; ALK1) are transforming growth factor beta family receptors that integrate extracellular signals of bone morphogenic proteins (BMPs) and activins into Mothers Against Decapentaplegic homolog 1/5 (SMAD1/SMAD5) signaling complexes. Several activating mutations in ALK2 are implicated in fibrodysplasia ossificans progressiva (FOP), diffuse intrinsic pontine gliomas, and ependymomas. The ALK2 R206H mutation is also present in a subset of endometrial tumors, melanomas, non-small lung cancers, and colorectal cancers, and ALK2 expression is elevated in pancreatic cancer. Using DNA-encoded chemistry technology, we screened 3.94 billion unique compounds from our diverse DNA-encoded chemical libraries (DECLs) against the kinase domain of ALK2. Off-DNA synthesis of DECL hits and biochemical validation revealed nanomolar potent ALK2 inhibitors. Further structure-activity relationship studies yielded center for drug discovery (CDD)-2789, a potent [NanoBRET (NB) cell IC50: 0.54 μM] and metabolically stable analog with good pharmacological profile. Crystal structures of ALK2 bound with CDD-2281, CDD-2282, or CDD-2789 show that these inhibitors bind the active site through Van der Waals interactions and solvent-mediated hydrogen bonds. CDD-2789 exhibits high selectivity toward ALK2/ALK1 in KINOMEscan analysis and NB K192 assay. In cell-based studies, ALK2 inhibitors effectively attenuated activin A and BMP-induced Phosphorylated SMAD1/5 activation in fibroblasts from individuals with FOP in a dose-dependent manner. Thus, CDD-2789 is a valuable tool compound for further investigation of the biological functions of ALK2 and ALK1 and the therapeutic potential of specific inhibition of ALK2.
  
  
  
  
**TGFBR2**
-
2
of
10
  

### Neuropeptide Precursor VGF Promotes Liver Metastatic Colonization of Gαq Mutant Uveal Melanoma by Facilitating Tumor Microenvironment via Paracrine Loops.

2. Adv Sci (Weinh). 2024 Dec;11(46):e2407967. doi: 10.1002/advs.202407967 Epub 2024 Oct 18.
  
  
Ouyang S(1), Shi S(1), Ding W(1), Ge Y(1), Su Y(2), Mo J(1), Peng K(1), Zhang Q(1), Liu G(2), Xiao W ...
  
  
Author information: (1)National-Local Joint Engineering Laboratory of Druggability and New Drug Eval ...
  
  
Uveal melanoma (UM), the predominant primary ocular malignancy, often progresses to liver metastasis with limited therapeutic options. The interplay of the tumor microenvironment, encompassing secreted soluble factors, plays a crucial role in facilitating liver metastasis. In this study, the role is elucidated of the neural growth factor-inducible gene (VGF), a secreted neuropeptide precursor, in Gαq mutant UM. Employing a multiomics approach, encompassing transcriptomic and secretomic analyses, the intricate involvement of VGF in UM progression is unveiled. VGF is upregulated in Gαq mutant UM cells and associated with poor prognosis of UM patients. Targeting VGF significantly suppressed the growth of UM in vitro and in vivo. Further evidence shows that VGF is regulated by Gαq through MAPK/CREB pathway. Mechanistically, CREB modulates VGF expression by directly binding to consensus DNA response elements in the promoters of the VGF gene. Combined inhibition of Gαq and MEK remarkably reduces tumor burden in the UM xenograft model. Notably, VGF triggers liver metastatic colonization of UM and activates the fibrosis of hepatic stellate cells (HSCs), creating a favorable microenvironment, through an autocrine and paracrine loop. Furthermore, VGF directly binds to TGFBR2 and regulates TGF-β-SMAD signaling pathway, thereby regulating genes associated with endothelial-mesenchymal transition (EMT) to promote metastasis. Taken together, these findings identify VGF as a pivotal driver in the progression and metastasis of Gαq mutant UM and confers a promising therapeutic target and strategy for UM patients.
  
  
  
  
**TGFBR2**
-
3
of
10
  

### Asiaticoside modulates human NK cell functional fate by mediating metabolic flexibility in the tumor microenvironment.

3. Phytomedicine. 2024 Oct;133:155921. doi: 10.1016/j.phymed.2024.155921 Epub 2024 Aug 6.
  
  
Guo Y(1), Xu J(2), Jia Y(3), Tian Y(4), Zhang Y(2), Zhang J(5), Wang Y(6), Chen L(7).
  
  
Author information: (1)Cancer Institute, First Hospital of Jilin University, Changchun, 130012, PR C ...
  
  
BACKGROUND: Transforming growth factor-beta (TGF-β), an immunosuppressive cytokine, is often elevated in various tumors and inhibits the immune system's ability to combat tumor cells. Despite promising results from TGF-β inhibitor therapies, their clinical efficacy remains limited. PURPOSE: This study aimed to enhance the antitumor capabilities of natural killer (NK) cells in the presence of TGF-β by exploring the potential of asiaticoside, a natural compound with established clinical safety. STUDY DESIGN: The effects of asiaticoside on NK cells were investigated to determine its potential to counteract TGF-β-induced immunosuppression and elucidate the underlying mechanisms. METHODS: Natural compounds were screened using a Luminex assay to identify those promoting Interferon-γ (IFN-γ) secretion from NK cells. Asiaticoside-pretreated NK cells' cytotoxicity was assessed against K562, OVCAR8, and A2780 cells using organoids from ascites-derived ovarian cancer (OC) cells. In vivo efficacy was evaluated with B16 melanoma lung metastasis and subcutaneous tumor models in C57BL/6 mice, using asiaticoside as a 50 mg/kg injection. The compound's ability to enhance NK cell-driven anti-neoplastic responses was further assessed in an OC murine model. Effects on TGF-β/SMAD pathways and mitochondrial functions were examined through various microscopy and metabolomic techniques. The involvement of the mTOR/DRP1 axis in asiaticoside-mediated restoration of mitochondrial oxidation in NK cells after TGF-β suppression was determined using the mTOR inhibitor rapamycin and the DRP1 inhibitor Mdivi-1. RESULTS: Asiaticoside-treated NK cells retained their ability to suppress tumor growth and metastasis despite TGF-β presence. Asiaticoside downregulated TGF-β receptors 1 (TGFBR1) expression, impaired the protein stability of TGFBR1 and TGF-β receptors 2 (TGFBR2), and reduced SMAD2 phosphorylation, preventing SMAD2 translocation from the mitochondria. This preserved mitochondrial respiration and maintained NK cell antitumor activity. CONCLUSION: The study concludes that asiaticoside has significant potential as a strategy for "priming" NK cells in cellular immunotherapy. By demonstrating that asiaticoside degrades the TGF-β receptor, leading to reduced phosphorylation of SMAD2 and preventing its mitochondrial translocation, thereby maintaining mitochondrial integrity. Meantime, asiaticoside counteracts TGF-β-induced suppression of mitochondrial oxidative and aerobic respiration through the mTOR/DRP1 pathways. The research uncovers a previously unreported pathway for preserving mitochondrial respiration and NK cell functionality. A detailed mechanistic insight into how asiaticoside functions at the molecular level was explored. Its ability to counteract the immunosuppressive effects of TGF-β makes it a valuable candidate for enhancing the effectiveness of immunotherapies in treating a variety of tumors with elevated TGF-β levels.
  
  
  
  
**TGFBR2**
-
4
of
10
  

### Localized in vivo gene editing of murine cancer-associated fibroblasts.

4. bioRxiv [Preprint]. 2024 Jul 16:2024.07.11.603114. doi: 10.1101/2024.07.11.603114
  
  
Kuhn NF(1)(2), Zaleta-Linares I(1)(2), Nyberg WA(3), Eyquem J(2)(3), Krummel MF(1)(2).
  
  
Author information: (1)Department of Pathology, University of California, San Francisco, CA 94143, U ...
  
  
Discovering the role of fibroblasts residing in the tumor microenvironment (TME) requires controlled, localized perturbations because fibroblasts play critical roles in regulating immunity and tumor biology at multiple sites. Systemic perturbations can lead to unintended, confounding secondary effects, and methods to locally genetically engineer fibroblasts are lacking. To specifically investigate murine stromal cell perturbations restricted to the TME, we developed an adeno-associated virus (AAV)-based method to target any gene-of-interest in fibroblasts at high efficiency (>80%). As proof of concept, we generated single (sKO) and double gene KOs (dKO) of Osmr, TGFBR2, and Il1r1 in cancer-associated fibroblasts (CAFs) and investigated how their cell states and those of other cells of the TME subsequently change in mouse models of melanoma and pancreatic ductal adenocarcinoma (PDAC). Furthermore, we developed an in vivo knockin-knockout (KIKO) strategy to achieve long-term tracking of CAFs with target gene KO via knocked-in reporter gene expression. This validated in vivo gene editing toolbox is fast, affordable, and modular, and thus holds great potential for further exploration of gene function in stromal cells residing in tumors and beyond.
  
  
  
  
**TGFBR2**
-
5
of
10
  

### CRISPR-mediated TGFBR2 knockout renders human ovarian cancer tumor-infiltrating lymphocytes resistant to TGF-β signaling.

5. J Immunother Cancer. 2022 Jul;10(7):e003750. doi: 10.1136/jitc-2021-003750
  
  
Fix SM(1), Forget MA(1), Sakellariou-Thompson D(1), Wang Y(1), Griffiths TM(2), Lee M(3), Haymaker C ...
  
  
Author information: (1)Melanoma Medical Oncology, The University of Texas MD Anderson Cancer Center, ...
  
  
BACKGROUND: The correlation between elevated T-cell infiltration and improved survival of ovarian cancer (OvCa) patients suggests that endogenous tumor-infiltrating lymphocytes (TIL) possess some degree of antitumor activity that can be harnessed for OvCa immunotherapy. We previously optimized a protocol for ex vivo OvCa TIL expansion for adoptive cell therapy, which is now being tested in a clinical trial at our institution (NCT03610490). Building on this success, we embarked on genetic modification of OvCa TIL to overcome key immunosuppressive factors present in the tumor microenvironment. Here, we present the preclinical optimization of CRISPR/Cas9-mediated knockout of the TGF-β receptor 2 (TGFBR2) in patient-derived OvCa TIL. METHODS: OvCa TILs were generated from four patients' tumor samples obtained at surgical resection and subjected to CRISPR/Cas9-mediated knockout of TGFBR2 before undergoing a rapid expansion protocol. TGFBR2-directed gRNAs were comprehensively evaluated for their TGFBR2 knockout efficiency and off-target activity. Furthermore, the impact of TGFBR2 knockout on TIL expansion, function, and downstream signaling was assayed. RESULTS: TGFBR2 knockout efficiencies ranging from 59±6% to 100%±0% were achieved using 5 gRNAs tested in four independent OvCa TIL samples. TGFBR2 knockout TIL were resistant to immunosuppressive TGF-β signaling as evidenced by a lack of SMAD phosphorylation, a lack of global transcriptional changes in response to TGF-β stimulation, equally strong secretion of proinflammatory cytokines in the presence and absence of TGF-β, and improved cytotoxicity in the presence of TGF-β. CRISPR-modification itself did not alter the ex vivo expansion efficiency, immunophenotype, nor the TCR clonal diversity of OvCa TIL. Importantly for clinical translation, comprehensive analysis of CRISPR off-target effects revealed no evidence of off-target activity for our top two TGFBR2-targeting gRNAs. CONCLUSIONS: CRISPR/Cas9-mediated gene knockout is feasible and efficient in patient-derived OvCa TIL using clinically-scalable methods. We achieved efficient and specific TGFBR2 knockout, yielding an expanded OvCa TIL product that was resistant to the immunosuppressive effects of TGF-β. This study lays the groundwork for clinical translation of CRISPR-modified TIL, providing opportunities for engineering more potent TIL therapies not only for OvCa treatment, but for the treatment of other solid cancers as well.
  
  
  
  
**TGFBR2**
-
6
of
10
  

### Tumor in the Crossfire: Inhibiting TGF-β to Enhance Cancer Immunotherapy.

6. BioDrugs. 2022 Mar;36(2):153-180. doi: 10.1007/s40259-022-00521-1 Epub 2022 Mar 30.
  
  
Tschernia NP(1), Gulley JL(2).
  
  
Author information: (1)Genitourinary Malignancies Branch, Medical Oncology Service, National Cancer ...
  
  
Cancer immunotherapy using monoclonal antibodies targeting immune checkpoints has undoubtedly revolutionized the cancer treatment landscape in the last decade. Immune checkpoint inhibitors can elicit long-lasting, previously unheard-of responses in a number of tumor entities. Yet, even in such tumors as metastatic melanoma and non-small cell-lung cancer, in which immune checkpoint inhibition has become the first-line treatment of choice, only a minority of patients will benefit considerably from these treatments. This has been attributed to a number of factors, including an immune-suppressive tumor microenvironment (TME). Using different modalities to break these barriers is of utmost importance to expand the population of patients that benefit from immune checkpoint inhibition. The multifunctional cytokine transforming growth factor-β (TGF-β) has long been recognized as an immune-suppressive factor in the TME. A considerable number of drugs have been developed to target TGF-β, yet most of these have since been discontinued. The combination of anti-TGF-β agents with immune checkpoint inhibitors now has the potential to revive this target as a viable immunomodulatory therapeutic approach. Currently, a limited number of small molecular inhibitor and monoclonal antibody candidates that target TGF-β are in clinical development in combination with the following immune checkpoint inhibitors: SRK 181, an antibody inhibiting the activation of latent TGF-β1; NIS 793, a monoclonal antibody targeting TGF-β; and SHR 1701, a fusion protein consisting of an anti-PD-L1 monoclonal antibody fused with the extracellular domain of human TGF-β receptor II. Several small molecular inhibitors are also in development and are briefly reviewed: LY364947, a pyrazole-based small molecular inhibitor of the serine-threonine kinase activity of TGFβRI; SB-431542, an inhibitor targeting several TGF-β superfamily Type I activin receptor-like kinases as well as TGF-β1-induced nuclear Smad3 localization; and galunisertib, an oral small molecular inhibitor of the TGFβRI kinase. One of the most advanced agents in this area is bintrafusp alfa, a bifunctional fusion protein composed of the extracellular domain of TGF-β receptor II fused to a human IgG1 mAb blocking PD-L1. Bintrafusp alfa is currently in advanced clinical development and as an agent in this space with the most clinical experience, is a focused highlight of this review.
  
  
  
  
**TGFBR2**
-
7
of
10
  

### Engineering Cancer Antigen-Specific T Cells to Overcome the Immunosuppressive Effects of TGF-β.

7. J Immunol. 2022 Jan 1;208(1):169-180. doi: 10.4049/jimmunol.2001357 Epub 2021 Dec 1.
  
  
Silk JD(1), Abbott RJM(2), Adams KJ(2), Bennett AD(2), Brett S(3), Cornforth TV(2), Crossland KL(2), ...
  
  
Author information: (1)Adaptimmune Ltd., Milton Park, Abingdon, United Kingdom; jonathan.silk@enarab ...
  
  
Adoptive T cell therapy with T cells expressing affinity-enhanced TCRs has shown promising results in phase 1/2 clinical trials for solid and hematological tumors. However, depth and durability of responses to adoptive T cell therapy can suffer from an inhibitory tumor microenvironment. A common immune-suppressive agent is TGF-β, which is secreted by tumor cells and cells recruited to the tumor. We investigated whether human T cells could be engineered to be resistant to inhibition by TGF-β. Truncating the intracellular signaling domain from TGF-β receptor (TGFβR) II produces a dominant-negative receptor (dnTGFβRII) that dimerizes with endogenous TGFβRI to form a receptor that can bind TGF-β but cannot signal. We previously generated specific peptide enhanced affinity receptor TCRs recognizing the HLA-A\*02-restricted peptides New York esophageal squamous cell carcinoma 1 (NY-ESO-1)157-165/l-Ag family member-1A (TCR: GSK3377794, formerly NY-ESO-1c259) and melanoma Ag gene A10254-262 (TCR: ADP-A2M10, formerly melanoma Ag gene A10c796). In this article, we show that exogenous TGF-β inhibited in vitro proliferation and effector functions of human T cells expressing these first-generation high-affinity TCRs, whereas inhibition was reduced or abolished in the case of second-generation TCRs coexpressed with dnTGFβRII (e.g., GSK3845097). TGF-β isoforms and a panel of TGF-β-associated genes are overexpressed in a range of cancer indications in which NY-ESO-1 is commonly expressed, particularly in synovial sarcoma. As an example, immunohistochemistry/RNAscope identified TGF-β-positive cells close to T cells in tumor nests and stroma, which had low frequencies of cells expressing IFN-γ in a non-small cell lung cancer setting. Coexpression of dnTGFβRII may therefore improve the efficacy of TCR-transduced T cells.
  
  
  
  
**TGFBR2**
-
8
of
10
  

### Berberine Suppresses EMT in Liver and Gastric Carcinoma Cells through Combination with TGFβR Regulating TGF-β/Smad Pathway.

8. Oxid Med Cell Longev. 2021 Oct 18;2021:2337818. doi: 10.1155/2021/2337818 eCollection 2021.
  
  
Du H(1)(2), Gu J(1)(2), Peng Q(1)(2), Wang X(1)(2), Liu L(1)(2), Shu X(1)(2), He Q(1)(2), Tan Y(1)(2 ...
  
  
Author information: (1)Department of Biochemistry and Molecular Biology School of Basic Medical Scie ...
  
  
Berberine (BBR), a natural alkaloid derived from Coptis, has anticancer activity. Some researchers have found that it could restrain epithelial-mesenchymal transition (EMT) of melanoma, neuroblastoma, and other tumor cells. However, it is unclear whether BBR can reverse EMT in hepatocellular carcinoma (HCC) and gastric carcinoma (GC). In our study, BBR inhibited the migration and invasion of HepG2, MGC803, and SGC7901 cells in a dose-dependent manner. Transcription sequencing assays showed that Vimentin, MMP, and Smad3 were downregulated, but Smad2, Smad6, TAB2, ZO-1, and claudin 7 were upregulated when treated with BBR. GO Enrichment analysis of KEGG pathway showed that BBR significantly inhibited TGF-β/Smad at 12 h, then, PI3K/Akt and Wnt/β-catenin signaling pathways at 24 h, which were closely related to the proliferation, migration, and EMT. The results of the transcriptome sequencing analysis were verified by Western Blot. It showed that the expression of epithelial marker E-cadherin and ZO-1 remarkably augmented with BBR treatment, as well as declined mesenchymal markers, including N-cadherin and Vimentin, decreased transcription factor Snail and Slug. The effects of BBR were similar to those of the PI3K inhibitor LY294002 and TGF-β receptor inhibitor SB431542. Furthermore, β-catenin and phosphorylation of AKT, Smad2, and Smad3 were changed dose-dependently by BBR treatment, which upregulated p-Smad2 and downregulated the others. Combined with LY or SB, respectively, BBR could enhance the effects of the two inhibitors. Simultaneously, IGF-1 and TGF-β, which is the activator of PI3K/AKT and TGF-β/Smad, respectively, could reverse the anti-EMT effect of BBR. The Molecular Docking results showed BBR had a high affinity with the TGF-β receptor I (TGFβR1), and the binding energy was -7.5 kcal/mol, which is better than the original ligand of TGFβR1. Although the affinity of BBR with TGF-β receptor II (TGFβR2) was lower than the original ligand of TGFβR2, the more considerable negative binding energy (-8.54 kcal/mol) was obtained. BBR upregulated p-Smad2, which was different from other reports, indicating that the function of Smad2 was relatively complex. Combination BBR with SB could enhance the effect of the inhibitor on EMT, and the results indicated that BBR binding to TGFβR was not competitive with SB to TGFβR since different binding amino acid sites. Our experiments demonstrated BBR increased p-Smad2 and decreased p-Smad3 by binding to TGFβR1 and TGβFR2 inhibiting TGF-β/Smad, then, PI3K/AKT and other signaling pathways to restrain EMT, metastasis, and invasion in tumor cells. The effect of BBR was similar on the three tumor cells.
  
  
  
  
**TGFBR2**
-
9
of
10
  

### Progression of melanoma is suppressed by targeting all transforming growth factor‑β isoforms with an Fc chimeric receptor.

9. Oncol Rep. 2021 Sep;46(3):197. doi: 10.3892/or.2021.8148 Epub 2021 Jul 23.
  
  
Kodama S(1), Podyma-Inoue KΑ(2), Uchihashi T(1), Kurioka K(1), Takahashi H(2), Sugauchi A(1), Takaha ...
  
  
Author information: (1)The First Department of Oral and Maxillofacial Surgery, Graduate School of De ...
  
  
Melanoma is an aggressive type of cancer originating from the skin that arises from neoplastic changes in melanocytes. Transforming growth factor‑β (TGF‑β) is a pleiotropic cytokine and is known to contribute to melanoma progression by inducing the epithelial‑mesenchymal transition (EMT) program and creating an environment that favors tumor progression. There are three TGF‑β isoforms, TGF‑β1, TGF‑β2 and TGF‑β3, all of which engage in pro‑tumorigenic activities by activating SMAD signaling pathways. All TGF‑β isoforms activate signaling pathways by binding to their TGF‑β type I (TβRI) and type II (TβRII) receptors. Thus, effective targeting of all TGF‑β isoforms is of great importance. In the present study, chimeric proteins comprising the extracellular domains of TβRI and/or TβRII fused with the Fc portion of human immunoglobulin (IgG) were validated in the melanoma context. The Fc chimeric receptor comprising both TβRI and TβRII (TβRI‑TβRII‑Fc) effectively trapped all TGF‑β isoforms. Conversely, TβRII‑Fc chimeric receptor, that comprises TβRII only, was able to interact with TGF‑β1 and TGF‑β3 isoforms, but not with TGF‑β2, which is a poor prognostic factor for melanoma patients. Accordingly, it was revealed that TβRI‑TβRII‑Fc chimeric receptor suppressed the EMT program in melanoma cells in vitro induced by any of the three TGF‑β isoforms, as revealed by decreased expression of mesenchymal markers. Conversely, TβRII‑Fc chimeric receptor inhibited the EMT program induced by TGF‑β1 and TGF‑β3. In addition, it was established that tumor growth in subcutaneous mouse melanoma was inhibited by TβRI‑TβRII‑Fc chimeric receptor indicating that Fc chimeric receptor could be applied to modify the tumor microenvironment (TME) of melanoma. Therefore, designing of Fc chimeric receptors targeting TGF‑β signals that affect various components of the TME may result in the development of effective anti‑melanoma agents.
  
  
  
  
**TGFBR2**
-
10
of
10
  

### Canine Transforming Growth Factor-β Receptor 2-Ig: A Potential Candidate Biologic for Melanoma Treatment That Reverses Transforming Growth Factor-β1 Immunosuppression.

10. Front Vet Sci. 2021 Jun 14;8:656715. doi: 10.3389/fvets.2021.656715 eCollection 2021.
  
  
Takeuchi H(1), Konnai S(1)(2), Maekawa N(2), Takagi S(3)(4), Ohta H(3), Sasaki N(3), Kim S(3), Okaga ...
  
  
Author information: (1)Department of Disease Control, Faculty of Veterinary Medicine, Hokkaido Unive ...
  
  
Cancer cells can evade host immune systems via multiple mechanisms. Transforming growth factor beta 1 (TGF-β1) is an immunosuppressive cytokine that induces regulatory T cell (Tregs) differentiation and is involved in immune evasion mechanisms in cancer. The inhibition of the TGF-β1 signaling pathway can suppress cancer progression and metastasis through the modulation of anticancer immune responses. However, to best of our knowledge, no implementation of treatments targeting TGF-β1 has been reported in dog cancers. This study aimed to examine whether TGF-β1 is upregulated in canine cancers. We measured TGF-β1 concentrations in culture supernatants of canine melanoma cell lines and in serum samples from dogs with oral malignant melanoma. TGF-β1 production was observed in several cell lines, and serum TGF-β1 levels were elevated in dogs with oral malignant melanoma. Interestingly, the addition of recombinant TGF-β1 to canine peripheral blood mononuclear cell cultures decreased Th1 cytokine production and increased differentiation of CD4+CD25+Foxp3+ lymphocytes, suggesting that TGF-β1 is immunosuppressive in canine immune systems. We developed a decoy receptor for TGF-β, namely TGF-βRII-Ig, by identifying an open reading frame of the canine TGFBR2 gene. TGF-βRII-Ig was prepared as a recombinant fusion protein of the extracellular region of canine TGF-βRII and the Fc region of canine IgG-B. As expected, TGF-βRII-Ig bound to TGF-β1. In the presence of TGF-β1, the treatment with TGF-βRII-Ig increased Th1 cytokine production and decreased the differentiation of CD4+CD25+Foxp3+ lymphocytes. Our results suggest that TGF-βRII-Ig competitively inhibits the immunosuppressive effects of TGF-β1 and thereby activates immune responses. This study demonstrated the potential of TGF-βRII-Ig as a novel biologic for canine melanoma.
  
  
  
  
  

---

  

# AFF1

**Signature gene in:** Dendritic cells, Plasma cells
  
**Differentially expressed in:** no cell type
  
  
**AFF1**
-
1
of
10
  

### Effective Utilization of a Customized Targeted Hybrid Capture RNA Sequencing in the Routine Molecular Categorization of Adolescent and Adult B-Lineage Acute Lymphoblastic Leukemia: A Real-World Experience.

1. Mol Diagn Ther. 2025 May;29(3):407-418. doi: 10.1007/s40291-025-00779-5 Epub 2025 Apr 5.
  
  
Sreedharanunni S(#)(1), Thakur V(#)(2), Balakrishnan A(2), Sachdeva MUS(2), Kaur P(2), Raina S(2), J ...
  
  
Author information: (1)Department of Hematology, Postgraduate Institute of Medical Education and Res ...
  
  
INTRODUCTION: Recent World Health Organization (WHO) and International Consensus Classifications have introduced numerous molecular entities in B-lineage acute lymphoblastic leukemia (B-ALL), necessitating comprehensive genomic characterization by detecting gene fusions, expression, mutations, and exon deletions. While whole-genome plus transcriptome sequencing is the ideal strategy, it remains cost-prohibitive for routine use. This study reports a cost-effective and reasonably efficient alternate approach integrating a customized targeted hybrid capture RNA sequencing (RNAseq) into the routine workup. METHODOLOGY: A total of 95 consecutive adolescent/adult B-ALL cases negative for common chimeric gene fusions (CGF) (BCR::ABL1, KMT2A::AFF1, TCF3::PBX1, and ETV6::RUNX1) were analyzed using a customized 69-gene targeted RNAseq panel. In total, three fusion detection pipelines, the Trinity Cancer Transcriptome Analysis Toolkit (CTAT) Mutations pipeline, and the Toblerone alignment tool were employed, and the results were compared with fluorescence in situ hybridization (FISH)/multiplex ligation-dependent probe amplification (MLPA) testing. RESULTS: RNAseq identified fusions in 43% of cases (including BCR::ABL1-like: 15.8% and IGH::DUX4: 10.5%), demonstrating superior detection of cryptic intrachromosomal rearrangements. Somatic variants were detected in 30% of cases (including rat sarcoma (RAS) pathway and Janus kinase (JAK)-signal transducers and activators of transcription (STAT) variants in 18% and 5.3% respectively), and IKZF1 deletions were detected in 25% (77% concordance with MLPA). The integration of targeted RNAseq and comprehensive bioinformatic analysis with flow-cytometry-based ploidy analysis and FISH-based IGH rearrangements helped categorize 79% of common CGF-negative B-ALL. The BCR::ABL1/BCR::ABL1-like group showed a higher frequency of pathogenic IKZF1 deletions (50% versus 21.7%; p = 0.011), measurable residual disease (92% versus 51%; p = 0.009), and poorer overall survival (8.6 versus 22.8 months; p = 0.07). DISCUSSION AND CONCLUSIONS: Effective utilization of RNAseq data by comprehensive bioinformatic analysis to test fusions, mutations, and deletions, supported by only minimal supplementary FISH testing, provides a practical, cost-effective solution for the molecular characterization of B-ALL in real-world scenarios until a single alternative and cost-effective test is available.
  
  
  
  
**AFF1**
-
2
of
10
  

### Cryptic Rearrangement of the KMT2A Gene in a B-cell Acute Lymphoblastic Leukemia.

2. Anticancer Res. 2025 Mar;45(3):921-928. doi: 10.21873/anticanres.17479
  
  
Brunetti M(1), Andersen K(1), Spetalen S(2)(3), Tjønnfjord GE(1)(3)(4), Heim S(1)(3), Micci F(1).
  
  
Author information: (1)Section for Cancer Cytogenetics, Institute for Cancer Genetics and Informatic ...
  
  
BACKGROUND/AIM: A 30-year-old female diagnosed with B cell acute lymphoblastic leukemia (B-ALL) had a normal karyotype at diagnosis. CASE REPORT: The case was investigated further by fluorescence in situ hybridization (FISH), array comparative genomic hybridization (aCGH), and reverse-transcription polymerase chain reaction (RT-PCR) followed by Cycle sequencing. The diagnostic karyotype was normal (46,XX), but FISH studies on tumor cells using a KMT2A break-apart probes showed that the proximal part of KMT2A was inserted into an apparently normal chromosome 4 with concomitant loss of the distal part of the probe. aCGH identified losses within 11q23.3 and 4q21.3q22.1 with the breakpoints mapping inside the KMT2A and AFF1 loci. The presence of the putative KMT2A::AFF1 fusion gene was confirmed by FISH analysis and RT-PCR/Cycle sequencing; an in-frame fusion was detected between KMT2A (exon 9) and AFF1 (exon 6). The patient underwent allogenic stem cell transplantation and reached complete remission. CONCLUSION: This case highlights the need to supplement banding cytogenetics with appropriate molecular (cyto)genetic techniques whenever the karyotype does not reveal characteristic aberrations. Although KMT2A rearrangements in both lymphoblastic and myeloid acute leukemias usually arise through karyotypically visible chromosomal recombinations, this is not always the case.
  
  
  
  
**AFF1**
-
3
of
10
  

### Genetic Diversity in KMT2A -r and KMT2A -Wt Groups: Assessing the Prognostic Value of Markers in BCP-ALL Among Infants.

3. Int J Lab Hematol. 2025 Jun;47(3):472-480. doi: 10.1111/ijlh.14442 Epub 2025 Feb 25.
  
  
Ilyasova K(1), Zerkalenkova E(1), Soldatkina O(1), Kazakova A(1), Myakova N(1), Roumiantseva J(1), F ...
  
  
Author information: (1)Dmitry Rogachev National Research and Clinical Centre for Pediatric Hematolog ...
  
  
BACKGROUND/OBJECTIVES: Infant BCP-ALL is classified into KMT2A-r and KMT2A-wt groups, both showing heterogeneity. KMT2A rearrangements indicate poor prognosis, but outcomes vary by fusion partner. The KMT2A-wt group includes cases in the B-other ALL subgroup, with unclear prognostic significance. We aim to improve understanding of molecular subtypes in KMT2A-r and KMT2A-wt, focusing on NUTM1 and PAX5 rearrangements. METHODS: We analyzed 175 infants (aged 0-365 days) diagnosed with BCP-ALL from 2010 to 2023 at the Dmitry Rogachev National Medical Research Center of Pediatric Hematology, Oncology and Immunology. Genomic aberrations were identified by karyotyping, FISH and RNA-seq. RNA-seq was performed using the Illumina, and gene fusions were validated by Sanger sequencing. RESULTS: There was no difference in survival based on KMT2A partner genes. The KMT2A::AFF1 group showed similar outcomes to other partners, with 2-year EFS of 36% (95% CI, 21%-59%) versus 37% (95% CI, 23%-60%) (log-rank test, p = 0.9). In the KMT2A-wt group (n = 33, 17.7% of cases), NUTM1-r (n = 9) and PAX5-r (n = 10) accounted for 27% and 30.3%, respectively. The NUTM1-r and PAX5-r groups showed excellent survival rates, with 2-year EFS of 80% (95% CI, 52%-100%) and 100% (95% CI, 100%-100%), respectively, but the small cohort size limit the statistical power of the analysis (log-rank test, p = 0.9). CONCLUSIONS: Survival in the KMT2A-r group did not differ by fusion partner. NUTM1 rearrangements showed a favorable prognosis, and PAX5-rearranged patients had better outcomes than previously reported. In the NUTM1-r group, the most common fusion, BRD9:NUTM1, showed variability in breakpoints (Exons 3, 8, and 14 of BRD9).
  
  
  
  
**AFF1**
-
4
of
10
  

### A Case of Childhood Acute Lymphoblastic Leukemia With Retinitis Pigmentosa-like Fundus Findings.

4. Cureus. 2025 Jan 7;17(1):e77094. doi: 10.7759/cureus.77094 eCollection 2025 Jan.
  
  
Okujima N(1), Iimori H(1), Shiraishi A(1).
  
  
Author information: (1)Department of Ophthalmology, Ehime University School of Medicine, Toon, JPN.
  
  
Ophthalmic manifestations are a common occurrence in leukaemia cases and may result from a number of factors, including direct leukemic infiltration, haematologic abnormalities, central nervous system (CNS) involvement, or treatment-related effects. This case report discusses a paediatric patient with acute lymphoblastic leukaemia (ALL) who developed recurrent ocular symptoms, including retinitis pigmentosa-like findings, which is degeneration with pigment neopigmentation in the mid- to peripheral fundus, during remission, underscoring the necessity of continuous monitoring. A four-year-old female with a history of ALL, diagnosed at two years and three months of age, presented with ophthalmic complications during remission. The leukaemia was characterised by positivity for CD20, CD19, and the mature B-cell antigen, as well as a KMT2A-AFF1 gene rearrangement. Despite the administration of an intensive chemotherapy regimen and CAR-T cell therapy, the patient experienced a relapse and subsequently underwent umbilical cord blood transplantation, resulting in the attainment of molecular remission. However, at three years and seven months of age, a notable increase in the size of the left orbital tumour was observed. At three years and 11 months of age, the patient exhibited retinal changes that were consistent with retinitis pigmentosa, as confirmed by fundus photography. Additional findings included moderate left pupil dilation, an absent light reflex, optic disc swelling, dilated retinal vessels, and a serous retinal detachment. The presence of KMT2A-AFF1 mRNA in the anterior chamber fluid served to confirm the occurrence of an ocular relapse, a finding that was also evident in the bone marrow analysis. The patient was treated with salvage chemotherapy and localised radiation therapy (24 Gy) to the left eye, resulting in partial resolution of symptoms. At the four-year and three-month follow-up, retinitis pigmentosa-like changes with proliferative alterations persisted, although the serous retinal detachment had resolved. This case demonstrates that ophthalmic manifestations can occur even during the remission phase of leukaemia, emphasising the importance of regular, multidisciplinary follow-up.
  
  
  
  
**AFF1**
-
5
of
10
  

### Next-generation sequencing RNA fusion panel for the diagnosis of haematological malignancies.

5. Pathology. 2025 Apr;57(3):340-347. doi: 10.1016/j.pathol.2024.09.009 Epub 2024 Nov 16.
  
  
Ngo TQ(1), Goh AFN(2), Dorwal P(3), Leong E(4), Shortt J(5), Fedele PL(6), Gilbertson M(6), Fong CY( ...
  
  
Author information: (1)Department of Haematology, Monash Health, Clayton, Vic, Australia; Department ...
  
  
Haematological malignancies are being increasingly defined by gene rearrangements, which have traditionally been detected by karyotype, fluorescent in situ hybridisation (FISH) or reverse-transcriptase polymerase chain reaction (RT-PCR). However, these traditional methods may miss cryptic gene rearrangements and are limited by the number of gene rearrangements screened at any one time. A next-generation sequencing (NGS) RNA fusion panel is an evolving technology that can identify multiple fusion transcripts in a single molecular assay, even without prior knowledge of breakpoints or fusion partners. We explored the utility of the Illumina TruSight RNA Fusion Panel for use in haematological malignancies by sequencing 30 peripheral blood or bone marrow aspirate samples. Secondary and tertiary analyses were performed using the Illumina DRAGEN RNA pipeline and PierianDx Clinical Genomics Workspace platform. Our RNA fusion panel was able to reliably detect known fusion transcripts, such as BCR::ABL1, ETV6::RUNX1 and KMT2A::AFF1, in acute lymphoblastic leukaemia (ALL), KMT2A::MLLT3, KMT2A::MLLT6, PML::RARA and CBFB::MYH11 in acute myeloid leukaemia (AML), and FIP1L1::PDGFRA in myeloid/lymphoid neoplasm with eosinophilia (MLN-Eo). In addition, it was able to detect rare KAT6A::CREBBP and CHIC2::ETV6 fusions, which could not be confirmed by traditional methods. The assay had a transcript limit of detection of approximately 5-10% of positive controls. These findings confirm the unique utility of the NGS-based RNA fusion panel as a diagnostic tool to identify gene rearrangements that drive haematological malignancies. It can identify novel and rare gene rearrangements to assist with diagnosis, prognostication and treatment decisions.
  
  
  
  
**AFF1**
-
6
of
10
  

### MLL-AF9 regulates transcriptional initiation in mixed lineage leukemic cells.

6. J Biol Chem. 2024 Aug;300(8):107566. doi: 10.1016/j.jbc.2024.107566 Epub 2024 Jul 11.
  
  
Yang Z(1), Zhang G(2), Zhao R(1), Tian T(1), Zhi J(1), Wei G(2), Roeder RG(3), Jing L(4), Yu M(5).
  
  
Author information: (1)Sheng Yushou Center of Cell Biology and Immunology, School of Life Sciences a ...
  
  
Mixed lineage leukemia-fusion proteins (MLL-FPs) are believed to maintain gene activation and induce MLL through aberrantly stimulating transcriptional elongation, but the underlying mechanisms are incompletely understood. Here, we show that both MLL1 and AF9, one of the major fusion partners of MLL1, mainly occupy promoters and distal intergenic regions, exhibiting chromatin occupancy patterns resembling that of RNA polymerase II in HEL, a human erythroleukemia cell line without MLL1 rearrangement. MLL1 and AF9 only coregulate over a dozen genes despite of their co-occupancy on thousands of genes. They do not interact with each other, and their chromatin occupancy is also independent of each other. Moreover, AF9 deficiency in HEL cells decreases global TBP occupancy while decreases CDK9 occupancy on a small number of genes, suggesting an accessory role of AF9 in CDK9 recruitment and a possible major role in transcriptional initiation via initiation factor recruitment. Importantly, MLL1 and MLL-AF9 occupy promoters and distal intergenic regions, exhibiting identical chromatin occupancy patterns in MLL cells, and MLL-AF9 deficiency decreased occupancy of TBP and TFIIE on major target genes of MLL-AF9 in iMA9, a murine acute myeloid leukemia cell line inducibly expressing MLL-AF9, suggesting that it can also regulate initiation. These results suggest that there is no difference between MLL1 and MLL-AF9 with respect to location and size of occupancy sites, contrary to what people have believed, and that MLL-AF9 may also regulate transcriptional initiation in addition to widely believed elongation.
  
  
  
  
**AFF1**
-
7
of
10
  

### Sequential drug treatment targeting cell cycle and cell fate regulatory programs blocks non-genetic cancer evolution in acute lymphoblastic leukemia.

7. Genome Biol. 2024 May 31;25(1):143. doi: 10.1186/s13059-024-03260-4
  
  
Malyukova A(#)(1), Lahnalampi M(#)(2), Falqués-Costa T(3), Pölönen P(2), Sipola M(2), Mehtonen J(2), ...
  
  
Author information: (1)Department of Cell and Molecular Biology, Karolinska Institutet, Biomedicum, ...
  
  
BACKGROUND: Targeted therapies exploiting vulnerabilities of cancer cells hold promise for improving patient outcome and reducing side-effects of chemotherapy. However, efficacy of precision therapies is limited in part because of tumor cell heterogeneity. A better mechanistic understanding of how drug effect is linked to cancer cell state diversity is crucial for identifying effective combination therapies that can prevent disease recurrence. RESULTS: Here, we characterize the effect of G2/M checkpoint inhibition in acute lymphoblastic leukemia (ALL) and demonstrate that WEE1 targeted therapy impinges on cell fate decision regulatory circuits. We find the highest inhibition of recovery of proliferation in ALL cells with KMT2A-rearrangements. Single-cell RNA-seq and ATAC-seq of RS4;11 cells harboring KMT2A::AFF1, treated with the WEE1 inhibitor AZD1775, reveal diversification of cell states, with a fraction of cells exhibiting strong activation of p53-driven processes linked to apoptosis and senescence, and disruption of a core KMT2A-RUNX1-MYC regulatory network. In this cell state diversification induced by WEE1 inhibition, a subpopulation transitions to a drug tolerant cell state characterized by activation of transcription factors regulating pre-B cell fate, lipid metabolism, and pre-BCR signaling in a reversible manner. Sequential treatment with BCR-signaling inhibitors dasatinib, ibrutinib, or perturbing metabolism by fatostatin or AZD2014 effectively counteracts drug tolerance by inducing cell death and repressing stemness markers. CONCLUSIONS: Collectively, our findings provide new insights into the tight connectivity of gene regulatory programs associated with cell cycle and cell fate regulation, and a rationale for sequential administration of WEE1 inhibitors with low toxicity inhibitors of pre-BCR signaling or metabolism.
  
  
  
  
**AFF1**
-
8
of
10
  

### Optimized cytogenetic risk-group stratification of KMT2A-rearranged pediatric acute myeloid leukemia.

8. Blood Adv. 2024 Jun 25;8(12):3200-3213. doi: 10.1182/bloodadvances.2023011771
  
  
van Weelderen RE(1)(2), Harrison CJ(3), Klein K(1)(2)(4), Jiang Y(1), Abrahamsson J(5), Alonzo T(6), ...
  
  
Author information: (1)Princess Máxima Center for Pediatric Oncology, Utrecht, The Netherlands. (2)E ...
  
  
A comprehensive international consensus on the cytogenetic risk-group stratification of KMT2A-rearranged (KMT2A-r) pediatric acute myeloid leukemia (AML) is lacking. This retrospective (2005-2016) International Berlin-Frankfurt-Münster Study Group study on 1256 children with KMT2A-r AML aims to validate the prognostic value of established recurring KMT2A fusions and additional cytogenetic aberrations (ACAs) and to define additional, recurring KMT2A fusions and ACAs, evaluating their prognostic relevance. Compared with our previous study, 3 additional, recurring KMT2A-r groups were defined: Xq24/KMT2A::SEPT6, 1p32/KMT2A::EPS15, and 17q12/t(11;17)(q23;q12). Across 13 KMT2A-r groups, 5-year event-free survival probabilities varied significantly (21.8%-76.2%; P < .01). ACAs occurred in 46.8% of 1200 patients with complete karyotypes, correlating with inferior overall survival (56.8% vs 67.9%; P < .01). Multivariable analyses confirmed independent associations of 4q21/KMT2A::AFF1, 6q27/KMT2A::AFDN, 10p12/KMT2A::MLLT10, 10p11.2/KMT2A::ABI1, and 19p13.3/KMT2A::MLLT1 with adverse outcomes, but not those of 1q21/KMT2A::MLLT11 and trisomy 19 with favorable and adverse outcomes, respectively. Newly identified ACAs with independent adverse prognoses were monosomy 10, trisomies 1, 6, 16, and X, add(12p), and del(9q). Among patients with 9p22/KMT2A::MLLT3, the independent association of French-American-British-type M5 with favorable outcomes was confirmed, and those of trisomy 6 and measurable residual disease at end of induction with adverse outcomes were identified. We provide evidence to incorporate 5 adverse-risk KMT2A fusions into the cytogenetic risk-group stratification of KMT2A-r pediatric AML, to revise the favorable-risk classification of 1q21/KMT2A::MLLT11 to intermediate risk, and to refine the risk-stratification of 9p22/KMT2A::MLLT3 AML. Future studies should validate the associations between the newly identified ACAs and outcomes and unravel the underlying biological pathogenesis of KMT2A fusions and ACAs.
  
  
  
  
**AFF1**
-
9
of
10
  

### Lineage switch of KMT2A-rearranged adult B-lineage acute lymphoblastic leukemia following bispecific T-cell engager and monoclonal antibody therapy.

9. J Hematop. 2023 Jun;16(2):103-109. doi: 10.1007/s12308-023-00539-6 Epub 2023 Mar 22.
  
  
Wu JR(1), Shih PC(1), Li C(1), Chao HL(1), Wang HC(1), Chiang YM(1), Liu YJ(1), Hsu SC(1)(2), Yao CY ...
  
  
Author information: (1)Department of Laboratory Medicine, National Taiwan University Hospital, Colle ...
  
  
Adult B-lineage acute lymphoblastic leukemia (B-ALL) with t(4;11)(q21;q23) is very rare. It is characterized by mixed-lineage leukemia and has the potential for lineage switching during the treatment course. We report the disease course of a patient with B-ALL with t(4;11)(q21;q23) to demonstrate that close monitoring of cell morphology and immunophenotyping is necessary to capture the lineage switch at an early stage. Cell morphology, immunophenotyping, and cytogenetics were used to evaluate the patient's disease status. A 36-year-old woman was diagnosed with B-ALL with t(4;11)(q21;q23), which encodes the KMT2A::AFF1 fusion. After the initial induction chemotherapy, her disease remained refractory, and the patient received salvage immunotherapy with blinatumomab and inotuzumab ozogamicin. However, the ALL did not respond. Repeated bone marrow examinations unexpectedly revealed the emergence of a major population of monoblasts, in addition to a minor population of the original B lymphoblasts. The patient was diagnosed with disease evolution from B-ALL to mixed-phenotype acute leukemia (MPAL, B/myeloid). We present this case to highlight the potential of KMT2A-rearranged B-ALL to undergo lineage switch following B-cell targeted therapy. Patients with this kind of B-ALL should therefore be closely monitored to capture potential changes in the nature of the disease and prompt appropriate treatment.
  
  
  
  
**AFF1**
-
10
of
10
  

### Four Additional Doses of PEG-L-Asparaginase During the Consolidation Phase in the AIEOP-BFM ALL 2009 Protocol Do Not Improve Outcome and Increase Toxicity in High-Risk ALL: Results of a Randomized Study.

10. J Clin Oncol. 2024 Mar 10;42(8):915-926. doi: 10.1200/JCO.23.01388 Epub 2023 Dec 14.
  
  
Conter V(1), Valsecchi MG(2)(3), Cario G(4), Zimmermann M(5), Attarbaschi A(6)(7), Stary J(8), Niggl ...
  
  
Author information: (1)Tettamanti Center, Fondazione IRCCS San Gerardo dei Tintori, Monza, Italy. (2 ...
  
  
PURPOSE: The AIEOP-BFM ALL 2009 protocol included, at the end of the induction phase, a randomized study of patients with high-risk (HR) ALL to investigate if an intensive exposure to pegylated L-asparaginase (PEG-ASNASE, 2,500 IU/sqm once a week × 4) on top of BFM consolidation phase IB allowed us to decrease minimal residual disease (MRD) and improve outcome. PATIENTS AND METHODS: A total of 1,097 patients presented, from June 2010 to February 2017, with one or more of the following HR criteria: KMT2A::AFF1 rearrangement, hypodiploidy, prednisone poor response, poor bone marrow response at day 15 (Flow MRD ≥10%), or no complete remission (CR) at the end of induction. Of them, 809 (85.1%) were randomly assigned to receive (404) or not receive (405) four weekly doses of PEG-ASNASE. RESULTS: By intention to treat (ITT) analysis, there was no significant difference in the proportion of patients with polimerase chain reaction MRD ≥5 × 10^-4^ at the end of phase IB in the experimental versus control arm (13.9% v 17.0%, P = .25). The 5-year event-free survival (median follow-up 6.3 years) by ITT in the experimental and control arms was 70.4% (2.3) versus 75.0% (2.2; P = .18), and the 5-year overall survival was 81.5% (2.0) versus 84.0% (1.9; P = .25), respectively. The corresponding 5-year cumulative incidence of death in CR was 9.5% (1.5) versus 5.7% (1.2; P = .08), and that of relapse was 17.7% (1.9) versus 17.2% (1.9), respectively (P = .94). Adverse reactions in phase IB occurred in 22.2% and 8.9% of patients in the experimental and control arm, respectively (P < .001). CONCLUSION: Additional PEG-ASNASE in phase IB did not translate into a benefit for decreasing relapse incidence but was associated with higher toxicity. Further improvements with conventional chemotherapy might be difficult in the context of intensive treatment protocols.
  
  
  
  
  

---

  

# AFF3

**Signature gene in:** B cells
  
**Differentially expressed in:** no cell type
  
  
**AFF3**
-
1
of
10
  

### WNT11 Promotes immune evasion and resistance to Anti-PD-1 therapy in liver metastasis.

1. Nat Commun. 2025 Feb 7;16(1):1429. doi: 10.1038/s41467-025-56714-z
  
  
Jiang W(#)(1)(2)(3), Guan B(#)(4), Sun H(#)(4), Mi Y(#)(5), Cai S(1)(6)(7), Wan R(2)(3), Li X(6)(7), ...
  
  
Author information: (1)Cancer Institute, Fudan University Shanghai Cancer Center, No. 270 Dong'an Ro ...
  
  
Liver metastasis (LM) poses a significant challenge in cancer treatment, with limited available therapeutic options and poor prognosis. Understanding the dynamics of tumor microenvironment (TME) and immune interactions is crucial for developing effective treatments. We find that WNT11 promoted CD8+ T-cell exclusion and suppression, which was correlated with poor prognosis in LM. Mechanistically, WNT11-overexpressing tumor cells directly reduce CD8+ T-cell recruitment and activity by decreasing CXCL10 and CCL4 expression through CAMKII-mediated β-catenin/AFF3 downregulation. WNT11-overexpressing tumor cells promote immunosuppressive macrophage polarization by inducing IL17D expression via the CAMKII/NF-κB pathway, which result in CD8+ T-cell suppression. Moreover, CAMKII inhibition increases the efficacy of anti-PD-1 therapy in mouse model of LM. Serum expression of WNT11 is identified as a potential minimally invasive biomarker in the management of colorectal cancer-LM with immunotherapy. Our findings highlight WNT11/CAMKII axis as a critical regulator of the TME and a promising target for immunotherapy in patients with LM.
  
  
  
  
**AFF3**
-
2
of
10
  

### Integrated mendelian randomization analyses highlight AFF3 as a novel eQTL-mediated susceptibility gene in renal cancer and its potential mechanisms.

2. BMC Cancer. 2024 Jun 17;24(1):739. doi: 10.1186/s12885-024-12513-1
  
  
Wang Q(1), Chen S(1), Wang G(1), Zhang T(1), Gao Y(2).
  
  
Author information: (1)Department of Urology, Jianhu Clinical Medical College of Yangzhou University ...
  
  
BACKGROUNDS: A growing number of expression quantitative trait loci (eQTLs) have been found to be linked with tumorigenesis. In this article, we employed integrated Mendelian randomization (MR) analyses to identify novel susceptibility genes in renal cancer (RC) and reveal their potential mechanisms. METHODS: Two-sample MR analyses were performed to infer causal relationships between eQTLs, metabolites, and RC risks through the "TwoSampleMR" R package. Sensitivity analyses, such as heterogeneity, pleiotropy, and leave-one-out analysis, were used to assess the stability of our outcomes. Summary-data-based MR (SMR) analyses were used to verify the causal relationships among cis-eQTLs and RC risks via the SMR 1.3.1 software. RESULTS: Our results provided the first evidence for AFF3 eQTL elevating RC risks, suggesting its oncogenic roles (IVW method; odds ratio (OR) = 1.0005; 95% confidence interval (CI) = 1.0001-1.0010; P = 0.0285; heterogeneity = 0.9588; pleiotropy = 0.8397). Further SMR analysis validated the causal relationships among AFF3 cis-eQTLs and RC risks (P < 0.05). Moreover, the TCGA-KIRC, the ICGC-RC, and the GSE159115 datasets verified that the AFF3 gene was more highly expressed in RC tumors than normal control via scRNA-sequencing and bulk RNA-sequencing (P < 0.05). Gene set enrichment analysis (GSEA) analysis identified six potential biological pathways of AFF3 involved in RC. As for the potential mechanism of AFF3 in RC, we concluded in this article that AFF3 eQTL could negatively modulate the levels of the X-11,315 metabolite (IVW method; OR = 0.9127; 95% CI = 0.8530-0.9765; P = 0.0081; heterogeneity = 0.4150; pleiotropy = 0.8852), exhibiting preventive effects against RC risks (IVW method; OR = 0.9987; 95% CI = 0.9975-0.9999; P = 0.0380; heterogeneity = 0.5362; pleiotropy = 0.9808). CONCLUSIONS: We concluded that AFF3 could serve as a novel eQTL-mediated susceptibility gene in RC and reveal its potential mechanism of elevating RC risks via negatively regulating the X-11,315 metabolite levels.
  
  
  
  
**AFF3**
-
3
of
10
  

### Comprehensive analysis of the functions, prognostic and diagnostic values of RNA binding proteins in head and neck squamous cell carcinoma.

3. J Stomatol Oral Maxillofac Surg. 2024 Sep;125(4S):101937. doi: 10.1016/j.jormas.2024.101937 Epub 2024 Jun 4.
  
  
Liu H(1), Ye Z(1), Wang X(2), Wu Y(3), Deng C(4).
  
  
Author information: (1)School of Stomatology, Wannan Medical College, Wuhu, China; Anhui Provincial ...
  
  
BACKGROUND: Accumulating evidence has suggested that RNA binding protein (RBP) dysregulation plays an essential role during tumorigenesis. Here, we sought to explore the potential biological functions and clinical significance of RBP and develop diagnostic and prognostic signatures based on RBP in patients with head and neck squamous cell carcinoma (HNSCC). METHODS: The differently expressed RBPs between HNSCC samples and their normal counterparts were identified using the Limma package. The immunohistochemistry (IHC) images of several RBPs were collected from the Human Protein Atlas database. The diagnostic signature based on RBP was built by LASSO-logistic regression and random forest. The prognostic signature based on RBP was constructed by LASSO and stepwise Cox regression analysis in the training cohort and validated in the validation cohort. RESULTS: Eighty-four aberrantly expressed RBPs were obtained, comprising 41 up-regulated and 43 down-regulated RBPs. Seven RBP genes (CPEB3, PDCD4, ENDOU, PARP12, DNMT3B, IGF2BP1, EXO1) were identified as diagnostic-related hub genes. They were used to establish a diagnostic RBP signature risk score (DRBPS) model by the coefficients in least absolute shrinkage and selection operator (LASSO)-logistic regression analysis and showed high specificity and sensitivity in the training (area under the receiver operating characteristic curve (AUC) = 0.998), and in all validation cohorts (AUC > 0.95 for all). Similarly, seven RBP genes (MKRN3, ZC3H12D, EIF5A2, AFF3, SIDT1, RBM24, and NR0B1) were identified as prognosis-associated hub genes by LASSO and stepwise multiple Cox regression analyses and were used to construct the prognostic model named as PRBPS. The AUC of the time-dependent receiver operator characteristic curve of the prognostic model was 0.664 at 3 years and 0.635 at 5 years in the training cohort and 0.720, 0.777 in the validation cohort, showing a favorable predictive efficacy for prognosis in HNSCC. CONCLUSIONS: Our results demonstrate the value of consideration of RBP in the diagnosis and prognosis for HNSCC and provide a novel insight into understanding the potential role of dysregulated RBP in HNSCC.
  
  
  
  
**AFF3**
-
4
of
10
  

### The differential expression of AFF3 in cervical cancer and its correlation with clinicopathological features and prognosis.

4. J Obstet Gynaecol. 2024 Dec;44(1):2333784. doi: 10.1080/01443615.2024.2333784 Epub 2024 Apr 11.
  
  
Zhang Y(1), Li L(2), Han Q(1), Wen L(1).
  
  
Author information: (1)Department of Gynaecology and Obstetrics, JiLin Provinc YanBian University Ho ...
  
  
BACKGROUND: Cervical cancer (CC) is the second most common malignancy in women, and identifying biomarkers of CC is crucial for prognosis prediction. Here, we investigated the expression of AF4/FMR2 Family Member 3 (AFF3) in CC and its association with clinicopathological features and prognosis. METHODS: Tumour and adjacent tissues, along with clinicopathological features and follow-up information, were collected from 78 patients. AFF3 expression was assessed using quantitative real-time polymerase chain reaction and Western blotting. The correlation between AFF3 expression and CC symptoms was using chi-square test. The 5-year overall survival (OS) was analysed using the Kaplan-Meier method. The Univariate analysis of prognostic risk factors was conducted using the COX proportional hazards model, followed by multivariate COX regression analysis including variables with p < 0.01. RESULTS: AFF3 expression was downregulated in CC, and its levels were correlated with lymph node metastasis (LNM) and International Federation of Gynaecology and Obstetrics (FIGO) stage. Patients with low AFF3 expression had a lower 5-year OS rate (52.78%, 19/36). Postoperative survival was reduced in patients with histological grade 3 (G3), myometrial invasion (depth ≥ 1/2), lymphovascular space invasion, LNM, and advanced FIGO stage. Low expression of AFF3 (HR: 2.848, 95% CI: 1.144-7.090) and histological grade G3 (HR: 4.393, 95% CI: 1.663-11.607) were identified as independent prognostic risk factors in CC patients. CONCLUSION: Low expression of AFF3 and histological G3 are independent predictors of poor prognosis in CC patients, suggesting that AFF3 could serve as a potential biomarker for prognostic assessment in CC.
  
  
  
  
**AFF3**
-
5
of
10
  

### Molecular Profiling of Axial Spondyloarthritis Patients Reveals an Association between Innate and Adaptive Cell Populations and Therapeutic Response to Tumor Necrosis Factor Inhibitors.

5. Biomolecules. 2024 Mar 21;14(3):382. doi: 10.3390/biom14030382
  
  
Sobral D(1), Fernandes AF(2), Bernardes M(3)(4), Pinto P(5), Santos H(6)(7), Lagoas-Gomes J(8), Tava ...
  
  
Author information: (1)Applied Molecular Biosciences Unit, Life Sciences Department, Sciences and Te ...
  
  
This study aims at identifying molecular biomarkers differentiating responders and non-responders to treatment with Tumor Necrosis Factor inhibitors (TNFi) among patients with axial spondyloarthritis (axSpA). Whole blood mRNA and plasma proteins were measured in a cohort of biologic-naïve axSpA patients (n = 35), pre and post (14 weeks) TNFi treatment with adalimumab. Differential expression analysis was used to identify the most enriched pathways and in predictive models to distinguish responses to TNFi. A treatment-associated signature suggests a reduction in inflammatory activity. We found transcripts and proteins robustly differentially expressed between baseline and week 14 in responders. C-reactive protein (CRP) and Haptoglobin (HP) proteins showed strong and early decrease in the plasma of axSpA patients, while a cluster of apolipoproteins (APOD, APOA2, APOA1) showed increased expression at week 14. Responders to TNFi treatment present higher levels of markers of innate immunity at baseline, and lower levels of adaptive immunity markers, particularly B-cells. A logistic regression model incorporating ASDAS-CRP, gender, and AFF3, the top differentially expressed gene at baseline, enabled an accurate prediction of response to adalimumab in our cohort (AUC = 0.97). In conclusion, innate and adaptive immune cell type composition at baseline may be a major contributor to response to adalimumab in axSpA patients. A model including clinical and gene expression variables should also be considered.
  
  
  
  
**AFF3**
-
6
of
10
  

### Loss of AR-regulated AFF3 contributes to prostate cancer progression and reduces ferroptosis sensitivity by downregulating ACSL4 based on single-cell sequencing analysis.

6. Apoptosis. 2024 Oct;29(9-10):1679-1695. doi: 10.1007/s10495-024-01941-w Epub 2024 Mar 13.
  
  
Fan A(#)(1), Li Y(#)(1), Zhang Y(#)(1), Meng W(2), Pan W(2), Chen M(2), Ma Z(3), Chen W(4).
  
  
Author information: (1)Department of Urology, Zhongshan Hospital, Fudan University, Shanghai, 200030 ...
  
  
Prostate cancer (PCa) is one of the most common cancers affecting the health of men worldwide. Castration-resistant prostate cancer (CRPC), the advanced and refractory phase of prostate cancer, has multiple mechanisms of resistance to androgen deprivation therapy (ADT) such as AR mutations, aberrant androgen synthase, and abnormal expression of AR-related genes. Based on the research of the AR pathway, new drugs for the treatment of CRPC have been developed in clinical practice, such as Abiraterone and enzalutamide. However, many areas in this pathway are still worth exploring. In this study, single-cell sequencing analysis was utilized to scrutinize significant genes in the androgen receptor (AR) pathway related to CRPC. Our analysis of single-cell sequencing combined with bulk-cell sequencing revealed a substantial downregulation of AR-regulated AFF3 in CRPC. Overexpression of AFF3 restricted the proliferation and migration of prostate cancer cells whilst also increasing their sensitivity towards enzalutamide, while knockdown of AFF3 had the opposite effect. To elucidate the mechanism of tumor inhibition by AFF3, we applied GSVA and GSEA to investigate the metabolic pathways related to AFF3 and revealed that AFF3 had an impact on fatty acids metabolism and ferroptosis through the regulation of ACSL4 protein expression. Based on correlation analysis and flow cytometry, we can speculate that AFF3 can impact the sensitivity of the CRPC cell lines to the ferroptosis inducer (RSL3) by regulating ACSL4. Therefore, our findings may provide new insights into the mechanisms of drug resistance in CRPC, and AFF3 may serve as a novel prognostic biomarker in prostate cancer.
  
  
  
  
**AFF3**
-
7
of
10
  

### Feasibility of whole-exome sequencing in fine-needle aspiration specimens of papillary thyroid microcarcinoma for the identification of novel gene mutations.

7. Clin Genet. 2024 May;105(5):567-572. doi: 10.1111/cge.14494 Epub 2024 Feb 7.
  
  
Ma L(1), Gao L(1), Hu Y(2), Li X(2), Liu C(2), Ji J(1), Shi X(1), Pan A(1), An Y(1), Luo N(1), Xia Y ...
  
  
Author information: (1)Department of Ultrasound, State Key Laboratory of Complex Severe and Rare Dis ...
  
  
Genetic profiling is important for assisting the management of papillary thyroid microcarcinoma (PTMC). Although whole-exome sequencing (WES) of surgically resected PTMC tissue has been performed and revealed potential prognostic biomarkers, its application in PTMC fine-needle aspiration (FNA) specimens has not been explored. This study aimed to evaluate the feasibility of WES using FNA specimens of PTMC. Five PTMC patients were enrolled with clinical characteristics gathered. Fine aspiration cytology needle (23 gauges) was used to collect FNA biopsy with ultrasound guidance. WES analysis of FNA specimens from five PTMC patients and matched blood samples was performed. The WES of FNA samples yielded an average sequencing depth of 281× and average coverage of 99.5%. We identified 534 somatic single-nucleotide variants and 13 indels in total, and per sample, we found a mean of 24 exonic mutations, which affected a total of 120 genes. In the PTMC FNA samples, the most frequently mutated genes were BRAF and ANKRD18B, and the four driver genes were BRAF, AFF3, SRCAP, and EGFR. We also identified several germline cancer predisposing gene mutations. The results suggest that WES of FNA specimens is feasible for PTMC and can identify novel genetic mutations.
  
  
  
  
**AFF3**
-
8
of
10
  

### Machine learning algorithm integrates bulk and single-cell transcriptome sequencing to reveal immune-related personalized therapy prediction features for pancreatic cancer.

8. Aging (Albany NY). 2023 Dec 12;15(23):14109-14140. doi: 10.18632/aging.205293 Epub 2023 Dec 12.
  
  
Zang L(1), Zhang B(1), Zhou Y(2), Zhang F(3), Tian X(3), Tian Z(1), Chen D(4)(5)(6), Miao Q(1).
  
  
Author information: (1)Department of General Surgery, Taiyuan Central Hospital, Taiyuan 030009, Shan ...
  
  
Pancreatic cancer (PC) is a digestive malignancy with worse overall survival. Tumor immune environment (TIME) alters the progression and proliferation of various solid tumors. Hence, we aimed to detect the TIME-related classifier to facilitate the personalized treatment of PC. Based on the 1612 immune-related genes (IRGs), we classified patients into Immune\_rich and Immune\_desert subgroups via consensus clustering. Patients in distinct subtypes exhibited a difference in sensitivity to immune checkpoint blockers (ICB). Next, the immune-related signature (IRS) model was established based on 8 IRGs (SYT12, TNNT1, TRIM46, SMPD3, ANLN, AFF3, CXCL9 and RP1L1) and validated its predictive efficiency in multiple cohorts. RT-qPCR experiments demonstrated the differential expression of 8 IRGs between tumor and normal cell lines. Patients who gained lower IRS score tended to be more sensitive to chemotherapy and immunotherapy, and obtained better overall survival compared to those with higher IRS scores. Moreover, scRNA-seq analysis revealed that fibroblast and ductal cells might affect malignant tumor cells via MIF-(CD74+CD44) and SPP1-CD44 axis. Eventually, we identified eight therapeutic targets and one agent for IRS high patients. Our study screened out the specific regulation pattern of TIME in PC, and shed light on the precise treatment of PC.
  
  
  
  
**AFF3**
-
9
of
10
  

### The Underlying Mechanism Involved in Gefitinib Resistance and Corresponding Experiment Validation in Lung Cancer.

9. Mediators Inflamm. 2023 May 9;2023:9658912. doi: 10.1155/2023/9658912 eCollection 2023.
  
  
Song P(1)(2), Zhou J(3), Wu K(1), Wang W(1), Gu S(1).
  
  
Author information: (1)Department of Thoracic Surgery, Shanghai Tongji Hospital, School of Medicine, ...
  
  
BACKGROUND: Gefitinib resistance remains a major problem in the treatment of lung cancer. However, the underlying mechanisms involved in gefitinib resistance are largely unclear. METHODS: Open-accessed data of lung cancer patients were downloaded from The Cancer Genome Atlas Program and Gene Expression Omnibus databases. CCK8, colony formation, and 5-ethynyl-2'-deoxyuridine assays were utilized to evaluate the cell proliferation ability. Transwell and wound-healing assays were utilized to evaluate the cell invasion and migration ability. Quantitative real-time PCR was utilized to detect the RNA level of specific genes. RESULTS: Here, we obtained the expression profile data of wild and gefitinib-resistant cells. Combined with the data from the TCGA and GDSC databases, we identified six genes, RNF150, FAT3, ANKRD33, AFF3, CDH2, and BEX1, that were involved in gefitinib resistance in both cell and tissue levels. We found that most of these genes were expressed in the fibroblast of the NSCLC microenvironment. Hence, we also comprehensively investigated the role of fibroblast in the NSCLC microenvironment, including its biological effect and cell interaction. Ultimately, CDH2 was selected for further analysis for its prognosis correlation. In vitro experiments presented the cancer-promoting role of CDH2 in NSCLC. Moreover, cell viability detection showed that the inhibition of CDH2 could significantly decrease the IC50 of gefitinib in NSCLC cells. GSEA showed that CDH2 could significantly affect the pathway activity of PI3K/AKT/mTOR signaling. CONCLUSIONS: This study is aimed at investigating the underlying mechanism involved in gefitinib resistance to lung cancer. Our research has improved researchers' understanding of gefitinib resistance. Meanwhile, we found that CDH2 could lead to gefitinib resistance through PI3K/AKT/mTOR signaling.
  
  
  
  
**AFF3**
-
10
of
10
  

### Whole Exome Sequencing of a Patient with a Milder Phenotype of Xeroderma Pigmentosum Group C.

10. Medicina (Kaunas). 2023 Apr 3;59(4):699. doi: 10.3390/medicina59040699
  
  
Seo JI(1), Nishigori C(2), Ahn JJ(3), Ryu JY(3), Lee J(4), Lee MH(1), Kim SK(5), Jeong KH(1).
  
  
Author information: (1)Department of Dermatology, College of Medicine, Kyung Hee University, Seoul 0 ...
  
  
A 17-year-old female Korean patient (XP115KO) was previously diagnosed with Xeroderma pigmentosum group C (XPC) by Direct Sanger sequencing, which revealed a homozygous nonsense mutation in the XPC gene (rs121965088: c.1735C > T, p.Arg579Ter). While rs121965088 is associated with a poor prognosis, our patient presented with a milder phenotype. Hence, we conducted whole-exome sequencing in the patient and her family members to detect coexisting mutations that may have resulted in a milder phenotype of rs121965088 through genetic interaction. Materials and Methods: the whole-exome sequencing analysis of samples obtained from the patient and her family members (father, mother, and brother) was performed. To identify the underlying genetic cause of XPC, the extracted DNA was analyzed using Agilent's SureSelect XT Human All Exon v5. The functional effects of the resultant variants were predicted using the SNPinfo web server, and structural changes in the XPC protein using the 3D protein modeling program SWISS-MODEL. Results: Eight biallelic variants, homozygous in the patient and heterozygous in her parents, were detected. Four were found in the XPC gene: one nonsense variant (rs121965088: c.1735C > T, p.Arg579Ter) and three silent variants (rs2227998: c.2061G > A, p. Arg687Arg; rs2279017: c.2251-6A > C, intron; rs2607775: c.-27G > C, 5'UTR). The remaining four variants were found in non-XP genes, including one frameshift variant [rs72452004 of olfactory receptor family 2 subfamily T member 35 (OR2T35)], three missense variants [rs202089462 of ALF transcription elongation factor 3 (AFF3), rs138027161 of TCR gamma alternate reading frame protein (TARP), and rs3750575 of annexin A7 (ANXA7)]. Conclusions: potential candidates for genetic interactions with rs121965088 were found. The rs2279017 and rs2607775 of XPC involved mutations in the intron region, which affected RNA splicing and protein translation. The genetic variants of AFF3, TARP, and ANXA7 are all frameshift or missense mutations, inevitably disturbing the translation and function of the resultant proteins. Further research on their functions in DNA repair pathways may reveal undiscovered cellular relationships within xeroderma pigmentosum.
  
  
  
  
  

---

  

# BTG1

**Signature gene in:** CD8+ T cells, Tregs
  
**Differentially expressed in:** no cell type
  
  
**BTG1**
-
1
of
10
  

### Rituximab-Chidamide combination chemotherapy enhances autophagy to overcome drug resistance in diffuse large B-cell lymphoma.

1. Int Immunopharmacol. 2025 May 27;156:114578. doi: 10.1016/j.intimp.2025.114578 Epub 2025 Apr 19.
  
  
Wu Z(1), Wang D(1), Fu D(2), Ning D(3), Gu S(4).
  
  
Author information: (1)Department of Hematology, General Hospital of Northern Theater Command, Sheny ...
  
  
Diffuse large B-cell lymphoma (DLBCL) is a challenging malignancy, particularly when resistance to standard therapies such as Rituximab develops. This study investigates the combined therapeutic effects of Rituximab and Chidamide on DLBCL, focusing on drug resistance mechanisms and autophagy regulation. Using high-throughput proteomics and transcriptomic analyses, key proteins and signaling pathways were identified. BTG1 emerged as a signature gene, while autophagy-related genes such as BECN1, ATG5, HSPA8, PTEN, and MAPK8 were highlighted as pivotal players. In vitro experiments using Rituximab-sensitive and -resistant DLBCL cell lines (Raji and Raji-4RH) demonstrated that Chidamide significantly inhibited cell proliferation in a dose- and time-dependent manner, induced G0/G1 phase arrest, and enhanced autophagy. Mechanistically, Chidamide upregulated histone acetylation and autophagy-related proteins while reducing p62 levels, synergistically promoting autophagy with Rituximab. In vivo mouse models confirmed the combined treatment's efficacy in suppressing tumor growth. These findings suggest that the BTG1/BECN1/ATG5 signaling axis plays a critical role in enhancing autophagy and reversing Rituximab resistance. The combination of Chidamide and Rituximab presents a promising therapeutic strategy, offering new insights into overcoming drug resistance in DLBCL.
  
  
  
  
**BTG1**
-
2
of
10
  

### Single-cell sequencing exposes mast cell-derived CD52's anti-tumor action in breast cancer through the IL-6/JAK/STAT3 axis.

2. Int J Biol Macromol. 2025 Apr 5;310(Pt 3):142879. doi: 10.1016/j.ijbiomac.2025.142879 Online ahead of print.
  
  
Jia Y(1), Peng Z(1), Tian X(1), Guan Y(2), Han Y(1), Ji D(1), Lan B(1), Xu B(3), Fan Y(4).
  
  
Author information: (1)Department of Medical Oncology, National Cancer Center/National Clinical Rese ...
  
  
The aggressive nature and rapid progression of triple-negative breast cancer (TNBC), coupled with a high likelihood of recurrence and mortality, underscore the critical need for effective treatments. While immunotherapy presents promising advantages for those with triple-negative breast cancer (TNBC), its efficacy is not universal. This disparity highlights the importance of investigating survival outcomes and prognostic factors for those TNBC patients who don't respond well to immunotherapy. Our study leverages both bulk and single-cell RNA sequencing data to conduct an in-depth analysis, revealing that genes associated with mast cells (PCMT1, VDAC1, YWHAB, BRD4, BTG1, and CD52) are pivotal in prognostication for TNBC patients. Laboratory experiments have further substantiated our findings, demonstrating that the overexpression of CD52 in mast cells impedes the proliferation, invasion, and metastasis of breast cancer cells. Further anti-CD52 treatment inhibiting breast tumor growth in vivo. Additionally, we have discovered that CD52 elicits its antitumor effects by meditating the IL-6/JAK/STAT3 signaling pathway. These insights not only enhance the prognostic significance of mast cells in TNBC but also pave the way for the development of novel targeted immunotherapy strategies.
  
  
  
  
**BTG1**
-
3
of
10
  

### Factors affecting chemotherapy response after the first relapse of B-cell acute lymphoblastic leukemia in pediatric patients.

3. Am J Transl Res. 2025 Feb 15;17(2):897-912. doi: 10.62347/YDNO1939 eCollection 2025.
  
  
Dong SW(1), Zhang H(2), Wang J(3), Huang SY(4), Wang HC(5).
  
  
Author information: (1)Department of Pediatrics, The First Affiliated Hospital of Xinjiang Medical U ...
  
  
OBJECTIVE: To investigate the factors affecting chemotherapy efficacy following first relapse in pediatric B-cell acute lymphoblastic leukemia (B-ALL). METHODS: A retrospective investigation was conducted on the clinical data from 254 pediatric patients with B-ALL treated at the First Affiliated Hospital of Xinjiang Medical University, Red Star Hospital of the 13th Division of Xinjiang Production and Construction Corps and Chengdu Women's and Children's Central Hospital between August 2016 and September 2022. Patients were divided into a Good Response (GR) group and a Poor Response (PR) group based on minimal residual disease (MRD) levels post-relapse treatment. The demographic data, blood and cytokine profiles, cytogenetic/molecular alterations, and therapeutic interventions were analyzed. Factors influencing response were screened using univariate and multivariate logistic regression models. RESULTS: The GR group showed significantly higher white blood cell (WBC) counts (8.24 ± 2.21 × 10^3^/µL) compared to the PR group (7.50 ± 1.88 × 10^3^/µL; P = 0.004). Elevated levels of tumor necrosis factor-alpha (TNF-α) (22.78 ± 4.31 vs. 20.94 ± 4.28 pg/mL; P < 0.001) and interleukin-6 (IL-6) (112.48 ± 21.09 vs. 106.31 ± 20.77 pg/mL; P = 0.020) were linked to poor outcome. Hypodiploidy and combined genetic alterations in Ikaros family zinc finger 1 (IKZF1), nuclear receptor subfamily 3 group C member 1 (NR3C1), and B-cell translocation gene 1 (BTG1) were associated with poor response (P = 0.032 and P = 0.003, respectively). Blinatumomab usage was associated with improved outcome (P = 0.030). Multivariate analysis revealed that higher TNF-α and IL-6 levels were independent risk factors of PR, while higher WBC count was a protective factor. CONCLUSION: Chemotherapy efficacy in relapsed pediatric B-ALL is influenced by hematologic, cytokine, and genetic factors.
  
  
  
  
**BTG1**
-
4
of
10
  

### Evaluation of the clinical significance of BTG1 gene expression and pepsinogen in serum and cancerous tissue and gastric atrophy.

4. Arch Physiol Biochem. 2025 Feb 23:1-10. doi: 10.1080/13813455.2025.2458560 Online ahead of print.
  
  
Paridar Y(1), Hosseinpour H(2), Mard-Soltani M(3), Pouria Mehr S(3), Shakerian N(3), Alinezhad Dezfu ...
  
  
Author information: (1)Gastroenterology Clinic, Dezful University of Medical Sciences, Dezful, Iran. ...
  
  
INTRODUCTION: This study aimed to assess the expression changes of BTG1, PGI, and PGII in tissues and serum of patients with gastric cancer, atrophic gastritis, and healthy individuals. METHODS: QRT-PCR was used to measure BTG1, PGI, and PGII expression in 30 cancers, 30 atrophic gastritis, and 30 healthy tissue samples. Serum levels of PGI and PGII were measured using ELISA. Statistical tests included the Mann-Whitney U and independent T-test. Covariates like tumour stage and H. pylori status were considered. RESULTS: BTG1 expression was significantly lower in cancer and gastritis tissues. Serum PGI and PGII levels were significantly reduced in cancer patients (P ≤ 0.001). DISCUSSION: The PGI/PGII ratio in serum emerged as a strong non-invasive biomarker for distinguishing cancer from healthy individuals. While BTG1 provides insights into gastric carcinogenesis, its clinical utility is limited due to the need for tissue samples. The serum-based PGI/PGII ratio shows greater promise as a non-invasive screening tool for GC.
  
  
  
  
**BTG1**
-
5
of
10
  

### miRNA-503 inhibition exerts anticancer effects and reduces tumor growth in mesothelioma.

5. J Exp Clin Cancer Res. 2025 Feb 22;44(1):65. doi: 10.1186/s13046-025-03283-0
  
  
Piccioni M(#)(1), Di Meo F(#)(1)(2), Valentino A(#)(3), Campani V(#)(4), Arigoni M(5), Tanori M(6), ...
  
  
Author information: (1)Institute of Biosciences and Bio-Resources, CNR, Naples, Italy. (2)Aix Marsei ...
  
  
BACKGROUND: Malignant mesothelioma (MM) is a rare and aggressive form of cancer that affects the mesothelial surfaces, associated with exposure to asbestos fibres. To date, no cure is available for MM and therapeutically approved treatments are based on the use of platinum compounds often used in combination with other drugs. We have previously analysed the efficacy of a cisplatin/piroxicam (CDDP/P) combined treatment showing that this treatment was able to reduce in vivo tumor growth. Several studies reported that platinum-drug sensitivity in cancer is connected to modulation of the expression of non-coding RNAs. In this study we analysed if the CDDP/P treatment was able to modulate miRNAs expression in MM. METHODS: miRNA sequencing performed on MSTO-211 H cells treated with CDDP with CDDP/P led us to identify miRNA-503 - downregulated by CDDP/P - as a novel miRNA that acts as an oncomiR in MM. The effect of miRNA-503 inhibition was evaluated in vitro in mesothelioma cells analysing apoptosis induction and reduction of cancer properties. Inhibition of miR-503 expression in vivo, was analysed in ectopic mouse model of MM by using LNP encapsulating anti-mir-503 and miR-503 expression was evaluated in human MM samples. RESULTS: In vitro and in vivo analysis confirmed miR-503 acts as oncogene in MM since its inhibition was able to reduce cell cancer properties and tumor growth in ectopic mouse model of MM. Its expression was found upregulated in human MM patients compared to normal pleura. Bioinformatic analysis indicated BTG1, CCNG1, EDG1, and TIMP2 as putative target genes of miRNA-503. These genes showed an opposite expression compared to miR-503 levels both in cells and in MM samples. Finally, microarray analysis indicated that miR-503 inhibition affected the expression of the well-known MM biomarkers: CXCL8, SERPINE1 and Osteopontin. CONCLUSIONS: Our study is the first reporting an oncomiR role for miR-503 in MM and suggests that its inactivation could have a clinical value in MM patients. This study reveals that miRNA-503 acts as an oncomiR in MM suggesting that its inhibition, through LNP delivery, has the potential to be considered as a novel therapeutic strategy in MM.
  
  
  
  
**BTG1**
-
6
of
10
  

### Single-cell transcriptomics analysis reveals dynamic changes and prognostic signature in tumor microenvironment of PDAC.

6. Sci Rep. 2025 Feb 2;15(1):4025. doi: 10.1038/s41598-025-86950-8
  
  
Li Y(#)(1), Ding Z(#)(2), Cheng T(1), Hu Y(1), Zhong F(3), Ren S(4), Wang S(5).
  
  
Author information: (1)School of Life Science and Food Engineering, Huaiyin Institute of Technology, ...
  
  
Pancreatic ductal adenocarcinoma (PDAC) is a highly lethal malignant tumor characterized by a complex tumor microenvironment (TME) with significant heterogeneity, posing immense challenges for devising effective therapeutic strategies. This study aims to elucidate the dynamic changes in the TME during PDAC progression and develop a prognostic model using single-cell RNA sequencing (scRNA-seq) data. We utilized a previously published comprehensive dataset comprising 31 samples (including 8 PDAC I, 9 PDAC II, 6 PDAC III, and 8 PDAC IV) to characterize the changes in TME composition with PDAC progression through advanced scRNA-seq analysis. We found that as cancer progresses, immune cells gradually become a predominant component in late-stage PDAC. We defined a novel Treg and exhausted T cell signature gene, TNFRSF4. Additionally, we identified a prognostic gene set (RPS10, MIF, MT-ATP6, CSTB, IFI30, NPC2, BTG1, CTSD, FCGR2A, SEC61G, IER3, HSPB1, HMOX1, and ZFP36L1) and differentiated high-risk from low-risk PDAC patients based on median risk score threshold. Based on these findings, we developed a novel prognostic model that identifies poorer prognosis in high-risk groups. Furthermore, our analysis revealed significant interactions between cells at different stages of PDAC and identified three promising therapeutic agents (XR-11576, Ixabepilone, and AMONAFIDE) based on correlated genes. Finally, molecular docking studies validated their potential by confirming stable binding with key protein targets. This study not only provides insights into the evolving TME of PDAC but also offers a new prognostic model and potential therapeutic strategies, contributing to improved management and treatment of this aggressive cancer.
  
  
  
  
**BTG1**
-
7
of
10
  

### Comparison of differences in transcriptional and genetic profiles between intra-central nervous system and extra-central nervous system large B-cell lymphoma.

7. Neoplasia. 2025 Feb;60:101119. doi: 10.1016/j.neo.2024.101119 Epub 2024 Dec 28.
  
  
Wang S(1), Chen H(2), Dai B(1), Zheng K(3), Zheng J(3), Zhu Y(4), Yuan Y(1), Ding T(1), Wang Q(1), X ...
  
  
Author information: (1)Department of Hematology, Huashan Hospital, Fudan University, Shanghai 200040 ...
  
  
Primary central nervous system diffused large B-cell lymphoma (PCNS-DLBCL) is a rare type of non-Hodgkin lymphoma restricted to the central nervous system (CNS). To explore its specific pathogenesis and therapeutic targets, we performed multi-omics sequencing on tumor samples from patients diagnosed with PCNS-DLBCL, secondary CNS-DLBCL or extracranial (ec) DLBCL.By single-cell RNA sequencing, highly proliferated and dark zone (DZ)-related B cell subclusters, MKI67\_B1, PTTG1\_B2 and BTG1\_B3, were predominant significantly in PCNS-DLBCL. Compared to SCNS-DLBCL and ecDLBCL, an immune-suppressive tumor microenvironment was observed in PCNS-DLBCL by analysis of immune-stimulating/inhibitory ligand‒receptor (L-R) pairs. By performing whole-exome sequencing in 93 patients, mutations enriched in BCR-NFkB and TLR pathways and the cooperation of these two pathways were found to be predominant in PCNS-DLBCL comparing to nonGCB-ecDLBCL. In summary, our study provides comprehensive insights into the transcriptomic and genetic characteristics of PCNS-DLBCL in contrast to ecDLBCL and will help dissect the oncogenic mechanism of this disease.
  
  
  
  
**BTG1**
-
8
of
10
  

### Identification of genomic biomarkers of disease progression and survival in primary CNS lymphoma.

8. Blood Adv. 2025 Mar 11;9(5):1117-1131. doi: 10.1182/bloodadvances.2024014460
  
  
Geng H(1)(2), Mo S(3), Chen L(4), Ballapuram A(4), Tsang M(5), Lu M(4), Rauschecker AM(6), Wen KW(2) ...
  
  
Author information: (1)Department of Laboratory Medicine, University of California, San Francisco, S ...
  
  
The determination of the genetic subtypes of primary central nervous system lymphoma (PCNSL) and their relationship to differential chemoimmunotherapeutic response has not been established. There is a particular need for genomic biomarkers that identify patients with newly diagnosed PCNSL at high risk of early progression and death. We applied targeted next-generation sequencing for detection of recurrent single-nucleotide variants, copy number alterations, and zygosity abnormalities in diagnostic specimens from 78 patients with PCNSL treated with a standard methotrexate-based regimen, to identify prognostically significant molecular subgroups. All patients received induction immunochemotherapy, and 44 proceeded to dose-intensive consolidation. Genomic aberrations at 4 loci were associated with 91% of lymphoma progression events and all 15 deaths: (1) chromosome 6p copy-neutral loss of heterozygosity (CN-LOH) or focal homozygous deletion (HD) at 6p21.3, and mutations of tumor suppressor genes (2) BTG1, (3) ETV6, and (4) TP53. Cox regression multivariate analysis demonstrated a high risk of progression in patients with aberrations at these loci. Genomic aberrations at these loci were also associated with significantly shorter survival. Lower expression of HLA-DR was associated with 6p CN-LOH/6p21.3 HD and inferior prognosis. These genomic aberrations identify a high-risk molecular subgroup that may inform risk stratification in PCNSL. Further elucidation of the mechanisms of therapeutic resistance associated with the high-risk genetic phenotype is requisite to facilitate precision medicine and progress in therapy.
  
  
  
  
**BTG1**
-
9
of
10
  

### [Efficacy and safety analysis of the OR-CHOP regimen for the treatment of MCD subtype diffuse large B cell lymphoma in the real-world setting].

9. Zhonghua Xue Ye Xue Za Zhi. 2024 Sep 14;45(9):827-831. doi: 10.3760/cma.j.cn121090-20240607-00212
  
  
[Article in Chinese; Abstract available in Chinese from the publisher]
  
  
Yin H(1), Hua W(1), Shen HR(1), Wu JZ(1), Li Y(1), Wang L(1), Liang JH(1), Li JY(1), Xu W(1).
  
  
Author information: (1)The First Affiliated Hospital of Nanjing Medical University (Department of Hematology, Jiangsu Province Hospital), Nanjing 210029, China.
  
  
  
  
**BTG1**
-
10
of
10
  

### Unraveling Copy Number Alterations in Pediatric B-Cell Acute Lymphoblastic Leukemia: Correlation with Induction Phase Remission Using MLPA.

10. Asian Pac J Cancer Prev. 2024 Jul 1;25(7):2421-2426. doi: 10.31557/APJCP.2024.25.7.2421
  
  
Aisyi M(1), Andriastuti M(2), Kosasih AS(3), Utomo ARH(4), Saputra F(5), Tjitra Sari T(2), Sjakti HA ...
  
  
Author information: (1)Department of Pediatric Hematology-Oncology, Dharmais Cancer Center Hospital. ...
  
  
OBJECTIVE: Acute Lymphoblastic Leukemia (ALL) is the most common malignancy occurring in children. Copy number alterations (CNA) like PAX5, CDKN2A/2B, PAR1 Region, ETV6, IKZF1, BTG1, and RB1 gene deletion are important genetic events that define and prognosticate B-cell ALL. Thus, this study aimed to evaluate associations of CNA with induction phase remission status in childhood B-cell ALL. METHODS: This study was observational with a cross-sectional design at the Dharmais Cancer Hospital, Harapan Kita Mother and Children Hospital, and Tangerang Regional Public Hospital. We evaluated 74 pediatric B-cell ALL cases with 1-18-year-olds. Genomic DNA was analyzed by Multiplex Ligation Dependent Probe Amplification Assay (MLPA). This study used the P335 ALL-IKZF1 panel kit, which contains several ALL-related genes. The patient's clinical and laboratory characteristics were collected from medical records from January to December 2019. RESULT: We observed gene copy number alteration in children with B-Cell ALL. PAX5 was the most commonly observed gene deletion, followed by CDKN21/2B, ETV6, IKZF1, BTG1, RB1, and PAR1 Region. Based on gene mutations, only the PAX5 had a significant association with the remission status of pediatric B-cell ALL (p-value <0.05; OR = 3.91). It showed that patients with PAX5 gene mutations have 3.9 times the risk of no remission and/or relapse compared to those without PAX5 gene mutations. CONCLUSION: Patients with mutations in the PAX5 gene have a higher chance of not achieving remission and/or experiencing relapse than those without such mutations. The MLPA method can be utilized for examining copy number alterations, which is valuable for achieving more precise stratification in diagnosis.. Further research is needed to expand upon this finding.
  
  
  
  
  

---

  

# CHD2

**Signature gene in:** Dendritic cells
  
**Differentially expressed in:** no cell type
  
  
**CHD2**
-
1
of
10
  

### In Vivo Multiplexed Modeling Reveals Diverse Roles of the TBX2 Subfamily and Egr1 in Ras -Driven Lung Adenocarcinoma.

1. bioRxiv [Preprint]. 2025 Mar 17:2025.03.15.642187. doi: 10.1101/2025.03.15.642187
  
  
Khalil A, Dinh T, Parks M, Obeng RC, Gryder B, Kresak A, Wang Y, Maltas J, Bedrock M, Wei X, Faber Z ...
  
  
The TBX2 subfamily of T-box transcription factors (including Tbx2 , Tbx3 , Tbx4 , Tbx5 ) plays an es ...
  
  
DOI: 10.1101/2025.03.15.642187 PMCID: PMC11956923 PMID: 40166332
  
  
  
  
**CHD2**
-
2
of
10
  

### Pilot study: Initial investigation suggests differences in EMT-associated gene expression in breast tumor regions.

2. Comput Struct Biotechnol J. 2025 Jan 30;27:548-555. doi: 10.1016/j.csbj.2025.01.027 eCollection 2025.
  
  
King KL(1), Abdollahi H(2), Dinkel Z(1), Akins A(1), Valafar H(2), Dunn H(1).
  
  
Author information: (1)Department of Bioengineering, Clemson University, Clemson, SC, USA. (2)Depart ...
  
  
Triple negative breast cancer (TNBC) is the most aggressive subtype and disproportionately affects African American women. The development of breast cancer is highly associated with interactions between tumor cells and the extracellular matrix (ECM), and recent research suggests that cellular components of the ECM vary between racial groups. This pilot study aimed to evaluate gene expression in TNBC samples from patients who identified as African American and Caucasian using traditional statistical methods and emerging Machine Learning (ML) approaches. ML enables the analysis of complex datasets and the extraction of useful information from small datasets. We selected four regions of interest from tumor biopsy samples and used laser microdissection to extract tissue for gene expression characterization via RT-qPCR. Both parametric and non-parametric statistical analyses identified genes differentially expressed between the two ethnic groups. Out of 40 genes analyzed, 4 were differentially expressed in the edge of tumor (ET) region and 8 in the ECM adjacent to the tumor (ECMT) region. In addition to statistical approach, ML was used to generate decision trees (DT) for a broader analysis of gene expression and ethnicity. Our DT models achieved 83.33 % accuracy and identified the most significant genes, including CD29 and EGF from the ET region and SNAI1 and CHD2 from the ECMT region. All significant genes were analyzed for pathway enrichment using MSigDB and Gene Ontology databases, most notably the epithelial to mesenchymal transition and cell motility pathways. This pilot study highlights key genes of interest that are differentially expressed in African American and Caucasian TNBC samples.
  
  
  
  
**CHD2**
-
3
of
10
  

### Molecular Profiling of Sinonasal Adenoid Cystic Carcinoma: Canonical and Noncanonical Gene Fusions and Mutation.

3. Am J Surg Pathol. 2025 Mar 1;49(3):227-242. doi: 10.1097/PAS.0000000000002349 Epub 2025 Jan 6.
  
  
Skálová A(1)(2), Bradová M(1)(2), Agaimy A(3), Laco J(4), Badual C(5), Ihrler S(6), Damjanov I(7), R ...
  
  
Author information: (1)Department of Pathology, Charles University, Faculty of Medicine in Pilsen. ( ...
  
  
Adenoid cystic carcinomas (AdCC) of salivary gland origin have long been categorized as fusion-defined carcinomas owing to the almost universal presence of the gene fusion MYB::NFIB , or less commonly MYBL1::NFIB. Sinonasal AdCC is an aggressive salivary gland malignancy with no effective systemic therapy. Therefore, it is urgent to search for potentially targetable genetic alterations associated with AdCC. We have searched the authors' registries and selected all AdCCs arising in the sinonasal tract. The tumors were examined histologically, immunohistochemically, by next generation sequencing (NGS) and/or fluorescence in situ hybridization (FISH) looking for MYB/MYBL1 and/or NFIB gene fusions or any novel gene fusions and/or mutations. In addition, all tumors were tested for HPV by genotyping using (q)PCR. Our cohort comprised 88 cases of sinonasal AdCC, predominantly characterized by canonical MYB::NFIB (49 cases) and MYBL1::NFIB (9 cases) fusions. In addition, noncanonical fusions EWSR1::MYB ; ACTB::MYB; ESRRG::DNM3 , and ACTN4::MYB were identified by NGS, each of them in 1 case. Among nine fusion-negative AdCCs, FISH detected rearrangements in MYB (7 cases) , NFIB (1 case), and EWSR1 (1 case). Six AdCCs lacked fusions or gene rearrangements, while 11 cases were unanalyzable. Mutational analysis was performed by NGS in 31/88 (35%) AdCCs. Mutations in genes with established roles in oncogenesis were identified in 21/31 tumors (68%), including BCOR (4/21; 19%), NOTCH1 (3/21; 14%), EP300 (3/21; 14%), SMARCA4 (2/21; 9%), RUNX1 (2/21; 9%), KDM6A (2/21; 9%), SPEN (2/21; 9%), and RIT1, MGA, RB1, PHF6, PTEN, CREBBP, DDX41, CHD2, ROS1, TAF1, CCD1, NF1, PALB2, AVCR1B, ARID1A, PPM1D, LZTR1, GEN1 , PDGFRA , each in 1 case (1/21; 5%). Additional 24 cases exhibited a spectrum of gene mutations of uncertain pathogenetic significance. No morphologic differences were observed between AdCCs with MYBL1::NFIB and MYB::NFIB fusions. Interestingly, mutations in the NOTCH genes were seen in connection with both canonical and noncanonical fusions, and often associated with high-grade histology or metatypical phenotype, as well as with poorer clinical outcome. Noncanonical fusions were predominantly observed in metatypical AdCCs. These findings emphasize the value of comprehensive molecular profiling in correlating morphologic characteristics, genetic landscape, and clinical behavior in AdCC.
  
  
  
  
**CHD2**
-
4
of
10
  

### Pathogenic variants of mycosis fungoides identified using next-generation sequencing.

4. J Hematop. 2024 Dec;17(4):209-214. doi: 10.1007/s12308-024-00607-5 Epub 2024 Sep 19.
  
  
Shrestha S(1), Newsom K(1), Chaffin JM(1), Seifert RP(2).
  
  
Author information: (1)Department of Pathology, Immunology and Laboratory Medicine, University of Fl ...
  
  
Mycosis fungoides (MF), the predominant form of cutaneous T-cell lymphoma (CTCL), poses diagnostic challenges due to its clinical and histological resemblance to benign skin disorders. Delayed diagnosis contributes to therapeutic delays, prompting exploration of advanced diagnostics tools. Next-generation sequencing (NGS) may enhance disease detection by identifying pathogenic variants common to CTCL but absent in benign inflammatory disorders. We aim to discuss novel and common pathogenic variants in CTCL to enhance the utility of NGS as a diagnostic adjunct. This pilot study employed (NGS) to identify pathogenic variants in 10 MF cases. Cases were selected based on PCR-confirmed T-cell receptor clonality, with adequate DNA for NGS. GatorSeq NGS Panel, Illumina NextSeq500, and QIAGEN Clinical Insight QCI software facilitated sequencing, analysis, and variant interpretation. NGS revealed eight novel mutations in genes including HLA-DRB1, AK2, ITPKB, HLA-B, TYRO3, and CHD2. Additionally, previously reported MF-associated mutations such as DNMT3A, STAT5B, and SOCS1 (mouse study only) were detected as well. Detected variants were involved in apoptotic, NF-kB, JAK-STAT, and TCR signaling pathways, providing insights into MF pathogenesis. Mutations in genes like APC, AK2, TYRO3, and ITPKB that regulate tumor proliferation and apoptosis were noted. MF cases were associated with HLA gene mutations. NGS may enhance MF diagnosis, as the detection of pathogenic variants, particularly those known to occur in MF, favors a neoplastic diagnosis over an inflammatory diagnosis. Continuing this work may lead to the discovery of therapeutic targets.
  
  
  
  
**CHD2**
-
5
of
10
  

### ALK-positive large B-cell lymphoma: a clinicopathological and molecular characteristics analysis of seven cases.

5. Pathology. 2024 Dec;56(7):961-968. doi: 10.1016/j.pathol.2024.05.014 Epub 2024 Aug 13.
  
  
Wang X(1), Yi H(2), Liu Q(1), Guo T(3), Li A(1), Ouyang B(1), Li Y(1), Zhang Y(1), Xu H(1), Dong L(1 ...
  
  
Author information: (1)Department of Pathology, Ruijin Hospital, Shanghai Jiao Tong University Schoo ...
  
  
Anaplastic lymphoma kinase-positive large B-cell lymphoma (ALK+ LBCL) is a rare and highly aggressive lymphoma with characteristic ALK rearrangements. Various fusion genes involving ALK have been demonstrated, but the influence of the ALK fusion partners on ALK protein expression and the genetic characteristics of ALK+ LBCL remain relatively unknown. In this study, we conducted an extensive clinicopathological and molecular analysis on seven cases of ALK+ LBCL to explore the correlation between ALK fusion genes and ALK protein expression, thereby enriching the genetic characteristics of this tumour. We integrated the findings from clinical, histopathological/immunophenotypic, and molecular studies, including three samples subjected to next-generation sequencing, and six cases underwent RNA-based ALK fusion gene detection. We identified five distinct types of ALK fusion genes, including CLTC, NPM1, PABPC1, SEC31A, and TFG. Notably, only the NPM1::ALK fusion showed nuclear and cytoplasmic ALK staining, and the remaining four fusion genes resulted in cytoplasmic ALK staining. Our analysis revealed that the CLTC::ALK fusion resulted in a unique cytoplasmic perinuclear Golgi zone focal granular heterogeneous staining pattern of ALK. Additionally, we identified six potentially clinically significant gene mutations, including TET2, CHD2, DTX1, KMT2D, LRP1B, and XPO1. Furthermore, in all cases, the absence of 5-hydroxymethylcytosine (5hmC) was observed. We present seven cases of ALK+ LBCL, discussing the correlation between fusion genes and ALK protein expression, and enhancing our understanding of the genetic attributes of this tumour. This study also shows the loss of 5hmC in nearly all seven ALK+ LBCL cases, independently of TET2 mutations.
  
  
  
  
**CHD2**
-
6
of
10
  

### Comprehensive Analysis of the Expression, Prognosis, and Immune Infiltrates for Chromodomain-Helicase-DNA-Binding Proteins in Breast Tumor.

6. Asian Pac J Cancer Prev. 2024 May 1;25(5):1547-1558. doi: 10.31557/APJCP.2024.25.5.1547
  
  
Hasanlu M(1), Amiri-Dashatan N(2), Farahani M(3), Koushki M(4), Ahmadi H(5)(6), Parsamanesh N(7), Ah ...
  
  
Author information: (1)Department of Internal Medicine, Vali-e-Asr Hospital, School of Medicine, Zan ...
  
  
BACKGROUND: Several recent studies suggest that chromodomain-helicase -DNA-binding domains (CHDs) are linked with cancers. We explored the association between chromodomain-Helicase-DNA-binding domain proteins and breast cancer (BrCa) and introduced potential prognostic markers using various databases. MATERIALS AND METHODS: We analyzed the expression of the CHD family and their prognostic value in BrCa by mining UALCAN, TIMER, and Kaplan-Meier plotter databases. The association of CHD expression and immune infiltrating abundance was studied via the TIMER database. In addition, microRNAs related to the CHD family were identified by using the MirTarBase online database. RESULTS: The present study indicated that compared to normal tissues, BrCa tissues showed increased mRNA levels of CHD3/4/7 but decreased CHD2/5/9 expression. Interestingly, We also found a positive correlation between CHD gene expression and the infiltration of macrophage, neutrophil, and dendritic cells in BrCa, except CHD3/5. The Kaplan-Meier Plotter analysis suggested that high expression levels of CHD1/2/3/4/6/8/9 were significantly related to shorter relapse-free survival (RFS), while higher mRNA expression of CHD1, CHD2, CHD8, and CHD9 was significantly associated with longer overall survival of BrCa patients. The miRNAs of hsa-miR-615-3p and hsa-let-7b-5p were identified as being more correlated with the CHD family. CONCLUSION: The altered expression of some CHD members was significantly related to clinical cancer outcomes, and CHD1/2/8/9 could serve as potential prognostic biomarkers to improve the survival of BrCa patients. However, to evaluate the studied CHD members in detail are needed further investigations including experimental validation.
  
  
  
  
**CHD2**
-
7
of
10
  

### CHD2 Regulates Neuron-Glioma Interactions in Pediatric Glioma.

7. Cancer Discov. 2024 Sep 4;14(9):1732-1754. doi: 10.1158/2159-8290.CD-23-0012
  
  
Zhang X(#)(1)(2)(3)(4), Duan S(#)(1)(2)(3)(4), Apostolou PE(5), Wu X(6), Watanabe J(7)(8), Gallitto ...
  
  
Author information: (1)Institute for Cancer Genetics, Columbia University Irving Medical Center, New ...
  
  
High-grade gliomas (HGG) are deadly diseases for both adult and pediatric patients. Recently, it has been shown that neuronal activity promotes the progression of multiple subgroups of HGG. However, epigenetic mechanisms that govern this process remain elusive. Here we report that the chromatin remodeler chromodomain helicase DNA-binding protein 2 (CHD2) regulates neuron-glioma interactions in diffuse midline glioma (DMG) characterized by onco-histone H3.1K27M. Depletion of CHD2 in H3.1K27M DMG cells compromises cell viability and neuron-to-glioma synaptic connections in vitro, neuron-induced proliferation of H3.1K27M DMG cells in vitro and in vivo, activity-dependent calcium transients in vivo, and extends the survival of H3.1K27M DMG-bearing mice. Mechanistically, CHD2 coordinates with the transcription factor FOSL1 to control the expression of axon-guidance and synaptic genes in H3.1K27M DMG cells. Together, our study reveals a mechanism whereby CHD2 controls the intrinsic gene program of the H3.1K27M DMG subtype, which in turn regulates the tumor growth-promoting interactions of glioma cells with neurons. Significance: Neurons drive the proliferation and invasion of glioma cells. Here we show that chromatin remodeler chromodomain helicase DNA-binding protein 2 controls the epigenome and expression of axon-guidance and synaptic genes, thereby promoting neuron-induced proliferation of H3.1K27M diffuse midline glioma and the pathogenesis of this deadly disease.
  
  
  
  
**CHD2**
-
8
of
10
  

### Genome-wide somatic mutation analysis of sinonasal adenocarcinoma with and without wood dust exposure.

8. Genes Environ. 2024 May 6;46(1):12. doi: 10.1186/s41021-024-00306-8
  
  
Sipilä LJ(1)(2)(3), Katainen R(1)(2)(4), Aavikko M(1)(2)(4), Ravantti J(1)(2)(5), Donner I(6), Lehto ...
  
  
Author information: (1)Department of Medical and Clinical Genetics, University of Helsinki, Biomedic ...
  
  
BACKGROUND: Sinonasal adenocarcinoma is a rare cancer, encompassing two different entities, the intestinal-type sinonasal adenocarcinoma (ITAC) and the non-intestinal-type sinonasal adenocarcinoma (non-ITAC). Occurrence of ITAC is strongly associated with exposure to hardwood dusts. In countries with predominant exposure to softwood dust the occurrence of sinonasal adenocarcinomas is lower and the relative amount of non-ITACs to ITACs is higher. The molecular mechanisms behind the tumorigenic effects of wood dust remain largely unknown. METHODS: We carried out whole-genome sequencing of formalin-fixed paraffin-embedded (FFPE) samples of sinonasal adenocarcinomas from ten wood dust-exposed and six non-exposed individuals, with partial tobacco exposure data. Sequences were analyzed for the presence of mutational signatures matching COSMIC database signatures. Driver mutations and CN variant regions were characterized. RESULTS: Mutation burden was higher in samples of wood dust-exposed patients (p = 0.016). Reactive oxygen species (ROS) damage-related mutational signatures were almost exclusively identified in ITAC subtype samples (p = 0.00055). Tobacco smoke mutational signatures were observed in samples of patients with tobacco exposure or missing information, but not in samples from non-exposed patients. A tetraploidy copy number (CN) signature was enriched in ITAC subtype (p = 0.042). CN variation included recurrent gains in COSMIC Cancer Gene Census genes TERT, SDHA, RAC1, ETV1, PCM1, and MYC. Pathogenic variants were observed most frequently in TP53, NF1, CHD2, BRAF, APC, and LRP1B. Driver mutations and copy number gains did not segregate by subtype. CONCLUSIONS: Our analysis identified distinct mutational characteristics in ITAC and non-ITAC. Mutational signature analysis may eventually become useful for documentation of occupation-related cancer, while the exact mechanisms behind wood dust-driven carcinogenesis remain elusive. The presence of homologous recombination deficiency signatures implies a novel opportunity for treatment, but further studies are needed.
  
  
  
  
**CHD2**
-
9
of
10
  

### A CIC-related-epigenetic factors-based model associated with prediction, the tumor microenvironment and drug sensitivity in osteosarcoma.

9. Sci Rep. 2024 Jan 15;14(1):1308. doi: 10.1038/s41598-023-49770-2
  
  
Yu B(1)(2), Geng C(3), Wu Z(3), Zhang Z(3), Zhang A(3), Yang Z(1), Huang J(1), Xiong Y(3), Yang H(4) ...
  
  
Author information: (1)Key Laboratory of Tumor Immunological Prevention and Treatment of Yunnan Prov ...
  
  
Osteosarcoma is generally considered a cold tumor and is characterized by epigenetic alterations. Although tumor cells are surrounded by many immune cells such as macrophages, T cells may be suppressed, be inactivated, or not be presented due to various mechanisms, which usually results in poor prognosis and insensitivity to immunotherapy. Immunotherapy is considered a promising anti-cancer therapy in osteosarcoma but requires more research, but osteosarcoma does not currently respond well to this therapy. The cancer immunity cycle (CIC) is essential for anti-tumor immunity, and is epigenetically regulated. Therefore, it is possible to modulate the immune microenvironment of osteosarcoma by targeting epigenetic factors. In this study, we explored the correlation between epigenetic modulation and CIC in osteosarcoma through bioinformatic methods. Based on the RNA data from TARGET and GSE21257 cohorts, we identified epigenetic related subtypes by NMF clustering and constructed a clinical prognostic model by the LASSO algorithm. ESTIMATE, Cibersort, and xCell algorithms were applied to analyze the tumor microenvironment. Based on eight epigenetic biomarkers (SFMBT2, SP140, CBX5, HMGN2, SMARCA4, PSIP1, ACTR6, and CHD2), two subtypes were identified, and they are mainly distinguished by immune response and cell cycle regulation. After excluding ACTR6 by LASSO regression, the prognostic model was established and it exhibited good predictive efficacy. The risk score showed a strong correlation with the tumor microenvironment, drug sensitivity and many immune checkpoints. In summary, our study sheds a new light on the CIC-related epigenetic modulation mechanism of osteosarcoma and helps search for potential drugs for osteosarcoma treatment.
  
  
  
  
**CHD2**
-
10
of
10
  

### Chronic Lymphocytic Leukemia IGHV Somatic Hypermutation Detection by Targeted Capture Next-Generation Sequencing.

10. Clin Chem. 2024 Jan 4;70(1):273-284. doi: 10.1093/clinchem/hvad147
  
  
Grants JM(1), May C(1), Bridgers J(1), Huang S(1), Gillis S(1), Meissner B(2), Boyle M(2), Ben-Neria ...
  
  
Author information: (1)Michael Smith Genome Sciences Centre, BC Cancer, Vancouver, BC, Canada. (2)Ce ...
  
  
BACKGROUND: Somatic hypermutation (SHM) status of the immunoglobulin heavy variable (IGHV) gene plays a crucial role in determining the prognosis and treatment of patients with chronic lymphocytic leukemia (CLL). A common approach for determining SHM status is multiplex polymerase chain reaction and Sanger sequencing of the immunoglobin heavy locus; however, this technique is low throughput, is vulnerable to failure, and does not allow multiplexing with other diagnostic assays. METHODS: Here we designed and validated a DNA targeted capture approach to detect immunoglobulin heavy variable somatic hypermutation (IGHV SHM) status as a submodule of a larger next-generation sequencing (NGS) panel that also includes probes for ATM, BIRC3, CHD2, KLHL6, MYD88, NOTCH1, NOTCH2, POT1, SF3B1, TP53, and XPO1. The assay takes as input FASTQ files and outputs a report containing IGHV SHM status and V allele usage following European Research Initiative on CLL guidelines. RESULTS: We validated the approach on 35 CLL patient samples, 34 of which were characterized using Sanger sequencing. The NGS panel identified the IGHV SHM status of 34 of 35 CLL patients. We showed 100% sensitivity and specificity among the 33 CLL samples with both NGS and Sanger sequencing calls. Furthermore, we demonstrated that this panel can be combined with additional targeted capture panels to detect prognostically important CLL single nucleotide variants, insertions/deletions, and copy number variants (TP53 copy number loss). CONCLUSIONS: A targeted capture approach to IGHV SHM detection can be integrated into broader sequencing panels, allowing broad CLL prognostication in a single molecular assay.
  
  
  
  
  

---

  

# AC006160.1

**Signature gene in:** Stromal cells
  
**Differentially expressed in:** Stromal cells
  
  
**AC006160.1**
-
1
of
4
  

### Construction and validation of a prognostic model for bladder cancer based on disulfidptosis-related lncRNAs.

1. Medicine (Baltimore). 2024 Jul 5;103(27):e38750. doi: 10.1097/MD.0000000000038750
  
  
Yang X(1), Zhang Y(2), Liu J(1), Feng Y(1).
  
  
Author information: (1)Department of Urology, Suining Central Hospital, Suining, Sichuan, China. (2) ...
  
  
BACKGROUND: Bladder cancer (BLCA) is a prevalent and aggressive cancer associated with high mortality and poor prognosis. Currently, studies on the role of disulfidptosis-related long non-coding RNAs (DRLs) in BLCA are limited. This study aims to construct a prognostic model based on DRLs to improve the accuracy of survival predictions for patients and identify novel targets for therapeutic intervention in BLCA management. METHODS: Transcriptomic and clinical datasets for patients with BLCA were obtained from The Cancer Genome Atlas. Using multivariate Cox regression and least absolute shrinkage and selection operator techniques, a risk prognostic signature defined by DRLs was developed. The model's accuracy and prognostic relevance were assessed through Kaplan-Meier survival plots, receiver operating characteristic curves, concordance index, and principal component analysis. Functional and pathway enrichment analyses, including Gene Ontology, Kyoto Encyclopedia of Genes and Genomes, and Gene Set Enrichment Analysis, were conducted to elucidate the underlying biological processes. Immune cell infiltration was quantified using the CIBERSORT algorithm. Differences and functions of immune cells in different risk groups were evaluated through single-sample Gene Set Enrichment Analysis. The Tumor Immune Dysfunction and Exclusion predictor and tumor mutational burden (TMB) assessments were utilized to gauge the likelihood of response to immunotherapy. Drug sensitivity predictions were made using the Genomics of Drug Sensitivity in Cancer database. RESULTS: A robust 8-DRL risk prognostic model, comprising LINC00513, SMARCA5-AS1, MIR4435-2HG, MIR4713HG, AL122035.1, AL359762.3, AC006160.1, and AL590428.1, was identified as an independent prognostic indicator. This model demonstrated strong predictive power for overall survival in patients with BLCA, revealing significant disparities between high- and low-risk groups regarding tumor microenvironment, immune infiltration, immune functions, TMB, Tumor Immune Dysfunction and Exclusion scores, and drug susceptibility. CONCLUSION: This study introduces an innovative prognostic signature of 8 DRLs, offering a valuable prognostic tool and potential therapeutic targets for bladder carcinoma. The findings have significant implications for TMB, the immune landscape, and patient responsiveness to immunotherapy and targeted treatments.
  
  
  
  
**AC006160.1**
-
2
of
4
  

### Ferroptosis-related lncRNAs: Distinguishing heterogeneity of the tumour microenvironment and predicting immunotherapy response in bladder cancer.

2. Heliyon. 2024 May 31;10(11):e32018. doi: 10.1016/j.heliyon.2024.e32018 eCollection 2024 Jun 15.
  
  
Yang Z(1), Li X(1), Zhou L(1), Luo Y(2), Zhan N(2), Ye Y(1), Liu Z(2), Zhang X(2), Qiu T(2), Lin L(2 ...
  
  
Author information: (1)Department of Urology, The First Affiliated Hospital of Wenzhou Medical Unive ...
  
  
Ferroptosis, a cell death pathway dependent on iron, has been shown in research to play a role in the development, advancement, and outlook of tumours through ferroptosis-related lncRNAs (FRLRs). However, the value of the FRLRs in bladder cancer (BLCA) has not been thoroughly investigated. This research project involved developing a predictive model using ten specific FRLRs (AC099850.4, AL731567.1, AL133415.1, AC021321.1, SPAG5-AS1, HMGA2-AS1, RBMS3-AS3, AC006160.1, AL583785.1, and AL662844.4) through univariate COX and LASSO regression techniques. The validation of this signature as a standalone predictor was confirmed in a group of 65 patients from the urology bladder tumour database at the First Affiliated Hospital of Wenzhou Medical University in Wenzhou, China. Patients were categorized based on their median risk score into either a low-risk group or a high-risk group. Enrichment analysis identified possible molecular mechanisms that could explain the variations in clinical outcomes observed in high-risk and low-risk groups. Moreover, we explored the correlation between FLPS and immunotherapy-related indicators. The ability of FLPS to forecast the effectiveness of immunotherapy was validated by the elevated levels of immune checkpoint genes (PD-L1, CTLA4, and PD-1) in the group at high risk. We also screened the crucial FRLR (HMGA2-AS1) through congruent expression and prognostic conditions and established a ceRNA network, indicating that HMGA2-AS1 may affect epithelial-mesenchymal transition by modulating the Wnt signalling pathway through the ceRNA mechanism. We identified the top five mRNAs (NFIB, NEGR1, JAZF1, JCAD, and ESM1) based on random forest algorithm and analysed the relationship between HMGA2-AS1, the top five mRNAs, and immunotherapy, and their interactions with drug sensitivities. Our results suggest that patients with BLCA have a greater sensitivity to four drugs (dasatinib, pazopanib, erismodegib and olaparib). Our study provides new insights into the TME, key signalling pathways, genome, and potential therapeutic targets of BLCA, with future guidance for immunotherapy and targeted precision drugs.
  
  
  
  
**AC006160.1**
-
3
of
4
  

### Metformin and long non-coding RNAs in breast cancer.

3. J Transl Med. 2023 Feb 27;21(1):155. doi: 10.1186/s12967-023-03909-x
  
  
Gholami M(1)(2), Klashami ZN(1), Ebrahimi P(3), Mahboobipour AA(4), Farid AS(2), Vahidi A(1), Zoughi ...
  
  
Author information: (1)Metabolic Disorders Research Center, Endocrinology and Metabolism Molecular-C ...
  
  
Breast cancer (BC) is the second most common cancer and cause of death in women. In recent years many studies investigated the association of long non-coding RNAs (lncRNAs), as novel genetic factors, on BC risk, survival, clinical and pathological features. Recent studies also investigated the roles of metformin treatment as the firstline treatment for type 2 diabetes (T2D) played in lncRNAs expression/regulation or BC incidence, outcome, mortality and survival, separately. This comprehensive study aimed to review lncRNAs associated with BC features and identify metformin-regulated lncRNAs and their mechanisms of action on BC or other types of cancers. Finally, metformin affects BC by regulating five BC-associated lncRNAs including GAS5, HOTAIR, MALAT1, and H19, by several molecular mechanisms have been described in this review. In addition, metformin action on other types of cancers by regulating ten lncRNAs including AC006160.1, Loc100506691, lncRNA-AF085935, SNHG7, HULC, UCA1, H19, MALAT1, AFAP1-AS1, AC026904.1 is described.
  
  
  
  
**AC006160.1**
-
4
of
4
  

### Ferroptosis-Related Long Noncoding RNAs Have Excellent Predictive Ability for Multiomic Characteristics of Bladder Cancer.

4. Oxid Med Cell Longev. 2022 Aug 29;2022:9316847. doi: 10.1155/2022/9316847 eCollection 2022.
  
  
Liu J(1)(2), Cui J(2)(3), Zhao S(1), Wu M(1), Wang J(1)(2), Zhang Y(1)(2), Jin B(1)(2), Wang J(1)(2) ...
  
  
Author information: (1)Department of Urology, Beijing Hospital, National Center of Gerontology, Inst ...
  
  
BACKGROUND: The role of ferroptosis-related long non-coding RNAs (lncRNAs) in bladder cancer remains elusive. This study is aimed at examining the prognostic role of ferroptosis-related lncRNAs in bladder cancer. MATERIALS AND METHODS: The transcriptomic matrix and clinical information of patients with bladder cancer were obtained from The Cancer Genome Atlas (TCGA) database. A ferroptosis-related lncRNA signature was developed via the least absolute shrinkage and selection operator (LASSO) analysis using data from the training cohort, and the signature was further validated using data from the test cohort. The role of AC006160.1, the most significant lncRNA in the risk signature, was examined in various cell lines including SV-HUC-1, BIU-87, HT-1376, T24, RT4, RT-112, 5637, and UMUC3. The pcDNA3.1-AC006160.1 plasmid was constructed and transfected into the bladder cancer cell lines T24 and BIU-87. In addition, cell proliferation, colony formation, transwell, and wound healing assays were performed to examine the biological function of AC006160.1 in T24 and BIU-87 cell lines. RESULTS: Two clusters were identified through consensus clustering based on prognostic ferroptosis-related lncRNAs. A 5-lncRNA risk signature was successfully constructed using data from the training cohort and validated using data from the test cohort. The risk signature had excellent ability to predict survival outcomes, clinical stages, pathological grades, expression of immune checkpoints, and immunotherapeutic responses in bladder cancer samples. Furthermore, AC006160.1 expression was found to be lower in the cancer cell lines BIU-87, T24, RT4, RT-112, and 5637 than in the normal control cell line SV-HUC-1. Cell proliferation, colony formation, transwell migration, and wound healing assays validated that overexpression of AC006160.1 significantly inhibited the proliferation and invasion abilities of both T24 and BIU-87 cells. Drug sensitivity analysis revealed that patients with high expression of AC006160.1 were sensitive to metformin and methotrexate, and the results were further validated via in vitro drug experiments. CONCLUSIONS: Ferroptosis-related lncRNAs play a vital role in predicting the multiomic characteristics of bladder cancer. The lncRNA AC006160.1 serves as a protective factor for the development of bladder cancer.
  
  
  
  
  

---

  

# AHDC1

**Signature gene in:** Stromal cells
  
**Differentially expressed in:** Stromal cells
  
  
**AHDC1**
-
1
of
7
  

### Using evolutionary constraint to define novel candidate driver genes in medulloblastoma.

1. Proc Natl Acad Sci U S A. 2023 Aug 15;120(33):e2300984120. doi: 10.1073/pnas.2300984120 Epub 2023 Aug 7.
  
  
Roy A(#)(1), Sakthikumar S(#)(2)(3), Kozyrev SV(2), Nordin J(1)(2), Pensch R(2), Mäkeläinen S(2), Pe ...
  
  
Author information: (1)Department of Immunology, Genetics and Pathology, Science for Life Laboratory ...
  
  
Current knowledge of cancer genomics remains biased against noncoding mutations. To systematically search for regulatory noncoding mutations, we assessed mutations in conserved positions in the genome under the assumption that these are more likely to be functional than mutations in positions with low conservation. To this end, we use whole-genome sequencing data from the International Cancer Genome Consortium and combined it with evolutionary constraint inferred from 240 mammals, to identify genes enriched in noncoding constraint mutations (NCCMs), mutations likely to be regulatory in nature. We compare medulloblastoma (MB), which is malignant, to pilocytic astrocytoma (PA), a primarily benign tumor, and find highly different NCCM frequencies between the two, in agreement with the fact that malignant cancers tend to have more mutations. In PA, a high NCCM frequency only affects the BRAF locus, which is the most commonly mutated gene in PA. In contrast, in MB, >500 genes have high levels of NCCMs. Intriguingly, several loci with NCCMs in MB are associated with different ages of onset, such as the HOXB cluster in young MB patients. In adult patients, NCCMs occurred in, e.g., the WASF-2/AHDC1/FGR locus. One of these NCCMs led to increased expression of the SRC kinase FGR and augmented responsiveness of MB cells to dasatinib, a SRC kinase inhibitor. Our analysis thus points to different molecular pathways in different patient groups. These newly identified putative candidate driver mutations may aid in patient stratification in MB and could be valuable for future selection of personalized treatment options.
  
  
  
  
**AHDC1**
-
2
of
7
  

### AT-hook DNA-binding motif-containing protein one knockdown downregulates EWS-FLI1 transcriptional activity in Ewing's sarcoma cells.

2. PLoS One. 2022 Oct 4;17(10):e0269077. doi: 10.1371/journal.pone.0269077 eCollection 2022.
  
  
Kitagawa T(1), Kobayashi D(2)(3), Baron B(4), Okita H(5), Miyamoto T(6), Takai R(1), Paudel D(1), Oh ...
  
  
Author information: (1)Advanced Research Promotion Center, Health Sciences University of Hokkaido, K ...
  
  
Ewing's sarcoma is the second most common bone malignancy in children or young adults and is caused by an oncogenic transcription factor by a chromosomal translocation between the EWSR1 gene and the ETS transcription factor family. However, the transcriptional mechanism of EWS-ETS fusion proteins is still unclear. To identify the transcriptional complexes of EWS-ETS fusion transcription factors, we applied a proximal labeling system called BioID in Ewing's sarcoma cells. We identified AHDC1 as a proximal protein of EWS-ETS fusion proteins. AHDC1 knockdown showed a reduced cell growth and transcriptional activity of EWS-FLI1. AHDC1 knockdown also reduced BRD4 and BRG1 protein levels, both known as interacting proteins of EWS-FLI1. Our results suggest that AHDC1 supports cell growth through EWS-FLI1.
  
  
  
  
**AHDC1**
-
3
of
7
  

### Whole genome sequencing identifies rare germline variants enriched in cancer related genes in first degree relatives of familial pancreatic cancer patients.

3. Clin Genet. 2021 Nov;100(5):551-562. doi: 10.1111/cge.14038 Epub 2021 Aug 3.
  
  
Tan M(1)(2)(3), Brusgaard K(1)(4), Gerdes AM(5), Mortensen MB(1)(3)(6), Detlefsen S(1)(3)(7), Schaff ...
  
  
Author information: (1)Department of Clinical Research, University of Southern Denmark, Odense, Denm ...
  
  
First-degree relatives (FDRs) of familial pancreatic cancer (FPC) patients have increased risk of developing pancreatic ductal adenocarcinoma (PDAC). Investigating and understanding the genetic basis for PDAC susceptibility in FPC predisposed families may contribute toward future risk-assessment and management of high-risk individuals. Using a Danish cohort of 27 FPC families, we performed whole-genome sequencing of 61 FDRs of FPC patients focusing on rare genetic variants that may contribute to familial aggregation of PDAC. Statistical analysis was performed using the gnomAD database as external controls. Through analysis of heterozygous premature truncating variants (PTV), we identified cancer-related genes and cancer-driver genes harboring multiple germline mutations. Association analysis detected 20 significant genes with false discovery rate, q < 0.05 including: PALD1, LRP1B, COL4A2, CYLC2, ZFYVE9, BRD3, AHDC1, etc. Functional annotation showed that the significant genes were enriched by gene clusters encoding for extracellular matrix and associated proteins. PTV genes were over-represented by functions related to transport of small molecules, innate immune system, ion channel transport, and stimuli-sensing channels. In conclusion, FDRs of FPC patients carry rare germline variants related to cancer pathogenesis that may contribute to increased susceptibility to PDAC. The identified variants may potentially be useful for risk prediction of high-risk individuals in predisposed families.
  
  
  
  
**AHDC1**
-
4
of
7
  

### Xia-Gibbs Syndrome: A Review of Literature.

4. Cureus. 2020 Dec 29;12(12):e12352. doi: 10.7759/cureus.12352
  
  
Goyal C(1), Naqvi WM(1), Sahu A(1), Aujla AS(2).
  
  
Author information: (1)Physiotherapy, Datta Meghe Institute of Medical Sciences, Wardha, IND. (2)Pae ...
  
  
Xia-Gibbs syndrome (XGS) is a rare genetic disorder that has been discovered as a distinct clinical entity in the recent past. The occurrence has been attributed to the mutation of AT Hook DNA binding motif Containing 1 (AHDC1) gene that is carried on chromosome 1p36. The concerned gene participates in deoxyribonucleic acid (DNA) repair apart from other crucial functions. The mutation results in dysfunction that leads to neurodevelopmental delay. The spectrum of manifestations constitutes intellectual disabilities, hypotonia, expressive language delay, sleep difficulties, and short stature. Dysmorphic facial features include depressed nasal bridge, hypertelorism, down-slanting or up-slanting palpebral fissures, horizontal eyebrows, dysplastic dentition, thin upper lip vermilion, and micrognathia. The phenotype is still expanding. The condition may range from mild to severe dysfunction depending on the area and site of genetic aberration but variation is evident. Thus, the correlation between genotype and phenotype is largely unclear. XGS should be considered as a differential diagnosis for patients presenting with intellectual as well as developmental disabilities. Whole-exome sequencing (WES) is the genetic test that is largely used for the confirmation of diagnosis. Less is known about the natural history as only a few adults with XGS have been documented in the literature. Age-appropriate cancer screening is recommended for patients with XGS as the gene mutation alters DNA repair mechanisms that may trigger tumour formation. The management of patients diagnosed with XGS is an area that needs investigation. Though use of growth hormone replacement therapy and physiotherapy intervention have been reported as effective in previous studies, research on effective means of care of these patients is warranted on a larger number of patients. We present a review of current literature on what is known about XGS that would facilitate to identify knowledge gaps for paving a way for further studies. This, in turn, will help in provision of early and effective rehabilitation services for patients with XGS.
  
  
  
  
**AHDC1**
-
5
of
7
  

### LINC01133 promotes the progression of cervical cancer by sponging miR-4784 to up-regulate AHDC1.

5. Cancer Biol Ther. 2019;20(12):1453-1461. doi: 10.1080/15384047.2019.1647058 Epub 2019 Aug 7.
  
  
Feng Y(1), Qu L(2), Wang X(2), Liu C(2).
  
  
Author information: (1)Department of gynecology, Henan Provincial People's Hospital, People's Hospit ...
  
  
Cervical cancer, as the deadliest gynecological tumor with high risk of incidence, manifests aberrantly expressed lncRNAs in the malignant cellular processes. Long intergenic non-protein coding RNA 1133 (LINC01133) has been acknowledged to actively participate in aggressive tumor phenotypes. Our study focused on the identification of the function and corresponding mechanism of the novel molecule, LINC01133 in cervical cancer. LINC01133 expression profile was validated by digging The Cancer Genome Atlas (TCGA) database and qRT-PCR analysis. A considerably up-regulated expression of LINC01133 was unveiled. The results of CCK-8, trypan blue exclusion, EdU and transwell migration assays manifested the facilitating property of LINC01133 in cervical cancer. The epithelial-mesenchymal transition (EMT) was also exacerbated by LINCO1133. Apoptotic rate of cervical cancer cells was promoted after silencing LINCO1133. Mechanically, LINC01133 functioning as a ceRNA targeted miR-4784 to augment AHDC1 expression. Finally, LINCO1133/miR-4784 aggravated the malignant growth and aggressiveness and EMT of cervical cancer in an AHDC1-dependant way.
  
  
  
  
**AHDC1**
-
6
of
7
  

### The phenotypic spectrum of Xia-Gibbs syndrome.

6. Am J Med Genet A. 2018 Jun;176(6):1315-1326. doi: 10.1002/ajmg.a.38699 Epub 2018 Apr 25.
  
  
Jiang Y(1)(2), Wangler MF(2)(3), McGuire AL(4), Lupski JR(1)(2)(3)(5), Posey JE(2), Khayat MM(1)(2), ...
  
  
Author information: (1)Human Genome Sequencing Center, Baylor College of Medicine, Houston, Texas. ( ...
  
  
Xia-Gibbs syndrome (XGS: OMIM # 615829) results from de novo truncating mutations within the AT-Hook DNA Binding Motif Containing 1 gene (AHDC1). To further define the phenotypic and molecular spectrum of this disorder, we established an XGS Registry and recruited patients from a worldwide pool of approximately 60 probands. Additional de novo truncating mutations were observed among 25 individuals, extending both the known number of mutation sites and the range of positions within the coding region that were sensitive to alteration. Detailed phenotypic examination of 20 of these patients via clinical records review and data collection from additional surveys showed a wider age range than previously described. Data from developmental milestones showed evidence for delayed speech and that males were more severely affected. Neuroimaging from six available patients showed an associated thinning of the corpus callosum and posterior fossa cysts. An increased risk of both scoliosis and seizures relative to the population burden was also observed. Data from a modified autism screening tool revealed that XGS shares significant overlap with autism spectrum disorders. These details of the phenotypic heterogeneity of XGS implicate specific genotype/phenotype correlations and suggest potential clinical management guidelines.
  
  
  
  
**AHDC1**
-
7
of
7
  

### Whole-Exome Sequencing Identifies a de novo AHDC1 Mutation in a Colombian Patient with Xia-Gibbs Syndrome.

7. Mol Syndromol. 2017 Nov;8(6):308-312. doi: 10.1159/000479357 Epub 2017 Sep 8.
  
  
García-Acero M(1), Acosta J(2).
  
  
Author information: (1)Instituto de Genética Humana, Pontificia Universidad Javeriana, Bogotá, Colom ...
  
  
Xia-Gibbs syndrome is an autosomal dominant multisystem developmental disorder characterized by global developmental delay, hypotonia, obstructive sleep apnea, seizures, retrocerebellar cysts, delayed myelination, micrognathia, and mild dysmorphic features. Using whole-exome sequencing, we identified a de novo AHDC1 frameshift mutation c.2030\_2030delG (p.G677Afs\*52) in a Colombian patient, which was absent in both parents. Furthermore, we summarized the phenotypes of patients reported in the literature.
  
  
  
  
  

---

  

# AL513327.1

**Signature gene in:** MDM
  
**Differentially expressed in:** MDM
  
  
**AL513327.1**
-
1
of
1
  

### Novel Prognosis and Therapeutic Response Model of Immune-Related lncRNA Pairs in Clear Cell Renal Cell Carcinoma.

1. Vaccines (Basel). 2022 Jul 21;10(7):1161. doi: 10.3390/vaccines10071161
  
  
Wang G(1), Liu P(2), Li J(1), Jin K(1), Zheng X(1), Xie L(1)(3).
  
  
Author information: (1)Department of Urology, First Affiliated Hospital, School of Medicine, Zhejian ...
  
  
Clear cell renal cell carcinoma (ccRCC) is the most common type of renal carcinoma. It is particularly important to accurately judge the prognosis of patients. Since most tumor prediction models depend on the specific expression level of related genes, a better model therefore needs to be constructed. To provide an immune-related lncRNA (irlncRNAs) tumor prognosis model that is independent of the specific gene expression levels, we first downloaded and sorted out the data on ccRCC in the TCGA database and screened irlncRNAs using co-expression analysis and then obtained the differently expressed irlncRNA (DEirlncRNA) pairs by means of univariate analysis. In addition, we modified LASSO penalized regression. Subsequently, the ROC curve was drawn, and we compared the area under the curve, calculated the Akaike information standard value of the 5-year receiver operating characteristic curve, and determined the cut-off point to establish the best model to distinguish the high- or low-disease-risk group of ccRCC. Subsequently, we reassessed the model from the perspectives of survival, clinic-pathological characteristics, tumor-infiltrating immune cells, chemotherapeutics efficacy, and immunosuppressed biomarkers. A total of 17 DEirlncRNAs pairs (AL031710.1|AC104984.5, AC020907.4|AC127-24.4,AC091185.1|AC005104.1, AL513218.1|AC079015.1, AC104564.3|HOXB-AS3, AC003070.1|LINC01355, SEMA6A-AS1|CR936218.1, AL513327.1|AS005785.1, AC084876.1|AC009704.2, IGFL2-AS1|PRDM16-DT, AC011462.4|MMP25-AS1, AL662844.3I|TGB2-AS1, ARHGAP27P1|AC116914.2, AC093788.1|AC007098.1, MCF2L-AS1|AC093001.1, SMIM25|AC008870.2, and AC027796.4|LINC00893) were identified, all of which were included in the Cox regression model. Using the cut-off point, we can better distinguish patients according to different factors, such as survival status, invasive clinic-pathological features, tumor immune infiltration, whether they are sensitive to chemotherapy or not, and expression of immunosuppressive biomarkers. We constructed the irlncRNA model by means of pairing, which can better eliminate the dependence on the expression level of the target genes. In other words, the signature established by pairing irlncRNA regardless of expression levels showed promising clinical prediction value.
  
  
  
  
  

---

  

# CC2D2B

**Signature gene in:** Stromal cells
  
**Differentially expressed in:** Stromal cells
  
  
**CC2D2B**
-
1
of
1
  

### Microarray expression profiling identifies genes, including cytokines, and biofunctions, as diapedesis, associated with a brain metastasis from a papillary thyroid carcinoma.

1. Am J Cancer Res. 2016 Oct 1;6(10):2140-2161. eCollection 2016.
  
  
Schulten HJ(1), Hussein D(2), Al-Adwani F(3), Karim S(1), Al-Maghrabi J(4), Al-Sharif M(5), Jamal A( ...
  
  
Author information: (1)Center of Excellence in Genomic Medicine Research, King Abdulaziz UniversityJ ...
  
  
Brain metastatic papillary thyroid carcinomas (PTCs) are afflicted with unfavorable prognosis; however, the underlying molecular genetics of these rare metastases are virtually unknown. In this study, we compared whole transcript microarray expression profiles of a BRAF mutant, brain metastasis from a PTC, including its technical replicate (TR), with eight non-brain metastatic PTCs and eight primary brain tumors. The top 95 probe sets (false discovery rate (FDR) p-value < 0.05 and fold change (FC) > 2) that were differentially expressed between the brain metastatic PTC, including the TR, and both, non-brain metastatic PTCs and primary brain tumors were in the vast majority upregulated and comprise, e.g. ROS1, MYBPH, SLC18A3, HP, SAA2-SAA4, CP, CCL20, GFAP, RNU1-120P, DMBT1, XDH, CXCL1, PI3, and NAPSA. Cytokines were represented by 10 members in the top 95 probe sets. Pathway and network analysis (p-value < 0.05 and FC > 2) identified granulocytes adhesion and diapedesis as top canonical pathway. Most significant upstream regulators were lipopolysaccharide, TNF, NKkB (complex), IL1A, and CSF2. Top networks categorized under diseases & functions were entitled migration of cells, cell movement, cell survival, apoptosis, and proliferation of cells. Probe sets that were significantly shared between the brain metastatic PTC, the TR, and primary brain tumors include CASP1, CASP4, C1R, CC2D2B, RNY1P16, WDR72, LRRC2, ZHX2, CITED1, and the noncoding transcript AK128523. Taken together, this study identified a set of candidate genes and biofunctions implicated in, so far nearly uncharacterized, molecular processes of a brain metastasis from a PTC.
  
  
  
  
  

---

  

# CHKA

**Signature gene in:** MDM
  
**Differentially expressed in:** MDM
  
  
**CHKA**
-
1
of
10
  

### Core Molecular Clock Factors Regulate Osteosarcoma Stem Cell Survival and Behavior via CSC/EMT Pathways and Lipid Droplet Biogenesis.

1. Cells. 2025 Mar 31;14(7):517. doi: 10.3390/cells14070517
  
  
Bhoumik S(1), Lee Y(1)(2)(3)(4).
  
  
Author information: (1)Department of Translational Medicine and Physiology, Elson S. Floyd College o ...
  
  
The circadian clock, an intrinsic 24 h cellular timekeeping system, regulates fundamental biological processes, including tumor physiology and metabolism. Cancer stem cells (CSCs), a subpopulation of cancer cells with self-renewal and tumorigenic capacities, are implicated in tumor initiation, recurrence, and metastasis. Despite growing evidence for the circadian clock's involvement in regulating CSC functions, its precise regulatory mechanisms remain largely unknown. Here, using a human osteosarcoma (OS) model (143B), we have shown that core molecular clock factors are critical for OS stem cell survival and behavior via direct modulation of CSC and lipid metabolic pathways. In single-cell-derived spheroid formation assays, 143B OS cells exhibited robust spheroid-forming capacity under 3D culture conditions. Furthermore, siRNA-mediated depletion of core clock components (i.e., BMAL1, CLOCK, CRY1/2, PER1/2)-essential positive and negative elements of the circadian clock feedback loop-significantly reduced spheroid formation in 143B CSCs isolated from in vivo OS xenografts. In contrast, knockdown of the secondary clock-stabilizing factor genes NR1D1 and NR1D2 had little effect. We also found that knockdown of BMAL1, CLOCK, or CRY1/2 markedly impaired the migration and invasion capacities of 143B CSCs. At the molecular level, silencing of BMAL1, CLOCK, or CRY1/2 distinctly altered the expression of genes associated with stem cell properties and the epithelial-mesenchymal transition (EMT) in 143B CSCs. In addition, disruption of BMAL1, CLOCK, or CRY1/2 expression significantly reduced lipid droplet formation by downregulating the expression of genes involved in lipogenesis (e.g., DGAT1, FASN, ACSL4, PKM2, CHKA, SREBP1), which are closely linked to CSC/EMT processes. Furthermore, transcriptomic analysis of human OS patient samples revealed that compared with other core clock genes, CRY1 was highly expressed in OS tumors relative to controls, and its expression exhibited strong positive correlations with patient prognosis, survival, and LD biogenesis gene expression. These findings highlight the critical role of the molecular circadian clock in regulating CSC properties and metabolism, underscoring the therapeutic potential of targeting the core clock machinery to enhance OS treatment outcomes.
  
  
  
  
**CHKA**
-
2
of
10
  

### PLD2 is a marker for MASLD-HCC with early-stage fibrosis: revealed by lipidomic and gene expression analysis.

2. Metabolomics. 2025 Mar 10;21(2):39. doi: 10.1007/s11306-025-02226-2
  
  
Sun J(#)(1)(2)(3), Dahboul F(#)(1), Pujos-Guillot E(4), Petera M(4), Chu-Van E(5), Colsch B(5), Weil ...
  
  
Author information: (1)INRAE, Human Nutrition Unit 1019, Clermont Auvergne University, Clermont-Ferr ...
  
  
INTRODUCTION: Metabolic steatotic liver disease (MASLD) can progress to hepatocellular carcinoma (HCC). 25% of MASLD-HCCs occur in the absence of fibrosis. OBJECTIVES: This study aimed to explore lipid metabolic pathways through "omics" and to identify biomarkers of MASLD-HCC based on the degree of fibrosis. METHODS: Our cohort included 79 pairs of MASLD-HCC tumor tissues (TT) and adjacent non-tumor human liver tissues (NTT), which were divided into two groups according to fibrosis degree (F0F2 n = 45 and F3F4 n = 34). Lipidomic analysis (n = 52) using liquid chromatography high-resolution mass spectrometry (LC-HRMS/MS) and gene expression analysis (n = 79) using RT-qPCR were performed. For each group, TT was compared with NTT. Five healthy liver tissues were used as calibrators in gene expression analysis. RESULTS: Using LC-HRMS/MS, 130 lipids were putatively annotated, 30 of which showed a significant difference between TT and NTT. In MASLD-HCC-F0F2, ceramide levels decreased. While sphingomyelin, most phosphatidylcholine and phosphatidylethanolamine species were increased. In contrast, in MASLD-HCC-F3F4, most lipid contents decreased. Based on lipidomic data, a panel of 18 genes related to lipid metabolism was analyzed. The expression of six genes, ACAT2, DGAT2, ACOX1, CHKA, PLD1, and PLD2, was exclusively upregulated in MASLD-HCC-F0F2. Taken together, these data support the existence of two MASLD-HCC lipid metabolic phenotypes, according to the degree of fibrosis. CONCLUSION: In conclusion, our results allow: (1) discriminate two phenotypes of MASLD-HCC according to fibrosis level; (2) propose PLD as a potential drug target for MASLD-HCC-F0F2 patients, and suggest that PLD inhibitor could be evaluated in combination with immunotherapy treatment.
  
  
  
  
**CHKA**
-
3
of
10
  

### Chloroquine Suppresses Colorectal Cancer Progression via Targeting CHKA and PFKM to inhibit the PI3K/AKT Pathway and the Warburg Effect.

3. Int J Biol Sci. 2025 Jan 27;21(4):1619-1631. doi: 10.7150/ijbs.101921 eCollection 2025.
  
  
Liu Y(1), Zhu Y(1), Gu L(1), Li K(2), Ma A(1), Liu L(1), Meng Y(1), Zhang J(1), Shen S(1), Shi Q(1), ...
  
  
Author information: (1)State Key Laboratory for Quality Ensurance and Sustainable Use of Dao-di Herb ...
  
  
Colorectal cancer (CRC) is the second leading cause of cancer-related death worldwide and has become a recognized global health problem. Therefore, the search for new anti-CRC agents or the exploration of new effective drug targets for CRC therapy is urgent. Chloroquine (CQ) is a widely-used antimalarial drug and has shown anti-proliferative effects in CRC. However, the underlying mechanisms are not well understood, particularly as the direct targets of CQ have not been identified. In this study, choline kinase alpha (CHKA) and ATP-dependent 6-phosphofructokinase, muscle type (PFKM) were identified and verified as the binding targets of CQ. CQ specifically binds to CHKA, inhibits its expression and enzymatic activity, and downregulates the downstream phosphorylation of PI3K and AKT, thereby suppressing tumor cell proliferation and inducing apoptosis. CQ also binds to PFKM and inhibits its expression and activity, thereby blocking the Warburg effect. In addition, the downregulation of CHKA can decrease the expression of PFKM and inhibit its activity, thereby blocking the Warburg effect. These observations shed new light on the antitumor mechanisms of CQ and provide new evidence for the close relationship between the PI3K/AKT signaling pathway and the Warburg effect, providing new therapeutic targets for treating CRC.
  
  
  
  
**CHKA**
-
4
of
10
  

### Choline kinase alpha regulates autophagy-associated exosome release to promote glioma cell progression.

4. Biochem Biophys Res Commun. 2025 Feb;746:151269. doi: 10.1016/j.bbrc.2024.151269 Epub 2024 Dec 31.
  
  
Li J(1), Zhao Y(1), Wu X(1), Zou Y(2), Liu Y(2), Ma H(3).
  
  
Author information: (1)School of Clinical Medicine, Ningxia Medical University, Yinchuan, China. (2) ...
  
  
Glioma is the most common primary intracranial malignant tumor in adults, with a poor prognosis. Exosomes released by tumor cells play a crucial role in tumor development, metastasis, angiogenesis, and other biological processes. Despite this significance, the precise molecular mechanisms governing exosome secretion and their impact on tumor progression remain incompletely understood. While Choline Kinase Alpha (CHKA) has been implicated in promoting various types of tumors, its specific role in glioma pathogenesis remains unclear. Our study initially demonstrates that CHKA enhances the proliferation, migration, and invasion abilities of glioma cells. Interestingly, CHKA also stimulates the release of exosomes from glioma cells. Mechanistically, reduced CHKA expression hampers exosome secretion by elevating autophagy levels in gliomas, whereas counteracting the autophagy elevation resulting from CHKA downregulation restores the release of exosomes. Notably, exosomes derived from glioma cells with normal CHKA expression exhibit a greater capacity to promote glioma progression compared to those derived from cells with low CHKA expression. Overall, our findings suggest that CHKA modulates exosome secretion via an autophagy-dependent pathway, thereby facilitating the proliferation, migration, and invasion of glioma cells.
  
  
  
  
**CHKA**
-
5
of
10
  

### Disulfiram/Cu targeting FOXO6 modulates sensitivity of hepatocellular carcinoma to lenvatinib via disrupt choline metabolic.

5. Cell Signal. 2025 Mar;127:111563. doi: 10.1016/j.cellsig.2024.111563 Epub 2024 Dec 16.
  
  
Wu S(1), Weng J(1), Pan Y(2), Wen Z(3), Zeng J(4), Lou Y(1), Tong S(1), Liao P(5), Li N(6), Yu Z(7), ...
  
  
Author information: (1)Zhejiang Key Laboratory of Intelligent Cancer Biomarker Discovery and Transla ...
  
  
Disulfiram/Cu(DSF/Cu) has a known pharmacokinetic and safety profile, exerting a strong antitumor effect. Oral tyrosine kinase inhibitors including lenvatinib are approved as first-line therapy for treating advanced unresectable hepatocellular carcinoma (HCC). These patients still have limited survival due to drug resistance. Disulfiram/Cu and lenvatinib are the promising antitumor treatments. In this study, we studied whether Disulfiram/Cu increased lenvatinib sensitivity in HCC cells. Moreover, the potential drug targets of Disulfiram/Cu and associated mechanisms were explored. We mainly investigated Autophagic flux was determined via immunofluorescence analysis and confocal microscopy. p-PI3K, p-AKT, p62, LC3B, FOXO6, and CHKA proteins associated with autophagy were detected by immunoblotting. In addition, antitumour activity of Disulfiram/Cu in combination with lenvatinib was examined in vivo through construction of the nude mouse transplant tumor model. Furthermore, our results show disulfiram/Cu combined with lenvatinib exerted the synergistic impact on treating HCC in vitro. Mechanistically, transcriptome combined with metabolome reveals Disulfiram/Cu targeting FOXO6 induction of autophagy mediated inhibits cell growth in hepatocellular carcinoma by downregulating CHKα for inhibiting AKT pathway activation while blocking choline metabolic reprogramming in HCC. These effects mostly explain the tumor-promoting effect of FOXO6 on HCC. In general, the results illustrate the mechanistic associations between metabolites and tumor cell malignant phenotype, contributing to developing new anti-HCC pharmacological treatments by Inhibiting FOXO6 for disrupting choline metabolic pathway.
  
  
  
  
**CHKA**
-
6
of
10
  

### Phosphocholine inhibits proliferation and reduces stemness of endometrial cancer cells by downregulating mTOR-c-Myc signaling.

6. Cell Mol Life Sci. 2024 Dec 16;82(1):3. doi: 10.1007/s00018-024-05517-4
  
  
Gong K(#)(1)(2), Zheng Y(#)(1)(2), Liu Y(1), Zhang T(1), Song Y(1)(2), Chen W(1), Guo L(1)(2), Zhou ...
  
  
Author information: (1)Department of Gynecology and Obstetrics, Guangzhou Women and Children's Medic ...
  
  
BACKGROUND: Endometrial cancer (EC) represents a serious health concern among women globally. Excessive activation of the protooncogene c-Myc (c-Myc) is associated with the proliferation and stemness of EC cells. Phosphocholine (PC), which is synthesized by choline kinase alpha (CHKA) catalysis, is upregulated in EC tumor tissues. The present study aimed to investigate the effect of PC accumulation on EC cells and clarify the relationship between PC accumulation and c-Myc activity in EC. METHODS: The c-Myc and CHKA expression in EC tumor tissues were examined using immunohistochemistry. Cell Counting Kit-8 assay, colony formation assay, flow cytometry, western blotting, BrdU staining, and tumorsphere formation assay were used to assess the effect of PC accumulation on EC cells. The mechanism by which PC accumulation inhibits c-Myc was evaluated using RNA-sequencing. Patient-derived organoid (PDO) models were utilised to explore the preclinical efficacy of PC against EC cells. RESULTS: PC accumulation suppressed EC cell proliferation and stemness by inhibiting the activation of the mammalian target of rapamycin (mTOR)-c-Myc signaling. PC accumulation promoted excessive reactive oxygen species production, which reduced the expression of GTPase HRAS. This, in turn, inhibited the mTOR-c-Myc axis and induced EC cell apoptosis. Finally, PC impeded proliferation and downregulated the expression of the mTOR-MYC signaling in EC PDO models. CONCLUSIONS: PC accumulation impairs the proliferation ability and stem cell characteristics of EC cells by inhibiting the activated mTOR-c-Myc axis, potentially offering a promising strategy to enhance the efficacy of EC clinical therapy through the promotion of PC accumulation in tumor cells.
  
  
  
  
**CHKA**
-
7
of
10
  

### Choline kinases: Enzymatic activity, involvement in cancer and other diseases, inhibitors.

7. Int J Cancer. 2025 Apr 1;156(7):1314-1325. doi: 10.1002/ijc.35286 Epub 2024 Dec 11.
  
  
Korbecki J(1)(2), Bosiacki M(2), Kupnicka P(2), Barczak K(3), Ziętek P(4), Chlubek D(2), Baranowska- ...
  
  
Author information: (1)Department of Anatomy and Histology, Collegium Medicum, University of Zielona ...
  
  
One of the aspects of tumor metabolism that distinguish it from healthy tissue is the phosphorylation of choline by choline kinases, which initiates the synthesis of phosphatidylcholine. Presently, there is a lack of comprehensive reviews discussing the current understanding of the role of choline kinase in cancer processes, as well as studies on the anti-tumor properties of choline kinase inhibitors. To address these gaps, this review delves into the enzymatic and non-enzymatic properties of CHKα and CHKβ and explores their precise involvement in cancer processes, particularly cancer cell proliferation. Additionally, we discuss clinical aspects of choline kinases in various tumor types, including pancreatic ductal adenocarcinoma, ovarian cancer, lung adenocarcinoma, lymphoma, leukemia, hepatocellular carcinoma, colon adenocarcinoma, and breast cancer. We examine the potential of CHKα inhibitors as anti-tumor drugs, although they are not yet in the clinical trial phase. Finally, the paper also touches upon the significance of choline kinases in non-cancerous diseases.
  
  
  
  
**CHKA**
-
8
of
10
  

### Integrative multiomic analysis identifies distinct molecular subtypes of NAFLD in a Chinese population.

8. Sci Transl Med. 2024 Nov 6;16(772):eadh9940. doi: 10.1126/scitranslmed.adh9940 Epub 2024 Nov 6.
  
  
Ding J(1)(2)(3)(4), Liu H(5), Zhang X(1)(2)(3)(4), Zhao N(1)(2)(3)(4), Peng Y(1)(2)(3)(4), Shi J(6), ...
  
  
Author information: (1)Department of Gastroenterology, the First Affiliated Hospital (Southwest Hosp ...
  
  
Nonalcoholic fatty liver disease (NAFLD) has become a common health care burden worldwide. The high heterogeneity of NAFLD remains elusive and impairs outcomes of clinical diagnosis and pharmacotherapy. Several NAFLD classifications have been proposed on the basis of clinical, genetic, alcoholic, or serum metabolic analyses. Yet, accurately predicting the progression of NAFLD to cirrhosis or hepatocellular carcinoma (HCC) in patients remains a challenge. Here, on the basis of a Chinese cohort of patients, we classified NAFLD into three distinct molecular subtypes (NAFLD-mSI, NAFLD-mSII, and NAFLD-mSIII) using integrative multiomics including whole-genome sequencing (WGS), proteomics, phosphoproteomics, lipidomics, and metabolomics across a broad range of liver, blood, and urine specimens. We found that NAFLD-mSI had higher expression of CYP1A2 and CYP3A4, which alleviate hepatic steatosis through mediating free fatty acid/bile acid-mTOR-FXR/PPARα signaling. NAFLD-mSII displayed an elevated risk of liver cirrhosis along with increased hepatic infiltration of M1 and M2 macrophages because of lipid-triggered hepatic CCL2 and CRP production. NAFLD-mSIII exhibited a potential risk for HCC development by increased transcription of CEBPB- and ERCC3-regulated oncogenes because of activation of the EGF-EGFR/CHKA/PI3K-PDK1-AKT cascade. Next, we validated the existence of these three NAFLD molecular subtypes in an external cohort comprising 92 patients with NAFLD across three different Chinese hospitals. These findings may aid in understanding the molecular features underlying NAFLD heterogeneity, thereby facilitating clinical diagnosis and treatment strategies with the aim of preventing the development of liver cirrhosis and HCC.
  
  
  
  
**CHKA**
-
9
of
10
  

### Targeted inhibition of CHKα and mTOR in models of pancreatic ductal adenocarcinoma: A novel regimen for metastasis.

9. Cancer Lett. 2024 Nov 28;605:217280. doi: 10.1016/j.canlet.2024.217280 Epub 2024 Sep 28.
  
  
Liu J(1), Jiang B(2), Xu W(3), Liu Q(3), Huang H(3), Chang X(4), Ma G(5), Xu X(5), Zhou L(3), Xiao G ...
  
  
Author information: (1)Department of General Surgery, Key Laboratory of Research in Pancreatic Tumor ...
  
  
Pancreatic ductal adenocarcinoma (PDAC) is a highly metastatic malignancy for which there are currently no effective anti-metastatic therapies. Herein, we employed single-cell RNA sequencing and metabolomics analysis to demonstrate that metastatic cells highly express focal adhesion kinase (FAK), which promotes metastasis by remodeling choline kinase α (CHKα)-dependent choline metabolism. We designed a novel CHKα inhibitor, CHKI-03, and verified its efficacy in inhibiting metastasis in multiple preclinical models. Classical and newly synthesized small-molecule inhibitors have previously been used to assess the therapeutic potential of targeting mTOR and CHKα in various animal models. Mechanistically, FAK activated mTOR and its downstream HIF-1α, thereby elevating CHKα expression and promoting the proliferation, migration, and invasion of PDAC cells, as well as tumor growth and metastasis. Consistently, high expression levels of both FAK and CHKα are correlated with poor prognosis in patients with PDAC. Notably, CHK1-03 inhibited CHKα expression and also suppressed mTORC1 phosphorylation, disrupting the mTORC1-CHKα positive feedback loop. In addition, the combination of CHKI-03 and the mTORC1 inhibitor rapamycin synergistically inhibited tumor growth and metastasis in PDX models. The combination of CHKI-03 and rapamycin demonstrates considerable therapeutic efficacy in PDO models resistant to gemcitabine. Our findings reveal a pivotal mechanism underlying PDAC metastasis regulated by mTORC1-CHKα loop-dependent choline metabolism reprogramming, highlighting the therapeutic potential of this novel regimen for treating PDAC metastasis.
  
  
  
  
**CHKA**
-
10
of
10
  

### Elevated choline drives KLF5-dominated transcriptional reprogramming to facilitate liver cancer progression.

10. Oncogene. 2024 Oct;43(42):3121-3136. doi: 10.1038/s41388-024-03150-w Epub 2024 Sep 9.
  
  
Li X(#)(1), Hu Z(#)(1), Shi Q(1), Qiu W(1), Liu Y(1), Liu Y(1), Huang S(1)(2), Liang L(1), Chen Z(3) ...
  
  
Author information: (1)Fudan University Shanghai Cancer Center and Institutes of Biomedical Sciences ...
  
  
An increase in the total choline-containing compound content is a common characteristic of cancer cells, and aberrant choline metabolism in cancer is closely associated with malignant progression. However, the potential role of choline-induced global transcriptional changes in cancer cells remains unclear. In this study, we reveal that an elevated choline content facilitates hepatocellular carcinoma (HCC) cell proliferation by reprogramming Krüppel-like factor 5 (KLF5)-dominated core transcriptional regulatory circuitry (CRC). Mechanistically, choline administration leads to elevated S-adenosylmethionine (SAM) levels, inducing the formation of H3K4me1 within the super-enhancer (SE) region of KLF5 and activating its transcription. KLF5, as a key transcription factor (TF) of CRC established by choline, further transactivates downstream genes to facilitate HCC cell cycle progression. Additionally, KLF5 can increase the expression of choline kinase-α (CHKA) and CTP:phosphocholine cytidylyltransferase (CCT) resulting in a positive feedback loop to promote HCC cell proliferation. Notably, the histone deacetylase inhibitor (HDACi) vorinostat (SAHA) significantly suppressed KLF5 expression and liver tumor growth in mice, leading to a prolonged lifespan. In conclusion, these findings highlight the epigenetic regulatory mechanism of the SE-driven key regulatory factor KLF5 conducted by choline metabolism in HCC and suggest a potential therapeutic strategy for HCC patients with high choline content.
  
  
  
  
  

---

  

# CNTN4

**Signature gene in:** Stromal cells
  
**Differentially expressed in:** Stromal cells
  
  
**CNTN4**
-
1
of
10
  

### Identification of Genetic Factors Related With Nonhereditary Colorectal Polyposis and Its Recurrence Through Genome-Wide Association Study.

1. J Gastroenterol Hepatol. 2025 Feb;40(2):482-490. doi: 10.1111/jgh.16840 Epub 2024 Dec 4.
  
  
Ji JH(1), Lee SH(2), Jeon CI(3), Jang J(1), Park J(1), Park SJ(1), Park JJ(1)(4), Cheon JH(1), Jee S ...
  
  
Author information: (1)Department of Internal Medicine, Institute of Gastroenterology, Severance Hos ...
  
  
BACKGROUND: Many patients with colorectal polyposis demonstrate negative results in germline mutation test. This study aimed to uncover genetic variants associated with nonhereditary colorectal polyposis using a genome-wide association study (GWAS). METHODS: At a single referral university hospital, between January 2012 and September 2021, 638 patients with ≥ 10 biopsy-proven cumulative polyps on colonoscopy without germline mutations related to hereditary colorectal cancer or polyposis were included. The control group comprised 1863 individuals from the Korea Medical Institute, each having undergone at least two colonoscopies, all of which were normal. This study utilized GWAS to identify susceptibility loci for nonhereditary colorectal polyposis. Genetic differences between patients with and without ≥ 10 polyp recurrences were analyzed using Cox proportional hazards models. RESULTS: GWAS revealed 71 novel risk single-nucleotide polymorphisms (SNPs) not seen in previous colorectal cancer and polyp GWAS. Five genes (UPF3A, BICRA, CBWD6, PDE4DIP, and ABCC4) overlapping seven SNPs (rs566295755, rs2770288, rs1012003, rs201270202, rs71264659, rs1699813, and rs149368557), previously linked to colorectal cancer, were identified as significant risk factors for nonhereditary colorectal polyposis. Two novel genes (CNTN4 and CNTNAP3B), not previously associated with colorectal diseases, were identified. Three SNPs (rs149368557, rs12438834, and rs9707935) were significantly associated with higher risk of recurrence of polyposis. The gene overlapping with rs149368557 was ABCC4, which was also significantly associated with an increased risk of nonhereditary colorectal polyposis. CONCLUSION: This study identified 71 novel risk variants for nonhereditary colorectal polyposis, with three SNPs (rs149368557, rs12438834, and rs9707935) indicating significant associations with increased risk of polyposis recurrence.
  
  
  
  
**CNTN4**
-
2
of
10
  

### Contactin-4 suppresses antitumor T cell responses by engaging amyloid precursor protein.

2. Sci Immunol. 2024 Oct 11;9(100):eadk7237. doi: 10.1126/sciimmunol.adk7237 Epub 2024 Oct 11.
  
  
Jeon BN(1), Kim S(2), Kim Y(2), Yu H(1), Park C(1), Kim G(1), Ha Y(1), Kim GY(1), Kim H(1), Palucka ...
  
  
Author information: (1)Genome and Company, 8F Gwanggyo Flax Desian, 50 Changnyong-daero, 256beon-gil ...
  
  
Immune checkpoint inhibitors have substantial advanced tumor treatment, but their limited benefits and strong responses in only a subset of patients remain challenging. In this study, we explored the immunomodulatory function of contactin-4 (CNTN4). CNTN4 was highly expressed in tumor tissues, and expression impaired the antitumor function of T cells. CNTN4 bound to amyloid precursor protein (APP) on T cells, which attenuated conjugation between cancer cells and T cells, and diminished T cell receptor signaling cascades. We developed an anti-CNTN4 antibody (GENA-104A16) and an anti-APP antibody (5A7) that blocked the binding between CNTN4 and APP. Administration of either GENA-104A16 or 5A7 promoted antitumor T cell responses in a syngeneic mouse model and increased tumor-infiltrating lymphocytes in vivo. Furthermore, elevated CNTN4 levels were associated with poor prognosis and negatively correlated with various cytotoxic immune-related markers. These results suggest that CNTN4-APP is an inhibitory checkpoint in T cells and represents a promising therapeutic strategy for cancer immunotherapy.
  
  
  
  
**CNTN4**
-
3
of
10
  

### Proteomics of left ventricular structure in the Multi-Ethnic Study of Atherosclerosis.

3. ESC Heart Fail. 2025 Feb;12(1):239-249. doi: 10.1002/ehf2.15073 Epub 2024 Sep 12.
  
  
Peterson TE(1), Lima JAC(1), Shah SJ(2), Bluemke DA(3), Bertoni AG(4), Liu Y(5), Ngo D(6), Varadaraj ...
  
  
Author information: (1)Division of Cardiology, Department of Medicine, Johns Hopkins University, Bal ...
  
  
AIMS: Proteomic profiling offers an expansive approach to biomarker discovery and mechanistic hypothesis generation for LV remodelling, a critical component of heart failure (HF). We sought to identify plasma proteins cross-sectionally associated with left ventricular (LV) size and geometry in a diverse population-based cohort without known cardiovascular disease (CVD). METHODS AND RESULTS: Among participants of the Multi-Ethnic Study of Atherosclerosis (MESA), we quantified plasma abundances of 1305 proteins using an aptamer-based platform at exam 1 (2000-2002) and exam 5 (2010-2011) and assessed LV structure by cardiac magnetic resonance (CMR) at the same time points. We used multivariable linear regression with robust variance to assess cross-sectional associations between plasma protein abundances and LV structural characteristics at exam 1, reproduced findings in later-life at exam 5, and explored relationships of associated proteins using annotated enrichment analysis. We studied 763 participants (mean age 60 ± 10 years at exam 1; 53% female; 19% Black race; 31% Hispanic ethnicity). Following adjustment for renal function and traditional CVD risk factors, plasma levels of 3 proteins were associated with LV mass index at both time points with the same directionality (FDR < 0.05): leptin (LEP), renin (REN), and cathepsin-D (CTSD); 20 with LV end-diastolic volume index: LEP, NT-proBNP, histone-lysine N-methyltransferase (EHMT2), chordin-like protein 1 (CHRDL1), tumour necrosis factor-inducible gene 6 protein (TNFAIP6), NT-3 growth factor receptor (NTRK3), c5a anaphylatoxin (C5), neurogenic locus notch homologue protein 3 (NOTCH3), ephrin-B2 (EFNB2), osteomodulin (OMD), contactin-4 (CNTN4), gelsolin (GSN), stromal cell-derived factor 1 (CXCL12), calcineurin subunit B type 1 (PPP3R1), insulin-like growth factor 1 receptor (IGF1R), bone sialoprotein 2 (IBSP), interleukin-11 (IL-11), follistatin-related protein 1 (FSTL1), periostin (POSTN), and biglycan (BGN); and 4 with LV mass-to-volume ratio: RGM domain family member B (RGMB), transforming growth factor beta receptor type 3 (TGFBR3), ephrin-A2 (EFNA2), and cell adhesion molecule 3 (CADM3). Functional annotation implicated regulation of the PI3K-Akt pathway, bone morphogenic protein signalling, and cGMP-mediated signalling. CONCLUSIONS: We report proteomic profiling of LV size and geometry, which identified novel associations and reinforced previous findings on biomarker candidates for LV remodelling and HF. If validated, these proteins may help refine risk prediction and identify novel therapeutic targets for HF.
  
  
  
  
**CNTN4**
-
4
of
10
  

### Clinicopathological characteristics of pheochromocytoma/paraganglioma and screening of prognostic markers.

4. J Surg Oncol. 2023 Sep;128(4):510-518. doi: 10.1002/jso.27358 Epub 2023 Jun 5.
  
  
Lei J(1), Qu T(1), Cha L(1), Tian L(1), Qiu F(1), Guo W(1), Cao J(1), Sun C(1), Zhou B(1).
  
  
Author information: (1)Department of Hepatobiliary and Pancreatic Surgery, Department of Retroperito ...
  
  
BACKGROUND: Malignant pheochromocytoma/paraganglioma (PCPG) is lethal and difficult to diagnose before metastasis. This study is aiming to characterize the PCPG and explore novel prognostic markers. METHODS: Clinical data of patients with pathologically confirmed invasive and noninvasive PCPG were collected and analyzed. Then, the differentially expressed genes (DEGs) and HUB genes were identified by R package "limma" in GSE67066-GPL570. Afterward, the prognostic markers were screened out using R packages of "survival" and "survminer" based on the TCGA data. RESULTS: The 34 invasive PCPGs were characterized by irregular contour and unclear boundary on CT and capsule/extracapsule tissue invasion on pathology compared with the 42 noninvasive PCPGs. Then, 29 upregulated and 30 downregulated DEGs were identified in malignant PCPG compared with benign, which were mainly enriched in the terms of calcium ion binding, neuron cell-cell adhesion, axon, regulation of hormone levels, and regulation of secretion by cell. Of which, nine DEGs were furtherly selected as the HUB genes. Finally, CNTN4 and SH3GL2 were found to be highly expressed in malignant PCPGs and negatively correlated with progression-free interval. CONCLUSIONS: Malignant PCPGs tend to be aggressive in imaging and pathology. The high expression of CNTN4 and SH3GL2 in PCPGs may indicate a poor prognosis.
  
  
  
  
**CNTN4**
-
5
of
10
  

### MiR-148a-3p attenuates apoptosis and inflammation by targeting CNTN4 in atherosclerosis.

5. Ann Transl Med. 2022 Nov;10(22):1201. doi: 10.21037/atm-22-3768
  
  
Wang K(1), Huang XT(2), Miao YP(3), Bai XL(3), Jin F(3).
  
  
Author information: (1)Department of Neurosurgery, The Affiliated Hospital of Inner Mongolia Medical ...
  
  
BACKGROUND: Atherosclerosis (AS) seriously affects human health. The role of microRNAs (miRNAs) in the pathogenesis and progression of AS has become a focus of research. Our goal was to identify the biological effect of differentially expressed miRNAs (DE-miRNAs) in AS. METHODS: To analyze differentially expressed genes (DEGs), including differentially expressed mRNAs (DE-mRNAs) and DE-miRNAs, in AS by using the Gene Expression Omnibus (GEO) database and limma package. DEGs protein-protein interaction (PPI) network and functional enrichment analysis were constructed by using the search tool for the retrieval of interacting genes/proteins (STRING) database, Cytoscape software and Cytoscape plugin "ClueGO2.5.6". We established a coexpression network of dysregulated miRNAs and mRNAs to predict the function of miRNAs by using miRWalk database and Pearson correlation coefficient (PCC) analysis. Cellular experiments were used to validate the results of bioinformatics. RESULTS: First, 69 common DEGs were obtained from datasets GSE43292 and GSE97210 using the limma package in R. Next, a DEG PPI network was constructed. Functional enrichment analysis of DEGs showed that 11 functional pathways were significantly enriched, such as positive regulation of monocyte chemotaxis. Seven common DE-miRNAs were obtained from the GSE99685 dataset and DE-mRNAs predicted miRNAs through the miRWalk database. The miRNA-mRNA network constructed using Cytoscape software suggested that miR-148a-3p targeted contactin 4 (CNTN4). Quantitative real-time polymerase chain reaction (qRT-PCR) assay results indicated that miR-148a-3p was downregulated and CNTN4 was upregulated in the THP-1 + phorbol 12-myristate 13-acetate (PMA) + oxidized low-density lipoprotein (oxLDL) group compared with the THP-1 + PMA group. qRT-PCR, flow cytometry, and enzyme-linked immunosorbent assay (ELISA) found that upregulated miR-148a-3p significantly inhibited the expression of CNTN4, cell apoptosis, and interleukin-6 (IL-6) and tumor necrosis factor-alpha (TNF-α) concentrations in oxLDL-induced THP-1 macrophages. In addition, a dual-luciferase reporter assay demonstrated that CNTN4 was a target gene of miR-148a-3p. CONCLUSIONS: Overall, these findings suggested that miR-148a-3p inhibited oxLDL-induced cell apoptosis and inflammation via targeting CNTN4 in THP-1 macrophages.
  
  
  
  
**CNTN4**
-
6
of
10
  

### Clinical significance of germline telomere length and associated genetic factors in patients with neuroblastoma.

6. Sci Rep. 2022 Jul 28;12(1):12954. doi: 10.1038/s41598-022-17246-4
  
  
Bae JS(#)(1), Lee JW(#)(2), Joung JG(3), Cho HW(2), Ju HY(2), Yoo KH(2), Koo HH(2), Sung KW(4).
  
  
Author information: (1)Research Institute for Future Medicine, Samsung Medical Center, Seoul, Republ ...
  
  
Studies investigating the relationship between germline telomere length and the clinical characteristics of tumors are very limited. This study evaluated the relationship between germline telomere length and the clinical characteristics of neuroblastoma. In addition, a genome-wide association study (GWAS) was performed to investigate the genetic factors associated with germline telomere length. The germline telomere length of peripheral blood mononuclear cells from 186 patients with neuroblastoma was measured by quantitative polymerase chain reaction. The association between germline telomere length and clinical characteristics, including long-term survival, was investigated. For the GWAS, genotyping was performed with a high-density bead chip (Illumina, San Diego, CA, USA). After strict quality-control checks of the samples, an association analysis was conducted. The result showed that longer germline telomeres were significantly associated with longer event-free survival (P = 0.032). To identify significantly assocated genetic markers for germline telomere length, genome wide association analysis was performed. As a result, several single nucleotide polymorphisms located in HIVEP3, LRRTM4, ADGRV1, RAB30, and CHRNA4 genes were discovered. During gene-based analysis (VEGAS2 tool), the CNTN4 gene had the most significant association with germline telomere length (P = 1.0E-06). During gene ontology analysis, susceptible genes associated with germline telomere length were mainly distributed in neurite morphogenesis and neuron development. A longer germline telomere length is associated with favorable prognostic factors at diagnosis and eventually better event-free survival in patients with neuroblastoma. In addition, the GWAS demonstrated that genetic markers and genes related to germline telomere length are associated with neurite morphogenesis and neuron development. Further research with larger cohorts of patients and functional investigations are needed.
  
  
  
  
**CNTN4**
-
7
of
10
  

### Pediatric Pan-Central Nervous System Tumor Methylome Analyses Reveal Immune-Related LncRNAs.

7. Front Immunol. 2022 May 4;13:853904. doi: 10.3389/fimmu.2022.853904 eCollection 2022.
  
  
Li Y(1), Xu S(1), Xu D(1), Pan T(1), Guo J(1), Gu S(1), Lin Q(1), Li X(1)(2), Li K(1), Xiang W(1).
  
  
Author information: (1)College of Biomedical Information and Engineering, NHC Key Laboratory of Cont ...
  
  
Pediatric central nervous system (CNS) tumors are the second most common cancer diagnosis among children. Long noncoding RNAs (lncRNAs) emerge as critical regulators of gene expression, and they play fundamental roles in immune regulation. However, knowledge on epigenetic changes in lncRNAs in diverse types of pediatric CNS tumors is lacking. Here, we integrated the DNA methylation profiles of 2,257 pediatric CNS tumors across 61 subtypes with lncRNA annotations and presented the epigenetically regulated landscape of lncRNAs. We revealed the prevalent lncRNA methylation heterogeneity across pediatric pan-CNS tumors. Based on lncRNA methylation profiles, we refined 14 lncRNA methylation clusters with distinct immune microenvironment patterns. Moreover, we found that lncRNA methylations were significantly correlated with immune cell infiltrations in diverse tumor subtypes. Immune-related lncRNAs were further identified by investigating their correlation with immune cell infiltrations and potentially regulated target genes. LncRNA with methylation perturbations potentially regulate the genes in immune-related pathways. We finally identified several candidate immune-related lncRNA biomarkers (i.e., SSTR5-AS1, CNTN4-AS1, and OSTM1-AS1) in pediatric cancer for further functional validation. In summary, our study represents a comprehensive repertoire of epigenetically regulated immune-related lncRNAs in pediatric pan-CNS tumors, and will facilitate the development of immunotherapeutic targets.
  
  
  
  
**CNTN4**
-
8
of
10
  

### Value of Immunohistochemical Expression of Apelin, Succinate Dehydrogenase B, Chromogranin B, Human Epidermal Growth Factor Receptor-2, Contactin 4, and Succinyl-CoA Synthetase Subunit Beta in Differentiating Metastatic From Non-Metastatic Pheochromocytoma and Paraganglioma.

8. Front Endocrinol (Lausanne). 2022 Apr 28;13:882906. doi: 10.3389/fendo.2022.882906 eCollection 2022.
  
  
Wang Y(1)(2), Chen D(1), Pang Y(1), Xu X(1), Guan X(1)(2), Liu L(1)(2).
  
  
Author information: (1)Department of Urology, Xiangya Hospital, Central South University, Changsha, ...
  
  
OBJECTIVE: We aimed to retrospectively collect pathologically identified pheochromocytoma and paraganglioma (PPGL) tumor tissues from our center and investigate the expression of apelin and succinyl-CoA synthetase subunit beta (SUCLG2), human epidermal growth factor receptor-2 (HER2 or ERBB-2), contactin 4 (CNTN4), chromogranin B (CHGB), and succinate dehydrogenase B (SDHB) in metastatic and non-metastatic PPGLs, for exploring their roles in the diagnosis of metastatic PPGLs. METHODS: A total of 369 patients with pathologically and surgically confirmed PPGLs at Xiangya Hospital, Central South University, between June 2010 and June 2020 were retrospectively included. Sixty patients-12 patients with metastatic PPGLs and 48 patients with non-metastatic PPGLs-were selected through propensity score matching (1:4) to reduce the effect of PPGL type, sex, and age. We observed and quantified the expression of apelin, SDHB, CHGB, ERBB-2, CNTN4, and SUCLG2 in paraffin-embedded samples using immunohistochemical staining. RESULTS: No significant differences were observed between the metastatic group and non-metastatic group with respect to the expression of CNTN4 and SUCLG2. The expression of apelin, SDHB, CHGB, and ERBB-2 was significantly different between the two groups. The expression of apelin, SDHB, and CHGB was significantly lower in the metastatic group than that in the non-metastatic group (P < 0.001). ERBB-2 expression was significantly higher in the metastatic group than in the non-metastatic group (P = 0.042). Kaplan-Meier analysis revealed that patients with negative expression of apelin, SDHB, and CHGB showed significantly lower metastasis-free survival than those with positive expression. Multivariate Cox analysis revealed that SDHB and CHGB levels were independently associated with metastasis-free survival. CONCLUSION: The expression levels of apelin, CHGB, SDHB, and ERBB-2 may be predictive biomarkers for the diagnosis of metastatic PPGLs. Patients with negative expression of apelin, CHGB, and SDHB should be subjected to frequent postoperative follow-up procedures.
  
  
  
  
**CNTN4**
-
9
of
10
  

### Several Fusion Genes Identified in a Spermatic Cord Leiomyoma With Rearrangements of Chromosome Arms 3p and 21q.

9. Cancer Genomics Proteomics. 2021 Jul-Aug;18(4):531-542. doi: 10.21873/cgp.20278
  
  
Panagopoulos I(1), Gorunova L(2), Andersen K(2), Lobmaier I(3), Heim S(2)(4).
  
  
Author information: (1)Section for Cancer Cytogenetics, Institute for Cancer Genetics and Informatic ...
  
  
BACKGROUND/AIM: Benign smooth-muscle tumors, leiomyomas, occur in nearly every organ but are most common in the uterus. Whereas much is known about the genetics of uterine leiomyomas, little genetic information exists about leiomyomas of other organs. Here, we report and discuss the genetic findings in a para-testicular leiomyoma. MATERIALS AND METHODS: Cytogenetic, array comparative genomic hybridization (aCGH) RNA sequencing, reverse-transcription polymerase chain reaction (RT- PCR), and Sanger sequencing analyses were performed on a leiomyoma of the spermatic cord removed from a 61-year-old man. RESULTS: The karyotype was 48~50,XY,add(3) (p21),+4,+7,+8,+9,add(21)(q22)[cp9]/46,XY[2]. aCGH confirmed the trisomies and also detected multiple gains and losses from 3p and 21q. RNA sequencing detected the chimeras ARHGEF3-CACNA2D2, TRAK1-TIMP4, ITPR1- DT-NR2C2, CLASP2-IL17RD, ZNF621-LARS2, CNTN4- RHOA, and NR2C2-CFAP410. All chimeras were confirmed by RT-PCR and Sanger sequencing. CONCLUSION: Our data, together with those previously published, indicate that a group of leiomyomas may be cytogenetically characterized by aberrations of 3p and the formation of fusion genes.
  
  
  
  
**CNTN4**
-
10
of
10
  

### Multi-omics analysis of genomics, epigenomics and transcriptomics for molecular subtypes and core genes for lung adenocarcinoma.

10. BMC Cancer. 2021 Mar 9;21(1):257. doi: 10.1186/s12885-021-07888-4
  
  
Zhao Y(1), Gao Y(2), Xu X(3), Zhou J(4), Wang H(5).
  
  
Author information: (1)Department II of Radiotherapy, Cangzhou Central Hospital, No.16 Xinhua West R ...
  
  
BACKGROUND: Lung adenocarcinoma (LUAD) is the most frequently diagnosed histological subtype of lung cancer. Our purpose was to explore molecular subtypes and core genes for LUAD using multi-omics analysis. METHODS: Methylation, transcriptome, copy number variation (CNV), mutations and clinical feature information concerning LUAD were retrieved from The Cancer Genome Atlas Database (TCGA). Molecular subtypes were conducted via the "iClusterPlus" package in R, followed by Kaplan-Meier survival analysis. Correlation between iCluster subtypes and immune cells was analyzed. Core genes were screened out by integration of methylation, CNV and gene expression, which were externally validated by independent datasets. RESULTS: Two iCluster subtypes were conducted for LUAD. Patients in imprinting centre 1 (iC1) subtype had a poorer prognosis than those in iC2 subtype. Furthermore, iC2 subtype had a higher level of B cell infiltration than iC1 subtype. Two core genes including CNTN4 and RFTN1 were screened out, both of which had higher expression levels in iC2 subtype than iC1 subtype. There were distinct differences in CNV and methylation of them between two subtypes. After validation, low expression of CNTN4 and RFTN1 predicted poorer clinical outcomes for LUAD patients. CONCLUSION: Our findings comprehensively analyzed genomics, epigenomics, and transcriptomics of LUAD, offering novel underlying molecular mechanisms for LUAD. Two multi-omics-based core genes (CNTN4 and RFTN1) could become potential therapeutic targets for LUAD.
  
  
  
  
  

---

  

# DPEP2

**Signature gene in:** MDM
  
**Differentially expressed in:** MDM
  
  
**DPEP2**
-
1
of
7
  

### Generation of novel lipid metabolism-based signatures to predict prognosis and immunotherapy response for colorectal adenocarcinoma.

1. Sci Rep. 2024 Jul 26;14(1):17158. doi: 10.1038/s41598-024-67549-x
  
  
Wang Y(1), Yao J(2), Zhang Z(2), Wei L(2), Wang S(3).
  
  
Author information: (1)Department of Oncology and Hematology, Suzhou Kowloon Hospital, Shanghai Jiao ...
  
  
Erratum in Sci Rep. 2024 Sep 11;14(1):21206. doi: 10.1038/s41598-024-72216-2.
  
  
  
  
**DPEP2**
-
2
of
7
  

### Neutrophil infiltration associated genes on the prognosis and tumor immune microenvironment of lung adenocarcinoma.

2. Front Immunol. 2023 Dec 22;14:1304529. doi: 10.3389/fimmu.2023.1304529 eCollection 2023.
  
  
Liu R(#)(1)(2), Zhu G(#)(1)(2), Sun Y(#)(3), Li M(2), Hu Z(1)(2), Cao P(1)(2), Li X(1)(2), Song Z(1) ...
  
  
Author information: (1)Department of Lung Cancer Surgery, Tianjin Medical University General Hospita ...
  
  
The neutrophils exhibit both anti-tumor and pro-tumor effects in cancers. The correlation between neutrophils and tumor development in lung adenocarcinoma (LUAD) is still uncertain, possibly due to a lack of specific neutrophil infiltration evaluation methods. In this study, we identified 30 hub genes that were significantly associated with neutrophil infiltration in LUAD through data mining, survival analysis, and multiple tumor-infiltrating immune cells (TICs) analysis, including TIMER, CIBERSORT, QUANTISEQ, XCELL, and MCPCOUNTER. Consensus clustering analysis showed that these 30 hub genes were correlated with clinical features in LUAD. We further developed a neutrophil scoring system based on these hub genes. The neutrophil score was significantly correlated with prognosis and tumor immune microenvironment (TIME) in LUAD. It was also positively associated with PD-L1 expression and negatively associated with tumor mutational burden (TMB). When combined with the neutrophil score, the predictive capacity of PD-L1 and TMB for prognosis was significantly improved. Thus, the 30 hub genes might play an essential role in the interaction of neutrophils and LUAD, and the neutrophil scoring system might effectually assess the infiltration of neutrophils. Furthermore, we verified the expression of these 30 genes in the LUAD tumor tissues collected from our department. We further found that overexpressed TNFAIP6 and TLR6 and downregulated P2RY13, SCARF1, DPEP2, PRAM1, CYP27A1, CFP, GPX3, and NCF1 in LUAD tissue might be potentially associated with neutrophils pro-tumor effects. The following in vitro experiments demonstrated that TNFAIP6 and TLR6 were significantly overexpressed, and P2RY13 and CYP27A1 were significantly downregulated in LUAD cell lines, compared to BEAS-2B cells. Knocking down TNFAIP6 in A549 and PC9 resulted in the upregulation of FAS, CCL3, and ICAM-1, and the downregulation of CCL2, CXCR4, and VEGF-A in neutrophils when co-culturing with the conditioned medium (CM) from LUAD cells. Knocking down TNFAIP6 in LUAD also led to an elevated early apoptosis rate of neutrophils. Therefore, overexpressed TNFAIP6 in LUAD cancer cells might lead to neutrophils "N2" polarization, which exhibited pro-tumor effects. Further research based on the genes identified in this pilot study might shed light on neutrophils' effects on LUAD in the future.
  
  
  
  
**DPEP2**
-
3
of
7
  

### Dipeptidase‑2 is a prognostic marker in lung adenocarcinoma that is correlated with its sensitivity to cisplatin.

3. Oncol Rep. 2023 Aug;50(2):161. doi: 10.3892/or.2023.8598 Epub 2023 Jul 14.
  
  
Wang Y(#)(1), Zhang T(#)(1), Du H(#)(2), Yang M(1), Xie G(3), Liu T(1), Deng S(1), Yuan W(1), He S(1 ...
  
  
Author information: (1)College of Laboratory Medicine, Chengdu Medical College, Chengdu, Sichuan 610 ...
  
  
Lung cancer accounts for the highest percentage of cancer morbidity and mortality worldwide, and lung adenocarcinoma (LUAD) is the most prevalent subtype. Although numerous therapies have been developed for lung cancer, patient prognosis is limited by tumor metastasis and more effective treatment targets are urgently required. In the present study, gene expression profiles were extracted from the Gene Expression Omnibus database and mRNA expression data were downloaded from The Cancer Genome Atlas database. In addition, TIMER 2.0 database was used to analyze the expression of genes in normal and multiple tumor tissues. Protein expression was confirmed using the Human Protein Atlas database and LUAD cell lines, sphere formation assay, western blotting, and a xenograft mouse model were used to confirm the bioinformatics analysis. Dipeptidase‑2 (DPEP2) expression was significantly decreased in LUAD and was negatively associated with prognosis. DPEP2 overexpression substantially inhibited epithelial‑mesenchymal transition (EMT) as well as LUAD cell metastasis, and limited the expression of the cancer stem cell transformation markers, CD44 and CD133. In addition, DPEP2 improved LUAD sensitivity to cisplatin by inhibiting EMT; this was verified in vitro and in vivo. These data indicated that DPEP2 upregulates E‑cadherin, thereby regulating cell migration, cancer stem cell transformation, and cisplatin resistance, ultimately affecting the survival of patients with LUAD. Overall, the findings of the present suggest that DPEP2 is important in the development of LUAD and can be used both as a prognostic marker and a target for future therapeutic research.
  
  
  
  
**DPEP2**
-
4
of
7
  

### Integrated Single-Cell and Transcriptome Sequencing Analyses Identify Dipeptidase 2 as an Immune-Associated Prognostic Biomarker for Lung Adenocarcinoma.

4. Pharmaceuticals (Basel). 2023 Jun 12;16(6):871. doi: 10.3390/ph16060871
  
  
Zhang X(1), Zhang Y(2), Gao X(3), Zhang Y(1), Chen Y(1).
  
  
Author information: (1)NHC Key Laboratory of Cancer Proteomics & State Local Joint Engineering Labor ...
  
  
Dipeptidase 2 (DPEP2) is a dipeptidyl peptidase that plays an important role in the hydrolysis of leukotriene D4 (LTD4) to leukotriene E4 (LTE4). Previous studies have suggested that LTD4 promotes tumor progression and survival in non-small cell lung cancer (NSCLC). Therefore, we hypothesized that DPEP2 may play a pivotal role in this tumor. Given that lung adenocarcinoma (LUAD) is the most common subtype of NSCLC, our study aimed to examine the expression and function of DPEP2 in LUAD. Based on bioinformatics and the analysis of clinical samples, our findings revealed that DPEP2 is highly expressed in normal lung tissues, but downregulated in LUAD tissues, and its expression levels were significantly associated with clinical indicators of tumor grade and prognosis. Pathway enrichment analysis showed that DPEP2 is involved in biological processes such as chemokine signaling pathways, leukocyte trans-endothelial migration, and humoral immune responses in LUAD. In addition, DPEP2 expression was significantly associated with various immune cells, especially monocytes-macrophages. Single-cell transcriptome data further confirmed the expression of DPEP2 dominantly in macrophages from normal lung tissues. Analysis of the TCIA database revealed that high DPEP2 expression is associated with a stronger response to immune checkpoint inhibitors such as CTLA4 and PD1, and determines sensitivity to LUAD therapeutic agents. Furthermore, we found that DPEP2 inhibits the migration and invasion of LUAD cells. Therefore, DPEP2 may serve as a potential immune biomarker and therapeutic target for LUAD, providing novel therapeutic approaches for this disease.
  
  
  
  
**DPEP2**
-
5
of
7
  

### An immune indicator based on BTK and DPEP2 identifies hot and cold tumors and clinical treatment outcomes in lung adenocarcinoma.

5. Sci Rep. 2023 Mar 29;13(1):5153. doi: 10.1038/s41598-023-32276-2
  
  
Han T(#)(1), Liu Y(#)(1)(2), Wu J(3)(4)(5), Bai Y(1)(2), Zhou J(1), Hu C(1), Zhang W(1), Guo J(1), W ...
  
  
Author information: (1)School of Medicine, Anhui University of Science and Technology, Huainan, 2320 ...
  
  
In lung adenocarcinoma (LUAD), immune heterogeneity of hot and cold tumors has been recognized as one of the major factors affecting immunotherapy and other common treatments. However, there is still a lack of biomarkers that can effectively identify the immunophenotype of cold and hot tumors. First, the immune signatures were obtained based on literature mining, including macrophage/monocyte, IFN-γ response, TGF-β response, IL12 response, lymphocyte activation, and ECM/Dve/immune response. Subsequently, LUAD patients were further clustered into different immune phenotypes based on these immune signatures. Next, the key genes related to the immune phenotypes were screened by WGCNA analysis, univariate analysis, and lasso-cox analysis, and the risk signature was established via the key genes. In additional, we compared the clinicopathological characteristics, drug sensitivity, the abundance of immune infiltration, and the efficacy of immunotherapy and commonly used therapies between patients in the high- and low-risk groups in LUAD. LUAD patients were divided into immune hot phenotype and immune cold phenotype groups. The clinical presentation showed that patients with the immune hot phenotype had higher immunoactivity (including higher MHC, CYT, immune, stromal, ESTIMATE scores, higher abundance of immune cell infiltration, higher abundance of TIL, and enrichment of immune-enriched subtypes) and better survival outcomes than those with the immune cold phenotype. Subsequently, WGCNA analysis, univariate analysis, and lasso-cox analysis identified the genes highly associated with the immune phenotype: BTK and DPEP2. The risk signature, consisting of BTK and DPEP2, is highly correlated with the immune phenotype. High-risk scores were enriched in patients with immune cold phenotype and low-risk scores were enriched in patients with immune hot phenotype. Compared to the high-risk group, the low-risk group had better clinical performance, higher drug sensitivity, and a higher degree of immunoactivity, as well as better efficacy in receiving immunotherapy and common adjuvant therapy. This study developed an immune indicator consisting of BTK and DPEP2 based on the heterogeneity of hot and cold Immunophenotypes of the tumor microenvironment. This indicator has good efficacy in predicting prognosis and assessing the efficacy of immunotherapy, chemotherapy, and radiotherapy. It has the potential to facilitate personalized and precise treatment of LUAD in the future.
  
  
  
  
**DPEP2**
-
6
of
7
  

### Characterization of the fatty acid metabolism-related genes in lung adenocarcinoma to guide clinical therapy.

6. BMC Pulm Med. 2022 Dec 23;22(1):486. doi: 10.1186/s12890-022-02286-3
  
  
Huang G(1), Zhang J(2), Gong L(3), Wang X(3), Zhang B(4), Liu D(5).
  
  
Author information: (1)State Key Laboratory of Respiratory Disease and National Clinical Research Ce ...
  
  
BACKGROUND: Lung adenocarcinoma (LUAD) is a common cancer with a bad prognosis. Numerous investigations have indicated that the metabolism of fatty acids plays an important role in the occurrence, progression, and treatment of cancer. Consequently, the objective of the current investigation was to elucidate the role and prognostic significance of genes associated with fatty acid metabolism in patients diagnosed with LUAD. MATERIALS AND METHODS: The data files were acquired from The Cancer Genome Atlas database and GSE31210 dataset. Univariate Cox and least absolute shrinkage and selection operator regression analyses were conducted to establish a prognostic risk scoring model depending on fatty acid metabolism-associated genes to predict the prognosis of patients with LUAD. pRRophetic algorithm was utilized to evaluate the potential therapeutic agents. Gene set variation analysis combined with cell-type identification based on the estimation of relative subsets of RNA transcript and single-sample gene set enrichment analysis was used to determine the association between immune cell infiltration and risk score. Tumor immune dysfunction and exclusion algorithm was employed to predict immunotherapeutic sensitivity. RESULTS: To forecast the prognosis of patients with LUAD, a risk scoring model based on five genes associated with fatty acid metabolism was developed, including LDHA, ALDOA, CYP4B1, DPEP2, and HPGDS. Using the risk score algorithm, patients were divided into higher- and lower-risk categories. Patients classified as minimal risk showed superior prognosis than those with elevated risk. In addition, individuals in the higher-risk group had a proclivity toward chemoresistance and amenable to immunotherapy. CONCLUSION: The prognostic risk scoring model aids in estimating the prognosis of LUAD patients. It may also provide new insights into LUAD carcinogenesis and therapeutic strategies.
  
  
  
  
**DPEP2**
-
7
of
7
  

### Microarray-based gene expression profiling and DNA copy number variation analysis of temporal fossa arachnoid cysts.

7. Cerebrospinal Fluid Res. 2010 Feb 26;7:6. doi: 10.1186/1743-8454-7-6
  
  
Aarhus M(1), Helland CA, Lund-Johansen M, Wester K, Knappskog PM.
  
  
Author information: (1)Centre for Medical Genetics and Molecular Medicine, Haukeland University Hosp ...
  
  
BACKGROUND: Intracranial arachnoid cysts (AC) are membranous sacs filled with CSF-like fluid that are commonly found in the temporal fossa. The majority of ACs are congenital. Typical symptoms are headache, dizziness, and dyscognition. Little is known about genes that contribute to the formation of the cyst membranes. METHODS: In order to identify differences in gene expression between normal arachnoid membrane (AM) and cyst membrane, we have performed a high-resolution mRNA microarray analysis. In addition we have screened DNA from AC samples for chromosomal duplications or deletions using DNA microarray-based copy number variation analysis. RESULTS: The transcriptome consisting of 33096 gene probes showed a near-complete similarity in expression between AC and AM samples. Only nine genes differed in expression between the two tissues: ASGR1, DPEP2, SOX9, SHROOM3, A2BP1, ATP10D, TRIML1, NMU were down regulated, whereas BEND5 was up regulated in the AC samples. Three of the AC samples had unreported human DNA copy number variations, all DNA gains. CONCLUSIONS: Extending results of previous anatomical studies, the present study has identified a small subset of differentially expressed genes and DNA alterations in arachnoid cysts compared to normal arachnoid membrane.
  
  
  
  
  

---

  

# EFHD1

**Signature gene in:** Stromal cells
  
**Differentially expressed in:** Stromal cells
  
  
**EFHD1**
-
1
of
10
  

### EFHD1 Activates SIK3 to Limit Colorectal Cancer Initiation and Progression via the Hippo Pathway.

1. J Cancer. 2025 Jan 20;16(4):1348-1362. doi: 10.7150/jca.103229 eCollection 2025.
  
  
Huang Q(1)(2), Tang X(1)(2), Gan C(3), Deng Q(1)(2), Zhi S(1)(2), Huang Q(1), Zheng X(1), Li X(4), P ...
  
  
Author information: (1)Institute of Cardiovascular Disease, Meizhou People's Hospital, Meizhou Acade ...
  
  
Colorectal cancer (CRC) is one of the most commonly diagnosed cancers, with high rates of metastasis and lethality. EF-hand domain-containing protein D1 (EFHD1) and salt-inducible kinase 3 (SIK3) have been studied in several cancer types. Aberrant expression of EFHD1 and SIK3 has been observed in CRC, but little research has addressed their regulatory abilities and signaling pathways. In this study, we aimed to explore the efficacy of EFHD1 in inhibiting CRC proliferation and metastasis and to elucidate the underlying mechanisms involved in the upregulation of SIK3 expression. Cell viability, colony formation, wound healing, Transwell assay, orthotopic xenograft, and pulmonary metastasis mouse models were used to detect the antiproliferative and anti-metastatic effects of EFHD1 against CRC in vitro and in vivo. The Gene Expression Profiling Interactive Analysis (GEPIA) database was used to determine EFHD1 and SIK3 expression in CRC. The regulatory roles of EFHD1 and SIK3 in mediating anti-metastatic effects in CRC were measured using western blotting, immunohistochemical, and immunofluorescence analyses. The results showed that EFHD1 expression was significantly repressed in the clinical CRC samples. EFHD1 markedly suppressed cell proliferation, migration, and invasion in vitro and inhibited tumor growth and metastasis in vivo. Analysis of the GEPIA database revealed that EFHD1 expression positively correlated with SIK3 expression. SIK3 overexpression inhibited the migration of CRC cells, and SIK3 knockdown partially eliminated the inhibitory effects of EFHD1 on CRC metastasis. EFHD1 exerted anti-metastatic effects against CRC via upregulating SIK3 and inhibiting epithelial-mesenchymal transition (EMT) processing through modulating the Hippo signaling pathway. Collectively, these findings identify EFHD1 as a potent SIK3 agonist and highlight the EFHD1-SIK3 axis as a key modulator of the Hippo signaling pathway in CRC. EFHD1 serves as a novel regulator and is worthy of further development as a novel therapeutic target in CRC.
  
  
  
  
**EFHD1**
-
2
of
10
  

### EFHD1 promotes osteosarcoma proliferation and drug resistance by inhibiting the opening of the mitochondrial membrane permeability transition pore (mPTP) by binding to ANT3.

2. Cell Mol Life Sci. 2024 May 25;81(1):236. doi: 10.1007/s00018-024-05254-8
  
  
Shen X(#)(1), Ma M(#)(1), Mi R(#)(2), Zhuang J(1), Song Y(1), Yang W(1), Li H(1), Lu Y(2), Yang B(1) ...
  
  
Author information: (1)Department of Orthopedics, The Eighth Affiliated Hospital of Sun Yat-sen Univ ...
  
  
Chemoresistance is the main obstacle in the clinical treatment of osteosarcoma (OS). In this study, we investigated the role of EF-hand domain-containing protein 1 (EFHD1) in OS chemotherapy resistance. We found that the expression of EFHD1 was highly correlated with the clinical outcome after chemotherapy. We overexpressed EFHD1 in 143B cells and found that it increased their resistance to cell death after drug treatment. Conversely, knockdown of EFHD1 in 143BR cells (a cisplatin-less-sensitive OS cell line derived from 143B cells) increased their sensitivity to treatment. Mechanistically, EFHD1 bound to adenine nucleotide translocase-3 (ANT3) and inhibited its conformational change, thereby inhibiting the opening of the mitochondrial membrane permeability transition pore (mPTP). This effect could maintain mitochondrial function, thereby favoring OS cell survival. The ANT3 conformational inhibitor carboxyatractyloside (CATR), which can promote mPTP opening, enhanced the chemosensitivity of EFHD1-overexpressing cells when combined with cisplatin. The ANT3 conformational inhibitor bongkrekic acid (BKA), which can inhibit mPTP opening, restored the resistance of EFHD1 knockdown cells. In conclusion, our results suggest that EFHD1-ANT3-mPTP might be a promising target for OS therapy in the future.
  
  
  
  
**EFHD1**
-
3
of
10
  

### EFHD1 expression is correlated with tumor-infiltrating neutrophils and predicts prognosis in gastric cancer.

3. Heliyon. 2023 Oct 14;9(10):e21062. doi: 10.1016/j.heliyon.2023.e21062 eCollection 2023 Oct.
  
  
Zhao B(1), Wang S(2), Xue L(1), Wang Q(3), Liu Y(1), Xu Q(1), Xue Q(4).
  
  
Author information: (1)Department of Pathology, Nantong Tumor Hospital, Nantong Fifth People's Hospi ...
  
  
BACKGROUND: Gastric cancer (GC) ranks third in terms of mortality worldwide. The tumor microenvironment is critical for the progression of gastric cancer. This study investigated the association between EF-hand domain containing 1 (EFHD1) expression and its clinical significance in the tumor microenvironment (TME) of gastric cancer. METHODS: We used bioinformatic analyses to assess the relevance of EFHD1 mRNA in the TME of gastric carcinoma tissues and its relationship with clinical features. Therefore, we performed multiplex immunohistochemistry analyses to determine the potential role of the EFHD1 protein in the TME of gastric cancer. RESULTS: EFHD1 expression increased dramatically in gastric cancer tissues compared to levels in non-cancerous tissue samples (t = 6.246, P < 0.001). The EFHD1 protein presentation was associated with invasion depth (χ2 = 19.120, P < 0.001) and TNM stages (χ2 = 14.468, P = 0.002). Notably, EFHD1 protein expression was significantly related to CD66b + neutrophil infiltration of the intratumoral (r = 0.420, P < 0.001) and stromal (r = 0.367, P < 0.001) TME in gastric cancer. Additionally, Cox regression analysis revealed that EFHD1 was an independent prognostic predictor (hazard ratio [HR] = 2.262, P < 0.001) in patients with gastric cancer. CONCLUSIONS: Our study revealed the pattern of EFHD1 overexpression in the TME of patients with gastric cancer and demonstrated its utility as a biomarker for unfavorable clinical outcomes, thereby providing a potential immunotherapy target.
  
  
  
  
**EFHD1**
-
4
of
10
  

### EFHD1, a novel mitochondrial regulator of tumor metastasis in clear cell renal cell carcinoma.

4. Cancer Sci. 2023 May;114(5):2029-2040. doi: 10.1111/cas.15749 Epub 2023 Feb 26.
  
  
Meng K(1)(2), Hu Y(1), Wang D(1), Li Y(1), Shi F(1), Lu J(3), Wang Y(1), Cao Y(3), Zhang CZ(1), He Q ...
  
  
Author information: (1)MOE Key Laboratory of Tumor Molecular Biology and Key Laboratory of Functiona ...
  
  
The biological function of many mitochondrial proteins in mechanistic detail has not been well investigated in clear cell renal cell carcinoma (ccRCC). A seven-mitochondrial-gene signature was generated by Lasso regression analysis to improve the prediction of prognosis of patients with ccRCC, using The Cancer Genome Atlas and Clinical Proteomic Tumor Analysis Consortium cohort. Among those seven genes, EFHD1 is less studied and its role in the progression of ccRCC remains unknown. The decreased expression of EFHD1 was validated in clinical samples and was correlated with unfavorable outcome. Overexpression of EFHD1 in ccRCC cells resulted in the reduction of mitochondrial Ca2+ , and the inhibition of cell migration and invasion in vitro and tumor metastasis in vivo. Mechanistically, EFHD1 physically bound to the core mitochondrial calcium transporter (mitochondrial calcium uniporter, MCU) through its N-terminal domain. The interaction between EFHD1 and MCU suppressed the uptake of Ca2+ into mitochondria, and deactivated the Hippo/YAP signaling pathway. Further data revealed that the ectopic expression of EFHD1 upregulated STARD13 to enhance the phosphorylation of YAP protein at Ser-127. The knockdown of STARD13 or the overexpression of MCU partly abrogated the EFHD1-mediated induction of phosphorylation of YAP at Ser-127 and suppression of cell migration. Taken together, the newly identified EFHD1-MCU-STARD13 axis participates in the modulation of the Hippo/YAP pathway and serves as a novel regulator in the progression of ccRCC.
  
  
  
  
**EFHD1**
-
5
of
10
  

### Mitochondrial 1555 G>A variant as a potential risk factor for childhood glioblastoma.

5. Neurooncol Adv. 2022 Apr 13;4(1):vdac045. doi: 10.1093/noajnl/vdac045 eCollection 2022 Jan-Dec.
  
  
Li S(1), Gai X(2), Myint SS(1), Arroyo K(1), Morimoto L(3), Metayer C(3), de Smith AJ(1), Walsh KM(4 ...
  
  
Author information: (1)Center for Genetic Epidemiology, Department of Population and Public Health S ...
  
  
BACKGROUND: Childhood glioblastoma multiforme (GBM) is a highly aggressive disease with low survival, and its etiology, especially concerning germline genetic risk, is poorly understood. Mitochondria play a key role in putative tumorigenic processes relating to cellular oxidative metabolism, and mitochondrial DNA variants were not previously assessed for association with pediatric brain tumor risk. METHODS: We conducted an analysis of 675 mitochondrial DNA variants in 90 childhood GBM cases and 2789 controls to identify enrichment of mitochondrial variant associated with GBM risk. We also performed this analysis for other glioma subtypes including pilocytic astrocytoma. Nuclear-encoded mitochondrial gene variants were also analyzed. RESULTS: We identified m1555 A>G was significantly associated with GBM risk (adjusted OR 29.30, 95% CI 5.25-163.4, P-value 9.5 X 10-4). No association was detected for other subtypes. Haplotype analysis further supported the independent risk contributed by m1555 G>A, instead of a haplogroup joint effect. Nuclear-encoded mitochondrial gene variants identified significant associations in European (rs62036057 in WWOX, adjusted OR = 2.99, 95% CI 1.88-4.75, P-value = 3.42 X 10-6) and Hispanic (rs111709726 in EFHD1, adjusted OR = 3.57, 95% CI 1.99-6.40, P-value = 1.41 X 10-6) populations in ethnicity-stratified analyses. CONCLUSION: We report for the first time a potential role played by a functional mitochondrial ribosomal RNA variant in childhood GBM risk, and a potential role for both mitochondrial and nuclear-mitochondrial DNA polymorphisms in GBM tumorigenesis. These data implicate cellular oxidative metabolic capacity as a contributor to the etiology of pediatric glioblastoma.
  
  
  
  
**EFHD1**
-
6
of
10
  

### Identification of Hub Genes Associated With Clear Cell Renal Cell Carcinoma by Integrated Bioinformatics Analysis.

6. Front Oncol. 2021 Sep 30;11:726655. doi: 10.3389/fonc.2021.726655 eCollection 2021.
  
  
Huang H(1)(2)(3), Zhu L(2)(3), Huang C(3)(4), Dong Y(2)(3), Fan L(2)(3), Tao L(1)(3), Peng Z(1)(3), ...
  
  
Author information: (1)Department of Nephrology, Xiangya Hospital Central South University, Changsha ...
  
  
BACKGROUND: Clear cell renal cell carcinoma (ccRCC) is a common genitourinary cancer type with a high mortality rate. Due to a diverse range of biochemical alterations and a high level of tumor heterogeneity, it is crucial to select highly validated prognostic biomarkers to be able to identify subtypes of ccRCC early and apply precision medicine approaches. METHODS: Transcriptome data of ccRCC and clinical traits of patients were obtained from the GSE126964 dataset of Gene Expression Omnibus and The Cancer Genome Atlas Kidney Renal Clear Cell Carcinoma (TCGA-KIRC) database. Weighted gene co-expression network analysis (WGCNA) and differentially expressed gene (DEG) screening were applied to detect common differentially co-expressed genes. Gene Ontology, Kyoto Encyclopedia of Genes and Genomes analysis, survival analysis, prognostic model establishment, and gene set enrichment analysis were also performed. Immunohistochemical analysis results of the expression levels of prognostic genes were obtained from The Human Protein Atlas. Single-gene RNA sequencing data were obtained from the GSE131685 and GSE171306 datasets. RESULTS: In the present study, a total of 2,492 DEGs identified between ccRCC and healthy controls were filtered, revealing 1,300 upregulated genes and 1,192 downregulated genes. Using WGCNA, the turquoise module was identified to be closely associated with ccRCC. Hub genes were identified using the maximal clique centrality algorithm. After having intersected the hub genes and the DEGs in GSE126964 and TCGA-KIRC dataset, and after performing univariate, least absolute shrinkage and selection operator, and multivariate Cox regression analyses, ALDOB, EFHD1, and ESRRG were identified as significant prognostic factors in patients diagnosed with ccRCC. Single-gene RNA sequencing analysis revealed the expression profile of ALDOB, EFHD1, and ESRRG in different cell types of ccRCC. CONCLUSIONS: The present results demonstrated that ALDOB, EFHD1, and ESRRG may act as potential targets for medical therapy and could serve as diagnostic biomarkers for ccRCC.
  
  
  
  
**EFHD1**
-
7
of
10
  

### Aberrant promoter methylation of PPP1R3C and EFHD1 in plasma of colorectal cancer patients.

7. Cancer Med. 2014 Oct;3(5):1235-45. doi: 10.1002/cam4.273 Epub 2014 May 24.
  
  
Takane K(1), Midorikawa Y, Yagi K, Sakai A, Aburatani H, Takayama T, Kaneda A.
  
  
Author information: (1)Department of Digestive Surgery and Pathology, Nihon University School of Med ...
  
  
Aberrant DNA methylation is a common epigenetic alteration involved in colorectal cancer (CRC). In our previous study, we performed methylated DNA immunoprecipitation-on-chip analysis combined with gene re-expression analysis by 5-aza-2'-deoxycytidine treatment, to identify methylation genes in CRC genome widely. Among these genes, 12 genes showed aberrant hypermethylation frequently in >75% of 149 CRC samples but did not in normal samples. In this study, we aim to find out any of these methylation genes to be utilized for CRC detection using plasma DNA samples. Primers for methylation-specific PCR and pyrosequencing were designed for seven of the 12 genes. Among them, PPP1R3C and EFHD1 were rarely hypermethylated in peripheral blood cells, but frequently hypermethylated in 24 CRC tissue samples and their corresponding plasma samples. In plasma samples, PPP1R3C was methylated in 81% (97/120) of CRC patients, but only in 19% (18/96) of noncancer patients (P = 6 × 10(-20) , Fisher's exact test). In combined analysis with EFHD1, both genes were methylated in 53% (64/120) of CRC patients, but only in 4% (4/96) of noncancer patients (P = 2 × 10(-16) ), giving high specificity of 96%. At least one of the two genes was methylated in 90% (108/120) of CRC patients, and 36% (35/96) of control patients, giving high sensitivity of 90%. Compared with low sensitivity of carcinoembryonic antigen (17% at stage I, 40% at stage II) and CA19-9 (0% at stage I, 13% at stage II) for early-stage CRCs, sensitivity of aberrant methylation was significantly higher: PPP1R3C methylation at 92% (11/12) for stage I and 77% (23/30) for stage II, and methylation of at least one gene at 100% (12/12) for stage I and 87% (26/30) for stage II. PPP1R3C methylation or its combined use of EFHD1 methylation was highly positive in CRC plasma samples, and they might be useful in detection of CRC, especially for early-stage CRCs.
  
  
  
  
**EFHD1**
-
8
of
10
  

### Gene expression signatures differentiate adenocarcinoma of lung and breast origin in effusions.

8. Hum Pathol. 2012 May;43(5):684-94. doi: 10.1016/j.humpath.2011.06.015 Epub 2011 Sep 19.
  
  
Davidson B(1), Stavnes HT, Risberg B, Nesland JM, Wohlschlaeger J, Yang Y, Shih IeM, Wang TL.
  
  
Author information: (1)Division of Pathology, Norwegian Radium Hospital, Oslo University Hospital, N ...
  
  
Lung and breast adenocarcinoma at advanced stages commonly involve the serosal cavities, giving rise to malignant effusions. The aim of the present study was to compare the global gene expression patterns of metastases from these 2 malignancies, to expand and improve the diagnostic panel of biomarkers currently available for their differential diagnosis, as well as to define type-specific biological targets. Gene expression profiles of 7 breast and 4 lung adenocarcinoma effusions were analyzed using the HumanRef-8 BeadChip from Illumina. Differentially expressed candidate genes were validated using quantitative real-time polymerase chain reaction and immunohistochemistry. Unsupervised hierarchical clustering using all 54,675 genes in the array separated lung from breast adenocarcinoma samples. We identified 289 unique probes that were significantly differentially expressed in the 2 cancers by greater than 2-fold using moderated t statistics, of which 65 and 224 were overexpressed in breast and lung adenocarcinoma, respectively. Genes overexpressed in breast adenocarcinoma included TFF1, TFF3, FOXA1, CA12, PITX1, RARRES1, CITED4, MYC, TFAP2A, EFHD1, TOB1, SPDEF, FASN, and TH. Genes overexpressed in lung adenocarcinoma included TITF1, SFTPG, MMP7, EVA1, GPR116, HOP, SCGB3A2, and MET. The differential expression of 15 genes was validated by quantitative real-time PCR, and differences in 8 gene products were confirmed by immunohistochemistry. Expression profiling distinguishes breast adenocarcinoma from lung adenocarcinoma and identifies genes that are differentially expressed in these 2 tumor types. The molecular signatures unique to these cancers may facilitate their differential diagnosis and may provide a molecular basis for therapeutic target discovery.
  
  
  
  
**EFHD1**
-
9
of
10
  

### HNF4 alpha orchestrates a set of 14 genes to down-regulate cell proliferation in kidney cells.

9. Biol Chem. 2008 Feb;389(2):179-87. doi: 10.1515/BC.2008.011
  
  
Grigo K(1), Wirsing A, Lucas B, Klein-Hitpass L, Ryffel GU.
  
  
Author information: (1)Institut für Zellbiologie (Tumorforschung), Universitätsklinikum Essen, Unive ...
  
  
Abstract Few genes are known to be involved in renal cell carcinoma (RCC) development and progression. The cell-specific transcription factor hepatocyte nuclear factor 4 alpha (HNF4 alpha) is down-regulated in RCC and we have shown that HNF4 alpha inhibits cell proliferation in the embryonic kidney cell line HEK293. To clarify the possible tumor suppressor activity of HNF4 alpha we analyzed the whole human expression profile in HEK293 cells upon HNF4 alpha induction. By comparing induced and uninduced cells, we identified 1411 differentially expressed genes. Using RNA interference, we screened 56 HNF4 alpha-regulated genes for their possible role in mediating inhibition of cell proliferation triggered by HNF4 alpha. We demonstrate that 14 of these regulated genes are able to contribute to the inhibitory effect of HNF4 alpha on cell proliferation, including well-known cancer genes, such as CDKN1A (p21), TGFA, MME (NEP) and ADAMTS1. In addition, the genes SEPP1, THEM2, BPHL, DSC2, ANK3, ALDH6A1, EPHX2, NELL2, EFHD1 and PROS1 are also part of the network of HNF4 alpha target genes that regulate proliferation in HEK293 cells. Therefore, we postulate that HNF4 alpha orchestrates, at least, these 14 genes to regulate cell proliferation in HEK293 cells and that down-regulation of HNF4 alpha could contribute to the progression of kidney cancer.
  
  
  
  
**EFHD1**
-
10
of
10
  

### HNF4alpha reduces proliferation of kidney cells and affects genes deregulated in renal cell carcinoma.

10. Oncogene. 2005 Sep 22;24(42):6418-31. doi: 10.1038/sj.onc.1208794
  
  
Lucas B(1), Grigo K, Erdmann S, Lausen J, Klein-Hitpass L, Ryffel GU.
  
  
Author information: (1)Institut für Zellbiologie (Tumorforschung), Universitätsklinikum Essen, D-451 ...
  
  
Hepatocyte nuclear factor 4alpha (HNF4alpha) is a tissue-specific transcription factor known to regulate a large number of genes in hepatocytes and pancreatic beta cells. Although HNF4alpha is highly expressed in some sections of the kidney, little is known about its role in this organ and about HNF4alpha-regulated genes in the kidney cells. The abundance and activity of HNF4alpha are frequently reduced in renal cell carcinoma (RCC) indicating some tumor suppressing function of HNF4alpha in renal cells. To determine the potential role of HNF4alpha in RCC, we used Flp recombinase-mediated gene integration to generate human embryonic kidney cells (HEK293) that conditionally express wild-type or mutated HNF4alpha. Expression of wild-type HNF4alpha but not of the mutants led to reduction of proliferation and alterations of cell morphology. These effects were reversible and induced at physiological concentrations of HNF4alpha. Using gene expression profiling by microarrays, we determined genes regulated by HNF4alpha. Interestingly, many of the genes regulated by HNF4alpha have been shown to be deregulated in RCC microarray studies. These genes (ACY1, WT1, SELENBP1, COBL, EFHD1, AGXT2L1, ALDH5A1, THEM2, ABCB1, FLJ14146, CSPG2, TRIM9 and HEY1) are good candidates for genes whose activity is changed upon the decrease of HNF4alpha in RCC.
  
  
  
  
  

---

  

# FAM53B

**Signature gene in:** MDM
  
**Differentially expressed in:** MDM
  
  
**FAM53B**
-
1
of
7
  

### FAM53B promotes pancreatic ductal adenocarcinoma metastasis by regulating macrophage M2 polarization.

1. World J Gastrointest Oncol. 2024 Apr 15;16(4):1479-1499. doi: 10.4251/wjgo.v16.i4.1479
  
  
Pei XZ(1), Cai M(1), Jiang DW(1), Chen SH(1), Wang QQ(1), Lu HM(2), Lu YF(3).
  
  
Author information: (1)Department of Hepatological Surgery, The First Hospital of Jiaxing, Jiaxing 3 ...
  
  
BACKGROUND: Our study investigated the role of FAM53B in regulating macrophage M2 polarization and its potential mechanisms in promoting pancreatic ductal adenocarcinoma (PDAC) metastasis. AIM: To further investigate the role of FAM53B in regulating macrophage M2 polarization and its potential mechanism in promoting PDAC metastasis. Our goal is to determine how FAM53B affects macrophage M2 polarization and to define its underlying mechanism in PDAC metastasis. METHODS: Cell culture and various experiments, including protein analysis, immunohistochemistry, and animal model experiments, were conducted. We compared FAM53B expression between PDAC tissues and healthy tissues and assessed the correlation of FAM53B expression with clinical features. Our study analyzed the role of FAM53B in macrophage M2 polarization in vitro by examining the expression of relevant markers. Finally, we used a murine model to study the role of FAM53B in PDAC metastasis and analyzed the potential underlying mechanisms. RESULTS: Our research showed that there was a significant increase in FAM53B levels in PDAC tissues, which was linked to adverse tumor features. Experimental findings indicated that FAM53B can enhance macrophage M2 polarization, leading to increased anti-inflammatory factor release. The results from the mouse model further supported the role of FAM53B in PDAC metastasis, as blocking FAM53B prevented tumor cell invasion and metastasis. CONCLUSION: FAM53B promotes PDAC metastasis by regulating macrophage M2 polarization. This discovery could lead to the development of new strategies for treating PDAC. For example, interfering with the FAM53B signaling pathway may prevent cancer spread. Our research findings also provide important information for expanding our understanding of PDAC pathogenesis.
  
  
  
  
**FAM53B**
-
2
of
7
  

### The circ\_FAM53B-miR-183-5p-CCDC6 axis modulates the malignant behaviors of papillary thyroid carcinoma cells.

2. Mol Cell Biochem. 2022 Nov;477(11):2627-2641. doi: 10.1007/s11010-022-04465-6 Epub 2022 May 22.
  
  
Zhang C(1), Gu H(2), Liu D(3), Tong F(3), Wei H(3), Zhou D(3), Fang J(3), Dai X(3), Tian H(3).
  
  
Author information: (1)Department of Pathology, Chongqing university Fuling Hospital, No. 2, Gaosunt ...
  
  
Papillary thyroid carcinoma (PTC) is a common thyroid malignancy. Circular RNAs (circRNAs) have been implicated in the development of PTC. Here, we explored the function and mechanism of circRNA family with sequence similarity 53, member B (circ\_FAM53B) in PTC pathogenesis. Circ\_FAM53B, microRNA (miR)-183-5p and coiled-coil domain containing 6 (CCDC6) levels were gauged by quantitative reverse transcription polymerase chain reaction (qRT-PCR) or Western blotting. The direct relationship between miR-183-5p and circ\_FAM53B or CCDC6 was verified by dual-luciferase reporter and RNA immunoprecipitation (RIP) assays. Our data showed that circ\_FAM53B expression was reduced in PTC tissues and cells. Circ\_FAM53B expression restrained proliferation, migration, and invasion and triggered apoptosis of PTC cells, as well as hindered HUVEC tube formation. Circ\_FAM53B repressed miR-183-5p expression. MiR-183-5p re-expression reversed the effects of circ\_FAM53B on cell behaviors. MiR-183-5p targeted and inhibited CCDC6, and circ\_FAM53B upregulated CCDC6 through miR-183-5p competition. MiR-183-5p knockdown repressed cell proliferation, migration, invasion, and tube formation and facilitated apoptosis by upregulating CCDC6. Furthermore, circ\_FAM53B reduced tumor growth in vivo. Collectively, our findings suggest that circ\_FAM53B affects PTC cell biological behaviors via the miR-183-5p-CCDC6 axis.
  
  
  
  
**FAM53B**
-
3
of
7
  

### Clinical M2 macrophages-related genes to aid therapy in pancreatic ductal adenocarcinoma.

3. Cancer Cell Int. 2021 Oct 30;21(1):582. doi: 10.1186/s12935-021-02289-w
  
  
Xu Q(#)(1)(2), Chen S(#)(1), Hu Y(#)(1), Huang W(3).
  
  
Author information: (1)The Second Affiliated Hospital, Yuying Children's Hospital of Wenzhou Medical ...
  
  
BACKGROUND: Increasing evidence supports that infiltration M2 Macrophages act as pivotal player in tumor progression of pancreatic ductal adenocarcinoma (PDAC). Nonetheless, comprehensive analysis of M2 Macrophage infiltration and biological roles of hub genes (FAM53B) in clinical outcome and immunotherapy was lack. METHOD: The multiomic data of PDAC samples were downloaded from distinct datasets. CIBERSORT algorithm was performed to uncover the landscape of TIME. Weighted gene co-expression network analysis (WGCNA) was performed to identify candidate module and significant genes associated with M2 Macrophages. Kaplan-Meier curve and receiver operating characteristic (ROC) curves were applied for prognosis value validation. Mutation data was analyzed by using "maftools" R package. Gene set variation analysis (GSVA) was employed to assign pathway activity estimates to individual sample. Immunophenoscore (IPS) was implemented to estimate immunotherapeutic significance of risk score. The half-maximal inhibitory concentration (IC50) of chemotherapeutic drugs was predicted by using the pRRophetic algorithm. Finally, quantitative real-time polymerase chain reaction was used to determine FAM53B mRNA expression and TIMER database was utilized to uncover its possible role in immune infiltration of PDAC. RESULTS: Herein, 17,932 genes in 234 samples (214 tumor and 20 normal) were extracted from three platforms. Taking advantage of WGCNA, significant module (royalblue) and 135 candidate genes were considered as M2 Macrophages-related genes. Subsequently, risk signature including 5 hub genes was developed by multiple analysis, which exhibited excellent prognostic performance. Besides, comprehensive prognostic nomogram was constructed to quantitatively estimate risk. Then, intrinsic link between risk score with tumor mutation burden (TMB) was explored. Additionally, risk score significantly correlated with diversity of tumor immune microenvironment (TIME). PDAC samples within different risk presented diverse signaling pathways activity and experienced significantly distinct sensitivity to administering chemotherapeutic or immunotherapeutic agents. Finally, the biological roles of FAM53B were revealed in PDAC. CONCLUSIONS: Taken together, comprehensive analyses of M2 Macrophages profiling will facilitate prognostic prediction, delineating complexity of TIME, and contribute insight into precision therapy for PDAC.
  
  
  
  
**FAM53B**
-
4
of
7
  

### Analysis of Genetic Alterations Related to DNA Methylation in Testicular Germ Cell Tumors Based on Data Mining.

4. Cytogenet Genome Res. 2021;161(6-7):382-394. doi: 10.1159/000516385 Epub 2021 Aug 25.
  
  
Yu X(1), Han Y(2), Liu S(1), Jiang W(1), Song Y(1), Tong J(1), Qiao T(1), Lv Z(1)(3)(4), Li D(1).
  
  
Author information: (1)Department of Nuclear Medicine, Shanghai Tenth People's Hospital, Tongji Univ ...
  
  
Embryonal carcinoma (EC) and seminoma (SE) are both derived from germ cell neoplasia in situ but show big differences in growth patterns and clinical prognosis. Epigenetic regulation may play an important role in the development of EC and SE. This study investigated the DNA methylation-based genetic alterations between EC and SE by analyzing the datasets of mRNA expression and DNA methylation profiling. The datasets were downloaded from the Gene Expression Omnibus database. The differentially expressed genes (DEGs) were identified between EC and SE by limma package in R environment. Gene function enrichment analysis of the DEGs was performed on the DAVID tool, the results of which suggested differences in capability of pluripotency and genomic stability between EC and SE. The minfi package and wANNOVAR tool were used to identify differentially methylated genes. A total of 37 genes were discovered with both mRNA expression and the accordant DNA methylation changes. The findings were verified by the sequencing data from The Cancer Genome Atlas database, and Kaplan-Meier survival analysis was performed. Finally, 5 genes (PRDM1, LMO2, FAM53B, HCN4, and FAM124B) were found that showed both low expression and high methylation in EC, and were significantly associated with relapse-free survival. The findings of methylation-based genetic features between EC and SE might be helpful in studying the role of DNA methylation in cancer development.
  
  
  
  
**FAM53B**
-
5
of
7
  

### Super-Enhancer Associated Five-Gene Risk Score Model Predicts Overall Survival in Multiple Myeloma Patients.

5. Front Cell Dev Biol. 2020 Dec 3;8:596777. doi: 10.3389/fcell.2020.596777 eCollection 2020.
  
  
Qi T(1)(2)(3), Qu J(2)(3), Tu C(4), Lu Q(2)(3), Li G(2)(3), Wang J(2)(3), Qu Q(1)(5).
  
  
Author information: (1)Department of Pharmacy, Xiangya Hospital, Central South University, Changsha, ...
  
  
Multiple myeloma (MM) is a malignant plasma cell tumor with high heterogeneity, characterized by anemia, hypercalcemia, renal failure, and lytic bone lesions. Although various powerful prognostic factors and models have been exploited, the development of more accurate prognosis and treatment for MM patients is still facing many challenges. Given the essential roles of super-enhancer (SE) associated genes in the tumorigenesis of MM, we tried to initially screen and identify the significant prognostic factors from SE associated genes in MM by the least absolute shrinkage and selection operator (Lasso) penalized Cox regression, univariate and multivariate Cox regression analysis using GSE24080 and GSE9782 datasets. Risk score model of five genes including CSGALNACT1, FAM53B, TAPBPL, REPIN1, and DDX11, was further constructed and the Kaplan-Meier (K-M) curves showed that the low-risk group seems to have better clinical outcome of survival compared to the high-risk group. Time-dependent receiver operating characteristic (ROC) curves presented the favorable performance of the model. An interactive nomogram consisting of the five-gene risk group and eleven clinical traits was established and identified by calibration curves. Therefore, the risk score model of SE associated five genes developed here could be used to predict the prognosis of MM patients, which may assist the clinical treatment of MM patients in the future.
  
  
  
  
**FAM53B**
-
6
of
7
  

### Upregulation of circular RNA circ‑FAM53B predicts adverse prognosis and accelerates the progression of ovarian cancer via the miR‑646/VAMP2 and miR‑647/MDM2 signaling pathways.

6. Oncol Rep. 2019 Dec;42(6):2728-2737. doi: 10.3892/or.2019.7366 Epub 2019 Oct 10.
  
  
Sun D(1), Liu J(1), Zhou L(2).
  
  
Author information: (1)Department of Gynecology, Heilongjiang Provincial Hospital, Harbin, Heilongji ...
  
  
Ovarian cancer (OC) is a common cancer of the human genital system. Circular RNAs (circRNAs) play an important role in carcinogenesis and progression of various cancers. The present study aimed to clarify the expression profile and functions of circ‑FAM53B in the progression of OC and reveal its underlying mechanisms. Relative levels of circ‑FAM53B in OC specimens and cell lines were determined by quantitative real‑time polymerase chain reaction (qRT‑PCR). The clinical significance of circ‑FAM53B in OC patients was analyzed through Fisher's exact test, Kaplan‑Meier curves, and Cox regression analysis. Subsequently, the regulatory effects of circ‑FAM53B on the proliferative, apoptotic, migratory, and invasive potential of OC cells were determined by loss/gain‑of‑function experiments. Mechanistically, bioinformatics analysis and luciferase reporter gene assay were used to reveal the potential molecular mechanisms of circ‑FAM53B in OC. circ‑FAM53B was overexpressed in OC specimens and cells and correlated with clinical severity and poor prognosis of OC patients. The overexpression of circ‑FAM53B accelerated the proliferation, migration, and invasion of HO8910 cells; however, it decreased the number of apoptotic cells. Silencing of circ‑FAM53B induced the opposite effect. Through dual‑luciferase reporter gene assay and functional experiments, the potential functions of circ‑FAM53B/miRNA‑646/vesicle‑associated membrane protein 2 (VAMP2) and circ‑FAM53B/miRNA‑647/mouse double minute 2 (MDM2) in mediating the progression of OC were identified. Collectively, the present results indicated that circ‑FAM53B could be a competing endogenous RNA (ceRNA) to competitively sponge miR‑646 and miR‑647 to upregulate VAMP2 and MDM2 expression at the post‑transcriptional level, thus mediating the cellular behaviors of OC cells.
  
  
  
  
**FAM53B**
-
7
of
7
  

### The reconstruction of transcriptional networks reveals critical genes with implications for clinical outcome of multiple myeloma.

7. Clin Cancer Res. 2011 Dec 1;17(23):7402-12. doi: 10.1158/1078-0432.CCR-11-0596 Epub 2011 Sep 2.
  
  
Agnelli L(1), Forcato M, Ferrari F, Tuana G, Todoerti K, Walker BA, Morgan GJ, Lombardi L, Bicciato ...
  
  
Author information: (1)Department of Medical Sciences, University of Milan and Hematology, Fondazion ...
  
  
Comment in Clin Cancer Res. 2011 Dec 1;17(23):7210-2. doi: 10.1158/1078-0432.CCR-11-2316.
  
  
  
  
  

---

  

# FARP2

**Signature gene in:** Stromal cells
  
**Differentially expressed in:** Stromal cells
  
  
**FARP2**
-
1
of
9
  

### Plexin-A2 enables the proliferation and the development of tumors from glioblastoma derived cells.

1. Cell Death Dis. 2023 Jan 19;14(1):41. doi: 10.1038/s41419-023-05554-0
  
  
Toledano S(1), Sabag AD(2), Ilan N(1), Liburkin-Dan T(1), Kessler O(1), Neufeld G(3).
  
  
Author information: (1)Cancer research center, The Bruce Rappaport Faculty of Medicine, Technion-Isr ...
  
  
The semaphorin guidance factors receptor plexin-A2 transduces sema6A and sema6B signals and may mediate, along with plexin-A4, the anti-angiogenic effects of sema6A. When associated with neuropilins plexin-A2 also transduces the anti-angiogenic signals of sema3B. Here we show that inhibition of plexin-A2 expression in glioblastoma derived cells that express wild type p53 such as U87MG and A172 cells, or in primary human endothelial cells, strongly inhibits cell proliferation. Inhibition of plexin-A2 expression in U87MG cells also results in strong inhibition of their tumor forming ability. Knock-out of the plexin-A2 gene in U87MG cells using CRISPR/Cas9 inhibits cell proliferation which is rescued following plexin-A2 re-expression, or expression of a truncated plexin-A2 lacking its extracellular domain. Inhibition of plexin-A2 expression results in cell cycle arrest at the G2/M stage, and is accompanied by changes in cytoskeletal organization, cell flattening, and enhanced expression of senescence associated β-galactosidase. It is also associated with reduced AKT phosphorylation and enhanced phosphorylation of p38MAPK. We find that the pro-proliferative effects of plexin-A2 are mediated by FARP2 and FYN and by the GTPase activating (GAP) domain located in the intracellular domain of plexin-A2. Point mutations in these locations inhibit the rescue of cell proliferation upon re-expression of the mutated intracellular domain in the knock-out cells. In contrast re-expression of a plexin-A2 cDNA containing a point mutation in the semaphorin binding domain failed to inhibit the rescue. Our results suggest that plexin-A2 may represent a novel target for the development of anti-tumorigenic therapeutics.
  
  
  
  
**FARP2**
-
2
of
9
  

### Pathogenic nsSNPs that increase the risks of cancers among the Orang Asli and Malays.

2. Sci Rep. 2021 Aug 9;11(1):16158. doi: 10.1038/s41598-021-95618-y
  
  
Khoruddin NA(1)(2), Noorizhab MN(1)(3), Teh LK(1)(3), Mohd Yusof FZ(1)(2), Salleh MZ(4)(5).
  
  
Author information: (1)Integrative Pharmacogenomics Institute (iPROMISE), Universiti Teknologi MARA ...
  
  
Single-nucleotide polymorphisms (SNPs) are the most common genetic variations for various complex human diseases, including cancers. Genome-wide association studies (GWAS) have identified numerous SNPs that increase cancer risks, such as breast cancer, colorectal cancer, and leukemia. These SNPs were cataloged for scientific use. However, GWAS are often conducted on certain populations in which the Orang Asli and Malays were not included. Therefore, we have developed a bioinformatic pipeline to mine the whole-genome sequence databases of the Orang Asli and Malays to determine the presence of pathogenic SNPs that might increase the risks of cancers among them. Five different in silico tools, SIFT, PROVEAN, Poly-Phen-2, Condel, and PANTHER, were used to predict and assess the functional impacts of the SNPs. Out of the 80 cancer-related nsSNPs from the GWAS dataset, 52 nsSNPs were found among the Orang Asli and Malays. They were further analyzed using the bioinformatic pipeline to identify the pathogenic variants. Three nsSNPs; rs1126809 (TYR), rs10936600 (LRRC34), and rs757978 (FARP2), were found as the most damaging cancer pathogenic variants. These mutations alter the protein interface and change the allosteric sites of the respective proteins. As TYR, LRRC34, and FARP2 genes play important roles in numerous cellular processes such as cell proliferation, differentiation, growth, and cell survival; therefore, any impairment on the protein function could be involved in the development of cancer. rs1126809, rs10936600, and rs757978 are the important pathogenic variants that increase the risks of cancers among the Orang Asli and Malays. The roles and impacts of these variants in cancers will require further investigations using in vitro cancer models.
  
  
  
  
**FARP2**
-
3
of
9
  

### ROCK2 inhibition triggers the collective invasion of colorectal adenocarcinomas.

3. EMBO J. 2019 Jul 15;38(14):e99299. doi: 10.15252/embj.201899299 Epub 2019 Jun 18.
  
  
Libanje F(1), Raingeaud J(1), Luan R(1), Thomas Z(1), Zajac O(1), Veiga J(2), Marisa L(3), Adam J(4) ...
  
  
Author information: (1)INSERM U-981, Gustave Roussy, Villejuif, France. (2)Cell Biology Program, Mem ...
  
  
The metastatic progression of cancer is a multi-step process initiated by the local invasion of the peritumoral stroma. To identify the mechanisms underlying colorectal carcinoma (CRC) invasion, we collected live human primary cancer specimens at the time of surgery and monitored them ex vivo. This revealed that conventional adenocarcinomas undergo collective invasion while retaining their epithelial glandular architecture with an inward apical pole delineating a luminal cavity. To identify the underlying mechanisms, we used microscopy-based assays on 3D organotypic cultures of Caco-2 cysts as a model system. We performed two siRNA screens targeting Rho-GTPases effectors and guanine nucleotide exchange factors. These screens revealed that ROCK2 inhibition triggers the initial leader/follower polarization of the CRC cell cohorts and induces collective invasion. We further identified FARP2 as the Rac1 GEF necessary for CRC collective invasion. However, FARP2 activation is not sufficient to trigger leader cell formation and the concomitant inhibition of Myosin-II is required to induce invasion downstream of ROCK2 inhibition. Our results contrast with ROCK pro-invasive function in other cancers, stressing that the molecular mechanism of metastatic spread likely depends on tumour types and invasion mode.
  
  
  
  
**FARP2**
-
4
of
9
  

### Anti-FIRΔexon2, a splicing variant form of PUF60, autoantibody is detected in the sera of esophageal squamous cell carcinoma.

4. Cancer Sci. 2019 Jun;110(6):2004-2013. doi: 10.1111/cas.14024 Epub 2019 May 20.
  
  
Kobayashi S(1)(2), Hiwasa T(3), Ishige T(2), Rahmutulla B(4), Kano M(1), Hoshino T(5), Minamoto T(6) ...
  
  
Author information: (1)Department of Frontier Surgery, Graduate School of Medicine, Chiba University ...
  
  
Anti-PUF60 autoantibodies are reportedly detected in the sera of patients with dermatomyositis and Sjögren's syndrome; however, little is known regarding its existence in the sera of cancer patients. FIR, a splicing variant of the PUF60 gene, is a transcriptional repressor of c-myc. In colorectal cancer, there is an overexpression of the dominant negative form of FIR, in which exon 2 is lacking (FIRΔexon2). Previously, large-scale SEREX (serological identification of antigens by recombinant cDNA expression cloning) screenings have identified anti-FIR autoantibodies in the sera of cancer patients. In the present study, we revealed the presence and significance of anti-FIR (FIR/FIRΔexon2) Abs in the sera of patients with esophageal squamous cell carcinoma (ESCC). Our results were validated by an amplified luminescence proximity homogeneous assay using sera of patients with various cancer types. We revealed that anti-FIRΔexon2 Ab had higher sensitivity than anti-FIR Ab. Receiver operating characteristic (ROC) analysis was applied for evaluating the use of anti-FIRΔexon2 Ab as candidate markers such as anti-p53 Ab and carcinoembryonic antigen, and the highest area under the ROC curve was observed in the combination of anti-FIRΔexon2 Ab and anti-p53 Ab. In summary, our results suggest the use of anti-FIRΔexon2 Ab in combination with the anti-p53 Ab as a predictive marker for ESCC. The area under the ROC curve was further increased in the advanced stage of ESCC. The value of anti-FIRΔexon2 autoantibody as novel clinical indicator against ESCC and as a companion diagnostic tool is discussed.
  
  
  
  
**FARP2**
-
5
of
9
  

### Endothelial RhoGEFs: A systematic analysis of their expression profiles in VEGF-stimulated and tumor endothelial cells.

5. Vascul Pharmacol. 2015 Nov;74:60-72. doi: 10.1016/j.vph.2015.10.003 Epub 2015 Oct 17.
  
  
Hernández-García R(1), Iruela-Arispe ML(2), Reyes-Cruz G(3), Vázquez-Prado J(4).
  
  
Author information: (1)Department of Pharmacology,CINVESTAV-IPN,México D.F.,Mexico. (2)Department of ...
  
  
Rho guanine nucleotide exchange factors (RhoGEFs) integrate cell signaling inputs into morphological and functional responses. However, little is known about the endothelial repertoire of RhoGEFs and their regulation. Thus, we assessed the expression of 81 RhoGEFs (70 homologous to Dbl and 11 of the DOCK family) in endothelial cells. Further, in the case of DH-RhoGEFs, we also determined their responses to VEGF exposure in vitro and in the context of tumors. A phylogenetic analysis revealed the existence of four groups of DH-RhoGEFs and two of the DOCK family. Among them, we found that the most abundant endothelial RhoGEFs were: Tuba, FGD5, Farp1, ARHGEF17, TRIO, P-Rex1, ARHGEF15, ARHGEF11, ABR, FARP2, ARHGEF40, ALS, DOCK1, DOCK7 and DOCK6. Expression of RASGRF2 and PREX2 increased significantly in response to VEGF, but most other RhoGEFs were unaffected. Interestingly murine endothelial cells isolated from tumors showed that all four phylogenetic subgroups of DH-RhoGEFs were altered when compared to non-tumor endothelial cells. In summary, our results provide a detailed assessment of RhoGEFs expression profiles in the endothelium and set the basis to systematically address their regulation in vascular signaling.
  
  
  
  
**FARP2**
-
6
of
9
  

### Sendai virus-mediated gene transfer of the c-myc suppressor far-upstream element-binding protein-interacting repressor suppresses head and neck cancer.

6. Gene Ther. 2015 Apr;22(4):297-304. doi: 10.1038/gt.2014.123 Epub 2015 Jan 15.
  
  
Tanaka N(1), Araki K(1), Mizokami D(1), Miyagawa Y(1), Yamashita T(1), Tomifuji M(1), Ueda Y(2), Ino ...
  
  
Author information: (1)Department of Otorhinolaryngology - Head and Neck Surgery, National Defense M ...
  
  
Far-upstream element-binding protein-interacting repressor (FIR) is a transcription factor that inhibits c-Myc expression and has been shown to have antitumor effects in some malignancies. Here, we evaluated the antitumor effects of FIR using fusion gene-deleted Sendai virus (SeV/ΔF) as a nontransmissible vector against head and neck squamous cell carcinoma (HNSCC). Using in vitro and in vivo xenograft mouse models, we observed efficient expression of green fluorescent protein (GFP) following transduction with the SeV/ΔF vector encoding GFP (GFP-SeV/ΔF) into HNSCC cells. In vitro and in vivo studies revealed that administration of the FIR-encoded SeV/ΔF (FIR-SeV/ΔF) vector exerted significant antitumor effects, suppressed c-Myc expression and induced apoptosis in HNSCC. Additionally, the antitumor effects of FIR or the expression of GFP following administration of the FIR- or GFP-SeV/ΔF vector, respectively, were dependent on the multiplicity of infection or titer. Furthermore, the SeV/ΔF vector itself had no cytotoxic effects. Therefore, the SeV/ΔF vector may be safe and useful for the treatment of HNSCC, allowing for high-titer SeV/ΔF vector administration for anticancer gene therapy. In addition, SeV/ΔF vector-mediated FIR gene therapy demonstrated effective tumor suppression in HNSCC, suggesting that this therapy may have the potential for clinical use as a novel strategy for HNSCC treatment.
  
  
  
  
**FARP2**
-
7
of
9
  

### Non-transmissible Sendai virus vector encoding c-myc suppressor FBP-interacting repressor for cancer therapy.

7. World J Gastroenterol. 2014 Apr 21;20(15):4316-28. doi: 10.3748/wjg.v20.i15.4316
  
  
Matsushita K(1), Shimada H(1), Ueda Y(1), Inoue M(1), Hasegawa M(1), Tomonaga T(1), Matsubara H(1), ...
  
  
Author information: (1)Kazuyuki Matsushita, Fumio Nomura, Department of Molecular Diagnosis, Graduat ...
  
  
AIM: To investigate a novel therapeutic strategy to target and suppress c-myc in human cancers using far up stream element (FUSE)-binding protein-interacting repressor (FIR). METHODS: Endogenous c-Myc suppression and apoptosis induction by a transient FIR-expressing vector was examined in vivo via a HA-tagged FIR (HA-FIR) expression vector. A fusion gene-deficient, non-transmissible, Sendai virus (SeV) vector encoding FIR cDNA, SeV/dF/FIR, was prepared. SeV/dF/FIR was examined for its gene transduction efficiency, viral dose dependency of antitumor effect and apoptosis induction in HeLa (cervical squamous cell carcinoma) cells and SW480 (colon adenocarcinoma) cells. Antitumor efficacy in a mouse xenograft model was also examined. The molecular mechanism of the anti-tumor effect and c-Myc suppression by SeV/dF/FIR was examined using Spliceostatin A (SSA), a SAP155 inhibitor, or SAP155 siRNA which induce c-Myc by increasing FIR∆exon2 in HeLa cells. RESULTS: FIR was found to repress c-myc transcription and in turn the overexpression of FIR drove apoptosis through c-myc suppression. Thus, FIR expressing vectors are potentially applicable for cancer therapy. FIR is alternatively spliced by SAP155 in cancer cells lacking the transcriptional repression domain within exon 2 (FIR∆exon2), counteracting FIR for c-Myc protein expression. Furthermore, FIR forms a complex with SAP155 and inhibits mutual well-established functions. Thus, both the valuable effects and side effects of exogenous FIR stimuli should be tested for future clinical application. SeV/dF/FIR, a cytoplasmic RNA virus, was successfully prepared and showed highly efficient gene transduction in in vivo experiments. Furthermore, in nude mouse tumor xenograft models, SeV/dF/FIR displayed high antitumor efficiency against human cancer cells. SeV/dF/FIR suppressed SSA-activated c-Myc. SAP155 siRNA, potentially produces FIR∆exon2, and led to c-Myc overexpression with phosphorylation at Ser62. HA-FIR suppressed endogenous c-Myc expression and induced apoptosis in HeLa and SW480 cells. A c-myc transcriptional suppressor FIR expressing SeV/dF/FIR showed high gene transduction efficiency with significant antitumor effects and apoptosis induction in HeLa and SW480 cells. CONCLUSION: SeV/dF/FIR showed strong tumor growth suppression with no significant side effects in an animal xenograft model, thus SeV/dF/FIR is potentially applicable for future clinical cancer treatment.
  
  
  
  
**FARP2**
-
8
of
9
  

### SAP155-mediated c-myc suppressor far-upstream element-binding protein-interacting repressor splicing variants are activated in colon cancer tissues.

8. Cancer Sci. 2013 Feb;104(2):149-56. doi: 10.1111/cas.12058 Epub 2012 Dec 4.
  
  
Kajiwara T(1), Matsushita K, Itoga S, Tamura M, Tanaka N, Tomonaga T, Matsubara H, Shimada H, Habara ...
  
  
Author information: (1)Department of Molecular Diagnosis, Chiba University Graduate School of Medici ...
  
  
The c-myc transcriptional suppressor, far-upstream element (FUSE)-binding protein (FBP)-interacting repressor (FIR), is alternatively spliced in colorectal cancer tissue (Matsushita et al., Cancer Res 2006). Recently, the knockdown of SAP155 pre-mRNA-splicing factor, a subunit of SF3b, was reported to disturb FIR pre-mRNA splicing and yield FIRΔexon2, an exon 2-spliced variant of FIR, which lacks c-myc repression activity. In the present study, novel splicing variants of FIR, Δ3 and Δ4, were also generated by SAP155 siRNA, and these variants were found to be activated in human colorectal cancer tissue. Furthermore, the expression levels of FIR variant mRNA were examined in the peripheral blood of colorectal cancer patients and healthy volunteers to assess its potency for tumor detection. As expected, circulating FIR variant mRNA in the peripheral blood of cancer patients were significantly overexpressed compared to that in healthy volunteers. In particular, the area under the receiving operating characteristic curve of FIR, FIRΔexon2 or FIRΔexon2/FIR, was greater than those of conventional carcinoembryonic antigen or carbohydrate antigen 19-9. In addition, FIRΔexon2 or FIR mRNA expression in the peripheral blood was significantly reduced after operative removal of colorectal tumors. Thus, circulating FIR and FIRΔexon2 mRNA are potential novel screening markers for colorectal cancer testing with conventional carcinoembryonic antigen and or carbohydrate antigen 19-9. Taken together, our results indicate that overexpression of FIR and its splicing variants in colorectal cancer directs feed-forward or addicted circuit c-myc transcriptional activation. Clinical implications for colorectal cancers of novel FIR splicing variants are also discussed in the present paper.
  
  
  
  
**FARP2**
-
9
of
9
  

### Common variants at 2q37.3, 8q24.21, 15q21.3 and 16q24.1 influence chronic lymphocytic leukemia risk.

9. Nat Genet. 2010 Feb;42(2):132-6. doi: 10.1038/ng.510 Epub 2010 Jan 10.
  
  
Crowther-Swanepoel D(1), Broderick P, Di Bernardo MC, Dobbins SE, Torres M, Mansouri M, Ruiz-Ponte C ...
  
  
Author information: (1)Institute of Cancer Research, Sutton, Surrey, UK.
  
  
To identify new risk variants for chronic lymphocytic leukemia (CLL), we conducted a genome-wide association study of 299,983 tagging SNPs, with validation in four additional series totaling 2,503 cases and 5,789 controls. We identified four new risk loci for CLL at 2q37.3 (rs757978, FARP2; odds ratio (OR) = 1.39; P = 2.11 x 10(-9)), 8q24.21 (rs2456449; OR = 1.26; P = 7.84 x 10(-10)), 15q21.3 (rs7169431; OR = 1.36; P = 4.74 x 10(-7)) and 16q24.1 (rs305061; OR = 1.22; P = 3.60 x 10(-7)). We also found evidence for risk loci at 15q25.2 (rs783540, CPEB1; OR = 1.18; P = 3.67 x 10(-6)) and 18q21.1 (rs1036935; OR = 1.22; P = 2.28 x 10(-6)). These data provide further evidence for genetic susceptibility to this B-cell hematological malignancy.
  
  
  
  
  

---

  

# GRM8

**Signature gene in:** Stromal cells
  
**Differentially expressed in:** Stromal cells
  
  
**GRM8**
-
1
of
10
  

### Escape from TGF-β-induced senescence promotes aggressive hallmarks in epithelial hepatocellular carcinoma cells.

1. Mol Oncol. 2025 Mar 14. doi: 10.1002/1878-0261.70021 Online ahead of print.
  
  
Kalyoncu M(1), Demirci D(1), Eris S(1)(2), Dayanc B(1)(2), Cakiroglu E(1)(2), Basol M(1)(2), Uysal M ...
  
  
Author information: (1)Izmir Biomedicine and Genome Center, Turkey. (2)Department of Genomics and Mo ...
  
  
Transforming growth factor-β (TGF-β) signaling and cellular senescence are key hallmarks of hepatocellular carcinoma (HCC) pathogenesis. Despite provoking senescence-associated growth arrest in epithelial HCC cells, elevated TGF-β activity paradoxically correlates with increased aggressiveness and poor prognosis in advanced tumors. Whether the transition between these dichotomous functions involves modulation of the senescence phenotype during disease progression remains elusive. Exploiting the epithelial HCC cell line Huh7 as a robust model, we demonstrate that chronic exposure to TGF-β prompts escape from Smad3-mediated senescence, leading to the development of TGF-β resistance. This altered state is characterized by an optimal proliferation rate and the acquisition of molecular and functional traits of less-differentiated mesenchymal cells, coinciding with differential growth capacity in 2D and 3D culture conditions, epithelial-to-mesenchymal transition (EMT), and increased invasiveness in vitro, and metastasis in vivo. Mechanistically, resistant cells exhibit defective activation and nuclear trafficking of Smad molecules, particularly Smad3, as ectopic activation of the TGF-β/Smad3 axis is able to reinstate TGF-β sensitivity. An integrated transcriptomic landscape reveals both shared and distinct gene signatures associated with senescent and TGF-β resistant states. Importantly, genetic ablation and molecular studies identify microtubule affinity regulating kinase 1 (MARK1) and glutamate metabotropic receptor 8 (GRM8) as critical modulators of the resistance phenomenon, potentially by impairing spatiotemporal signaling dynamics of Smad activity. Our findings unveil a novel phenomenon wherein epithelial HCC cells may exploit senescence plasticity as a mechanism to oppose TGF-β anti-tumor responses and progress towards more aggressive HCC phenotypes.
  
  
  
  
**GRM8**
-
2
of
10
  

### Exploration of prognostic and treatment markers in hepatocellular carcinoma via GPCR-related genes analysis.

2. Heliyon. 2024 Apr 15;10(9):e29659. doi: 10.1016/j.heliyon.2024.e29659 eCollection 2024 May 15.
  
  
Wang Y(1), Jin B(1), Wu X(1), Xing J(1), Zhang B(1), Chen X(1), Liu X(1), Wan X(1), Du S(1).
  
  
Author information: (1)Department of Liver Surgery, Peking Union Medical College Hospital, Chinese A ...
  
  
BACKGROUND: G protein-coupled receptors (GPCRs), the biggest family of signaling receptors, account for 34 % of all the drug targets approved by the Food and Drug Administration (FDA). It has been gradually recognized that GPCRs are of significance for tumorigenesis, but in-depth studies are still required to explore specific mechanisms. In this study, the role of GPCRs in hepatocellular carcinoma (HCC) was elucidated, and GPCR-related genes were employed for building a risk-score model for the prognosis and treatment efficacy prediction of HCC patients. METHODS: Patients' data on HCC were sourced from the Liver Hepatocellular Carcinoma-Japan (LIRI-JP) and The Cancer Genome Atlas (TCGA) databases, while GPCR-related genes were obtained from the Molecular Signatures Database (MSigDB). Univariant and multivariant Cox regression analyses, as well as least absolute shrinkage and selection operator (LASSO) were performed with the aim of identifying differentially expressed GPCR-related genes and grouping patients. Differential expression and functional enrichment analyses were performed; protein-protein interaction (PPI) mechanisms were explored; hub genes and micro ribonucleic acid (miRNA)-target gene regulatory networks were constructed. The tumor immune dysfunction and exclusion (TIDE) algorithm was utilized to evaluate immune infiltration levels and genetic variations. Sensitivity to immunotherapy and common antitumor drugs was predicted via the database Genomics of Drug Sensitivity in Cancer (GDSC). RESULTS: A GPCR-related risk score containing eight GPCR-related genes (atypical chemokine receptor 3 (ACKR3), C-C chemokine receptor type 3 (CCR3), CCR7, frizzled homolog 5 (FZD5), metabotropic glutamate receptor 8 (GRM8), hydroxycarboxylic acid receptor 1 (HCAR1), 5-hydroxytryptamine receptor 5A (HTR5A) and nucleotide-binding oligomerization domain-like receptor family pyrin domain containing 6 (NLRP6)) was set up. In addition, patients were classified into groups with high and low risks. Patients in the high-risk group exhibited a worse prognosis but demonstrated a more favorable immunotherapy response rate compared with those in the low-risk group. Distinct sensitivity to chemotherapeutic drugs was observed. A clinical prediction model on the basis of GPCR-related risk scores was constructed. Areas under the curves (AUC) corresponding to one-, three- and five-year survival were 0.731, 0.765 and 0.731, respectively. CONCLUSIONS: In this study, an efficient HCC prognostic prediction model was constructed by only GPCR-related genes, which are all potential targets for HCC treatment.
  
  
  
  
**GRM8**
-
3
of
10
  

### Changes in DNA methylation profile in liver tissue during progression of HCV-induced fibrosis to hepatocellular carcinoma.

3. Vavilovskii Zhurnal Genet Selektsii. 2023 Mar;27(1):72-82. doi: 10.18699/VJGB-23-10
  
  
Goncharova IA(1), Zarubin AA(1), Babushkina NP(1), Koroleva IA(1), Nazarenko MS(1).
  
  
Author information: (1)Research Institute of Medical Genetics, Tomsk National Research Medical Cente ...
  
  
In this study we compared methylation levels of 27,578 CpG sites between paired samples of the tumor and surrounding liver tissues with various degrees of damage (fibrosis, cirrhosis) in HCV-induced hepatocellular carcinoma (HCC) patients, as well as between tumor and normal tissue in non-viral HCC patients, using GSE73003 and GSE37988 data from GEODataSets (https://www.ncbi.nlm.nih.gov/). A significantly lower number of differentially methylated sites (DMS) were found between HCC of non-viral etiology and normal liver tissue, as well as between HCC and fibrosis (32 and 40), than between HCC and cirrhosis (2450 and 2304, respectively, according to GSE73003 and GSE37988 datasets). As the pathological changes in the tissue surrounding the tumor progress, the ratio of hyper-/hypomethylated DMSs in the tumor decreases. Thus, in tumor tissues compared with normal/fibrosis/cirrhosis of the liver, 75/62.5/47.7 % (GSE73003) and 16 % (GSE37988) of CpG sites are hypermethylated, respectively. Persistent hypermethylation of the ZNF154 and ZNF540 genes, as well as CCL20 hypomethylation, were registered in tumor tissue in relation to both liver fibrosis and liver cirrhosis. Protein products of the EDG4, CCL20, GPR109A, and GRM8 genes, whose CpG sites are characterized by changes in DNA methylation level in tumor tissue in the setting of cirrhosis and fibrosis, belong to "Signaling by G-protein-coupled receptors (GPCRs)" category. However, changes in the methylation level of the "driver" genes for oncopathology (АРС, CDKN2B, GSTP1, ELF4, TERT, WT1) are registered in tumor tissue in the setting of liver cirrhosis but not fibrosis. Among the genes hypermethylated in tumor tissue in the setting of liver cirrhosis, the most represented biological pathways are developmental processes, cell-cell signaling, transcription regulation, Wnt-protein binding. Genes hypomethylated in liver tumor tissue in the setting of liver cirrhosis are related to olfactory signal transduction, neuroactive ligand-receptor interaction, keratinization, immune response, inhibition of serine proteases, and zinc metabolism. The genes hypermethylated in the tumor are located at the 7p15.2 locus in the HOXA cluster region, and the hypomethylated CpG sites occupy extended regions of the genome in the gene clusters of olfactory receptors (11p15.4), keratin and keratin-associated proteins (12q13.13, 17q21.2, and 21q22.11), epidermal differentiation complex (1q21.3), and immune system function loci 9p21.3 (IFNA, IFNB1, IFNW1 cluster) and 19q13.41-19q13.42 (KLK, SIGLEC, LILR, KIR clusters). Among the genes of fibrogenesis or DNA repair, cg14143055 (ADAMDEC1) is located in the binding region of the HOX gene family transcription factors (TFs), while cg05921699 (CD79A), cg06196379 (TREM1) and cg10990993 (MLH1) are located in the binding region of the ZNF protein family transcription factor (TF). Thus, the DNA methylation profile in the liver in HCV-induced HCC is unique and differs depending on the degree of surrounding tissue lesion - liver fibrosis or liver cirrhosis.
  
  
  
  
**GRM8**
-
4
of
10
  

### A Novel Prognostic Model for Identifying the Risk of Hepatocellular Carcinoma Based on Angiogenesis Factors.

4. Front Genet. 2022 Mar 18;13:857215. doi: 10.3389/fgene.2022.857215 eCollection 2022.
  
  
Gao Y(1), Liu J(1), Zhao D(1), Diao G(1).
  
  
Author information: (1)Department of Hepatobiliary Surgery, the Fifth Medical Center of Chinese PLA ...
  
  
Hepatocellular carcinoma (HCC) is the most common primary liver cancer with poor prognosis. An optimized stratification of HCC patients to discriminate clinical benefit regarding different degrees of malignancy is urgently needed because of no effective and reliable prognostic biomarkers currently. HCC is typically characterized by rich vascular. The dysregulated vascular endothelial growth factor was proved a pivotal regulator of the development of HCC. Therefore, we investigated the capability of angiogenic factors (AFs) in stratifying patients and constructed a prognostic risk model. A total of 6 prognostic correlated AFs (GRM8, SPC25, FSD1L, SLC386A, FAM72A and SLC39A10) were screened via LASSO Cox regression, which provided the basis for developing a novel prognostic risk model. Based on the risk model, HCC patients were subdivided into high-risk and low-risk groups. Kaplan-Meier curve indicated that patients in the high-risk group have a lower survival rate compared with those in the low-risk group. The prognostic model showed good predictive efficacy, with AUCs reaching 0.802 at 1 year, 0.694 at 2 years, and 0.672 at 3 years. Univariate and multivariate cox regression analysis demonstrated that the risk score had significant prognostic value and was an independent prognostic factor for HCC. Moreover, this model also showed a good diagnostic positive rate in the ICGC-LIRI-JP and GSE144269. Finally, we demonstrated the efficacy of the AF-risk model in HCC patients following sorafenib adjuvant chemotherapy. And revealed the underlying molecular features involving tumor stemness, immune regulation, and genomic alterations associated with the risk score. Based on a large population, we established a novel prognostic model based on 6 AFs to help identify HCC patients with a greater risk of death. The model may provide a reference for better clinical management of HCC patients in the era of cancer precision medicine.
  
  
  
  
**GRM8**
-
5
of
10
  

### Metabotropic Glutamate Receptor 8 Is Regulated by miR-33a-5p and Functions as an Oncogene in Breast Cancer.

5. J Oncol. 2021 Dec 14;2021:8002087. doi: 10.1155/2021/8002087 eCollection 2021.
  
  
Zhang C(1), Xie S(1), Yuan S(1), Zhang Y(2), Bai Y(1), Chu L(1), Wu Z(1), Guo N(1), Wang Q(1), Zhang ...
  
  
Author information: (1)Department of General Surgery, PLA No. 988 Hospital of Joint Logistics Suppor ...
  
  
It has been reported that glutamate metabotropic receptor 8 (GRM8) is closely implicated in the progression of human neuroblastoma, lung cancer, and glioma, but its role in breast cancer remains unknown. Thus, the present study was performed to uncover it. Immunohistochemistry, real-time PCR (RT-PCR), and western blotting experiments were performed to test GRM8 expression levels in tissues and cells. Cell functions were assessed by Cell Count Kit 8 (CCK-8), flow cytometry, wound healing, transwell chambers, and in vivo xenotransplantation experiments. The relationship between miR-33a-5p and GRM8 was evaluated by luciferase gene reporter and western blotting assay. The results showed that GRM8 expression was increased in breast cancer tissues and cells, which was closely associated with lower overall survival rate. Ectopic expression of GRM8 significantly enhanced cell growth, migration, and invasion and tumorigenesis and repressed cell apoptosis. In addition, GRM8 was under the negative regulation of miR-33a-5p, which was downregulated in breast cancer tissues and served as a tumor suppressor. Moreover, overexpression of GRM8 abrogated the inhibitive role of miR-33a-5p played in breast cancer. Collectively, this study reveals that GRM8 functions as an oncogene in breast cancer and is regulated by miR-33a-5p.
  
  
  
  
**GRM8**
-
6
of
10
  

### Neuronal exposure induces neurotransmitter signaling and synaptic mediators in tumors early in brain metastasis.

6. Neuro Oncol. 2022 Jun 1;24(6):914-924. doi: 10.1093/neuonc/noab290
  
  
Deshpande K(1)(2)(3), Martirosian V(1)(2)(3), Nakamura BN(4)(2), Iyer M(1)(2)(3), Julian A(1)(2)(3), ...
  
  
Author information: (1)Department of Neurological Surgery, University of Southern California, Los An ...
  
  
BACKGROUND: Brain metastases (BM) are responsible for neurological decline and poor overall survival. Although the pro-metastatic roles of glial cells, and the acquisition of neuronal attributes in established BM tumors have been described, there are no studies that investigate the initial interplay between neurons and brain-seeking tumor cells. The aim of this study was to characterize early tumor-neuron interactions and the induced CNS-adaptive changes in tumor cells prior to macro-colonization. METHODS: Utilizing pure neuronal cultures and brain-naïve and patient-derived BM tumor cells, we surveyed the early induction of mediators of neurotransmitter (NT) and synaptic signaling in breast and lung tumor cells. Reliance on microenvironmental GABA in breast-to-brain metastatic cells (BBMs) was assessed in vitro and in vivo. RESULTS: Coculture with neurons induces early expression of classical NT receptor genes (HTR4, GRIA2, GRIN2B, GRM4, GRM8, DRD1) and neuronal synaptic mediators (CNR1, EGR2, ARC, NGFR, NRXN1) in breast and lung cancer cells. NT-dependent classification of tumor cells within the neuronal niche shows breast cancer cells become GABAergic responsive brain metastases (GRBMs) and transition from relying on autocrine GABA, to paracrine GABA from adjacent neurons; while autocrine Dopaminergic breast and lung tumor cells persist. In vivo studies confirm reliance on paracrine GABA is an early CNS-acclimation strategy in breast cancer. Moreover, neuronal contact induces early resurgence in Reelin expression in tumor cells through epigenetic activation, facilitating CNS adaptation. CONCLUSION: Tumor-neuron interactions allow for CNS adaptation early in the course of brain metastasis.
  
  
  
  
**GRM8**
-
7
of
10
  

### 3D disorganization and rearrangement of genome provide insights into pathogenesis of NAFLD by integrated Hi-C, Nanopore, and RNA sequencing.

7. Acta Pharm Sin B. 2021 Oct;11(10):3150-3164. doi: 10.1016/j.apsb.2021.03.022 Epub 2021 Apr 6.
  
  
Xu L(1), Yin L(1), Qi Y(1), Tan X(1), Gao M(1), Peng J(1)(2)(3).
  
  
Author information: (1)College of Pharmacy, Dalian Medical University, Dalian 116044, China. (2)Key ...
  
  
The three-dimensional (3D) conformation of chromatin is integral to the precise regulation of gene expression. The 3D genome and genomic variations in non-alcoholic fatty liver disease (NAFLD) are largely unknown, despite their key roles in cellular function and physiological processes. High-throughput chromosome conformation capture (Hi-C), Nanopore sequencing, and RNA-sequencing (RNA-seq) assays were performed on the liver of normal and NAFLD mice. A high-resolution 3D chromatin interaction map was generated to examine different 3D genome hierarchies including A/B compartments, topologically associated domains (TADs), and chromatin loops by Hi-C, and whole genome sequencing identifying structural variations (SVs) and copy number variations (CNVs) by Nanopore sequencing. We identified variations in thousands of regions across the genome with respect to 3D chromatin organization and genomic rearrangements, between normal and NAFLD mice, and revealed gene dysregulation frequently accompanied by these variations. Candidate target genes were identified in NAFLD, impacted by genetic rearrangements and spatial organization disruption. Our data provide a high-resolution 3D genome interaction resource for NAFLD investigations, revealed the relationship among genetic rearrangements, spatial organization disruption, and gene regulation, and identified candidate genes associated with these variations implicated in the pathogenesis of NAFLD. The newly findings offer insights into novel mechanisms of NAFLD pathogenesis and can provide a new conceptual framework for NAFLD therapy.
  
  
  
  
**GRM8**
-
8
of
10
  

### A Case of Adult Pancreatoblastoma With Novel APC Mutation and Genetic Heterogeneity.

8. Front Oncol. 2021 Aug 27;11:725290. doi: 10.3389/fonc.2021.725290 eCollection 2021.
  
  
Suemitsu Y(1), Ono Y(2)(3), Mizukami Y(2)(3), Ye J(4), Yamakawa K(4), Takamoto T(5), Nakano-Narusawa ...
  
  
Author information: (1)Department of Pathology, Japanese Red Cross Medical Center, Shibuya, Japan. ( ...
  
  
BACKGROUND: Pancreatoblastoma is a rare malignant epithelial neoplasm of the pancreas that mainly occurs in children and involves abnormalities in the WNT/β-catenin pathway, such as CTNNB1 mutation. However, the molecular abnormalities in adult pancreatoblastoma are not well known. CASE PRESENTATION: An elderly man, who underwent elective distal pancreatectomy and splenectomy, was referred to our hospital with a mass in the tail of the pancreas. Histologically, the lesion revealed proliferation of clear, basophilic, and cartilaginous tumor cells with lymphatic metastasis. Each of the morphologically distinct tumor components showed different immunohistochemical patterns, indicating heterogeneous differentiation, including epithelial (both acinar and ductal), mesenchymal, and neuroendocrine differentiation. All tumor components showed nuclear expression of β-catenin and cyclin D1. Per next-generation sequencing (NGS), the clear and basophilic tumor cells shared mutations in APC, GRM8, LAMP1, and AKA9. Among the mutations, APC, c.1816\_1817insA showed the highest frequency in both cell types, indicating that APC mutation was a driver mutation of the tumor. A diagnosis of PB was rendered. SUMMARY: In conclusion, the clear and basophilic cells of the tumor were supposedly derived from the same clone and subsequently acquired additional mutations. This is the first report of clonal evolution in pancreatoblastoma.
  
  
  
  
**GRM8**
-
9
of
10
  

### The underlying molecular mechanism and drugs for treatment in adrenal cortical carcinoma.

9. Int J Med Sci. 2021 Jun 16;18(13):3026-3038. doi: 10.7150/ijms.60261 eCollection 2021.
  
  
Ma C(1), Xiong J(1), Su H(1), Li H(1).
  
  
Author information: (1)Department of Urology, Peking Union Medical College Hospital, Peking Union Me ...
  
  
Purpose: The study aimed to predict and explore the possible clinical value and mechanism of genetic markers in adrenal cortical carcinoma using a bioinformatics analysis method. Methods: The RNA-seqs and miRNAs data were downloaded from TCGA database to identify the differentially expressed genes and differentially expressed miRNAs. The hub-genes were screened by building protein-protein interaction sub-networks with 12 topological analysis methods. We conducted the receiver operating characteristic curve to elevate the diagnostic value of hub-genes in distinguishing the death and alive groups. The survival analysis of hub-genes and key miRNAs were conducted using Kaplan-Meier curves. Furthermore, most significant small molecules were identified as therapeutic candidates for adrenal cortical carcinoma by the CMap analysis. Results: Compared to survival group, we found 475 up-regulated genes and 354 genes and the key pathways leading to the death of different ACC individual patients. Then we used 12 topological analysis methods to found the most possible 22 hub-genes. Among these hub-genes, nine hub-genes (C3, CXCL5, CX3CR1, GRM8, HCAR2, HTR1B, SUCNR1, PTGER3 and SSTR1) could be used to distinguish the death and survival groups for patients. We also revealed that mRNA expressions of 12 genes (C3, CXCL8, CX3CR1, GNAT3, GNGT1, GRM8, HCAR2, HTR1B, HTR1D, PTGER3, SSTR1 and SUCNR1) and four key miRNAs (hsa-mir-330, hsa-mir-489, hsa-mir-508 and hsa-mir-513b) were related to survival. Three most small molecules were identified (H-9, AZ-628 and phensuximide) as potential therapeutic drugs for adrenal cortical carcinoma. Conclusion: The hub-genes expression was significant useful in adrenal cortical carcinoma, provide new diagnostic, prognosis and therapeutic approaches for adrenal cortical carcinoma. Furthermore, we also explore the possible miRNAs involved in regulation of hub-genes.
  
  
  
  
**GRM8**
-
10
of
10
  

### Novel Molecular Hallmarks of Group 3 Medulloblastoma by Single-Cell Transcriptomics.

10. Front Oncol. 2021 Mar 18;11:622430. doi: 10.3389/fonc.2021.622430 eCollection 2021.
  
  
Qin C(1), Pan Y(1), Li Y(1), Li Y(1), Long W(1), Liu Q(1).
  
  
Author information: (1)Department of Neurosurgery in Xiangya Hospital, Central South University, Cha ...
  
  
Medulloblastoma (MB) is a highly heterogeneous and one of the most malignant pediatric brain tumors, comprising four subgroups: Sonic Hedgehog, Wingless, Group 3, and Group 4. Group 3 MB has the worst prognosis of all MBs. However, the molecular and cellular mechanisms driving the maintenance of malignancy are poorly understood. Here, we employed high-throughput single-cell and bulk RNA sequencing to identify novel molecular features of Group 3 MB, and found that a specific cell cluster displayed a highly malignant phenotype. Then, we identified the glutamate receptor metabotropic 8 (GRM8), and AP-1 complex subunit sigma-2 (AP1S2) genes as two critical markers of Group 3 MB, corresponding to its poor prognosis. Information on 33 clinical cases was further utilized for validation. Meanwhile, a global map of the molecular cascade downstream of the MYC oncogene in Group 3 MB was also delineated using single-cell RNA sequencing. Our data yields new insights into Group 3 MB molecular characteristics and provides novel therapeutic targets for this relentless disease.
  
  
  
  
  

---

  

# HIVEP3

**Signature gene in:** Stromal cells
  
**Differentially expressed in:** Stromal cells
  
  
**HIVEP3**
-
1
of
10
  

### Placental methylation and pro-inflammatory protein levels in cord blood.

1. Placenta. 2024 Dec;158:231-239. doi: 10.1016/j.placenta.2024.10.067 Epub 2024 Oct 30.
  
  
van Otterdijk SD(1), Binder AM(2), Michels KB(3).
  
  
Author information: (1)Institute for Prevention and Cancer Epidemiology, Faculty of Medicine and Med ...
  
  
INTRODUCTION: The neonates' inflammatory response may in part be shaped during development by the placental epigenome, but evidence is scarce. We investigated the association between placental DNA methylation and pro-inflammatory proteins in cord blood. METHODS: A total of 249 mother-child dyads from the Harvard Epigenetic Birth Cohort were included in this study. Genome-wide methylation in placental DNA was assessed using the Illumina Human Methylation 450 Bead Chip array and verified by pyrosequencing. Cord blood inflammation markers assessed were interleukin-6, interleukin-8 and tumor necrosis factor α, intercellular adhesion molecule 1, serum amyloid A, and C-reactive protein. RESULTS: We identified differential placental DNA methylation of three loci in the HIVEP3 gene shore region that were associated with TNFα protein levels in cord blood. TNFα levels were associated with mode of delivery, gestational age and birth order. Three other loci located in the open sea region of the BCL11B gene were associated with SAA protein levels in cord blood. SAA levels were associated with birthweight, gestational age, and infant sex. CONCLUSIONS: Our results suggest a potential role for HIVEP3 and BCL11B placental DNA methylation in the acute immune response of the neonate. These immune markers were correlated with several mother and child characteristics.
  
  
  
  
**HIVEP3**
-
2
of
10
  

### HIVEP3 as a potential prognostic factor promotes the development of acute myeloid leukemia.

2. Growth Factors. 2023 Feb;41(1):43-56. doi: 10.1080/08977194.2022.2158329 Epub 2022 Dec 26.
  
  
Tang Y(1), Xu G(2), Hu B(3), Zhu Y(4).
  
  
Author information: (1)Department of Pediatrics, The Second Affiliated Hospital of Jiaxing Universit ...
  
  
Acute myeloid leukemia (AML) is a common malignancy worldwide. Human immune deficiency virus type 1 enhancer-binding protein 3 (HIVEP3) was verified to play a vital role in types of cancers. However, the functional role of HIVEP3 in AML was rarely reported. In this study, CCK-8, colony formation assay, flow cytometry, and Trans-well chamber experiments were applied for detecting cell proliferation, apoptosis, and invasion in AML cells. The expression of proteins related to TGF-β/Smad signaling pathway was determined by western blot. Our data showed that the expression level of HIVEP3 was closely related to the risk classification and prognosis of AML patients. Moreover, HIVEP3 was highly expressed in AML patients and cells. Knockdown of HIVEP3 significantly repressed cell proliferation invasion, and enhanced cell apoptosis in HL-60 and THP-1 cells. In addition, HIVEP3 donwreglation could inhibit the TGF-β/Smad signaling pathway. TGF-β overexpression could reverse the inhibition effects of HIVEP3 knockdown on AML development and the TGF-β/Smad signaling pathway. These findings indicated that HIVEP3 contributed to the progression of AML via regulating the TGF-β/Smad signaling pathway and had a prognostic value for AML.
  
  
  
  
**HIVEP3**
-
3
of
10
  

### Clinical significance of germline telomere length and associated genetic factors in patients with neuroblastoma.

3. Sci Rep. 2022 Jul 28;12(1):12954. doi: 10.1038/s41598-022-17246-4
  
  
Bae JS(#)(1), Lee JW(#)(2), Joung JG(3), Cho HW(2), Ju HY(2), Yoo KH(2), Koo HH(2), Sung KW(4).
  
  
Author information: (1)Research Institute for Future Medicine, Samsung Medical Center, Seoul, Republ ...
  
  
Studies investigating the relationship between germline telomere length and the clinical characteristics of tumors are very limited. This study evaluated the relationship between germline telomere length and the clinical characteristics of neuroblastoma. In addition, a genome-wide association study (GWAS) was performed to investigate the genetic factors associated with germline telomere length. The germline telomere length of peripheral blood mononuclear cells from 186 patients with neuroblastoma was measured by quantitative polymerase chain reaction. The association between germline telomere length and clinical characteristics, including long-term survival, was investigated. For the GWAS, genotyping was performed with a high-density bead chip (Illumina, San Diego, CA, USA). After strict quality-control checks of the samples, an association analysis was conducted. The result showed that longer germline telomeres were significantly associated with longer event-free survival (P = 0.032). To identify significantly assocated genetic markers for germline telomere length, genome wide association analysis was performed. As a result, several single nucleotide polymorphisms located in HIVEP3, LRRTM4, ADGRV1, RAB30, and CHRNA4 genes were discovered. During gene-based analysis (VEGAS2 tool), the CNTN4 gene had the most significant association with germline telomere length (P = 1.0E-06). During gene ontology analysis, susceptible genes associated with germline telomere length were mainly distributed in neurite morphogenesis and neuron development. A longer germline telomere length is associated with favorable prognostic factors at diagnosis and eventually better event-free survival in patients with neuroblastoma. In addition, the GWAS demonstrated that genetic markers and genes related to germline telomere length are associated with neurite morphogenesis and neuron development. Further research with larger cohorts of patients and functional investigations are needed.
  
  
  
  
**HIVEP3**
-
4
of
10
  

### HIVEP3 cooperates with ferroptosis gene signatures to confer adverse prognosis in acute myeloid leukemia.

4. Cancer Med. 2022 Dec;11(24):5050-5065. doi: 10.1002/cam4.4806 Epub 2022 May 10.
  
  
Zhang X(1), Zhang X(2), Liu K(1), Li W(1), Wang J(1), Liu P(1), Ma W(1).
  
  
Author information: (1)Department of Clinical Laboratory Medicine, The First Affiliated Hospital of ...
  
  
BACKGROUND: The human immunodeficiency virus type I enhancer binding protein (HIVEP) family, which contains zinc finger and acid-rich (ZAS) domains, has been demonstrated to be implicated in vital biological processes, such as cell survival, tumor necrosis factor (TNF) signaling, and tumor formation. However, its expression patterns, prognostic relevance, and functional implications in acute myeloid leukemia (AML) remain elusive. METHODS: We inspected HIVEP mRNA expression levels in datasets from The Cancer Genome Atlas (TCGA) and GSE24006. Survival analyses were orchestrated using the web-based bioinformatics platforms and R studio in two AML cohorts. Prognostic value and capacity were assessed by Cox regression analyses. Association of HIVEP3 expression levels with clinical characteristics were analyzed with R and UALCAN. Subsequentially, functional enrichment analyses were operated to interpret HIVEP3 co-expressed gene clusters. A prognostic gene signature was created by the least absolute shrinkage and selection operator (LASSO) regression algorithm. Moreover, bone marrow aspirate smears of AML patients were stained for HIVEP3 by immunohistochemistry (IHC). HIVEP3 expression was examined by qRT-PCR in leukemia cell lines treated with ferroptosis compounds in vitro. RESULTS: Augmented transcriptional levels of HIVEP2 and 3 were noted in AML patients (p<0.001). HIVEP3 not only could confer adverse prognosis independently in AML patients, but also was associated with AML subtypes, age, cytogenetic risk, and disease-related molecules. Co-expressed gene clusters of HIVEP3 were enriched in functional pathways related to AML leukemogenesis, such as ribosome, metabolism, and calcium signaling. Combined with multiple tumorigenesis signaling pathways, we proposed an integrated LASSO model with HIVEP3 and ferroptosis regulators AIFM2 and LPCAT3, to predict the outcome for AML patients. Furthernore, altered HIVEP3 expression at the mRNA or protein level was confirmed in sorted leukemia cells and blast cells in bone marrow tissues. In vitro experiments authenticated the involvement of HIVEP3 in ferroptosis signaling pathways. CONCLUSIONS: Our findings suggest that HIVEP3 is a de novo independent prognostic indicator, and the crosstalk between HIVEP3 and ferroptosis signaling pathways may inspire a specific perspective on the oncological network of AML.
  
  
  
  
**HIVEP3**
-
5
of
10
  

### Characterization of epigenetic and transcriptional landscape in infantile hemangiomas with ATAC-seq and RNA-seq.

5. Epigenomics. 2020 Jun;12(11):893-905. doi: 10.2217/epi-2020-0060 Epub 2020 Mar 30.
  
  
Li X(1), Chen Y(2), Fu C(1), Li H(1), Yang K(1), Bi J(1), Huo R(1).
  
  
Author information: (1)Department of Plastic Surgery, Shandong Provincial Hospital Affiliated to Sha ...
  
  
Aim: This study was conducted to reveal epigenetic landscape in infantile hemangiomas (IHs) and identify transcription factors (TFs) and their downstream genes active in IHs. Materials & methods: We performed Assay for Transposase Accessible Chromatin (ATAC-seq) with RNA-seq in three pairs of IHs and their adjacent normal tissues. Functions of candidate TFs were investigated in human umbilical vein endothelial cells (HUVECs). Results: Chromatin of IH tissues is less compact. Some candidate genes and TFs were identified. In HUVECs, SPDEF inhibited cell viability and tube formation, and promoted apoptosis; SOX4 exerted the opposite effect. SPDEF may act through EPHA5, ZBTB46 and SASH1; SOX4 may act through MMP12 and HIVEP3. Conclusion: Epigenetics plays a role in IHs. SPDEF and SOX4 may act in IHs.
  
  
  
  
**HIVEP3**
-
6
of
10
  

### Decoding Somatic Driver Gene Mutations and Affected Signaling Pathways in Human Medulloblastoma Subgroups.

6. J Cancer. 2018 Nov 17;9(24):4596-4610. doi: 10.7150/jca.27993 eCollection 2018.
  
  
Robbins CJ(1), Bou-Dargham MJ(1), Sanchez K(1), Rosen MC(1), Sang QA(1).
  
  
Author information: (1)Department of Chemistry & Biochemistry, Institute of Molecular Biophysics, Fl ...
  
  
Medulloblastoma is the most common malignant pediatric brain tumor. Prior studies have concentrated their efforts studying the four molecular subgroups: SHH, Wnt, group 3, and group 4. SHH and Wnt are driven by their canonical pathways. Groups 3 and 4 are highly metastatic and associated with aberrations in epigenetic regulators. Recent developments in the field have revealed that these subgroups are not as homogenous as previously believed. The objective of this study is to investigate the involvement of somatic driver gene mutations in these medulloblastoma subgroups. We obtained medulloblastoma data from the Catalogue of Somatic Mutations in Cancer (COSMIC), which contains distinct samples that were not previously studied in a large cohort. We identified somatic driver gene mutations and the signaling pathways affected by these driver genes for medulloblastoma subgroups using bioinformatics tools. We have revealed novel infrequent drivers in these subgroups that contribute to our understanding of tumor heterogeneity in medulloblastoma. Normally SHH signaling is activated in the SHH subgroup, however, we determined gain-of-function mutations in ubiquitin ligase (CUL1) that inhibit Gli-mediated transcription. This suggests a potential hindrance in SHH signaling for some patients. For group 3, gain-of-function in the inhibitor of proinflammatory cytokines (HIVEP3) suggests an immunosuppressive phenotype and thus a more hostile tumor microenvironment. Surprisingly, group 4 tumors possess mutations that may prompt the activation of Wnt signaling through gain-of-function mutations in MUC16 and PCDH9. These infrequent mutations detected in this study could be due to subclonal or spatially restricted alterations. The investigation of aberrant driver gene mutations can lead to the identification of new drug targets and a greater understanding of human medulloblastoma heterogeneity.
  
  
  
  
**HIVEP3**
-
7
of
10
  

### Increased sensitivity of rheumatoid synoviocytes to Schnurri-3 expression in TNF-α and IL-17A induced osteoblastic differentiation.

7. Bone. 2016 Jun;87:89-96. doi: 10.1016/j.bone.2016.04.008 Epub 2016 Apr 9.
  
  
Lavocat F(1), Osta B(1), Miossec P(2).
  
  
Author information: (1)Department of clinical Immunology and Rheumatology, Immunogenomics and Inflam ...
  
  
OBJECTIVE: To compare the effects of TNF-α and IL-17A on osteogenic differentiation of isolated fibroblast-like synoviocytes (FLS) from healthy donors, osteoarthritis (OA) and rheumatoid arthritis (RA) patients. METHODS: FLS were cultured in osteogenic medium, with and without TNF-α and/or IL-17A. Extracellular matrix mineralization was evaluated by alizarin red staining and alkaline phosphatase activity (ALP) measurement. mRNA expression was analyzed by qRT-PCR for Wnt5a, BMP2 and Runx2, genes associated with osteogenesis, for DKK1 and RANKL, genes associated with osteogenesis inhibition and Schnurri-3, a new critical gene in the cross talk with osteoclasts. IL-6 and IL-8 production was measured by ELISA. RESULTS: In osteogenic medium, matrix mineralization and increased ALP activity indicated that FLS can undergo osteogenic differentiation, which was increased with TNF-α and IL-17A. The expression of osteogenesis activators (BMP2 and Wnt5a) was increased with cytokines and that of the osteogenesis inhibitor DKK1 was decreased. There was no difference between all three cell types. In contrast, RA FLS were particularly sensitive to the synergistic increase of Shn3 with TNF-α and IL-17A. Levels of IL-6 and IL-8 were also higher for RA-FLS, compared to healthy and OA FLS. CONCLUSION: IL-17A and/or TNF-α treatment favor an osteogenesis induction in isolated FLS, independent of their origin. RA-FLS were more sensitive to the synergistic increase of Schnurri-3 expression. Combined with the higher levels of inflammation, this may in turn activate osteoclastogenesis, leading to increased bone destruction seen in destructive arthritis.
  
  
  
  
**HIVEP3**
-
8
of
10
  

### Combined overexpression of HIVEP3 and SOX9 predicts unfavorable biochemical recurrence-free survival in patients with prostate cancer.

8. Onco Targets Ther. 2014 Jan 24;7:137-46. doi: 10.2147/OTT.S55432 eCollection 2014.
  
  
Qin GQ(1), He HC(2), Han ZD(2), Liang YX(2), Yang SB(2), Huang YQ(2), Zhou L(2), Fu H(2), Li JX(1), ...
  
  
Author information: (1)Central Hospital of Panyu District, Guangzhou, People's Republic of China ; D ...
  
  
BACKGROUND: To clarify the involvement of HIVEP3 and SOX9 coexpression in prostate cancer (PCa). METHODS: A small interfering RNA was used to knockdown SOX9 expression in a PCa cell line and to analyze the effects of SOX9 inhibition on the expression of HIVEP3 in vitro. Then, HIVEP3 and SOX9 expression patterns in the human PCa tissues were detected using quantitative reverse transcription polymerase chain reaction (qRT-PCR) analysis and immunohistochemistry. RESULTS: We found that the downregulation of SOX9 could inhibit the expression of HIVEP3 in the PCa cells in vitro. In addition, both HIVEP3 and SOX9 messenger RNA expression levels in the PCa tissues were significantly higher than those in the noncancerous prostate tissues (P=0.006 and P<0.001, respectively). Moreover, the immunohistochemical staining scores of HIVEP3 in the PCa tissues with PSA failure were significantly higher than those without (P=0.042); the increased SOX9 protein expression was more frequently found in the PCa tissues with a high Gleason score (P=0.045) and a high clinical stage (P=0.012). The tumors showing the HIVEP3-high/SOX9-high expression more frequently had PSA failure (P=0.024). When the patients with an HIVEP3 overexpression combined with the SOX9 overexpression, this group had a worse biochemical recurrence-free survival (P<0.001). Furthermore, the multivariate analysis showed that the HIVEP3/SOX9 coexpression was an independent predictor of an unfavorable biochemical recurrence-free survival. CONCLUSION: Our data offer the convincing evidence for the first time that a combined analysis of HIVEP3 and SOX9 may help to predict the tumor progression and prognosis of PCa patients. In particular, the overexpression of HIVEP3 in PCa might partly explain the poor prognosis of patients with an upregulation of SOX9.
  
  
  
  
**HIVEP3**
-
9
of
10
  

### ZAS3 promotes TNFα-induced apoptosis by blocking NFϰB-activated expression of the anti-apoptotic genes TRAF1 and TRAF2.

9. BMB Rep. 2011 Apr;44(4):267-72. doi: 10.5483/BMBRep.2011.44.4.267
  
  
Shin DH(1), Park KW, Wu LC, Hong JW.
  
  
Author information: (1)The Graduate School of East-West Medical Science, Kyung Hee University, Yongi ...
  
  
ZAS3 is a large zinc finger transcription repressor that binds the ϰB-motif via two signature domains of ZASN and ZASC. A loss-of-function study showed that lack of ZAS3 protein induced accelerated cell proliferation and tumorigenesis. Conversely, gain-of-function studies showed that ZAS3 repressed NFϰB-activated transcription by competing with NFϰB for the ϰB-motif. Based on these observations, we hypothesize that ZAS3 promotes apoptosis by interrupting anti-apoptotic activity of NFϰB. Here, we present evidence that upon TNFα stimulation, ZAS3 inhibits NFϰB-mediated cell survival and promotes caspase-mediated apoptosis. The inhibitory effect of ZAS3 on NFϰB activity is mediated by neither direct association with NFϰB nor disrupting nuclear localization of NFϰB. Instead, ZAS3 repressed the expression of two key anti-apoptotic genes of NFϰB, TRAF1 and TRAF2, thereby sensitizing cells to TNFα-induced cell death. Taken together, our data suggest that ZAS3 is a tumor suppressor gene and therefore serves as a novel therapeutic target for developing anti-cancer drugs.
  
  
  
  
**HIVEP3**
-
10
of
10
  

### Developmental anomalies and neoplasia in animals and cells deficient in the large zinc finger protein KRC.

10. Genes Chromosomes Cancer. 2002 Dec;35(4):287-98. doi: 10.1002/gcc.10128
  
  
Allen CE(1), Muthusamy N, Weisbrode SE, Hong JW, Wu LC.
  
  
Author information: (1)Program of Molecular, Cellular, and Developmental Biology, College of Medicin ...
  
  
The large zinc finger protein KRC binds to the signal sequences of V(D)J recombination and the kappaB motif. Disruption of KRC expression in cell lines resulted in increased cell proliferation, anchorage independence of growth, and uncoupling of nuclear division and cell division. In this report, the function of KRC was studied in a RAG2-deficient blastocyst complementation animal model. KRC-deficient embryonic stem cells were generated by homologous recombination and were introduced into RAG2(-/-) blastocysts to generate KRC(-/-);RAG2(-/-) chimeric mice. The lymphoid compartments of chimeras examined at 5 weeks of age were developed, suggesting that KRC is not essential for V(D)J recombination development. However, by 6 months of age, there was a marked deficit in CD4(+)CD8(+) thymocytes in the chimeras, suggesting that KRC may be involved in T-lymphocyte survival. Additionally, one chimera developed anomalies, including postaxial polydactyly, hydronephrosis, and an extragonadal malignant teratoma. DNA analysis showed that the teratoma was derived from KRC(-/-) embryonic stem cells. The teratoma had compound tissue organization and was infiltrated with B lymphocytes. Subsequently, several immortalized KRC-deficient cell lines were established from the teratoma. In this study, growth anomalies and neoplasia were observed in animals and cells deficient in KRC, and other studies have shown allelic loss occurring at the chromosomal region of the human KRC counterpart in various tumors. We propose that KRC may be a previously unidentified tumor-suppresser gene.
  
  
  
  
  

---

  

# ITPR2

**Signature gene in:** B cells, Plasma cells
  
**Differentially expressed in:** Plasma cells
  
  
**ITPR2**
-
1
of
10
  

### A model-free and distribution-free multi-omics integration approach for detecting novel lung adenocarcinoma genes.

1. Sci Rep. 2024 Aug 3;14(1):17996. doi: 10.1038/s41598-023-45813-w
  
  
Zhao S(1), Qi C(#)(2), Zhao G(#)(2), Wang Y(#)(2), Fu G(#)(3).
  
  
Author information: (1)Binghamton University, Department of Mathematics and Statistics, Binghamton, ...
  
  
Detection of important genes affecting lung adenocarcinoma (LUAD) is critical to finding effective therapeutic targets for this highly lethal cancer. However, many existing approaches have focused on single outcomes or phenotypic associations, which may not be as thorough as investigating molecular transcript levels within cells. In this article, we apply a novel multivariate rank-distance correlation-based gene selection procedure (MrDcGene) to LUAD multi-omics data downloaded from The Cancer Genome Atlas (TCGA). MrDcGene provides additional opportunities for detecting novel susceptibility genes as it leverages information from multiple platforms, while efficiently handling challenges such as high dimensionality, low signal-to-noise ratio, unknown distributions, and non-linear structures, etc. Notably, the MrDcGene method is able to detect two different scenarios, i.e., strong association strength with a few gene expressions and weak association strength with several gene expressions. After thoroughly exploring the association between gene expression (GE) and multiple other platforms, including reverse phase protein array (RPPA), miRNA, copy number variation (CNV) and DNA methylation (ME), we detect several novel genes that may play an important role in LUAD (ZNF133, CCDC159, YWHAZ, HNRNPR. ITPR2, PTHLH, and WIPI2). In addition, we quantitatively validate several other susceptibility genes that were reported in the literature using different methods and studies. The accuracy of the MrDcGene approach is theoretically assured and empirically demonstrated by the simulation studies.
  
  
  
  
**ITPR2**
-
2
of
10
  

### ScHGSC-IGDC: Identifying genes with differential correlations of high-grade serous ovarian cancer based on single-cell RNA sequencing analysis.

2. Heliyon. 2024 Jun 13;10(12):e32909. doi: 10.1016/j.heliyon.2024.e32909 eCollection 2024 Jun 30.
  
  
Li Y(1)(2)(3), Wang Q(1)(2)(3), Zheng X(1)(2)(3), Xu B(1)(2)(3), Hu W(2)(3)(4), Zhang J(5), Kong X(6 ...
  
  
Author information: (1)Tumor Biological Diagnosis and Treatment Center, The Third Affiliated Hospita ...
  
  
Due to the high heterogeneity of ovarian cancer (OC), it occupies the main cause of cancer-related death among women. As the most aggressive and frequent subtype of OC, high-grade serous cancer (HGSC) represents around 70 % of all patients. With the booming progress of single-cell RNA sequencing (scRNA-seq), unique and subtle changes among different cell states have been identified including novel risk genes and pathways. Here, our present study aims to identify differentially correlated core genes between normal and tumor status through HGSC scRNA-seq data analysis. R package high-dimension Weighted Gene Co-expression Network Analysis (hdWGCNA) was implemented for building gene interaction networks based on HGSC scRNA-seq data. DiffCorr was integrated for identifying differentially correlated genes between tumor and their adjacent normal counterparts. Software Cytoscape was implemented for constructing and visualizing biological networks. Real-time qPCR (RT-qPCR) was utilized to confirm expression pattern of new genes. We introduced ScHGSC-IGDC (Identifying Genes with Differential Correlations of HGSC based on scRNA-seq analysis), an in silico framework for identifying core genes in the development of HGSC. We detected thirty-four modules in the network. Scores of new genes with opposite correlations with others such as NDUFS5, TMSB4X, SERPINE2 and ITPR2 were identified. Further survival and literature validation emphasized their great values in the HGSC management. Meanwhile, RT-qPCR verified expression pattern of NDUFS5, TMSB4X, SERPINE2 and ITPR2 in human OC cell lines and tissues. Our research offered novel perspectives on the gene modulatory mechanisms from single cell resolution, guiding network based algorithms in cancer etiology field.
  
  
  
  
**ITPR2**
-
3
of
10
  

### RNA Sequencing of Intraoperative Peritumoral Tissues Reveals Potential Pathways Involved in Glioma-Related Seizures.

3. J Mol Neurosci. 2023 Jun;73(6):437-447. doi: 10.1007/s12031-023-02125-y Epub 2023 Jun 2.
  
  
Kumar K(1), Dubey V(2), Zaidi SS(2), Tripathi M(1), Siraj F(3), Sharma MC(4), Chandra PS(5), Doddama ...
  
  
Author information: (1)Department of Neurology, AIIMS, New Delhi, India. (2)Department of Biophysics ...
  
  
Tumor-induced changes in the peritumoral neocortex play a crucial role in generation of seizures. This study aimed to investigate the molecular mechanisms potentially involved in peritumoral epilepsy in low-grade gliomas (LGGs). Intraoperative peritumoral brain tissues resected from LGG patients with seizures (pGRS) or without seizures (pGNS) were used for RNA sequencing (RNA-seq). Comparative transcriptomics was performed to identify differentially expressed genes (DEGs) in pGRS compared to pGNS using deseq2 and edgeR packages (R). Gene set enrichment analysis (GSEA) using Gene Ontology terms and Kyoto Encyclopedia of Genes & Genomes (KEGG) pathways was performed using the clusterProfiler package (R). The expression of key genes was validated at the transcript and protein levels in the peritumoral region using real-time PCR and immunohistochemistry, respectively. A total of 1073 DEGs were identified in pGRS compared to pGNS, of which 559 genes were upregulated and 514 genes were downregulated (log2 fold-change ≥ 2, padj < 0.001). The DEGs in pGRS were highly enriched in the "Glutamatergic Synapse" and "Spliceosome" pathways, with increased expression of GRIN2A (NR2A), GRIN2B (NR2B), GRIA1 (GLUR1), GRIA3 (GLUR3), GRM5, CACNA1C, CACNA1A, and ITPR2. Moreover, increased immunoreactivity was observed for NR2A, NR2B, and GLUR1 proteins in the peritumoral tissues of GRS. These findings suggest that altered glutamatergic signaling and perturbed Ca2+ homeostasis may be potential causes of peritumoral epilepsy in gliomas. This explorative study identifies important genes/pathways that merit further characterization for their potential involvement in glioma-related seizures.
  
  
  
  
**ITPR2**
-
4
of
10
  

### Identification of the novel therapeutic targets and biomarkers associated of prostate cancer with cancer-associated fibroblasts (CAFs).

4. Front Oncol. 2023 Mar 3;13:1136835. doi: 10.3389/fonc.2023.1136835 eCollection 2023.
  
  
Zhai X(1), Chen X(1), Wan Z(1), Ge M(1), Ding Y(1), Gu J(1), Hua J(1), Guo D(1), Tan M(1), Xu D(1).
  
  
Author information: (1)Urology Centre, Shuguang Hospital Affiliated to Shanghai University of Tradit ...
  
  
Globally, prostate cancer remains a leading cause of mortality and morbidity despite advances in treatment. Research on prostate cancer has primarily focused on the malignant epithelium, but the tumor microenvironment has recently been recognized as an important factor in the progression of prostate cancer. Cancer-associated fibroblasts (CAFs) play an important role in prostate cancer progression among multiple cell types in the tumor microenvironment. In order to develop new treatments and identify predictive and prognostic biomarkers for CAFs, further research is needed to understand the mechanism of action of prostate cancer and CAF. In this work, we performed the single-cell RNA sequence analysis to obtain the biomarkers for CAFs, and ten genes were finally regarded as the marker genes for CAFs. Based on the ssGSEA algorithm, the prostate cancer cohort was divided into low- and high-CAFs groups. Further analysis revealed that the CAFs-score is associated with many immune-related cells and immune-related pathways. In addition, between the low- and high-CAFs tissues, a total of 127 hub genes were discovered, which is specific in CAFs. After constructing the prognostic prediction model, SLPI, VSIG2, CENPF, SLC7A1, SMC4, and ITPR2 were finally regarded as the key genes in the prognosis of patients with prostate cancer. Each patient was assigned with the risk score as follows: SLPI\* 0.000584811158157081 + VSIG2 \* -0.01190627068889 + CENPF \* -0.317826812875334 + SLC7A1 \* -0.0410213995358753 + SMC4 \* 0.202544454923637 + ITPR2 \* -0.0824652047622673 + TOP2A \* 0.140312081524807 + OR51E2 \* -0.00136602095885459. The GSVA revealed the biological features of CAFs, many cancer-related pathways, such as the adipocytokine signaling pathway, ERBB signaling pathway, GnRH signaling pathway, insulin signaling pathway, mTOR signaling pathway and PPAR signaling pathway are closely associated with CAFs. As a result of these observations, similar transcriptomics may be involved in the transition from normal fibroblasts to CAFs in adjacent tissues. As one of the biomarkers for CAFs, CENPF can promote the proliferation ability of prostate cancer cells. The overexpress of CENPF could promote the proliferation ability of prostate cancer cells. In conclusion, we discuss the potential prognostic and therapeutic value of CAF-dependent pathways in prostate cancer.
  
  
  
  
**ITPR2**
-
5
of
10
  

### Genomic profiling and network-level understanding uncover the potential genes and the pathways in hepatocellular carcinoma.

5. Front Genet. 2022 Nov 21;13:880440. doi: 10.3389/fgene.2022.880440 eCollection 2022.
  
  
El-Kafrawy SA(1)(2), El-Daly MM(1)(2), Bajrai LH(1)(3), Alandijany TA(1)(2), Faizo AA(1)(2), Mobashi ...
  
  
Author information: (1)Special Infectious Agents Unit-BSL3, King Fahd Medical Research Centre, King ...
  
  
Data integration with phenotypes such as gene expression, pathways or function, and protein-protein interactions data has proven to be a highly promising technique for improving human complex diseases, particularly cancer patient outcome prediction. Hepatocellular carcinoma is one of the most prevalent cancers, and the most common cause is chronic HBV and HCV infection, which is linked to the majority of cases, and HBV and HCV play a role in multistep carcinogenesis progression. We examined the list of known hepatocellular carcinoma biomarkers with the publicly available expression profile dataset of hepatocellular carcinoma infected with HCV from day 1 to day 10 in this study. The study covers an overexpression pattern for the selected biomarkers in clinical hepatocellular carcinoma patients, a combined investigation of these biomarkers with the gathered temporal dataset, temporal expression profiling changes, and temporal pathway enrichment following HCV infection. Following a temporal analysis, it was discovered that the early stages of HCV infection tend to be more harmful in terms of expression shifting patterns, and that there is no significant change after that, followed by a set of genes that are consistently altered. PI3K, cAMP, TGF, TNF, Rap1, NF-kB, Apoptosis, Longevity regulating pathway, signaling pathways regulating pluripotency of stem cells, Cytokine-cytokine receptor interaction, p53 signaling, Wnt signaling, Toll-like receptor signaling, and Hippo signaling pathways are just a few of the most commonly enriched pathways. The majority of these pathways are well-known for their roles in the immune system, infection and inflammation, and human illnesses like cancer. We also find that ADCY8, MYC, PTK2, CTNNB1, TP53, RB1, PRKCA, TCF7L2, PAK1, ITPR2, CYP3A4, UGT1A6, GCK, and FGFR2/3 appear to be among the prominent genes based on the networks of genes and pathways based on the copy number alterations, mutations, and structural variants study.
  
  
  
  
**ITPR2**
-
6
of
10
  

### Inositol 1,4,5-trisphosphate receptor gene variants are related to the risk of breast cancer in a Chinese population.

6. J Gene Med. 2023 Feb;25(2):e3463. doi: 10.1002/jgm.3463 Epub 2022 Nov 22.
  
  
Chen J(1)(2), Zhou J(1), Jiang Y(1)(2), Wang Y(1), Chen C(1)(2), Jiang T(3), Du J(1).
  
  
Author information: (1)Department of Epidemiology, International Joint Research Center on Environmen ...
  
  
BACKGROUND: Mammalian inositol 1,4,5-trisphosphate receptor (ITPR) genes encode ubiquitously expressed endoplasmic reticulum Ca2+ channels that have recently been shown to be closely linked to the pathogenesis of several cancers. However, few studies to date have explored associations between ITPR gene family single nucleotide polymorphisms (SNPs) and breast cancer risk. METHODS: In the present case-control study, 12 SNPs in the potential functional regions of the ITPR1, ITPR2, and ITPR3 genes were genotyped using an Illumina Infinium® Beadchip in 2095 Chinese women (1032 cases and 1063 controls). RESULTS: Multivariate logistic regression analyses indicated that a missense SNP in the ITPR3 coding region (rs2229642) was significantly related to breast cancer risk when using an additive model in this study (rs2229642-adjusted odds ratio = 1.40, 95% confidence interval = 1.12-1.74, p = 2.97 × 10^-3^ ). Expression quantitative trait loci analyses indicated that the SNP rs2229642 was associated with reduced ITPR3 expression levels (p = 3.2 × 10^-7^ ) and with marked reductions in the expressions of several proximal genes, including BAK1, GRM4, HLA-DOB, and UQCC2 (p = 0.013, 0.018, 3.4 × 10^-3^ , 3.8 × 10^-5^ ), suggesting that it may further regulate other genes associated with oncogenic susceptibility. Kaplan-Meier analyses indicated that the patients with higher ITPR3 expression exhibited significantly poorer outcomes compared to the patients with lower expression of this gene (hazard ratio = 1.11, 95% confidence interval = 1-1.23, p = 0.046). CONCLUSIONS: The results indicated that genetic variant in the coding region of ITPR3 gene may regulate the expressions of its host and some other cancer-related genes, as well as act as potential predictive biomarker for susceptibility to breast cancer in the Chinese population.
  
  
  
  
**ITPR2**
-
7
of
10
  

### Transcriptional ITPR3 as potential targets and biomarkers for human pancreatic cancer.

7. Aging (Albany NY). 2022 May 17;14(10):4425-4444. doi: 10.18632/aging.204080 Epub 2022 May 17.
  
  
Zheng W(1)(2)(3), Bai X(4), Zhou Y(1)(2), Yu L(1)(2), Ji D(2)(5), Zheng Y(6), Meng N(1), Wang H(1), ...
  
  
Author information: (1)Department of Hepatopancreatobiliary Surgery, Second Affiliated Hospital of H ...
  
  
Inositol 1,4,5-Triphosphate Receptor Family (ITPRs) are necessary intracellular Ca2+-release channel encoders and participate in mammalian cell physiological and pathological processes. Previous studies have suggested that ITPRs participate in tumorigenesis of multiple cancers. Nevertheless, the diverse expression profiles and prognostic significance of three ITPRs in pancreatic cancer have yet to be uncovered. In this work, we examined the expression levels and survival dates of ITPRs in patients with pancreatic cancer. As a result, we identified that ITPR1 and ITPR3 expression levels are significantly elevated in cancerous specimens. Survival data revealed that over-expression of ITPR2 and ITPR3 resulted in unfavourable overall survival and pathological stage. The multivariate Cox logistic regression analysis showed that ITPR3 could be an independent risk factor for PAAD patient survival. Moreover, to investigate how ITPRs work, co-expressed genes, alterations, protein-protein interaction, immune infiltration, methylation, and functional enrichment of ITPRs were also analyzed. Then, we evaluated these findings in clinical samples. Moreover, the gain and loss of function of ITPR3 were also conducted. The electron microscope assay was employed to explore the role of ITPR3 in pancreatic cancer cell lines' endoplasmic reticulum stress. In summary, our findings demonstrated that ITPR3 has the potential to be drug targets and biomarkers for human pancreatic cancer.
  
  
  
  
**ITPR2**
-
8
of
10
  

### The multifaceted regulation of mitophagy by endogenous metabolites.

8. Autophagy. 2022 Jun;18(6):1216-1239. doi: 10.1080/15548627.2021.1975914 Epub 2021 Sep 29.
  
  
Zhang T(1)(2)(3), Liu Q(1)(2)(3), Gao W(2)(3)(4), Sehgal SA(5), Wu H(1)(2)(3).
  
  
Author information: (1)State Key Laboratory of Agricultural Microbiology, College of Veterinary Medi ...
  
  
Owing to the dominant functions of mitochondria in multiple cellular metabolisms and distinct types of regulated cell death, maintaining a functional mitochondrial network is fundamental for the cellular homeostasis and body fitness in response to physiological adaptations and stressed conditions. The process of mitophagy, in which the dysfunctional or superfluous mitochondria are selectively engulfed by autophagosome and subsequently degraded in lysosome, has been well formulated as one of the major mechanisms for mitochondrial quality control. To date, the PINK1-PRKN-dependent and receptors (including proteins and lipids)-dependent pathways have been characterized to determine the mitophagy in mammalian cells. The mitophagy is highly responsive to the dynamics of endogenous metabolites, including iron-, calcium-, glycolysis-TCA-, NAD+-, amino acids-, fatty acids-, and cAMP-associated metabolites. Herein, we summarize the recent advances toward the molecular details of mitophagy regulation in mammalian cells. We also highlight the key regulations of mammalian mitophagy by endogenous metabolites, shed new light on the bidirectional interplay between mitophagy and cellular metabolisms, with attempting to provide a perspective insight into the nutritional intervention of metabolic disorders with mitophagy deficit.Abbreviations: acetyl-CoA: acetyl-coenzyme A; ACO1: aconitase 1; ADCYs: adenylate cyclases; AMPK: AMP-activated protein kinase; ATM: ATM serine/threonine kinase; BCL2L1: BCL2 like 1; BCL2L13: BCL2 like 13; BNIP3: BCL2 interacting protein 3; BNIP3L: BCL2 interacting protein 3 like; Ca2+: calcium ion; CALCOCO2: calcium binding and coiled-coil domain 2; CANX: calnexin; CO: carbon monoxide; CYCS: cytochrome c, somatic; DFP: deferiprone; DNM1L: dynamin 1 like; ER: endoplasmic reticulum; FKBP8: FKBP prolyl isomerase 8; FOXO3: forkhead box O3; FTMT: ferritin mitochondrial; FUNDC1: FUN14 domain containing 1; GABA: γ-aminobutyric acid; GSH: glutathione; HIF1A: hypoxia inducible factor 1 subunit alpha; IMMT: inner membrane mitochondrial protein; IRP1: iron regulatory protein 1; ISC: iron-sulfur cluster; ITPR2: inositol 1,4,5-trisphosphate type 2 receptor; KMO: kynurenine 3-monooxygenase; LIR: LC3 interacting region; MAM: mitochondria-associated membrane; MAP1LC3: microtubule associated protein 1 light chain 3; MFNs: mitofusins; mitophagy: mitochondrial autophagy; mPTP: mitochondrial permeability transition pore; MTOR: mechanistic target of rapamycin kinase; NAD+: nicotinamide adenine dinucleotide; NAM: nicotinamide; NMN: nicotinamide mononucleotide; NO: nitric oxide; NPA: Niemann-Pick type A; NR: nicotinamide riboside; NR4A1: nuclear receptor subfamily 4 group A member 1; NRF1: nuclear respiratory factor 1; OPA1: OPA1 mitochondrial dynamin like GTPase; OPTN: optineurin; PARL: presenilin associated rhomboid like; PARPs: poly(ADP-ribose) polymerases; PC: phosphatidylcholine; PHB2: prohibitin 2; PINK1: PTEN induced kinase 1; PPARG: peroxisome proliferator activated receptor gamma; PPARGC1A: PPARG coactivator 1 alpha; PRKA: protein kinase AMP-activated; PRKDC: protein kinase, DNA-activated, catalytic subunit; PRKN: parkin RBR E3 ubiquitin protein ligase; RHOT: ras homolog family member T; ROS: reactive oxygen species; SIRTs: sirtuins; STK11: serine/threonine kinase 11; TCA: tricarboxylic acid; TP53: tumor protein p53; ULK1: unc-51 like autophagy activating kinase 1; VDAC1: voltage dependent anion channel 1.
  
  
  
  
**ITPR2**
-
9
of
10
  

### Ten-eleven translocation 1 mediated-DNA hydroxymethylation is required for myelination and remyelination in the mouse brain.

9. Nat Commun. 2021 Aug 24;12(1):5091. doi: 10.1038/s41467-021-25353-5
  
  
Zhang M(#)(1), Wang J(#)(1), Zhang K(#)(1), Lu G(1), Liu Y(1), Ren K(1), Wang W(1), Xin D(2), Xu L(3 ...
  
  
Author information: (1)Department of Neurobiology, School of Basic Medicine, Fourth Military Medical ...
  
  
Ten-eleven translocation (TET) proteins, the dioxygenase for DNA hydroxymethylation, are important players in nervous system development and diseases. However, their role in myelination and remyelination after injury remains elusive. Here, we identify a genome-wide and locus-specific DNA hydroxymethylation landscape shift during differentiation of oligodendrocyte-progenitor cells (OPC). Ablation of Tet1 results in stage-dependent defects in oligodendrocyte (OL) development and myelination in the mouse brain. The mice lacking Tet1 in the oligodendrocyte lineage develop behavioral deficiency. We also show that TET1 is required for remyelination in adulthood. Transcriptomic, genomic occupancy, and 5-hydroxymethylcytosine (5hmC) profiling reveal a critical TET1-regulated epigenetic program for oligodendrocyte differentiation that includes genes associated with myelination, cell division, and calcium transport. Tet1-deficient OPCs exhibit reduced calcium activity, increasing calcium activity rescues the differentiation defects in vitro. Deletion of a TET1-5hmC target gene, ITPR2, impairs the onset of OPC differentiation. Together, our results suggest that stage-specific TET1-mediated epigenetic programming and intracellular signaling are important for proper myelination and remyelination in mice.
  
  
  
  
**ITPR2**
-
10
of
10
  

### Characteristics of genetic alterations of peripheral T-cell lymphoma in childhood including identification of novel fusion genes: the Japan Children's Cancer Group (JCCG).

10. Br J Haematol. 2021 Aug;194(4):718-729. doi: 10.1111/bjh.17639 Epub 2021 Jul 13.
  
  
Ohki K(1), Kiyokawa N(1), Watanabe S(1), Iwafuchi H(1)(2), Nakazawa A(3), Ishiwata K(4), Ogata-Kawat ...
  
  
Author information: (1)Department of Pediatric Hematology and Oncology Research, National Research I ...
  
  
Peripheral T-cell lymphoma (PTCL) is a group of heterogeneous non-Hodgkin lymphomas showing a mature T-cell or natural killer cell phenotype, but its molecular abnormalities in paediatric patients remain unclear. By employing next-generation sequencing and multiplex ligation-dependent probe amplification of tumour samples from 26 patients, we identified somatic alterations in paediatric PTCL including Epstein-Barr virus (EBV)-negative (EBV- ) and EBV-positive (EBV+ ) patients. As recurrent mutational targets for PTCL, we identified several previously unreported genes, including TNS1, ZFHX3, LRP2, NCOA2 and HOXA1, as well as genes previously reported in adult patients, e.g. TET2, CDKN2A, STAT3 and TP53. However, for other reported mutations, VAV1-related abnormalities were absent and mutations of NRAS, GATA3 and JAK3 showed a low frequency in our cohort. Concerning the association of EBV infection, two novel fusion genes: STAG2-AFF2 and ITPR2-FSTL4, and deletion and alteration of CDKN2A/2B, LMO1 and HOXA1 were identified in EBV- PTCL, but not in EBV+ PTCL. Conversely, alterations of PCDHGA4, ADAR, CUL9 and TP53 were identified only in EBV+ PTCL. Our observations suggest a clear difference in the molecular mechanism of onset between paediatric and adult PTCL and a difference in the characteristics of genetic alterations between EBV- and EBV+ paediatric PTCL.
  
  
  
  
  

---

  

# KIAA1755

**Signature gene in:** Stromal cells
  
**Differentially expressed in:** Stromal cells
  
  
**KIAA1755**
-
1
of
4
  

### High Expression Levels of the Long Non-Coding RNAs Lnc-IRF2-3 and Lnc-KIAA1755-4 Are Markers of Poor Prognosis in Chronic Lymphocytic Leukemia.

1. Int J Mol Sci. 2025 Jan 29;26(3):1153. doi: 10.3390/ijms26031153
  
  
Tosic N(1), Tomic Vujovic K(2), Vukovic V(2)(3), Kotur N(1), Stankovic B(1), Marjanovic I(1), Antic ...
  
  
Author information: (1)Institute of Molecular Genetics and Genetic Engineering, University of Belgra ...
  
  
Long non-coding RNAs (lncRNAs) play complex roles at multiple levels of gene regulation, thus modulating key cellular processes involved in the pathogenesis and progression of cancer. Aberrant expression of lncRNAs has been reported in various malignancies, including chronic lymphocytic leukemia (CLL). We investigated the expression of lnc-IRF2-3 and lnc-KIAA1755-4 in peripheral blood mononuclear cells of 112 previously untreated CLL patients by quantitative reverse-transcriptase polymerase chain reaction. Both lncRNAs were found to be overexpressed in CLL samples in comparison to healthy controls, and their high levels were associated with adverse clinico-biological characteristics of patients at diagnosis. High lnc-IRF2-3 expression was associated with high leukocyte and lymphocyte counts, high β2-microglobulin, advanced Binet stage, unfavorable cytogenetics, CD38-positivity and IGHV-unmutated status. Regarding lnc-KIAA1755-4, its high expression was associated with high leukocyte count, lymphocyte count, β2-microglobulin, lactate dehydrogenase and low hemoglobin, as well as with IGHV-unmutated status. In addition, we observed shorter time to first treatment and overall survival of patients expressing high levels of both lncRNAs in comparison to low-expressing patients. In summary, our study showed that high lnc-IRF2-3 and lnc-KIAA1755-4 expression at diagnosis predicts poor survival in CLL. The mechanisms of their upregulation, as well as their specific targets in CLL cells, remain to be elucidated.
  
  
  
  
**KIAA1755**
-
2
of
4
  

### The value of a three-microRNA panel in serum for prostate cancer screening.

2. Int J Biol Markers. 2024 Mar;39(1):70-79. doi: 10.1177/03936155231213660 Epub 2023 Nov 13.
  
  
Lin S(1)(2), Sun C(1)(3), Li R(1)(3), Lu C(1)(3), Li X(1)(2), Wen Z(1)(2), Ge Z(1)(2), Chen W(1)(2), ...
  
  
Author information: (1)Department of Urology, Guangdong and Shenzhen Key Laboratory of Reproductive ...
  
  
BACKGROUND: Globally, prostate cancer is the second most common malignancy in males. Serum microRNAs (miRNAs) may function as non-invasive and innovative biomarkers for various cancers. Our study aimed to determine potential miRNAs for prostate cancer screening. METHODS: A three-stage study was accomplished to ascertain crucial miRNAs as markers. In the screening stage, we searched PubMed for aberrantly expressed miRNAs relevant to prostate cancer and selected them as candidate miRNAs. In training and validation stages, with serum specimens from 112 prostate cancer patients and 112 healthy controls, expressions of candidate miRNAs were identified through quantitative reverse transcription-polymerase chain reaction. The diagnostic capabilities of miRNAs were determined by receiver operating characteristic curves. Bioinformatic analysis was utilized to explore the function of the critical miRNAs. RESULTS: Expression of six serum miRNAs (miR-34b-3p, miR-556-5p, miR-200c-3p, miR-361-5p, miR-369-3p, miR-485-3p) were significantly altered in prostate cancer patients contrasted with healthy controls. The optimal combination of critical miRNAs is a three-miRNA panel (miR-34b-3p, miR-200c-3p, and miR-361-5p) with good diagnostic capability. FLRT2, KIAA1755, LDB3, and NTRK3 were identified as the potential genes targeted by the three-miRNA panel. CONCLUSIONS: The three-miRNA panel may perform as an innovative and promising serum marker for prostate cancer screening.
  
  
  
  
**KIAA1755**
-
3
of
4
  

### High-throughput assessment of the antibody profile in ovarian cancer ascitic fluids.

3. Oncoimmunology. 2019 Jun 4;8(9):e1614856. doi: 10.1080/2162402X.2019.1614856 eCollection 2019.
  
  
Antony F(1), Deantonio C(1), Cotella D(1), Soluri MF(1), Tarasiuk O(1), Raspagliesi F(2), Adorni F(3 ...
  
  
Author information: (1)Department of Health Sciences, and Interdisciplinary Research Center of Autoi ...
  
  
The identification of effective biomarkers for early diagnosis, prognosis, and response to treatments remains a challenge in ovarian cancer (OC) research. Here, we present an unbiased high-throughput approach to profile ascitic fluid autoantibodies in order to obtain a tumor-specific antigen signature in OC. We first reported the reactivity of immunoglobulins (Igs) purified from OC patient ascites towards two different OC cell lines. Using a discovery set of Igs, we selected tumor-specific antigens from a phage display cDNA library. After biopanning, 700 proteins were expressed as fusion protein and used in protein array to enable large-scale immunoscreening with independent sets of cancer and noncancerous control. Finally, the selected antigens were validated by ELISA. The initial screening identified eight antigenic clones: CREB3, MRPL46, EXOSC10, BCOR, HMGN2, HIP1R, OLFM4, and KIAA1755. These antigens were all validated by ELISA in a study involving ascitic Igs from 153 patients (69 with OC, 34 with other cancers and 50 without cancer), with CREB3 showing the highest sensitivity (86.95%) and specificity (98%). Notably, we were able to identify an association between the tumor-associated (TA) antibody response and the response to a first-line tumor treatment (platinum-based chemotherapy). A stronger association was found by combining three antigens (BCOR, CREB3, and MRLP46) as a single antibody signature. Measurement of an ascitic fluid antibody response to multiple TA antigens may aid in the identification of new prognostic signatures in OC patients and shift attention to new potentially relevant targets.
  
  
  
  
**KIAA1755**
-
4
of
4
  

### lncRNA profiling in early-stage chronic lymphocytic leukemia identifies transcriptional fingerprints with relevance in clinical outcome.

4. Blood Cancer J. 2016 Sep 9;6(9):e468. doi: 10.1038/bcj.2016.77
  
  
Ronchetti D(1), Manzoni M(1), Agnelli L(1), Vinci C(1), Fabris S(2), Cutrona G(3), Matis S(3), Colom ...
  
  
Author information: (1)Department of Oncology and Hemato-Oncology, University of Milan, Milan, Italy ...
  
  
Long non-coding RNAs (lncRNAs) represent a novel class of functional RNA molecules with an important emerging role in cancer. To elucidate their potential pathogenetic role in chronic lymphocytic leukemia (CLL), a biologically and clinically heterogeneous neoplasia, we investigated lncRNAs expression in a prospective series of 217 early-stage Binet A CLL patients and 26 different subpopulations of normal B-cells, through a custom annotation pipeline of microarray data. Our study identified a 24-lncRNA-signature specifically deregulated in CLL compared with the normal B-cell counterpart. Importantly, this classifier was validated on an independent data set of CLL samples. Belonging to the lncRNA signature characterizing distinct molecular CLL subgroups, we identified lncRNAs recurrently associated with adverse prognostic markers, such as unmutated IGHV status, CD38 expression, 11q and 17p deletions, and NOTCH1 mutations. In addition, correlation analyses predicted a putative lncRNAs interplay with genes and miRNAs expression. Finally, we generated a 2-lncRNA independent risk model, based on lnc-IRF2-3 and lnc-KIAA1755-4 expression, able to distinguish three different prognostic groups in our series of early-stage patients. Overall, our study provides an important resource for future studies on the functions of lncRNAs in CLL, and contributes to the discovery of novel molecular markers with clinical relevance associated with the disease.
  
  
  
  
  

---

  

# LINC01934

**Signature gene in:** CD4+ T cells, Tregs
  
**Differentially expressed in:** Tregs
  
  
**LINC01934**
-
1
of
1
  

### Identification and Verification of Immune Subtype-Related lncRNAs in Clear Cell Renal Cell Carcinoma.

1. Front Oncol. 2022 Jun 2;12:888502. doi: 10.3389/fonc.2022.888502 eCollection 2022.
  
  
Wang Z(1), Chen Z(2), Guo T(3), Hou M(4), Wang J(1), Guo Y(5), Du T(1), Zhang X(1), Wang N(1), Ding ...
  
  
Author information: (1)Department of Urology, Henan Provincial People's Hospital, Zhengzhou Universi ...
  
  
BACKGROUND: According to clinical study results, immune checkpoint blockade (ICB) treatment enhances the survival outcome of patients with clear cell renal cell carcinoma (ccRCC). Previous research has divided ccRCC patients into immune subtypes with distinct ICB response rates. However, the study on the association between lncRNAs and ccRCC immune subtypes is lacking. METHODS: Differentially expressed lncRNAs/mRNAs between two major immune subgroups were calculated. A weighted gene co-expression network analysis (WGCNA) was conducted to establish the lncRNA-mRNA co-expression network and select the key lncRNAs. Then, prognostic lncRNAs were selected from the network by the bioinformatics method. Next, the risk-score was estimated by lncRNA expression and their coefficients. Finally, a nomogram based on lncRNAs and clinical parameters was created to predict the prognosis of ccRCC. RESULTS: LncRNAs and mRNAs associated with ccRCC immune subtypes were identified. The lncRNAs and mRNAs from a gene module closely linked to the immune subtype were used to construct a network. The KEGG pathways enriched in the network were related to immune system activation processes. These 8 lncRNAs (AL365361.1, LINC01934, AC090152.1, PCED1B-AS1, LINC00426, AC007728.2, AC243829.4, and LINC00158) were found to be positively correlated with immune cells of the tumor microenvironment. The C-index of the nomogram was 0.777, and the calibration curve data suggests that the nomogram has a high degree of discriminating capacity. CONCLUSION: In summary, we discovered core lncRNAs linked with immune subtypes and created corresponding lncRNA-mRNA networks. These lncRNAs are anticipated to have predictive significance for ccRCC and may provide insight into novel biomarkers for the disease.
  
  
  
  
  

---

  

# MEG8

**Signature gene in:** Stromal cells
  
**Differentially expressed in:** Stromal cells
  
  
**MEG8**
-
1
of
10
  

### MEG8 as an antagonistic pleiotropic mechanism in breast cancer.

1. Cell Death Discov. 2024 Dec 20;10(1):509. doi: 10.1038/s41420-024-02272-0
  
  
Verdugo-Sivianes EM(1)(2)(3), Espinosa-Sánchez A(1)(2), Cases I(4), Rojas AM(4), Otero-Albiol D(1)(2 ...
  
  
Author information: (1)Instituto de Biomedicina de Sevilla (IBIS), Hospital Universitario Virgen del ...
  
  
Cellular senescence connects aging and cancer. Cellular senescence is a common program activated by cells in response to various types of stress. During this process, cells lose their proliferative capacity and undergo distinct morphological and metabolic changes. Senescence itself constitutes a tumor suppression mechanism and plays a significant role in organismal aging by promoting chronic inflammation. Additionally, age is one of the major risk factors for developing breast cancer. Therefore, while senescence can suppress tumor development early in life, it can also lead to an aging process that drives the development of age-related pathologies, suggesting an antagonistic pleiotropic effect. In this work, we identified Rian/MEG8 as a potential biomarker connecting aging and breast cancer for the first time. We found that Rian/MEG8 expression decreases with age; however, it is high in mice that age prematurely. We also observed decreased MEG8 expression in breast tumors compared to normal tissue. Furthermore, MEG8 overexpression reduced the proliferative and stemness properties of breast cancer cells both in vitro and in vivo by activating apoptosis. MEG8 could exemplify the antagonistic pleiotropic theory, where senescence is beneficial early in life as a tumor suppression mechanism due to increased MEG8, resulting in fewer breast tumors at an early age. Conversely, this effect could be detrimental later in life due to aging and cancer, when MEG8 is reduced and loses its tumor-suppressive role.
  
  
  
  
**MEG8**
-
2
of
10
  

### Identifying key genes and functionally enriched pathways in acute myeloid leukemia by weighted gene co-expression network analysis.

2. J Appl Genet. 2025 May;66(2):347-362. doi: 10.1007/s13353-024-00881-0 Epub 2024 Jul 9.
  
  
Jian J(1)(2), Yuan C(1), Hao H(3).
  
  
Author information: (1)Qilu Hospital of Shandong University, Qingdao, 266035, Shandong, China. (2)Qi ...
  
  
Acute myeloid leukemia (AML) is characterized by the uncontrolled proliferation of myeloid leukemia cells in the bone marrow and other hematopoietic tissues and is highly heterogeneous. While with the progress of sequencing technology, understanding of the AML-related biomarkers is still incomplete. The purpose of this study is to identify potential biomarkers for prognosis of AML. Based on WGCNA analysis of gene mutation expression, methylation level distribution, mRNA expression, and AML-related genes in public databases were employed for investigating potential biomarkers for the prognosis of AML. This study screened a total of 6153 genes by analyzing various changes in 103 acute myeloid leukemia (AML) samples, including gene mutation expression, methylation level distribution, mRNA expression, and AML-related genes in public databases. Moreover, seven AML-related co-expression modules were mined by WGCNA analysis, and twelve biomarkers associated with the AML prognosis were identified from each top 10 genes of the seven co-expression modules. The AML samples were then classified into two subgroups, the prognosis of which is significantly different, based on the expression of these twelve genes. The differentially expressed 7 genes of two subgroups (HOXB-AS3, HOXB3, SLC9C2, CPNE8, MEG8, S1PR5, MIR196B) are mainly involved in glucose metabolism, glutathione biosynthesis, small G protein-mediated signal transduction, and the Rap1 signaling pathway. With the utilization of WGCNA mining, seven gene co-expression modules were identified from the TCGA database, and there are unreported genes that may be potential driver genes of AML and may be the direction to identify the possible molecular signatures to predict survival of AML patients and help guide experiments for potential clinical drug targets.
  
  
  
  
**MEG8**
-
3
of
10
  

### Differential effect of the duration of exposure on the carcinogenicity of cadmium in MCF10A mammary epithelial cells.

3. Food Chem Toxicol. 2024 Apr;186:114523. doi: 10.1016/j.fct.2024.114523 Epub 2024 Feb 19.
  
  
Zimta AA(1), Cenariu D(2), Tigu AB(2), Moldovan C(2), Jurj A(3), Pirlog R(3), Pop C(4), Gurzau ES(5) ...
  
  
Author information: (1)MedFuture-Research Center for Advanced Medicine, "Iuliu Haţieganu" University ...
  
  
The carcinogenic role of cadmium (Cd2+) in breast cancer is still debatable. Current data points to duration of exposure as the most important element. In our study, we designed an in vitro model to investigate the effects of 3 weeks versus 6 weeks of low-level CdCl2 exposure on MCF10A cells. Our results demonstrated that after 3 weeks of CdCl2 exposure the cells displayed significant changes in the DNA integrity, but there was no development of malignant features. Interestingly, after 6 weeks of exposure, the cells significantly increased their invasion, migration and colony formation capacities. Additionally, MCF10A cells exposed for 6 weeks to CdCl2 had many dysregulated genes (4905 up-regulated and 4262 down-regulated). As follows, Cd-induced phenotypical changes are accompanied by a profound modification of the transcriptomic landscape. Furthermore, the molecular alterations driving carcinogenesis in MCF10A cells exposed to CdCl2 were found to be influenced by the duration of exposure, as in the case of MEG8. This long non-coding RNA was down-regulated at 3 weeks, but up-regulated at 6 weeks of exposure. In conclusion, even very low levels of Cd (0.5 μM) can have significant carcinogenic effects on breast cells in the case of subchronic exposure.
  
  
  
  
**MEG8**
-
4
of
10
  

### Long Non-Coding RNAs: Bridging Cancer-Associated Thrombosis and Clinical Outcome of Ovarian Cancer Patients.

4. Int J Mol Sci. 2023 Dec 21;25(1):140. doi: 10.3390/ijms25010140
  
  
Marques IS(1)(2), Tavares V(1)(3)(4), Savva-Bordalo J(5), Rei M(6), Liz-Pimenta J(3)(7), de Melo IG( ...
  
  
Author information: (1)Molecular Oncology and Viral Pathology Group, Research Center of IPO Porto (C ...
  
  
Ovarian cancer (OC) and venous thromboembolism (VTE) have a close relationship, in which tumour cells surpass the haemostatic system to drive cancer progression. Long non-coding RNAs (lncRNAs) have been implicated in VTE pathogenesis, yet their roles in cancer-associated thrombosis (CAT) and their prognostic value are unexplored. Understanding how these lncRNAs influence venous thrombogenesis and ovarian tumorigenesis may lead to the identification of valuable biomarkers for VTE and OC management. Thus, this study evaluated the impact of five lncRNAs, namely MALAT1, TUG1, NEAT1, XIST and MEG8, on a cohort of 40 OC patients. Patients who developed VTE after OC diagnosis had worse overall survival compared to their counterparts (log-rank test, p = 0.028). Elevated pre-chemotherapy MEG8 levels in peripheral blood cells (PBCs) predicted VTE after OC diagnosis (Mann-Whitney U test, p = 0.037; Χ2 test, p = 0.033). In opposition, its low levels were linked to a higher risk of OC progression (adjusted hazard ratio (aHR) = 3.00; p = 0.039). Furthermore, low pre-chemotherapy NEAT1 levels in PBCs were associated with a higher risk of death (aHR = 6.25; p = 0.008). As for the remaining lncRNAs, no significant association with VTE incidence, OC progression or related mortality was observed. Future investigation with external validation in larger cohorts is needed to dissect the implications of the evaluated lncRNAs in OC patients.
  
  
  
  
**MEG8**
-
5
of
10
  

### Dysregulation of non-coding RNAs in Wilms tumor.

5. Pathol Res Pract. 2023 Jun;246:154523. doi: 10.1016/j.prp.2023.154523 Epub 2023 May 8.
  
  
Taheri M(1), Hussen BM(2), Abdullah SR(3), Ghafouri-Fard S(4), Jamali E(5), Shojaei S(6).
  
  
Author information: (1)Institute of Human Genetics, Jena University Hospital, Jena, Germany; Urology ...
  
  
Wilms tumor (WT) as the most frequent pediatric tumor of kidney has been shown to be associated with dysregulation of non-coding RNAs. miR-200c, miR-155-5p, miR-1180, miR-22-3p, miR-483-5p, miR-140-5p, miR-92a-3p, miR-483-3p, miR-572, miR-539 and miR-613 are among dysregulated miRNAs in this tumor. Moreover, a number of long non-coding RNAs such as CRNDE, XIST, SNHG6, MEG3, LINC00667, MEG8, DLGAP1-AS2 and SOX21-AS1 have been shown to be dysregulated in WT. Finally, distinct studies have reported down-regulation of circCDYL and up-regulation of circ0093740 and circSLC7A6 in this tumor. Dysregulation of these transcripts represents a new avenue for identification of the pathetiology of this pediatric tumor as well as design of targeted therapies.
  
  
  
  
**MEG8**
-
6
of
10
  

### MicroRNA‑203a‑3p suppresses endothelial cell proliferation and invasion, and promotes apoptosis in hemangioma by inactivating the VEGF‑mediated PI3K/AKT pathway.

6. Exp Ther Med. 2022 Sep 1;24(5):644. doi: 10.3892/etm.2022.11581 eCollection 2022 Nov.
  
  
Hu Z(1), Zhuo L(2), Li Y(3), Duan D(4), Guo J(5).
  
  
Author information: (1)Department of General Surgery II (Department of Plastic Surgery), Affiliated ...
  
  
Our previous study demonstrated that microRNA-203a-3p (miR-203a-3p) was involved in the regulation of long non-coding RNA MEG8-mediated the progression of hemangioma, which is a benign tumor characterized by endothelial hyperplasia in the blood vessels and primarily occurring in infants and females. Therefore, the present study aimed to further investigate the effects of miR-203a-3p on endothelial cell proliferation, invasion and apoptosis, as well as its underlying mechanism in hemangioma. Human hemangioma endothelial cells (HemECs) were first transfected with either miR-203a-3p mimics or a miR-203a-3p inhibitor. Subsequently, vascular endothelial growth factor A (VEGFA) was overexpressed in these cells. Cell proliferation (by Cell Counting Kit-8 assay), apoptosis (by TUNEL assay), invasion (by Transwell assay) and PI3K/AKT signaling (by western blot) were assessed following transfection of these cells. Notably, transfection with miR-203a-3p mimics caused a reduction in cell proliferation, invasion and in the phosphorylation levels of PI3K and AKT, and promoted cell apoptosis in HemECs. By contrast, transfection with the miR-203a-3p inhibitor exerted the opposite effects compared with those of the miR-203a-3p mimics. miR-203a-3p was revealed to directly suppress VEGFA expression in HemECs. VEGFA overexpression alone increased cell proliferation and invasion, but decreased apoptosis. Furthermore, VEGFA co-transfection reversed the effects mediated by miR-203a-3p mimics transfection in HemECs. Mechanistically, miR-203a-3p was demonstrated to inactivate the PI3K/AKT pathway, whereas VEGFA overexpression produced the opposite effect. VEGFA co-transfection also attenuated the miR-203a-3p mimics-induced inactivation of PI3K/AKT signaling in HemECs. In conclusion, these data suggested that miR-203a-3p may inhibit endothelial cell proliferation and invasion, and promote apoptosis by inactivating VEGFA and PI3K/AKT signaling in hemangioma. These findings also implicated miR-203 as a possible treatment option for this disease.
  
  
  
  
**MEG8**
-
7
of
10
  

### Comprehensive bioinformatic analyses of lncRNA-mediated ceRNA network for uterine corpus endometrial carcinoma.

7. Transl Cancer Res. 2022 Jul;11(7):1994-2012. doi: 10.21037/tcr-22-249
  
  
Cai Y(#)(1), Cui J(#)(1), Wang Z(1), Wu H(2).
  
  
Author information: (1)Medical School of Nantong University, Nantong, China. (2)Department of Medica ...
  
  
BACKGROUND: Given that long non-coding RNAs (lncRNAs) involved in the tumor initiation or progression of the endometrium and that competing endogenous RNA (ceRNA) plays an important role in increasingly more biological processes, lncRNA-mediated ceRNA is likely to function in the pathogenesis of uterine corpus endometrial carcinoma (UCEC). Our present study aimed to explore the potential molecular mechanisms for the prognosis of UCEC through a lncRNA-mediated ceRNA network. METHODS: The transcriptome profiles and corresponding clinical profiles of UCEC dataset were retrieved from Clinical Proteomic Tumor Analysis Consortium (CPTAC) and The Cancer Genome Atlas (TCGA) databases respectively. Differentially expressed genes (DEGs) in UCEC samples were identified via "Edge R" package. Then, an integrated bioinformatics analysis including functional enrichment analysis, tumor infiltrating immune cell (TIIC) analysis, Kaplan-Meier curve, Cox regression analysis were conducted to analyze the prognostic biomarkers. RESULTS: In the CPTAC dataset of UCEC, a ceRNA network comprised of 36 miRNAs, 123 lncRNAs and 124 targeted mRNAs was established, and 8 of 123 prognostic-related Differentially Expressed long noncoding RNAs (DElncRNAs) were identified. While in the TCGA dataset, a ceRNA network comprised of 38 miRNAs, 83 lncRNAs and 110 targeted mRNAs was established, and 2 of 83 prognostic-related DElncRNAs were identified. After filtered by risk grouping and Cox regression analysis, 10 prognostic-related lncRNAs including LINC00443, LINC00483, C2orf48, TRBV11-2, MEG-8 were identified. In addition, 33 survival-related Differentially Expressed messenger RNA (DEmRNAs) in two ceRNA networks were further validated in the Human Protein Atlas Portal (HPA) database. Finally, six lncRNA/miRNA/mRNA axes were established to elucidate prognostic regulatory roles in UCEC. CONCLUSIONS: Several prognostic lncRNAs are identified and prognostic model of lncRNA-mediated ceRNA network is constructed, which promotes the understanding of UCEC development mechanisms and potential therapeutic targets.
  
  
  
  
**MEG8**
-
8
of
10
  

### MEG8: An Indispensable Long Non-coding RNA in Multiple Cancers.

8. Curr Pharm Des. 2022;28(20):1688-1694. doi: 10.2174/1381612828666220516090245
  
  
Du Z(1)(2), Tan F(1)(2), Chen J(1)(2), Wang B(1)(2), Liu Y(1)(2), Zhao F(1)(2), Wu Y(1)(2), Yuan C(1 ...
  
  
Author information: (1)Third-grade Pharmacological Laboratory on Traditional Chinese Medicine, State ...
  
  
BACKGROUND: As a member of long non-coding RNAs (lncRNAs), maternally expressed gene 8 (MEG8) has been found involving in the progression of a variety of cancers and playing a regulatory role. Therefore, MEG8 may turn into a new therapeutic target for cancer in the future. The purpose of this review is to illustrate the molecular mechanism and physiological function of MEG8 in various cancers. METHODS: We retrieved and analyzed related articles about MEG8, lncRNAs, and cancers, and then summarize the pathophysiological mechanisms of MEG8 in cancer development. RESULTS: LncRNA MEG8 participates in various cancers progression, thus influencing the proliferation, migration, and invasion of cancers. However, the expression of MEG8 is abnormally upregulated in non-small cell lung cancer (NSCLC), pancreatic cancer (PC), liver cancer (HCC), pituitary adenoma (PA) and hemangioma (HA), and inhibited in colorectal cancer (CRC), ovarian cancer (OC) and giant cell tumor (GCT), suggesting its clinical value in cancer therapy. CONCLUSION: LncRNA MEG8 is expected to be a new therapeutic target or biomarker for a wide range of cancers in the future.
  
  
  
  
**MEG8**
-
9
of
10
  

### Long non-coding RNA MEG8 induced by PLAG1 promotes clear cell renal cell carcinoma through the miR-495-3p/G3BP1 axis.

9. Pathol Res Pract. 2022 Jan;229:153734. doi: 10.1016/j.prp.2021.153734 Epub 2021 Dec 5.
  
  
Shan G(1), Huang T(2), Tang T(3).
  
  
Author information: (1)Department of Urology, Renmin Hospital of Wuhan University, Wuhan, Hubei, Chi ...
  
  
Clear cell renal cell carcinoma (ccRCC) is recognized as one of the most lethal malignancies among the urological system, with constantly increasing mortality. While the molecular mechanisms underlying ccRCC progression are still poorly understood, the molecular and functional role of lncRNA in multiple diseases has been well demonstrated. In this study, we hypothesized that lncRNA MEG8 might participate in ccRCC development. At first, we found that MEG8 expression was increased in ccRCC tumor tissues and cells. Next, we demonstrated that MEG8 knockdown suppressed cell viability, migration, and invasion in vitro and inhibited tumor growth in vivo. Subsequently, we utilized bioinformatics analysis, ChIP, and luciferase assays, and we found that PLAG1 could transcriptionally regulate MEG8 in ccRCC cells. Furthermore, MEG8 promoted G3BP1 expression to aggravate ccRCC tumorigenic properties through sponging miR-495-3p. Our study identified a novel PLAG1/MEG8/miR-495-3p/G3BP1 network in ccRCC development, which might be a promising direction for developing new diagnoses or therapeutic agents for ccRCC.
  
  
  
  
**MEG8**
-
10
of
10
  

### Identifying and characterizing lincRNA genomic clusters reveals its cooperative functions in human cancer.

10. J Transl Med. 2021 Dec 14;19(1):509. doi: 10.1186/s12967-021-03179-5
  
  
Zhou H(1), Gao Y(1), Li X(1), Shang S(1), Wang P(1), Zhi H(1), Guo S(1), Sun D(1), Liu H(1), Li X(2) ...
  
  
Author information: (1)College of Bioinformatics Science and Technology, Harbin Medical University, ...
  
  
BACKGROUND: Emerging evidence has revealed that some long intergenic non-coding RNAs (lincRNAs) are likely to form clusters on the same chromosome, and lincRNA genomic clusters might play critical roles in the pathophysiological mechanism. However, the comprehensive investigation of lincRNA clustering is rarely studied, particularly the characterization of their functional significance across different cancer types. METHODS: In this study, we firstly constructed a computational method basing a sliding window approach for systematically identifying lincRNA genomic clusters. We then dissected these lincRNA genomic clusters to identify common characteristics in cooperative expression, conservation among divergent species, targeted miRNAs, and CNV frequency. Next, we performed comprehensive analyses in differentially-expressed patterns and overall survival outcomes for patients from The Cancer Genome Atlas (TCGA) and The Genotype-Tissue Expression (GTEx) across multiple cancer types. Finally, we explored the underlying mechanisms of lincRNA genomic clusters by functional enrichment analysis, pathway analysis, and drug-target interaction. RESULTS: We identified lincRNA genomic clusters according to the algorithm. Clustering lincRNAs tended to be co-expressed, highly conserved, targeted by more miRNAs, and with similar deletion and duplication frequency, suggesting that lincRNA genomic clusters may exert their effects by acting in combination. We further systematically explored conserved and cancer-specific lincRNA genomic clusters, indicating they were involved in some important mechanisms of disease occurrence through diverse approaches. Furthermore, lincRNA genomic clusters can serve as biomarkers with potential clinical significance and involve in specific pathological processes in the development of cancer. Moreover, a lincRNA genomic cluster named Cluster127 in DLK1-DIO3 imprinted locus was discovered, which contained MEG3, MEG8, MEG9, MIR381HG, LINC02285, AL132709.5, and AL132709.1. Further analysis indicated that Cluster127 may have the potential for predicting prognosis in cancer and could play their roles by participating in the regulation of PI3K-AKT signaling pathway. CONCLUSIONS: Clarification of the lincRNA genomic clusters specific roles in human cancers could be beneficial for understanding the molecular pathogenesis of different cancer types.
  
  
  
  
  

---

  

# NHSL1

**Signature gene in:** MDM
  
**Differentially expressed in:** MDM
  
  
**NHSL1**
-
1
of
7
  

### PPP2R1A regulates migration persistence through the NHSL1-containing WAVE Shell Complex.

1. Nat Commun. 2023 Jun 15;14(1):3541. doi: 10.1038/s41467-023-39276-w
  
  
Wang Y(1), Chiappetta G(#)(2), Guérois R(#)(3), Liu Y(#)(4), Romero S(#)(1), Boesch DJ(4), Krause M( ...
  
  
Author information: (1)Laboratory of Structural Biology of the Cell (BIOC), CNRS UMR7654, École Poly ...
  
  
The RAC1-WAVE-Arp2/3 signaling pathway generates branched actin networks that power lamellipodium protrusion of migrating cells. Feedback is thought to control protrusion lifetime and migration persistence, but its molecular circuitry remains elusive. Here, we identify PPP2R1A by proteomics as a protein differentially associated with the WAVE complex subunit ABI1 when RAC1 is activated and downstream generation of branched actin is blocked. PPP2R1A is found to associate at the lamellipodial edge with an alternative form of WAVE complex, the WAVE Shell Complex, that contains NHSL1 instead of the Arp2/3 activating subunit WAVE, as in the canonical WAVE Regulatory Complex. PPP2R1A is required for persistence in random and directed migration assays and for RAC1-dependent actin polymerization in cell extracts. PPP2R1A requirement is abolished by NHSL1 depletion. PPP2R1A mutations found in tumors impair WAVE Shell Complex binding and migration regulation, suggesting that the coupling of PPP2R1A to the WAVE Shell Complex is essential to its function.
  
  
  
  
**NHSL1**
-
2
of
7
  

### Nance-Horan Syndrome-like 1 protein negatively regulates Scar/WAVE-Arp2/3 activity and inhibits lamellipodia stability and cell migration.

2. Nat Commun. 2021 Sep 28;12(1):5687. doi: 10.1038/s41467-021-25916-6
  
  
Law AL(1)(2), Jalal S(#)(1), Pallett T(#)(1), Mosis F(1), Guni A(1), Brayford S(3), Yolland L(3), Ma ...
  
  
Author information: (1)Krause Group, Randall Centre for Cell and Molecular Biophysics, King's Colleg ...
  
  
Cell migration is important for development and its aberrant regulation contributes to many diseases. The Scar/WAVE complex is essential for Arp2/3 mediated lamellipodia formation during mesenchymal cell migration and several coinciding signals activate it. However, so far, no direct negative regulators are known. Here we identify Nance-Horan Syndrome-like 1 protein (NHSL1) as a direct binding partner of the Scar/WAVE complex, which co-localise at protruding lamellipodia. This interaction is mediated by the Abi SH3 domain and two binding sites in NHSL1. Furthermore, active Rac binds to NHSL1 at two regions that mediate leading edge targeting of NHSL1. Surprisingly, NHSL1 inhibits cell migration through its interaction with the Scar/WAVE complex. Mechanistically, NHSL1 may reduce cell migration efficiency by impeding Arp2/3 activity, as measured in cells using a Arp2/3 FRET-FLIM biosensor, resulting in reduced F-actin density of lamellipodia, and consequently impairing the stability of lamellipodia protrusions.
  
  
  
  
**NHSL1**
-
3
of
7
  

### Integrative genomic analysis of salivary duct carcinoma.

3. Sci Rep. 2020 Sep 14;10(1):14995. doi: 10.1038/s41598-020-72096-2
  
  
Kim Y(1)(2)(3), Song S(4), Lee M(5), Swatloski T(6), Kang JH(1)(2), Ko YH(7), Park WY(2), Jeong HS(8 ...
  
  
Author information: (1)Department of Health Science and Technology, Samsung Advanced Institute for H ...
  
  
Salivary duct carcinoma (SDC) is one of the most aggressive subtypes of salivary gland cancers. Conventional chemotherapy and/or radiation have shown only limited clinical efficacy in the treatment of recurrent or metastatic SDC. Currently, clinically approved targeted-therapeutics are not generally applicable except in very limited cases, and there exists a strong need for the development of treatment against this unique tumor type. To further interrogate genomic features of SDC, we have conducted multi-omic profiling of the SDC to describe the genomic alterations prevalent in this disease. Whole-genome sequencing, whole exome-sequencing and transcriptome sequencing were performed on a discovery cohort of 10 SDC samples. Targeted genomic profiling was performed in additional 32 SDC samples to support the findings obtained from the original discovery cohort. The cancer cohort was characterized by an average mutation burden of 85 somatic exonic mutations per tumor sample. The cohort harbored a mutational signature of BRCA and APOBEC/AID. Several genes, including TP53, RB1, SMAD4, HRAS, APC, PIK3CA and GNAQ were recurrently somatically altered in SDC. A novel fusion gene, generated by genomic rearrangement, MYB-NHSL1, was also noted. Our findings represent a significant layer in the systematic understanding of potentially clinically useful genomic and molecular targets for a subset of recurrent/metastatic SDC.
  
  
  
  
**NHSL1**
-
4
of
7
  

### Analysis of microarray-identified genes and microRNAs associated with drug resistance in ovarian cancer.

4. Int J Clin Exp Pathol. 2015 Jun 1;8(6):6847-58. eCollection 2015.
  
  
Zou J(1), Yin F(2), Wang Q(1), Zhang W(1), Li L(1).
  
  
Author information: (1)Department of Gynecologic Oncology, Affiliated Tumor Hospital of Guangxi Medi ...
  
  
The aim of this study was to identify potential microRNAs and genes associated with drug resistance in ovarian cancer through web-available microarrays. The drug resistant-related microRNA microarray dataset GS54665 and mRNA dataset GSE33482, GSE28646, and GSE15372 were downloaded from the Gene Expression Omnibus database. Dysregulated microRNAs/genes were screened with GEO2R and were further identified in SKOV3 (SKOV3/DDP) and A2780 (A2780/DDP) cells by real-time quantitative PCR (qRT-PCR), and then their associations with drug resistance was analyzed by comprehensive bioinformatic analyses. Nine microRNAs (microRNA-199a-5p, microRNA-199a-3p, microRNA-199b-3p, microRNA-215, microRNA-335, microRNA-18b, microRNA-363, microRNA-645 and microRNA-141) and 38 genes were identified to be differentially expressed in drug-resistant ovarian cancer cells, with seven genes (NHSL1, EPHA3, USP51, ZSCAN4, EPHA7, SNCA and PI15) exhibited exactly the same expression trends in all three microarrays. Biological process annotation and pathway enrichment analysis of the 9 microRNAs and 38 genes identified several drug resistant-related signaling pathways, and the microRNA-mRNA interaction revealed the existence of a targeted regulatory relationship between the 9 microRNAs and most of the 38 genes. The expression of 9 microRNAs and the 7 genes by qRT-PCR in SKOV3/DDP and A2780/DDP cells indicating a consistent expression profile with the microarrays. Among those, the expression of EPHA7 and PI15 were negatively correlated with that of microRNA-141, and they were also identified as potential targets of this microRNA via microRNA-mRNA interaction. We thus concluded that microRNA-141, EPHA7, and PI15 might jointly participate in the regulation of drug resistance in ovarian cancer and serve as potential targets in targeted therapies.
  
  
  
  
**NHSL1**
-
5
of
7
  

### Whole exome sequencing in a case of sporadic multiple meningioma reveals shared NF2, FAM109B, and TPRXL mutations, together with unique SMARCB1 alterations in a subset of tumor nodules.

5. Cancer Genet. 2015 Jun;208(6):327-32. doi: 10.1016/j.cancergen.2015.03.012 Epub 2015 Apr 11.
  
  
Torres-Martín M(1), Kusak ME(2), Isla A(3), Burbano RR(4), Pinto GR(5), Melendez B(6), Castresana JS ...
  
  
Author information: (1)Research Unit, La Paz University Hospital, IdiPAZ, Madrid, Spain. Electronic ...
  
  
Meningiomas are common intracranial tumors derived from arachnoid cells. Multiple meningiomas are occasionally present even in patients with no history of neurofibromatosis type 2, a condition that can cause the formation of this neoplasm. Previous studies have shown that most multiple meningiomas are monoclonal in origin. In this study, exome sequencing was performed on four meningiomas and the corresponding peripheral blood DNA from a 61-year-old woman with sporadic multiple meningioma. At least three common mutational events (at the NF2, FAM109B, and TPRXL genes) were detected in the tumors' DNA when they were compared with the lymphocyte DNA from the patient as control. Additionally, an array of unique mutations was detected in each tumor, including in SMARCB1 in two of the samples, a gene whose alteration leads to the development of meningioma. Mutations in other genes, such as IRS4, GULP1, NHSL1, and C10orf53, accounted for one alteration in each meningioma nodule. Our data suggest a monoclonal origin of the meningiomas in this patient, although the numerous alterations contained in each sample indicated multiple secondary variable changes in each tumor nodule. Whether the alterations described in this work are drivers of tumorigenesis or are simply passengers requires further study.
  
  
  
  
**NHSL1**
-
6
of
7
  

### An optimized predictor panel for colorectal cancer diagnosis based on the combination of tumor-associated antigens obtained from protein and phage microarrays.

6. J Proteomics. 2012 Aug 3;75(15):4647-55. doi: 10.1016/j.jprot.2012.03.004 Epub 2012 Mar 18.
  
  
Barderas R(1), Babel I, Díaz-Uriarte R, Moreno V, Suárez A, Bonilla F, Villar-Vázquez R, Capellá G, ...
  
  
Author information: (1)Functional Proteomics Laboratory, Centro de Investigaciones Biológicas (CIB-C ...
  
  
Humoral response in cancer patients appears early in cancer progression and can be used for diagnosis, including early detection. By using human recombinant protein and T7 phage microarrays displaying colorectal cancer (CRC)-specific peptides, we previously selected 6 phages and 6 human recombinant proteins as tumor-associated antigens (TAAs) with high diagnostic value. After completing validation in biological samples, TAAs were classified according to their correlation, redundancy in reactivity patterns and multiplex diagnostic capabilities. For predictor model optimization, TAAs were reanalyzed with a new set of samples. A combination of three phages displaying peptides homologous to GRN, NHSL1 and SREBF2 and four proteins PIM1, MAPKAPK3, FGFR4 and ACVR2B, achieved an area under the curve (AUC) of 94%, with a sensitivity of 89.1% and specificity of 90.0%, to correctly predict the presence of cancer. For early colorectal cancer stages, the AUC was 90%, with a sensitivity of 88.2% and specificity of 82.6%. In summary, we have defined an optimized predictor panel, combining TAAs from different sources, with highly improved accuracy and diagnostic value for colorectal cancer. This article is part of a Special Issue entitled: Translational Proteomics.
  
  
  
  
**NHSL1**
-
7
of
7
  

### Identification of MST1/STK4 and SULF1 proteins as autoantibody targets for the diagnosis of colorectal cancer by using phage microarrays.

7. Mol Cell Proteomics. 2011 Mar;10(3):M110.001784. doi: 10.1074/mcp.M110.001784 Epub 2011 Jan 12.
  
  
Babel I(1), Barderas R, Diaz-Uriarte R, Moreno V, Suarez A, Fernandez-Aceñero MJ, Salazar R, Capellá ...
  
  
Author information: (1)Functional Proteomics Laboratory, Centro de Investigaciones Biológicas (CIB-C ...
  
  
The characterization of the humoral response in cancer patients is becoming a practical alternative to improve early detection. We prepared phage microarrays containing colorectal cancer cDNA libraries to identify phage-expressed peptides recognized by tumor-specific autoantibodies from patient sera. From a total of 1536 printed phages, 128 gave statistically significant values to discriminate cancer patients from control samples. From this, 43 peptide sequences were unique following DNA sequencing. Six phages containing homologous sequences to STK4/MST1, SULF1, NHSL1, SREBF2, GRN, and GTF2I were selected to build up a predictor panel. A previous study with high-density protein microarrays had identified STK4/MST1 as a candidate biomarker. An independent collection of 153 serum samples (50 colorectal cancer sera and 103 reference samples, including healthy donors and sera from other related pathologies) was used as a validation set to study prediction capability. A combination of four phages and two recombinant proteins, corresponding to MST1 and SULF1, achieved an area under the curve of 0.86 to correctly discriminate cancer from healthy sera. Inclusion of sera from other different neoplasias did not change significantly this value. For early stages (A+B), the corrected area under the curve was 0.786. Moreover, we have demonstrated that MST1 and SULF1 proteins, homologous to phage-peptide sequences, can replace the original phages in the predictor panel, improving their diagnostic accuracy.
  
  
  
  
  

---

  

# PCSK5

**Signature gene in:** Stromal cells
  
**Differentially expressed in:** Stromal cells
  
  
**PCSK5**
-
1
of
10
  

### PCSK5 downregulation promotes the inhibitory effect of andrographolide on glioblastoma through regulating STAT3.

1. Mol Cell Biochem. 2025 Jan;480(1):521-533. doi: 10.1007/s11010-024-04977-3 Epub 2024 Mar 29.
  
  
Gong H(#)(1), Yang X(#)(1), An L(1), Zhang W(1), Liu X(1), Shu L(1), Yang L(2).
  
  
Author information: (1)Department of Immunology, Basic Medical College, Guizhou Medical University, ...
  
  
Proprotein convertase subtilisin/kexin type 5 (PCSK5) is a member of the proprotein convertase (PC) family, which processes immature proteins into functional proteins and plays an important role in the process of cell migration and transformation. Andrographolide is a non-peptide compound with PC inhibition and antitumor activity. Our research aimed to investigate the functional role of PCSK5 downregulation combined with Andro on GBM progression. Results from the cancer genome atlas (TCGA) and clinical samples revealed a significant upregulation of PCSK5 in GBM tissues than in non-tumor brain tissues. Higher expression of PCSK5 was correlated with advanced GBM stages and worse patient prognosis. PCSK5 knockdown attenuated the epithelial-mesenchymal transition (EMT)-like properties of GBM cells induced by IL-6. PCSK5 knockdown in combination with Andro treatment significantly inhibited the proliferation and invasion of GBM cells in vitro, as well as tumor growth in vivo. Mechanistically, PCSK5 downregulation reduced the expression of p-STAT3 and Matrix metalloproteinases (MMPs), which could be rescued by the p-STAT3 agonist. STAT3 silencing downregulated the expression of MMPs without affecting PCSK5. Furthermore, Andro in combination with PCSK5 silencing significantly inhibited STAT3/MMPs axis. These observations provided evidence that PCSK5 functioned as a potential tumor promoter by regulating p-STAT3/MMPs and the combination of Andro with PCSK5 silencing might be a good strategy to prevent GBM progression.
  
  
  
  
**PCSK5**
-
2
of
10
  

### Dysregulation of the Skin-Liver Axis in Prurigo Nodularis: An Integrated Genomic, Transcriptomic, and Population-Based Analysis.

2. Genes (Basel). 2024 Jan 23;15(2):146. doi: 10.3390/genes15020146
  
  
Marani M(1), Madan V(1), Le TK(1), Deng J(1), Lee KK(1), Ma EZ(1), Kwatra SG(1).
  
  
Author information: (1)Department of Dermatology, Johns Hopkins University School of Medicine, Balti ...
  
  
Pruritus has long been linked to hepatic dysfunction; however, there are limited data characterizing the association between liver disease and prurigo nodularis (PN), a chronic inflammatory skin disease featuring severe pruritis. We thus conducted a cross-sectional analysis of hepatic comorbidities in PN patients using TriNetX, a large global health research network. This analysis revealed that PN patients had a higher risk (p < 0.001) of developing liver cirrhosis, acute and subacute hepatic failure, inflammatory liver disease, chronic hepatitis, nonalcoholic steatohepatitis, portal hypertension, fatty liver, chronic passive congestion of the liver, and hepatocellular carcinoma compared with healthy controls. The cumulative incidence of liver disease was about three times higher in PN patients compared with healthy controls. These findings provided the basis for translational studies to investigate a genetic mechanism for this association. Cutaneous transcriptomic analysis performed on PN patients revealed the dysregulation of genes related to hepatic failure in lesional PN compared with both nonlesional PN and control skin. Similarly, gene set variation analysis (GSVA) revealed a significantly increased (p < 0.05) activation of liver metabolism, chronic hepatic failure, acute hepatic failure, cholestatic liver disease, polycystic liver disease, and hepatocellular carcinoma pathways in lesional PN compared with control skin. A subsequent genome-wide association study (GWAS) identified shared single-nucleotide polymorphisms (SNPs) in the genes AR, EDIL3, MACROD2, PCSK5, RUNX1T1, TENM4, and ZEB2 between PN and liver disease from the FinnGen cohort. Significant dysregulation of the skin-liver axis in PN patients may explain the increased incidence and severity of hepatic comorbidities and help identify future therapeutic targets for PN.
  
  
  
  
**PCSK5**
-
3
of
10
  

### Gene expression profiles to analyze the anticancer and carcinogenic effects of arsenic in bladder cancer.

3. Am J Transl Res. 2023 Oct 15;15(10):5984-5996. eCollection 2023.
  
  
Han X(1)(2)(3), Zhang T(3), Ma Q(3), Chang R(3), Xin S(3), Yu Q(4)(5), Zhang G(2), Wang Y(2)(4).
  
  
Author information: (1)Department of Oncology and Chemistry, The First Affiliated Hospital, Jinan Un ...
  
  
OBJECTIVES: Arsenic is one of the greatest hazards as an environmental carcinogen. At the same time it is also a promising anticancer agent, that can be used to treat acute promyelocytic leukemia (APL) and some other tumors. Arsenic trioxide (ATO) exerts its therapeutic effect by promoting degradation of an oncogenic protein that drives the growth of APL cells. However, the molecular mechanisms that govern these paradoxical effects of arsenic in bladder cancer remain unclear. We speculate that they share the common mechanism that arsenic binds to the target proteins and subsequently impacts the expression of downstream genes. METHODS: To address this issue, three Gene Set Enrichments (GSE) were loaded from the Gene Expression Omnibus (GEO) database with four expression matrices. Three of them were mice samples at exposure times of 1, 2, and 12 weeks, and the last was a human urothelial cell (HUC1) sample. Differentially expressed genes (DEGs) from 4 expression groups were identified at iDEP and analyzed at Metascape and Cytoscape for signaling pathway analysis and protein-protein interaction (PPI) analysis. The web-portals UALCAN and GEPIA were used to analyze the role of DEGs in the crosstalk between carcinogenic and anticancer effects. The putative downstream genes of arsenic binding proteins were retrieved using the Cistrome Data Browser. Real-time PCR was used to validate the expression of DEGs. RESULTS: The signaling pathways referred to lipid metabolism. Responses to various stimuli or hormones were overrepresented in 4 expression matrices. The PPI network emphasized the role of KRAS and TNF signaling in different groups. Furthermore, BDKRB2, FOS, NR4A1, PLAU, SH3BGRL, and F10 played an important role in the crosstalk between carcinogenic and anticancer effects in bladder cancer. Arsenic may impact the activity of ACTB, BACH1, NME2, RBBP4, PARP1, and PML by direct binding, and thus influence the expression of downstream genes such as PAX6, MLLT11, LTBP1, PCSK5, ZFP36, COL8A2, and IL1R2. CONCLUSION: Arsenic exerted carcinogenic and anticancer functions by altering the expression of crosstalk genes such as BDKRB2, FOS, NR4A1, PLAU, SH3BGRL, and F10, and these were due to arsenic binding proteins.
  
  
  
  
**PCSK5**
-
4
of
10
  

### Identifying Genetic Signatures Associated with Oncogene-Induced Replication Stress in Osteosarcoma and Screening for Potential Targeted Drugs.

4. Biochem Genet. 2024 Jun;62(3):1690-1715. doi: 10.1007/s10528-023-10497-4 Epub 2023 Sep 6.
  
  
Gao M(1)(2), Liu W(2), Li T(2), Song Z(1)(2), Wang X(#)(3), Zhang X(#)(4)(5).
  
  
Author information: (1)School of Medicine, Nankai University, Tianjin, China. (2)Department of Ortho ...
  
  
Osteosarcoma is the most common type of primary malignant bone tumor. Due to the lack of selectivity and sensitivity of chemotherapy drugs to tumor cells, coupled with the use of large doses, chemotherapy drugs often have systemic toxicity. The use of modern sequencing technology to screen tumor markers in a large number of tumor samples is a common method for screening highly specific and selective anti-tumor drugs. This study aims to identify potential biomarkers using the latest reported gene expression signatures of oncogene-induced replication stress (ORS) in aggressive cancers, and potential anti-osteosarcoma drugs were screened in different drug databases. In this study, we obtained 89 osteosarcoma-related samples in the TARGET database, all of which included survival information. According to the median expression of each of six reported ORS gene markers (NAT10/DDX27/ZNF48/C8ORF33/MOCS3/MPP6), we divided 89 osteosarcoma gene expression datasets into a high expression group and a low expression group and then performed a differentially expressed gene (DEG) analysis. The coexisting genes of 6 groups of DEGs were used as replication stress-related genes (RSGs) of osteosarcoma. Then, key RSGs were screened using LASSO regression, a Cox risk proportional regression prognostic model and a tenfold cross-validation test. GSE21257 datasets collected from the Gene Expression Omnibus (GEO) database were used to verify the prognostic model. The final key RSGs selected were used in the L1000PWD and DGIdb databases to mine potential drugs. After further validation by the prognostic model, we identified seven genes associated with ORS in osteosarcoma as key RSGs, including transcription factor 7 like 2 (TCF7L2), solute carrier family 27 member 4 (SLC27A4), proprotein convertase subtilisin/kexin type 5 (PCSK5), nucleolar protein 6 (NOL6), coiled-coil-coil-coil-coil-helix domain containing 4 (CHCHD4), eukaryotic translation initiation factor 3 subunit B (EIF3B), and synthesis of cytochrome C oxidase 1 (SCO1). Then, we screened the seven key RSGs in two drug databases and found six potential anti-osteosarcoma drugs (D GIdb database: repaglinide, tacrolimus, sirolimus, cyclosporine, and hydrochlorothiazide; L1000PWD database: the small molecule VU-0365117-1). Seven RSGs (TCF7L2, SLC27A4, PCSK5, NOL6, CHCHD4, EIF3B, and SCO1) may be associated with the ORS gene signatures in osteosarcoma. Repaglinide, tacrolimus, sirolimus, cyclosporine, hydrochlorothiazide and the small molecule VU-0365117-1 are potential therapeutic drugs for osteosarcoma.
  
  
  
  
**PCSK5**
-
5
of
10
  

### Inactivation of Notch4 Attenuated Pancreatic Tumorigenesis in Mice.

5. Cancer Res Commun. 2022 Dec 12;2(12):1601-1616. doi: 10.1158/2767-9764.CRC-22-0106 eCollection 2022 Dec.
  
  
Saeki K(#)(1)(2), Qiu W(#)(1)(2), Friedman RA(1)(3), Pan S(1)(4), Lu J(1)(5), Ichimiya S(1)(5), Chio ...
  
  
Author information: (1)Herbert Irving Comprehensive Cancer Center, Columbia University Irving Medica ...
  
  
Expression of the Notch family of receptors is often upregulated in pancreatic ductal adenocarcinoma (PDAC). In this study, we focused on Notch4, which had not been investigated in PDAC. We generated KC (LSL-KrasG12D;p48-Cre), N4 - / - KC (Notch4- / -;LSL-KrasG12D;p48-Cre), PKC (p16fl/fl;LSL-KrasG12D;p48-Cre), and N4 - / - PKC (Notch4-/ -; p16fl/f l;LSL-KrasG12D;p48-Cre) genetically engineered mouse models (GEMM). We performed caerulein treatment in both KC and N4 - / - KC mice, and the development of acinar-to-ductal metaplasia (ADM) and pancreatic intraepithelial neoplasia (PanIN) lesions were significantly diminished in the N4 - / - KC than in the KC GEMM (P = 0.01). This in vivo result was validated by in vitro ADM induction of the explant cultures of pancreatic acinar cells from the N4 - / - KC and KC mice (P < 0.001), confirming that Notch4 is an important contributor to early pancreatic tumorigenesis. To evaluate the role of Notch4 in the later stage of pancreatic tumorigenesis, we compared the PKC and N4 - / - PKC mice. The N4 - / - PKC mice had better overall survival (P = 0.012) and significantly reduced tumor burden (PanIN: P = 0.018 at 2 months, PDAC: P = 0.039 at 5 months) compared with the PKC GEMM. RNA-sequencing analysis of pancreatic tumor cell lines derived from the PKC and N4 - / - PKC GEMMs revealed that 408 genes were differentially expressed (FDR < 0.05) and PCSK5 is a potential downstream effector of the Notch4 signaling pathway (P < 0.001). Low expression of PCSK5 positively correlates with good survival in patients with PDAC (P = 0.028). We have identified a novel role for Notch4 signaling with tumor-promoting function in pancreatic tumorigenesis. Our study also uncovered a novel association between PCSK5 and Notch4 signaling in PDAC. SIGNIFICANCE: We demonstrated that global inactivation of Notch4 significantly improved the survival of an aggressive mouse model for PDAC and provided preclinical evidence that Notch4 and PCSK5 are novel targets for PDAC therapies.
  
  
  
  
**PCSK5**
-
6
of
10
  

### Coordinated reprogramming of renal cancer transcriptome, metabolome and secretome associates with immune tumor infiltration.

6. Cancer Cell Int. 2023 Jan 5;23(1):2. doi: 10.1186/s12935-022-02845-y
  
  
Poplawski P(1), Alseekh S(2)(3), Jankowska U(4), Skupien-Rabian B(4), Iwanicka-Nowicka R(5)(6), Koss ...
  
  
Author information: (1)Department of Biochemistry and Molecular Biology, Centre of Postgraduate Medi ...
  
  
BACKGROUND: Clear cell renal cell carcinoma (ccRCC) is the most common subtype of renal cancer. The molecules (proteins, metabolites) secreted by tumors affect their extracellular milieu to support cancer progression. If secreted in amounts detectable in plasma, these molecules can also serve as useful, minimal invasive biomarkers. The knowledge of ccRCC tumor microenvironment is fragmentary. In particular, the links between ccRCC transcriptome and the composition of extracellular milieu are weakly understood. In this study, we hypothesized that ccRCC transcriptome is reprogrammed to support alterations in tumor microenvironment. Therefore, we comprehensively analyzed ccRCC extracellular proteomes and metabolomes as well as transcriptomes of ccRCC cells to find molecules contributing to renal tumor microenvironment. METHODS: Proteomic and metabolomics analysis of conditioned media isolated from normal kidney cells as well as five ccRCC cell lines was performed using mass spectrometry, with the following ELISA validation. Transcriptomic analysis was done using microarray analysis and validated using real-time PCR. Independent transcriptomic and proteomic datasets of ccRCC tumors were used for the analysis of gene and protein expression as well as the level of the immune infiltration. RESULTS: Renal cancer secretome contained 85 proteins detectable in human plasma, consistently altered in all five tested ccRCC cell lines. The top upregulated extracellular proteins included SPARC, STC2, SERPINE1, TGFBI, while downregulated included transferrin and DPP7. The most affected extracellular metabolites were increased 4-hydroxy-proline, succinic acid, cysteine, lactic acid and downregulated glutamine. These changes were associated with altered expression of genes encoding the secreted proteins (SPARC, SERPINE1, STC2, DPP7), membrane transporters (SLC16A4, SLC6A20, ABCA12), and genes involved in protein trafficking and secretion (KIF20A, ANXA3, MIA2, PCSK5, SLC9A3R1, SYTL3, and WNTA7). Analogous expression changes were found in ccRCC tumors. The expression of SPARC predicted the infiltration of ccRCC tumors with endothelial cells. Analysis of the expression of the 85 secretome genes in > 12,000 tumors revealed that SPARC is a PanCancer indicator of cancer-associated fibroblasts' infiltration. CONCLUSIONS: Transcriptomic reprogramming of ccRCC supports the changes in an extracellular milieu which are associated with immune infiltration. The proteins identified in our study represent valuable cancer biomarkers detectable in plasma.
  
  
  
  
**PCSK5**
-
7
of
10
  

### A more novel and robust gene signature predicts outcome in patients with esophageal squamous cell carcinoma.

7. Clin Res Hepatol Gastroenterol. 2022 Dec;46(10):102033. doi: 10.1016/j.clinre.2022.102033 Epub 2022 Oct 17.
  
  
Ma C(1), Luo H(2).
  
  
Author information: (1)Department of Thoracic Surgery, The First Affiliated Hospital of Zhengzhou Un ...
  
  
BACKGROUND: Esophageal squamous cell carcinoma (ESCC) is a life-threatening thoracic tumor with a poor prognosis. The tumor microenvironment (TME) mainly comprises tumor cells and tumor-infiltrating immune cells mixed with stromal components. The latest research has displayed that tumor immune cell infiltration (ICI) is closely connected with the ESCC patients' clinical prognosis. This study was designed to construct a gene signature based on the ICI of ESCC to predict prognosis. METHODS: Based on the selection criteria we set, the eligible ESCC cases from the GSE53625 and TCGA-ESCA datasets were chosen for the training cohort and the validation cohort, respectively. Unsupervised clustering detailed grouped ESCC cases of the training cohort based on the ICI profile. We determined the differential expression genes (DEGs) between the ICI clusters, and, subsequently, we adopted the univariate Cox analysis to recognize DEGs with prognostic potential. These screened DEGs underwent a Lasso regression, which then generated a gene signature. The harvested signature's predictive ability was further examined by the Kaplan-Meier analysis, Cox analysis, ROC, IAUC, and IBS. More importantly, we listed similar studies in the most recent year and compared theirs with ours. We performed the functional annotation, immune relevant signature correlation analysis, and immune infiltrating analysis to thoroughly understand the functional mechanism of the signature and the immune cells' roles in the gene signature's predicting capacity. RESULTS: A sixteen-gene signature (ARSD, BCAT1, BIK, CLDN11, DLEU7-AS1, GGH, IGFBP2, LINC01037, LINC01446, LINC01497, M1AP, PCSK2, PCSK5, PPP2R2A, TIGD7, and TMSB4X) was generated from the Lasso model. We then confirmed the signature as having solid and stable prognostic capacity by several statistical methods. We revealed the superiority of our signature after comparing it to our predecessors, and the GSEA uncovered the specifically mechanism of action related to the gene signature. Two immune relevant signatures, including GZMA and LAG3 were identified associating with our signature. The immune-infiltrating analysis identified crucial roles of resting mast cells, which potentially support the sixteen-gene signature's prognosis ability. CONCLUSIONS: We discovered a robust sixteen-gene signature that can accurately predict ESCC prognosis. The immune relevant signatures, GZMA and LAG3, and resting mast cells infiltrating were closely linked to the sixteen-gene signature's ability.
  
  
  
  
**PCSK5**
-
8
of
10
  

### Aberrant APOBEC3C expression induces characteristic genomic instability in pancreatic ductal adenocarcinoma.

8. Oncogenesis. 2022 Jun 24;11(1):35. doi: 10.1038/s41389-022-00411-9
  
  
Qian Y(#)(1)(2)(3)(4), Gong Y(#)(1)(2)(3)(4), Zou X(#)(1)(2)(3)(4), Liu Y(1)(2)(3)(4), Chen Y(1)(2)( ...
  
  
Author information: (1)Department of Pancreatic Surgery, Fudan University Shanghai Cancer Center, 20 ...
  
  
Pancreatic ductal adenocarcinoma (PDAC) is a well-known lethal and heterogeneous disease. Apolipoprotein B mRNA-editing enzyme catalytic polypeptide-like (APOBEC) is an important mutagenic driver that has seldom been investigated in PDAC. Therefore, this study investigated the significance of APOBEC3C in PDAC. First, cytosine deamination-associated mutation signatures were identified in the PDAC cohorts from TCGA and Fudan University Shanghai Cancer Center (FUSCC) datasets, and C > X-enriched kataegis regions were identified in the FUSCC cohort (12 to 27 counts per sample). Patients were stratified according to APOBEC3C expression, and high APOBEC3C expression was found to correlate with a higher motif enrichment score of 5'-CC-3' and an elevated kataegis count within PCSK5 and NES genes. Second, we compared APOBEC expression in PDAC and normal pancreatic tissues and found that APOBEC3C was substantially upregulated in PDAC. APOBEC3C-overexpressing cell lines were generated to substantiate the effects of APOBEC3C on PDAC genome, including alterations in single-nucleotide variant (SNV) classes (higher proportion of C > T conversions) and the formation of kataegis regions (newly occurring kataegis regions detected in ACHE and MUC6 genes). Three different PDAC cohorts (FUSCC, TCGA, and QCMG) were analysed to evaluate the prognostic value of APOBEC3C, and APOBEC3C overexpression predicted shorter survival. Finally, the APOBEC3C overexpression correalted with the PDAC tumour microenvironment (TME) remodelling, APOBEC3C expression was associated with the invasion of CD4 + T lymphocytes and CD8 + T lymphocytes (cytotoxic T lymphocytes, CTLs), indicating enhanced immune activity and validating the practicality of APOBEC3C for guiding immunotherapy.
  
  
  
  
**PCSK5**
-
9
of
10
  

### Genetic Alterations in Papillary Thyroid Carcinoma With Hashimoto(')s Thyroiditis： ANK3, an Indolent Maintainer of Papillary Thyroid Carcinoma.

9. Front Oncol. 2022 May 12;12:894786. doi: 10.3389/fonc.2022.894786 eCollection 2022.
  
  
Zeng C(1), Long J(1), Deng C(1), Xie L(1), Ma H(1), Guo Y(1), Liu S(1), Deng M(2).
  
  
Author information: (1)Department of Pathology, The Eighth Affiliated Hospital, Sun Yat-sen Universi ...
  
  
Hashimoto's thyroiditis (TH) is a risk factor for the occurrence of papillary thyroid carcinoma (PTC), which is considered to be the most common type of thyroid cancer. In recent years, the prevalence of PTC with TH has been increasing, but little is known about the genetic alteration in PTC with TH. This study analyzed the mutation spectrum and mutation signature of somatic single nucleotide variants (SNV) for 10 non-tumor and tumor pair tissues of PTC with TH using whole-exome sequencing. The ANK3 protein expression was evaluated by immunohistochemistry in PTC with TH and PTC samples. Moreover, the functional role of ANK3 in PTC cells was determined by CCK-8 proliferation assay, colony formation assays, cell cycle analysis, cell invasion and migration and in vivo study through overexpression assay. Our results showed three distinct mutational signatures and the C>T/G>A substitution was the most common type of SNV. Gene-set enrichment analysis showed that most of the significantly mutated genes were enriched in the regulation of actin cytoskeleton signaling. Moreover, NCOR2, BPTF, ANK3, and PCSK5 were identified as the significantly mutated genes in PTC with TH, most of which have not been previously characterized. Unexpectedly, it was found that ANK3 was overexpressed in cytoplasm close to the membrane of PTC cells with TH and in almost all PTC cases, suggesting its role as a diagnostic marker of PTC. Ectopic expression of ANK3 suppressed invasion and migration, increased apoptosis of B-CPAP and TPC-1 cells. Moreover, our findings revealed that enhanced ANK3 expression inhibits growth of PTC cells both in vitro and in vivo. Ectopic expression of ANK3 significantly enhanced E-cadherin protein expression and inhibited PTC progression, at least in part, by suppression of epithelial-mesenchymal transition (EMT). Our study shows that ANK3 exerts an anti-oncogenic role in the development of PTC and might be an indolent maintainer of PTC.
  
  
  
  
**PCSK5**
-
10
of
10
  

### Identification of key tumor stroma-associated transcriptional signatures correlated with survival prognosis and tumor progression in breast cancer.

10. Breast Cancer. 2022 May;29(3):541-561. doi: 10.1007/s12282-022-01332-6 Epub 2022 Jan 12.
  
  
Uddin MN(1)(2)(3)(4), Wang X(5)(6)(7).
  
  
Author information: (1)Biomedical Informatics Research Lab, School of Basic Medicine and Clinical Ph ...
  
  
BACKGROUND: The aberrant expression of stromal gene signatures in breast cancer has been widely studied. However, the association of stromal gene signatures with tumor immunity, progression, and clinical outcomes remains lacking. METHODS: Based on eight breast tumor stroma (BTS) transcriptomics datasets, we identified differentially expressed genes (DEGs) between BTS and normal breast stroma. Based on the DEGs, we identified dysregulated pathways and prognostic hub genes, hub oncogenes, hub protein kinases, and other key marker genes associated with breast cancer. Moreover, we compared the enrichment levels of stromal and immune signatures between breast cancer patients with bad and good clinical outcomes. We also investigated the association between tumor stroma-related genes and breast cancer progression. RESULTS: The DEGs included 782 upregulated and 276 downregulated genes in BTS versus normal breast stroma. The pathways significantly associated with the DEGs included cytokine-cytokine receptor interaction, chemokine signaling, T cell receptor signaling, cell adhesion molecules, focal adhesion, and extracellular matrix-receptor interaction. Protein-protein interaction network analysis identified the stromal hub genes with prognostic value in breast cancer, including two oncogenes (COL1A1 and IL21R), two protein kinases encoding genes (PRKACA and CSK), and a growth factor encoding gene (PLAU). Moreover, we observed that the patients with bad clinical outcomes were less enriched in stromal and antitumor immune signatures (CD8 + T cells and tumor-infiltrating lymphocytes) but more enriched in tumor cells and immunosuppressive signatures (MDSCs and CD4 + regulatory T cells) compared with the patients with good clinical outcomes. The ratios of CD8 + /CD4 + regulatory T cells were lower in the patients with bad clinical outcomes. Furthermore, we identified the tumor stroma-related genes, including MCM4, SPECC1, IMPA2, and AGO2, which were gradually upregulated through grade I, II, and III breast cancers. In contrast, COL14A1, ESR1, SLIT2, IGF1, CH25H, PRR5L, ABCA6, CEP126, IGDCC4, LHFP, MFAP3, PCSK5, RAB37, RBMS3, SETBP1, and TSPAN11 were gradually downregulated through grade I, II, and III breast cancers. It suggests that the expression of these stromal genes has an association with the progression of breast cancers. These progression-associated genes also displayed an expression association with recurrence-free survival in breast cancer patients. CONCLUSIONS: This study identified tumor stroma-associated biomarkers correlated with deregulated pathways, tumor immunity, tumor progression, and clinical outcomes in breast cancer. Our findings provide new insights into the pathogenesis of breast cancer.
  
  
  
  
  

---

  

# PIK3IP1

**Signature gene in:** CD8+ T cells, MDM, Tregs
  
**Differentially expressed in:** MDM
  
  
**PIK3IP1**
-
1
of
10
  

### Therapeutic implications and comprehensive insights into cellular senescence and aging in the tumor microenvironment of sarcoma.

1. Discov Oncol. 2025 Jan 15;16(1):50. doi: 10.1007/s12672-025-01757-3
  
  
Zan P(#)(1), Zhang Y(#)(2), Zhu Y(#)(3), Chen Q(4), Duan Z(2), Guan Y(2), Liu K(5), Shang A(6), Li Z ...
  
  
Author information: (1)Department of Orthopedics, Shanghai General Hospital, School of Medicine, Sha ...
  
  
Sarcoma (SARC), a diverse group of stromal tumors arising from mesenchymal tissues, is often associated with a poor prognosis. Emerging evidence indicates that senescent cells within the tumor microenvironment (TME) significantly contribute to cancer progression and metastasis. Although the influence of senescence on SARC has been partially acknowledged, it has yet to be fully elucidated. In this study, we revealed that senescence level and age were associated with TME, immune treatment indicators, and prognosis in SARC. Utilizing the weighted gene co-expression network analysis and least absolute shrinkage and selection operator algorithm, we identified senescence-related genes and developed a senescence predictor. Three genes, RAD54, PIK3IP1, and TRIP13, were selected to construct a multiple linear regression model. Validation cohorts, immunohistochemistry, and quantitative reverse transcription polymerase chain reaction confirmed that the predictor derived from these three genes possessed prognostic and pathological relevance. Our senescence predictor provides comprehensive insights into the molecular mechanisms of SARC and identifies potential biomarkers for prognosis, paving the way for effective treatments. The results of this study hold promise for developing therapeutic strategies tailored to the unique characteristics of SARC.
  
  
  
  
**PIK3IP1**
-
2
of
10
  

### A novel necroptosis-related genes signature to predict prognosis and treatment response in bladder cancer.

2. Front Mol Biosci. 2024 Nov 25;11:1493411. doi: 10.3389/fmolb.2024.1493411 eCollection 2024.
  
  
Yao D(#)(1)(2)(3), Yu W(#)(1)(2)(3), Ma X(1)(2)(3), Tian J(1)(2)(3).
  
  
Author information: (1)Department of Urology, Lanzhou University Second Hospital, Lanzhou, China. (2 ...
  
  
BACKGROUND: Necroptosis, a form of programmed inflammatory cell death, plays a crucial role in tumor development, necrosis, metastasis, and immune response. This study aimed to explore the role of necroptosis in BLCA and construct a new prognostic model to guide clinical treatment and predict individualized treatment response. METHODS: The transcriptome profiling and the corresponding clinical data of BLCA patients were obtained from the Cancer Genome Atlas database (TCGA) and GEO databases. Univariate, multivariate and LASSO Cox regression analyses were used to identify and construct prognostic features associated with necroptosis. We constructed and validated a prognostic model associated with the patient's overall survival (OS). A nomogram was established to predict the survival rates of BLCA patients. Finally, the correlation between risk scores and tumor immune microenvironment, somatic mutations, immunotherapy, and chemotherapy was comprehensively analyzed. RESULTS: The study found two distinct NRG clusters and three gene subtypes, with significant differences in pathway enrichment and immune cell infiltration associated with different NRG clusters in the TME. In addition, we screened out six necroptosis prognosis-related genes (including PPP2R3A; CERCAM; PIK3IP1; CNTN1; CES1 and CD96) to construct a risk score prognostic model. Significant differences in overall survival rate, immune cell infiltration status, and somatic mutations existed between the high and low-risk scores in BLCA patients. Finally, drug sensitivity analysis showed that high-risk patients benefited more from immunotherapy and chemotherapy drugs. CONCLUSION: This study explores the importance of necroptosis in the prognosis of patients with BLCA, and the prognostic features associated with necroptosis that we identified can serve as new biomarkers to help develop more precise treatment strategies.
  
  
  
  
**PIK3IP1**
-
3
of
10
  

### Multi-omics features of immunogenic cell death in gastric cancer identified by combining single-cell sequencing analysis and machine learning.

3. Sci Rep. 2024 Sep 18;14(1):21751. doi: 10.1038/s41598-024-73071-x
  
  
Dai SL(#)(1), Pan JQ(#)(2), Su ZR(3).
  
  
Author information: (1)Department of General Surgery, Deqing People's Hospital, Deqing Campus, Sir R ...
  
  
Gastric cancer (GC) is a prevalent malignancy with high mortality rates. Immunogenic cell death (ICD) is a unique form of programmed cell death that is closely linked to antitumor immunity and plays a critical role in modulating the tumor microenvironment (TME). Nevertheless, elucidating the precise effect of ICD on GC remains a challenging endeavour. ICD-related genes were identified in single-cell sequencing datasets and bulk transcriptome sequencing datasets via the AddModuleScore function, weighted gene co-expression network (WGCNA), and differential expression analysis. A robust signature associated with ICD was constructed using a machine learning computational framework incorporating 101 algorithms. Furthermore, multiomics analysis, including single-cell sequencing analysis, bulk transcriptomic analysis, and proteomics analysis, was conducted to verify the correlation of these hub genes with the immune microenvironment features of GC and with GC invasion and metastasis. We screened 59 genes associated with ICD and developed a robust ICD-related gene signature (ICDRS) via a machine learning computational framework that integrates 101 different algorithms. Furthermore, we identified five key hub genes (SMAP2, TNFAIP8, LBH, TXNIP, and PIK3IP1) from the ICDRS. Through single-cell analysis of GC tumor s, we confirmed the strong correlations of the hub genes with immune microenvironment features. Among these five genes, LBH exhibited the most significant associations with a poor prognosis and with the invasion and metastasis of GC. Finally, our findings were validated through immunohistochemical staining of a large clinical sample set, and the results further supported that LBH promotes GC cell invasion by activating the epithelial-mesenchymal transition (EMT) pathway.
  
  
  
  
**PIK3IP1**
-
4
of
10
  

### PIK3IP1: structure, aberration, function, and regulation in diseases.

4. Eur J Pharmacol. 2024 Aug 15;977:176753. doi: 10.1016/j.ejphar.2024.176753 Epub 2024 Jun 17.
  
  
Jia Y(1), He P(1), Ma X(1), Lv K(1), Liu Y(2), Xu Y(3).
  
  
Author information: (1)Key Laboratory of Advanced Drug Preparation Technologies, Ministry of Educati ...
  
  
Phosphoinositide 3-kinase (PI3K) pathway, controlling diverse functions in cells, is one of the most frequently dysregulated pathways in cancer. Several negative regulators have been reported to intricately constrain the overactivation of PI3K pathway. Phosphatidylinoinosidine-3-kinase interacting protein 1 (PIK3IP1), as a unique transmembrane protein, is a newly discovered negative regulator of PI3K pathway. PIK3IP1 negatively regulates PI3K activity by directly binding to the p110 catalytic subunit of PI3K. It has been reported that PIK3IP1 is frequently low expressed in tumors and autoimmune diseases. In tumor cells and impaired cardiomyocyte, PIK3IP1 inhibits cell proliferation and survival. Consistently, the expression of PIK3IP1 is related with the condition of cancer. In addition, PIK3IP1 inhibits the inflammatory response and immune function via maintaining the quiescent state of immune cells. Thus, low expression of PIK3IP1 represents the severe condition of autoimmune diseases. PIK3IP1 is regulated by transcription factors, epigenetic factors or micro-RNAs to facilitate its normal function in different cellular contexts. This review integrates the total findings on PIK3IP1 in different disease, and summaries the structure, biological functions and regulatory mechanisms of PIK3IP1.
  
  
  
  
**PIK3IP1**
-
5
of
10
  

### CircPDE5A-encoded novel regulator of the PI3K/AKT pathway inhibits esophageal squamous cell carcinoma progression by promoting USP14-mediated de-ubiquitination of PIK3IP1.

5. J Exp Clin Cancer Res. 2024 Apr 24;43(1):124. doi: 10.1186/s13046-024-03054-3
  
  
Lei K(#)(1)(2), Liang R(#)(1)(2), Liang J(#)(1)(2), Lu N(#)(1)(3), Huang J(1)(2), Xu K(1)(2), Tan B( ...
  
  
Author information: (1)Guangdong Provincial Key Laboratory of Malignant Tumor Epigenetics and Gene R ...
  
  
BACKGROUND: Esophageal squamous cell carcinoma (ESCC) is a common gastrointestinal tumor and has become an important global health problem. The PI3K/AKT signaling pathway plays a key role in the development of ESCC. CircRNAs have been reported to be involved in the regulation of the PI3K/AKT pathway, but the underlying mechanisms are unclear. Therefore, this study aimed to identify protein-coding circRNAs and investigate their functions in ESCC. METHODS: Differential expression of circRNAs between ESCC tissues and adjacent normal tissues was identified using circRNA microarray analysis. Thereafter, LC-MS/MS was used to identify circPDE5A-encoded novel protein PDE5A-500aa. Molecular biological methods were used to explore the biological functions and regulatory mechanisms of circPDE5A and PDE5A-500aa in ESCC. Lastly, circRNA-loaded nanoplatforms were constructed to investigate the therapeutic translation value of circPDE5A. RESULTS: We found that circPDE5A expression was down-regulated in ESCC cells and tissues and that it was negatively associated with advanced clinicopathological stages and poorer prognosis in ESCC. Functionally, circPDE5A inhibited ESCC proliferation and metastasis in vitro and in vivo by encoding PDE5A-500aa, a key regulator of the PI3K/AKT signaling pathway in ESCC. Mechanistically, PDE5A-500aa interacted with PIK3IP1 and promoted USP14-mediated de-ubiquitination of the k48-linked polyubiquitin chain at its K198 residue, thereby attenuating the PI3K/AKT pathway in ESCC. In addition, Meo-PEG-S-S-PLGA-based reduction-responsive nanoplatforms loaded with circPDE5A and PDE5A-500aa plasmids were found to successfully inhibit the growth and metastasis of ESCC in vitro and in vivo. CONCLUSION: The novel protein PDE5A-500aa encoded by circPDE5A can act as an inhibitor of the PI3K/AKT signaling pathway to inhibit the progression of ESCC by promoting USP14-mediated de-ubiquitination of PIK3IP1 and may serve as a potential target for the development of therapeutic agents.
  
  
  
  
**PIK3IP1**
-
6
of
10
  

### PRMT5 supports multiple oncogenic pathways in mantle cell lymphoma.

6. Blood. 2023 Sep 7;142(10):887-902. doi: 10.1182/blood.2022019419
  
  
Sloan SL(1)(2), Brown F(1), Long M(1)(2), Weigel C(1), Koirala S(1), Chung JH(1), Pray B(1)(2), Vill ...
  
  
Author information: (1)Division of Hematology, Department of Internal Medicine, College of Medicine, ...
  
  
Mantle cell lymphoma (MCL) is an incurable B-cell malignancy with an overall poor prognosis, particularly for patients that progress on targeted therapies. Novel, more durable treatment options are needed for patients with MCL. Protein arginine methyltransferase 5 (PRMT5) is overexpressed in MCL and plays an important oncogenic role in this disease via epigenetic and posttranslational modification of cell cycle regulators, DNA repair genes, components of prosurvival pathways, and RNA splicing regulators. The mechanism of targeting PRMT5 in MCL remains incompletely characterized. Here, we report on the antitumor activity of PRMT5 inhibition in MCL using integrated transcriptomics of in vitro and in vivo models of MCL. Treatment with a selective small-molecule inhibitor of PRMT5, PRT-382, led to growth arrest and cell death and provided a therapeutic benefit in xenografts derived from patients with MCL. Transcriptional reprograming upon PRMT5 inhibition led to restored regulatory activity of the cell cycle (p-RB/E2F), apoptotic cell death (p53-dependent/p53-independent), and activation of negative regulators of B-cell receptor-PI3K/AKT signaling (PHLDA3, PTPROt, and PIK3IP1). We propose pharmacologic inhibition of PRMT5 for patients with relapsed/refractory MCL and identify MTAP/CDKN2A deletion and wild-type TP53 as biomarkers that predict a favorable response. Selective targeting of PRMT5 has significant activity in preclinical models of MCL and warrants further investigation in clinical trials.
  
  
  
  
**PIK3IP1**
-
7
of
10
  

### MLL4 Regulates the Progression of Non-Small-Cell Lung Cancer by Regulating the PI3K/AKT/SOX2 Axis.

7. Cancer Res Treat. 2023 Jul;55(3):778-803. doi: 10.4143/crt.2022.1042 Epub 2023 Jan 26.
  
  
Yang Y(1)(2)(3), Qiu R(1)(2)(3), Weng Q(1)(2)(3), Xu Z(1)(2)(3), Song J(1)(2)(3), Zhao S(1)(2)(3), M ...
  
  
Author information: (1)Key Laboratory of Imaging Diagnosis and Minimally Invasive Intervention Resea ...
  
  
PURPOSE: Mixed-lineage leukemia protein 4 (MLL4/KMT2D) is a histone methyltransferase, and its mutation has been reported to be associated with a poor prognosis in many cancers, including lung cancer. We investigated the function of MLL4 in lung carcinogenesis. MATERIALS AND METHODS: RNA sequencing (RNA-seq) in A549 cells transfected with control siRNA or MLL4 siRNA was performed. Also, we used EdU incorporation assay, colony formation assays, growth curve analysis, transwell invasion assays, immunohistochemical staining, and in vivo bioluminescence assay to investigate the function of MLL4 in lung carcinogenesis. RESULTS: We found that MLL4 expression was downregulated in non-small cell lung cancer (NSCLC) tissues compared to adjacent normal tissues and tended to decrease with disease stage progression. We analyzed the transcriptomes in control and MLL4- deficient cells using high-throughput RNA deep sequencing (RNA-seq) and identified a cohort of target genes, such as SOX2, ATF1, FOXP4, PIK3IP1, SIRT4, TENT5B, and LFNG, some of which are related to proliferation and metastasis. Our results showed that low expression of MLL4 promotes NSCLC cell proliferation and metastasis and is required for the maintenance of NSCLC stem cell properties. CONCLUSION: Our findings identify an important role of MLL4 in lung carcinogenesis through transcriptional regulation of PIK3IP1, affecting the PI3K/AKT/SOX2 axis, and suggest that MLL4 could be a potential prognostic indicator and target for NSCLC therapy.
  
  
  
  
**PIK3IP1**
-
8
of
10
  

### Upregulation of PIK3IP1 monitors the anti-cancer activity of PI3Kα inhibitors in gastric cancer cells.

8. Biochem Pharmacol. 2023 Jan;207:115380. doi: 10.1016/j.bcp.2022.115380 Epub 2022 Dec 12.
  
  
Ma XB(1), Wang Y(1), Jia YJ(1), Liu YJ(1), Tian YQ(1), Liu Y(2), Hou GQ(3), Xu YC(4), Liu HM(5).
  
  
Author information: (1)Key Laboratory of Advanced Drug Preparation Technologies, Ministry of Educati ...
  
  
Gastric cancer remains one of the most malignant cancers in the world. The target-based drugs approved by FDA for gastric cancer treatment include only three targets and benefit a small portion of gastric cancer patients. PIK3CA, a confirmed oncogene, mutates in 7-25% gastric cancer patients. PI3Kα inhibitor BYL719 has been approved for treating specific breast cancer. However, there is no comprehensive study about PI3Kα inhibitor in gastric cancer. In this study, we found pharmacological inhibition or knockdown of PI3Kα effectively inhibited the proliferation of partial gastric cancer cells. Then, we systematically explored the potential biomarkers for predicting or monitoring treatment response according to previous reports and found that basal expression of several receptor tyrosine kinases were related with the sensitivity of gastric cancer cells to BYL719. Next, RNA-seq technique was utilized and showed that BYL719 inhibited Myc targets V2 gene set in sensitive gastric cancer cells, and western blotting further verified that c-Myc was only inhibited in sensitive gastric cancer cells. More importantly, we firstly found BYL719 significantly elevated the expression of PIK3IP1 in sensitive gastric cancer cells, which was also observed in NCI-N87 cell derived xenograft mice models. Meanwhile, knockdown of PIK3IP1 partially rescued the cell growth inhibited by BYL719 in sensitive gastric cancer cells, suggesting the important role of PIK3IP1 in the antitumor activity of BYL719. In conclusion, our study provides biological evidence that PI3Kα is a promising target in specific gastric cancer and the elevation of PIK3IP1 could supply as a biomarker that monitoring treatment response.
  
  
  
  
**PIK3IP1**
-
9
of
10
  

### Targeting EZH2 Promotes Chemosensitivity of BCL-2 Inhibitor through Suppressing PI3K and c-KIT Signaling in Acute Myeloid Leukemia.

9. Int J Mol Sci. 2022 Sep 27;23(19):11393. doi: 10.3390/ijms231911393
  
  
Yang C(1), Gu Y(1), Ge Z(1), Song C(2)(3).
  
  
Author information: (1)Department of Hematology, Zhongda Hospital, School of Medicine, Southeast Uni ...
  
  
Acute myeloid leukemia (AML) is one of the most common hematological malignancies with high heterogeneity, characterized by a differentiating block at the early progenitor stage. The selective BCL-2 inhibitor, Venetoclax (Ven), has shown exciting clinical results in a certain group of AML patients. However, Ven alone is insufficient to reach an enduringly complete response, which leads to the concern of Ven resistance. Alternative combined therapies with Ven are demanded in AML. Here, we reported the synergistic effect and molecular mechanism of the enhancer of zeste homolog 2 (EZH2) inhibitor DZNeP with Ven in AML cells. Results showed that the combination of DZNeP with Ven significantly induces cell proliferation arrest compared to single-drug control in AML cells and primary samples, and CalcuSyn analysis showed their significant synergy. The combination also significantly promotes apoptosis and increases the expression of pro-apoptotic proteins. The whole transcriptome analysis showed that phosphoinositide-3-kinase-interacting protein1 (PIK3IP1), the PI3K/AKT/mTOR signaling suppressor, is upregulated upon DZNeP treatment. Moreover, EZH2 is upregulated but PIK3IP1 is downregulated in 88 newly diagnosed AML cohorts compared to 70 healthy controls, and a higher expression of EZH2 is associated with poor outcomes in AML patients. Particularly, the combination of DZNeP with Ven dramatically eliminated CD117 (c-KIT) (+) AML blasts, suggesting the effect of the combination on tumor stem cells. In summary, our data indicated that DZNeP increases the sensitivity of Ven in AML by affecting PI3K and c-KIT signaling in AML. Our results also suggested that the therapeutic targeting of both EZH2 and BCL-2 provides a novel potential combined strategy against AML.
  
  
  
  
**PIK3IP1**
-
10
of
10
  

### Characterization of 22q12 Microdeletions Causing Position Effect in Rare NF2 Patients with Complex Phenotypes.

10. Int J Mol Sci. 2022 Sep 2;23(17):10017. doi: 10.3390/ijms231710017
  
  
Tritto V(1), Eoli M(2), Paterra R(2), Redaelli S(3), Moscatelli M(4), Rusconi F(1), Riva P(1).
  
  
Author information: (1)Dipartimento di Biotecnologie Mediche e Medicina Traslazionale, Università de ...
  
  
Neurofibromatosis type 2 is an autosomal dominant tumor-prone disorder mainly caused by NF2 point mutations or intragenic deletions. Few individuals with a complex phenotype and 22q12 microdeletions have been described. The 22q12 microdeletions' pathogenic effects at the genetic and epigenetic levels are currently unknown. We here report on 22q12 microdeletions' characterization in three NF2 patients with different phenotype complexities. A possible effect of the position was investigated by in silico analysis of 22q12 topologically associated domains (TADs) and regulatory elements, and by expression analysis of 12 genes flanking patients' deletions. A 147 Kb microdeletion was identified in the patient with the mildest phenotype, while two large deletions of 561 Kb and 1.8 Mb were found in the other two patients, showing a more severe symptomatology. The last two patients displayed intellectual disability, possibly related to AP1B1 gene deletion. The microdeletions change from one to five TADs, and the 22q12 chromatin regulatory landscape, according to the altered expression levels of four deletion-flanking genes, including PIK3IP1, are likely associated with an early ischemic event occurring in the patient with the largest deletion. Our results suggest that the identification of the deletion extent can provide prognostic markers, predictive of NF2 phenotypes, and potential therapeutic targets, thus overall improving patient management.
  
  
  
  
  

---

  

# PPM1B

**Signature gene in:** MDM
  
**Differentially expressed in:** MDM
  
  
**PPM1B**
-
1
of
10
  

### PPM1B degradation mediated by TRIM25 ubiquitination modulates cell cycle and promotes gastric cancer growth.

1. Sci Rep. 2025 Feb 20;15(1):6160. doi: 10.1038/s41598-025-89519-7
  
  
Zhu GW(1)(2)(3), Chen H(#)(1)(2)(3), Liu SY(#)(1)(2)(3), Lin PH(1)(2)(3)(4), Lin CL(1)(2)(3)(4), Ye ...
  
  
Author information: (1)Department of Gastrointestinal Surgery 2 Section, the First Affiliated Hospit ...
  
  
Protein phosphatase PPM1B, a member of the serine/threonine phosphatase family, has been implicated in various human cancers. In this study, our objective was to investigate the role of PPM1B in GC growth and explore the underlying mechanisms. Our findings revealed that PPM1B expression was downregulated in GC tissues, and higher levels of PPM1B expression were associated with improved overall survival in GC patients. Overexpression of PPM1B significantly inhibited cell proliferation, induced G1 phase cell cycle arrest, and suppressed tumor growth. Conversely, knockdown or knockout of PPM1B yielded opposite effects. Mechanistically, we identified that PPM1B exerted its inhibitory role in GC cell growth and cell cycle regulation through the TRIM25/PPM1B/CDK2 signaling pathway. Specifically, we demonstrated that TRIM25 physically interacts with PPM1B, leading to enhanced degradation of PPM1B and subsequent modulation of CDK2 phosphorylation and GC cell growth. PPM1B emerges as a potential prognostic biomarker and therapeutic target in GC. These findings hold clinical significance by offering opportunities to improve diagnosis and treatment strategies for GC patients. Furthermore, this study provides novel insights into the pathogenesis and progression of GC, expanding our understanding of this disease.
  
  
  
  
**PPM1B**
-
2
of
10
  

### Magnesium-dependent-protein phosphatase 1B regulates the protein arginine methyltransferase 5 through the modulation of myosin phosphatase.

2. J Biol Chem. 2025 Feb;301(2):108107. doi: 10.1016/j.jbc.2024.108107 Epub 2024 Dec 18.
  
  
Keller I(1), Ungvári Á(1), Major E(1), Horváth D(1), Kónya Z(1), Tóth E(1), Erdődi F(1), Kiss A(1), ...
  
  
Author information: (1)Department of Medical Chemistry, Faculty of Medicine, University of Debrecen, ...
  
  
Dysregulation of the expression levels and the activity of kinases/phosphatases is an intrinsic hallmark of tumor transformation and progression, as either as a primary cause or consequence. The myosin phosphatase (MP)/protein arginine methyltransferase 5 (PRMT5)/histone (H4) pathway is an oncogenic signaling pathway downregulating the gene expression of tumor suppressors. However, the upstream regulators of the pathway are unknown. We show that the Mg2+-dependent protein phosphatase 1 B (PP2CB or PPM1B) interacts and regulates MP through the MYPT1 regulatory subunit, and this interplay results in the inactivation of the tumorigenic pathway driven by PRMT5. The phospho-Thr696 inhibitory residues of the MYPT1 regulatory subunit of MP was dephosphorylated by PPM1B. The inhibition of PPM1B by sanguinarine resulted in the deactivation of MP and the increased activity of PRMT5 leading to increased symmetric dimethylation of histone H4 in HeLa cells. The overexpression of the PPM1B had the opposite action. The overexpression of PPM1B decreased the colonization activity of HeLa cells through modulation of MP. Finally, human cervical carcinoma biopsies showed almost complete elimination of PPM1B compared to their healthy control counterparts. The phosphorylation of the inhibitory MYPT1pT696 and the regulatory PRMT5pT80 residues and the symmetric dimethylation of H4 were elevated in the cancer biopsies and it resulted in a decrease in retinoblastoma protein expression. The results indicate a tumor suppressor role of the PPM1B/MP axis via inhibition of PRMT5, thereby regulating gene expression through H4 arginine dimethylation. Collectively, PPM1B is a tumor suppressor and a possible tumor marker for cervical carcinoma.
  
  
  
  
**PPM1B**
-
3
of
10
  

### Application of large-scale and multicohort plasma proteomics data to discover novel causal proteins in gastric cancer.

3. Discov Oncol. 2024 Oct 18;15(1):570. doi: 10.1007/s12672-024-01460-9
  
  
Tang W(1), Ma X(2).
  
  
Author information: (1)College of Liberal Arts and Sciences, University of Florida, Gainesville, USA ...
  
  
PURPOSES: Gastric cancer (GC) is one of the most common malignant tumors threatening human beings and has a poor prognosis. Therefore, exploring unveiled biomarkers or therapeutic targets for the diagnosis and treatment of GC is crucial. METHODS: A total of 5772 protein quantitative trait loci (pQTL) were aggregated from four latest large-scale proteomics cohorts. Two-sample Mendelian randomization (two-sample MR) was utilized to identify the causal effect of blood plasma proteins on GC. Heterogeneity, pleiotropy, and directionality analyses were employed to evaluate proteins identified via two-sample MR. The robustness of results was further validated via colocalization. The drug targets of proteins were evaluated to reveal the compounds that can interfere with these proteins. RESULTS: Ten proteins with potential causations in relation to GC were identified: LY6D, SLURP1, MLN, PSCA, THSD1, CFTR, PPM1B, KDM3A, TSC1, and HCG22. Among these proteins, LY6D, SLURP1, and THSD1 were considered as the most reliable biomarkers of GC due to their significant H4 posterior probabilities in colocalization analysis and absence of pleiotropy. Compound 35, nitrosamide, and 0175029-0000 were potential drugs or small molecules targeting LY6D, SLURP1, and THSD1, respectively. CONCLUSION: This study identified several plasma proteins as potential biomarkers of GC and provided data support and new insights into the early diagnosis, intervention, and therapeutic targets of GC.
  
  
  
  
**PPM1B**
-
4
of
10
  

### MLKL promotes hepatocarcinogenesis through inhibition of AMPK-mediated autophagy.

4. Cell Death Differ. 2024 Aug;31(8):1085-1098. doi: 10.1038/s41418-024-01314-5 Epub 2024 May 23.
  
  
Yu X(1)(2), Feng M(1)(2), Guo J(2), Wang H(1)(2), Yu J(3), Zhang A(1)(2), Wu J(1)(2), Han Y(4), Sun ...
  
  
Author information: (1)Department of Gastroenterology, Renmin Hospital, School of Basic Medical Scie ...
  
  
The pseudokinase mixed lineage kinase domain-like (MLKL) is an essential component of the activation of the necroptotic pathway. Emerging evidence suggests that MLKL plays a key role in liver disease. However, how MLKL contributes to hepatocarcinogenesis has not been fully elucidated. Herein, we report that MLKL is upregulated in a diethylnitrosamine (DEN)-induced murine HCC model and is associated with human hepatocellular carcinomas. Hepatocyte-specific MLKL knockout suppresses the progression of hepatocarcinogenesis. Conversely, MLKL overexpression aggravates the initiation and progression of DEN-induced HCC. Mechanistic study reveals that deletion of MLKL significantly increases the activation of autophagy, thereby protecting against hepatocarcinogenesis. MLKL directly interacts with AMPKα1 and inhibits its activity independent of its necroptotic function. Mechanistically, MLKL serves as a bridging molecule between AMPKα1 and protein phosphatase 1B (PPM1B), thus enhancing the dephosphorylation of AMPKα1. Consistently, MLKL expression correlates negatively with AMPKα1 phosphorylation in HCC patients. Taken together, our findings highlight MLKL as a novel AMPK gatekeeper that plays key roles in inhibiting autophagy and driving hepatocarcinogenesis, suggesting that the MLKL-AMPKα1 axis is a potential therapeutic target for HCC.
  
  
  
  
**PPM1B**
-
5
of
10
  

### Epithelial-mesenchymal transition induced by tumor cell-intrinsic PD-L1 signaling predicts a poor response to immune checkpoint inhibitors in PD-L1-high lung cancer.

5. Br J Cancer. 2024 Jul;131(1):23-36. doi: 10.1038/s41416-024-02698-4 Epub 2024 May 10.
  
  
Jeong H(1)(2), Koh J(3), Kim S(4), Song SG(3)(5), Lee SH(1)(5)(6)(7), Jeon Y(2), Lee CH(1)(5)(6)(7), ...
  
  
Author information: (1)Cancer Research Institute, Seoul National University, Seoul, Republic of Kore ...
  
  
Erratum in Br J Cancer. 2024 Sep;131(4):779-780. doi: 10.1038/s41416-024-02798-1.
  
  
  
  
**PPM1B**
-
6
of
10
  

### Modulation of YBX1-mediated PANoptosis inhibition by PPM1B and USP10 confers chemoresistance to oxaliplatin in gastric cancer.

6. Cancer Lett. 2024 Apr 10;587:216712. doi: 10.1016/j.canlet.2024.216712 Epub 2024 Feb 15.
  
  
Lin C(1), Lin P(2), Yao H(2), Liu S(2), Lin X(2), He R(2), Teng Z(2), Zuo X(3), Li Y(3), Ye J(4), Zh ...
  
  
Author information: (1)Department of Gastrointestinal Surgery 2 Section, Institute of Abdominal Surg ...
  
  
Gastric cancer (GC) is a common malignant tumor of the digestive tract, and chemoresistance significantly impacts GC patients' prognosis. PANoptosis has been associated with oxaliplatin-induced cell death. However, the direct regulatory role of YBX1 in cellular chemoresistance through PANoptosis remains unclear. In this study, we investigated the impact of YBX1 on regulating PANoptosis and its influence on the resistance of gastric cancer cells to oxaliplatin. Through overexpression and silencing experiments, we assessed YBX1's effect on proliferation and PANoptosis regulation in gastric cancer cells. Additionally, we identified PPM1B and USP10 as interacting proteins with YBX1 and confirmed their influence on YBX1 molecular function and protein expression levels. Our results demonstrate that YBX1 suppresses PANoptosis, leading to enhanced resistance of gastric cancer cells to oxaliplatin. Furthermore, we found that PPM1B and USP10 play critical roles in regulating YBX1-mediated PANoptosis inhibition. PPM1B directly interacts with YBX1, causing dephosphorylation of YBX1 at serine 314 residue. This dephosphorylation process affects the deubiquitination of YBX1 mediated by USP10, resulting in decreased YBX1 protein expression levels and impacting PANoptosis and oxaliplatin resistance in gastric cancer cells. Additionally, we discovered that the 314th amino acid of YBX1 has a profound impact on its own protein expression abundance, thereby affecting the functionality of YBX1. In conclusion, our study reveals the significance of PPM1B-mediated dephosphorylation of YBX1 and USP10-mediated deubiquitination in regulating PANoptosis and sensitivity to oxaliplatin in gastric cancer cells. These findings offer a potential therapeutic strategy for patients with oxaliplatin-resistant gastric cancer.
  
  
  
  
**PPM1B**
-
7
of
10
  

### RBM10 regulates the tumorigenic potential of human cancer cells by modulating PPM1B and YBX1 activities.

7. Exp Cell Res. 2024 Feb 15;435(2):113932. doi: 10.1016/j.yexcr.2024.113932 Epub 2024 Jan 20.
  
  
Li Y(1), Wei D(2), Chen Z(1), Chen Y(1), Deng Y(1), Li M(1), Zhao Y(3), Niu K(4).
  
  
Author information: (1)CAS Key Laboratory of Genomic and Precision Medicine, Beijing Institute of Ge ...
  
  
RNA binding protein RBM10 participates in various RNA metabolism, and its decreased expression or loss of function by mutation has been identified in many human cancers. However, how its dysregulation contributes to human cancer pathogenesis remains to be determined. Here, we found that RBM10 expression was decreased in breast tumors, and breast cancer patients with low RBM10 expression presented poorer survival rates. RBM10 depletion in breast cancer cells significantly promotes the cellular proliferation and migration. We further demonstrated that RBM10 forms a triple complex with YBX1 and phosphatase 1B (PPM1B), in which PPM1B serves as the phosphatase of YBX1. RBM10 knock-down markedly attenuated association between YBX1 and PPM1B, leading to elevated levels of YBX1 phosphorylation and its nuclear translocation. Furthermore, cancer cells with RBM10 depletion had a significantly accelerated tumor growth in nude mice. Importantly, these enhanced tumorigenic phenotypes can be reversed by overexpression of PPM1B. Our findings provide the mechanistic bases for functional loss of RBM10 in promoting tumorigenicity, and are potentially useful in the development of combined therapeutic strategies for cancer patients with defective RBM10.
  
  
  
  
**PPM1B**
-
8
of
10
  

### Overexpression of PPM1B inhibited chemoresistance to temozolomide and proliferation in glioma cells.

8. Cell Biol Int. 2024 Feb;48(2):143-153. doi: 10.1002/cbin.12092 Epub 2023 Oct 5.
  
  
Yu Y(1)(2), Liu Q(3), Ran Q(3), Cao F(3).
  
  
Author information: (1)Neurosurgery Department, People's Hospital of Honghuagang District of Zunyi, ...
  
  
Protein phosphatase magnesium-dependent 1B (PPM1B) functions as IKKβ phosphatases to terminate nuclear factor kappa B (NF-κB) signaling. NF-κB signaling was constitutively activated in glioma cells. At present, little is known about the role of PPM1B in glioma. In the current study, we found that the expression of PPM1B was reduced in glioma tissues and cells, and decreased expression of PPM1B was related to poor overall survival of patients. Overexpression of PPM1B inhibited the proliferation and promoted apoptosis of glioma cells. Moreover, PPM1B overexpression reduced the phosphorylation of IKKβ and inhibited the nuclear localization of NF-κBp65. PDTC, an inhibitor of NF-κB signaling, reversed PPM1B-knockdown-induced cell proliferation. Furthermore, overexpression of PPM1B enhanced the sensitivity of glioma cells to temozolomide. In vivo experiments showed that overexpression of PPM1B could inhibit tumor growth, improve the survival rate of nude mice, and enhance the sensitivity to temozolomide. In conclusion, PPM1B suppressed glioma cell proliferation and the IKKβ-NF-κB signaling pathway, and enhanced temozolomide sensitivity of glioma cells.
  
  
  
  
**PPM1B**
-
9
of
10
  

### TXLNA enhances TBK1 phosphorylation by suppressing PPM1B recruitment.

9. Biochim Biophys Acta Mol Cell Res. 2023 Oct;1870(7):119550. doi: 10.1016/j.bbamcr.2023.119550 Epub 2023 Jul 26.
  
  
Tian X(1), Zhang Z(1), Ding M(2).
  
  
Author information: (1)School of Life Science and Technology, China Pharmaceutical University, Nanji ...
  
  
In recent years, there has been a notable increase in cancer incidence and mortality, and immune abnormalities have been closely linked to malignancy development. TANK-binding kinase 1 (TBK1) is a non-classical IκB kinase that regulates interferon and NF-κB signaling pathways and plays a crucial role in innate immunity. Recent studies have shown high expression levels of TBK1 and increased activity in various tumor cells, suggesting its involvement in the development and progression of multiple cancers. Targeting TBK1 for tumor therapy may be a possibility. However, little is known about the abnormal activation and dynamic regulation of TBK1 in cancer. First, we utilized the BioID biotinylation technique combined with TMT-based quantitative proteomics to analyze the TBK1 interacting proteins. Our results revealed that TXLNA interacts with TBK1 and binds to the α-helical scaffold of TBK1. The expression of TXLNA could affect the S172 phosphorylation of TBK1. PPM1B is a phosphatase that can dephosphorylate TBK1 S172, so we used the APEX2 proximity labeling technique combined with TMT-based quantitative proteomics to explore the interacting proteins of PPM1B and search for the regulatory pathway of TXLNA on TBK1 phosphorylation. We found that PPM1B interacts with TXLNA. Based on these results, we further found that TXLNA impairs the binding of PPM1B to TBK1, inhibiting the dephosphorylation of TBK1 and contributing to the abnormal enhancement of TBK1 activity in cancer cells. This study sheds light on the potential mechanism of aberrant activation and dynamic regulation of TBK1 in tumors and provides a potential target for tumor therapy.
  
  
  
  
**PPM1B**
-
10
of
10
  

### Pleckstrin-2-promoted PPM1B degradation plays an important role in transforming growth factor-β-induced breast cancer cell invasion and metastasis.

10. Cancer Sci. 2023 Jun;114(6):2429-2444. doi: 10.1111/cas.15791 Epub 2023 Mar 30.
  
  
Du L(1)(2), Li J(1)(2), Tian Y(3)(4), Feng R(1)(2).
  
  
Author information: (1)Department of Biochemistry and Molecular Biology, College of Life Sciences, P ...
  
  
Transforming growth factor-β (TGF-β) is known to promote breast cancer cell migration, invasion, and dissemination; however, the underlying molecular mechanisms are not yet well characterized. Here, we report that TGF-β induces pleckstrin-2 (PLEK2) expression by Smad3 and signal transducer and activator of transcription 3 (STAT3) activating PLEK2 promoter activity. Higher PLEK2 expression is associated with poor prognosis in breast cancer patients. Overexpression and knockout experiments in MDA-MB-231 and MCF-7 breast cancer cells revealed that PLEK2 promotes cell migration, invasion, and dissemination in 2D and 3D cell culture. Moreover, PLEK2 promotes metastasis of breast cancer cells in vivo. Pleckstrin-2 localizes to the cell membrane and cell protrusions following TGF-β treatment. Furthermore, inhibition of PI3K phosphorylation abolishes TGF-β- and PLEK2-induced cell invasion. The carboxyl-terminal PH domain of PLEK2 is critical for TGF-β- and PLEK2-induced Akt activation and plays an important role in cell invasion. Pleckstrin-2 interacts with PPM1B and promotes its ubiquitin-dependent degradation. The PLEK2-PPM1B axis utilizes nuclear factor-κB signaling to promote cell migration and invasion. Our data implicate the TGF-β-STAT3/Smad3-PLEK2-PPM1B signaling cascade in TGF-β-induced breast cancer cell migration and invasion. These findings suggest that PLEK2/PPM1B could represent novel targets for the intervention of breast cancer metastasis.
  
  
  
  
  

---

  

# PRKG1

**Signature gene in:** Stromal cells
  
**Differentially expressed in:** Stromal cells
  
  
**PRKG1**
-
1
of
10
  

### Long non-coding RNA PRKG1-AS1 promotes cell proliferation and migration in lung adenocarcinoma.

1. Respir Res. 2025 Feb 21;26(1):64. doi: 10.1186/s12931-025-03122-y
  
  
Jiao T(1), Wen H(1), Zeng L(1), Chen Y(1), Shi J(1), Yang S(2).
  
  
Author information: (1)Department of Respiratory and Critical Care Medicine, The Second Affiliated H ...
  
  
In previous studies, we have discovered a significant correlation between cGMP-dependent protein kinase I antisense RNA 1 (PRKG1-AS1) and the prognosis of lung adenocarcinoma (LUAD). Through analysis of The Cancer Genome Atlas (TCGA) database and expression data from non-small cell lung cancer tissues and cells, we found that PRKG1-AS1 is overexpressed in LUAD tissues. High expression of PRKG1-AS1 is associated with poor prognosis in LUAD patients. Cox regression analysis revealed that PRKG1-AS1 is an independent factor affecting the prognosis of LUAD. Gene Set Enrichment Analysis (GSEA) results indicated that PRKG1-AS1 might participate in various cancer-related biological processes and signaling pathways, including immune response. Furthermore, our study demonstrated that knockdown of PRKG1-AS1 expression inhibited proliferation and metastasis in LUAD. Correlation analysis between PRKG1-AS1 and protein-coding genes (PCGs) revealed a positive correlation between PRKG1-AS1 and dickkopf-1 (DKK1). Downregulation of PRKG1-AS1 led to decreased expression of DKK1, which is highly expressed in LUAD and is associated with poor prognosis. In summary, our findings suggest that PRKG1-AS1 may function as an oncogene contributing to the development of LUAD and holds significant prognostic value.
  
  
  
  
**PRKG1**
-
2
of
10
  

### The clinical significance and anti-tumor role of PRKG1 in bladder cancer.

2. Front Immunol. 2024 Jul 30;15:1442555. doi: 10.3389/fimmu.2024.1442555 eCollection 2024.
  
  
Jin L(#)(1), Chen T(#)(2), Sun H(#)(3), Dai G(1), Yao Q(1), Yuan F(1), Liu X(1), Xue B(1).
  
  
Author information: (1)Department of Urology, the Second Affiliated Hospital of Soochow University, ...
  
  
INTRODUCTION: cGMP-dependent protein kinase 1 (PRKG1) has shown to be associated with some tumorigenesis, while the role of PRKG1 in bladder cancer is unclear. METHODS: To investigate the biological and clinical significance of PRKG1 in bladder cancer, we detected the expression of PRKG1 and explored the function of PRKG1 in bladder cancer cells. The PRKG1 transcripts data was downloaded from The Cancer Genome Atlas (TCGA) database, and immunohistochemistry staining was conducted on formalin-fixed paraffin-embedded (FFPE) sample tissues. Relationship between clinical characteristics of patients and expression of PRKG1 was analyzed in FFPE samples, TCGA database, and GSE19423 dataset. PRKG1 was over-expressed, and cell proliferation, migration, invasion, apoptosis, and spheroidizing ability were then detected. Chemosensitivity to cisplatin was detected with cell viability, and half-maximal drug inhibitory concentration (IC50) was calculated. In addition, the relation between PRKG1 expression and the infiltration level of tumor immune cells in tumor microenvironment were analyzed. RESULTS: The results showed expression of PRKG1 was lower in bladder cancer, compared with normal tissues both at protein and transcript levels. Lower PRKG1 expression was related to higher tumor grade, T stage, and muscle invasion, also predicted worse overall survival and recurrence free survival in patients treated with Bacillus Calmette-Guerin (BCG) intravesical immunotherapy. Analysis of tumor immune cells infiltration showed lower PRKG1 was associated with non-inflamed tumor microenvironment. CONCLUSION: The present study firstly identified the anti-tumor role and tumor immune regulatory role of PRKG1, also found loss of PRKG1 could be used as a prognosis factor. The present study provided a potential biomarker and therapy target to bladder cancer.
  
  
  
  
**PRKG1**
-
3
of
10
  

### Cancer-associated fibroblast-derived gene signature discriminates distinct prognoses by integrated single-cell and bulk RNA-seq analyses in breast cancer.

3. Aging (Albany NY). 2024 May 9;16(9):8279-8305. doi: 10.18632/aging.205817 Epub 2024 May 9.
  
  
Fang Z(1), Han YL(2), Gao ZJ(1), Yao F(1).
  
  
Author information: (1)Department of Breast and Thyroid Surgery, Renmin Hospital of Wuhan University ...
  
  
BACKGROUND: Cancer-associated fibroblasts (CAFs) are one of the most predominant cellular subpopulations in the tumor stroma and play an integral role in cancer occurrence and progression. However, the prognostic role of CAFs in breast cancer remains poorly understood. METHODS: We identified a number of CAF-related biomarkers in breast cancer by combining single-cell and bulk RNA-seq analyses. Based on univariate Cox regression as well as Least Absolute Shrinkage and Selection Operator (LASSO) regression analysis, a novel CAF-associated prognostic model was developed. Breast cancer patients were grouped according to the median risk score and further analyzed for outcome, clinical characteristic, pathway activity, genomic feature, immune landscape, and drug sensitivity. RESULTS: A total of 341 CAF-related biomarkers were identified from single-cell and bulk RNA-seq analyses. We eventually screened eight candidate prognostic genes, including CERCAM, EMP1, SDC1, PRKG1, XG, TNN, WLS, and PDLIM4, and constructed the novel CAF-related prognostic model. Grouped by the median risk score, high-risk patients showed a significantly worse prognosis and exhibited distinct pathway activities such as uncontrolled cell cycle progression, angiogenesis, and activation of glycolysis. In addition, the combined risk score and tumor mutation burden significantly improved the ability to predict patient prognosis. Importantly, patients in the high-risk group had a higher infiltration of M2 macrophages and a lower infiltration of CD8+ T cells and activated NK cells. Finally, we calculated the IC50 for a range of anticancer drugs and personalized the treatment regimen for each patient. CONCLUSION: Integrating single-cell and bulk RNA-seq analyses, we identified a list of compositive CAF-associated biomarkers and developed a novel CAF-related prognostic model for breast cancer. This robust CAF-derived gene signature acts as an excellent predictor of patient outcomes and treatment responses in breast cancer.
  
  
  
  
**PRKG1**
-
4
of
10
  

### Identification of miRNAs and target genes associated with lymph node metastasis in cervical cancer using bioinformatics analysis.

4. Toxicol Mech Methods. 2023 Oct;33(8):625-635. doi: 10.1080/15376516.2023.2207644 Epub 2023 May 11.
  
  
Ding Y(1), Wu X(2), Yang X(3).
  
  
Author information: (1)Department of Gynecology, Ankang City Central Hospital, Ankang, PR China. (2) ...
  
  
This study was designed to identify the differentially expressed miRNAs (DEMs) and genes (DEGs) in metastatic cervical cancer using bioinformatic tools. In this study, fifty-seven DEMs (48 downregulated and 9 upregulated) were identified, among which miR-4459 and miR-3195 expression was negatively associated with overall survival of cervical cancer patients. Then, 476 target DEGs were determined, and protein-protein interaction (PPI) network was constructed. Seventeen hub genes (LONRF2, CCNE2, AURKA, SYT1, NEGR1, PPP1R12B, GABRP, RAD51, CDK1, FBLN5, PRKG1, CDC6, CACNA1C, MEOX2, ANLN, MYLK, and EDNRB) were finally selected to construct the miRNA-hub gene network. Overall, our study discovered the key miRNAs and mRNAs related to lymph node metastasis (LNM) in cervical cancer, which helps discover candidate therapeutic targets for cervical cancer.
  
  
  
  
**PRKG1**
-
5
of
10
  

### Molecular Subtyping in Muscle-Invasive Bladder Cancer on Predicting Survival and Response of Treatment.

5. Biomedicines. 2022 Dec 27;11(1):69. doi: 10.3390/biomedicines11010069
  
  
Bejrananda T(1), Saetang J(1)(2), Sangkhathat S(1)(3)(4).
  
  
Author information: (1)Department of Surgery, Faculty of Medicine, Prince of Songkla University, Son ...
  
  
Molecular classifications for urothelial bladder cancer appear to be promising in disease prognostication and prediction. This study investigated the novel molecular subtypes of muscle invasive bladder cancer (MIBC). Tumor samples and normal tissues of MIBC patients were submitted for transcriptome sequencing. Expression profiles were clustered using K-means clustering and principal component analysis. The molecular subtypes were also applied to The Cancer Genome Atlas (TCGA) dataset and analyzed for clinical outcome correlation. Three molecular subtypes of MIBC were discovered, clusters A, B, and C. The most differentially upregulated genes in cluster A were BDKRB1, EDNRA, AVPR1A, PDGFRB, and TNC, while the most upregulated genes in cluster C were collagen-related genes, PDGFRB, and PRKG1. For cluster B, COL6A3, COL1A2, COL6A2, tenascin C, and fibroblast growth factor 2 were statistically suppressed. When the centroids of clustering on PCA were applied to TCGA data, the clustering significantly predicted survival outcomes. Cluster B had the best overall survival (OS), and cluster C was associated with poor OS but exhibited the best response to perioperative chemotherapy. Among all groups, cluster B had a better pathologic response to neoadjuvant chemotherapy (40%). Based on the results of the present study, the novel clusters of subtype MIBC appear potentially suitable for integration into clinical practice.
  
  
  
  
**PRKG1**
-
6
of
10
  

### Downexpression of miR-200c-3p Contributes to Achalasia Disease by Targeting the PRKG1 Gene.

6. Int J Mol Sci. 2022 Dec 30;24(1):668. doi: 10.3390/ijms24010668
  
  
Micale L(1), Fusco C(1), Nardella G(1), Palmieri O(2), Latiano T(2), Gioffreda D(2), Tavano F(2), Pa ...
  
  
Author information: (1)Division of Medical Genetics, Fondazione IRCCS Casa Sollievo della Sofferenza ...
  
  
Achalasia is an esophageal smooth muscle motility disorder with unknown pathogenesis. Taking into account our previous results on the downexpression of miR-200c-3p in tissues of patients with achalasia correlated with an increased expression of PRKG1, SULF1, and SYDE1 genes, our aim was to explore the unknown biological interaction between these genes and human miR-200c-3p and if this relation could unravel their functional role in the etiology of achalasia. To search for putative miR-200c-3p binding sites in the 3'-UTR of PRKG1, SULF1 and SYDE1, a bioinformatics tool was used. To test whether PRKG1, SULF1, and SYDE1 are targeted by miR-200c-3p, a dual-luciferase reporter assay and quantitative PCR on HEK293 and fibroblast cell lines were performed. To explore the biological correlation between PRKG1 and miR-200c-3p, an immunoblot analysis was carried out. The overexpression of miR-200c-3p reduced the luciferase activity in cells transfected with a luciferase reporter containing a fragment of the 3'-UTR regions of PRKG1, SULF1, and SYDE1 which included the miR-200c-3p seed sequence. The deletion of the miR-200c-3p seed sequence from the 3'-UTR fragments abrogated this reduction. A negative correlation between miR-200c-3p and PRKG1, SULF1, and SYDE1 expression levels was observed. Finally, a reduction of the endogenous level of PRKG1 in cells overexpressing miR-200c-3p was detected. Our study provides, for the first time, functional evidence about the PRKG1 gene as a direct target and SULF1 and SYDE1 as potential indirect substrates of miR-200c-3p and suggests the involvement of NO/cGMP/PKG signaling in the pathogenesis of achalasia.
  
  
  
  
**PRKG1**
-
7
of
10
  

### lncRNAs UC.145 and PRKG1-AS1 Determine the Functional Output of DKK1 in Regulating the Wnt Signaling Pathway in Gastric Cancer.

7. Cancers (Basel). 2022 May 11;14(10):2369. doi: 10.3390/cancers14102369
  
  
Yoon JH(1), Byun H(1), Ivan C(2)(3), Calin GA(4), Jung D(1), Lee S(1).
  
  
Author information: (1)Division of Gastroenterology, Department of Internal Medicine, Institute of G ...
  
  
DKK1 inhibits the canonical Wnt signaling pathway that is known to be involved in various cancers. However, whether DKK1 acts as an oncogene or tumor suppressor gene remains controversial. Furthermore, the DKK1-regulating mechanism in gastric cancer has not yet been defined. The aim of this study was to explore whether the ultraconserved region UC.145 regulates epigenetic changes in DKK1 expression in gastric cancer. Microarray analysis revealed that UC.145 exhibited the highest binding affinity to EZH2, a histone methyltransferase. The effects of UC.145 inactivation were assessed in gastric cancer cell lines using siRNA. The results indicated that UC.145 triggers DKK1 methylation via interaction with EZH2 and is involved in the canonical Wnt signaling pathway. Additionally, interaction between UC.145 and another long non-coding RNA adjacent to DKK1, PRKG1-AS1, induced a synergistic effect on Wnt signaling. The regulation of these three genes was closely associated with patient overall survival. Inactivation of UC.145 induced apoptosis and inhibited the growth and migratory, invasive, and colony-forming abilities of gastric cancer cells. The study findings provide insights into Wnt signaling in gastric cancer and support UC.145 as a potential novel predictive biomarker for the disease.
  
  
  
  
**PRKG1**
-
8
of
10
  

### High HSPA8 expression predicts adverse outcomes of acute myeloid leukemia.

8. BMC Cancer. 2021 Apr 29;21(1):475. doi: 10.1186/s12885-021-08193-w
  
  
Li J(1), Ge Z(2)(3).
  
  
Author information: (1)Department of Hematology, Zhongda Hospita, Medical School of Southeast Univer ...
  
  
BACKGROUND: Acute myeloid leukemia (AML) remains one of the most common hematological malignancies, posing a serious challenge to human health. HSPA8 is a chaperone protein that facilitates proper protein folding. It contributes to various activities of cell function and also is associated with various types of cancers. To date, the role of HSPA8 in AML is still undetermined. METHODS: In this study, public datasets available from the TCGA (Cancer Genome Atlas) and GEO (Gene Expression Omnibus) were mined to discover the association between the expression of HSPA8 and clinical phenotypes of CN-AML. A series of bioinformatics analysis methods, including functional annotation and miRNA-mRNA regulation network analysis, were employed to investigate the role of HSPA8 in CN-AML. RESULTS: HSPA8 was highly expressed in the AML patients compared to the healthy controls. The high HSPA8 expression had lower overall survival (OS) rate than those with low HSPA8 expression. High expression of HSPA8 was also an independent prognostic factor for overall survival (OS) of CN-AML patients by multivariate analysis. The differential expressed genes (DEGs) associated with HSPA8 high expression were identified, and they were enriched PI3k-Akt signaling, cAMP signaling, calcium signaling pathway. HSPA8 high expression was also positively associated with micro-RNAs (hsa-mir-1269a, hsa-mir-508-3p, hsa-mir-203a), the micro-RNAs targeted genes (VSTM4, RHOB, HOBX7) and key known oncogenes (KLF5, RAN, and IDH1), and negatively associated with tumor suppressors (KLF12, PRKG1, TRPS1, NOTCH1, RORA). CONCLUSIONS: Our research revealed HSPA8 as a novel potential prognostic factor to predict the survival of CN-AML patients. Our data also revealed the possible carcinogenic mechanism and the complicated microRNA-mRNA network associated with the HSPA8 high expression in AML.
  
  
  
  
**PRKG1**
-
9
of
10
  

### Identification of hub lncRNAs in head and neck cancer based on weighted gene co-expression network analysis and experiments.

9. FEBS Open Bio. 2021 Jul;11(7):2060-2073. doi: 10.1002/2211-5463.13134 Epub 2021 May 21.
  
  
Lina S(1)(2).
  
  
Author information: (1)Department of Endodontics, School and Hospital of Stomatology, China Medical ...
  
  
Head and neck squamous cell carcinoma (HNSCC) ranks as the sixth most common cancer among systemic malignant tumors, with 600 000 new cases occurring every year worldwide. Since HNSCC has high heterogeneity and complex pathogenesis, no effective prognostic indicator has yet been identified. Here, we aimed to identify a lncRNA signature associated with the prognosis of HNSCC as a potential new biomarker. LncRNA expression data were downloaded from The Cancer Genome Atlas database. A polygenic risk score model was constructed by using Lasso-Cox regression analysis. Weighted gene co-expression network analysis (WGCNA) was applied to analyze the co-expression modules of lncRNAs associated with the prognosis of HNSCC. The robustness of the signature was validated in testing and external cohorts. Polymerase chain reaction was performed to detect the expression levels of identified lncRNAs in cancer and adjacent tissues. We constructed an 8-lncRNA signature (LINC00567, LINC00996, MTOR-AS1, PRKG1-AS1, RAB11B-AS1, RPS6KA2-AS1, SH3BP5-AS1, ZNF451-AS1) that could be used as an independent prognostic factor of HNSCC. The signature showed strong robustness and had stable prediction performance in different cohorts. WGCNA results showed that modules related to risk score mainly participated in biological processes such as blood vessel development, positive regulation of catabolic processes, and regulation of growth. The prognostic risk score model based on lncRNA for HNSCC may help clinicians conduct individualized treatment plans.
  
  
  
  
**PRKG1**
-
10
of
10
  

### The bivariate NRIP1/ZEB2 RNA marker permits non-invasive presymptomatic screening of pre-eclampsia.

10. Sci Rep. 2020 Dec 14;10(1):21857. doi: 10.1038/s41598-020-79008-4
  
  
Manders V(1)(2), Visser A(1), Keijser R(2), Min N(1)(2), Poutsma A(1), Mulders J(1), van den Berkmor ...
  
  
Author information: (1)Department of Clinical Chemistry, Amsterdam UMC, VU University Medical Center ...
  
  
Using genome-wide transcriptome analysis by RNA sequencing of first trimester plasma RNA, we tested whether the identification of pregnancies at risk of developing pre-eclampsia with or without preterm birth or growth restriction is possible between weeks 9-14, prior to the appearance of clinical symptoms. We implemented a metaheuristic approach in the self-learning SVM algorithm for differential gene expression analysis of normal pregnancies (n = 108), affected pregnancies (n = 34) and non-pregnant controls (n = 19). Presymptomatic candidate markers for affected pregnancies were validated by RT-qPCR in first trimester samples (n = 34) from an independent cohort. PRKG1 was significantly downregulated in a subset of pregnancies with birth weights below the 10thpercentile as shared symptom. The NRIP1/ZEB2 ratio was found to be upregulated in pregnancies with pre-eclampsia or trisomy 21. Complementary quantitative analysis of both the linear and circular forms of NRIP1 permitted discrimination between pre-eclampsia and trisomy 21. Pre-eclamptic pregnancies showed an increase in linear NRIP1 compared to circular NRIP1, while trisomy 21 pregnancies did not.
  
  
  
  
  

---

  

# RABGAP1L

**Signature gene in:** B cells, Dendritic cells, Plasma cells
  
**Differentially expressed in:** B cells
  
  
**RABGAP1L**
-
1
of
10
  

### A novel prognostic model based on telomere-related lncRNAs in gastric cancer.

1. Transl Cancer Res. 2024 Sep 30;13(9):4608-4624. doi: 10.21037/tcr-24-295 Epub 2024 Sep 27.
  
  
Ding X(1), Zhang Y(1), You S(1).
  
  
Author information: (1)Department of General Surgery, The Fourth Affiliated Hospital of Soochow Univ ...
  
  
BACKGROUND: Telomeres are specialized structures at the ends of chromosomes that are important for their protection. Over time, long non-coding RNAs (lncRNAs) have gradually come into the spotlight as essential biomarkers of proliferation, migration, and invasion of human malignant tumors. Nevertheless, the impact of telomere-related lncRNAs (TRLs) in gastric cancer is currently unknown. In the present study, we screen the TRLs and identify a prognostic TRLs signature in gastric cancer. METHODS: First, telomere-related genes (TRGs) were retrieved from the website, and RNA sequencing (RNA-seq) data and clinical data of stomach adenocarcinoma (STAD) patients were gathered from The Cancer Genome Atlas (TCGA) database. Gastric cancer patients' lncRNAs and overall survival (OS) were found to be related using univariate Cox regression analysis. Next, least absolute shrinkage and selection operator (LASSO) regression analysis and multifactorial Cox regression analysis were used to further screen telomere-related differentially expressed lncRNAs (TRDELs), and finally six lncRNAs were obtained, including LINC01537, CFAP61-AS1, DIRC1, RABGAP1L-IT1, DBH-AS1, and REPIN1-AS1. According to these six TRDELs, a prognostic model for gastric cancer was constructed. The samples were divided into the training group and the testing group at random, and the reliability of prognostic model was validated in both groups and overall samples. In addition, we performed Kaplan-Meier (K-M) survival curve analysis, independent prognostic analysis, and functional enrichment analysis to validate the predictive value and independence of the model, as well as immune cell correlation analysis, clustering analysis, and principal component analysis (PCA) to further explore the relationship between this model and the tumor cells. Finally, we performed the drug sensitivity analysis to identify a few small molecules that may have a therapeutic effect on gastric cancer. RESULTS: Finally, we constructed a prognostic model for gastric cancer consisting of six TRDELs. According to the K-M curve, the prognosis of the low-risk group was noticeably superior than that of the high-risk group. Multivariate Cox regression analysis suggested that risk score was an independent prognostic element. Receiver operating characteristic (ROC) curves, nomogram, and calibration curve indicated that the prognostic model had good predictive ability. Functional enrichment analysis demonstrated major pathways with high- and low-risk groups. Next, both tumor microenvironment (TME) and immune correlation analysis showed discrepancy in the high- and low-risk groups. Through drug sensitivity analysis, we screened four small molecules that might be beneficial for gastric cancer treatment. CONCLUSIONS: A prognostic model consisting of these six TRDELs was capable to predict the prognosis of gastric cancer patients.
  
  
  
  
**RABGAP1L**
-
2
of
10
  

### Machine learning with in silico analysis markedly improves survival prediction modeling in colon cancer patients.

2. Cancer Med. 2023 Mar;12(6):7603-7615. doi: 10.1002/cam4.5420 Epub 2022 Nov 7.
  
  
Lee CJ(1), Baek B(2), Cho SH(3), Jang TY(1), Jeon SE(1), Lee S(1), Lee H(2), Nam JS(1)(4).
  
  
Author information: (1)School of Life Sciences, Gwangju Institute of Science and Technology, Gwangju ...
  
  
BACKGROUND: Predicting the survival of cancer patients provides prognostic information and therapeutic guidance. However, improved prediction models are needed for use in diagnosis and treatment. OBJECTIVE: This study aimed to identify genomic prognostic biomarkers related to colon cancer (CC) based on computational data and to develop survival prediction models. METHODS: We performed machine-learning (ML) analysis to screen pathogenic survival-related driver genes related to patient prognosis by integrating copy number variation and gene expression data. Moreover, in silico system analysis was performed to clinically assess data from ML analysis, and we identified RABGAP1L, MYH9, and DRD4 as candidate genes. These three genes and tumor stages were used to generate survival prediction models. Moreover, the genes were validated by experimental and clinical analyses, and the theranostic application of the survival prediction models was assessed. RESULTS: RABGAP1L, MYH9, and DRD4 were identified as survival-related candidate genes by ML and in silico system analysis. The survival prediction model using the expression of the three genes showed higher predictive performance when applied to predict the prognosis of CC patients. A series of functional analyses revealed that each knockdown of three genes reduced the protumor activity of CC cells. In particular, validation with an independent cohort of CC patients confirmed that the coexpression of MYH9 and DRD4 gene expression reflected poorer clinical outcomes in terms of overall survival and disease-free survival. CONCLUSIONS: Our survival prediction approach will contribute to providing information on patients and developing a therapeutic strategy for CC patients.
  
  
  
  
**RABGAP1L**
-
3
of
10
  

### Identification and validation of a 9-gene signature for the prognosis of ovarian cancer by integrated bioinformatical analysis.

3. Ann Transl Med. 2022 Oct;10(19):1059. doi: 10.21037/atm-22-3752
  
  
Chen S(1), Yang M(1), Yang H(2), Tang Q(1), Gu C(3), Wei W(2).
  
  
Author information: (1)Department of Reproductive Medicine, Meizhou People's Hospital, Meizhou, Chin ...
  
  
BACKGROUND: Ovarian cancer (OC) is the most lethal malignancy among gynecological cancers worldwide. It is urgent to identify effective biomarkers for the prognosis and diagnosis of OC. METHODS: We analyzed 4 OC Gene Expression Omnibus (GEO) data sets to detect differentially expressed genes (DEGs). To explore potential correlations between the gene sets and clinical features, we conducted weighted gene co-expression network analysis (WGCNA). Hub genes were identified from the key modules by univariate Cox regression, least absolute shrinkage and selection operator (LASSO), and multivariate Cox regression analyses and risk scores were calculated based on the expressions of the hub genes. Univariate and multivariate Cox regression analyses were conducted to determine the values of the diagnoses for OC patients. We also determined the predictive value of the long non-coding RNA (lncRNA) score in response to immunotherapy and chemotherapeutic drugs. RESULTS: DEGs were analyzed between the OC and normal ovarian tissues and prognostic modules were identified by a WGCNA. Nine hub genes chose from the prognostic modules were determined the prognostic values in OC. The risk scores were calculated based on the expression of hub genes, and patients with high-risk scores had poor survival. Univariate and multivariate Cox regression analyses showed that the risk score was an independent prognostic factor for OC. Additionally, the levels of hub genes were also found to be related to immune cell inﬁltration in OC microenvironments. An immunotherapy cohort showed that high-risk scores enhanced the response to anti-programmed death-ligand 1 (PD-L1) immunotherapy and was remarkably correlated with the inflamed immune phenotype, and had significant therapeutic advantages and clinical benefits. Further, patients with high-risk scores were more sensitive to midostaurin. CONCLUSIONS: We identiﬁed the risk score including protein phosphatase, Mg2+/Mn2+ dependent 1K (PPM1K), protein phosphatase 1 catalytic subunit alpha (PPP1CA), exostosin glycosyltransferase 1 (EXT1), RAB GTPase activating protein 1 like (RABGAP1L), mitotic arrest deficient 2 like 1 (MAD2L1), xeroderma pigmentosum complementation group C (XPC), Egl-9 family hypoxia inducible factor 3 (EGLN3), cyclin D1 binding protein 1 (CCNDBP1), and zinc finger protein 25 (ZNF25), and validated their prognostic and predicted values for OC.
  
  
  
  
**RABGAP1L**
-
4
of
10
  

### Dysregulation of Circulatory Levels of lncRNAs in Parkinson's Disease.

4. Mol Neurobiol. 2023 Jan;60(1):317-328. doi: 10.1007/s12035-022-03086-w Epub 2022 Oct 20.
  
  
Huang T(1), Zhao JY(#)(1), Pan RR(#)(1), Jiang T(#)(1), Fu XX(2), Huang Q(1), Wang XX(1), Gong PY(1) ...
  
  
Author information: (1)Department of Neurology, Nanjing First Hospital, Nanjing Medical University, ...
  
  
Emerging evidence suggested that long non-coding RNAs (lncRNAs) were involved in Parkinson's disease (PD) pathogenesis. Herein, we used gene expression profiles from GEO database to construct a PD-specific ceRNA network. Functional enrichment analysis suggested that ceRNA network might participate in the development of PD. PPI networks were constructed, and the ceRNA subnetwork based on five hub genes was set up. In a cohort of 32 PD patients and 31 healthy controls, the expression of 10 DElncRNAs (TTC3-AS1, LINC01259, ZMYND10-AS1, CHRM3-AS1, MYO16-AS1, AGBL5-IT1, HOTAIRM1, RABGAP1L-IT1, HLCS-IT1, and LINC00393) were further verified. Consistent with the microarray data, LINC01259 expression was significantly lower in PD patients compared with controls (P = 0.008). Intriguingly, such a difference was only observed among male patients and male controls when dividing study participants based on their gender (P = 0.016). However, the expression of other lncRNAs did not differ significantly between the two groups. Receiver operating characteristic (ROC) curve analysis revealed that the diagnostic power of LINC01259 was 0.694 for PD and 0.677 for early-stage PD. GSEA enrichment analysis revealed that LINC01259 was mainly enriched in biological processes associated with immune function and inflammatory response. Moreover, LINC01259 expression was not correlated with age of patients, disease duration, disease stage, MDS-UPDRS score, MDS-UPDRS III score, MMSE score, and MOCA score. The current study provides further evidence for the dysregulation of lncRNAs in circulating leukocytes of PD patients, revealing that LINC01259 has clinical potential as a novel immune and inflammatory biomarker for PD and early-stage PD diagnosis.
  
  
  
  
**RABGAP1L**
-
5
of
10
  

### Circulatory Endothelin 1-Regulating RNAs Panel: Promising Biomarkers for Non-Invasive NAFLD/NASH Diagnosis and Stratification: Clinical and Molecular Pilot Study.

5. Genes (Basel). 2021 Nov 18;12(11):1813. doi: 10.3390/genes12111813
  
  
Albadawy R(1), Agwa SHA(2), Khairy E(3), Saad M(4), El Touchy N(1), Othman M(5), El Kassas M(6), Mat ...
  
  
Author information: (1)Gastroentrology, Hepatology & Infectious Disease Department, Faculty of Medic ...
  
  
Nonalcoholic fatty liver disease (NAFLD) is one of the major seeds of liver cirrhosis and hepatocellular carcinoma. There is no convenient reliable non-invasive early diagnostic tool available for NAFLD/NASH diagnosis and stratification. Recently, the role of cytosolic sensor, stimulator of interferon genes (STING) signaling pathway in pathogenesis of nonalcoholic steatohepatitis (NASH) has been evidenced in research. We have selected EDN1/TNF/MAPK3/EP300/hsa-miR-6888-5p/lncRNA RABGAP1L-DT-206 RNA panel from bioinformatics microarrays databases related to STING pathway and NAFLD/NASH pathogenesis. We have used reverse-transcriptase real-time polymerase chain reaction to assess the expression of the serum RNAs panel in NAFLD/NASH without suspicion of advanced fibrosis, NAFLD/with NASH patients with suspicion of advanced fibrosis and controls. Additionally, we have assessed the diagnostic performance of the Ribonucleic acid (RNA) panel. We have detected upregulation of the EDN1 regulating RNAs panel expression in NAFLD/NASH cases compared to healthy controls. We concluded that this circulatory RNA panel could enable us to discriminate NAFLD/NASH cases from controls, and also NAFLD/NASH cases (F1, F2) from advanced fibrosis stages (F3, F4).
  
  
  
  
**RABGAP1L**
-
6
of
10
  

### The oncogene AAMDC links PI3K-AKT-mTOR signaling with metabolic reprograming in estrogen receptor-positive breast cancer.

6. Nat Commun. 2021 Mar 26;12(1):1920. doi: 10.1038/s41467-021-22101-7
  
  
Golden E(#)(1)(2), Rashwan R(#)(1)(2)(3), Woodward EA(#)(1)(2), Sgro A(1)(2)(4), Wang E(1)(2), Sorol ...
  
  
Author information: (1)Cancer Epigenetics Group, The Harry Perkins Institute of Medical Research, Th ...
  
  
Adipogenesis associated Mth938 domain containing (AAMDC) represents an uncharacterized oncogene amplified in aggressive estrogen receptor-positive breast cancers. We uncover that AAMDC regulates the expression of several metabolic enzymes involved in the one-carbon folate and methionine cycles, and lipid metabolism. We show that AAMDC controls PI3K-AKT-mTOR signaling, regulating the translation of ATF4 and MYC and modulating the transcriptional activity of AAMDC-dependent promoters. High AAMDC expression is associated with sensitization to dactolisib and everolimus, and these PI3K-mTOR inhibitors exhibit synergistic interactions with anti-estrogens in IntClust2 models. Ectopic AAMDC expression is sufficient to activate AKT signaling, resulting in estrogen-independent tumor growth. Thus, AAMDC-overexpressing tumors may be sensitive to PI3K-mTORC1 blockers in combination with anti-estrogens. Lastly, we provide evidence that AAMDC can interact with the RabGTPase-activating protein RABGAP1L, and that AAMDC, RABGAP1L, and Rab7a colocalize in endolysosomes. The discovery of the RabGAP1L-AAMDC assembly platform provides insights for the design of selective blockers to target malignancies having the AAMDC amplification.
  
  
  
  
**RABGAP1L**
-
7
of
10
  

### Development of small-molecule tropomyosin receptor kinase (TRK) inhibitors for NTRK fusion cancers.

7. Acta Pharm Sin B. 2021 Feb;11(2):355-372. doi: 10.1016/j.apsb.2020.05.004 Epub 2020 May 23.
  
  
Jiang T(1), Wang G(1), Liu Y(1), Feng L(1), Wang M(1), Liu J(1), Chen Y(2), Ouyang L(1).
  
  
Author information: (1)State Key Laboratory of Biotherapy and Cancer Center, West China Hospital, an ...
  
  
Erratum in Acta Pharm Sin B. 2022 Jun;12(6):2963-2964. doi: 10.1016/j.apsb.2022.02.028.
  
  
  
  
**RABGAP1L**
-
8
of
10
  

### High-resolution 400K oligonucleotide array comparative genomic hybridization analysis of neurofibromatosis type 1-associated cutaneous neurofibromas.

8. Gene. 2015 Mar 10;558(2):220-6. doi: 10.1016/j.gene.2014.12.064 Epub 2015 Jan 3.
  
  
Asai A(1), Karnan S(2), Ota A(3), Takahashi M(4), Damdindorj L(2), Konishi Y(2), Hossain E(2), Konis ...
  
  
Author information: (1)Department of Biochemistry, Aichi Medical University School of Medicine, Naga ...
  
  
Neurofibromatosis type 1 (NF1) is a genetic disorder where affected individuals develop benign or malignant nervous system tumors. To date, NF1 is caused by mutations in the NF1 tumor suppressor gene located at chromosome band 17q11.2. In this study, we aimed to characterize novel recurrent regional chromosomal imbalances and tumor-related candidate genes in NF1-associated cutaneous neurofibromas. Nine cutaneous neurofibromas from NF1 patients were screened for recurrent chromosomal imbalances using high-resolution 400K oligonucleotide array comparative genomic hybridization (aCGH). All the cases exhibited at least one sub-microscopic abnormality. Regions of recurrent chromosomal imbalances in a least one third of cases were loss of 1q13.2 (33%, FAM19A3), 1q21.1 (44%, RABGAP1L), 2q37.1 (56%, INPP5D), 3p25.1 (67%, CHCHD4), 4p15.32 (56%, FGFBP1), 5q11.2 (56%, ARL15), 6q22.31 (56%, NKAIN2), 6q22.33 (67%, ARHGAP18), 6q25.1 (67%, UST), 7q13 (56%, ADCY1), 12q13.13 (44%, KRT71), 19q13.32 (56%, GRLF1), and 20p11.21 (56%, NLP) and gain of 2p23.3 (76%, C2orf53), 8q22.3 (44%, ODF1) and 8q24.3 (67%, ARC). Several chromosomal imbalances, including loss of 7q11.23, 13q14.1, 14q32.13, 17p12, and 17q11.2 were detected at a lower frequency. We also confirmed that these chromosomal imbalances were not detected in the patient-matched lymphocyte DNAs. Amongst the 6 tumor-related candidate genes (RABGAP1L, ADCY1, SLIT2, GRLF1, UST, and ARC) identified in the regions of recurrent chromosomal imbalances, the gene expression changes of UST (down-regulation) and ARC (up-regulation) were found to be significantly associated with copy number alterations. The novel recurrent chromosomal imbalances and the altered expression levels of the tumor-related candidate genes may be associated with the development of NF1-associated benign cutaneous neurofibromas.
  
  
  
  
**RABGAP1L**
-
9
of
10
  

### New routes to targeted therapy of intrahepatic cholangiocarcinomas revealed by next-generation sequencing.

9. Oncologist. 2014 Mar;19(3):235-42. doi: 10.1634/theoncologist.2013-0352 Epub 2014 Feb 21.
  
  
Ross JS(1), Wang K, Gay L, Al-Rohil R, Rand JV, Jones DM, Lee HJ, Sheehan CE, Otto GA, Palmer G, Yel ...
  
  
Author information: (1)Department of Pathology and Laboratory Medicine, Albany Medical College, Alba ...
  
  
BACKGROUND: Intrahepatic cholangiocarcinoma (ICC) is a subtype of primary liver cancer that is rarely curable by surgery and is rapidly increasing in incidence. Relapsed ICC has a poor prognosis, and current systemic nontargeted therapies are commonly extrapolated from those used in other gastrointestinal malignancies. We hypothesized that genomic profiling of clinical ICC samples would identify genomic alterations that are linked to targeted therapies and that could facilitate a personalized approach to therapy. METHODS: DNA sequencing of hybridization-captured libraries was performed for 3,320 exons of 182 cancer-related genes and 36 introns of 14 genes frequently rearranged in cancer. Sample DNA was isolated from 40 μm of 28 formalin-fixed paraffin-embedded ICC specimens and sequenced to high coverage. RESULTS: The most commonly observed alterations were within ARID1A (36%), IDH1/2 (36%), and TP53 (36%) as well as amplification of MCL1 (21%). Twenty cases (71%) harbored at least one potentially actionable alteration, including FGFR2 (14%), KRAS (11%), PTEN (11%), CDKN2A (7%), CDK6 (7%), ERBB3 (7%), MET (7%), NRAS (7%), BRCA1 (4%), BRCA2 (4%), NF1 (4%), PIK3CA (4%), PTCH1 (4%), and TSC1 (4%). Four (14%) of the ICC cases featured novel gene fusions involving the tyrosine kinases FGFR2 and NTRK1 (FGFR2-KIAA1598, FGFR2-BICC1, FGFR2-TACC3, and RABGAP1L-NTRK1). CONCLUSION: Two thirds of patients in this study harbored genomic alterations that are associated with targeted therapies and that have the potential to personalize therapy selection for to individual patients.
  
  
  
  
**RABGAP1L**
-
10
of
10
  

### RABGAP1L gene rearrangement resulting from a der(Y)t(Y;1)(q12;q25) in acute myeloid leukemia arising in a child with Klinefelter syndrome.

10. Virchows Arch. 2009 Mar;454(3):311-6. doi: 10.1007/s00428-009-0732-z Epub 2009 Jan 28.
  
  
Roberti MC(1), La Starza R, Surace C, Sirleto P, Pinto RM, Pierini V, Crescenzi B, Mecucci C, Angion ...
  
  
Author information: (1)Cytogenetics and Molecular Genetics, Bambino Gesù Children's Hospital, Piazza ...
  
  
In this study, we report the molecular cytogenetic characterization of an acute myeloid leukemia with a der(Y)t(Y;1)(q12;q25) in bone marrow cells in a child with Klinefelter syndrome. Conventional cytogenetics demonstrated the unbalanced translocation, i.e., a trisomic 1q25-qter juxtaposed to Yq12 replaced the terminal segment of chromosome Y was acquired and present only on bone marrow cells. Fluorescence in situ hybridization showed that the breakpoint at 1q25 disrupted RABGAP1L, a strongly expressed gene in CFU-GEMM, erythroid cells, and megakaryocytes, while the Yq12 breakpoint fell within the heterochromatic region. As der(Y)t(Y;1)(q12;q25) was an isolated cytogenetic change, RABGAP1L rearrangement as well as gene(s) dosage effects correlated to 1q25-qter trisomy, and Yq12-qter loss may make a major contribution to leukemogenesis and/or disease progression.
  
  
  
  
  

---

  

# RHBDF2

**Signature gene in:** MDM
  
**Differentially expressed in:** MDM
  
  
**RHBDF2**
-
1
of
10
  

### Identification of novel candidate predisposing genes in familial nonmedullary thyroid carcinoma implicating DNA damage repair pathways.

1. Int J Cancer. 2025 Jan 1;156(1):130-144. doi: 10.1002/ijc.35159 Epub 2024 Sep 9.
  
  
Pires C(1)(2), Marques IJ(1)(2), Saramago A(1), Moura MM(1), Pojo M(1), Cabrera R(3), Santos C(4), R ...
  
  
Author information: (1)Unidade de Investigação em Patobiologia Molecular, Instituto Português de Onc ...
  
  
The genetic basis of nonsyndromic familial nonmedullary thyroid carcinoma (FNMTC) is still poorly understood, as the susceptibility genes identified so far only account for a small percentage of the genetic burden. Recently, germline mutations in DNA repair-related genes have been reported in cases with thyroid cancer. In order to clarify the genetic basis of FNMTC, 94 genes involved in hereditary cancer predisposition, including DNA repair genes, were analyzed in 48 probands from FNMTC families, through targeted next-generation sequencing (NGS). Genetic variants were selected upon bioinformatics analysis and in silico studies. Structural modeling and network analysis were also performed. In silico results of NGS data unveiled likely pathogenic germline variants in 15 families with FNMTC, in genes encoding proteins involved in DNA repair (ATM, CHEK2, ERCC2, BRCA2, ERCC4, FANCA, FANCD2, FANCF, and PALB2) and in the DICER1, FLCN, PTCH1, BUB1B, and RHBDF2 genes. Structural modeling predicted that most missense variants resulted in the disruption of networks of interactions between residues, with implications for local secondary and tertiary structure elements. Functional annotation and network analyses showed that the involved DNA repair proteins functionally interact with each other, within the same DNA repair pathway and across different pathways. MAPK activation was a common event in tumor progression. This study supports that rare germline variants in DNA repair genes may be accountable for FNMTC susceptibility, with potential future utility in patients' clinical management, and reinforces the relevance of DICER1 in disease etiology.
  
  
  
  
**RHBDF2**
-
2
of
10
  

### Cryo-EM reveals that iRhom2 restrains ADAM17 protease activity to control the release of growth factor and inflammatory signals.

2. Mol Cell. 2024 Jun 6;84(11):2152-2165.e5. doi: 10.1016/j.molcel.2024.04.025 Epub 2024 May 22.
  
  
Lu F(1), Zhao H(2), Dai Y(3), Wang Y(3), Lee CH(4), Freeman M(5).
  
  
Author information: (1)Sir William Dunn School of Pathology, University of Oxford, South Parks Road, ...
  
  
A disintegrin and metalloprotease 17 (ADAM17) is a membrane-tethered protease that triggers multiple signaling pathways. It releases active forms of the primary inflammatory cytokine tumor necrosis factor (TNF) and cancer-implicated epidermal growth factor (EGF) family growth factors. iRhom2, a rhomboid-like, membrane-embedded pseudoprotease, is an essential cofactor of ADAM17. Here, we present cryoelectron microscopy (cryo-EM) structures of the human ADAM17/iRhom2 complex in both inactive and active states. These reveal three regulatory mechanisms. First, exploiting the rhomboid-like hallmark of TMD recognition, iRhom2 interacts with the ADAM17 TMD to promote ADAM17 trafficking and enzyme maturation. Second, a unique iRhom2 extracellular domain unexpectedly retains the cleaved ADAM17 inhibitory prodomain, safeguarding against premature activation and dysregulated proteolysis. Finally, loss of the prodomain from the complex mobilizes the ADAM17 protease domain, contributing to its ability to engage substrates. Our results reveal how a rhomboid-like pseudoprotease has been repurposed during evolution to regulate a potent membrane-tethered enzyme, ADAM17, ensuring the fidelity of inflammatory and growth factor signaling.
  
  
  
  
**RHBDF2**
-
3
of
10
  

### Increased Expression of Inactive Rhomboid Protein 2 in Circulating Monocytes after Acute Myocardial Infarction.

3. J Cardiovasc Transl Res. 2024 Oct;17(5):1059-1066. doi: 10.1007/s12265-024-10519-5 Epub 2024 May 14.
  
  
van Dijck P(1), Hannemann C(1)(2)(3), Dreger H(2)(4)(5), Stangl V(1)(2), Stangl K(1)(2), Ludwig A(1) ...
  
  
Author information: (1)Department of Cardiology, Angiology and Intensive Care Medicine, Campus Mitte ...
  
  
Increased TNF-α levels following acute myocardial infarction (AMI) contribute to impaired recovery of myocardial function. Interaction of inactive rhomboid protein 2 (iRhom2) with TNF-α converting enzyme (TACE) is required for TNF-α shedding from immune cells. We hypothesized that iRhom2 expression increases in circulating monocytes following AMI. Transcript levels of iRhom2, TACE and TNF-α were evaluated by quantitative real-time PCR in isolated monocytes of 50 AMI patients at admission (d1) and 3 days (d3) after. We observed a significant increase in levels of iRhom2 mRNA expression in monocytes between d1-3, while TNF-α and TACE mRNA expression remained unchanged. At d3, iRhom2 mRNA expression positively correlated with levels of intermediate monocytes or serum TNF-α, and negatively with LV systolic function. iRhom2 may contribute to regulation of post-infarction inflammation and is associated with LV dysfunction following AMI. iRhom2 modulation should be evaluated as a potential therapeutic strategy to attenuate cardiac remodeling following AMI.
  
  
  
  
**RHBDF2**
-
4
of
10
  

### DNA methylation: The epigenetic mechanism of Alzheimer's disease.

4. Ibrain. 2023 Aug 10;9(4):463-472. doi: 10.1002/ibra.12121 eCollection 2023 Winter.
  
  
Qin HY(1), Liu JY(1), Fang CL(2), Deng YP(1), Zhang Y(3).
  
  
Author information: (1)Department of Anesthesiology Southwest Medical University Luzhou Sichuan Chin ...
  
  
Nowadays, with the development of the social health care system, there is an increasing trend towards an aging society. The incidence of Alzheimer's disease (AD) is also on the rise. AD is a kind of neurodegenerative disease that can be found in any age group. For years, scientists have been committing to discovering the cause of AD. DNA methylation is one of the most common epigenetic mechanisms in mammals and plays a vital role in the pathogenesis of several diseases, including tumors. Studying chemical changes in the epigenome, or DNA methylation can help us understand the effects of our environment and life on diseases, such as smoking, depression, and menopause, which may affect people's chances of developing Alzheimer's or other diseases. Recent studies have identified some crucial genes like ANK1, RHBDF2, ABCA7, and BIN1, linking DNA methylation to AD. This review focuses on elucidating the relationship between DNA methylation and the pathogenesis of AD and provides an outlook on possible targeted therapeutic modalities.
  
  
  
  
**RHBDF2**
-
5
of
10
  

### iRhom2 regulates ectodomain shedding and surface expression of the major histocompatibility complex (MHC) class I.

5. Cell Mol Life Sci. 2024 Apr 4;81(1):163. doi: 10.1007/s00018-024-05201-7
  
  
Calligaris M(1)(2), Spanò DP(1)(3), Bonelli S(1)(3), Müller SA(4)(5), Carcione C(6), D'apolito D(6), ...
  
  
Author information: (1)Department of Research IRCCS ISMETT (Istituto Mediterraneo per i Trapianti e ...
  
  
Proteolytic release of transmembrane proteins from the cell surface, the so called ectodomain shedding, is a key process in inflammation. Inactive rhomboid 2 (iRhom2) plays a crucial role in this context, in that it guides maturation and function of the sheddase ADAM17 (a disintegrin and metalloproteinase 17) in immune cells, and, ultimately, its ability to release inflammatory mediators such as tumor necrosis factor α (TNFα). Yet, the macrophage sheddome of iRhom2/ADAM17, which is the collection of substrates that are released by the proteolytic complex, is only partly known. In this study, we applied high-resolution proteomics to murine and human iRhom2-deficient macrophages for a systematic identification of substrates, and therefore functions, of the iRhom2/ADAM17 proteolytic complex. We found that iRhom2 loss suppressed the release of a group of transmembrane proteins, including known (e.g. CSF1R) and putative novel ADAM17 substrates. In the latter group, shedding of major histocompatibility complex class I molecules (MHC-I) was consistently reduced in both murine and human macrophages when iRhom2 was ablated. Intriguingly, it emerged that in addition to its shedding, iRhom2 could also control surface expression of MHC-I by an undefined mechanism. We have demonstrated the biological significance of this process by using an in vitro model of CD8+ T-cell (CTL) activation. In this model, iRhom2 loss and consequent reduction of MHC-I expression on the cell surface of an Epstein-Barr virus (EBV)-transformed lymphoblastoid cell line dampened activation of autologous CTLs and their cell-mediated cytotoxicity. Taken together, this study uncovers a new role for iRhom2 in controlling cell surface levels of MHC-I by a dual mechanism that involves regulation of their surface expression and ectodomain shedding.
  
  
  
  
**RHBDF2**
-
6
of
10
  

### Mechanistic insight on the role of iRhom2-TNF-α-BAFF signaling pathway in various autoimmune disorders.

6. Adv Biol Regul. 2024 May;92:101011. doi: 10.1016/j.jbior.2023.101011 Epub 2023 Dec 14.
  
  
Dwivedi SD(1), Shukla R(1), Yadav K(2), Rathor LS(1), Singh D(1), Singh MR(3).
  
  
Author information: (1)University Institute of Pharmacy, Pt. Ravishankar Shukla University, Raipur ( ...
  
  
iRhom2 is a crucial cofactor involved in upregulation of TNF receptors (TNFRs) and the pro-inflammatory cytokine tumor necrosis factor (TNF-) from the cell surface by ADAM17. Tumor necrosis factor- α converting enzyme (TACE) is another name given to ADAM17. Many membrane attached biologically active molecules are cleaved by this enzyme which includes TNFRs and the pro-inflammatory cytokine tumor necrosis factor- α. The TNF receptors are of two types TNFR1 and TNFR2. iRhom2 belongs to the pseudo-protease class of rhomboid family, its abundance is observed in the immune cells. Biological activity of ADAM17 is affected in multiple levels by the iRhom2. ADAM17 is trafficked into the Golgi apparatus by the action of iRhom2, where it gets matured proteolytically and is stimulated to perform its function on the cell surface. This process of activation of ADAM17 results in the protection of the organism from the cascade of inflammatory reactions, as this activation blocks the TNF- α mediated secretion responsible for inflammatory responses produced. Present paper illustrates about the iRhom2-TNF-α-BAFF signaling pathway and its correlation with several autoimmune disorders such as Rheumatoid Arthritis, Systemic Lupus Erythematosus, Hemophilia Arthropathy, Alzheimer's disease and Tylosis with esophageal cancer etc.
  
  
  
  
**RHBDF2**
-
7
of
10
  

### Analysis of the function of ADAM17 in iRhom2 curly-bare and tylosis with esophageal cancer mutant mice.

7. J Cell Sci. 2023 Jul 1;136(13):jcs260910. doi: 10.1242/jcs.260910 Epub 2023 Jul 7.
  
  
Rabinowitsch AI(1)(2), Maretzky T(3), Weskamp G(4), Haxaire C(4), Tueshaus J(5)(6)(7), Lichtenthaler ...
  
  
Author information: (1)Tri-Institutional MD/PhD Program, Memorial Sloan Kettering Cancer Center, Wei ...
  
  
Tylosis with oesophageal cancer (TOC) is a rare familial disorder caused by cytoplasmic mutations in inactive rhomboid 2 (iRhom2 or iR2, encoded by RHBDF2). iR2 and the related iRhom1 (or iR1, encoded by Rhbdf1) are key regulators of the membrane-anchored metalloprotease ADAM17, which is required for activating EGFR ligands and for releasing pro-inflammatory cytokines such as TNFα (or TNF). A cytoplasmic deletion in iR2, including the TOC site, leads to curly coat or bare skin (cub) in mice, whereas a knock-in TOC mutation (toc) causes less severe alopecia and wavy fur. The abnormal skin and hair phenotypes of iR2cub/cub and iR2toc/toc mice depend on amphiregulin (Areg) and Adam17, as loss of one allele of either gene rescues the fur phenotypes. Remarkably, we found that iR1-/- iR2cub/cub mice survived, despite a lack of mature ADAM17, whereas iR2cub/cub Adam17-/- mice died perinatally, suggesting that the iR2cub gain-of-function mutation requires the presence of ADAM17, but not its catalytic activity. The iR2toc mutation did not substantially reduce the levels of mature ADAM17, but instead affected its function in a substrate-selective manner. Our findings provide new insights into the role of the cytoplasmic domain of iR2 in vivo, with implications for the treatment of TOC patients.
  
  
  
  
**RHBDF2**
-
8
of
10
  

### RHBDF2 is correlated with immune infiltrates in hepatocellular carcinoma and may have potential as a biomarker.

8. FEBS Open Bio. 2023 May;13(5):881-897. doi: 10.1002/2211-5463.13598 Epub 2023 Apr 5.
  
  
Gong H(1), Xie H(1), Huangfu Z(1), Zhang S(1), Tang Y(1), Xiao M(1), Li M(1), Wang Y(1).
  
  
Author information: (1)Department of Pathology, Molecular Medicine and Cancer Research Center, Basic ...
  
  
Immune checkpoint inhibitors have been approved for the treatment of advanced hepatocellular carcinoma (HCC). However, immunotherapy requires the identification of suitable biomarkers to guide treatment. The gene for rhomboid 5 homolog 2 (IRHom2), which encodes the rhombus protease iRhom2, activates the MAP3K7-dependent pathway and promotes hepatic steatosis. Thus, we hypothesized an involvement of this gene in HCC. We report that RHBDF2 expression is dramatically upregulated in HCC. RHBDF2 upregulation is associated with tumor stage, lymph node metastasis, tumor protein P53 mutation, and worse prognoses in HCC patients. Gene Ontology, Kyoto Encyclopedia of Genes and Genomes and gene set enrichment analysis enrichment analysis indicated that RHBDF2 was involved in immune signal pathways. Moreover, RHBDF2 expression was positively related not only to immune infiltration, but also to immune cell markers. Examination of several types of HCC infiltrated by immune cells revealed that the group with high expression of RHBDF2 showed the worst outcomes. Therefore, RHBDF2 may have potential as a novel biomarker for predicting prognosis and is related to immune infiltrates in HCC.
  
  
  
  
**RHBDF2**
-
9
of
10
  

### The prognostic value of RHBDF2 in Pan-Cancer, and its correlation with cell Adhesion of Hepatocellular Carcinoma.

9. Biotechnol Genet Eng Rev. 2024 Oct;40(2):1024-1048. doi: 10.1080/02648725.2023.2191092 Epub 2023 Mar 21.
  
  
Gong H(1), Zhang Y(2), Chen X(1), Cao X(1), Tang L(2), Wang Y(1).
  
  
Author information: (1)Department of Pathology, Molecular Medicine and Cancer Research Center, Basic ...
  
  
The impact of RHBDF2 on the expression and potential function in many cancers is still unknown. Therefore, the expression and methylation modification of RHBDF2 were evaluated across TCGA cancers in this study. Moreover, two methods, COX regression and Kaplan-Meier, were utilized for analyses of the prognoses of RHBDF2 in patients. Besides, the association between RHBDF2 and immune microenvironment, mutation, tumor mutation burden and microsatellite instability was analyzed with Pearson correlation. We verified RHBDF2 expression in hepatocellular carcinoma (HCC) compared with normal cell and tissue samples, detected the effects of RHBDF2 knockdown on biological functions in HCC cells, and detected CD4, CD8 and CD68 expression in hepatocellular carcinoma tissues and paired normal tissues. Given these results, the significant mRNA overexpression and promoter hypomethylation of RHBDF2 in various tumor types was showed, and a clear relationship between RHBDF2 overexpression and unfavourable overall survival and progression-free survival was observed, including liver hepatocellular carcinoma (LIHC), glioma (GBMLGG) and pancreatic adenocarcinoma (PAAD). Additionally, hypomethylation of RHBDF2 can affect the overall survival in some tumors. Furthermore, a clear correlation between RHBDF2 and infiltration of immune cells, immune-related molecules, TMB and MSI was observed. Besides, RHBDF2 expression is upregulated in HCC cells and tissues, and RHBDF2 knockdown could decrease the cell adhesion ability of HCC cells. More importantly, the expression of CD4, CD8 and CD68 was higher in HCC tissues. Altogether, the research denoted that RHBDF2 can be a prognostic biomarker for cancers according to these results and participate in cell adhesion of HCC cells.
  
  
  
  
**RHBDF2**
-
10
of
10
  

### Investigating isoform switching in RHBDF2 and its role in neoplastic growth in breast cancer.

10. PeerJ. 2022 Nov 25;10:e14124. doi: 10.7717/peerj.14124 eCollection 2022.
  
  
Masood M(1)(2), Masood MBE(1), Us Subah N(1), Shabbir M(3), Paracha RZ(1), Rafiq M(1).
  
  
Author information: (1)School of Interdisciplinary Engineering and Sciences, National University of ...
  
  
BACKGROUND: Breast cancer is the second leading cause of cancer-related deaths globally, and its prevalence rates are increasing daily. In the past, studies predicting therapeutic drug targets for cancer therapy focused on the assumption that one gene is responsible for producing one protein. Therefore, there is always an immense need to find promising and novel anti-cancer drug targets. Furthermore, proteases have an integral role in cell proliferation and growth because the proteolysis mechanism is an irreversible process that aids in regulating cellular growth during tumorigenesis. Therefore, an inactive rhomboid protease known as iRhom2 encoded by the gene RHBDF2 can be considered an important target for cancer treatment. Speculatively, previous studies on gene expression analysis of RHBDF2 showed heterogenous behaviour during tumorigenesis. Consistent with this, several studies have reported the antagonistic role of iRhom2 in tumorigenesis, i.e., either they are involved in negative regulation of EGFR ligands via the ERAD pathway or positively regulate EGFR ligands via the EGFR signalling pathway. Additionally, different opinions suggest iRhom2 mediated cleavage of EGFR ligands takes place TACE dependently or TACE independently. However, reconciling these seemingly opposing roles is still unclear and might be attributed to more than one transcript isoform of iRhom2. METHODS: To observe the differences at isoform resolution, the current strategy identified isoform switching in RHBDF2 via differential transcript usage using RNA-seq data during breast cancer initiation and progression. Furthermore, interacting partners were found via correlation and enriched to explain their antagonistic role. RESULTS: Isoform switching was observed at DCIS, grade 2 and grade 3, from canonical to the cub isoform. Neither EGFR nor ERAD was found enriched. However, pathways leading to TACE-dependent EGFR signalling pathways were more observant, specifically MAPK signalling pathways, GPCR signalling pathways, and toll-like receptor pathways. Nevertheless, it was noteworthy that during CTCs, the cub isoform switches back to the canonical isoform, and the proteasomal degradation pathway and cytoplasmic ribosomal protein pathways were significantly enriched. Therefore, it could be inferred that cub isoform functions during cancer initiation in EGFR signalling. In contrast, during metastasis, where invasion is the primary task, the isoform switches back to the canonical isoform.
  
  
  
  
  

---

  

# RIC8B

**Signature gene in:** Stromal cells
  
**Differentially expressed in:** Stromal cells
  
  
**RIC8B**
-
1
of
4
  

### Elp3-mediated codon-dependent translation promotes mTORC2 activation and regulates macrophage polarization.

1. EMBO J. 2022 Sep 15;41(18):e109353. doi: 10.15252/embj.2021109353 Epub 2022 Aug 3.
  
  
Chen D(1)(2)(3), Nemazanyy I(4), Peulen O(1)(5), Shostak K(1)(2)(3), Xu X(1)(2)(3), Tang SC(1)(2)(3) ...
  
  
Author information: (1)Interdisciplinary Cluster for Applied Genoproteomics, University of Liege, Li ...
  
  
Macrophage polarization is a process whereby macrophages acquire distinct effector states (M1 or M2) to carry out multiple and sometimes opposite functions. We show here that translational reprogramming occurs during macrophage polarization and that this relies on the Elongator complex subunit Elp3, an enzyme that modifies the wobble uridine base U34 in cytosolic tRNAs. Elp3 expression is downregulated by classical M1-activating signals in myeloid cells, where it limits the production of pro-inflammatory cytokines via FoxO1 phosphorylation, and attenuates experimental colitis in mice. In contrast, alternative M2-activating signals upregulate Elp3 expression through a PI3K- and STAT6-dependent signaling pathway. The metabolic reprogramming linked to M2 macrophage polarization relies on Elp3 and the translation of multiple candidates, including the mitochondrial ribosome large subunit proteins Mrpl3, Mrpl13, and Mrpl47. By promoting translation of its activator RIC8B in a codon-dependent manner, Elp3 also regulates mTORC2 activation. Elp3 expression in myeloid cells further promotes Wnt-driven tumor initiation in the intestine by maintaining a pool of tumor-associated macrophages exhibiting M2 features. Collectively, our data establish a functional link between tRNA modifications, mTORC2 activation, and macrophage polarization.
  
  
  
  
**RIC8B**
-
2
of
4
  

### The CREB Transcription Factor Controls Transcriptional Activity of the Human RIC8B Gene.

2. J Cell Biochem. 2016 Aug;117(8):1797-805. doi: 10.1002/jcb.25479 Epub 2016 Feb 2.
  
  
Maureira A(1), Sánchez R(1), Valenzuela N(1), Torrejón M(1), Hinrichs MV(1), Olate J(1), Gutiérrez J ...
  
  
Author information: (1)Departamento de Bioquímica y Biología Molecular, Facultad de Ciencias Biológi ...
  
  
Proper regulation of gene expression is essential for normal development, cellular growth, and differentiation. Differential expression profiles of mRNA coding for vertebrate Ric-8B during embryo and adult stages have been observed. In addition, Ric-8B is expressed in few cerebral nuclei subareas. These facts point to a dynamic control of RIC8B gene expression. In order to understand the transcriptional regulation of this gene, we searched for cis-elements in the sequence of the human RIC8B promoter region, identifying binding sites for the basic/leucine zipper (bZip) CREB transcription factor family (CRE sites) and C/EBP transcription factor family (C/EBP sites). CRE sites were found clustered near the transcription start site, while the C/EBP sites were found clustered at around 300 bp upstream the CRE sites. Here, we demonstrate the ability of CREB1 and C/EBPβ to bind their respective elements identified in the RIC8B promoter. Comparative protein-DNA interaction analyses revealed only the proximal elements as high affinity sites for CREB1 and only the distal elements as high affinity sites for C/EBPβ. Chromatin immunoprecipitation analyses, carried out using a human neuroblastoma cell line, confirmed the preferential association of CREB to the proximal region of the RIC8B promoter. By performing luciferase reporter assays, we found the CRE sites as the most relevant elements for its transcriptional activity. Taken together, these data show the existence of functional CREB and C/EBP binding sites in the human RIC8B gene promoter, a particular distribution of these sites and demonstrate a relevant role of CREB in stimulating transcriptional activity of this gene. J. Cell. Biochem. 117: 1797-1805, 2016. © 2016 Wiley Periodicals, Inc.
  
  
  
  
**RIC8B**
-
3
of
4
  

### A functional N-terminal domain in C/EBPβ-LAP\* is required for interacting with SWI/SNF and to repress Ric-8B gene transcription in osteoblasts.

3. J Cell Physiol. 2014 Oct;229(10):1521-8. doi: 10.1002/jcp.24595
  
  
Aguilar R(1), Grandy R, Meza D, Sepulveda H, Pihan P, van Wijnen AJ, Lian JB, Stein GS, Stein JL, Mo ...
  
  
Author information: (1)Center for Biomedical Research and FONDAP Center for Genome Regulation, Facul ...
  
  
The chromatin remodeling complex SWI/SNF and the transcription factor C/EBPβ play critical roles in osteoblastic cells as they jointly control transcription of a number of bone-related target genes. The largest C/EBPβ isoform, LAP\*, possesses a short additional N-terminal domain that has been proposed to mediate the interaction of this factor with SWI/SNF in myeloid cells. Here we examine the requirement of a functional N-terminus in C/EBPβ-LAP\* for binding SWI/SNF and for recruiting this complex to the Ric-8B gene to mediate transcriptional repression. We find that both C/EBPβ-LAP\* and SWI/SNF simultaneously bind to the Ric-8B promoter in differentiating osteoblasts that repress Ric-8B expression. This decreased expression of Ric-8B is not accompanied by significant changes in histone acetylation at the Ric-8B gene promoter sequence. A single aminoacid change at the C/EBPβ-LAP\* N-terminus (R3L) that inhibits C/EBPβ-LAP\*-SWI/SNF interaction, also prevents SWI/SNF recruitment to the Ric-8B promoter as well as C/EBPβ-LAP\*-dependent repression of the Ric-8B gene. Inducible expression of the C/EBPβ-LAP\*R3L protein in stably transfected osteoblastic cells demonstrates that this mutant protein binds to C/EBPβ-LAP\*-target promoters and competes with the endogenous C/EBPβ factor. Together our results indicate that a functional N-terminus in C/EBPβ-LAP\* is required for interacting with SWI/SNF and for Ric-8B gene repression in osteoblasts.
  
  
  
  
**RIC8B**
-
4
of
4
  

### Odorant receptor genes are expressed in olfactory neuroblastoma.

4. Genet Mol Res. 2013 Sep 10;12(3):3479-87. doi: 10.4238/2013.September.10.4
  
  
Gonzalez-Kristeller DC(1), Gutiyama LM, Campos AH, Soares FA, Brentani H, Malnic B.
  
  
Author information: (1)Departamento de Bioquímica, Instituto de Química, Universidade de São Paulo, ...
  
  
Olfactory neuroblastoma (ONB) is a malignant tumor found in the human nasal cavity. These tumors are rare and poorly characterized at the molecular level. In this study, we asked whether olfactory-specific genes are expressed in ONBs by using reverse-transcriptase-polymerase chain reaction. We found that the olfactory marker protein and the RIC-8B genes, which are specifically expressed in mature olfactory neurons, are expressed in ONBs. Importantly, we also found that ONBs express a large variety of odorant receptor genes, representative of different odorant receptor gene subfamilies. Our results show that the ONBs express genes that are normally expressed in mature olfactory neurons and indicate that they are derived from progenitor or immature cells in the olfactory epithelium and not from a clonal expansion of a single or few mature olfactory neurons.
  
  
  
  
  

---

  

# SESTD1

**Signature gene in:** Endothelial cells
  
**Differentially expressed in:** Endothelial cells
  
  
**SESTD1**
-
1
of
3
  

### Modelling of miRNA-mRNA Network to Identify Gene Signatures with Diagnostic and Prognostic Value in Gastric Cancer: Evidence from In-Silico and In-Vitro Studies.

1. Rep Biochem Mol Biol. 2024 Jul;13(2):281-300. doi: 10.61186/rbmb.13.2.281
  
  
Jahantab MB(1), Salehi M(2), Koushki M(3)(4), Farrokhi Yekta R(5), Amiri-Dashatan N(5)(6), Rezaei-Ta ...
  
  
Author information: (1)Clinical Research Development Unit, Shahid Jalil Hospital, Yasuj University o ...
  
  
BACKGROUND: Gastric cancer (GC) is a prevalent malignancy with high recurrence. Advances in systems biology have identified molecular pathways and biomarkers. This study focuses on discovering gene and miRNA biomarkers for diagnosing and predicting survival in GC patients. METHODS: Three sets of genes (GSE19826, GSE81948, and GSE112369) and two sets of miRNA expression (GSE26595, GSE78775) were obtained from the Gene Expression Omnibus (GEO), and subsequently, differentially expressed genes (DEGs) and miRNAs (DEMs) were identified. Functional pathway enrichment, DEG-miR-TF-protein-protein interaction network, DEM-mRNA network, ROC curve, and survival analyses were performed. Finally, qRT-PCR was applied to validate our results. RESULTS: From the high-throughput profiling studies of GC, we investigated 10 candidate mRNA and 7 candidate miRNAs as potential biomarkers. Expression analysis of these hubs revealed that 5 miRNAs (including miR-141-3p, miR-204-5p, miR-338-3p, miR-609, and miR-369-5p) were significantly upregulated compared to the controls. The genes with the highest degree included 6 upregulated and 4 downregulated genes in tumor samples compared to controls. The expression of miR-141-3p, miR-204-5p, SESTD1, and ANTXR1 were verified in vitro from these hub DEMs and DEGs. The findings indicated a decrease in the expression of miR-141-3p and miR-204-5p and increased expression of SESTD1 and ANTXR1 in GC cell lines compared to the GES-1 cell line. CONCLUSIONS: The current investigation successfully recognized a set of prospective miRNAs and genes that may serve as potential biomarkers for GC's early diagnosis and prognosis.
  
  
  
  
**SESTD1**
-
2
of
3
  

### Multiplatform discovery and regulatory function analysis of structural variations in non-small cell lung carcinoma.

2. Cell Rep. 2021 Sep 7;36(10):109660. doi: 10.1016/j.celrep.2021.109660
  
  
Xia L(1), Wang Z(2), Wu X(1), Zeng T(1), Luo W(3), Hu X(1), Ni Y(4), Che G(5), Liu L(5), Zhang W(6), ...
  
  
Author information: (1)Frontier Science Center for Disease Molecular Network, State Key Laboratory o ...
  
  
Non-small cell lung carcinoma (NSCLC), the most common form of lung cancer, is the leading cause of cancer-related death worldwide. We perform whole-genome sequencing (WGS) on samples from 43 primary patients with NSCLC and matched normal samples and analyze their matched open chromatin data and transcriptome data. Our results indicate that next-generation sequencing (NGS) and the Bionano Genomics (BNG) platform should be viewed as complementary technologies in terms of structural variations detection. By creating a framework integrating these two platforms, we detect high-technical-confidence somatic structural variations (SVs) in NSCLC cases, which could aid in the efficient investigation of new candidate oncogenes, such as TRIO and SESTD1. Our findings highlight the impact of somatic SVs on NSCLC oncogenesis and lay a foundation for exploring associations among somatic SVs, gene expression, and regulatory networks in patients with NSCLC.
  
  
  
  
**SESTD1**
-
3
of
3
  

### Regulation of vesicle transport and cell motility by Golgi-localized Dbs.

3. Small GTPases. 2014;5(4):1-12. doi: 10.4161/sgtp.28570
  
  
Fitzpatrick ER(1), Hu T, Ciccarelli BT, Whitehead IP.
  
  
Author information: (1)a Department of Microbiology and Molecular Genetics; The New Jersey Medical S ...
  
  
DBS/MCF2L has been recently identified as a risk locus for osteoarthritis. It encodes a guanine nucleotide exchange factor (Dbs) that has been shown to regulate both normal and tumor cell motility. In the current study, we have determined that endogenous Dbs is predominantly expressed as 2 isoforms, a 130 kDa form (Dbs-130) that is localized to the Golgi complex, and an 80 kDa form (Dbs-80) that is localized to the endoplasmic reticulum (ER). We have previously described an inhibitor that binds to the RhoGEF domain of Dbs and blocks its transforming activity. Here we show that the inhibitor localizes to the Golgi, where it specifically interacts with Dbs-130. Inhibition of endogenous Dbs-130 activity is associated with reduced levels of activated Cdc42, enlarged Golgi, and resistance to Brefeldin A-mediated Golgi dispersal, suggesting a role for Dbs in vesicle transport. Cells treated with the inhibitor exhibit normal protein transport from the ER to the Golgi, but are defective in transport from the Golgi to the plasma membrane. Inhibition of Dbs-130 in MDA-MB-231 human breast tumor cells limits motility in both transwell and wound healing assays, but appears to have no effect on the organization of the microtubule cytoskeleton. The reduced motility is associated with a failure to reorient the Golgi toward the leading edge. This is consistent with the Golgi localization, and suggests that the Dbs-130 regulates aspects of the secretory pathway that are required to support cell polarization during directed migration.
  
  
  
  
  

---

  

# SMAP2

**Signature gene in:** CD4+ T cells, CD8+ T cells, MDM, Tregs
  
**Differentially expressed in:** CD8+ T cells, MDM
  
  
**SMAP2**
-
1
of
5
  

### Multi-omics features of immunogenic cell death in gastric cancer identified by combining single-cell sequencing analysis and machine learning.

1. Sci Rep. 2024 Sep 18;14(1):21751. doi: 10.1038/s41598-024-73071-x
  
  
Dai SL(#)(1), Pan JQ(#)(2), Su ZR(3).
  
  
Author information: (1)Department of General Surgery, Deqing People's Hospital, Deqing Campus, Sir R ...
  
  
Gastric cancer (GC) is a prevalent malignancy with high mortality rates. Immunogenic cell death (ICD) is a unique form of programmed cell death that is closely linked to antitumor immunity and plays a critical role in modulating the tumor microenvironment (TME). Nevertheless, elucidating the precise effect of ICD on GC remains a challenging endeavour. ICD-related genes were identified in single-cell sequencing datasets and bulk transcriptome sequencing datasets via the AddModuleScore function, weighted gene co-expression network (WGCNA), and differential expression analysis. A robust signature associated with ICD was constructed using a machine learning computational framework incorporating 101 algorithms. Furthermore, multiomics analysis, including single-cell sequencing analysis, bulk transcriptomic analysis, and proteomics analysis, was conducted to verify the correlation of these hub genes with the immune microenvironment features of GC and with GC invasion and metastasis. We screened 59 genes associated with ICD and developed a robust ICD-related gene signature (ICDRS) via a machine learning computational framework that integrates 101 different algorithms. Furthermore, we identified five key hub genes (SMAP2, TNFAIP8, LBH, TXNIP, and PIK3IP1) from the ICDRS. Through single-cell analysis of GC tumor s, we confirmed the strong correlations of the hub genes with immune microenvironment features. Among these five genes, LBH exhibited the most significant associations with a poor prognosis and with the invasion and metastasis of GC. Finally, our findings were validated through immunohistochemical staining of a large clinical sample set, and the results further supported that LBH promotes GC cell invasion by activating the epithelial-mesenchymal transition (EMT) pathway.
  
  
  
  
**SMAP2**
-
2
of
5
  

### Discovery and Validation of Circulating EVL mRNA as a Prognostic Biomarker in Pancreatic Cancer.

2. J Oncol. 2021 Apr 20;2021:6656337. doi: 10.1155/2021/6656337 eCollection 2021.
  
  
Du Y(1), Yao K(2), Feng Q(3), Mao F(2), Xin Z(1), Xu P(4), Yao J(2)(4).
  
  
Author information: (1)Clinic Medical College, Dalian Medical University, Dalian 116000, China. (2)C ...
  
  
BACKGROUND: Circulating plasma mRNAs can be analyzed to identify putative cancer biomarkers. This study was conducted in an effort to detect plasma mRNA biomarkers capable of predicting pancreatic cancer (PACA) patient prognosis. Material and Methods. First, prognostic mRNAs that were differentially expressed in PACA in The Cancer Genome Atlas (TCGA) were established, after which microarray expression profiles from PACA patient plasma samples were utilized to specifically identify potential prognostic plasma mRNA biomarkers associated with this cancer type. In total, plasma samples were then collected from 79 PACA patients and 19 healthy controls to confirm differential mRNA expression via qPCR, while Kaplan-Meier analyses were used to examine the link between mRNA expression and patient overall survival. RESULTS: In total, three prognostic differentially expressed genes were identified in PACA patient plasma samples, including SMAP2, PTPN6, and EVL (Ena/VASP-like). Plasma EVL levels were confirmed via qPCR to be correlated with tumor pathology (p < 0.01), while the overall survival of patients with low plasma EVL levels was poor (p < 0.01). Multivariate Cox regression analyses further confirmed that plasma EVL levels were independent predictors of PACA patient prognosis. CONCLUSION: We found that PACA is associated with the downregulation of plasma EVL mRNA levels, indicating that this mRNA may be a viable biomarker associated with patient prognosis.
  
  
  
  
**SMAP2**
-
3
of
5
  

### Small-Molecule Activators of Protein Phosphatase 2A for the Treatment of Castration-Resistant Prostate Cancer.

3. Cancer Res. 2018 Apr 15;78(8):2065-2080. doi: 10.1158/0008-5472.CAN-17-0123 Epub 2018 Jan 22.
  
  
McClinch K(#)(1), Avelar RA(#)(2), Callejas D(#)(2), Izadmehr S(#)(1)(3), Wiredja D(4), Perl A(2), S ...
  
  
Author information: (1)Department of Medicine, Division of Hematology and Medical Oncology, Tisch Ca ...
  
  
Primary prostate cancer is generally treatable by androgen deprivation therapy, however, later recurrences of castrate-resistant prostate cancer (CRPC) that are more difficult to treat nearly always occur due to aberrant reactivation of the androgen receptor (AR). In this study, we report that CRPC cells are particularly sensitive to the growth-inhibitory effects of reengineered tricyclic sulfonamides, a class of molecules that activate the protein phosphatase PP2A, which inhibits multiple oncogenic signaling pathways. Treatment of CRPC cells with small-molecule activators of PP2A (SMAP) in vitro decreased cellular viability and clonogenicity and induced apoptosis. SMAP treatment also induced an array of significant changes in the phosphoproteome, including most notably dephosphorylation of full-length and truncated isoforms of the AR and downregulation of its regulatory kinases in a dose-dependent and time-dependent manner. In murine xenograft models of human CRPC, the potent compound SMAP-2 exhibited efficacy comparable with enzalutamide in inhibiting tumor formation. Overall, our results provide a preclinical proof of concept for the efficacy of SMAP in AR degradation and CRPC treatment.Significance: A novel class of small-molecule activators of the tumor suppressor PP2A, a serine/threonine phosphatase that inhibits many oncogenic signaling pathways, is shown to deregulate the phosphoproteome and to destabilize the androgen receptor in advanced prostate cancer. Cancer Res; 78(8); 2065-80. ©2018 AACR.
  
  
  
  
**SMAP2**
-
4
of
5
  

### Involvement of small ArfGAP1 (SMAP1), a novel Arf6-specific GTPase-activating protein, in microsatellite instability oncogenesis.

4. Oncogene. 2014 May 22;33(21):2758-67. doi: 10.1038/onc.2013.211 Epub 2013 Jun 10.
  
  
Sangar F(1), Schreurs AS(1), Umaña-Diaz C(1), Clapéron A(1), Desbois-Mouthon C(1), Calmel C(1), Maug ...
  
  
Author information: (1)1] INSERM, UMR\_S 938, Saint-Antoine Research Center, Paris, France [2] UPMC U ...
  
  
Small ArfGAP1 (stromal membrane-associated protein 1, SMAP1), a GTPase-activating protein specific for ADP-ribosylation factor 6 (Arf6), which is a small GTPase acting on membrane trafficking and actin remodeling, is frequently mutated in various tumors displaying microsatellite instability (MSI), notably in MSI colorectal cancers (CRC). Genotyping of 93 MSI CRCs (40 stage II, 32 stage III and 21 stage IV) allowed us to underscore that SMAP1 mutation frequency was inversely correlated with disease stage (P=0.01). Analysis of 46 cancer cell lines showed that SMAP1 mutations occurred only in MSI tumors, and consisted exclusively in short insertion or deletion in the coding 10-adenine repeat, generating a premature termination codon located downstream the ArfGAP domain. SMAP1 transcript levels were significant decreased (P=0.006), and truncated SMAP1 protein could not be detected in cells displaying biallelic SMAP1 mutations, owing to its sensitivity to proteasome degradation. To investigate the role of SMAP1 mutations, we used the SMAP1-null HCT116 cell line and we established three isogenic SMAP1-complemented clones. Cell proliferation was first assessed in vivo using subcutaneous xenografts into immunodeficient mice. Tumors developed in all animals regardless of the cell line injected, but tumor volumes were significantly smaller for both SMAP1-complemented clones compared with HCT116 (P<0.0001, at the time of killing). In vitro, SMAP1 mutations also increased cell clonogenicity (P=0.02-0.04), cell proliferation (P=0.008) by shortening the G2/M phase and decreased cell invasiveness (P=0.03-0.003). In keeping, SMAP1-complemented HCT116 gained several mesenchymal markers (Snail, Slug and vimentin) considered as a hallmark of epithelial-to-mesenchymal transition. These observations are reminiscent of some clinical characteristics of MSI CRCs, notably their larger size and lower rate of metastasis. Our observations suggest that SMAP1 loss-of-function mutations in MSI CRC may contribute to the emerging oncogenic pathway involving abnormal Arf6 regulation.
  
  
  
  
**SMAP2**
-
5
of
5
  

### Smap1 deficiency perturbs receptor trafficking and predisposes mice to myelodysplasia.

5. J Clin Invest. 2013 Mar;123(3):1123-37. doi: 10.1172/JCI63711 Epub 2013 Feb 22.
  
  
Kon S(1), Minegishi N, Tanabe K, Watanabe T, Funaki T, Wong WF, Sakamoto D, Higuchi Y, Kiyonari H, A ...
  
  
Author information: (1)Department of Molecular Immunology, Institute of Development, Aging and Cance ...
  
  
The formation of clathrin-coated vesicles is essential for intracellular membrane trafficking between subcellular compartments and is triggered by the ARF family of small GTPases. We previously identified SMAP1 as an ARF6 GTPase-activating protein that functions in clathrin-dependent endocytosis. Because abnormalities in clathrin-dependent trafficking are often associated with oncogenesis, we targeted Smap1 in mice to examine its physiological and pathological significance. Smap1-deficent mice exhibited healthy growth, but their erythroblasts showed enhanced transferrin endocytosis. In mast cells cultured in SCF, Smap1 deficiency did not affect the internalization of c-KIT but impaired the sorting of internalized c-KIT from multivesicular bodies to lysosomes, resulting in intracellular accumulation of undegraded c-KIT that was accompanied by enhanced activation of ERK and increased cell growth. Interestingly, approximately 50% of aged Smap1-deficient mice developed anemia associated with morphologically dysplastic cells of erythroid-myeloid lineage, which are hematological abnormalities similar to myelodysplastic syndrome (MDS) in humans. Furthermore, some Smap1-deficient mice developed acute myeloid leukemia (AML) of various subtypes. Collectively, to our knowledge these results provide the first evidence in a mouse model that the deregulation of clathrin-dependent membrane trafficking may be involved in the development of MDS and subsequent AML.
  
  
  
  
  

---

  

# SPTLC2

**Signature gene in:** MDM
  
**Differentially expressed in:** MDM
  
  
**SPTLC2**
-
1
of
10
  

### SPTLC2 drives an EGFR-FAK-HBEGF signaling axis to promote ovarian cancer progression.

1. Oncogene. 2025 Mar;44(10):679-693. doi: 10.1038/s41388-024-03249-0 Epub 2024 Dec 7.
  
  
Zhai X(#)(1)(2), Shen N(#)(1), Guo T(#)(3), Wang J(#)(1), Xie C(#)(1), Cao Y(1), Liu L(1), Yan Y(4), ...
  
  
Author information: (1)Institute of Cancer Stem Cell, Dalian Medical University Cancer Center, No. 9 ...
  
  
The epidermal growth factor receptor (EGFR) signaling pathway is frequently associated with ovarian cancer (OC) progression. However, inhibition of EGFR signaling in OC patients achieved limited therapeutic effects, highlighting the need to define the mechanism of EGFR deregulation in OC development. Herein we showed that serine palmitoyltransferase long chain base subunit 2 (SPTLC2) acts as a positive regulator in the EGFR signaling pathway in OC. Phenotypically, depletion of SPTLC2 suppressed clonogenic growth and migration of OC cells in vitro and in ovo, as well as metastasis in OC xenograft models, whereas overexpression of SPTLC2 yielded opposite effects. Mechanistically, SPTLC2 drives an EGFR-FAK-HBEGF signaling axis via binding with EGFR. Notably, the serine palmitoyltransferase activity of SPTLC2 is critical for regulation of the EGFR-FAK-HBEGF signaling axis and activity in OC progression. Clinically, high SPTLC2 expression is associated with high-grade serous ovarian cancer and metastasis. Collectively, our findings establish an oncogenic role of SPTLC2 in OC growth and progression though upregulation of EGFR signaling and suggest that SPTLC2 represents a potential therapeutic target in EGFR-driven ovarian cancer patients.
  
  
  
  
**SPTLC2**
-
2
of
10
  

### Serine enrichment in tumors promotes regulatory T cell accumulation through sphinganine-mediated regulation of c-Fos.

2. Sci Immunol. 2024 Apr 19;9(94):eadg8817. doi: 10.1126/sciimmunol.adg8817 Epub 2024 Apr 19.
  
  
Ma S(1), Sandhoff R(2), Luo X(3), Shang F(4)(5), Shi Q(6), Li Z(3), Wu J(1), Ming Y(1), Schwarz F(7) ...
  
  
Author information: (1)Key Laboratory of Immune Response and Immunotherapy, Center for Advanced Inte ...
  
  
CD4+ regulatory T (Treg) cells accumulate in the tumor microenvironment (TME) and suppress the immune system. Whether and how metabolite availability in the TME influences Treg cell differentiation is not understood. Here, we measured 630 metabolites in the TME and found that serine and palmitic acid, substrates required for the synthesis of sphingolipids, were enriched. A serine-free diet or a deficiency in SPTLC2, the rate-limiting enzyme catalyzing sphingolipid synthesis, suppressed Treg cell accumulation and inhibited tumor growth. Sphinganine, an intermediate metabolite in sphingolipid synthesis, physically interacted with the transcription factor c-Fos. Sphinganine c-Fos interactions enhanced the genome-wide recruitment of c-Fos to regions near the transcription start sites of target genes including Pdcd1 (encoding PD-1), which promoted Pdcd1 transcription and increased inducible Treg cell differentiation in vitro in a PD-1-dependent manner. Thus, Sptlc2-mediated sphingolipid synthesis translates the extracellular information of metabolite availability into nuclear signals for Treg cell differentiation and limits antitumor immunity.
  
  
  
  
**SPTLC2**
-
3
of
10
  

### Risk model based on genes regulating the response of tumor cells to T-cell-mediated killing in esophageal squamous cell carcinoma.

3. Aging (Albany NY). 2024 Feb 1;16(3):2494-2516. doi: 10.18632/aging.205495 Epub 2024 Feb 1.
  
  
Zhang X(1)(2), Yu C(1)(2), Zhou S(1)(2), Zhang Y(1)(2), Tian B(1)(2), Bian Y(1)(2), Wang W(1)(2), Li ...
  
  
Author information: (1)Department of Gastroenterology, Changhai Hospital, Naval Medical University, ...
  
  
Immune checkpoint inhibitors (ICIs) represent a promising therapeutic approach for esophageal squamous cell carcinoma (ESCC). However, the subpopulations of ESCC patients expected to benefit from ICIs have not been clearly defined. The anti-tumor cytotoxic activity of T cells is an important pharmacological mechanism of ICIs. In this study, the prognostic value of the genes regulating tumor cells to T cell-mediated killing (referred to as GRTTKs) in ESCC was explored by using a comprehensive bioinformatics approach. Training and validation datasets were obtained from The Cancer Genome Atlas (TCGA) and Gene Expression Omnibus (GEO), respectively. A prognostic risk scoring model was developed by integrating prognostic GRTTKs from TCGA and GEO datasets using a ridge regression algorithm. Patients with ESCC were divided into high- and low-risk groups based on eight GRTTKs (EIF4H, CDK2, TCEA1, SPTLC2, TMEM209, RGP1, EIF3D, and CAPZA3) to predict overall survival in the TCGA cohort. Using Kaplan-Meier curves, receiver operating characteristic curves, and C-index analysis, the high reliability of the prognostic risk-scoring model was certified. The model scores served as independent prognostic factors, and combining clinical staging with risk scoring improved the predictive value. Patients in the high-risk group exhibited abundant immune cell infiltration, including immune checkpoint expression, antigen presentation capability, immune cycle gene expression, and high tumor inflammation signature scores. The high-risk group exhibited a greater response to immunotherapy and neoadjuvant chemotherapy than the low-risk group. Drug sensitivity analysis demonstrated lower IC50 for AZD6244 and PD.0332991 in high-risk groups and lower IC50 for cisplatin, ATRA, QS11, and vinorelbine in the low-risk group. Furthermore, the differential expression of GRTTK-related signatures including CDK2, TCEA1, and TMEM209 were verified in ESCC tissues and paracancerous tissues. Overall, the novel GRTTK-based prognostic model can serve as indicators to predict the survival status and immunotherapy response of patients with ESCC, thereby providing guidance for the development of personalized treatment strategies.
  
  
  
  
**SPTLC2**
-
4
of
10
  

### Triptolide Inhibits the Biological Processes of HUVECs and HepG2 Cells via the Serine Palmitoyltransferase Long Chain Base Subunit 2/Sphingosine-1-Phosphate Signaling Pathway.

4. Dis Markers. 2022 Nov 18;2022:9119423. doi: 10.1155/2022/9119423 eCollection 2022.
  
  
Jia L(1)(2)(3), Zhu S(1)(2)(3), Zhu M(1)(2)(3), Huang L(1)(2)(3), Xu S(1)(2)(3), Luo Y(1)(2)(3), Xia ...
  
  
Author information: (1)Clinical Pharmacy & Pharmacology Research Institute, Affiliated Hospital of G ...
  
  
Triptolide (TP) has demonstrated innumerous biological effects and pharmacological potential against different cancer types. Hepatocellular carcinoma has a high incidence in men, and its incidence is increasing year by year. Studies have shown that angiogenesis plays an important role in the formation of tumors and that angiogenesis is closely related to tumor growth and metastasis. Deregulation of sphingolipids signaling has been associated with several pathological conditions, including cancer. In the present study, we aimed at exploring the potential molecular mechanism of TP's antivascular and antitumor effects in vitro from the perspective of sphinolipids. Human umbilical vein endothelial cells (HUVECs) and HepG2 cells were, respectively, treated with different concentrations of TP and transfected. Then, the effect of HUVECs on HepG2 cells was investigated using a three-dimensional coculture model system. CCK-8 assay was performed for cell proliferation. Cell migration and invasion abilities were assessed using the transwell assay. Cell adhesion and tube formation were detected by Matrigel. RT-PCR and western blotting were used to detect the mRNA and protein expression. The S1P production was measured via ELISA assay. Our results showed that TP inhibited HUVECs and HepG2 cells proliferation, migration, invasion, adhesion, angiogenesis, and serine palmitoyltransferase long chain base subunit 2 (SPTLC2) expression; upregulating SPTLC2 facilitated the proliferation, migration, invasion, adhesion, angiogenesis, and sphingosine-1-phosphate (S1P) production of HUVECs and HepG2 cells, while interfering with SPTLC2 expression inhibited them; HUVECs facilitated the proliferation, migration, invasion, S1P production, S1PR1, and S1PR2 expression of HepG2 cells, while S1PR3 expression was decreased. In conclusion, SPTLC2 may be associated with the antivascular and antitumor effects of TP, and SPTLC2 is expected to become a new marker for tumor therapy. HUVECs can promote the proliferation, migration, and invasion of HepG2 cells, which may be related to the S1P/sphingosine-1-phosphate receptor (S1PR) signaling pathway.
  
  
  
  
**SPTLC2**
-
5
of
10
  

### Deoxy-sphingolipids, oxidative stress, and vitamin C correlate with qualitative and quantitative patterns of small fiber dysfunction and degeneration.

5. Pain. 2022 Sep 1;163(9):1800-1811. doi: 10.1097/j.pain.0000000000002580 Epub 2022 Jan 19.
  
  
Dohrn MF(1)(2), Dumke C(1), Hornemann T(3), Nikolin S(4), Lampert A(5), Espenkott V(6), Vollert J(7) ...
  
  
Author information: (1)Department of Neurology, Medical Faculty, RWTH Aachen University, Aachen, Ger ...
  
  
Defined by dysfunction or degeneration of Aδ and C fibers, small fiber neuropathies (SFNs) entail a relevant health burden. In 50% of cases, the underlying cause cannot be identified or treated. In 100 individuals (70% female individuals; mean age: 44.8 years) with an idiopathic, skin biopsy-confirmed SFN, we characterized the symptomatic spectrum and measured markers of oxidative stress (vitamin C, selenium, and glutathione) and inflammation (transforming growth factor beta, tumor necrosis factor alpha), as well as neurotoxic 1-deoxy-sphingolipids. Neuropathic pain was the most abundant symptom (95%) and cause of daily life impairment (72%). Despite the common use of pain killers (64%), the painDETECT questionnaire revealed scores above 13 points in 80% of patients. In the quantitative sensory testing (QST), a dysfunction of Aδ fibers was observed in 70% and of C fibers in 44%, affecting the face, hands, or feet. Despite normal nerve conduction studies, QST revealed Aβ fiber involvement in 46% of patients' test areas. Despite absence of diabetes mellitus or mutations in SPTLC1 or SPTLC2 , plasma 1-deoxy-sphingolipids were significantly higher in the sensory loss patient cluster when compared with those in patients with thermal hyperalgesia ( P < 0.01) or those in the healthy category ( P < 0.1), correlating inversely with the intraepidermal nerve fiber density (1-deoxy-SA: P < 0.05, 1-deoxy-SO: P < 0.01). Patients with arterial hypertension, overweight (body mass index > 25 kg/m 2 ), or hyperlipidemia showed significantly lower L-serine (arterial hypertension: P < 0.01) and higher 1-deoxy-sphingolipid levels (arterial hypertension: P < 0.001, overweight: P < 0.001, hyperlipidemia: P < 0.01). Lower vitamin C levels correlated with functional Aβ involvement ( P < 0.05). Reduced glutathione was lower in patients with Aδ dysfunction ( P < 0.05). Idiopathic SFNs are heterogeneous. As a new pathomechanism, plasma 1-deoxy-sphingolipids might link the metabolic syndrome with small fiber degeneration.
  
  
  
  
**SPTLC2**
-
6
of
10
  

### Elevated ATGL in colon cancer cells and cancer stem cells promotes metabolic and tumorigenic reprogramming reinforced by obesity.

6. Oncogenesis. 2021 Nov 29;10(11):82. doi: 10.1038/s41389-021-00373-4
  
  
Iftikhar R(#)(1), Penrose HM(#)(1), King AN(1), Samudre JS(1), Collins ME(1), Hartono AB(1), Lee SB( ...
  
  
Author information: (1)Department of Pathology and Laboratory Medicine, Tulane University School of ...
  
  
Erratum in Oncogenesis. 2022 Mar 15;11(1):14. doi: 10.1038/s41389-022-00388-5.
  
  
  
  
**SPTLC2**
-
7
of
10
  

### [Clinicopathological features and molecular genetic changes of lung salivary gland-type clear cell carcinoma].

7. Zhonghua Bing Li Xue Za Zhi. 2021 Jul 8;50(7):728-733. doi: 10.3760/cma.j.cn112151-20210129-00129
  
  
[Article in Chinese; Abstract available in Chinese from the publisher]
  
  
Xue QQ(1), Huang Y(2), Zuo SY(3), Zheng Q(1), Ji G(1), Zhou XY(1), Wu CY(2), Li Y(1).
  
  
Author information: (1)Department of Pathology, Fudan University Shanghai Cancer Center; Department of Oncology, Shanghai Medical College, Fudan University, Shanghai 200032, China. (2)Department of Pathology, Tongji University Shanghai Pulmonary Hospital, Shanghai 200433, China. (3)Department of Pathology, the Second People's Hospital of Liaocheng, Shandong Province, Liaocheng 252600, China.
  
  
  
  
**SPTLC2**
-
8
of
10
  

### Serine restriction alters sphingolipid diversity to constrain tumour growth.

8. Nature. 2020 Oct;586(7831):790-795. doi: 10.1038/s41586-020-2609-x Epub 2020 Aug 12.
  
  
Muthusamy T(1), Cordes T(#)(1), Handzlik MK(#)(1), You L(#)(1), Lim EW(1), Gengatharan J(1), Pinto A ...
  
  
Author information: (1)Department of Bioengineering, University of California San Diego, La Jolla, C ...
  
  
Comment in Signal Transduct Target Ther. 2020 Nov 24;5(1):274. doi: 10.1038/s41392-020-00401-6.
  
  
  
  
**SPTLC2**
-
9
of
10
  

### Decreased SPTLC1 expression predicts worse outcomes in ccRCC patients.

9. J Cell Biochem. 2020 Feb;121(2):1552-1562. doi: 10.1002/jcb.29390 Epub 2019 Sep 12.
  
  
Zhu WK(1)(2)(3), Xu WH(2)(3), Wang J(2)(3), Huang YQ(2)(3), Abudurexiti M(2)(3), Qu YY(2)(3), Zhu YP ...
  
  
Author information: (1)Institutes of Biomedical Science, Fudan University, Shanghai, China. (2)Depar ...
  
  
OBJECTIVE: Serine palmitoyltransferase, long chain base subunit 1 (SPTLC1) catalyzes the first step in sphingolipid synthesis and has been implicated in the progression of various cancers. However, its role in clear cell renal cell carcinoma (ccRCC) remains unclear. Here, we investigated the expression and prognostic value of SPTLC1 in ccRCC. METHODS: Three ccRCC patient cohorts were studied. ccRCC and adjacent normal kidney tissue samples were obtained from 183 patients at the Fudan University Shanghai Cancer Center (FUSCC) and subjected to immunohistochemical staining and quantitative reverse-transcription polymerase chain reaction to evaluate SPTLC1 protein and messenger RNA (mRNA) expression. Two validation cohorts consisting of mRNA and clinicopathological data sets from patients with ccRCC were obtained from the Cancer Genome Atlas (TCGA, n = 429) and Oncomine (n = 178) databases. Associations between low and high SPTLC1 mRNA and protein expression and survival were evaluated using the Kaplan-Meier method and log-rank test. Independent prognostic factors were identified using univariate and multivariate Cox regression analysis. RESULTS: SPTLC1 mRNA or protein were expressed at significantly lower levels in ccRCC tissues compared with normal kidney tissues in all three patient cohorts (P < .001). Low SPTLC1 expression was significantly associated with shorter overall survival in the FUSCC (P = .041) and Oncomine (P < .001) cohorts, and was significantly associated with shorter overall survival (P < .0001) and progression-free survival (P < .001) in the TCGA cohort. Bioinformatics analysis identified 10 genes significantly coregulated with SPTLC1 in ccRCC, most of which contributed to sphingomyelin metabolism (SPTLC2, SPTLC3, SPTSSA, SPTSSB, ORMDL1, ORMDL2, ORMDL3, ZDHHC9, GOLGA7B, and KDSR). Functional enrichment analysis predicted that SPTLC1 and its network play significant roles in inflammatory, hypoxia, and interferon gamma responses, and in allograft rejection pathways. CONCLUSION: Low SPTLC1 expression is significantly associated with disease progression and poor survival in patients with ccRCC, suggesting that SPTLC1 may function as a tumor suppressor. Thus, SPTLC1 could be a potential new biomarker and/or therapeutic target for ccRCC.
  
  
  
  
**SPTLC2**
-
10
of
10
  

### Aster glehni Extract Containing Caffeoylquinic Compounds Protects Human Keratinocytes through the TRPV4-PPARδ-AMPK Pathway.

10. Evid Based Complement Alternat Med. 2018 Dec 9;2018:9616574. doi: 10.1155/2018/9616574 eCollection 2018.
  
  
Lee YJ(1), Jang YN(1), Han YM(1), Kim HM(1)(2), Jin C(3), Kim HJ(3), Seo HS(1)(2).
  
  
Author information: (1)Cardiovascular Center, Korea University Guro Hospital, 148, Gurodong-ro, Guro ...
  
  
Aster glehni (AG) has been used in cooking and as a medicine to treat various diseases for over hundreds of years in Korea. To speculate the protective effects of AG on skin barrier, we estimated the protein levels of biomarkers related to skin barrier protection in human keratinocytes, HaCaT cells treated with sodium dodecyl sulfate (SDS), or 2,4-dinitrochlorobenzene (DNCB). The protein levels for keratin, involucrin, defensin, tumor necrosis factor alpha (TNFα), peroxisome proliferator-activated receptor delta (PPARδ), 5' adenosine monophosphate-activated protein kinase (AMPK), serine palmitoyltransferase long chain base subunit 2 (SPTLC2), and transient receptor potential cation channel subfamily V member 4 (TRPV4) were evaluated using western blotting or immunocytochemistry in HaCaT cells. AG extract increased the protein levels of PPARδ, phosphorylated AMPK, SPTLC2, keratin, involucrin, and defensin compared to the SDS or DNCB control group. However, TNFα expression increased by SDS or DNCB was decreased with AG extract. The order of action of each regulatory biomarker in AG pathway was identified TRPV4→PPARδ→AMPK from antagonist and siRNA treatment studies. AG can ameliorate the injury of keratinocytes caused by SDS or DNCB through the sequential regulation of TRPV4→PPARδ→AMPK pathway.
  
  
  
  
  

---

  

# SYTL3

**Signature gene in:** CD4+ T cells, CD8+ T cells, Tregs
  
**Differentially expressed in:** CD8+ T cells, Tregs
  
  
**SYTL3**
-
1
of
4
  

### Coordinated reprogramming of renal cancer transcriptome, metabolome and secretome associates with immune tumor infiltration.

1. Cancer Cell Int. 2023 Jan 5;23(1):2. doi: 10.1186/s12935-022-02845-y
  
  
Poplawski P(1), Alseekh S(2)(3), Jankowska U(4), Skupien-Rabian B(4), Iwanicka-Nowicka R(5)(6), Koss ...
  
  
Author information: (1)Department of Biochemistry and Molecular Biology, Centre of Postgraduate Medi ...
  
  
BACKGROUND: Clear cell renal cell carcinoma (ccRCC) is the most common subtype of renal cancer. The molecules (proteins, metabolites) secreted by tumors affect their extracellular milieu to support cancer progression. If secreted in amounts detectable in plasma, these molecules can also serve as useful, minimal invasive biomarkers. The knowledge of ccRCC tumor microenvironment is fragmentary. In particular, the links between ccRCC transcriptome and the composition of extracellular milieu are weakly understood. In this study, we hypothesized that ccRCC transcriptome is reprogrammed to support alterations in tumor microenvironment. Therefore, we comprehensively analyzed ccRCC extracellular proteomes and metabolomes as well as transcriptomes of ccRCC cells to find molecules contributing to renal tumor microenvironment. METHODS: Proteomic and metabolomics analysis of conditioned media isolated from normal kidney cells as well as five ccRCC cell lines was performed using mass spectrometry, with the following ELISA validation. Transcriptomic analysis was done using microarray analysis and validated using real-time PCR. Independent transcriptomic and proteomic datasets of ccRCC tumors were used for the analysis of gene and protein expression as well as the level of the immune infiltration. RESULTS: Renal cancer secretome contained 85 proteins detectable in human plasma, consistently altered in all five tested ccRCC cell lines. The top upregulated extracellular proteins included SPARC, STC2, SERPINE1, TGFBI, while downregulated included transferrin and DPP7. The most affected extracellular metabolites were increased 4-hydroxy-proline, succinic acid, cysteine, lactic acid and downregulated glutamine. These changes were associated with altered expression of genes encoding the secreted proteins (SPARC, SERPINE1, STC2, DPP7), membrane transporters (SLC16A4, SLC6A20, ABCA12), and genes involved in protein trafficking and secretion (KIF20A, ANXA3, MIA2, PCSK5, SLC9A3R1, SYTL3, and WNTA7). Analogous expression changes were found in ccRCC tumors. The expression of SPARC predicted the infiltration of ccRCC tumors with endothelial cells. Analysis of the expression of the 85 secretome genes in > 12,000 tumors revealed that SPARC is a PanCancer indicator of cancer-associated fibroblasts' infiltration. CONCLUSIONS: Transcriptomic reprogramming of ccRCC supports the changes in an extracellular milieu which are associated with immune infiltration. The proteins identified in our study represent valuable cancer biomarkers detectable in plasma.
  
  
  
  
**SYTL3**
-
2
of
4
  

### Androgen Receptor Signaling Reduces the Efficacy of Bacillus Calmette-Guérin Therapy for Bladder Cancer via Modulating Rab27b-Induced Exocytosis.

2. Mol Cancer Ther. 2020 Sep;19(9):1930-1942. doi: 10.1158/1535-7163.MCT-20-0050 Epub 2020 Jul 31.
  
  
Mizushima T(1)(2)(3)(4)(5), Jiang G(1)(2), Kawahara T(1)(3)(4)(6), Li P(3)(4), Han B(3)(4), Inoue S( ...
  
  
Author information: (1)Department of Pathology & Laboratory Medicine, University of Rochester Medica ...
  
  
Although intravesical bacillus Calmette-Guérin (BCG) immunotherapy has been the gold standard for nonsurgical management of non-muscle-invasive bladder cancer, a considerable number of patients exhibit resistance to the adjuvant treatment with unexplained mechanisms. This study aimed to investigate whether and how androgen receptor (AR) signals modulate BCG cytotoxicity in bladder cancer. AR knockdown or overexpression in bladder cancer lines resulted in induction or reduction, respectively, in intracellular BCG quantity and its cytotoxic activity. Microarray screening identified Rab27b, a small GTPase known to mediate bacterial exocytosis, which was upregulated in BCG-resistant cells and downregulated in AR-shRNA cells. Knockdown of Rab27b, or its effector SYTL3, or overexpression of Rab27b also induced or reduced, respectively, BCG quantity and cytotoxicity. In addition, treatment with GW4869, which was previously shown to inhibit Rab27b-dependent secretion, induced them and reduced Rab27b expression in bladder cancer cells. Meanwhile, AR expression was upregulated in BCG-resistant lines, compared with respective controls. In a mouse orthotopic xenograft model, Rab27b/SYTL3 knockdown or GW4869 treatment enhanced the amount of BCG within tumors and its suppressive effect on tumor growth. Moreover, in non-muscle-invasive bladder cancer specimens from patients subsequently undergoing BCG therapy, positivity of AR/Rab27b expression was associated with significantly higher risks of tumor recurrence. AR activation thus correlates with resistance to BCG treatment, presumably via upregulating Rab27b expression. Mechanistically, it is suggested that BCG elimination from urothelial cells is induced by Rab27b/SYTL3-mediated exocytosis. Accordingly, Rab27b inactivation, potentially via antiandrogenic drugs and/or exocytosis inhibition are anticipated to sensitize the efficacy of BCG therapy, especially in patients with BCG-refractory AR/Rab27b-positive bladder cancer.
  
  
  
  
**SYTL3**
-
3
of
4
  

### The Search for Molecular Markers in a Gene-Orphan Case Study of a Pediatric Spinal Cord Pilocytic Astrocytoma.

3. Cancer Genomics Proteomics. 2020 Mar-Apr;17(2):117-130. doi: 10.21873/cgp.20172
  
  
Martinelli C(1), Gabriele F(1), Manai F(1), Ciccone R(2)(3), Novara F(3), Sauta E(4), Bellazzi R(4), ...
  
  
Author information: (1)Department of Biology and Biotechnology, University of Pavia, Pavia, Italy. ( ...
  
  
BACKGROUND/AIM: We herein presented a case of pediatric spinal cord pilocytic astrocytoma diagnosed on the basis of histopathological and clinical findings. MATERIALS AND METHODS: Given the paucity of data on genetic features for this tumor, we performed exome, array CGH and RNA sequencing analysis from nucleic acids isolated from a unique and not repeatable very small amount of a formalin-fixed, paraffin-embedded (FFPE) specimen. RESULTS: DNA mutation analysis, comparing tumor and normal lymphocyte peripheral DNA, evidenced few tumor-specific single nucleotide variants in DEFB119, MUC5B, NUDT1, LTBP3 and CPSF3L genes. Differently, tumor DNA was not characterized by for the main pilocytic astrocytoma gene variations, including BRAFV600E. An inframe trinucleotides insertion involving DLX6 or lnc DLX6-AS1 genes was scored in 44.9% of sequenced reads; the temporal profile of this variation on the expression of DLX-AS1 was investigated in patient's urine-derived exosomes, reporting no significant variation in the one-year molecular follow-up. Array CGH identified a tumor microdeletion at the 6q25.3 chromosomal region, spanning 1,01 Mb and comprising ZDHHC14, SNX9, TULP4 and SYTL3 genes. The expression of these genes did not change in urine-derived exosomes during the one-year investigation period. Finally, RNAseq did not reveal any of the common pilocytic BRAF-KIAA1549 genes fusion events. CONCLUSION: To our knowledge, the present report is one of the first described gene-orphan case studies of a pediatric spinal cord pilocytic astrocytoma.
  
  
  
  
**SYTL3**
-
4
of
4
  

### Whole-Exome Sequencing Study of Thyrotropin-Secreting Pituitary Adenomas.

4. J Clin Endocrinol Metab. 2017 Feb 1;102(2):566-575. doi: 10.1210/jc.2016-2261
  
  
Sapkota S(1), Horiguchi K(1), Tosaka M(2), Yamada S(3), Yamada M(1).
  
  
Author information: (1)Department of Medicine and Molecular Science and. (2)Department of Neurosurge ...
  
  
CONTEXT: Thyrotropin (TSH)-secreting pituitary adenomas (TSHomas) are a rare cause of hyperthyroidism, and the genetic aberrations responsible remain unknown. OBJECTIVE: To identify somatic genetic abnormalities in TSHomas. DESIGN AND SETTING: A single-nucleotide polymorphism (SNP) array analysis was performed on 8 TSHomas. Four tumors with no allelic losses or limited loss of heterozygosity were selected, and whole-exome sequencing was performed, including their corresponding blood samples. Somatic variants were confirmed by Sanger sequencing. A set of 8 tumors was also assessed to validate candidate genes. PATIENTS: Twelve patients with sporadic TSHomas were examined. RESULTS: The overall performance of whole-exome sequencing was good, with an average coverage of each base in the targeted region of 97.6%. Six DNA variants were confirmed as candidate driver mutations, with an average of 1.5 somatic mutations per tumor. No mutations were recurrent. Two of these mutations were found in genes with an established role in malignant tumorigenesis (SMOX and SYTL3), and 4 had unknown roles (ZSCAN23, ASTN2, R3HDM2, and CWH43). Similarly, an SNP array analysis revealed frequent chromosomal regions of copy number gains, including recurrent gains at loci harboring 4 of these 6 genes. CONCLUSIONS: Several candidate somatic mutations and changes in copy numbers for TSHomas were identified. The results showed no recurrence of mutations in the tumors studied but a low number of mutations, thereby highlighting their benign nature. Further studies on a larger cohort of TSHomas, along with the use of epigenetic and transcriptomic approaches, may reveal the underlying genetic lesions.
  
  
  
  
  

---

  

# TMCC3

**Signature gene in:** Endothelial cells
  
**Differentially expressed in:** Endothelial cells
  
  
**TMCC3**
-
1
of
5
  

### NamiRNA-enhancer network of miR-492 activates the NR2C1-TGF-β/Smad3 pathway to promote epithelial-mesenchymal transition of pancreatic cancer.

1. Carcinogenesis. 2023 May 26;44(2):153-165. doi: 10.1093/carcin/bgac102
  
  
Liu S(1), He X(1), Di Y(2), Li Q(1), Li F(1), Ma Y(1), Chen L(3), Gao Y(1)(4), Xu J(1), Yang S(5), X ...
  
  
Author information: (1)Scientific research Center, Shanghai Public Health Clinical Center, Fudan Uni ...
  
  
Pancreatic cancer (PaCa) is one of the most fatal malignancies of the digestive system, and most patients are diagnosed at advanced stages due to the lack of specific and effective tumor-related biomarkers for the early detection of PaCa. miR-492 has been found to be upregulated in PaCa tumor tissue and may serve as a potential therapeutic target. However, the molecular mechanisms by which miR-492 promotes PaCa tumor growth and progression are unclear. In this study, we first found that miR-492 in enhancer loci activated neighboring genes (NR2C1/NDUFA12/TMCC3) and promoted PaCa cell proliferation, migration, and invasion in vitro. We also observed that miR-492-activating genes significantly enriched the TGF-β/Smad3 signaling pathway in PaCa to promote epithelial-mesenchymal transition (EMT) during tumorigenesis and development. Using CRISPR-Cas9 and ChIP assays, we further observed that miR-492 acted as an enhancer trigger, and that antagomiR-492 repressed PaCa tumorigenesis in vivo, decreased the expression levels of serum TGF-β, and suppressed the EMT process by downregulating the expression of NR2C1. Our results demonstrate that miR-492, as an enhancer trigger, facilitates PaCa progression via the NR2C1-TGF-β/Smad3 pathway.
  
  
  
  
**TMCC3**
-
2
of
5
  

### Outcomes Following GD2-Directed Postconsolidation Therapy for Neuroblastoma After Cessation of Random Assignment on ANBL0032: A Report From the Children's Oncology Group.

2. J Clin Oncol. 2022 Dec 10;40(35):4107-4118. doi: 10.1200/JCO.21.02478 Epub 2022 Jul 15.
  
  
Desai AV(1), Gilman AL(2), Ozkaynak MF(3), Naranjo A(4), London WB(5), Tenney SC(4), Diccianni M(6), ...
  
  
Author information: (1)University of Chicago, Chicago, IL. (2)ICON plc, Raleigh, NC. (3)Maria Fareri ...
  
  
PURPOSE: Postconsolidation immunotherapy including dinutuximab, granulocyte-macrophage colony-stimulating factor, and interleukin-2 improved outcomes for patients with high-risk neuroblastoma enrolled on the randomized portion of Children's Oncology Group study ANBL0032. After random assignment ended, all patients were assigned to immunotherapy. Survival and toxicities were assessed. PATIENTS AND METHODS: Patients with a pre-autologous stem cell transplant (ASCT) response (excluding bone marrow) of partial response or better were eligible. Demographics, stage, tumor biology, pre-ASCT response, and adverse events were summarized using descriptive statistics. Event-free survival (EFS) and overall survival (OS) from time of enrollment (up to day +200 from last ASCT) were evaluated. RESULTS: From 2009 to 2015, 1,183 patients were treated. Five-year EFS and OS for the entire cohort were 61.1 ± 1.9% and 71.9 ± 1.7%, respectively. For patients ≥ 18 months old at diagnosis with International Neuroblastoma Staging System stage 4 disease (n = 662) 5-year EFS and OS were 57.0 ± 2.4% and 70.9 ± 2.2%, respectively. EFS was superior for patients with complete response/very good partial response pre-ASCT compared with those with PR (5-year EFS: 64.2 ± 2.2% v 55.4 ± 3.2%, P = .0133); however, OS was not significantly different. Allergic reactions, capillary leak, fever, and hypotension were more frequent during interleukin-2-containing cycles than granulocyte-macrophage colony-stimulating factor-containing cycles (P < .0001). EFS was superior in patients with higher peak dinutuximab levels during cycle 1 (P = .034) and those with a high affinity FCGR3A genotype (P = .0418). Human antichimeric antibody status did not correlate with survival. CONCLUSION: Analysis of a cohort assigned to immunotherapy after cessation of random assignment on ANBL0032 confirmed previously described survival and toxicity outcomes. EFS was highest among patients with end-induction complete response/very good partial response. Among patients with available data, higher dinutuximab levels and FCGR3A genotype were associated with superior EFS. These may be predictive biomarkers for dinutuximab therapy.
  
  
  
  
**TMCC3**
-
3
of
5
  

### Transmembrane and coiled-coil domain family 3 (TMCC3) regulates breast cancer stem cell and AKT activation.

3. Oncogene. 2021 Apr;40(16):2858-2871. doi: 10.1038/s41388-021-01729-1 Epub 2021 Mar 19.
  
  
Wang YH(1), Chan YT(1), Hung TH(1), Hung JT(1), Kuo MW(1), Wang SH(1), Huang Y(2)(3), Lin YJ(1), Che ...
  
  
Author information: (1)Institute of Stem Cell and Translational Cancer Research, Chang Gung Memorial ...
  
  
Cancer stem cells (CSC) play a pivotal role in cancer metastasis and resistance to therapy. Previously, we compared the phosphoproteomes of breast cancer stem cells (BCSCs) enriched subpopulation and non-BCSCs sorted from breast cancer patient-derived xenograft (PDX), and identified a function unknown protein, transmembrane and coiled-coil domain family 3 (TMCC3) to be a potential enrichment marker for BCSCs. We demonstrated greater expression of TMCC3 in BCSCs than non-BCSCs and higher expression of TMCC3 in metastatic lymph nodes and lungs than in primary tumor of breast cancer PDXs. TMCC3 silencing suppressed mammosphere formation, ALDH activity and cell migration in vitro, along with reduced tumorigenicity and metastasis in vivo. Mechanistically, we found that AKT activation was reduced by TMCC3 silencing, but enhanced by TMCC3 overexpression. We further demonstrated that TMCC3 interacted directly with AKT through its 1-153 a.a. domain by cell-free biochemical assay in vitro and co-immunoprecipitation and interaction domain mapping assays in vivo. Based on domain truncation studies, we showed that the AKT-interacting domain of TMCC3 was essential for TMCC3-induced AKT activation, self-renewal, and metastasis. Clinically, TMCC3 mRNA expression in 202 breast cancer specimens as determined by qRT-PCR assay showed that higher TMCC3 expression correlated with poorer clinical outcome of breast cancer, including early-stage breast cancer. Multivariable analysis identified TMCC3 expression as an independent risk factor for survival. These findings suggest that TMCC3 is crucial for maintenance of BCSCs features through AKT regulation, and TMCC3 expression has independent prognostic significance in breast cancer. Thus, TMCC3 may serve as a new target for therapy directed against CSCs.
  
  
  
  
**TMCC3**
-
4
of
5
  

### Identification of gene and microRNA changes in response to smoking in human airway epithelium by bioinformatics analyses.

4. Medicine (Baltimore). 2019 Sep;98(38):e17267. doi: 10.1097/MD.0000000000017267
  
  
Huang J(1), Jiang W(2), Tong X(1), Zhang L(1), Zhang Y(2), Fan H(1).
  
  
Author information: (1)Department of Respiratory and Critical Care Medicine, West China Hospital/Wes ...
  
  
Smoking is a substantial risk factor for many respiratory diseases. This study aimed to identify the gene and microRNA changes related to smoking in human airway epithelium by bioinformatics analysis.From the Gene Expression Omnibus (GEO) database, the mRNA datasets GSE11906, GSE22047, GSE63127, and microRNA dataset GSE14634 were downloaded, and were analyzed using GEO2R. Functional enrichment analysis of the differentially expressed genes (DEGs) was enforced using DAVID. The protein-protein interaction (PPI) network and differentially expressed miRNAs (DEMs)- DEGs network were executed by Cytoscape.In total, 107 DEGs and 10 DEMs were determined. Gene Ontology (GO) analysis revealed that DEGs principally enriched in oxidation-reduction process, extracellular space and oxidoreductase activity. Kyoto Encyclopedia of Genes and Genomes (KEGG) pathway demonstrated that DEGs were principally enriched in metabolism of xenobiotics by cytochrome P450 and chemical carcinogenesis. The PPI network revealed 15 hub genes, including NQO1, CYP1B1, AKR1C1, CYP1A1, AKR1C3, CEACAM5, MUCL1, B3GNT6, MUC5AC, MUC12, PTGER4, CALCA, CBR1, TXNRD1, and CBR3. Cluster analysis showed that these hub genes were associated with adenocarcinoma in situ, squamous cell carcinoma, cell differentiation, inflammatory response, oxidative DNA damage, oxidative stress response and tumor necrosis factor. Hsa-miR-627-5p might have the most target genes, including ITLN1, TIMP3, PPP4R4, SLC1A2, NOVA1, RNFT2, CLDN10, TMCC3, EPHA7, SRPX2, PPP1R16B, GRM1, HS3ST3A1, SFRP2, SLC7A11, and KLHDC8A.We identified several molecular changes induced by smoking in human airway epithelium. This study may provide some candidate genes and microRNAs for assessing the risk of lung diseases caused by smoking.
  
  
  
  
**TMCC3**
-
5
of
5
  

### Proteomics Profiling of CLL Versus Healthy B-cells Identifies Putative Therapeutic Targets and a Subtype-independent Signature of Spliceosome Dysregulation.

5. Mol Cell Proteomics. 2018 Apr;17(4):776-791. doi: 10.1074/mcp.RA117.000539 Epub 2018 Jan 24.
  
  
Johnston HE(1)(2), Carter MJ(1), Larrayoz M(3), Clarke J(4), Garbis SD(2)(5), Oscier D(6), Strefford ...
  
  
Author information: (1)From the ‡Antibody and Vaccine Group, Cancer Sciences Unit, Faculty of Medici ...
  
  
Chronic lymphocytic leukemia (CLL) is a heterogeneous B-cell cancer exhibiting a wide spectrum of disease courses and treatment responses. Molecular characterization of RNA and DNA from CLL cases has led to the identification of important driver mutations and disease subtypes, but the precise mechanisms of disease progression remain elusive. To further our understanding of CLL biology we performed isobaric labeling and mass spectrometry proteomics on 14 CLL samples, comparing them with B-cells from healthy donors (HDB). Of 8694 identified proteins, ∼6000 were relatively quantitated between all samples (q<0.01). A clear CLL signature, independent of subtype, of 544 significantly overexpressed proteins relative to HDB was identified, highlighting established hallmarks of CLL (e.g. CD5, BCL2, ROR1 and CD23 overexpression). Previously unrecognized surface markers demonstrated overexpression (e.g. CKAP4, PIGR, TMCC3 and CD75) and three of these (LAX1, CLEC17A and ATP2B4) were implicated in B-cell receptor signaling, which plays an important role in CLL pathogenesis. Several other proteins (e.g. Wee1, HMOX1/2, HDAC7 and INPP5F) were identified with significant overexpression that also represent potential targets. Western blotting confirmed overexpression of a selection of these proteins in an independent cohort. mRNA processing machinery were broadly upregulated across the CLL samples. Spliceosome components demonstrated consistent overexpression (p = 1.3 × 10^-21^) suggesting dysregulation in CLL, independent of SF3B1 mutations. This study highlights the potential of proteomics in the identification of putative CLL therapeutic targets and reveals a subtype-independent protein expression signature in CLL.
  
  
  
  
  

---

  

# TPRG1

**Signature gene in:** MDM
  
**Differentially expressed in:** MDM
  
  
**TPRG1**
-
1
of
10
  

### Long Non-coding RNA TPRG1-AS1 Interacts With CLTC in Liver Cancer Cells.

1. Anticancer Res. 2024 Nov;44(11):4813-4824. doi: 10.21873/anticanres.17307
  
  
Moon SU(#)(1), Shah M(#)(1), Thao TT(#)(1), Woo HG(2)(3).
  
  
Author information: (1)Department of Physiology, Ajou University School of Medicine, Suwon, Republic ...
  
  
BACKGROUND/AIM: Certain long non-coding RNAs (lncRNAs), identified as potential tumor suppressors, have shown potential in inhibiting tumor growth. Here, we investigated a novel mechanism involving the direct interaction between lncRNA TPRG1-AS1 and Clathrin Heavy Chain (CLTC) in the Epidermal Growth Factor (EGF) signaling pathway for its tumor-suppressive effects. MATERIALS AND METHODS: Our research revealed a direct physical interaction between TPRG1-AS1 and CLTC through RNA pulldown and RNA immunoprecipitation (RIP)-qPCR, which subsequently influenced the EGF signaling pathway. We confirmed phenotype changes by cell viability, sphere formation, and invasion. We confirmed the mechanism underlying these phenotypic changes through immunoblotting and immunocytochemistry. RESULTS: Firstly, we confirmed a reduction in the phenotype associated with the overexpression of TPRG1-AS1 (TPRG1-AS1oe) interacting with CLTC in the presence of EGF signaling. Next, it was observed that TPRG1-AS1oe suppressed EGF downstream signaling, specifically MAPK8 and MAPK14, in relation to CLTC. Moreover, we verified that overexpressed TPRG1-AS1 binds to CLTC and suppresses EGF downstream signaling using a custom HEX probe. CONCLUSION: Collectively our study uncovered a novel regulatory axis wherein TPRG1-AS1 interacts with CLTC, consequently attenuating EGF downstream signaling, particularly through the MAPK8 and MAPK14 pathways. These complex interactions ultimately lead to a reduction in the phenotype of liver cancer cells.
  
  
  
  
**TPRG1**
-
2
of
10
  

### A Circadian Rhythm-related Signature to Predict Prognosis, Immune Infiltration, and Drug Response in Breast Cancer.

2. Curr Med Chem. 2025;32(3):608-626. doi: 10.2174/0109298673320179240803071001
  
  
Chu M(1), Huang J(1), Wang Q(1), Fang Y(1), Cui D(1), Jin Y(1)(2).
  
  
Author information: (1)Department of Medical Genetics, School of Basic Medical Sciences, Nanjing Med ...
  
  
PURPOSE: Circadian rhythm-related genes (CRRGs) play essential roles in cancer occurrence and development. However, the prognostic significance of CRRGs in breast cancer (BC) has not been fully elucidated. Our study aimed to develop a prognostic gene signature based on CRRGs that can accurately and stably predict the prognosis of BC. METHODS: The transcriptome data and clinical information for BC patients were obtained from The Cancer Genome Atlas (TCGA) and Gene Expression Omnibus (GEO) databases. A consensus unsupervised clustering analysis was carried out to investigate the roles of CRRGs in BC. A CRRGs-related prognostic risk model was established by using logistic least absolute shrinkage and selection operator (LASSO) Cox regression and univariate Cox regression analyses. Kaplan-Meier (KM) curves analysis, time-dependent receptor operation characteristics (ROC) curves analysis, and nomogram were plotted to evaluate the predictive efficacy of the model. The relevance of risk score to the immune cell infiltration, tumor burden mutation (TMB), and therapeutic response was assessed. RESULTS: A risk model comprising six CRRGs (SLC44A4, SLC16A6, TPRG1, FABP7, GLYATL2, and FDCSP) was constructed and validated, demonstrating an effective predictor for the prognosis of BC. The low-risk group displayed a higher expression of immune checkpoint genes and a lower burden of tumor mutation. Additionally, drug sensitivity analysis demonstrated that the prognostic signature may serve as a potential chemosensitivity predictor. CONCLUSION: We established a CRRGs-related risk signature, which is of great value in predicting the prognosis of patients with BC and guiding the treatment for BC.
  
  
  
  
**TPRG1**
-
3
of
10
  

### Meta-analysis of genome-wide association studies for cancer therapy-related cardiovascular dysfunction and functional mapping highlight an intergenic region close to TP63.

3. Sci Rep. 2024 Aug 8;14(1):18413. doi: 10.1038/s41598-024-69064-5
  
  
Martínez-Campelo L(1), Blanco-Verea A(2), López-Fernández T(3), Martínez-Monzonís A(4), Buño A(5), M ...
  
  
Collaborators: Sendón JL, Soto AB, Albendea MC, Espinosa E, Batlle JF, López-Fernández T, de Sá EL, ...
  
  
Author information: (1)Instituto de Investigación Sanitaria de Santiago, Xenética Cardiovascular, Santiago De Compostela, Spain. laura\_94.mc@hotmail.com. (2)Instituto de Investigación Sanitaria de Santiago, Xenética Cardiovascular, Santiago De Compostela, Spain. (3)Hospital Universitario La Paz, Servicio de Cardiología, Madrid, Spain. (4)Hospital Clínico Universitario de Santiago de Compostela, Servicio de Cardiología, CIBER de Enfermedades Cardiovasculares, Santiago De Compostela, Spain. (5)Hospital Universitario La Paz, Servicio de Análisis Clínicos, IdiPaz, Madrid, Spain. (6)Hospital Universitario La Paz, Servicio de Oncología, Madrid, Spain. (7)Department of Cancer Biology, Mayo Clinic Florida, Jacksonville, USA. (8)Department of Quantitative Health Sciences, Mayo Clinic Florida, Jacksonville, USA. (9)Spanish National Cancer Research Centre, Human Cancer Genetics Programme, Madrid, Spain. (10)Department of Medical Oncology, Integrated Cancer Center Ghent, Ghent, Belgium. (11)Center for Oncological Research (CORE), Integrated Personalized and Precision Oncology Network (IPPON), University of Antwerp, Wilrijk, Belgium. (12)Department of Medical Oncology, University Hospital Ramón y Cajal, Madrid, Spain. (13)Centro Singular de Investigación en Medicina Molecular y Enfermedades Crónicas, Universidade de Santiago de Compostela, CIBER de Enfermedades Raras, Madrid, Spain. (14)Fundación Pública Galega de Medicina Xenómica, Grupo de Medicina Xenómica, Santiago De Compostela, Spain. (15)Instituto de Investigación Hospital Universitario La Paz (IdiPaz), Madrid, Spain.
  
  
  
  
**TPRG1**
-
4
of
10
  

### CCND1-associated ceRNA network reveal the critical pathway of TPRG1-AS1-hsa-miR-363-3p-MYO1B as a prognostic marker for head and neck squamous cell carcinoma.

4. Sci Rep. 2023 Jul 22;13(1):11831. doi: 10.1038/s41598-023-38847-7
  
  
Li Z(1), Qiu X(2), He Q(3), Fu X(3), Ji F(3), Tian X(1).
  
  
Author information: (1)Department of Pharyngolaryngology Head and Neck Surgery, The First Affiliated ...
  
  
Head and neck squamous cell carcinoma (HNSC) is one of the leading causes of cancer death globally, yet there are few useful biomarkers for early identification and prognostic prediction. Previous studies have confirmed that CCND1 amplification is closely associated with head and neck oncogenesis, and the present study explored the ceRNA network associated with CCND1. Gene expression profiling of the Head and Neck Squamous Cell Carcinoma (HNSC) project of The Cancer Genome Atlas (TCGA) program identified the TPRG1-AS1-hsa-miR-363-3P-MYO1B gene regulatory axis associated with CCND1. Further analysis of the database showed that MYOB was regulated by methylation in head and neck tumors, and functional enrichment analysis showed that MYO1B was involved in "actin filament organization" and "cadherin binding ". Immune infiltration analysis suggested that MYO1B may influence tumorigenesis and prognosis by regulating the immune microenvironment of HNSC. MYO1B enhanced tumor spread through the EMT approach, according to epithelial mesenchymal transition (EMT) characterisation. We analyzed both herbal and GSCALite databases and found that CCND1 and MYO1B have the potential as predictive biomarkers for the treatment of HNSC patients. RT-qPCR validated bioinformatic predictions of gene expression in vitro cell lines. In conclusion, we found a CCND1-related ceRNA network and identified the novel TPRG1-AS1-hsa-miR-363-3p-MYO1B pathway as a possible HNSC diagnostic biomarker and therapeutic target.
  
  
  
  
**TPRG1**
-
5
of
10
  

### LncRNA HOTAIR as a ceRNA is related to breast cancer risk and prognosis.

5. Breast Cancer Res Treat. 2023 Aug;200(3):375-390. doi: 10.1007/s10549-023-06982-4 Epub 2023 Jun 9.
  
  
Qian L(#)(1), Li L(#)(2), Li Y(3), Li S(4), Zhang B(5), Zhu Y(6)(7), Yang B(8).
  
  
Author information: (1)Department of Surgical Oncology, The First Affiliated Hospital of Bengbu Medi ...
  
  
PURPOSE: Breast cancer (BC) is one of the biggest threats to women's health. LncRNA HOTAIR is related to the recurrence and metastasis of BC. Whether HOTAIR can serve as an effective biomarker to distinguish BC patients with different prognosis need to be further studied. METHODS: The miRNA and mRNA expression profile data of BC patients were downloaded from TCGA database. Univariate Cox regression was used to screen differential expression genes (DEGs). The miRcode database and miRWalk database were used to predict miRNA binding to HOTAIR and binding sites of miRNAs, respectively. Kaplan-Meier (KM) analysis was used to estimate the overall survival rate of BC patients. Finally, qRT-PCR and western blot were applied to evaluate the expression level of HOTAIR and mRNAs between BC cells and normal mammary cells. RESULTS: The patients with high HOTAIR expression had poor prognosis in BC. Totally 10 genes correlated with BC prognosis were identified from 170 DEGs, among which PAX7, IYD, ZIC2, MS4A1, TPRXL, CD24, LHX1 were positively correlated with HOTAIR, while CHAD, NPY1R, TPRG1 were opposite. The levels of IYD, ZIC2, CD24 mRNA and protein were increased in BC tissues and BC cells. In BC cells, the levels of IYD, ZIC2 and CD24 mRNA and protein were significantly increased in HOTAIR overexpressed group. HOTAIR had the strongest interaction with hsa-miR-129-5p, followed by hsa-miR-107. CONCLUSION: HOTAIR regulated the expression of downstream genes by interacting with 8 miRNAs and ultimately affected the prognosis of BC patients.
  
  
  
  
**TPRG1**
-
6
of
10
  

### Expression analysis of novel long non-coding RNAs for invasive ductal and invasive lobular breast carcinoma cases.

6. Pathol Res Pract. 2023 Apr;244:154391. doi: 10.1016/j.prp.2023.154391 Epub 2023 Feb 28.
  
  
Aktan Ç(1), Küçükaslan AŞ(1), Türk BA(2), Yildirim I(3).
  
  
Author information: (1)Beykent University, Faculty of Medicine, Department of Medical Biology, 34500 ...
  
  
AIM: Long non-coding RNAs (LncRNAs) serve as important regulatory molecules of gene expression and protein functionality at multiple biological levels, and their deregulation plays a key role in tumorigenesis including in breast cancer metastasis. Therefore, in this study, we aim to compare the expression of novel lncRNAs in the landscape of invasive ductal carcinoma (IDC) and invasive lobular (ILC) carcinoma of breast. MAIN METHODS: We have designed an in-silico approach to find the lncRNAs that regulate the breast cancer. Then, we used the clinical samples to carry out the verification of our in silico finding. In the present study, the tissues of breast cancer were deparaffinized. RNA was extracted by the TRIzole method. After synthesizing cDNA from the extracted RNA, expression levels of lncRNAs were analyzed by qPCR using primers specifically designed and validated for the targeted lncRNAs. In this study, breast biopsy materials from 41 female patients with IDC and 10 female patients with ILC were examined histopathological and expression changes of candidate lncRNAs were investigated in line with the findings. The results were analyzed using IBM SPSS Statistics 25 version. RESULTS: The mean age of the cases was 53.78 ± 14.96. The minimum age was 29, while the maximum age was 87. While 27 of the cases were pre-menopausal, 24 cases were post-menopausal. The number of hormone receptor-positive cases was found to be 40, 35, and 27 for ER, PR, and cerb2/neu, respectively. While the expressions of LINC00501, LINC00578, LINC01209, LINC02015, LINC02584, ABCC5-AS1, PEX5L-AS2, SHANK2-AS3 and SOX2-OT showed significant differences (p < 0.05), the expressions of LINC01206, LINC01994, SHANK2-AS1, and TPRG1-AS2 showed no significant differences (p > 0.05). In addition, it was determined that the regulation of all lncRNAs could be able to involve in the development of cancer such as the NOTCH1, NFKB, and estrogen receptor signalings. CONCLUSION: As a result, it was thought that the discovery of novel lncRNAs might be an important player in the diagnosis, prognosis and therapeutic development of breast cancer.
  
  
  
  
**TPRG1**
-
7
of
10
  

### The oncogenic role of TFAP2A in bladder urothelial carcinoma via a novel long noncoding RNA TPRG1-AS1/DNMT3A/CRTAC1 axis.

7. Cell Signal. 2023 Feb;102:110527. doi: 10.1016/j.cellsig.2022.110527 Epub 2022 Nov 21.
  
  
He J(1), Dong C(2), Zhang H(2), Jiang Y(2), Liu T(2), Man X(3).
  
  
Author information: (1)Department of Surgical Oncology and Breast Surgery, The First Hospital of Chi ...
  
  
BACKGROUND: Overexpression of TFAP2A has been linked to increased lymph node metastasis in basal-squamous bladder cancer. However, its downstream targets in bladder urothelial carcinoma (BLCA), the most malignant cancer of the urinary tract, remain unclear. In the current study, we aim to explore the function and mechanism of TFAP2A in BLCA. METHODS: TFAP2A expression and the prognostic significance in BLCA was analyzed using TCGA and GTEX projects. TFAP2A was knocked-down in BLCA cells to study its impact on glucose uptake, lactate and ATP production, expression of HK2, and the number of vascular meshes formed by HUVEC. The target long noncoding RNAs (lncRNAs) of TFAP2A were predicted by bioinformatics tools, followed by ChIP-qPCR and luciferase assays. The downstream targets of TPRG1-AS1 were analyzed by microarray analysis. Rescue experiments were conducted for validation. RESULTS: TFAP2A upregulation in BLCA predicted dismal survival of patients. Loss of TFAP2A inhibited glycolysis (as evidenced by reduced glucose uptake, lactate, ATP production, and the expression of HK2) and angiogenesis (decreased number of vascular meshes formed by HUVEC). TFAP2A promoted the transcription of TPRG1-AS1. TPRG1-AS1 reversed the inhibitory effect of TFAP2A knockdown on glycolysis and angiogenesis in BLCA cells. TPRG1-AS1 inhibited the transcription of CRTAC1 by recruiting a DNA methyltransferase to the promoter of CRTAC1 and increasing the DNA methylation of its promoter. CRTAC1 inhibited glycolysis and angiogenesis in BLCA cells. TFAP2A silencing curbed tumor growth in vivo via the TPRG1-AS1/CRTAC1 axis. CONCLUSION: TFAP2A reduces CRTAC1 expression by promoting TPRG1-AS1 transcription, thereby expediting BLCA glycolysis and angiogenesis.
  
  
  
  
**TPRG1**
-
8
of
10
  

### Long Noncoding RNA TPRG1-AS1 Suppresses Migration of Vascular Smooth Muscle Cells and Attenuates Atherogenesis via Interacting With MYH9 Protein.

8. Arterioscler Thromb Vasc Biol. 2022 Nov;42(11):1378-1397. doi: 10.1161/ATVBAHA.122.318158 Epub 2022 Sep 29.
  
  
Ren X(#)(1), Zhu H(#)(1), Deng K(1), Ning X(1), Li L(1), Liu D(1), Yang B(1), Shen C(2), Wang X(3), ...
  
  
Author information: (1)Key Laboratory of Cardiovascular Epidemiology and Department of Epidemiology, ...
  
  
BACKGROUND: Migration of human aortic smooth muscle cells (HASMCs) contributes to the pathogenesis of atherosclerosis. This study aims to functionally characterize long noncoding RNA TPRG1-AS1 (tumor protein p63 regulated 1, antisense 1) in HASMCs and reveal the underlying mechanism of TPRG1-AS1 in HASMCs migration, neointima formation, and subsequent atherosclerosis. METHODS: The expression of TPRG1-AS1 in atherosclerotic plaques was verified a series of in silico analysis and quantitative real-time polymerase chain reaction analysis. Northern blot, rapid amplification of cDNA ends and Sanger sequencing were used to determine its full length. In vitro transcription-translation assay was used to investigate the protein-coding capacity of TPRG1-AS1. RNA fluorescent in situ hybridization was used to confirm its subcellular localization. Loss- and gain-of-function studies were used to investigate the function of TPRG1-AS1. Furthermore, the effect of TPRG1-AS1 on the pathological response was evaluated in carotid balloon injury model, wire injury model, and atherosclerosis model, respectively. RESULTS: TPRG1-AS1 was significantly increased in atherosclerotic plaques. TPRG1-AS1 did not encode any proteins and its full length was 1279nt, which was bona fide a long noncoding RNA. TPRG1-AS1 was mainly localized in cytoplasmic and perinuclear regions in HASMCs. TPRG1-AS1 directly interacted with MYH9 (myosin heavy chain 9) protein in HASMCs, promoted MYH9 protein degradation through the proteasome pathway, hindered F-actin stress fiber formation, and finally inhibited HASMCs migration. Vascular smooth muscle cell-specific transgenic overexpression of TPRG1-AS1 significantly reduced neointima formation, and attenuated atherosclerosis in apolipoprotein E knockout (Apoe-/-) mice. CONCLUSIONS: This study demonstrated that TPRG1-AS1 inhibited HASMCs migration through interacting with MYH9 protein and consequently suppressed neointima formation and atherosclerosis.
  
  
  
  
**TPRG1**
-
9
of
10
  

### Tumor protein P63 Regulated 1 contributes to inflammation and cell proliferation of cystitis glandularis through regulating the NF-кB/cyclooxygenase-2/prostaglandin E2 axis.

9. Bosn J Basic Med Sci. 2022 Feb 1;22(1):100-109. doi: 10.17305/bjbms.2021.6763
  
  
Hong T(1), Piao S(2), Sun L(2), Tao Y(2), Ke M(2).
  
  
Author information: (1)Department of Urology, Taizhou Hospital of Zhejiang Province, Shaoxing Univer ...
  
  
Cystitis glandularis is characterized by chronic inflammation and hyperproliferation of bladder mucosa, and contributes to progression of bladder adenocarcinoma. TPRG1 (Tumor Protein P63 Regulated 1) is related to cellular inﬂammatory response, and dysregulation of TPRG1 in tumor tissues is associated with tumor early recurrence. The effect of TPRG1 on cystitis glandularis was investigated in this study. Firstly, bladder specimen were isolated from patients with cystitis glandularis and E. coli-induced cystitis rat. Expression of TPRG1 was found to be up-regulated in the bladder specimen. Moreover, adeno-associated virus (AAV)-mediated silence of TPRG1 was delivered into rat, and data from hematoxylin and eosin (H and E) staining showed that injection with AAV-shTPRG1 ameliorated E. coli-induced histological changes in bladder tissues of rats, and suppressed the inflammatory response. Secondly, TPRG1 was also increased in primary cystitis glandularis cells. Knockdown of TPRG1 decreased cell proliferation of primary cystitis glandularis cells, and suppressed the migration. Thirdly, cyclooxygenase-2 (COX-2) was up-regulated in the bladder specimen isolated from patients with cystitis glandularis and E. coli-induced cystitis rat. Injection with AAV-shTPRG1 reduced protein expression of COX-2, p65 and prostaglandin E2 (PGE2) in the bladder specimen. Lastly, interference of COX-2 attenuated TPRG1 over-expression-induced increase of cell proliferation and migration in the primary cystitis glandularis cells. In conclusion, TPRG1 promoted inflammation and cell proliferation of cystitis glandularis through activation of NF-кB/COX2/PGE2 axis.
  
  
  
  
**TPRG1**
-
10
of
10
  

### Immune Microenvironment Signatures as Biomarkers to Predict Early Recurrence of Stage Ia-b Lung Cancer.

10. Front Oncol. 2021 Jul 28;11:680287. doi: 10.3389/fonc.2021.680287 eCollection 2021.
  
  
Wang Q(1)(2), Zhou D(1)(2), Wu F(3), Liang Q(4), He Q(5), Peng M(1)(2), Yao T(1)(2), Hu Y(1)(2), Qia ...
  
  
Author information: (1)Department of Thoracic Surgery, The Second Xiangya Hospital of Central South ...
  
  
INTRODUCTION: Approximately 30% of patients diagnosed with stage Ia-b NSCLC die of recurrent disease after surgery. This study aimed to identify immune-related biomarkers that might predict tumor recurrence in stage Ia-b NSCLC within 40 months after curative resection. METHODS: Gene expression data of stage Ia-b NSCLC samples was retrieved from the TCGA database, the GEO databases, and the Second Xiangya hospital (XXEYY) database. 22 types of tumors infiltrating immune cells and the expression of immune-associated genes were investigated using CIBERSORT, immunohistochemical staining, and GSEA analyses in a total of 450 patients (80 in the training cohort and 370 in the validation cohorts). Recurrence-related immune features were selected based on the LASSO Cox regression model. RESULTS: High density of Tregs, Macrophages M0 and M1 cell could be observed in recurrence group while the memory B cell was more frequently enriched in controls, yet Tregs alone was significantly associated with tumor early recurrence in TCGA cohort, XYEYY cohort and GSE37745 dataset. A handful of immune-related genes were identified in the recurrence group. Based on Lasso regression analysis, the expressions of five immune-related genes, RLTPR, SLFN13, MIR4500HG, HYDIN and TPRG1 were closely correlated with tumor early recurrence. In the training cohort (TCGA), the combination of these five genes has sensitivity and specificity of 85% and 85%, with AUC of 0.91 (95% CI 0.84-0.98) for lung cancer early recurrence prediction, whereas in validation cohorts, the sensitivity and specificity using this panel was 61-89% and 54-82%, with AUC of 0.62-0.84. CONCLUSION: Our study demonstrated that the immune microenvironment signatures were closely related to tumor early recurrence. Compared to tumor-infiltrating lymphocytes, the expression of five immune-related genes could be robust biomarkers to predict early recurrence of stage Ia-b NSCLC after curative resection.
  
  
  
  
  

---

  

# TTC39B

**Signature gene in:** Stromal cells
  
**Differentially expressed in:** Stromal cells
  
  
**TTC39B**
-
1
of
5
  

### MT1H inhibits the growth of gastric cancer by regulating SLC6A19/TTC39B/ADM2 and activating p53-dependent autophagy.

1. Sci Rep. 2025 Mar 18;15(1):9339. doi: 10.1038/s41598-025-91319-y
  
  
Xing Y(#)(1)(2), Li G(#)(1)(2), Li G(2), Xu J(3), Zhang T(2), Li M(1), Gao C(1)(2), Fu M(4), Zheng P ...
  
  
Author information: (1)Marshall B. J. Medical Research Center, Zhengzhou University, Zhengzhou, 4500 ...
  
  
Metallothioneins (MTs) are a class of cysteine-rich proteins that actively participate in the cellular defense against free radicals. However, owing to the high heterogeneity among different MTs, comprehensive investigations are needed to determine the biological activities and distribution patterns of each MT in different tissues. In the present study, ectopic expression of MT1H significantly inhibited the proliferation of gastric cancer cells. Mechanistically, MT1H was transported into the nucleus and regulated the expression of key genes involved in nutrient transportation and homeostasis, such as SLC6A19, TTC39B, and ADM2, and thereby activating the p53 and autophagy pathways. Additionally, survival analysis of data from the TCGA and other databases revealed that gastric cancer patients with high expression of MT1H had longer survival. Furthermore, MT1H was undetectable in most gastric cell lines, but its expression was increased upon treatment with dexamethasone (Dexa) and the metal ion zinc. Therefore, MT1H emerges as a valuable tumor suppressor, a biomarker for the prognosis, and a promising therapeutic target in gastric cancer patients.
  
  
  
  
**TTC39B**
-
2
of
5
  

### TTC39B destabilizes retinoblastoma protein promoting hepatic lipogenesis in a sex-specific fashion.

2. J Hepatol. 2022 Feb;76(2):383-393. doi: 10.1016/j.jhep.2021.09.021 Epub 2021 Sep 30.
  
  
Hsieh J(1), Molusky MM(2), McCabe KM(2), Fotakis P(2), Xiao T(2), Tascau L(2), Zeana-Schliep L(2), D ...
  
  
Author information: (1)Division of Molecular Medicine, Department of Medicine, Columbia University, ...
  
  
BACKGROUND & AIMS: Molecular mechanisms underlying the different susceptibility of men and women to non-alcoholic fatty liver disease (NAFLD) are poorly understood. The TTC39B locus encodes a scaffolding protein, associates with gynecological disorders and its deletion protects mice from diet-induced steatohepatitis. This study aimed to elucidate the molecular mechanisms linking TTC39B (T39) to the expression of lipogenic genes and to explore sex-specific effects. METHODS: Co-expression in HEK293A cells validated the novel T39/pRb interaction predicted by a protein-protein interaction algorithm. T39 was knocked down using an antisense oligonucleotide (ASO) in mice with dietary NAFLD and a genetic deficiency of pRb or its downstream effector E2F1, as well as in primary human hepatocytes. RESULTS: T39 interacts with pRb via its C-terminal TPR domain and promotes its proteasomal degradation. In female mice, T39 deficiency reduces the mRNA of lipogenic genes, especially Pnpla3, in a pRb- and E2F1-dependent manner. In contrast, in male mice, T39 deficiency results in a much smaller reduction in lipogenic gene expression that is independent of pRb/E2F1. T39 also interacts with VAPB via an N-terminal FFAT motif and stabilizes the interaction of VAPB with SCAP. Ovariectomy abolishes the effect of T39 knockdown on the hepatic pRb/E2F1/Pnpla3 axis. In both sexes T39 knockdown reduces SCAP independently of pRb. In primary human hepatocytes, T39 knockdown reduces expression of PNPLA3 and other lipogenic genes in women but not men. CONCLUSIONS: We have uncovered a conserved sexual dimorphism in the regulation of hepatic lipogenic genes, with effects of T39 mediated through pRb/E2F1 in females and VAPB/SCAP in both sexes. T39 inhibition could be a novel strategy to downregulate PNPLA3 and treat NAFLD in women. LAY SUMMARY: In females, the protein TTC39B degrades a tumor suppressor in the liver to promote the synthesis of new fat and the expression of a major genetic risk factor for non-alcoholic fatty liver disease. TTC39B is a potential therapeutic target for non-alcoholic fatty liver disease, especially in women.
  
  
  
  
**TTC39B**
-
3
of
5
  

### BRCA2 loss-of-function germline mutations are associated with esophageal squamous cell carcinoma risk in Chinese.

3. Int J Cancer. 2020 Feb 15;146(4):1042-1051. doi: 10.1002/ijc.32619 Epub 2019 Aug 26.
  
  
Ko JM(1), Ning L(1), Zhao XK(2), Chai AWY(1), Lei LC(1), Choi SSA(1), Tao L(1), Law S(3), Kwong A(3) ...
  
  
Author information: (1)Department of Clinical Oncology, University of Hong Kong, Hong Kong, People's ...
  
  
Esophageal squamous cell carcinoma (ESCC) occurs with highest frequency in China with over 90% mortality, highlighting the need for early detection and improved treatment strategies. We aimed to identify ESCC cancer predisposition gene(s). Our study included 4,517 individuals. The discovery phase using whole-exome sequencing (WES) included 186 familial ESCC patients from high-risk China. Targeted gene sequencing validation of 598 genes included 3,289 Henan and 1,228 moderate-risk Hong Kong Chinese. A WES approach identified BRCA2 loss-of-function (LOF) mutations in 3.23% (6/186) familial ESCC patients compared to 0.21% (9/4300) in the ExAC East Asians (odds ratio [OR] = 15.89, p = 2.48 × 10^-10^ ). BRCA2 LOF mutation frequency in the combined Henan cohort has significantly higher prevalence (OR = 10.55, p = 0.0035). Results were independently validated in an ESCC Hong Kong cohort (OR = 10.64, p = 0.022). One Hong Kong pedigree was identified to carry a BRCA2 LOF mutation. BRCA2 inactivation in ESCC was via germline LOF mutations and wild-type somatic allelic loss via loss of heterozygosity. Gene-based association analysis, including LOF mutations and rare deleterious missense variants defined with combined annotation dependent depletion score ≥30, confirmed the genetic predisposition role of BRCA2 (OR = 9.50, p = 3.44 × 10^-5^ ), and provided new evidence for potential association of ESCC risk with DNA repair genes (POLQ and MSH2), inflammation (TTC39B) and angiogenesis (KDR). Our findings are the first to provide compelling evidence of the role of BRCA2 in ESCC genetic susceptibility in Chinese, suggesting defective homologous recombination is an underlying cause in ESCC pathogenesis, which is amenable to therapeutic options based on synthetic lethality approaches such as targeting BRCA2 with PARP1 inhibitors in ESCC.
  
  
  
  
**TTC39B**
-
4
of
5
  

### (-)-Epigallocatechin-3-Gallate Ameliorates Atherosclerosis and Modulates Hepatic Lipid Metabolic Gene Expression in Apolipoprotein E Knockout Mice: Involvement of TTC39B.

4. Front Pharmacol. 2018 Mar 9;9:195. doi: 10.3389/fphar.2018.00195 eCollection 2018.
  
  
Wang W(1), Zhang ZZ(2), Wu Y(1), Wang RQ(1), Chen JW(1), Chen J(1), Zhang Y(1), Chen YJ(1), Geng M(1 ...
  
  
Author information: (1)School of Life Sciences, Hefei Normal University, Hefei, China. (2)State Key ...
  
  
Erratum in Front Pharmacol. 2018 May 01;9:459. doi: 10.3389/fphar.2018.00459.
  
  
  
  
**TTC39B**
-
5
of
5
  

### Germline polymorphisms in an enhancer of PSIP1 are associated with progression-free survival in epithelial ovarian cancer.

5. Oncotarget. 2016 Feb 9;7(6):6353-68. doi: 10.18632/oncotarget.7047
  
  
French JD(1), Johnatty SE(1), Lu Y(1), Beesley J(1), Gao B(2), Kalimutho M(1), Henderson MJ(3), Russ ...
  
  
Author information: (1)QIMR Berghofer Medical Research Institute, Brisbane, Australia. (2)Department ...
  
  
Women with epithelial ovarian cancer (EOC) are usually treated with platinum/taxane therapy after cytoreductive surgery but there is considerable inter-individual variation in response. To identify germline single-nucleotide polymorphisms (SNPs) that contribute to variations in individual responses to chemotherapy, we carried out a multi-phase genome-wide association study (GWAS) in 1,244 women diagnosed with serous EOC who were treated with the same first-line chemotherapy, carboplatin and paclitaxel. We identified two SNPs (rs7874043 and rs72700653) in TTC39B (best P=7x10^-5^, HR=1.90, for rs7874043) associated with progression-free survival (PFS). Functional analyses show that both SNPs lie in a putative regulatory element (PRE) that physically interacts with the promoters of PSIP1, CCDC171 and an alternative promoter of TTC39B. The C allele of rs7874043 is associated with poor PFS and showed increased binding of the Sp1 transcription factor, which is critical for chromatin interactions with PSIP1. Silencing of PSIP1 significantly impaired DNA damage-induced Rad51 nuclear foci and reduced cell viability in ovarian cancer lines. PSIP1 (PC4 and SFRS1 Interacting Protein 1) is known to protect cells from stress-induced apoptosis, and high expression is associated with poor PFS in EOC patients. We therefore suggest that the minor allele of rs7874043 confers poor PFS by increasing PSIP1 expression.
  
  
  
  
  

---

  

# UEVLD

**Signature gene in:** Stromal cells
  
**Differentially expressed in:** Stromal cells
  
  
**UEVLD**
-
1
of
1
  

### Targeted next generation sequencing identifies somatic mutations and gene fusions in papillary thyroid carcinoma.

1. Oncotarget. 2017 Jul 11;8(28):45784-45792. doi: 10.18632/oncotarget.17412
  
  
Lu Z(1), Zhang Y(2), Feng D(2), Sheng J(2), Yang W(1)(3), Liu B(2).
  
  
Author information: (1)Key Laboratory of Carcinogenesis and Translational Research (Ministry of Educ ...
  
  
138 papillary thyroid carcinoma (PTC) samples were assessed for somatic mutation profile and fusion genes by targeted resequencing using a cancer panel (ThyGenCapTM) targeting 244 cancer-related genes and 20 potential fusion genes. At least one genetic alteration (including mutations and fusion genes) was observed in 118/138 (85.5%) samples. The most frequently mutated gene was BRAF V600E (57.2%). Moreover, we identified 11 fusion genes including eight previously reported ones and three novel fusion genes, UEVLD-RET, OSBPL9-BRAF, and SQSTM1-NTRK3. Alterations affecting the mitogen-activated protein kinase (MAPK) signaling pathway components were seen in 69.6% of the PTC cases and all of these driver mutations were mutually exclusive. Univariate analysis ascertained that the fusion genes were strongly associated with distinct clinicopathological characteristics, such as young age, local invasion, extensive metastasis, and disease stage. In conclusion, our approach facilitated simultaneous high-throughput detection of gene fusions and somatic mutations in PTC samples.
  
  
  
  
  

---

  

# ABCB1

**Signature gene in:** Endothelial cells
  
**Differentially expressed in:** Endothelial cells
  
  
**ABCB1**
-
1
of
10
  

### Growth Hormone Upregulates Melanoma Drug Resistance and Migration via Melanoma-Derived Exosomes.

1. Cancers (Basel). 2024 Jul 24;16(15):2636. doi: 10.3390/cancers16152636
  
  
Kulkarni P(1)(2)(3), Basu R(1), Bonn T(1)(4), Low B(1)(3), Mazurek N(1)(5), Kopchick JJ(1)(2)(6).
  
  
Author information: (1)Edison Biotechnology Institute, Ohio University, Athens, OH 45701, USA. (2)Mo ...
  
  
Drug resistance in melanoma is a major hindrance in cancer therapy. Growth hormone (GH) plays a pivotal role in contributing to the resistance to chemotherapy. Knocking down or blocking the GH receptor has been shown to sensitize the tumor cells to chemotherapy. Extensive studies have demonstrated that exosomes, a subset of extracellular vesicles, play an important role in drug resistance by transferring key factors to sensitize cancer cells to chemotherapy. In this study, we explore how GH modulates exosomal cargoes from melanoma cells and their role in drug resistance. We treated the melanoma cells with GH, doxorubicin, and the GHR antagonist, pegvisomant, and analyzed the exosomes released. Additionally, we administered these exosomes to the recipient cells. The GH-treated melanoma cells released exosomes with elevated levels of ABC transporters (ABCC1 and ABCB1), N-cadherin, and MMP2, enhancing drug resistance and migration in the recipient cells. GHR antagonism reduced these exosomal levels, restoring drug sensitivity and attenuating migration. Overall, our findings highlight a novel role of GH in modulating exosomal cargoes that drive chemoresistance and metastasis in melanoma. This understanding provides insights into the mechanisms of GH in melanoma chemoresistance and suggests GHR antagonism as a potential therapy to overcome chemoresistance in melanoma treatment.
  
  
  
  
**ABCB1**
-
2
of
10
  

### The Blocking of Drug Resistance Channels by Selected Hydrophobic Statins in Chemoresistance Human Melanoma.

2. Biomolecules. 2023 Nov 21;13(12):1682. doi: 10.3390/biom13121682
  
  
Placha W(1), Suder P(2), Panek A(3), Bronowicka-Adamska P(1), Zarzycka M(1), Szczygieł M(4), Zagajew ...
  
  
Author information: (1)Department of Biochemistry, Faculty of Medicine, Jagiellonian University Medi ...
  
  
Despite the development of modern drugs, drug resistance in oncology remains the main factor limiting the curability of patients. This paper shows the use of a group of hydrophobic statins to inhibit drug resistance (Pgp protein). In a chemoresistance melanoma cell model, viability, necroptosis with DNA damage, the absorption of the applied pharmaceuticals, and the functional activity of the ABCB1 drug transporter after administration of docetaxel or docetaxel with a selected hydrophobic statin were studied. Taxol-resistant human melanoma cells from three stages of development were used as a model: both A375P and WM239A metastatic lines and radial growth phase WM35 cells. An animal model (Mus musculus SCID) was developed for the A375P cell line. The results show that hydrophobic statins administered with docetaxel increase the accumulation of the drug in the tumor cell a.o. by blocking the ABCB1 channel. They reduce taxol-induced drug resistance. The tumor size reduction was observed after the drug combination was administrated. It was shown that the structural similarity of statins is of secondary importance, e.g., pravastatin and simvastatin. Using cytostatics in the presence of hydrophobic statins increases their effectiveness while reducing their overall toxicity.
  
  
  
  
**ABCB1**
-
3
of
10
  

### Acquired and intrinsic resistance to vemurafenib in BRAF(V600E) -driven melanoma brain metastases.

3. FEBS Open Bio. 2024 Jan;14(1):96-111. doi: 10.1002/2211-5463.13730 Epub 2023 Nov 30.
  
  
Zhang P(1)(2)(3), Kuil LE(1)(4), Buil LCM(1)(5), Freriks S(1)(5), Beijnen JH(6)(7), van Tellingen O( ...
  
  
Author information: (1)Division of Pharmacology, The Netherlands Cancer Institute, Amsterdam, The Ne ...
  
  
BRAFV600 -mutated melanoma brain metastases (MBMs) are responsive to BRAF inhibitors, but responses are generally less durable than those of extracranial metastases. We tested the hypothesis that the drug efflux transporters P-glycoprotein (P-gp; ABCB1) and breast cancer resistance protein (BCRP; ABCG2) expressed at the blood-brain barrier (BBB) offer MBMs protection from therapy. We intracranially implanted A375 melanoma cells in wild-type (WT) and Abcb1a/b;Abcg2-/- mice, characterized the tumor BBB, analyzed drug levels in plasma and brain lesions after oral vemurafenib administration, and determined the efficacy against brain metastases and subcutaneous lesions. Although contrast-enhanced MRI demonstrated that the integrity of the BBB is disrupted in A375 MBMs, vemurafenib achieved greater antitumor efficacy against MBMs in Abcb1a/b;Abcg2-/- mice compared with WT mice. Concordantly, P-gp and BCRP are expressed in MBM-associated brain endothelium both in patients and in A375 xenografts and expression of these transporters limited vemurafenib penetration into A375 MBMs. Although initially responsive, A375 MBMs rapidly developed therapy resistance, even in Abcb1a/b;Abcg2-/- mice, and this was unrelated to pharmacokinetic or target inhibition issues. Taken together, we demonstrate that both intrinsic and acquired resistance can play a role in MBMs.
  
  
  
  
**ABCB1**
-
4
of
10
  

### Evolving Acquired Vemurafenib Resistance in a BRAF V600E Mutant Melanoma PDTX Model to Reveal New Potential Targets.

4. Cells. 2023 Jul 24;12(14):1919. doi: 10.3390/cells12141919
  
  
Tóvári J(1)(2), Vári-Mező D(1)(2), Surguta SE(1)(2), Ladányi A(2)(3), Kigyós A(4), Cserepes M(1)(2).
  
  
Author information: (1)Department of Experimental Pharmacology, National Institute of Oncology, 1122 ...
  
  
Malignant melanoma is challenging to treat, and metastatic cases need chemotherapy strategies. Targeted inhibition of commonly mutant BRAF V600E by inhibitors is efficient but eventually leads to resistance and progression in the vast majority of cases. Numerous studies investigated the mechanisms of resistance in melanoma cell lines, and an increasing number of in vivo or clinical data are accumulating. In most cases, bypassing BRAF and resulting reactivation of the MAPK signaling, as well as alternative PI3K-AKT signaling activation are reported. However, several unique changes were also shown. We developed and used a patient-derived tumor xenograft (PDTX) model to screen resistance evolution in mice in vivo, maintaining tumor heterogeneity. Our results showed no substantial activation of the canonical pathways; however, RNAseq and qPCR data revealed several altered genes, such as GPR39, CD27, SLC15A3, IFI27, PDGFA, and ABCB1. Surprisingly, p53 activity, leading to apoptotic cell death, was unchanged. The found biomarkers can confer resistance in a subset of melanoma patients via immune modulation, microenvironment changes, or drug elimination. Our resistance model can be further used in testing specific inhibitors that could be used in future drug development, and combination therapy testing that can overcome inhibitor resistance in melanoma.
  
  
  
  
**ABCB1**
-
5
of
10
  

### Complex mixtures of pesticides and metabolites modulate the malignant phenotype of murine melanoma B16-F1 cells.

5. Environ Sci Pollut Res Int. 2023 Apr;30(16):47366-47380. doi: 10.1007/s11356-023-25603-7 Epub 2023 Feb 4.
  
  
de Almeida Roque A(1), da Luz JZ(1), Santurio MTK(1), Neto FF(1), de Oliveira Ribeiro CA(2).
  
  
Author information: (1)Laboratory of Cell Toxicology, Department of Cellular and Molecular Biology, ...
  
  
Pesticides use increased worldwide with a record in Brazil. Although several works addressed the effects of pesticides on living organisms, only a few considered their mixture, and even fewer tried to unravel their role in tumoral progression. Due to the relevance of cancer, in the present study, the effects of the mixture of pesticides widely used in Brazil (Glyphosate, 2,4-dichlorophenoxyacetic acid, Mancozeb, Atrazine, Acephate, and Paraquat) and their main metabolites (Aminomethylphosphonic Acid, 2,4-diclorophenol, Ethylenethiourea, Desethylatrazine, Methamidophos, and Paraquat) were investigated on the malignancy phenotype of murine melanoma B16-F1 cells after acute (24 h) and chronic (15 days) exposures. The tested concentrations were based on the Acceptable Daily Intake (ADI) value established by Brazilian legislation. The set of results showed that these chemicals modulate important parameters of tumor progression, affecting the expression of genes related to tumor aggressiveness (Mmp14 and Cd44) and multidrug resistance (ABCB1, Abcc1, and Abcc4), as well as tissue inhibitors of metalloproteinases (Timp1, Timp2, and Timp3). These findings revealed an absence of cytotoxicity but showed modulation of migration, invasion, and colonization capacity of B16-F1 cells. Together, the results point to some negative ways that exposure to pesticides can affect the progression of melanoma and raise a concern related to the increasing trend in pesticide use in Brazil, as the country is one of the major world food suppliers.
  
  
  
  
**ABCB1**
-
6
of
10
  

### Encorafenib Acts as a Dual-Activity Chemosensitizer through Its Inhibitory Effect on ABCC1 Transporter In Vitro and Ex Vivo.

6. Pharmaceutics. 2022 Nov 24;14(12):2595. doi: 10.3390/pharmaceutics14122595
  
  
Zhang Y(1), Vagiannis D(1), Budagaga Y(1), Sabet Z(1), Hanke I(2), Rozkoš T(3), Hofman J(1).
  
  
Author information: (1)Department of Pharmacology and Toxicology, Faculty of Pharmacy in Hradec Král ...
  
  
Encorafenib (LGX818, trade name Braftovi), a novel BRAF inhibitor, has been approved for the treatment of melanoma and colorectal cancer. In the present work, we evaluated encorafenib's possible antagonistic effects on the pharmacokinetic mechanisms of multidrug resistance (MDR), as well as its perpetrator role in drug interactions. Firstly, encorafenib potently inhibited the efflux function of the ABCC1 transporter in drug accumulation assays, while moderate and null interaction levels were recorded for ABCB1 and ABCG2, respectively. In contrast, the mRNA expression levels of all the tested transporters were not altered by encorafenib. In the drug combination studies, we found that daunorubicin and topotecan resistances were synergistically attenuated by the encorafenib-mediated interaction in A431-ABCC1 cells. Notably, further experiments in ex vivo patient-derived explants confirmed the MDR-modulating ability of encorafenib. Advantageously, the overexpression of tested drug efflux transporters failed to hinder the antiproliferative activity of encorafenib. In addition, no significant modulation of the CYP3A4 enzyme's activity by encorafenib was observed. In conclusion, our work indicated that encorafenib can act as an effective chemosensitizer targeting the ABCC1-induced MDR. Our in vitro and ex vivo data might provide valuable information for designing the novel effective scheme applicable in the clinical pharmacotherapy of BRAF-mutated/ABCC1-expressing tumors.
  
  
  
  
**ABCB1**
-
7
of
10
  

### Heme Oxygenase-1 Has a Greater Effect on Melanoma Stem Cell Properties Than the Expression of Melanoma-Initiating Cell Markers.

7. Int J Mol Sci. 2022 Mar 25;23(7):3596. doi: 10.3390/ijms23073596
  
  
Kusienicka A(1), Bukowska-Strakova K(1)(2), Cieśla M(1), Nowak WN(1), Bronisz-Budzyńska I(1), Seretn ...
  
  
Author information: (1)Department of Medical Biotechnology, Faculty of Biophysics, Biochemistry and ...
  
  
Melanoma-initiating cells (MICs) contribute to the tumorigenicity and heterogeneity of melanoma. MICs are identified by surface and functional markers and have been shown to display cancer stem cell (CSC) properties. However, the existence of MICs that follow the hierarchical CSC model has been questioned by studies showing that single unselected melanoma cells are highly tumorigenic in xenotransplantation assays. Herein, we characterize cells expressing MIC markers (CD20, CD24, CD133, Sca-1, ABCB1, ABCB5, ALDHhigh) in the B16-F10 murine melanoma cell line. We use flow cytometric phenotyping, single-cell sorting followed by in vitro clonogenic assays, and syngeneic in vivo serial transplantation assays to demonstrate that the expression of MIC markers does not select CSC-like cells in this cell line. Previously, our group showed that heme-degrading enzyme heme oxygenase-1 (HO-1) can be upregulated in melanoma and increase its aggressiveness. Here, we show that HO-1 activity is important for non-adherent growth of melanoma and HO-1 overexpression enhances the vasculogenic mimicry potential, which can be considered protumorigenic activity. However, HO-1 overexpression decreases clone formation in vitro and serial tumor initiation in vivo. Thus, HO-1 plays a dual role in melanoma, improving the progression of growing tumors but reducing the risk of melanoma initiation.
  
  
  
  
**ABCB1**
-
8
of
10
  

### Spheroid-induced heterogeneity and plasticity of uveal melanoma cells.

8. Cell Oncol (Dordr). 2022 Apr;45(2):309-321. doi: 10.1007/s13402-022-00671-y Epub 2022 Apr 11.
  
  
Chen Y(1), Lu X(2), Gao L(3), Dean DC(4), Liu Y(5).
  
  
Author information: (1)Hunan Key Laboratory of Ophthalmology, Eye Center of Xiangya Hospital, Centra ...
  
  
PURPOSE: The mechanism underlying cancer heterogeneity and plasticity remains elusive, in spite of the fact that multiple hypotheses have been put forward. We intended to clarify this heterogeneity in uveal melanoma (UM) by looking for evidence of cancer stem cell involvement and a potential role of ZEB1 in cancer cell plasticity. METHODS: Spheroids derived from human UM cells as well as xenograft tumors in nude mice were dissected for signs of heterogeneity and plasticity. Two human UM cell lines were studied: the epithelioid type C918 cell line and the spindle type OCM1 cell line. We knocked down ZEB1 in both cell lines to investigate its involvement in the regulation of stem-like cell formation and vascularization by qRT-PCR, immunohistochemistry, flow cytometry, electrophoretic mobility shift assay (EMSA) and chromatin immunoprecipitation (ChIP) assays. RESULTS: We found that a small side population (SP) in OCM1 showed stem cell-like properties such as heterogeneity, remote dissemination and nuclear dye exclusion after spheroid formation in vitro. ZEB1 regulated UM stem cell generation indirectly by promoting cell proliferation to form large size tumors in vivo and spheroid in vitro, and directly by binding to stemness genes such as TERT and ABCB1. In addition, we found that ZEB1 participates in vasculogenic mimicry system formation through the regulation of CD34 and VE-cadherin expression. CONCLUSIONS: From our data we conclude that cancer stem cells may contribute to UM heterogeneity and plasticity and that ZEB1 may play a regulatory role in it.
  
  
  
  
**ABCB1**
-
9
of
10
  

### Dabrafenib inhibits ABCG2 and cytochrome P450 isoenzymes; potential implications for combination anticancer therapy.

9. Toxicol Appl Pharmacol. 2022 Jan 1;434:115797. doi: 10.1016/j.taap.2021.115797 Epub 2021 Nov 13.
  
  
Sorf A(1), Vagiannis D(1), Ahmed F(1), Hofman J(1), Ceckova M(2).
  
  
Author information: (1)Department of Pharmacology and Toxicology, Faculty of Pharmacy in Hradec Kral ...
  
  
Dabrafenib is a BRAF inhibitor used in combination treatment of malignant melanoma and non-small cell lung carcinoma. In this study, we aimed to characterize its interactions with cytochrome P450 (CYP) isoenzymes and ATP-binding cassette (ABC) efflux transporters that have critical impact on the pharmacokinetics of drugs and play a role in drug resistance development. Using accumulation assays, we showed that dabrafenib inhibited ABCG2 and, less potently, ABCB1 transporter. We also confirmed dabrafenib as a CYP2C8, CYP2C9, CYP3A4, and CYP3A5 inhibitor. Importantly, inhibition of ABCG2 and CYP3A4 by dabrafenib led to the potentiation of cytotoxic effects of mitoxantrone and docetaxel toward respective resistant cell lines in drug combination studies. On the contrary, the synergistic effect was not consistently observed in ABCB1-expressing models. We further demonstrated that mRNA levels of ABCB1, ABCG2, ABCC1, and CYP3A4 were increased after 24 h and 48 h exposure to dabrafenib. Overall, our data confirm dabrafenib as a drug frequently and potently interacting with ABC transporters and CYP isoenzymes. This feature should be addressed with caution when administering dabrafenib to patients with polypharmacy but also could be utilized advantageously when designing new dabrafenib-containing drug combinations to improve the therapeutic outcome in drug-resistant cancer.
  
  
  
  
**ABCB1**
-
10
of
10
  

### ABCB1 in dermatology: roles in skin diseases and their treatment.

10. J Mol Med (Berl). 2021 Nov;99(11):1527-1538. doi: 10.1007/s00109-021-02105-y Epub 2021 Aug 9.
  
  
Weng HJ(1)(2)(3), Tsai TF(4).
  
  
Author information: (1)Department of Dermatology, Taipei Medical University-Shuang Ho Hospital, New ...
  
  
Adenosine triphosphate-binding cassette subfamily B member 1 (ABCB1), also known as permeability glycoprotein, multidrug-resistant protein 1, or cluster of differentiation 243 (CD243), is a crucial protein for purging foreign substances from cells. The functions of ABCB1 have been investigated extensively for their roles in cancer, stem cells, and drug resistance. Abundant pharmacogenetic studies have been conducted on ABCB1 and its association with treatment responsiveness to various agents, particularly chemotherapeutic and immunomodulatory agents. However, its functions in the skin and implications on dermatotherapeutics are far less reported. In this article, we reviewed the roles of ABCB1 in dermatology. ABCB1 is expressed in the skin and its appendages during drug delivery and transport. It is associated with treatment responsiveness to various agents, including topical steroids, methotrexate, cyclosporine, azathioprine, antihistamines, antifungal agents, colchicine, tacrolimus, ivermectin, tetracycline, retinoid acids, and biologic agents. Moreover, genetic variation in ABCB1 is associated with the pathogenesis of several dermatoses, including psoriasis, atopic dermatitis, melanoma, bullous pemphigoid, Behçet disease, and lichen planus. Further investigation is warranted to elucidate the roles of ABCB1 in dermatology and the possibility of enhancing therapeutic efficacy through ABCB1 manipulation.
  
  
  
  
  

---

  

# ADIPOR2

**Signature gene in:** Endothelial cells
  
**Differentially expressed in:** Endothelial cells
  
  
**ADIPOR2**
-
1
of
1
  

### Adiponectin receptor expression in human malignant tissues.

1. Horm Cancer. 2010 Jun;1(3):136-45. doi: 10.1007/s12672-010-0017-7
  
  
Chou SH(1), Tseleni-Balafouta S, Moon HS, Chamberland JP, Liu X, Kavantzas N, Mantzoros CS.
  
  
Author information: (1)Division of Endocrinology, Diabetes, & Metabolism, Department of Medicine, Be ...
  
  
Adiponectin has been proposed to be a mediator of obesity-associated malignancies and to have direct antineoplastic effects acting via adiponectin receptors AdipoR1 and ADIPOR2. We describe herein the expression of AdipoR1 and ADIPOR2 in several cancers not previously studied. We used immunohistochemistry to assess expression of adiponectin receptors in archival specimens of renal cell carcinoma (n = 64), hepatocellular carcinoma (n = 123), melanoma (n = 20), cholangiocarcinoma (n = 20), transitional cell carcinoma of the bladder (n = 24), ovarian epithelial carcinoma (n = 63), cervical squamous cell carcinoma (n = 49), and adrenocortical carcinoma (n = 48). To compare expression in malignant versus nonmalignant tissues, we also studied AdipoR1 and ADIPOR2 expression in pairs of renal cell carcinoma and adjacent healthy kidney tissue specimens by immunohistochemistry. We also studied mRNA expression in 45 specimens of renal cell carcinoma by real-time polymerase chain reaction. Finally, we utilized Western blotting to confirm the presence of adiponectin receptors and subsequently studied cell signaling pathways of adiponectin in the renal cancer cell line 786-O. Cancers associated with obesity were significantly more likely to express AdipoR1 than cancers not associated with obesity. Of the specimens of renal cell carcinoma, which is strongly associated with obesity, 93.8% expressed AdipoR1 compared to 44.9% of the specimens of cervical cell carcinoma, which is not associated with obesity (p < 0.001). There was no difference in the expression of adiponectin receptors or their mRNA between malignant and benign kidney tissue specimens. Overall, there were no correlations between expression of adiponectin receptors or their mRNA and tumor prognostic factors. Finally, Western blotting confirmed the presence of AdipoR1 in the renal cancer cell line 786-O, and adiponectin activates in vitro several signaling pathways in this cell line. In summary, we report for the first time expression of AdipoR1 and ADIPOR2 in the above cancers and that AdipoR1 is more ubiquitously expressed in obesity-associated cancers.
  
  
  
  
  

---

  

# AGO4

**Signature gene in:** Stromal cells
  
**Differentially expressed in:** Stromal cells
  
  
**AGO4**
-
1
of
1
  

### Identifying an lncRNA-Related ceRNA Network to Reveal Novel Targets for a Cutaneous Squamous Cell Carcinoma.

1. Biology (Basel). 2021 May 13;10(5):432. doi: 10.3390/biology10050432
  
  
Xu Y(1), Dong Y(1), Deng Y(1), Qi Q(2), Wu M(3), Liang H(3), She Q(1), Guo Q(3).
  
  
Author information: (1)Department of Dermatology, Tongji Medical College, Huazhong University of Sci ...
  
  
A cutaneous squamous cell carcinoma (cSCC) derived from keratinocytes is the second most common cause of non-melanoma skin cancer. The accumulation of the mutational burden of genes and cellular DNA damage caused by the risk factors (e.g., exposure to ultraviolet radiation) contribute to the aberrant proliferation of keratinocytes and the formation of a cSCC. A cSCC encompasses a spectrum of diseases that range from recursor actinic keratosis (AK) and squamous cell carcinoma (SCC) in situ (SCCIS) to invasive cSCCs and further metastatic SCCs. Emerging evidence has revealed that lncRNAs are involved in the biological process of a cSCC. According to the ceRNA regulatory theory, lncRNAs act as natural miRNA sponges and interact with miRNA response elements, thereby regulating the mRNA expression of their down-stream targets. This study was designed to search for the potential lncRNAs that may become potential therapeutic targets or biomarkers of a cSCC. Considering the spirit of the study to be adequately justified, we collected microarray-based datasets of 19 cSCC tissues and 12 normal skin samples from the GEO database (GSE42677 and GSE45164). After screening the differentially expressed genes via a limma package, we identified 24 differentially expressed lncRNAs (DElncRNAs) and 3221 differentially expressed mRNAs (DEmRNAs). The miRcode, miRTarBase, miRDB and TargetScan databases were used to predict miRNAs that could interact with DElncRNAs and DEmRNAs. A total of 137 miRNA-lncRNA and 221 miRNA-mRNA pairs were retained in the ceRNA network, consisting of 31 miRNAs, 11 DElncRNAs and 155 DEmRNAs. For the functional analysis, the top enriched biological process was enhancer sequence-specific DNA binding in Gene Ontology (GO) terms. The FoxO signaling pathway, autophagy and cellular senescence were the top enrichment terms based on a Kyoto Encyclopedia of Genes and Genomes (KEGG) analysis. The combination of a STRING tool and Cytoscape software (plug-in MCODE) identified five core mRNAs and built a core mRNA-associated ceRNA network. The expression for five identified core mRNAs and their related nine lncRNAs was validated using the external dataset GSE7553. Finally, one lncRNA HLA-F-AS1 and three mRNAs named AGO4, E2F1 and CCND1 were validated with the same expression patterns. We speculate that lncRNA HLA-F-AS1 may sponge miR-17-5p or miR-20b-5p to regulate the expression of CCND1 and E2F1 in the cSCC. The present study may provide potential diagnostic and therapeutic targets for cSCC patients.
  
  
  
  
  

---

  

# ATF3

**Signature gene in:** Stromal cells
  
**Differentially expressed in:** Stromal cells
  
  
**ATF3**
-
1
of
10
  

### Thiotaurine inhibits melanoma progression by enhancing Ca(2+) overload-induced cellular apoptosis.

1. J Dermatol Sci. 2025 Apr;118(1):29-37. doi: 10.1016/j.jdermsci.2025.03.001 Epub 2025 Mar 15.
  
  
Wang D(1), Xie A(1), Luo J(2), Li L(3), Zhang Z(1), Deng W(1), Yang B(1), Chang Y(4), Liang Y(5).
  
  
Author information: (1)Dermatology Hospital, Southern Medical University, Guangzhou, China. (2)Depar ...
  
  
BACKGROUND: Melanoma is the most dangerous type of skin cancer with poor therapy outcomes. Since malignant cells are more susceptible to Ca2+ overload than normal cells, activating Ca2+ overload-mediated apoptosis may be a promising strategy to inhibit melanoma progression. Hydrogen sulfide (H2S) donors can regulate Ca2+ channels, but their effects on melanoma cells remain unclear. OBJECTIVE: To explore the effects of Thiotaurine (TTAU), an H2S donor, on melanoma cells and its underlying mechanisms. METHODS: We tested the effect of TTAU by culturing melanoma cells in vitro and establishing the xenograft model of mice in vivo. Cell proliferation and apoptosis were assessed using the CCK-8 test and flow cytometry. Molecules involved in apoptosis or Ca2+-related signal transduction were analyzed by western blotting. Immunofluorescence was used to measure Ca2+ levels, mitochondrial damage, and reactive oxygen species (ROS). RESULTS: TTAU significantly reduced melanoma cell viability and induced apoptosis both in vitro and in vivo. Mechanistically, TTAU increased intracellular Ca2+, upregulated transient receptor potential vanilloid 1(TRPV1), and decreased activating transcription factor 3(ATF3) by nuclear factor of activated T cell cytoplasmic 1(NFATc1). TTAU also caused mitochondrial damage and ROS overproduction, which also promoted apoptosis. CONCLUSION: We first elucidate that TTAU inhibits melanoma progression by activating Ca2+ influx-NFATc1-ATF3 signaling and aggravating mitochondrial oxidative stress, in which TRPV1 may act as an amplifier for Ca2+ influx. Our research is expected to provide new ideas for the treatment of tumors such as melanoma, as well as the clinical application of reactive sulfur species-based drugs.
  
  
  
  
**ATF3**
-
2
of
10
  

### ATF3 regulates CDC42 transcription and influences cytoskeleton remodeling, thus inhibiting the proliferation, migration and invasion of malignant skin melanoma cells.

2. Melanoma Res. 2025 Feb 1;35(1):37-49. doi: 10.1097/CMR.0000000000001011 Epub 2024 Nov 26.
  
  
Niu L(1), Liu S(2), Shen J(3), Chang J(3), Li X(1), Zhang L(1).
  
  
Author information: (1)Department of Dermatology, Affiliated Hospital of Hebei Engineering Universit ...
  
  
Cutaneous malignant melanoma (CMM) is one of the most aggressive and lethal types of skin cancer. Cytoskeletal remodeling is a key factor in the progression of CMM. Previous research has shown that activating transcription factor 3 (ATF3) inhibits metastasis in bladder cancer by regulating actin cytoskeleton remodeling through gelsolin. However, whether ATF3 plays a similar role in cytoskeletal remodeling in CMM cells remains unknown. Various gene and protein expression analyses were performed using techniques such as reverse transcription quantitative PCR, western blot, immunofluorescent staining, and immunohistochemical staining. CMM viability, migration, and invasion were examined through cell counting kit-8 and transwell assays. The interactions between cell division cycle 42 (CDC42) and ATF3 were investigated using chromatin immunoprecipitation and dual-luciferase reporter assays. CDC42 was upregulated in CMM tissues and cells. Cytoskeletal remodeling of CMM cells, as well as CMM cell proliferation, migration, and invasion, were inhibited by CDC42 or ATF3. ATF3 targeted the CDC42 promoter region to regulate its transcriptional activity. ATF3 suppresses cytoskeletal remodeling in CMM cells, thereby inhibiting CMM progression and metastasis through CDC42. This research may provide a foundation for using ATF3 as a therapeutic target for CMM.
  
  
  
  
**ATF3**
-
3
of
10
  

### FATP2 activates PI3K/Akt/mTOR pathway by inhibiting ATF3 and promotes the occurrence and development of bladder cancer.

3. Cell Signal. 2024 May;117:111087. doi: 10.1016/j.cellsig.2024.111087 Epub 2024 Feb 3.
  
  
Jiang M(1), Chen R(2), Hu B(3), Xiong S(4), Li S(4), Fu B(5), Liu X(6).
  
  
Author information: (1)Department of Urology, The First Affiliated Hospital of Nanchang University, ...
  
  
Bladder cancer (BLCA) is ranked among the main causes of mortality in male cancer patients, and research into targeted therapies guided by its genomics and molecular biology has been a prominent focus in BLCA studies. Fatty acid transporter protein 2 (FATP2), a member of the FATPs family,is a key contributor to the progression of cancers such as hepatocellular carcinomas and melanomas.However,its role in BLCA remains poorly understand. This study delved into the function of FATP2 in BLCA through a succession of experiments in vivo and in vitro, employing techniques as quantitative real-time polymerase chain reaction (qRT-PCR), RNA sequencing, transwell assays, immunofluorescence, western blot,and others to dissect its mechanistic actions. The findings revealed that an oncogenic function is executed by FATP2 in bladder cancer, significantly impacting the proliferation and migration capabilities, thereby affecting the prognosis of BLCA patients. Furthermore, A suppression that relies on both time and concentration of BLCA proliferation and migration, trigger of apoptosis, and blockage of the cell cycle at the G2/M phase were observed when the inhibitor of FATP2, Lipofermata, was applied. It was unveiled through subsequent investigations that ATF3 expression is indirectly promoted by Lipofermata through the inhibition of FATP2, ultimately inhibiting the signal transduction of the PI3K/Akt/mTOR pathway. This effect was also responsible for the inhibitory impact on BLCA proliferation. Therefore, FATP2 emerges as an auspicious and emerging molecular target with potential applications in precision therapy in BLCA.
  
  
  
  
**ATF3**
-
4
of
10
  

### ITGB2-ICAM1 axis promotes liver metastasis in BAP1-mutated uveal melanoma with retained hypoxia and ECM signatures.

4. Cell Oncol (Dordr). 2024 Jun;47(3):951-965. doi: 10.1007/s13402-023-00908-4 Epub 2023 Dec 27.
  
  
Li J(1)(2), Cao D(1)(2), Jiang L(3), Zheng Y(3), Shao S(4), Zhuang A(#)(5)(6), Xiang D(#)(7)(8)(9).
  
  
Author information: (1)State Key Laboratory of Systems Medicine for Cancer, Shanghai Cancer Institut ...
  
  
PURPOSE: Uveal melanoma (UM) with BAP1 inactivating mutations has a high risk of metastasis, but the mechanism behind BAP1 deficiency driving UM metastasis is unknown. METHODS: We analyzed the single-cell RNA sequencing (scRNA-Seq) data comprised primary and metastatic UM with or without BAP1 mutations (MUTs) to reveal inter- and intra-tumor heterogeneity among different groups. Then, an immune-competent mouse liver metastatic model was used to explore the role of ITGB2-ICAM1 in BAP1-associated UM metastasis. RESULTS: Cluster 1 tumor cells expressed high levels of genes linked to tumor metastasis, such as GDF15, ATF3, and CDKN1A, all of which are associated with poor prognosis. The strength of communication between terminally exhausted CD8+ T cells and GDF15hiATF3hiCDKN1Ahi tumor cells was enhanced in BAP1-mutated UM, with CellChat analysis predicting strong ITGB2-ICAM1 signaling between them. High expression of either ITGB2 or ICAM1 was a worse prognostic indicator. Using an immune-competent mouse liver metastatic model, we indicated that inhibiting either ICAM1 or ITGB2 prevented liver metastasis in the BAP1-mutated group in vivo. The inhibitors primarily inhibited hypoxia- and ECM-related pathways indicated by changes in the expression of genes such as ADAM8, CAV2, ENO1, PGK1, LOXL2, ITGA5, and VCAN. etc. CONCLUSION: This study suggested that the ITGB2-ICAM1 axis may play a crucial role for BAP1-associated UM metastasis by preserving hypoxia- and ECM- related signatures, which provide a potential strategy for preventing UM metastasis in patients with BAP1 mutation.
  
  
  
  
**ATF3**
-
5
of
10
  

### Inhibition of TCA cycle improves the anti-PD-1 immunotherapy efficacy in melanoma cells via ATF3-mediated PD-L1 expression and glycolysis.

5. J Immunother Cancer. 2023 Sep;11(9):e007146. doi: 10.1136/jitc-2023-007146
  
  
Liu N(1)(2)(3)(4)(5), Yan M(1)(2)(3)(4)(5), Tao Q(1)(2)(3)(4)(5), Wu J(1)(2)(3)(4)(5), Chen J(1)(2)( ...
  
  
Author information: (1)Department of Dermatology, Xiangya Hospital, Central South University, Changs ...
  
  
BACKGROUND: anti-Programmed Death-1 (anti-PD-1) immunotherapy has shown promising manifestation in improving the survival rate of patients with advanced melanoma, with its efficacy closely linked to Programmed cell death-Ligand 1 (PD-L1) expression. However, low clinical efficacy and drug resistance remain major challenges. Although the metabolic alterations from tricarboxylic acid (TCA) cycle to glycolysis is a hallmark in cancer cells, accumulating evidence demonstrating TCA cycle plays critical roles in both tumorigenesis and treatment. METHODS: The plasma levels of metabolites in patients with melanoma were measured by nuclear magnetic resonance (NMR) spectroscopy. The effect of pyruvate dehydrogenase subunit 1 (PDHA1) and oxoglutarate dehydrogenase (OGDH) on immunotherapy was performed by B16F10 tumor-bearing mice. Flow cytometry analyzed the immune microenvironment. RNA sequencing analyzed the global transcriptome alterations in CPI613-treated melanoma cells. The regulation of PD-L1 and glycolysis by PDHA1/OGDH-ATF3 signaling were confirmed by Quantitative real-time polymerase chain reaction (qRT-PCR), western blotting, dual-luciferase reporter gene, Chromatin immunoprecipitation (ChIP)-quantitative PCR and Seahorse assay. The relationship between PDHA1/OGDH-ATF3-glycolysis and the efficacy of melanoma anti-PD-1 immunotherapy was verified in the clinical database and single-cell RNA-seq (ScRNA-Seq). RESULTS: In our study, the results showed that significant alterations in metabolites associated with glycolysis and the TCA cycle in plasma of patients with melanoma through NMR technique, and then, PDHA1 and OGDH, key enzymes for regulation TCA cycle, were remarkable raised in melanoma and negatively related to anti-PD-1 efficacy through clinical database analysis as well as ScRNA-Seq. Inhibition of PDHA1 and OGDH by either shRNA or pharmacological inhibitor by CPI613 dramatically attenuated melanoma progression as well as improved the therapeutic efficacy of anti-PD-1 against melanoma. Most importantly, suppression of TCA cycle remarkably raises PD-L1 expression and glycolysis flux through AMPK-CREB-ATF3 signaling. CONCLUSIONS: Taken together, our results demonstrated the role of TCA cycle in immune checkpoint blockade and provided a novel combination strategy for anti-PD-1 immunotherapy in melanoma treatment.
  
  
  
  
**ATF3**
-
6
of
10
  

### Effervescent cannabidiol solid dispersion-doped dissolving microneedles for boosted melanoma therapy via the "TRPV1-NFATc1-ATF3" pathway and tumor microenvironment engineering.

6. Biomater Res. 2023 May 18;27(1):48. doi: 10.1186/s40824-023-00390-x
  
  
Shi J(#)(1), Ma Q(#)(1), Su W(1), Liu C(1)(2), Zhang H(1)(2), Liu Y(1)(2), Li X(1)(2), Jiang X(1), G ...
  
  
Author information: (1)Affiliated Hospital of Integrated Traditional Chinese and Western Medicine, N ...
  
  
BACKGROUND: Conventional dissolving microneedles (DMNs) face significant challenges in anti-melanoma therapy due to the lack of active thrust to achieve efficient transdermal drug delivery and intra-tumoral penetration. METHODS: In this study, the effervescent cannabidiol solid dispersion-doped dissolving microneedles (Ef/CBD-SD@DMNs) composed of the combined effervescent components (CaCO3 & NaHCO3) and CBD-based solid dispersion (CBD-SD) were facilely fabricated by the "one-step micro-molding" method for boosted transdermal and tumoral delivery of cannabidiol (CBD). RESULTS: Upon pressing into the skin, Ef/CBD-SD@DMNs rapidly produce CO2 bubbles through proton elimination, significantly enhancing the skin permeation and tumoral penetration of CBD. Once reaching the tumors, Ef/CBD-SD@DMNs can activate transient receptor potential vanilloid 1 (TRPV1) to increase Ca2+ influx and inhibit the downstream NFATc1-ATF3 signal to induce cell apoptosis. Additionally, Ef/CBD-SD@DMNs raise intra-tumoral pH environment to trigger the engineering of the tumor microenvironment (TME), including the M1 polarization of tumor-associated macrophages (TAMs) and increase of T cells infiltration. The introduction of Ca2+ can not only amplify the effervescent effect but also provide sufficient Ca2+ with CBD to potentiate the anti-melanoma efficacy. Such a "one stone, two birds" strategy combines the advantages of effervescent effects on transdermal delivery and TME regulation, creating favorable therapeutic conditions for CBD to obtain stronger inhibition of melanoma growth in vitro and in vivo. CONCLUSIONS: This study holds promising potential in the transdermal delivery of CBD for melanoma therapy and offers a facile tool for transdermal therapies of skin tumors.
  
  
  
  
**ATF3**
-
7
of
10
  

### Integrative Analysis Reveals the Expression Pattern of SOX9 in Satellite Glial Cells after Sciatic Nerve Injury.

7. Brain Sci. 2023 Feb 7;13(2):281. doi: 10.3390/brainsci13020281
  
  
Liu K(1), Ma W(1), Yang J(1)(2), Liu W(1), Zhang S(1), Zhu K(1), Liu J(1), Xiang X(1), Wang G(1), Wu ...
  
  
Author information: (1)Institute of Neuroscience, Faculty of Basic Medical Science, Kunming Medical ...
  
  
BACKGROUND: Several complex cellular and gene regulatory processes are involved in peripheral nerve repair. This study uses bioinformatics to analyze the differentially expressed genes (DEGs) in the satellite glial cells of mice following sciatic nerve injury. METHODS: R software screens differentially expressed genes, and the WebGestalt functional enrichment analysis tool conducts Gene Ontology (GO) enrichment and Kyoto Encyclopedia of Genes and Genomics (KEGG) pathway analysis. The Search Tool for the Retrieval of Interacting Genes/Proteins constructs protein interaction networks, and the cytoHubba plug-in in the Cytoscape software predicts core genes. Subsequently, the sciatic nerve injury model of mice was established and the dorsal root ganglion satellite glial cells were isolated and cultured. Satellite glial cells-related markers were verified by immunofluorescence staining. Real-time polymerase chain reaction assay and Western blotting assay were used to detect the mRNA and protein expression of Sox9 in satellite glial cells. RESULTS: A total of 991 DEGs were screened, of which 383 were upregulated, and 508 were downregulated. The GO analysis revealed the processes of biosynthesis, negative regulation of cell development, PDZ domain binding, and other biological processes were enriched in DEGs. According to the KEGG pathway analysis, DEGs are primarily involved in steroid biosynthesis, hedgehog signaling pathway, terpenoid backbone biosynthesis, American lateral skeleton, and melanoma pathways. According to various cytoHubba algorithms, the common core genes in the protein-protein interaction network are ATF3, Mmp2, and Sox9. Among these, Sox9 was reported to be involved in the central nervous system and the generation and development of astrocytes and could mediate the transformation between neurogenic and glial cells. The experimental results showed that satellite glial cell marker GS were co-labeled with Sox9; stem cell characteristic markers Nestin and p75NTR were labeled satellite glial cells. The mRNA and protein expression of Sox9 in satellite glial cells were increased after sciatic nerve injury. CONCLUSIONS: In this study, bioinformatics was used to analyze the DEGs of satellite glial cells after sciatic nerve injury, and transcription factors related to satellite glial cells were screened, among which Sox9 may be associated with the fate of satellite glial cells.
  
  
  
  
**ATF3**
-
8
of
10
  

### Effect of Sun exposure-induced ferroptosis mechanisms on pathology and potential biological processes of primary melanoma by microarray data analysis.

8. Front Genet. 2022 Sep 26;13:998792. doi: 10.3389/fgene.2022.998792 eCollection 2022.
  
  
Gao Y(1), Hou Q(1), Guo R(1), Ying J(1), Xiong J(1), Jiang H(1).
  
  
Author information: (1)Department of Plastic Surgery, Shanghai East Hospital, Tongji University Scho ...
  
  
Objectives: Sunlight exposure is an important environmental factor in the pathogenesis of skin cutaneous melanoma (SKCM). Ultraviolet (UV) from sunlight can cause excessive intracellular production of reactive oxygen species (ROS), resulting in damage from oxidative stress to cells. As a major iron-rich and ROS-producing organelle, mitochondria are considered as an important place for cell ferroptosis. Thus, the pathology and potential biological process of UV exposure-induced ferroptosis in the development of SKCM has aroused our strong interest. Methods: Gene expression profile datasets of melanoma cell line datasets (GSE31909) and UV-irradiated mitochondria dataset (GSE3632) were downloaded from the Gene Expression Omnibus (GEO) database, and ferroptosis-related genes were obtained from the FerrDb v2 database. After identifying the common differentially expressed genes (DEGs), comprehensive analyzes were performed, including functional annotation, protein-protein interaction (PPI) network construction, hub gene identification, and gene and tissue protein expression levels, survival analysis, and immune cell infiltration analysis. Results: A total of 14 common DEGs was identified for subsequent analyses. Seven DEGs, including PSMB4, CRELD2, CDKN2A, TIMP1, NDRG1, ATF3 and JUND, have consistent performance in mRNA and protein expression in normal skin and SKCM tissues can be regarded as a good biomarker with SKCM diagnostic effectiveness. Functional enrichment analysis results indicate that HIF-1 signaling pathway and angiogenesis involved in the pathogenesis and development of SKCM. Induction of ferroptosis in tumor cells by enhancing the function of CD8+ T cells is expected to be an effective intervention to promote tumor therapy. Conclusion: Our study reveals the pathogenesis and potential biological processes of UV exposure-induced ferroptosis in the development of SKCM, which may provide potential immunotherapy targets for SKCM treatment via tumor cell ferroptosis mechanisms.
  
  
  
  
**ATF3**
-
9
of
10
  

### NRF2 mediates melanoma addiction to GCDH by modulating apoptotic signalling.

9. Nat Cell Biol. 2022 Sep;24(9):1422-1432. doi: 10.1038/s41556-022-00985-x Epub 2022 Sep 1.
  
  
Verma S(1), Crawford D(2), Khateb A(1), Feng Y(1), Sergienko E(3), Pathria G(1)(4), Ma CT(3), Olson ...
  
  
Author information: (1)Cancer Center, Sanford Burnham Prebys Medical Discovery Institute, La Jolla, ...
  
  
Tumour dependency on specific metabolic signals has been demonstrated and often guided numerous therapeutic approaches. We identify melanoma addiction to the mitochondrial protein glutaryl-CoA dehydrogenase (GCDH), which functions in lysine metabolism and controls protein glutarylation. GCDH knockdown induced cell death programmes in melanoma cells, an activity blocked by inhibition of the upstream lysine catabolism enzyme DHTKD1. The transcription factor NRF2 mediates GCDH-dependent melanoma cell death programmes. Mechanistically, GCDH knockdown induces NRF2 glutarylation, increasing its stability and DNA binding activity, with a concomitant transcriptional upregulation of ATF4, ATF3, DDIT3 and CHAC1, resulting in cell death. In vivo, inducible inactivation of GCDH effectively inhibited melanoma tumour growth. Correspondingly, reduced GCDH expression correlated with improved survival of patients with melanoma. These findings identify melanoma cell addiction to GCDH, limiting apoptotic signalling by controlling NRF2 glutarylation. Inhibiting the GCDH pathway could thus represent a therapeutic approach to treat melanoma.
  
  
  
  
**ATF3**
-
10
of
10
  

### The MITF regulatory network in melanoma.

10. Pigment Cell Melanoma Res. 2022 Sep;35(5):517-533. doi: 10.1111/pcmr.13053 Epub 2022 Jul 9.
  
  
Chauhan JS(1), Hölzel M(2), Lambert JP(3)(4)(5), Buffa FM(6), Goding CR(1).
  
  
Author information: (1)Ludwig Institute for Cancer Research, Nuffield Department of Clinical Medicin ...
  
  
Bidirectional interactions between plastic tumor cells and the microenvironment critically impact tumor evolution and metastatic dissemination by enabling cancer cells to adapt to microenvironmental stresses by switching phenotype. In melanoma, a key determinant of phenotypic identity is the microphthalmia-associated transcription factor MITF that promotes proliferation, suppresses senescence, and anticorrelates with immune infiltration and therapy resistance. What determines whether MITF can activate or repress genes associated with specific phenotypes, or how signaling regulating MITF might impact immune infiltration is poorly understood. Here, we find that MITF binding to genes associated with high MITF is via classical E/M-box motifs, but genes downregulated when MITF is high contain FOS/JUN/AP1/ATF3 sites. Significantly, the repertoire of MITF-interacting factors identified here includes JUN and ATF3 as well as many previously unidentified interactors. As high AP1 activity is a hallmark of MITFLow , invasive, slow-cycling, therapy resistant cells, the ability of MITF to repress AP1-regulated genes provides an insight into how MITF establishes and maintains a pro-proliferative phenotype. Moreover, although β-catenin has been linked to immune exclusion, many Hallmark β-catenin signaling genes are associated with immune infiltration. Instead, low MITF together with Notch signaling is linked to immune infiltration in both mouse and human melanoma tumors.
  
  
  
  
  

---

  

# ATXN1

**Signature gene in:** Endothelial cells
  
**Differentially expressed in:** Endothelial cells
  
  
**ATXN1**
-
1
of
1
  

### A novel population of local pericyte precursor cells in tumor stroma that require Notch signaling for differentiation.

1. Microvasc Res. 2015 Sep;101:38-47. doi: 10.1016/j.mvr.2015.05.004 Epub 2015 Jun 17.
  
  
Patenaude A(1), Woerher S(2), Umlandt P(1), Wong F(1), Ibrahim R(1), Kyle A(3), Unger S(1), Fuller M ...
  
  
Author information: (1)Genome Sciences Centre, British Columbia Cancer Agency, Vancouver, British Co ...
  
  
Pericytes are perivascular support cells, the origin of which in tumor tissue is not clear. Recently, we identified a Tie1(+) precursor cell that differentiates into vascular smooth muscle, in a Notch-dependent manner. To understand the involvement of Notch in the ontogeny of tumor pericytes we used a novel flow immunophenotyping strategy to define CD146(+)/CD45(-)/CD31(-/lo) pericytes in the tumor stroma. This strategy combined with ex vivo co-culture experiments identified a novel pericyte progenitor cell population defined as Sca1(hi)/CD146(-)/CD45(-)/CD31(-). The differentiation of these progenitor cells was stimulated by co-culture with endothelial cells. Overexpression of the Notch ligand Jagged1 in endothelial cells further stimulated the differentiation of Sca1(hi)/CD146(-)/CD45(-)/CD31(-) cells into pericytes, while inhibition of Notch signaling with a γ-secretase inhibitor reduced this differentiation. However, Notch inhibition specifically in Tie1-expressing cells did not change the abundance of pericytes in tumors, suggesting that the pericyte precursor is distinct from the vascular smooth muscle cell precursor. Transplant experiments showed that the bone marrow contributes minimally to tumor pericytes. Immunophenotyping revealed that Sca1(hi)/CD146(-)/CD45(-)/CD31(-) cells have greater potential to differentiate into pericytes and have increased expression of classic mesenchymal stem cell markers (CD13, CD44, Nt5e and Thy-1) compared to Sca1(-/lo)/CD146(-)/CD45(-)/CD31(-) cells. Our results suggest that a local Sca1(hi)/CD146(-)/CD45(-)/CD31(-) pericyte progenitor resides in the tumor microenvironment and requires Notch signaling for differentiation into mature pericytes.
  
  
  
  
  

---

  

# AXL

**Signature gene in:** MDM
  
**Differentially expressed in:** MDM
  
  
**AXL**
-
1
of
10
  

### Loss of fatty acid-binding protein 7 promotes B16F10 melanoma metastasis.

1. Sci Rep. 2025 Mar 26;15(1):10495. doi: 10.1038/s41598-024-80874-5
  
  
Wannakul T(1)(2), Miyazaki H(3), Maekawa M(1), Kagawa Y(1), Yamamoto Y(1)(4), Owada Y(1).
  
  
Author information: (1)Department of Organ Anatomy, Graduate School of Medicine, Tohoku University, ...
  
  
Melanoma possesses the characteristic phenotypic plasticity, enhancing its metastatic formation and drug resistance. Lipid and fatty acid metabolism are usually altered to support melanoma progression and can be targeted for therapeutic development. Fatty acid binding protein 7 (FABP7) is highly expressed in melanomas and is shown to support its proliferation, migration, and invasion, but the mechanisms remain unclear. Our study aimed to link FABP7 to lipid metabolism and phenotypic shift in melanomas. We established the Fabp7-knockout (KO) B16F10 melanoma cells, which showed an enhanced invasion through matrix-coated membrane, without significant change in proliferation. Similar outcomes were obtained when using RNA interference targeting FABP7. Fabp7-KO cells injected into mice exhibited slower primary tumor growth, but formed higher metastatic foci count in the lungs. We also discovered a higher saturation in overall lipids, phosphatidylcholines, and triacylglycerols. We observed transcriptional shifts toward the invasive MITFLow/AXLHigh phenotype, with upregulation of transforming growth factor-beta (TGF-β) receptor mRNAs. In conclusion, FABP7 may help balancing lipid saturation and maintain the proliferative state of melanomas, mitigating invasiveness and metastatic formation.
  
  
  
  
**AXL**
-
2
of
10
  

### From fat to fear: how lipid powers cancer spread.

2. Genes Dev. 2025 Mar 14;39(7-8):423-5. doi: 10.1101/gad.352753.125 Online ahead of print.
  
  
Larue L(1).
  
  
Author information: (1)Normal and Pathological Development of Melanocytes, U1021, Institut National ...
  
  
Metastasis and therapy resistance drive cancer-related deaths, with melanoma cells exhibiting phenotypic plasticity that allows them to switch between proliferative and invasive states. In this issue of Genes & Development, Chocarro-Calvo and colleagues (doi:10.1101/gad.351985.124) reveal that oleic acid activates AXL, a receptor involved in metastasis and therapy resistance, linking lipid metabolism to melanoma aggressiveness. They demonstrate that MITFLow/AXLHigh cells induce lipolysis in human adipose tissue via WNT5A secretion, compensating for lipid synthesis deficiencies. The study highlights distinct lipid uptake mechanisms in melanoma subpopulations and suggests that targeting AXL-driven lipid uptake could provide therapeutic opportunities. These findings have broad implications, indicating that metabolic cues influence AXL activation in other cancers.
  
  
  
  
**AXL**
-
3
of
10
  

### BRAF(V600E)-PROTAC versus inhibitors in melanoma cells: Deep transcriptomic characterisation.

3. Clin Transl Med. 2025 Mar;15(3):e70251. doi: 10.1002/ctm2.70251
  
  
Alhassan SO(1), Abd Elmageed ZY(2), Errami Y(3), Wang G(4), Abi-Rached JA(5), Kandil E(5), Zerfaoui ...
  
  
Author information: (1)Department of Gastrointestinal Oncology, Moffitt Cancer Center Magnolia Campu ...
  
  
AIMS: This study compares the suppression of Mitogen-activated protein kinase (MAPK) signalling and early resistance potential between a proteolysis-targeting chimera (PROTAC) and inhibitors targeting BRAFV600E. METHODS: We performed a detailed in silico analysis of the transcriptomic landscape of the A375 melanoma cell line treated with a PROTAC and BRAFV600E inhibitors from RNA sequencing data. The study assessed gene dysregulation, MAPK and Phosphoinositide-3-kinase (PI3K/AKT) pathway inhibition, and cell survival. Key genes uniquely dysregulated by PROTAC treatment were validated by qPCR. Furthermore, analysis was performed to evaluate dedifferentiation and early resistance signatures to understand melanoma drug-induced plasticity. RESULTS: PROTAC-treated cells showed significantly lower MAPK pathway activity, strong cell cycle arrest and elevated apoptotic gene expression compared to inhibitor-treated cells, with no effect on the PI3K/AKT pathway. A high microphtalmia-associated transcription factor (MITF)/Tyrosine-Protein Kinase Receptor (AXL) ratio in PROTAC-treated cells indicated reduced early drug resistance. BRAF degradation induced a melanocytic-transitory phenotype. Although PROTAC and inhibitor treatments caused overlapping transcriptomic changes, key differences were observed. PROTAC treatment enriched processes such as epithelial‒mesenchymal transition, inflammatory responses, and Tumor necrosis factor-Alpha (TNF-α) and IL2/STAT5 signalling. CONCLUSION: PROTAC-targeting BRAFV600E demonstrates enhanced MAPK suppression, reduced early resistance and distinct transcriptional effects compared to traditional inhibitors. It represents a promising strategy for overcoming resistance in melanoma treatment.
  
  
  
  
**AXL**
-
4
of
10
  

### Fatty acid uptake activates an AXL-CAV1-β-catenin axis to drive melanoma progression.

4. Genes Dev. 2025 Apr 1;39(7-8):463-489. doi: 10.1101/gad.351985.124
  
  
Chocarro-Calvo A(#)(1)(2), Jociles-Ortega M(#)(2), García-Martinez JM(2), Louphrasitthiphol P(1), Ca ...
  
  
Author information: (1)Ludwig Institute for Cancer Research, Nuffield Department of Clinical Medicin ...
  
  
Interaction between the tumor microenvironment and cancer cell plasticity drives intratumor phenotypic heterogeneity and underpins disease progression and nongenetic therapy resistance. Phenotype-specific expression of the AXL receptor tyrosine kinase is a pivotal player in dormancy, invasion, and resistance to treatment. However, although the AXL ligand GAS6 is present within tumors, how AXL is activated in metastasizing cells remains unclear. Here, using melanoma as a model, we reveal that AXL is activated by exposure to human adipocytes and to oleic acid, a monounsaturated fatty acid abundant in lymph and in adipocytes. AXL activation triggers SRC-dependent formation and nuclear translocation of a β-catenin-CAV1 complex required for melanoma invasiveness. Remarkably, only undifferentiated AXLHigh melanoma cells engage in symbiosis with human adipocytes, in part by triggering WNT5a-mediated lipolysis, leading to AXL-dependent, but FATP-independent, fatty acid uptake and nuclear localization of the β-catenin-CAV1 complex. Significantly, human melanomas in the vicinity of adipocytes exhibit high levels of nuclear CAV1. The results unveil an AXL- and CAV1-dependent mechanism through which a nutritional input drives phenotype-specific activation of a prometastasis program. Given the key role of AXL in a broad range of cancers, the results offer major insights into the mechanisms of cancer cell dormancy and therapy resistance.
  
  
  
  
**AXL**
-
5
of
10
  

### Mechanisms of Resistance to Anti-PD-1 Immunotherapy in Melanoma and Strategies to Overcome It.

5. Biomolecules. 2025 Feb 12;15(2):269. doi: 10.3390/biom15020269
  
  
Zielińska MK(1)(2), Ciążyńska M(3)(4), Sulejczak D(5), Rutkowski P(1), Czarnecka AM(1)(5).
  
  
Author information: (1)Department of Soft Tissue/Bone Sarcoma and Melanoma, Maria Sklodowska-Curie N ...
  
  
Resistance to anti-PD-1 therapy in melanoma remains a major obstacle in achieving effective and durable treatment outcomes, highlighting the need to understand and address the underlying mechanisms. The first key factor is innate anti-PD-1 resistance signature (IPRES), an expression of a group of genes associated with tumor plasticity and immune evasion. IPRES promotes epithelial-to-mesenchymal transition (EMT), increasing melanoma cells' invasiveness and survival. Overexpressed AXL, TWIST2, and WNT5a induce phenotypic changes. The upregulation of pro-inflammatory cytokines frequently coincides with EMT-related changes, further promoting a resistant and aggressive tumor phenotype. Inflamed tumor microenvironment may also drive the expression of resistance. The complexity of immune resistance development suggests that combination therapies are necessary to overcome it. Furthermore, targeting epigenetic regulation and exploring novel approaches such as miR-146a modulation may provide new strategies to counter resistance in melanoma.
  
  
  
  
**AXL**
-
6
of
10
  

### Changes in AXL and/or MITF melanoma subpopulations in patients receiving immunotherapy.

6. Immunooncol Technol. 2024 Nov 15;24:101009. doi: 10.1016/j.iotech.2024.101009 eCollection 2024 Dec.
  
  
Willemsen M(1), Bulgarelli J(2), Chauhan SK(3), Lereim RR(3), Angeli D(4), Grisendi G(5), Krebbers G ...
  
  
Author information: (1)Department of Dermatology and Netherlands Institute for Pigment Disorders, Am ...
  
  
BACKGROUND: Tumor heterogeneity is a hurdle to effective therapy, as illustrated by the 'mixed responses' frequently seen in immunotherapy-treated patients. Previously, AXL+ tumor cells were identified to be highly resistant to targeted therapy, whereas more differentiated MITF+ tumor cells do respond to RAF and MEK inhibitors. PATIENTS AND METHODS: In this study, we analyzed tumor heterogeneity and explored the presence of the previously described AXL+ or MITF+ melanoma subpopulations in metastatic tissues by NanoString gene expression analysis, single-cell RNA sequencing and in situ multiplex immunofluorescence. Furthermore, we analyzed how these subpopulations correlate with immunological pressure and response to immunotherapy by immunomodulating antibodies or autologous tumor lysate-loaded dendritic cell vaccination. RESULTS: Our data demonstrate large interpatient variability and variable therapy-induced changes independent of the type of therapy. We identify the presence of previously described AXL+ and MITF+ subpopulations in metastatic tissues both at the mRNA level and in situ at the protein level, and demonstrate that MITF+ melanoma cells are significantly decreased upon immunotherapy, while AXL+ melanoma cell numbers are stable. MITF+ tumor cells showed the most significant inverse correlation with CD8+ T cells. Our patient cohort also shows that immunotherapy-induced changes in the abundance of AXL+ or MITF+ tumor cells did not correlate with improved survival. CONCLUSIONS: Overall, this study suggests that more differentiated MITF+ tumors are efficiently targeted by immunotherapy, while AXL+ tumor cells may be more resistant, analogous to their response to targeted therapy.
  
  
  
  
**AXL**
-
7
of
10
  

### BRAF-Mutated Melanoma Cell Lines Develop Distinct Molecular Signatures After Prolonged Exposure to AZ628 or Dabrafenib: Potential Benefits of the Antiretroviral Treatments Cabotegravir or Doravirine on BRAF-Inhibitor-Resistant Cells.

7. Int J Mol Sci. 2024 Nov 6;25(22):11939. doi: 10.3390/ijms252211939
  
  
Zanrè V(1), Bellinato F(2), Cardile A(1), Passarini C(1), Di Bella S(3), Menegazzi M(1).
  
  
Author information: (1)Section of Biochemistry, Department of Neuroscience, Biomedicine and Movement ...
  
  
Melanoma is an aggressive cancer characterized by rapid growth, early metastasis, and poor prognosis, with resistance to current therapies being a significant issue. BRAF mutations drive uncontrolled cell division by activating the MAPK pathway. In this study, A375 and FO-1, BRAF-mutated melanoma cell lines, were treated for 4-5 months with RAF inhibitor dabrafenib or AZ628, leading to drug resistance over time. The resistant cells showed altered molecular signatures, with differences in cell cycle regulation and the propensity of cell death. Dabrafenib-resistant cells maintained high proliferative activity, while AZ628-resistant cells, especially A375 cells, exhibited slow-cycling, and a senescent-like phenotype with high susceptibility to ferroptosis, a form of cell death driven by iron. Antiretroviral drugs doravirine and cabotegravir, known for their effects on human endogenous retroviruses, were tested for their impact on these resistant melanoma cells. Both drugs reduced cell viability and colony formation in resistant cell lines. Doravirine was particularly effective in reactivating apoptosis and reducing cell growth in highly proliferative resistant cells by increasing tumor-suppressor proteins p16Ink4a and p27Kip1. These findings suggest that antiretroviral drugs can influence apoptosis and cell proliferation in RAF-inhibitor-resistant melanoma cells, offering potential therapeutic strategies for overcoming drug resistance.
  
  
  
  
**AXL**
-
8
of
10
  

### Secreted Apoe rewires melanoma cell state vulnerability to ferroptosis.

8. Sci Adv. 2024 Oct 18;10(42):eadp6164. doi: 10.1126/sciadv.adp6164 Epub 2024 Oct 16.
  
  
More S(1)(2), Bonnereau J(1)(2), Wouters D(3)(4)(5), Spotbeen X(4)(6), Karras P(7)(8), Rizzollo F(1) ...
  
  
Author information: (1)Cell Death Research and Therapy Laboratory, VIB-KU Leuven Center for Cancer B ...
  
  
A major therapeutic barrier in melanoma is the coexistence of diverse cellular states marked by distinct metabolic traits. Transitioning from a proliferative to an invasive melanoma phenotype is coupled with increased ferroptosis vulnerability. However, the regulatory circuits controlling ferroptosis susceptibility across melanoma cell states are unknown. In this work, we identified Apolipoprotein E (APOE) as the top lipid-metabolism gene segregating the melanoma MITFhigh/AXLlow proliferative/ferroptosis-resistant from MITFlow/AXLhigh invasive/ferroptosis-sensitive state. Mechanistically, ApoE secreted by the MITFhigh/AXLlow cells protects the invasive phenotype from ferroptosis-inducing agents by reducing the content of peroxidation-prone polyunsaturated fatty acids and boosting GPX4 levels both in vitro and in vivo. Whole-exome sequencing indicates that APOEhigh expression in patients with melanoma is associated with resistance to ferroptosis, regardless of APOE germline status. In aggregate, we found a ferroptosis-resistance mechanism between melanoma cell states relying on secreted ApoE and APOEhigh expression as a potential biomarker for poor ferroptosis response in melanoma.
  
  
  
  
**AXL**
-
9
of
10
  

### Sex-dependent effects in the aged melanoma tumor microenvironment influence invasion and resistance to targeted therapy.

9. Cell. 2024 Oct 17;187(21):6016-6034.e25. doi: 10.1016/j.cell.2024.08.013 Epub 2024 Sep 6.
  
  
Chhabra Y(1), Fane ME(2), Pramod S(3), Hüser L(3), Zabransky DJ(4), Wang V(3), Dixit A(3), Zhao R(5) ...
  
  
Author information: (1)Department of Biochemistry and Molecular Biology, Johns Hopkins Bloomberg Sch ...
  
  
There is documented sex disparity in cutaneous melanoma incidence and mortality, increasing disproportionately with age and in the male sex. However, the underlying mechanisms remain unclear. While biological sex differences and inherent immune response variability have been assessed in tumor cells, the role of the tumor-surrounding microenvironment, contextually in aging, has been overlooked. Here, we show that skin fibroblasts undergo age-mediated, sex-dependent changes in their proliferation, senescence, ROS levels, and stress response. We find that aged male fibroblasts selectively drive an invasive, therapy-resistant phenotype in melanoma cells and promote metastasis in aged male mice by increasing AXL expression. Intrinsic aging in male fibroblasts mediated by EZH2 decline increases BMP2 secretion, which in turn drives the slower-cycling, highly invasive, and therapy-resistant melanoma cell phenotype, characteristic of the aged male TME. Inhibition of BMP2 activity blocks the emergence of invasive phenotypes and sensitizes melanoma cells to BRAF/MEK inhibition.
  
  
  
  
**AXL**
-
10
of
10
  

### The classification of melanocytic gene signatures.

10. Pigment Cell Melanoma Res. 2024 Nov;37(6):854-863. doi: 10.1111/pcmr.13189 Epub 2024 Jul 28.
  
  
Hu M(1), Coleman S(2), Judson-Torres RL(1)(3)(4), Tan AC(1)(2)(3).
  
  
Author information: (1)Department of Oncological Sciences, University of Utah, Salt Lake City, Utah, ...
  
  
Gene expression profiling technologies have revolutionized cell biology, enabling researchers to identify gene signatures linked to various biological attributes of melanomas, such as pigmentation status, differentiation state, proliferative versus invasive capacity, and disease progression. Although the discovery of gene signatures has significantly enhanced our understanding of melanocytic phenotypes, reconciling the numerous signatures reported across independent studies and different profiling platforms remains a challenge. Current methods for classifying melanocytic gene signatures depend on exact gene overlap and comparison with unstandardized baseline transcriptomes. In this study, we aimed to categorize published gene signatures into clusters based on their similar patterns of expression across clinical cutaneous melanoma specimens. We analyzed nearly 800 melanoma samples from six gene expression repositories and developed a classification framework for gene signatures that is resilient against biases in gene identification across profiling platforms and inconsistencies in baseline standards. Using 39 frequently cited published gene signatures, our analysis revealed seven principal classes of gene signatures that correlate with previously identified phenotypes: Differentiated, Mitotic/MYC, AXL, Amelanotic, Neuro, Hypometabolic, and Invasive. Each class is consistent with the phenotypes that the constituent gene signatures represent, and our classification method does not rely on overlapping genes between signatures. To facilitate broader application, we created WIMMS (what is my melanocytic signature, available at https://wimms.tanlab.org/), a user-friendly web application. WIMMS allows users to categorize any gene signature, determining its relationship to predominantly cited signatures and its representation within the seven principal classes.
  
  
  
  
  

---

  

# BCL11B

**Signature gene in:** CD8+ T cells
  
**Differentially expressed in:** CD8+ T cells
  
  
**BCL11B**
-
1
of
6
  

### Network-based analysis of heterogeneous patient-matched brain and extracranial melanoma metastasis pairs reveals three homogeneous subgroups.

1. Comput Struct Biotechnol J. 2024 Feb 17;23:1036-1050. doi: 10.1016/j.csbj.2024.02.013 eCollection 2024 Dec.
  
  
Grützmann K(1), Kraft T(1), Meinhardt M(2), Meier F(3)(4), Westphal D(3)(4), Seifert M(1)(4).
  
  
Author information: (1)Institute for Medical Informatics and Biometry, Faculty of Medicine, TU Dresd ...
  
  
Melanoma, the deadliest form of skin cancer, can metastasize to different organs. Molecular differences between brain and extracranial melanoma metastases are poorly understood. Here, promoter methylation and gene expression of 11 heterogeneous patient-matched pairs of brain and extracranial metastases were analyzed using melanoma-specific gene regulatory networks learned from public transcriptome and methylome data followed by network-based impact propagation of patient-specific alterations. This innovative data analysis strategy allowed to predict potential impacts of patient-specific driver candidate genes on other genes and pathways. The patient-matched metastasis pairs clustered into three robust subgroups with specific downstream targets with known roles in cancer, including melanoma (SG1: RBM38, BCL11B, SG2: GATA3, FES, SG3: SLAMF6, PYCARD). Patient subgroups and ranking of target gene candidates were confirmed in a validation cohort. Summarizing, computational network-based impact analyses of heterogeneous metastasis pairs predicted individual regulatory differences in melanoma brain metastases, cumulating into three consistent subgroups with specific downstream target genes.
  
  
  
  
**BCL11B**
-
2
of
6
  

### Integrative RNA profiling of TBEV-infected neurons and astrocytes reveals potential pathogenic effectors.

2. Comput Struct Biotechnol J. 2022 May 30;20:2759-2777. doi: 10.1016/j.csbj.2022.05.052 eCollection 2022.
  
  
Selinger M(1)(2), Věchtová P(1), Tykalová H(1)(2), Ošlejšková P(1), Rumlová M(3), Štěrba J(1), Grubh ...
  
  
Author information: (1)Faculty of Science, University of South Bohemia in České Budějovice, Branišov ...
  
  
Tick-borne encephalitis virus (TBEV), the most medically relevant tick-transmitted flavivirus in Eurasia, targets the host central nervous system and frequently causes severe encephalitis. The severity of TBEV-induced neuropathogenesis is highly cell-type specific and the exact mechanism responsible for such differences has not been fully described yet. Thus, we performed a comprehensive analysis of alterations in host poly-(A)/miRNA/lncRNA expression upon TBEV infection in vitro in human primary neurons (high cytopathic effect) and astrocytes (low cytopathic effect). Infection with severe but not mild TBEV strain resulted in a high neuronal death rate. In comparison, infection with either of TBEV strains in human astrocytes did not. Differential expression and splicing analyses with an in silico prediction of miRNA/mRNA/lncRNA/vd-sRNA networks found significant changes in inflammatory and immune response pathways, nervous system development and regulation of mitosis in TBEV Hypr-infected neurons. Candidate mechanisms responsible for the aforementioned phenomena include specific regulation of host mRNA levels via differentially expressed miRNAs/lncRNAs or vd-sRNAs mimicking endogenous miRNAs and virus-driven modulation of host pre-mRNA splicing. We suggest that these factors are responsible for the observed differences in the virulence manifestation of both TBEV strains in different cell lines. This work brings the first complex overview of alterations in the transcriptome of human astrocytes and neurons during the infection by two TBEV strains of different virulence. The resulting data could serve as a starting point for further studies dealing with the mechanism of TBEV-host interactions and the related processes of TBEV pathogenesis.
  
  
  
  
**BCL11B**
-
3
of
6
  

### BCL11B prevents fatal autoimmunity by promoting T(reg) cell program and constraining innate lineages in T(reg) cells.

3. Sci Adv. 2019 Aug 7;5(8):eaaw0480. doi: 10.1126/sciadv.aaw0480 eCollection 2019 Aug.
  
  
Drashansky TT(1), Helm E(1), Huo Z(2), Curkovic N(1), Kumar P(1), Luo X(1), Parthasarathy U(1), Zuni ...
  
  
Author information: (1)Department of Anatomy and Cell Biology, College of Medicine, University of Fl ...
  
  
Regulatory T (Treg) cells are essential for peripheral tolerance and rely on the transcription factor (TF) Foxp3 for their generation and function. Several other TFs are critical for the Treg cell program. We found that mice deficient in BCL11B TF solely in Treg cells developed fatal autoimmunity, and Bcl11b-deficient Treg cells had severely altered function. BCL11B KO Treg cells showed decreased functional marker levels in homeostatic conditions, inflammation, and tumors. BCL11B controlled expression of essential Treg program genes at steady state and in inflammation. BCL11B bound to genomic regulatory regions of Treg program genes in both human and mouse Treg cells, overlapping with Foxp3 binding; these genes showed altered chromatin accessibility in the absence of BCL11B. Additionally, BCL11B restrained myeloid and NK cell programs in Treg cells. Our study provides new mechanistic insights on the Treg cell program and identity control, with major implications for therapies in autoimmunity and cancer.
  
  
  
  
**BCL11B**
-
4
of
6
  

### Targeting CD147 for T to NK Lineage Reprogramming and Tumor Therapy.

4. EBioMedicine. 2017 Jun;20:98-108. doi: 10.1016/j.ebiom.2017.05.022 Epub 2017 May 18.
  
  
Geng JJ(1), Tang J(2), Yang XM(2), Chen R(1), Zhang Y(2), Zhang K(3), Miao JL(3), Chen ZN(4), Zhu P( ...
  
  
Author information: (1)Department of Clinical Immunology, Xijing Hospital, Fourth Military Medical U ...
  
  
CD147 is highly expressed on the surface of numerous tumor cells to promote invasion and metastasis. Targeting these cells with CD147-specific antibodies has been validated as an effective approach for lung and liver cancer therapy. In the immune system, CD147 is recognized as a co-stimulatory receptor and impacts the outcome of thymic selection. Using T cell-specific deletion, we showed here that in thymus CD147 is indispensable for the stable αβ T cell lineage commitment: loss of CD147 biases both multipotent DN (double negative) and fully committed DP (double positive) cells into innate NK-like lineages. Mechanistically, CD147 deficiency results in impaired Wnt signaling and expression of BCL11B, a master transcription factor in determining T cell identity. In addition, functional blocking of CD147 by antibody phenocopies genetic deletion to enrich NK-like cells in the periphery. Furthermore, using a melanoma model and orthotopic liver cancer transplants, we showed that the augmentation of NK-like cells strongly associates with resistance against tumor growth upon CD147 suppression. Therefore, besides its original function in tumorigenesis, CD147 is also an effective surface target for immune modulation in tumor therapy.
  
  
  
  
**BCL11B**
-
5
of
6
  

### TNF-α-dependent hematopoiesis following BCL11B deletion in T cells restricts metastatic melanoma.

5. J Immunol. 2014 Feb 15;192(4):1946-53. doi: 10.4049/jimmunol.1301976 Epub 2014 Jan 20.
  
  
Uddin MN(1), Zhang Y, Harton JA, MacNamara KC, Avram D.
  
  
Author information: (1)Center for Immunology and Microbial Disease, Albany Medical College, Albany, ...
  
  
Using several tumor models, we demonstrate that mice deficient in BCL11B in T cells, although having reduced numbers of T cells in the peripheral lymphoid organs, developed significantly less tumors compared with wild-type mice. BCL11B(-/-) CD4(+) T cells, with elevated TNF-α levels, but not the BCL11B(-/-) CD8(+) T cells, were required for the reduced tumor burden, as were NK1.1(+) cells, found in increased numbers in BCL11B(F/F)/CD4-Cre mice. Among NK1.1(+) cells, the NK cell population was predominant in number and was the only population displaying elevated granzyme B levels and increased degranulation, although not increased proliferation. Although the number of myeloid-derived suppressor cells was increased in the lungs with metastatic tumors of BCL11B(F/F)/CD4-Cre mice, their arginase-1 levels were severely reduced. The increase in NK cell and myeloid-derived suppressor cell numbers was associated with increased bone marrow and splenic hematopoiesis. Finally, the reduced tumor burden, increased numbers of NK cells in the lung, and increased hematopoiesis in BCL11B(F/F)/CD4-Cre mice were all dependent on TNF-α. Moreover, TNF-α treatment of wild-type mice also reduced the tumor burden and increased hematopoiesis and the numbers and activity of NK cells in the lung. In vitro treatment with TNF-α of lineage-negative hematopoietic progenitors increased NK and myeloid differentiation, further supporting a role of TNF-α in promoting hematopoiesis. These studies reveal a novel role for TNF-α in the antitumor immune response, specifically in stimulating hematopoiesis and increasing the numbers and activity of NK cells.
  
  
  
  
**BCL11B**
-
6
of
6
  

### Reprogramming of T cells to natural killer-like cells upon BCL11B deletion.

6. Science. 2010 Jul 2;329(5987):85-9. doi: 10.1126/science.1188063 Epub 2010 Jun 10.
  
  
Li P(1), Burke S, Wang J, Chen X, Ortiz M, Lee SC, Lu D, Campos L, Goulding D, Ng BL, Dougan G, Hunt ...
  
  
Author information: (1)Wellcome Trust Sanger Institute, Hinxton, Cambridge CB10 1HH, UK.
  
  
Comment in Science. 2010 Jul 2;329(5987):44-5. doi: 10.1126/science.1191664. Nat Rev Clin Oncol. 2010 Sep;7(9):483. doi: 10.1038/nrclinonc.2010.125.
  
  
  
  
  

---

  

# CCDC88C

**Signature gene in:** CD8+ T cells
  
**Differentially expressed in:** CD8+ T cells
  
  
**CCDC88C**
-
1
of
1
  

### Prognostic Relevance of CCDC88C (DAPLE) Transcripts in the Peripheral Blood of Patients with Cutaneous Melanoma.

1. Sci Rep. 2018 Dec 21;8(1):18036. doi: 10.1038/s41598-018-36173-x
  
  
Dunkel Y(1), Reid AL(2), Ear J(1), Aznar N(1)(3), Millward M(4)(5), Gray E(2), Pearce R(2), Ziman M( ...
  
  
Author information: (1)Department of Medicine, University of California, San Diego, La Jolla, Califo ...
  
  
A loss of balance between G protein activation and deactivation has been implicated in the initiation of melanomas, and non-canonical Wnt signaling via the Wnt5A/Frizzled (FZD) pathway has been shown to be critical for the switch to an invasive phenotype. DAPLE [CCDC88C], a cytosolic guanine nucleotide exchange modulator (GEM) which enhances non-canonical Wnt5A/FZD signaling via activation of trimeric G protein, Gαi, has been shown to serve opposing roles-as an inducer of EMT and invasiveness and a potent tumor suppressor-via two isoforms, V1 (full-length) and V2 (short spliced isoform), respectively. Here we report that the relative abundance of these isoforms in the peripheral circulation, presumably largely from circulating tumor cells (CTCs), is a prognostic marker of cutaneous melanomas. Expression of V1 is increased in both the early and late clinical stages (p < 0.001, p = 0.002, respectively); V2 is decreased exclusively in the late clinical stage (p = 0.003). The two isoforms have opposing prognostic effects: high expression of V2 increases relapse-free survival (RFS; p = 0.014), whereas high expression of V1 tends to decrease RFS (p = 0.051). Furthermore, these effects are additive, in that melanoma patients with a low V2-high V1 signature carry the highest risk of metastatic disease. We conclude that detection of DAPLE transcripts in the peripheral blood (i.e., liquid biopsies) of patients with melanoma may serve as a prognostic marker and an effective strategy for non-invasive long-term follow-up of patients with melanoma.
  
  
  
  
  

---

  

# CCND3

**Signature gene in:** CD4+ T cells, CD8+ T cells, MDM, Tregs
  
**Differentially expressed in:** CD8+ T cells, MDM
  
  
**CCND3**
-
1
of
10
  

### Deciphering the predictive value of senescence-related signature in lung adenocarcinoma: Implications for antitumor immunity and immunotherapy efficacy.

1. Heliyon. 2024 Aug 8;10(16):e35940. doi: 10.1016/j.heliyon.2024.e35940 eCollection 2024 Aug 30.
  
  
Guo Y(1)(2)(3), Wang Y(4), Duan J(2)(3), Wan R(2)(3), Chang G(5), Zhang X(2)(3), Ma Z(2)(3), Bai H(2 ...
  
  
Author information: (1)Department of Clinical Research, State Key Laboratory of Oncology in South Ch ...
  
  
OBJECTIVE: The senescence process is pivotal in both the onset and advancement of lung adenocarcinoma (LUAD), influencing cell growth, immune evasion, the potential for metastasis, and resistance to treatments. Senescent cells' dual nature, both harmful and advantageous, adds complexity to understanding their expression patterns and clinical relevance in LUAD. In this study, we sought to evaluate the predictive value of the senescence-related signature in survival outcomes and immunotherapy efficacy in patients with LUAD. MATERIALS AND METHODS: We integrated data from 1449 LUAD cases sourced from different publicly accessible datasets and a clinical cohort of Chinese LUAD patients. The Cox regression analysis employing the least absolute shrinkage and selection operator (LASSO) was performed on 156 senescence-associated genes to develop the senescence-related signature. Kaplan-Meier analysis and time-dependent receiver operating characteristic curves were utilizaed to assess the prognostic significance of the senescence-related signature. Functional annotation, immune infiltration analysis, and gene set variation analysis were applied to investigate the association of the senescence-related signature with anti-tumor immunity in LUAD. Immunotherapy cohorts of non-small cell lung cancer, urothelial carcinoma, skin cutaneous melanoma, and glioblastoma patients were included to assess the senescence-related signature in predicting immunotherapy efficacy. RESULTS: The senescence-related signature, which encompasses seven senescence-related genes, namely, FOXM1, VDAC1, PPP3CA, MAPK13, PIK3CD, RRAS, and CCND3, was identified to have predictive significance across multiple LUAD cohorts and demonstrated a negative association with antitumor immunity and tumor-infiltrating neutrophils. Patients exhibiting low expression levels of the senescence-related signature responded more favorably to immune checkpoint inhibitors in various solid tumors, including LUAD. Inhibiting FOXM1 pharmacologically with thiostrepton produced tumor-suppressive effects and improved immunotherapy responses in a Lewis lung carcinoma mouse model. CONCLUSIONS: The senescence-related signature demonstrates potential in predicting patient prognosis and immunotherapy efficacy in LUAD.
  
  
  
  
**CCND3**
-
2
of
10
  

### A centrosome-related gene signature for predicting the overall survival of uveal melanoma.

2. Transl Cancer Res. 2024 Feb 29;13(2):771-781. doi: 10.21037/tcr-23-1486 Epub 2024 Feb 28.
  
  
Yu X(#)(1), Guo X(#)(2), Li W(3), Li J(1)(4).
  
  
Author information: (1)Department of Breast and Thyroid Surgery, Renmin Hospital of Wuhan University ...
  
  
BACKGROUND: Centrosome aberration (CA) plays a vital role in tumorigenesis and metastasis under pathophysiological conditions. The existence of CA was first reported in uveal melanoma (UVM) recently. Our study aimed to investigate the association of centrosome-related genes with UVM prognosis. METHODS: The Cancer Genome Atlas (TCGA)-UVM and Gene Expression Omnibus series (GSE) 22138 were included in the study. Least absolute shrinkage and selection operator (LASSO) and Cox regression were combined to screen out key genes and construct a centrosome-related gene signature. Kaplan-Meier (KM) survival curves were used to evaluate the survival differences between the 2 groups. Gene enrichment, immune infiltration, and mutation profile were used to explore the underlying mechanism. RESULTS: A centrosome-related gene signature was constructed: Risk score =-3.27071 × MAP6 - 5.03735 × CCDC40 - 2.68459 × PRKCD + 1.826349 × IGFBP4 + 11.66582 × RAB6C - 4.86899 × CCND3. The survival possibilities of the two groups were significantly different. The high-risk group showed cancer progression, inflammation, and immune restriction characteristics when compared with the low-risk group. BAP1 mutation was associated with high risk and SF3B1 mutation was associated with low risk according to the signature. CONCLUSIONS: Our study first investigated the role of centrosome-related genes in UVM overall survival (OS). We then constructed a centrosome-related gene signature for UVM, which provides new insights into the role of CA in UVM and identifies novel centrosome-related biomarkers.
  
  
  
  
**CCND3**
-
3
of
10
  

### Quantitative Proteomic Analysis Revealed Broad Roles of N(6)-Methyladenosine in Heat Shock Response.

3. J Proteome Res. 2021 Jul 2;20(7):3611-3620. doi: 10.1021/acs.jproteome.1c00191 Epub 2021 May 27.
  
  
Miao W(1), Yang YY(1), Wang Y(1).
  
  
Author information: (1)Department of Chemistry, University of California, Riverside, California 9252 ...
  
  
As optimum temperature is essential for all living organisms, heat shock represents a challenging problem for their survival. Therefore, cellular response to heat shock is among the most extensively investigated stress response pathways; however, how the human proteome responds to heat shock has not been comprehensively investigated. In this study, we employed stable isotope labeling by amino acids in cell culture (SILAC), together with liquid chromatography-tandem mass spectrometry (LC-MS/MS) analysis, to fulfill an in-depth analysis of the alterations in the human proteome in M14 human melanoma cells in response to heat shock stress. We found that, after heat shock, 284 and 278 out of the 4319 quantified proteins were with substantially diminished and elevated expressions, respectively. We also examined the alterations in human kinome after heat shock by using our recently developed targeted proteomic method relying on parallel-reaction monitoring. Our results showed that the expression levels of 11 and 22 kinase proteins were increased and decreased, respectively, by at least 1.5-fold upon heat shock. By interrogating publicly available RNA-seq and m6A sequencing data, we observed that the elevated expression of more than 30 proteins, including CHEK1 and CCND3 kinases, could occur via an m6A-mediated mechanism. Furthermore, our results from single-base elongation and ligation-based quantitative polymerase chain reaction (qPCR) amplification (SELECT) and luciferase reporter assays revealed that heat shock gave rise to elevated m6A levels at A280 and A286 sites in the 5'-untranslated region of HSPH1 mRNA, thereby leading to increased translation of HSPH1 protein. Together, our discovery and targeted proteomic methods revealed the reprogramming of human proteome and kinome upon heat shock stress and provided insights into cellular responses toward heat shock stress.
  
  
  
  
**CCND3**
-
4
of
10
  

### Treatment of melanoma with selected inhibitors of signaling kinases effectively reduces proliferation and induces expression of cell cycle inhibitors.

4. Med Oncol. 2017 Dec 6;35(1):7. doi: 10.1007/s12032-017-1069-0
  
  
Ciołczyk-Wierzbicka D(1), Gil D(2), Laidler P(2).
  
  
Author information: (1)Chair of Medical Biochemistry, Jagiellonian University Medical College, ul. K ...
  
  
Cancer treatment often tends to involve direct targeting enzymes essential for the growth and proliferation of cancer cells. The aim of this study was the recognition of the possible role of selected protein kinases: PI3K, ERK1/2, and mTOR in cell proliferation and cell cycle in malignant melanoma. We investigated the role of protein kinase inhibitors: U0126 (ERK1/2), LY294002 (PI3K), rapamycin (mTOR), everolimus (mTOR), GDC-0879 (B-RAF), and CHIR-99021 (GSK3beta) in cell proliferation and expression of crucial regulatory cell cycle proteins in human melanoma cells: WM793 (VGP) and Lu1205 (metastatic). They were used either individually or in various combinations. The study on the effect of signaling kinases inhibitors on proliferation-BrdU ELISA test after 48-72 h. Their effect on the expression of cell cycle regulatory proteins: cyclin D1 and D3, cyclin-dependent kinase CDK4 and CDK6, and cell cycle inhibitors: p16, p21, and p27, was studied at the protein level (western blot). Treatment of melanoma cells with protein kinase inhibitors led to significantly decreased cell proliferation except the use of a GSK-3β kinase inhibitors-CHIR-99021. The significant decrease in the expression of selected cyclins and cyclin-dependent kinases (CDKs) with parallel increase in the expression of some of cyclin-dependent kinases inhibitors and in consequence meaningful reduction in melanoma cell proliferation by the combinations of inhibitors of signaling kinases clearly showed the crucial role of AKT, ERK 1/2, and mTOR signal transduction in melanoma progression. The results unanimously indicate those pathways as an important target for treatment of melanoma.
  
  
  
  
**CCND3**
-
5
of
10
  

### Combination of pan-RAF and MEK inhibitors in NRAS mutant melanoma.

5. Mol Cancer. 2015 Feb 3;14(1):27. doi: 10.1186/s12943-015-0293-5
  
  
Atefi M(1), Titz B(2), Avramis E(3), Ng C(4), Wong DJ(5), Lassen A(6), Cerniglia M(7), Escuin-Ordina ...
  
  
Author information: (1)Department of Medicine, Division of Hematology-Oncology, University of Califo ...
  
  
BACKGROUND: Approximately 20% of melanomas contain a mutation in NRAS. However no direct inhibitor of NRAS is available. One of the main signaling pathways downstream of NRAS is the MAPK pathway. In this study we investigated the possibility of blocking oncogenic signaling of NRAS by inhibiting two signaling points in the MAPK pathway. METHODS: Fourteen NRAS mutated human melanoma cell lines were treated with a pan-RAF inhibitor (PRi, Amgen Compd A), a MEK inhibitor (MEKi, trametinib) or their combination and the effects on proliferation, cell cycle progression, apoptosis, transcription profile and signaling of the cells were investigated. RESULTS: The majority of the cell lines showed a significant growth inhibition, with high levels of synergism of the PRi and MEKi combination. Sensitive cell lines showed induction of apoptosis by the combination treatment and there was a correlation between p-MEK levels and synergistic effect of the combination treatment. Proliferation of sensitive cell lines was blocked by the inhibition of the MAPK pathway, which also blocked expression of cyclin D1. However, in resistant cell lines, proliferation was blocked by combined inhibition of the MAPK pathway and cyclin D3, which is not regulated by the MAPK pathway. Resistant cell lines also showed higher levels of p-GSK3β and less perturbation of the apoptotic profile upon the treatment in comparison with the sensitive cell lines. CONCLUSIONS: The combination of PRi + MEKi can be an effective regimen for blocking proliferation of NRAS mutant melanomas when there is higher activity of the MAPK pathway and dependence of proliferation and survival on this pathway.
  
  
  
  
**CCND3**
-
6
of
10
  

### Identification of melanoma biomarkers based on network modules by integrating the human signaling network with microarrays.

6. J Cancer Res Ther. 2014 Nov;10 Suppl:C114-24. doi: 10.4103/0973-1482.145816
  
  
Huang C, Sheng Y, Jia J, Chen L(1).
  
  
Author information: (1)Department of Dermatology, Huashan Hospital, Shanghai Medical College, Fudan ...
  
  
BACKGROUND: Melanoma is a leading cause of cancer death. Thus, accurate prognostic biomarkers that will assist rational treatment planning need to be identified. METHODS: Microarray analysis of melanoma and normal tissue samples was performed to identify differentially expressed modules (DEMs) from the signaling network and ultimately detect molecular markers to support histological examination. Network motifs were extracted from the human signaling network. Then, significant expression-correlation differential modules were identified by comparing the network module expression-correlation differential scores under normal and disease conditions using the gene expression datasets. Finally, we obtained DEMs by the Wilcoxon rank test and considered the average gene expression level in these modules as the classification features for diagnosing melanoma. RESULTS: In total, 99 functional DEMs were identified from the signaling network and gene expression profiles. The area under the curve scores for cancer module genes, melanoma module genes, and whole network modules are 92.4%, 90.44%, and 88.45%, respectively. The classification efficiency rates for nonmodule features are 71.04% and 79.38%, which correspond to the features of cancer genes and melanoma cancer genes, respectively. Finally, we acquired six significant molecular biomarkers, namely, module 10 (CALM3, Ca 2+ , PKC, PDGFRA, phospholipase-g, PIB5PA, and phosphatidylinositol-3-kinase), module 14 (SRC, Src homology 2 domain-containing [SHC], SAM68, GIT1, transcription factor-4, CBLB, GRB2, VAV2, LCK, YES, PTCH2, downstream of tyrosine kinase [DOK], and KIT), module 16 (ELK3, p85beta, SHC, ZFYVE9, TGFBR1, TGFBR2, CITED1, SH3KBP1, HCK, DOK, and KIT), module 45 (RB, CCND3, CCNA2, CDK4, and CDK6), module 75 (PCNA, CDK4, and CCND1), and module 114 (PSD93, NMDAR, and FYN). CONCLUSION: We explored the gene expression profile and signaling network in a global view and identified DEMs that can be used as diagnostic or prognostic markers for melanoma.
  
  
  
  
**CCND3**
-
7
of
10
  

### Detection of copy number alterations in metastatic melanoma by a DNA fluorescence in situ hybridization probe panel and array comparative genomic hybridization: a southwest oncology group study (S9431).

7. Clin Cancer Res. 2008 May 15;14(10):2927-35. doi: 10.1158/1078-0432.CCR-07-4068
  
  
Moore SR(1), Persons DL, Sosman JA, Bobadilla D, Bedell V, Smith DD, Wolman SR, Tuthill RJ, Moon J, ...
  
  
Author information: (1)City of Hope, Duarte, California 91010, USA.
  
  
PURPOSE: Gene copy number alteration (CNA) is common in malignant melanoma and is associated with tumor development and progression. The concordance between molecular cytogenetic techniques used to determine CNA has not been evaluated on a large set of loci in malignant melanoma. EXPERIMENTAL DESIGN: A panel of 16 locus-specific fluorescence in situ hybridization (FISH) probes located on eight chromosomes was used to identify CNA in touch preparations of frozen tissue samples from 19 patients with metastatic melanoma (SWOG-9431). A subset (n = 11) was analyzed using bacterial artificial chromosome (BAC) array comparative genomic hybridization (aCGH) of DNA isolated directly from touch-preparation slides. RESULTS: By FISH, most samples showed loss near or at WISP3/6p21, CCND3/6q22, and CDKN2A/9p21 (>75% of samples tested). More than one third of CDKN2A/9p21 losses were biallelic. Gains of NEDD9/6p24, MET/7q31, and MYC/8q24 were common (57%, 47%, and 41%, respectively) and CNA events involving 9p21/7p12.3 and MET were frequently coincident, suggesting gain of the whole chromosome 7. Changes were confirmed by aCGH, which also uncovered many discreet regions of change, larger than a single BAC. Overlapping segments observed in >45% of samples included many of the loci analyzed in the FISH study, in addition to other WNT pathway members, and genes associated with TP53 pathways and DNA damage response, repair, and stability. CONCLUSIONS: This study outlines a set of CNAs at the gene and regional level, using FISH and aCGH, which may provide a benchmark for future studies and may be important in selection of individual therapy for patients with metastatic malignant melanoma.
  
  
  
  
**CCND3**
-
8
of
10
  

### Cyclin D3 expression in melanoma cells is regulated by adhesion-dependent phosphatidylinositol 3-kinase signaling and contributes to G1-S progression.

8. J Biol Chem. 2006 Sep 1;281(35):25644-51. doi: 10.1074/jbc.M600197200 Epub 2006 Jun 29.
  
  
Spofford LS(1), Abel EV, Boisvert-Adamo K, Aplin AE.
  
  
Author information: (1)Center for Cell Biology and Cancer Research, Albany Medical College, Albany, ...
  
  
D-type cyclins regulate G1 cell cycle progression by enhancing the activities of cyclin-dependent kinases (CDKs), and their expression is frequently altered in malignant cells. We and others have previously shown that cyclin D1 is up-regulated in melanoma cells through adhesion-independent MEK-ERK1/2 signaling initiated by mutant B-RAF. Here, we describe the regulation and role of cyclin D3 in human melanoma cells. Cyclin D3 expression was enhanced in a cell panel of human melanoma cell lines compared with melanocytes and was regulated by fibronectin-mediated phosphatidylinositol 3-kinase/Akt signaling but not MEK activity. RNA interference experiments demonstrated that cyclin D3 contributed to G1-S cell cycle progression and proliferation in melanoma cells. Overexpression of cyclin D1 did not recover the effects of cyclin D3 knockdown. Finally, immunoprecipitation studies showed that CDK6 is a major binding partner for cyclin D3, whereas CDK4 preferentially associated with cyclin D1. Together, these findings demonstrate that cyclin D3 is an important regulator of melanoma G1-S cell cycle progression and that D-type cyclins are differentially regulated in melanoma cells.
  
  
  
  
**CCND3**
-
9
of
10
  

### Suppression of C8161 melanoma metastatic ability by chromosome 6 induces differentiation-associated tyrosinase and decreases proliferation on adhesion-restrictive substrates mediated by overexpression of p21WAF1 and down-regulation of bcl-2 and cyclin D3.

9. Biochem Biophys Res Commun. 2001 Feb 16;281(1):159-65. doi: 10.1006/bbrc.2001.4330
  
  
Strasberg Rieber M(1), Welch DR, Rieber M.
  
  
Author information: (1)IVIC, Tumor Cell Biology, Apartado 21827, Caracas, 1020 A, Venezuela.
  
  
Metastatic tumors grow under conditions that restrict proliferation of non-metastatic, more differentiated cells. To investigate this prediction, we developed a simple adhesion-restrictive assay which allows proliferation of human metastatic C8161 melanoma, but prevents growth of neo 6.3/C8161 cells in which metastasis is suppressed by introduction of neo-tagged chromosome 6. We show that tyrosinase, a key enzyme in melanocytic cell differentiation, and expression of chromosome 6-encoded cell cycle modulators like p21WAF1 and cyclin D3 is selectively increased in C8161 tumors in which metastasisis is suppressed by chromosome 6. In the latter cells, growth arrest evidenced only under adhesion-restrictive conditions correlated with down-regulation of cyclin D3 and anti-apoptotic bcl-2. No comparable growth arrest or down-regulation was detected under comparable conditions in metastatic cells, which showed activation of invasion-associated MMP-9 92 kDa gelatinase B. Our data suggests that the metastasis-suppressing effects of chromosome 6 involving increased differentiation-associated tyrosinase and growth arrest on adhesion-restrictive substrates; are partly mediated by modulation of growth regulators, like p21WAF1 and cyclin D3.
  
  
  
  
**CCND3**
-
10
of
10
  

### Levels of cyclin D1 and D3 in malignant melanoma: deregulated cyclin D3 expression is associated with poor clinical outcome in superficial melanoma.

10. Clin Cancer Res. 2000 Sep;6(9):3614-20.
  
  
Flørenes VA(1), Faye RS, Maelandsmo GM, Nesland JM, Holm R.
  
  
Author information: (1)Department of Pathology, Institute for Cancer Research, The Norwegian Radium ...
  
  
We examined 172 primary (110 superficial and 62 nodular) and 73 metastatic melanomas, as well as 10 benign nevi, for protein expression of cyclin D1 and cyclin D3 and evaluated the relationship between deregulated protein levels and clinical outcome. For both proteins, a heterogeneous nuclear staining pattern was observed. Cyclin D3 was expressed by 96% of primary and 97% of metastatic melanomas. The corresponding percentages for cyclin D1 were 62% and 29%, respectively. In benign nevi, only rare cyclin D3-positive cells and no cyclin D1-positive cells were observed. High levels of cyclin D3 (>5% of the cells stained) were detected in 26 of 62 (42%) nodular melanomas and in 22 of 110 (20%) superficial tumors, whereas no such difference was observed with respect to cyclin D1. In superficial melanomas, a significant concordant staining pattern was observed between cyclin D1 and cyclin D3 (P = 0.0009), cyclin D1 and Ki-67 (P = 0.0001), cyclin D1 and cyclin A (P = 0.02), cyclin D3 and Ki-67 (P < 0.00001), and cyclin D3 and cyclin A (P = 0.002). Kaplan-Meier analysis revealed that high levels of cyclin D3 were an indicator of early relapse and decreased overall survival for patients with superficial (P = 0.001 and P = 0.009, respectively) but not nodular (P = 0.64 and P = 0.23) melanoma. Cyclin D1 did not have any impact on disease-free and overall survival for either of the subtypes. In conclusion, our results suggest that deregulation of cyclin D3 expression leading to increased proliferation may be a prognostic factor for superficial melanoma, whereas deregulated cell cycle machinery seems to have little impact on disease progression of nodular melanoma.
  
  
  
  
  

---

  

# CD163

**Signature gene in:** MDM
  
**Differentially expressed in:** MDM
  
  
**CD163**
-
1
of
10
  

### Gastric SMARCA4-deficient undifferentiated tumors: clinicopathological analysis of two cases in a single center.

1. Diagn Pathol. 2025 Apr 24;20(1):51. doi: 10.1186/s13000-025-01651-0
  
  
Cheng X(1)(2), Chen S(1)(2), Jiang X(1)(2), Li T(1)(3), Zhang H(1)(4), Huang F(5)(6), Bao T(7)(8).
  
  
Author information: (1)Shexian Branch, Second Affiliated Hospital, Zhejiang University School of Med ...
  
  
OBJECTIVES: Gastric SMARCA4-deficient undifferentiated tumors are rare and have a poor prognosis. We analyzed two cases of gastric SMARCA4-deficient undifferentiated tumors with clinicopathologic characteristics, treatment and flow-up. METHODS: Immunohistochemistry was used to evaluate the expression of BRG1 (SAMRCA4), SMARCB1 (INI-1), CKpan, Ki-67, CD3, CD20, CD163, PD-1, and PD-L1 in gastric SMARCA4-deficient undifferentiated tumors. Additionally, the clinical characteristics, imaging features, diagnosis, and treatment were analyzed. RESULTS: Two elderly male patients (69 and 61 years old) with a large ulcerated mass located in the gastric fundus and cardia. Histologically, the tumor is of low adhesion, diffusely infiltrating lamellar growth, without any percentage of epithelial differentiation zones, and with little stromal component. Tumor cells round, oval, a small amount of irregular shape, easy to see mitotic figures. Some of them had obvious nucleoli, and a few had multiple nucleoli. The cytoplasm varies, and some cells are more abundant. Significant vascular and neural invasion. BRG1(SMARCA4) was absent, INI-1 was present, and Ki-67 proliferation index was highly expressed (≥ 80%). The remaining sarcoma-specific markers were negative. In case 1, the epithelial markers were negative and the PD-L1 combined positive score was 5. In case 2, CKpan was weakly expressed in only a dozen cells, and the PDL1 CPS was 10. The two patients received chemotherapy and anti-PD1 immunotherapy after radical gastrectomy for gastric cancer. The postoperative follow-up time of the two patients was 16 (case 1) and 3 months (case 2), respectively. The general condition was good, no recurrence or metastasis was observed, and the plasma tumor markers were in the normal range. CONCLUSIONS: Large SMARCA4-deficient tumors are more likely to have massive necrosis on the surface, leading to negative biopsy results. This tumor has a diffuse lamellar growth and needs to be differentiated from a variety of tumors with similar morphology, such as lymphoma, malignant melanoma, neuroendocrine carcinoma and undifferentiated sarcoma. The tumor cells were negative or only slightly positive for CKpan increases the difficulty of pathological diagnosis of this disease. However, loss of BRG1 (SMARCA4) expression can confirm the diagnosis. Chemotherapy combined with anti-PD1 treatment may have potential benefits in the management of gastric SMARCA4-deficient undifferentiated tumors. However, given the rarity of these tumors and the limited number of cases in our study, further research with larger cohorts is needed to validate these preliminary results.
  
  
  
  
**CD163**
-
2
of
10
  

### MAGE-A4 induces non-small cell lung cancer and tumor-promoting plasma cell accumulation.

2. Sci Adv. 2025 Feb 14;11(7):eads4227. doi: 10.1126/sciadv.ads4227 Epub 2025 Feb 12.
  
  
Armstrong D(1)(2), Chang CY(1)(2), Hong MJ(2), Green L(3), Shen Y(4)(5), Hudson W(4)(5), Mauk KE(2), ...
  
  
Author information: (1)Translation Biology and Molecular Medicine Program, Baylor College of Medicin ...
  
  
Adaptive immunity is critical in eliminating tumors, but cancer-intrinsic factors can subvert this function. Melanoma antigen-A4 (MAGE-A4), a cancer-testis antigen, is expressed in solid tumors and correlates with poor survival, but its role in tumorigenesis and antitumor immunity remains unclear. We found that expression of MAGE-A4 was highly associated with the loss of PTEN, a tumor suppressor, in human non-small cell lung cancers (NSCLC). Here, we show that constitutive expression of human MAGE-A4 with Pten loss in mouse airway epithelia results in metastatic adenocarcinoma. Tumors showed distinct enrichment in IgA+ CD138+ CXCR4+ plasma cells (PCs) and increased expression of CXCL12 in endothelial cells. Consistently, human NSCLC expressing MAGE-A4 showed increased CD138+ IgA+ PCs surrounding tumors. Abrogation of PCs decreased tumor burden, increased activated T cell infiltration, and reduced CD163+CD206+ macrophages in the MAGE-A4-induced lung tumors. These findings suggest MAGE-A4 promotes NSCLC tumorigenesis, in part, through the recruitment and retention of IgA+ PCs in the lungs.
  
  
  
  
**CD163**
-
3
of
10
  

### Reprogramming the melanoma and immunosuppressive myeloid cells with esomeprazole-loaded PLGA nanoparticles.

3. iScience. 2024 Dec 9;28(1):111559. doi: 10.1016/j.isci.2024.111559 eCollection 2025 Jan 17.
  
  
Cerioli N(1), Bououdina W(2), Mereu A(1), Natsaridis E(3), Salsetta J(1), Cova A(1), Lupoli G(1), D' ...
  
  
Author information: (1)Translational Immunology Unit, Fondazione IRCCS Istituto Nazionale dei Tumori ...
  
  
Proton pump inhibitors have been explored for potentiating cancer therapies via reverting the tumor acidity and promoting the activation of anti-tumor immune responses. To regulate the intracellular pH of melanoma and immunosuppressive myeloid cells, we developed poly(L-lactide-co-glycolide) nanoparticles loaded with esomeprazole (ESO-NPs). The effect of ESO-NPs on melanoma cells was observed as alkalinization and reduction of melanin content accompanied by a decrease of microphthalmia-associated transcription factor (MITF), poliovirus receptor (PVR), and programmed death ligand 1 (PD-L1) immune checkpoint expression. ESO-NP treatment of melanoma-patient-derived and in vitro-induced myeloid-derived suppressor cells (MDSCs) reduced the expression of immunosuppression-associated molecules PD-L1, CD206, and CD163 on patient-derived myeloid cells while inducing the expression of co-stimulatory molecule CD86 and HLA-DR in the in vitro model. Our findings suggest that reprogramming the intracellular pH of melanoma and immune-suppression-associated myeloid cells with ESO-NPs can modulate the expression of proteins involved in resistance to cancer therapy and immunosuppression, thus potentially improving the response to immunotherapies.
  
  
  
  
**CD163**
-
4
of
10
  

### Programmed Death Ligand-1 in Melanoma and Extracellular Vesicles Promotes Local and Regional Immune Suppression through M2-like Macrophage Polarization.

4. Am J Pathol. 2025 Feb;195(2):306-320. doi: 10.1016/j.ajpath.2024.09.011 Epub 2024 Oct 29.
  
  
Huang L(1), Yang J(2), Zhu J(1), Wang H(1), Dong L(1), Guo Y(1), Chen Y(3), Zhang F(4), Xu DJ(1), Ou ...
  
  
Author information: (1)Department of Pathology and Laboratory Medicine, Perelman School of Medicine ...
  
  
Tumor-associated macrophages (TAMs) play dual roles (both pro- and antitumor) in tumor progression. TAMs induce programmed death ligand-1 (PD-L1) expression in cancer cells. However, the regulatory effects of PD-L1 in melanoma cells on TAM phenotypical switching remain underexplored. Herein, CD163 and MRC1 levels were significantly elevated in metastatic melanomas compared with those in primary melanomas, correlating with CD274 expression and predicted patient clinical outcomes. To study the mechanisms regulating M2-like polarization, PD-L1 was knocked out in both YUMM1.7 and B16-F10 melanoma cells. Knocking out PD-L1 (PD-L1KO) in melanoma resulted in a decelerated in vivo growth rate, accompanied by a significantly increased M1/M2 ratio, more dendritic cells, and enhanced activation of CD8+ T cells compared with wild-type (WT) melanoma cells. These alterations were associated with decreased expression of M2-associated chemokines (CCL2, CCL3, and CXCL2) and cytokines (IL6, IL10, and TGFB1). Mice harboring PD-L1KO melanomas exhibited elevated levels of CD8+ T cells in both the tumor-draining lymph nodes and the bloodstream compared with mice with PD-L1WT melanomas. Treatment with extracellular vesicles (EVs) derived from PD-L1KO melanoma resulted in a reduced tumor growth rate and fewer M2-like macrophages in the tumors compared with EVs from PD-L1WT melanomas. Therefore, these data suggest that PD-L1 in melanoma and melanoma-derived EVs induces M2-like polarization, contributing to local and regional immune suppression.
  
  
  
  
**CD163**
-
5
of
10
  

### A Case Series of Primary Cutaneous Sarcomatoid Carcinoma With Aberrant Smooth Muscle Actin Expression: A Clinicopathologic and Immunophenotypic Study.

5. Am J Dermatopathol. 2025 Feb 1;47(2):83-92. doi: 10.1097/DAD.0000000000002800 Epub 2024 Aug 14.
  
  
Shafi S(1), Gru AA(2)(3), Roman J(4), Maupin J(1), Plaza JA(5).
  
  
Author information: (1)Pathologist, Department of Pathology, The Ohio State University Wexner Medica ...
  
  
Primary cutaneous sarcomatoid squamous cell carcinoma can show significant histologic overlap with other malignant spindle cell tumors, posing a diagnostic challenge. Even with a wide array of immunohistochemical markers, the exact line of differentiation can be a challenge to identify in some cases. The picture is further complicated by the aberrant expression of myofibroblastic markers [such as smooth muscle actin (SMA)] by these neoplasms, along with a concomitant loss of conventional epithelial markers. The histologic differential diagnoses of primary cutaneous sarcomatoid squamous cell carcinoma include desmoplastic melanoma, leiomyosarcoma, and spindle cell atypical fibroxanthoma/pleomorphic dermal sarcoma (AFX/PDS). A retrospective analysis of 16 cases of PCSSCCs with SMA expression, obtained from large academic institutions, was performed and is summarized below. The tumors were in the scalp (6 cases), arm (4 cases), leg (2 cases), face (2 cases), hand (1 case), and neck (1 case). Immunohistochemical studies were performed in all cases with the following antibodies: AE1/AE3, CAM 5.2, MNF-116, p63, p40, CK5/6, S-100, SOX10, SMA, desmin, calponin, H-caldesmon, CD10, CD68, CD163, and CD34. Histopathologically, all cases were classified as high-grade malignant poorly differentiated neoplasms. Tumors were characterized by an infiltrative neoplasm that involved the entire reticular dermis and, in 7 cases, the subcutaneous fat. Three cases were associated with a well-differentiated squamous cell component. The neoplasms were composed of atypical spindle and epithelioid cells arranged in long and intersecting fascicles. All neoplasms were positive for epithelial markers (at least 1 marker), and all cases were strongly positive for SMA. Our data emphasize the diagnostic utility of multiple epithelial markers as a first screening tool in the detection and workup of malignant cutaneous sarcomatoid neoplasms. Awareness of SMA expression in these tumors can complicate its diagnosis, and it is important to recognize this aberrant immunophenotype to facilitate definitive diagnosis and avoid misdiagnosis.
  
  
  
  
**CD163**
-
6
of
10
  

### MAGE-A4-Responsive Plasma Cells Promote Non-Small Cell Lung Cancer.

6. bioRxiv [Preprint]. 2024 Jul 16:2024.07.10.602985. doi: 10.1101/2024.07.10.602985
  
  
Armstrong D, Chang CY, Hong MJ, Green L, Hudson W, Shen Y, Song LZ, Jammi S, Casal B, Creighton CJ, ...
  
  
Adaptive immunity is critical to eliminate malignant cells, while multiple tumor-intrinsic factors c ...
  
  
DOI: 10.1101/2024.07.10.602985 PMCID: PMC11275715 PMID: 39071307
  
  
  
  
**CD163**
-
7
of
10
  

### [Aqueous extract of Chuan Xiong Rhizoma enhances inhibitory effect of temozolomide against brain metastasis of melanoma in mice].

7. Nan Fang Yi Ke Da Xue Xue Bao. 2024 Jun 20;44(6):1088-1097. doi: 10.12122/j.issn.1673-4254.2024.06.09
  
  
[Article in Chinese]
  
  
Zhao Q(1), Zhang Z(1), Zhou X(1), Rong X(1), Liu X(1), Zhao X(1), Wang H(1), Pang J(1), Li S(1), Li ...
  
  
Author information: (1)School of Pharmacy, Bengbu Medical University, Bengbu 233030, China. (2)Anhui Provincial Key Laboratory of New Technology for Chinese Herbal Pieces Manufacturing, Bozhou 236800, China. (3)Postdoctoral Workstation at Anhui Xiehecheng Pharmaceutical Yinpian Co. Ltd, Bozhou 236800, China.
  
  
  
  
**CD163**
-
8
of
10
  

### Giant thigh hemosiderotic/aneurysmal dermatofibroma: Case report with radiologic-pathologic correlation.

8. Rev Esp Patol. 2024 Jul-Sep;57(3):217-224. doi: 10.1016/j.patol.2024.04.001 Epub 2024 May 20.
  
  
Pena-Burgos EM(1), D'Angelo C(2), Díez-Corral MC(2), Sánchez Villanueva E(3), Tapia-Viñe M(4), Pozo- ...
  
  
Author information: (1)Príncipe de Asturias University Hospital, Pathology Department, Spain. Electr ...
  
  
Hemosiderotic/aneurysmal variant of dermatofibroma (DF) is infrequent and may be misdiagnosed with malignant lesions. We report the case of a giant (7.6cm) subcutaneous hemosiderotic/aneurysmal DF (H/ADF) of the thigh in a 53-year-old female patient. Internal arterial and venous hypervascularity was seen by spectral Doppler ultrasound. Magnetic resonance image showed a discrete homogeneous hypointense in T1-weighted images (WI) and T2-WI mass, with hyperintense areas in fat-suppressed T2-WI. The histology revealed a monotonous fusocelular proliferation without atypia, positive for CD163, factor XIIIa and CD10. Widely distributed hemosiderin pigment and two blood-filled pseudovascular spaces lacking endothelial lining were present. H/ADF was diagnosed. The mass was removed but surgical margins were affected. The patient did not present local relapse or distant metastasis. H/ADF are unusual cutaneous soft tissue tumours that can be clinically, radiologically and histopathologically confused with malignant lesions such as melanomas, vascular lesions or sarcomas, especially in giant cases.
  
  
  
  
**CD163**
-
9
of
10
  

### Proliferating macrophages in human tumours show characteristics of monocytes responding to myelopoietic growth factors.

9. Front Immunol. 2024 Jun 5;15:1412076. doi: 10.3389/fimmu.2024.1412076 eCollection 2024.
  
  
Park SM(1)(2), Chen CJ(1)(2), Verdon DJ(1)(2), Ooi MPY(1), Brooks AES(1)(2), Martin RCW(3), Mathy JA ...
  
  
Author information: (1)School of Biological Sciences, The University of Auckland, Auckland, New Zeal ...
  
  
Macrophages play essential roles in maintaining tissue homeostasis and immune defence. However, their extensive infiltration into tumours has been linked to adverse outcomes in multiple human cancers. Within the tumour microenvironment (TME), tumour-associated macrophages (TAMs) promote tumour growth and metastasis, making them prime targets for cancer immunotherapy. Recent single-cell analysis suggest that proliferating TAMs accumulate in human cancers, yet their origins and differentiation pathways remain uncertain. Here, we show that a subpopulation of CD163+ TAMs proliferates in situ within the TME of melanoma, lung cancer, and breast cancer. Consistent with their potential role in suppressing anti-tumour activities of T cells, CD163+ TAMs express a range of potent immunosuppressive molecules, including PD-L1, PD-L2, IL-10, and TGF-β. Other phenotypic markers strongly suggested that these cells originate from CD14+ CCR2+ monocytes, a cell population believed to have minimal capacity for proliferation. However, we demonstrate in vitro that certain myelopoietic cytokines commonly available within the TME induce robust proliferation of human monocytes, especially the combination of interleukin 3 (IL-3) and Macrophage Colony-Stimulating Factor 1 (M-CSF). Monocytic cells cultured with these cytokines efficiently modulate T cell proliferation, and their molecular phenotype recapitulates that of CD163+ TAMs. IL-3-driven proliferation of monocytic cells can be completely blocked by IL-4, associated with the induction of CDKN1A, alongside the upregulation of transcription factors linked to dendritic cell function, such as BATF3 and IRF4. Taken together, our work suggests several novel therapeutic routes to reducing immunosuppressive TAMs in human tumours, from blocking chemokine-mediated recruitment of monocytes to blocking their proliferation.
  
  
  
  
**CD163**
-
10
of
10
  

### Integrative molecular and spatial analysis reveals evolutionary dynamics and tumor-immune interplay of in situ and invasive acral melanoma.

10. Cancer Cell. 2024 Jun 10;42(6):1067-1085.e11. doi: 10.1016/j.ccell.2024.04.012 Epub 2024 May 16.
  
  
Liu H(1), Gao J(2), Feng M(3), Cheng J(3), Tang Y(4), Cao Q(3), Zhao Z(3), Meng Z(3), Zhang J(4), Zh ...
  
  
Author information: (1)Peking University-Yunnan Baiyao International Medical Research Center, Peking ...
  
  
In acral melanoma (AM), progression from in situ (AMis) to invasive AM (iAM) leads to significantly reduced survival. However, evolutionary dynamics during this process remain elusive. Here, we report integrative molecular and spatial characterization of 147 AMs using genomics, bulk and single-cell transcriptomics, and spatial transcriptomics and proteomics. Vertical invasion from AMis to iAM displays an early and monoclonal seeding pattern. The subsequent regional expansion of iAM exhibits two distinct patterns, clonal expansion and subclonal diversification. Notably, molecular subtyping reveals an aggressive iAM subset featured with subclonal diversification, increased epithelial-mesenchymal transition (EMT), and spatial enrichment of APOE+/CD163+ macrophages. In vitro and ex vivo experiments further demonstrate that APOE+CD163+ macrophages promote tumor EMT via IGF1-IGF1R interaction. Adnexal involvement can predict AMis with higher invasive potential whereas APOE and CD163 serve as prognostic biomarkers for iAM. Altogether, our results provide implications for the early detection and treatment of AM.
  
  
  
  
  

---

  

# CDH13

**Signature gene in:** Endothelial cells
  
**Differentially expressed in:** Endothelial cells
  
  
**CDH13**
-
1
of
10
  

### The Role of T-Cadherin (CDH13) in Treatment Options with Garcinol in Melanoma.

1. Cancers (Basel). 2024 May 12;16(10):1853. doi: 10.3390/cancers16101853
  
  
Staebler S(1), Hoechst S(1), Thongmao A(1), Schneider N(1), Bosserhoff AK(1), Kuphal S(1).
  
  
Author information: (1)Institute of Biochemistry, Friedrich Alexander University Erlangen-Nürnberg, ...
  
  
Targeted therapies with chemotherapeutic agents and immunotherapy with checkpoint inhibitors are among the systemic therapies recommended in the guidelines for clinicians to treat melanoma. Although there have been constant improvements in the treatment of melanoma, resistance to the established therapies continues to occur. Therefore, the purpose of this study was to explore the function of garcinol with regards to specific cancer properties such as proliferation and apoptosis. Garcinol, a natural compound isolated from the plant also known as mangosteen (Garcinia mangostana), is a newly discovered option for cancer treatment. Numerous pharmaceutical substances are derived from plants. For example, the derivates of camptothecin, extracted from the bark of the Chinese tree of happiness (Camptotheca acuminate), or paclitaxel, extracted from the bark of the Western yew tree (Taxus brevifolia), are used as anti-cancer drugs. Here, we show that garcinol reduced proliferation and induced apoptosis in melanoma cell lines. In addition, we found that those cells that are positive for the expression of the cell-cell adhesion molecule T-cadherin (CDH13) respond more sensitively to treatment with garcinol. After knock-down experiments with an siRNA pool against T-cadherin, the sensitivity to garcinol decreased and proliferation and anti-apoptotic behavior of the cells was restored. We conclude that patients who are T-cadherin-positive could especially benefit from a therapy with garcinol.
  
  
  
  
**CDH13**
-
2
of
10
  

### PTEN Hamartoma Tumor Syndrome: Skin Manifestations and Insights Into Their Molecular Pathogenesis.

2. Front Med (Lausanne). 2021 Jul 27;8:688105. doi: 10.3389/fmed.2021.688105 eCollection 2021.
  
  
Innella G(1)(2), Bonora E(1), Neri I(3), Virdi A(3), Guglielmo A(3), Pradella LM(1), Ceccarelli C(4) ...
  
  
Author information: (1)Department of Medical and Surgical Sciences, Center for Studies on Hereditary ...
  
  
Germline PTEN pathogenic variants cause a spectrum of disorders collectively labeled PTEN Hamartoma Tumor Syndrome (PHTS) and featured by hamartomas, developmental anomalies and increased cancer risk. Studies on experimental models provided evidence that PTEN is a "haploinsufficient" tumor-suppressor gene, however, mechanisms involved in the pathogenesis of clinical manifestations in PHTS patients remain elusive. Beyond analyzing clinical and molecular features of a series of 20 Italian PHTS patients, we performed molecular investigations to explore the mechanisms involved in the pathogenesis of PTEN-associated manifestations, with special focus on mucocutaneous manifestations. Typical mucocutaneous features were present in all patients assessed, confirming that these are the most important clue to the diagnosis. The most frequent were papules located in the trunk or extremities (73.7%), oral mucosa papules (68.4%), acral/palmoplantar keratosis and facial papules (both 57.9%), according with literature data. Molecular analyses on one trichilemmoma suggested that the wild-type PTEN allele was retained and expressed, reinforcing the evidence that PTEN does not require a second somatic hit to initiate pathogenic processes. Unexpectedly, one patient also displayed a cutaneous phenotype consistent with atypical mole/melanoma syndrome; no variants were detected in known melanoma genes, but Whole Exome Sequencing showed the rare truncating variant c.495G>A in the CDH13 gene that might have cooperated with PTEN-haploinsufficiency to generate such phenotype. Our findings confirm the reproducibility of known PHTS manifestations in real-world practice, highlighting the role of mucocutaneous manifestations in facilitating prompt diagnosis of the syndrome, and provide some insights into the pathogenic process induced by PTEN alterations, which may contribute to its understanding.
  
  
  
  
**CDH13**
-
3
of
10
  

### Genome-wide association study identifies CDH13 as a susceptibility gene for rhododendrol-induced leukoderma.

3. Pigment Cell Melanoma Res. 2020 Nov;33(6):826-833. doi: 10.1111/pcmr.12904 Epub 2020 Jun 29.
  
  
Okamura K(1), Abe Y(1), Naka I(2), Ohashi J(2), Yagami A(3)(4), Matsunaga K(3)(5), Kobayashi Y(6), F ...
  
  
Author information: (1)Department of Dermatology, Faculty of Medicine, Yamagata University, Yamagata ...
  
  
Racemic RS-4-(4-hydroxyphenyl)-2-butanol (rhododendrol; trade name: Rhododenol [RD]), which is used in topical skin-lightening cosmetics, was unexpectedly reported in Japan to induce leukoderma or vitiligo called RD-induced leukoderma (RIL) after repeated application. To our knowledge, no studies have investigated chemical-induced vitiligo pathogenesis on a genome-wide scale. Here, we conducted a genome-wide association study (GWAS) for 147 cases and 112 controls. CDH13, encoding a glycosylphosphatidylinositol-anchored protein called T-cadherin (T-cad), was identified as the strongest RIL susceptibility gene. RD sensitivity was remarkably increased by T-cad knockdown in cultured normal human melanocytes. Furthermore, we confirmed tyrosinase upregulation and downregulation of the anti-apoptotic molecules (BCL-2 and BCL-XL), suggesting that T-cad is associated with RD via tyrosinase or apoptotic pathway regulation. Finally, monobenzyl ether of hydroquinone sensitivity also tended to increase with T-cad knockdown, suggesting that the T-cad could be a candidate susceptibility gene for RIL and other chemical-induced vitiligo forms. This is the first GWAS for chemical-induced vitiligo, and it could be a useful model for studying the disease's genetic aspects.
  
  
  
  
**CDH13**
-
4
of
10
  

### [Establishment of cisplatin-resistant melanoma cell line with stable expression of T-cadherin and its biological characteristics].

4. Zhong Nan Da Xue Xue Bao Yi Xue Ban. 2019 Nov 28;44(11):1214-1221. doi: 10.11817/j.issn.1672-7347.2019.180649
  
  
[Article in Chinese; Abstract available in Chinese from the publisher]
  
  
Lu H(1), Guo J(1), He L(1), Liu L(1), Duan X(1).
  
  
Author information: (1)Department of Dermatology, Affiliated Hospital of Chengde Medical College, Chengde Hebei 067000, China.
  
  
  
  
**CDH13**
-
5
of
10
  

### Aberrant DNA methylation is associated with aggressive clinicopathological features and poor survival in cutaneous melanoma.

5. Br J Dermatol. 2018 Aug;179(2):394-404. doi: 10.1111/bjd.16254 Epub 2018 May 25.
  
  
de Unamuno Bustos B(1), Murria Estal R(2), Pérez Simó G(2), Simarro Farinos J(2), Pujol Marco C(1), ...
  
  
Author information: (1)Department of Dermatology, Hospital Universitari i Politecnic La Fe, Valencia ...
  
  
Comment in Br J Dermatol. 2018 Aug;179(2):250-251. doi: 10.1111/bjd.16772.
  
  
  
  
**CDH13**
-
6
of
10
  

### DNA methylation and gene deletion analysis of brain metastases in melanoma patients identifies mutually exclusive molecular alterations.

6. Neuro Oncol. 2014 Nov;16(11):1499-509. doi: 10.1093/neuonc/nou107 Epub 2014 Jun 26.
  
  
Marzese DM(1), Scolyer RA(1), Roqué M(1), Vargas-Roig LM(1), Huynh JL(1), Wilmott JS(1), Murali R(1) ...
  
  
Author information: (1)Department of Molecular Oncology, John Wayne Cancer Institute, Santa Monica, ...
  
  
BACKGROUND: The brain is a common target of metastases for melanoma patients. Little is known about the genetic and epigenetic alterations in melanoma brain metastases (MBMs). Unraveling these molecular alterations is a key step in understanding their aggressive nature and identifying novel therapeutic targets. METHODS: Genome-wide DNA methylation analyses of MBMs (n = 15) and normal brain tissues (n = 91) and simultaneous multigene DNA methylation and gene deletion analyses of metastatic melanoma tissues (99 MBMs and 43 extracranial metastases) were performed. BRAF and NRAS mutations were evaluated in MBMs by targeted sequencing. RESULTS: MBMs showed significant epigenetic heterogeneity. RARB, RASSF1, ESR1, APC, PTEN, and CDH13 genes were frequently hypermethylated. Deletions were frequently detected in the CDKN2A/B locus. Of MBMs, 46.1% and 28.8% had BRAF and NRAS missense mutations, respectively. Compared with lung and liver metastases, MBMs exhibited higher frequency of CDH13 hypermethylation and CDKN2A/B locus deletion. Mutual exclusivity between hypermethylated genes and CDKN2A/B locus deletion identified 2 clinically relevant molecular subtypes of MBMs. CDKN2A/B deletions were associated with multiple MBMs and frequently hypermethylated genes with shorter time to brain metastasis. CONCLUSIONS: Melanoma cells that colonize the brain harbor numerous genetically and epigenetically altered genes. This study presents an integrated genomic and epigenomic analysis that reveals MBM-specific molecular alterations and mutually exclusive molecular subtypes.
  
  
  
  
**CDH13**
-
7
of
10
  

### Loss of T-cadherin (CDH-13) regulates AKT signaling and desensitizes cells to apoptosis in melanoma.

7. Mol Carcinog. 2014 Aug;53(8):635-47. doi: 10.1002/mc.22018 Epub 2013 Apr 26.
  
  
Bosserhoff AK(1), Ellmann L, Quast AS, Eberle J, Boyle GM, Kuphal S.
  
  
Author information: (1)Institute of Pathology, Molecular Pathology, University of Regensburg, Regens ...
  
  
An understanding of signaling pathways is a basic requirement for the treatment of melanoma. Currently, kinases are at the center of melanoma therapies. According to our research, additional alternative molecules are equally important for development of melanoma. In this regard, cancer progression is, among other factors, driven by an altered adhesion via cadherins. For instance, the de-regulated expression of the adhesion molecule T-cadherin is found in various cancer types, including melanoma, and influences migration and invasion. T-cadherin is thought to affect cellular function largely through its signaling and not its adhesion properties because the molecule is anchored into the cell membrane by a glycosylphosphatidylinositol (GPI) moiety. However, detailed knowledge about the consequences of the loss of T-cadherin in melanoma is currently lacking. For this reason, we were interested in assessing which signaling pathways are initiated by T-cadherin. The tumor growth of subcutaneously injected T-cadherin-positive melanoma cells was diminished compared with T-cadherin-negative cells in nude mice. The difference in tumor volume was not due to decreased proliferation but rather due to increased apoptosis. After the expression of T-cadherin was induced, we detected V-AKT murine thymoma viral oncogene homolog (AKT) and FoxO3a hypophosphorylation accompanied by the downregulation of the antiapoptotic molecules BCL-2, BCL-x and Clusterin. Furthermore, we detected a diminished transcriptional activity of CREB and AP-1. We demonstrated that T-cadherin functions as a pro-apoptotic tumor suppressor that antagonizes AKT/CREB/AP-1/FoxO3a signaling, whereas NFκB, TCF/LEF and mTOR are not part of the T-cadherin signaling pathway. Notably, we found that the restoration of T-cadherin in melanoma cells causes sensitization to apoptosis induced by CD95/Fas antibody CH-11.
  
  
  
  
**CDH13**
-
8
of
10
  

### BRN2 is a transcriptional repressor of CDH13 (T-cadherin) in melanoma cells.

8. Lab Invest. 2012 Dec;92(12):1788-800. doi: 10.1038/labinvest.2012.140 Epub 2012 Oct 15.
  
  
Ellmann L(1), Joshi MB, Resink TJ, Bosserhoff AK, Kuphal S.
  
  
Author information: (1)Institute of Pathology, Molecular Pathology, University of Regensburg, Regens ...
  
  
T-cadherin (cadherin 13, H-cadherin, gene name CDH13) has been proposed to act as a tumor-suppressor gene as its expression is significantly diminished in several types of carcinomas, including melanomas. Allelic loss and promoter hypermethylation have been proposed as mechanisms for silencing of CDH13. However, they do not account for loss of T-cadherin expression in all carcinomas, and other genetic or epigenetic alterations can be presumed. The present study investigated transcriptional regulation of CDH13 in melanoma. Bioinformatical analysis pointed to the presence of known BRN2 (also known as POU3F2 and N-Oct-3)-binding motifs in the CDH13 promoter sequence. We found an inverse correlation between BRN2 and T-cadherin protein and transcript expression. Reporter gene analysis and electrophoretic mobility shift assays in melanoma cells demonstrated that CDH13 is a direct target of BRN2 and that BRN2 is a functional transcriptional repressor of CDH13 promoter activity. The regulatory binding element of BRN2 was located -219 bp of the CDH13 promoter proximal to the start codon and was identified as 5'-CATGCAAAA-3'. Ectopic expression of BRN2 in BRN2-negative/T-cadherin-positive melanoma cells resulted in suppression of CDH13 promoter activity, whereas BRN2 knockdown in BRN2-positive/T-cadherin-negative melanoma cells resulted in re-expression of T-cadherin transcripts and protein. Transcriptional repression of CDH13 by BRN2 may participate in malignant transformation of melanoma by increasing invasion and migration potentials of melanoma cells. The study has identified CDH13 as a novel direct BRN2 transcriptional target gene and has advanced knowledge of mechanisms underlying loss of T-cadherin expression in melanoma.
  
  
  
  
**CDH13**
-
9
of
10
  

### E-cadherin cell-cell communication in melanogenesis and during development of malignant melanoma.

9. Arch Biochem Biophys. 2012 Aug 1;524(1):43-7. doi: 10.1016/j.abb.2011.10.020 Epub 2011 Nov 9.
  
  
Kuphal S(1), Bosserhoff AK.
  
  
Author information: (1)Institute of Pathology, University of Regensburg, D-93053 Regensburg, Germany ...
  
  
Cell-cell communication is necessary for the crosstalk between cells that constitute multicellular organisms and is essential for cells to coordinate their physiological behavior to create cohesive tissues. Cellular crosstalk is not only controlled by molecules, like growth factors, hormones, ions and G-proteins, etc. but also by cell-cell contacts. These contacts are essential for intercellular communication and are involved in survival, apoptosis, proliferation, differentiation and homeostasis of entire tissues. In polarized epithelia of vertebrates, the adherent junction is part of the tripartite junctional complex that is localized at the juxtaluminal region, which includes tight junctions (including claudins, occludins, and zonula occludens proteins), desmosomal junctions (including desmogleins), and adherent junctions. In focus of the manuscript are adherent molecules of the cadherin superfamily of the skin. In the normal epidermis, melanocytes and keratinocytes are mostly connected via E-cadherin, P-cadherin and H-cadherin [1-3]. Melanocytes that reside in the basal layer of the epidermis predominantly contain E-cadherin and H-cadherin, whereas those that reside in the hair follicles are rich in P-cadherin [2]. The regulation and role of E-cadherin during melanoma development will be the focus of this review.
  
  
  
  
**CDH13**
-
10
of
10
  

### Melanoblasts in culture as an in vitro system to determine molecular changes in melanoma.

10. Exp Dermatol. 2011 May;20(5):435-40. doi: 10.1111/j.1600-0625.2011.01271.x
  
  
Bosserhoff AK(1), Ellmann L, Kuphal S.
  
  
Author information: (1)Institute of Pathology, University of Regensburg, Regensburg, Germany.
  
  
Many consolidated findings have revealed that cancer formation resembles events of embryonic development. In particular, the network of transcription factors and adhesion molecules is very similar when comparing neural crest-derived melanoblasts and melanoma cells. The main difference is found in the manifestation of distinct genes in melanoma, whereas in neural crest cells gene expression is tightly regulated to promote either a migratory or stationary phenotype. We established a cell culture system to generate melanoblast-related cells (MBrc) out of melanocytes as originally described by Cook et al. First, we confirmed the typical gene expression pattern of BRN-2, SOX10, PAX3 and EDNRB. Furthermore, we identified enhanced migration and proliferation similar to that of melanoma cells. Our intention of using this system was to classify the known 'melanoma-associated genes' into a subgroup of genes solely regulated by the differentiation process and a second subgroup that is unaffected by differentiation and is potentially important to the stabilization of a melanoma phenotype. The expression of melanoma-associated genes (N-cadherin, MUC-18, integrin β3, α3, α5, αv, SLUG, TBX3, HIF1-α, BMP-4 and bFGF) was enhanced in MBrc which were de-differentiated out of melanocytes. E-cadherin, H-cadherin and β-catenin, prevalently found to be downregulated in melanoma, were diminished in MBrc. Remarkably, the transcription factor SNAIL was unaffected by differentiation and could be one key molecule in early melanoma development that is of prevailing importance. In summary, we feel that the analysis of MBrc generated in a reproducible system will give new insight into the role and importance of melanoma-associated genes.
  
  
  
  
  

---

  

# CELF2

**Signature gene in:** Monocytes
  
**Differentially expressed in:** Monocytes
  
  
**CELF2**
-
1
of
1
  

### Identification of prognostic genes in uveal melanoma microenvironment.

1. PLoS One. 2020 Nov 16;15(11):e0242263. doi: 10.1371/journal.pone.0242263 eCollection 2020.
  
  
Luo H(1)(2), Ma C(1)(3).
  
  
Author information: (1)Charité-Universitätsmedizin Berlin, Corporate Member of Freie Universität Ber ...
  
  
BACKGROUND: Uveal melanoma (UM) is the most common primary intraocular malignancy in adults. Many previous studies have demonstrated that the infiltrating of immune and stromal cells in the tumor microenvironment contributes significantly to prognosis. METHODS: Dataset TCGA-UVM, download from TCGA portal, was taken as the training cohort, and GSE22138, obtained from GEO database, was set as the validation cohort. ESTIMATE algorithm was applied to find intersection differentially expressed genes (DEGs) among tumor microenvironment. Kaplan-Meier analysis and univariate Cox regression model were performed on intersection DEGs to initial screen for potential prognostic genes. Then these genes entered into the validation cohort for validation using the same methods as that in the training cohort. Moreover, we conducted correlation analyses between the genes obtained in the validation cohort and the status of chromosome 3, chromosome 8q, and tumor metastasis to get prognosis genes. At last, the immune infiltration analysis was performed between the prognostic genes and 6 main kinds of tumor-infiltrating immune cells (TICs) for understanding the role of the genes in the tumor microenvironment. RESULTS: 959 intersection DEGs were found in the UM microenvironment. Kaplan-Meier and Cox analysis was then performed in the training and validation cohorts on these DEGs, and 52 genes were identified with potential prognostic value. After comparing the 52 genes to chromosome 3, chromosome 8q, and metastasis, we obtained 21 genes as the prognostic genes. The immune infiltration analysis showed that Neutrophil had the potential prognostic ability, and almost every prognostic gene we had identified was correlated with abundances of Neutrophil and CD8+ T Cell. CONCLUSIONS: Identifying 21 prognosis genes (SERPINB9, EDNRB, RAPGEF3, HFE, RNF43, ZNF415, IL12RB2, MTUS1, NEDD9, ZNF667, AZGP1, WARS, GEM, RAB31, CALHM2, CA12, MYEOV, CELF2, SLCO5A1, ISM1, and PAPSS2) could accurately identify patients' prognosis and had close interactions with Neutrophil in the tumor environment, which may provide UM patients with personalized prognosis prediction and new treatment insights.
  
  
  
  
  

---

  

# CERS6

**Signature gene in:** Dendritic cells
  
**Differentially expressed in:** Dendritic cells
  
  
**CERS6**
-
1
of
5
  

### Exercise Promotes Pro-Apoptotic Ceramide Signaling in a Mouse Melanoma Model.

1. Cancers (Basel). 2022 Sep 2;14(17):4306. doi: 10.3390/cancers14174306
  
  
Lee J(1), Savage H(1)(2), Maegawa S(1), Ballarò R(1), Pareek S(1)(2), Guerrouahen BS(1), Gopalakrish ...
  
  
Author information: (1)Department of Pediatrics Research, The University of Texas MD Anderson Cancer ...
  
  
Ceramides are essential sphingolipids that mediate cell death and survival. Low ceramide content in melanoma is one mechanism of drug resistance. Thus, increasing the ceramide content in tumor cells is likely to increase their sensitivity to cytotoxic therapy. Aerobic exercise has been shown to modulate ceramide metabolism in healthy tissue, but the relationship between exercise and ceramide in tumors has not been evaluated. Here, we demonstrate that aerobic exercise causes tumor cell apoptosis and accumulation of pro-apoptotic ceramides in B16F10 but not BP melanoma models using mice. B16F10 tumor-bearing mice were treated with two weeks of moderate treadmill exercise, or were control, unexercised mice. A reverse-phase protein array was used to identify canonical p53 apoptotic signaling as a key pathway upregulated by exercise, and we demonstrate increased apoptosis in tumors from exercised mice. Consistent with this finding, pro-apoptotic C16-ceramide, and the ceramide generating enzyme ceramide synthase 6 (CERS6), were higher in B16F10 tumors from exercised mice, while pro-survival sphingosine kinase 1 (Sphk1) was lower. These data suggest that exercise contributes to B16F10 tumor cell death, possibly by modulating ceramide metabolism toward a pro-apoptotic ceramide/sphingosine-1-phosphate balance. However, these results are not consistent in BP tumors, demonstrating that exercise can have different effects on tumors of different patient or mouse origin with the same diagnosis. This work indicates that exercise might be most effective as a therapeutic adjuvant with therapies that kill tumor cells in a ceramide-dependent manner.
  
  
  
  
**CERS6**
-
2
of
5
  

### Comprehensive analysis of LASS6 expression and prognostic value in ovarian cancer.

2. J Ovarian Res. 2021 Sep 7;14(1):117. doi: 10.1186/s13048-021-00868-z
  
  
Xing J(1), Yi J(2).
  
  
Author information: (1)Department of Neurosurgery, The Affiliated Traditional Chinese Medicine Hospi ...
  
  
BACKGROUND: Ceramide plays an important role in the occurrence and development of tumor. The synthesis of ceramide needs the participation of LASS. Current studies have shown that different LASS family members play different functions in tumors, especially LASS6, has been proved to play a key role in breast cancer, gastric cancer, melanoma and so on, but the research on ovarian cancer is very limited. METHODS: Bioinformatics web resources, including Oncomine, UALCAN, Kaplan-Meier Plotter and TIMER were used to analyze the expression profile, prognostic value and immune infiltration of LASS6. The related genes of LASS6 in ovarian cancer were mined by Regulome Explorer and LinkedOmics database, and cluster analysis was done by DAVID. The PPI network involving LASS6 was constructed by STRING database. Finally, the correlation between 10 genes and LASS6 was analyzed by GEPIA database, and their prognostic value in ovarian cancer was analyzed by Kaplan-Meier plotter. RESULTS: The expression of LASS6 was up-regulated in ovarian cancer, which was related to the progression and poor prognosis of ovarian cancer. Through GO/KEGG cluster analysis, we also found that LASS6 may affect calcium ion channel and its transport pathways. The analysis of regulatory network involved in LASS6 showed that the high mRNAs of 7 key genes were associated with poor prognosis of OS in patients with ovarian cancer, among which DEGS1 was the most significant. CONCLUSIONS: LASS6 may play an important role in the regulation of calcium pathway and become a new therapeutic target and potential prognostic marker in ovarian cancer.
  
  
  
  
**CERS6**
-
3
of
5
  

### Aging-dependent mitochondrial dysfunction mediated by ceramide signaling inhibits antitumor T cell response.

3. Cell Rep. 2021 May 4;35(5):109076. doi: 10.1016/j.celrep.2021.109076
  
  
Vaena S(1), Chakraborty P(2), Lee HG(1), Janneh AH(1), Kassir MF(1), Beeson G(3), Hedley Z(2), Yalci ...
  
  
Author information: (1)Departments of Biochemistry and Molecular Biology, Medical University of Sout ...
  
  
We lack a mechanistic understanding of aging-mediated changes in mitochondrial bioenergetics and lipid metabolism that affect T cell function. The bioactive sphingolipid ceramide, induced by aging stress, mediates mitophagy and cell death; however, the aging-related roles of ceramide metabolism in regulating T cell function remain unknown. Here, we show that activated T cells isolated from aging mice have elevated C14/C16 ceramide accumulation in mitochondria, generated by ceramide synthase 6, leading to mitophagy/mitochondrial dysfunction. Mechanistically, aging-dependent mitochondrial ceramide inhibits protein kinase A, leading to mitophagy in activated T cells. This aging/ceramide-dependent mitophagy attenuates the antitumor functions of T cells in vitro and in vivo. Also, inhibition of ceramide metabolism or PKA activation by genetic and pharmacologic means prevents mitophagy and restores the central memory phenotype in aging T cells. Thus, these studies help explain the mechanisms behind aging-related dysregulation of T cells' antitumor activity, which can be restored by inhibiting ceramide-dependent mitophagy.
  
  
  
  
**CERS6**
-
4
of
5
  

### Silencing of CERS6 increases the invasion and glycolysis of melanoma WM35, WM451 and SK28 cell lines via increased GLUT1-induced downregulation of WNT5A.

4. Oncol Rep. 2016 May;35(5):2907-15. doi: 10.3892/or.2016.4646 Epub 2016 Mar 2.
  
  
Tang Y(1), Cao K(2), Wang Q(2), Chen J(1), Liu R(1), Wang S(1), Zhou J(1), Xie H(3).
  
  
Author information: (1)Department of Plastic Surgery, The Third Xiangya Hospital of Central South Un ...
  
  
Ceramide synthases (CerSs) have been shown to regulate numerous aspects of cancer development. CERS6 has been suggested to be involved in cancer etiology. However, little is known concerning the exact effect of CERS6 on the malignant behavior of melanoma, including glycolysis, proliferation and invasion. In the present study, we found that the expression of CERS6 was low in the melanoma cell lines, including WM35, WM451 and SK-28, and the expression level was related to the malignanct behavior of the melanoma cell lines. We constructed overexpression and silencing models of CERS6 in three melanoma cell lines and found that silencing of CERS6 promoted the ability of proliferation and invasion in the melanoma cell lines. Additionally, downregulation of CERS6 upregulated the activity of glycolysis-related enzyme, and enhanced the expression of glycolysis-related genes, including GLUT1 and MCT1. Furthermore, we identified the genes whose expression levels were changed after silencing of CERS6 by gene microarray. The expression of glycolysis-related gene SLC2A1 (also known as GLUT1) was found to be upregulated, while notably WNT5A was downregulated. The altered expression of GLUT1 and WNT5A was verified by qPCR and western blotting. Furthermore, silencing of GLUT1 in the melanoma cells resulted in the increased expression of WNT5A and the decreased ability of invasion and proliferation in the melanoma cells. Collectively, silencing of CERS6 induced the increased expression of GLUT1, which downregulated the expression of WNT5A and enhanced the invasion and proliferation of melanoma cells. Thus, CERS6 may provide a novel therapeutic target for melanoma treatment.
  
  
  
  
**CERS6**
-
5
of
5
  

### Histone deacetylase inhibitors interact with melanoma differentiation associated-7/interleukin-24 to kill primary human glioblastoma cells.

5. Mol Pharmacol. 2013 Aug;84(2):171-81. doi: 10.1124/mol.113.086553 Epub 2013 May 9.
  
  
Hamed HA(1), Yacoub A, Park MA, Archer K, Das SK, Sarkar D, Grant S, Fisher PB, Dent P.
  
  
Author information: (1)Department of Neurosurgery, Virginia Commonwealth University, Richmond, VA, U ...
  
  
We presently demonstrate that histone deacetylase inhibitors (HDACIs) enhance toxicity of melanoma differentiation-associated gene-7/interleukin 24 (mda-7/IL-24) in invasive primary human glioblastoma multiforme (GBM) cells. Additionally, a method is described to augment the efficacy of adenoviral delivery of mda-7/IL-24 in these cells. HDACIs synergized with melanoma differentiation-associated (MDA)-7/IL-24 killing GBM cells. Enhanced lethality correlated with increased autophagy that was dependent on the expression of ceramide synthase 6. HDACIs interacted with MDA-7/IL-24 prolonging generation of reactive oxygen species and Ca(2+). Quenching of reactive oxygen species and Ca(2+) blocked HDACI and MDA-7/IL-24 killing. In vivo MDA-7/IL-24 prolonged the survival of animals carrying orthotopic tumors, and HDACIs enhanced survival further. A serotype 5/3 adenovirus more effectively delivers mda-7/IL-24 to GBM tumors than a serotype 5 virus. Hence, we constructed a serotype 5/3 adenovirus that conditionally replicates in tumor cells expressing MDA-7/IL-24, in which the adenoviral early region 1A (E1A) gene was driven by the cancer-specific promoter progression elevated gene-3 [Ad.5/3 (INGN 241)-PEG-E1A-mda-7; also called Ad.5/3-CTV (cancer terminator virus)]. Ad.5/3-CTV increased the survival of mice carrying GBM tumors to a significantly greater extent than did a nonreplicative virus Ad.5/3-mda-7. Ad.5/3-CTV exhibited no toxicity in the brains of Syrian hamsters. Collectively our data demonstrate that HDACIs enhance MDA-7/IL-24 lethality, and adenoviral delivery of mda-7/IL-24 combined with tumor-specific viral replication is an effective preclinical GBM therapeutic.
  
  
  
  
  

---

  

# CFLAR

**Signature gene in:** CD4+ T cells, CD8+ T cells, Dendritic cells, Tregs
  
**Differentially expressed in:** CD8+ T cells
  
  
**CFLAR**
-
1
of
10
  

### The deubiquitinase Usp27x as a novel regulator of CFLIP(L) protein expression and sensitizer to death-receptor-induced apoptosis.

1. Apoptosis. 2022 Feb;27(1-2):112-132. doi: 10.1007/s10495-021-01706-9 Epub 2022 Jan 19.
  
  
Dold MN(1), Ng X(1), Alber C(1), Gentle IE(1), Häcker G(1)(2), Weber A(3).
  
  
Author information: (1)Faculty of Medicine, Institute of Medical Microbiology and Hygiene, Medical C ...
  
  
Death receptors are transmembrane proteins that can induce the activation of caspase-8 upon ligand binding, initiating apoptosis. Recent work has highlighted the great molecular complexity of death receptor signalling, in particular through ubiquitination/deubiquitination. We have earlier defined the deubiquitinase Ubiquitin-Specific Protease 27x (Usp27x) as an enzyme capable of stabilizing the pro-apoptotic Bcl-2 family member Bim. Here, we report that enhanced expression of Usp27x in human melanoma cells leads to the loss of cellular FLICE-like inhibitory protein (CFLIP) and sensitizes to Tumor necrosis factor receptor 1 (TNF-R1) or Toll-like receptor 3 (TLR3)-induced extrinsic apoptosis through enabling enhanced processing of caspase-8. The loss of cFLIPL upon overexpression of Usp27x was not due to reduced transcription, could be partially counteracted by blocking the ubiquitin proteasome system and was independent of the known cFLIPL destabilizing ubiquitin E3-ligases Itch and DTX1. Instead, Usp27x interacted with the E3-ligase TRIM28 and reduced ubiquitination of TRIM28. Reduction of cFLIPL protein levels by Usp27x-induction depended on TRIM28, which was also required for polyI:C-induced cell death. This work defines Usp27x as a novel regulator of cFLIPL protein expression and a deubiquitinase in fine tuning death receptor signalling pathways to execute apoptosis.
  
  
  
  
**CFLAR**
-
2
of
10
  

### Ethanol extract of asiasari radix preferentially induces apoptosis in G361 human melanoma cells by differential regulation of p53.

2. BMC Complement Altern Med. 2019 Aug 28;19(1):231. doi: 10.1186/s12906-019-2609-2
  
  
Park KH(1), Choi JH(1)(2), Song YS(2), Kim GC(1), Hong JW(3).
  
  
Author information: (1)Department of Anatomy and Cell Biology, School of Dentistry, Yangsan Campus o ...
  
  
BACKGROUND: In Korea and China, asiasari radix (AR) is widely used as a traditional anti-inflammatory and analgesic agent. After its skin-regenerating and hair loss-preventing activities were identified, several types of AR extracts were used for aesthetic purposes. Nevertheless, the effect of ARE on various types of skin cancers was not fully studied yet. METHODS: In this study, we tested the effect of an ethanolic AR extract (ARE) on G361 human melanoma and HaCaT human keratinocyte cell lines. After ARE exposure, cell growth and the expression patterns of proteins and genes were monitored. RESULTS: The ARE-mediated cell growth inhibition was greater in G361 cells than in HaCaT cells due to differences in its cell growth regulation effects. Interestingly, ARE treatment induced caspase-3-mediated apoptosis in G361 cells, but not in HaCaT cells. Furthermore, ARE reduced the expression of p53 and p21 proteins in G361 cells, whereas it induced their expression in HaCaT cells. ARE induced cell death in G361 cells through the reactive oxygen species (ROS)-dependent regulation of p53 and p21 in G361 cells. Microarray analysis showed that ARE regulates Mouse double minute 2 homolog (MDM2) and CASP8 and FADD-like apoptosis regulator (CFLAR) gene expression in G361 and HaCaT cells differently. CONCLUSION: The treatment of ARE preferentially induces apoptosis in melanoma cells by the ROS-dependent differential regulation of p53 level. Therefore, ARE can be used as a new medicinal option for melanoma.
  
  
  
  
**CFLAR**
-
3
of
10
  

### C-FLIP and the NOXA/Mcl-1 axis participate in the synergistic effect of pemetrexed plus cisplatin in human choroidal melanoma cells.

3. PLoS One. 2017 Sep 1;12(9):e0184135. doi: 10.1371/journal.pone.0184135 eCollection 2017.
  
  
Zhao X(1), Kong F(2), Wang L(1), Zhang H(1).
  
  
Author information: (1)Department of Ophthalmology, the Second Hospital of Shandong University, Jina ...
  
  
Choroidal melanoma is the most common primary malignant intraocular tumor, and very few effective therapies are available to treat it. Our study aimed to understand whether pemetrexed plus cisplatin exerts a beneficial synergistic effect in human choroidal melanoma cells and to delineate the underlying molecular mechanism. To accomplish these aims, we treated choroidal melanoma cells with pemetrexed and cisplatin and assessed cell survival with SRB and MTT assays. Proteins were detected using western blotting analysis. NOXA and CHOP were knocked down with siRNA. We found that pemetrexed or cisplatin alone inhibited survival and induced apoptosis in human choroidal melanoma cells. Furthermore, the expression levels of C-FLIP, an anti-apoptotic protein in the extrinsic apoptosis pathway, and Mcl-1, an anti-apoptotic protein in the intrinsic apoptosis pathway, were decreased by pemetrexed or cisplatin respectively, while the expression of a pro-apoptotic protein in the intrinsic apoptosis pathway, NOXA, was up-regulated. Moreover, pemetrexed or cisplatin alone increased the protein expression of the endoplasmic reticulum stress markers IRE1α, Bip and CHOP. Silencing CHOP expression reduced NOXA expression. These findings suggest that the pemetrexed or cisplatin induced intrinsic apoptosis via activation of the ER stress response. Importantly, combining the two compounds more strongly induced apoptosis. Following the cotreatment, CHOP and NOXA expression increased, while C-FLIP and Mcl-1 expression decreased, and these effects were more pronounced than when using either compound alone. This result suggests that pemetrexed and cisplatin synergistically activate ER stress response-induced apoptosis in choroidal melanoma cells. To summarize, the C-FLIP and NOXA/Mcl-1 axis participated in the synergistic effect of pemetrexed plus cisplatin in human choroidal melanoma cells. Intrinsic apoptosis was induced via activation of the ER stress response. Our study provides important mechanistic insights into potential cancer treatment with pemetrexed plus cisplatin and enriches our understanding of human choroidal melanoma.
  
  
  
  
**CFLAR**
-
4
of
10
  

### Suppression of c‑FLIPL promotes JNK activation in malignant melanoma cells.

4. Mol Med Rep. 2016 Mar;13(3):2904-8. doi: 10.3892/mmr.2016.4856 Epub 2016 Feb 3.
  
  
Tian F(1), Hu Y(1), Sun X(1), Lu G(1), Li Y(2), Yang J(2), Tao J(2).
  
  
Author information: (1)Department of Dermatology, Sixth People's Hospital of Zhengzhou, Zhengzhou, H ...
  
  
The up‑regulation of cellular Fas‑associated death domain‑like interleukin‑1β‑converting enzyme (FLICE)‑like inhibitory protein (c‑FLIP) has been reported in various tumor types, and has been previously shown to be associated with the clinicopathological features of melanoma. To assess its potential role in cancer therapy, the present study evaluated the effects of short hairpin (sh)RNAs of different c‑FLIP isoforms on cellular proliferation and c‑Jun N‑terminal kinase (JNK) signaling. Human c‑FLIP shRNA plasmids were constructed and transfected into the A875 melanoma cell line. It was observed that c‑FLIP shRNA exhibited strong inhibitory effects against the levels of phosphorylated‑JNK and inhibited cellular proliferation in A875 cells. Thus, this indicated that c‑FLIP long form shRNA serves a specific inhibitory role in cellular proliferation through inducing the activation of the JNK pathway in A875 cells. The present study provided insight into the development of RNAi based therapies for melanoma.
  
  
  
  
**CFLAR**
-
5
of
10
  

### The overriding of TRAIL resistance by the histone deacetylase inhibitor MS-275 involves c-myc up-regulation in cutaneous, uveal, and mucosal melanoma.

5. Int Immunopharmacol. 2015 Sep;28(1):313-21. doi: 10.1016/j.intimp.2015.06.024 Epub 2015 Jun 27.
  
  
Venza M(1), Visalli M(2), Oteri R(2), Agliano F(2), Morabito S(2), Teti D(3), Venza I(1).
  
  
Author information: (1)Department of Experimental Specialized Medical and Surgical and Odontostomato ...
  
  
Malignant melanoma is a highly aggressive tumor which may occur in the skin, eye, and mucous membranes. The prognosis of melanoma remains poor in spite of therapeutic advances, emphasizing the importance of innovative treatment modalities. Currently, tumor necrosis factor-related apoptosis-inducing ligand (TRAIL) is showing promising clinical responses, however its use is hampered by intrinsic or acquired melanoma resistance to apoptosis. Recently, we showed that the combination of TRAIL with the class I-specific histone deacetylase inhibitor (HDACi) MS-275 was a privileged way to override TRAIL resistance through down-regulation of cellular Fas-associated death domain (FADD)-like interleukin-1beta-converting enzyme-inhibitory protein (C-FLIP). Here, we elucidated the underlying mechanism and provided evidence that a crucial step in the C-FLIP downregulation triggered by MS-275 implies the up-regulation of c-myc, a transcriptional repressor of C-FLIP. Notably, MS-275 caused H3 histone acetylation at the promoter of c-myc and increased its binding to the C-FLIP promoter, that in turn led to reduced C-FLIP gene transcription. Knockdown of c-myc prevented the MS-275-mediated downregulation of C-FLIP and hindered TRAIL-plus MS-275-induced apoptosis. Findings reported here provide additional knowledge tools for a more aware and effective molecular therapy of melanoma.
  
  
  
  
**CFLAR**
-
6
of
10
  

### HDAC Inhibition Overcomes Acute Resistance to MEK Inhibition in BRAF-Mutant Colorectal Cancer by Downregulation of c-FLIPL.

6. Clin Cancer Res. 2015 Jul 15;21(14):3230-3240. doi: 10.1158/1078-0432.CCR-14-2701 Epub 2015 Mar 26.
  
  
Carson R(#)(1), Celtikci B(#)(1), Fenning C(1), Javadi A(1), Crawford N(1), Carbonell LP(1), Lawler ...
  
  
Author information: (1)Centre for Cancer Research and Cell Biology, School of Medicine, Dentistry an ...
  
  
PURPOSE: Activating mutations in the BRAF oncogene are found in 8% to 15% of colorectal cancer patients and have been associated with poor survival. In contrast with BRAF-mutant (MT) melanoma, inhibition of the MAPK pathway is ineffective in the majority of BRAFMT colorectal cancer patients. Therefore, identification of novel therapies for BRAFMT colorectal cancer is urgently needed. EXPERIMENTAL DESIGN: BRAFMT and wild-type (WT) colorectal cancer models were assessed in vitro and in vivo. Small-molecule inhibitors of MEK1/2, MET, and HDAC were used, overexpression and siRNA approaches were applied, and cell death was assessed by flow cytometry, Western blotting, cell viability, and caspase activity assays. RESULTS: Increased c-MET-STAT3 signaling was identified as a novel adaptive resistance mechanism to MEK inhibitors (MEKi) in BRAFMT colorectal cancer models in vitro and in vivo. Moreover, MEKi treatment resulted in acute increases in transcription of the endogenous caspase-8 inhibitor c-FLIPL in BRAFMT cells, but not in BRAFWT cells, and inhibition of STAT3 activity abrogated MEKi-induced c-FLIPL expression. In addition, treatment with c-FLIP-specific siRNA or HDAC inhibitors abrogated MEKi-induced upregulation of c-FLIPL expression and resulted in significant increases in MEKi-induced cell death in BRAFMT colorectal cancer cells. Notably, combined HDAC inhibitor/MEKi treatment resulted in dramatically attenuated tumor growth in BRAFMT xenografts. CONCLUSIONS: Our findings indicate that c-MET/STAT3-dependent upregulation of c-FLIPL expression is an important escape mechanism following MEKi treatment in BRAFMT colorectal cancer. Thus, combinations of MEKi with inhibitors of c-MET or C-FLIP (e.g., HDAC inhibitors) could be potential novel treatment strategies for BRAFMT colorectal cancer.
  
  
  
  
**CFLAR**
-
7
of
10
  

### NF-κB Regulation of C-FLIP Promotes TNFα-Mediated RAF Inhibitor Resistance in Melanoma.

7. J Invest Dermatol. 2015 Jul;135(7):1839-1848. doi: 10.1038/jid.2015.91 Epub 2015 Mar 9.
  
  
Shao Y(1), Le K(2), Cheng H(2), Aplin AE(3).
  
  
Author information: (1)Key Laboratory of Biomedical Information Engineering of the Ministry of Educa ...
  
  
Targeted inhibitors elicit heterogeneous clinical responses in genetically stratified groups of patients. Although most studies focus on tumor intrinsic properties, factors in the tumor microenvironment were recently found to modulate the response to inhibitors. Here, we show that in cutaneous BRAF V600E melanoma, the cytokine tumor necrosis factor-α (TNFα) blocks RAF inhibitor-induced apoptosis via activation of NF-κB. Several NF-κB-dependent factors are upregulated following TNFα and RAF inhibitor treatment. Of these factors, we show that death receptor inhibitor cellular caspase 8 (FLICE)-like inhibitory protein (C-FLIP) is required for TNFα-induced protection against RAF inhibitor. Overexpression of c-FLIP\_S or c-FLIP\_L isoform decreased RAF inhibitor-induced apoptosis in the absence of TNFα. Importantly, targeting NF-κB enhances response to RAF inhibitor in vitro and in vivo. Together, our results show mechanistic evidence for cytokine-mediated resistance to RAF inhibitor and provide a preclinical rationale for the strategy of cotargeting the RAF/MEK/ERK1/2 pathway and the TNFα/NF-κB axis to treat mutant BRAF melanomas.
  
  
  
  
**CFLAR**
-
8
of
10
  

### Gene expression profiling identifies IRF4-associated molecular signatures in hematological malignancies.

8. PLoS One. 2014 Sep 10;9(9):e106788. doi: 10.1371/journal.pone.0106788 eCollection 2014.
  
  
Wang L(1), Yao ZQ(2), Moorman JP(2), Xu Y(3), Ning S(1).
  
  
Author information: (1)Center for Inflammation, Infectious Diseases and Immunity, Quillen College of ...
  
  
The lymphocyte-specific transcription factor Interferon (IFN) Regulatory Factor 4 (IRF4) is implicated in certain types of lymphoid and myeloid malignancies. However, the molecular mechanisms underlying its interactions with these malignancies are largely unknown. In this study, we have first profiled molecular signatures associated with IRF4 expression in associated cancers, by analyzing existing gene expression profiling datasets. Our results show that IRF4 is overexpressed in melanoma, in addition to previously reported contexts including leukemia, myeloma, and lymphoma, and that IRF4 is associated with a unique gene expression pattern in each context. A pool of important genes involved in B-cell development, oncogenesis, cell cycle regulation, and cell death including BATF, LIMD1, CFLAR, PIM2, and CCND2 are common signatures associated with IRF4 in non-Hodgkin B cell lymphomas. We confirmed the correlation of IRF4 with LIMD1 and CFLAR in a panel of cell lines derived from lymphomas. Moreover, we profiled the IRF4 transcriptome in the context of EBV latent infection, and confirmed several genes including IFI27, IFI44, GBP1, and ARHGAP18, as well as CFLAR as novel targets for IRF4. These results provide valuable information for understanding the IRF4 regulatory network, and improve our knowledge of the unique roles of IRF4 in different hematological malignancies.
  
  
  
  
**CFLAR**
-
9
of
10
  

### c-FLIPp43 induces activation of the nuclear factor‑κB signaling pathway in a dose-dependent manner in the A375 melanoma cell line.

9. Mol Med Rep. 2014 Sep;10(3):1438-42. doi: 10.3892/mmr.2014.2364 Epub 2014 Jul 7.
  
  
Wang Y(1), Tu Y(1), Lu J(2), Tao J(1), Li Y(1).
  
  
Author information: (1)Department of Dermatology, Affiliated Union Hospital, Tongji Medical College, ...
  
  
In order to investigate the role of c‑FLIPp43 in the regulation of the nuclear factor (NF)‑κB signaling pathway in melanoma cell lines, a eukaryotic expression vector for c‑FLIPp43 was constructed with the pCMV‑Tag2B plasmid. The monoclonal A375 cells with stable expression of c‑FLIPp43 were obtained by G418 selection and were identified with western blot analysis. The protein level of NF‑κBp65 in the A375 cell line with stable expression of c‑FLIPp43 was examined by western blot analysis. The translocation of NF‑κBp65 was examined using immunofluorescence. The A375 cell lines were transfected with the pCMV‑Tag2B‑cFLIPp43 vector at different doses and the activation of the NF‑κB signaling pathway was examined by the dual‑luciferase reporter assay system. The stable expression of c‑FLIPp43 in the A375 cell lines transfected with the pCMV‑Tag2B‑cFLIPp43 vector increased the protein level of NF‑κBp65 compared with in the A375 cell lines transfected with the empty vector. Transfection of the cells using the pCMV‑Tag2B‑cFLIPp43 vector increased the amount of NF‑κBp65 in the nucleus in a dose‑dependent manner. In conclusion, the transfection of the c‑FLIPp43 expression vector induces the protein expression of NF‑κBp65 and promotes the activation of the NF‑κB signaling pathway in the A375 melanoma cell line.
  
  
  
  
**CFLAR**
-
10
of
10
  

### Class I-specific histone deacetylase inhibitor MS-275 overrides TRAIL-resistance in melanoma cells by downregulating C-FLIP.

10. Int Immunopharmacol. 2014 Aug;21(2):439-46. doi: 10.1016/j.intimp.2014.05.024 Epub 2014 Jun 2.
  
  
Venza I(1), Visalli M(2), Oteri R(2), Teti D(3), Venza M(1).
  
  
Author information: (1)Department of Experimental Specialized Medical and Surgical and Odontostomato ...
  
  
Tumor-necrosis factor-related apoptosis-inducing ligand (TRAIL) has selective killing effect toward malignant cells; however some human melanomas are intrinsically resistant. In this study, we have shown that class I-specific histone deacetylase inhibitor (HDACi) MS-275 can synergize with TRAIL to induce apoptosis in TRAIL-resistant cell lines and to enhance susceptibility of sensitive cells. Conversely, class II-selective HDACi MC1575 has shown no effect on the resistance of melanoma cells and was able exclusively to increase TRAIL-induced cell death in responsive cells. Both the HDACis variably increased DR4, DR5, and procaspase 8 expression, regardless whether cells were TRAIL-sensitive or TRAIL-resistant. However, only MS-275 markedly decreased the expression levels of both the long and short C-FLIP isoforms. RNAi-mediated C-FLIP silencing resulted in caspase 8-dependent apoptosis in survivor cells which was comparable to that observed following MS-275 treatment. Accordingly, enforced expression of ectopic C-FLIP has abolished the cooperative induction of apoptosis by the combination of MS-275 and TRAIL. These data indicate that C-FLIP is a critical regulator of death ligand sensitivity in melanoma. Inhibition of class I HDAC isoenzymes 1, 2 and 3 has resulted to be functionally important for C-FLIP downregulation by MS-275. In contrast, knockdown of class II HDACs has had no effect on C-FLIP expression, thus explaining the dual incapacity of MC1575 to inhibit C-FLIP expression and sensitize cells resistant to TRAIL. The data reported here suggest that MS-275 represents a promising therapeutic approach in combination with TRAIL for treatment of cutaneous and uveal melanoma due to its ability to reduce C-FLIP expression.
  
  
  
  
  

---

  

# CHD3

**Signature gene in:** CD4+ T cells, Stromal cells
  
**Differentially expressed in:** Stromal cells
  
  
**CHD3**
-
1
of
1
  

### Prognostic and Immunotherapeutic Value of Regulatory T Cell Marker Gene Signature in Melanoma.

1. Iran J Allergy Asthma Immunol. 2025 Mar 10;24(2):237-253. doi: 10.18502/ijaai.v24i2.18150
  
  
Liu Y(1), Liu J(2), Jiang K(3), Cheng X(4), Di S(5), Tang J(6), Luo B(7).
  
  
Author information: (1)The First Clinical Medical College, Nanjing Medical University, Nanjing, Chin ...
  
  
Regulatory T cells (Tregs) are central to establishing an immunosuppressive tumor microenvironment (TME), which promotes cancer progression and influences therapeutic outcomes. However, the prognostic significance of Treg-related genes (TRGs) in predicting immunotherapy response in melanoma remains insufficiently characterized. This study seeks to elucidate the role of TRGs in the antitumor immune response of melanoma. The ordinary transcriptome and single-cell RNA sequencing (scRNA-seq) data were obtained from the gene expression omnibus and the cancer genome atlas databases. A multi-tiered quality control process was applied to scRNA-seq data, followed by cell annotation, cell-cell communication, and enrichment analysis to investigate Treg function in the melanoma microenvironment. Weighted gene coexpression network analysis (WGCNA) was employed to identify modules associated with Treg infiltration. Key prognostic genes were identified using univariate Cox regression analysis and integrated into a prognostic model through least absolute shrinkage and selection operator and stepwise regression methods. The analysis revealed a Treg-related gene signature (TRGS) comprising CHD3, FOSB, SEMA4D, PSME1, FYN, PRKACB, and ARID5A. Higher TRGS-based risk scores were significantly associated with worse prognoses, immune cell infiltration, and stromal scores. TRGS was identified as an independent prognostic indicator for melanoma, offering novel insights into the role of Tregs in modulating the TME. This study highlights the potential clinical utility of TRGs in melanoma diagnostics and personalized immunotherapy, providing a robust foundation for future therapeutic strategies.
  
  
  
  
  

---

  

# CPM

**Signature gene in:** MDM
  
**Differentially expressed in:** MDM
  
  
**CPM**
-
1
of
10
  

### Bodman MA(1), Al Aboud AM(2).

1. Melanocytic Nevi.
  
  
In: StatPearls [Internet]. Treasure Island (FL): StatPearls Publishing; 2025 Jan–. 2023 Jul 17.
  
  
Author information: (1)Kent State University CPM (2)King Abdullah Medical City
  
  
Pigmented lesions of the skin are commonly called freckles. They include solar lentigo, congenital nevi, mucosal nevi, and special nevi of the palms and soles. This activity will help to differentiate the various pedal nevi from acral lentiginous melanoma clinically. Plantar melanomas are characteristically late to be diagnosed, have a poorer response to treatment, and have a significantly higher mortality rate when compared to more proximal melanomas.
  
  
  
  
**CPM**
-
2
of
10
  

### Stromal-driven and Amyloid β-dependent induction of neutrophil extracellular traps modulates tumor growth.

2. Nat Commun. 2021 Jan 29;12(1):683. doi: 10.1038/s41467-021-20982-2
  
  
Munir H(1), Jones JO(1)(2), Janowitz T(3)(4)(5), Hoffmann M(6), Euler M(6), Martins CP(1)(7), Welsh ...
  
  
Author information: (1)MRC Cancer Unit, Hutchison/MRC Research Centre, University of Cambridge, Box ...
  
  
Tumors consist of cancer cells and a network of non-cancerous stroma. Cancer-associated fibroblasts (CAF) are known to support tumorigenesis, and are emerging as immune modulators. Neutrophils release histone-bound nuclear DNA and cytotoxic granules as extracellular traps (NET). Here we show that CAFs induce NET formation within the tumor and systemically in the blood and bone marrow. These tumor-induced NETs (t-NETs) are driven by a ROS-mediated pathway dependent on CAF-derived Amyloid β, a peptide implicated in both neurodegenerative and inflammatory disorders. Inhibition of NETosis in murine tumors skews neutrophils to an anti-tumor phenotype, preventing tumor growth; reciprocally, t-NETs enhance CAF activation. Mirroring observations in mice, CAFs are detected juxtaposed to NETs in human melanoma and pancreatic adenocarcinoma, and show elevated amyloid and β-Secretase expression which correlates with poor prognosis. In summary, we report that CAFs drive NETosis to support cancer progression, identifying Amyloid β as the protagonist and potential therapeutic target.
  
  
  
  
**CPM**
-
3
of
10
  

### An overview on recent in vivo biological application of cerium oxide nanoparticles.

3. Asian J Pharm Sci. 2020 Sep;15(5):558-575. doi: 10.1016/j.ajps.2019.10.005 Epub 2019 Nov 27.
  
  
Stephen Inbaraj B(1), Chen BH(1).
  
  
Author information: (1)Department of Food Science, Fu Jen Catholic University, Taipei 242.
  
  
Cerium oxide nanoparticles (CNPs) possess a great potential as therapeutic agents due to their ability to self-regenerate by reversibly switching between two valences +3 and +4. This article reviews recent articles dealing with in vivo studies of CNPs towards Alzheimer's disease, obesity, liver inflammation, cancer, sepsis, amyotrophic lateral sclerosis, acute kidney injury, radiation-induced tissue damage, hepatic ischemia reperfusion injury, retinal diseases and constipation. In vivo anti-cancer studies revealed the effectiveness of CNPs to reduce tumor growth and angiogenesis in melanoma, ovarian, breast and retinoblastoma cancer cell-induced mice, with their conjugation with folic acid, doxorubicin, CPM, or CXC receptor-4 antagonist ligand eliciting higher efficiency. After conjugation with triphenylphosphonium or magnetite nanoparticles, CNPs were shown to combat Alzheimer's disease by reducing amyloid-β, glial fibrillary acidic protein, inflammatory and oxidative stress markers in mice. By improving muscle function and longevity, the citrate/EDTA-stabilized CNPs could ameliorate amyotrophic lateral sclerosis. Also, they could effectively reduce obesity in mice by scavenging ROS and reducing adipogenesis, triglyceride synthesis, GAPDH enzyme activity, leptin and insulin levels. In CCl4-induced rats, stress signaling pathways due to inflammatory cytokines, liver enzymes, oxidative and endoplasmic reticulum messengers could be attenuated by CNPs. Commercial CNPs showed protective effects on rats with hepatic ischemia reperfusion and peritonitis-induced hepatic/cardiac injuries by decreasing oxidative stress and hepatic/cardiac inflammation. The same CNPs could improve kidney function by diminishing renal superoxide, hyperglycemia and tubular damage in peritonitis-induced acute kidney injury in rats. Radiation-induced lung and testicular tissue damage could be alleviated in mice, with the former showing improvement in pulmonary distress and bronchoconstriction and the latter exhibiting restoration in spermatogenesis rate and spermatid/spermatocyte number. Through enhancement of gastrointestinal motility, the CNPs could alleviate constipation in both young and old rats. They could also protect rat from light-induced retinal damage by slowing down neurodegenerative process and microglial activation.
  
  
  
  
**CPM**
-
4
of
10
  

### State of clinical research of radiotherapy/chemoradiotherapy and immune checkpoint inhibitor therapy combinations in solid tumours-a German radiation oncology survey.

4. Eur J Cancer. 2019 Feb;108:50-54. doi: 10.1016/j.ejca.2018.11.026 Epub 2019 Jan 12.
  
  
Käsmann L(1), Eze C(2), Dantes M(2), Roengvoraphoj O(2), Niyazi M(2), Belka C(3), Manapov F(3).
  
  
Author information: (1)Department of Radiation Oncology, University Hospital, LMU Munich, Munich, Ge ...
  
  
Combinations of immune checkpoint inhibitors (ICIs) with radiotherapy and/or chemoradiotherapy are currently under investigation in many cancer types and clinical settings. In this survey, we solicited members of the German Radiation Oncology Society and young DEGRO (working group of DEGRO e.V.) to review the current status of research in this field and underline critical issues such as oncological benefit, treatment toxicity and obstacles in clinical research. The responses represent 14 different departments of radiation oncology at German university hospitals. Respondents of the same department were analysed for congruence. Sixty-one percent of all respondents perform radiotherapy/chemoradiotherapy and ICI therapy combination studies at their institutions and participate in multicentre studies. Combinations were investigated mainly in head and neck tumours (95%), lung cancer (57%), malignant melanoma (48%) and tumours of the upper gastrointestinal tract (9%). Combination of chemoradiotherapy with checkpoint inhibitors was only tested in head and neck cancers (52%), non-small-cell lung cancer (NSCLC) (8.70%) and malignant melanoma (4%). A combination of radiotherapy/chemoradiotherapy with ICIs is assumed to be effective or very effective by >85% of all respondents. The treatment of intracranial metastatic disease by this combination is assumed to be very effective by most respondents (61%). The present survey shows great acceptance of new combined modality treatment paradigm. ICIs with radiotherapy and/or chemoradiotherapy are under investigation at >75% of all participating centres. Head and neck tumours, NSCLC and malignant melanoma are the most frequently tested cancer types.
  
  
  
  
**CPM**
-
5
of
10
  

### Primary Spindle Cell Malignant Melanoma of Esophagus: An Unusual Finding.

5. J Clin Diagn Res. 2016 Feb;10(2):OD03-4. doi: 10.7860/JCDR/2016/16121.7188 Epub 2016 Feb 1.
  
  
Rawandale NA(1), Suryawanshi KH(2).
  
  
Author information: (1)Assistant Professor, Department of Medicine, S.B.H.G.M.C. Dhule, Maharashtra, ...
  
  
Malignant melanoma of esophagus is usually a metastatic tumour rather than a primary tumour. Primary malignant melanoma accounts for less than 0.2% of all esophageal neoplasm. We report a case of primary spindle cell malignant melanoma of esophagus in a 69-year-old male who presented with history of dysphagia since 1 month. Radiological examinations revealed polypoidal growth at lateral aspect of esophagus. Biopsy was reported as grade III squamous cell carcinoma. Video assisted thoracoscopic esophagectomy was performed. Histopathological examination along with immunohistochemistry gave confirmed diagnosis of primary spindle cell malignant melanoma of esophagus. Though a rare entity, due to its aggressive nature and poor prognosis primary malignant melanoma should be one of the differential diagnoses in a patient with polypoidal esophageal mass lesion. Despite radical surgical treatment prognosis is extremely poor.
  
  
  
  
**CPM**
-
6
of
10
  

### Assessing good operating conditions for intraoperative imaging of melanoma sentinel nodes by a portable gamma camera.

6. Phys Med. 2015 Feb;31(1):92-7. doi: 10.1016/j.ejmp.2014.10.079 Epub 2014 Nov 8.
  
  
Riccardi L(1), Gabusi M(2), Bignotto M(3), Gregianin M(4), Vecchiato A(5), Paiusco M(6).
  
  
Author information: (1)Medical Physics Unit, Veneto Institute of Oncology IOV - IRCCS, Padua, Italy. ...
  
  
OBJECTIVE: To provide guidance for reliable identification of low-activity sentinel nodes in the setting of melanoma surgery using a commercial hand-held gamma camera. METHODS: The average uptake of (99m)Tc nanocolloid by sentinel nodes was evaluated in 95 excised nodes using a Sentinella 102(®) (Oncovision, Valencia, Spain) portable gamma camera. The device sensitivity was assessed for different source depths and collimator distances, imaging an 8-mm sphere filled with a known-activity solution of (99m)Tc. Five nuclear medicine physicians were asked to identify the source at different activity levels and positions within the field of view. For each image the number of signal counts inside a circular region of interest (ROI) was measured, while the variability of ROI counts among operators was assessed. The number of counts providing a minimal, near-constant inter-operator variability was determined as a criterion for a consistent identification of the source. Either the minimum activity or the acquisition time needed to collect the appropriate statistics were then calculated. RESULTS: The median SN uptake (0.5%) turned out to be compatible with values reported in the literature. The sensitivity of the compact gamma camera ranged from ∼25 CPM/kBq to ∼1 CPM/kBq. A total of 50 counts in the ROI circumscribing the lymph node-simulating sphere appeared to be a robust criterion for identification of the source. CONCLUSIONS: Ten megabecquerels of injected activity at the time of surgery and one minute of acquisition allows reliable identification of sentinel nodes for collimator-to-source distances up to 10 cm.
  
  
  
  
**CPM**
-
7
of
10
  

### What is a sentinel node? Re-evaluating the 10% rule for sentinel lymph node biopsy in melanoma.

7. J Surg Oncol. 2007 Jun 15;95(8):623-8. doi: 10.1002/jso.20729
  
  
Kroon HM(1), Lowe L, Wong S, Fullen D, Su L, Cimmino V, Chang AE, Johnson T, Sabel MS.
  
  
Author information: (1)Department of Surgery, University of Michigan Comprehensive Cancer Center, An ...
  
  
INTRODUCTION: Many surgeons use the "10% rule" to define whether a lymph node is a sentinel node (SLN) when staging malignant melanoma. However, this increases the number of SLN removed and the time and cost of the procedure. We examined the impact of raising this threshold on the accuracy of the procedure. METHODS: We reviewed the records of 561 patients with melanoma (624 basins) who underwent SLN with technetium Tc99 labeled sulfur colloid using a definition of a SLN as 10% of that of the node with the highest counts per minute (CPM). RESULTS: Of the 624 basins, 154 (25%) were positive for metastases. An average of 1.9 nodes per basin were removed (range 1-6). Metastases were found in the hottest node in 137 cases (89% of positive basins, 97% of basins overall). Increasing the threshold above 10% decreased the number of nodes excised and the costs involved, but incrementally raised the number of false negative cases above baseline (a 4% increase for a "20% rule," 5% for a "30% rule," 6% for a "40% rule," and 7% for a "50% rule"). Taking only the hottest node would raise the false negative rate by 11%. CONCLUSIONS: Although using thresholds higher than 10% for the definition of a SLN will minimize the extent of surgery and decrease the costs associated with the procedure, it will compromise the accuracy of the procedure and is not recommended.
  
  
  
  
**CPM**
-
8
of
10
  

### The Finger iridectomy technique: small incision biopsy of anterior segment tumours.

8. Br J Ophthalmol. 2005 Aug;89(8):946-9. doi: 10.1136/bjo.2004.062653
  
  
Finger PT(1), Latkany P, Kurli M, Iacob C.
  
  
Author information: (1)The New York Eye Cancer Center,115 East 61st Street, New York City, NY 10021, ...
  
  
AIMS: To develop a minimally invasive, maximally effective method to biopsy anterior segment tumours. METHODS: A 25 gauge aspiration cutter (vitrector) was used to biopsy anterior segment tumours. The probe was introduced under sodium hyaluronate 1% and through a 1 mm incision. Aspiration (600 mm Hg) cutting (300 CPM) was performed to obtain specimens for cytology and histopathology. RESULTS: Diagnostic material was obtained in nine of 10 (90%) cases. Diagnoses included iris naevus, iris stroma, malignant melanoma, melanocytoma, epithelial inclusion cyst, and sarcoid granuloma. All corneal wounds were self sealing. One patient developed a transient postoperative increase in intraocular pressure. Within the follow up of this study, no patients suffered intraocular haemorrhage, infection, cataract or vision loss. CONCLUSION: The Finger iridectomy technique was a minimally invasive and very effective biopsy technique. Aspiration cutting yielded relatively large pieces of tissue (and cells) used for cytopathological and histopathological evaluation. Small incision surgery allowed for rapid rehabilitation and no significant complications.
  
  
  
  
**CPM**
-
9
of
10
  

### In vivo studies of pharmacokinetics and efficacy of Bismuth-213 labeled antimelanoma monoclonal antibody 9.2.27.

9. Cancer Biol Ther. 2005 Jul;4(7):763-8. doi: 10.4161/cbt.4.7.1868 Epub 2005 Jul 25.
  
  
Rizvi SM(1), Qu CF, Song YJ, Raja C, Allen BJ.
  
  
Author information: (1)Center for Experimental Radiation Oncology, Cancer Care Center, St. George Ho ...
  
  
OBJECTIVES: Key objectives of the study were to determine the pharmacokinetics and efficacy of the alpha emitting Bismuth-213 labeled 9.2.27 alpha-immunoconjugate (AIC). METHODS: Balb/c nude mice were injected with varying doses of AIC to determine the pharmacokinetics of the AIC. The results were normalized to percent counts per minute (CPM) per gram per 3.7 MBq of the AIC (%CPM/g/3.7MBq) for each organ. Efficacy was determined by injected varying doses of AIC to different stages of tumor growth for intra-lesional, systemic and multiple dose TAT. RESULTS: Biodistribution studies showed similar pharmacokinetics for blood and brain, liver, kidneys, spleen, gut, heart, lungs and bone marrow, indicating that there was no retention of AIC. This is particularly important for brain (due to the presence of NG2+ cells) as the antibody 9.2.27 may reach NG2 positive cells. Tumor growth at 2-days post-inoculation was completely inhibited by TAT. The response to TAT was inversely proportional to tumor growth, i.e., a reduction in response was observed with increased tumor burden. A multiple dose regime was found to be more effective than single dose. CONCLUSIONS: TAT is effective for the treatment of micrometastatic melanoma, when the tumor is preangiogenic in the form of isolated cells or cell clusters. There is no evidence of retention of AIC in brain, kidneys and other vital organs.
  
  
  
  
**CPM**
-
10
of
10
  

### Biological activity and binding of estradiol to SK-Mel 23 human melanoma cells.

10. Braz J Med Biol Res. 2004 Jun;37(6):901-5. doi: 10.1590/s0100-879x2004000600016 Epub 2004 May 27.
  
  
Sarti MS(1), Visconti MA, Castrucci AM.
  
  
Author information: (1)Departamento de Fisiologia, Instituto de Biociências, Universidade de São Pau ...
  
  
Patients expressing estradiol receptors in melanoma cells have been reported to have a better prognosis. We therefore decided to investigate the in vitro effects of beta-estradiol and tamoxifen on the growth and tyrosinase activity of SK-Mel 23 human melanoma cells. Twenty-four-hour treatment with 0.4 nM beta-estradiol inhibited cell proliferation in 30% (0.70 +/- 0.03 x 10(5) cells) and increased tyrosinase activity in 50% (7130.5 +/- 376.5 CPM/10(5) cells), as compared to untreated cells (1.0 +/- 0.05 x 10(5) cells and 4769 +/- 25.5 CPM/10(5) cells, respectively). Both responses were completely (100%) blocked by 1 microM tamoxifen. Higher concentrations (up to 1.6 nM) or longer treatments (up to 72 h) did not result in a larger effect of the hormone on proliferation or tyrosinase activity. Competition binding assays demonstrated the presence of binding sites to [2,4,6,7-3H]-beta-estradiol, and that the tritiated analogue was displaced by the unlabeled hormone (1 nM to 100 microM, Kd = 0.14 microM, maximal displacement of 93%) or by 10 microM tamoxifen (displacement of 60%). Beta-estradiol also increased the phosphorylated state of two proteins of 16 and 46 kDa, after 4-h treatment, as determined by Western blot. The absorbance of each band was 1.9- and 4-fold the controls, respectively, as determined with Image-Pro Plus software. Shorter incubation periods with beta-estradiol did not enhance phosphorylation; after 6-h treatment with the hormone, the two proteins returned to the control phosphorylation levels. The growth inhibition promoted by estradiol may explain the better prognosis of melanoma-bearing women as compared to men, and open new perspectives for drug therapy.
  
  
  
  
  

---

  

# ELL2

**Signature gene in:** Stromal cells
  
**Differentially expressed in:** Stromal cells
  
  
**ELL2**
-
1
of
1
  

### FOXD1 Is a Transcription Factor Important for Uveal Melanocyte Development and Associated with High-Risk Uveal Melanoma.

1. Cancers (Basel). 2022 Jul 28;14(15):3668. doi: 10.3390/cancers14153668
  
  
van den Bosch QCC(1)(2), Nguyen JQN(1)(2), Brands T(1)(2), van den Bosch TPP(3), Verdijk RM(3)(4), P ...
  
  
Author information: (1)Department of Ophthalmology, Erasmus MC Cancer Center, Erasmus MC University ...
  
  
Uveal melanoma (UM) is a deadly ocular malignancy, originating from uveal melanocytes. Although much is known regarding prognostication in UM, the exact mechanism of metastasis is mostly unknown. Metastatic tumor cells are known to express a more stem-like RNA profile which is seen often in cell-specific embryonic development to induce tumor progression. Here, we identified novel transcription regulators by reanalyzing publicly available single cell RNA sequencing experiments. We identified five transcription regulators of interest: ELL2, KDM5B, REXO4, RBFOX2 and FOXD1. Our most significant finding is FOXD1, as this gene is nearly exclusively expressed in high-risk UM and its expression is associated with a poor prognosis. Even within the BAP1-mutated UM, the expression of FOXD1 is correlated with poor survival. FOXD1 is a novel factor which could potentially be involved in the metastatic capacity of high-risk UM. Elucidating the function of FOXD1 in UM could provide insight into the malignant transformation of uveal melanocytes, especially in high-risk UM.
  
  
  
  
  

---

  

# FKBP5

**Signature gene in:** CD4+ T cells, CD8+ T cells, MDM, Monocytes, Stromal cells, Tregs, Tumor cells
  
**Differentially expressed in:** CD4+ T cells, CD8+ T cells, MDM, Monocytes, Stromal cells, Tregs
  
  
**FKBP5**
-
1
of
10
  

### FKBP51 plays an essential role in Akt ubiquitination that requires Hsp90 and PHLPP.

1. Cell Death Dis. 2023 Feb 13;14(2):116. doi: 10.1038/s41419-023-05629-y
  
  
Tufano M(#)(1), Marrone L(#)(1), D'Ambrosio C(2), Di Giacomo V(1), Urzini S(1), Xiao Y(3), Matuozzo ...
  
  
Author information: (1)Department of Molecular Medicine and Medical Biotechnology, University of Nap ...
  
  
FKBP51 plays a relevant role in sustaining cancer cells, particularly melanoma. This cochaperone participates in several signaling pathways. FKBP51 forms a complex with Akt and PHLPP, which is reported to dephosphorylate Akt. Given the recent discovery of a spliced FKBP51 isoform, in this paper, we interrogate the canonical and spliced isoforms in regulation of Akt activation. We show that the TPR domain of FKBP51 mediates Akt ubiquitination at K63, which is an essential step for Akt activation. The spliced FKBP51, lacking such domain, cannot link K63-Ub residues to Akt. Unexpectedly, PHLPP silencing does not foster phosphorylation of Akt, and its overexpression even induces phosphorylation of Akt. PHLPP stabilizes levels of E3-ubiquitin ligase TRAF6 and supports K63-ubiquitination of Akt. The interactome profile of FKBP51 from melanoma cells highlights a relevant role for PHLPP in improving oncogenic hallmarks, particularly, cell proliferation.
  
  
  
  
**FKBP5**
-
2
of
10
  

### Alternative splicing of FKBP5 gene exerts control over T lymphocyte expansion.

2. J Cell Biochem. 2024 Dec;125(12):e30364. doi: 10.1002/jcb.30364 Epub 2023 Jan 16.
  
  
Marrone L(1), D'Agostino M(1), Cesaro E(1), di Giacomo V(1), Urzini S(1), Romano MF(1), Romano S(1).
  
  
Author information: (1)Department of Molecular Medicine and Medical Biotechnology, University of Nap ...
  
  
FKBP51 is constitutively expressed by immune cells. As other FKBP family members, FKBP51 acts as a coreceptor for the natural products FK506 and rapamycin, which exhibit immunosuppressive effects. However, little is known about the intrinsic role of this large FKBP in the primary function of lymphocytes, that is, the adaptive immune response against foreign antigens, for example, pathogens. This paper aimed to investigate whether FKBP51 expression was modulated during lymphocyte activation. Moreover, as we recently identified a splicing isoform of FKBP51, namely FKBP51s, we also measured this splice protein, along with the canonical one, at different times of a peripheral blood mononuclear cell culture stimulated via T cell receptor. Our results show that the two FKBP51 isoforms were alternatively induced during the proliferative burst. Canonical FKBP51 increased in the time window between 48 and 96 h and its expression levels correlated with cyclin D levels. FKBP51s transiently increased earlier, at 24-36 h to reappearing later, at 120 h, when cyclin D expression returned at resting levels and proliferation ceased. Interestingly, within these two specific timeframes, FKBP51s accumulated in the nucleus. Here FKBP51s colocalized with the Foxp3 transcription factor at 36 h. Regulatory T cell (Treg) counts significantly decreased when FKBP51s was downmodulated. The coculture suppression assay suggested that FKBP51s supports the suppressive capability of Tregs. At 120 h, chromatin immunoprecipitation experiments found FKBP51s linked to CCND1 gene, suggesting a possible effect on gene transcription regulation, as previously demonstrated in melanoma. In conclusion, our study shows that FKBP5 isoforms are upregulated during lymphocyte activation, albeit on different timeframes. The activation of canonical FKBP51 coincides with proliferation hallmarks; FKBP5 splicing occurs early to sustain Treg development and late when proliferation ceases.
  
  
  
  
**FKBP5**
-
3
of
10
  

### Alternative macrophage polarisation associated with resistance to anti-PD1 blockade is possibly supported by the splicing of FKBP51 immunophilin in melanoma patients.

3. Br J Cancer. 2020 Jun;122(12):1782-1790. doi: 10.1038/s41416-020-0840-8 Epub 2020 Apr 22.
  
  
Troiani T(1), Giunta EF(1), Tufano M(2), Vigorito V(2), Arrigo P(2), Argenziano G(3), Ciardiello F(1 ...
  
  
Author information: (1)Department of Precision Medicine, Oncology Unit, University of Campania Luigi ...
  
  
BACKGROUND: FKBP51 immunophilin is abundantly expressed by immune cells. Co-inhibitory immune receptor signalling generates the splicing isoform FKBP51s. Tregs stained by FKBP51s are increased in melanoma patients and their counts are associated with anti-CTLA-4 response. An expansion of FKBP51s+PD-L1+ monocytes was measured in a group of non-responding patients to anti-CTLA-4. The aim of this work was to confirm the predictive value of response of FKBP51s+Tregs in a cohort of patients undergoing anti-PD1 treatment and shed light on a monocyte subset co-expressing PD-L1/FKBP51s. METHODS: Co-cultures of organoids and autologous lymphocytes were used to confirm that tumour T-cell interaction can induce FKBP51s. PBMC immunophenotype and flow cytometry served to assess and monitor FKBP51s+Treg and FKBP51s+PD-L1+ monocytes in 22 advanced melanoma patients treated with anti-PD1. Silencing and overexpression of FKBP51s in human macrophages served to address the protein role in the tolerant macrophages' behaviour. RESULTS: FKBP51s+Tregs count was increased in responders and had a prognostic value. Non-responders showed an early increase in FKBP51s+ PD-L1+ monocytes during anti-PD1 treatment. Manipulation of FKBP51s modulated the macrophage-phenotype, with forced protein expression promoting aspects associated with tolerance. CONCLUSIONS: FKBP51s may guide in the selection and monitoring of melanoma patient candidates to immune-checkpoint-targeted therapy. Manipulation of FKBP51s may overcome resistance.
  
  
  
  
**FKBP5**
-
4
of
10
  

### Force-induced gene up-regulation does not follow the weak power law but depends on H3K9 demethylation.

4. Sci Adv. 2020 Apr 1;6(14):eaay9095. doi: 10.1126/sciadv.aay9095 eCollection 2020 Apr.
  
  
Sun J(1), Chen J(1)(2), Mohagheghian E(1), Wang N(1).
  
  
Author information: (1)Department of Mechanical Science and Engineering, The Grainger College of Eng ...
  
  
Mechanical forces play important roles in development, physiology, and diseases, but how force is transduced into gene transcription remains elusive. Here, we show that transcription of transgene DHFR or endogenous genes egr-1 and Cav1 is rapidly up-regulated in response to cyclic forces applied via integrins at low frequencies but not at 100 Hz. Gene up-regulation does not follow the weak power law with force frequency. Force-induced transcription up-regulation at the nuclear interior is associated with demethylation of histone H3 lysine-9 trimethylation (H3K9me3), whereas no transcription up-regulation near the nuclear periphery is associated with H3K9me3 that inhibits Pol II recruitment to the promoter site. H3K9me3 demethylation induces Pol II recruitment and increases force-induced transcription of egr-1 and Cav1 at the nuclear interior and activates mechano-nonresponsive gene FKBP5 near the nuclear periphery, whereas H3K9me3 hypermethylation has opposite effects. Our findings demonstrate that rapid up-regulation of endogenous mechanoresponsive genes depends on H3K9me3 demethylation.
  
  
  
  
**FKBP5**
-
5
of
10
  

### TRAF2 and FKBP51 as possible markers for identification of suitable melanoma tumors for tumor necrosis factor-α inhibition.

5. Melanoma Res. 2019 Apr;29(2):145-150. doi: 10.1097/CMR.0000000000000553
  
  
Romano S(1), D'Arrigo P(1), Tufano M(1), Staibano S(2), Rea A(1), Merolla F(3), Ilardi G(2), Petrell ...
  
  
Author information: (1)Department of Molecular Medicine and Medical Biotechnologies. (2)Department o ...
  
  
Tumor necrosis factor-α (TNF-α) is a pleiotropic cytokine, whose role in melanoma is controversial. Although high-dose TNF-α is approved for the treatment of patients with in transit-metastatic melanoma confined to the limb, diverse preclinical models of melanoma have shown that TNF-α can induce cell invasion. Biomarkers that can differentiate between the dual role of TNF-α are needed. TRAF2 is critical to TNF receptor-induced activation of nuclear factor-κB (NF-κB), allowing shifting from death to survival-signaling cascades. The large immunophilin FKBP51 acts as a scaffold and catalyst in the IκB kinase complex assembly and activation. Here, using microscopy and an electrophoretic mobility-shift assay, we provide further evidence in support of the essential role of FKBP51 in sustaining the TNF-α NF-κB signaling in melanoma. Through the cross-linking reaction with the chemical linker disuccinimidyl glutarate, we show that a direct interaction occurs between FKBP51 and TRAF2 in melanoma cells. Immunohistochemistry of tumor samples from 24 patients with cutaneous melanomas showed a correlation between the expressions of the two proteins. Given the association of FKBP51 and TRAF2 with TNF-α-induced NF-κB signaling and their correlation in tumor samples, we propose that the two proteins can be exploited as useful markers for the identification of those melanoma tumors that can benefit from TNF-α inhibition. Future studies will address this hypothesis.
  
  
  
  
**FKBP5**
-
6
of
10
  

### FKBP51s signature in peripheral blood mononuclear cells of melanoma patients as a possible predictive factor for immunotherapy.

6. Cancer Immunol Immunother. 2017 Sep;66(9):1143-1151. doi: 10.1007/s00262-017-2004-0 Epub 2017 Apr 22.
  
  
Romano S(1), Simeone E(2), D'Angelillo A(1), D'Arrigo P(1), Russo M(1), Capasso M(1)(3), Lasorsa VA( ...
  
  
Author information: (1)Department of Molecular Medicine and Medical Biotechnologies, Federico II Uni ...
  
  
The inhibitory immune checkpoint PD-L1/PD1 promotes the alternative splicing of the FKBP5 gene, resulting in increased expression of its variant 4 in the peripheral blood mononuclear cells of melanoma patients. The variant 4 transcript is translated into the truncated FKBP51s protein. Given the importance of co-inhibitory signalling in tumour immune escape, here we tested the potential for using FKBP51s expression to predict immunotherapy outcomes. To do this, we immunophenotyped PBMCs from 118 melanoma patients and 77 age- and sex-matched healthy controls. Blood samples were collected before patients underwent ipilimumab treatment. In 64 of the 118 patients, FKBP51s expression was also assessed in regulatory T cells (Tregs). We found that each PBMC subset analysed contained an FKBP51spos fraction, and that this fraction was greater in the melanoma patients than healthy controls. In CD4 T lymphocytes, the FKBP51sneg fraction was significantly impaired. Tregs count was increased in melanoma patients, which is in line with previous studies. Also, by analyses of FKBP51s in Tregs, we identified a subgroup of ipilimumab nonresponder patients (p = 0.002). In conclusion, FKBP51s-based immunophenotyping of melanoma patients revealed several profiles related to a negative immune regulatory control and identified an unknown Treg subset. These findings are likely to be useful in the selection of the patients that are candidate for immunotherapy.
  
  
  
  
**FKBP5**
-
7
of
10
  

### FKBP51 Immunohistochemical Expression: A New Prognostic Biomarker for OSCC?

7. Int J Mol Sci. 2017 Feb 18;18(2):443. doi: 10.3390/ijms18020443
  
  
Russo D(1), Merolla F(2), Mascolo M(3), Ilardi G(4), Romano S(5), Varricchio S(6), Napolitano V(7), ...
  
  
Author information: (1)Department of Advanced Biomedical Sciences, University "Federico II", 80131 N ...
  
  
Up-to-date, several molecular markers of prognosis have been studied in Oral Squamous Cell Carcinoma (OSCC), but none entered in the clinical setting. Therapy of OSCC tumors mainly relies on surgery, radiotherapy and partially on chemotherapy; there is an urgent need for biomarkers able to better stratify OSCC patients' risk to address targeted therapeutic strategies. The role of immune response in the pathogenesis and biological behavior of OSCC has been investigated by several authors, and promising results have been obtained with immune checkpoint inhibitors. We already investigated the role of the immune modulator FK506-binding protein 51 (FKBP51), a FK506-binding immunophilin, in cutaneous melanoma biology, and its expression in several human solid tumors. In the present study, we aimed to assess the value of FKBP51 expression in OSCC tumor cells as a marker of outcome. We collected clinical data from 72 patients who underwent surgery for Squamous Cell Carcinoma (SCC) of the tongue, floor, lips and palate. FKBP51 expression was assessed by immunohistochemistry on paraffin-embedded tumor tissues. In addition, we evaluated the human papillomavirus (HPV) status of primary tumors by immunohistochemistry, viral subtyping and In Situ Hybridization (ISH) assay. We found that high FKBP51-expressing tumors characterized the OSCCs with the worst prognosis: the high immunohistochemical expression of FKBP51 associated with death occurring within five years from the diagnosis with a sensitivity of 88.46% and a specificity of 91.67%. The estimated positive predictive value of the test was 88.45% and negative predictive value 91.67%. We tested FKBP51 mRNA presence, by RT-PCR assay, in a selected series of OSCC tumors, and we found that mRNA correlated well to the protein expression and to the clinical outcome. Applying the Bayes formula, we estimated an 88% probability of dying within five years from the diagnosis of OSCC patients with a high FKBP51 immunohistochemical (IHC) test result (>51% of FKBP51 positive tumor cells). On the basis of our analysis, we propose tumor tissue expression of FKBP51 protein as a reliable prognostic marker for OSCC tumors.
  
  
  
  
**FKBP5**
-
8
of
10
  

### FKBP51 regulates cell motility and invasion via RhoA signaling.

8. Cancer Sci. 2017 Mar;108(3):380-389. doi: 10.1111/cas.13153
  
  
Takaoka M(1), Ito S(1), Miki Y(1)(2), Nakanishi A(1).
  
  
Author information: (1)Department of Molecular Genetics, Medical Research Institute, Tokyo Medical a ...
  
  
FK506 binding protein 51 (FKBP51), a member of the immunophilin family, is involved in multiple signaling pathways, tumorigenesis, and chemoresistance. FKBP51 expression correlates with metastatic potential in melanoma and prostate cancer. However, the functions of FKBP51, particularly involving the regulation of cell motility and invasion, are not fully understood. We discovered two novel interacting partner proteins of FKBP51, i.e., deleted in liver cancer 1 (DLC1) and deleted in liver cancer 2 (DLC2), using immunoprecipitation and mass spectrometry. DLC1 and DLC2 are Rho GTPase-activating proteins that are frequently downregulated in various cancers. Next, we demonstrated that overexpression of FKBP51 enhances cell motility and invasion of U2OS cells via upregulation of RhoA activity and enhanced Rho-ROCK signaling. Moreover, FKBP51-depleted cells displayed a cortical distribution of actin filaments and decreased cell motility and invasion. Consistent with this phenotype, FKBP51 depletion caused a downregulation of RhoA activity. Considered together, our results demonstrate that FKBP51 positively controls cell motility by promoting RhoA and ROCK activation; thus, we have revealed a novel role for FKBP51 in cytoskeletal rearrangement and cell migration and invasion.
  
  
  
  
**FKBP5**
-
9
of
10
  

### FKBP51 employs both scaffold and isomerase functions to promote NF-κB activation in melanoma.

9. Nucleic Acids Res. 2015 Aug 18;43(14):6983-93. doi: 10.1093/nar/gkv615 Epub 2015 Jun 22.
  
  
Romano S(1), Xiao Y(2), Nakaya M(3), D'Angelillo A(4), Chang M(3), Jin J(3), Hausch F(5), Masullo M( ...
  
  
Author information: (1)Department of Molecular Medicine and Medical Biotechnologies, Federico II Uni ...
  
  
Melanoma is the most aggressive skin cancer; its prognosis, particularly in advanced stages, is disappointing largely due to the resistance to conventional anticancer treatments and high metastatic potential. NF-κB constitutive activation is a major factor for the apoptosis resistance of melanoma. Several studies suggest a role for the immunophilin FKBP51 in NF-κB activation, but the underlying mechanism is still unknown. In the present study, we demonstrate that FKBP51 physically interacts with IKK subunits, and facilitates IKK complex assembly. FKBP51-knockdown inhibits the binding of IKKγ to the IKK catalytic subunits, IKK-α and -β, and attenuates the IKK catalytic activity. Using FK506, an inhibitor of the FKBP51 isomerase activity, we found that the IKK-regulatory role of FKBP51 involves both its scaffold function and its isomerase activity. Moreover, FKBP51 also interacts with TRAF2, an upstream mediator of IKK activation. Interestingly, both FKBP51 TPR and PPIase domains are required for its interaction with TRAF2 and IKKγ, whereas only the TPR domain is involved in interactions with IKKα and β. Collectively, these results suggest that FKBP51 promotes NF-κB activation by serving as an IKK scaffold as well as an isomerase. Our findings have profound implications for designing novel melanoma therapies based on modulation of FKBP51.
  
  
  
  
**FKBP5**
-
10
of
10
  

### Molecular Aspects of FKBP51 that Enable Melanoma Dissemination.

10. Curr Mol Pharmacol. 2015;9(2):141-7. doi: 10.2174/1874467208666150519115242
  
  
D'Angelillo A, Staibano S, Russo M, Romano MF(1), Romano S.
  
  
Author information: (1)Department of Molecular Medicine and Medical Biotechnologies, Federico II Uni ...
  
  
FKBP51 (FKBP5 Official Symbol) is large molecular weight member of the FK506 binding protein family, a subfamily of the immunophilin proteins. FKBP51 exerts multiple biological functions in the cell, including modulation of steroid hormone response, immune regulation, cell proliferation, regulation of pAkt levels and control of NF-κB activation. Several lines of evidence support a role for this protein in cancer biology, especially in resistance to chemo- and radio-therapy. Recent research studies highlighted functions of FKBP51 in promoting the epithelial to mesenchymal transition (EMT) transdifferentiation program in melanoma. This process, which is classically regulated by Transforming Growth Factor (TGF)-β, enables cancer cells to disseminate from primary tumors and spread to distant locations, acquiring resistance to therapy and self-renewal capability. This last, in turn, is crucial to their subsequent expansion at sites of dissemination. The aim of the present article is to review recent literature data that involve FKBP51 in the mechanisms that switch the TGF-β from a tumor suppressor to a pro-metastatic invader.
  
  
  
  
  

---

  

# FN1

**Signature gene in:** Stromal cells
  
**Differentially expressed in:** Stromal cells
  
  
**FN1**
-
1
of
10
  

### Unveiling a novel cancer hallmark by evaluation of neural infiltration in cancer.

1. Brief Bioinform. 2025 Mar 4;26(2):bbaf082. doi: 10.1093/bib/bbaf082
  
  
Dong Q(1)(2), Guo Y(1)(3), Lv C(1), Ren L(3), Chen B(1), Wang Y(3), Liu Y(3), Liu M(1), Liu K(1), Zh ...
  
  
Author information: (1)Department of Systems Biology, College of Bioinformatics Science and Technolo ...
  
  
Cancer cells acquire necessary functional capabilities for malignancy through the influence of the nervous system. We evaluate the extent of neural infiltration within the tumor microenvironment (TME) across multiple cancer types, highlighting its role as a cancer hallmark. We identify cancer-related neural genes using 40 bulk RNA-seq datasets across 10 cancer types, developing a predictive score for cancer-related neural infiltration (C-Neural score). Cancer samples with elevated C-Neural scores exhibit perineural invasion, recurrence, metastasis, higher stage or grade, or poor prognosis. Epithelial cells show the highest C-Neural scores among all cell types in 55 single-cell RNA sequencing datasets. The epithelial cells with high C-Neural scores (epi-highCNs) characterized by increased copy number variation, reduced cell differentiation, higher epithelial-mesenchymal transition scores, and elevated metabolic level. Epi-highCNs frequently communicate with Schwann cells by FN1 signaling pathway. The co-culture experiment indicates that Schwann cells may facilitate cancer progression through upregulation of VDAC1. Moreover, C-Neural scores positively correlate with the infiltration of antitumor immune cells, indicating potential response for immunotherapy. Melanoma patients with high C-Neural scores may benefit from trametinib. These analyses illuminate the extent of neural influence within TME, suggesting potential role as a cancer hallmark and offering implications for effective therapeutic strategies against cancer.
  
  
  
  
**FN1**
-
2
of
10
  

### Hypomethylation of CD3D promoter induces immune cell infiltration and supports malignant phenotypes in uveal melanoma.

2. FASEB J. 2023 Oct;37(10):e23128. doi: 10.1096/fj.202300505RR
  
  
Zhang C(1), Wu S(2).
  
  
Author information: (1)Department of Strabismus and Pediatric Ophthalmology, the Second Hospital of ...
  
  
Alterations in DNA methylation in malignant diseases have been heralded as promising targets for diagnostic, prognostic, and predictive values. This study was based on epigenetic alterations and immune cell infiltration analysis to investigate the mechanism of CD3D methylation in uveal melanoma (UM). Bioinformatics analysis was performed on transcriptome data, 450 K methylation data, and clinical information of UM patients from the TCGA database. Stromal and immune cell infiltration was evaluated by calculating the StromalScore and ImmuneScore of UM samples. UM samples were divided into high and low StromalScore and ImmuneScore groups, followed by differential and enrichment analyses. PPI network construction and correlation analysis was used to identify the core prognosis-related genes. The bioinformatics analysis results were confirmed in UM cell experiments. StromalScore and ImmuneScore were significantly associated with the prognosis of UM patients. CD3D, IRF1, CCL3, and FN1 were identified as core genes driven by methylation that affected the prognosis of UM patients. CD3D expression showed the highest correlation with its methylation and was closely related to the four key immune cells in UM development. CD3D was hypomethylated and abundantly expressed in UM cells, while silencing of CD3D inhibited the proliferation, migration, and invasion of UM cells in vitro. In summary, this study identifies hypomethylation of CD3D promoter in UM, which was associated with immune cell infiltration of UM.
  
  
  
  
**FN1**
-
3
of
10
  

### A comprehensive identification of potential molecular targets and small drugs candidate for melanoma cancer using bioinformatics and network-based screening approach.

3. J Biomol Struct Dyn. 2024 Sep;42(14):7349-7369. doi: 10.1080/07391102.2023.2240409 Epub 2023 Aug 3.
  
  
Khan DA(1), Adhikary T(2), Sultana MT(2), Toukir IA(3).
  
  
Author information: (1)Department of Genetic Engineering and Biotechnology, Jashore University of Sc ...
  
  
Melanoma is the third most common malignant skin tumor and has increased in morbidity and mortality over the previous decade due to its rapid spread into the bloodstream or lymphatic system. This study used integrated bioinformatics and network-based methodologies to reliably identify molecular targets and small molecular medicines that may be more successful for Melanoma diagnosis, prognosis and treatment. The statistical LIMMA approach utilized for bioinformatics analysis in this study found 246 common differentially expressed genes (cDEGs) between case and control samples from two microarray gene-expression datasets (GSE130244 and GSE15605). Protein-protein interaction network study revealed 15 cDEGs (PTK2, STAT1, PNO1, CXCR4, WASL, FN1, RUNX2, SOCS3, ITGA4, GNG2, CDK6, BRAF, AGO2, GTF2H1 and AR) to be critical in the development of melanoma (KGs). According to regulatory network analysis, the most important transcriptional and post-transcriptional regulators of DEGs and hub-DEGs are ten transcription factors and three miRNAs. We discovered the pathogenetic mechanisms of MC by studying DEGs' biological processes, molecular function, cellular components and KEGG pathways. We used molecular docking and dynamics modeling to select the four most expressed genes responsible for melanoma malignancy to identify therapeutic candidates. Then, utilizing the Connectivity Map (CMap) database, we analyzed the top 4-hub-DEGs-guided repurposable drugs. We validated four melanoma cancer drugs (Fisetin, Epicatechin Gallate, 1237586-97-8 and PF 431396) using molecular dynamics simulation with their target proteins. As a result, the results of this study may provide resources to researchers and medical professionals for the wet-lab validation of MC diagnosis, prognosis and treatments.Communicated by Ramaswamy H. Sarma.
  
  
  
  
**FN1**
-
4
of
10
  

### Optimization of topical curcumin spanlastics for melanoma treatment.

4. Pharm Dev Technol. 2023 Jun;28(5):425-439. doi: 10.1080/10837450.2023.2204926 Epub 2023 May 9.
  
  
Ismail S(1), Garhy D(1), Ibrahim HK(2).
  
  
Author information: (1)Department of Pharmaceutics, Egyptian Drug Authority (EDA) Formerly Known as ...
  
  
The aim of the study was to enhance curcumin skin permeability through the preparation of spanlastics. Spanlastics were prepared using the ethanol injection technique through a central composite design using Span 60 concentration (X1), edge activator type (X2), and its concentration (X3) as the independent variables. The spanlastics were characterized for particle size (PS), encapsulation efficiency (EE), and dissolution efficiency (%DE24h). Formulae with the highest desirability (FN1 and FN2) were prepared and further characterized. They were elastic, spherical, nonirritant, and compatible with the used excipients. They had particle sizes of 147 and 198 nm, encapsulation efficiencies of 84.00% and 89.63%, zeta potential values of -45.50 and -39.10 mV, permeation enhancement ratios of 11.51 and 8.34 folds, and amounts retained of 7.25 and 10.44 µg/cm2 after 24 h, respectively. Formulae FN1 and FN2 showed cytotoxic effects after 48 h on human melanoma A375 with IC50 of 10.9 and 75.6 μg/mL, respectively. They increased the apoptotic effect confirming the success of the spanlastics to be a potential delivery for melanoma treatment.
  
  
  
  
**FN1**
-
5
of
10
  

### New panel of biomarkers to discriminate between amelanotic and melanotic metastatic melanoma.

5. Front Oncol. 2023 Jan 26;12:1061832. doi: 10.3389/fonc.2022.1061832 eCollection 2022.
  
  
Militaru IV(1), Rus AA(1), Munteanu CVA(2), Manica G(1), Petrescu SM(1).
  
  
Author information: (1)Department of Molecular Cell Biology, Institute of Biochemistry, Bucharest, R ...
  
  
Melanoma is a form of skin cancer that can rapidly invade distant organs. A distinctive feature of melanomas is their pigmentation status, as melanin is present in most skin melanomas, whilst many metastatic tumors could become amelanotic. Besides the obvious malfunction of the key genes of the melanin pathway, the amelanotic tumors could bear a characteristic molecular signature accounting for their aggressivity. Using mass spectrometry-based proteomics we report here a distinctive panel of biomarkers for amelanotic aggressive melanoma that differ from the less invasive pigmented cells. The developed method allows the label-free quantification of proteins identified by LC-MS/MS analysis. We found a set of proteins comprising AHNAK, MYOF, ANXA1, CAPN2, ASPH, EPHA2, THBS1, TGM2, ACTN4 along with proteins involved in cell adhesion/migration (integrins, PLEC, FSCN1, FN1) that are highly expressed in amelanotic melanoma. Accompanying the down regulation of pigmentation specific proteins such as tyrosinase and TYRP1, these biomarkers are highly specific for a type of highly invasive melanoma. Interestingly, the LC-MS/MS proteomics analysis in hypoxia revealed that the abundance of this specific set of proteins found in normoxia was rather unaltered in these conditions. These biomarkers could therefore predict a metastatic behaviour for the amelanotic cells in the early stages of the tumor development and thus serve in melanoma prognostic. Applying this algorithm to related databases including melanoma samples published by independent laboratories/public databases we confirm the specificity of the newly found signatures. Overall, we begin to unravel the molecular alterations in the amelanotic melanoma and how basic proteomics offers insights into how to assess the clinical, pathological and misdiagnosis differences between the main subtypes of melanoma.
  
  
  
  
**FN1**
-
6
of
10
  

### Bidirectional regulation between tumor cell-intrinsic PD-L1 and TGF-β1 in epithelial-to-mesenchymal transition in melanoma.

6. Transl Cancer Res. 2022 Oct;11(10):3698-3710. doi: 10.21037/tcr-22-292
  
  
Li Z(#)(1), Wang F(#)(1), Dang J(2), Cheng F(1), Zheng F(3).
  
  
Author information: (1)Institute of Hematology, Union Hospital, Tongji Medical College, Huazhong Uni ...
  
  
BACKGROUND: Transforming growth factor-β1 (TGF-β1) is the predominant form of TGF-β and induces epithelial-to-mesenchymal transition (EMT) in melanoma. Tumor cell-intrinsic programmed death ligand-1 (PD-L1) plays a crucial role in maintenance of the EMT in melanoma. However, the relationship among tumor cell-intrinsic PD-L1, TGF-β1 and EMT is very complicated. METHODS: We investigated the bidirectional regulation between cell-intrinsic PD-L1 and TGF-β1 in melanoma, and explored the role of PD-L1 in TGF-β1-induced EMT and tumor progression. RESULTS: We found that TGF-β1 upregulated PD-L1 expression in B16-F0 and B16-F10 melanoma cells. Interestingly, PD-L1 also enhanced the intracellular TGF-β1 mRNA levels and induced the secretion of TGF-β1. Immunohistochemical staining revealed that PD-L1 protein expression was co-localized with α-smooth muscle actin (SMA) protein expression in melanoma, suggesting that PD-L1 was associated with EMT. By using shRNA lentivirus to knockdown PD-L1 (PD-L1-shRNA) in melanoma cell lines, we showed that TGF-β1-induced EMT was significantly inhibited in PD-L1-shRNA melanoma cells, which was characterized by the lower fibronectin (FN1) mRNA and higher E-cadherin (CDH1) mRNA levels (both are EMT markers) than that in control. TGF-β1-induced melanoma cell proliferation and migration were also markedly inhibited in PD-L1-shRNA cells. Consistent with the observation in vitro, PD-L1 knockdown inhibited tumor growth and repressed TGF-β1-induced EMT characterized by reduction of FN1 and increase of CDH1 in mouse model. CONCLUSIONS: The present study demonstrated a bidirectional regulation between cell-intrinsic PD-L1 and TGF-β1 in melanoma, which may help in designing promising combinations which include targeting TGF-β1 signaling along with PD-L1.
  
  
  
  
**FN1**
-
7
of
10
  

### Minimally invasive skin sampling and transcriptome analysis using microneedles for skin type biomarker research.

7. Skin Res Technol. 2022 Mar;28(2):322-335. doi: 10.1111/srt.13135 Epub 2022 Jan 10.
  
  
Kim SH(1), Kim JH(1), Lee SJ(1), Jung MS(1), Jeong DH(2), Lee KH(1)(3).
  
  
Author information: (1)Cutis Biomedical Research Center Co. Ltd., Seoul, Republic of Korea. (2)Rapha ...
  
  
BACKGROUND: Minimally invasive skin sampling is used in various fields. In this study, we examined whether it was possible to obtain skin specimens using biocompatible microneedles composed of sodium hyaluronate and performed transcriptome analysis. MATERIALS AND METHODS: Thirty-three subjects with different skin conditions, such as skin aging, skin hydration, skin pigmentation, oily skin and sensitive skin, were recruited. Skin types were evaluated based on age, non-invasive measurement devices, 10% lactic acid stinging test and visual assessment; the skin specimens were sampled from the face using microneedles. Total RNA was extracted, and microarray was performed. Correlations between various biomarkers and skin condition parameters were analysed. RESULTS: Several skin-type biomarkers are correlated with age, non-invasive device measurements, LAST score and visual assessment of acne lesions. Representatively, COL1A1 (Collagen type 1 alpha 1 chain), FN1 (Fibronectin 1) and PINK1 (PTEN-induced putative kinase protein 1) for skin aging, FLG (Filaggrin), KLF4 (Kruppel-like factor 4) and LOR (Loricrin) for skin hydration, GPNMB (Glycoprotein non-metastatic melanoma protein B), MLANA (Melan-A) and TYR (Tyrosinase) for skin pigmentation, IGF1 (insulin-like growth factor-1), MPZL3 (Myelin protein zero like 3) and AQP3 (Aquaporin 3) for oily skin and PGF (placental growth factor), CYR61 (cysteine-rich angiogenic inducer 61), RBP4 (retinol-binding protein 4), TAC1 (Tachykinin precursor 1), CAMP (Cathelicidin antimicrobial peptide), MMP9 (Matrix metallopeptidase 9), MMP3, MMP12 and CCR1 (C-C motif chemokine receptor 1) for sensitive skin. CONCLUSION: Microneedle skin sampling is a new and minimally invasive option for transcriptome analysis of human skin and can be applied for diagnosis and treatment efficacy evaluation, as well as skin type classification.
  
  
  
  
**FN1**
-
8
of
10
  

### Screening and identification of key genes and pathways in metastatic uveal melanoma based on gene expression using bioinformatic analysis.

8. Medicine (Baltimore). 2020 Oct 23;99(43):e22974. doi: 10.1097/MD.0000000000022974
  
  
Xie J(1), Wu Z(1), Xu X(2), Liang G(1), Xu J(1).
  
  
Author information: (1)Department of Ophthalmology. (2)Zhejiang Provincial Key Lab of Geriatrics & G ...
  
  
The current study aimed to elucidate the molecular mechanisms and identify the potential key genes and pathways for metastatic uveal melanoma (UM) using bioinformatics analysis.Gene expression microarray data from GSE39717 included 39 primary UM tissue samples and 2 metastatic UM tissue samples. Differentially expressed genes (DEGs) were generated using Gene Expression Omnibus 2R. Gene ontology (GO) and Kyoto Encyclopedia of Genes and Genomes (KEGG) pathway enrichment analyses were performed using the online Database for Annotation, Visualization and Integrated Discovery (DAVID) tool. The web-based STRING tool was adopted to construct a protein--protein interaction (PPI) network. The MCODE tool in Cytoscape was used to generate significant modules of the PPI network.A total of 213 DEGs were identified. GO and KEGG analyses revealed that the upregulated genes were mainly enriched in extracellular matrix organization and blood coagulation cascades, while the downregulated DEGs were mainly related to protein binding, negative regulation of ERK cascade, nucleus and chromatin modification, and lung and renal cell carcinoma. The most significant module was extracted from the PPI network. GO and KEGG enrichment analyses of the module revealed that the genes were mainly enriched in the extracellular region and space organization, blood coagulation process, and PI3K-Akt signaling pathway. Hub genes, including FN1, APOB, F2, SERPINC1, SERPINA1, APOA1, FGG, PROC, ITIH2, VCAN, TFPI, CXCL8, CDH2, and HP, were identified from DEGs. Survival analysis and hierarchical clustering results revealed that most of the hub genes were associated with prognosis and clinical progression.Results of this bioinformatics analysis may provide predictive biomarkers and potential candidate therapeutic targets for individuals with metastatic UM.
  
  
  
  
**FN1**
-
9
of
10
  

### Cancer cell line-specific protein profiles in extracellular vesicles identified by proteomics.

9. PLoS One. 2020 Sep 4;15(9):e0238591. doi: 10.1371/journal.pone.0238591 eCollection 2020.
  
  
Guerreiro EM(1), Øvstebø R(2), Thiede B(3), Costea DE(4)(5), Søland TM(1)(6), Kanli Galtung H(1).
  
  
Author information: (1)Institute of Oral Biology, Faculty of Dentistry, University of Oslo, Oslo, No ...
  
  
Extracellular vesicles (EVs), are important for intercellular communication in both physiological and pathological processes. To explore the potential of cancer derived EVs as disease biomarkers for diagnosis, monitoring, and treatment decision, it is necessary to thoroughly characterize their biomolecular content. The aim of the study was to characterize and compare the protein content of EVs derived from three different cancer cell lines in search of a specific molecular signature, with emphasis on proteins related to the carcinogenic process. Oral squamous cell carcinoma (OSCC), pancreatic ductal adenocarcinoma (PDAC) and melanoma brain metastasis cell lines were cultured in CELLine AD1000 flasks. EVs were isolated by ultrafiltration and size-exclusion chromatography and characterized. Next, the isolated EVs underwent liquid chromatography-mass spectrometry (LC-MS) analysis for protein identification. Functional enrichment analysis was performed for a more general overview of the biological processes involved. More than 600 different proteins were identified in EVs from each particular cell line. Here, 14%, 10%, and 24% of the identified proteins were unique in OSCC, PDAC, and melanoma vesicles, respectively. A specific protein profile was discovered for each cell line, e.g., EGFR in OSCC, Muc5AC in PDAC, and FN1 in melanoma vesicles. Nevertheless, 25% of all the identified proteins were common to all cell lines. Functional enrichment analysis linked the proteins in each data set to biological processes such as "biological adhesion", "cell motility", and "cellular component biogenesis". EV proteomics discovered cancer-specific protein profiles, with proteins involved in processes promoting tumor progression. In addition, the biological processes associated to the melanoma-derived EVs were distinct from the ones linked to the EVs isolated from OSCC and PDAC. The malignancy specific biomolecular cues in EVs may have potential applications as diagnostic biomarkers and in therapy.
  
  
  
  
**FN1**
-
10
of
10
  

### ALCAM (CD166) as a gene expression marker for human mesenchymal stromal cell characterisation.

10. Gene X. 2020 Mar 14;5:100031. doi: 10.1016/j.gene.2020.100031 eCollection 2020 Dec.
  
  
Brinkhof B(1), Zhang B(1), Cui Z(1), Ye H(1), Wang H(1)(2).
  
  
Author information: (1)Institute of Biomedical Engineering, Department of Engineering Science, Unive ...
  
  
BACKGROUND: Human mesenchymal stromal cells (MSCs) phenotypically share their positive expression of the International Society for Cell and Gene Therapy (ISCT) markers CD73, CD90 and CD105 with fibroblasts. Fibroblasts are often co-isolated as an unwanted by-product from biopsy and they can rapidly overgrow the MSCs in culture. Indeed, many other surface markers have been proposed, though no unique MSC specific marker has been identified yet. Quantitative PCR (qPCR) is a precise, efficient and rapid method for gene expression analysis. To identify a marker suitable for accurate MSC characterisation, qPCR was exploited. METHODS AND RESULTS: Two commercially obtained bone marrow (BM) derived MSCs and an hTERT immortalised BM-MSC line (MSC-TERT) have been cultured for different days and at different oxygen levels before RNA extraction. Together with RNA samples previous extracted from umbilical cord derived MSCs and MSC-TERT cells cultured in 2D or 3D, this heterogeneous sample set was quantitatively analysed for the expression levels of 18 candidate MSC marker genes. The expression levels in MSCs were compared with the expression levels in fibroblasts to verify the differentiation capability of these genes between MSCs and fibroblasts. None of the ISCT markers could differentiate between fibroblasts and MSCs. A total of six other genes (ALCAM, CLIC1, EDIL3, EPHA2, NECTIN2, and TMEM47) were identified as possible biomarkers for accurate identification of MSCs. CONCLUSION: Justified by considerations on expression level, reliability and specificity, Activated-Leukocyte Cell Adhesion Molecule (ALCAM) was the best candidate for improving the biomarker set of MSC identification.
  
  
  
  
  

---

  

# FOXO1

**Signature gene in:** CD8+ T cells
  
**Differentially expressed in:** CD8+ T cells
  
  
**FOXO1**
-
1
of
10
  

### Comparison of the Efficacy of Photodynamic Therapy Versus Cisplatin Application.

1. J Lasers Med Sci. 2024 Dec 24;15:e67. doi: 10.34172/jlms.2024.67 eCollection 2024.
  
  
Arjmand B(1)(2), Bahadorimonfared A(3), Jahani Sherafat S(4), Moravvej H(5), Rezaei M(6)(7), Daneshi ...
  
  
Author information: (1)Cell Therapy and Regenerative Medicine Research Center, Endocrinology and Met ...
  
  
Introduction: Photodynamic therapy (PDT) is a photochemical treatment that involves the use of light and photosensitizer. This method is applied as a therapeutic approach against several types of cancer. The main aim of this study is to compare the efficacy of PDT with that of cisplatin (a well-known chemotherapy agent) through protein-protein interaction (PPI) network analysis. Methods: Gene expression profiles of human melanoma A375 cells from the Gene Expression Omnibus (GEO) were selected for analysis via directed PPI network analysis. The significant differentially expressed genes (DEGs) were identified and assessed based on co-expression interactions. The critical DEGs were introduced by considering out-degree and in-degree values. Results: Two directed PPI networks for upregulated and downregulated DEGs were constructed. TP53 was identified as a critical upregulated gene in response to cisplatin in comparison with PDT. EGFR, PPARG, MMP9, PTGS2, FOXO1, and RUNX2 were highlighted as the crucial downregulated genes due to the effect of cisplatin on the gene expression of the treated cells. Conclusion: Cisplatin directly targets key cellular functions such as cell growth, differentiation, migration, and invasion. It seems that the combination of cisplatin and PDT is a suitable method for treating cancers because cisplatin targets the key genes responsible for cancer development, while PDT intensifies the effect of cisplatin and reduces its side effects.
  
  
  
  
**FOXO1**
-
2
of
10
  

### Rhabdomyosarcoma Harboring NRAS or HRAS Mutation Arising in Giant Congenital Melanocytic Nevus: Report of 2 Cases.

2. Am J Dermatopathol. 2025 Feb 6. doi: 10.1097/DAD.0000000000002934 Online ahead of print.
  
  
Kletskaya I(1), Konovalov D(2), Druy A(2), Abasov R(2), Salomatina A(2), Zaitseva M(2), Kazaryan G(3 ...
  
  
Author information: (1)Russian Children's Clinical Hospital of Pirogov's Russian National Research M ...
  
  
Nonmelanoma malignancies associated with congenital melanocytic nevi (CMN) are extremely rare, with only 12 reported cases of rhabdomyosarcoma (RMS) to date. We present 2 additional cases of RMS arising in giant CMN, with immunohistochemical and molecular biologic investigations. The first case was a 32-year-old woman with a personal history of melanoma in giant CMN who, after successful treatment and long remission, presented with a new 1-cm nodule within the CMN. Microscopically, the atypical areas exhibited a round cell/alveolar morphology with immunoreactivity for desmin and myogenin, and lacked PAX3/7::FOXO1 fusions typical for alveolar RMS on a reverse transcription polymerase chain reaction analysis. An identical NRAS p.Q61R mutation with comparable variant allele frequency (32% and 44%) was identified in both the nevus and the RMS tissue by next-generation sequencing. The second patient was a 5-year-old girl with a rapidly growing, bleeding, ulcerated 3 × 4 cm interscapular mass within a giant CMN that histologically seemed as a proliferation of pleomorphic spindle, polygonal and epithelioid cells with marked pleomorphism immunoreactive for myogenin, muscle-specific actin, and smooth muscle actin. Next-generation sequencing yielded an HRAS p.Q61R mutation with limited variant allele frequency (7%) in the RMS component, while ATRX p.Q2193\* variant was detected in the nevus. Our study is apparently the first report of NRAS and HRAS mutations in tumors with RMS phenotype arisen in CMN.
  
  
  
  
**FOXO1**
-
3
of
10
  

### An Optimized NGS Workflow Defines Genetically Based Prognostic Categories for Patients with Uveal Melanoma.

3. Biomolecules. 2025 Jan 18;15(1):146. doi: 10.3390/biom15010146
  
  
Massimino M(1)(2), Tirrò E(2), Stella S(2)(3), Tomarchio C(2)(3), Di Bella S(4), Vitale SR(2), Conti ...
  
  
Author information: (1)Department of General Surgery and Medical-Surgical Specialties, University of ...
  
  
BACKGROUND: Despite advances in uveal melanoma (UM) diagnosis and treatment, about 50% of patients develop distant metastases, thereby displaying poor overall survival. Molecular profiling has identified several genetic alterations that can stratify patients with UM into different risk categories. However, these genetic alterations are currently dispersed over multiple studies and several methodologies, emphasizing the need for a defined workflow that will allow standardized and reproducible molecular analyses. METHODS: Following the findings published by "The Cancer Genome Atlas-UM" (TCGA-UM) study, we developed an NGS-based gene panel (called the UMpanel) that classifies mutation sets in four categories: initiating alterations (CYSLTR2, GNA11, GNAQ and PLCB4), prognostic alterations (BAP1, EIF1AX, SF3B1 and SRSF2), emergent biomarkers (CDKN2A, CENPE, FOXO1, HIF1A, RPL5 and TP53) and chromosomal abnormalities (imbalances in chromosomes 1, 3 and 8). RESULTS: Employing commercial gene panels, reference mutated DNAs and Sanger sequencing, we performed a comparative analysis and found that our methodological approach successfully predicted survival with great specificity and sensitivity compared to the TCGA-UM cohort that was used as a validation group. CONCLUSIONS: Our results demonstrate that a reproducible NGS-based workflow translates into a reliable tool for the clinical stratification of patients with UM.
  
  
  
  
**FOXO1**
-
4
of
10
  

### Zinc dampens antitumor immunity by promoting Foxp3(+) regulatory T cells.

4. Front Immunol. 2024 Aug 23;15:1389387. doi: 10.3389/fimmu.2024.1389387 eCollection 2024.
  
  
Narayan S(1), Dalal R(1), Rizvi ZA(1)(2), Awasthi A(1)(2).
  
  
Author information: (1)Centre for Immunobiology and Immunotherapy, Translational Health Science and ...
  
  
INTRODUCTION: The role of zinc (Zn) in tumor development and immune modulation has always been paradoxical. This study redefines our understanding of the impact of Zn on cancer progression and therapeutic strategies. METHODS: We investigated the effects of dietary Zn levels on tumor progression and immune responses. This included examining the impact of both high and deficient dietary Zn, as well as Zn chelation, on tumor growth and immune cell populations. Specifically, we analyzed the frequency of Foxp3+ regulatory T-cells (Tregs) and identified the role of FOXO1 in Zn-mediated effects on Tregs. Additionally, we explored the therapeutic potential of clioquinol (CQ) in enhancing α-PD-1 immunotherapy responses, particularly in melanoma. RESULTS: Our findings show that high dietary Zn promotes tumor progression by fostering a protumorigenic environment mediated by T cells. Increased Zn intake was found to facilitate tumor progression by increasing Foxp3+ Treg frequency. In contrast, deficiency in dietary Zn and chelation of tissue Zn emerged as potent drivers of antitumor immunity. We pinpointed FOXO1 as the master regulator governing the influence of Zn on Tregs. DISCUSSION: These results reveal a novel mechanistic insight into how Zn influences tumor progression and immune regulation. The identification of FOXO1 as a key regulator opens new avenues for understanding the role of Zn in cancer biology. Furthermore, we introduce a promising therapeutic approach by showing that administering clioquinol (CQ) significantly enhances α-PD-1 immunotherapy response, particularly in melanoma. These revelations transform our comprehension of the multifaceted role of Zn in tumorigenesis and immune regulation, highlighting innovative possibilities for cancer therapy.
  
  
  
  
**FOXO1**
-
5
of
10
  

### Comprehensive pan-cancer analysis of mitochondrial outer membrane permeabilisation activity reveals positive immunomodulation and assists in identifying potential therapeutic targets for immunotherapy resistance.

5. Clin Transl Med. 2024 Jun;14(6):e1735. doi: 10.1002/ctm2.1735
  
  
Chen Q(1)(2), Gao F(3), Wu J(1)(4), Zhang K(1)(2), Du T(1)(2), Chen Y(1), Cai R(1)(2), Zhao D(1)(2), ...
  
  
Author information: (1)State Key Laboratory of Oncology in South China, Guangdong Provincial Clinica ...
  
  
BACKGROUND: Mitochondrial outer membrane permeabilisation (MOMP) plays a pivotal role in cellular death and immune activation. A deeper understanding of the impact of tumour MOMP on immunity will aid in guiding more effective immunotherapeutic strategies. METHODS: A comprehensive pan-cancer dataset comprising 30 cancer-type transcriptomic cohorts, 20 immunotherapy transcriptomic cohorts and three immunotherapy scRNA-seq datasets was collected and analysed to determine the influence of tumour MOMP activity on clinical prognosis, immune infiltration and immunotherapy effectiveness. Leveraging 65 scRNA-Seq datasets, the MOMP signature (MOMP.Sig) was developed to accurately reflect tumour MOMP activity. The clinical predictive value of MOMP.Sig was explored through machine learning models. Integration of the MOMP.Sig model and a pan-cancer immunotherapy CRISPR screen further investigated potential targets to overcome immunotherapy resistance, which subsequently underwent clinical validation. RESULTS: Our research revealed that elevated MOMP activity reduces mortality risk in cancer patients, drives the formation of an anti-tumour immune environment and enhances the response to immunotherapy. This finding emphasises the potential clinical application value of MOMP activity in immunotherapy. MOMP.Sig, offering a more precise indicator of tumour cell MOMP activity, demonstrated outstanding predictive efficacy in machine-learning models. Moreover, with the assistance of the MOMP.Sig model, FOXO1 was identified as a core modulator that promotes immune resistance. Finally, these findings were successfully validated in clinical immunotherapy cohorts of skin cutaneous melanoma and triple-negative breast cancer patients. CONCLUSIONS: This study enhances our understanding of MOMP activity in immune modulation, providing valuable insights for more effective immunotherapeutic strategies across diverse tumours.
  
  
  
  
**FOXO1**
-
6
of
10
  

### The emerging role of T helper 9 (Th9) cells in immunopathophysiology: A comprehensive review of their effects and responsiveness in various disease states.

6. Int Rev Immunol. 2024;43(6):341-360. doi: 10.1080/08830185.2024.2364586 Epub 2024 Jun 12.
  
  
Khokhar M(1), Purohit P(1).
  
  
Author information: (1)Department of Biochemistry, All India Institute of Medical Sciences (AIIMS), ...
  
  
Th9 cells, a subset of T-helper cells producing interleukin-9 (IL-9), play a vital role in the adaptive immune response and have diverse effects in different diseases. Regulated by transcription factors like PU.1 and IRF4, and cytokines such as IL-4 and TGF-β, Th9 cells drive tissue inflammation. This review focuses on their emerging role in immunopathophysiology. Th9 cells exhibit immune-mediated cancer cell destruction, showing promise in glioma and cervical cancer treatment. However, their role in breast and lung cancer is intricate, requiring a deeper understanding of pro- and anti-tumor aspects. Th9 cells, along with IL-9, foster T cell and immune cell proliferation, contributing to autoimmune disorders. They are implicated in psoriasis, atopic dermatitis, and infections. In allergic reactions and asthma, Th9 cells fuel pro-inflammatory responses. Targeting FOXO1 may regulate innate and adaptive immune responses, alleviating disease symptoms. This comprehensive review outlines Th9 cells' evolving immunopathophysiological role, emphasizing the necessity for further research to grasp their effects and potential therapeutic applications across diseases.
  
  
  
  
**FOXO1**
-
7
of
10
  

### TRIM11 attenuates Treg cell differentiation by p62-selective autophagic degradation of AIM2.

7. Cell Rep. 2023 Oct 31;42(10):113231. doi: 10.1016/j.celrep.2023.113231 Epub 2023 Oct 6.
  
  
Yu T(1), Yang X(2), Fu Q(3), Liang J(4), Wu X(3), Sheng J(5), Chen Y(5), Xiao L(2), Wu Y(6), Nie D(5 ...
  
  
Author information: (1)The Second Affiliated Hospital, The State Key Laboratory of Respiratory Disea ...
  
  
Ubiquitination is an important protein modification that regulates diverse biological processes, including CD4+ T cell differentiation and functions. However, the function of most E3 ubiquitin ligases in CD4+ T cell differentiation and CD4+ T cell-mediated pathological diseases remains unclear. In this study, we find that tripartite motif-containing motif 11 (TRIM11) specifically negatively regulates regulatory T (Treg) cell differentiation in CD4+ T cells and promotes autoimmune disease development in an AIM2-dependent manner. Mechanistically, TRIM11 interacts with absent in melanoma 2 (AIM2) and promotes the selective autophagic degradation of AIM2 by inducing AIM2 ubiquitination and binding to p62 in CD4+ T cells. AIM2 attenuates AKT and FOXO1 phosphorylation, MYC signaling, and glycolysis, thereby promoting the stability of Treg cells during experimental autoimmune encephalomyelitis (EAE). Our findings suggest that TRIM11 serves as a potential target for immunotherapeutic intervention for dysregulated immune responses that lead to autoimmunity and cancers.
  
  
  
  
**FOXO1**
-
8
of
10
  

### Basigin is necessary for normal decidualization of human uterine stromal cells.

8. Hum Reprod. 2022 Nov 24;37(12):2885-2898. doi: 10.1093/humrep/deac229
  
  
Yang S(1)(2), Bi J(1), Drnevich J(3), Li K(1), Nowak RA(1).
  
  
Author information: (1)Department of Animal Sciences, University of Illinois, Urbana, IL, USA. (2)De ...
  
  
STUDY QUESTION: Does basigin (BSG) regulate human endometrial stromal cell (HESC) decidualization in vitro? SUMMARY ANSWER: BSG regulates HESCs proliferation and decidualization. WHAT IS KNOWN ALREADY: Studies have shown that in the human endometrium, BSG expression is menstrual-cycle dependent and its expression was significantly lower in uterine endometrium during the luteal phase of women experiencing multiple implantation failures after IVF than in women with normal fertility. STUDY DESIGN, SIZE, DURATION: We utilized a telomerase-immortalized HESCs in an in vitro cell culture model system to investigate whether BSG regulates decidualization of stromal cells. Further, we used microarray analysis to identify changes in the gene expression profile of HESCs treated with BSG small interfering RNA (siRNA). All experiments were repeated at least three times. PARTICIPANTS/MATERIALS, SETTING, METHODS: The effect of BSG knockdown (using siRNA) on HESC proliferation was determined by counting cell number and by tritiated thymidine incorporation assays. The effect of BSG on decidualization of HESCs was determined by RT-qPCR for the decidualization markers insulin-like growth factor-binding protein 1 (IGFBP1) and prolactin (PRL). Immunoblotting was used to determine the effect of BSG siRNA on the expression of MMP-2,3. Microarray analysis was used to identify BSG-regulated genes in HESCs at Day 6 of decidualization. Functional and pathway enrichment analyses were then carried out on the differentially expressed genes (DEGs). The STRING online database was used to analyze protein-protein interaction (PPI) between DEG-encoded proteins, and CytoScape software was used to visualize the interaction. MCODE and CytoHubba were used to construct functional modules and screen hub genes separately. Several BSG-regulated genes identified in the microarray analysis were confirmed by qPCR. MAIN RESULTS AND THE ROLE OF CHANCE: Knockdown of BSG expression in cultured stromal cells by siRNA significantly (P < 0.05) inhibited HESC proliferation, disrupted cell decidualization and down-regulated MMP-2 and MMP-3 expression. Microarray analysis identified 721 genes that were down-regulated, and 484 genes up-regulated with P < 0.05 in BSG siRNA treated HESCs. GO term enrichment analysis showed that the DEGs were significantly enriched in cell communication, signaling transduction and regulation, response to stimulus, cell adhesion, anatomical structure morphogenesis, extracellular matrix organization, as well as other functional pathways. KEGG pathway analysis identified upregulated gene enriched in pathways such as the MAPK signaling pathway, colorectal cancer, melanoma and axon guidance. In contrast, downregulated genes were mainly enriched in pathways including ECM-receptor interaction, PI3K-Akt signaling pathway, pathways in cancer, antigen processing, type I diabetes mellitus and focal adhesion. The top 10 hub nodes were identified using 12 methods analyses. The hub genes that showed up in two methods were screened out. Among these genes, upregulated genes included EGFR, HSP90AA1, CCND1, PXN, PRKACB, MGAT4A, EVA1A, LGALS1, STC2, HSPA4; downregulated genes included WNT4/5, FOXO1, CDK1, PIK3R1, IGF1, JAK2, LAMB1, ITGAV, HGF, MXRA8, TMEM132A, UBE2C, QSOX1, ERBB2, GNB4, HSP90B1, LAMB2, LAMC1 and ITGA1. Hub genes and module genes involved in the top three modules of PPI analysis were analyzed through the string database. Analysis showed that hub and module genes were related mainly to the WNT signaling pathway, PI3K-AKT signaling pathway and pathways in cancer. LARGE SCALE DATA: The microarray data set generated in this study has been published online at databank.illinois.edu. LIMITATIONS, REASONS FOR CAUTION: Most of the findings were obtained using an in vitro cell culture system that may not necessarily reflect in vivo functions. WIDER IMPLICATIONS OF THE FINDINGS: Our results demonstrate that BSG plays a vital role in decidualization and that downregulation of BSG in the uterine endometrium may be associated with infertility in women. The identified hub genes and pathways increase our understanding of the genetic etiology and molecular mechanisms underlying the regulation of decidualization by BSG. STUDY FUNDING/COMPETING INTEREST(S): This work was supported by the NIH U54 HD40093 (R.A.N.). The authors have no competing interests to declare.
  
  
  
  
**FOXO1**
-
9
of
10
  

### RSK2 promotes melanoma cell proliferation and vemurafenib resistance via upregulating cyclin D1.

9. Front Pharmacol. 2022 Sep 20;13:950571. doi: 10.3389/fphar.2022.950571 eCollection 2022.
  
  
Wu HZ(1)(2), Li LY(1)(2), Jiang SL(1), Li YZ(1)(3), Shi XM(1)(2), Sun XY(1)(2), Li Z(1), Cheng Y(1)( ...
  
  
Author information: (1)Department of Pharmacy, The Second Xiangya Hospital, Central South University ...
  
  
BRAF inhibitors are commonly used in targeted therapies for melanoma patients harboring BRAFV600E mutant. Despite the benefit of vemurafenib therapy, acquired resistance during or after treatment remains a major obstacle in BRAFV600E mutant melanoma. Here we found that RSK2 is overexpressed in melanoma cells and the high expression of RSK2 indicates poor overall survival (OS) in melanoma patients. Overexpression of RSK2 leads to vemurafenib resistance, and the deletion of RSK2 inhibits cell proliferation and sensitizes melanoma cells to vemurafenib. Mechanistically, RSK2 enhances the phosphorylation of FOXO1 by interacting with FOXO1 and promoting its subsequent degradation, leading to upregulation of cyclin D1 in melanoma cells. These results not only reveal the presence of a RSK2-FOXO1-cyclin D1 signaling pathway in melanoma, but also provide a potential therapeutic strategy to enhance the efficacy of vemurafenib against cancer.
  
  
  
  
**FOXO1**
-
10
of
10
  

### The FoxO4/DKK3 axis represses IFN-γ expression by Th1 cells and limits antimicrobial immunity.

10. J Clin Invest. 2022 Sep 15;132(18):e147566. doi: 10.1172/JCI147566
  
  
Chen X(1)(2), Hu J(3)(4), Wang Y(5), Lee Y(2), Zhao X(1), Lu H(1)(6), Zhu G(1), Wang H(7), Jiang Y(1 ...
  
  
Author information: (1)Institute for Immunology and School of Medicine, Tsinghua University, Beijing ...
  
  
Forkhead box O transcriptional factors, especially FOXO1 and FoxO3a, play critical roles in physiologic and pathologic immune responses. However, the function of FoxO4, another main member of the FoxO family, in lymphoid cells is still poorly understood. Here, we showed that loss of FoxO4 in T cells augmented IFN-γ production of Th1 cells in vitro. Correspondingly, conditional deletion of FoxO4 in CD4+ T cells enhanced T cell-specific responses to Listeria monocytogenes infection in vivo. Genome-wide occupancy and transcriptomic analyses identified Dkk3 (encoding the Dickkopf-3 protein) as a direct transcriptional target of FoxO4. Consistent with the FoxO4-DKK3 relationship, recombinant DKK3 protein restored normal levels of IFN-γ production in FoxO4-deficient Th1 cells through the downregulation of lymphoid enhancer-binding factor 1 (Lef1) expression. Together, our data suggest a potential FoxO4/DKK3 axis in Th1 cell differentiation, providing what we believe to be an important insight and supplement for FoxO family proteins in T lymphocyte biology and revealing a promising target for the treatment of immune-related diseases.
  
  
  
  
  

---

  

# FOXO3

**Signature gene in:** Endothelial cells
  
**Differentially expressed in:** Endothelial cells
  
  
**FOXO3**
-
1
of
10
  

### Transcriptomic analysis of genes associated with vitamin D receptor signalling reveals differences between skin cancers.

1. Exp Dermatol. 2024 Oct;33(10):e15160. doi: 10.1111/exd.15160
  
  
Ocanha-Xavier JP(1)(2), Xavier-Junior JCC Jr(1)(3)(4), Miot HA(5), da Silva MG(1), Marques MEA(1).
  
  
Author information: (1)Department of Pathology, São Paulo State University-UNESP, Botucatu, São Paul ...
  
  
Vitamin D activates the vitamin D receptor (VDR), which dimerizes preferentially with the retinoid X receptor-α (RXRα). This heterodimer connects with genetic elements responsive to vitamin D, inhibiting or stimulating gene activity. We performed Nanostring® analysis of VDR/RXRα to compare the mRNA expression of this heterodimer and their correlated transcriptomes in non-melanoma skin cancer (basal cell carcinomas (BCC) and squamous cell carcinomas (SCC)) and melanocytic lesions (intradermal nevi (IN), and melanomas (MM)) with control skin. To evaluate VDR, RXRα and other 22 correlated genes in BCC, SCC, IN and MM, paraffin samples had their transcriptomes analysed using Nanostring®, a platform that allows multiple mRNA analyses. There were 46 samples, including 11 BCC, 10 SCC, 10 IN, 12 MM and 3 pools of control skins. Most mRNAs differed between the lesion groups and the control group. BCC and SCC NCOR2 were upregulated; in MM and IN, RXRγ was higher than in the control group. TP53, FOXO3 and MED1 showed a significant difference when we compared the BCC group to the SCC group. Melanoma and intradermal nevi differed only in AhR. VDR and RXRα were lower than the control in all groups. The panel shows a clear difference between the non-melanocytic cancers and, on the other hand, a slight difference between the melanocytic lesions. The study of vitamin D's influence through its receptor and RXRα is an exciting issue for understanding the importance of this pathway, and the present study can impact the prevention and treatment strategies, mainly in non-melanocytic tumours.
  
  
  
  
**FOXO3**
-
2
of
10
  

### Photobiomodulation Using 830 nm Lighting-Emitting Diode Inhibits Melanogenesis via FOXO3a in Human Melanocyte.

2. Pigment Cell Melanoma Res. 2024 Sep;37(5):681-692. doi: 10.1111/pcmr.13193 Epub 2024 Aug 21.
  
  
Dan Y(1), Chen L(1), Jin S(1), Xing X(1), Zhu Y(1), Jiang M(1), Zhang C(1), Xiang LF(1).
  
  
Author information: (1)Department of Dermatology, Huashan Hospital, Fudan University, Shanghai, PR C ...
  
  
Photobiomodulation (PBM) using 830 nm light-emitting diode (LED) benefits tissue regeneration, wound healing and neural stimulation. However, there is not much exploration of its effect on melanocytes and ex vivo skin model. This study aims to investigate the mechanism behind the anti-melanogenic activity of 830 nm LED and provides evidence for its activity in human ex vivo skin model. Our results showed that 830 nm LED at fluences ranging from 5 to 20 J/cm2 inhibited melanosome maturation and reduced melanin content, tyrosinase activity and melanogenesis-related proteins. 830 nm LED inhibited the phosphorylation of AKT and its downstream FOXO3a, leading to nuclear translocation of FOXO3a. Furthermore, FOXO3a knockdown and AKT activator like SC79 could reverse the melanogenesis inhibition phenotype induced by 830 nm LED. In human ex vivo skin model, Fontana-Masson staining revealed a decrease in epidermal basal pigmentation after 830 nm LED irradiation. Taken together, 830 nm LED demonstrated the anti-melanogenic activity via FOXO3a.
  
  
  
  
**FOXO3**
-
3
of
10
  

### FOXO3 Inhibits the Cisplatin Resistance and Progression of Melanoma Cells by Promoting CDKN1C Transcription.

3. Appl Biochem Biotechnol. 2024 Nov;196(11):7834-7848. doi: 10.1007/s12010-024-04909-3 Epub 2024 Apr 3.
  
  
Yang C(#)(1), Yan Z(#)(2), Sun Z(1), Hu F(1), Xu W(3).
  
  
Author information: (1)Department of Oncology, Xiangyang Central Hospital, Affiliated Hospital of Hu ...
  
  
BACKGROUND: Forkhead box O3 (FOXO3) and cyclin dependent kinase inhibitor 1 C Gene (CDKN1C) have been shown to be involved in the melanoma process, but their roles in the cisplatin (DDP) resistance of melanoma remain unclear. METHODS: The mRNA levels of CDKN1C and FOXO3 were measured using quantitative real-time PCR. The protein levels of CDKN1C, FOXO3 and mitochondrial oxidative phosphorylation (mtOXPHOS)-related markers were determinant by western blot analysis. The DDP resistance, proliferation, and apoptosis of melanoma cells were assessed by cell counting kit 8 assay, colony formation assay and flow cytometry. Glucose consumption, lactate production and ATP level were detected to assess glycolysis. The regulation of FOXO3 on CDKN1C was confirmed by ChIP assay and dual-luciferase reporter assay. In vivo experiments were performed to evaluate the effect of FOXO3 on DDP sensitivity in melanoma tumor tissues. RESULTS: CDKN1C and FOXO3 were downregulated in chemoresistant melanoma tissues, and their low expression levels were related to the poor prognosis of melanoma patients. Overexpression of CDKN1C and FOXO3 repressed DDP resistance, proliferation, and glycolysis, while promoted apoptosis and mtOXPHOS in DDP-resistant melanoma cells. Further analysis suggested that FOXO3 could bind to CDKN1C promoter region to enhance its transcription. Besides, CDKN1C knockdown reversed the regulation of FOXO3 on melanoma cell DDP resistance and progression. Moreover, FOXO3 overexpression enhanced the DDP sensitivity of melanoma tumor tissues in vivo. CONCLUSION: FOXO3 promoted the transcription of CDKN1C, thereby inhibiting the DDP resistance and progression of melanoma cells.
  
  
  
  
**FOXO3**
-
4
of
10
  

### SIRT1-mediated deacetylation of FOXO3a transcription factor supports pro-angiogenic activity of interferon-deficient tumor-associated neutrophils.

4. Int J Cancer. 2022 Apr 1;150(7):1198-1211. doi: 10.1002/ijc.33871 Epub 2021 Nov 24.
  
  
Bordbari S(1), Mörchen B(2), Pylaeva E(1), Siakaeva E(1), Spyra I(1), Domnich M(1), Droege F(1), Kan ...
  
  
Author information: (1)Department of Otorhinolaryngology, University Hospital Essen, University Duis ...
  
  
Angiogenesis plays an important role during tumor growth and metastasis. We could previously show that Type I interferon (IFN)-deficient tumor-associated neutrophils (TANs) show strong pro-angiogenic activity, and stimulate tumor angiogenesis and growth. However, the exact mechanism responsible for their pro-angiogenic shift is not clear. Here, we set out to delineate the molecular mechanism and factors regulating pro-angiogenic properties of neutrophils in the context of Type I IFN availability. We demonstrate that neutrophils from IFN-deficient (Ifnar1-/- ) mice efficiently release pro-angiogenic factors, such as VEGF, MMP9 or BV8, and thus significantly support the vascular normalization of tumors by increasing the maturation of perivascular cells. Mechanistically, we could show here that the expression of pro-angiogenic factors in neutrophils is controlled by the transcription factor forkhead box protein O3a (FOXO3a), which activity depends on its post-translational modifications, such as deacetylation or phosphorylation. In TANs isolated from Ifnar1-/- mice, we observe significantly elevated SIRT1, resulting in SIRT1-mediated deacetylation of FOXO3a, its nuclear retention and activation. Activated FOXO3a supports in turn the transcription of pro-angiogenic genes in TANs. In the absence of SIRT1, or after its inhibition in neutrophils, elevated kinase MEK/ERK and PI3K/AKT activity is observed, leading to FOXO3a phosphorylation, cytoplasmic transfer and inactivation. In summary, we have found that FOXO3a is a key transcription factor controlling the angiogenic switch of neutrophils. Post-translational FOXO3a modifications regulate its transcriptional activity and, as a result, the expression of pro-angiogenic factors supporting development of vascular network in growing tumors. Therefore, targeting FOXO3a activity could provide a novel strategy of antiangiogenic targeted therapy for cancer.
  
  
  
  
**FOXO3**
-
5
of
10
  

### Determination of hypoxia signature to predict prognosis and the tumor immune microenvironment in melanoma.

5. Mol Omics. 2021 Apr 1;17(2):307-316. doi: 10.1039/d0mo00159g Epub 2021 Feb 24.
  
  
Shou Y(1), Yang L, Yang Y, Zhu X, Li F, Xu J.
  
  
Author information: (1)Department of Dermatology, Huashan Hospital, Fudan University, 12 Wulumuqi Zh ...
  
  
Melanoma is one of the highly malignant skin tumors, the incidence and death of which continue to increase. The hypoxic microenvironment drives tumor growth, progression, and heterogeneity; it also triggers a cascade of immunosuppressive responses and affects the levels of T cells, macrophages, and natural killer cells. Here, we aim to develop a hypoxia-based gene signature for prognosis evaluation and help evaluate the status of hypoxia and the immune microenvironment in melanoma. Based on the data from The Cancer Genome Atlas (TCGA) and Gene Expression Omnibus (GEO) database, we performed integrated bioinformatics to analyze the hypoxia-related genes. Using Lasso Cox regression, a hypoxia model was constructed. The receiver operating characteristic and the Kaplan-Meier curve were used to evaluate the predictive capacity of the model. With the CIBERSORT algorithm, the abundance of 22 immune cells in the melanoma microenvironment was analyzed. A total of 20 hypoxia-related genes were significantly related to prognosis in the log-rank test. Lasso regression showed that FBP1, SDC3, FOXO3, IGFBP1, S100A4, EGFR, ISG20, CP, PPARGC1A, KIF5A, and DPYSL4 displayed the best features. Based on these genes, a hypoxia model was established, and the area under the curve for the model was 0.734. Furthermore, the hypoxia score was identified as an independent prognostic factor. Besides, the hypoxia score could also predict the immune microenvironment in melanoma. Down-regulated activated CD4 memory T cells, CD8 T cells, and M1-like macrophages, and up-regulated Tregs were observed in patients with a high hypoxia score. The hypoxia-related genes were identified, and the hypoxia score was found to be a prognostic factor for overall survival and a predictor for the immune microenvironment. Our findings provide new ideas for evaluation and require further validation in clinical practice.
  
  
  
  
**FOXO3**
-
6
of
10
  

### Pristimerin-induced uveal melanoma cell death via inhibiting PI3K/Akt/FoxO3a signalling pathway.

6. J Cell Mol Med. 2020 Jun;24(11):6208-6219. doi: 10.1111/jcmm.15249 Epub 2020 Apr 29.
  
  
Yan F(1)(2), Liao R(1)(3), Silva M(1), Li S(1), Jiang Y(1), Peng T(1), Lazarovici P(4), Zheng W(1).
  
  
Author information: (1)Faculty of Health Sciences, University of Macau, Macau, China. (2)School of M ...
  
  
Uveal melanoma (UM) is a highly invasive intraocular malignancy with high mortality. Presently, there is no FDA-approved standard for the treatment of metastatic UM. Pristimerin is a natural quinine methide triterpenoid compound with anti-angiogenic, anti-cancer and anti-inflammatory activities. However, Pristimerin potential cytotoxic effect on UM was poorly investigated. In the present study, we found the migration and invasion of UM-1 cells were inhibited by Pristimerin which also caused a rapid increase of ROS, decreased mitochondrial membrane potential, induced the accumulation of cells in G0/G1 phase, ending with apoptotic cell death. Pristimerin inhibited Akt and FoxO3a phosphorylation and induced nuclear accumulation of FoxO3a in UM-1 cells, increased the expression of pro-apoptotic proteins Bim、p27Kip1 , cleaved caspase-3, PARP and Bax, and decreased the expression of Cyclin D1 and Bcl-2. LY294002 or Akt-siRNA inhibited the PI3K/Akt/FoxO3a pathway and promoted the Pristimerin-induced apoptosis, while Pristimerin effects were partially abolished in FoxO3a knockdown UM-1 cell cultures. Taken together, present results showed that Pristimerin induced apoptotic cell death through inhibition of PI3K/Akt/FoxO3a pathway in UM-1 cells. These findings indicate that Pristimerin may be considered as a potential chemotherapeutic agent for patients with UM.
  
  
  
  
**FOXO3**
-
7
of
10
  

### FOXO3a‑SIRT6 axis suppresses aerobic glycolysis in melanoma.

7. Int J Oncol. 2020 Mar;56(3):728-742. doi: 10.3892/ijo.2020.4964 Epub 2020 Jan 17.
  
  
Dong Z(1), Yang J(1), Li L(1), Tan L(1), Shi P(1), Zhang J(1), Zhong X(1), Ge L(1), Wu Z(2), Cui H(1 ...
  
  
Author information: (1)State Key Laboratory of Silkworm Genome Biology, Institute of Sericulture and ...
  
  
Melanoma, the most aggressive human skin tumor, has a very short survival time, and there are currently no effective treatments. Alterations in cell metabolism, such as enhanced aerobic glycolysis, have been identified as hallmarks of cancer cells. In the present study, bioinformatics studies using online databases revealed that FOXO3a expression was lower in melanoma tissues compared with normal tissues and nevus. Additionally, Kaplan‑Meier analysis showed that high expression of FOXO3a predicted an improved prognosis for patients with melanoma. Furthermore, Pearson correlation analysis indicated that the expression of FOXO3a was positively correlated with SIRT6 expression and negatively correlated with the expression levels of a number of glycolysis‑associated genes. Chromatin immunoprecipitation and luciferase assays showed that FOXO3a was enriched in the SIRT6 promoter region and promoted its transcription. Then, SIRT6 was overexpressed in FOXO3a‑knockdown MV3 cells and downregulated in FOXO3a‑overexpressing MV3 cells by using lentivirus‑mediated stable infection. The results showed that SIRT6 knockdown or overexpression rescued the effects of FOXO3a overexpression or knockdown, respectively, on glycolysis, as determined by glucose uptake, glucose consumption and lactate production assays, the expression of glycolytic genes and glucose stress flux tests. SIRT6 overexpression also suppressed FOXO3a knockdown‑induced tumor growth in a mouse model. The present findings indicated that the FOXO3a‑SIRT6 regulatory axis inhibited glucose metabolism and tumor cell proliferation in melanoma, and provided novel insight into potential therapeutic strategies to treat this disease.
  
  
  
  
**FOXO3**
-
8
of
10
  

### Characterization of MicroRNAs Regulating FOXO Expression.

8. Methods Mol Biol. 2019;1890:13-28. doi: 10.1007/978-1-4939-8900-3\_2
  
  
Hanniford D(1)(2), Hernando E(3)(4).
  
  
Author information: (1)Department of Pathology, New York University Langone Health, New York, NY, US ...
  
  
MicroRNAs are critical post-transcriptional regulators of a majority of genes, of which the FOXO family of transcription factors is no exception. Here, we describe generalizable methods, including 3' UTR reporter assays and western blotting after microRNA manipulation, to test if a candidate miRNA (miR-182) directly targets a candidate (FOXO3) gene product. We also provide guidance on candidate miRNA selection and unbiased miRNA-target identification methods.
  
  
  
  
**FOXO3**
-
9
of
10
  

### Inhibition of mTORC2 enhances UVB-induced apoptosis in keratinocytes through a mechanism dependent on the FOXO3a transcriptional target NOXA but independent of TRAIL.

9. Cell Signal. 2018 Dec;52:35-47. doi: 10.1016/j.cellsig.2018.08.018 Epub 2018 Aug 30.
  
  
Feehan RP(1), Nelson AM(2), Shantz LM(3).
  
  
Author information: (1)Department of Cellular & Molecular Physiology, Penn State College of Medicine ...
  
  
The primary cause of non-melanoma skin cancer (NMSC) is ultraviolet B (UVB) radiation. We have shown previously that mTORC2 inhibition sensitizes keratinocytes to UVB-induced apoptosis mediated by the transcription factor FOXO3a. FOXO3a is a key regulator of apoptosis and a tumor suppressor in several cancer types. Activation of FOXO3a promotes apoptosis through the coordinated expression of a variety of target genes, including TRAIL and NOXA. We hypothesized that in the setting of mTORC2 inhibition, the UVB-induced expression of these factors would lead to apoptosis in a FOXO3a-dependent manner. Using spontaneously immortalized human keratinocytes (HaCaT cells), we observed that both TRAIL and NOXA expression increased in cells exposed to UVB and the TOR kinase inhibitor Torin 2. Similar to knockdown of FOXO3a, NOXA knockdown reversed the sensitization to UVB-induced apoptosis caused by mTORC2 inhibition. In contrast, loss of TRAIL by either knockdown or knockout actually enhanced expression of nuclear FOXO3a, which maintained apoptosis. These surprising results are not due to faulty death receptor signaling in HaCaT cells, as we found that the cells undergo extrinsic apoptosis in response to treatment with recombinant TRAIL. Even more striking, TRAIL knockout cells were sensitized to recombinant TRAIL-induced apoptosis compared to wild-type HaCaT cells, with the largest increase occurring in the presence of mTORC2 inhibition. Taken together, these studies provide strong evidence that mTORC2 controls UVB-induced apoptosis by regulating NOXA expression downstream of FOXO3a. Moreover, FOXO3a transcriptional activation by mTORC2 inhibitors may be a valuable target for prevention or therapy of NMSC, especially in cases with low endogenous TRAIL.
  
  
  
  
**FOXO3**
-
10
of
10
  

### NOVA1 acts as an oncogene in melanoma via regulating FOXO3a expression.

10. J Cell Mol Med. 2018 May;22(5):2622-2630. doi: 10.1111/jcmm.13527 Epub 2018 Mar 2.
  
  
Yu X(1), Zheng H(1), Chan MTV(2), Wu WKK(2)(3).
  
  
Author information: (1)Department of Dermatology, Peking Union Medical College Hospital, Chinese Aca ...
  
  
Increasing studies have suggested that dysregulation of RNA-binding proteins (RBPs) contributes to cancer progression. Neuro-oncological ventral antigen 1 (NOVA1) is a novel RBP and plays an important role in tumour development. However, the expression and role of NOVA1 in melanoma remain unknown. In this study, we indicated that NOVA1 expression was up-regulated in melanoma samples and cell lines. Moreover, we demonstrated that knockdown of NOVA1 suppressed melanoma cell proliferation, migration and invasion in both A375 and A875 cell lines. In addition, we showed that suppressed expression of NOVA1 enhanced forkhead box O3a (FOXO3a) expression while inhibited AKT expression in melanoma cell. Furthermore, we demonstrated that inhibited expression of FoxO3A rescued NOVA1-mediated cell proliferation, migration and invasion in melanoma cell line A375. These results suggested that NOVA1 acted as an oncogene in the development of melanoma partly through regulating FoxO3A expression.
  
  
  
  
  

---

  

# GALM

**Signature gene in:** CD8+ T cells
  
**Differentially expressed in:** CD8+ T cells
  
  
**GALM**
-
1
of
1
  

### Neobavaisoflavone Inhibits Melanogenesis through the Regulation of Akt/GSK-3β and MEK/ERK Pathways in B16F10 Cells and a Reconstructed Human 3D Skin Model.

1. Molecules. 2020 Jun 9;25(11):2683. doi: 10.3390/molecules25112683
  
  
Kim DE(1), Chang BY(1), Ham SO(2), Kim YC(3), Kim SY(1).
  
  
Author information: (1)Institute of Pharmaceutical Research and Development, College of Pharmacy, Wo ...
  
  
Previous studies have confirmed the anti-melanogenic effect of the aerial part of Pueraria lobata, however, due to its inherent color, P. lobata has limited commercial use. In this study, an extract (GALM-DC) of the aerial part of P. lobata having improved color by the use of activated carbon was obtained. Furthermore, the active compound neobavaisoflavone (NBI) was identified from GALM-DC. The effect of NBI on melanogenesis, tyrosinase activity, α-glucosidase activity, and mechanism of action in melanocytes was investigated. Tyrosinase activity, melanin contents and the expression of melanin-related genes and proteins were determined in B16F10 cells. NBI reduced melanin synthesis and tyrosinase activity. Furthermore, NBI treatment reduced the mRNA and protein expression levels of MITF, TRP-1, and tyrosinase. NBI also works by phosphorylating and activating proteins that inhibit melanogenesis, such as GSK3β and ERK. Specific inhibitors of Akt/GSK-3β (LY294002) and MEK/ERK (PD98059) signaling prevented the inhibition of melanogenesis by NBI. NBI inhibited melanin production through the regulation of MEK/ERK and Akt/GSK-3β signaling pathways in α-MSH-stimulated B16F10 cells. NBI suppresses tyrosinase activity and melanogenesis through inhibition of α-glucosidase activity. Besides, NBI significantly reduced melanogenesis in a reconstructed human 3D skin model. In conclusion, these results suggest that NBI has potential as a skin-whitening agent for hyperpigmentation.
  
  
  
  
  

---

  

# GCLC

**Signature gene in:** MDM
  
**Differentially expressed in:** MDM
  
  
**GCLC**
-
1
of
10
  

### Assessment of thermal and solvent stable SPME fibers for metabolomics studies performed in living systems.

1. Talanta. 2025 May 15;287:127646. doi: 10.1016/j.talanta.2025.127646 Epub 2025 Jan 31.
  
  
Kahremanoğlu K(1), Jaroch K(2), Szeliska P(2), Filipiak W(2), Charemski B(2), Żuchowska K(2), Çetin ...
  
  
Author information: (1)Department of Chemistry, Middle East Technical University, Ankara, 06800, Tür ...
  
  
Solid phase microextraction (SPME), as a sampling/sample preparation technique, offers unique solutions for the most challenging applications, including metabolomics studies of living systems. However, for global metabolomics it is critical to use an SPME sampler facilitating the extraction of both volatiles and nonvolatiles, which at the same time is compatible with thermal and solvent-assisted desorption. As a promising universal coating, recently hydrophilic-lipophilic balanced (HLB) particles immobilized in PTFE have been introduced as a new SPME sampler to provide a wide-range of analyte coverage and compatibility with solvent and thermal desorption. Thus, making it suitable for both gas and liquid chromatography (GC/LC) based applications. However, its potential in metabolomics has not been investigated to date. In this study, HLB/PTFE SPME fibers were prepared, evaluated with selected polar and non-polar metabolites relevant to biological systems, and validated for cell-line studies. The validation proved that these fibers can extract a wide-range of molecules (LogP: 4.2 to 15.6) with acceptable accuracy (≤19% RE%) and repeatability (intra-day ≤17% and inter-day 12% RSD%). The LOQ was determined to vary between 150.0 and 500.0 ng/mL. Upon validation, the fibers were used in a proof-of-concept study for extraction of endometabolome and exometabolome of melanoma B16F10 and lung cancer LL2 cell lines. The metabolome studies showed that HLB/PTFE fibers provide lower coverage, but for some compounds higher extraction efficiency compared to HLB/PAN fibers used in LC-based metabolomics. Fibers also proved suitable for GC-MS analysis, allowing for the detection of 36 volatile organic compounds in the headspace of the cell lines and RPMI medium.
  
  
  
  
**GCLC**
-
2
of
10
  

### Differential Induction of Reactive Oxygen Species and Expression of Antioxidant Enzymes in Human Melanocytes Correlate with Melanin Content: Implications on the Response to Solar UV and Melanoma Susceptibility.

2. Antioxidants (Basel). 2022 Jun 20;11(6):1204. doi: 10.3390/antiox11061204
  
  
Upadhyay PR(1)(2), Starner RJ(2), Swope VB(2), Wakamatsu K(3), Ito S(3), Abdel-Malek ZA(2).
  
  
Author information: (1)Division of Pharmaceutical Sciences, College of Pharmacy, University of Cinci ...
  
  
Constitutive pigmentation determines the response to sun exposure and the risk for melanoma, an oxidative stress-driven tumor. Using primary cultures of human melanocytes, we compared the effects of constitutive pigmentation on their antioxidant response to solar UV. The quantitation of eumelanin and pheomelanin showed that the eumelanin content and eumelanin to pheomelanin ratio correlated inversely with the basal levels of reactive oxygen species (ROS). Irradiation with 7 J/cm2 solar UV increased ROS generation without compromising melanocyte viability. Among the antioxidant enzymes tested, the basal levels of heme oxygenase-1 (HO-1) and the glutamate cysteine ligase catalytic subunit and modifier subunit (GCLC and GCLM) correlated directly with the eumelanin and total melanin contents. The levels of HO-1 and GCLM decreased at 6 h but increased at 24 h post-solar UV. Consistent with the GCLC and GCLM levels, the basal glutathione (GSH) content was significantly lower in light than in dark melanocytes. The expression of HMOX1, GCLC, GCLM, and CAT did not correlate with the melanin content and was reduced 3 h after solar UV irradiation, particularly in lightly pigmented melanocytes. Solar UV increased p53 and lipid peroxidation, which correlated inversely with the eumelanin and total melanin contents. These intrinsic differences between light and dark melanocytes should determine their antioxidant response and melanoma risk.
  
  
  
  
**GCLC**
-
3
of
10
  

### Integrated analysis of single-cell and bulk RNA sequencing data reveals a pan-cancer stemness signature predicting immunotherapy response.

3. Genome Med. 2022 Apr 29;14(1):45. doi: 10.1186/s13073-022-01050-w
  
  
Zhang Z(#)(1)(2), Wang ZX(#)(1)(2)(3), Chen YX(#)(1)(2), Wu HX(#)(1)(2), Yin L(1)(2), Zhao Q(1)(2), ...
  
  
Author information: (1)Department of Medical Oncology, Sun Yat-sen University Cancer Center, State K ...
  
  
BACKGROUND: Although immune checkpoint inhibitor (ICI) is regarded as a breakthrough in cancer therapy, only a limited fraction of patients benefit from it. Cancer stemness can be the potential culprit in ICI resistance, but direct clinical evidence is lacking. METHODS: Publicly available scRNA-Seq datasets derived from ICI-treated patients were collected and analyzed to elucidate the association between cancer stemness and ICI response. A novel stemness signature (Stem.Sig) was developed and validated using large-scale pan-cancer data, including 34 scRNA-Seq datasets, The Cancer Genome Atlas (TCGA) pan-cancer cohort, and 10 ICI transcriptomic cohorts. The therapeutic value of Stem.Sig genes was further explored using 17 CRISPR datasets that screened potential immunotherapy targets. RESULTS: Cancer stemness, as evaluated by CytoTRACE, was found to be significantly associated with ICI resistance in melanoma and basal cell carcinoma (both P < 0.001). Significantly negative association was found between Stem.Sig and anti-tumor immunity, while positive correlations were detected between Stem.Sig and intra-tumoral heterogenicity (ITH) / total mutational burden (TMB). Based on this signature, machine learning model predicted ICI response with an AUC of 0.71 in both validation and testing set. Remarkably, compared with previous well-established signatures, Stem.Sig achieved better predictive performance across multiple cancers. Moreover, we generated a gene list ranked by the average effect of each gene to enhance tumor immune response after genetic knockout across different CRISPR datasets. Then we matched Stem.Sig to this gene list and found Stem.Sig significantly enriched 3% top-ranked genes from the list (P = 0.03), including EMC3, BECN1, VPS35, PCBP2, VPS29, PSMF1, GCLC, KXD1, SPRR1B, PTMA, YBX1, CYP27B1, NACA, PPP1CA, TCEB2, PIGC, NR0B2, PEX13, SERF2, and ZBTB43, which were potential therapeutic targets. CONCLUSIONS: We revealed a robust link between cancer stemness and immunotherapy resistance and developed a promising signature, Stem.Sig, which showed increased performance in comparison to other signatures regarding ICI response prediction. This signature could serve as a competitive tool for patient selection of immunotherapy. Meanwhile, our study potentially paves the way for overcoming immune resistance by targeting stemness-associated genes.
  
  
  
  
**GCLC**
-
4
of
10
  

### The anti-melanogenic effects of 3-O-ethyl ascorbic acid via Nrf2-mediated α-MSH inhibition in UVA-irradiated keratinocytes and autophagy induction in melanocytes.

4. Free Radic Biol Med. 2021 Sep;173:151-169. doi: 10.1016/j.freeradbiomed.2021.07.030 Epub 2021 Jul 24.
  
  
Chen SJ(1), Hseu YC(2), Gowrisankar YV(3), Chung YT(3), Zhang YZ(4), Way TD(4), Yang HL(5).
  
  
Author information: (1)Institute of Nutrition, College of Health Care, China Medical University, Tai ...
  
  
3-O-ethyl ascorbic acid (EAA) is an ether-derivative of ascorbic acid, known to inhibit tyrosinase activity, and is widely used in skincare formulations. Nevertheless, the molecular mechanisms underlying the EAA's effects are poorly understood. Here, the anti-melanogenic activity of EAA was demonstrated through Nrf2-mediated α-MSH inhibition in UVA-irradiated keratinocytes (HaCaT) and autophagy induction and inhibition of α-MSH-stimulated melanogenesis in melanocytes (B16F10). EAA pretreatment increased the HaCaT cell viability but suppressed ROS-mediated p53/POMC/α-MSH pathways in UVA-irradiated cells. Further, the conditioned medium from EAA-pretreated and UVA-irradiated HaCaT cells suppressed the MITF-CREB-tyrosinase pathways leading to the inhibition of melanin synthesis in B16F10 cells. EAA treatment increased nuclear Nrf2 translocation via the p38, PKC, and ROS pathways leading to HO-1, γ-GCLC, and NQO-1 antioxidant expression in HaCaT cells. However, Nrf2 silencing reduced the EAA-mediated anti-melanogenic activity, evidenced by impaired antioxidant gene expression and uncontrolled ROS (H202) generation following UVA irradiation. In B16F10 cells, EAA-induced autophagy was shown by enhanced LC3-II levels, AVO formation, Beclin-1 upregulation, and activation of p62/SQSTM1. Further, EAA-induced anti-melanogenic activity was substantially decreased in autophagy inhibitor (3-MA) pretreated or LC3 knockdown B16F10 cells. Notably, transmission electron microscopy data showed increased melanosome-engulfing autophagosomes in EAA-treated B16F10 cells. Moreover, EAA also down-regulated MC1R, TRP-1/-2, tyrosinase expressions, and melanin synthesis by suppressing the cAMP-CREB-mediated MITF expression in B16F10 cells stimulated with α-MSH. In vivo studies on the zebrafish model further confirmed that EAA inhibited tyrosinase expression/activity and endogenous pigmentation. In conclusion, 3-O-ethyl ascorbic acid is an effective skin-whitening agent and could be used as a topical agent for cosmetic purposes.
  
  
  
  
**GCLC**
-
5
of
10
  

### The anti-melanogenic effects of ellagic acid through induction of autophagy in melanocytes and suppression of UVA-activated α-MSH pathways via Nrf2 activation in keratinocytes.

5. Biochem Pharmacol. 2021 Mar;185:114454. doi: 10.1016/j.bcp.2021.114454 Epub 2021 Feb 3.
  
  
Yang HL(1), Lin CP(2), Vudhya Gowrisankar Y(2), Huang PJ(3), Chang WL(2), Shrestha S(1), Hseu YC(4).
  
  
Author information: (1)Institute of Nutrition, College of Healthcare, China Medical University, Taic ...
  
  
Ellagic acid (EA) is a natural phenol antioxidant in different fruits, vegetables, and nuts. As a copper iron chelator from the tyrosinase enzyme's active site, EA was reported to inhibit melanogenesis in melanocytes. Here, we demonstrated the anti-melanogenic mechanisms of EA through autophagy induction in melanoma B16F10 cells and the role of Nrf2 and UVA (3 J/cm2)-activated α-melanocyte stimulating hormone (α-MSH) pathways in keratinocyte HaCaT cells. In vitro data showed that EA suppressed the tyrosinase activity and melanogenesis by suppressing cAMP-mediated CREB and MITF signaling mechanisms in α-MSH-stimulated B16F10 cells. ERK, JNK, and AKT pathways were involved in this EA-regulated MITF downregulation. Notably, EA induced autophagy in B16F10 cells was evidenced from increased LC3-II accumulation, p62/SQSTM1 activation, ATG4B downregulation, acidic vesicular organelle (AVO) formation, PI3K/AKT/mTOR inhibition, and Beclin-1/Bcl-2 dysregulation. Interestingly, 3-MA (an autophagy inhibitor) pretreatment or LC3 silencing (siRNA transfection) of B16F10 cells significantly reduced EA-induced anti-melanogenic activity. Besides this, in UVA-irradiated keratinocyte HaCaT cells, EA suppressed ROS production and α-MSH generation. Moreover, EA mediated the activation and nuclear translocation of Nrf2, leading to antioxidant γ-GCLC, HO-1, and NQO-1 protein expression in HaCaT cells. However, Nrf2 knockdown has significantly impaired this effect, and there was an uncontrolled ROS generation following UVA irradiation. JNK, PKC, and ROS pathways were involved in the activation of Nrf2 in HaCaT cells. In vivo experiments using the zebrafish model confirmed that EA inhibited tyrosinase activity and endogenous pigmentation. In conclusion, ellagic acid is an effective skin-whitening agent and might be used as a topical applicant.
  
  
  
  
**GCLC**
-
6
of
10
  

### The Skin-Whitening Effects of Ectoine via the Suppression of α-MSH-Stimulated Melanogenesis and the Activation of Antioxidant Nrf2 Pathways in UVA-Irradiated Keratinocytes.

6. Antioxidants (Basel). 2020 Jan 10;9(1):63. doi: 10.3390/antiox9010063
  
  
Hseu YC(1)(2)(3)(4), Chen XZ(1), Vudhya Gowrisankar Y(1), Yen HR(3)(4)(5)(6), Chuang JY(7), Yang HL( ...
  
  
Author information: (1)Department of Cosmeceutics, College of Biopharmaceutical and Food Sciences, C ...
  
  
Ultraviolet A (UVA)-irradiation induced reactive oxygen species (ROS) production mediates excessive melanogenesis in skin cells leading to pigmentation. We demonstrated the depigmenting and anti-melanogenic effects of Ectoine, a natural bacterial osmolyte, in UVA-irradiated human (HaCaT) keratinocytes, and the underlying molecular mechanisms were elucidated. HaCaT cells were pre-treated with low concentrations of Ectoine (0.5-1.5 μM) and assayed for various depigmenting and anti-melanogenic parameters. This pre-treatment significantly downregulated ROS generation, α-melanocyte-stimulating hormone (α-MSH) production, and proopiomelanocortin (POMC) expression in UVA-irradiated HaCaT cells. Also, antioxidant heme oxygenase-1 (HO-1), NAD(P)H dehydrogenase [quinone 1] (NQO-1), and γ-glutamate-cysteine ligase catalytic subunit (γ-GCLC) protein expressions were mediated via the nuclear translocation of nuclear factor erythroid 2-related factor 2 (Nrf2) whose knockdown indeed impaired this effect signifying the importance of the Nrf2 pathway. Ectoine was mediating the activation of Nrf2 via the p38, protein kinase B (also known as AKT), protein kinase C (PKC), and casein kinase II protein kinase (CKII) pathways. The conditioned medium obtained from the Ectoine pre-treated and UVA-irradiated HaCaT cells downregulated the tyrosinase, tyrosinase-related protein-1 and -2 (TRP-1/-2), cyclic AMP (c-AMP) protein kinase, c-AMP response element-binding protein (CREB), and microphthalmia-associated transcription factor (MITF) expressions leading to melanoma B16F10 cells having inhibited melanin synthesis. Interestingly, this anti-melanogenic effect in α-MSH-stimulated B16F10 cells was observable only at 50-400 μM concentrations of Ectoine, signifying the key role played by Ectoine (0.5-1 μM)-treated keratinocytes in skin whitening effects. We concluded that Ectoine could be used as an effective topical natural cosmetic agent with depigmenting and anti-melanogenic efficacy.
  
  
  
  
**GCLC**
-
7
of
10
  

### The in vitro and in vivo depigmenting activity of Coenzyme Q10 through the down-regulation of α-MSH signaling pathways and induction of Nrf2/ARE-mediated antioxidant genes in UVA-irradiated skin keratinocytes.

7. Biochem Pharmacol. 2019 Jun;164:299-310. doi: 10.1016/j.bcp.2019.04.015 Epub 2019 Apr 13.
  
  
Hseu YC(1), Ho YG(2), Mathew DC(2), Yen HR(3), Chen XZ(2), Yang HL(4).
  
  
Author information: (1)Department of Cosmeceutics, College of Biopharmaceutical and Food Sciences, C ...
  
  
Coenzyme CoQ10 (CoQ10), a ubiquinone compound, has been reported to inhibit tyrosinase activity and melanin production in melanoma B16F10 cells. However, the molecular mechanism underlying this inhibitory effect is poorly understood. In this paper we aimed to investigate the molecular mechanisms involved in the anti-melanogenic activity of CoQ10 (1-2 μM) in UVA (5 J/cm2)-irradiated keratinocyte HaCaT cells and α-MSH stimulated B16-F10 cells. It was observed that CoQ10 suppressed p53/POMC, α-MSH production as well as inhibited ROS generation in UVA-irradiated keratinocyte HaCaT cells. CoQ10 down-regulated the melanin synthesis in α-MSH-stimulated B16-F10 cells by suppressing the MITF expression by down regulating the cAMP mediated CREB signaling cascades. Furthermore, in vivo evidence demonstrated the inhibitory effect of CoQ10 on endogenous pigmentation in zebrafish. Increased nuclear Nrf2 translocation accompanied by the induction of HO-1 and γ-GCLC genes were observed in CoQ10 treated keratinocyte HaCaT cells. Notably, silencing of Nrf2 (siRNA transfection) significantly diminished CoQ10-mediated anti-melanogenic activity, as evidenced by impaired antioxidant HO-1 gene, uncontrolled ROS generation, and α-MSH production following UVA irradiation. To conclude, CoQ10 is an effective de-pigmention or skin-whitening agent and could be used in cosmetics for topical application.
  
  
  
  
**GCLC**
-
8
of
10
  

### Efficient therapeutic delivery by a novel cell-permeant peptide derived from KDM4A protein for antitumor and antifibrosis.

8. Oncotarget. 2016 Aug 2;7(31):49075-49090. doi: 10.18632/oncotarget.8682
  
  
Wang H(1)(2)(3), Ma JL(2), Yang YG(1)(2), Song Y(1)(2)(3), Wu J(1)(2)(3), Qin YY(2)(3), Zhao XL(2)(3 ...
  
  
Author information: (1)The Institute of Cell Therapy, China Three Gorges University, Yichang 443002, ...
  
  
Cell-penetrating peptide (CPP) based delivery have provided immense potential for the therapeutic applications, however, most of nonhuman originated CPPs carry the risk of possible cytotoxicity and immunogenicity, thus may restricting to be used. Here, we describe a novel human-derived CPP, denoted hPP10, and hPP10 has cell-penetrating properties evaluated by CellPPD web server, as well as In-Vitro and In-Vivo analysis. In vitro studies showed that hPP10-FITC was able to penetrate into various cells including primary cultured cells, likely through an endocytosis pathway. And functionalized macromolecules, such as green fluorescent protein (GFP), tumor-specific apoptosis inducer Apoptin as well as biological active enzyme GCLC (Glutamate-cysteine ligase, catalytic subunit) can be delivered by hPP10 in vitro and in vivo. Collectively, our results suggest that hPP10 provide a novel and versatile tool to deliver exogenous proteins or drugs for clinical applications as well as reprogrammed cell-based therapy.
  
  
  
  
**GCLC**
-
9
of
10
  

### Photoprotection by dietary phenolics against melanogenesis induced by UVA through Nrf2-dependent antioxidant responses.

9. Redox Biol. 2016 Aug;8:79-90. doi: 10.1016/j.redox.2015.12.006 Epub 2015 Dec 19.
  
  
Chaiprasongsuk A(1), Onkoksoong T(1), Pluemsamran T(1), Limsaengurai S(1), Panich U(2).
  
  
Author information: (1)Department of Pharmacology, Faculty of Medicine Siriraj Hospital, Mahidol Uni ...
  
  
Dietary phenolics may play a protective role in UV-mediated skin pigmentation through their antioxidant and UV-absorbing actions. In this study, we investigated whether genetic silencing of Nrf2, regulating the transcription of antioxidant genes, affected melanogenesis in primary human epidermal melanocytes (HEMn) and B16F10 melanoma cells subjected to UVA (8J/cm(2)) exposure. Then, we explored the antimelanogenic actions of phenolics; caffeic acid (CA) and ferulic acid (FA) providing partial UVA protection; quercetin (QU) and rutin (RU) providing strong UVA protection and; avobenzone (AV), an efficient UVA filter, in association with modulation of Nrf2-mediated antioxidant defenses in response to UVA insults in B16F10 cells. Upon oxidative insults, Nrf2 silencing promoted melanogenesis in both HEMn and B16F10 cells irradiated with UVA. Stimulation of melanogenesis by UVA correlated with increased ROS and oxidative DNA damage (8-OHdG), GSH depletion as well as a transient downregulation of Nrf2 nuclear translocation and of Nrf2-ARE signaling in B16F10 cells. All test compounds exerted antimelanogenic effects with respect to their abilities to reverse UVA-mediated oxidative damage as well as downregulation of Nrf2 activity and its target antioxidants (GCLC, GST and NQO1) in B16F10 cells. In conclusion, defective Nrf2 may promote melanogenesis under UVA irradiation through oxidative stress mechanisms. Compounds with antioxidant and/or UVA absorption properties could protect against UVA-induced melanogenesis through indirect regulatory effect on Nrf2-ARE pathway.
  
  
  
  
**GCLC**
-
10
of
10
  

### Protective effect of AVS073, a polyherbal formula, against UVA-induced melanogenesis through a redox mechanism involving glutathione-related antioxidant defense.

10. BMC Complement Altern Med. 2013 Jul 5;13:159. doi: 10.1186/1472-6882-13-159
  
  
Panich U(1), Pluemsamran T, Tangsupa-a-nan V, Wattanarangsan J, Phadungrakwittaya R, Akarasereenont ...
  
  
Author information: (1)Department of Pharmacology, Faculty of Medicine Siriraj Hospital, Mahidol Uni ...
  
  
BACKGROUND: Ayurved Siriraj Brand Wattana formula (AVS073), a Thai herbal formula, has traditionally been used for health promotion and prevention of age-related problems. Ultraviolet A (UVA) is recognized to play a vital role in stimulation of melanin synthesis responsible for abnormal skin pigmentation possibly mediated by photooxidative stress. We thus aimed to study the inhibitory effect of AVS073 extracts on UVA-induced melanogenesis via a redox mechanism involving glutathione (GSH) synthesis and glutathione S-transferase (GST) using human melanoma (G361) cell culture. METHODS: The standardization of AVS073 extracts was carried out by TLC and UHPLC to obtain fingerprinting profiles of the formula, which identified several phenolic compounds including gallic acid (GA) in the formula. Antimelanogenic actions of AVS073 (up to 60 μg/ml) and GA (up to 10 μg/ml) were investigated by measuring tyrosinase activity and mRNA as well as melanin level in G361 cells irradiated with UVA. Moreover, antioxidant actions of the herbal formula and GA were determined by evaluating oxidant formation and modulation of GSH-related antioxidant defenses including GSH content, GST activity and mRNA level of γ-glutamate cysteine ligase catalytic (γ-GCLC) and modifier (γ-GCLM) subunit and GST. RESULTS: AVS073 extracts and GA, used as a reference compound, suppressed UVA-augmented tyrosinase activity and mRNA and melanin formation. In addition, pretreatment with AVS073 and GA was able to inhibit cellular oxidative stress, GSH depletion, GST inactivation and downregulation of γ-GCLC, γ-GCLM and GST mRNA in G361 cells exposed to UVA radiation. CONCLUSIONS: AVS073 formula exerted antimelanogenic effects possibly through improving the redox state by upregulation of GSH and GST. Moreover, pharmacological activity of the polyherbal formula would be attributed to combined action of different phenolic compounds present in the formula.
  
  
  
  
  

---

  

# HCLS1

**Signature gene in:** MDM
  
**Differentially expressed in:** MDM
  
  
**HCLS1**
-
1
of
1
  

### Ubiquitination-related biomarkers in metastatic melanoma patients and their roles in tumor microenvironment.

1. Front Oncol. 2023 May 19;13:1170190. doi: 10.3389/fonc.2023.1170190 eCollection 2023.
  
  
Zhang L(1), Shi Z(1), Zhang F(1), Chen B(1), Qiu W(1), Cai L(1), Lin X(1).
  
  
Author information: (1)Department of Dermatology and Venereology, The First Affiliated Hospital of W ...
  
  
BACKGROUND: Skin cutaneous melanoma (SKCM) is the deadliest type of cutaneous malignancy. Ubiquitination is a process of protein sorting and degradation that exhibits multiple functions in the progression of various tumors. This study aimed to characterize a set of genes for ubiquitination in SKCM. METHODS: The expression patterns of ubiquitin-associated genes (URGs) and the corresponding clinical information in SKCM tissues were comprehensively analyzed based on The Cancer Genome Atlas (TCGA) database. We performed univariate and multivariate Cox proportional regression models to characterize the risk scores and identify four critical genes related to prognostic ubiquitination (HCLS1, CORO1A, NCF1 and CCRL2), which were used to construct the prognostic signatures. We also studied the effects of HCLS1, CORO1A and CCRL2 on tumor metastasis-related indicators at the cellular level through in vitro experiments. RESULTS: SKCM patients in the low-risk group showing a longer survival than those in the high-risk group. Characteristic risk scores correlated with several clinicopathological variables and reflected the infiltration of multiple immune cells. In addition, the knockdown of CLS1, CORO1A and CCRL2 affected cellular malignant biological behavior through the EMT signaling pathway. CONCLUSION: This study provides a novel and prospective strategy to improve the clinical survival of SKCM patients.
  
  
  
  
  

---

  

# HIPK2

**Signature gene in:** MDM
  
**Differentially expressed in:** MDM
  
  
**HIPK2**
-
1
of
1
  

### Cisplatin fails to induce puma mediated apoptosis in mucosal melanomas.

1. Oncotarget. 2015;6(12):9887-96. doi: 10.18632/oncotarget.3195
  
  
Fritsche MK(1), Metzler V(1), Becker K(2), Plettenberg C(3), Heiser C(1), Hofauer B(1), Knopf A(1).
  
  
Author information: (1)Technische Universität München, Hals-Nasen-Ohrenklinik und Poliklinik, 81675 ...
  
  
OBJECTIVES: Mucosal melanomas (MM) are aggressive subtypes of common melanomas. It remains unclear whether limitations in their resectability or their distinctive molecular mechanisms are responsible for the aggressive phenotype. METHODS: In total, 112 patients with cutaneous melanomas (CM) and 27 patients with MM were included. Clinical parameters were analysed using Chi square, Fisher exact and student's t-test. Survival rates were calculated by Kaplan-Meier. Analysis of p53, p21, Mdm2, HIPK2, Gadd45, Puma, Bax, Casp9 and Cdk1 via quantitative PCR and immunohistochemistry (IHC) was performed. TP53 induction after cisplatin treatment was analysed in 10 cell lines (melanocytes, four MM and five CM) using western blot (WB) and qPCR. RESULTS: The overall/recurrence-free survival differed significantly between MM (40 months and 30 months) and CM (90 months and 107 months; p < 0.001). IHC and WB confirmed high p53 expression in all melanomas. HIPK2 and Gadd45 showed significantly higher expressions in CM (p < 0.005; p = 0.004). QPCR and WB of wild-type cell lines demonstrated no differences for p53, p21, Mdm2, Bax and Casp9. WB failed to detect Puma in MM, while Cdk1 regulation occurred exclusively in MM. CONCLUSIONS: The aggressive phenotype of MM did not appear to be due to differential expressions of p53, p21, Mdm2, Bax or Casp9. A non-functional apoptosis in MM may have further clinical implications.
  
  
  
  
  

---

  

# IRAK3

**Signature gene in:** MDM
  
**Differentially expressed in:** MDM
  
  
**IRAK3**
-
1
of
3
  

### Age-related transcriptome changes in melanoma patients with tumor-positive sentinel lymph nodes.

1. Aging (Albany NY). 2020 Dec 29;12(24):24914-24939. doi: 10.18632/aging.202435 Epub 2020 Dec 29.
  
  
Menefee DS(1), McMasters A(1), Pan J(2), Li X(3), Xiao D(1), Waigel S(4), Zacharias W(4)(5), Rai SN( ...
  
  
Author information: (1)The Hiram C. Polk, Jr., MD. Department of Surgery, University of Louisville S ...
  
  
Age is an important factor for determining the outcome of melanoma patients. Sentinel lymph node (SLN) status is also a strong predictor of survival for melanoma. Paradoxically, older melanoma patients have a lower incidence of SLN metastasis but a higher mortality rate when compared with their younger counterparts. The mechanisms that underlie this phenomenon remain unknown. This study uses three independent datasets of RNA samples from patients with melanoma metastatic to the SLN to identify age-related transcriptome changes in SLNs and their association with outcome. Microarray was applied to the first dataset of 97 melanoma patients. NanoString was performed in the second dataset to identify the specific immune genes and pathways that are associated with recurrence in younger versus older patients. qRT-PCR analysis was used in the third dataset of 36 samples to validate the differentially expressed genes (DEGs) from microarray and NanoString. These analyses show that FOS, NR4A, and ITGB1 genes were significantly higher in older melanoma patients with positive SLNs. IRAK3- and Wnt10b-related genes are the major pathways associated with recurrent melanoma in younger and older patients with tumor-positive SLNs, respectively. This study aims to elucidate age-related differences in SLNs in the presence of nodal metastasis.
  
  
  
  
**IRAK3**
-
2
of
3
  

### Induction of IRAK-M in melanoma induces caspase-3 dependent apoptosis by reducing TRAF6 and calpastatin levels.

2. Commun Biol. 2020 Jun 12;3(1):306. doi: 10.1038/s42003-020-1033-y
  
  
Geng D(1), Ciavattone N(2), Lasola JJ(2), Shrestha R(3), Sanchez A(4), Guo J(4), Vlk A(2), Younis R( ...
  
  
Author information: (1)Department of Medicine, Division of Medical Oncology, University of Colorado ...
  
  
Melanoma represents the most serious type of skin cancer. Although recent years have seen advances using targeted and immunotherapies, most patients remain at high risk for tumor recurrence. Here we show that IRAK-M, a negative regulator of MyD88 signaling, is deficient or low in melanoma and expression levels correlate with patient survival. Inducing IRAK-M expression using genetic approaches or epigenetic modifiers initiates apoptosis by prompting its interaction with TRAF6 via IRAK-M's C-terminal domain. This complex recruits and degrades calpastatin which stimulates calpain activity and triggers caspase-3-dependent but caspase-8,-9-independent apoptosis. Using a drug screen, we identified compounds that induced IRAK-M expression. Administration of IRAK-M-inducing drugs reduced tumor growth in mice but was ineffective against IRAK-M knock-down tumors. These results uncover a previously uncharacterized apoptosis pathway, emphasize IRAK-M as a potential therapeutic target and suggest that the anticancer activity of certain drugs could do so through their ability to induce IRAK-M expression.
  
  
  
  
**IRAK3**
-
3
of
3
  

### Blood mRNA expression profiling predicts survival in patients treated with tremelimumab.

3. Clin Cancer Res. 2014 Jun 15;20(12):3310-8. doi: 10.1158/1078-0432.CCR-13-2906 Epub 2014 Apr 10.
  
  
Saenger Y(1), Magidson J(2), Liaw B(2), de Moll E(2), Harcharik S(2), Fu Y(2), Wassmann K(2), Fisher ...
  
  
Author information: (1)Authors' Affiliations: Division of Hematology and Oncology, Tisch Cancer Inst ...
  
  
PURPOSE: Tremelimumab (ticilimumab, Pfizer), is a monoclonal antibody (mAb) targeting cytotoxic T lymphocyte-associated antigen-4 (CTLA-4). Ipilimumab (Yervoy, BMS), another anti-CTLA-4 antibody, is approved by the U.S. Federal Drug Administration (FDA). Biomarkers are needed to identify the subset of patients who will achieve tumor control with CTLA-4 blockade. EXPERIMENTAL DESIGN: Pretreatment peripheral blood samples from 218 patients with melanoma who were refractory to prior therapy and receiving tremelimumab in a multicenter phase II study were measured for 169 mRNA transcripts using reverse transcription polymerase chain reaction (RT-PCR). A two-class latent model yielded a risk score based on four genes that were highly predictive of survival (P < 0.001). This signature was validated in an independent population of 260 treatment-naïve patients with melanoma enrolled in a multicenter phase III study of tremelimumab. RESULTS: Median follow-up was 297 days for the training population and 386 days for the test population. Expression levels of the 169 genes were closely correlated across the two populations (r = 0.9939). A four-gene model, including cathepsin D (CTSD), phopholipase A2 group VII (PLA2G7), thioredoxin reductase 1 (TXNRD1), and interleukin 1 receptor-associated kinase 3 (IRAK3), predicted survival in the test population (P = 0.001 by log-rank test). This four-gene model added to the predictive value of clinical predictors (P < 0.0001). CONCLUSIONS: Expression levels of CTSD, PLA2G7, TXNRD1, and IRAK3 in peripheral blood are predictive of survival in patients with melanoma treated with tremelimumab. Blood mRNA signatures should be further explored to define patient subsets likely to benefit from immunotherapy.
  
  
  
  
  

---

  

# ITGA4

**Signature gene in:** Tregs
  
**Differentially expressed in:** Tregs
  
  
**ITGA4**
-
1
of
10
  

### Evaluation of VLA-4 (integrin α4β1) as a shared target for radiopharmaceutical therapy across solid tumors.

1. Mol Cancer Ther. 2025 Feb 6. doi: 10.1158/1535-7163.MCT-24-0370 Online ahead of print.
  
  
Reyes-González JM(1), Rajkumar H(2), Lee W(3), Baidoo KE(4), Edinger RS(5), Diehl G(6), Nambiar D(4) ...
  
  
Author information: (1)National Cancer Institute, Bethesda, United States. (2)University of Pittsbur ...
  
  
Radiopharmaceutical therapy (RPT) is a promising approach to treating solid tumors, but therapeutic advances are impeded by the lack of broadly expressed targets and shared molecular vulnerability across different tumor types. We evaluate VLA-4 (integrin α4β1) as a potential target for RPT in solid tumors using radiolabeled copper-64 ([64Cu]Cu-) and copper-67 ([67Cu]Cu-CB-TE1A1P-PEG4-LLP2A) LLP2A, a peptidomimetic ligand of VLA-4, for preclinical imaging and RPT testing. Expression analysis of ITGA4, encoding the alpha 4 subunit of VLA-4, revealed overexpression in hematological malignancies and various solid tumors compared to healthy tissue. Flow cytometry showed medium to high VLA-4 expression in 77% of tested cancer cell lines. PET/CT imaging with [64Cu]Cu-LLP2A demonstrated tracer uptake in tumors and lymphoid tissues. In toxicity studies, [67Cu]Cu-LLP2A administered at 37-74 MBq was well-tolerated, causing only thymic atrophy without long-term hematological or tissue toxicity. Tumor dose response was observed in B16-F10 melanoma models. Using orthotopic syngeneic models (B16-F10, B78, 4T1, GL261, TH-MYCN, and E2A-PBX1) and human cancer models (SK-MEL-37, 143B, and IMR-5), we conducted PET/CT imaging and biodistribution studies, with selected models used for [64Cu]Cu-LLP2A dosimetry calculations. Given VLA-4's broad expression across cancer tissues, demonstrated on-target effects, acceptable toxicity profile, and favorable dosimetry in preclinical models, further investigation of [67Cu]Cu-LLP2A as an RPT agent is warranted.
  
  
  
  
**ITGA4**
-
2
of
10
  

### A comprehensive identification of potential molecular targets and small drugs candidate for melanoma cancer using bioinformatics and network-based screening approach.

2. J Biomol Struct Dyn. 2024 Sep;42(14):7349-7369. doi: 10.1080/07391102.2023.2240409 Epub 2023 Aug 3.
  
  
Khan DA(1), Adhikary T(2), Sultana MT(2), Toukir IA(3).
  
  
Author information: (1)Department of Genetic Engineering and Biotechnology, Jashore University of Sc ...
  
  
Melanoma is the third most common malignant skin tumor and has increased in morbidity and mortality over the previous decade due to its rapid spread into the bloodstream or lymphatic system. This study used integrated bioinformatics and network-based methodologies to reliably identify molecular targets and small molecular medicines that may be more successful for Melanoma diagnosis, prognosis and treatment. The statistical LIMMA approach utilized for bioinformatics analysis in this study found 246 common differentially expressed genes (cDEGs) between case and control samples from two microarray gene-expression datasets (GSE130244 and GSE15605). Protein-protein interaction network study revealed 15 cDEGs (PTK2, STAT1, PNO1, CXCR4, WASL, FN1, RUNX2, SOCS3, ITGA4, GNG2, CDK6, BRAF, AGO2, GTF2H1 and AR) to be critical in the development of melanoma (KGs). According to regulatory network analysis, the most important transcriptional and post-transcriptional regulators of DEGs and hub-DEGs are ten transcription factors and three miRNAs. We discovered the pathogenetic mechanisms of MC by studying DEGs' biological processes, molecular function, cellular components and KEGG pathways. We used molecular docking and dynamics modeling to select the four most expressed genes responsible for melanoma malignancy to identify therapeutic candidates. Then, utilizing the Connectivity Map (CMap) database, we analyzed the top 4-hub-DEGs-guided repurposable drugs. We validated four melanoma cancer drugs (Fisetin, Epicatechin Gallate, 1237586-97-8 and PF 431396) using molecular dynamics simulation with their target proteins. As a result, the results of this study may provide resources to researchers and medical professionals for the wet-lab validation of MC diagnosis, prognosis and treatments.Communicated by Ramaswamy H. Sarma.
  
  
  
  
**ITGA4**
-
3
of
10
  

### Defining the altered glycoproteomic space of the early secretory pathway by class I mannosidase pharmacological inhibition.

3. Front Mol Biosci. 2023 Jan 9;9:1064868. doi: 10.3389/fmolb.2022.1064868 eCollection 2022.
  
  
Munteanu CVA(1), Chirițoiu GN(2), Petrescu AJ(1), Petrescu ȘM(2).
  
  
Author information: (1)Department of Bioinformatics and Structural Biochemistry, Institute of Bioche ...
  
  
N-glycosylation is a key process for various biological functions like protein folding, maturation and sorting for the conventional secretory compartment, cell-cell communication and immune response. This is usually accomplished by a complex system of mannosidases in which those from class I have an outstanding role, commonly involved in the early protein sorting associated to the Endoplasmic Reticulum (ER) in the N-glycan dependent quality control (ERQC) and ER-associated degradation (ERAD). Although these are vital processes in maintaining cellular homeostasis, large-scale analysis studies for this pool of molecules, further denoted as proteins from the early secretory pathway (ESP), were limited addressed. Here, using a custom workflow employing a combination of glycomics and deglycoproteomics analyses, using lectin affinity and selective Endoglycosidase H (Endo H) digestion, we scrutinize the steady-state oligomannosidic glycoprotein load and delineate ESP fraction in melanoma cells. All of these were assessed by applying our workflow for glycosite relative quantification of both the peptide chain and carbohydrate structure in cells with inhibited activity of class I mannosidases after kifunensine treatment. We found that most of the ESP are transient clients involved in cell communication via extracellular matrix, particularly integrin-mediated communication which adopt Man9 N-glycans in kifunensine-treated cells. Moreover, our results reveal that core-fucosylation is decreased subsequent inhibition of class I mannosidases and this could be explained by a general lower protein level of FUT8, the enzyme responsible for fucosylation. By comparing our data with results obtained following downregulation of a key mannosidase in misfolded protein degradation, we mapped both novel and previously suggested endogenous substrate candidates like PCDH2, HLA-B, LAMB2 or members of the integrin family of proteins such as ITGA1 and ITGA4, thus validating the findings obtained using our workflow regarding accumulation and characterization of ESP transitory members following mannosidase class I inhibition. This workflow and the associated dataset not only allowed us to investigate the oligomannosidic glycoprotein fraction but also to delineate differences mediated at glycosite-level upon kifunensine treatment and outline the potential associated cellular responses.
  
  
  
  
**ITGA4**
-
4
of
10
  

### A strategy for uncovering germline variants altering anti-tumor CD8 T cell response.

4. J Genet Genomics. 2023 May;50(5):353-361. doi: 10.1016/j.jgg.2023.01.001 Epub 2023 Jan 20.
  
  
Ulaganathan VK(1), Vasileva MH(2).
  
  
Author information: (1)Klinik für Dermatologie & Allergology, Universitätsmedizin Göttingen, Götting ...
  
  
Among many factors known to alter the outcomes of T cell receptor (TCR)-induced proximal signaling, the role of human germline variants in dictating the individuality of the anti-tumor CD8 T cell response has remained challenging to address. Here, we describe a convenient strategy for molecular and functional characterization of phosphotyrosine-altering non-synonymous single nucleotide variations (pTyr-SNVs) that directly impact TCR-induced proximal phosphotyrosine motif-based signaling pathways. We devise an experimental co-cultivation set-up comprising a C57BL/6 mouse-derived metastatic melanoma cell line engineered to constitutively present ovalbumin (OVA) antigens and retrovirally engineered syngeneic major histocompatibility complex (MHC) Class I restricted OVA TCR-transgenic CD8 T cells (OT-I). Using the synthetic version of pTyr-SNV rs1178800678-G/T-encoding integrin alpha 4 (ITGA4) p.S1027I variant as a prototype, we show that under identical TCR stimulation conditions, genetically determined membrane-proximal immunoreceptor tyrosin activation motif (ITAM) results in increased tyrosine phosphorylation of 70 kDa zeta-chain-associated protein (ZAP70) and the levels of cytotoxic effector molecule granzyme B (GZMB), which in turn result in enhanced cytotoxic activity against metastatic melanoma cell line. This strategy paves the way for rapid molecular and functional characterization of anti-tumor immune response-linked germline pTyr-SNVs so as to improve our understanding of the genetic basis of individual-to-individual differences in anti-tumor CD8 T cell response.
  
  
  
  
**ITGA4**
-
5
of
10
  

### Identification of Therapeutic Targets and Prognostic Biomarkers Among Integrin Subunits in the Skin Cutaneous Melanoma Microenvironment.

5. Front Oncol. 2021 Sep 30;11:751875. doi: 10.3389/fonc.2021.751875 eCollection 2021.
  
  
Nurzat Y(1), Su W(1), Min P(1), Li K(1), Xu H(1), Zhang Y(1).
  
  
Author information: (1)Department of Plastic and Reconstructive Surgery, Shanghai Ninth People's Hos ...
  
  
The roles of different integrin alpha/beta (ITGA/ITGB) subunits in skin cutaneous melanoma (SKCM) and their underlying mechanisms of action remain unclear. Oncomine, UALCAN, GEPIA, STRING, GeneMANIA, cBioPortal, TIMER, TRRUST, and Webgestalt analysis tools were used. The expression levels of ITGA3, ITGA4, ITGA6, ITGA10, ITGB1, ITGB2, ITGB3, ITGB4, and ITGB7 were significantly increased in SKCM tissues. The expression levels of ITGA1, ITGA4, ITGA5, ITGA8, ITGA9, ITGA10, ITGB1, ITGB2, ITGB3, ITGB5, ITGB6 and ITGB7 were closely associated with SKCM metastasis. The expression levels of ITGA1, ITGA4, ITGB1, ITGB2, ITGB6, and ITGB7 were closely associated with the pathological stage of SKCM. The expression levels of ITGA6 and ITGB7 were closely associated with disease-free survival time in SKCM, and the expression levels of ITGA6, ITGA10, ITGB2, ITGB3, ITGB6, ITGB7, and ITGB8 were markedly associated with overall survival in SKCM. We also found significant correlations between the expression of integrin subunits and the infiltration of six types of immune cells (B cells, CD8+ T cells, CD4+T cells, macrophages, neutrophils, and dendritic cells). Finally, Gene Ontology (GO) enrichment analysis and Kyoto Encyclopedia of Genes and Genomes (KEGG) pathway analysis were performed, and protein-protein interaction (PPI) networks were constructed. We have identified abnormally-expressed genes and gene regulatory networks associated with SKCM, improving understanding of the underlying pathogenesis of SKCM.
  
  
  
  
**ITGA4**
-
6
of
10
  

### Blockade of MCAM/CD146 impedes CNS infiltration of T cells over the choroid plexus.

6. J Neuroinflammation. 2018 Aug 22;15(1):236. doi: 10.1186/s12974-018-1276-4
  
  
Breuer J(1), Korpos E(2)(3), Hannocks MJ(2)(3), Schneider-Hohendorf T(1), Song J(2)(3), Zondler L(4) ...
  
  
Author information: (1)Clinic of Neurology with Institute of Translational Neurology, University of ...
  
  
BACKGROUND: Very late antigen 4 (VLA-4; integrin α4β1) is critical for transmigration of T helper (TH) 1 cells into the central nervous system (CNS) under inflammatory conditions such as multiple sclerosis (MS). We have previously shown that VLA-4 and melanoma cell adhesion molecule (MCAM) are important for trans-endothelial migration of human TH17 cells in vitro and here investigate their contribution to pathogenic CNS inflammation. METHODS: Antibody blockade of VLA-4 and MCAM is assessed in murine models of CNS inflammation in conjunction with conditional ablation of α4-integrin expression in T cells. Effects of VLA-4 and MCAM blockade on lymphocyte migration are further investigated in the human system via in vitro T cell transmigration assays. RESULTS: Compared to the broad effects of VLA-4 blockade on encephalitogenic T cell migration over endothelial barriers, MCAM blockade impeded encephalitogenic T cell migration in murine models of MS that especially depend on CNS migration across the choroid plexus (CP). In transgenic mice lacking T cell α4-integrin expression (CD4::Itga4-/-), MCAM blockade delayed disease onset. Migration of MCAM-expressing T cells through the CP into the CNS was restricted, where laminin 411 (composed of α4, β1, γ1 chains), the proposed major ligand of MCAM, is detected in the endothelial basement membranes of murine CP tissue. This finding was translated to the human system; blockade of MCAM with a therapeutic antibody reduced in vitro transmigration of MCAM-expressing T cells across a human fibroblast-derived extracellular matrix layer and a brain-derived endothelial monolayer, both expressing laminin α4. Laminin α4 was further detected in situ in CP endothelial-basement membranes in MS patients' brain tissue. CONCLUSIONS: Our findings suggest that MCAM-laminin 411 interactions facilitate trans-endothelial migration of MCAM-expressing T cells into the CNS, which seems to be highly relevant to migration via the CP and to potential future clinical applications in neuroinflammatory disorders.
  
  
  
  
**ITGA4**
-
7
of
10
  

### Nanoscale Tuning of VCAM-1 Determines VLA-4-Dependent Melanoma Cell Plasticity on RGD Motifs.

7. Mol Cancer Res. 2018 Mar;16(3):528-542. doi: 10.1158/1541-7786.MCR-17-0272 Epub 2017 Dec 8.
  
  
Amschler K(#)(1), Kossmann E(#)(1), Erpenbeck L(1), Kruss S(2), Schill T(1), Schön M(1), Möckel SMC( ...
  
  
Author information: (1)Department of Dermatology, Venereology and Allergology, University Medical Ce ...
  
  
The biophysical fine-tuning of cancer cell plasticity is crucial for tumor progression but remains largely enigmatic. Although vascular cell adhesion molecule-1 (VCAM-1/CD106) has been implicated in melanoma progression, here its presentation on endothelial cells was associated with diminished melanoma cell spreading. Using a specific nanoscale modulation of VCAM-1 (tunable from 70 to 670 ligands/μm²) next to integrin ligands (RGD motifs) in a bifunctional system, reciprocal regulation of integrin α4 (ITGA4/VLA-4/CD49D)-dependent adhesion and spreading of melanoma cells was found. As the VCAM-1/VLA-4 receptor pair facilitated adhesion, while at the same time antagonizing RGD-mediated spreading, melanoma cell morphogenesis on these bifunctional matrices was directly regulated by VCAM-1 in a dichotomic and density-dependent fashion. This was accompanied by concordant regulation of F-actin cytoskeleton remodeling, Rac1-expression, and paxillin-related adhesion formation. The novel function of VCAM-1 was corroborated in vivo using two murine models of pulmonary metastasis. The regulation of melanoma cell plasticity by VCAM-1 highlights the complex regulation of tumor-matrix interactions.Implications: Nanotechnology has revealed a novel dichotomic function of the VCAM-1/VLA-4 interaction on melanoma cell plasticity, as nanoscale tuning of this interaction reciprocally determines adhesion and spreading in a ligand density-dependent manner. Mol Cancer Res; 16(3); 528-42. ©2017 AACR.
  
  
  
  
**ITGA4**
-
8
of
10
  

### Whole-blood RNA transcript-based models can predict clinical response in two large independent clinical studies of patients with advanced melanoma treated with the checkpoint inhibitor, tremelimumab.

8. J Immunother Cancer. 2017 Aug 15;5(1):67. doi: 10.1186/s40425-017-0272-z
  
  
Friedlander P(1), Wassmann K(2), Christenfeld AM(2), Fisher D(3), Kyi C(4), Kirkwood JM(5), Bhardwaj ...
  
  
Author information: (1)Division of Hematology and Medical Oncology, Tisch Cancer Institute, Icahn Sc ...
  
  
BACKGROUND: Tremelimumab is an antibody that blocks CTLA-4 and demonstrates clinical efficacy in a subset of advanced melanoma patients. An unmet clinical need exists for blood-based response-predictive gene signatures to facilitate clinically effective and cost-efficient use of such immunotherapeutic interventions. METHODS: Peripheral blood samples were collected in PAXgene® tubes from 210 treatment-naïve melanoma patients receiving tremelimumab in a worldwide, multicenter phase III study (discovery dataset). A central panel of radiologists determined objective response using RECIST criteria. Gene expression for 169 mRNA transcripts was measured using quantitative PCR. A 15-gene pre-treatment response-predictive classifier model was identified. An independent population (N = 150) of refractory melanoma patients receiving tremelimumab after chemotherapy enrolled in a worldwide phase II study (validation dataset). The classifier model, using the same genes, coefficients and constants for objective response and one-year survival after treatment, was applied to the validation dataset. RESULTS: A 15-gene pre-treatment classifier model (containing ADAM17, CDK2, CDKN2A, DPP4, ERBB2, HLA-DRA, ICOS, ITGA4, LARGE, MYC, NAB2, NRAS, RHOC, TGFB1, and TIMP1) achieved an area under the curve (AUC) of 0.86 (95% confidence interval 0.81 to 0.91, p < 0.0001) for objective response and 0.6 (95% confidence interval 0.54 to 0.67, p = 0.0066) for one-year survival in the discovery set. This model was validated in the validation set with AUCs of 0.62 (95% confidence interval 0.54 to 0.70 p = 0.0455) for objective response and 0.68 for one-year survival (95% confidence interval 0.59 to 0.75 p = 0.0002). CONCLUSIONS: To our knowledge, this is the largest blood-based biomarker study of a checkpoint inhibitor, tremelimumab, which demonstrates a validated pre-treatment mRNA classifier model that predicts clinical response. The data suggest that the model captures a biological signature representative of genes needed for a robust anti-cancer immune response. It also identifies non-responders to tremelimumab at baseline prior to treatment.
  
  
  
  
**ITGA4**
-
9
of
10
  

### Oncofetal Chondroitin Sulfate Glycosaminoglycans Are Key Players in Integrin Signaling and Tumor Cell Motility.

9. Mol Cancer Res. 2016 Dec;14(12):1288-1299. doi: 10.1158/1541-7786.MCR-16-0103 Epub 2016 Sep 21.
  
  
Clausen TM(1)(2)(3)(4), Pereira MA(5), Al Nakouzi N(2)(3), Oo HZ(2)(3)(6), Agerbæk MØ(5)(2)(3), Lee ...
  
  
Author information: (1)Centre for Medical Parasitology at Department of International Health, Immuno ...
  
  
Many tumors express proteoglycans modified with oncofetal chondroitin sulfate glycosaminoglycan chains (ofCS), which are normally restricted to the placenta. However, the role of ofCS in cancer is largely unknown. The function of ofCS in cancer was analyzed using the recombinant ofCS-binding VAR2CSA protein (rVAR2) derived from the malaria parasite, Plasmodium falciparum We demonstrate that ofCS plays a key role in tumor cell motility by affecting canonical integrin signaling pathways. Binding of rVAR2 to tumor cells inhibited the interaction of cells with extracellular matrix (ECM) components, which correlated with decreased phosphorylation of Src kinase. Moreover, rVAR2 binding decreased migration, invasion, and anchorage-independent growth of tumor cells in vitro Mass spectrometry of ofCS-modified proteoglycan complexes affinity purified from tumor cell lines on rVAR2 columns revealed an overrepresentation of proteins involved in cell motility and integrin signaling, such as integrin-β1 (ITGB1) and integrin-α4 (ITGA4). Saturating concentrations of rVAR2 inhibited downstream integrin signaling, which was mimicked by knockdown of the core chondroitin sulfate synthesis enzymes β-1,3-glucuronyltransferase 1 (B3GAT1) and chondroitin sulfate N-acetylgalactosaminyltransferase 1 (CSGALNACT1). The ofCS modification was highly expressed in both human and murine metastatic lesions in situ and preincubation or early intravenous treatment of tumor cells with rVAR2 inhibited seeding and spreading of tumor cells in mice. This was associated with a significant increase in survival of the animals. These data functionally link ofCS modifications with cancer cell motility and further highlights ofCS as a novel therapeutic cancer target. IMPLICATIONS: The cancer-specific expression of ofCS aids in metastatic phenotypes and is a candidate target for therapy. Mol Cancer Res; 14(12); 1288-99. ©2016 AACR.
  
  
  
  
**ITGA4**
-
10
of
10
  

### Gene expression profile of B 16(F10) murine melanoma cells exposed to hypoxic conditions in vitro.

10. Gene Expr. 2006;13(3):191-203. doi: 10.3727/000000006783991818
  
  
Olbryt M(1), Jarzab M, Jazowiecka-Rakus J, Simek K, Szala S, Sochanik A.
  
  
Author information: (1)Department of Tumor Biology, Maria Skłodowska-Curie Memorial Cancer Center an ...
  
  
Hypoxia is an important feature of tumor microenvironment, exerting far-reaching effects on cells and contributing to cancer progression. Previous studies have established substantial differences in hypoxia response between various cell lines. Investigating this phenomenon in melanoma cells contributes to a better understanding of cell lineage-specific hypoxia response and could point out novel hypoxia-regulated genes. We investigated transcriptional activity of B 16(F10) murine melanoma cells cultured for 24 h under hypoxic (nominal 1% O2, 15 samples including controls) and hypoxia-mimicking conditions (cobalt chloride, 100 or 200 microM, 6 samples including controls). Gene expression profiles were analyzed using MG-U74Av2 oligonucleotide microarrays. Data analysis revealed 2541 probesets (FDR <5%) for 1% oxygen experiment and 364 probesets (FDR <5%) for cobalt chloride, which showed differences in expression levels. Analysis of hypoxia-regulated genes (true hypoxia, 1% O2) by stringent Family-Wise Error Rate estimation indicated 454 significantly changed transcripts (p < 0.05). The most upregulated genes were Lgals3, Selenbpl, Nppb (more than ten-fold increase). We observed significant differences in expression levels of genes regulating glycolysis (Pfkp, Hk2, Aldo3, Eno2), apoptosis (Bnip3, Bnip31, Cdknla), transcription (Bhlhb2, Sap30, Atf3, Mxil), angiogenesis (Vegfa, Adm, Anxa2, Ctgf), adhesion (Pkp2, ITGA4, Mcam), migration (Cnn2, Tmsb4x), and other processes. Both true hypoxia and hypoxia mimicry induced HIF-1-regulated genes. However, unsupervised analysis (Singular Value Decomposition) revealed distinct differences in gene expression between these two experimental conditions. Contrary to hypoxia, cobalt chloride caused suppression of gene expression rather than stimulation, especially concerning transcripts related to proliferation, immune response, DNA repair, and melanin biosynthesis.
  
  
  
  
  

---

  

# ITPRIP

**Signature gene in:** Stromal cells
  
**Differentially expressed in:** Stromal cells
  
  
**ITPRIP**
-
1
of
1
  

### Long Noncoding RNA ITPRIP-1 Positively Regulates the Innate Immune Response through Promotion of Oligomerization and Activation of MDA5.

1. J Virol. 2018 Aug 16;92(17):e00507-18. doi: 10.1128/JVI.00507-18 Print 2018 Sep 1.
  
  
Xie Q(#)(1), Chen S(#)(1), Tian R(1), Huang X(1), Deng R(1), Xue B(1), Qin Y(1), Xu Y(1), Wang J(1), ...
  
  
Author information: (1)Institute of Pathogen Biology and Immunology of College of Biology, State Key ...
  
  
Emerging evidence indicates that long noncoding RNAs (lncRNAs) regulate various biological processes, especially innate and adaptive immunity. However, the relationship between lncRNAs and the interferon (IFN) pathway remains largely unknown. Here, we report that lncRNA ITPRIP-1 (lncITPRIP-1) is involved in viral infection and plays a crucial role in the virus-triggered IFN signaling pathway through the targeting of melanoma differentiation-associated gene 5 (MDA5). LncITPRIP-1 can be induced by viral infection, which is not entirely dependent on the IFN signal. Besides, there is no coding potential found in the lncITPRIP-1 transcript. LncITPRIP-1 binds to the C terminus of MDA5, and it possesses the ability to boost the oligomerization of both the full length and the 2 caspase activation and recruitment domains of MDA5 in a K63-linked polyubiquitination-independent manner. Amazingly, we also found that MDA5 can suppress hepatitis C virus (HCV) replication independently of IFN signaling through its C-terminal-deficient domain bound to viral RNA, in which lncITPRIP-1 plays a role as an assistant. In addition, the expression of lncITPRIP-1 is highly consistent with MDA5 expression, indicating that lncITPRIP-1 may function as a cofactor of MDA5. All the data suggest that lncITPRIP-1 enhances the innate immune response to viral infection through the promotion of oligomerization and activation of MDA5. Our study discovers the first lncRNA ITPRIP-1 involved in MDA5 activation.IMPORTANCE Hepatitis C virus infection is a global health issue, and there is still no available vaccine, which makes it urgent to reveal the underlying mechanisms of HCV and host factors. Although RIG-I has been recognized as the leading cytoplasmic sensor against HCV for a long time, recent findings that MDA5 regulates the IFN response to HCV have emerged. Our work validates the significant role of MDA5 in IFN signaling and HCV infection and proposes the first lncRNA inhibiting HCV replication by promoting the activation of MDA5 and mediating the association between MDA5 and HCV RNA, the study of which may shed light on the MDA5 function and treatment for hepatitis C patients. Our suggested model of how lncITPRIP-1 orchestrates signal transduction for IFN production illustrates the essential role of lncRNAs in virus elimination.
  
  
  
  
  

---

  

# KLF2

**Signature gene in:** Endothelial cells
  
**Differentially expressed in:** Endothelial cells
  
  
**KLF2**
-
1
of
5
  

### Peripheral-derived regulatory T cells contribute to tumor-mediated immune suppression in a nonredundant manner.

1. Proc Natl Acad Sci U S A. 2024 Sep 3;121(36):e2404916121. doi: 10.1073/pnas.2404916121 Epub 2024 Aug 29.
  
  
Hossain MM(1), King P(1), Hackett J(2), Gerard HC(1), Niwinski R(1), Wu L(3), Van Kaer L(3), Dyson G ...
  
  
Author information: (1)Department of Biochemistry, Microbiology and Immunology, Wayne State Universi ...
  
  
Identifying tumor-mediated mechanisms that impair immunity is instrumental for the design of new cancer therapies. Regulatory T cells (Tregs) are a key component of cancer-derived immune suppression; however, these lymphocytes are necessary to prevent systemic autoimmunity in mice and humans, and thus, direct targeting of Tregs is not a clinical option for cancer patients. We have previously demonstrated that excising transcription factor Kruppel-like factor 2 (KLF2) within the T cell lineage blocks the generation of peripheral-derived Tregs (pTregs) without impairing production of thymic-derived Tregs. Using this mouse model, we have now demonstrated that eliminating pTregs is sufficient to delay/prevent tumor malignancy without causing autoimmunity. Cancer-bearing mice that expressed KLF2 converted tumor-specific CD4+ T cells into pTregs, which accumulated in secondary lymphoid organs and impaired further T cell effector activity. In contrast, pTreg-deficient mice retained cancer-specific immunity, including improved T cell infiltration into "cold" tumors, reduced T cell exhaustion in tumor beds, restricted generation of tumor-associated myeloid-derived suppressor cells, and the continued production of circulating effector T cells that arose in a cancer-dependent manner. Results indicate that tumor-specific pTregs are critical for early stages of cancer progression and blocking the generation of these inhibitory lymphocytes safely delays/prevents malignancy in preclinical models of melanoma and prostate cancer.
  
  
  
  
**KLF2**
-
2
of
5
  

### Longitudinal single-cell analysis of a patient receiving adoptive cell therapy reveals potential mechanisms of treatment failure.

2. Mol Cancer. 2022 Dec 14;21(1):219. doi: 10.1186/s12943-022-01688-5
  
  
Qu R(1), Kluger Y(1), Yang J(1), Zhao J(1), Hafler DA(2), Krause DS(3), Bersenev A(3), Bosenberg M(4 ...
  
  
Author information: (1)Department of Pathology, Yale School of Medicine, 333 Cedar St, New Haven, CT ...
  
  
Adoptive cell therapy (ACT) using tumor infiltrating lymphocytes (TIL) is being studied in multiple tumor types. However, little is known about clonal cell expansion in vitro and persistence of the ACT product in vivo. We performed single-cell RNA and T-Cell Receptor (TCR) sequencing on serial blood and tumor samples from a patient undergoing ACT, who did not respond. We found that clonal expansion varied during preparation of the ACT product, and only one expanded clone was preserved in the ACT product. The TCR of the preserved clone which persisted and remained activated for five months was previously reported as specific for cytomegalovirus and had upregulation of granzyme family genes and genes associated with effector functions (HLA-DQB1, LAT, HLA-DQA1, and KLRD1). Clones that contracted during TIL preparation had features of exhaustion and apoptosis. At disease progression, all previously detected clonotypes were detected. New clonotypes appearing in blood or tumor at disease progression were enriched for genes associated with cytotoxicity or stemness (FGFBP2, GNLY, GZMH, GZMK, IL7R, SELL and KLF2), and these might be harnessed for alternative cellular therapy or cytokine therapy. In-depth single-cell analyses of serial samples from additional ACT-treated patients is warranted, and viral- versus tumor-specificity should be carefully analyzed.
  
  
  
  
**KLF2**
-
3
of
5
  

### Efficient Suppression of NRAS-Driven Melanoma by Co-Inhibition of ERK1/2 and ERK5 MAPK Pathways.

3. J Invest Dermatol. 2020 Dec;140(12):2455-2465.e10. doi: 10.1016/j.jid.2020.03.972 Epub 2020 May 4.
  
  
Adam C(1), Fusi L(1), Weiss N(1), Goller SG(1), Meder K(1), Frings VG(1), Kneitz H(1), Goebeler M(1) ...
  
  
Author information: (1)Department of Dermatology, Venereology and Allergology, University Hospital W ...
  
  
Cutaneous melanoma is a highly malignant tumor typically driven by somatic mutation in the oncogenes BRAF or NRAS, leading to uncontrolled activation of the MEK/ERK MAPK pathway. Despite the availability of immunotherapy, MAPK pathway‒targeting regimens are still a valuable treatment option for BRAF-mutant melanoma. Unfortunately, patients with NRAS mutation do not benefit from such therapies owing to the lack of targetable BRAF mutations and a high degree of intrinsic and acquired resistance toward MEK inhibition. Here, we demonstrate that concomitant inhibition of ERK5 removes this constraint and effectively sensitizes NRAS-mutant melanoma cells for MAPK pathway‒targeting therapy. Using approved MEK inhibitors or a pharmacologic ERK inhibitor, we demonstrate that MAPK inhibition triggers a delayed activation of ERK5 through a PDGFR inhibitor-sensitive pathway in NRAS-mutant melanoma cells, resulting in sustained proliferation and survival. ERK5 phosphorylation also occurred naturally in NRAS-mutant melanoma cells and correlated with nuclear localization of its stem cell-associated effector KLF2. Importantly, MEK/ERK5 co-inhibition prevented long-term growth of human NRAS-mutant melanoma cells in vitro and effectively repressed tumor progression in a xenotransplant mouse model. Our findings suggest MEK/ERK5 cotargeting as a potential treatment option for NRAS-mutant melanoma, which currently is not amenable for targeted therapies.
  
  
  
  
**KLF2**
-
4
of
5
  

### [New molecular pathological strategies for malignant iris tumors].

4. Ophthalmologe. 2019 Apr;116(4):324-331. doi: 10.1007/s00347-018-0840-8
  
  
[Article in German]
  
  
Kakkassery V(1), Jünemann AM(2), Scheef BO(2), Grisanti S(3), Heindl LM(4)(5).
  
  
Author information: (1)Klinik für Augenheilkunde, Universitätsklinikum Schleswig-Holstein, Campus Lübeck, Universität zu Lübeck, Ratzeburger Allee 160, 23538, Lübeck, Deutschland. vinodh.kakkassery@gmail.com. (2)Klinik und Poliklinik für Augenheilkunde, Universitätsmedizin Rostock, Rostock, Deutschland. (3)Klinik für Augenheilkunde, Universitätsklinikum Schleswig-Holstein, Campus Lübeck, Universität zu Lübeck, Ratzeburger Allee 160, 23538, Lübeck, Deutschland. (4)Zentrum für Augenheilkunde, Universitätsklinikum zu Köln, Köln, Deutschland. (5)Centrum für Integrierte Onkologie (CIO) Köln-Bonn, Köln-Bonn, Deutschland.
  
  
  
  
**KLF2**
-
5
of
5
  

### A MicroRNA302-367-Erk1/2-Klf2-S1pr1 Pathway Prevents Tumor Growth via Restricting Angiogenesis and Improving Vascular Stability.

5. Circ Res. 2017 Jan 6;120(1):85-98. doi: 10.1161/CIRCRESAHA.116.309757 Epub 2016 Oct 18.
  
  
Pi J(1), Tao T(1), Zhuang T(1), Sun H(1), Chen X(1), Liu J(1), Cheng Y(1), Yu Z(1), Zhu HH(1), Gao W ...
  
  
Author information: (1)From the Key Laboratory of Arrhythmias of the Ministry of Education of China, ...
  
  
RATIONALE: Angiogenic hypersprouting and leaky vessels are essential for tumor growth. MicroRNAs have unique therapeutic advantages by targeting multiple pathways of tumor-associated angiogenesis, but the function of individual miRNAs of miR302-367 cluster in angiogenesis and tumors has not yet been fully evaluated. OBJECTIVE: To investigate the functions of miR302-367 in developmental angiogenesis and tumor angiogenesis and explore the molecular mechanisms of microRNA for the treatment of pathological neovascularization-related diseases. METHODS AND RESULTS: Here, we show that miR302-367 elevation in endothelial cells reduces retinal sprouting angiogenesis and promotes vascular stability in vivo, ex vivo, and in vitro. Erk1/2 is identified as direct target of miR302-367, and downregulation of Erk1/2 on miR302-367 elevation in endothelial cells increases the expression of KLF2 and in turn S1pr1 and its downstream target VE-cadherin, suppressing angiogenesis and improving vascular stability. Conversely, both pharmacological blockade and genetic deletion of S1pr1 in endothelial cells reverse the antiangiogenic and vascular stabilizing effect of miR302-367 in mice. Tumor angiogenesis shares features of developmental angiogenesis, and endothelial specific elevation of miR302-367 reduces tumor growth by restricting sprout angiogenesis and decreasing vascular permeability via the same Erk1/2-Klf2-S1pr1 pathways. CONCLUSIONS: MiR302-367 regulation of an Erk1/2-Klf2-S1pr1 pathway in the endothelium advances our understanding of angiogenesis, meanwhile also provides opportunities for therapeutic intervention of tumor growth.
  
  
  
  
  

---

  

# MARCH3

**Signature gene in:** Endothelial cells
  
**Differentially expressed in:** Endothelial cells
  
  
**MARCH3**
-
1
of
1
  

### 3rd International Conference on Melanoma. Venice, Italy, 31 March-3 April 1993. Abstracts.

1. Melanoma Res. 1993 Mar;3 Suppl 1:1-112.
  
  
[No authors listed]
  
  
PMID: 8096413 [Indexed for MEDLINE]
  
  
NA
  
  
  
  
  

---

  

# MEG3

**Signature gene in:** Stromal cells
  
**Differentially expressed in:** Stromal cells
  
  
**MEG3**
-
1
of
10
  

### Novel Biological Strategies for Melanoma Therapy: A Focus on lncRNAs and Their Targeting.

1. Cancers (Basel). 2025 Apr 9;17(8):1273. doi: 10.3390/cancers17081273
  
  
Orlandella FM(1)(2), Arcone R(1), Luciano N(1)(2), Salvatore G(1)(2), Motti ML(1).
  
  
Author information: (1)Department of Medical, Human Movement and Well-Being Sciences, University of ...
  
  
Increasing evidence revealed that restoring the correct expression of lncRNAs could have implications in the management of melanoma patients. In this context, here, we aim to dissect the main characteristics of lncRNAs altered in melanoma and their crosstalk with the signaling pathways involved in the progression of this disease. We also highlight the role of nucleic acid-based techniques and natural compounds (i.e., phytochemicals) as a therapeutic tool to increase or silence their expression in cancer cells. Finally, we explore the advances in nanotechnologies as delivery systems to efficiently carry these chemicals into cancer cells, thus limiting their potential off-target effects. The analysis of the literature showed that HOTAIR, MALAT1, and H19 are the oncogenic lncRNAs most studied in melanoma, while MEG3 is an important tumor suppressor decreased in this cancer. The aberrant expression of these lncRNAs affects several hallmarks of cancer, e.g., proliferation, motility, and epithelial to mesenchymal transition, promoting the melanoma plasticity and drug resistance. In this frame, siRNA, antisense oligonucleotide, and CRISPR-Cas9 genome editing appear to be the most effective nucleic acid strategies to restore the physiologic expression of lncRNA, while curcumin, resveratrol, and quercetin are the main phytochemicals able to target and influence the expression of lncRNAs altered in cancer. Overall, this study provides a comprehensive overview regarding the role of lncRNAs in the phenotype plasticity of melanoma cells and their potential targeting using RNA-based therapy and natural products.
  
  
  
  
**MEG3**
-
2
of
10
  

### HGF facilitates methylation of MEG3, potentially implicated in vemurafenib resistance in melanoma.

2. J Gene Med. 2024 Jan;26(1):e3644. doi: 10.1002/jgm.3644 Epub 2023 Dec 10.
  
  
Jia X(1), Feng H(2), He S(2), Chen X(3), Feng H(4), Chen M(5), Hu X(2).
  
  
Author information: (1)Department of Pathology, Department of Pathology, Lhasa People's Hospital, Lh ...
  
  
BACKGROUND: Melanoma, a frequently encountered cutaneous malignancy characterized by a poor prognosis, persists in presenting formidable challenges despite the advancement in molecularly targeted drugs designed to improve survival rates significantly. Unfortunately, as more therapeutic choices have developed over time, the gradual emergence of drug resistance has become a notable impediment to the effectiveness of these therapeutic interventions. The hepatocyte growth factor (HGF)/c-met signaling pathway has attracted considerable attention, associated with drug resistance stemming from multiple potential mutations within the c-met gene. The activation of the HGF/c-met pathway operates in an autocrine manner in melanoma. Notably, a key player in the regulatory orchestration of HGF/c-met activation is the long non-coding RNA MEG3. METHODS: Melanoma tissues were collected to measure MEG3 expression. In vitro validation was performed on MEG3 to prove its oncogenic roles. Bioinformatic analyses were conducted on the TCGA database to build the MEG3-related score. The immune characteristics and mutation features of the MEG3-related score were explored. RESULTS: We revealed a negative correlation between HGF and MEG3. In melanoma cells, HGF inhibited MEG3 expression by augmenting the methylation of the MEG3 promoter. Significantly, MEG3 exhibits a suppressive impact on the proliferation and migration of melanoma cells, concurrently inhibiting c-met expression. Moreover, a predictive model centered around MEG3 demonstrates notable efficacy in forecasting critical prognostic indicators, immunological profiles, and mutation statuses among melanoma patients. CONCLUSIONS: The present study highlights the potential of MEG3 as a pivotal regulator of c-met, establishing it as a promising candidate for targeted drug development in the ongoing pursuit of effective therapeutic interventions.
  
  
  
  
**MEG3**
-
3
of
10
  

### Gambogenic Acid Inhibits Invasion and Metastasis of Melanoma through Regulation of lncRNA MEG3.

3. Biol Pharm Bull. 2023;46(10):1385-1393. doi: 10.1248/bpb.b23-00156
  
  
Wang M(1), Tu Y(1), Liu C(1), Cheng H(1), Zhang M(2), Li Q(1).
  
  
Author information: (1)Key Laboratory of Xin'an Medicine, Ministry of Education, Anhui Key Laborator ...
  
  
Cutaneous melanoma is an aggressive cancer, which is the most common type of melanoma. In our previous studies, gambogenic acid (GNA) inhibited the proliferation and migration of melanoma cells. Maternally expressed gene 3 (MEG3) is a long noncoding RNA (lncRNA) that has been shown to have inhibitory effects in a variety of cancers. However, the mechanisms in melanoma progression need to be further investigated. In the current study, we investigated the inhibitory effect of GNA on melanoma and its molecular mechanism through a series of cell and animal experiments. We found that GNA could improve epithelial mesenchymal transition by up-regulating the expression of the lncRNA MEG3 gene, thereby inhibiting melanoma metastasis in vitro and in vivo.
  
  
  
  
**MEG3**
-
4
of
10
  

### Cytoglobin inhibits non-thermal plasma-induced apoptosis in melanoma cells through regulation of the NRF2-mediated antioxidant response.

4. Redox Biol. 2022 Sep;55:102399. doi: 10.1016/j.redox.2022.102399 Epub 2022 Jul 14.
  
  
De Backer J(1), Lin A(2), Berghe WV(3), Bogaerts A(4), Hoogewijs D(5).
  
  
Author information: (1)Protein Chemistry, Proteomics and Epigenetic Signaling (PPES) Research Group, ...
  
  
Melanoma arises from pigment-producing cells called melanocytes located in the basal layers of the epidermis of the skin. Cytoglobin (CYGB) is a ubiquitously expressed hexacoordinated globin that is highly enriched in melanocytes and frequently downregulated during melanomagenesis. Previously, we showed that non-thermal plasma (NTP)-produced reactive oxygen and nitrogen species (RONS) lead to the formation of an intramolecular disulfide bridge that would allow CYGB to function as a redox-sensitive protein. Here, we investigate the cytotoxic effect of indirect NTP treatment in two melanoma cell lines with divergent endogenous CYGB expression levels, and we explore the role of CYGB in determining treatment outcome. Our findings are consistent with previous studies supporting that NTP cytotoxicity is mediated through the production of RONS and leads to apoptotic cell death in melanoma cells. Furthermore, we show that NTP-treated solutions elicit an antioxidant response through the activation of nuclear factor erythroid 2-related factor 2 (NRF2). The knockdown and overexpression of CYGB respectively sensitizes and protects melanoma cells from RONS-induced apoptotic cell death. The presence of CYGB enhances heme-oxygenase 1 (HO-1) and NRF2 protein expression levels, whereas the absence impairs their expression. Moreover, analysis of the CYGB-dependent transcriptome demonstrates the tumor suppressor long non-coding RNA maternally expressed 3 (MEG3) as a hitherto undescribed link between CYGB and NRF2. Thus, the presence of CYGB, at least in melanoma cells, seems to play a central role in determining the therapeutic outcome of RONS-inducing anticancer therapies, like NTP-treated solutions, possessing both tumor-suppressive and oncogenic features. Hence, CYGB expression could be of interest either as a biomarker or as a candidate for future targeted therapies in melanoma.
  
  
  
  
**MEG3**
-
5
of
10
  

### Upregulation LncRNA MEG3 expression suppresses proliferation and metastasis in melanoma via miR-208/SOX4.

5. Mol Cell Biochem. 2023 Feb;478(2):407-414. doi: 10.1007/s11010-022-04515-z Epub 2022 Jul 15.
  
  
Yang Y(#)(1), Jin L(#)(1)(2), He J(1), Wang R(1), Wang Y(1), Bai J(1), Chen Y(3), Luo Z(4).
  
  
Author information: (1)Department of Dermatology, The 1st Medical Center of the PLA General Hospital ...
  
  
Long non-coding RNAs (lncRNAs) regulate gene expression and play a significant role in cancer progression. Previously, downregulation of lncRNA MEG3 was shown to associate with poor clinical outcomes in melanoma patients. The basis for this association has not been described and the aims of this study were to identify a role for lncRNA MEG3 in melanoma and to describe its regulatory mechanism of action. RT-qPCR was used to detect lncRNA MEG3 expression in melanoma cells and tissues. Luciferase reporter assays were used to identify lncRNA MEG3 downstream targets. Melanoma cells were transfected with various expression vectors and these transfected cells were assessed for; migration, colony formation, proliferation, in vivo tumorigenesis, and metastatic potential. Melanoma cell lines were found to be sensitive to lncRNA MEG3 expression levels and overexpression was found to inhibit melanoma cell proliferation and invasion, both in vitro and in vivo. Luciferase reporter assays identified miR-208 and SOX4 as downstream targets of lncRNA MEG3. Overexpression of miR-208 and silencing of SOX4 rescued invasion and proliferation by cells that overexpressed lncRNA MEG3. Moreover, lncRNA MEG3 inhibited cancer stem cell differentiation and suppressed melanoma progression and metastasis through inhibition of miR-208 by SOX4.
  
  
  
  
**MEG3**
-
6
of
10
  

### Development and validation of an RNA sequencing panel for gene fusions in soft tissue sarcoma.

6. Cancer Sci. 2022 May;113(5):1843-1854. doi: 10.1111/cas.15317 Epub 2022 Mar 10.
  
  
Hu W(1), Yuan L(2), Zhang X(1), Ni Y(3)(4), Hong D(5), Wang Z(6), Li X(3), Ling Y(3), Zhang C(3), De ...
  
  
Author information: (1)State Key Laboratory of Oncology in South China, Collaborative Innovation Cen ...
  
  
Gene fusions are one of the most common genomic alterations in soft tissue sarcomas (STS), which contain more than 70 subtypes. In this study, a custom-designed RNA sequencing panel including 67 genes was developed and validated to identify gene fusions in STS. In total, 92 STS samples were analyzed using the RNA panel and 95.7% (88/92) successfully passed all the quality control parameters. Fusion transcripts were detected in 60.2% (53/88) of samples, including three novel fusions (MEG3-PLAG1, SH3BP1-NTRK1, and RPSAP52-HMGA2). The panel demonstrated excellent analytic accuracy, with 93.9% sensitivity and 100% specificity. The intra-assay, inter-assay, and personnel consistencies were all 100.0% in four samples and three replicates. In addition, different variants of ESWR1-FLI, COL1A1-PDGFB, NAB2-STAT6, and SS18-SSX were also identified in the corresponding subtypes of STS. In combination with histological and molecular diagnosis, 14.8% (13/88) patients finally changed preliminary histology-based classification. Collectively, this RNA panel developed in our study shows excellent performance on RNA from formalin-fixed, paraffin-embedded samples and can complement DNA-based assay, thereby facilitating precise diagnosis and novel fusion detection.
  
  
  
  
**MEG3**
-
7
of
10
  

### LncRNA POU3F3 promotes melanoma cell proliferation by downregulating lncRNA MEG3.

7. Discov Oncol. 2021 Jul 9;12(1):21. doi: 10.1007/s12672-021-00414-9
  
  
Liu Y(1)(2), Zhuang Y(1)(2), Fu X(1)(2), Li C(3).
  
  
Author information: (1)Department of Hand and Microvascular Surgery, Shenzhen People's Hospital, The ...
  
  
BACKGROUND: LncRNA POU3F3 (POU3F3) is overexpressed and plays oncogenic roles in esophageal squamous-cell carcinomas. LncRNA MEG3 (MEG3) has been characterized as a tumor suppressive lncRNA in different types of cancer. Our preliminary deep sequencing analysis revealed the inverse correlation between POU3F3 and MEG2 across melanoma tissues, indicating the interaction between them in melanoma. Therefore, this study was performed to investigate the crosstalk between POU3F3 and MEG3 in melanoma. METHODS: Tumor and adjacent healthy tissues collected from 60 melanoma patients were subjected to RNA extractions and RT-qPCRs to analyze the differential expression of POU3F3 and MEG2 in melanoma. In melanoma cells, POU3F3 and MEG2 were overexpressed to study the interactions between them. CCK-8 assays were performed to analyze the roles of POU3F3 and MEG2 in regulating melanoma cell proliferation. RESULTS: We found that POU3F3 was upregulated, while lncRNA MEG3 was downregulated in melanoma. Expression levels of POU3F3 and MEG3 were inversely correlated across tumor tissues. In vitro experiments showed that POU3F3 overexpression decreased MEG3 expression in melanoma cells, while MEG3 overexpression failed to affect POU3F3. POU3F3 overexpression increased melanoma cell proliferation, while MEG3 overexpression decreased melanoma cell proliferation. In addition, rescue experiments showed that MEG3 overexpression attenuated the enhancing effects of POU3F3 overexpression. CONCLUSION: POU3F3 may promote melanoma cell proliferation by downregulating MEG3.
  
  
  
  
**MEG3**
-
8
of
10
  

### Correction to: LncRNA MEG3 promotes melanoma growth, metastasis and formation through modulating miR-21/E-cadherin axis.

8. Cancer Cell Int. 2020 May 11;20:158. doi: 10.1186/s12935-020-01239-2 eCollection 2020.
  
  
Wu L(1), Zhu L(1), Li Y(1), Zheng Z(1), Lin X(1)(2), Yang C(1).
  
  
Author information: (1)1Department of Dermatology, The Sixth Affiliated Hospital, Sun Yat-sen Univer ...
  
  
Erratum for Cancer Cell Int. 2020 Jan 10;20:12. doi: 10.1186/s12935-019-1087-4.
  
  
  
  
**MEG3**
-
9
of
10
  

### LncRNA MEG3 promotes melanoma growth, metastasis and formation through modulating miR-21/E-cadherin axis.

9. Cancer Cell Int. 2020 Jan 10;20:12. doi: 10.1186/s12935-019-1087-4 eCollection 2020.
  
  
Wu L(1), Zhu L(1), Li Y(1), Zheng Z(1), Lin X(1)(2), Yang C(1).
  
  
Author information: (1)1Department of Dermatology, The Sixth Affiliated Hospital, Sun Yat-sen Univer ...
  
  
Erratum in Cancer Cell Int. 2020 May 11;20:158. doi: 10.1186/s12935-020-01239-2.
  
  
  
  
**MEG3**
-
10
of
10
  

### Long non-coding RNA MEG3 promotes cerebral ischemia-reperfusion injury through increasing pyroptosis by targeting miR-485/AIM2 axis.

10. Exp Neurol. 2020 Mar;325:113139. doi: 10.1016/j.expneurol.2019.113139 Epub 2019 Nov 30.
  
  
Liang J(1), Wang Q(1), Li JQ(1), Guo T(2), Yu D(3).
  
  
Author information: (1)Department of Neurology, Affiliated Haikou Hospital of Xiangya Medical Colleg ...
  
  
OBJECTIVE: Inflammasome contributes to ischemic brain injury by inducing pyroptosis and inflammation. The aim of this study is to unravel the mechanism of long non-coding RNA (lncRNA) maternally expressed gene 3 (MEG3)-mediated regulation of absent in melanoma 2 (AIM2) inflammasome during cerebral ischemia/reperfusion (I/R). METHODS: In vivo middle cerebral artery occlusion (MCAO) rat model and in vitro oxygen-glucose deprivation/reperfusion (OGD/R)-treated neurocytes model were generated. TTC, H&E staining and TUNEL were performed to assess the cerebral ischemic injury. LDH and MTT assays were used to detect cell viability and cytotoxicity. qRT-PCR was used to detect the expression levels of MEG3, miR-485 and AIM2. Immunohistochemistry (IHC) and immunofluorescence were conducted to detect the AIM2 expression. ELISA and Western blotting were performed to determine the secretion and protein levels of inflammasome signaling proteins. Dual luciferase reporter assay and Ago2-RIP were used to validate the direct interaction among MEG3, miR-485 and AIM2. RESULTS: In both MCAO rats and OGD/R-treated neurocytes, MEG3 and AIM2 were significantly up-regulated, whereas miR-485 was down-regulated. MCAO induces pyroptosis and release of IL-1β and IL-18 in ischemia brain. MEG3 acted as a molecular sponge to suppress miR-485, and AIM2 was identified as a direct target of miR-485. Knockdown of MEG3 inhibited OGD/R-induced pyroptosis and inflammation, and lack of MEG3 inhibited caspase1 signaling and decreased the expression of AIM2, ASC, cleaved-caspase1 and GSDMD-N. While overexpression of MEG3 exerted opposite effects. CONCLUSION: MEG3/miR-485/AIM2 axis contributes to pyroptosis via activating caspase1 signaling during cerebral I/R, suggesting that this axis may be a potent therapeutic target in ischemic stroke.
  
  
  
  
  

---

  

# MERTK

**Signature gene in:** MDM
  
**Differentially expressed in:** MDM
  
  
**MERTK**
-
1
of
10
  

### Permanent Efferocytosis Prevention by Terminating MERTK Recycle on Tumor-Associated Macrophages for Cancer Immunotherapy.

1. J Am Chem Soc. 2025 Apr 28. doi: 10.1021/jacs.5c05640 Online ahead of print.
  
  
Huang P(1), Liu Y(2)(3), Zhao C(2)(3), Wang C(2)(3), Wang L(2)(3), Luo M(2)(3), Wang W(2)(3), Shan W ...
  
  
Author information: (1)Department of Diagnostic Radiology, Yong Loo Lin School of Medicine, National ...
  
  
Efferocytosis of apoptotic tumor cells by tumor-associated macrophages mediated through the phosphatidylserine (PtdSer)/MER proto-oncogene tyrosine kinase (MERTK) axis can exacerbate tumor immunosuppression, and conversely, prevention of efferocytosis via blocking PtdSer-MerTK association using prevalent antibodies represents a promising strategy for reversing tumor immunosuppression and boosting antitumor immunity. However, it remains unclear whether the antibody blockade can induce durable efferocytosis prevention and achieve sustained tumor growth inhibition. Here, we have shown that utilizing PtdSer and MERTK antibodies induced only a transient rather than a persistent efferocytosis prevention effect, and little enhancement was observed even after improving antibody enrichment in tumor sites. Further mechanistic studies suggested that degradation of anti-MerTK antibody and recycling of the MERTK receptor to the cell membrane would compromise the therapeutic benefits of antibody blockade. Based on these findings, we developed a CRISPR/Cas9 gene editing system deployed using Cas9 mRNA and MERTK sgRNA to permanently knock out MERTK, which achieved durable efferocytosis prevention, elicited persistent in situ vaccination immune responses via enhancing X-ray irradiation-induced immunogenic cell death, and led to sustained tumor suppression effects together with anti-PtdSer antibody and X-ray irradiation treatment in multiple B16 melanoma tumor models. Our findings provide a reliable gene-editing-mediated strategy for long-term modulating MERTK homeostasis and overcoming MerTK-dependent cancer immune evasion, generating adaptive antitumor immune responses for sustained cancer immunotherapy.
  
  
  
  
**MERTK**
-
2
of
10
  

### Preliminary Study of Radionuclide-Labeled MerTK-Targeting PET Imaging Agents for the Diagnosis of Melanoma.

2. J Med Chem. 2024 Nov 14;67(21):19813-19825. doi: 10.1021/acs.jmedchem.4c02156 Epub 2024 Nov 1.
  
  
Wang C(1)(2), Liu H(1)(2), Yang Y(1)(2), Sun Q(1)(2), Yin L(1)(2), Yang L(1)(2), Wang X(3), Zhao W(4 ...
  
  
Author information: (1)Department of Nuclear Medicine, Nuclear Medicine and Molecular Imaging Key La ...
  
  
MERTK PET imaging holds potential as a promising approach for assessing tumor aggressiveness and monitoring treatment response. In this study, we synthesized a series of 18F- and 68Ga-labeled tracers derived from MERTK inhibitors for detection of MERTK expression. Among the synthesized agents, the dimeric compounds [68Ga]10 and [68Ga]12 demonstrated good in vivo and in vitro stability, high affinities to the MERTK receptor, and good MerTK-targeting specificity. Notably, [68Ga]10 exhibited a tumor uptake of 2.6 ± 0.2%ID/g at 1 h p. i. in B16F10 tumor-bearing mice, nearly tripling the uptake of its monomeric counterpart [68Ga]3. A similar enhancement was observed with [68Ga]12 compared to its monomeric analog [68Ga]6. Additionally, [18F]14 achieved a tumor uptake of 7.6 ± 0.5%ID/g at 2 h p. i., outperforming the previously reported [18F]15. Biodistribution analysis further validated the results, highlighting their potential for clinical investigation.
  
  
  
  
**MERTK**
-
3
of
10
  

### MERTK(+) macrophages promote melanoma progression and immunotherapy resistance through AhR-ALKAL1 activation.

3. Sci Adv. 2024 Oct 4;10(40):eado8366. doi: 10.1126/sciadv.ado8366 Epub 2024 Oct 4.
  
  
Wu N(1)(2), Li J(3), Li L(1)(2), Yang L(1)(2), Dong L(1)(2), Shen C(1)(2), Sha S(1)(2), Fu Y(1)(2), ...
  
  
Author information: (1)Department of Dermatology, Union Hospital, Tongji Medical College, Huazhong U ...
  
  
Despite our increasing understanding of macrophage heterogeneity, drivers of macrophage phenotypic and functional polarization in the microenvironment are not fully elucidated. Here, our single-cell RNA sequencing data identify a subpopulation of macrophages expressing high levels of the phagocytic receptor MER proto-oncogene tyrosine kinase (MERTK+ macrophages), which is closely associated with melanoma progression and immunotherapy resistance. Adoptive transfer of the MERTK+ macrophages into recipient mice notably accelerated tumor growth regardless of macrophage depletion. Mechanistic studies further revealed that ALK And LTK Ligand 1 (ALKAL1), a target gene of aryl hydrocarbon receptor (AhR), facilitated MERTK phosphorylation, resulting in heightened phagocytic activity of MERTK+ macrophages and their subsequent polarization toward an immunosuppressive phenotype. Specifically targeted delivery of AhR antagonist to tumor-associated macrophages with mannosylated micelles could suppress MERTK expression and improved the therapeutic efficacy of anti-programmed cell death ligand 1 therapy. Our findings shed light on the regulatory mechanism of MERTK+ macrophages and provide strategies for improving the efficacy of melanoma immunotherapy.
  
  
  
  
**MERTK**
-
4
of
10
  

### MERTK Induces Dysfunctional Dendritic Cells by Metabolic Reprogramming.

4. Cancer Immunol Res. 2024 Sep 3;12(9):1268-1285. doi: 10.1158/2326-6066.CIR-23-0666
  
  
Zewdie EY(1)(2), Edwards GM(1), Hunter DM(1), Earp HS(1)(2)(3), Holtzhausen A(1)(2).
  
  
Author information: (1)UNC Lineberger Comprehensive Cancer Center, Chapel Hill, North Carolina. (2)D ...
  
  
Checkpoint inhibitors, specifically anti-programmed cell death protein 1 (PD1), have shown success in treating metastatic melanoma; however, some patients develop resistance. Dendritic cells (DC) play a key role in initiating an immune response, but in certain circumstances they become ineffective. We investigated the role of MERTK, a receptor tyrosine kinase responsible for myeloid cell clearance of dead cells, in the regulation of DC function and metabolism in the tumor microenvironment. Tumors resistant to anti-PD1 exhibited increased levels of MERTK+ DCs. Treating wild-type DCs with apoptotic melanoma cells in vitro resulted in increased MERTK expression, elevated mitochondrial respiration and fatty acid oxidation, and reduced T-cell stimulatory capacity, all characteristics of dysfunctional DCs. In contrast, dead cells had only limited effect on the metabolism of MerTK-deficient DCs, which instead maintained an antigen-presenting, stimulatory phenotype. The efficacy of anti-PD1 to slow tumor progression and induce antigen specific T-cell infiltration was markedly increased in mice with selective ablation of MERTK in the DC compartment, suggesting the possibility of therapeutically targeting MERTK to modulate DC metabolism and function and enhance anti-PD1 therapy.
  
  
  
  
**MERTK**
-
5
of
10
  

### Targeting Tyro3, Axl, and MERTK Receptor Tyrosine Kinases Significantly Sensitizes Triple-Negative Breast Cancer to CDK4/6 Inhibition.

5. Cancers (Basel). 2024 Jun 18;16(12):2253. doi: 10.3390/cancers16122253
  
  
Demirsoy S(1), Tran H(2), Liu J(2), Li Y(3), Yang S(3), Aregawi D(1), Glantz MJ(1), Jacob NK(2), Wal ...
  
  
Author information: (1)Departments of Neurosurgery, Penn State University, Hershey, PA 17033, USA. ( ...
  
  
Triple-negative breast cancer (TNBC) is the most aggressive subtype with high metastasis and mortality rates. Given the lack of actionable targets such as ER and HER2, TNBC still remains an unmet therapeutic challenge. Despite harboring high CDK4/6 expression levels, the efficacy of CDK4/6 inhibition in TNBC has been limited due to the emergence of resistance. The resistance to CDK4/6 inhibition is mainly mediated by RB1 inactivation. Since our aim is to overcome resistance to CDK4/6 inhibition, in this study, we primarily used the cell lines that do not express RB1. Following a screening for activated receptor tyrosine kinases (RTKs) upon CDK4/6 inhibition, we identified the TAM (Tyro3, Axl, and MERTK) RTKs as a crucial therapeutic vulnerability in TNBC. We show that targeting the TAM receptors with a novel inhibitor, sitravatinib, significantly sensitizes TNBC to CDK4/6 inhibitors. Upon prolonged HER2 inhibitor treatment, HER2+ breast cancers suppress HER2 expression, physiologically transforming into TNBC-like cells. We further show that the combined treatment is highly effective against drug-resistant HER2+ breast cancer as well. Following quantitative proteomics and RNA-seq data analysis, we extended our study into the immunophenotyping of TNBC. Given the roles of the TAM receptors in promoting the creation of an immunosuppressive tumor microenvironment (TME), we further demonstrate that the combination of CDK4/6 inhibitor abemaciclib and sitravatinib modifies the immune landscape of TNBC to favor immune checkpoint blockade. Overall, our study offers a novel and highly effective combination therapy against TNBC and potentially treatment-resistant HER2+ breast cancer that can be rapidly moved to the clinic.
  
  
  
  
**MERTK**
-
6
of
10
  

### Inhibition of CSF-1R and IL-6R prevents conversion of cDC2s into immune incompetent tumor-induced DC3s boosting DC-driven therapy potential.

6. Cell Rep Med. 2024 Feb 20;5(2):101386. doi: 10.1016/j.xcrm.2023.101386 Epub 2024 Jan 18.
  
  
Becker AMD(1), Decker AH(1), Flórez-Grau G(1), Bakdash G(1), Röring RJ(2), Stelloo S(3), Vermeulen M ...
  
  
Author information: (1)Department of Tumor Immunology, Radboud Institute for Molecular Life Sciences ...
  
  
The human dendritic cell (DC) family has recently been expanded by CD1c+CD14+CD163+ DCs, introduced as DC3s. DC3s are found in tumors and peripheral blood of cancer patients. Here, we report elevated frequencies of CD14+ cDC2s, which restore to normal frequencies after tumor resection, in non-small cell lung cancer patients. These CD14+ cDC2s phenotypically resemble DC3s and exhibit increased PD-L1, MERTK, IL-10, and IDO expression, consistent with inferior T cell activation ability compared with CD14- cDC2s. In melanoma patients undergoing CD1c+ DC vaccinations, increased CD1c+CD14+ DC frequencies correlate with reduced survival. We demonstrate conversion of CD5+/-CD1c+CD14- cDC2s to CD14+ cDC2s by tumor-associated factors, whereas monocytes failed to express CD1c under similar conditions. Targeted proteomics identified IL-6 and M-CSF as dominant drivers, and we show that IL-6R and CSF1R inhibition prevents tumor-induced CD14+ cDC2s. Together, this indicates cDC2s as direct pre-cursors of DC3-like CD1c+CD14+ DCs and provides insights into the importance and modulation of CD14+ DC3s in anti-tumor immune responses.
  
  
  
  
**MERTK**
-
7
of
10
  

### Intrinsic disorder may drive the interaction of PROS1 and MERTK in uveal melanoma.

7. Int J Biol Macromol. 2023 Oct 1;250:126027. doi: 10.1016/j.ijbiomac.2023.126027 Epub 2023 Jul 26.
  
  
Djulbegovic M(1), Taylor Gonzalez DJ(1), Antonietti M(1), Uversky VN(2), Shields CL(3), Karp CL(4).
  
  
Author information: (1)Bascom Palmer Eye Institute, University of Miami, Miami, FL, USA. (2)Departme ...
  
  
BACKGROUND: Class 2 uveal melanomas are associated with the inactivation of the BRCA1 ((breast cancer type 1 susceptibility protein)-associated protein 1 (BAP1)) gene. Inactivation of BAP1 promotes the upregulation of vitamin K-dependent protein S (PROS1), which interacts with the tyrosine-protein kinase Mer (MERTK) receptor on M2 macrophages to induce an immunosuppressive environment. METHODS: We simulated the interaction of PROS1 with MERTK with ColabFold. We evaluated PROS1 and MERTK for the presence of intrinsically disordered protein regions (IDPRs) and disorder-to-order (DOT) regions to understand their protein-protein interaction (PPI). We first evaluated the structure of each protein with AlphaFold. We then analyzed specific sequence-based features of the PROS1 and MERTK with a suite of bioinformatics tools. RESULTS: With high-resolution, moderate confidence, we successfully modeled the interaction between PROS1 and MERTK (predicted local distance difference test score (pDLLT) = 70.68). Our structural analysis qualitatively demonstrated IDPRs (i.e., spaghetti-like entities) in PROS1 and MERK. PROS1 was 23.37 % disordered, and MERTK was 23.09 % disordered, classifying them as moderately disordered and flexible proteins. PROS1 was significantly enriched in cysteine, the most order-promoting residue (p-value <0.05). Our IUPred analysis demonstrated that there are two disorder-to-order transition (DOT) regions in PROS1. MERTK was significantly enriched in proline, the most disorder-promoting residue (p-value <0.05), but did not contain DOT regions. Our STRING analysis demonstrated that the PPI network between PROS1 and MERTK is more complex than their assumed one-to-one binding (p-value <2.0 × 10^-6^). CONCLUSION: Our findings present a novel prediction for the interaction between PROS1 and MERTK. Our findings show that PROS1 and MERTK contain elements of intrinsic disorder. PROS1 has two DOT regions that are attractive immunotherapy targets. We recommend that IDPRs and DOT regions found in PROS1 and MERTK should be considered when developing immunotherapies targeting this PPI.
  
  
  
  
**MERTK**
-
8
of
10
  

### Axl contributes to efficient migration and invasion of melanoma cells.

8. PLoS One. 2023 Mar 29;18(3):e0283749. doi: 10.1371/journal.pone.0283749 eCollection 2023.
  
  
Shao H(1)(2), Teramae D(1)(2), Wells A(1)(2).
  
  
Author information: (1)Department of Pathology, University of Pittsburgh, Pittsburgh, Pennsylvania, ...
  
  
Axl, a member of the TAM receptor family has been broadly suggested to play a key role in tumor metastasis. However, the function of Axl in the invasion and metastasis of melanoma, the most lethal skin cancer, remains largely unknown. In the present study, we found that melanoma cell lines present variable protein levels of Axl and Tyro3; interestingly, MERTK is not noted at detectable levels in any of tested MGP (metastatic growth phase) cell lines. Treatment with recombinant human Gas6 significantly activates Akt in the Axl-expressing WM852 and IgR3 lines but just slightly in WM1158. IgR3, WM852 and WM1158 demonstrate different autocrine signaling. Knockdown of Axl by siRNA or the treatment with Axl-specific inhibitor R428 dramatically inhibits the migration and invasion of both IgR3 and WM852 in vitro. These findings suggest that Axl enhances the invasion of melanoma cells.
  
  
  
  
**MERTK**
-
9
of
10
  

### Preclinical Evaluation of Trabectedin in Combination With Targeted Inhibitors for Treatment of Metastatic Uveal Melanoma.

9. Invest Ophthalmol Vis Sci. 2022 Dec 1;63(13):14. doi: 10.1167/iovs.63.13.14
  
  
Glinkina K(1), Nemati F(2), Teunisse AFAS(1), Gelmi MC(3), Etienne V(2), Kuipers MJ(1), Alsafadi S(4 ...
  
  
Author information: (1)Department of Cell and Chemical Biology, Leiden University Medical Center, Le ...
  
  
PURPOSE: Uveal melanoma (UM) is considered a rare disease; yet, it is the most common intraocular malignancy in adults. Although the primary tumor may be efficiently managed, more than 50% of patients with UM develop distant metastases. The mortality at the first year after diagnosis of metastatic UM has been estimated at 81%, and the poor prognosis has not improved in the past years due to the lack of effective therapies. METHODS: In order to search for novel therapeutic possibilities for metastatic UM, we performed a small-scale screen of targeted drug combinations. We verified the targets of the tested compounds by western blotting and PCR and clarified the mechanism of action of the selected combinations by caspase 3 and 7 activity assay and flow cytometry. The best two combinations were tested in a mouse patient-derived xenograft (PDX) UM model as putative therapeutics for metastatic UM. RESULTS: Combinations of the multitarget drug trabectedin with either the CK2/CLK double-inhibitor CX-4945 (silmitasertib) or the c-MET/TAM (TYRO3, Axl, MERTK) receptor inhibitors foretinib and cabozantinib demonstrated synergistic effects and induced apoptosis (relative caspase 3 and 7 activity increased up to 20.5-fold in UM cell lines). In the case of the combination of foretinib and cabozantinib, inhibition of the TAM receptors, but not c-Met, was essential to inhibit the growth of UM cells. Monotreatment with trabectedin inhibited tumor growth by 42%, 49%, and 35% in the MM26, MM309, and MM339 PDX mouse models, respectively. CONCLUSIONS: Trabectedin alone or in combination with cabozantinib inhibited tumor growth in PDX UM mouse models. Blocking of MERTK, rather than TYRO3, activity inhibited UM cell growth and synergized with trabectedin.
  
  
  
  
**MERTK**
-
10
of
10
  

### BAP1 Loss Promotes Suppressive Tumor Immune Microenvironment via Upregulation of PROS1 in Class 2 Uveal Melanomas.

10. Cancers (Basel). 2022 Jul 28;14(15):3678. doi: 10.3390/cancers14153678
  
  
Kaler CJ(1), Dollar JJ(1), Cruz AM(1), Kuznetsoff JN(1), Sanchez MI(1), Decatur CL(1), Licht JD(2), ...
  
  
Author information: (1)Bascom Palmer Eye Institute, Sylvester Comprehensive Cancer Center and Interd ...
  
  
Uveal melanoma (UM) is the most common primary cancer of the eye and is associated with a high rate of metastatic death. UM can be stratified into two main classes based on metastatic risk, with class 1 UM having a low metastatic risk and class 2 UM having a high metastatic risk. Class 2 UM have a distinctive genomic, transcriptomic, histopathologic, and clinical phenotype characterized by biallelic inactivation of the BAP1 tumor-suppressor gene, an immune-suppressive microenvironment enriched for M2-polarized macrophages, and poor response to checkpoint-inhibitor immunotherapy. To identify potential mechanistic links between BAP1 loss and immune suppression in class 2 UM, we performed an integrated analysis of UM samples, as well as genetically engineered UM cell lines and uveal melanocytes (UMC). Using RNA sequencing (RNA-seq), we found that the most highly upregulated gene associated with BAP1 loss across these datasets was PROS1, which encodes a ligand that triggers phosphorylation and activation of the immunosuppressive macrophage receptor MERTK. The inverse association between BAP1 and PROS1 in class 2 UM was confirmed by single-cell RNA-seq, which also revealed that MERTK was upregulated in CD163+ macrophages in class 2 UM. Using ChIP-seq, BAP1 knockdown in UM cells resulted in an accumulation of H3K27ac at the PROS1 locus, suggesting epigenetic regulation of PROS1 by BAP1. Phosphorylation of MERTK in RAW 264.7 monocyte-macrophage cells was increased upon coculture with BAP1-/- UMCs, and this phosphorylation was blocked by depletion of PROS1 in the UMCs. These findings were corroborated by multicolor immunohistochemistry, where class 2/BAP1-mutant UMs demonstrated increased PROS1 expression in tumor cells and increased MERTK phosphorylation in CD163+ macrophages compared with class 1/BAP1-wildtype UMs. Taken together, these findings provide a mechanistic link between BAP1 loss and the suppression of the tumor immune microenvironment in class 2 UMs, and they implicate the PROS1-MERTK pathway as a potential target for immunotherapy in UM.
  
  
  
  
  

---

  

# MTSS1

**Signature gene in:** Stromal cells
  
**Differentially expressed in:** Stromal cells
  
  
**MTSS1**
-
1
of
6
  

### The mechanisms behind the dual role of long non-coding RNA (lncRNA) metastasis suppressor-1 in human tumors: Shedding light on the molecular mechanisms.

1. Pathol Res Pract. 2024 Apr;256:155189. doi: 10.1016/j.prp.2024.155189 Epub 2024 Feb 16.
  
  
Abdullaev B(1), Alsaab HO(2), Hjazi A(3), Alkhafaji AT(4), Alawadi AH(5), Hamzah HF(6).
  
  
Author information: (1)Research Department of Biotechnology, New Uzbekistan University, Mustaqillik ...
  
  
When the expression levels of metastasis suppressor-1 (MTSS1) were discovered to be downregulated in a metastatic cancer cell line in 2002, it was proposed that MTSS1 functioned as a suppressor of metastasis. The 755 amino acid long protein MTSS1 connects to actin and organizes the cytoskeleton. Its gene is located on human chromosome 8q24. The suppressor of metastasis in metastatic cancer was first found to be MTSS1. Subsequent reports revealed that MTSS1 is linked to the prevention of metastasis in a variety of cancer types, including hematopoietic cancers like diffuse large B cell lymphoma and esophageal, pancreatic, and stomach cancers. Remarkably, conflicting results have also been documented. For instance, it has been reported that MTSS1 expression levels are elevated in a subset of melanomas, hepatocellular carcinoma associated with hepatitis B, head and neck squamous cell carcinoma, and lung squamous cell carcinoma. This article provides an overview of the pathological effects of lncRNA MTSS1 dysregulation in cancer. In order to facilitate the development of MTSS1-based therapeutic targeting, we also shed light on the current understanding of MTS1.
  
  
  
  
**MTSS1**
-
2
of
6
  

### Metastasis suppressor 1 (MTSS1) expression is associated with reduced in-vivo metastasis and enhanced patient survival in lung adenocarcinoma.

2. Clin Exp Metastasis. 2018 Feb;35(1-2):15-23. doi: 10.1007/s10585-017-9869-3 Epub 2017 Dec 7.
  
  
Taylor MD(1)(2), Bollt O(3), Iyer SC(3)(4), Robertson GP(3)(4)(5)(6)(7)(8).
  
  
Author information: (1)Department of Surgery, The Pennsylvania State University College of Medicine, ...
  
  
Metastasis suppressor 1 (MTSS1) has been shown to be a metastasis suppressor in a number of cancers. However, its role in lung adenocarcinoma is largely unknown. To evaluate the significance of MTSS1 expression on lung adenocarcinoma metastatic properties, the gain or loss of MTSS1 in in vivo and in vitro experiments were employed. Using an in vivo orthotopic mouse xenograft model mimicking human disease progression, stable overexpression of MTSS1 in lung adenocarcinoma cells resulted in a significant decrease in metastatic burden. Stable overexpression of MTSS1 in NCI-H1299 decreased in vitro lung adenocarcinoma invasion and migration while knockdown of MTSS1 in A549 resulted in a significant increase in cell invasion and migration. Using The Cancer Genome Atlas dataset of over 500 patient lung adenocarcinoma specimens, we demonstrated a 20% increase in 5-year survival associated with preserved intratumoral MTSS expression. MTSS1 expression in lung adenocarcinoma is associated with decreased metastatic burden, as assessed by an in vivo orthotopic model, and correlates with a 20% survival advantage at 5 years following diagnosis. In vitro data suggests MTSS1 regulates lung adenocarcinoma through augmentation of cell invasion and migration.
  
  
  
  
**MTSS1**
-
3
of
6
  

### Reprogramming human A375 amelanotic melanoma cells by catalase overexpression: Upregulation of antioxidant genes correlates with regression of melanoma malignancy and with malignant progression when downregulated.

3. Oncotarget. 2016 Jul 5;7(27):41154-41171. doi: 10.18632/oncotarget.9273
  
  
Bracalente C(1)(2), Ibañez IL(1)(2), Berenstein A(3), Notcovich C(1), Cerda MB(1), Klamt F(4), Chern ...
  
  
Author information: (1)Departamento de Micro y Nanotecnología, Comisión Nacional de Energía Atómica, ...
  
  
Reactive oxygen species (ROS) are implicated in tumor transformation. The antioxidant system (AOS) protects cells from ROS damage. However, it is also hijacked by cancers cells to proliferate within the tumor. Thus, identifying proteins altered by redox imbalance in cancer cells is an attractive prognostic and therapeutic tool. Gene expression microarrays in A375 melanoma cells with different ROS levels after overexpressing catalase were performed. Dissimilar phenotypes by differential compensation to hydrogen peroxide scavenging were generated. The melanotic A375-A7 (A7) upregulated TYRP1, CNTN1 and UCHL1 promoting melanogenesis. The metastatic A375-G10 (G10) downregulated MTSS1 and TIAM1, proteins absent in metastasis. Moreover, differential coexpression of AOS genes (EPHX2, GSTM3, MGST1, MSRA, TXNRD3, MGST3 and GSR) was found in A7 and G10. Their increase in A7 improved its AOS ability and therefore, oxidative stress response, resembling less aggressive tumor cells. Meanwhile, their decrease in G10 revealed a disruption in the AOS and therefore, enhanced its metastatic capacity.These gene signatures, not only bring new insights into the physiopathology of melanoma, but also could be relevant in clinical prognostic to classify between non aggressive and metastatic melanomas.
  
  
  
  
**MTSS1**
-
4
of
6
  

### p63 controls cell migration and invasion by transcriptional regulation of MTSS1.

4. Oncogene. 2016 Mar 24;35(12):1602-8. doi: 10.1038/onc.2015.230 Epub 2015 Jun 29.
  
  
Giacobbe A(1), Compagnone M(1), Bongiorno-Borbone L(1), Antonov A(2), Markert EK(3), Zhou JH(4), Ann ...
  
  
Author information: (1)Department of Experimental Medicine and Surgery, University of Rome 'Tor Verg ...
  
  
Metastasis is a multistep cell-biological process, which is orchestrated by many factors, including metastasis activators and suppressors. Metastasis Suppressor 1 (MTSS1) was originally identified as a metastasis suppressor protein whose expression is lost in metastatic bladder and prostate carcinomas. However, recent findings indicate that MTSS1 acts as oncogene and pro-migratory factor in melanoma tumors. Here, we identify and characterized a molecular mechanism controlling MTSS1 expression, which impinges on a pro-tumorigenic role of MTSS1 in breast tumors. We found that in normal and in cancer cell lines ΔNp63 is able to drive the expression of MTSS1 by binding to a p63-binding responsive element localized in the MTSS1 locus. We reported that ΔNp63 is able to drive the migration of breast tumor cells by inducing the expression of MTSS1. Notably, in three human breast tumors data sets the MTSS1/p63 co-expression is a negative prognostic factor on patient survival, suggesting that the MTSS1/p63 axis might be functionally important to regulate breast tumor progression.
  
  
  
  
**MTSS1**
-
5
of
6
  

### Tissue-based microarray expression of genes predictive of metastasis in uveal melanoma and differentially expressed in metastatic uveal melanoma.

5. J Ophthalmic Vis Res. 2013 Oct;8(4):303-7.
  
  
Demirci H(1), Reed D(1), Elner VM(1).
  
  
Author information: (1)Department of Ophthalmology and Visual Sciences, W.K. Kellogg Eye Center, Uni ...
  
  
PURPOSE: To screen the microarray expression of CDH1, ECM1, EIF1B, FXR1, HTR2B, ID2, LMCD1, LTA4H, MTUS1, RAB31, ROBO1, and SATB1 genes which are predictive of primary uveal melanoma metastasis, and NFKB2, PTPN18, MTSS1, GADD45B, SNCG, HHIP, IL12B, CDK4, RPLP0, RPS17, RPS12 genes that are differentially expressed in metastatic uveal melanoma in normal whole human blood and tissues prone to metastatic involvement by uveal melanoma. METHODS: We screened the GeneNote and GNF BioGPS databases for microarray analysis of genes predictive of primary uveal melanoma metastasis and those differentially expressed in metastatic uveal melanoma in normal whole blood, liver, lung and skin. RESULTS: Microarray analysis showed expression of all 22 genes in normal whole blood, liver, lung and skin, which are the most common sites of metastases. In the GNF BioGPS database, data for expression of the HHIP gene in normal whole blood and skin was not complete. CONCLUSIONS: Microarray analysis of genes predicting systemic metastasis of uveal melanoma and genes differentially expressed in metastatic uveal melanoma may not be used as a biomarker for metastasis in whole blood, liver, lung, and skin. Their expression in tissues prone to metastasis may suggest that they play a role in tropism of uveal melanoma metastasis to these tissues.
  
  
  
  
**MTSS1**
-
6
of
6
  

### MTSS1 is a metastasis driver in a subset of human melanomas.

6. Nat Commun. 2014 Mar 17;5:3465. doi: 10.1038/ncomms4465
  
  
Mertz KD(1), Pathria G(2), Wagner C(2), Saarikangas J(3), Sboner A(4), Romanov J(5), Gschaider M(5), ...
  
  
Author information: (1)1] Division of Immunology, Allergy and Infectious Diseases (DIAID), Departmen ...
  
  
In cancers with a highly altered genome, distinct genetic alterations drive subsets rather than the majority of individual tumours. Here we use a sequential search across human tumour samples for transcript outlier data points with associated gene copy number variations that correlate with patient's survival to identify genes with pro-invasive functionality. Employing loss and gain of function approaches in vitro and in vivo, we show that one such gene, MTSS1, promotes the ability of melanocytic cells to metastasize and engages actin dynamics via Rho-GTPases and cofilin in this process. Indeed, high MTSS1 expression defines a subgroup of primary melanomas with unfavourable prognosis. These data underscore the biological, clinical and potential therapeutic implications of molecular subsets within genetically complex cancers.
  
  
  
  
  

---

  

# MTUS1

**Signature gene in:** Endothelial cells
  
**Differentially expressed in:** Endothelial cells
  
  
**MTUS1**
-
1
of
3
  

### Identification of prognostic genes in uveal melanoma microenvironment.

1. PLoS One. 2020 Nov 16;15(11):e0242263. doi: 10.1371/journal.pone.0242263 eCollection 2020.
  
  
Luo H(1)(2), Ma C(1)(3).
  
  
Author information: (1)Charité-Universitätsmedizin Berlin, Corporate Member of Freie Universität Ber ...
  
  
BACKGROUND: Uveal melanoma (UM) is the most common primary intraocular malignancy in adults. Many previous studies have demonstrated that the infiltrating of immune and stromal cells in the tumor microenvironment contributes significantly to prognosis. METHODS: Dataset TCGA-UVM, download from TCGA portal, was taken as the training cohort, and GSE22138, obtained from GEO database, was set as the validation cohort. ESTIMATE algorithm was applied to find intersection differentially expressed genes (DEGs) among tumor microenvironment. Kaplan-Meier analysis and univariate Cox regression model were performed on intersection DEGs to initial screen for potential prognostic genes. Then these genes entered into the validation cohort for validation using the same methods as that in the training cohort. Moreover, we conducted correlation analyses between the genes obtained in the validation cohort and the status of chromosome 3, chromosome 8q, and tumor metastasis to get prognosis genes. At last, the immune infiltration analysis was performed between the prognostic genes and 6 main kinds of tumor-infiltrating immune cells (TICs) for understanding the role of the genes in the tumor microenvironment. RESULTS: 959 intersection DEGs were found in the UM microenvironment. Kaplan-Meier and Cox analysis was then performed in the training and validation cohorts on these DEGs, and 52 genes were identified with potential prognostic value. After comparing the 52 genes to chromosome 3, chromosome 8q, and metastasis, we obtained 21 genes as the prognostic genes. The immune infiltration analysis showed that Neutrophil had the potential prognostic ability, and almost every prognostic gene we had identified was correlated with abundances of Neutrophil and CD8+ T Cell. CONCLUSIONS: Identifying 21 prognosis genes (SERPINB9, EDNRB, RAPGEF3, HFE, RNF43, ZNF415, IL12RB2, MTUS1, NEDD9, ZNF667, AZGP1, WARS, GEM, RAB31, CALHM2, CA12, MYEOV, CELF2, SLCO5A1, ISM1, and PAPSS2) could accurately identify patients' prognosis and had close interactions with Neutrophil in the tumor environment, which may provide UM patients with personalized prognosis prediction and new treatment insights.
  
  
  
  
**MTUS1**
-
2
of
3
  

### Altered expression profile of circular RNAs in conjunctival melanoma.

2. Epigenomics. 2019 May;11(7):787-804. doi: 10.2217/epi-2019-0029 Epub 2019 Mar 20.
  
  
Shang Q(1)(2), Li Y(1)(2), Wang H(1)(2), Ge S(1)(2), Jia R(1)(2).
  
  
Author information: (1)Department of Ophthalmology, Ninth People's Hospital, Shanghai Jiao Tong Univ ...
  
  
Aim: We aimed to explore the roles of circular RNA (circRNA) in conjunctival melanoma. Materials & methods: The altered circRNAs were identified by RNA sequencing. The function of one circRNA, circMTUS1, was explored by several experiments in conjunctival melanoma. Bioinformatic analyses and an RNA immunoprecipitation assay were performed to further study the downstream mechanism of circMTUS1. Results: We identified 9300 different circRNAs in conjunctival melanoma tissues compared with adjacent normal tissues. CircMTUS1 was upregulated in conjunctival melanoma. Silencing of circMTUS1 inhibited conjunctival melanoma proliferation in vitro and in vivo. CircMTUS1 may serve as an oncogene by binding to hsa-miR-622 and hsa-miR-1208 to regulate several tumor-related pathways. Conclusion: We demonstrated that circMTUS1 may serve as an oncogene to promote tumor proliferation.
  
  
  
  
**MTUS1**
-
3
of
3
  

### Tissue-based microarray expression of genes predictive of metastasis in uveal melanoma and differentially expressed in metastatic uveal melanoma.

3. J Ophthalmic Vis Res. 2013 Oct;8(4):303-7.
  
  
Demirci H(1), Reed D(1), Elner VM(1).
  
  
Author information: (1)Department of Ophthalmology and Visual Sciences, W.K. Kellogg Eye Center, Uni ...
  
  
PURPOSE: To screen the microarray expression of CDH1, ECM1, EIF1B, FXR1, HTR2B, ID2, LMCD1, LTA4H, MTUS1, RAB31, ROBO1, and SATB1 genes which are predictive of primary uveal melanoma metastasis, and NFKB2, PTPN18, MTSS1, GADD45B, SNCG, HHIP, IL12B, CDK4, RPLP0, RPS17, RPS12 genes that are differentially expressed in metastatic uveal melanoma in normal whole human blood and tissues prone to metastatic involvement by uveal melanoma. METHODS: We screened the GeneNote and GNF BioGPS databases for microarray analysis of genes predictive of primary uveal melanoma metastasis and those differentially expressed in metastatic uveal melanoma in normal whole blood, liver, lung and skin. RESULTS: Microarray analysis showed expression of all 22 genes in normal whole blood, liver, lung and skin, which are the most common sites of metastases. In the GNF BioGPS database, data for expression of the HHIP gene in normal whole blood and skin was not complete. CONCLUSIONS: Microarray analysis of genes predicting systemic metastasis of uveal melanoma and genes differentially expressed in metastatic uveal melanoma may not be used as a biomarker for metastasis in whole blood, liver, lung, and skin. Their expression in tissues prone to metastasis may suggest that they play a role in tropism of uveal melanoma metastasis to these tissues.
  
  
  
  
  

---

  

# NFIA

**Signature gene in:** Stromal cells
  
**Differentially expressed in:** Stromal cells
  
  
**NFIA**
-
1
of
3
  

### LncRNA LINC00518 Acts as an Oncogene in Uveal Melanoma by Regulating an RNA-Based Network.

1. Cancers (Basel). 2020 Dec 21;12(12):3867. doi: 10.3390/cancers12123867
  
  
Barbagallo C(1), Caltabiano R(2), Broggi G(2), Russo A(3), Puzzo L(2), Avitabile T(3), Longo A(3), R ...
  
  
Author information: (1)Department of Biomedical and Biotechnological Sciences-Section of Biology and ...
  
  
Uveal melanoma (UM) is the most common primary intraocular malignant tumor in adults; little is known about the contribution of non-coding RNAs (ncRNAs) to UM pathogenesis. Competitive endogenous RNA (ceRNA) networks based on RNA-RNA interactions regulate physiological and pathological processes. Through a combined approach of in silico and experimental biology, we investigated the expression of a set of long non-coding RNAs (lncRNAs) in patient biopsies, identifying LINC00518 as a potential oncogene in UM. The detection of LINC00518 dysregulation associated with several in vitro functional assays allowed us to investigate its ceRNA regulatory network and shed light on its potential involvement in cancer-related processes, such as epithelial to mesenchymal transition (EMT) and CoCl2-induced hypoxia-like response. In vitro transient silencing of LINC00518 impaired cell proliferation and migration, and affected mRNA expression of LINGO2, NFIA, OTUD7B, SEC22C, and VAMP3. A "miRNA sponge" and "miRNA protector" model have been hypothesized for LINC00518-induced regulation of mRNAs. In vitro inhibition of MITF suggested its role as a potential activator of LINC00518 expression. Comprehensively, LINC00518 may be considered a new oncogene in UM and a potential target for RNA-based therapeutic approaches.
  
  
  
  
**NFIA**
-
2
of
3
  

### Novel Methylation Patterns Predict Outcome in Uveal Melanoma.

2. Life (Basel). 2020 Oct 20;10(10):248. doi: 10.3390/life10100248
  
  
Ferrier ST(1), Burnier JV(1)(2)(3).
  
  
Author information: (1)Cancer Research Program, Research Institute of the McGill University Health C ...
  
  
Uveal melanoma (UM) is the most common intraocular tumor in adults. Despite effective local treatments, 50% of patients develop metastasis. Better ways to determine prognosis are needed as well as new therapeutic targets. Epigenetic changes are important events driving cancer progression; however, few studies exist on methylation changes in UM. Our aim was to identify methylation events associated with UM prognosis. Matched clinical, genetic, and methylation data for 80 UM cases were obtained from The Cancer Genome Atlas (TCGA). Top differentially methylated loci were sorted through hierarchical clustering based on methylation patterns, and these patterns were compared to tumor characteristics, genomic aberrations, and patient outcome. Hierarchical clustering revealed two distinct groups. These classifications effectively separated high and low-risk cases, with significant differences between groups in patient survival (p < 0.0001) and correlation with known prognostic factors. Major differences in methylation of specific genes, notably NFIA, HDAC4, and IL12RB2, were also seen. The methylation patterns identified in this study indicate potential novel prognostic indicators of UM and highlight the power of methylation changes in predicting outcome. The methylation events enriched in the high-risk group suggest that epigenetic modulating drugs may be useful in reducing metastatic potential, and that specific differentially methylated loci could act as biomarkers of therapeutic response.
  
  
  
  
**NFIA**
-
3
of
3
  

### Analysis of the regulation of fatty acid binding protein 7 expression in human renal carcinoma cell lines.

3. BMC Mol Biol. 2011 Jul 19;12:31. doi: 10.1186/1471-2199-12-31
  
  
Takaoka N(1), Takayama T, Teratani T, Sugiyama T, Mugiya S, Ozono S.
  
  
Author information: (1)Department of Urology, Hamamatsu University School of Medicine, Hamamatsu, Sh ...
  
  
BACKGROUND: Improving the treatment of renal cell carcinoma (RCC) will depend on the development of better biomarkers for predicting disease progression and aiding the design of appropriate therapies. One such marker may be fatty acid binding protein 7 (FABP7), also known as B-FABP and BLBP, which is expressed normally in radial glial cells of the developing central nervous system and cells of the mammary gland. Melanomas, glioblastomas, and several types of carcinomas, including RCC, overexpress FABP7. The abundant expression of FABP7 in primary RCCs compared to certain RCC-derived cell lines may allow the definition of the molecular components of FABP7's regulatory system. RESULTS: We determined FABP7 mRNA levels in six RCC cell lines. Two were highly expressed, whereas the other and the embryonic kidney cell line (HEK293) were weakly expressed FABP7 transcripts. Western blot analysis of the cell lines detected strong FABP7 expression only in one RCC cell line. Promoter activity in the RCC cell lines was 3- to 21-fold higher than that of HEK293. Deletion analysis demonstrated that three FABP7 promoter regions contributed to upregulated expression in RCC cell lines, but not in the HEK293 cell. Competition analysis of gel shifts indicated that OCT1, OCT6, and nuclear factor I (NFI) bound to the FABP7 promoter region. Supershift experiments indicated that BRN2 (POU3F2) and NFI bound to the FABP7 promoter region as well. There was an inverse correlation between FABP7 promoter activity and BRN2 mRNA expression. The FABP7-positive cell line's NFI-DNA complex migrated faster than in other cell lines. Levels of NFIA mRNA were higher in the HEK293 cell line than in any of the six RCC cell lines. In contrast, NFIC mRNA expression was lower in the HEK293 cell line than in the six RCC cell lines. CONCLUSIONS: Three putative FABP7 promoter regions drive reporter gene expression in RCC cell lines, but not in the HEK293 cell line. BRN2 and NFI may be key factors regulating the expression of FABP7 in certain RCC-derived cell lines.
  
  
  
  
  

---

  

# OMD

**Signature gene in:** Stromal cells
  
**Differentially expressed in:** Stromal cells
  
  
**OMD**
-
1
of
4
  

### Mathematical prediction with pretreatment growth rate of metastatic cancer on outcomes: implications for the characterization of oligometastatic disease.

1. Front Oncol. 2023 May 29;13:1061881. doi: 10.3389/fonc.2023.1061881 eCollection 2023.
  
  
Shin Y(1), Chang JS(2)(3), Kim Y(1), Shin SJ(4), Kim J(2), Kim TH(5), Liu M(3), Olson R(6), Kim JS(7 ...
  
  
Author information: (1)Department of Biomedical Engineering and of Biomedicine & Health Sciences, Co ...
  
  
BACKGROUND: Oligometastatic disease (OMD) represents an indolent cancer status characterized by slow tumor growth and limited metastatic potential. The use of local therapy in the management of the condition continues to rise. This study aimed to investigate the advantage of pretreatment tumor growth rate in addition to baseline disease burden in characterizing OMDs, generally defined by the presence of ≤ 5 metastatic lesions. METHODS: The study included patients with metastatic melanoma treated with pembrolizumab. Gross tumor volume of all metastases was contoured on imaging before (TP-1) and at the initiation of pembrolizumab (TP0). Pretreatment tumor growth rate was calculated by an exponential ordinary differential equation model using the sum of tumor volumes at TP-1 and TP0 and the time interval between TP-1. and TP0. Patients were divided into interquartile groups based on pretreatment growth rate. Overall survival, progression-free survival, and subsequent progression-free survival were the study outcomes. RESULTS: At baseline, median cumulative volume and number of metastases were 28.4 cc (range, 0.4-1194.8 cc) and 7 (range, 1-73), respectively. The median interval between TP-1 and TP0 was -90 days and pretreatment tumor growth rate (×10-2 days-1) was median 4.71 (range -0.62 to 44.1). The slow-paced group (pretreatment tumor growth rate ≤ 7.6 ×10-2 days-1, the upper quartile) had a significantly higher overall survival rate, progression-free survival, and subsequent progression-free survival compared to those of the fast-paced group (pretreatment tumor growth rate > 7.6 ×10-2 days-1). Notably, these differences were prominent in the subgroup with >5 metastases. CONCLUSION: Pretreatment tumor growth rate is a novel prognostic metric associated with overall survival, progression-free survival, and subsequent progression-free survival among metastatic melanoma patients, especially patients with >5 metastases. Future prospective studies should validate the advantage of disease growth rate plus disease burden in better defining OMDs.
  
  
  
  
**OMD**
-
2
of
4
  

### A Comparison of 2 Disease Burden Assessment Methods (3D Volume Versus the Number of Lesions) for Prognostication of Survival in Metastatic Melanoma: Implications for the Characterization of Oligometastatic Disease.

2. Int J Radiat Oncol Biol Phys. 2022 Dec 1;114(5):883-891. doi: 10.1016/j.ijrobp.2022.08.040 Epub 2022 Aug 23.
  
  
Kim J(1), Chang JS(2), Sung W(3), Kim JS(4), Kim TH(5), Choi SH(6), Kim KH(1), Ko H(7), Lee HS(8), J ...
  
  
Author information: (1)Department of Radiation Oncology, Yonsei Cancer Center, Heavy Ion Therapy Res ...
  
  
PURPOSE: Oligometastatic disease (OMD), generally defined by the presence of ≤5 metastatic lesions, represents an intermediate state between localized and widespread metastatic disease. This study aimed to question the conventional definition of OMD and assess the significance of the total volume and loci of metastases in characterizing OMD using an unselected metastatic melanoma cohort. METHODS AND MATERIALS: We identified 86 consecutive patients with metastatic melanoma who received pembrolizumab monotherapy from 2015 to 2020. We retrospectively contoured the gross tumor volumes of all metastatic lesions on baseline and follow-up imaging. The number, total volume, and loci information of metastases was collected. The primary endpoint was overall survival. A density histogram plot was used for tumor characteristic descriptions, and classification analysis using the decision tree and random forest methods was performed to determine the optimal combination of prognostic factors in the clinical setting. RESULTS: A total of 2728 gross tumor volumes were delineated. On baseline imaging, the median number and total volume of metastases was 7 (interquartile range, 3-17) and 28.4 cc (interquartile range, 8.4-88.78), respectively. The lymph node was the most common metastatic site (n = 46, 54%), followed by the lungs (n = 32, 37%), liver (n = 23, 27%), and bones (n = 21, 24%). Two-year overall survival rates of patients with 1 to 5, 6 to 10, 11 to 20, and >20 metastases were 58%, 47%, 31%, and 14%, respectively, and with ≤10, 11 to 30, 31 to 130, and >130 cc of metastatic volume were 64%, 43%, 33%, and 25%, respectively. K-adaptive partitioning revealed that the optimal cutoff was 20 and 37.9 cc. Decision tree and random forest analyses revealed that volume and loci (brain and liver metastases) were the most important factors (Harrell's C-index, 0.78). CONCLUSIONS: The OMD state could represent a continuous spectrum of disease burden instead of a binary phenomenon. We propose integrating the volumetric and spatial information of metastases into the characterization of OMD and the stratification tool of clinical trials in the metastatic setting, although external validation studies are needed.
  
  
  
  
**OMD**
-
3
of
4
  

### Safety and Efficacy of Checkpoint Inhibition in Patients With Melanoma and Preexisting Autoimmune Disease : A Cohort Study.

3. Ann Intern Med. 2021 May;174(5):641-648. doi: 10.7326/M20-3419 Epub 2021 Feb 16.
  
  
van der Kooij MK(1), Suijkerbuijk KPM(2), Aarts MJB(3), van den Berkmortel FWPJ(4), Blank CU(5), Boe ...
  
  
Author information: (1)Leiden University Medical Center, Leiden, the Netherlands (M.K.V., O.M.D., E. ...
  
  
Comment in Ann Intern Med. 2021 Sep;174(9):1345-1346. doi: 10.7326/L21-0442. Ann Intern Med. 2021 Sep;174(9):1345. doi: 10.7326/L21-0441.
  
  
  
  
**OMD**
-
4
of
4
  

### Local treatment of oligometastatic disease: current role.

4. Br J Radiol. 2019 Aug;92(1100):20180835. doi: 10.1259/bjr.20180835 Epub 2019 Jun 6.
  
  
Winkelmann MT(1), Clasen S(1), Pereira PL(2), Hoffmann R(1).
  
  
Author information: (1)1 Department of Diagnostic and Interventional Radiology, University Hospital ...
  
  
The presence of distant metastases has long been a predictor of poor outcome in solid cancer. However, in an oncologic situation called oligometastatic disease (OMD), multiple studies have revealed a survival benefit with aggressive treatment of these metastases. Besides surgery and radiation therapy, local thermal therapies have developed into a treatment option for OMD. Most studies concerning local therapy of OMD are available for colorectal cancer, which is therefore the focus of this article. Furthermore, this review gives a basic overview of the most popular ablation techniques for treatment of OMD.
  
  
  
  
  

---

  

# OPRM1

**Signature gene in:** Tregs
  
**Differentially expressed in:** Tregs
  
  
**OPRM1**
-
1
of
3
  

### Pan-Cancer Analysis and Validation of Opioid-Related Receptors Reveals the Immunotherapeutic Value of Toll-Like Receptor 4.

1. Int J Gen Med. 2023 Nov 24;16:5527-5548. doi: 10.2147/IJGM.S434076 eCollection 2023.
  
  
Wu S(1), Chen J(1), Huang E(2)(3)(4), Zhang Y(1).
  
  
Author information: (1)Department of Anesthesiology, the Second Affiliated Hospital, Shantou Univers ...
  
  
INTRODUCTION: The relationship between the expression of opioid-associated receptors and cancer outcomes is complex and varies among studies. METHODS: This study focused on six opioid-related receptors (OPRM1, OPRD1, OPRK1, OPRL1, OGFR, and TLR4) and their impact on cancer patient survival. Bioinformatics analysis was conducted on 33 cancer types from The Cancer Genome Atlas database to examine their expression, clinical correlations, mechanisms in the tumor microenvironment, and potential for immunotherapy. Due to significantly lower expression of OPRM1, OPRD1, and OPRK1 compared to OGFR and TLR4, the analysis concentrated on the latter two genes. RESULTS: OGFR was highly expressed in 16 tumor types, while TLR4 showed low expression in 13. Validation from external samples, the Gene Expression Omnibus, and the Human Protein Atlas supported these findings. The diagnostic value of these two genes was demonstrated using the Genotype-Tissue Expression database. Univariate Cox regression models and Kaplan-Meier curves confirmed OGFR's impact on prognosis in a cancer type-specific manner, while high TLR4 expression was associated with a favorable prognosis. Analysis of the tumor microenvironment using a deconvolution algorithm linked OGFR to CD8+ T cells and TLR4 to macrophages. Single-cell datasets further validated this correlation. In 25 immune checkpoint blockade treatment cohorts, TLR4 expression showed promise as an immunotherapy efficacy predictor in non-small cell lung cancer, urothelial carcinoma, and melanoma. CONCLUSION: In a pan-cancer analysis of 33 tissues, OGFR was consistently highly expressed, while TLR4 had low expression. Both genes have diagnostic and prognostic significance and are linked to immune cells in the tumor microenvironment. TLR4 has potential as an immunotherapeutic response marker.
  
  
  
  
**OPRM1**
-
2
of
3
  

### Methadone-Not a magic bullet in melanoma therapy.

2. Exp Dermatol. 2018 Jun;27(6):694-696. doi: 10.1111/exd.13543
  
  
Brüggen MC(1)(2), Mangana J(1)(2), Irmisch A(1)(2), French LE(1)(2), Levesque MP(1)(2), Cheng PF(1)( ...
  
  
Author information: (1)Department of Dermatology, Zurich University Hospital, Zurich, Switzerland. ( ...
  
  
Methadone (Met) mainly acts as a μ-opioid receptor agonist. Recent evidence pointing towards the role of Met in sensitization of certain cancer cell lines to chemotherapeutic agents has promoted the hypothesis that Met may be a useful adjuvant to cancer chemotherapy. We wanted to address whether Met has, alone or in combination with a chemotherapeutic agent, an effect on melanoma cell viability in vitro. Only a small fraction (4.3%) of our 102 melanoma biobank cell lines with RNA-sequencing data showed expression of the main receptor for Met (OPRM1). We assessed the viability of melanoma cell lines with high, medium or low/no OPRM1 expression (OPRM1high , OPRM1med , OPRM1neg ) 72 hours after treatment with Met alone or combined with cisplatin (Cis). Our analyses show that Met alone did not affect cell viability. While Cis/Met treatment did not have an effect on viability of OPRM1med or OPRM1neg cell lines, it resulted in a slightly decreased cell viability of OPRM1high cells. Clinically, concurrent temozolomide/Met treatment did not have an effect in our single-case report of a patient suffering from uveal melanoma. Taken together, our findings do not provide evidence for recommending Met as an adjuvant to chemotherapy in patients with melanoma.
  
  
  
  
**OPRM1**
-
3
of
3
  

### PLoS One. 2008 Feb 6;3(2):e1554. doi: 10.1371/journal.pone.0001554.

3. RETRACTED ARTICLE
  
  
High-affinity naloxone binding to filamin a prevents mu opioid receptor-Gs coupling underlying opioi ...
  
  
Wang HY(1), Frankfurt M, Burns LH.
  
  
Author information: (1)Department of Physiology and Pharmacology, City University of New York Medical School, New York, New York, USA. hywang@sci.ccny.cuny.edu
  
  
  
  
  

---

  

# P4HA2

**Signature gene in:** Stromal cells
  
**Differentially expressed in:** Stromal cells
  
  
**P4HA2**
-
1
of
1
  

### Targeting HIF-activated collagen prolyl 4-hydroxylase expression disrupts collagen deposition and blocks primary and metastatic uveal melanoma growth.

1. Oncogene. 2021 Aug;40(33):5182-5191. doi: 10.1038/s41388-021-01919-x Epub 2021 Jul 3.
  
  
Kaluz S(#)(1)(2), Zhang Q(#)(3)(4), Kuranaga Y(5), Yang H(3), Osuka S(1)(5)(6), Bhattacharya D(1), D ...
  
  
Author information: (1)Department of Neurosurgery, Emory University, Atlanta, GA, USA. (2)Winship Ca ...
  
  
Uveal melanoma (UM) is the most prevalent primary intraocular malignancy in adults, and patients that develop metastases (~50%) survive <1 year, highlighting the urgent need for new therapies. TCGA has recently revealed that a hypoxia gene signature is associated with poor UM patient prognosis. Here we show that expression of hypoxia-regulated collagen prolyl-4-hydroxylase genes P4HA1 and P4HA2 is significantly upregulated in UM patients with metastatic disease and correlates with poor prognosis, suggesting these enzymes might be key tumor drivers. We targeted hypoxia-induced expression of P4HA1/2 in UM with KCN1, a hypoxia inducible factor-1 (HIF-1) pathway inhibitor and found potent inhibition of primary and metastatic disease and extension of animal survival, without overt side effects. At the molecular level, KCN1 antagonized hypoxia-induced expression of P4HA1 and P4HA2, which regulate collagen maturation and deposition in the extracellular matrix. The treatment decreased prolyl hydroxylation, induced proteolytic cleavage and rendered a disordered structure to collagen VI, the main collagen produced by UM, and reduced UM cell invasion. Together, these data demonstrate that extracellular collagen matrix formation can be targeted in UM by inhibiting hypoxia-induced P4HA1 and P4HA2 expression, warranting further development of this strategy in patients with uveal melanoma.
  
  
  
  
  

---

  

# PAG1

**Signature gene in:** Stromal cells
  
**Differentially expressed in:** Stromal cells
  
  
**PAG1**
-
1
of
4
  

### Single cell sequencing analysis constructed the N7-methylguanosine (m7G)-related prognostic signature in uveal melanoma.

1. Aging (Albany NY). 2023 Mar 14;15(6):2082-2096. doi: 10.18632/aging.204592 Epub 2023 Mar 14.
  
  
Xie J(1), Chen L(2), Cao Y(3), Ma C(4), Zhao W(5), Li J(6), Yao W(3), Hu Y(7), Wang M(1), Shi J(1).
  
  
Author information: (1)Department of Burn and Plastic Surgery, The First Affiliated Hospital of Nanj ...
  
  
BACKGROUND: Uveal melanoma is a highly malignant tumor in the eye. Its recurrence and metastasis are common, and the prognosis is poor. METHODS: The transcriptome data of UVM were downloaded from TCGA database, and the single cell sequencing dataset GSE139829 was downloaded from GEO database. Weighted co-expression network analysis was used to explore the modules associated with m7G. Lasso regression was used to construct M7G-related prognostic signature. Immune infiltration analysis was used to explore the significance of the model in the tumor immune microenvironment. Finally, cell assays were used to explore the function of key genes in the MUM-2B and OCM-1 cell lines of UVM. RESULTS: The prognostic signature was constructed by Cox regression and Lasso regression. Patients could be divided into high-risk group and low-risk group by this signature, and the high-risk group had worse prognosis (P<0.05). Cell experiments showed that the proliferation, invasion and migration ability of UVM cell lines were significantly decreased after the knockdown of PAG1, a key gene in signature, which proved that PAG1 might be a potential target of UVM. CONCLUSIONS: Our study explored the significance of m7G in UVM, provided biomarkers for its diagnosis and treatment.
  
  
  
  
**PAG1**
-
2
of
4
  

### Exosome-dependent immune surveillance at the metastatic niche requires BAG6 and CBP/p300-dependent acetylation of p53.

2. Theranostics. 2019 Aug 14;9(21):6047-6062. doi: 10.7150/thno.36378 eCollection 2019.
  
  
Schuldner M(1)(2), Dörsam B(1), Shatnyeva O(2), Reiners KS(2)(3), Kubarenko A(3), Hansen HP(2), Fink ...
  
  
Author information: (1)Experimental Tumor Research, Center for Tumor Biology and Immunology, Clinic ...
  
  
Extracellular vesicles released by tumor cells contribute to the reprogramming of the tumor microenvironment and interfere with hallmarks of cancer including metastasis. Notably, melanoma cell-derived EVs are able to establish a pre-metastatic niche in distant organs, or on the contrary, exert anti-tumor activity. However, molecular insights into how vesicles are selectively packaged with cargo defining their specific functions remain elusive. Methods: Here, we investigated the role of the chaperone Bcl2-associated anthogene 6 (BAG6, synonym Bat3) for the formation of pro- and anti-tumor EVs. EVs collected from wildtype cells and BAG6-deficient cells were characterized by mass spectrometry and RNAseq. Their tumorigenic potential was analyzed using the B-16V transplantation mouse melanoma model. Results: We demonstrate that EVs from B-16V cells inhibit lung metastasis associated with the mobilization of Ly6Clow patrolling monocytes. The formation of these anti-tumor-EVs was dependent on acetylation of p53 by the BAG6/CBP/p300-acetylase complex, followed by recruitment of components of the endosomal sorting complexes required for transport (ESCRT) via a P(S/T)AP double motif of BAG6. Genetic ablation of BAG6 and disruption of this pathway led to the release of a distinct EV subtype, which failed to suppress metastasis but recruited tumor-promoting neutrophils to the pre-metastatic niche. Conclusion: We conclude that the BAG6/CBP/p300-p53 axis is a key pathway directing EV cargo loading and thus a potential novel microenvironmental therapeutic target.
  
  
  
  
**PAG1**
-
3
of
4
  

### IFN-γ inhibits basal and α-MSH-induced melanogenesis.

3. Pigment Cell Melanoma Res. 2014 Mar;27(2):201-8. doi: 10.1111/pcmr.12190 Epub 2013 Nov 25.
  
  
Son J(1), Kim M, Jou I, Park KC, Kang HY.
  
  
Author information: (1)Department of Pharmacology, Ajou University School of Medicine, Suwon, Korea; ...
  
  
Inflammatory cytokines are closely related to pigmentary changes. In this study, the effects of IFN-γ on melanogenesis were investigated. IFN-γ inhibits basal and α-MSH-induced melanogenesis in B16 melanoma cells and normal human melanocytes. MITF mRNA and protein expressions were significantly inhibited in response to IFN-γ. IFN-γ inhibited CREB binding to the MITF promoter but did not affect CREB phosphorylation. Instead, IFN-γ inhibited the association of CBP and CREB through the increased association between CREB binding protein (CBP) and STAT1. These findings suggest that IFN-γ inhibits both basal and α-MSH-induced melanogenesis by inhibiting MITF expression. The inhibitory action of IFN-γ in α-MSH-induced melanogenesis is likely to be associated with the sequestration of CBP via the association between CBP and STAT1. These data suggest that IFN-γ plays a role in controlling inflammation- or UV-induced pigmentary changes.
  
  
  
  
**PAG1**
-
4
of
4
  

### Melanomas and melanoma cell lines do not express HLA-G, and the expression cannot be induced by gammaIFN treatment.

4. Tissue Antigens. 2000 Jul;56(1):30-7. doi: 10.1034/j.1399-0039.2000.560104.x
  
  
Frumento G(1), Franchello S, Palmisano GL, Nicotra MR, Giacomini P, Loke YW, Geraghty DE, Maio M, Ma ...
  
  
Author information: (1)Laboratory of Immunogenetics, IST National Institute for Cancer Research, Gen ...
  
  
HLA-G is an effective ligand of natural killer (NK) inhibitory receptors, HLA-G transcripts have been detected in several human tumors, and cytokines like gamma interferon (IFN) enable HLA-G molecules to be expressed. These findings are particularly upsetting in case of melanomas: IFN treatment is frequently included in melanoma therapeutic protocols, and downregulation of classical class I molecules occurs in nearly half of these tumors. Therefore, a melanoma cell downregulating classical class I and de novo expressing HLA-G, either constitutively or upon IFN treatment, is probably a stealthy target for the immune system, having inhibited both the cytotoxic T lymphocyte (CTL) and the NK activity. To elucidate this point we have investigated the expression of HLA-G molecules in 45 melanoma cell lines before and after gammaIFN treatment. Analysis was performed by immunofluorescence and flow cytometry, using the anti-HLA-G MoAbs 87G and G233, by Western blot, using the anti-HLA-G MEM/G1 MoAb and PAG1 antiserum, and by RT-PCR analysis. In addition, 8 melanoma tissues from patients free from therapy and 6 nevi were studied by immunohistochemistry using the 87G MoAb. No evidence was gathered of HLA-G expression, neither constitutive nor, in cell lines, after gammaIFN treatment. We therefore conclude that HLA-G expression is an uncommon event in melanomas, and that a therapy including IFNs cannot harm the patient by inducing the de novo expression of HLA-G molecules at least in its G1 isoform.
  
  
  
  
  

---

  

# PARP4

**Signature gene in:** CD8+ T cells
  
**Differentially expressed in:** CD8+ T cells
  
  
**PARP4**
-
1
of
1
  

### PARP4 deficiency enhances sensitivity to ATM inhibitor by impairing DNA damage repair in melanoma.

1. Cell Death Discov. 2025 Jan 30;11(1):35. doi: 10.1038/s41420-025-02296-0
  
  
Li Y(#)(1), Liu Y(#)(1), Ma J(1), Yang Y(1), Yue Q(1), Zhu G(1), Guo W(1), Gao T(1), Shi Q(2), Li C( ...
  
  
Author information: (1)Department of Dermatology, Xijing Hospital, Fourth Military Medical Universit ...
  
  
Besides the important pathogenic mechanisms of melanoma, including BRAF-driven and immunosuppressive microenvironment, genomic instability and abnormal DNA double-strand breaks (DSB) repair are significant driving forces for its occurrence and development. This suggests investigating novel therapeutic strategies from the synthetic lethality perspective. Poly (ADP-ribose) polymerase 4 (PARP4) is known to be a member of the PARP protein family. The low expression of PARP4 is significantly associated with defective DSB repair markers and poor prognosis in melanoma. Further research revealed that PARP4 plays a role in DSB repair by regulating the non-homologous end joining (NHEJ) pathway through its involvement in Ku80 mono-ADP-ribosylation. Moreover, from a synthetic lethality perspective, PARP4 expression is associated with ATM inhibitor sensitivity. Overall, our study provides new and valuable insights into the function of PARP4 and melanoma pathogenesis and suggests that ATM inhibitor may be a promising therapeutic approach for treating melanoma with low PARP4 expression.
  
  
  
  
  

---

  

# PARP8

**Signature gene in:** CD4+ T cells, CD8+ T cells, Tregs
  
**Differentially expressed in:** CD8+ T cells
  
  
**PARP8**
-
1
of
1
  

### Identification and validation of immunogenic cell death-related score in uveal melanoma to improve prediction of prognosis and response to immunotherapy.

1. Aging (Albany NY). 2023 May 3;15(9):3442-3464. doi: 10.18632/aging.204680 Epub 2023 May 3.
  
  
Li X(1)(2), Kang J(3), Yue J(3), Xu D(2), Liao C(4), Zhang H(5), Zhao J(5), Liu Q(5), Jiao J(5), Wan ...
  
  
Author information: (1)Department of Central Laboratory, Shanxi Provincial People’s Hospital, Taiyua ...
  
  
BACKGROUND: Immunogenic cell death (ICD) could activate innate and adaptive immune response. In this work, we aimed to develop an ICD-related signature in uveal melanoma (UVM) patients and facilitate assessment of their prognosis and immunotherapy. METHODS: A set of machine learning methods, including non-negative matrix factorization (NMF) method and least absolute shrinkage and selection operator (LASSO) logistic regression model, and bioinformatics analytic tools were integrated to construct an ICD-related risk score (ICDscore). CIBERSORT and ESTIMATE algorithms were used to evaluate the infiltration of immune cells. The Genomics of Drug Sensitivity in Cancer (GDSC), cellMiner and tumor immune dysfunction and exclusion (TIDE) databases were used for therapy sensitivity analyses. The predictive performance between ICDscore with other mRNA signatures was also compared. RESULTS: The ICDscore could predict the prognosis of UVM patients in both the training and four validating cohorts. The ICDscore outperformed 19 previously published signatures. Patients with high ICDscore exhibited a substantial increase in immune cell infiltration and expression of immune checkpoint inhibitor-related genes, leading to a higher response rate to immunotherapy. Furthermore, the downregulation of poly (ADP-ribose) polymerase family member 8 (PARP8), a critical gene involved in the development of the ICDscore, resulted in decreased cell proliferation and slower migration of UVM cells. CONCLUSION: In conclusion, we developed a robust and powerful ICD-related signature for evaluating the prognosis and benefits of immunotherapy that could serve as a promising tool to guide decision-making and surveillance for UVM patients.
  
  
  
  
  

---

  

# PDGFC

**Signature gene in:** MDM
  
**Differentially expressed in:** MDM
  
  
**PDGFC**
-
1
of
7
  

### Pharmacological inhibition of PDGF-C/neuropilin-1 interaction: A novel strategy to reduce melanoma metastatic potential.

1. Biomed Pharmacother. 2024 Jul;176:116766. doi: 10.1016/j.biopha.2024.116766 Epub 2024 May 23.
  
  
Ceci C(1), Ruffini F(2), Falconi M(3), Atzori MG(1), Falzon A(1), Lozzi F(1), Iacovelli F(3), D'Atri ...
  
  
Author information: (1)Department of Systems Medicine, University of Rome Tor Vergata, Rome, Italy. ...
  
  
Activation of neuropilin-1 (NRP-1) by platelet derived growth factor (PDGF)-C sustains melanoma invasiveness. Therefore, in the search of novel agents capable of reducing melanoma spreading, PDGF-C/NRP-1 interaction was investigated as a potential druggable target. Since the PDGF-C region involved in NRP-1 binding is not yet known, based on the sequence and structural homology between PDGF-C and vascular endothelial growth factor-A (VEGF-A), we hypothesized that the NRP-1 b1 domain region involved in the interaction with VEGF-A might also be required for PDGF-C binding. Hence, this region was selected from the protein crystal structure and used as target in the molecular docking procedure. In the following virtual screening, compounds from a DrugBank database were used as query ligands to identify agents potentially capable of disrupting NRP-1/PDGF-C interaction. Among the top 45 candidates with the highest affinity, five drugs were selected based on the safety profile, lack of hormonal effects, and current availability in the market: the antipsychotic pimozide, antidiabetic gliclazide, antiallergic cromolyn sodium, anticancer tyrosine kinase inhibitor entrectinib, and antihistamine azelastine. Analysis of drug influence on PDGF-C in vitro binding to NRP-1 and PDGF-C induced migration of human melanoma cells expressing NRP-1, indicated gliclazide and entrectinib as the most specific agents that were active at clinically achievable and non-toxic concentrations. Both drugs also reverted PDGF-C ability to stimulate extracellular matrix invasion by melanoma cells resistant to BRAF inhibitors. The inhibitory effect on tumor cell motility involved a decrease of p130Cas phosphorylation, a signal transduction pathway activated by PDGF-C-mediated stimulation of NRP-1.
  
  
  
  
**PDGFC**
-
2
of
7
  

### Targeting of PDGF-C/NRP-1 autocrine loop as a new strategy for counteracting the invasiveness of melanoma resistant to braf inhibitors.

2. Pharmacol Res. 2023 Jun;192:106782. doi: 10.1016/j.phrs.2023.106782 Epub 2023 Apr 29.
  
  
Ruffini F(1), Ceci C(2), Atzori MG(1), Caporali S(3), Levati L(3), Bonmassar L(3), Cappellini GCA(4) ...
  
  
Author information: (1)Department of Systems Medicine, University of Rome Tor Vergata, Rome, Italy. ...
  
  
Melanoma resistance to BRAF inhibitors (BRAFi) is often accompanied by a switch from a proliferative to an invasive phenotype. Therefore, the identification of signaling molecules involved in the development of metastatic properties by resistant melanoma cells is of primary importance. We have previously demonstrated that activation of neuropilin-1 (NRP-1) by platelet-derived growth factor (PDGF)-C confers melanoma cells with an invasive behavior similar to that of BRAFi resistant tumors. Aims of the present study were to evaluate the role of PDGF-C/NRP-1 autocrine loop in the acquisition of an invasive and BRAFi-resistant phenotype by melanoma cells and the effect of its inhibition on drug resistance and extracellular matrix (ECM) invasion. Furthermore, we investigated whether PDGF-C serum levels were differentially modulated by drug treatment in metastatic melanoma patients responsive or refractory to BRAFi as single agents or in combination with MEK inhibitors (MEKi). The results indicated that human melanoma cells resistant to BRAFi express higher levels of PDGF-C and NRP-1 as compared to their susceptible counterparts. Overexpression occurs early during development of drug resistance and contributes to the invasive properties of resistant cells. Accordingly, silencing of NRP-1 or PDGF-C reduces tumor cell invasiveness. Analysis of PDGF-C in the serum collected from patients treated with BRAFi or BRAFi+MEKi, showed that in responders PDGF-C levels decrease after treatment and raise again at tumor progression. Conversely, in non-responders treatment does not affect PDGF-C serum levels. Thus, blockade of NRP-1 activation by PDGF-C might represent a new therapeutic approach to counteract the invasiveness of BRAFi-resistant melanoma.
  
  
  
  
**PDGFC**
-
3
of
7
  

### Platelet-derived growth factor-C promotes human melanoma aggressiveness through activation of neuropilin-1.

3. Oncotarget. 2017 Jun 27;8(40):66833-66848. doi: 10.18632/oncotarget.18706 eCollection 2017 Sep 15.
  
  
Ruffini F(1), Levati L(1), Graziani G(2), Caporali S(1), Atzori MG(2), D'Atri S(1), Lacal PM(1).
  
  
Author information: (1)Laboratory of Molecular Oncology, "Istituto Dermopatico dell'Immacolata"-IRCC ...
  
  
Despite recent progress in advanced melanoma therapy, identification of signalling pathways involved in melanoma switch from proliferative to invasive states is still crucial to uncover new therapeutic targets for improving the outcome of metastatic disease. Neuropilin-1 (NRP-1), a co-receptor for vascular endothelial growth factor-A (VEGF-A) tyrosine kinase receptors (VEGFRs), has been suggested to play a relevant role in melanoma progression. NRP-1 can be activated by VEGF-A also in the absence of VEGFRs, triggering specific signal transduction pathways (e.g. p130Cas phosphorylation). Since melanoma cells co-expressing high levels of NRP-1 and platelet derived growth factor-C (PDGF-C) show a highly invasive behaviour and PDGF-C shares homology with VEGF-A, in this study we have investigated whether PDGF-C directly interacts with NRP-1 and promotes melanoma aggressiveness. Results demonstrate that PDGF-C specifically binds in vitro to NRP-1. In melanoma cells expressing NRP-1 but lacking PDGFRα, PDGF-C stimulates extra-cellular matrix (ECM) invasion and induces p130Cas phosphorylation. Blockade of PDGF-C function by neutralizing antibodies or reduction of its secretion by specific siRNA inhibit ECM invasion and vasculogenic mimicry. Moreover, PDGF-C silencing significantly down-modulates the expression of Snail, a transcription factor involved in tumour invasiveness that is highly expressed in NRP-1 positive melanoma cells. In conclusion, our results demonstrate for the first time a direct activation of NRP-1 by PDGF-C and strongly suggest that autocrine and/or paracrine stimulation of NRP-1 by PDGF-C might contribute to the acquisition of a metastatic phenotype by melanoma cells.
  
  
  
  
**PDGFC**
-
4
of
7
  

### Platelet-derived growth factor C and calpain-3 are modulators of human melanoma cell invasiveness.

4. Oncol Rep. 2013 Dec;30(6):2887-96. doi: 10.3892/or.2013.2791 Epub 2013 Oct 10.
  
  
Ruffini F(1), Tentori L, Dorio AS, Arcelli D, D'Amati G, D'Atri S, Graziani G, Lacal PM.
  
  
Author information: (1)Laboratory of Molecular Oncology, 'Istituto Dermopatico dell'Immacolata'- IRC ...
  
  
The molecular mechanisms responsible for the elevated metastatic potential of malignant melanoma are still not fully understood. In order to shed light on the molecules involved in the acquisition by melanoma of a highly aggressive phenotype, we compared the gene expression profiles of two cell clones derived from the human cutaneous metastatic melanoma cell line M14: a highly invasive clone (M14C2/MK18) and a clone (M14C2/C4) with low ability to invade the extracellular matrix (ECM). The highly invasive phenotype of M14C2/MK18 cells was correlated with overexpression of neuropilin-1, activation of a vascular endothelial growth factor (VEGF)-A/VEGFR-2 autocrine loop and secretion of matrix metalloprotease-2. Moreover, in an in vivo murine model, M14C2/MK18 cells displayed a higher growth rate as compared with M14C2/C4 cells, even though in vitro both clones possessed comparable proliferative potential. Microarray analysis in M14C2/MK18 cells showed a strong upregulation of platelet-derived growth factor (PDGF)-C, a cytokine that contributes to angiogenesis, and downregulation of calpain-3, a calcium-dependent thiol-protease that regulates specific signalling cascade components. Inhibition of PDGF-C with a specific antibody resulted in a significant decrease in ECM invasion by M14C2/MK18 cells, confirming the involvement of PDGF-C in melanoma cell invasiveness. Moreover, the PDGF-C transcript was found to be upregulated in a high percentage of human melanoma cell lines (17/20), whereas only low PDGF-C levels were detected in a few melanocytic cultures (2/6). By contrast, inhibition of calpain-3 activity in M14C2/C4 control cells, using a specific chemical inhibitor, markedly increased ECM invasion, strongly suggesting that downregulation of calpain-3 plays a role in the acquisition of a highly invasive phenotype. The results indicate that PDGF-C upregulation and calpain-3 downregulation are involved in the aggressiveness of malignant melanoma and suggest that modulators of these proteins or their downstream effectors may synergise with VEGF‑A therapies in combating tumour-associated angiogenesis and melanoma spread.
  
  
  
  
**PDGFC**
-
5
of
7
  

### MET expression in melanoma correlates with a lymphangiogenic phenotype.

5. Hum Mol Genet. 2012 Aug 1;21(15):3387-96. doi: 10.1093/hmg/dds171 Epub 2012 May 7.
  
  
Swoboda A(1), Schanab O, Tauber S, Bilban M, Berger W, Petzelbauer P, Mikula M.
  
  
Author information: (1)Skin and Endothelium Research Division, Department of Dermatology, Medical Un ...
  
  
Melanomas contain high frequencies of tumorigenic cells and their tumorigenic capacity resides in several distinct subpopulations within melanoma. Since their metastatic potential is linked to their ability to recruit lymphatic vessels, we aimed at identifying lymphangiogenic subpopulations by comparative in vitro analysis of single cell clones derived from a melanoma of a single patient. Selected lymphangiogenic clones were then grafted into severe combined immunodeficient mice, where they induced lymphangiogenesis and metastasized into sentinel nodes, whereas non-lymphangiogenic clones from the same patient did not metastasize. Transcriptome analysis revealed high expression of vascular endothelial growth factor C (VEGF-C) and platelet derived growth factor C (PDGF-C) as well as of the met proto-oncogene (MET) and its targets to be associated with this lymphangiogenic phenotype. Screening of a set of independently isolated melanoma cell lines from other patients confirmed this association between expression of high levels of MET and of VEGF-C and PDGF-C. Hence, we provide a model to screen for the lymphangiogenic potential of tumor cells. We show that the lymphangiogenic potential is heterogeneously distributed among melanoma cells within one given tumor and is associated with activation of MET signaling.
  
  
  
  
**PDGFC**
-
6
of
7
  

### Melanoma cells control HA synthesis in peritumoral fibroblasts via PDGF-AA and PDGF-CC: impact on melanoma cell proliferation.

6. J Invest Dermatol. 2012 Feb;132(2):385-93. doi: 10.1038/jid.2011.325 Epub 2011 Oct 13.
  
  
Willenberg A(1), Saalbach A, Simon JC, Anderegg U.
  
  
Author information: (1)Department of Dermatology, Venerology and Allergology, University of Leipzig, ...
  
  
The microenvironment surrounding tumors has an important role in tumor progression. Fibroblasts (Fbs) of the tumor stroma receive signals from tumors and transform the extracellular matrix, thus supporting tumor growth, motility, and metastasis. The matrix component hyaluronan (HA) has a pivotal role in tumor progression. Here we analyzed the cell populations that synthesize HA in human malignant melanoma (MM) cells and studied the regulatory network between MM cells and stromal Fbs controlling HA synthesis. Tissue analysis indicated that Fbs are the main source of HA in the stroma of melanoma, whereas MM themselves synthesize only minute amounts of HA. In vitro, Fb-derived HA is mainly produced by hyaluronan synthase 2 (HAS2) and enhances proliferation of MM. Proteins secreted by MM can further increase HA synthesis in Fbs in a phosphatidylinositol 3/mitogen-activated protein-kinase-dependent manner. Melanoma cell-derived platelet-derived growth factor (PDGF)-AA and PDGF-CC were identified as major mediators that signal through PDGFR-α and thus induce HAS2-mediated HA synthesis in Fbs. In conclusion, we have identified a complex interaction of MM with its surrounding microenvironment by demonstrating that MM by the release of PDGF-AA and PDGF-CC upregulate HA synthesis in Fbs, which in turn stimulates MM proliferation in a paracrine manner.
  
  
  
  
**PDGFC**
-
7
of
7
  

### Paracrine signaling by platelet-derived growth factor-CC promotes tumor growth by recruitment of cancer-associated fibroblasts.

7. Cancer Res. 2009 Jan 1;69(1):369-78. doi: 10.1158/0008-5472.CAN-08-2724
  
  
Anderberg C(1), Li H, Fredriksson L, Andrae J, Betsholtz C, Li X, Eriksson U, Pietras K.
  
  
Author information: (1)Ludwig Institute for Cancer Research Ltd., Stockholm Branch, Nobels Väg 3, St ...
  
  
Cancer results from the concerted performance of malignant cells and stromal cells. Cell types populating the microenvironment are enlisted by the tumor to secrete a host of growth-promoting cues, thus upholding tumor initiation and progression. Platelet-derived growth factors (PDGF) support the formation of a prominent tumor stromal compartment by as of yet unidentified molecular effectors. Whereas PDGF-CC induces fibroblast reactivity and fibrosis in a range of tissues, little is known about the function of PDGF-CC in shaping the tumor-stroma interplay. Herein, we present evidence for a paracrine signaling network involving PDGF-CC and PDGF receptor-alpha in malignant melanoma. Expression of PDGFC in a mouse model accelerated tumor growth through recruitment and activation of different subsets of cancer-associated fibroblasts. In seeking the molecular identity of the supporting factors provided by cancer-associated fibroblasts, we made use of antibody arrays and an in vivo coinjection model to identify osteopontin as the effector of the augmented tumor growth induced by PDGF-CC. In conclusion, we establish paracrine signaling by PDGF-CC as a potential drug target to reduce stromal support in malignant melanoma.
  
  
  
  
  

---

  

# PDIA6

**Signature gene in:** Stromal cells
  
**Differentially expressed in:** Stromal cells
  
  
**PDIA6**
-
1
of
3
  

### Melanoma RBPome identification reveals PDIA6 as an unconventional RNA-binding protein involved in metastasis.

1. Nucleic Acids Res. 2022 Aug 12;50(14):8207-8225. doi: 10.1093/nar/gkac605
  
  
Mestre-Farràs N(1), Guerrero S(1), Bley N(2), Rivero E(1), Coll O(1), Borràs E(1)(3), Sabidó E(1)(3) ...
  
  
Author information: (1)Centre for Genomic Regulation (CRG), The Barcelona Institute of Science and T ...
  
  
RNA-binding proteins (RBPs) have been relatively overlooked in cancer research despite their contribution to virtually every cancer hallmark. Here, we use RNA interactome capture (RIC) to characterize the melanoma RBPome and uncover novel RBPs involved in melanoma progression. Comparison of RIC profiles of a non-tumoral versus a metastatic cell line revealed prevalent changes in RNA-binding capacities that were not associated with changes in RBP levels. Extensive functional validation of a selected group of 24 RBPs using five different in vitro assays unveiled unanticipated roles of RBPs in melanoma malignancy. As proof-of-principle we focused on PDIA6, an ER-lumen chaperone that displayed a novel RNA-binding activity. We show that PDIA6 is involved in metastatic progression, map its RNA-binding domain, and find that RNA binding is required for PDIA6 tumorigenic properties. These results exemplify how RIC technologies can be harnessed to uncover novel vulnerabilities of cancer cells.
  
  
  
  
**PDIA6**
-
2
of
3
  

### Protein disulfide isomerases: Impact of thapsigargin treatment on their expression in melanoma cell lines.

2. Int J Biol Macromol. 2015 Aug;79:44-8. doi: 10.1016/j.ijbiomac.2015.04.029 Epub 2015 Apr 23.
  
  
Silva Z(1), Veríssimo T(1), Videira PA(2), Novo C(3).
  
  
Author information: (1)CEDOC, Chronic Diseases Research Center, NOVA Medical School/Faculdade Ciênci ...
  
  
Anti-cancer treatments usually elevate the content of unfolded or misfolded proteins in the endoplasmic reticulum (ER). Here we aimed to get insights into the relation between sensitivity of melanoma cell lines to the ER stress inducer thapsigargin (THG) and the genetic expression of protein disulfide isomerase family members (PDIs). The expression of PDIs was analysed by flow cytometry and real-time PCR. The results showed that SK-MEL-30, the less THG sensitive cell line, displays higher basal PDIs' expression levels and the sensitivity is increased by the PDIs inhibitor bacitracin. While SK-MEL-30 PDIs' expression is not THG dose-dependent, an increase in glucose related protein 78 (GRP78), PDIA5, PDIA6, and thioredoxin-related-transmembrane proteins' (TMX3 and TMX4) expression, in response to higher drug concentrations, was observed in MNT-1. The differences in PDIs' gene expression in MNT-1 suggest a different response to ER stress compared to the other cell lines and highlight the importance of understanding the diversity among cancer cells.
  
  
  
  
**PDIA6**
-
3
of
3
  

### Targets of protective tumor immunity.

3. Ann N Y Acad Sci. 2009 Sep;1174:74-80. doi: 10.1111/j.1749-6632.2009.04938.x
  
  
Dranoff G(1).
  
  
Author information: (1)Department of Medical Oncology and Cancer Vaccine Center, Dana-Farber Cancer ...
  
  
The identification of antigens associated with tumor destruction is a major goal of cancer immunology. Several genetic and biochemical techniques have revealed a broad range of gene products that elicit immune recognition in cancer patients, but the biologic importance of these responses in most cases is poorly understood. While some targets are linked to tumor regressions in the context of adoptive cellular therapies or cancer vaccinations, the possible roles of immunity to most antigens in disease pathogenesis and clinical outcomes remain to be elucidated. One strategy for characterizing antigens that elicit clinically significant immune recognition involves the study of patients who achieve durable clinical benefits from immune treatments. Through this approach, we uncovered the immunogenicity of major histocompatibility chain-related protein A (MICA), which is a ligand for NKG2D, and ERP5, a protein disulfide isomerase involved in MICA shedding. Our findings suggest that components of the NKG2D pathway may be attractive targets for therapeutic monoclonal antibodies.
  
  
  
  
  

---

  

# PER1

**Signature gene in:** MDM
  
**Differentially expressed in:** MDM
  
  
**PER1**
-
1
of
10
  

### Interplay of Light, Melatonin, and Circadian Genes in Skin Pigmentation Regulation.

1. Pigment Cell Melanoma Res. 2025 Jan;38(1):e13220. doi: 10.1111/pcmr.13220
  
  
Bertolesi GE(1)(2), Debnath N(1)(2), Heshami N(1)(2), Bui R(1)(2), Zadeh-Haghighi H(2)(3), Simon C(2 ...
  
  
Author information: (1)Department of Cell Biology and Anatomy, Alberta Children's Hospital Research ...
  
  
Circadian regulation of skin pigmentation is essential for thermoregulation, ultraviolet (UV) protection, and synchronization of skin cell renewal. This regulation involves both cell-autonomous photic responses and non-cell-autonomous hormonal control, particularly through melatonin produced in a light-sensitive manner. Photosensitive opsins, cryptochromes, and melatonin regulate circadian rhythms in skin pigment cells. We studied light/dark cycles and melatonin coordination in melanin synthesis and cell proliferation of Xenopus laevis melanophores. In vivo, tadpole pigmentation shows robust circadian regulation mainly hormone-driven, in that isolated melanophores respond strongly to melatonin but only slightly to light. Melanophore proliferation is faster in the dark and slower with melatonin as compared to a 12/12 light/dark cycle. Expression of circadian core genes (clock, bmal1, PER1, per2, per3, cry1, cry2, and cry4) in melatonin-treated cells during the light phase mimics dark phase expression. Overexpression of individual Crys did not affect melanization or cell proliferation, likely due to their cooperative actions. Melanin synthesis was inhibited by circadian cycle deregulation through (a) pharmacological inhibition of Cry1 and Cry2 degradation with KL001, (b) continuous light or dark conditions, and (c) melatonin treatment. Our findings suggest that circadian cycle regulation, rather than proliferative capacity, alters melanization of melanophores.
  
  
  
  
**PER1**
-
2
of
10
  

### Disruptions of Circadian Genes in Cutaneous Melanoma-An In Silico Analysis of Transcriptome Databases.

2. Int J Mol Sci. 2023 Jun 14;24(12):10140. doi: 10.3390/ijms241210140
  
  
Lesicka M(1), Nedoszytko B(2)(3), Reszka E(1).
  
  
Author information: (1)Department of Translational Research, Nofer Institute of Occupational Medicin ...
  
  
Circadian genes are a set of genes that regulate the body's internal clock and influence various physiological processes, including sleep-wake cycles, metabolism and immune function. Skin cutaneous melanoma (SKCM) is a type of skin cancer that arises from the pigment-producing cells in the skin and is the most deadly form of skin cancer. This study has investigated the relevance of circadian gene expression and immune infiltrations in the outcomes of cutaneous melanoma patients. In the present study, in silico methods based on the GEPIa, TIMER 2.0 and cBioPortal databases were performed, so as to investigate the transcript level and prognostic value of 24 circadian genes in SKCM and their relationship with the immune infiltration level. The in silico analysis showed that significantly more than half of the investigated circadian genes have an altered transcript pattern in cutaneous melanoma compared to normal skin. The mRNA levels of TIMELES and BHLHE41 were upregulated, whereas those of NFIL3, BMAL1, HLF, TEF, RORA, RORC, NR1D1, PER1, PER2, PER3, CRY2 and BHLHE40 were downregulated. The presented research shows that SKCM patients with at least one alteration of their circadian genes have decreased overall survival. Additionally, majority of the circadian genes are significantly corelated with the immune cells' infiltration level. The strongest correlation was found for neutrophils and was followed by circadian genes: NR1D2 r = 0.52 p < 0.0001, BMAL1 r = 0.509 p < 0.0001; CLOCK r = 0.45 p < 0.0001; CSNKA1A1 r = 0.45 p < 0.0001; RORA r = 0.44 p < 0.0001. The infiltration level of immune cells in skin tumors has been associated with patient prognosis and treatment response. Circadian regulation of immune cell infiltration may further contribute to these prognostic and predictive markers. Examining the correlation between circadian rhythm and immune cell infiltration can provide valuable insights into disease progression and guide personalized treatment decisions.
  
  
  
  
**PER1**
-
3
of
10
  

### Histone lactylation drives oncogenesis by facilitating m(6)A reader protein YTHDF2 expression in ocular melanoma.

3. Genome Biol. 2021 Mar 16;22(1):85. doi: 10.1186/s13059-021-02308-z
  
  
Yu J(#)(1), Chai P(#)(1), Xie M(#)(1), Ge S(1), Ruan J(1), Fan X(1), Jia R(2).
  
  
Author information: (1)Department of Ophthalmology, Shanghai Key Laboratory of Orbital Diseases and ...
  
  
BACKGROUND: Histone lactylation, a metabolic stress-related histone modification, plays an important role in the regulation of gene expression during M1 macrophage polarization. However, the role of histone lactylation in tumorigenesis remains unclear. RESULTS: Here, we show histone lactylation is elevated in tumors and is associated with poor prognosis of ocular melanoma. Target correction of aberrant histone lactylation triggers therapeutic efficacy both in vitro and in vivo. Mechanistically, histone lactylation contributes to tumorigenesis by facilitating YTHDF2 expression. Moreover, YTHDF2 recognizes the m6A modified PER1 and TP53 mRNAs and promotes their degradation, which accelerates tumorigenesis of ocular melanoma. CONCLUSION: We reveal the oncogenic role of histone lactylation, thereby providing novel therapeutic targets for ocular melanoma therapy. We also bridge histone modifications with RNA modifications, which provides novel understanding of epigenetic regulation in tumorigenesis.
  
  
  
  
**PER1**
-
4
of
10
  

### Gender-specific associations between polymorphisms of the circadian gene RORA and cutaneous melanoma susceptibility.

4. J Transl Med. 2021 Feb 6;19(1):57. doi: 10.1186/s12967-021-02725-5
  
  
Benna C(1)(2), Rajendran S(3), Spiro G(3), Menin C(4), Dall'Olmo L(3)(5), Rossi CR(3)(5), Mocellin S ...
  
  
Author information: (1)Department of Surgery Oncology and Gastroenterology, University of Padova, Pa ...
  
  
BACKGROUND: Melanoma is the deadliest of skin cancers and has an increasing annual incidence worldwide. It is a multi-factorial disease most likely arising from both genetic predisposition and environmental exposure to ultraviolet light. Genetic variability of the components of the biological circadian clock is recognized to be a risk factor for different type of cancers. Moreover, two variants of a clock gene, RORA, have been associated with melanoma patient's prognosis. Our aim is to test the hypothesis that specific single nucleotide polymorphisms (SNPs) of the circadian clock genes may significantly influence the predisposition to develop cutaneous melanoma or the outcome of melanoma patients. METHODS: We genotyped 1239 subjects, 629 cases of melanoma and 610 healthy controls in 14 known SNPs of seven selected clock genes: AANAT, CLOCK, NPAS2, PER1, PER2, RORA, and TIMELESS. Genotyping was conducted by q-PCR. Multivariate logistic regression was employed for susceptibility of melanoma assessment, modeled additively. Subgroup analysis was performed by gender. For the female subgroup, a further discrimination was performed by age. For prognosis of melanoma assessment, multivariate Cox proportional hazard regression was employed. The Benjamini-Hochberg method was utilized as adjustment for multiple comparisons. RESULTS: We identified two RORA SNPs statistically significant with respect to the association with melanoma susceptibility. Considering the putative role of RORA as a nuclear steroid hormone receptor, we conducted a subgroup analysis by gender. Interestingly, the RORA rs339972 C allele was associated with a decreased predisposition to develop melanoma only in the female subgroup (OR 0.67; 95% CI 0.51-0.88; P = 0.003) while RORA rs10519097 T allele was associated with a decreased predisposition to develop melanoma only in the male subgroup (OR 0.62; 95% CI 0.44-0.87; P = 0.005). Moreover, the RORA rs339972 C allele had a decreased susceptibility to develop melanoma only in females aged over 50 years old (OR 0.67; 95% CI 0.54-0.83; P = 0.0002). None of the studied SNPs were significantly associated with the prognosis. CONCLUSIONS: Overall, we cannot ascertain that circadian pathway genetic variation is involved in melanoma susceptibility or prognosis. Nevertheless, we identified an interesting relationship between melanoma susceptibility and RORA polymorphisms acting in sex-specific manner and which is worth further future investigation.
  
  
  
  
**PER1**
-
5
of
10
  

### Chronic shift-lag promotes NK cell ageing and impairs immunosurveillance in mice by decreasing the expression of CD122.

5. J Cell Mol Med. 2020 Dec;24(24):14583-14595. doi: 10.1111/jcmm.16088 Epub 2020 Nov 13.
  
  
Zeng X(1), Liang C(2), Yao J(1).
  
  
Author information: (1)Medical Research Center and Department of Laboratory Medicine, Shunde Hospita ...
  
  
Long-term subjection to shift work increases the risk of cancer. The purpose of the present study was to explore the mechanism by which chronic circadian disruption impairs natural killer (NK) cell immunosurveillance. Mice were subjected to light-dark reverse every 4 days for 12 weeks to disrupt normal circadian rhythm. NK cell development and function were evaluated by flow cytometry. The mRNA and protein levels of period 1 (PER1) and per2 were suppressed, while circadian locomotor output cycle kaput (CLOCK) was increased in the shifted mice, indicating successful generation of the circadian rhythm disruption mouse model. Chronic shift-lag promoted NK cell ageing, which is likely due to the reduction in Ly49 family receptor expression in shifted NK. We further studied the effects of circadian rhythm disruption on NK cell function. Chronic shift-lag inhibited NK cell secretion of granular CD107a and interferon gamma. Moreover, chronic shift-lag attenuated the clearance of MHC-I-deficient tumour cells by NK cells in vivo and promoted lung metastasis of B16F10 melanomas. Furthermore, chronic shift-lag reduced NK cell killing function, which may be due to the suppression of Eomes transcription factor expression, which inhibiting the transcription of CD122. In conclusion, our findings suggest that chronic circadian disruption attenuates NK cell cytolytic activity by decreasing the expression of CD122.
  
  
  
  
**PER1**
-
6
of
10
  

### Melanopsin mediates UVA-dependent modulation of proliferation, pigmentation, apoptosis, and molecular clock in normal and malignant melanocytes.

6. Biochim Biophys Acta Mol Cell Res. 2020 Oct;1867(10):118789. doi: 10.1016/j.bbamcr.2020.118789 Epub 2020 Jul 6.
  
  
de Assis LVM(1), Mendes D(2), Silva MM(2), Kinker GS(3), Pereira-Lima I(1), Moraes MN(4), Menck CFM( ...
  
  
Author information: (1)Laboratory of Comparative Physiology of Pigmentation, Department of Physiolog ...
  
  
Cutaneous melanocytes and melanoma cells express several opsins, of which melanopsin (OPN4) detects temperature and UVA radiation. To evaluate the interaction between OPN4 and UVA radiation, normal and malignant Opn4WT and Opn4KO melanocytes were exposed to three daily low doses (total 13.2 kJ/m2) of UVA radiation. UVA radiation led to a reduction of proliferation in both Opn4WT cell lines; however, only in melanoma cells this effect was associated with increased cell death by apoptosis. Daily UVA stimuli induced persistent pigment darkening (PPD) in both Opn4WT cell lines. Upon Opn4 knockout, all UVA-induced effects were lost in three independent clones of Opn4KO melanocytes and melanoma cells. PER1 bioluminescence was reduced after 1st and 2nd UVA radiations in Opn4WT cells. In Opn4KO melanocytes and melanoma cells, an acute increase of PER1 expression was seen immediately after each stimulus. We also found that OPN4 expression is downregulated in human melanoma compared to normal skin, and it decreases with disease progression. Interestingly, metastatic melanomas with low expression of OPN4 present increased expression of BMAL1 and longer overall survival. Collectively, our findings reinforce the functionality of the photosensitive system of melanocytes that may subsidize advancements in the understanding of skin related diseases, including cancer.
  
  
  
  
**PER1**
-
7
of
10
  

### The circadian clock regulates cisplatin-induced toxicity and tumor regression in melanoma mouse and human models.

7. Oncotarget. 2018 Feb 20;9(18):14524-14538. doi: 10.18632/oncotarget.24539 eCollection 2018 Mar 6.
  
  
Dakup PP(1), Porter KI(1), Little AA(1), Gajula RP(1), Zhang H(1), Skornyakov E(2)(3), Kemp MG(4), V ...
  
  
Author information: (1)Department of Pharmaceutical Sciences, College of Pharmacy, Washington State ...
  
  
Cisplatin is one of the most commonly used chemotherapeutic drugs; however, toxicity and tumor resistance limit its use. Studies using murine models and human subjects have shown that the time of day of cisplatin treatment influences renal and blood toxicities. We hypothesized that the mechanisms responsible for these outcomes are driven by the circadian clock. We conducted experiments using wild-type and circadian disrupted PER1/2-/- mice treated with cisplatin at selected morning (AM) and evening (PM) times. Wild-type mice treated in the evening showed an enhanced rate of removal of cisplatin-DNA adducts and less toxicity than the morning-treated mice. This temporal variation in toxicity was lost in the PER1/2-/- clock-disrupted mice, suggesting that the time-of-day effect is linked to the circadian clock. Observations in blood cells from humans subjected to simulated day and night shift schedules corroborated this view. PER1/2-/- mice also exhibited a more robust immune response and slower tumor growth rate, indicating that the circadian clock also influences the immune response to melanoma tumors. Our findings indicate that cisplatin chronopharmacology involves the circadian clock control of DNA repair as well as immune responses, and thus affects both cisplatin toxicity and tumor growth. This has important implications for chronochemotherapy in cancer patients, and also suggests that influencing the circadian clock (e.g., through bright light treatment) may be explored as a tool to improve patient outcomes.
  
  
  
  
**PER1**
-
8
of
10
  

### Melanopsin, a Canonical Light Receptor, Mediates Thermal Activation of Clock Genes.

8. Sci Rep. 2017 Oct 25;7(1):13977. doi: 10.1038/s41598-017-13939-3
  
  
Moraes MN(1), de Assis LVM(1), Magalhães-Marques KK(1), Poletini MO(2), de Lima LHRG(1), Castrucci A ...
  
  
Author information: (1)Department of Physiology, Institute of Biosciences, University of São Paulo, ...
  
  
Melanopsin (OPN4) is a photo-pigment found in a small subset of intrinsically photosensitive ganglion cells (ipRGCs) of the mammalian retina. These cells play a role in synchronizing the central circadian pacemaker to the astronomical day by conveying information about ambient light to the hypothalamic suprachiasmatic nucleus, the site of the master clock. We evaluated the effect of a heat stimulus (39.5 °C) on clock gene (PER1 and Bmal1) expression in cultured murine Melan-a melanocytes synchronized by medium changes, and in B16-F10 melanoma cells, in the presence of the selective OPN4 antagonist AA92593, or after OPN4 knockdown by small interfering RNA (siRNA). In addition, we evaluated the effects of heat shock on the localization of melanopsin by immunocytochemistry. In both cell lines melanopsin was found in a region capping the nucleus and heat shock did not affect its location. The heat-induced increase of PER1 expression was inhibited when melanopsin was pharmacologically blocked by AA92593 as well as when its protein expression was suppressed by siRNA in both Melan-a and B16-F10 cells. These data strongly suggest that melanopsin is required for thermo-reception, acting as a thermo-opsin that ultimately feeds the local circadian clock in mouse melanocytes and melanoma cells.
  
  
  
  
**PER1**
-
9
of
10
  

### Estradiol differently affects melanin synthesis of malignant and normal melanocytes: a relationship with clock and clock-controlled genes.

9. Mol Cell Biochem. 2016 Oct;421(1-2):29-39. doi: 10.1007/s11010-016-2781-3 Epub 2016 Aug 18.
  
  
Poletini MO(1), de Assis LV(2), Moraes MN(2), Castrucci AM(3).
  
  
Author information: (1)Department of Physiology and Biophysics, Institute of Biological Sciences, Fe ...
  
  
Melanin production within melanocytes is regulated, among others, by estradiol, whose effects on melanogenesis are still not completely elucidated. Here we show that although 10(-7) M 17β-estradiol (E2) increased tyrosinase mRNA levels in B16-F10 malignant melanocytes, there was a transient decrease and abolishment of the temporal variation of melanin content. Both parameters were much higher in the malignant than in normal Melan-a cells. Considering that silencing clock machinery in human melanocytes increases melanogenesis, we investigated clock gene expression in those cell lines. Except for Melan-a Bmal1 and B16-F10 Per2 expression of control cells, PER1, Per2, and Bmal1 expression increased independently of cell type or E2 treatment after 24 h. However, melanoma cells showed a marked increase in PER1 and Bma11 expression in response to E2 at the same time points, what may rule out E2 as a synchronizer agent since the expression of those genes were not in antiphase. Next, we investigated the expression of Xpa, a clock-controlled gene, which in Melan-a cells, peaked at 18 h, and E2 treatment shifted this peak to 24 h, whereas B16-F10 Xpa expression peaked at 24 h in both control and E2 group, and it was higher compared to Melan-a cells in both groups. Therefore, malignant and normal melanocytes display profound differences on core elements of the local clock, and how they respond to E2, what is most probably determinant of the differences seen on melanin synthesis and Tyrosinase and Xpa expression. Understanding these processes at the molecular level could bring new strategies to treat melanoma.
  
  
  
  
**PER1**
-
10
of
10
  

### The effect of white light on normal and malignant murine melanocytes: A link between opsins, clock genes, and melanogenesis.

10. Biochim Biophys Acta. 2016 Jun;1863(6 Pt A):1119-33. doi: 10.1016/j.bbamcr.2016.03.001 Epub 2016 Mar 3.
  
  
de Assis LV(1), Moraes MN(1), da Silveira Cruz-Machado S(2), Castrucci AM(3).
  
  
Author information: (1)Laboratory of Comparative Physiology of Pigmentation, Department of Physiolog ...
  
  
The skin possesses a photosensitive system comprised of opsins whose function is not fully understood, and clock genes which exert an important regulatory role in skin biology. Here, we evaluated the presence of opsins in normal (Melan-a cells) and malignant (B16-F10 cells) murine melanocytes. Both cell lines express Opn2, Opn4--for the first time reported in these cell types--as well as S-opsin. OPN4 protein was found in a small area capping the cell nuclei of B16-F10 cells kept in constant dark (DD); twenty-four hours after the white light pulse (WLP), OPN4 was found in the cell membrane. Despite the fact that B16-F10 cells expressed less Opn2 and Opn4 than Melan-a cells, our data indicate that the malignant melanocytes exhibited increased photoresponsiveness. The clock gene machinery is also severely downregulated in B16-F10 cells as compared to Melan-a cells. PER1, Per2, and Bmal1 expression increased in B16-F10 cells in response to WLP. Although no response in clock gene expression to WLP was observed in Melan-a cells, gene correlational data suggest a minor effect of WLP. In contrast to opsins and clock genes, melanogenesis is significantly upregulated in malignant melanocytes in comparison to Melan-a cells. Tyrosinase expression increased after WLP only in B16-F10 cells; however no increase in melanin content after WLP was seen in either cell line. Our findings may prove useful in the treatment and the development of new pharmacological approaches of depigmentation diseases and skin cancer.
  
  
  
  
  

---

  

# PIP4K2A

**Signature gene in:** CD8+ T cells, Tregs
  
**Differentially expressed in:** CD8+ T cells
  
  
**PIP4K2A**
-
1
of
1
  

### Exploring new mechanisms in cancer molecular pathways and pathogenic cell transformation: PIP4K2A as a prognostic marker and therapeutic target in cutaneous malignant melanoma.

1. Discov Oncol. 2024 Nov 23;15(1):697. doi: 10.1007/s12672-024-01555-3
  
  
Luo WF(#)(1), Song DM(#)(2), Shen T(2), He YB(3), Du HY(4), Si MJ(#)(5), Fang LW(#)(6).
  
  
Author information: (1)Jinzhou Medical University, The First Affiliated Hospital of Jinzhou Medical ...
  
  
BACKGROUND: Cutaneous malignant melanoma is a very aggressive and metastatic form of skin cancer, typically linked with poor outcomes. Advances in genomic analysis have underscored the crucial role of T cells in tumor immunity. Immune checkpoint inhibitors have notably transformed melanoma treatment by boosting T cell activity. Studies of gene expression have found that the phosphatidylinositol-4-phosphate kinase 2A (PIP4K2A) gene is abnormally expressed in various tumors, indicating its potential role in tumor progression. Utilizing single-cell sequencing and machine learning, researchers can now explore the complex interactions between T cells and melanoma cells at a genomic level. This study aimed to investigate the role of the PIP4K2A gene in cutaneous malignant melanoma, with a focus on its influence on T cell-mediated immune responses. METHODS: Samples from cutaneous melanoma patients were analysed by single-cell transcriptome for differentially expressed genes and signalling pathways associated with cutaneous melanoma. Then, genes were identified and predictive models were built based on the transcriptomic data using machine learning models to assess whether the expression level of PIP4K2A could effectively predict the malignancy and prognosis of cutaneous melanoma. In addition, we also performed drug therapy predictive analysis and immunotherapy analysis.Finally, the critical role of PIP4K2A in cutaneous melanoma was further confirmed by immunohistochemistry. RESULTS: The PIP4K2A gene exhibited a significantly elevated expression level in cutaneous malignant melanoma, showing a strong correlation with the clinical stage and patient prognosis. At the therapeutic level, high PIP4K2A expression is less responsive to immunotherapy, and this gene is a risk factor for drug therapy in cutaneous malignant melanoma. Additionally, our experimental outcomes validated this observation. CONCLUSIONS: The PIP4K2A gene could be a crucial prognostic marker for cutaneous malignant melanoma, as it significantly affects T cell activity within the tumor microenvironment. This study offers essential insights into melanoma pathogenesis and assists in pinpointing new early diagnostic markers and therapeutic targets. Utilizing advanced genomic tools and computational techniques, the research enhances our understanding of T cell dynamics in melanoma, facilitating the development of personalized medicine and more effective immunotherapy strategies.
  
  
  
  
  

---

  

# PLCB1

**Signature gene in:** Stromal cells
  
**Differentially expressed in:** Stromal cells
  
  
**PLCB1**
-
1
of
1
  

### Novel Genetic Risk Variants Associated with Oral Tongue Squamous Cell Carcinoma.

1. Head Neck Pathol. 2025 Apr 25;19(1):45. doi: 10.1007/s12105-025-01784-0
  
  
Nikkilä R(1)(2)(3), Mäkitie A(1)(3), Joensuu H(4), Markkanen S(5)(6), Elenius K(7)(8)(9), Monni O(4) ...
  
  
Author information: (1)Department of Otorhinolaryngology - Head and Neck Surgery, University of Hels ...
  
  
PURPOSE: Limited data from genome-wide association studies (GWAS) focusing on oral tongue squamous cell carcinoma (OTSCC) are available. The present study was conducted to explore genetic associations for OTSCC. METHODS: A GWAS on 376 cases of OTSCC was conducted using the FinnGen Data Freeze-12 dataset. The case-cohort included 205 males and 171 females. Cases with malignancies involving the base of the tongue or lingual tonsil were excluded from the case-cohort. Individuals with no recorded history of malignancy were used as controls (n = 407,067). A Phenome-wide association study (PheWAS) was performed for the lead variants to assess their co-associations with other cancers. RESULTS: GWAS analysis identified three genome-wide significant loci associated with OTSCC (p < 5 × 10^-8^), located at 5p15.33 (rs27067 near gene LINC01511), 10q24 (rs1007771191 near RPS3AP36), and 20p12.3 (rs1438070080 near PLCB1), respectively. PheWAS showed associations of rs27067 mainly with prostate cancer (OR = 1.06, p = 5.41 × 10^-7^), and seborrheic keratosis (OR = 1.11, p = 1.51 × 10^-11^). A co-directional effect with melanoma was also observed (OR = 0.93, p = 6.24 × 10^-5^). CONCLUSION: The GWAS detected two novel genetic associations with OTSCC. Further research is needed to identify the genes at these loci that contribute to the molecular pathogenesis of OTSCC.
  
  
  
  
  

---

  

# PPP2R5C

**Signature gene in:** CD8+ T cells, Endothelial cells
  
**Differentially expressed in:** CD8+ T cells
  
  
**PPP2R5C**
-
1
of
1
  

### The protein phosphatase 2A subunit Bgamma gene is identified to be differentially expressed in malignant melanomas by subtractive suppression hybridization.

1. Melanoma Res. 2001 Dec;11(6):577-85. doi: 10.1097/00008390-200112000-00004
  
  
Deichmann M(1), Polychronidis M, Wacker J, Thome M, Näher H.
  
  
Author information: (1)Department of Dermatology, University Clinics of Heidelberg, Vossstrasse 2, 6 ...
  
  
Several genes implicated in the development of various malignancies appear to be of minor relevance in melanoma. We therefore aimed to find a tumour suppressor candidate involved in this malignancy by comparing gene expression in uncultured primary melanoma specimens with those in acquired melanocytic naevi, from which quite often melanomas are known to arise. Applying the subtractive suppression hybridization technique, we generated a subtracted library of candidate genes downregulated in melanoma. Among the cDNA fragments identical to known genes, this library included a cDNA fragment 630 bp in length that is identical to the gene for the human protein phosphatase 2A (PP2A) regulatory subunit B (B56) gamma isoform (PP2A-Bgamma, PPP2R5C). On further evaluation of 15 primary melanoma and 16 acquired melanocytic naevus tissue specimens from independent patients using semiquantitative reverse transcription-polymerase chain reaction (RT-PCR) analysis, expression of this gene was found to be suppressed in melanomas compared with naevi; the difference was statistically significant. As PP2A is known to be a major cellular serine-threonine phosphatase, and has been implicated not only in the regulation of cell growth and division but also in the control of gene transcription and growth factor signal transduction, alterations in the pattern of the regulatory subunits may affect substrate specificity and subcellular localization of the PP2A holoenzyme in melanoma cells.
  
  
  
  
  

---

  

# PRKCB

**Signature gene in:** B cells, CD8+ T cells
  
**Differentially expressed in:** CD8+ T cells
  
  
**PRKCB**
-
1
of
1
  

### Histologic and Genetic Features of 51 Melanocytic Neoplasms With Protein Kinase C Fusion Genes.

1. Mod Pathol. 2023 Nov;36(11):100286. doi: 10.1016/j.modpat.2023.100286 Epub 2023 Jul 19.
  
  
de la Fouchardière A(1), Pissaloux D(2), Houlier A(3), Paindavoine S(3), Tirode F(4), LeBoit PE(5), ...
  
  
Author information: (1)Department of Biopathology, Centre Léon Bérard, Lyon, France; Department of R ...
  
  
Fusion genes involving homologs of protein kinase C (PKC) have been identified in a variety of tumors. We report the clinical and histologic presentation of 51 cutaneous melanocytic neoplasms with a PKC fusion gene (involving PRKCA in 35 cases, PRKCB in 15 cases, and PRKCG in a single case). Most tumors were in young adults (median age, 29.5 years; range, 1-73 years) but some presented in newborns. Histologically, 42 tumors were classified as benign, presenting predominantly as biphasic dermal proliferation (88%) with nests of small melanocytes surrounded by fibrosis with haphazardly arranged spindled and dendritic melanocytes, resembling those reported as "combined blue nevi." Most tumors (60%) were heavily pigmented and in 15%, hyperpigmented epithelioid melanocytes were present at the dermoepidermal junction. Two lesions were paucicellular and showed marked sclerosis. Three tumors, including 2 proliferating nodules, were considered intermediate grade. Six tumors had sheets of atypical melanocytes infiltrating the dermis and were classified as melanomas. Two of the melanomas displayed loss of BAP1 nuclear expression. The median follow-up time was 12 months, with 1 patient alive with metastatic disease and 1 dying of their melanoma. These results suggest that melanocytic tumors with PKC fusion genes have characteristic histopathologic features, which are more similar to blue nevi than to pigmented epithelioid melanocytomas. As is the case with GNA-mutated blue nevi, they can progress to melanomas via BAP1 inactivation and metastasize.
  
  
  
  
  

---

  

# PSTPIP2

**Signature gene in:** MDM
  
**Differentially expressed in:** MDM
  
  
**PSTPIP2**
-
1
of
1
  

### The nonreceptor tyrosine kinase SYK drives caspase-8/NLRP3 inflammasome-mediated autoinflammatory osteomyelitis.

1. J Biol Chem. 2020 Mar 13;295(11):3394-3400. doi: 10.1074/jbc.RA119.010623 Epub 2019 Nov 12.
  
  
Dasari TK(1), Geiger R(2), Karki R(2), Banoth B(2), Sharma BR(2), Gurung P(3), Burton A(2), Kannegan ...
  
  
Author information: (1)Department of Immunology, St. Jude Children's Research Hospital, Memphis, Ten ...
  
  
Comment in J Biol Chem. 2020 Mar 13;295(11):3401-3402. doi: 10.1074/jbc.H120.012867.
  
  
  
  
  

---

  

# PTPRJ

**Signature gene in:** CD8+ T cells
  
**Differentially expressed in:** CD8+ T cells
  
  
**PTPRJ**
-
1
of
5
  

### Integrated genomic analyses of acral and mucosal melanomas nominate novel driver genes.

1. Genome Med. 2022 Jun 16;14(1):65. doi: 10.1186/s13073-022-01068-0
  
  
Wang M(1)(2), Banik I(1)(2), Shain AH(1)(2), Yeh I(3)(4)(5), Bastian BC(6)(7)(8).
  
  
Author information: (1)Department of Dermatology, University of California San Francisco, San Franci ...
  
  
BACKGROUND: Acral and mucosal melanomas are aggressive subtypes of melanoma, which have a significantly lower burden of somatic mutations than cutaneous melanomas, but more frequent copy number variations, focused gene amplifications, and structural alterations. The landscapes of their genomic alterations remain to be fully characterized. METHODS: We compiled sequencing data of 240 human acral and mucosal melanoma samples from 11 previously published studies and applied a uniform pipeline to call tumor cell content, ploidy, somatic and germline mutations, as well as CNVs, LOH, and SVs. We identified genes that are significantly mutated or recurrently affected by CNVs and implicated in oncogenesis. We further examined the difference in the frequency of recurrent pathogenic alterations between the two melanoma subtypes, correlation between pathogenic alterations, and their association with clinical features. RESULTS: We nominated PTPRJ, mutated and homozygously deleted in 3.8% (9/240) and 0.8% (2/240) of samples, respectively, as a probable tumor suppressor gene, and FER and SKP2, amplified in 3.8% and 11.7% of samples, respectively, as probable oncogenes. We further identified a long tail of infrequent pathogenic alterations, involving genes such as CIC and LZTR1. Pathogenic germline mutations were observed on MITF, PTEN, ATM, and PRKN. We found BRAF V600E mutations in acral melanomas with fewer structural variations, suggesting that they are distinct and related to cutaneous melanomas. Amplifications of PAK1 and GAB2 were more commonly observed in acral melanomas, whereas SF3B1 R625 codon mutations were unique to mucosal melanomas (12.9%). Amplifications at 11q13-14 were frequently accompanied by fusion to a region on chromosome 6q12, revealing a recurrent novel structural rearrangement whose role remains to be elucidated. CONCLUSIONS: Our meta-analysis expands the catalog of driver mutations in acral and mucosal melanomas, sheds new light on their pathogenesis and broadens the catalog of therapeutic targets for these difficult-to-treat cancers.
  
  
  
  
**PTPRJ**
-
2
of
5
  

### Cross-species genomic landscape comparison of human mucosal melanoma with canine oral and equine melanoma.

2. Nat Commun. 2019 Jan 21;10(1):353. doi: 10.1038/s41467-018-08081-1
  
  
Wong K(1), van der Weyden L(1), Schott CR(2), Foote A(3), Constantino-Casas F(4), Smith S(5), Dobson ...
  
  
Author information: (1)Wellcome Sanger Institute, Wellcome Trust Genome Campus, Hinxton, Cambridge, ...
  
  
Mucosal melanoma is a rare and poorly characterized subtype of human melanoma. Here we perform a cross-species analysis by sequencing tumor-germline pairs from 46 primary human muscosal, 65 primary canine oral and 28 primary equine melanoma cases from mucosal sites. Analysis of these data reveals recurrently mutated driver genes shared between species such as NRAS, FAT4, PTPRJ, TP53 and PTEN, and pathogenic germline alleles of BRCA1, BRCA2 and TP53. We identify a UV mutation signature in a small number of samples, including human cases from the lip and nasal mucosa. A cross-species comparative analysis of recurrent copy number alterations identifies several candidate drivers including MDM2, B2M, KNSTRN and BUB1B. Comparison of somatic mutations in recurrences and metastases to those in the primary tumor suggests pervasive intra-tumor heterogeneity. Collectively, these studies suggest a convergence of some genetic changes in mucosal melanomas between species but also distinctly different paths to tumorigenesis.
  
  
  
  
**PTPRJ**
-
3
of
5
  

### Somatic inactivating PTPRJ mutations and dysregulated pathways identified in canine malignant melanoma by integrated comparative genomic analysis.

3. PLoS Genet. 2018 Sep 6;14(9):e1007589. doi: 10.1371/journal.pgen.1007589 eCollection 2018 Sep.
  
  
Hendricks WPD(1), Zismann V(1), Sivaprakasam K(1)(2), Legendre C(1), Poorman K(3)(4), Tembe W(1), Pe ...
  
  
Author information: (1)Integrated Cancer Genomics Division, Translational Genomics Research Institut ...
  
  
Canine malignant melanoma, a significant cause of mortality in domestic dogs, is a powerful comparative model for human melanoma, but little is known about its genetic etiology. We mapped the genomic landscape of canine melanoma through multi-platform analysis of 37 tumors (31 mucosal, 3 acral, 2 cutaneous, and 1 uveal) and 17 matching constitutional samples including long- and short-insert whole genome sequencing, RNA sequencing, array comparative genomic hybridization, single nucleotide polymorphism array, and targeted Sanger sequencing analyses. We identified novel predominantly truncating mutations in the putative tumor suppressor gene PTPRJ in 19% of cases. No BRAF mutations were detected, but activating RAS mutations (24% of cases) occurred in conserved hotspots in all cutaneous and acral and 13% of mucosal subtypes. MDM2 amplifications (24%) and TP53 mutations (19%) were mutually exclusive. Additional low-frequency recurrent alterations were observed amidst low point mutation rates, an absence of ultraviolet light mutational signatures, and an abundance of copy number and structural alterations. Mutations that modulate cell proliferation and cell cycle control were common and highlight therapeutic axes such as MEK and MDM2 inhibition. This mutational landscape resembles that seen in BRAF wild-type and sun-shielded human melanoma subtypes. Overall, these data inform biological comparisons between canine and human melanoma while suggesting actionable targets in both species.
  
  
  
  
**PTPRJ**
-
4
of
5
  

### Multi-omics Analysis of Serum Samples Demonstrates Reprogramming of Organ Functions Via Systemic Calcium Mobilization and Platelet Activation in Metastatic Melanoma.

4. Mol Cell Proteomics. 2017 Jan;16(1):86-99. doi: 10.1074/mcp.M116.063313 Epub 2016 Nov 22.
  
  
Muqaku B(1), Eisinger M(1), Meier SM(1), Tahir A(1), Pukrop T(2), Haferkamp S(2), Slany A(1), Reichl ...
  
  
Author information: (1)From the ‡Department of Analytical Chemistry, Faculty of Chemistry, Universit ...
  
  
Pathophysiologies of cancer-associated syndromes such as cachexia are poorly understood and no routine biomarkers have been established, yet. Using shotgun proteomics, known marker molecules including PMEL, CRP, SAA, and CSPG4 were found deregulated in patients with metastatic melanoma. Targeted analysis of 58 selected proteins with multiple reaction monitoring was applied for independent data verification. In three patients, two of which suffered from cachexia, a tissue damage signature was determined, consisting of nine proteins, PLTP, CD14, TIMP1, S10A8, S10A9, GP1BA, PTPRJ, CD44, and C4A, as well as increased levels of glycine and asparagine, and decreased levels of polyunsaturated phosphatidylcholine concentrations, as determined by targeted metabolomics. Remarkably, these molecules are known to be involved in key processes of cancer cachexia. Based on these results, we propose a model how metastatic melanoma may lead to reprogramming of organ functions via formation of platelet activating factors from long-chain polyunsaturated phosphatidylcholines under oxidative conditions and via systemic induction of intracellular calcium mobilization. Calcium mobilization in platelets was demonstrated to alter levels of several of these marker molecules. Additionally, platelets from melanoma patients proved to be in a rather exhausted state, and platelet-derived eicosanoids implicated in tumor growth were found massively increased in blood from three melanoma patients. Platelets were thus identified as important source of serum protein and lipid alterations in late stage melanoma patients. As a result, the proposed model describes the crosstalk between lipolysis of fat tissue and muscle wasting mediated by oxidative stress, resulting in the metabolic deregulations characteristic for cachexia.
  
  
  
  
**PTPRJ**
-
5
of
5
  

### TNF-alpha and melphalan modulate a specific group of early expressed genes in a murine melanoma model.

5. Cytokine. 2013 May;62(2):217-25. doi: 10.1016/j.cyto.2013.02.022 Epub 2013 Mar 25.
  
  
de Lima VC(1), de Carvalho AF, Morato-Marques M, Hashimoto VL, Spilborghs GM, Marques SM, Landman G, ...
  
  
Author information: (1)Laboratório de Inflamação e Câncer, Fundação Antônio Prudente, Hospital AC Ca ...
  
  
BACKGROUND: Cutaneous melanoma displays high morbidity and mortality rates. Isolated limb perfusion with melphalan (Mel) is used for the treatment of non-resectable, locally advanced extremity melanomas. When combined with tumor necrosis factor alpha (TNF-alpha) treatment, the complete response varies between 70% and 90%. The mechanisms underlying the effects of Mel and TNF-alpha are not completely understood. We evaluated the impact of systemic Mel and TNF-alpha administration on tumor growth, analyzed the morphological changes promoted by each treatment, and identified early expressed genes in response to Mel and TNF-alpha treatment, either alone or in combination, in a murine melanoma model. METHODS: Six- to eight-week-old male mice were subcutaneously inoculated with B16F10 melanoma cells and then intravenously injected with TNF-alpha, melphalan or a combination of both drugs when the tumors reached 1.0 cm(2). Tumor growth was monitored every other day, and histological analysis was performed when the tumors reached 3.0 cm(2). Total RNA was extracted from the resected tumors and submitted to amplification, labeling and hybridization on an oligonucleotide microarray (Fox Chase Cancer Center). Tumor growth and histological parameters were compared using ANOVA. Survival curves were calculated using the Kaplan-Meier method. Two-way ANOVA was used to identify differentially expressed genes among the various treatments, and Dunn's test was used for pair-wise comparisons. RESULTS: Systemic administration of Mel impaired tumor growth (p<0.001), improved animal survival (p<0.001), and decreased mitotic rate (p=0.049). Treatment with TNF-alpha alone had no impact, neither on tumor growth, nor on survival, but it increased necrosis (p<0.024) and decreased mitotic rates (p=0.001) in the tumors. Combined treatment with Mel and TNF-alpha had similar effects in tumor growth, survival, necrosis and mitotic rate as observed with individual treatments. Moreover, 118 genes were found differentially expressed by microarray analysis and 10% of them were validated by RT- real time PCR. In our model we found that the treatments regulate genes that play important roles in tumorigenesis such as cell adhesion (Pard3, Pecam1, Ilk, and Dlg5), proliferation (Tcfe3 and Polr1e), cell motility (Kifap3, Palld, and Arhgef6), apoptosis (Bcl2l11), and angiogenesis (Flt1 and PTPRJ). CONCLUSIONS: Our data reproduces, in mice, some of the features observed in melanoma patients treated with the combination of Mel and TNF-alpha. The identification of genes with altered expression by these drugs both individually and in combination might help in the understanding of their mechanism of action and, as a consequence, improved strategies that could impact their clinical application.
  
  
  
  
  

---

  

# RBMS1

**Signature gene in:** Stromal cells
  
**Differentially expressed in:** Stromal cells
  
  
**RBMS1**
-
1
of
1
  

### RBMS1: Shedding light on ocular melanoma's dark prognosis.

1. Exp Eye Res. 2024 Oct;247:110058. doi: 10.1016/j.exer.2024.110058 Epub 2024 Aug 22.
  
  
Yaghy A(1), Perera DC(2).
  
  
Author information: (1)Department of Ophthalmology and Visual Sciences, University of Massachusetts ...
  
  
DOI: 10.1016/j.exer.2024.110058 PMID: 39181227 [Indexed for MEDLINE]
  
  
  
  
  

---

  

# SFRP4

**Signature gene in:** Stromal cells
  
**Differentially expressed in:** Stromal cells
  
  
**SFRP4**
-
1
of
1
  

### Finding Novel Anti-carcinomas Compounds by Targeting SFRP4 Through Molecular Modeling, Docking and Dynamic Simulation Studies.

1. Curr Comput Aided Drug Des. 2018;14(2):160-173. doi: 10.2174/1573409914666180112100122
  
  
Hassan M(1), Azhar M(2)(3), Abbas Q(4), Raza H(1), Moustafa AA(5), Shahzadi S(6), Ashraf Z(7), Seo S ...
  
  
Author information: (1)Department of Biological Sciences, College of Natural Sciences, Kongju Nation ...
  
  
BACKGROUND: Secreted Frizzled-Related Protein 4 (SFRP4) is a glycoprotein that acts as a competitor of both canonical and non-canonical Wnt pathways. SFRP4 is mostly expressed in ovary and plays a significant role as a target molecule to cure ovarian carcinoma. OBJECTIVE: Multiple chemical agonists are being used to cure ovary melanoma. We are interested in theoretically analyzing the compounds through computational approaches for their potential inhibitory effects against SFRP4. METHODS: Compounds were sketched in Chemsketch drawing tool and minimized through chimera tool. Because the crystal structure of SFRP4 is not available in Protein Data Bank, homology modeling approach was used to predict Three-Dimensional (3D) crystal structure of SFRP4. Moreover, multiple computational approaches such as molecular docking and Molecular Dynamic (MD) simulations along with various online tools were employed to screen the best inhibitor against ovary melanoma. RESULTS: The docking results showed that 1d and 1e compounds revealed significant binding energy values (-9.10 and -9.00 kcal/mol, respectively) compared with the standard drugs such as cis-platin and docetaxel (-3.30, -10.80 kcal/mol), respectively. Moreover, MD simulation results showed that 1d has little fluctuations throughout the simulation period as depicted by the root mean square deviation and root mean square fluctuation graphs. CONCLUSION: The present in-silico study provides a deeper insight into the structural attributes of 1d compound and its overall molecular interactions against SFRP4 and gives a hypothetical gateway to use this compound as a potential inhibitor against ovarian carcinoma.
  
  
  
  
  

---

  

# SH3PXD2B

**Signature gene in:** MDM
  
**Differentially expressed in:** MDM
  
  
**SH3PXD2B**
-
1
of
1
  

### The role of Tks adaptor proteins in invadopodia formation, growth and metastasis of melanoma.

1. Oncotarget. 2016 Nov 29;7(48):78473-78486. doi: 10.18632/oncotarget.12954
  
  
Iizuka S(1)(2), Abdullah C(1)(2)(3), Buschman MD(1)(4), Diaz B(1)(5), Courtneidge SA(1)(2)(6).
  
  
Author information: (1)Sanford Burnham Prebys Medical Discovery Institute, La Jolla, CA, USA. (2)Dep ...
  
  
Metastatic cancer cells are characterized by their ability to degrade and invade through extracellular matrix. We previously showed that the Tks adaptor proteins, Tks4 and Tks5, are required for invadopodia formation and/or function in Src-transformed fibroblasts and a number of human cancer cell types. In this study, we investigated the role of Tks adaptor proteins in melanoma cell invasion and metastasis. Knockdown of either Tks4 or Tks5 in both mouse and human melanoma cell lines resulted in a decreased ability to form invadopodia and degrade extracellular matrix. In addition, Tks-knockdown melanoma cells had decreased proliferation in a 3-dimensional type l collagen matrix, but not in 2-dimensional culture conditions. We also investigated the role of Tks proteins in melanoma progression in vivo using xenografts and experimental metastasis assays. Consistent with our in vitro results, reduction of Tks proteins markedly reduced subcutaneous melanoma growth as well as metastatic growth in the lung. We explored the clinical relevance of Tks protein expression in human melanoma specimens using a tissue microarray. Compared to non-malignant nevi, both Tks proteins were highly expressed in melanoma tissues. Moreover, metastatic melanoma cases showed higher expression of Tks5 than primary melanoma cases. Taken together, these findings suggest the importance of Tks adaptor proteins in melanoma growth and metastasis in vivo, likely via functional invadopodia formation.
  
  
  
  
  

---

  

# SLA

**Signature gene in:** CD4+ T cells, CD8+ T cells, MDM, Tregs
  
**Differentially expressed in:** CD8+ T cells, MDM
  
  
**SLA**
-
1
of
10
  

### Microcavity well-plate for automated parallel bioelectronic analysis of 3D cell cultures.

1. Biosens Bioelectron. 2024 Apr 15;250:116042. doi: 10.1016/j.bios.2024.116042 Epub 2024 Jan 14.
  
  
Zitzmann FD(1), Schmidt S(2), Frank R(2), Weigel W(2), Meier M(3), Jahnke HG(4).
  
  
Author information: (1)Centre for Biotechnology and Biomedicine, Biochemical Cell Technology, Leipzi ...
  
  
Three-dimensional (3D) in vitro cell culture models serve as valuable tools for accurately replicating cellular microenvironments found in vivo. While cell culture technologies are rapidly advancing, the availability of non-invasive, real-time, and label-free analysis methods for 3D cultures remains limited. To meet the demand for higher-throughput drug screening, there is a demanding need for analytical methods that can operate in parallel. Microelectrode systems in combination with microcavity arrays (MCAs), offer the capability of spatially resolved electrochemical impedance analysis and field potential monitoring of 3D cultures. However, the fabrication and handling of small-scale MCAs have been labour-intensive, limiting their broader application. To overcome this challenge, we have established a process for creating MCAs in a standard 96-well plate format using high-precision selective laser etching. In addition, to automate and ensure the accurate placement of 3D cultures on the MCA, we have designed and characterized a plug-in tool using SLA-3D-printing. To characterize our new 96-well plate MCA-based platform, we conducted parallel analyses of human melanoma 3D cultures and monitored the effect of cisplatin in real-time by impedance spectroscopy. In the following we demonstrate the capabilities of the MCA approach by analysing contraction rates of human pluripotent stem cell-derived cardiomyocyte aggregates in response to cardioactive compounds. In summary, our MCA system significantly expands the possibilities for label-free analysis of 3D cell and tissue cultures, offering an order of magnitude higher parallelization capacity than previous devices. This advancement greatly enhances its applicability in real-world settings, such as drug development or clinical diagnostics.
  
  
  
  
**SLA**
-
2
of
10
  

### Sulfated Lactosyl Archaeol Archaeosomes Synergize with Poly(I:C) to Enhance the Immunogenicity and Efficacy of a Synthetic Long Peptide-Based Vaccine in a Melanoma Tumor Model.

2. Pharmaceutics. 2021 Feb 12;13(2):257. doi: 10.3390/pharmaceutics13020257
  
  
Akache B(1), Agbayani G(1), Stark FC(1), Jia Y(1), Dudani R(1), Harrison BA(1), Deschatelets L(1), C ...
  
  
Author information: (1)Human Health Therapeutics, National Research Council Canada, Ottawa, ON K1A 0 ...
  
  
Cancer remains a leading cause of morbidity and mortality worldwide. While novel treatments have improved survival outcomes for some patients, new treatment modalities/platforms are needed to combat a wider variety of tumor types. Cancer vaccines harness the power of the immune system to generate targeted tumor-specific immune responses. Liposomes composed of glycolipids derived from archaea (i.e., archaeosomes) have been shown to be potent adjuvants, inducing robust, long-lasting humoral and cell-mediated immune responses to a variety of antigens. Herein, we evaluated the ability of archaeosomes composed of sulfated lactosyl archaeol (SLA), a semi-synthetic archaeal glycolipid, to enhance the immunogenicity of a synthetic long peptide-based vaccine formulation containing the dominant CD8+ T cell epitope, SIINFEKL, from the weakly immunogenic model antigen ovalbumin. One advantage of immunizing with long peptides is the ability to include multiple epitopes, for example, the long peptide antigen was also designed to include the immediately adjacent CD4+ epitope, TEWTSSNVMEER. SLA archaeosomes were tested alone or in combination with the toll-like receptor 3 (TLR3) agonist Poly(I:C). Overall, SLA archaeosomes synergized strongly with Poly(I:C) to induce robust antigen-specific CD8+ T cell responses, which were highly functional in an in vivo cytolytic assay. Furthermore, immunization with this vaccine formulation suppressed tumor growth and extended mouse survival in a mouse melanoma tumor model. Overall, the combination of SLA archaeosomes and Poly(I:C) appears to be a promising adjuvant system when used along with long peptide-based antigens targeting cancer.
  
  
  
  
**SLA**
-
3
of
10
  

### Mechanistic insight into the induction of cellular immune responses by encapsulated and admixed archaeosome-based vaccine formulations.

3. Hum Vaccin Immunother. 2020 Sep 1;16(9):2183-2195. doi: 10.1080/21645515.2020.1788300 Epub 2020 Aug 5.
  
  
Agbayani G(1), Jia Y(1), Akache B(1), Chandan V(1), Iqbal U(1), Stark FC(1), Deschatelets L(1), Lam ...
  
  
Author information: (1)Human Health Therapeutics, National Research Council Canada , Ottawa, ON, Can ...
  
  
Archaeosomes are liposomes formulated using total polar lipids (TPLs) or semi-synthetic glycolipids derived from archaea. Conventional archaeosomes with entrapped antigen exhibit robust adjuvant activity as demonstrated by increased antigen-specific humoral and cell-mediated responses and enhanced protective immunity in various murine infection and cancer models. However, antigen entrapment efficiency can vary greatly resulting in antigen loss during formulation and variable antigen:lipid ratios. In order to circumvent this, we recently developed an admixed archaeosome formulation composed of a single semi-synthetic archaeal lipid (SLA, sulfated lactosylarchaeol) which can induce similarly robust adjuvant activity as an encapsulated formulation. Herein, we evaluate and compare the mechanisms involved in the induction of early innate and antigen-specific responses by both admixed (Adm) and encapsulated (Enc) SLA archaeosomes. We demonstrate that both archaeosome formulations result in increased immune cell infiltration, enhanced antigen retention at injection site and increased antigen uptake by antigen-presenting cells and other immune cell types, including neutrophils and monocytes following intramuscular injection to mice using ovalbumin as a model antigen. In vitro studies demonstrate SLA in either formulation is preferentially taken up by macrophages. Although the encapsulated formulation was better able to induce antigen-specific CD8+ T cell activation by dendritic cells in vitro, both encapsulated and admixed formulations gave equivalently enhanced protection from tumor challenge when tested in vivo using a B16-OVA melanoma model. Despite some differences in the immunostimulatory profile relative to the SLA (Enc) formulation, SLA (Adm) induces strong in vivo immunogenicity and efficacy, while offering an ease of formulation.
  
  
  
  
**SLA**
-
4
of
10
  

### Simplified Admix Archaeal Glycolipid Adjuvanted Vaccine and Checkpoint Inhibitor Therapy Combination Enhances Protection from Murine Melanoma.

4. Biomedicines. 2019 Nov 23;7(4):91. doi: 10.3390/biomedicines7040091
  
  
Stark FC(1), Agbayani G(1), Sandhu JK(1), Akache B(1), McPherson C(1), Deschatelets L(1), Dudani R(1 ...
  
  
Author information: (1)Human Health Therapeutics, National Research Council of Canada, Montreal Rd. ...
  
  
Archaeosomes are liposomes composed of natural or synthetic archaeal lipids that when used as adjuvants induce strong long-lasting humoral and cell-mediated immune responses against entrapped antigens. However, traditional entrapped archaeosome formulations have only low entrapment efficiency, therefore we have developed a novel admixed formulation which offers many advantages, including reduced loss of antigen, consistency of batch-to-batch production as well as providing the option to formulate the vaccine immediately before use, which is beneficial for next generation cancer therapy platforms that include patient specific neo-antigens or for use with antigens that are less stable. Herein, we demonstrate that, when used in combination with anti-CTLA-4 and anti-PD-1 checkpoint therapy, this novel admixed archaeosome formulation, comprised of preformed sulfated lactosyl archaeol (SLA) archaeosomes admixed with OVA antigen (SLA-OVA (adm)), was as effective at inducing strong CD8+ T cell responses and protection from a B16-OVA melanoma tumor challenge as the traditionally formulated archaeosomes with encapsulated OVA protein. Furthermore, archaeosome vaccine formulations combined with anti-CTLA-4 and anti-PD-1 therapy, induced OVA-CD8+ T cells within the tumor and immunohistochemical analysis revealed the presence of CD8+ T cells associated with dying or dead tumor cells as well as within or around tumor blood vessels. Overall, archaeosomes constitute an attractive option for use with combinatorial checkpoint inhibitor cancer therapy platforms.
  
  
  
  
**SLA**
-
5
of
10
  

### Exploring variation in low-value care: a multilevel modelling study.

5. BMC Health Serv Res. 2019 May 30;19(1):345. doi: 10.1186/s12913-019-4159-1
  
  
Badgery-Parker T(1), Feng Y(2), Pearson SA(3), Levesque JF(4), Dunn S(5), Elshaug AG(2).
  
  
Author information: (1)Faculty of Medicine and Health, School of Public Health, Menzies Centre for H ...
  
  
BACKGROUND: Whether patients receive low-value hospital care (care that is not expected to provide a net benefit) may be influenced by unmeasured factors at the hospital they attend or the hospital's Local Health District (LHD), or the patients' areas of residence. Multilevel modelling presents a method to examine the effects of these different levels simultaneously and assess their relative importance to the outcome. Knowing which of these levels has the greatest contextual effects can help target further investigation or initiatives to reduce low-value care. METHODS: We conducted multilevel logistic regression modelling for nine low-value hospital procedures. We fit a series of six models for each procedure. The baseline model included only episode-level variables with no multilevel structure. We then added each level (hospital, LHD, Statistical Local Area [SLA] of residence) separately and used the change in the c statistic from the baseline model as a measure of the contribution of the level to the outcome. We then examined the variance partition coefficients (VPCs) and median odds ratios for a model including all three levels. Finally, we added level-specific covariates to examine if they were associated with the outcome. RESULTS: Analysis of the c statistics showed that hospital was more important than LHD or SLA in explaining whether patients receive low-value care. The greatest increases were 0.16 for endoscopy for dyspepsia, 0.13 for colonoscopy for constipation, and 0.14 for sentinel lymph node biopsy for early melanoma. SLA gave a small increase in c compared with the baseline model, but no increase over the model with hospital. The VPCs indicated that hospital accounted for most of the variation not explained by the episode-level variables, reaching 36.8% (95% CI, 31.9-39.0) for knee arthroscopy. ERCP (8.5%; 95% CI, 3.9-14.7) and EVAR (7.8%; 95% CI, 2.9-15.8) had the lowest residual variation at the hospital level. The variables at the hospital, LHD and SLA levels that were available for this study generally showed no significant effect. CONCLUSIONS: Investigations into the causes of low-value care and initiatives to reduce low-value care might best be targeted at the hospital level, as the high variation at this level suggests the greatest potential to reduce low-value care.
  
  
  
  
**SLA**
-
6
of
10
  

### Safety and biodistribution of sulfated archaeal glycolipid archaeosomes as vaccine adjuvants.

6. Hum Vaccin Immunother. 2018 Jul 3;14(7):1746-1759. doi: 10.1080/21645515.2017.1423154 Epub 2018 Jan 30.
  
  
Akache B(1), Stark FC(1), Iqbal U(1), Chen W(1), Jia Y(1), Krishnan L(1), McCluskie MJ(1).
  
  
Author information: (1)a Human Health Therapeutics, National Research Council Canada , Ottawa , Cana ...
  
  
Archaeosomes are liposomes comprised of ether lipids derived from various archaea. Unlike conventional ester-linked liposomes, archaeosomes exhibit high pH and thermal stability. As adjuvants, archaeosomes can induce robust, long-lasting humoral and cell-mediated immune responses and enhance protection in murine models of infectious disease and cancer. Archaeosomes constituted with total polar lipids (TPL) of various archaea are relatively complex, comprising >10 different lipid compounds. Archaeosomes can be constituted with semi-synthetic glycerolipids built on ether-linked isoprenoid phytanyl cores with varied synthetic glycol- and amino-head groups. However, such semi-synthetic archaeosomes involve many synthetic steps to arrive at the final desired glycolipid composition. We have developed a novel archaeosome formulation comprising a sulfated saccharide group covalently linked to the free sn-1 hydroxyl backbone of an archaeal core lipid (sulfated S-lactosylarchaeol, SLA) mixed with uncharged glycolipid (lactosylarchaeol, LA). This new class of adjuvants can be easily synthesized and retains strong immunostimulatory activity for induction of cell-mediated immunity following systemic immunization. Herein, we demonstrate the safety of SLA/LA archaeosomes following intramuscular injection to mice and evaluate the immunogenicity, in vivo distribution and cellular uptake of antigen (ovalbumin) encapsulated into SLA/LA archaeosomes. Overall, we have found that semi-synthetic sulfated glycolipid archaeosomes are a safe and effective novel class of adjuvants capable of inducing strong antigen-specific immune responses in mice and protection against subsequent B16 melanoma tumor challenge. A key step in their mechanism of action appears to be the recruitment of immune cells to the injection site and the subsequent trafficking of antigen to local draining lymph nodes.
  
  
  
  
**SLA**
-
7
of
10
  

### Sulfated archaeal glycolipid archaeosomes as a safe and effective vaccine adjuvant for induction of cell-mediated immunity.

7. Hum Vaccin Immunother. 2017 Dec 2;13(12):2772-2779. doi: 10.1080/21645515.2017.1316912 Epub 2017 May 24.
  
  
McCluskie MJ(1), Deschatelets L(1), Krishnan L(1).
  
  
Author information: (1)a Human Health Therapeutics , National Research Council Canada , Ottawa , Can ...
  
  
Archaeosomes are liposomal vesicles composed of ether glycerolipids unique to the domain of Archaea. Unlike conventional ester-linked liposomes, archaeosomes exhibit high stability and possess strong adjuvant and immunostimulatory properties making them an attractive vaccine delivery vehicle. Traditionally comprised of total polar lipids (TPL) or semi-synthetic phospho-glycerolipids of ether-linked isoprenoid phytanyl cores with varied glycol- and amino-head groups, archaeosomes can induce robust and long-lasting humoral and cell-mediated immune responses against antigenic cargo and provide protection in murine models of infectious disease and cancer. However, traditional TPL archaeosome formulations are relatively complex comprising several lipid species. Semi-synthetic archaeosomes tested previously contain a combination of several phospho-glycolipids (negative and neutral charged) to produce a stable, uniform-sized liposome formulation. Moreover, they involve many synthetic steps to arrive at the final desired glycolipid composition. Herein, we present a novel adjuvant formulation comprising a sulfated saccharide group covalently linked to the free sn-1 hydroxyl backbone of an archaeal core lipid (sulfated S-lactosylarchaeol, SLA). SLA individually or mixed with uncharged glyolipid (lactosylarchaeol, LA) constituted efficacious carrier vesicles for entrapped antigens (ovalbumin or melanoma associated tyrosinase-related protein 2 [TRP-2]) and induction of strong cell-mediated responses in mice and protection against subsequent B16 melanoma tumor challenge. Thus, semi-synthetic sulfated glycolipid archaeosomes represent a new class of adjuvants that will potentially ease manufacturing and scale-up, while retaining immunostimulatory activity.
  
  
  
  
**SLA**
-
8
of
10
  

### Multiple clear cell acanthomas and a sebaceous lymphadenoma presenting in a patient with Cowden syndrome - a case report.

8. J Cutan Pathol. 2017 Jan;44(1):79-82. doi: 10.1111/cup.12823 Epub 2016 Oct 20.
  
  
Potenziani S(1), Applebaum D(2), Krishnan B(1)(3), Gutiérrez C(1), Diwan AH(1)(2).
  
  
Author information: (1)Department of Pathology & Immunology, Baylor College of Medicine, Houston, TX ...
  
  
Cowden syndrome (CS) is an uncommon autosomal dominant multiorgan/system genodermatosis. It is characterized by the development of multiple hamartomas of endodermal, mesodermal and ectodermal origin, an increased lifetime risk of breast, thyroid, endometrial and other cancers and an identifiable germline mutation. Mucocutaneous hamartomas are the most common lesions seen and mainly include facial trichilemmomas, oral mucosal papillomas and benign acral keratoses. Herein, we report a case of a 63-year-old Caucasian male with a long-established diagnosis of CS and history of thyroid cancer, colonic polyps, and innumerable trichilemmomas, seborrheic keratoses, squamous papillomas and non-melanoma skin cancers excised in the past. He presented in four separate occasions with small skin-colored papulonodular lesions that upon excision revealed to be clear cell acanthomas. He also developed a tumor in the preauricular area that was completely resected and was found to be a sebaceous lymphadenoma (SLA) of the parotid gland. This is to our knowledge, the second report of clear cell acanthoma and also the second reported case of SLA in a patient with CS.
  
  
  
  
**SLA**
-
9
of
10
  

### Stereotactic laser ablation as treatment for brain metastases that recur after stereotactic radiosurgery: a multiinstitutional experience.

9. Neurosurg Focus. 2016 Oct;41(4):E11. doi: 10.3171/2016.7.FOCUS16227
  
  
Ali MA(1), Carroll KT(1), Rennert RC(1), Hamelin T(2), Chang L(3), Lemkuil BP(3), Sharma M(4), Barnh ...
  
  
Author information: (1)Departments of 1 Neurosurgery. (2)Radiology, and. (3)Anesthesia, University o ...
  
  
OBJECTIVE Therapeutic options for brain metastases (BMs) that recur after stereotactic radiosurgery (SRS) remain limited. METHODS The authors provide the collective experience of 4 institutions where treatment of BMs that recurred after SRS was performed with stereotactic laser ablation (SLA). RESULTS Twenty-six BMs (in 23 patients) that recurred after SRS were treated with SLA (2 patients each underwent 2 SLAs for separate lesions, and a third underwent 2 serial SLAs for discrete BMs). Histological findings in the BMs treated included the following: breast (n = 6); lung (n = 6); melanoma (n = 5); colon (n = 2); ovarian (n = 1); bladder (n = 1); esophageal (n = 1); and sarcoma (n = 1). With a median follow-up duration of 141 days (range 64-794 days), 9 of the SLA-treated BMs progressed despite treatment (35%). All cases of progression occurred in BMs in which < 80% ablation was achieved, whereas no disease progression was observed in BMs in which ≥ 80% ablation was achieved. Five BMs were treated with SLA, followed 1 month later by adjuvant SRS (5 Gy daily × 5 days). No disease progression was observed in these patients despite ablation efficiency of < 80%, suggesting that adjuvant hypofractionated SRS enhances the efficacy of SLA. Of the 23 SLA-treated patients, 3 suffered transient hemiparesis (13%), 1 developed hydrocephalus requiring temporary ventricular drainage (4%), and 1 patient who underwent SLA of a 28.9-cm3 lesion suffered a neurological deficit requiring an emergency hemicraniectomy (4%). Although there is significant heterogeneity in corticosteroid treatment post-SLA, most patients underwent a 2-week taper. CONCLUSIONS Stereotactic laser ablation is an effective treatment option for BMs in which SRS fails. Ablation of ≥ 80% of BMs is associated with decreased risk of disease progression. The efficacy of SLA in this setting may be augmented by adjuvant hypofractionated SRS.
  
  
  
  
**SLA**
-
10
of
10
  

### Maintenance of Efficacy and Continuing Safety of Golimumab for Active Ulcerative Colitis: PURSUIT-SC Maintenance Study Extension Through 1 Year.

10. Clin Transl Gastroenterol. 2016 Apr 28;7(4):e168. doi: 10.1038/ctg.2016.24
  
  
Gibson PR(1), Feagan BG(2), Sandborn WJ(3), Marano C(4), Strauss R(4), Johanns J(4), Padgett L(4), C ...
  
  
Author information: (1)Department of Gastroenterology, Alfred Hospital, Monash University and Alfred ...
  
  
OBJECTIVES: The safety and efficacy of subcutaneous golimumab through 2 years of maintenance therapy was evaluated in patients with moderate-to-severe ulcerative colitis (UC). METHODS: Patients completing treatment through week 52 (placebo, golimumab 50, 100, every-4-weeks (q4w)) and evaluations at week 54 were eligible for this long-term extension (LTE) trial. Patients receiving placebo or golimumab 50 mg with worsening disease during the LTE could receive golimumab 100 mg. Efficacy assessments included the Mayo physician's global assessment (PGA) subscore, inflammatory bowel disease questionnaire (IBDQ), and corticosteroid use. Patients who were randomized to golimumab at PURSUIT-Maintenance baseline and continued receiving golimumab during the LTE were analyzed for efficacy (using intention-to-treat and "as observed" analyses; N=195) and safety (N=200). Patients treated with golimumab at any time from induction baseline through week 104 (N=1240) constituted the overall safety population. RESULTS: Baseline demographics and disease characteristics of patients entering the LTE receiving golimumab were similar to those of all patients randomized to golimumab maintenance at baseline. At week 104, 80.5% (157/195) of patients had a PGA=0/1 (range weeks 56-104: 80.5-91.8%) and 56.4% (110/195) had a PGA=0 (weeks 56-104: range: 53.8-58.5%). Through week 104, 86% of patients maintained inactive or mild disease activity. Among 174 corticosteroid-free patients at week 54, 88.5% remained corticosteroid-free at week 104. At week 104, 62.2% (120/193) had an IBDQ score ≥170. Tuberculosis, opportunistic infection, and malignancy rates were low, and the overall safety profile was similar to that reported through week 54. Two non-melanoma skin cancers, one metastatic colon cancer, and two deaths (biventricular heart dysfunction, sepsis) occurred between weeks 54 and 104. CONCLUSION: Subcutaneous golimumab q4w through 2 years maintained clinical benefit and reduced corticosteroid use among patients who did well in the maintenance study. No new safety signals were observed.
  
  
  
  
  

---

  

# SLC11A1

**Signature gene in:** MDM
  
**Differentially expressed in:** MDM
  
  
**SLC11A1**
-
1
of
3
  

### Transcriptomic signatures in peripheral CD4(+)T-lymphocytes may reflect melanoma staging and immunotherapy responsiveness prior to ICI initiation.

1. Front Immunol. 2025 Mar 28;16:1529707. doi: 10.3389/fimmu.2025.1529707 eCollection 2025.
  
  
Palli E(#)(1), Lavigne M(#)(2), Verginis P(2), Alissafi T(3), Anastasopoulou A(1), Lyrarakis G(1), K ...
  
  
Author information: (1)First Department of Internal Medicine, Laikon General Hospital, National and ...
  
  
BACKGROUND AND PURPOSE: Promoting adaptive immunity with ICIs has drastically improved melanoma prognosis, but not for all patients. Some cases relapse in the first few months, while others keep durable benefit, even after immunotherapy discontinuation. To identify cellular/molecular signatures in peripheral blood that could differentiate advanced from metastatic melanoma and predict dynamics for primary/secondary immune escape, we examined 100 consecutive patients with stage III/IV melanoma scheduled to start ICIs. MATERIALS AND METHODS: At melanoma diagnosis, a multiparameter flow cytometric analysis and purification scheme using standard conjugated antibodies were performed for all individuals prior to ICI initiation. In each stage(III/IV) according to their RFS/PFS, we retrospectively selected the cases with the clearest clinical outcomes and focused our analysis on the extreme responders(n=7) and non-responders(n=7) to characterize the transcriptomes of circulating CD4+T-cells by bulk RNA-seq, Differential Expression Analysis(DEA)and Gene Ontology(GO)enrichment analysis. Based on our selected patient cohort, we examined for differentially expressed genes(DEGs)and key-pathways that appear preferentially activated in stage III vs. IV melanoma, and in long vs. short immunotherapy responders. RESULTS: Although circulating immune-cells did not numerically differ in both sets of analysis(staging and ICI responsiveness), DEA and GO data showed that patients could be clustered separately, identifying 189vs.92 DEGs in stage IV/III and 101vs.47 DEGs in early progressors/long responders. These DEGs were functionally implicated in distinct pathways. For metastatic cases: inflammatory response(logp-value=-9.2:ADGRE5/2,CYBA,GRN,HMOX1,IRF5,ITGAM), adaptive immunity(logp-value=-7.7:CD1C,CD74,CYBB,NCF2,CTSA,S100A8/9,BCL3,FCER1G), T-cell activation(logp-value=-6.3:BCL3,CD1C,CD74,FCER1G,FGL2)and lipid metabolism/catabolism(logp-value=-2.5/-2.6:ARF3,GPX1,MVD,OCRL,PCCB,CTSA,PNPLA2,NAGLU,GBA2,ABHD4); while in early-progressors to ICIs: immune effector processing(logp-value=-13.7:BCL6,FGR,HLA-DQA1/DQB1,HLA-DRA,HLA-DRB1/DRB5,NKG7,SLC11A1,TYROBP,SPON2,HAVCR2),PD-1(logp-value=-10.2:HLA-DQA1/DQB1,HLA-DRA,HLA-DRB1/DRB5)and IFN signaling(logp-value=-8.5: HLA-DQA1/DQB1,HLA-DRA,HLA-DRB1/DRB5,NCAM1,IFITM3),positive regulation of T-cell activation(logp-value=-7.7:BCL6,HLA-DQA1/DQB1,HLA-DRA,HLA-DRB1/DRB5,SASH3,HAVCR2)and CD28 co-stimulation(logp-value=-10.3:HLA-DQA1/DQB1,HLA-DRA,HLA-DRB1/DRB5), supporting an immune-mediated behavior. CONCLUSIONS: Specific pathways and marker genes in the peripheral CD4+T-cells may predetermine melanoma staging and immunotherapy resistance.
  
  
  
  
**SLC11A1**
-
2
of
3
  

### Genes Involved in Immune Reinduction May Constitute Biomarkers of Response for Metastatic Melanoma Patients Treated with Targeted Therapy.

2. Biomedicines. 2022 Jan 26;10(2):284. doi: 10.3390/biomedicines10020284
  
  
Berciano-Guerrero MA(1)(2), Lavado-Valenzuela R(2)(3), Moya A(3)(4), delaCruz-Merino L(5), Toscano F ...
  
  
Author information: (1)Medical Oncology, IBIMA, Medical Oncology Intercenter Unit, Hospital Regional ...
  
  
Targeted therapy in metastatic melanoma often achieves a major tumour regression response and significant long-term survival via the release of antigens that reinduce immunocompetence. The biomarkers thus activated may guide the prediction of response, but this association and its mechanism have yet to be established. Blood samples were collected from nineteen consecutive patients with metastatic melanoma before, during, and after treatment with targeted therapy. Differential gene expression analysis was performed, which identified the genes involved in the treatment, both in the first evaluation of response and during progression. Although clinical characteristics of the patients were poorer than those obtained in pivotal studies, radiological responses were similar to those reported previously (objective response rate: 73.7%). In the first tumour assessment, the expression of some genes increased (CXCL-10, SERPING1, PDL1, and PDL2), while that of others decreased (ARG1, IL18R1, IL18RAP, IL1R1, ILR2, FLT3, SLC11A1, CD163, and S100A12). The analysis of gene expression in blood shows that some are activated and others inhibited by targeted therapy. This response pattern may provide biomarkers of the immune reinduction response, which could be used to study potential combination treatments. Nevertheless, further studies are needed to validate these results.
  
  
  
  
**SLC11A1**
-
3
of
3
  

### Altered expression of the iron transporter Nramp1 (SLC11A1) during fetal development of the retinal pigment epithelium in microphthalmia-associated transcription factor Mitf(mi) and Mitf(vitiligo) mouse mutants.

3. Exp Eye Res. 2008 Feb;86(2):419-33. doi: 10.1016/j.exer.2007.11.015 Epub 2007 Dec 4.
  
  
Gelineau-van Waes J(1), Smith L, van Waes M, Wilberding J, Eudy JD, Bauer LK, Maddox J.
  
  
Author information: (1)Department of Genetics, Cell Biology & Anatomy, University of Nebraska Medica ...
  
  
Microphthalmia-associated transcription factor (Mitf) is expressed in neural crest cell-derived melanocytes, and in the retinal pigment epithelium (RPE) during ocular development. Mutations in Mitf are associated with auditory/visual/pigmentary syndromes in humans. Mitf(mi/mi) mouse mutants lack pigmentation, and are microphthalmic, while Mitf(vit/vit) mouse mutants display abnormal RPE pigmentation, and progressive retinal degeneration. Microarray analysis was used to identify novel downstream gene targets/pathways in the RPE that are altered by mutations in the transcription factor Mitf. Using the Affymetrix platform, gene expression profiles were generated using the eyes of E13.5 mouse fetuses that were wildtype, heterozygous, or homozygous for the Mitf(mi) mutation. In a separate experiment, eyes from E13.5 mouse fetuses homozygous for the Mitf(vit) mutation were compared to eyes from the C57BL/6 control background strain. Statistical analyses were performed using robust multiarray average, mixed-effects ANOVA and random-variance t-tests. Altered expression of genes involved in pigment formation, melanosome biogenesis/transport, and redox homeostasis were observed. Twelve genes were commonly mis-regulated in the eyes of both Mitf mutants: 10 of these genes were downregulated in both mutants relative to controls, while 2 of the genes (Nramp1 (SLC11A1) and epoxide hydrolase) were downregulated in Mitf(mi/mi) mutants, and conversely, upregulated in Mitf(vit/vit) mutants. Quantitative RT-PCR and immunohistochemistry were used to confirm altered gene/protein expression. RPE expression of the Fe(+2) iron transporter Nramp1 (SLC11A1) has not previously been reported. Fe(+2) is an important co-factor utilized by the iron-dependent isomerohydrolase RPE65 in the retinoid visual cycle. However, excess accumulation of Fe(+2) in the RPE has recently been associated with oxidative damage and age-related macular degeneration. Abnormal pigmentation and increased activity of SLC11A1 in the RPE of Mitf(vit) mice may contribute to the pathology and progressive retinal degeneration observed in these mutants.
  
  
  
  
  

---

  

# SLC1A3

**Signature gene in:** MDM
  
**Differentially expressed in:** MDM
  
  
**SLC1A3**
-
1
of
1
  

### Generation of an immortalized astrocyte cell line from H-2Kb-tsA58 mice to study the role of astrocytes in brain metastasis.

1. Int J Oncol. 2009 Oct;35(4):665-72. doi: 10.3892/ijo\_00000378
  
  
Langley RR(1), Fan D, Guo L, Zhang C, Lin Q, Brantley EC, McCarty JH, Fidler IJ.
  
  
Author information: (1)Department of Cancer Biology, Unit 173, The University of Texas MD Anderson C ...
  
  
Astrocytes play a critical role in maintaining cerebral homeostasis and their dysregulation is thought to contribute to the pathogenesis of several diseases, including brain cancer and metastasis. Similar to the human disease, we found that lung and melanoma metastases in the mouse brain are accompanied by a reactive gliosis. To begin to study the biology of astrocytes and examine how these cells might contribute to metastasis formation and progression in the brain, we generated a conditionally immortal astrocyte cell line from H-2Kb-tsA58 mice. Astrocytes grown in culture expressed glial fibrillary acid protein (GFAP), glutamate receptor 1, and the N-methyl-D-aspartate (NMDA) receptor. Astrocytes also expressed the glial-specific transporters excitatory amino acid transporter 1 (EAAT1) and EAAT2. Astrocytes grown under permissive conditions (33 degrees C) expressed SV40 large T antigen and had a doubling time of 36 h, whereas expression of SV40 large T antigen was negligible in astrocytes grown at 37 degrees C for 72 h, which coincided with a plateau in cell division. In a co-culture assay with human lung adenocarcinoma cells (PC14-PE6), astrocytes activated programs in the tumor cells that signal for cell division and survival. Hence, the immortalized cell line will be useful for studying the role of astrocytes in disease processes in the brain, such as metastasis.
  
  
  
  
  

---

  

# SLC8A1

**Signature gene in:** Monocytes
  
**Differentially expressed in:** Monocytes
  
  
**SLC8A1**
-
1
of
1
  

### Publisher Correction: LKB1-SIK2 loss drives uveal melanoma proliferation and hypersensitivity to SLC8A1 and ROS inhibition.

1. EMBO Mol Med. 2024 Sep;16(9):2262-2267. doi: 10.1038/s44321-024-00114-1
  
  
Proteau S(1)(2), Krossa I(#)(1)(2), Husser C(#)(1)(2), Guéguinou M(3)(4), Sella F(5), Bille K(1)(2), ...
  
  
Author information: (1)University Côte d'Azur, Nice, France. (2)Inserm, Biology and Pathologies of m ...
  
  
Erratum for EMBO Mol Med. 2023 Dec 7;15(12):e17719. doi: 10.15252/emmm.202317719.
  
  
  
  
  

---

  

# SORBS1

**Signature gene in:** Endothelial cells
  
**Differentially expressed in:** Endothelial cells
  
  
**SORBS1**
-
1
of
1
  

### The adapter protein c-Cbl-associated protein (CAP) protects from acute CVB3-mediated myocarditis through stabilization of type I interferon production and reduced cytotoxicity.

1. Basic Res Cardiol. 2014 May;109(3):411. doi: 10.1007/s00395-014-0411-3 Epub 2014 Apr 24.
  
  
Valaperti A(1), Nishii M, Liu Y, Yang H, Naito K, Liu PP, Eriksson U.
  
  
Author information: (1)Division of Cardiology, Heart and Stroke/Richard Lewar Centre of Excellence, ...
  
  
c-Cbl-associated protein (CAP), also called SORBS1 or ponsin, has been described as an essential adapter protein in the insulin-signalling pathway. Here, we describe for the first time a unique protective role for CAP in viral myocarditis. Mortality and heart failure development were increased in CAP(-/-) mice compared to CAP(+/+) littermates after Coxsackievirus (CVB3) infection. Mechanistically, CAP protected from tissue apoptosis because of reduced CD8(+) T and natural killer cell cytotoxicity. Despite reduced cytotoxic elimination of CVB3-infected cells in CAP(+/+) hearts, however, CAP enhanced interferon regulatory factor 3 (IRF3)-dependent antiviral type I interferon production and decreased viral proliferation in vitro by binding to the cytoplasmic RIG-I-like receptor melanoma differentiation-associated protein 5 (MDA5). Taken together, these findings reveal a novel modulatory role for CAP in the heart as a key protein stabilizing antiviral type I interferon production, while protecting from excessive cytotoxic responses. Our study will help to define future strategies to develop treatments to limit detrimental responses during viral heart inflammation.
  
  
  
  
  

---

  

# ST3GAL1

**Signature gene in:** Endothelial cells
  
**Differentially expressed in:** Endothelial cells
  
  
**ST3GAL1**
-
1
of
7
  

### Screening the human miRNA interactome reveals coordinated up-regulation in melanoma, adding bidirectional regulation to miRNA networks.

1. Sci Adv. 2025 Jan 10;11(2):eadr0277. doi: 10.1126/sciadv.adr0277 Epub 2025 Jan 10.
  
  
Jame-Chenarboo F(1), Reyes JN(1), Twells NM(1), Ng HH(1), Macdonald D(1), Hernando E(2)(3), Mahal LK ...
  
  
Author information: (1)Department of Chemistry, University of Alberta, Edmonton, Canada. (2)Departme ...
  
  
Cellular protein expression is coordinated posttranscriptionally by an intricate regulatory network. The current presumption is that microRNAs (miRNAs) work by repression of functionally related targets within a system. In recent work, up-regulation of protein expression via direct interactions of messenger RNA with miRNA has been found in dividing cells, providing an additional mechanism of regulation. Herein, we demonstrate coordinated up-regulation of functionally coupled proteins by miRNA. We focused on CD98hc, the heavy chain of the amino acid transporter LAT-1, and α-2,3-sialyltransferases ST3GAL1 and ST3GAL2, which are critical for CD98hc stability in melanoma. Profiling miRNA regulation using our high-throughput miRFluR assay, we identified miRNA that up-regulated the expression of both CD98hc and either ST3GAL1 or ST3GAL2. These co-up-regulating miRNAs were enriched in melanoma datasets associated with transformation and progression. Our findings add co-up-regulation by miRNA into miRNA regulatory networks and add a bidirectional twist to the impact miRNAs have on protein regulation and glycosylation.
  
  
  
  
**ST3GAL1**
-
2
of
7
  

### Synthesis of α-Hydroxy-1,2,3-Triazole-linked Sialyltransferase Inhibitors and Evaluation of Selectivity Towards ST3GAL1, ST6GAL1 and ST8SIA2.

2. ChemMedChem. 2024 Aug 19;19(16):e202400088. doi: 10.1002/cmdc.202400088 Epub 2024 Jul 1.
  
  
Szabo R(1), Dobie C(1)(2), Montgomery AP(1), Steele H(1), Yu H(1)(2)(3), Skropeta D(1)(2).
  
  
Author information: (1)School of Chemistry & Molecular Bioscience, University of Wollongong, Wollong ...
  
  
Tumour-derived sialoglycans, bearing the charged nonulosonic sugar sialic acid at their termini, play a critical role in tumour cell adhesion and invasion, as well as evading cell death and immune surveillance. Sialyltransferases (ST), the enzymes responsible for the biosynthesis of sialylated glycans, are highly upregulated in cancer, with tumour hypersialylation strongly correlated with tumour growth, metastasis and drug resistance. As a result, desialylation of the tumour cell surface using either targeted delivery of a pan-ST inhibitor (or sialidase) or systemic delivery of a non-toxic selective ST inhibitors are being pursued as potential new anti-metastatic strategies against multiple cancers including pancreatic, ovarian, breast, melanoma and lung cancer. Herein, we have employed molecular modelling to give insights into the selectivity observed in a series of selective ST inhibitors that incorporate a uridyl ring in place of the cytidine of the natural donor (CMP-Neu5Ac) and replace the charged phosphodiester linker of classical ST inhibitors with a neutral α-hydroxy-1,2,3-triazole linker. The inhibitory activities of the nascent compounds were determined against recombinant human ST enzymes (ST3GAL1, ST6GAL1, ST8SIA2) showing promising activity and selectivity towards specific ST sub-types. Our ST inhibitors are non-toxic and show improved synthetic accessibility and drug-likeness compared to earlier nucleoside-based ST inhibitors.
  
  
  
  
**ST3GAL1**
-
3
of
7
  

### Development of a Novel, Potent, and Selective Sialyltransferase Inhibitor for Suppressing Cancer Metastasis.

3. Int J Mol Sci. 2024 Apr 12;25(8):4283. doi: 10.3390/ijms25084283
  
  
Tsai HE(1), Chen CL(1), Chang TT(2), Fu CW(1)(3), Chen WC(1)(4), Perez SJLP(1)(2)(5)(6), Hsiao PW(7) ...
  
  
Author information: (1)Institute of Chemistry, Academia Sinica, Taipei 115, Taiwan. (2)Biomedical Tr ...
  
  
Sialyltransferase-catalyzed membrane protein and lipid glycosylation plays a vital role as one of the most abundant post-translational modifications and diversification reactions in eukaryotes. However, aberrant sialylation has been associated with cancer malignancy and metastasis. Sialyltransferases thus represent emerging targets for the development of small molecule cancer drugs. Herein, we report the inhibitory effects of a recently discovered lithocholic acid derivative FCW393 on sialyltransferase catalytic activity, integrin sialyation, cancer-associated signal transduction, MDA-MB-231 and B16F10 cell migration and invasion, and in in vivo studies, on tumor growth, metastasis, and angiogenesis. FCW393 showed effective and selective inhibition of the sialyltransferases ST6GAL1 (IC50 = 7.8 μM) and ST3GAL3 (IC50 = 9.45 μM) relative to ST3GAL1 (IC50 > 400 μM) and ST8SIA4 (IC50 > 100 μM). FCW393 reduced integrin sialylation in breast cancer and melanoma cells dose-dependently and downregulated proteins associated with the integrin-regulated FAK/paxillin and GEF/Rho/ROCK pathways, and with the VEGF-regulated Akt/NFκB/HIF-1α pathway. FCW393 inhibited cell migration (IC50 = 2.6 μM) and invasion in in vitro experiments, and in in vivo studies of tumor-bearing mice, FCW393 reduced tumor size, angiogenesis, and metastatic potential. Based on its demonstrated selectivity, cell permeability, relatively low cytotoxicity (IC50 = 55 μM), and high efficacy, FCW393 shows promising potential as a small molecule experimental tool compound and a lead for further development of a novel cancer therapeutic.
  
  
  
  
**ST3GAL1**
-
4
of
7
  

### Integrated in vivo functional screens and multi-omics analyses identify α-2,3-sialylation as essential for melanoma maintenance.

4. bioRxiv [Preprint]. 2024 Mar 12:2024.03.08.584072. doi: 10.1101/2024.03.08.584072
  
  
Agrawal P(1)(2)(3), Chen S(4), de Pablos A(1)(2)(5), Jame-Chenarboo F(6), Miera Saenz de Vega E(7), ...
  
  
Author information: (1)Department of Pathology, NYU Grossman School of Medicine, New York. (2)Interd ...
  
  
Glycosylation is a hallmark of cancer biology, and altered glycosylation influences multiple facets of melanoma growth and progression. To identify glycosyltransferases, glycans, and glycoproteins essential for melanoma maintenance, we conducted an in vivo growth screen with a pooled shRNA library of glycosyltransferases, lectin microarray profiling of benign nevi and melanoma patient samples, and mass spectrometry-based glycoproteomics. We found that α-2,3 sialyltransferases ST3GAL1 and ST3GAL2 and corresponding α-2,3-linked sialosides are upregulated in melanoma compared to nevi and are essential for melanoma growth in vivo and in vitro. Glycoproteomics revealed that glycoprotein targets of ST3GAL1 and ST3GAL2 are enriched in transmembrane proteins involved in growth signaling, including the amino acid transporter Solute Carrier Family 3 Member 2 (SLC3A2/CD98hc). CD98hc suppression mimicked the effect of ST3GAL1 and ST3GAL2 silencing, inhibiting melanoma cell proliferation. We found that both CD98hc protein stability and its pro-survival effect in melanoma are dependent upon α-2,3 sialylation mediated by ST3GAL1 and ST3GAL2. In summary, our studies reveal that α-2,3-sialosides functionally contribute to melanoma maintenance, supporting ST3GAL1 and ST3GAL2 as novel therapeutic targets in these tumors.
  
  
  
  
**ST3GAL1**
-
5
of
7
  

### ST3 beta-galactoside alpha-2,3-sialyltransferase 1 (ST3GAL1) synthesis of Siglec ligands mediates anti-tumour immunity in prostate cancer.

5. Commun Biol. 2024 Mar 6;7(1):276. doi: 10.1038/s42003-024-05924-0
  
  
Garnham R(1), Geh D(1), Nelson R(2), Ramon-Gil E(1), Wilson L(2), Schmidt EN(3)(4), Walker L(2), Ada ...
  
  
Author information: (1)Newcastle University, Centre for Cancer, Newcastle University Biosciences Ins ...
  
  
Immune checkpoint blockade has yet to produce robust anti-cancer responses for prostate cancer. Sialyltransferases have been shown across several solid tumours, including breast, melanoma, colorectal and prostate to promote immune suppression by synthesising sialoglycans, which act as ligands for Siglec receptors. We report that ST3 beta-galactoside alpha-2,3-sialyltransferase 1 (ST3GAL1) levels negatively correlate with androgen signalling in prostate tumours. We demonstrate that ST3GAL1 plays an important role in modulating tumour immune evasion through the synthesises of sialoglycans with the capacity to engage the Siglec-7 and Siglec-9 immunoreceptors preventing immune clearance of cancer cells. Here, we provide evidence of the expression of Siglec-7/9 ligands and their respective immunoreceptors in prostate tumours. These interactions can be modulated by enzalutamide and may maintain immune suppression in enzalutamide treated tumours. We conclude that the activity of ST3GAL1 is critical to prostate cancer anti-tumour immunity and provide rationale for the use of glyco-immune checkpoint targeting therapies in advanced prostate cancer.
  
  
  
  
**ST3GAL1**
-
6
of
7
  

### ST3GAL1 is a target of the SOX2-GLI1 transcriptional complex and promotes melanoma metastasis through AXL.

6. Nat Commun. 2020 Nov 17;11(1):5865. doi: 10.1038/s41467-020-19575-2
  
  
Pietrobono S(1), Anichini G(1)(2), Sala C(3), Manetti F(4), Almada LL(5), Pepe S(1)(2), Carr RM(5), ...
  
  
Author information: (1)Core Research Laboratory - Institute for Cancer Research and Prevention (ISPR ...
  
  
Understanding the molecular events controlling melanoma progression is of paramount importance for the development of alternative treatment options for this devastating disease. Here we report a mechanism regulated by the oncogenic SOX2-GLI1 transcriptional complex driving melanoma invasion through the induction of the sialyltransferase ST3GAL1. Using in vitro and in vivo studies, we demonstrate that ST3GAL1 drives melanoma metastasis. Silencing of this enzyme suppresses melanoma invasion and significantly reduces the ability of aggressive melanoma cells to enter the blood stream, colonize distal organs, seed and survive in the metastatic environment. Analysis of glycosylated proteins reveals that the receptor tyrosine kinase AXL is a major effector of ST3GAL1 pro-invasive function. ST3GAL1 induces AXL dimerization and activation that, in turn, promotes melanoma invasion. Our data support a key role of the ST3GAL1-AXL axis as driver of melanoma metastasis, and highlight the therapeutic potential of targeting this axis to treat metastatic melanoma.
  
  
  
  
**ST3GAL1**
-
7
of
7
  

### Molecular cloning of a novel alpha2,3-sialyltransferase (ST3Gal VI) that sialylates type II lactosamine structures on glycoproteins and glycolipids.

7. J Biol Chem. 1999 Apr 23;274(17):11479-86. doi: 10.1074/jbc.274.17.11479
  
  
Okajima T(1), Fukumoto S, Miyazaki H, Ishida H, Kiso M, Furukawa K, Urano T, Furukawa K.
  
  
Author information: (1)Department of Biochemistry, Nagoya University School of Medicine, Tsurumai, N ...
  
  
A novel member of the human CMP-NeuAc:beta-galactoside alpha2, 3-sialyltransferase (ST) subfamily, designated ST3Gal VI, was identified based on BLAST analysis of expressed sequence tags, and a cDNA clone was isolated from a human melanoma line library. The sequence of ST3Gal VI encoded a type II membrane protein with 2 amino acids of cytoplasmic domain, 32 amino acids of transmembrane region, and a large catalytic domain with 297 amino acids; and showed homology to previously cloned ST3Gal III, ST3Gal IV, and ST3Gal V at 34, 38, and 33%, respectively. Extracts from L cells transfected with ST3Gal VI cDNA in a expression vector and a fusion protein with protein A showed an enzyme activity of alpha2, 3-sialyltransferase toward Galbeta1,4GlcNAc structure on glycoproteins and glycolipids. In contrast to ST3Gal III and ST3Gal IV, this enzyme exhibited restricted substrate specificity, i.e. it utilized Galbeta1,4GlcNAc on glycoproteins, and neolactotetraosylceramide and neolactohexaosylceramide, but not lactotetraosylceramide, lactosylceramide, or asialo-GM1. Consequently, these data indicated that this enzyme is involved in the synthesis of sialyl-paragloboside, a precursor of sialyl-Lewis X determinant.
  
  
  
  
  

---

  

# TBC1D16

**Signature gene in:** MDM
  
**Differentially expressed in:** MDM
  
  
**TBC1D16**
-
1
of
7
  

### Exploring the impact of deubiquitination on melanoma prognosis through single-cell RNA sequencing.

1. Front Genet. 2024 Dec 5;15:1509049. doi: 10.3389/fgene.2024.1509049 eCollection 2024.
  
  
Peng S(1), Xie J(2), He X(1).
  
  
Author information: (1)Department of Plastic Surgery, The Affiliated Friendship Plastic Surgery Hosp ...
  
  
BACKGROUND: Cutaneous melanoma, characterized by the malignant proliferation of melanocytes, exhibits high invasiveness and metastatic potential. Thus, identifying novel prognostic biomarkers and therapeutic targets is essential. METHODS: We utilized single-cell RNA sequencing data (GSE215120) from the Gene Expression Omnibus (GEO) database, preprocessing it with the Seurat package. Dimensionality reduction and clustering were executed through Principal Component Analysis (PCA) and Uniform Manifold Approximation and Projection (UMAP). Cell types were annotated based on known marker genes, and the AUCell algorithm assessed the enrichment of deubiquitination-related genes. Cells were categorized into DUB\_high and DUB\_low groups based on AUCell scores, followed by differential expression analysis. Importantly, we constructed a robust prognostic model utilizing various genes, which was evaluated in the TCGA cohort and an external validation cohort. RESULTS: Our prognostic model, developed using Random Survival Forest (RSF) and Ridge Regression methods, demonstrated excellent predictive performance, evidenced by high C-index and AUC values across multiple cohorts. Furthermore, analyses of immune cell infiltration and tumor microenvironment scores revealed significant differences in immune cell distribution and microenvironment characteristics between high-risk and low-risk groups. Functional experiments indicated that TBC1D16 significantly impacts the migration and proliferation of melanoma cells. CONCLUSION: This study highlights the critical role of deubiquitination in melanoma and presents a novel prognostic model that effectively stratifies patient risk. The model's strong predictive ability enhances clinical decision-making and provides a framework for future studies on the therapeutic potential of deubiquitination mechanisms in melanoma progression. Further validation and exploration of this model's applicability in clinical settings are warranted.
  
  
  
  
**TBC1D16**
-
2
of
7
  

### Characterisation of DNA methylation changes in EBF3 and TBC1D16 associated with tumour progression and metastasis in multiple cancer types.

2. Clin Epigenetics. 2019 Aug 5;11(1):114. doi: 10.1186/s13148-019-0710-5
  
  
Rodger EJ(1)(2), Chatterjee A(3)(4), Stockwell PA(5), Eccles MR(6)(7).
  
  
Author information: (1)Department of Pathology, Dunedin School of Medicine, University of Otago, 56 ...
  
  
BACKGROUND: Characteristic DNA methylation differences have been identified between primary and metastatic melanomas at EBF3 and/or TBC1D16 gene loci. To further evaluate whether these epigenetic changes may act more generally as drivers of tumour onset and metastasis, we have investigated DNA methylation changes involving EBF3 and TBC1D16 in additional publicly available data of multiple different tumour types. RESULTS: Promoter hypermethylation and gene body hypomethylation of EBF3 were observed in a number of metastatic tumour types, when compared to normal or primary tumour tissues, as well as in tumour vs normal tissues and in a colorectal primary/metastasis pair, although not all tumour samples or primary/metastasis cancer pairs exhibited altered patterns of EBF3 methylation. In addition, hypomethylation of TBC1D16 was observed in multiple tumours, including a breast cancer primary/metastasis pair, and to a lesser degree in melanoma, although again not all tumours or cancer primary/metastasis pairs exhibited altered patterns of methylation. CONCLUSIONS: These findings suggest characteristic DNA methylation changes in EBF3 and TBC1D16 are relatively common tumour-associated epigenetic events in multiple tumour types, which is consistent with a potential role as more general drivers of tumour progression.
  
  
  
  
**TBC1D16**
-
3
of
7
  

### Comprehensive DNA methylation study identifies novel progression-related and prognostic markers for cutaneous melanoma.

3. BMC Med. 2017 Jun 5;15(1):101. doi: 10.1186/s12916-017-0851-3
  
  
Wouters J(1)(2)(3)(4), Vizoso M(5), Martinez-Cardus A(5), Carmona FJ(5), Govaere O(1), Laguna T(5)(6 ...
  
  
Author information: (1)Translational Cell and Tissue Research, KU Leuven (University of Leuven), Leu ...
  
  
BACKGROUND: Cutaneous melanoma is the deadliest skin cancer, with an increasing incidence and mortality rate. Currently, staging of patients with primary melanoma is performed using histological biomarkers such as tumor thickness and ulceration. As disruption of the epigenomic landscape is recognized as a widespread feature inherent in tumor development and progression, we aimed to identify novel biomarkers providing additional clinical information over current factors using unbiased genome-wide DNA methylation analyses. METHODS: We performed a comprehensive DNA methylation analysis during all progression stages of melanoma using Infinium HumanMethylation450 BeadChips on a discovery cohort of benign nevi (n = 14) and malignant melanoma from both primary (n = 33) and metastatic (n = 28) sites, integrating the DNA methylome with gene expression data. We validated the discovered biomarkers in three independent validation cohorts by pyrosequencing and immunohistochemistry. RESULTS: We identified and validated biomarkers for, and pathways involved in, melanoma development (e.g., HOXA9 DNA methylation) and tumor progression (e.g., TBC1D16 DNA methylation). In addition, we determined a prognostic signature with potential clinical applicability and validated PON3 DNA methylation and OVOL1 protein expression as biomarkers with prognostic information independent of tumor thickness and ulceration. CONCLUSIONS: Our data underscores the importance of epigenomic regulation in triggering metastatic dissemination through the inactivation of central cancer-related pathways. Inactivation of cell-adhesion and differentiation unleashes dissemination, and subsequent activation of inflammatory and immune system programs impairs anti-tumoral defense pathways. Moreover, we identify several markers of tumor development and progression previously unrelated to melanoma, and determined a prognostic signature with potential clinical utility.
  
  
  
  
**TBC1D16**
-
4
of
7
  

### Targeting melanoma: unusual epigenetics reveals the dynamic rewiring of metastatic cells.

4. Epigenomics. 2015 Oct;7(7):1079-81. doi: 10.2217/epi.15.67 Epub 2015 Nov 6.
  
  
Vizoso M(1), Esteller M(1)(2)(3).
  
  
Author information: (1)Epigenetics & Biology Program (PEBC), Bellvitge Biomedical Research Institute ...
  
  
DOI: 10.2217/epi.15.67 PMID: 26541242 [Indexed for MEDLINE]
  
  
  
  
**TBC1D16**
-
5
of
7
  

### Epigenetic activation of a cryptic TBC1D16 transcript enhances melanoma progression by targeting EGFR.

5. Nat Med. 2015 Jul;21(7):741-50. doi: 10.1038/nm.3863 Epub 2015 Jun 1.
  
  
Vizoso M(1), Ferreira HJ(1), Lopez-Serra P(1), Carmona FJ(1), Martínez-Cardús A(1), Girotti MR(2), V ...
  
  
Author information: (1)Cancer Epigenetics and Biology Program, Bellvitge Biomedical Research Institu ...
  
  
Comment in Cell Cycle. 2015;14(18):2863-4. doi: 10.1080/15384101.2015.1076300.
  
  
  
  
**TBC1D16**
-
6
of
7
  

### TBC1D16 is a Rab4A GTPase activating protein that regulates receptor recycling and EGF receptor signaling.

6. Proc Natl Acad Sci U S A. 2012 Sep 25;109(39):15787-92. doi: 10.1073/pnas.1204540109 Epub 2012 Sep 10.
  
  
Goueli BS(1), Powell MB, Finger EC, Pfeffer SR.
  
  
Author information: (1)Department of Biochemistry, Stanford University School of Medicine, Stanford, ...
  
  
Rab4A is a master regulator of receptor recycling from endocytic compartments to the plasma membrane. The protein TBC1D16 is up-regulated in melanoma, and TBC1D16-overexpressing melanoma cells are dependent on TBC1D16. We show here that TBC1D16 enhances the intrinsic rate of GTP hydrolysis by Rab4A. TBC1D16 is both cytosolic and membrane associated; the membrane-associated pool colocalizes with transferrin and EGF receptors (EGFRs) and early endosome antigen 1, but not with LAMP1 protein. Expression of two TBC1D16 isoforms, but not the inactive R494A mutant, reduces transferrin receptor recycling but has no effect on transferrin receptor internalization. Expression of TBC1D16 alters GFP-Rab4A membrane localization. In HeLa cells, overexpression of TBC1D16 enhances EGF-stimulated EGFR degradation, concomitant with decreased EGFR levels and signaling. Thus, TBC1D16 is a GTPase activating protein for Rab4A that regulates transferrin receptor recycling and EGFR trafficking and signaling.
  
  
  
  
**TBC1D16**
-
7
of
7
  

### An integrated approach to uncover drivers of cancer.

7. Cell. 2010 Dec 10;143(6):1005-17. doi: 10.1016/j.cell.2010.11.013 Epub 2010 Dec 2.
  
  
Akavia UD(1), Litvin O, Kim J, Sanchez-Garcia F, Kotliar D, Causton HC, Pochanard P, Mozes E, Garraw ...
  
  
Author information: (1)Department of Biological Sciences, Columbia University, New York, NY 10027, U ...
  
  
Comment in Nat Rev Cancer. 2011 Feb;11(2):79.
  
  
  
  
  

---

  

# TCF4

**Signature gene in:** B cells
  
**Differentially expressed in:** B cells
  
  
**TCF4**
-
1
of
10
  

### A TCF4-dependent gene regulatory network confers resistance to immunotherapy in melanoma.

1. Cell. 2024 Jan 4;187(1):166-183.e25. doi: 10.1016/j.cell.2023.11.037
  
  
Pozniak J(1), Pedri D(2), Landeloos E(3), Van Herck Y(4), Antoranz A(5), Vanwynsberghe L(6), Nowosad ...
  
  
Author information: (1)Laboratory for Molecular Cancer Biology, Center for Cancer Biology, VIB, Leuv ...
  
  
To better understand intrinsic resistance to immune checkpoint blockade (ICB), we established a comprehensive view of the cellular architecture of the treatment-naive melanoma ecosystem and studied its evolution under ICB. Using single-cell, spatial multi-omics, we showed that the tumor microenvironment promotes the emergence of a complex melanoma transcriptomic landscape. Melanoma cells harboring a mesenchymal-like (MES) state, a population known to confer resistance to targeted therapy, were significantly enriched in early on-treatment biopsies from non-responders to ICB. TCF4 serves as the hub of this landscape by being a master regulator of the MES signature and a suppressor of the melanocytic and antigen presentation transcriptional programs. Targeting TCF4 genetically or pharmacologically, using a bromodomain inhibitor, increased immunogenicity and sensitivity of MES cells to ICB and targeted therapy. We thereby uncovered a TCF4-dependent regulatory network that orchestrates multiple transcriptional programs and contributes to resistance to both targeted therapy and ICB in melanoma.
  
  
  
  
**TCF4**
-
2
of
10
  

### VE-Cadherin modulates β-catenin/TCF-4 to enhance Vasculogenic Mimicry.

2. Cell Death Dis. 2023 Feb 17;14(2):135. doi: 10.1038/s41419-023-05666-7
  
  
Delgado-Bellido D(#)(1)(2)(3), Zamudio-Martínez E(#)(1), Fernández-Cortés M(1), Herrera-Campos AB(1) ...
  
  
Author information: (1)Instituto de Parasitología y Biomedicina López Neyra, CSIC, Granada, Spain. ( ...
  
  
Vasculogenic Mimicry (VM) refers to the capacity to form a blood network from aggressive cancer cells in an independent way of endothelial cells, to provide nutrients and oxygen leading to enhanced microenvironment complexity and treatment failure. In a previous study, we demonstrated that VE-Cadherin and its phosphorylation at Y658 modulated kaiso-dependent gene expression (CCND1 and Wnt 11) through a pathway involving Focal Adhesion kinase (FAK). In the present research, using a proteomic approach, we have found that β-catenin/TCF-4 is associated with nuclear VE-cadherin and enhances the capacity of malignant melanoma cells to undergo VM in cooperation with VE-Cadherin; in addition, preventing the phosphorylation of Y658 of VE-cadherin upon FAK disabling resulted in VE-Cadherin/β-catenin complex dissociation, increased β-catenin degradation while reducing TCF-4-dependent genes transcription (C-Myc and Twist-1). Uveal melanoma cells knockout for VE-Cadherin loses β-catenin expression while the rescue of VE-Cadherin (but not of the phosphorylation defective VE-Cadherin Y658F mutant) permits stabilization of β-catenin and tumor growth reduction in vivo experiments. In vivo, the concomitant treatment with the FAK inhibitor PF-271 and the anti-angiogenic agent bevacizumab leads to a strong reduction in tumor growth concerning the single treatment. In conclusion, the anomalous expression of VE-Cadherin in metastatic melanoma cells (from both uveal and cutaneous origins), together with its permanent phosphorylation at Y658, favors the induction of the aggressive VM phenotype through the cooperation of β-catenin with VE-Cadherin and by enhancing TCF-4 genes-dependent transcription.
  
  
  
  
**TCF4**
-
3
of
10
  

### A Cancer Associated Fibroblasts-Related Six-Gene Panel for Anti-PD-1 Therapy in Melanoma Driven by Weighted Correlation Network Analysis and Supervised Machine Learning.

3. Front Med (Lausanne). 2022 Apr 11;9:880326. doi: 10.3389/fmed.2022.880326 eCollection 2022.
  
  
Tian L(1), Long F(1), Hao Y(2), Li B(2), Li Y(3), Tang Y(1), Li J(1), Zhao Q(1), Chen J(1), Liu M(1) ...
  
  
Author information: (1)Key Laboratory of Clinical Laboratory Diagnostics, College of Laboratory Medi ...
  
  
BACKGROUND: Melanoma is a highly aggressive skin cancer with a poor prognosis and mortality. Immune checkpoint blockade (ICB) therapy (e.g., anti-PD-1 therapy) has opened a new horizon in melanoma treatment, but some patients present a non-responsive state. Cancer-associated fibroblasts (CAFs) make up the majority of stromal cells in the tumor microenvironment (TME) and have an important impact on the response to immunotherapy. There is still a lack of identification of CAFs-related predictors for anti-PD-1 therapy, although the establishment of immunotherapy biomarkers is well underway. This study aims to explore the potential CAFs-related gene panel for predicting the response to anti-PD-1 therapy in melanoma patients and elucidating their potential effect on TME. METHODS: Three gene expression datasets from melanoma patients without anti-PD-1 treatment, in a total of 87 samples, were downloaded from Gene Expression Omnibus (GEO) as the discovery sets (GSE91061) and validation sets (GSE78220 and GSE122220). The CAFs-related module genes were identified from the discovery sets by weighted gene co-expression network analysis (WGCNA). Concurrently, we utilized differential gene analysis on the discovery set to obtain differentially expressed genes (DEGs). Then, CAFs-related key genes were screened with the intersection of CAFs-related module genes and DEGs, succeeded by supervised machine learning-based identification. As a consequence of expression analysis, gene set enrichment analysis, survival analysis, staging analysis, TME analysis, and correlation analysis, the multidimensional systematic characterizations of the key genes were uncovered. The diagnostic performance of the CAFs-related gene panel was assessed by receiver operating characteristic (ROC) curves in the validation sets. Eventually, the CAFs-related gene panel was verified by the expression from the single-cell analysis. RESULTS: The six-gene panel associated with CAFs were finally identified for predicting the response to anti-PD-1 therapy, including CDK14, SYNPO2, TCF4, GJA1, CPXM1, and TFPI. The multigene panel demonstrated excellent combined diagnostic performance with the area under the curve of ROC reaching 90.5 and 75.4% ~100% in the discovery and validation sets, respectively. CONCLUSION: Confirmed by clinical treatment outcomes, the identified CAFs-related genes can be used as a promising biomarker panel for prediction to anti-PD-1 therapy response, which may serve as new immunotherapeutic targets to improve survival outcomes of melanoma patients.
  
  
  
  
**TCF4**
-
4
of
10
  

### The Critical Role of TRIB2 in Cancer and Therapy Resistance.

4. Cancers (Basel). 2021 May 30;13(11):2701. doi: 10.3390/cancers13112701
  
  
Mayoral-Varo V(1), Jiménez L(1), Link W(1).
  
  
Author information: (1)Instituto de Investigaciones Biomédicas "Alberto Sols" (CSIC-UAM), Arturo Dup ...
  
  
The Tribbles pseudokinases family consists of TRIB1, TRIB2, TRIB3 and STK40 and, although evolutionarily conserved, they have distinctive characteristics. Tribbles members are expressed in a context and cell compartment-dependent manner. For example, TRIB1 and TRIB2 have potent oncogenic activities in vertebrate cells. Since the identification of Tribbles proteins as modulators of multiple signalling pathways, recent studies have linked their expression with several pathologies, including cancer. Tribbles proteins act as protein adaptors involved in the ubiquitin-proteasome degradation system, as they bridge the gap between substrates and E3 ligases. Between TRIB family members, TRIB2 is the most ancestral member of the family. TRIB2 is involved in protein homeostasis regulation of C/EBPα, β-catenin and TCF4. On the other hand, TRIB2 interacts with MAPKK, AKT and NFkB proteins, involved in cell survival, proliferation and immune response. Here, we review the characteristic features of TRIB2 structure and signalling and its role in many cancer subtypes with an emphasis on TRIB2 function in therapy resistance in melanoma, leukemia and glioblastoma. The strong evidence between TRIB2 expression and chemoresistance provides an attractive opportunity for targeting TRIB2.
  
  
  
  
**TCF4**
-
5
of
10
  

### PGC1α Loss Promotes Lung Cancer Metastasis through Epithelial-Mesenchymal Transition.

5. Cancers (Basel). 2021 Apr 8;13(8):1772. doi: 10.3390/cancers13081772
  
  
Oh TI(1)(2), Lee M(3)(4)(5), Lee YM(1), Kim GH(1)(2), Lee D(6), You JS(7), Kim SH(8), Choi M(8), Jan ...
  
  
Author information: (1)Department of Biomedical Chemistry, College of Biomedical & Health Science, K ...
  
  
PGC1α oppositely regulates cancer metastasis in melanoma, breast, and pancreatic cancer; however, little is known about its impact on lung cancer metastasis. Transcriptome and in vivo xenograft analysis show that a decreased PGC1α correlates with the epithelial-mesenchymal transition (EMT) and lung cancer metastasis. The deletion of a single Pgc1α allele in mice promotes bone metastasis of KrasG12D-driven lung cancer. Mechanistically, PGC1α predominantly activates ID1 expression, which interferes with TCF4-TWIST1 cooperation during EMT. Bioinformatic and clinical studies have shown that PGC1α and ID1 are downregulated in lung cancer, and correlate with a poor survival rate. Our study indicates that TCF4-TWIST1-mediated EMT, which is regulated by the PGC1α-ID1 transcriptional axis, is a potential diagnostic and therapeutic target for metastatic lung cancer.
  
  
  
  
**TCF4**
-
6
of
10
  

### The VE-Cadherin/β-catenin signalling axis regulates immune cell infiltration into tumours.

6. Cancer Lett. 2021 Jan 1;496:1-15. doi: 10.1016/j.canlet.2020.09.026 Epub 2020 Sep 28.
  
  
Zhao Y(1), Li J(1), Ting KK(1), Chen J(2), Coleman P(1), Liu K(2), Wan L(1), Moller T(3), Vadas MA(1 ...
  
  
Author information: (1)Centre for the Endothelium, Vascular Biology Program, Centenary Institute, Th ...
  
  
Vascular normalisation, the process that reverses the structural and functional abnormalities seen in tumour-associated vessels, is also accompanied by changes in leucocyte trafficking. Our previous studies have shown the normalisation effects of the agent CD5-2 which acts to stabilise VE-Cadherin leading to increased penetration of CD8+ T cells but decreased infiltration of neutrophils (CD11b+Gr1hi) into tumour parenchyma. In the present study, we demonstrate that VE-Cadherin stabilisation through CD5-2 treatment of purified endothelial cells (ECs) results in a similar leucocyte-selective regulation of transmigration, suggesting the existence of an endothelial specific intrinsic mechanism. Further, we show by RNA sequencing (RNA-seq)-based transcriptomic analysis, that treatment of ECs with CD5-2 regulates chemokines known to be involved in leucocyte transmigration, including upregulation of CCL2 and CXCL10 that facilitate CD8+ T cell transmigration. Both in vitro and in vivo mechanistic studies revealed that the increased CCL2 expression was dependent on expression of VE-Cadherin and downstream activation of the AKT/GSK3β/β-catenin/TCF4 signalling pathway. CD5-2 treatment also contributed to the reorganisation of the cytoskeleton, inducing reorganisation of stress fibres to circumferential actin, which previously has been described as associated with the stabilisation of the endothelial barrier, and amplification of the transcellular migration of CD8+ T cells. Thus, we propose that promotion of endothelial junctional integrity during vascular normalisation not only inhibits vascular leak but also resets the endothelial dependent regulation of immune cell infiltration.
  
  
  
  
**TCF4**
-
7
of
10
  

### Cinobufagin Suppresses Melanoma Cell Growth by Inhibiting LEF1.

7. Int J Mol Sci. 2020 Sep 13;21(18):6706. doi: 10.3390/ijms21186706
  
  
Kim GH(1)(2), Fang XQ(1), Lim WJ(1), Park J(1), Kang TB(3), Kim JH(4), Lim JH(1)(2).
  
  
Author information: (1)Department of Biomedical Chemistry, College of Biomedical & Health Science, K ...
  
  
Constitutive activation of the β-catenin dependent canonical Wnt signaling pathway, which enhances tumor growth and progression in multiple types of cancer, is commonly observed in melanoma. LEF1 activates β-catenin/TCF4 transcriptional activity, promoting tumor growth and progression. Although several reports have shown that LEF1 is highly expressed in melanoma, the functional role of LEF1 in melanoma growth is not fully understood. While A375, A2058, and G361 melanoma cells exhibit abnormally high LEF1 expression, lung cancer cells express lower LEF1 levels. A luciferase assay-based high throughput screening (HTS) with a natural compound library showed that cinobufagin suppressed β-catenin/TCF4 transcriptional activity by inhibiting LEF1 expression. Cinobufagin decreases LEF1 expression in a dose-dependent manner and Wnt/β-catenin target genes such as Axin-2, cyclin D1, and c-Myc in melanoma cell lines. Cinobufagin sensitively attenuates cell viability and induces apoptosis in LEF1 expressing melanoma cells compared to LEF1-low expressing lung cancer cells. In addition, ectopic LEF1 expression is sufficient to attenuate cinobufagin-induced apoptosis and cell growth retardation in melanoma cells. Thus, we suggest that cinobufagin is a potential anti-melanoma drug that suppresses tumor-promoting Wnt/β-catenin signaling via LEF1 inhibition.
  
  
  
  
**TCF4**
-
8
of
10
  

### Silencing TCF4 Sensitizes Melanoma Cells to Vemurafenib Through Inhibiting GLUT3-Mediated Glycolysis.

8. Onco Targets Ther. 2020 May 29;13:4905-4915. doi: 10.2147/OTT.S245531 eCollection 2020.
  
  
Liu C(1), He S(1), Zhang J(2), Li S(3), Chen J(4), Han C(1).
  
  
Author information: (1)Department of Burn and Plastic Surgery, The Third Xiangya Hospital of Central ...
  
  
BACKGROUND: Vemurafenib is a selective BRAF inhibitor with significant early effects in melanoma, but resistance will develop with the duration of treatment. Therefore, overcoming vemurafenib resistance can effectively improve the survival rate of melanoma. The transcriptional activity of TCF4 is necessary to maintain the malignant phenotype of cancer cells. However, the effect of TCF4 on melanoma sensitivity to vemurafenib and the underlying mechanism is unclear. METHODS: Vemurafenib-resistant A375 (A375/Vem) and SK-Mel-28 (SK-Mel-28/Vem) cells were constructed by administering increasing concentrations of vemurafenib, and the expression of TCF4 was examined in parent and vemurafenib-resistant cells. TCF4 loss-function cells models were established in A375/Vem and SK-Mel-28/Vem cells, respectively. Cell survival, clone formation, and cell apoptosis were assessed. The downstream target gene of TCF4 was verified by chromatin immunoprecipitation. Finally, the effect of TCF4 on melanoma cells glycolysis was investigated and were performed. RESULTS: TCF4 expression was increased in vemurafenib-resistant melanoma cells, and knocking down TCF4 could promote the sensitivity of melanoma cells to vemurafenib. Mechanism investigation revealed that TCF4 could interact with GLUT3 and silencing TCF4 could inhibit GLUT3 expression. In addition, overexpression of GLUT3 reversed the growth and glycolysis of tumor cells that were inhibited by TCF4 knockdown. CONCLUSION: Our study demonstrates that TCF4 downregulation sensitizes melanoma cells to vemurafenib through inhibiting GLUT3-mediated glycolysis. These findings support TCF4 as an oncogene and provide new mechanism by which TCF4 confers chemotherapy resistance in melanoma.
  
  
  
  
**TCF4**
-
9
of
10
  

### PRAME promotes epithelial-to-mesenchymal transition in triple negative breast cancer.

9. J Transl Med. 2019 Jan 3;17(1):9. doi: 10.1186/s12967-018-1757-3
  
  
Al-Khadairi G(1), Naik A(2), Thomas R(2), Al-Sulaiti B(2), Rizly S(2), Decock J(3)(4).
  
  
Author information: (1)College of Health and Life Sciences (CHLS), Hamad Bin Khalifa University (HBK ...
  
  
BACKGROUND: The triple negative breast cancer (TNBC) paradox marks a major challenge in the treatment-decision making process. TNBC patients generally respond better to neoadjuvant chemotherapy compared to other breast cancer patients; however, they have a substantial higher risk of disease recurrence. We evaluated the expression of the tumor-associated antigen PReferentially Antigen expressed in MElanoma (PRAME) as a prognostic biomarker in breast cancer and explored its role in cell migration and invasion, key hallmarks of progressive and metastatic disease. METHODS: TCGA and GTeX datasets were interrogated to assess the expression of PRAME in relation to overall and disease-free survival. The role of PRAME in cell migration and invasion was investigated using gain- and loss-of-function TNBC cell line models. RESULTS: We show that PRAME promotes migration and invasion of TNBC cells through changes in expression of E-cadherin, N-cadherin, vimentin and ZEB1, core markers of an epithelial-to-mesenchymal transition. Mechanistic analysis of PRAME-overexpressing cells showed an upregulation of 11 genes (SNAI1, TCF4, TWIST1, FOXC2, IL1RN, MMP2, SOX10, WNT11, MMP3, PDGFRB, and JAG1) and downregulation of 2 genes (BMP7 and TSPAN13). Gene ontology analyses revealed enrichment of genes that are dysregulated in ovarian and esophageal cancer and are involved in transcription and apoptosis. In line with this, interrogation of TCGA and GTEx data demonstrated an increased PRAME expression in ovarian and esophageal tumor tissues in addition to breast tumors where it is associated with worse survival. CONCLUSIONS: Our findings indicate that PRAME plays a tumor-promoting role in triple negative breast cancer by increasing cancer cell motility through EMT-gene reprogramming. Therefore, PRAME could serve as a prognostic biomarker and/or therapeutic target in TNBC.
  
  
  
  
**TCF4**
-
10
of
10
  

### Dimerization of p15RS mediated by a leucine zipper-like motif is critical for its inhibitory role on Wnt signaling.

10. J Biol Chem. 2018 May 18;293(20):7618-7628. doi: 10.1074/jbc.RA118.001969 Epub 2018 Apr 4.
  
  
Fan X(1)(2), Zhao J(1), Ren F(1), Wang Y(1), Feng Y(1), Ding L(1), Zhao L(3), Shang Y(3), Li J(4), N ...
  
  
Author information: (1)From the State Key Laboratory of Membrane Biology, School of Medicine and. (2 ...
  
  
We previously demonstrated that p15RS, a newly discovered tumor suppressor, inhibits Wnt/β-catenin signaling by interrupting the formation of β-catenin·TCF4 complex. However, it remains unclear how p15RS helps exert such an inhibitory effect on Wnt signaling based on its molecular structure. In this study, we reported that dimerization of p15RS is required for its inhibition on the transcription regulation of Wnt-targeted genes. We found that p15RS forms a dimer through a highly conserved leucine zipper-like motif in the coiled-coil terminus domain. In particular, residues Leu-248 and Leu-255 were identified as being responsible for p15RS dimerization, as mutation of these two leucines into prolines disrupted the homodimer formation of p15RS and weakened its suppression of Wnt signaling. Functional studies further confirmed that mutations of p15RS at these residues results in diminishment of its inhibition on cell proliferation and tumor formation. We therefore concluded that dimerization of p15RS governed by the leucine zipper-like motif is critical for its inhibition of Wnt/β-catenin signaling and tumorigenesis.
  
  
  
  
  

---

  

# TNFAIP3

**Signature gene in:** Tregs
  
**Differentially expressed in:** Tregs
  
  
**TNFAIP3**
-
1
of
10
  

### The Role of A20 in Cancer: Friend or Foe?

1. Cells. 2025 Apr 4;14(7):544. doi: 10.3390/cells14070544
  
  
Lee J(1), Cheong H(1).
  
  
Author information: (1)Division of Cancer Biology, Research Institute, National Cancer Center, Goyan ...
  
  
A20 is a ubiquitin-editing enzyme that has emerged as a key regulator of inflammatory signaling with paradoxical roles in cancer. Acting as both an oncogene and a tumor suppressor gene depending on the cellular context, A20 modulates important cell pathways, such as NF-κB signaling and autophagy. In this review, we summarize the dual roles of A20 in tumorigenesis, highlighting its ability to promote tumor progression in cancers, such as breast and melanoma, while functioning as a tumor suppressor in lymphomas and hepatocellular carcinoma. We discuss the interplay of A20 with autophagy, a process that is important for maintaining cellular homeostasis and influencing tumor dynamics. By integrating recent findings, we provide insight into how dysregulation of A20 and its associated pathways can either suppress or drive cancer development, which may lead to improved therapeutic intervention.
  
  
  
  
**TNFAIP3**
-
2
of
10
  

### ZSH-2208: A novel retinoid with potent anti-tumour effects on ESCC stem cells via RARγ-TNFAIP3 axis.

2. Clin Transl Med. 2025 Jan;15(1):e70148. doi: 10.1002/ctm2.70148
  
  
Chen R(1), Huang X(1), Hou J(1), Ni J(1), Zhao W(1), Li Q(2)(3), Jiao H(4), Cao X(1).
  
  
Author information: (1)Institute of Clinical Science, Zhongshan Hospital, Fudan University Shanghai ...
  
  
BACKGROUD: Oesophageal cancer ranks among the most prevalent malignant tumours globally, primarily consisting of oesophageal squamous cell carcinoma (ESCC). Cancer stem cells (CSCs) accelerate the progression ESCC via their strong self-renewal and tumourigenic capabilities, presenting significant clinical challenges due to increased risks of recurrence and drug resistance. METHODS: Our previous study has reported WYC-209, which is capable of inducing apoptosis of CSCs in melanoma and hepatoma, but is ineffective against ESCC. Additionally, clinical studies in ESCC still lack drug candidates that effectively target CSCs. Therefore, our team developed a series of novel retinoids that target retinoic acid receptors (RARs), with enhanced potency, broader efficacy and minimised toxic side effects against CSCs. Following iterative optimisation and pharmacological validation, ZSH-2208 was identified as the most promising candidate for effectively targeting ESCC tumour-repopulating cells (TRCs). Mechanistic exploration revealed that ZSH-2208 inhibits the growth of ESCC-TRCs through modulation of the RARγ-TNFAIP3 axis. The clinical significance of the key molecule TNFAIP3 in ESCC has also been demonstrated. RESULTS: This study introduces ZSH-2208, a novel retinoid specifically targeting ESCC-TRCs, which holds significant potential for clinical application in ESCC. KEY POINTS: The ESCC-TRCs replicates the characteristics of ESCC stem cells, which are inhibited by ZSH-2208. In vivo and in vitro experiments demonstrated that ZSH-2208, a novel RA analogue, effectively inhibits the growth of ESCC-TRCs through the RARγ-TNFAIP3 axis. Low levels of TNFIP3 protein may be associated with improved survival probability in ESCC patients.
  
  
  
  
**TNFAIP3**
-
3
of
10
  

### Somatic mutations in tumor-infiltrating lymphocytes impact on antitumor immunity.

3. Proc Natl Acad Sci U S A. 2024 Aug 27;121(35):e2320189121. doi: 10.1073/pnas.2320189121 Epub 2024 Aug 21.
  
  
Mukohara F(#)(1)(2), Iwata K(#)(1)(2), Ishino T(1)(3), Inozume T(4)(5)(6), Nagasaki J(1), Ueda Y(1), ...
  
  
Author information: (1)Department of Tumor Microenvironment, Faculty of Medicine, Dentistry and Phar ...
  
  
Immune checkpoint inhibitors (ICIs) exert clinical efficacy against various types of cancers by reinvigorating exhausted CD8+ T cells that can expand and directly attack cancer cells (cancer-specific T cells) among tumor-infiltrating lymphocytes (TILs). Although some reports have identified somatic mutations in TILs, their effect on antitumor immunity remains unclear. In this study, we successfully established 18 cancer-specific T cell clones, which have an exhaustion phenotype, from the TILs of four patients with melanoma. We conducted whole-genome sequencing for these T cell clones and identified various somatic mutations in them with high clonality. Among the somatic mutations, an SH2D2A loss-of-function frameshift mutation and TNFAIP3 deletion could activate T cell effector functions in vitro. Furthermore, we generated CD8+ T cell-specific TNFAIP3 knockout mice and showed that TNFAIP3 function loss in CD8+ T cell increased antitumor immunity, leading to remarkable response to PD-1 blockade in vivo. In addition, we analyzed bulk CD3+ T cells from TILs in additional 12 patients and identified an SH2D2A mutation in one patient through amplicon sequencing. These findings suggest that somatic mutations in TILs can affect antitumor immunity and suggest unique biomarkers and therapeutic targets.
  
  
  
  
**TNFAIP3**
-
4
of
10
  

### Gene Expression Profile Analysis of Human Epidermal Keratinocytes Expressing Human Papillomavirus Type 8 E7.

4. Pathol Oncol Res. 2022 May 18;28:1610176. doi: 10.3389/pore.2022.1610176 eCollection 2022.
  
  
Chen X(1), Li M(1), Tang Y(1)(2), Liang Q(1), Hua C(1), He H(3), Song Y(1), Cheng H(1).
  
  
Author information: (1)Department of Dermatology and Venereology, Zhejiang University School of Medi ...
  
  
Background: Human papillomavirus type 8 (HPV8) has been implicated in the progress of non-melanoma skin cancers and their precursor lesions. The HPV8 E7 oncoprotein plays a key role in the tumorigenesis of HPV-associated cutaneous tumors. However, the exact role of HPV8 E7 in human epidermal carcinogenesis has not been fully elucidated. Methods: To investigate the potential carcinogenic effects of HPV8 E7 on epithelial cells, we used RNA-sequencing technology to analyze the gene expression profile of HPV8 E7-overexpressed normal human epidermal keratinocytes (NHEKs). Results: RNA-sequencing revealed 831 differentially expressed genes (DEGs) between HPV8 E7-expressing NHEKs and control cells, among which, 631 genes were significantly upregulated, and 200 were downregulated. Gene ontology annotation enrichment analysis showed that HPV8 E7 mainly affected the expression of genes associated with protein heterodimerization activity, DNA binding, nucleosomes, and nucleosome assembly. Kyoto Encyclopedia of Genes and Genomes pathway enrichment analysis revealed that overexpression of HPV8 E7 affected the expression of gene clusters associated with viral carcinogenesis and transcriptional misregulation in cancer and necroptosis signaling pathways that reportedly play crucial roles in HPV infection promotion and cancer progression. We also found the DEGs, such as HKDC1 and TNFAIP3, were associated with epigenetic modifications, immune regulation, and metabolic pathways. Conclusion: Our results demonstrate that the pro-carcinogenic effect of HPV8 expression in epithelial cells may be attributed to the regulatory effect of oncogene E7 on gene expression associated with epigenetic modifications and immune and metabolic status-associated gene expression. Although our data are based on an in vitro experiment, it provides the theoretical evidence that the development of squamous cell carcinoma can be caused by HPV.
  
  
  
  
**TNFAIP3**
-
5
of
10
  

### Down-regulation of A20 promotes immune escape of lung adenocarcinomas.

5. Sci Transl Med. 2021 Jul 7;13(601):eabc3911. doi: 10.1126/scitranslmed.abc3911
  
  
Breitenecker K(1)(2)(3), Homolya M(1), Luca AC(1), Lang V(1), Trenk C(1), Petroczi G(1), Mohrherr J( ...
  
  
Author information: (1)Institute of Pharmacology, Center of Physiology and Pharmacology, Medical Uni ...
  
  
Inflammation is a well-known driver of lung tumorigenesis. One strategy by which tumor cells escape tight homeostatic control is by decreasing the expression of the potent anti-inflammatory protein tumor necrosis factor alpha-induced protein 3 (TNFAIP3), also known as A20. We observed that tumor cell intrinsic loss of A20 markedly enhanced lung tumorigenesis and was associated with reduced CD8+ T cell-mediated immune surveillance in patients with lung cancer and in mouse models. In mice, we observed that this effect was completely dependent on increased cellular sensitivity to interferon-γ (IFN-γ) signaling by aberrant activation of TANK-binding kinase 1 (TBK1) and increased downstream expression and activation of signal transducer and activator of transcription 1 (STAT1). Interrupting this autocrine feed forward loop by knocking out IFN-α/β receptor completely restored infiltration of cytotoxic T cells and rescued loss of A20 depending tumorigenesis. Downstream of STAT1, programmed death ligand 1 (PD-L1) was highly expressed in A20 knockout lung tumors. Accordingly, immune checkpoint blockade (ICB) treatment was highly efficient in mice harboring A20-deficient lung tumors. Furthermore, an A20 loss-of-function gene expression signature positively correlated with survival of melanoma patients treated with anti-programmed cell death protein 1. Together, we have identified A20 as a master immune checkpoint regulating the TBK1-STAT1-PD-L1 axis that may be exploited to improve ICB therapy in patients with lung adenocarcinoma.
  
  
  
  
**TNFAIP3**
-
6
of
10
  

### Casp8 acts through A20 to inhibit PD-L1 expression: The mechanism and its implication in immunotherapy.

6. Cancer Sci. 2021 Jul;112(7):2664-2678. doi: 10.1111/cas.14932 Epub 2021 May 20.
  
  
Zou J(1)(2), Xia H(1)(2), Zhang C(2), Xu H(1)(2), Tang Q(2), Zhu G(1)(2), Li J(1), Bi F(1)(2).
  
  
Author information: (1)Department of Abdominal Oncology, Cancer Center, West China Hospital, Sichuan ...
  
  
Immunotherapy targeting the PD-L1/PD-1 pathway is a novel type of clinical cancer treatment, but only small subsets of patients can benefit from it because of multiple factors. PD-L1/PD-1 expression is a biomarker for predicting the efficacy of anti-PD-L1/PD-1 therapy, which highlights the importance of understanding the regulatory mechanisms of PD-L1 expression in cancer cells. Casp8 is an apical caspase protease involved in mediating cell apoptosis, but it also has multiple nonapoptotic functions. Casp8 mutations are associated with increased risks of cancer, and low expression of Casp8 is closely connected with poor prognosis in patients with cancer. In addition, mutations of Casp8 in lymphocytes also lead to human immunodeficiency, thereby causing dysfunction of the innate immune system, but the roles of Casp8 in antitumor immunity remain unclear. Here, we found that knocking down Casp8 in mouse melanoma cells promoted tumor progression in an immune system-dependent manner. Mechanistically, Casp8 induced PD-L1 degradation by upregulating TNFAIP3 (A20) expression, a ubiquitin-editing enzyme that results in PD-L1 ubiquitination. In addition, compared with Casp8fl/fl mice, mice with conditional deletion of Casp8 in natural killer (NK) cells (Ncr1iCre/+ Casp8fl/fl mice) showed a decreased frequency of IFN-γ+ and CD107a+ NK cells but an increased frequency of PD-1+ and CTLA-4+ NK cells. Melanoma cells with Casp8 knocked down exhibited sensitivity to anti-PD-1 or anti-CTLA-4 antibody treatments, particularly in Ncr1iCre/+Casp8fl/fl mice. Together, the results indicate that Casp8 induces PD-L1 degradation by upregulating A20 expression and that decreased Casp8 expression is a potential biomarker for predicting the sensitivity to anti-PD-L1/PD-1 immunotherapy.
  
  
  
  
**TNFAIP3**
-
7
of
10
  

### The dual roles of A20 in cancer.

7. Cancer Lett. 2021 Jul 28;511:26-35. doi: 10.1016/j.canlet.2021.04.017 Epub 2021 Apr 29.
  
  
Shi Y(1), Wang X(2), Wang J(2), Wang X(2), Zhou H(3), Zhang L(2).
  
  
Author information: (1)Department of Immunology and Shandong Key Laboratory of Infection and Immunit ...
  
  
A20 is a prototypical anti-inflammatory molecule that is linked to multiple human diseases, including cancers. The role of A20 as a tumor suppressor was first discovered in B cell lymphomas. Subsequent studies revealed the dual roles of A20 in solid cancers. This review focuses on the roles of A20 in different cancer types to demonstrate that the effects of A20 are cancer type-dependent. A20 plays antitumor roles in colorectal carcinomas and hepatocellular carcinomas, whereas A20 acts as an oncogene in breast cancers, gastric cancers and melanomas. Moreover, the roles of A20 in the setting of glioma therapy are context-dependent. The action mechanisms of A20 in different types of cancer are summarized. Additionally, the role of A20 in antitumor immunity is discussed. Furthermore, some open questions in this rapidly advancing field are proposed. Exploration of the actions and molecular mechanisms of A20 in cancer paves the way for the application of A20-targeting approaches in future cancer therapy.
  
  
  
  
**TNFAIP3**
-
8
of
10
  

### A20 regulates the therapeutic effect of anti-PD-1 immunotherapy in melanoma.

8. J Immunother Cancer. 2020 Dec;8(2):e001866. doi: 10.1136/jitc-2020-001866
  
  
Guo W(#)(1), Ma J(#)(2), Guo S(#)(2), Wang H(#)(2), Wang S(#)(2)(3), Shi Q(2), Liu L(2), Zhao T(2), ...
  
  
Author information: (1)Department of Dermatology, Xijing Hospital, Fourth Military Medical Universit ...
  
  
BACKGROUND: The therapeutic effect of immune checkpoint blockers, especially the neutralizing antibodies of programmed cell death (PD-1) and its ligand programmed death ligand 1 (PD-L1), has been well verified in melanoma. Nevertheless, the dissatisfactory response rate and the occurrence of resistance significantly hinder the treatment effect. Inflammation-related molecules like A20 are greatly implicated in cancer immune response, but the role of tumorous A20 in antitumor immunity and immunotherapy efficacy remains elusive. METHODS: The association between tumorous A20 expression and the effect of anti-PD-1 immunotherapy was determined by immunoblotting, immunofluorescence staining and flow cytometry analysis of primary tumor specimens from melanoma patients. Preclinical mouse model, in vitro coculture system, immunohistochemical staining and flow cytometry analysis were employed to investigate the role of A20 in regulating the effect of anti-PD-1 immunotherapy. Bioinformatics, mass spectrum analysis and a set of biochemical analyzes were used to figure out the underlying mechanism. RESULTS: We first discovered that upregulated A20 was associated with impaired antitumor capacity of CD8+T cells and poor response to anti-PD-1 immunotherapy in melanoma patients. Subsequent functional studies in preclinical mouse model and in vitro coculture system proved that targeting tumorous A20 prominently improved the effect of immunotherapy through the invigoration of infiltrating CD8+T cells via the regulation of PD-L1. Mechanistically, A20 facilitated the ubiquitination and degradation of prohibitin to potentiate STAT3 activation and PD-L1 expression. Moreover, tumorous A20 expression was highly associated with the ratio of Ki-67 percentage in circulating PD-1+CD8+T cells to tumor burden. CONCLUSIONS: Together, our findings uncover a novel crosstalk between inflammatory molecules and antitumor immunity in melanoma, and highlight that A20 can be exploited as a promising target to bring clinical benefit to melanomas refractory to immune checkpoint blockade.
  
  
  
  
**TNFAIP3**
-
9
of
10
  

### A20 promotes melanoma progression via the activation of Akt pathway.

9. Cell Death Dis. 2020 Sep 23;11(9):794. doi: 10.1038/s41419-020-03001-y
  
  
Ma J(#)(1), Wang H(#)(1), Guo S(#)(1), Yi X(1), Zhao T(1), Liu Y(1), Shi Q(1), Gao T(1), Li C(2), Gu ...
  
  
Author information: (1)Department of Dermatology, Xijing Hospital, Fourth Military Medical Universit ...
  
  
Melanoma is the most life-threatening skin cancer with increasing incidence around the world. Although recent advances in targeted therapy and immunotherapy have brought revolutionary progress of the treatment outcome, the survival of patients with advanced melanoma remains unoptimistic, and metastatic melanoma is still an incurable disease. Therefore, to further understand the mechanism underlying melanoma pathogenesis could be helpful for developing novel therapeutic strategy. A20 is a crucial ubiquitin-editing enzyme implicated immunity regulation, inflammatory responses and cancer pathogenesis. Herein, we report that A20 played an oncogenic role in melanoma. We first found that the expression of A20 was significantly up-regulated in melanoma cell lines. Then, we showed that knockdown of A20 suppressed melanoma cell proliferation in vitro and melanoma growth in vivo through the regulation of cell-cycle progression. Moreover, A20 could potentiate the invasive and migratory capacities of melanoma cell in vitro and melanoma metastasis in vivo by promoting epithelial-mesenchymal transition (EMT). Mechanistically, we found that Akt activation mediated the oncogenic effect of A20 on melanoma development, with the involvement of glycolysis. What's more, the up-regulation of A20 conferred the acquired resistance to Vemurafenib in BRAF-mutant melanoma. Taken together, we demonstrated that up-regulated A20 promoted melanoma progression via the activation of Akt pathway, and that A20 could be exploited as a potential therapeutic target for melanoma treatment.
  
  
  
  
**TNFAIP3**
-
10
of
10
  

### IL-17RC is critically required to maintain baseline A20 production to repress JNK isoform-dependent tumor-specific proliferation.

10. Oncotarget. 2017 Jun 27;8(26):43153-43168. doi: 10.18632/oncotarget.17820
  
  
Yan C(1)(2), Lei Y(1)(2), Lin TJ(2)(3)(4), Hoskin DW(2)(5), Ma A(6), Wang J(1)(2)(3)(4).
  
  
Author information: (1)Canadian Center for Vaccinology, Halifax, Nova Scotia, Canada. (2)Department ...
  
  
The IL-17/IL-17R axis has controversial roles in cancer, which may be explained by tumor-specific results. Here, we describe a novel molecular mechanism underlying IL-17RC-controlled tumor-specific proliferation. Triggered by IL-17RC knockdown (KD), B16 melanoma and 4T1 carcinoma cells inversely altered homeostatic tumor proliferation and tumor growth in vitro and in vivo. In contrast to the existing dogma that IL-17RC-dependent signaling activates the JNK pathway, IL-17RC KD in both tumor cell lines caused aberrant expression and activation of different JNK isoforms along with markedly diminished levels of the ubiquitin-editing enzyme A20. We demonstrated that differential up-regulation of JNK1 and JNK2 in the two tumor cell lines was responsible for the reciprocal regulation of c-Jun activity and tumor-specific proliferation. Furthermore, we showed that A20 reconstitution of IL-17RCKD clones with expression of full-length A20, but not a truncation-mutant, reversed aberrant JNK1/JNK2 activities and tumor-specific proliferation. Collectively, our study reveals a critical role of IL-17RC in maintaining baseline A20 production and a novel role of the IL-17RC-A20 axis in controlling JNK isoform-dependent tumor-specific homeostatic proliferation.
  
  
  
  
  

---

  

# TNFRSF21

**Signature gene in:** Stromal cells
  
**Differentially expressed in:** Stromal cells
  
  
**TNFRSF21**
-
1
of
5
  

### Comprehensive Analysis of Necroptosis Landscape in Skin Cutaneous Melanoma for Appealing its Implications in Prognosis Estimation and Microenvironment Status.

1. J Pers Med. 2023 Jan 29;13(2):245. doi: 10.3390/jpm13020245
  
  
Cao X(1), He J(2), Chen A(2), Ran J(3), Li J(2), Chen D(4), Zhang H(1).
  
  
Author information: (1)Department of Plastic and Burn Surgery, The First Affiliated Hospital of Chon ...
  
  
PURPOSE: Due to poor prognosis and immunotherapy failure of skin cutaneous melanoma (SKCM), this study sought to find necroptosis-related biomarkers to predict prognosis and improve the situation with predicted immunotherapy drugs. EXPERIMENTAL DESIGN: The Cancer Genome Atlas (TCGA) and The Genotype-Tissue Expression Program (GTEx) database were utilized to recognize the differential necroptosis-related genes (NRGs). Univariate Cox (uni-Cox) and least absolute shrinkage and selection operator (LASSO) Cox analysis were utilized for prognostic signature establishment. The signature was verified in the internal cohort. To assess the signature's prediction performance, the area under the curve (AUC) of receiver operating characteristic (ROC) curves, Kaplan-Meier (K-M) analyses, multivariate Cox (multi-Cox) regression, nomogram, and calibration curves were performed. The molecular and immunological aspects were also reviewed using single-sample gene set enrichment analysis (ssGSEA). Cluster analysis was performed to identify the different types of SKCM. Finally, the expression of the signature gene was verified by immunohistochemical staining. RESULTS: On basis of the 67 NRGs, 4 necroptosis-related genes (FASLG, PLK1, EGFR, and TNFRSF21) were constructed to predict SKCM prognosis. The area's 1-, 3-, and 5-year OS under the AUC curve was 0.673, 0.649, and 0.677, respectively. High-risk individuals had significantly lower overall survival (OS) compared to low-risk patients. Immunological status and tumor cell infiltration in high-risk groups were significantly lower, indicating an immune system that was suppressed. In addition, hot and cold tumors could be obtained by cluster analysis, which is helpful for accurate treatment. Cluster 1 was considered a hot tumor and more susceptible to immunotherapy. Immunohistochemical results were consistent with positive and negative regulation of coefficients in signature. CONCLUSION: The results of this finding supported that NRGs could predict prognosis and help make a distinction between the cold and hot tumors for improving personalized therapy for SKCM.
  
  
  
  
**TNFRSF21**
-
2
of
5
  

### Identification of an endoplasmic reticulum stress-associated gene signature to predict the immune status and prognosis of cutaneous melanoma.

2. Medicine (Baltimore). 2022 Sep 9;101(36):e30280. doi: 10.1097/MD.0000000000030280
  
  
Chen R(1), Niu L(2), Wu L(1), He Y(1), Liu G(1), Hong K(3).
  
  
Author information: (1)Department of Hand Plastic Surgery, The First People's Hospital of Linping Di ...
  
  
Besides protecting normal cells from various internal and external perturbations, endoplasmic reticulum (ER) stress is also directly related to the pathogenesis of cutaneous melanoma (CM). However, due to the lack of specific molecular biomarkers, ER stress has not been considered a novel treatment target for CM. Here, we identified ER stress-related genes involved in the prognosis of CM patients and constructed an effective model for the prognostic prediction of these patients. First, gene expression data of CM and normal skin tissues from the Genotype-Tissue Expression (GTEx) and The Cancer Genome Atlas (TCGA) databases were retrieved to identify differentially expressed ER stress-related genes in CM. Meanwhile, an independent cohort obtained from the Gene Expression Omnibus (GEO) database was used for validation. The ER stress genes (ZBP1, DIABLO, GNLY, FASLG, AURKA, TNFRSF21, and CD40LG) that were associated with CM prognosis were incorporated into our prognostic model. The functional analyses indicated that the prognostic model was correlated with patient survival, gender, and cancer growth. Multivariate and univariate Cox regressions revealed that the constructed model could serve as an independent prognostic factor for CM patients. The pathway enrichment analysis showed that the risk model was enriched in different immunity and cancer progression-associated pathways. Moreover, the signature model was significantly connected with the immune subtypes, infiltration of immune cells, immune microenvironment, as well as tumor stem cells. The gene function analysis revealed that 7 ER stress genes were differentially expressed in CM patients and were significantly associated with prognosis and several antitumor drugs. Overall, our current model presented predictive value for the prognosis of CM patients and can be further used in the development of novel therapeutic strategies for CM.
  
  
  
  
**TNFRSF21**
-
3
of
5
  

### A Novel Necroptosis-Related Gene Signature in Skin Cutaneous Melanoma Prognosis and Tumor Microenvironment.

3. Front Genet. 2022 Jul 11;13:917007. doi: 10.3389/fgene.2022.917007 eCollection 2022.
  
  
Song B(1), Wu P(2), Liang Z(1), Wang J(1), Zheng Y(3), Wang Y(4), Chi H(5), Li Z(1), Song Y(1), Yin ...
  
  
Author information: (1)Department of Plastic Surgery, Xijing Hospital, Fourth Military Medical Unive ...
  
  
Background: Necroptosis has been identified recently as a newly recognized programmed cell death that has an impact on tumor progression and prognosis, although the necroptosis-related gene (NRGs) potential prognostic value in skin cutaneous melanoma (SKCM) has not been identified. The aim of this study was to construct a prognostic model of SKCM through NRGs in order to help SKCM patients obtain precise clinical treatment strategies. Methods: RNA sequencing data collected from The Cancer Genome Atlas (TCGA) were used to identify differentially expressed and prognostic NRGs in SKCM. Depending on 10 NRGs via the univariate Cox regression analysis usage and LASSO algorithm, the prognostic risk model had been built. It was further validated by the Gene Expression Omnibus (GEO) database. The prognostic model performance had been assessed using receiver operating characteristic (ROC) curves. We evaluated the predictive power of the prognostic model for tumor microenvironment (TME) and immunotherapy response. Results: We constructed a prognostic model based on 10 NRGs (FASLG, TLR3, ZBP1, TNFRSF1B, USP22, PLK1, GATA3, EGFR, TARDBP, and TNFRSF21) and classified patients into two high- and low-risk groups based on risk scores. The risk score was considered a predictive factor in the two risk groups regarding the Cox regression analysis. A predictive nomogram had been built for providing a more beneficial prognostic indicator for the clinic. Functional enrichment analysis showed significant enrichment of immune-related signaling pathways, a higher degree of immune cell infiltration in the low-risk group than in the high-risk group, a negative correlation between risk scores and most immune checkpoint inhibitors (ICIs), anticancer immunity steps, and a more sensitive response to immunotherapy in the low-risk group. Conclusions: This risk score signature could be applied to assess the prognosis and classify low- and high-risk SKCM patients and help make the immunotherapeutic strategy decision.
  
  
  
  
**TNFRSF21**
-
4
of
5
  

### Multi-Arm PEG/Peptidomimetic Conjugate Inhibitors of DR6/APP Interaction Block Hematogenous Tumor Cell Extravasation.

4. Adv Sci (Weinh). 2021 Jun;8(11):e2003558. doi: 10.1002/advs.202003558 Epub 2021 Mar 18.
  
  
Wang L(1), Shen Q(1), Liao H(2), Fu H(1), Wang Q(3), Yu J(1), Zhang W(1), Chen C(1), Dong Y(1), Yang ...
  
  
Author information: (1)State Key Laboratory of Oncogenes and Related Genes, Shanghai Cancer Institut ...
  
  
The binding of amyloid precursor protein (APP) expressed on tumor cells to death receptor 6 (DR6) could initiate the necroptosis pathway, which leads to necroptotic cell death of vascular endothelial cells (ECs) and results in tumor cells (TCs) extravasation and metastasis. This study reports the first inhibitor of DR6/APP interaction as a novel class of anti-hematogenous metastatic agent. By rationally utilizing three combined strategies including selection based on phage display library, d-retro-inverso modification, and multiple conjugation of screened peptidomimetic with 4-arm PEG, the polymer-peptidomimetic conjugate PEG-tAHP-DRI (tetra-(D-retro-inverso isomer of AHP-12) substitued 4-arm PEG5k ) is obtained as the most promising agent with the strongest binding potency (KD  = 51.12 × 10-9  m) and excellent pharmacokinetic properties. Importantly, PEG-tAHP-DRI provides efficient protection against TC-induced ECs necroptosis both in vitro and in vivo. Moreover, this ligand exhibits prominent anti-hematogenous metastatic activity in serval different metastatic mouse models (B16F10, 4T1, CT26, and spontaneous lung metastasis of 4T1 orthotopic tumor model) and displays no apparent detrimental effects in preliminary safety evaluation. Collectively, this study demonstrates the feasibility of exploiting DR6/APP interaction to regulate hematogenous tumor cells transendothelial migration and provides PEG-tAHP-DRI as a novel and promising inhibitor of DR6/APP interaction for developments of anti-hematogenous metastatic therapies.
  
  
  
  
**TNFRSF21**
-
5
of
5
  

### Epigenetic marks responsible for cadmium-induced melanoma cell overgrowth.

5. Toxicol In Vitro. 2015 Feb;29(1):242-50. doi: 10.1016/j.tiv.2014.10.020
  
  
Venza M(1), Visalli M, Biondo C, Oteri R, Agliano F, Morabito S, Teti D, Venza I.
  
  
Author information: (1)Department of Experimental Specialized Medical and Surgical and Odontostomato ...
  
  
Cadmium (Cd) is a human carcinogen that likely acts via epigenetic mechanisms. However, the precise role of Cd in melanoma remains to be defined. The goals of this study are to: (i) examine the effect of Cd on the proliferation rate of cutaneous and uveal melanoma cells; (ii) identify the genes affected by Cd exposure; (iii) understand whether epigenetic changes are involved in the response to Cd. The cell growth capacity increased at 48 h after Cd treatment at doses ranging from 0.5 to 10 μM. The research on the key genes regulating proliferation has shown that aberrant methylation is responsible for silencing of p16(INK4A) and caspase 8 in uveal and cutaneous melanoma cells, respectively. The methylation and expression patterns of p14(ARF), death receptors 4/5, and E-cadherin remained unmodified after Cd treatment in all the cell lines analyzed. Ectopic expression of p16(INK4A) abolished the overgrowth of uveal melanoma cells in response to Cd and the overexpression of caspase 8 drastically increased the apoptotic rate of Cd-treated cutaneous melanoma cells. In conclusion, our data suggest that hypermethylation of p16(INK4A) and caspase 8 represents the most common event linked to Cd-induced stimulation of cell growth and inhibition of cell death pathway in melanoma.
  
  
  
  
  

---

  

# UTRN

**Signature gene in:** B cells
  
**Differentially expressed in:** B cells
  
  
**UTRN**
-
1
of
3
  

### Circadian rhythm related genes identified through tumorigenesis and immune infiltration-guided strategies as predictors of prognosis, immunotherapy response, and candidate drugs in skin cutaneous malignant melanoma.

1. Front Immunol. 2025 Mar 21;16:1513750. doi: 10.3389/fimmu.2025.1513750 eCollection 2025.
  
  
Liu C(#)(1), Liu X(#)(2), Cao P(#)(3), Xin H(1), Li X(1), Zhu S(1).
  
  
Author information: (1)Center of Burns and Plastic Surgery and Dermatology, The 924th Hospital of Jo ...
  
  
BACKGROUND: Skin cutaneous malignant melanoma (SKCM) is among the most aggressive forms of skin cancer, notorious for its rapid progression and poor prognosis under late diagnosis. This study investigates the role of circadian rhythm-related genes (CRGs) in SKCM addressing a gap in understanding how CRGs affect tumor progression and patient outcomes. METHODS: An analysis of CRGs expression was conducted on SKCM samples derived from The Cancer Genome Atlas datasets(TCGA). Moreover, a correlation between various subtypes and their clinical features was identified. The study employed various bioinformatics methods, including differential expression analysis, consensus clustering, and survival analysis, to investigate the role of CRGs. The functional consequences of various CRG expression patterns were further investigated using immune infiltration analysis and gene set variation analysis (GSVA). A scoring system based on CRGs was developed to predict overall survival (OS) and treatment responses in SKCM patients. The predictive accuracy of this score system was then tested, and a nomogram was used to improve its clinical usefulness. RESULTS: Key findings from this study include significant genetic alterations in circadian rhythm-related genes (CRGs) in skin cutaneous melanoma (SKCM), such as mutations and CNVs. Two molecular subtypes with distinct clinical outcomes and immune profiles were identified. A prognostic model based on six CRGs (CMTM, TNPO1, CTBS, UTRN, HK2, and LIF) was developed and validated with TCGA and GEO datasets, showing high predictive accuracy for overall survival (OS). A high CRGs score correlated with poor OS, immune checkpoint expression, and reduced sensitivity to several chemotherapeutics, including AKT inhibitor VIII and Camptothecin. CONCLUSIONS: This work provides valuable insights into the circadian regulation of SKCM and underscores the potential of CRGs as biomarkers for prognosis and targets for therapeutic interventions. The novel molecular subtypes and CRGs prognostic scoring model introduced in this study offer significant contributions to the understanding and management of SKCM.
  
  
  
  
**UTRN**
-
2
of
3
  

### UTRN inhibits melanoma growth by suppressing p38 and JNK/c-Jun signaling pathways.

2. Cancer Cell Int. 2021 Feb 4;21(1):88. doi: 10.1186/s12935-021-01768-4
  
  
Zhou S(#)(1), Ouyang W(#)(2), Zhang X(3), Liao L(4), Pi X(1), Yang R(5), Mei B(6), Xu H(7), Xiang S( ...
  
  
Author information: (1)Department of Dermatology, The First People's Hospital of Foshan, 81 Lingnan ...
  
  
BACKGROUND: Utrophin (UTRN), as a tumor suppressor gene, is involved in various cancer progression. The function of UTRN in the melanoma process and the related molecular mechanisms are still unclear. Herein, we studied the function of UTRN in melanoma growth and the relevant molecular mechanisms. METHODS: Using the GEO database and UCSC Xena project, we compared the expression of UTRN in non-cancerous and melanoma tissues. Immunohistochemistry (IHC) staining, qRT-PCR and Western Blot (WB) were performed to evaluate UTRN expression in clinical samples. A total of 447 cases with UTRN expression data, patient characteristics and survival data were extracted from TCGA database and analyzed. After stable transduction and single cell cloning, the proliferation ability of A375 human melanoma cells was analyzed by Cell Counting Kit‑8 (CCK) and 5‑ethynyl‑2'‑deoxyuridine (EdU) incorporation assays. GSEA was performed to predict the mechanism by which UTRN regulated melanoma growth. Then WB analysis was used to assess the protein expression levels of pathway signaling in overexpression (EXP) melanoma cells. Epac activator 8-pCPT-2'-O-Me-cAMP was then used to evaluate the proliferation ability by activation of p38 and JNK/c-Jun signaling pathways. RESULTS: Data from GEO and UCSC Xena project indicated that UTRN expression was decreased in melanoma. Experiment on clinical samples further confirmed our finding. TCGA results showed that a reduced expression of UTRN in 447 melanoma samples was associated with advanced clinical characteristics (T stage, Clark level, ulceration), shorter survival time and poorer prognosis. In addition, up-regulated UTRN expression inhibited melanoma cell proliferation when compared to control group. MAPK signaling pathway was presented in both KEGG and BioCarta databases by using GSEA tool. WB results confirmed the down-regulated expression of p38, JNK1 and c-Jun in EXP group when compared to control group. Epac activator 8-pCPT-2'-O-Me-cAMP treatment could partially rescue proliferation of tumor cells. CONCLUSION: We have demonstrated that reduced UTRN predicted poorer prognosis and UTRN inhibited melanoma growth via p38 and JNK1/c-Jun pathways. Therefore, UTRN could serve as a tumor suppressor and novel prognostic biomarker for melanoma patients.
  
  
  
  
**UTRN**
-
3
of
3
  

### UTRN on chromosome 6q24 is mutated in multiple tumors.

3. Oncogene. 2007 Sep 13;26(42):6220-8. doi: 10.1038/sj.onc.1210432 Epub 2007 Mar 26.
  
  
Li Y(1), Huang J, Zhao YL, He J, Wang W, Davies KE, Nosé V, Xiao S.
  
  
Author information: (1)Department of Pathology, Brigham and Women's Hospital, Harvard Medical School ...
  
  
Though deletion of the long arm of chromosome 6 is one of the most common aberrations in tumors, its targeted gene(s) has not been convincingly identified. Using a functional screening approach, we found that UTRN (which encodes utrophin, a dystrophin-related protein) at 6q24, when expressed in an antisense orientation, induced cellular transformation, consistent with a tumor suppressor role. Northern blot analysis, semiquantitative reverse transcription-polymerase chain reaction (RT-PCR), and gene expression arrays all showed that UTRN expression was downregulated in primary tumors compared with matched normal tissues. Several UTRN neighbor genes were not affected in some tumors with UTRN downregulation, suggesting that UTRN was specifically targeted. RT-PCR, coupled with an in vitro transcription and translation assay, revealed inactivation mutations in 21/62 breast cancers, 4/20 neuroblastomas and 4/15 malignant melanomas. Most of the mutations were deletions involving one or more exons that led to the truncation of utrophin. Splicing errors were found in two cases, and nonsense mutation in one case. Overexpression of a wild-type UTRN in breast cancer cells inhibited tumor cell growth in vitro and reduced their tumor potential in nude mice. Our studies suggest that UTRN is a candidate tumor suppressor gene.
  
  
  
  
  

---

  

# ZFHX3

**Signature gene in:** Stromal cells
  
**Differentially expressed in:** Stromal cells
  
  
**ZFHX3**
-
1
of
1
  

### Interplay between Tumor Mutational Burden and Mutational Profile and Its Effect on Overall Survival: A Pilot Study of Metastatic Patients Treated with Immune Checkpoint Inhibitors.

1. Cancers (Basel). 2022 Nov 4;14(21):5433. doi: 10.3390/cancers14215433
  
  
Xavier CB(1), Lopes CDH(1), Awni BM(1), Campos EF(1), Alves JPB(2), Camargo AA(3), Guardia GDA(3), G ...
  
  
Author information: (1)Oncology Center, Hospital Sírio-Libanês, São Paulo 01308-050, Brazil. (2)Falc ...
  
  
Purpose: Solid tumors harboring tumor mutational burden (TMB) ≥10 mutations per megabase (mut/Mb) received agnostic approval for pembrolizumab. This work aims to analyze the somatic mutational profile’s influence on the outcomes of patients with TMB-high tumors treated with immune checkpoint inhibitors (ICIs). Methods: This post-hoc analysis evaluated clinical and molecular features of patients with solid tumors treated with ICIs that could be either monoclonal antibody directed against programmed cell death protein-1 or monoclonal antibody directed against programmed cell death ligand 1 (anti-PD-1/anti-PD-L1), monoclonal antibody directed against cytotoxic T lymphocyte-associated antigen (anti-CTLA-4) or a combined treatment regimen including one anti-PD-1/anti-PD-L1 and one anti-CTLA-4 (ICIs combination). We performed OS analysis for TMB thresholds of ≥10, ≥20, and <10 mut/Mb. We assessed OS according to the mutational profile for a TMB ≥ 10 mut/Mb cutoff. For genes correlated with OS at the univariate assessment, we conducted a Cox multivariate analysis adjusted by median TMB, sex, age, microsatellite instability (MSI), and histology. Results: A total of 1661 patients were investigated; 488 with a TMB ≥10 mut/Mb (29.4%). The median OS was 42 months for TMB ≥10 or 20 mut/Mb, and 15 months for TMB <10 mut/Mb (p < 0.005). Among TMB ≥10 mut/Mb patients, mutations in E2F3 or STK11 correlated with worse OS, and mutations in NTRK3, PTPRD, RNF43, TENT5C, TET1, or ZFHX3 with better OS. These associations were confirmed with univariate and multivariate analyses (p < 0.05). Melanoma histology and TMB above the median endowed patients with better OS (p < 0.05), while MSI status, age, and gender did not have a statistically significant effect on OS. Conclusion: Combining TMB and mutation profiles in key cancer genes can better qualify patients for ICI treatment and predict their OS.
  
  
  
  
  

---

  

# ZFP36L2

**Signature gene in:** CD8+ T cells
  
**Differentially expressed in:** CD8+ T cells
  
  
**ZFP36L2**
-
1
of
1
  

### Toxicogenomic and bioinformatics platforms to identify key molecular mechanisms of a curcumin-analogue DM-1 toxicity in melanoma cells.

1. Pharmacol Res. 2017 Nov;125(Pt B):178-187. doi: 10.1016/j.phrs.2017.08.018 Epub 2017 Sep 4.
  
  
Oliveira ÉA(1), Lima DS(2), Cardozo LE(2), Souza GF(3), de Souza N(1), Alves-Fernandes DK(1), Faião- ...
  
  
Author information: (1)Skin Biology Group, Clinical Chemistry and Toxicology Department, School of P ...
  
  
Melanoma is a highly invasive and metastatic cancer with high mortality rates and chemoresistance. Around 50% of melanomas are driven by activating mutations in BRAF that has led to the development of potent anti-BRAF inhibitors. However resistance to anti-BRAF therapy usually develops within a few months and consequently there is a need to identify alternative therapies that will bypass BRAF inhibitor resistance. The curcumin analogue DM-1 (sodium 4-[5-(4-hydroxy-3-methoxy-phenyl)-3-oxo-penta-1,4-dienyl]-2-methoxy-phenolate) has substantial anti-tumor activity in melanoma, but its mechanism of action remains unclear. Here we use a synthetic lethal genetic screen in Saccharomyces cerevisiae to identify 211 genes implicated in sensitivity to DM-1 toxicity. From these 211 genes, 74 had close human orthologues implicated in oxidative phosphorylation, insulin signaling and iron and RNA metabolism. Further analysis identified 7 target genes (ADK, ATP6V0B, PEMT, TOP1, ZFP36, ZFP36L1, ZFP36L2) with differential expression during melanoma progression implicated in regulation of tumor progression, cell differentiation, and epithelial-mesenchymal transition. Of these TOP1 and ADK were regulated by DM-1 in treatment-naïve and vemurafenib-resistant melanoma cells respectively. These data reveal that the anticancer effect of curcumin analogues is likely to be mediated via multiple targets and identify several genes that represent candidates for combinatorial targeting in melanoma.
  
  
  
  
  

---
