# Supplementary material for: Single-cell transcriptome analysis suggests cells of the tumor microenvironment as a major discriminator between brain and extracranial melanoma metastases
Source: Biol Direct. 2025 Sep 16;20:97. doi: 10.1186/s13062-025-00691-2 (PMC12439397; doi:10.1186/s13062-025-00691-2)
Supplement: Supplementary file 2 — Supplementary Material 2 [file 13062_2025_691_MOESM2_ESM.docx]

Supplementary Information

# Supplementary Tables

**Supplementary Table 1: Table containing aggregated expression values of 17500 genes in the 25 patient samples.** For each patient, gene expression values are means over single cells’ integrated expression values in the respective tissue. Each sheet contains data of one of the 17 cell types. Genes where a patient had less than 10 cells of respective cell type are not considered in further analyses and thus show no expression value in the table.

**Supplementary Table 2:** **Results from differential gene expression analysis comparing aggregated expression of MBM and ECM patient samples separately for each cell type.** Sheet one shows an overview of the results: cell type, biotype (protein-coding, lncRNA, both), number of significantly differentially expressed genes (FDR < 0.05), number of significant genes more/less highly expressed in MBM, summarized for all biotypes, and separated for protein-coding and lncRNAs. Sheet two compares the results to two alternative analyses: once excluding the five treated ECM samples, and once including all samples but adjusting for treatment. “whole” refers to the main analysis of the whole cohort (15 MBM, 10 ECM including 5 treated ECM patients), “naïve” refers to only untreated patients and “adjusted” are the results from modelling with “treatment” as cofactor. “num_tests” and “num_sign” represent the number of tests performed, and number of tests with significant results (FDR < 0.05), respectively. The last columns show correlations of log_2_-fold-changes and adjusted p-values between the different scenarios. The analysis failed in adjusting for treatment for many cell types resulting in missing values for their correlations. Sheet three contains gene annotation, including the cell type(s) a gene is differentially expressed and those where it is a signature gene (lowest 50 p-values). Annotation was taken from Ensembl v114. The last columns show annotations from COSMIC Cancer Gene Census v101. Subsequent sheets contain analysis results for each cell type. Note, log_2_-fold-changes (log2FC) are estimates from limma [1], which was used for differential gene expression testing, and are not directly calculated from the mean expression of the groups.

**Supplementary Table 3:** **Results from the functional enrichment analysis of genes in the different cell types.** Analysis was performed with gprofiler2 [2] based on the genes ranked by differential expression p-value comparing MBM with ECM with terms and pathways from Gene Ontology (GO) [3], KEGG [4] and WikiPathways [5]. Sheet one shows the number of significantly enriched terms/pathways (multi-testing adjusted p-value of <0.05, g_SCS correction). GO:BP and GO:MF stand for the GO categories biological process and molecular function. Sheet two shows the test results with the columns **term_size:** number of genes that are annotated to the term, **query_size**: number of genes included in the query, **intersection_size**: number of genes in input query that are annotated to corresponding term, **precision**: proportion of genes annotated to this term that are from the query (intersection_size/query_size​), **recall**: proportion of query genes annotated to this term (intersection_size/term_size​), **term_id**: unique identifier of enriched term in its source database, **term_name**: descriptive name of enriched term, **effective_size**: number of genes in background set also in the database, **source_order**: numeric order for the term within its data source. Sheet three shows, for each cell type and term source, the percentage of genes more highly expressed in MBM (Percent_up_in_MBM) out of the number of genes in the aggregated single-cell data (Number_of_genes).

**Supplementary Table 4: Log_2_-fold-changes of genes consistently differentially expressed in bulk tissue studies.** The table shows log_2_-fold-changes (MBM vs. ECM) for genes identified as differentially expressed in at least two of three previously published bulk studies [6–8]. Log_2_-fold-changes are shown for the original bulk studies and for our aggregated single-cell data (calculated as the sum of expression across cell types).

# Supplementary Figures


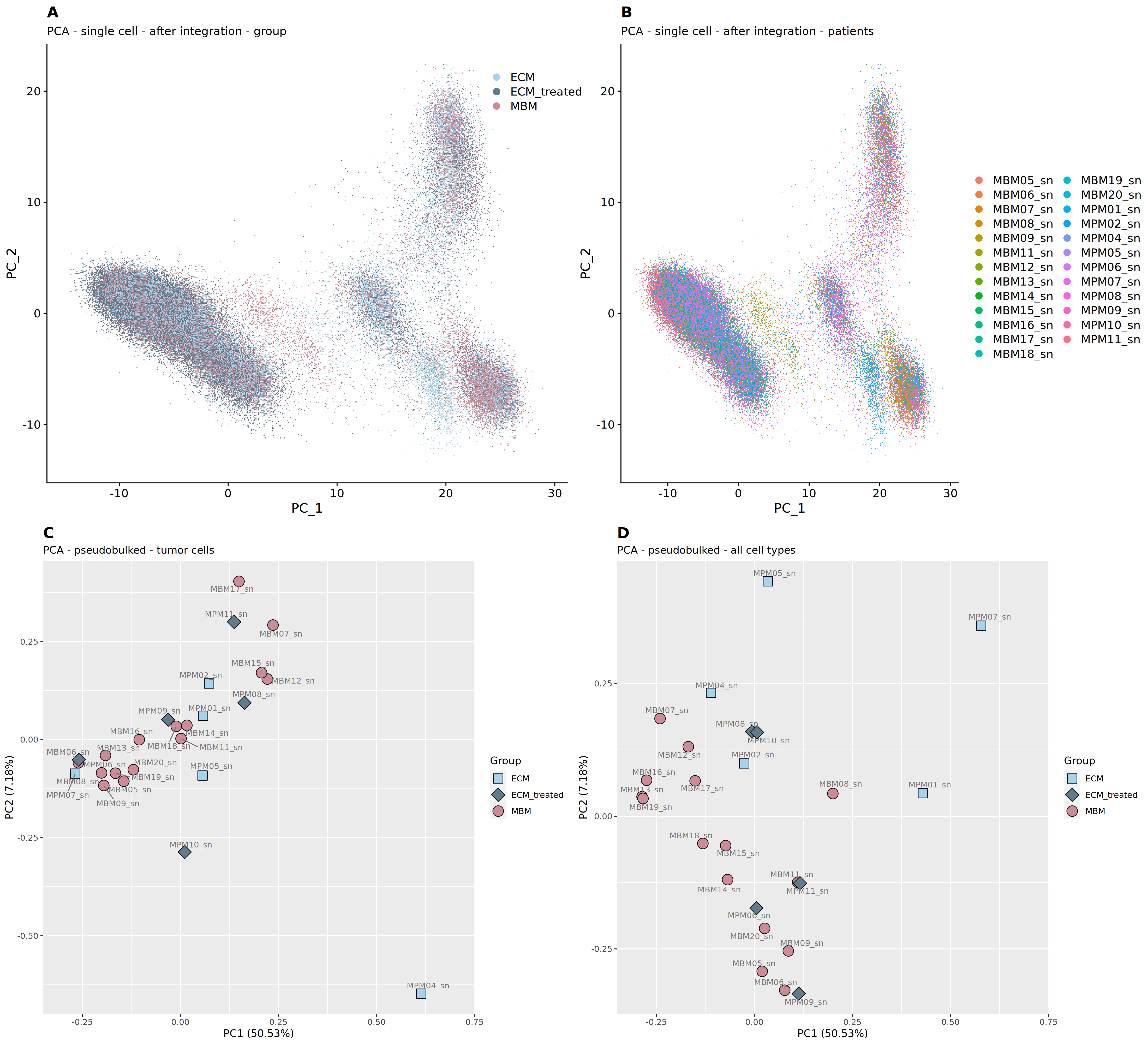


**Supplementary Figure 1: Principal component analyses of single cell and pseudobulked expression profiles.** Subfigures A and B show PCAs of the single cell data highlighting MBM, untreated and treated ECMs (A) and individual patients (B). Plots C and D show PCAs (NIPALS R package [9]) of pseudobulked expression profiles of the tumor cells (C) and the summed pseudobulked expression of all cell types (D) highlighting MBM, treatment-naïve ECM and treated ECM samples.


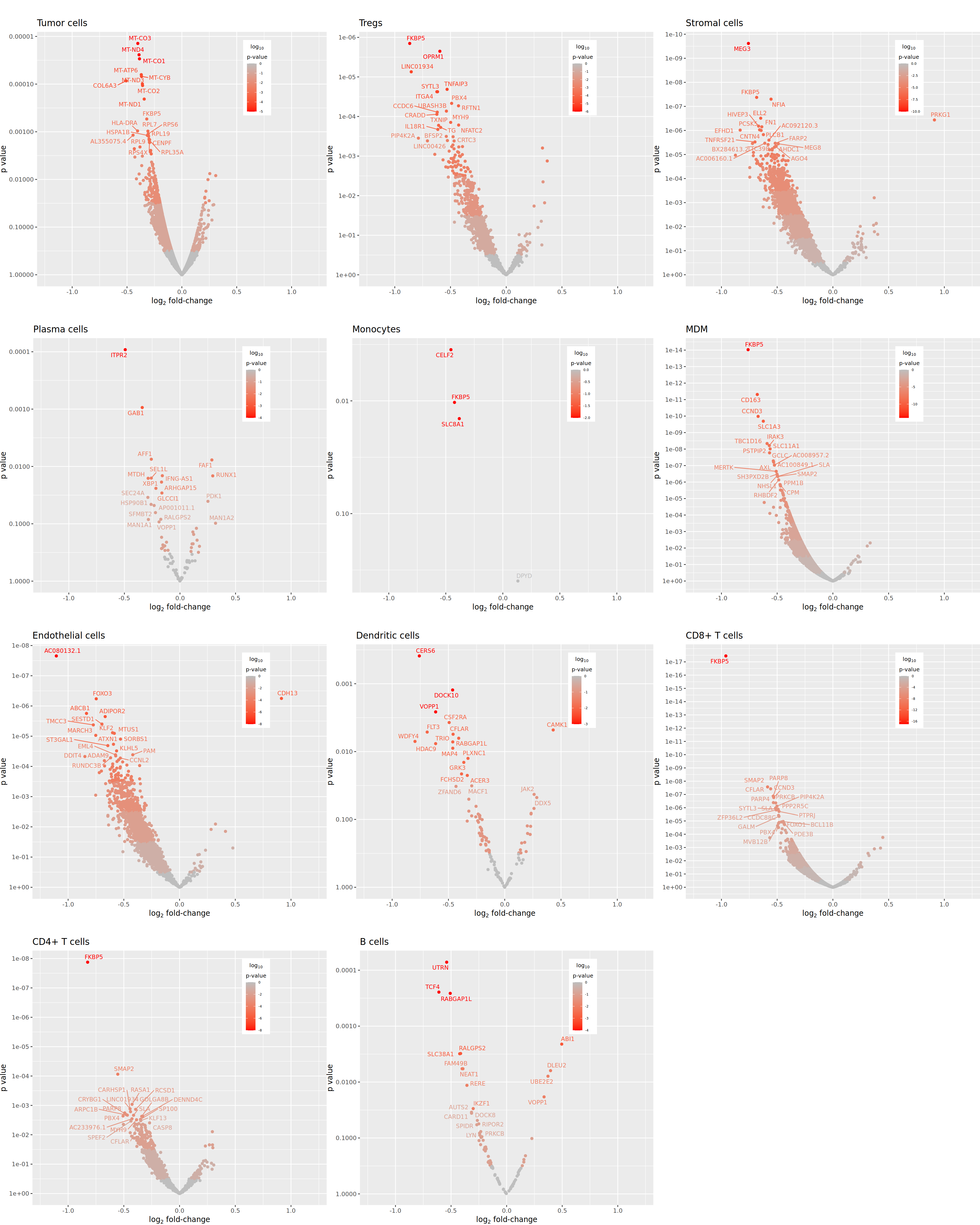


**Supplementary Figure 2: Volcano plots showing the results of the differential gene expression analysis comparing aggregated expression of the MBM and ECM patient samples across cell types.** Log_2_-fold-change and p-value of expression differences are plotted. The 20 genes with the lowest p-values are labeled for each cell type. A gray-to-red color scale indicates p-value magnitude within each plot.


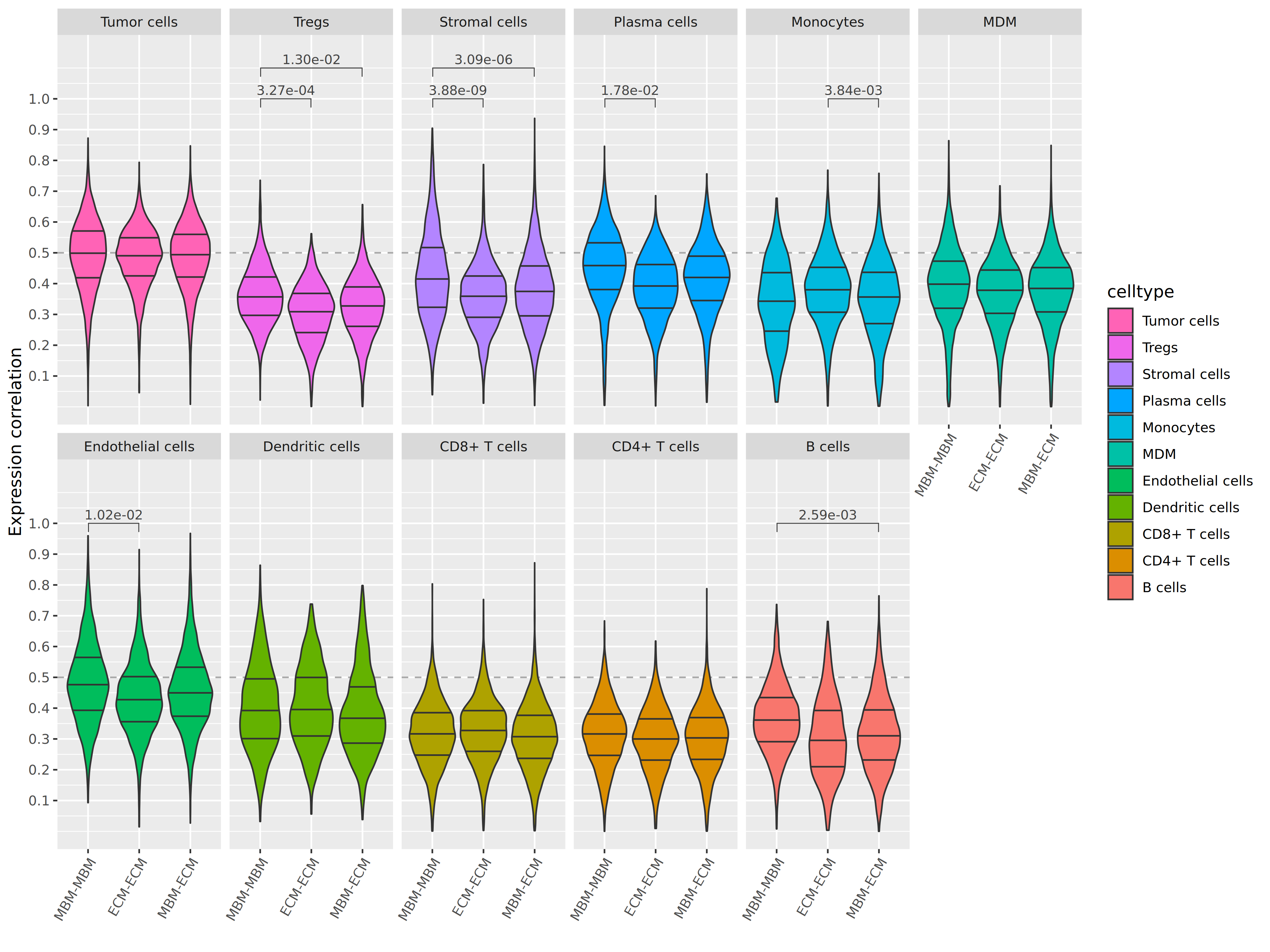


**Supplementary Figure 3A: Distribution of correlations between single cell gene expression profiles for patient groups and cell types.** We calculated gene expression correlations (Pearson) for each cell type by averaging the correlation of 100 randomly selected cell pairs from each possible sample pair. To test for significant correlation differences between groups, we used a mixed-effects model with patient pairs as a random effect and group as a fixed effect. Significant differences (FDR < 0.05) are indicated by brackets.


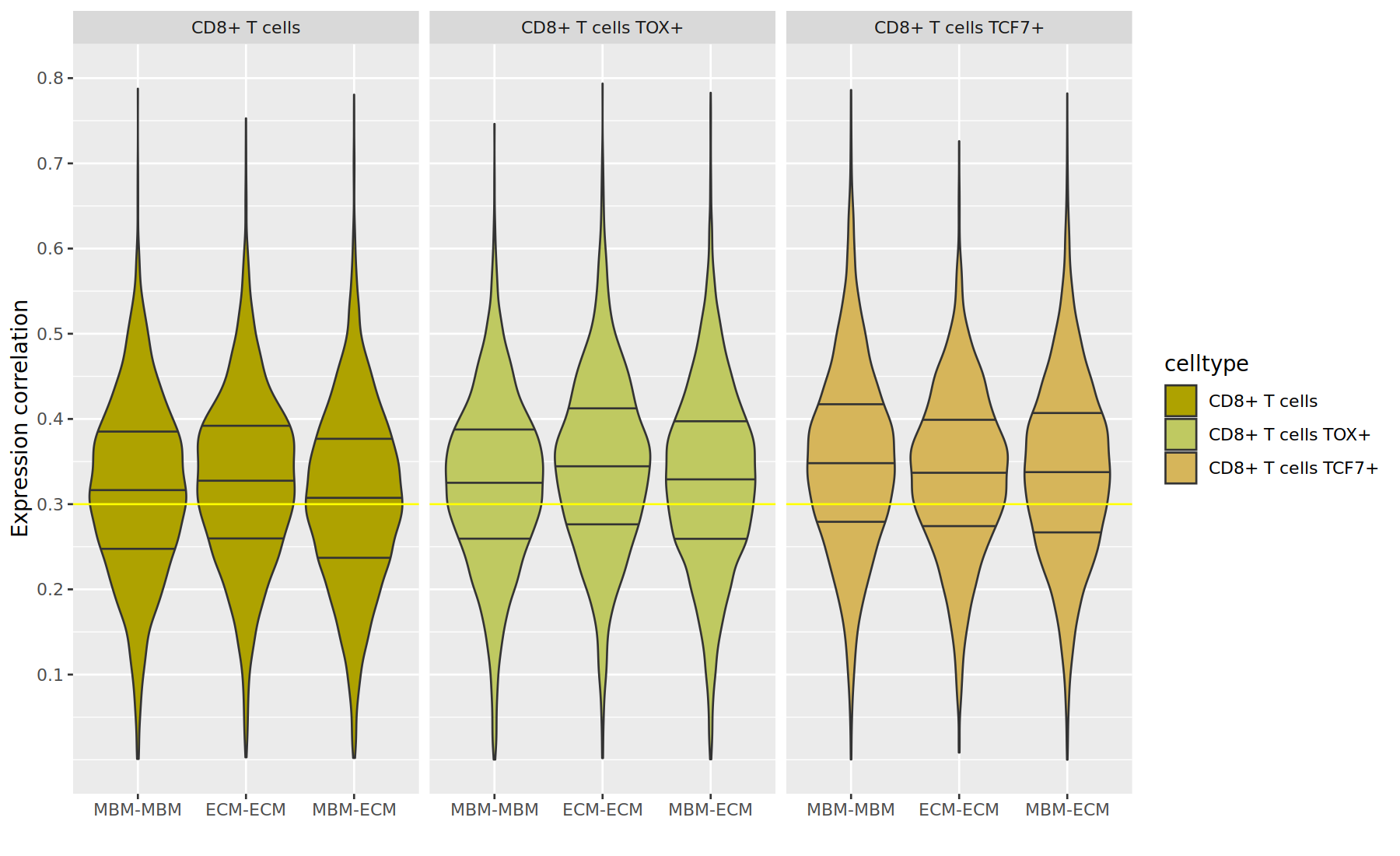


**Supplementary Figure 3B: Distribution of correlations between single cell gene expression profiles of patient groups of CD8+ T cells compared to the subtypes CD8+ TCF7+ and CD8+ TOX+.** We calculated gene expression correlations (Pearson) for each cell type by averaging the correlation of 100 randomly selected cell pairs from each possible sample pairs. The average correlation of the CD8+ T cell subtypes is slightly higher than that of CD8+ T cells (yellow line for orientation).


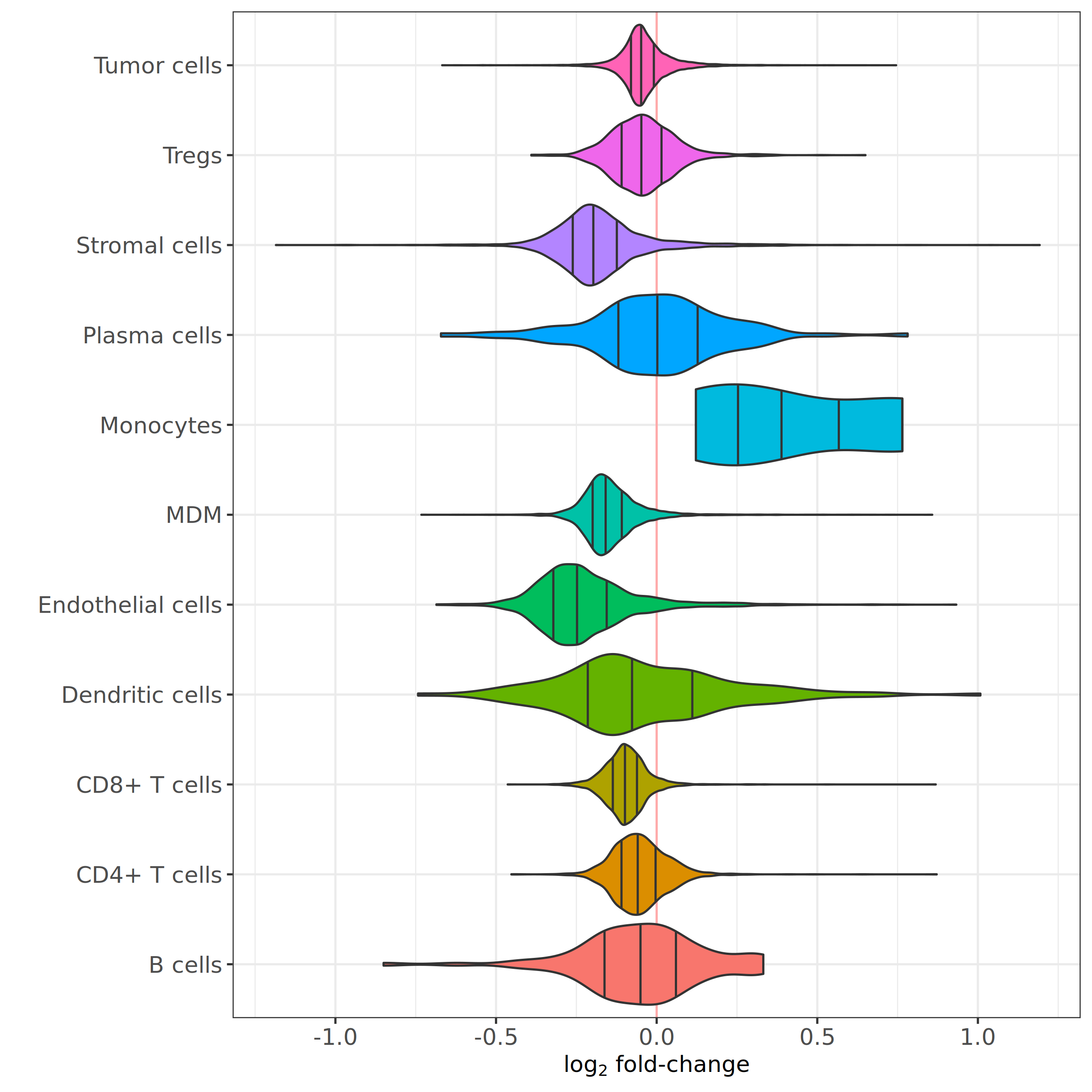


**Supplementary Figure 4: Results of the bias analysis using non-integrated expression data.** Preprocessed but non-integrated expression data was pseudobulked and subjected to the same differential expression analysis comparing MBM with ECM samples for test purpose. The resultant distribution of the log_2_-fold-changes of all tested genes showed the same trends as the main analysis (Figure 2B), indicating no bias from the integration.


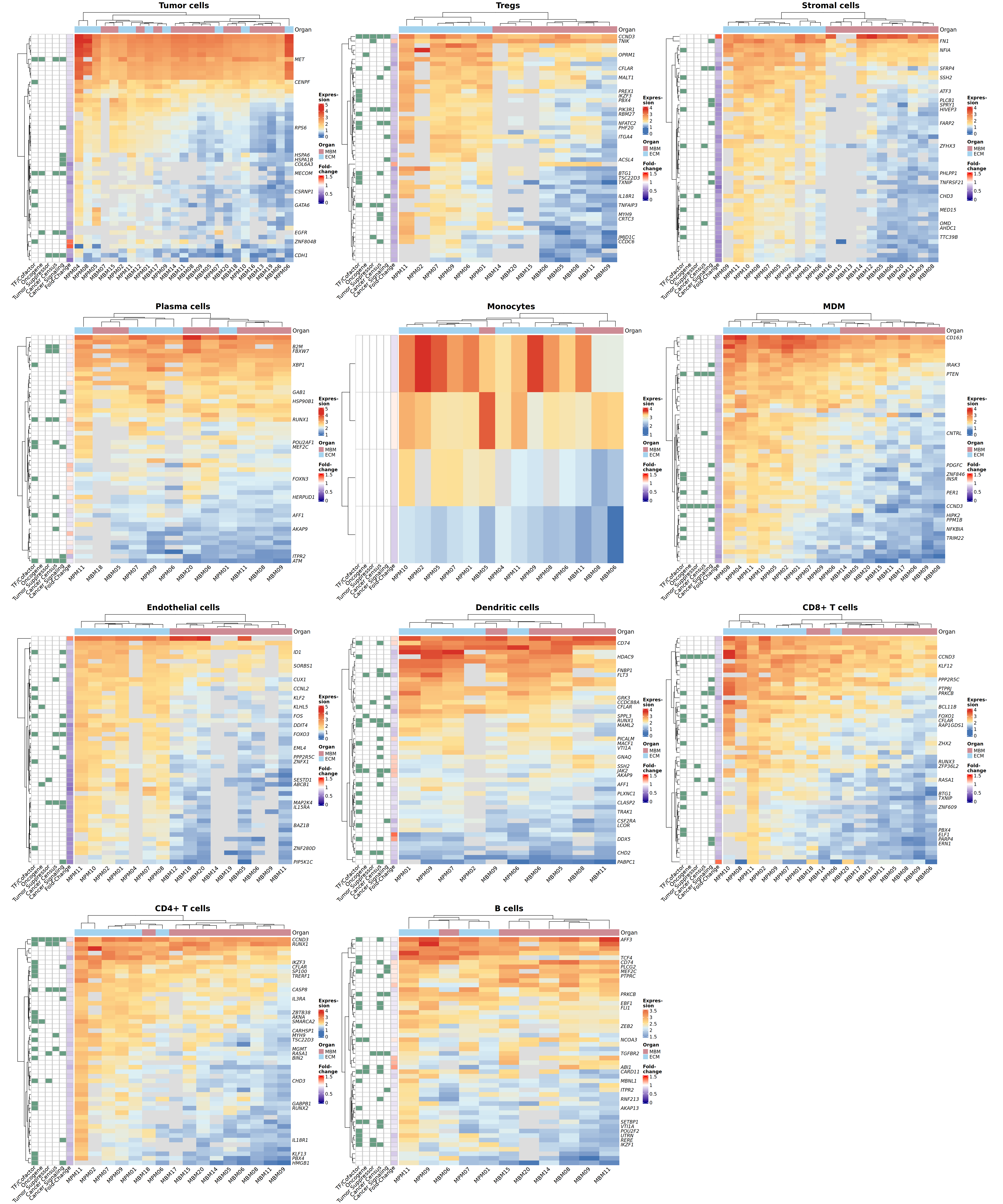


**Supplementary Figure 5:** **Heatmaps from hierarchical clustering of samples based on the top 50 differentially expressed genes (lowest p-value) of each cell type comparing MBM and ECM.** Euclidean distance and complete linkage were used. MBM and ECM samples were perfectly separated by the cell type specific signature genes of regulatory T cells, stromal cells, MDM and endothelial cells. Monocytes only have four genes in total. CD8+ T cells, CD4+ T cells, dendritic cells and B cells showed good separation. Gray areas represent genes not expressed or expressed in less than 10 cells. Fold changes were calculated as the ratio of average MBM to ECM gene expression. Cancer genes and their functions are highlighted.


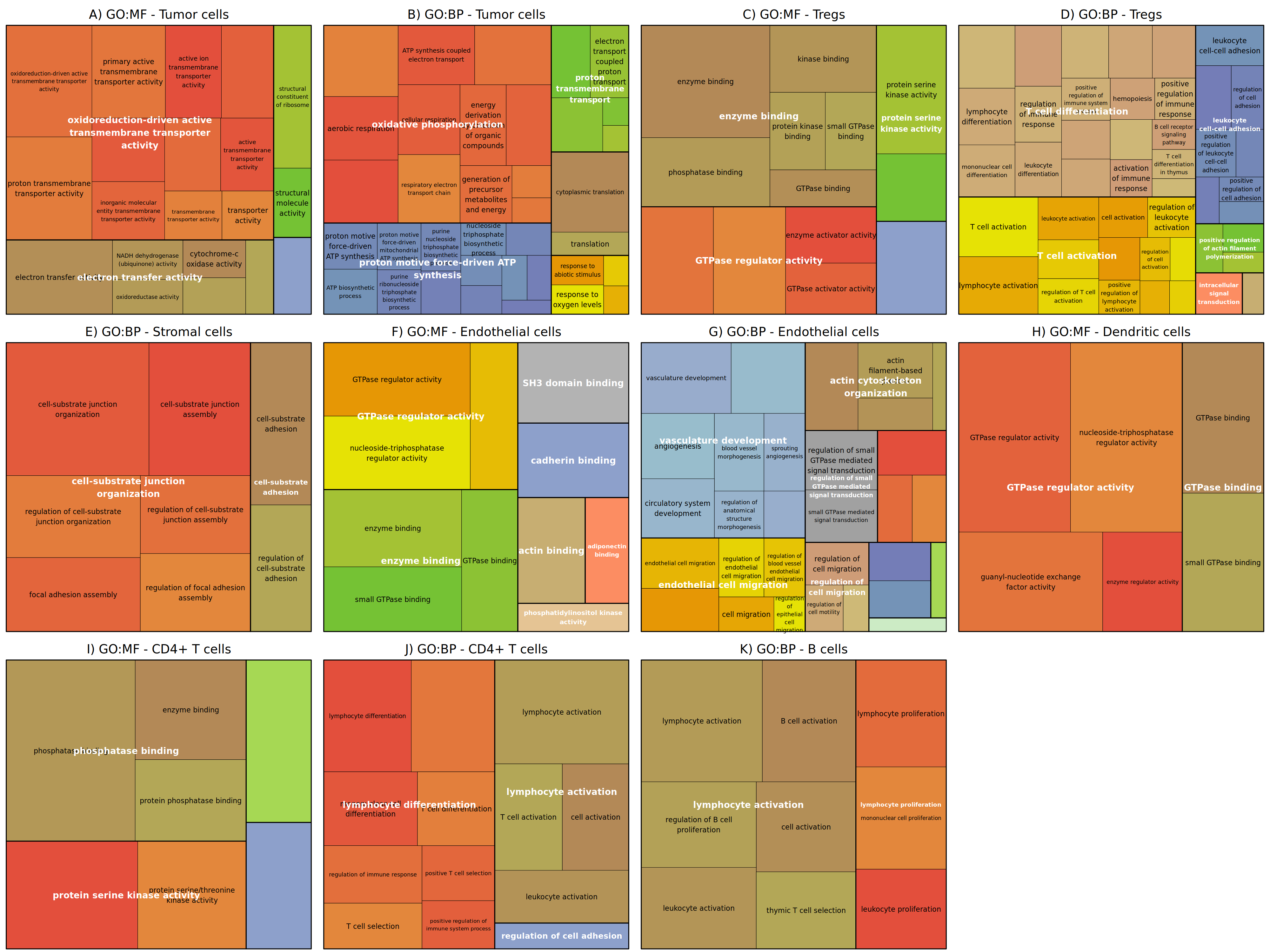


**Supplementary Figure 6: TreeMap visualization of functionally enriched Gene Ontology (GO) biological process (BP) and molecular function (MF) terms in the various cell types.** Enrichment analysis using gprofiler2 [2] was based on genes ranked by differential expression p-value (MBM vs. ECM) within each cell type. Following GO term redundancy reduction (rrvgo R package [10]), the TreeMap clustered enriched GO terms by semantic similarity, indicated by similar color shades and representative white text labels. Only cell types and GO categories with at least six enriched terms were used for plotting. The area size reflects the ranking score: -log_10_(adjusted p-value) from enrichment test (g:SCS correction). Color match across plots is coincidental.


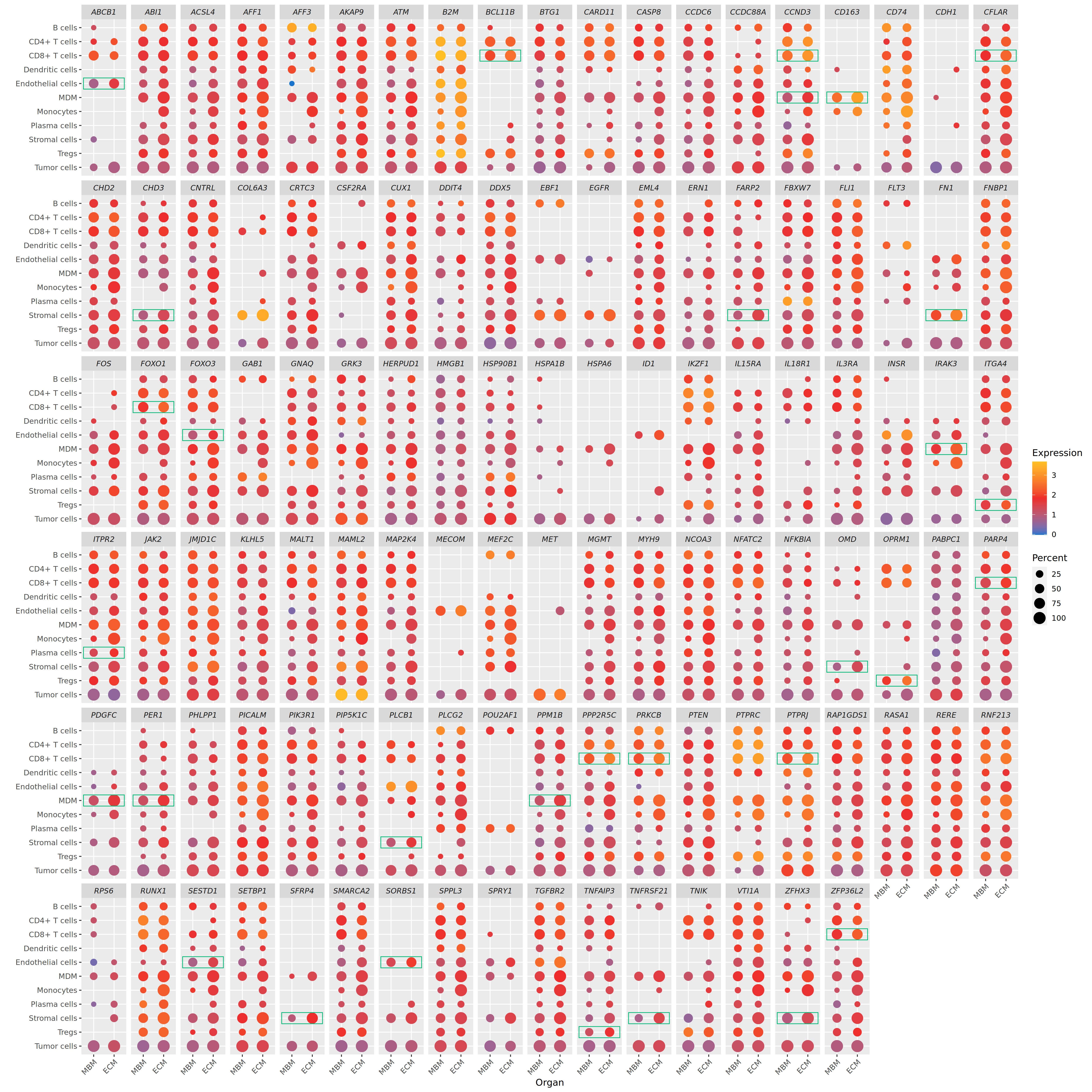


**Supplementary Figure 7: Expression patterns of cancer related signature genes across the various cell types.** Expression levels of signature genes in at least one cell type (differential expression MBM vs. ECM, top 50 genes with lowest p-value) that have at least one cancer-related annotation (oncogene, tumor suppressor, Cancer Gene Census, cancer signaling). Expression strength is indicated by color. Circle size represents the percentage of MBM and ECM patients expressing the gene. The green rectangles indicate that these genes are differentially expressed (MBM vs. ECM, FDR < 0.05) in the corresponding cell types.


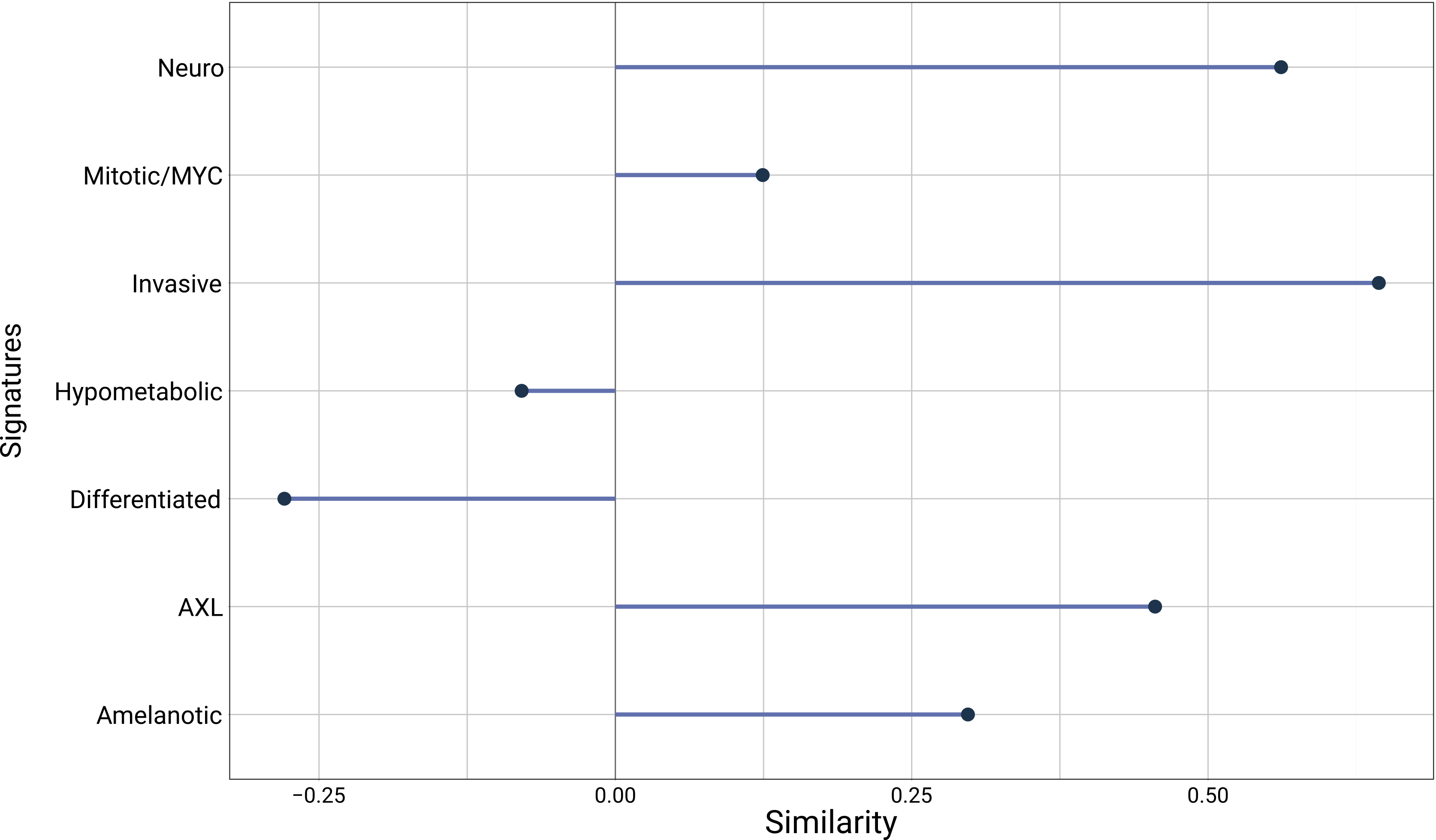
**Supplementary Figure 8: Associations of cancer-related signature genes across cell types with known signatures of melanocytic phenotypes**. The cancer-associated MBM vs. ECM signature genes (Figure 5, main manuscript) were analyzed with the help of WIMMS (What Is My Melanocytic Signature, https://wimms.tanlab.org/) to determine their association with seven principal classes of gene signatures that correlate with previously identified melanocytic phenotypes. The MBM vs. ECM signature shows positive associations with the Invasive, Neuro, AXL, Amelanotic, and Mitotic/MYC principal classes, and negative associations with the two principal classes Hypometabolic and Differentiated.


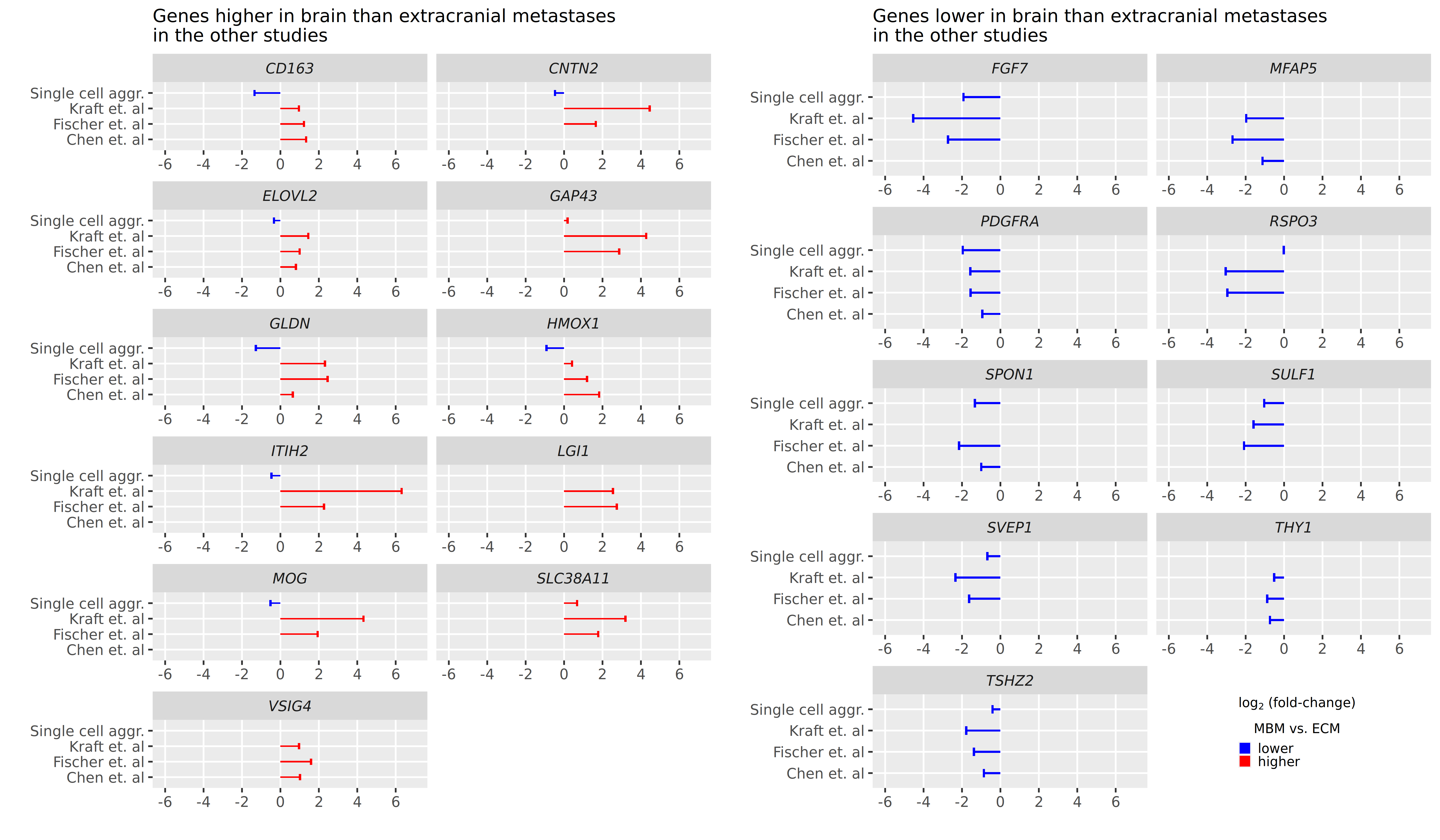


**Supplementary Figure 9: Log_2_-fold-changes of genes consistently differentially expressed in bulk tissue studies.** The plot compares log_2_-fold-changes (MBM vs. ECM) for genes identified as differentially expressed in at least two of three previously published bulk studies [6–8]. Log_2_-fold-changes are shown for the original bulk studies and for our aggregated single-cell data (calculated as the sum of expression across cell types).


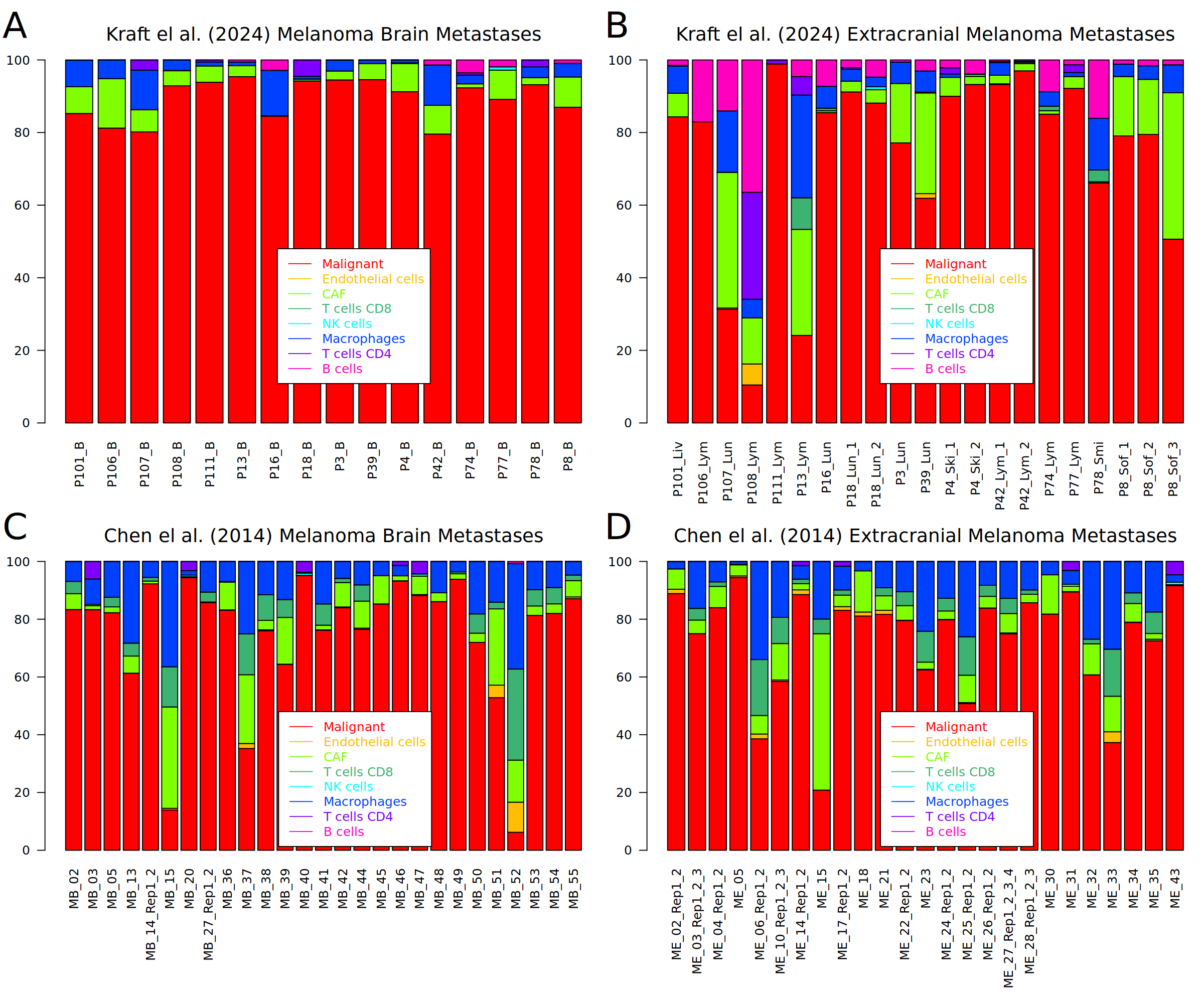
**Supplementary Figure 10: Deconvoluted cell fractions of bulk transcriptomes of melanoma metastases**. The bulk transcriptomes from melanoma metastases of Kraft et al. [8] and Chen et al. [6] were deconvoluted using CIBERSORTx to estimate the proportions of different potentially contained cell types. The required reference transcriptomes of the different cell types were taken from Tirosh et al. [11]. Malignant tumor cells (red) were predicted to be the most prevalent cell type in the analyzed bulk samples of both studies, except for a few metastasis samples shown in subpanels B, C, and D.


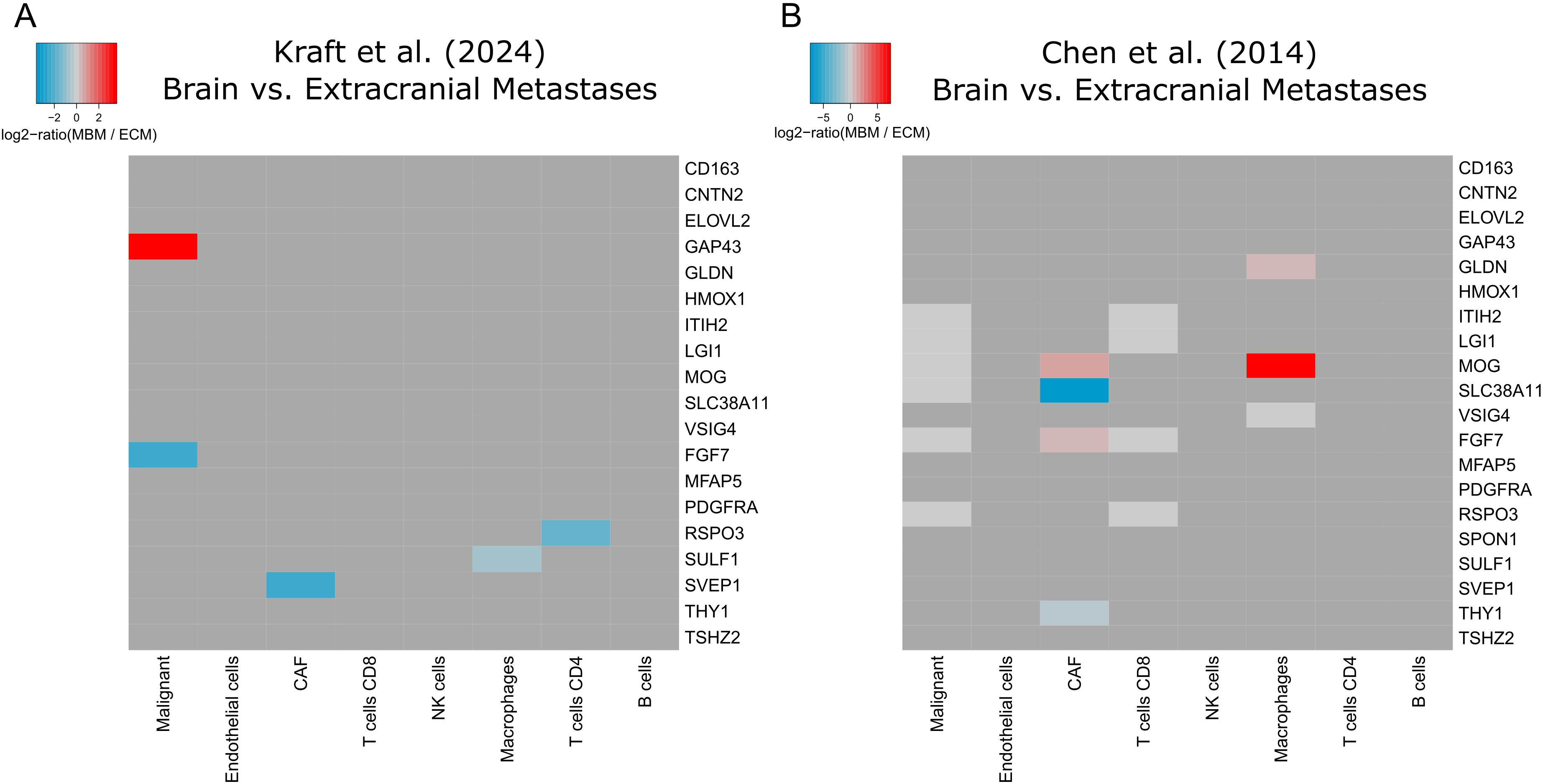


**Supplementary Figure 11: Estimated cell type-speciﬁc expression proﬁles of bulk transcriptomes of melanoma metastases.** The bulk transcriptomes from melanoma brain and extracranial metastases of Kraft et al. [8] and Chen et al. [6] were deconvoluted with CIBERSORTx to estimate their cell type-speciﬁc expression proﬁles. The required reference transcriptomes of the different cell types were taken from Tirosh et al. [11]. The estimates were used to compute gene-speciﬁc expression log_2_-ratios between brain and extracranial metastases. Reliable cell type-specific expression estimates were only possible for a few genes (blue to light grey to red), while such estimates were not possible for the majority of genes (dark grey). Only *FGF7* expression could be estimated across both studies, but its estimated expression levels differ.

# Appendix 1

**Comparison of our Methods and Results to the study of Biermann et al.**

| **Methods and Data** | **Biermann et al.** [12] | **Grützmann et al.** |
| --- | --- | --- |
| Main cohort | 21 MBM, 10 ECM patients with  snRNA-Seq and/or scRNA-Seq, TCR-Seq, SlideSeqV2 (spatial transcriptomics) | 15 MBM, 10 ECM patients with snRNA-Seq samples (6 samples were from scRNA-Seq or had no tumor cells) |
| Quality filtering | Filtered by num. of genes, num. of reads, mtRNA rate, doublets | Done in the same way |
| Preprocessing | Normalized, scaled, found variable features, neighbors and clusters | Done in the same way |
| Copy number alterations | Predicted with inferCNV [13] | Taken from Biermann et al. annotation |
| Cell type determination | Preliminary annotation with BlueprintEncodeData using R package SingleR [14], separation into immune/non-immune, tumor cells derived by inferCNV from non-immune cells, ‘FindAllMarkers’ [15] for cluster specific gene markers, manual annotation using known markers and public single-cell signatures | Taken from Biermann et al. annotation |
| Integration of samples | Canonical correlation analysis with 2000 genes | Reciprocal PCA with 17500 genes |
| Treatment of imputed values | Not removed | Removed |
| Expression aggregation | Not done | Aggregated the integrated single cell expression by cell type into one value per gene and sample |
| General analysis workflow after preprocessing | Individual analysis workflow for each cell type  Heterogeneous use of sn/scRNA-seq and integrated/non-integrated data | One unified workflow for all cell types |
| Differential gene expression MBM vs. ECM | Downsampled non-integrated data to have equal per-cell total counts between MBM and ECM (scuttle R package [16]) and downsampled to have equal numbers of cells  Then comparison with ‘FindMarkers’ with MAST [15] | Comparison with limma [1] using aggregated expression data |
| Gene set enrichment analysis of MBM vs. ECM differentially expressed genes | R package hypeR [17], pathway sets: GO BP, GO MF, Canonical Pathways, Hallmarks, Kegg, Reactome, PID, and WikiPathways | Functional enrichment with gprofiler2 [2], pathway sets: GO BP, GO MF, Kegg, WikiPathways |

**Further listing of methods and analyses, which differ between both studies**

| **Biermann et al.** |  | **Grützmann et al.** |
| --- | --- | --- |
| Diffusion component analysis with Destiny [18] to divide cell types into clusters (cell subtypes) |  | Evaluation of treatment status of ECM samples by excluding treated patients or cofactor adjustment |
| Benchmarking of integration methods Harmony [19], Conos [20], STACAS [21] |  | Comparison of differential gene expression of integrated and non-integrated expression data |
| Gene set enrichment of cell subtypes (hyper [17]) |  | Correlation analysis of aggregated and single cell data to estimate per cell type heterogeneity |
| FindMarkers with MAST for cell subtype comparison |  | Cluster analysis of cell type-specific signature genes (top 50 differentially expressed genes) |
| KINOMO [22] method for (meta)-program identification in tumor cells |  | Association of signature genes with cancer-related genes [23] |
| VIPER [24] and SCENIC [25] for transcription factor activity analysis in tumor cells |  | Analysis of signature genes for known melanoma signatures |
| ARACNe-AP [26] for master regulator analysis of tumor cells |  | Comparison of candidate genes of previous bulk RNA-seq studies |
| Enrichment analysis of MBM/ECM tumor signatures in bulk transcriptomes |  | Deconvolution of and comparison to previous bulk RNA-seq studies using CIBERSORTx [27] |
| Sequencing, confirmatory clustering and signature analysis in melanoma cell lines |  |  |
| Migration assays |  |  |
| Mice intracardiac injections of MBM cell lines |  |  |
| ATAC-seq of MBM and ECM cell lines |  |  |
| Analysis of public RNA-seq and proteomics data |  |  |
| Multiplexed immunofluorescence of further patient samples |  |  |
| RNA velocity [28], scVelo [29] analysis of MDM |  |  |
| ProjecTILs [30] for T cell analysis, T cell receptor sequencing and analysis |  |  |
| B cell chain analysis, immunofluorescence |  |  |
| Spatial transcriptomics, spatially regulated/variable gene analyses (Moran’s I , C-SIDE [31]) |  |  |

**Comparison of results**

|  | **Biermann et al.** | **Grützmann et al.** |
| --- | --- | --- |
| General findings | - Indications that data from fresh tissue scRNA-Seq and frozen, archived snRNA-Seq yield comparable global analysis outcomes  - Higher fraction of “dysfunctional” CD8+ T cells and myeloid cells in MBM compared to ECM  - Tumor cells show high transcriptional heterogeneity and variation between MBM and ECM  - Several indicators of MBM to have a neuronal-like phenotype program  - In part, several of the findings also seen in further experiments and public data | - Estimation of how a partially treated ECM group affects the analysis results  - Much more diverging global expression patterns in cells of microenvironment than in tumor cells  - Signature genes (50 genes with lowest p-value comparing MBM and ECM expression) of the microenvironment separated MBM from ECM, suggesting high contribution to MBM/ECM phenotype  - Cancer-associated signature genes associated with the known melanocytic signature classes Invasive, Neuro, AXL, Amelanotic, and Mitotic/MYC  - Candidate genes from previous bulk studies showed very mixed behavior in the data here  - No clear indicators of a neuronal-like phenotype of MBM found |
| Tumor cells | - MBM have more heterogeneous aneuploidy, higher chromosome instability  - MBM stronger MITF-high expression program  - ECM stronger AXL-high program  - MBM: tumorigenesis and maintenance, neural differentiation  - ECM: epithelial-to-mesenchymal transition  Gene set enrichment  - MBM: OxPhos, PI3K, insulin, PDFGRB, ERBB, KIT, TKI resistance  - ECM: EMT, cell adhesion, MTORC1 signaling  upstream transcription factor and protein analysis  - MBM: MITF, RELB  - ECM: CREB1, BACH1, SOX4  KINOMO  - identified programs of heterogeneity within each patient  - seven metaprograms (MP) shared across patients  - MP4 exclusive to ECM: glucose metabolism, hypoxia response, unfolded protein response, matrix proteins, cell cycle progression, TNF-alpha  - MP7 exclusive to MBM: neural development and differentiation, synapse function and formation  - Validation in other data sets: NCAM1, NELL1, LRRC1 (neuronal adhesion and differentiation), and ST6GALNAC3, TBXAS1, WNK4 consistently enriched/upregulated in MBM vs. ECM | - No differential expression between MBM and ECM  - Highly homogeneous global expression patterns in tumor cell expression within and between MBM and ECM  - Even higher homogeneity within MBM  - Signature genes did not separate MBM from ECM, suggesting low contribution to distinct MBM/ECM phenotype  - Signature genes encode transcription factors, co-factors, oncogenes, tumor suppressors and are involved in cancer signaling  - Oxidative phosphorylation, ATP synthesis, translation, proton transmembrane transport, MAPK signaling pathway, adherens junction, neuroinflammation enriched among tumor cell genes ranked by differential expression p-value comparing MBM with ECM  - Indicators of a more flexible energy metabolism in MBM than ECM tumor cells |
| Stromal cells | Not specifically analyzed | - Genes ranked by differential expression p-value comparing MBM with ECM are enriched for focal adhesion, cell-substrate junction/adhesion, and pathways crucial for cancer proliferation and migration: EGFR, PDGFR, VEGFR, and TGF beta signaling  - Signature genes separate MBM from ECM perfectly in cluster analysis |
| Endothelial cells | Not specifically analyzed | - Genes ranked by differential expression p-value are enriched for pathways of blood vessel development  - Signature genes separate MBM from ECM perfectly in cluster analysis |
| MDM cells | - Two major clusters discovered MDM-c1, MDM-c2 (FTL+ MDM)  Differential gene expression MDM-c1 vs. MDM-c2  - gene *FTL* most strongly different  - MDM FTL+: higher electron transport chain expression, ROX detoxification (GPX4), IFN-γ response, antigen presenting genes, …  - MDM-c1: higher efferocytosis receptor, several pro-tumorigenic marker genes (e.g. *CD163*)  scVelo and RNA velocity analysis: MDM-c1 may arise from earlier MDM FTL+ cells  MBM vs. ECM differential gene expression  - MBM: lower matrix protein gene and antigen presentation genes expression, higher expression of *CD163*, *TLR2*, *MERTK*, *AXL*, and *IL2RA*, higher expression of genes associated with pro-tumorigenic phenotype  - Differences preserved when stratifying into MDM FTL+ and MDM-c1  - MDM, esp. MDM-c1, more frequent in MBM vs. ECM | - Neuroinflammation, EGF EGFR signaling and others enriched among genes ranked by differential expression p-value  - Signature genes separate MBM from ECM perfectly in cluster analysis |
| Microglia | - 2 major clusters MG1, MG2  Diff. gene expression MG1 vs. MG2  - MG1:  - more higher expression of SPP1, chemokines, inflammatory cytokines  - Enrichment in MG1: inflammatory and activation pathways, others | Not specifically analyzed |
| T/NK cells | CD8+ T cells divided into 2 major populations TOX+ (dysfunctional) and TCF7 (effector memory T cells with progenitor-like function)  - TOX+: strong immune checkpoint expression, effector genes, chemokines  - TCF7+: naïve/memory phenotype marker gene expression, effector molecules, low immune checkpoint expression  - MBM: more TOX+ CD8+ T cells and CXCL13+ CD4+ T cells than ECM  - Analyzed TCR-seq data for clonal expansion  Integrated diffusion component analysis  - clonal expansion varied along TCF7+ to TOX+ CD8+ T cell trajectory indicating progressive loss of progenitor function and increased differentiation, same in MBM and ECM  Expanded vs. non-expanded CD8 T cells (DGE + enrichment)  - non-expanded: enrichment of IL7R, LTB, SELL, TCF7  - expanded: enrichment of cytokine production, cytotoxicity, co-stimulation, immune checkpoints, terminal differentiation  - suggests differences in immune checkpoint expression between MBM and ECM | - Signature genes of Tregs separate MBM from ECM perfectly in cluster analysis  - Enrichment of T cell receptor signaling, lymphocyte activation/differentiation, especially Th1/Th2/Th17 cell differentiation, PD-L1/PD-1 checkpoint, ErbB, MAPK, TNF, PI3K/Akt/mTOR, EGFR, VEGF, NF-kappa B signaling in CD4+, CD8+ and regulatory T cells in genes ranked by differential expression p-value  - CD4+ T cells enriched in multiple interleukin signaling pathways  - Neuroinflammation enriched among Tregs |
| B cells/plasma cells | - Identified naïve B cells, activated B cells, plasma cells  - Evidence that this differentiation takes place inside tumor  - Extra cohort of MBMs and ECMs: plasma cells are spatially restricted, plasma cell aggregates more abundant in MBM, may be similar to tertiary lymphoid structures in other tumors | - Enrichment of B cell activation, proliferation and development and EGF EGFR signaling in genes ranked by differential expression p-value  - Signature genes separate MBM from ECM well in cluster analysis |
| Spatial transcriptomics | - Fraction of malignant/non-malignant cells correlated with that of snRNA-seq, showed expected compositional variability  - Determined spatially variable genes with highest spatial autocorrelation  - Categorized genes by biol. function, determine spatial expression across cohort and asses cell type co-localization  - Again seen spatially restricted clusters of lymphoid aggregates dominated by plasma cells  - Big regions of cancer antigen presentation reciprocal to expression of TIMP1 (matrix metalloproteinase, potential role in promoting melanoma immune evasion)  - Tumor cells: spatially dichotomous expression of OxPhos and glycolysis metabolic pathways | Not analyzed |

# Additional File 1

**Additional-File-1-PubMed-abstracts-for-genes.html**

**Results from the literature search on differentially expressed genes and derived signature genes.** The NCBI PubMed database was searched for corresponding genes using the terms "gene symbol AND melanoma," and then "gene symbol AND tumor" if no initial results were found. Up to ten bibliographic entries were downloaded and compiled into this HTML file. Genes are presented alphabetically. It is indicated in which cell type they are differentially expressed or signature genes. For clarity, gene names, their synonyms, and keyword fragments (“melan”, “tumor”, “cancer”, and “neopla”) are highlighted.

# References

1. Ritchie ME, Phipson B, Wu D, Hu Y, Law CW, Shi W, et al. limma powers differential expression analyses for RNA-sequencing and microarray studies. Nucleic Acids Res. 2015;43:e47.

2. Peterson H, Kolberg L, Raudvere U, Kuzmin I, Vilo J. gprofiler2 -- an R package for gene list functional enrichment analysis and namespace conversion toolset g:Profiler. F1000Res [Internet]. 2020 [cited 2025 May 20];9:ELIXIR-709. Available from: https://pmc.ncbi.nlm.nih.gov/articles/PMC7859841/

3. Carbon S, Douglass E, Dunn N, Good B, Harris NL, Lewis SE, et al. The Gene Ontology Resource: 20 years and still GOing strong. Nucleic Acids Res [Internet]. 2019 [cited 2025 May 20];47:D330–8. Available from: https://pubmed.ncbi.nlm.nih.gov/30395331/

4. Kanehisa M, Goto S. KEGG: kyoto encyclopedia of genes and genomes. Nucleic Acids Res. 2000;28:27–30.

5. Agrawal A, Balcı H, Hanspers K, Coort SL, Martens M, Slenter DN, et al. WikiPathways 2024: next generation pathway database. Nucleic Acids Res [Internet]. 2024 [cited 2025 May 20];52:D679–89. Available from: https://pubmed.ncbi.nlm.nih.gov/37941138/

6. Chen G, Chakravarti N, Aardalen K, Lazar AJ, Tetzlaff MT, Wubbenhorst B, et al. Molecular profiling of patient-matched brain and extracranial melanoma metastases implicates the PI3K pathway as a therapeutic target. Clin Cancer Res. 2014;20:5537–46.

7. Fischer GM, Jalali A, Kircher DA, Lee W-C, McQuade JL, Haydu LE, et al. Molecular profiling reveals unique immune and metabolic features of melanoma brain metastases. Cancer Discov. 2019;9:628–45.

8. Kraft T, Grützmann K, Meinhardt M, Meier F, Westphal D, Seifert M. Personalized identification and characterization of genome-wide gene expression differences between patient-matched intracranial and extracranial melanoma metastasis pairs. Acta Neuropathol Commun [Internet]. 2024 [cited 2025 May 20];12. Available from: https://pubmed.ncbi.nlm.nih.gov/38671536/

9. Andrecut M. Parallel GPU implementation of iterative PCA algorithms. J Comput Biol [Internet]. 2009 [cited 2025 Aug 23];16:1593–9. Available from: www.liebertpub.com

10. Sayols S. rrvgo: a Bioconductor package for interpreting lists of Gene Ontology terms. MicroPubl Biol [Internet]. 2023 [cited 2025 May 20];2023. Available from: https://pubmed.ncbi.nlm.nih.gov/37151216/

11. Tirosh I, Izar B, Prakadan SM, Wadsworth MH, Treacy D, Trombetta JJ, et al. Dissecting the multicellular ecosystem of metastatic melanoma by single-cell RNA-seq. Science [Internet]. 2016 [cited 2025 May 20];352:189–96. Available from: https://pubmed.ncbi.nlm.nih.gov/27124452/

12. Biermann J, Melms JC, Amin AD, Wang Y, Caprio LA, Karz A, et al. Dissecting the treatment-naive ecosystem of human melanoma brain metastasis. Cell [Internet]. 2022 [cited 2023 Mar 10];185:2591-2608.e30. Available from: https://pubmed.ncbi.nlm.nih.gov/35803246/

13. Haas B, Tickle T. InferCNV https://github.com/broadinstitute/InferCNV. 2017.

14. Aran D, Looney AP, Liu L, Wu E, Fong V, Hsu A, et al. Reference-based analysis of lung single-cell sequencing reveals a transitional profibrotic macrophage. Nat Immunol. 2019;20:163–72.

15. Hafemeister C, Satija R. Normalization and variance stabilization of single-cell RNA-seq data using regularized negative binomial regression. Genome Biol. 2019;20:296.

16. McCarthy DJ, Campbell KR, Lun ATL, Wills QF. Scater: pre-processing, quality control, normalization and visualization of single-cell RNA-seq data in R. Bioinformatics. 2017;33:1179–86.

17. Federico A, Monti S. hypeR: an R package for geneset enrichment workflows. Bioinformatics. 2020;36:1307–8.

18. Angerer P, Haghverdi L, Büttner M, Theis FJ, Marr C, Buettner F. destiny: diffusion maps for large-scale single-cell data in R. Bioinformatics. 2016;32:1241–3.

19. Korsunsky I, Millard N, Fan J, Slowikowski K, Zhang F, Wei K, et al. Fast, sensitive and accurate integration of single-cell data with Harmony. Nat Methods. 2019;16:1289–96.

20. Barkas N, Petukhov V, Nikolaeva D, Lozinsky Y, Demharter S, Khodosevich K, et al. Joint analysis of heterogeneous single-cell RNA-seq dataset collections. Nat Methods. 2019;16:695–8.

21. Andreatta M, Carmona SJ. STACAS: Sub-Type Anchor Correction for Alignment in Seurat to integrate single-cell RNA-seq data. Bioinformatics. 2021;37:882–4.

22. Tagore S, Wang Y, Biermann J, Rabadan R, Azizi E, Izar B. KINOMO: A non-negative matrix factorization framework for recovering intra- and inter-tumoral heterogeneity from single-cell RNA-seq data. bioRxiv [Internet]. 2022 [cited 2025 Aug 22];2022.05.02.490362. Available from: https://www.biorxiv.org/content/10.1101/2022.05.02.490362v1

23. Hu M, Coleman S, Judson-Torres RL, Tan AC. The classification of melanocytic gene signatures. Pigment Cell Melanoma Res [Internet]. 2024 [cited 2025 Aug 22];37:854–63. Available from: https://pubmed.ncbi.nlm.nih.gov/39072997/

24. Alvarez MJ, Shen Y, Giorgi FM, Lachmann A, Ding BB, Ye BH, et al. Functional characterization of somatic mutations in cancer using network-based inference of protein activity. Nat Genet. 2016;48:838–47.

25. Aibar S, González-Blas CB, Moerman T, Huynh-Thu VA, Imrichova H, Hulselmans G, et al. SCENIC: single-cell regulatory network inference and clustering. Nat Methods. 2017;14:1083–6.

26. Lachmann A, Giorgi FM, Lopez G, Califano A. ARACNe-AP: gene network reverse engineering through adaptive partitioning inference of mutual information. Bioinformatics. 2016;32:2233–5.

27. Newman AM, Steen CB, Liu CL, Gentles AJ, Chaudhuri AA, Scherer F, et al. Determining cell type abundance and expression from bulk tissues with digital cytometry. Nat Biotechnol. 2019;37:773–82.

28. La Manno G, Soldatov R, Zeisel A, Braun E, Hochgerner H, Petukhov V, et al. RNA velocity of single cells. Nature. 2018;560:494–8.

29. Bergen V, Lange M, Peidli S, Wolf FA, Theis FJ. Generalizing RNA velocity to transient cell states through dynamical modeling. Nat Biotechnol. 2020;38:1408–14.

30. Andreatta M, Corria-Osorio J, Müller S, Cubas R, Coukos G, Carmona SJ. Interpretation of T cell states from single-cell transcriptomics data using reference atlases. Nat Commun. 2021;12:2965.

31. Cable DM, Murray E, Shanmugam V, Zhang S, Zou LS, Diao M, et al. Cell type-specific inference of differential expression in spatial transcriptomics. Nat Methods. 2022;19:1076–87.
